# Supplementary material for: Distinct oligodendrocyte populations have spatial preference and different responses to spinal cord injury
Source: Nat Commun. 2020 Nov 17;11:5860. doi: 10.1038/s41467-020-19453-x (PMC7673029; doi:10.1038/s41467-020-19453-x)
Supplement: Supplementary file 1 — Supplementary Information [file 41467_2020_19453_MOESM1_ESM.pdf]

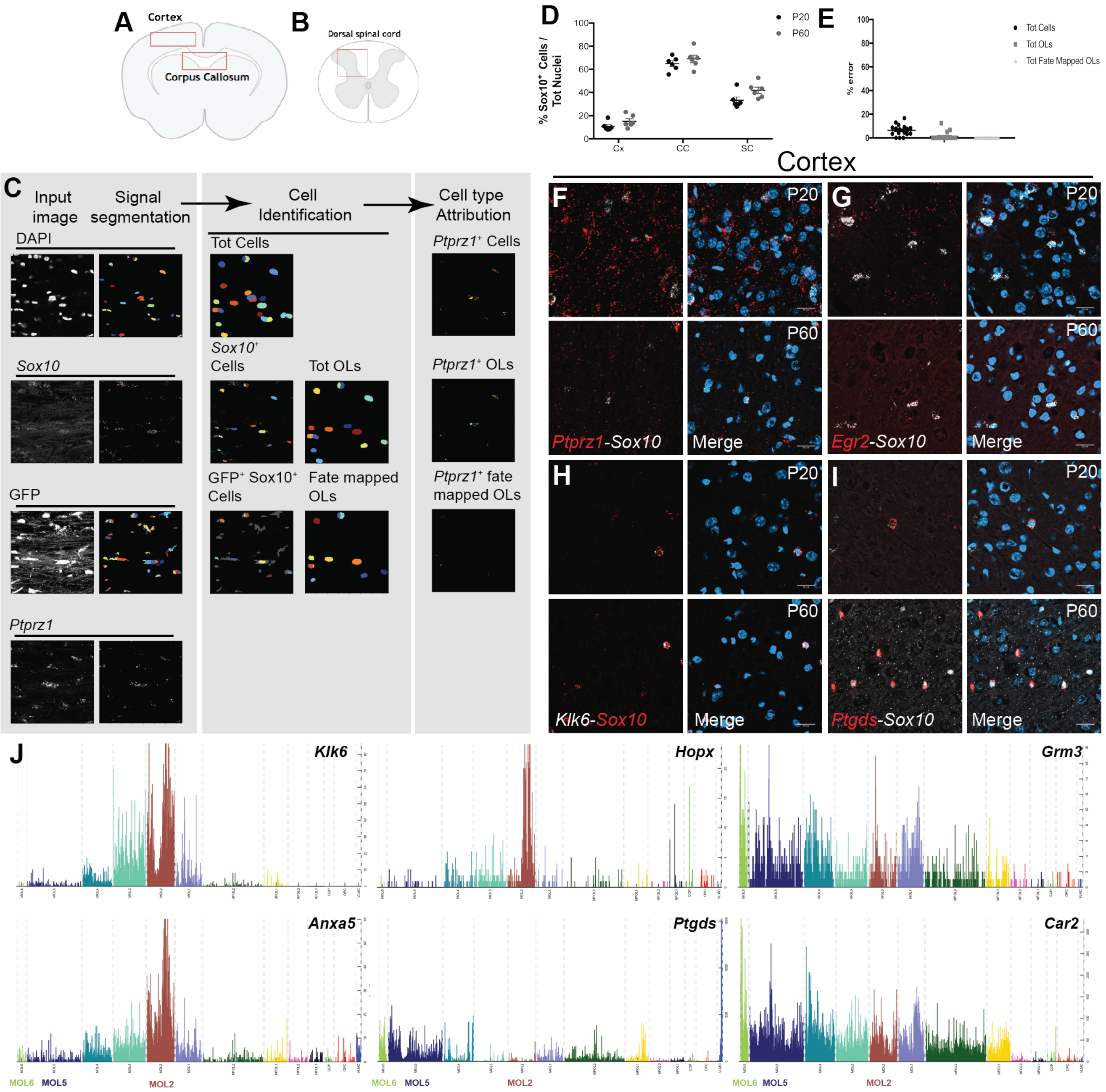

Supplementary Fig. 1. Spatial distribution of OL populations in the juvenile and adult central nervous system. (**A-B**) Schematics of coronal sections of the mouse brain (A) and spinal cord (B). Red squares highlight the systematically analyzed CNS regions. (**C**) Schematic overview and representative analyzed image output of the customized CellProfiler pipeline used in this study. (**D**) Percentage of the OL lineage cells (Sox10+ cells) calculated on the total number of nuclei shows the expected differential enrichment of the OL lineage in the analyzed regions. Data are presented as Mean ± SEM. n = 5-8 animals per condition, and can be assessed in the Source Data file. Exact p-values are reported in the Source Data file. (**E**) Percentage of the segmentation error of the used pipeline. Data are presented as Mean ± SEM, and can be assessed in the Source Data file. n = 5-18 images per condition. (**F-I**) Confocal representative images of the distribution of OPC-COPs (*Ptprz1*+ OL lineage cells, F), MOL1 (*Egr2*+ OL lineage cells, G), MOL2 (*Klk6*+ OL lineage cells, H), and MOL5/6 (*Ptgds*+ OL lineage cells, I) in the cortex of the juvenile (P20) and adult (P60). (**J**) Single-cell RNA-seq expression profiles of genes enriched in MOL2, MOL5 and MOL6 in the oligodendrocyte lineage in the juvenile and adult CNS at a single cell level, as in Marques et al, 2016[5]. Plots from http://linnarssonlab.org/oligodendrocytes/. Scale bar = 20µm. Cx = cortex, CC = corpus callosum, SC = spinal cord. Source data are provided as a Source Data file.

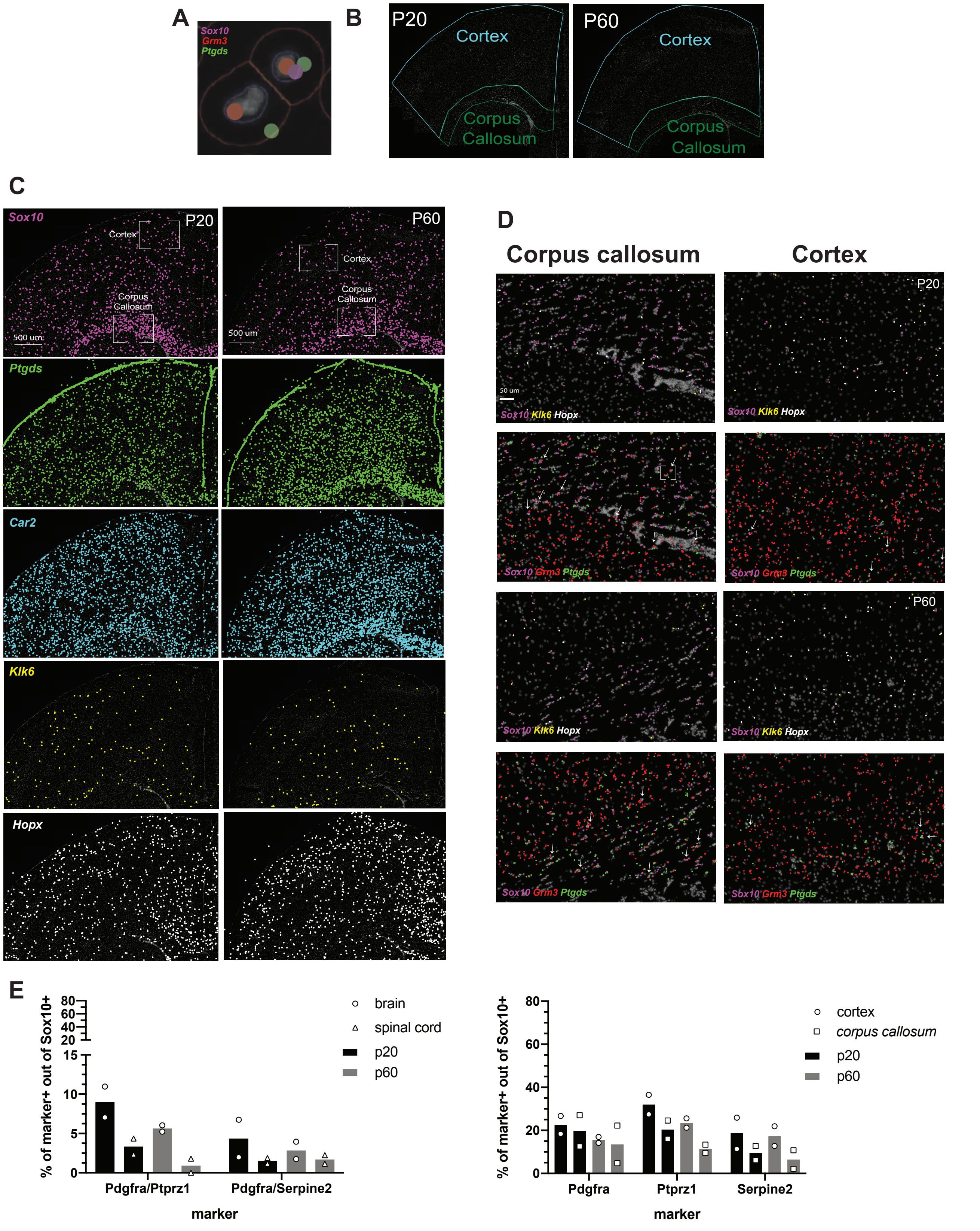

Supplementary Fig. 2. In Situ Sequencing (ISS) analysis of OPCs, MOL2 and MOL5/6 in P20 and P60 brain and spinal cord. (A) Close up of 2 segmented cells in the P20 corpus callosum (white rectangle in D), one cell triple positive for *Ptgds/Grm3/Sox10*, and the other cell double positive for *Ptgds/Grm3*. **(B)** Delineation of regions of interest (cortex and corpus callosum) defined for quantifications in ISS. **(C)** Representative images of the distribution of OL lineage cells (*Sox10*+) and cells positive *Ptgds, Car2, Klk6 and Hopx* in the analysed regions of the brain (cortex and corpus callosum) at P20 and P60. White rectangles highlight the regions shown in higher magnification in D. **(D)** High magnification images of corpus callosum and cortex regions depicted in C, with triple labelling of OL lineage cells (*Sox10*+) positive for *Ptgds/Grm3* (MOL5/6) and *Hopx/Klk6* (MOL2). White arrows exemplify cells triple labelled with *Sox10/Ptgds/Grm3.* **(E)** Quantification of OPC/COPs, subpopulations contribution to the OL lineage. Percentage of the OL lineage cells (*Sox10*+), also positive for *Pdgra,* and/or *Ptprz1* and/or *Serpine2* in the brain (corpus callosum and cortex) and spinal cord in juvenile (P20, black) and adulthood (P60, grey). One section per animal in 2 mice were analyzed. Source data are provided as a Source Data file.

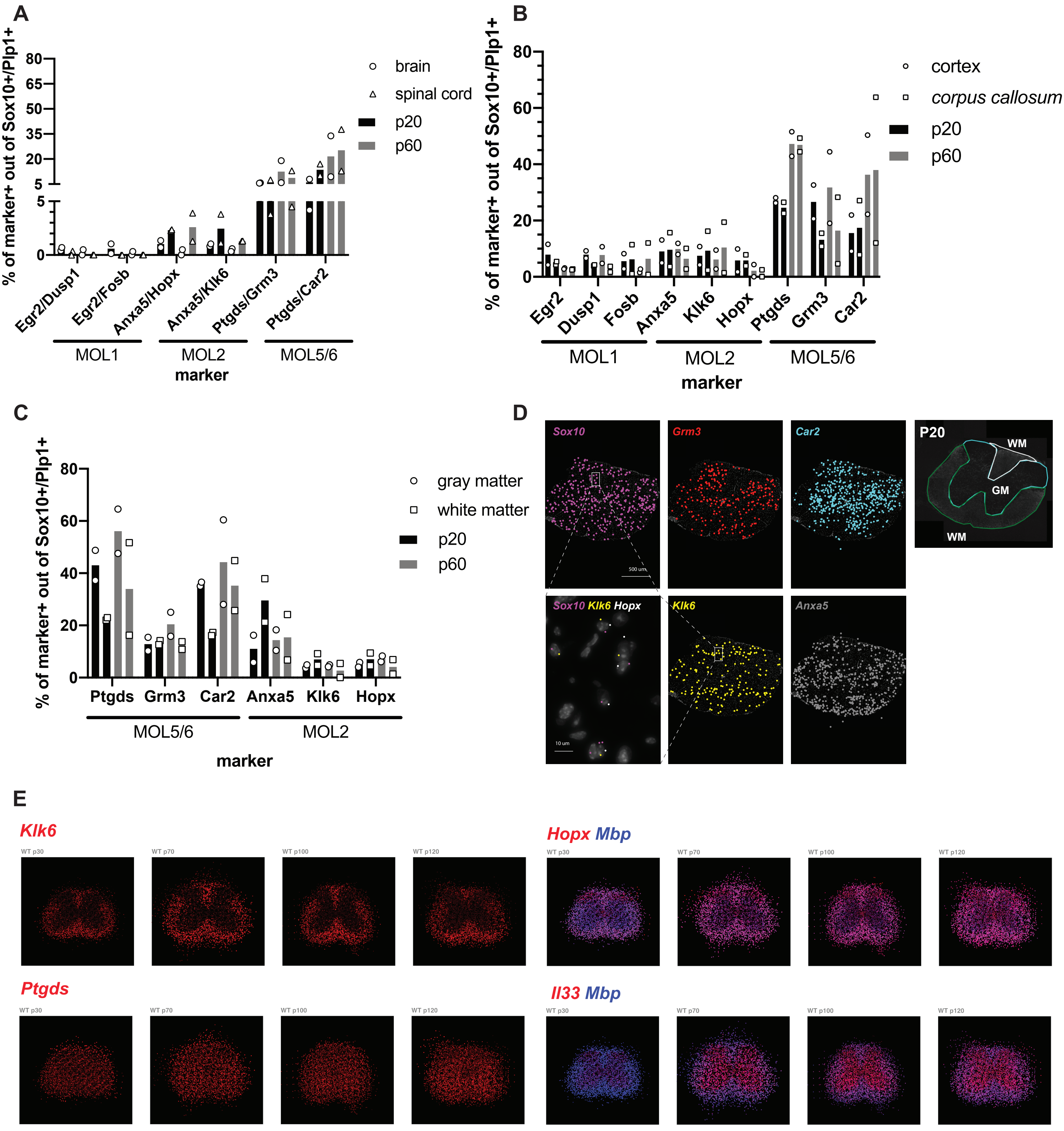

Supplementary Fig. 3. In Situ Sequencing (ISS) and spatial transcriptomics analysis of markers for MOL1, MOL2 and MOL5/6 in the brain and spinal cord at P20 and P60. (**A, B**) Quantification of MOL1 MOL2 and MOL5/6 subpopulations contribution to the OL lineage. **(A)** Percentage of the OL lineage cells (*Sox10*⁺ */Plp1*⁺), also positive for *Egr2/Dusp1* or *Egr2/Fosb* (MOL1), *Anxa5/Hopx* or *Anxa5/Klk6* (MOL2), *Ptgds/Grm3* or *Ptgds/Car2* (MOL5/6), in the brain (corpus callosum and cortex – circle) and spinal cord (triangle) in juvenile (P20, black) and adulthood (P60, grey). One section per animal in 2 mice were analyzed. **(B)** Percentage of the OL lineage cells (*Sox10*⁺ */Plp1*⁺), also positive for *Egr2, Dusp1* or *Fosb* (MOL1), *Anxa5, Klk6* or *Hopx* (MOL2), *Ptgds,* and/or *Grm3* and/or *Car2* (MOL5/6), in the corpus callosum (circle) and cortex (square) in juvenile (P20, black) and adulthood (P60, grey). One section per animal in 2 mice were analyzed. (**C**) Quantification of the MOL2 and MOL5/6 subpopulations contribution to the OL lineage. Percentage of the OL lineage cells (*Sox10*⁺ */Plp1*⁺), also positive for *Ptgds, Grm3* or *Car2* (MOL5/6), *Anxa5, Hopx* or *Klk6* (MOL2) in the grey matter (circle) and white matter (square) in juvenile (P20, dark) and adulthood (P60, grey) spinal cord, as delimited in Supplementary Fig. 3D. One section per animal in 2 mice were analyzed. **(D)** Representative images of the distribution of OL lineage cells (*Sox10*+) and cells positive *Ptgds, Grm3, Car2, Klk6 and Anx5* in the P20 spinal cord. White rectangles highlight a region shown in higher magnification with cells triple labeled with *Sox10/Klk6/Hopx*. **(E)** Spatial distribution of expression of *Klk6*, *Hopx*, *Ptgds*, *Il33 and Mbp* in the spinal cord of wild type mice from P30 to P120, as detected by spatial transcriptomics in Maniatis et. al., 2019[18] (https://als-st.nygenome.org/)[19]. Source data are provided as a Source Data file.

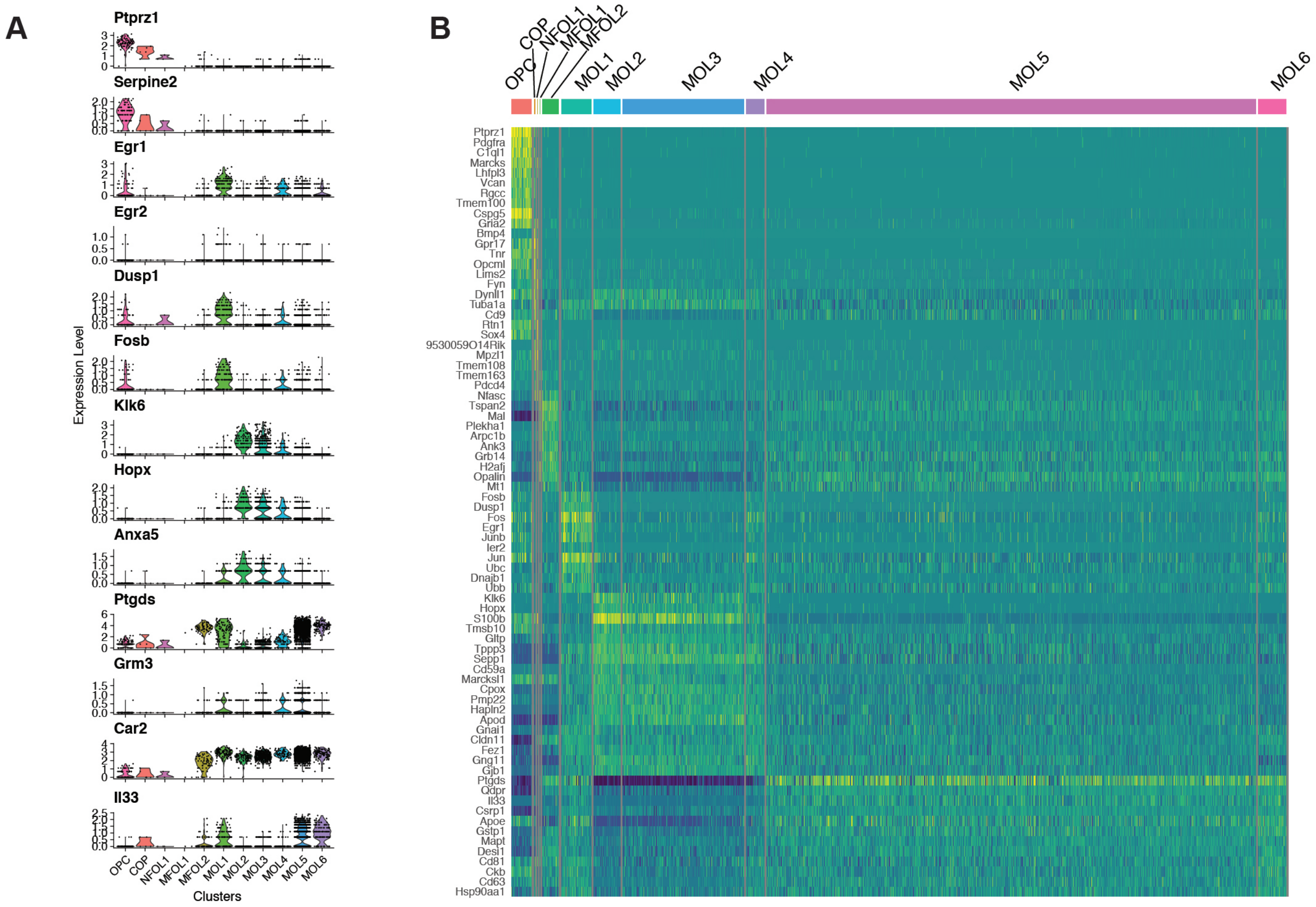

Supplementary Fig. 4. Louvain clustering analysis of the OL lineage cells. (**A**) Violin plots depicting the expression of canonical markers for OPCs, MOL1, MOL2 and MOL5/6, from P60 corpus callosum scRNA-Seq data (Figure 3); (**B**) Heat-map of differential gene expression highlighting the enriched gene modules (right) characterizing the identified clusters by Louvain clustering analysis, from P60 corpus callosum scRNA-Seq data (Figure 3). Distance matrix = Spearmann correlation. OPC = oligodendrocyte progenitor cell, COP = committed OPC, NFOL = newly formed oligodendrocyte, MFOL = myelin forming oligodendrocyte, MOL = mature oligodendrocyte.

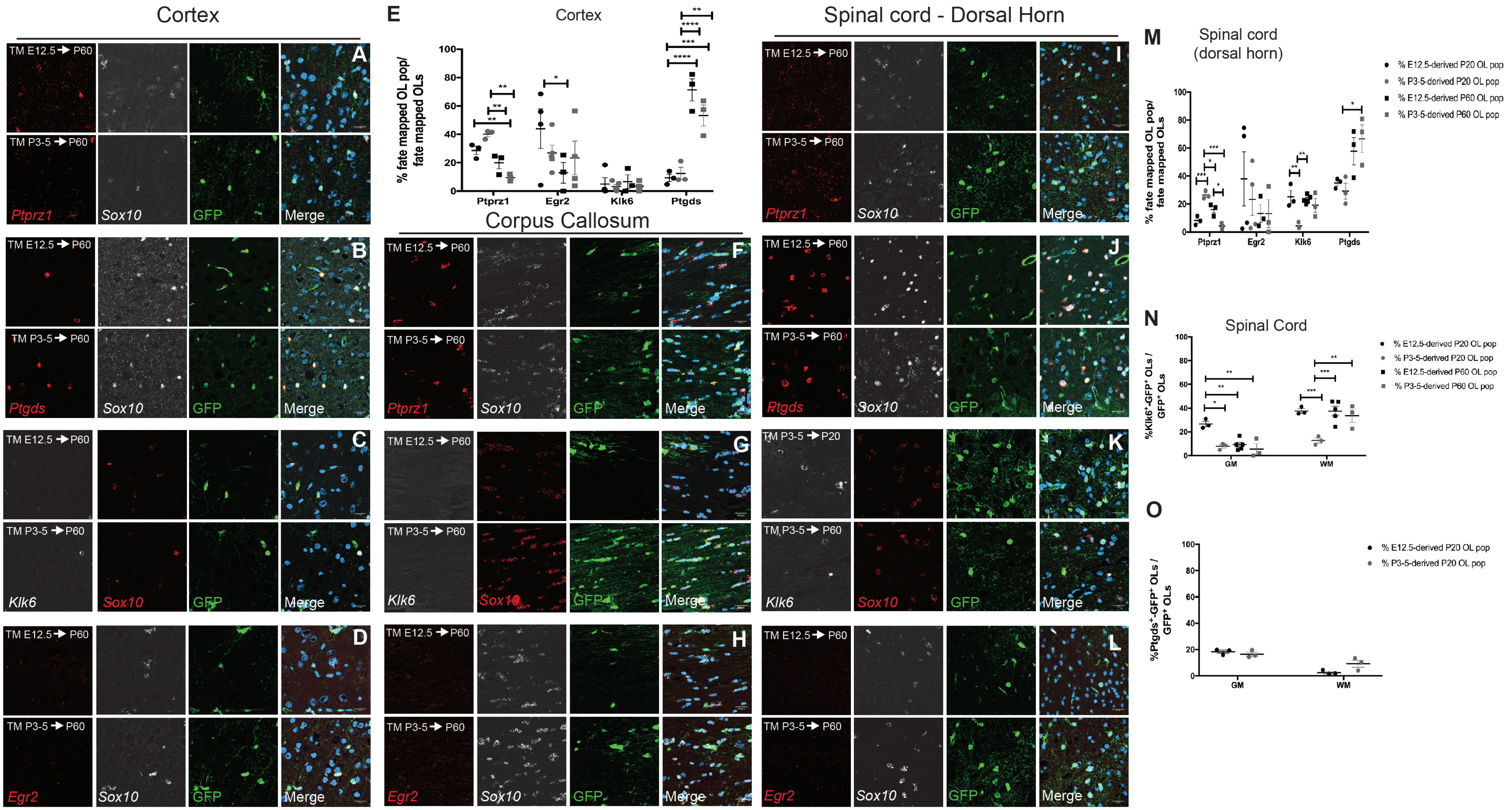

Supplementary Fig. 5. Pre- and postnatal OPCs equally contribute to the generation of MOL populations in the juvenile and adult central nervous system. (A-D, F-L) Confocal representative images show embryonically (TM E12.5)- and postnatally (TM P3-5)-derived OPCs-COPs (Ptprz1+-GFP+ OL lineage cells. A, F, I), MOL5/6 (Ptgds+-GFP+ OL lineage cells. B, J), MOL2 (Klk6+-GFP+ OL lineage cells. C, G, K), MOL1 (Egr2+-GFP+ OL lineage cells. D, H, L) in the adult (P60) cortex (A-D), corpus callosum (F-H), and dorsal horn of the spinal cord (I-L). Scale bar = 20 µm. **(E, M)** Percentages of the fate mapped OPCs-COPs (Ptprz1+-GFP+ OL lineage cells), MOL1 (Egr2+-GFP+ OL lineage cells), MOL2 (Klk6+-GFP+ OL lineage cells), and MOL5/6 (Ptgds+-GFP+ OL lineage cells) populations are calculated on the total number of fate mapped OL lineage cells (Sox10+-GFP+ cells) in the juvenile and adult cortex (E) and spinal cord (M). Data are presented as Mean ± SEM. n = 3-5 animals per condition, and can be assessed in the Source Data file. **(N-O)** Percentages of the embryonically (TM E12.5)- and postnatally (TM P3-5)-derived MOL2 (Klk6+-GFP+ OL lineage cells. N), and MOL5/6 (Ptgds+-GFP+ OL lineage cells. (O) populations are calculated on the total number of fate mapped OL lineage cells (Sox10+ -GFP+ cells) in the spinal cord white and grey matter. Data are presented as Mean ± SEM. n = 3-5 animals per condition, and can be assessed in the Source Data file. Asterisks indicate a significant difference between conditions (*p ≤ 0.05, **p ≤ 0.01, ***p ≤ 0.001, ****p ≤ 0.0001, 2-way ANOVA with Sidak's correction). Exact p-values are reported in the Source Data file. TM = tamoxifen, GFP = green fluorescent protein, GM = grey matter, WM = white matter. Source data are provided as a Source Data file.

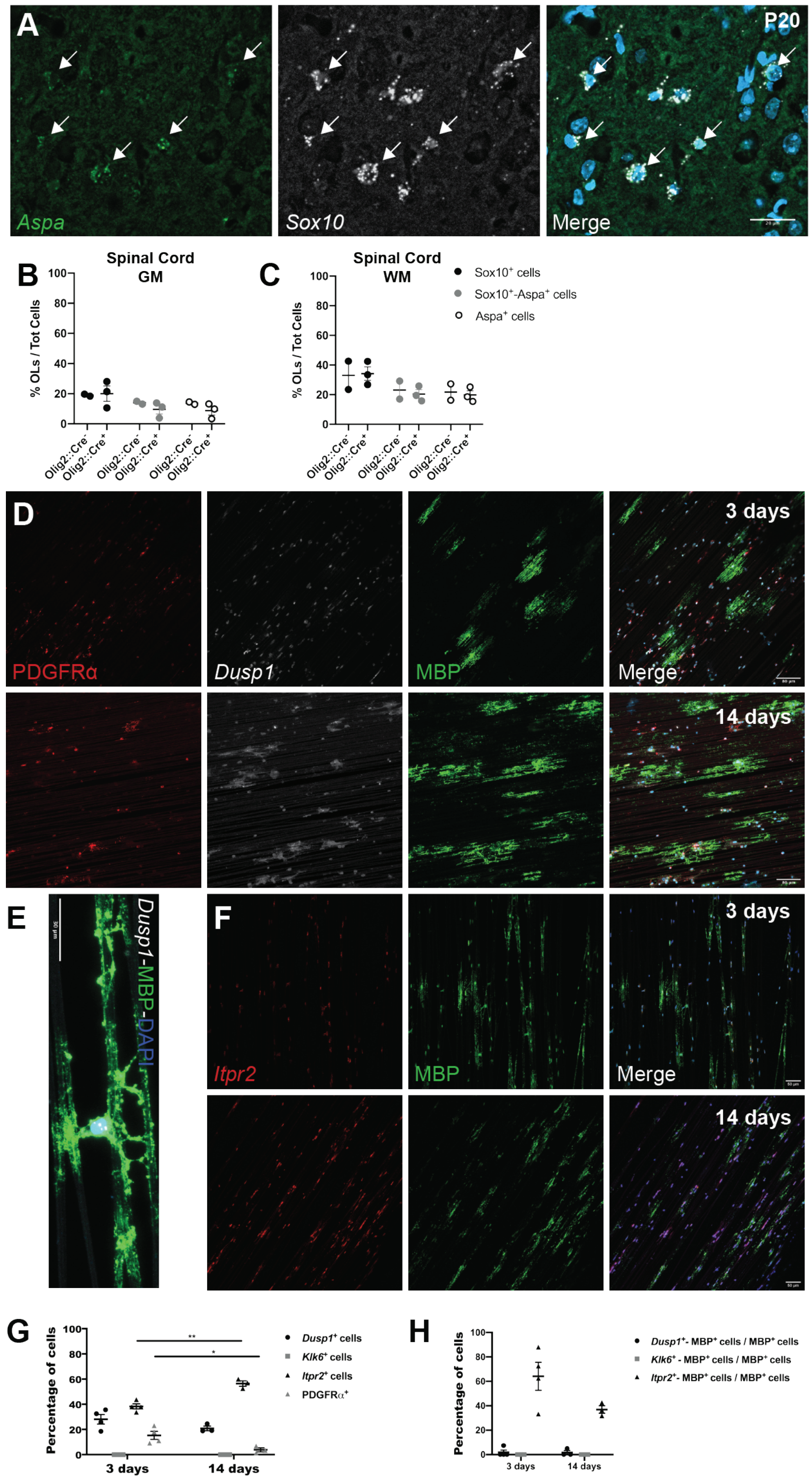

Supplementary Fig.6. Aspa is a specific marker to label mature oligodendrocytes. (**A**) Confocal representative images show that Aspa^{+} cells express Sox10 (a pan-marker for the OL lineage) in the juvenile (P20) grey matter of the spinal cord of the Olig2::Cre^{-}-TFEB$^{fl/fl}$ mice. Scale bar = 20 µm. (**B-C**) Quantification of the Sox10^{+}, Aspa^{+}-Sox10^{+}, and Aspa^{+} cells in the grey (B) and white (C) matter of the juvenile (Post-natal day (P) 20) spinal cord of Olig2::Cre^{-}-TFEB$^{fl/fl}$ and Olig2::Cre^{+}-TFEB$^{fl/fl}$ mice. Percentage of the population is calculated on the total number of nuclei (DAPI) in the analyzed region. Data are presented as Mean ± SEM. n = 2-3 animals per genotype, and can be assessed in the Supplementary Source Data file. Olig2::Cre^{-} = Olig2::Cre^{-}-TFEB$^{fl/fl}$, Olig2::Cre^{+} = Olig2::Cre^{+}-TFEB$^{fl/fl}$. (**D-E**) Confocal representative images show the PDGFRα^{+}, *Dusp1*$^{+}$ *Itpr2*$^{+}$ and MBP^{+} cells derived from brain OPCs cultured on microfibers for 3 or 14 days of differentiation in vitro. Scale bar = 80 µm. (**F**) Representative images of rare *Dusp1*$^{+}$MBP^{+} cell from brain OPCs 14 days *in vitro* with some processes enwrapping microfibers. (**G**) Quantification of the percentage of PDGFR-alpha^{+}, *Klk6*$^{+}$, *Dusp1*$^{+}$ and *Itpr2*$^{+}$ cells out of the total number of nuclei (DAPI^{+}). cultured on microfibers for 3 or 14 days of differentiation in vitro. (**H**) Quantification of the percentage of *Klk6*$^{+}$, *Dusp1*$^{+}$ and *Itpr2*$^{+}$ cells out of the MBP^{+} cells cultured on microfibers for 3 or 14 days of differentiation in vitro. n = 3-4 for G) and H), and can be assessed in the Source Data file. Each experiment was a biological replicate. Data are presented as Mean ± SEM. Asterisks indicate a significant difference between conditions for the individual markers (*p ≤ 0.05, **p ≤ 0.01, two-sided unpaired t-test). GM = grey matter, WM = white matter. Source data are provided as a Source Data file.

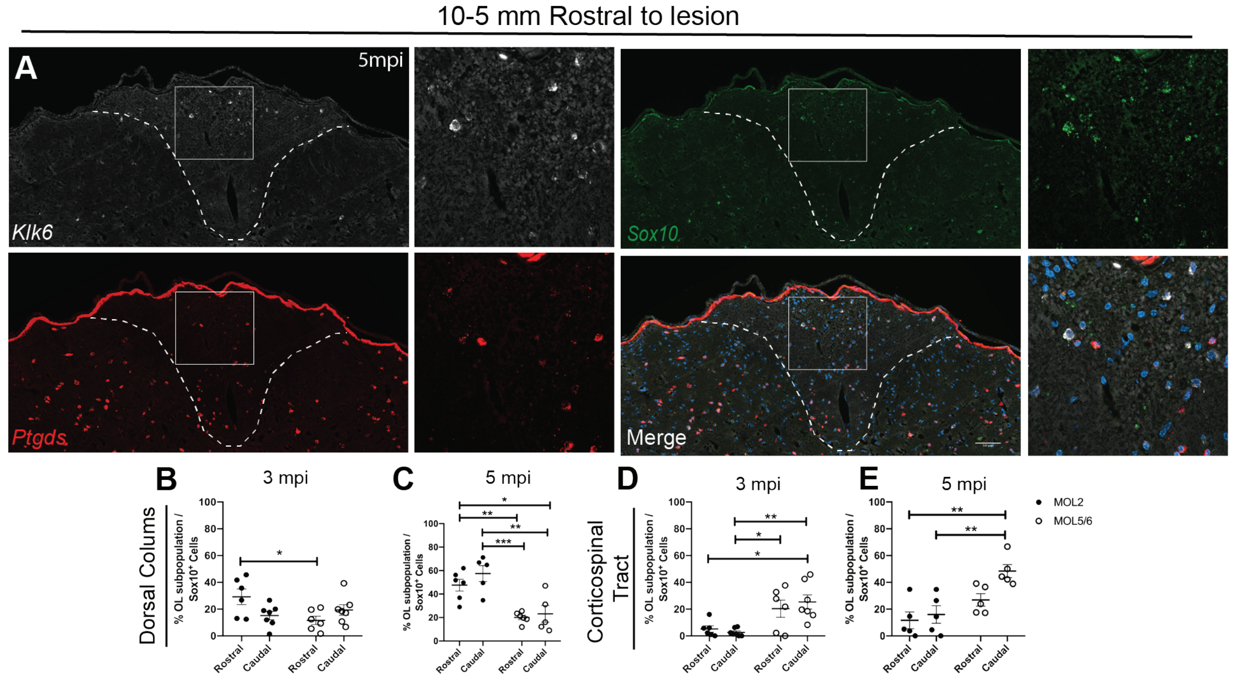

Supplementary Fig. 7. MOL2 and MOL5/6 populations maintain their spatial preference following traumatic spinal cord injury. (**A**) Confocal representative images of the dorsal spinal cord region 10-5 mm rostral to the injury site showing the maintained MOL2 (*Klk6*+ OL lineage cells) and MOL5/6 (*Ptgds*+ OL lineage cells) spatial preference 5 months post-injury. White dashed lines highlight the dorsal funiculi. Scale bar = 100 µm. (**B-E**) Percentages of the MOL2 (*Klk6*+ OL lineage cells) and MOL5/6 (*Ptgds*+ OL lineage cells) populations are calculated on the total number of OL lineage cells *(Sox10*+ cells) in the dorsal columns (B-C) and dorsal corticospinal tract (D-E) 5-10mm rostral and caudal to the injury site. Data are presented as Mean ± SEM. n = 5-7 animals per condition, and can be assessed in the Source Data file. Asterisks indicate a significant difference between conditions (*p ≤ 0.05, **p ≤ 0.01, ***p≤ 0.001, 2-way ANOVA with Sidak's correction). Exact p-values are reported in the Source Data file. mpi = months post-injury. Source data are provided as a Source Data file.

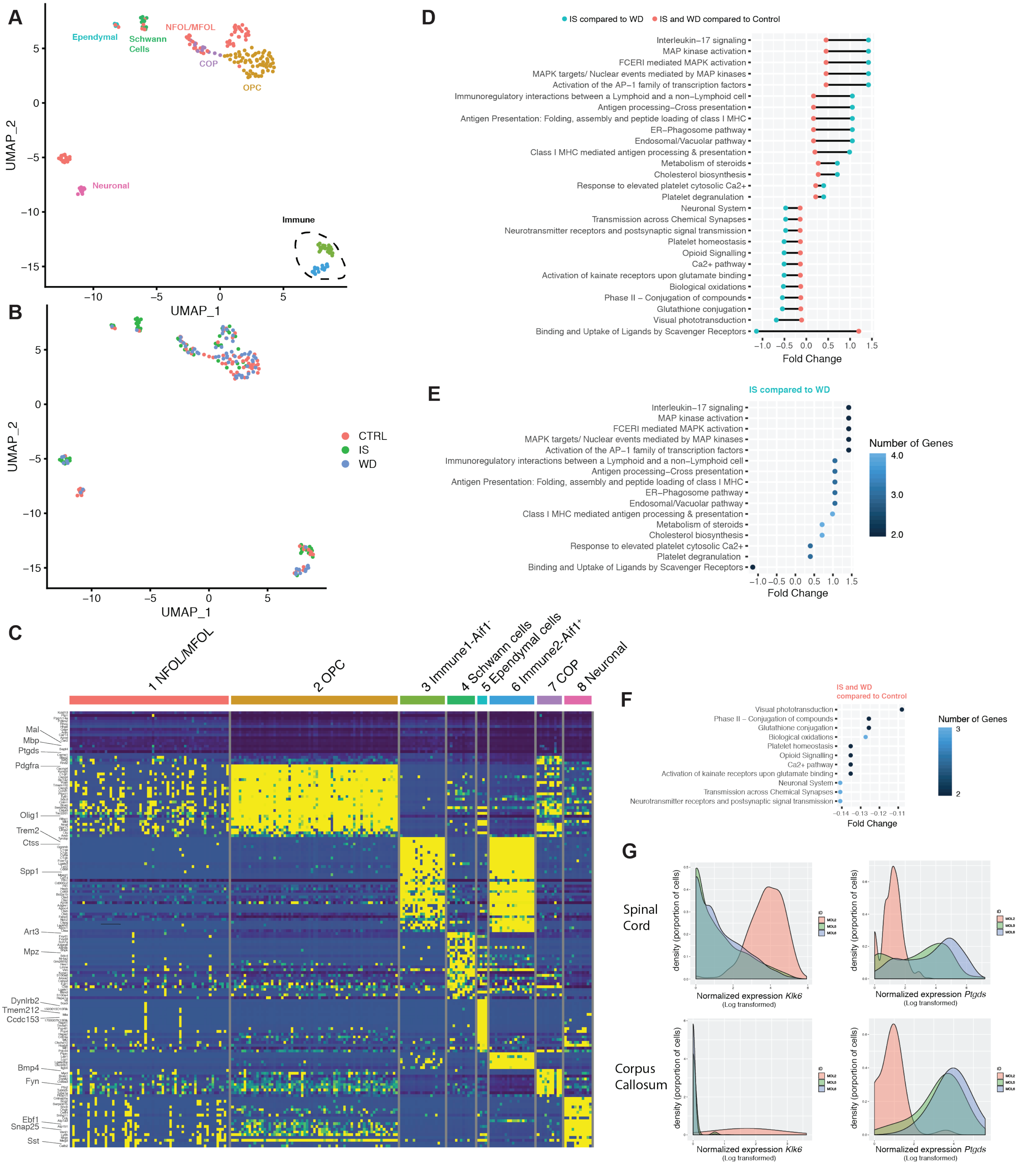

Supplementary Fig. 8. Louvain clustering analysis of the OPCs and non-OL lineage cells following spinal cord injury. (**A, B**) UMAP plots showing Sox10::Cre - GFP positive cells from laminectomy control, injury site, and Wallerian degeneration regions contributing to the clusters (A) and whether these cells were isolated from laminectomy control, injury site or Wallerian degenerati on regions (B), as determined by graph-based clustering (Seurat)[27,28] and integration cells with the other scRNA-Seq datasets[5,14]. (**C**) Heat-map of differential gene expression highlighting the enriched gene modules (left) characterizing the identified clusters (above) by Louvain clustering analysis, of OPCs and non-OL lineage cells in scRNA-seq data from spinal cord injury (Figure 6). Distance matrix = Spearmann correlation. (**D-F**) Cleveland plots illustrating the biological processes associated with differentially expressed genes in the OL lineage cells from the injury site compared to laminectomy control and Wallerian degeneration regions. (**G**) Density plots illustrating expression of *Klk6* and *Ptgds* in the MOL2, MOL5 and MOL6 populations in the spinal cord and corpus callosum single-cell RNA-seq data. CTRL = laminectomy control, IS = injury site, WD = Wallerian degeneration. OPC = oligodendrocyte progenitor cell, COP = committed OPC, NFOL = newly formed oligodendrocyte, MFOL = myelin forming oligodendrocyte, MOL = mature oligodendrocyte.

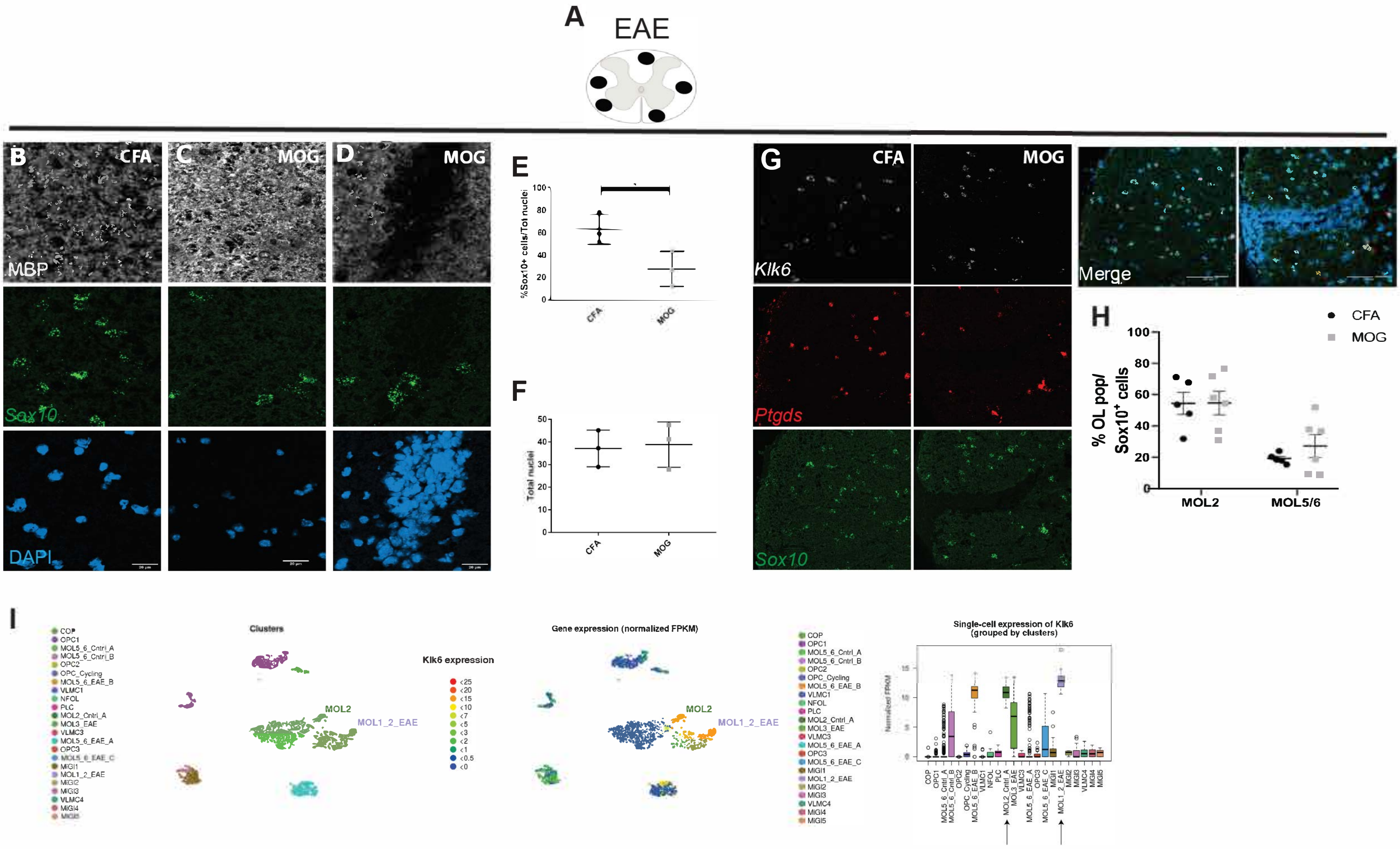

Supplementary Fig. 9. MOL2 and MOL5/6 populations respond to EAE in a similar manner. (A) Schematic of the lesion distribution in the Experimental Autoimmune encephalomyelitis (EAE) model. **(B-D, G)** Confocal representative images of the lateral white matter of the spinal cord showing the expression of MBP and distribution of Sox10• cells in vehicle control (CFA. B), non-lesione d (C), lesioned areas (D), and MOL2 and MOL.5/6 populations (G) in MOG-immunized mice. Scale bar= 20 (B-D) and 100 μm (G). **(E-F, H)** Percentages of the OL lineage cells *(Sox10'* cells. D), MOL2 *(Klk6+* OL lineage cells) and MOL.5/6 *(Ptgds+* OL lineage cells. **H),** and total nuclei number (E) in intact regions (CFA control) and peri-lesion areas of the lateral white matter. Data are presented as Mean ± SEM. n = 3-6 animals per condition and can be assessed in the Source Data file. Asterisks indicate a significant differenc e between conditions (*p < 0.05, 2-way ANOVA with Sidak's correction). Exact p-values are reported in the Source Data file. CFA = complete Freund's adjuvant, MOG = Myelin Oligodendrocyte Glycoprotein. **(I)** t-Distributed stochastic neighbor embedding (t-SNE) plots showing the OL lineage composition at the peak of disease in the EAE model, and high expression of *Klk6* in both ctrl and EAE-associated MOL2. Total number of cells is 794 for controls and 971 for EAE. Box plots include minimum, maximum, centre (median), and quartiles (01 and Q3). Data were previously published[7] and publicly searchable at https://castelobranco.shinyapps.io/E AE2017/. OPC = oligodendrocyte progenitor cell, COP = committed OPC, NFOL = newly formed oligodendrocyte, MFOL = myelin forming oligodendrocyte, MOL= mature oligodendrocyte. VLMC = vascular and leptomeningeal cell; MiGL = Microglia (or macrophages). FPKM = Fragments Per Kilobase of transcript per Million mapped reads. Source data are provided as a Source Data file.
