# Supplementary material for: Distinct oligodendrocyte populations have spatial preference and different responses to spinal cord injury
Source: Nat Commun. 2020 Nov 17;11:5860. doi: 10.1038/s41467-020-19453-x (PMC7673029; doi:10.1038/s41467-020-19453-x)
Supplement: Supplementary file 3 — Description of Additional Supplementary Files [file 41467_2020_19453_MOESM3_ESM.pdf]

Description of Additional Supplementary Files

Supplementary Data 1. Quantification of the OL subpopulations contribution to the OL lineage in the brain (corpus callosum and cortex) and spinal cord (grey and white matter) at P20 and P60, by ISS. n = 2 animals per stage and region**.**

Supplementary Data 2. Target sequences for padlock probe capturing arms for ISS.

Supplementary Data 3. Quantification of the OL subpopulations contribution to the OL lineage in the spinal cord. Percentage of the OL lineage cells (*Sox10*$^+$), MOL2 (*Klk6*$^+$ OL lineage cells), and MOL5/6 (*Ptgds*$^+$ OL lineage cells) contribution to the OL lineage (*Sox10*$^+$ cells) in the regions of interest in juvenile (P20) and adulthood (P60). We imaged 0.3, 0.13, and 0.4 mm2 of the sensorimotor cortex, corpus callosum, and dorsal spinal cord per tissue section, respectively. Minimum three sections per animal were analyzed. Data are presented as Mean ± SEM. n = 4-9 animals per condition, and can be assessed in the Source Data file.

Supplementary Data 4. Number of analyzed cells in P60 corpus callosum scRNAseq (Figure 3).

Supplementary Data 5. Differentially expressed genes in scRNAseq data from spinal cord injury, by MOL subpopulation and condition (spinal cord injury and Wallerian degeneration), using Wilcoxon rank sum test of all mature OL populations.

Supplementary Data 6. Signaling pathways upregulated in scRNAseq data from spinal cord injury, by MOL subpopulation and condition (spinal cord injury and Wallerian degeneration). Enrichment is calculated from a hypergeometric test.

Supplementary Data 7. Signaling pathways downregulated in scRNAseq data from spinal cord injury, by MOL subpopulation and condition (spinal cord injury and Wallerian degeneration). Enrichment is calculated from a hypergeometric test.
