# Supplementary material for: Distinct oligodendrocyte populations have spatial preference and different responses to spinal cord injury
Source: Nat Commun. 2020 Nov 17;11:5860. doi: 10.1038/s41467-020-19453-x (PMC7673029; doi:10.1038/s41467-020-19453-x)
Supplement: Supplementary file 8 — Supplementary Data5 [file 41467_2020_19453_MOESM8_ESM.pdf]

Info

Differential expression using Wilcoxon rank sum test of all mature OL populations

IS	Injury Site
WD	Wallerian Degeneration
Control	No Injury

All differential expression is limited to the mature oligodendrocyte populations.
Fig 6 e is based on information shown in the tables on the first two sheets.

Meaning of the columns

Column 1	Gene names
p_val	p-value calculated using wilcoxon rank sum test
avg_logFC	Log Fold change of the tested group
pct.1	Percent of cells expressing a given gene (refering to the first mention condition)
pct.2	Percent of cells expressing a given gene (refering to the second mention condition)
p_val_adj	FDR adjusted p-value

	p_val	avg_logFC	pct.1	pct.2	p_val_adj
B2m	1,35E-85	1,316538	0,949	0,462	2,13E-81
Aplp1	5,54E-84	-0,42917	1	1	8,74E-80
Fos	1,22E-74	2,051599	0,905	0,403	1,92E-70
H2-D1	7,84E-68	1,288385	0,917	0,538	1,24E-63
Egr1	2,27E-64	2,302329	0,58	0,076	3,58E-60
Gng11	1,5E-62	-0,80159	0,949	0,985	2,37E-58
Etv1	1,88E-58	-0,69559	0,914	0,975	2,97E-54
Ppp1r14a	3,9E-58	-0,53524	0,985	0,996	6,16E-54
Junb	7,49E-53	1,440736	0,679	0,234	1,18E-48
S100a16	1,08E-50	-0,59729	0,95	0,989	1,71E-46
AY036118	1,16E-50	0,61499	0,998	0,901	1,83E-46
Gpc1	1,68E-47	0,644288	0,778	0,392	2,65E-43
Gstm7	4,97E-47	-0,63021	0,829	0,932	7,85E-43
Epb41l3	2,95E-45	-0,49267	0,916	0,981	4,65E-41
Mag	2,49E-44	-0,33759	0,998	0,998	3,92E-40
C4b	5,49E-44	0,656461	0,998	0,679	8,66E-40
Apoe	4,38E-43	-1,19405	0,95	0,975	6,91E-39
Shisa4	5,62E-43	-0,53093	0,963	0,985	8,86E-39
Mt1	7,73E-43	0,913011	0,974	0,884	1,22E-38
Gstm5	1,42E-42	-0,55791	0,921	0,979	2,24E-38
Prdx1	1,44E-42	-0,41059	1	0,998	2,27E-38
Tfrc	1,97E-42	-0,4076	0,182	0,616	3,1E-38
Pdlim2	5,67E-42	-0,57112	0,949	0,992	8,95E-38
Hmgcs1	6,46E-42	1,002654	0,969	0,901	1,02E-37
Dhcr24	1,5E-41	0,757997	0,734	0,403	2,36E-37
Tmsb4x	2,82E-41	-0,42292	1	0,998	4,45E-37
Spock3	3,99E-41	-0,64991	0,793	0,901	6,29E-37
Nipa1	8,77E-41	-0,44135	0,873	0,966	1,38E-36
Pex5l	3,67E-40	-0,65982	0,954	0,983	5,79E-36
Fez1	3,77E-40	-0,39361	0,998	0,998	5,94E-36
Stmn1	4,17E-40	-0,37432	1	0,996	6,57E-36
Car2	1,01E-39	-0,39932	1	1	1,59E-35
Sept7	1,77E-39	-0,43243	0,998	0,998	2,79E-35
Bcas1	8,34E-39	0,6361	0,985	0,958	1,32E-34
Fth1	1,13E-38	-0,34139	1	1	1,78E-34
Cox6c	1,65E-38	-0,3622	0,987	0,996	2,6E-34
Tulp4	2,29E-38	0,414611	1	0,998	3,61E-34
Bmyc	9,37E-38	-0,51756	0,538	0,831	1,48E-33
Psat1	1,75E-37	-0,43398	0,95	0,983	2,77E-33
Cisd1	1,87E-37	-0,42041	0,818	0,954	2,95E-33
Ywhaq	1,89E-37	-0,33786	1	0,998	2,98E-33
Rida	2,65E-37	-0,48886	0,288	0,675	4,18E-33
Atg4c	9,69E-37	-0,42006	0,248	0,633	1,53E-32
H2-K1	1,36E-36	0,894032	0,659	0,329	2,14E-32
Mif	1,43E-36	-0,53765	0,839	0,935	2,25E-32
Nap1l5	1,84E-36	-0,5428	0,774	0,922	2,91E-32
Cyp2j6	5,24E-36	-0,46209	0,127	0,479	8,26E-32
Tmeff1	1,04E-35	-0,47199	0,884	0,96	1,64E-31
Ndufa4	1,19E-35	-0,37524	0,961	0,985	1,88E-31
Grhpr	1,6E-35	-0,48985	0,477	0,795	2,53E-31
Snrpn	2,92E-35	-0,49061	0,67	0,88	4,6E-31

Fis1	7,88E-35	-0,35198	0,987	0,985	1,24E-30
Ankub1	1,82E-34	-0,4251	0,185	0,553	2,87E-30
Glo1	2,81E-34	-0,42907	0,774	0,924	4,43E-30
Bpgm	5,54E-34	-0,36731	0,956	0,994	8,74E-30
Ncam1	7,54E-34	0,346163	1	0,998	1,19E-29
Psap	1,69E-33	0,328534	1	0,998	2,67E-29
Tmem258	1,72E-33	-0,48488	0,868	0,939	2,71E-29
Eml1	1,82E-33	-0,58699	0,563	0,808	2,88E-29
Jun	2,46E-33	1,201086	0,947	0,814	3,87E-29
Calm1	2,49E-33	-0,32878	0,998	0,998	3,93E-29
Mpc1	1,88E-32	-0,35837	0,978	0,987	2,97E-28
Ldlr	2,45E-32	0,87837	0,576	0,274	3,87E-28
Brd2	3,1E-32	0,574949	0,888	0,757	4,89E-28
Aspa	7,13E-32	-0,35228	0,974	0,994	1,12E-27
Gab1	8,17E-32	0,415996	0,98	0,949	1,29E-27
Chchd2	8,98E-32	-0,29595	0,998	0,998	1,42E-27
Apod	9,19E-32	-0,57425	1	0,996	1,45E-27
Hint1	1,75E-31	-0,36086	0,963	0,979	2,76E-27
Sept4	2,51E-31	-0,3794	1	0,996	3,96E-27
Gpm6b	4,29E-31	-0,27775	1	1	6,77E-27
Gfap	7,29E-31	-0,35871	0,075	0,371	1,15E-26
Mog	7,87E-31	-0,29993	0,996	0,998	1,24E-26
Vmp1	1,12E-30	-0,35602	0,971	0,989	1,77E-26
Ctsk	1,9E-30	-0,37331	0,207	0,553	3E-26
Kctd13	2,19E-30	-0,36847	0,971	0,989	3,46E-26
Fosb	3,55E-30	1,187103	0,382	0,082	5,6E-26
Selenof	4,39E-30	-0,304	0,969	0,973	6,93E-26
Ppia	6,59E-30	-0,29928	1	1	1,04E-25
Pkp4	8,34E-30	0,478827	0,917	0,743	1,32E-25
Fam102a	1,16E-29	0,409564	0,965	0,878	1,83E-25
Sdf2l1	1,74E-29	-0,36804	0,22	0,559	2,74E-25
Trf	2,01E-29	-0,35352	1	1	3,17E-25
Aldoc	2,21E-29	-0,66007	0,132	0,441	3,48E-25
Oaz1	3,97E-29	-0,31231	0,991	0,989	6,26E-25
Arhgap23	4,82E-29	0,366525	0,993	0,958	7,61E-25
Scarb2	7,87E-29	0,367772	1	0,983	1,24E-24
Egr2	8,27E-29	0,890041	0,279	0,023	1,3E-24
Agpat4	8,93E-29	-0,31295	0,987	0,998	1,41E-24
Cyp27a1	1,03E-28	-0,4453	0,273	0,603	1,63E-24
Srebf2	1,14E-28	0,442227	0,897	0,781	1,8E-24
Sptbn1	1,29E-28	0,394144	0,985	0,928	2,03E-24
Gsn	1,94E-28	-0,65144	0,954	0,949	3,06E-24
Plekhb1	1,96E-28	0,392221	1	0,996	3,1E-24
Socs3	2,27E-28	0,765137	0,426	0,127	3,59E-24
Sparc	3,58E-28	0,493102	0,943	0,73	5,65E-24
Klhl4	4,47E-28	-0,40261	0,549	0,819	7,05E-24
Atp5f1	6,19E-28	-0,33611	0,919	0,97	9,76E-24
Cfl1	6,51E-28	-0,24513	1	0,998	1,03E-23
Erbin	6,95E-28	0,460188	0,985	0,939	1,1E-23
Tmem125	7,48E-28	-0,39192	0,717	0,905	1,18E-23
Tcf4	7,91E-28	0,341555	0,998	0,992	1,25E-23
Spcs1	1,62E-27	-0,35064	0,868	0,93	2,55E-23

Inf2	2,03E-27	0,384083	0,991	0,941	3,2E-23
Tspan7	2,23E-27	-0,37725	0,259	0,576	3,52E-23
Mid1ip1	3,04E-27	-0,42946	0,941	0,968	4,8E-23
Omg	3,2E-27	-0,42873	0,666	0,865	5,05E-23
Cd81	3,87E-27	-0,31255	1	0,998	6,1E-23
Uqcr10	4,5E-27	-0,3066	0,938	0,973	7,09E-23
Serpind1	5,23E-27	-0,46319	0,083	0,354	8,25E-23
Ier2	6,47E-27	0,880627	0,312	0,051	1,02E-22
Paip2	1,09E-26	-0,28933	0,939	0,985	1,71E-22
Serf2	1,75E-26	-0,26677	0,994	0,994	2,75E-22
Fam171b	4,82E-26	-0,35232	0,93	0,981	7,61E-22
Mt2	6,07E-26	0,763622	0,4	0,122	9,57E-22
Ndrg2	7,95E-26	-0,36604	0,128	0,42	1,25E-21
Itih3	1,16E-25	-0,34712	0,088	0,357	1,83E-21
Dusp15	1,16E-25	-0,4558	0,437	0,728	1,84E-21
Qdpr	1,42E-25	-0,47805	0,993	0,987	2,25E-21
Atf4	1,63E-25	0,507464	0,829	0,648	2,57E-21
Itm2b	1,95E-25	-0,24332	1	0,998	3,07E-21
Abca2	2,05E-25	0,295968	0,998	0,998	3,24E-21
Sqle	2,2E-25	0,601407	0,761	0,551	3,47E-21
Agmo	2,64E-25	-0,36631	0,196	0,506	4,17E-21
Ptms	2,88E-25	0,452172	0,903	0,749	4,54E-21
Gatm	3,84E-25	-0,24826	1	0,998	6,06E-21
Atxn7l3b	4,33E-25	-0,28598	0,967	0,992	6,83E-21
Hnrnph1	5,64E-25	0,423516	0,899	0,772	8,9E-21
Cox5b	6,99E-25	-0,28542	0,965	0,981	1,1E-20
Eif1	7,97E-25	-0,24867	1	1	1,26E-20
Dnah14	1,24E-24	-0,37507	0,105	0,376	1,96E-20
Hist1h2bc	1,31E-24	-0,41047	0,807	0,922	2,06E-20
Rnf130	1,31E-24	-0,29214	0,963	0,994	2,07E-20
Mmd2	1,33E-24	-0,39893	0,145	0,439	2,1E-20
Cox7c	1,38E-24	-0,2799	0,998	0,998	2,18E-20
Cryab	1,48E-24	-0,29505	1	0,998	2,33E-20
Asrgl1	1,54E-24	-0,41088	0,594	0,793	2,43E-20
Pak7	1,64E-24	-0,38906	0,497	0,781	2,59E-20
Nkd1	1,86E-24	-0,33209	0,413	0,722	2,93E-20
Cystm1	1,87E-24	-0,30476	0,954	0,977	2,96E-20
Klf6	2,13E-24	0,759942	0,717	0,492	3,35E-20
Mrps21	2,52E-24	-0,31482	0,583	0,823	3,97E-20
Serpinb1a	3,45E-24	-0,51119	0,895	0,914	5,44E-20
Purb	3,86E-24	0,413202	0,949	0,848	6,09E-20
Efcab14	4,19E-24	-0,38861	0,982	0,983	6,61E-20
Cox5a	4,66E-24	-0,32767	0,736	0,911	7,35E-20
Ndufa3	5,71E-24	-0,27174	0,976	0,994	9,01E-20
Slc44a1	6,08E-24	0,273643	1	0,998	9,58E-20
Btg2	6,38E-24	1,053577	0,431	0,165	1,01E-19
Cd63	7,39E-24	0,42811	0,989	0,869	1,17E-19
Msmo1	9,37E-24	0,622421	0,927	0,766	1,48E-19
Gjc2	1,03E-23	-0,32875	0,95	0,992	1,63E-19
BC005537	1,04E-23	0,426387	0,892	0,772	1,65E-19
Timm8b	1,26E-23	-0,33398	0,618	0,838	1,98E-19
Higd1a	1,55E-23	-0,33433	0,554	0,823	2,45E-19

Npc1	1,97E-23	0,309906	1	0,981	3,11E-19
Epdr1	2,14E-23	-0,32021	0,275	0,593	3,37E-19
Lyz2	2,36E-23	-0,21698	0,05	0,281	3,72E-19
Jmjd1c	4,16E-23	0,558522	0,778	0,591	6,56E-19
Ndufb4	4,73E-23	-0,33204	0,855	0,926	7,46E-19
Ubl5	5,44E-23	-0,26495	0,93	0,975	8,58E-19
Cdk19	6,75E-23	0,371116	0,93	0,829	1,06E-18
Taldo1	8,25E-23	-0,27427	1	0,998	1,3E-18
Slc25a27	8,74E-23	-0,50447	0,284	0,557	1,38E-18
Bin1	1,09E-22	-0,33849	0,994	1	1,71E-18
Pon2	1,56E-22	-0,3456	0,47	0,736	2,47E-18
Ypel3	1,94E-22	-0,30497	0,866	0,956	3,06E-18
Rnf13	2,33E-22	-0,21865	0,998	0,998	3,68E-18
Mthfsl	2,44E-22	-0,29101	0,305	0,614	3,85E-18
1700063D(	2,45E-22	-0,40398	0,106	0,354	3,87E-18
Efhd1	2,62E-22	-0,35669	0,844	0,922	4,14E-18
Ctnnbip1	2,85E-22	-0,36525	0,637	0,838	4,5E-18
March1	3,54E-22	-0,34052	0,182	0,458	5,59E-18
Acyp2	3,8E-22	-0,34467	0,488	0,747	5,99E-18
Usmg5	3,85E-22	-0,29158	0,804	0,92	6,07E-18
Zeb2	4,31E-22	0,303082	1	0,998	6,81E-18
1810022K(	4,44E-22	-0,29723	0,494	0,776	7E-18
Ndufb9	4,85E-22	-0,2642	0,945	0,979	7,65E-18
Fam53b	4,97E-22	0,388546	0,914	0,8	7,84E-18
Smdt1	5,24E-22	-0,25536	0,976	0,987	8,27E-18
Fnta	5,43E-22	-0,34822	0,772	0,905	8,56E-18
Gde1	5,81E-22	-0,36364	0,89	0,949	9,17E-18
Tvp23b	9,54E-22	-0,28251	0,301	0,614	1,51E-17
Hspa8	9,97E-22	-0,22174	1	0,998	1,57E-17
Srp14	1,19E-21	-0,26565	0,963	0,979	1,87E-17
Inpp5f	1,27E-21	-0,33794	0,824	0,949	2,01E-17
Gm4524	1,44E-21	-0,20702	0,026	0,215	2,27E-17
B230118H(	1,71E-21	-0,30091	0,503	0,772	2,69E-17
2010107E(	1,75E-21	-0,31871	0,783	0,901	2,76E-17
Slc25a4	1,8E-21	-0,24645	0,994	0,998	2,84E-17
BC031181	2,38E-21	-0,28968	0,877	0,954	3,76E-17
Gstm1	2,45E-21	-0,43273	0,538	0,776	3,86E-17
Dusp1	2,7E-21	0,861229	0,45	0,203	4,26E-17
Mettl7a1	2,95E-21	-0,32485	0,549	0,81	4,65E-17
Depdc7	4,18E-21	-0,27836	0,161	0,432	6,6E-17
Slu7	4,46E-21	-0,39146	0,73	0,861	7,03E-17
Vdac2	5E-21	-0,28781	0,91	0,958	7,89E-17
Nudt19	5,42E-21	-0,26408	0,217	0,506	8,55E-17
Cpox	8,5E-21	-0,38892	0,974	0,973	1,34E-16
Pde6d	2,35E-20	-0,2924	0,633	0,846	3,71E-16
Zbtb20	2,58E-20	0,351286	0,996	0,985	4,07E-16
Ostc	3,72E-20	-0,27431	0,365	0,652	5,87E-16
1810037I1	4,35E-20	-0,26017	0,978	0,989	6,86E-16
Iscu	4,81E-20	-0,27311	0,325	0,618	7,59E-16
Jam3	7,09E-20	-0,26631	0,947	0,97	1,12E-15
Ndufa7	7,51E-20	-0,25244	0,912	0,966	1,18E-15
Adk	9,32E-20	-0,32691	0,506	0,764	1,47E-15

Cdc37l1	9,58E-20	-0,21433	0,996	0,998	1,51E-15
Adh5	9,96E-20	-0,27976	0,769	0,926	1,57E-15
Borcs5	1,43E-19	-0,2505	0,222	0,496	2,26E-15
Ptpre	1,99E-19	-0,28809	0,209	0,487	3,14E-15
Ddx3x	1,99E-19	0,419492	0,883	0,745	3,14E-15
Pex2	2,16E-19	-0,27926	0,545	0,797	3,41E-15
Gstp1	2,2E-19	0,56569	0,987	0,981	3,48E-15
Pin4	2,21E-19	-0,29431	0,556	0,768	3,49E-15
Ndufab1	2,28E-19	-0,28679	0,772	0,909	3,6E-15
Thsd7a	2,73E-19	-0,31079	0,185	0,451	4,31E-15
mt-Co1	3,56E-19	-0,19704	1	1	5,61E-15
Nudcd2	4,25E-19	-0,25191	0,226	0,498	6,71E-15
Cyb5a	4,42E-19	-0,30594	0,826	0,928	6,97E-15
Cycs	4,56E-19	-0,29253	0,646	0,844	7,19E-15
Il18	4,87E-19	-0,28648	0,462	0,728	7,68E-15
Idi1	5,03E-19	0,620959	0,802	0,627	7,94E-15
Cox4i1	5,36E-19	-0,19254	1	0,998	8,46E-15
Guk1	5,39E-19	-0,30619	0,523	0,766	8,5E-15
Dnajc15	5,7E-19	-0,27687	0,354	0,639	9E-15
1500011B(	5,78E-19	-0,27428	0,261	0,53	9,12E-15
Kif5b	6,25E-19	0,347913	0,963	0,873	9,86E-15
Golga7	8,18E-19	-0,26984	0,956	0,983	1,29E-14
Nat8f1	8,7E-19	-0,25517	0,156	0,403	1,37E-14
Insig1	9,01E-19	0,522087	0,87	0,711	1,42E-14
Plekhj1	9,02E-19	-0,29419	0,444	0,717	1,42E-14
Ndufaf2	1,04E-18	-0,27787	0,428	0,679	1,64E-14
Swi5	1,1E-18	-0,25277	0,921	0,954	1,73E-14
Fdft1	1,16E-18	0,448984	0,738	0,565	1,84E-14
Evi2a	1,19E-18	-0,26277	0,974	0,983	1,87E-14
Cntn2	1,26E-18	0,265018	0,996	0,994	1,99E-14
Mbp	1,32E-18	0,23148	1	0,998	2,09E-14
Psph	1,54E-18	-0,28706	0,385	0,665	2,43E-14
Lbh	1,55E-18	0,345118	0,956	0,797	2,45E-14
Efnb3	1,64E-18	-0,21299	0,996	0,998	2,59E-14
Ndufa8	1,66E-18	-0,25531	0,598	0,835	2,61E-14
Snx10	1,66E-18	0,382488	0,446	0,192	2,62E-14
Hcn2	1,71E-18	-0,46939	0,881	0,924	2,7E-14
Ywhaz	2,1E-18	0,297925	0,983	0,962	3,32E-14
3110021N:	2,11E-18	-0,2594	0,217	0,477	3,32E-14
Sft2d1	2,21E-18	-0,29082	0,706	0,85	3,48E-14
Atf3	2,28E-18	0,723959	0,235	0,044	3,6E-14
Tra2b	2,3E-18	0,386069	0,872	0,772	3,63E-14
Tubb4a	2,4E-18	-0,24264	1	0,998	3,79E-14
Kctd4	2,42E-18	-0,30491	0,332	0,612	3,82E-14
Mpc2	2,51E-18	-0,24543	0,853	0,93	3,97E-14
Rab3a	2,65E-18	-0,27743	0,4	0,667	4,19E-14
Vta1	2,72E-18	-0,29602	0,552	0,778	4,29E-14
Mcee	2,97E-18	-0,30382	0,477	0,726	4,69E-14
Gkap1	3,29E-18	-0,32619	0,756	0,895	5,19E-14
Aatk	3,55E-18	0,248304	0,996	0,996	5,6E-14
Khdrbs3	3,56E-18	-0,24433	0,15	0,392	5,61E-14
Tex52	3,8E-18	-0,24307	0,141	0,376	5,99E-14

Ndufc1	3,89E-18	-0,28332	0,811	0,914	6,13E-14
Zfp36l2	4,15E-18	0,472967	0,662	0,479	6,55E-14
Lamtor5	4,34E-18	-0,25879	0,565	0,81	6,85E-14
Abhd12	4,52E-18	-0,32386	0,806	0,886	7,14E-14
Mt3	4,63E-18	-0,24754	0,084	0,295	7,31E-14
Reep3	4,89E-18	0,267196	0,993	0,989	7,72E-14
Tmsb10	5,06E-18	0,519181	0,71	0,504	7,98E-14
Clic4	5,55E-18	0,277413	0,996	0,975	8,75E-14
Endod1	5,75E-18	0,273322	0,994	0,977	9,08E-14
Atp5k	6,61E-18	-0,23354	0,967	0,992	1,04E-13
Mrps33	7,02E-18	-0,29149	0,736	0,888	1,11E-13
Edil3	7,26E-18	0,224608	1	0,996	1,15E-13
Auh	7,36E-18	-0,2822	0,6	0,804	1,16E-13
Uqcrb	8,24E-18	-0,26627	0,772	0,899	1,3E-13
Adgrl3	8,34E-18	-0,24606	0,094	0,31	1,32E-13
Stard3nl	8,44E-18	-0,29448	0,6	0,821	1,33E-13
Glod4	8,48E-18	-0,29784	0,721	0,876	1,34E-13
Rap2a	9,01E-18	-0,30781	0,316	0,593	1,42E-13
Rpl13a	9,22E-18	0,344862	0,96	0,905	1,46E-13
Bola3	9,27E-18	-0,21828	0,235	0,5	1,46E-13
Ndufb3	9,31E-18	-0,27131	0,782	0,899	1,47E-13
Sys1	1,02E-17	-0,35054	0,905	0,935	1,62E-13
Fam96a	1,18E-17	-0,24402	0,284	0,551	1,86E-13
Fam177a	1,29E-17	-0,23328	0,119	0,34	2,04E-13
Psen2	1,49E-17	-0,29554	0,294	0,555	2,35E-13
Ppdpf	1,72E-17	-0,29978	0,53	0,751	2,71E-13
Papss1	1,76E-17	-0,25658	0,914	0,975	2,78E-13
Uqcrq	1,9E-17	-0,22388	0,947	0,981	2,99E-13
Pin1	1,91E-17	-0,25838	0,49	0,755	3,02E-13
Uqcr11	2,01E-17	-0,22315	0,947	0,975	3,17E-13
Iqsec1	2,04E-17	0,364327	0,936	0,838	3,22E-13
Selenoh	2,13E-17	-0,25504	0,25	0,504	3,36E-13
Ubc	2,21E-17	0,443305	1	0,996	3,49E-13
Col6a1	2,35E-17	0,39105	0,189	0,021	3,7E-13
Ech1	2,54E-17	-0,26834	0,743	0,886	4E-13
Btf3	2,79E-17	-0,20581	0,993	0,994	4,4E-13
Trappc6a	3,06E-17	-0,23903	0,191	0,435	4,83E-13
Atp10b	3,07E-17	-0,23062	0,13	0,357	4,84E-13
Plekhh2	3,36E-17	-0,228	0,136	0,369	5,3E-13
Cmtm5	3,73E-17	-0,22037	1	0,998	5,89E-13
Gnai1	3,74E-17	-0,26282	0,976	0,981	5,89E-13
Tspan3	3,9E-17	-0,22474	0,95	0,987	6,15E-13
Emc6	4,58E-17	-0,24692	0,422	0,696	7,22E-13
Pfdn4	4,62E-17	-0,21834	0,24	0,5	7,29E-13
Sppl2b	5,78E-17	-0,25234	0,169	0,407	9,12E-13
Gabarapl2	6,72E-17	-0,22302	0,967	0,977	1,06E-12
Map4k4	8,52E-17	0,266877	0,989	0,987	1,34E-12
Hopx	8,9E-17	-0,79193	0,576	0,724	1,4E-12
Tsc22d4	1,02E-16	-0,23946	0,998	0,998	1,61E-12
Hmgn2	1,03E-16	-0,28895	0,8	0,895	1,63E-12
Sik3	1,33E-16	0,303498	0,954	0,861	2,09E-12
Rab4a	1,36E-16	-0,29189	0,352	0,599	2,14E-12

Kif21a	1,42E-16	-0,25683	0,934	0,977	2,24E-12
Ndufb8	1,49E-16	-0,23354	0,89	0,945	2,35E-12
Gadd45g	1,51E-16	0,71445	0,233	0,051	2,39E-12
Timm8a1	1,63E-16	-0,23175	0,25	0,504	2,58E-12
Atp5c1	1,89E-16	-0,23136	0,923	0,958	2,97E-12
Cyp2j12	2,28E-16	-0,32508	0,279	0,515	3,6E-12
Wfdc18	2,34E-16	-0,32574	0,106	0,31	3,7E-12
Fbxo2	2,62E-16	-0,36861	0,343	0,576	4,13E-12
Klf13	2,7E-16	0,319109	0,993	0,992	4,26E-12
Atp5e	2,7E-16	-0,2099	0,969	0,987	4,26E-12
Rtn3	2,76E-16	-0,18654	0,998	0,996	4,36E-12
Mrpl34	2,87E-16	-0,2419	0,415	0,673	4,53E-12
Ddt	2,88E-16	-0,23904	0,409	0,681	4,54E-12
Rpa3	3,02E-16	-0,22465	0,198	0,439	4,76E-12
Eloc	3,04E-16	-0,23574	0,78	0,907	4,79E-12
Fam174a	3,05E-16	-0,25932	0,611	0,825	4,81E-12
Gipc1	3,38E-16	-0,30031	0,367	0,61	5,33E-12
Grb14	3,4E-16	-0,39577	0,945	0,954	5,37E-12
Chchd1	3,51E-16	-0,23747	0,583	0,819	5,54E-12
Gjb1	3,59E-16	-0,25872	0,983	0,97	5,67E-12
Glrb	3,6E-16	-0,28644	0,534	0,753	5,67E-12
Rsrp1	4,08E-16	-0,18644	0,998	0,998	6,44E-12
Coq2	4,08E-16	-0,2153	0,266	0,534	6,44E-12
Atp5j2	4,11E-16	-0,20162	0,965	0,983	6,48E-12
A230001M	4,15E-16	-0,26685	0,273	0,523	6,54E-12
Ldhb	4,22E-16	-0,53088	0,563	0,715	6,65E-12
Tuba1a	4,22E-16	-0,3069	1	0,998	6,66E-12
Srcin1	4,46E-16	0,310715	0,978	0,951	7,03E-12
Col6a2	4,52E-16	0,394831	0,222	0,044	7,13E-12
Aes	4,79E-16	-0,20611	0,985	0,994	7,56E-12
Prr5l	5E-16	-0,25751	0,895	0,96	7,89E-12
Gm42418	5,14E-16	0,245796	1	1	8,1E-12
Pdk2	5,18E-16	-0,29173	0,283	0,527	8,17E-12
Aig1	5,6E-16	-0,2195	0,912	0,958	8,83E-12
Phldb1	5,99E-16	0,287553	0,994	0,964	9,44E-12
Ptch1	6,54E-16	-0,27385	0,321	0,576	1,03E-11
Smim26	6,82E-16	-0,24246	0,549	0,787	1,08E-11
Bet1	7,28E-16	-0,26838	0,587	0,795	1,15E-11
Lmna	7,55E-16	0,368309	0,809	0,629	1,19E-11
Anks1b	7,71E-16	0,293516	0,969	0,876	1,22E-11
Hsd17b10	7,75E-16	-0,2842	0,497	0,707	1,22E-11
Nudt2	7,82E-16	-0,20511	0,207	0,451	1,23E-11
Pabpc1	8,04E-16	0,305419	0,91	0,816	1,27E-11
Ndufa11	8,91E-16	-0,25823	0,806	0,888	1,41E-11
2900097C:	8,94E-16	0,332912	0,862	0,778	1,41E-11
Ndufc2	1,01E-15	-0,24758	0,881	0,93	1,59E-11
Cox8a	1,07E-15	-0,20066	0,993	0,992	1,69E-11
Tma7	1,07E-15	-0,26282	0,785	0,89	1,69E-11
Masp1	1,12E-15	-0,28344	0,158	0,378	1,76E-11
Ndrg1	1,18E-15	0,210652	1	0,998	1,86E-11
Gca	1,18E-15	-0,2194	0,228	0,473	1,87E-11
Lars2	1,2E-15	0,343694	0,919	0,85	1,89E-11

Dnajc24	1,23E-15	-0,2348	0,484	0,736	1,94E-11
Sirt2	1,27E-15	-0,1846	1	0,998	2E-11
Sar1b	1,28E-15	-0,24625	0,571	0,776	2,02E-11
Sec62	1,34E-15	-0,20338	0,996	1	2,12E-11
Chchd3	1,38E-15	-0,2386	0,372	0,631	2,18E-11
Tob2	1,56E-15	0,401404	0,743	0,591	2,47E-11
Atp5g2	1,64E-15	-0,21755	0,961	0,973	2,59E-11
Frmd8	1,69E-15	-0,26643	0,916	0,958	2,66E-11
Dock9	1,73E-15	-0,33955	0,699	0,835	2,73E-11
Ndufs7	1,8E-15	-0,20753	0,949	0,97	2,84E-11
Lrrc4b	1,82E-15	0,306836	0,868	0,762	2,87E-11
Snhg20	1,89E-15	-0,22839	0,29	0,553	2,99E-11
Pigp	1,95E-15	-0,2774	0,549	0,755	3,07E-11
Nop10	1,98E-15	-0,22971	0,431	0,688	3,13E-11
Pfn2	2,01E-15	-0,26732	0,681	0,831	3,18E-11
Acyp1	2,04E-15	-0,22006	0,213	0,447	3,21E-11
Sparcl1	2,1E-15	-0,18955	0,024	0,167	3,32E-11
Rassf4	2,17E-15	-0,24727	0,123	0,329	3,43E-11
Fam162a	2,24E-15	-0,22809	0,242	0,487	3,53E-11
Apopt1	2,26E-15	-0,24898	0,378	0,622	3,57E-11
Cdh10	2,27E-15	-0,24076	0,182	0,405	3,58E-11
Csmd3	2,34E-15	-0,2337	0,325	0,586	3,7E-11
Cacybp	2,37E-15	-0,26225	0,606	0,785	3,75E-11
Stard3	2,38E-15	-0,26941	0,473	0,709	3,75E-11
Tmprss5	2,44E-15	-0,27787	0,204	0,43	3,86E-11
Dad1	2,56E-15	-0,22468	0,947	0,97	4,03E-11
Mrps28	2,57E-15	-0,21529	0,218	0,458	4,05E-11
Prrt1	2,63E-15	-0,23616	0,499	0,753	4,15E-11
Sp3os	2,64E-15	-0,19087	0,149	0,376	4,17E-11
Bloc1s2	2,66E-15	-0,21405	0,31	0,568	4,2E-11
Tmem14a	2,73E-15	-0,24779	0,358	0,601	4,31E-11
Rasal2	3,06E-15	-0,24963	0,376	0,629	4,83E-11
Ciapin1	3,1E-15	-0,20539	0,25	0,502	4,89E-11
Ncam2	3,38E-15	-0,26224	0,585	0,806	5,33E-11
Robo1	3,43E-15	-0,22647	0,194	0,428	5,41E-11
Ermn	3,72E-15	-0,17083	1	0,998	5,86E-11
Rab31	3,83E-15	0,291934	0,965	0,895	6,04E-11
Cox7a2	4,15E-15	-0,20732	0,985	0,979	6,55E-11
Ptgr2	4,17E-15	-0,24468	0,308	0,553	6,57E-11
Zfp36	4,59E-15	0,828489	0,4	0,211	7,24E-11
Npas3	4,82E-15	-0,22763	0,13	0,335	7,61E-11
Arhgef12	5,02E-15	0,341656	0,848	0,671	7,92E-11
Sdf2	5,07E-15	-0,21062	0,433	0,705	8E-11
Igsf11	5,1E-15	-0,27877	0,604	0,783	8,05E-11
Ssu72	5,28E-15	-0,23681	0,519	0,768	8,32E-11
Mrpl43	5,36E-15	-0,22097	0,536	0,781	8,46E-11
Amn1	5,4E-15	-0,21915	0,281	0,546	8,53E-11
Atp6v1g1	5,43E-15	-0,22677	0,956	0,981	8,56E-11
Rtl8a	5,63E-15	-0,24058	0,872	0,937	8,88E-11
Sf3b5	5,99E-15	-0,22603	0,541	0,751	9,45E-11
Cox6a1	7,47E-15	-0,19993	0,958	0,985	1,18E-10
Usp50	7,63E-15	-0,19674	0,16	0,376	1,2E-10

Sbno2	7,92E-15	0,620043	0,466	0,268	1,25E-10
Selenok	8,16E-15	-0,18077	1	1	1,29E-10
Emc4	8,47E-15	-0,23545	0,488	0,728	1,34E-10
Iah1	1,02E-14	-0,22417	0,194	0,418	1,61E-10
Ss18l2	1,04E-14	-0,36765	0,699	0,831	1,64E-10
Sgk3	1,13E-14	-0,25399	0,279	0,508	1,79E-10
Pum2	1,14E-14	0,31175	0,824	0,715	1,79E-10
C1d	1,21E-14	-0,21496	0,426	0,681	1,91E-10
Cbr1	1,22E-14	-0,19548	0,167	0,386	1,92E-10
Klf4	1,22E-14	0,779882	0,262	0,084	1,92E-10
Cltc	1,3E-14	0,249886	0,969	0,907	2,05E-10
Acot13	1,3E-14	-0,23813	0,356	0,603	2,06E-10
Gramd3	1,32E-14	-0,22115	0,209	0,439	2,08E-10
Hccs	1,32E-14	-0,23065	0,361	0,624	2,09E-10
Pxmp2	1,34E-14	-0,16503	0,061	0,228	2,11E-10
Atp6v1f	1,5E-14	-0,21903	0,921	0,956	2,37E-10
Cox7b	1,51E-14	-0,26928	0,741	0,859	2,38E-10
Pno1	1,52E-14	-0,22398	0,218	0,443	2,4E-10
Ap2a1	1,55E-14	-0,30868	0,939	0,951	2,44E-10
Prr13	1,57E-14	-0,20883	0,295	0,549	2,48E-10
Cpeb2	1,78E-14	0,2829	0,866	0,764	2,8E-10
Fhdc1	1,91E-14	-0,18579	0,066	0,236	3,02E-10
Gtf2b	1,96E-14	-0,20627	0,371	0,629	3,1E-10
Cd82	2E-14	0,255976	0,991	0,954	3,15E-10
Gins4	2,07E-14	-0,2306	0,437	0,675	3,26E-10
Ndufv2	2,09E-14	-0,22489	0,745	0,88	3,29E-10
Coro1c	2,2E-14	0,350002	0,767	0,629	3,47E-10
Cox17	2,21E-14	-0,24255	0,91	0,964	3,49E-10
Mdh1	2,24E-14	-0,23775	0,721	0,859	3,53E-10
Slc35a2	2,24E-14	-0,25358	0,516	0,734	3,54E-10
Ndufa5	2,5E-14	-0,22177	0,864	0,935	3,94E-10
Txndc12	2,51E-14	-0,23364	0,336	0,57	3,96E-10
Lrrc58	2,53E-14	0,277196	0,936	0,882	3,98E-10
Atp1a1	2,53E-14	0,239553	0,998	0,987	3,99E-10
Slirp	2,53E-14	-0,25459	0,554	0,753	3,99E-10
P2rx4	2,7E-14	-0,21943	0,277	0,536	4,26E-10
Ube2e3	2,99E-14	-0,25852	0,721	0,829	4,72E-10
Brk1	3,01E-14	-0,20295	0,868	0,947	4,74E-10
Lsm5	3,14E-14	-0,20954	0,484	0,724	4,96E-10
Selenop	3,73E-14	-0,40765	0,884	0,909	5,89E-10
Lamp1	3,75E-14	-0,15648	1	0,998	5,91E-10
Psmb5	3,77E-14	-0,23164	0,765	0,89	5,95E-10
Mtss1	3,9E-14	0,329616	0,839	0,728	6,16E-10
Tmem14c	4,12E-14	-0,22229	0,56	0,785	6,49E-10
Fn3k	4,12E-14	-0,23943	0,332	0,565	6,5E-10
Tmem165	4,17E-14	-0,20812	0,406	0,656	6,58E-10
Begain	4,17E-14	-0,15371	0,026	0,16	6,58E-10
Ostf1	4,34E-14	-0,20184	0,24	0,473	6,85E-10
Hagh	4,6E-14	-0,22953	0,532	0,753	7,26E-10
Arl6ip5	4,99E-14	-0,25267	0,637	0,823	7,88E-10
Npb	5,08E-14	-0,3166	0,022	0,152	8,02E-10
Rexo2	5,16E-14	-0,25053	0,813	0,895	8,14E-10

Btg1	5,28E-14	0,496837	0,492	0,304	8,32E-10
Enho	5,49E-14	-0,21873	0,2	0,418	8,67E-10
Churc1	5,58E-14	-0,25152	0,613	0,806	8,8E-10
Serpina3n	5,66E-14	0,085592	0,802	0,496	8,93E-10
Xrcc3	6,06E-14	-0,2236	0,266	0,506	9,56E-10
Kmt5a	6,15E-14	-0,26201	0,785	0,907	9,7E-10
Trim16	6,21E-14	-0,15883	0,028	0,162	9,79E-10
Pop5	6,34E-14	-0,21641	0,418	0,658	1E-09
Pyurf	6,35E-14	-0,17958	0,139	0,342	1E-09
Ndufs5	6,6E-14	-0,21436	0,967	0,979	1,04E-09
Cln3	6,8E-14	0,334623	0,782	0,66	1,07E-09
Abca1	6,88E-14	-0,28527	0,209	0,426	1,09E-09
Acy3	6,89E-14	-0,27548	0,112	0,293	1,09E-09
Tmem230	6,98E-14	-0,19951	0,286	0,523	1,1E-09
1110032A(	7,04E-14	-0,2116	0,283	0,525	1,11E-09
Enah	7,26E-14	-0,21546	0,171	0,384	1,15E-09
Tmcc3	7,3E-14	0,27648	0,998	0,989	1,15E-09
Vbp1	7,57E-14	-0,22414	0,815	0,911	1,19E-09
Rasal1	8,15E-14	-0,21545	0,103	0,291	1,28E-09
Myl12b	8,52E-14	-0,26396	0,501	0,719	1,34E-09
Anapc11	8,61E-14	-0,24111	0,89	0,93	1,36E-09
Aldoa	8,86E-14	-0,19835	0,994	0,998	1,4E-09
Cyp51	9,03E-14	0,451816	0,776	0,622	1,42E-09
Slc48a1	9,86E-14	-0,1692	0,998	1	1,55E-09
Bphl	9,92E-14	-0,17128	0,121	0,312	1,57E-09
Dhrs1	1E-13	-0,21241	0,472	0,717	1,58E-09
Slc24a2	1,02E-13	0,242975	1	0,989	1,61E-09
Dst	1,07E-13	0,274081	0,998	0,992	1,68E-09
Slc1a2	1,09E-13	-0,17791	0,028	0,16	1,73E-09
Ndufb6	1,1E-13	-0,22261	0,523	0,753	1,74E-09
Sec11c	1,14E-13	-0,17549	1	0,998	1,79E-09
Ddc	1,15E-13	-0,32604	0,286	0,511	1,81E-09
Psmd8	1,16E-13	-0,21248	0,585	0,806	1,83E-09
Tspan9	1,18E-13	-0,28724	0,165	0,365	1,86E-09
Emc7	1,23E-13	-0,2056	0,919	0,973	1,95E-09
1500011K:	1,33E-13	-0,22906	0,389	0,622	2,1E-09
Spp1	1,4E-13	-0,10606	0,002	0,101	2,21E-09
Fbxo36	1,44E-13	-0,19184	0,182	0,392	2,28E-09
Mrpl57	1,45E-13	-0,2159	0,305	0,555	2,28E-09
Arnt2	1,45E-13	0,310812	0,873	0,778	2,29E-09
Xist	1,58E-13	0,384946	0,714	0,428	2,49E-09
Atp5g1	1,73E-13	-0,19825	0,938	0,96	2,73E-09
Csrp1	1,76E-13	-0,21294	1	0,998	2,78E-09
Polr3h	1,79E-13	-0,20595	0,229	0,451	2,83E-09
Josd2	1,83E-13	-0,2017	0,996	0,996	2,89E-09
Ppp1r16b	1,92E-13	0,271649	0,994	0,968	3,02E-09
Klf7	1,95E-13	0,320954	0,914	0,827	3,07E-09
Fgfr2	2,03E-13	-0,21688	0,958	0,983	3,2E-09
Taf13	2,08E-13	-0,23785	0,457	0,669	3,29E-09
Tmem81	2,14E-13	-0,20973	0,145	0,333	3,37E-09
C230037L1	2,25E-13	-0,19396	0,171	0,376	3,56E-09
Tmem141	2,28E-13	-0,22207	0,215	0,432	3,6E-09

Aldh1a1	2,33E-13	-0,15971	0,061	0,219	3,68E-09
Cltb	2,34E-13	-0,24745	0,802	0,901	3,69E-09
Chmp2b	2,46E-13	-0,32158	0,494	0,698	3,89E-09
Map1lc3b	2,5E-13	-0,18403	0,963	0,979	3,94E-09
Grm7	2,5E-13	-0,19586	0,039	0,177	3,95E-09
Ndufa6	2,62E-13	-0,23555	0,807	0,909	4,13E-09
Slc6a9	2,62E-13	-0,23743	0,842	0,93	4,13E-09
Gss	2,73E-13	-0,25736	0,347	0,572	4,31E-09
S100a10	2,77E-13	0,251875	0,251	0,076	4,38E-09
Gpr85	2,86E-13	-0,16391	0,095	0,272	4,52E-09
Mrfap1	2,94E-13	-0,16973	0,993	0,998	4,64E-09
Anxa5	3,19E-13	0,117996	0,842	0,633	5,03E-09
0610009B2	3,23E-13	-0,18446	0,303	0,561	5,09E-09
Rnf7	3,28E-13	-0,21452	0,987	0,994	5,17E-09
Prdx6	3,29E-13	-0,22227	0,622	0,8	5,19E-09
Mrps18c	3,33E-13	-0,21236	0,393	0,622	5,25E-09
Crim1	3,35E-13	-0,19838	0,233	0,468	5,28E-09
Prdx3	3,38E-13	-0,18863	0,417	0,667	5,33E-09
Myrf	3,4E-13	0,214171	0,991	0,987	5,37E-09
Gltp	3,43E-13	-0,24613	0,996	0,996	5,41E-09
Ctnna3	3,5E-13	-0,20158	0,283	0,506	5,52E-09
Tppp3	3,72E-13	-0,35698	0,934	0,951	5,88E-09
Ndufa1	3,99E-13	-0,23287	0,567	0,77	6,29E-09
Dip2a	4,13E-13	0,286333	0,98	0,937	6,52E-09
2300009A0	4,19E-13	-0,16617	0,163	0,373	6,61E-09
Defb42	4,29E-13	-0,16953	0,066	0,224	6,77E-09
mt-Cytb	4,36E-13	-0,17741	1	0,998	6,88E-09
Tmem60	4,4E-13	-0,18089	0,418	0,677	6,94E-09
Kcnab1	4,44E-13	0,258824	0,242	0,076	7E-09
Sh3gl3	4,6E-13	-0,23719	0,343	0,586	7,26E-09
Yipf4	4,61E-13	-0,22963	0,626	0,8	7,27E-09
Agap1	4,71E-13	0,243414	0,983	0,977	7,43E-09
Atp1a2	4,88E-13	-0,1736	0,046	0,19	7,69E-09
Pdrg1	5E-13	-0,19824	0,301	0,53	7,89E-09
Ctnnb1	5,12E-13	0,282429	0,886	0,768	8,07E-09
Snca	5,19E-13	-0,20965	0,086	0,255	8,18E-09
Pla2g12a	5,25E-13	-0,18628	0,275	0,511	8,28E-09
Isca2	5,4E-13	-0,20369	0,262	0,481	8,51E-09
Echs1	5,45E-13	-0,21621	0,767	0,89	8,6E-09
Cox6b1	5,57E-13	-0,19842	0,974	0,983	8,78E-09
C1galt1c1	5,61E-13	-0,16779	0,237	0,466	8,84E-09
Ctsb	5,71E-13	0,259297	0,998	0,992	9,01E-09
Padi2	5,94E-13	0,27117	0,93	0,808	9,38E-09
Slc35b1	5,99E-13	-0,23237	0,466	0,692	9,44E-09
Sybu	6,04E-13	-0,18701	0,206	0,422	9,53E-09
Irf1	6,18E-13	0,460195	0,2	0,051	9,75E-09
Pomp	6,22E-13	-0,18868	0,87	0,949	9,81E-09
Dynll2	6,59E-13	0,336224	0,861	0,755	1,04E-08
Fbln2	6,87E-13	0,224455	0,191	0,044	1,08E-08
Edf1	6,98E-13	-0,20771	0,833	0,922	1,1E-08
Car14	7,06E-13	-0,24119	0,883	0,941	1,11E-08
Pgk1	7,25E-13	-0,23116	0,73	0,863	1,14E-08

Psmb2	7,28E-13	-0,2361	0,817	0,907	1,15E-08
Hapln2	7,43E-13	-0,25203	0,95	0,964	1,17E-08
Ddx3y	7,51E-13	-0,26937	0,229	0,451	1,19E-08
Tpst1	7,66E-13	-0,21828	0,539	0,764	1,21E-08
Mrpl18	7,77E-13	-0,193	0,479	0,709	1,23E-08
Mrpl55	8,03E-13	-0,19235	0,314	0,544	1,27E-08
Arl4d	8,23E-13	-0,18575	0,152	0,346	1,3E-08
Diablo	8,34E-13	-0,20805	0,543	0,745	1,32E-08
Stat3	8,74E-13	0,352898	0,798	0,648	1,38E-08
Ndufb10	8,8E-13	-0,19945	0,859	0,928	1,39E-08
5031439G(	8,93E-13	0,236878	0,996	0,96	1,41E-08
Cisd2	9,49E-13	-0,2106	0,56	0,768	1,5E-08
Cebpzos	9,56E-13	-0,19909	0,202	0,405	1,51E-08
Polr2c	9,6E-13	-0,17917	0,461	0,711	1,51E-08
Gyg	9,65E-13	-0,22792	0,33	0,546	1,52E-08
2700046A(	9,69E-13	-0,23119	0,101	0,268	1,53E-08
Asah2	9,7E-13	-0,1962	0,114	0,289	1,53E-08
Sec61g	9,82E-13	-0,20256	0,868	0,92	1,55E-08
Rab2a	1,02E-12	-0,21621	0,956	0,973	1,62E-08
Uqcrc2	1,04E-12	-0,21284	0,807	0,895	1,64E-08
Fasn	1,04E-12	0,291521	0,921	0,821	1,65E-08
Dcps	1,05E-12	-0,20779	0,461	0,688	1,66E-08
Abhd3	1,15E-12	-0,19169	0,185	0,388	1,82E-08
Vamp7	1,15E-12	-0,24572	0,376	0,599	1,82E-08
Cenpx	1,21E-12	-0,1891	0,338	0,576	1,91E-08
Fbxo44	1,21E-12	-0,2084	0,262	0,485	1,91E-08
Mrpl36	1,28E-12	-0,20388	0,286	0,519	2,03E-08
Ormdl2	1,29E-12	-0,2002	0,308	0,536	2,04E-08
Hbb-bs	1,32E-12	-2,84885	0	0,089	2,08E-08
4933431E2	1,36E-12	-0,25602	0,547	0,732	2,14E-08
Ube2n	1,37E-12	-0,21412	0,752	0,871	2,16E-08
Abca6	1,37E-12	-0,13439	0,055	0,203	2,16E-08
Gm42500	1,37E-12	-0,18382	0,105	0,276	2,17E-08
Sept9	1,45E-12	0,29062	0,383	0,196	2,29E-08
Dctn3	1,49E-12	-0,209	0,813	0,907	2,35E-08
Son	1,49E-12	0,21981	0,998	0,994	2,35E-08
Hdhd2	1,6E-12	-0,22013	0,528	0,738	2,52E-08
Taz	1,72E-12	-0,19783	0,372	0,616	2,71E-08
Tecr	1,76E-12	-0,17778	0,998	0,996	2,77E-08
Scamp3	1,79E-12	-0,17945	0,411	0,652	2,83E-08
Rtl8b	1,81E-12	-0,24703	0,664	0,821	2,85E-08
Hmbs	1,83E-12	-0,16195	0,156	0,357	2,88E-08
Pex7	1,85E-12	-0,2019	0,275	0,494	2,92E-08
Fgf22	1,86E-12	-0,15309	0,062	0,213	2,94E-08
Rnf113a2	2,18E-12	-0,16651	0,2	0,405	3,44E-08
Pnkd	2,2E-12	-0,22723	0,783	0,899	3,46E-08
Pllp	2,2E-12	-0,16491	1	0,998	3,47E-08
Lsm7	2,3E-12	-0,20151	0,488	0,728	3,63E-08
P4hb	2,31E-12	0,265641	0,89	0,797	3,64E-08
Sertad1	2,34E-12	0,331582	0,284	0,116	3,68E-08
Rraga	2,43E-12	-0,23756	0,539	0,713	3,84E-08
Higd2a	2,49E-12	-0,22827	0,732	0,854	3,94E-08

Cast	2,5E-12	0,22341	0,229	0,072	3,94E-08
Nsmce3	2,53E-12	-0,19184	0,455	0,7	3,99E-08
Stmn4	2,54E-12	-0,15033	1	0,998	4,01E-08
Hdac5	2,64E-12	0,289892	0,783	0,656	4,16E-08
Ccnl1	2,65E-12	0,395225	0,699	0,591	4,18E-08
Hmox2	2,82E-12	-0,22769	0,796	0,901	4,46E-08
Rab18	2,85E-12	-0,21814	0,793	0,892	4,49E-08
Bhlhe40	3,09E-12	0,35339	0,327	0,154	4,88E-08
Phgdh	3,19E-12	-0,21292	0,983	0,985	5,04E-08
Ddrgk1	3,3E-12	-0,19318	0,923	0,956	5,2E-08
Adam19	3,35E-12	-0,21029	0,226	0,437	5,29E-08
Zfyve21	3,58E-12	-0,21551	0,363	0,58	5,65E-08
Ddx6	3,66E-12	0,269911	0,919	0,88	5,77E-08
Zdhhc12	3,71E-12	-0,18742	0,172	0,361	5,85E-08
Man2a2	3,88E-12	0,251624	0,85	0,755	6,12E-08
Asf1a	3,93E-12	-0,20114	0,29	0,5	6,21E-08
Gm2115	3,95E-12	-0,12514	0,022	0,137	6,23E-08
Wbp1	3,96E-12	-0,20311	0,272	0,479	6,24E-08
Lamtor2	4,05E-12	-0,19669	0,675	0,835	6,4E-08
Rmdn1	4,24E-12	-0,17807	0,196	0,399	6,69E-08
Ndufb11	4,42E-12	-0,21142	0,93	0,97	6,97E-08
Cdk5rap3	4,82E-12	-0,18824	0,244	0,454	7,6E-08
Cerk	4,88E-12	0,332878	0,703	0,568	7,69E-08
Hacd1	5,03E-12	-0,19305	0,316	0,542	7,93E-08
Gm15446	5,05E-12	-0,11967	0,02	0,133	7,96E-08
Atp5h	5,32E-12	-0,18014	0,983	0,994	8,39E-08
Zfp536	5,38E-12	0,277709	0,954	0,914	8,49E-08
Fdps	5,48E-12	0,437166	0,875	0,789	8,65E-08
Atp5j	5,55E-12	-0,18412	0,96	0,979	8,76E-08
Micall1	5,55E-12	0,251959	0,989	0,966	8,76E-08
Tnrc6c	6,69E-12	0,305255	0,822	0,751	1,06E-07
Rab9	6,75E-12	-0,20353	0,461	0,681	1,06E-07
Pam16	6,98E-12	-0,18759	0,424	0,656	1,1E-07
Ccdc28b	7,24E-12	-0,18179	0,229	0,441	1,14E-07
Psmd6	7,24E-12	-0,18619	0,516	0,747	1,14E-07
Lyrm2	7,64E-12	-0,206	0,253	0,456	1,21E-07
Arl6	7,68E-12	-0,18244	0,196	0,392	1,21E-07
Hspb11	7,72E-12	-0,19264	0,251	0,462	1,22E-07
Tm2d1	8,15E-12	-0,20426	0,391	0,612	1,29E-07
Elovl5	8,34E-12	-0,25592	0,712	0,85	1,32E-07
Fcor	8,34E-12	-0,22727	0,341	0,559	1,32E-07
Sbds	8,52E-12	-0,20957	0,897	0,947	1,34E-07
2610524H(	8,65E-12	-0,15573	0,099	0,266	1,36E-07
H2-Q4	8,66E-12	0,278107	0,136	0,019	1,37E-07
Abhd6	8,7E-12	-0,18143	0,209	0,409	1,37E-07
Arsg	8,81E-12	-0,20117	0,908	0,947	1,39E-07
Tmem59	9,15E-12	-0,17783	0,976	0,989	1,44E-07
Elob	9,62E-12	-0,15696	0,989	0,987	1,52E-07
Pcdh15	9,82E-12	-0,12718	0,033	0,156	1,55E-07
1600012H(	1E-11	-0,17275	0,202	0,401	1,58E-07
Myl12a	1,06E-11	-0,2025	0,47	0,688	1,68E-07
Commd9	1,08E-11	-0,1863	0,409	0,629	1,71E-07

Slc2a1	1,11E-11	-0,23497	0,565	0,753	1,75E-07
Smim13	1,11E-11	-0,19186	0,363	0,599	1,75E-07
Atp6v1e1	1,15E-11	-0,18319	0,905	0,937	1,81E-07
Runx1	1,18E-11	0,275937	0,15	0,027	1,86E-07
Cacna1h	1,2E-11	0,207046	0,147	0,025	1,9E-07
Tm7sf3	1,21E-11	-0,19068	0,914	0,958	1,92E-07
Cept1	1,22E-11	-0,22019	0,459	0,686	1,93E-07
Gnai2	1,23E-11	0,199757	1	0,992	1,94E-07
Map7d2	1,24E-11	-0,16653	0,218	0,428	1,96E-07
Gadd45b	1,24E-11	0,717872	0,429	0,262	1,96E-07
Ssbp1	1,25E-11	-0,17995	0,305	0,534	1,97E-07
Cct2	1,28E-11	-0,19648	0,75	0,914	2,01E-07
Cnp	1,34E-11	-0,14799	1	1	2,12E-07
Pts	1,35E-11	-0,16023	0,18	0,371	2,13E-07
1110065P2	1,37E-11	-0,22998	0,484	0,681	2,16E-07
Lage3	1,4E-11	-0,18855	0,316	0,532	2,2E-07
Thyn1	1,4E-11	-0,15905	0,22	0,426	2,21E-07
Tmem199	1,41E-11	-0,15016	0,266	0,489	2,23E-07
Fam69a	1,46E-11	-0,1919	0,141	0,312	2,3E-07
Polr3e	1,49E-11	-0,20014	0,409	0,633	2,34E-07
4930402H1	1,52E-11	0,218197	0,985	0,924	2,39E-07
Lsm3	1,53E-11	-0,18205	0,442	0,667	2,42E-07
Dclk1	1,55E-11	-0,18829	0,103	0,27	2,45E-07
Csf1	1,58E-11	0,308641	0,864	0,774	2,49E-07
Fbxw17	1,65E-11	-0,18816	0,189	0,38	2,6E-07
Pycr2	1,67E-11	-0,22317	0,49	0,7	2,64E-07
Syne2	1,7E-11	0,220211	0,257	0,095	2,68E-07
Gjc3	1,71E-11	-0,21063	0,982	0,994	2,69E-07
Mcam	1,71E-11	0,359132	0,734	0,589	2,69E-07
1110004E0	1,72E-11	-0,14817	0,198	0,403	2,72E-07
Bfsp2	1,75E-11	-0,3308	0,464	0,643	2,77E-07
Dclk3	1,8E-11	-0,11444	0,028	0,143	2,83E-07
Cryzl2	1,97E-11	-0,15984	0,143	0,321	3,11E-07
Trappc2l	1,98E-11	-0,19705	0,55	0,755	3,12E-07
Nfkbiz	1,99E-11	0,194303	0,152	0,03	3,14E-07
Cldn14	2E-11	-0,21359	0,189	0,384	3,15E-07
Osbpl7	2,06E-11	-0,20419	0,299	0,506	3,24E-07
Metrn	2,07E-11	-0,23278	0,708	0,821	3,26E-07
Ppfibp2	2,07E-11	0,286242	0,879	0,829	3,26E-07
Ppt2	2,1E-11	-0,15665	0,147	0,327	3,32E-07
Arl2	2,14E-11	-0,17457	0,963	0,968	3,38E-07
Uap1l1	2,16E-11	0,271662	0,429	0,249	3,4E-07
Txndc17	2,23E-11	-0,21765	0,631	0,781	3,51E-07
Smim10l1	2,25E-11	-0,23321	0,572	0,738	3,54E-07
Rtn4	2,31E-11	-0,14791	1	0,998	3,65E-07
Ppa1	2,33E-11	-0,15881	0,11	0,274	3,67E-07
Selenbp1	2,37E-11	-0,15029	0,149	0,333	3,75E-07
Glul	2,43E-11	-0,26826	1	0,996	3,84E-07
Ppp5c	2,45E-11	-0,17204	0,314	0,538	3,87E-07
Nap1l3	2,5E-11	-0,15734	0,09	0,247	3,94E-07
Naa38	2,56E-11	-0,19833	0,475	0,686	4,03E-07
Pbdc1	2,59E-11	-0,17184	0,231	0,443	4,09E-07

Chchd5	2,61E-11	-0,16089	0,149	0,327	4,13E-07
Psenen	2,66E-11	-0,18643	0,912	0,958	4,19E-07
Ankra2	2,67E-11	-0,19054	0,393	0,624	4,21E-07
Gm12326	2,7E-11	-0,18948	0,123	0,291	4,27E-07
Ly6g6d	2,74E-11	-0,16892	0,141	0,319	4,32E-07
Sft2d3	2,79E-11	-0,16909	0,268	0,489	4,4E-07
Per1	2,87E-11	0,489238	0,42	0,247	4,54E-07
Fam234a	2,95E-11	-0,20412	0,352	0,57	4,65E-07
Pgam1	2,99E-11	-0,18949	0,842	0,92	4,71E-07
Lysmd1	2,99E-11	-0,14266	0,15	0,331	4,72E-07
Camk2n1	3,02E-11	-0,24123	0,406	0,612	4,77E-07
Tmem203	3,29E-11	-0,15065	0,125	0,295	5,2E-07
Mrpl41	3,43E-11	-0,17888	0,42	0,656	5,4E-07
Tmem5	3,45E-11	-0,21417	0,604	0,797	5,44E-07
Hotairm1	3,47E-11	-0,21486	0,306	0,508	5,47E-07
Smim27	3,5E-11	-0,19818	0,229	0,428	5,53E-07
Zbtb8os	3,57E-11	-0,16461	0,301	0,519	5,63E-07
Mapk10	3,58E-11	-0,16209	0,101	0,259	5,65E-07
Tomm7	3,58E-11	-0,1818	0,824	0,918	5,65E-07
Pla2g4a	3,72E-11	-0,1851	0,257	0,47	5,87E-07
Immp1l	3,76E-11	-0,16736	0,376	0,603	5,92E-07
Apbb1	3,81E-11	-0,19065	0,936	0,96	6,01E-07
Sumo1	3,82E-11	-0,20319	0,631	0,808	6,02E-07
Bpnt1	3,87E-11	-0,20412	0,53	0,736	6,11E-07
Hibadh	3,97E-11	-0,21881	0,56	0,736	6,27E-07
Sat2	4,01E-11	-0,17159	0,209	0,411	6,33E-07
Eef2	4,12E-11	0,189374	1	0,998	6,51E-07
Gmpr2	4,31E-11	-0,16877	0,229	0,43	6,8E-07
Zfp760	4,35E-11	-0,14929	0,105	0,266	6,85E-07
Slc25a38	4,57E-11	-0,16512	0,161	0,342	7,21E-07
Mrpl20	4,64E-11	-0,18773	0,761	0,88	7,31E-07
Gdpd1	4,73E-11	-0,19816	0,462	0,692	7,46E-07
0610040B1	4,74E-11	-0,13631	0,095	0,253	7,48E-07
Uqcrh	4,83E-11	-0,1783	0,947	0,977	7,62E-07
Impa1	4,97E-11	-0,16596	0,334	0,565	7,83E-07
Anxa2	5,25E-11	0,245047	0,433	0,236	8,28E-07
Ipp	5,51E-11	-0,11714	0,061	0,198	8,69E-07
Adssl1	5,73E-11	-0,26437	0,906	0,918	9,04E-07
Magt1	5,77E-11	-0,20479	0,906	0,958	9,11E-07
Hikeshi	5,79E-11	-0,17662	0,339	0,544	9,13E-07
Klc1	5,97E-11	0,287648	0,75	0,629	9,42E-07
Bsg	6,08E-11	-0,19163	0,941	0,968	9,6E-07
Dctpp1	6,35E-11	-0,16555	0,231	0,435	1E-06
Dpm3	6,39E-11	-0,19388	0,745	0,873	1,01E-06
Uqcrfs1	6,45E-11	-0,20169	0,58	0,772	1,02E-06
Srm	6,54E-11	-0,1873	0,257	0,468	1,03E-06
Golim4	6,69E-11	-0,23356	0,22	0,416	1,06E-06
Itgb3bp	6,97E-11	-0,16641	0,167	0,346	1,1E-06
Mea1	7,04E-11	-0,16133	0,288	0,498	1,11E-06
Nsmce1	7,19E-11	-0,16305	0,305	0,53	1,13E-06
Dpy30	7,33E-11	-0,1947	0,389	0,591	1,16E-06
Scp2	7,35E-11	-0,17358	0,912	0,951	1,16E-06

Glt8d1	7,4E-11	-0,17472	0,239	0,443	1,17E-06
Ccnh	7,68E-11	-0,17599	0,295	0,504	1,21E-06
Cmc4	7,73E-11	-0,16655	0,207	0,399	1,22E-06
Rpp30	7,83E-11	-0,14242	0,215	0,414	1,24E-06
Ddhd1	8,08E-11	0,352016	0,714	0,599	1,28E-06
Kazn	8,67E-11	0,236378	0,943	0,892	1,37E-06
Gtf2a2	8,76E-11	-0,16185	0,409	0,643	1,38E-06
Egr3	8,9E-11	0,318669	0,127	0,019	1,4E-06
Napa	9,01E-11	-0,18224	0,857	0,932	1,42E-06
Chac2	9,13E-11	-0,1428	0,105	0,262	1,44E-06
Ninj2	9,15E-11	-0,25113	0,527	0,711	1,44E-06
Naa20	9,24E-11	-0,16392	0,31	0,519	1,46E-06
Nde1	9,52E-11	-0,25191	0,512	0,698	1,5E-06
Arhgdia	9,57E-11	0,23317	0,993	0,973	1,51E-06
Polb	9,58E-11	-0,19031	0,495	0,711	1,51E-06
Sf3b6	9,63E-11	-0,17798	0,672	0,829	1,52E-06
Gstm6	1E-10	-0,14456	0,077	0,222	1,58E-06
Sdhaf1	1,04E-10	-0,15943	0,268	0,475	1,64E-06
Sdccag3	1,06E-10	-0,16943	0,374	0,599	1,68E-06
Abca8a	1,07E-10	-0,22015	0,165	0,342	1,69E-06
Dock10	1,08E-10	0,218663	0,987	0,97	1,71E-06
Mrps14	1,08E-10	-0,16449	0,306	0,53	1,71E-06
Ppib	1,13E-10	-0,16884	0,941	0,973	1,78E-06
Marcksl1	1,15E-10	0,274387	0,89	0,711	1,81E-06
1110008P1	1,15E-10	-0,1496	0,2	0,392	1,82E-06
Snap47	1,17E-10	-0,16797	0,391	0,62	1,85E-06
Dusp19	1,19E-10	-0,15938	0,213	0,403	1,87E-06
Tbc1d7	1,19E-10	-0,1688	0,255	0,456	1,88E-06
Ndufb5	1,2E-10	-0,18945	0,771	0,865	1,9E-06
Lancl1	1,2E-10	-0,1907	0,409	0,622	1,9E-06
Fus	1,21E-10	0,224741	0,987	0,992	1,91E-06
Dnajc19	1,25E-10	-0,20521	0,692	0,833	1,98E-06
Ifi27	1,28E-10	0,314814	0,884	0,743	2,03E-06
Acer2	1,29E-10	-0,10685	0,024	0,129	2,03E-06
Rpl24	1,36E-10	-0,13777	0,991	0,998	2,14E-06
Minpp1	1,36E-10	-0,1618	0,229	0,428	2,15E-06
Sptlc2	1,37E-10	-0,15347	0,365	0,597	2,16E-06
Bola1	1,42E-10	-0,1962	0,24	0,428	2,24E-06
Cyp2u1	1,42E-10	-0,15768	0,154	0,327	2,24E-06
Cers2	1,45E-10	-0,14741	0,994	0,998	2,29E-06
Cd9	1,46E-10	0,340442	0,952	0,863	2,31E-06
Plpp3	1,48E-10	0,319382	0,761	0,627	2,33E-06
Agbl3	1,49E-10	-0,14117	0,11	0,27	2,35E-06
Surf1	1,54E-10	-0,19169	0,486	0,692	2,43E-06
Myg1	1,54E-10	-0,14649	0,24	0,443	2,44E-06
Gm10076	1,55E-10	-0,14456	0,097	0,247	2,44E-06
Mad1l1	1,55E-10	-0,15378	0,209	0,399	2,45E-06
Irgm1	1,61E-10	0,475118	0,163	0,042	2,53E-06
Cnn3	1,61E-10	-0,21216	0,697	0,825	2,55E-06
Tmem128	1,71E-10	-0,17444	0,444	0,66	2,69E-06
Sgf29	1,73E-10	-0,13801	0,128	0,295	2,73E-06
Zfand5	1,78E-10	0,264916	0,963	0,93	2,82E-06

Dapk2	1,82E-10	-0,13278	0,07	0,207	2,88E-06
Sptssa	1,84E-10	-0,19029	0,813	0,897	2,9E-06
Sdhaf4	1,84E-10	-0,14117	0,127	0,291	2,9E-06
Ndufs4	1,85E-10	-0,18123	0,589	0,785	2,91E-06
Coa3	1,85E-10	-0,1788	0,666	0,816	2,92E-06
Pdzd11	1,88E-10	-0,17001	0,242	0,435	2,96E-06
Gm12216	1,88E-10	0,206392	0,106	0,011	2,97E-06
Rbbp9	1,92E-10	-0,16625	0,292	0,508	3,03E-06
Uqcc3	1,93E-10	-0,16684	0,327	0,536	3,04E-06
Amotl2	1,98E-10	0,303247	0,73	0,639	3,13E-06
Gm14305	2E-10	-0,13958	0,149	0,321	3,16E-06
Mrpl10	2,01E-10	-0,17237	0,279	0,481	3,18E-06
Cmc1	2,02E-10	-0,18759	0,262	0,449	3,18E-06
Cript	2,09E-10	-0,18782	0,776	0,871	3,29E-06
3830406C1	2,16E-10	-0,16753	0,275	0,485	3,4E-06
Asnsd1	2,17E-10	-0,14115	0,128	0,293	3,42E-06
Fah	2,19E-10	-0,21896	0,517	0,694	3,46E-06
Cdh19	2,25E-10	-0,16684	0,273	0,481	3,54E-06
Pigyl	2,35E-10	-0,18878	0,818	0,922	3,71E-06
Fgfrl1	2,42E-10	-0,25087	0,251	0,439	3,81E-06
Ndufa13	2,47E-10	-0,15558	0,974	0,981	3,9E-06
Uchl3	2,53E-10	-0,19186	0,402	0,618	4E-06
Aif1l	2,62E-10	-0,18398	0,328	0,538	4,14E-06
Tgoln1	2,64E-10	0,233159	0,921	0,863	4,17E-06
Trnt1	2,65E-10	-0,20168	0,466	0,66	4,18E-06
Sucla2	2,67E-10	-0,17471	0,328	0,536	4,21E-06
Aasdhppt	2,74E-10	-0,16869	0,231	0,422	4,32E-06
Mycbp	2,75E-10	-0,1347	0,099	0,251	4,34E-06
Pla2g16	2,77E-10	-0,14224	0,998	0,998	4,37E-06
Olig1	2,81E-10	-0,19536	0,976	0,977	4,43E-06
H3f3a	2,81E-10	-0,15449	1	1	4,44E-06
Tbrg1	2,83E-10	-0,15691	0,365	0,593	4,46E-06
Hoxd9	2,98E-10	-0,12033	0,051	0,175	4,7E-06
Kcnk13	2,98E-10	-0,21459	0,651	0,81	4,7E-06
Pcyt2	3E-10	0,26779	0,947	0,869	4,73E-06
Polr2k	3,02E-10	-0,17272	0,429	0,641	4,76E-06
Pacs2	3,08E-10	0,22792	0,998	0,987	4,86E-06
Pdia6	3,11E-10	-0,22758	0,688	0,821	4,9E-06
Myh14	3,14E-10	-0,20053	0,422	0,608	4,96E-06
Chchd4	3,18E-10	-0,14379	0,215	0,411	5,02E-06
Fryl	3,22E-10	0,2321	0,897	0,81	5,09E-06
Kctd5	3,28E-10	-0,14258	0,178	0,359	5,18E-06
Tm2d3	3,33E-10	-0,14599	0,295	0,506	5,25E-06
Vat1	3,42E-10	0,285108	0,67	0,544	5,39E-06
Krt10	3,42E-10	-0,14252	0,176	0,359	5,4E-06
Sytl2	3,44E-10	0,300548	0,859	0,77	5,43E-06
Tank	3,46E-10	-0,15598	0,169	0,342	5,46E-06
Mrpl58	3,47E-10	-0,19354	0,528	0,715	5,47E-06
Rnf122	3,48E-10	0,510661	0,505	0,382	5,49E-06
Tmem126a	3,5E-10	-0,15774	0,286	0,492	5,52E-06
Pdcd5	3,77E-10	-0,18087	0,747	0,846	5,95E-06
Cndp2	3,88E-10	-0,13958	0,246	0,447	6,12E-06

Smim8	3,95E-10	-0,16577	0,226	0,411	6,23E-06
Fam71a	4,07E-10	0,31451	0,084	0,002	6,41E-06
3110082J2	4,22E-10	-0,11064	0,024	0,124	6,67E-06
Mdp1	4,25E-10	-0,17444	0,284	0,483	6,7E-06
Galnt7	4,33E-10	-0,20352	0,464	0,665	6,84E-06
Gnas	4,35E-10	-0,1546	0,996	0,994	6,86E-06
Eef1e1	4,37E-10	-0,13684	0,242	0,443	6,9E-06
Emc2	4,4E-10	-0,1871	0,429	0,629	6,94E-06
1700123O	4,44E-10	-0,13226	0,191	0,38	7,01E-06
Polr2d	4,47E-10	-0,15631	0,172	0,342	7,05E-06
Snhg9	4,48E-10	-0,15947	0,196	0,378	7,07E-06
Pebp1	4,54E-10	-0,16243	0,974	0,987	7,17E-06
Clic5	4,54E-10	-0,15653	0,046	0,165	7,17E-06
Eif2s3y	4,6E-10	-0,19114	0,187	0,373	7,26E-06
Grina	4,69E-10	0,248717	0,798	0,69	7,39E-06
Lsamp	4,72E-10	-0,10881	0,024	0,124	7,45E-06
Mrps36	4,75E-10	-0,17054	0,457	0,671	7,49E-06
Cab39l	4,82E-10	-0,16224	0,194	0,373	7,6E-06
Nt5m	4,86E-10	-0,15821	0,149	0,312	7,66E-06
Pigz	4,93E-10	-0,24863	0,723	0,835	7,78E-06
Fam13c	4,98E-10	0,358905	0,708	0,559	7,86E-06
Tomm5	5,15E-10	-0,1718	0,389	0,601	8,12E-06
Zfp938	5,38E-10	-0,14121	0,099	0,245	8,49E-06
Twistnb	5,62E-10	-0,155	0,255	0,456	8,87E-06
Pcyt1b	5,62E-10	-0,15529	0,266	0,477	8,87E-06
Zfp638	5,7E-10	0,278997	0,846	0,768	8,99E-06
Mrpl48	5,79E-10	-0,21065	0,76	0,869	9,13E-06
Mthfs	5,81E-10	-0,14641	0,235	0,43	9,16E-06
Gm29455	5,85E-10	-0,16395	0,099	0,243	9,23E-06
Dhrs7b	5,91E-10	-0,15422	0,277	0,475	9,32E-06
Timm13	6E-10	-0,16999	0,881	0,947	9,46E-06
Pop7	6,06E-10	-0,14398	0,193	0,373	9,56E-06
Tmod2	6,25E-10	-0,31694	0,804	0,816	9,86E-06
Smpd1	6,27E-10	-0,20546	0,563	0,728	9,9E-06
Acadl	6,35E-10	-0,19557	0,461	0,646	1E-05
Mrps34	6,4E-10	-0,16879	0,308	0,502	1,01E-05
Irf2	6,55E-10	-0,17403	0,347	0,553	1,03E-05
Stk16	6,68E-10	-0,16995	0,376	0,584	1,05E-05
Polr2i	6,81E-10	-0,1591	0,411	0,627	1,07E-05
Hint3	7,01E-10	-0,18138	0,461	0,669	1,11E-05
Mettl23	7,03E-10	-0,15405	0,33	0,551	1,11E-05
Hmgcr	7,23E-10	0,453579	0,593	0,475	1,14E-05
Cmpk1	7,26E-10	-0,16427	0,426	0,639	1,14E-05
mt-Nd4	7,35E-10	-0,14509	1	0,998	1,16E-05
Sdhb	7,44E-10	-0,18731	0,617	0,785	1,17E-05
Cetn3	7,59E-10	-0,20983	0,705	0,831	1,2E-05
Cox14	7,69E-10	-0,16974	0,594	0,783	1,21E-05
Pde4b	7,7E-10	0,226836	0,985	0,939	1,22E-05
Ugp2	7,9E-10	-0,17619	0,521	0,724	1,25E-05
Pacsin1	7,91E-10	-0,20728	0,31	0,502	1,25E-05
1700029J0	7,98E-10	-0,12746	0,057	0,179	1,26E-05
Piga	8,08E-10	-0,15539	0,415	0,616	1,28E-05

Slc25a33	8,15E-10	-0,14124	0,095	0,236	1,29E-05
Mageh1	8,2E-10	-0,17567	0,294	0,492	1,29E-05
Taok1	8,28E-10	0,235923	0,895	0,844	1,31E-05
Gm48508	8,31E-10	-0,12948	0,079	0,215	1,31E-05
Tpgs2	8,53E-10	-0,15502	0,224	0,409	1,35E-05
Fbxo7	8,61E-10	-0,22238	0,745	0,835	1,36E-05
Ubac1	8,69E-10	-0,15674	0,328	0,53	1,37E-05
Rbx1	8,72E-10	-0,15258	0,928	0,964	1,38E-05
Lpcat2	8,81E-10	-0,31641	0,257	0,441	1,39E-05
Mrps12	8,82E-10	-0,14303	0,385	0,608	1,39E-05
Chmp1b	9,33E-10	-0,16339	0,488	0,696	1,47E-05
Exosc3	9,46E-10	-0,13297	0,097	0,241	1,49E-05
Mrps16	9,57E-10	-0,16416	0,394	0,605	1,51E-05
Med10	9,58E-10	-0,12894	0,275	0,479	1,51E-05
Dcun1d2	9,59E-10	-0,14113	0,139	0,304	1,51E-05
Cldnd1	9,76E-10	-0,14812	0,98	0,981	1,54E-05
Pdcl3	9,85E-10	-0,1477	0,262	0,456	1,55E-05
Dohh	1E-09	-0,16063	0,319	0,527	1,58E-05
Abcf3	1,02E-09	-0,13695	0,277	0,479	1,61E-05
Prpf31	1,03E-09	-0,15002	0,288	0,483	1,63E-05
Sec13	1,04E-09	-0,18916	0,495	0,69	1,64E-05
Ap4s1	1,06E-09	-0,16418	0,275	0,473	1,67E-05
Mad2l2	1,06E-09	-0,15641	0,16	0,323	1,68E-05
Gadd45gip	1,07E-09	-0,17122	0,404	0,618	1,69E-05
Folh1	1,1E-09	-0,15782	0,301	0,511	1,73E-05
Mrpl50	1,1E-09	-0,15119	0,207	0,386	1,74E-05
Lrrc1	1,11E-09	-0,15936	0,365	0,574	1,75E-05
Mobp	1,12E-09	-0,12673	1	0,998	1,77E-05
Pradc1	1,13E-09	-0,13654	0,163	0,333	1,78E-05
Pcmt1	1,14E-09	-0,19905	0,653	0,808	1,8E-05
Zfp329	1,14E-09	-0,11525	0,213	0,409	1,8E-05
D1Ertd622	1,15E-09	0,259385	0,927	0,895	1,82E-05
Tomm20	1,17E-09	-0,18401	0,908	0,943	1,84E-05
Aven	1,18E-09	-0,25	0,15	0,306	1,85E-05
Nufip2	1,19E-09	0,309565	0,725	0,622	1,87E-05
Sumo2	1,2E-09	-0,17058	0,892	0,945	1,89E-05
Gm12689	1,23E-09	-0,10525	0,033	0,137	1,95E-05
Sgms1	1,24E-09	-0,1738	0,218	0,401	1,95E-05
Fgfbp3	1,25E-09	-0,15833	0,127	0,278	1,97E-05
Glrx2	1,27E-09	-0,15657	0,473	0,692	2,01E-05
Dctn6	1,29E-09	-0,17505	0,501	0,707	2,04E-05
Dcdc2a	1,31E-09	-0,17232	0,139	0,3	2,06E-05
Cyp2j9	1,32E-09	-0,12016	0,044	0,156	2,08E-05
Cib1	1,32E-09	-0,17228	0,22	0,392	2,09E-05
Mcts1	1,35E-09	-0,15494	0,297	0,498	2,13E-05
Creld1	1,38E-09	-0,17382	0,215	0,384	2,18E-05
Nedd8	1,38E-09	-0,1733	0,873	0,928	2,18E-05
Coprs	1,39E-09	-0,14601	0,125	0,274	2,19E-05
4931406C(	1,4E-09	-0,16514	0,314	0,519	2,21E-05
Zc3hav1	1,42E-09	0,19173	0,237	0,097	2,24E-05
Gskip	1,42E-09	-0,14107	0,178	0,352	2,25E-05
Tmem50a	1,44E-09	-0,17491	0,943	0,968	2,27E-05

Nudt16	1,47E-09	-0,14419	0,149	0,31	2,31E-05
Izumo4	1,48E-09	-0,14498	0,229	0,418	2,34E-05
Cops5	1,5E-09	-0,17309	0,64	0,814	2,36E-05
Pop4	1,54E-09	-0,1951	0,527	0,692	2,43E-05
Aga	1,55E-09	-0,1618	0,22	0,401	2,44E-05
Dubr	1,57E-09	-0,1553	0,18	0,357	2,48E-05
Fam189a2	1,61E-09	-0,14432	0,121	0,274	2,54E-05
Pex13	1,64E-09	-0,1615	0,325	0,53	2,59E-05
Timm17b	1,64E-09	-0,16757	0,314	0,506	2,59E-05
2410015M	1,65E-09	-0,16763	0,763	0,869	2,6E-05
Selenow	1,66E-09	-0,13753	0,994	0,996	2,62E-05
Ift20	1,67E-09	-0,15992	0,615	0,793	2,64E-05
Dixdc1	1,69E-09	-0,17906	0,897	0,943	2,66E-05
Prima1	1,71E-09	-0,19407	0,439	0,65	2,7E-05
Zfp286	1,72E-09	-0,10129	0,026	0,122	2,71E-05
Zfp943	1,73E-09	-0,12457	0,09	0,228	2,73E-05
Bnip3	1,73E-09	-0,17279	0,402	0,612	2,73E-05
Galnt11	1,74E-09	-0,1508	0,231	0,414	2,75E-05
Garem2	1,74E-09	-0,13098	0,059	0,179	2,75E-05
Eps15	1,76E-09	-0,22422	0,844	0,903	2,78E-05
Inpp5a	1,93E-09	0,265061	0,596	0,468	3,04E-05
mt-Nd1	1,93E-09	0,167905	1	0,998	3,05E-05
Ufsp2	1,95E-09	-0,16074	0,299	0,498	3,08E-05
C030006K1	2,01E-09	-0,11811	0,092	0,232	3,17E-05
Wipi1	2,03E-09	-0,18563	0,822	0,907	3,21E-05
Ptp4a2	2,07E-09	0,208798	0,956	0,903	3,27E-05
Ctss	2,08E-09	-0,08971	0,018	0,108	3,28E-05
Med7	2,16E-09	-0,1453	0,213	0,388	3,41E-05
Taf10	2,22E-09	-0,18366	0,567	0,734	3,5E-05
Dpyd	2,3E-09	-0,14762	0,095	0,234	3,63E-05
Oard1	2,32E-09	-0,17373	0,31	0,508	3,67E-05
AU040320	2,4E-09	-0,15335	0,231	0,409	3,79E-05
Snrnp25	2,4E-09	-0,12022	0,117	0,268	3,79E-05
Mrpl15	2,41E-09	-0,14698	0,33	0,536	3,8E-05
Jpt2	2,5E-09	-0,22464	0,281	0,466	3,95E-05
Ndst1	2,57E-09	0,234736	0,683	0,563	4,05E-05
Slc25a11	2,57E-09	-0,13958	0,417	0,637	4,06E-05
Osbpl1a	2,6E-09	-0,17358	0,98	0,992	4,1E-05
Msl3	2,62E-09	-0,15997	0,244	0,42	4,13E-05
Elof1	2,62E-09	-0,13958	0,343	0,557	4,13E-05
Tmem143	2,7E-09	-0,12949	0,086	0,219	4,27E-05
Mrpl42	2,71E-09	-0,15529	0,479	0,686	4,27E-05
Atp5l	2,75E-09	-0,1556	0,976	0,981	4,33E-05
Atpaf1	2,75E-09	-0,13673	0,211	0,39	4,34E-05
E2f5	2,78E-09	-0,13806	0,16	0,323	4,38E-05
Phip	2,85E-09	0,262411	0,82	0,724	4,49E-05
Mrps10	2,86E-09	-0,12785	0,275	0,473	4,51E-05
Fam216a	2,92E-09	-0,12663	0,116	0,264	4,61E-05
Tgfbi	2,98E-09	0,26476	0,339	0,184	4,7E-05
Uqcc2	3,02E-09	-0,18434	0,782	0,871	4,76E-05
Uvssa	3,19E-09	-0,17275	0,182	0,346	5,04E-05
Phactr2	3,2E-09	-0,13813	0,169	0,338	5,04E-05

Inpp5j	3,25E-09	-0,12299	0,086	0,222	5,13E-05
Mzt1	3,27E-09	-0,15712	0,317	0,506	5,16E-05
Ndfip2	3,31E-09	-0,19425	0,763	0,863	5,23E-05
Srebf1	3,36E-09	-0,16745	0,47	0,669	5,31E-05
Dbil5	3,37E-09	-0,1163	0,037	0,139	5,32E-05
Tppp	3,4E-09	0,189901	0,996	0,977	5,37E-05
Gm4924	3,48E-09	-0,12454	0,084	0,217	5,49E-05
Thoc7	3,52E-09	-0,17293	0,363	0,568	5,56E-05
Ola1	3,57E-09	-0,1686	0,363	0,572	5,63E-05
Plcl1	3,57E-09	-0,17789	0,956	0,964	5,64E-05
Nkiras1	3,59E-09	-0,14973	0,391	0,593	5,67E-05
Faim2	3,66E-09	-0,19928	0,895	0,932	5,77E-05
Gm26802	3,7E-09	0,304627	0,086	0,006	5,83E-05
Eef1a1	3,76E-09	-0,1107	1	0,998	5,93E-05
Tyw5	3,76E-09	-0,12041	0,121	0,27	5,93E-05
Endog	3,9E-09	-0,12321	0,106	0,249	6,16E-05
Paics	3,92E-09	-0,17297	0,802	0,897	6,19E-05
Spry2	3,93E-09	-0,12744	0,196	0,376	6,2E-05
Trappc2	3,97E-09	-0,14897	0,383	0,589	6,27E-05
Ppp1r35	3,98E-09	-0,13101	0,226	0,409	6,28E-05
Bace1	4E-09	-0,23588	0,778	0,857	6,31E-05
Med30	4,12E-09	-0,17165	0,488	0,684	6,5E-05
Magoh	4,16E-09	-0,13566	0,292	0,487	6,56E-05
Ctso	4,24E-09	-0,13958	0,261	0,451	6,68E-05
Runx2	4,25E-09	0,14026	0,099	0,013	6,7E-05
Opcml	4,26E-09	-0,12464	0,09	0,224	6,72E-05
Tmem237	4,28E-09	-0,14334	0,303	0,504	6,76E-05
Mrps25	4,37E-09	-0,14872	0,383	0,599	6,89E-05
Ube2v2	4,4E-09	-0,13853	0,428	0,652	6,94E-05
Pick1	4,44E-09	-0,14713	0,308	0,502	7E-05
Gemin7	4,5E-09	-0,14459	0,332	0,527	7,1E-05
Mrpl51	4,61E-09	-0,16171	0,492	0,681	7,27E-05
Polr2l	4,68E-09	-0,14318	0,325	0,538	7,38E-05
Cdc123	4,69E-09	-0,15529	0,47	0,667	7,4E-05
Scrn1	4,74E-09	0,254106	0,532	0,401	7,49E-05
Pigq	4,76E-09	0,228595	0,761	0,665	7,51E-05
Ccnl2	4,8E-09	0,234445	0,809	0,73	7,57E-05
Smim19	4,83E-09	-0,16765	0,389	0,584	7,61E-05
Snx15	4,83E-09	-0,19733	0,75	0,886	7,62E-05
Ubac2	4,84E-09	-0,14943	0,233	0,409	7,64E-05
9330199G	4,87E-09	-0,10892	0,031	0,129	7,68E-05
Zer1	4,89E-09	-0,1764	0,497	0,698	7,71E-05
Rnf112	4,89E-09	-0,16493	0,266	0,454	7,72E-05
Hsbp1	4,9E-09	-0,15507	0,98	0,989	7,74E-05
Prox1os	4,96E-09	-0,19099	0,314	0,504	7,82E-05
Rnaseh1	4,97E-09	-0,12882	0,163	0,325	7,84E-05
Ugt8a	5,08E-09	-0,13875	0,998	0,998	8,01E-05
Elovl7	5,21E-09	-0,19904	0,952	0,973	8,23E-05
Cd320	5,24E-09	-0,12496	0,114	0,257	8,27E-05
Gm33594	5,36E-09	-0,16205	0,211	0,382	8,45E-05
Enpp1	5,62E-09	-0,15827	0,248	0,424	8,86E-05
Smim20	5,66E-09	-0,16071	0,367	0,557	8,92E-05

Clcn2	5,72E-09	-0,17165	0,29	0,468	9,03E-05
Hspa14	5,87E-09	-0,11588	0,226	0,416	9,26E-05
Vti1b	5,89E-09	-0,18855	0,528	0,705	9,28E-05
Stx8	5,91E-09	-0,14207	0,33	0,53	9,32E-05
Tmem38b	6,03E-09	-0,16792	0,361	0,557	9,51E-05
Tmbim4	6,04E-09	-0,16077	0,71	0,844	9,53E-05
Gcat	6,21E-09	-0,13958	0,209	0,382	9,79E-05
Ebp	6,21E-09	-0,17345	0,528	0,724	9,8E-05
Lap3	6,23E-09	-0,19878	0,967	0,973	9,83E-05
Fam204a	6,31E-09	-0,16475	0,521	0,728	9,95E-05
Znrd1	6,32E-09	-0,12887	0,279	0,475	9,97E-05
Mrps17	6,34E-09	-0,16338	0,394	0,591	0,0001
Dennd5a	6,37E-09	0,217146	0,978	0,932	0,000101
Snx1	6,38E-09	-0,16597	0,811	0,886	0,000101
Blvra	6,4E-09	-0,14099	0,24	0,42	0,000101
Git1	6,51E-09	0,207731	0,875	0,795	0,000103
Ybx3	6,53E-09	-0,18383	0,793	0,888	0,000103
Efr3b	6,56E-09	-0,14663	0,189	0,359	0,000104
Psma5	6,57E-09	-0,18172	0,664	0,774	0,000104
Gpd1	6,64E-09	-0,17237	0,521	0,696	0,000105
Cdc26	6,65E-09	-0,13261	0,374	0,593	0,000105
Ppp1cb	6,69E-09	0,207328	0,93	0,846	0,000106
Flnb	6,7E-09	0,291883	0,58	0,46	0,000106
Washc3	6,74E-09	-0,13662	0,182	0,35	0,000106
Tmco1	6,82E-09	-0,1833	0,657	0,812	0,000108
Kdm2a	6,9E-09	0,265545	0,701	0,597	0,000109
Hmgn3	6,95E-09	-0,15685	0,194	0,357	0,00011
Mrpl11	6,99E-09	-0,13395	0,402	0,62	0,00011
Nckap5	7E-09	0,195777	0,191	0,07	0,00011
Xrcc4	7,09E-09	-0,12915	0,18	0,346	0,000112
Pkd2l1	7,12E-09	-0,18798	0,086	0,213	0,000112
Tbcd	7,25E-09	-0,16281	0,272	0,456	0,000114
Itsn2	7,3E-09	0,247982	0,815	0,719	0,000115
Commd6	7,34E-09	-0,15652	0,635	0,81	0,000116
Foxn3	7,35E-09	0,208437	0,996	0,996	0,000116
Tmem192	7,39E-09	-0,15213	0,312	0,496	0,000117
Fra10ac1	7,56E-09	-0,12603	0,174	0,338	0,000119
Igtp	7,6E-09	0,330425	0,088	0,008	0,00012
Gm30025	7,62E-09	-0,13795	0,116	0,253	0,00012
Chordc1	7,62E-09	-0,17481	0,488	0,671	0,00012
Ppil3	7,64E-09	-0,14252	0,191	0,357	0,000121
Brms1	7,84E-09	-0,14088	0,295	0,487	0,000124
Slc2a8	7,87E-09	-0,13639	0,13	0,276	0,000124
Ppid	8,06E-09	-0,1619	0,527	0,722	0,000127
Cisd3	8,07E-09	-0,12065	0,134	0,285	0,000127
Steap3	8,11E-09	0,204813	0,217	0,084	0,000128
Yipf3	8,15E-09	-0,16584	0,49	0,684	0,000129
Hadhb	8,22E-09	-0,17332	0,666	0,81	0,00013
Bccip	8,24E-09	-0,14754	0,279	0,464	0,00013
Psmd13	8,47E-09	-0,16792	0,582	0,741	0,000134
Smim11	8,63E-09	-0,14627	0,411	0,612	0,000136
Tprkb	8,64E-09	-0,17633	0,503	0,688	0,000136

Unc50	8,69E-09	-0,17273	0,694	0,833	0,000137
Mrpl54	8,7E-09	-0,1665	0,369	0,553	0,000137
Dgcr6	8,83E-09	-0,1474	0,29	0,479	0,000139
Clns1a	8,85E-09	-0,14734	0,428	0,627	0,00014
Atraid	8,96E-09	-0,15743	0,866	0,916	0,000141
Atg3	8,99E-09	-0,17404	0,741	0,85	0,000142
Tmem144	9,13E-09	-0,19885	0,539	0,707	0,000144
Suclg1	9,39E-09	-0,16154	0,558	0,757	0,000148
Txnl4a	9,75E-09	-0,16396	0,435	0,618	0,000154
Jagn1	9,92E-09	-0,12509	0,354	0,57	0,000156
Tead1	1E-08	0,212881	0,194	0,074	0,000158
Park7	1,02E-08	-0,14597	0,943	0,977	0,000161
Ddr1	1,03E-08	0,172843	0,993	0,981	0,000162
Gm49012	1,03E-08	-0,10772	0,048	0,154	0,000163
Mtx2	1,04E-08	-0,15222	0,461	0,665	0,000164
Lamtor4	1,05E-08	-0,16309	0,606	0,759	0,000166
Mrpl40	1,06E-08	-0,1461	0,295	0,479	0,000168
Ublcp1	1,11E-08	-0,17927	0,341	0,515	0,000176
Zfp955b	1,15E-08	-0,09883	0,051	0,16	0,000181
Them6	1,17E-08	-0,10999	0,062	0,177	0,000185
Lsm8	1,18E-08	-0,13128	0,349	0,559	0,000186
Exosc7	1,18E-08	-0,12291	0,235	0,416	0,000187
Scmh1	1,21E-08	-0,14648	0,215	0,386	0,000191
Abat	1,21E-08	-0,12474	0,163	0,325	0,000191
Crip2	1,22E-08	0,304613	0,435	0,297	0,000193
Itgb1bp1	1,22E-08	-0,13031	0,284	0,47	0,000193
Fam92a	1,22E-08	-0,13286	0,259	0,441	0,000193
Pym1	1,27E-08	-0,12101	0,092	0,219	0,0002
Ost4	1,29E-08	-0,18	0,727	0,821	0,000204
Zbtb16	1,29E-08	-0,1364	0,125	0,266	0,000204
Igbp1	1,32E-08	-0,1408	0,327	0,523	0,000208
Rpsa	1,32E-08	0,238606	1	0,998	0,000209
Cnrip1	1,35E-08	-0,13341	0,136	0,283	0,000212
1810024B(	1,36E-08	-0,09404	0,048	0,154	0,000214
Abca5	1,38E-08	0,292978	0,466	0,321	0,000217
Mydgf	1,38E-08	-0,15376	0,558	0,747	0,000218
Efs	1,39E-08	0,227097	0,332	0,184	0,00022
Gm36855	1,4E-08	-0,10171	0,064	0,181	0,00022
Tbc1d5	1,41E-08	-0,1733	0,538	0,73	0,000222
AI480526	1,44E-08	-0,1453	0,191	0,357	0,000228
Ctnnal1	1,46E-08	-0,15281	0,251	0,424	0,000231
Ttll7	1,51E-08	-0,14551	1	0,996	0,000238
Dab1	1,53E-08	-0,0934	0,015	0,093	0,000241
Erlec1	1,54E-08	-0,148	0,31	0,502	0,000243
Yaf2	1,55E-08	-0,16335	0,389	0,572	0,000244
Rmdn3	1,55E-08	-0,12959	0,218	0,392	0,000244
Mrpl14	1,57E-08	-0,15421	0,477	0,669	0,000248
Mblac2	1,6E-08	-0,15081	0,218	0,384	0,000252
Dap3	1,62E-08	-0,11636	0,33	0,536	0,000256
Ccs	1,64E-08	-0,13836	0,352	0,557	0,000259
Ntmt1	1,64E-08	-0,13958	0,213	0,38	0,000259
Lsm1	1,65E-08	-0,13537	0,242	0,414	0,000261

Canx	1,67E-08	0,185537	0,994	0,987	0,000263
Cnih1	1,68E-08	-0,17328	0,628	0,77	0,000265
Gprc5b	1,7E-08	-0,16838	0,974	0,981	0,000268
Trnau1ap	1,7E-08	-0,14969	0,213	0,373	0,000269
Fundc2	1,7E-08	-0,14437	0,334	0,525	0,000269
Zfyve19	1,72E-08	-0,1304	0,161	0,319	0,000271
2700046G(	1,77E-08	-0,08926	0,062	0,177	0,000278
Enoph1	1,77E-08	-0,19894	0,606	0,745	0,000279
Mest	1,79E-08	-0,11954	0,09	0,217	0,000282
Sbsn	1,82E-08	-0,11451	0,094	0,222	0,000287
Dapk3	1,83E-08	-0,15406	0,292	0,468	0,000289
Commd4	1,87E-08	-0,16171	0,523	0,709	0,000294
Mcat	1,87E-08	-0,10734	0,09	0,217	0,000296
Tsc22d3	1,91E-08	-0,17126	0,4	0,584	0,000302
Scrg1	1,95E-08	-0,07226	0,011	0,084	0,000307
3110040N:	1,97E-08	-0,1228	0,092	0,217	0,00031
3222401L1	1,97E-08	-0,13958	0,217	0,395	0,00031
Fbxw5	2,04E-08	-0,1282	0,237	0,411	0,000322
Dnajb1	2,06E-08	0,642325	0,578	0,492	0,000324
Cby1	2,07E-08	-0,12438	0,18	0,338	0,000326
Slc16a1	2,09E-08	-0,18855	0,664	0,819	0,00033
Arid1a	2,12E-08	0,239247	0,69	0,61	0,000334
Scd3	2,2E-08	-0,15755	0,189	0,346	0,000347
Tex264	2,27E-08	-0,14674	0,479	0,686	0,000358
Kmt2e	2,27E-08	0,22729	0,927	0,886	0,000358
Ttc1	2,29E-08	-0,15902	0,464	0,656	0,000361
Ndufaf5	2,31E-08	-0,11581	0,084	0,207	0,000365
Armcx5	2,32E-08	-0,11959	0,141	0,289	0,000365
Lrrc8b	2,32E-08	0,217325	0,859	0,755	0,000367
Dnal4	2,34E-08	-0,1247	0,2	0,363	0,000369
Prelid3b	2,39E-08	-0,14087	0,297	0,479	0,000377
Ecsit	2,39E-08	-0,12966	0,211	0,378	0,000377
Med9	2,39E-08	-0,12888	0,171	0,325	0,000377
Rpl22l1	2,4E-08	-0,16381	0,701	0,825	0,000378
Gas2	2,4E-08	-0,11628	0,092	0,219	0,000379
Med8	2,46E-08	-0,13097	0,336	0,534	0,000389
Nup35	2,48E-08	-0,12948	0,206	0,369	0,000391
Znhit1	2,59E-08	-0,17426	0,574	0,741	0,000408
Hspe1	2,59E-08	-0,1522	0,861	0,922	0,000408
Taf1d	2,61E-08	0,265523	0,683	0,599	0,000412
Gas7	2,62E-08	0,197582	0,939	0,895	0,000413
Psma2	2,64E-08	-0,14077	0,853	0,922	0,000416
Pkig	2,64E-08	-0,13222	0,198	0,359	0,000417
Tmem80	2,66E-08	-0,16007	0,444	0,65	0,000419
Narfl	2,76E-08	-0,12403	0,156	0,306	0,000435
Creg2	2,78E-08	-0,10398	0,103	0,238	0,000439
D030056L:	2,86E-08	-0,12494	0,281	0,466	0,00045
Pdk3	2,86E-08	-0,14471	0,264	0,441	0,000451
Med31	2,86E-08	-0,13192	0,413	0,622	0,000452
Mdga2	2,91E-08	-0,15751	0,44	0,639	0,000459
Eef1akmt2	2,95E-08	-0,12481	0,182	0,342	0,000466
Derl2	2,97E-08	-0,14187	0,376	0,572	0,000469

Stxbp6	2,99E-08	-0,16933	0,561	0,73	0,000472
Jtb	3,01E-08	-0,15022	0,578	0,762	0,000474
Pold4	3,08E-08	-0,14982	0,198	0,352	0,000486
Tyro3	3,11E-08	0,231316	0,928	0,85	0,000491
Wdr83os	3,14E-08	-0,15418	0,462	0,658	0,000495
Morn2	3,17E-08	-0,12682	0,125	0,262	0,0005
Prim2	3,19E-08	-0,11069	0,075	0,194	0,000503
Pantr1	3,19E-08	-0,17206	0,288	0,468	0,000503
Scrn3	3,19E-08	-0,13091	0,209	0,373	0,000504
Wasf3	3,21E-08	-0,15376	0,121	0,255	0,000507
Prnp	3,22E-08	0,150633	1	0,987	0,000509
Psmg4	3,29E-08	-0,15882	0,288	0,458	0,000518
Eif2b3	3,35E-08	-0,13958	0,224	0,395	0,000529
Ndufaf3	3,39E-08	-0,14203	0,332	0,515	0,000534
Fbl	3,41E-08	0,308202	0,672	0,597	0,000538
Ahsa1	3,44E-08	-0,20656	0,732	0,819	0,000542
Stoml2	3,54E-08	-0,12812	0,316	0,5	0,000559
Uap1	3,58E-08	-0,15667	0,325	0,502	0,000564
Tm2d2	3,61E-08	-0,15487	0,708	0,829	0,000569
Mrps31	3,64E-08	-0,14097	0,248	0,411	0,000574
Gm42372	3,66E-08	-0,15752	0,182	0,335	0,000577
Tceal1	3,67E-08	-0,13682	0,237	0,405	0,000579
Gpr180	3,71E-08	-0,10925	0,128	0,27	0,000586
Cops6	3,74E-08	-0,16222	0,651	0,81	0,000589
Tmem229a	3,74E-08	-0,19047	0,894	0,939	0,000589
Pik3r1	3,75E-08	0,204445	0,978	0,947	0,000591
Map2k5	3,78E-08	-0,12253	0,224	0,392	0,000597
Macrod1	3,83E-08	-0,14742	0,288	0,456	0,000603
Ncbp2	3,83E-08	-0,15538	0,453	0,631	0,000604
1110059G	3,88E-08	-0,11605	0,233	0,405	0,000611
Ogdhl	3,9E-08	-0,17826	0,139	0,281	0,000616
Dhrs4	3,91E-08	-0,11454	0,147	0,293	0,000617
Nqo1	3,96E-08	-0,11899	0,064	0,175	0,000624
Imp3	4,02E-08	-0,14972	0,679	0,812	0,000634
Dmac1	4,02E-08	-0,13681	0,25	0,416	0,000634
Gpnmb	4,04E-08	-0,06672	0,011	0,082	0,000638
Sox11	4,1E-08	0,337485	0,106	0,021	0,000646
Rhbdd2	4,11E-08	-0,13244	0,224	0,386	0,000648
Hnrnpdl	4,11E-08	0,19307	0,963	0,96	0,000648
Ankrd28	4,12E-08	0,224218	0,934	0,84	0,00065
Arid5b	4,14E-08	0,283126	0,758	0,681	0,000652
Coa4	4,15E-08	-0,09747	0,057	0,165	0,000654
Hist3h2ba	4,18E-08	-0,19427	0,365	0,546	0,00066
Galnt12	4,2E-08	-0,09264	0,033	0,124	0,000662
Mrps24	4,24E-08	-0,15367	0,563	0,726	0,000669
Pcdh11x	4,24E-08	-0,12519	0,097	0,224	0,000669
Chst12	4,3E-08	-0,12479	0,187	0,346	0,000678
Zfp976	4,34E-08	-0,08432	0,018	0,097	0,000684
Msl3l2	4,45E-08	-0,13808	0,185	0,335	0,000702
Adora1	4,48E-08	-0,1688	0,547	0,732	0,000707
Smn1	4,49E-08	-0,10973	0,178	0,333	0,000709
Clock	4,53E-08	0,20365	0,772	0,637	0,000715

Rpl29	4,54E-08	-0,15041	0,96	0,973	0,000717
Atp1b3	4,58E-08	0,223877	0,998	0,989	0,000722
Tma16	4,6E-08	-0,1356	0,2	0,363	0,000726
Smim1	4,68E-08	-0,19272	0,479	0,652	0,000739
Idh3a	4,74E-08	-0,12498	0,345	0,538	0,000747
Map6	4,75E-08	0,260448	0,571	0,479	0,00075
Tmem178b	4,77E-08	0,224281	0,895	0,819	0,000752
Abhd16a	4,78E-08	-0,15505	0,455	0,641	0,000754
Dda1	4,79E-08	-0,15769	0,506	0,684	0,000756
Dnajb6	4,82E-08	-0,14154	0,818	0,901	0,00076
Dnajc17	4,83E-08	-0,10614	0,172	0,329	0,000761
Cdh20	4,84E-08	-0,14936	0,317	0,502	0,000763
Mtus1	4,88E-08	-0,17727	0,738	0,867	0,00077
Dctn2	4,94E-08	-0,14764	0,949	0,97	0,000779
Nktr	4,96E-08	0,204497	0,928	0,892	0,000782
Znhit3	5,04E-08	-0,12231	0,217	0,38	0,000795
Ccdc167	5,09E-08	-0,11757	0,13	0,272	0,000804
Nrn1l	5,16E-08	-0,09105	0,039	0,133	0,000814
Naxe	5,16E-08	-0,16436	0,618	0,776	0,000815
Lztfl1	5,27E-08	-0,15728	0,341	0,521	0,000831
Ccdc91	5,28E-08	-0,15029	0,161	0,304	0,000833
Mettl22	5,32E-08	-0,10363	0,114	0,247	0,00084
Snap23	5,35E-08	-0,14231	0,25	0,424	0,000843
Sh3bgrl3	5,38E-08	-0,12911	0,983	0,994	0,000848
9530059O1	5,46E-08	-0,13646	0,119	0,253	0,000861
Osgin2	5,47E-08	-0,1244	0,165	0,314	0,000864
Gosr1	5,48E-08	-0,13958	0,345	0,53	0,000865
Slc25a5	5,51E-08	-0,162	0,71	0,819	0,00087
Clcc1	5,54E-08	-0,13026	0,284	0,464	0,000873
Zfp830	5,56E-08	-0,12635	0,194	0,354	0,000878
Zfp934	5,69E-08	-0,1296	0,099	0,222	0,000898
Sik2	5,72E-08	-0,12271	0,242	0,424	0,000903
Chka	5,76E-08	0,549322	0,556	0,475	0,000909
Trub2	5,79E-08	-0,13251	0,217	0,382	0,000914
Carhsp1	5,91E-08	-0,14185	0,963	0,977	0,000932
March5	5,94E-08	-0,1329	0,418	0,612	0,000938
Dtymk	5,95E-08	-0,15021	0,354	0,538	0,000938
Ywhae	5,97E-08	-0,10884	1	0,998	0,000942
Gm28782	6,03E-08	-0,07007	0,011	0,08	0,000951
Ndufs8	6,17E-08	-0,16416	0,561	0,732	0,000973
Fam103a1	6,18E-08	-0,11763	0,259	0,435	0,000974
B4gat1	6,18E-08	-0,14468	0,613	0,783	0,000975
1600020E0	6,2E-08	-0,12599	0,174	0,323	0,000978
Rab10	6,3E-08	-0,12384	0,983	0,977	0,000993
Rogdi	6,33E-08	-0,13148	0,121	0,251	0,000999
Timm10b	6,43E-08	-0,15572	0,517	0,7	0,001014
Zfp606	6,43E-08	-0,13332	0,15	0,291	0,001015
St3gal4	6,45E-08	0,218201	0,833	0,738	0,001018
Ddx17	6,48E-08	0,176502	0,914	0,827	0,001022
Commd1	6,58E-08	-0,14202	0,646	0,819	0,001039
Pacsin3	6,58E-08	-0,15584	0,473	0,66	0,001039
Manf	6,61E-08	-0,20688	0,547	0,711	0,001043

mt-Nd3	6,61E-08	0,182003	1	0,998	0,001043
Rbm39	6,84E-08	0,158422	0,998	0,992	0,001079
Cinp	6,88E-08	-0,11265	0,217	0,386	0,001085
Ppp2r3c	6,94E-08	-0,1582	0,295	0,464	0,001095
Col6a3	6,97E-08	-0,08847	0,024	0,105	0,0011
Lsm12	7,06E-08	-0,13103	0,45	0,643	0,001114
Polr1d	7,26E-08	-0,14788	0,565	0,747	0,001146
BC003965	7,37E-08	-0,12974	0,347	0,534	0,001163
Nkiras2	7,38E-08	-0,1083	0,11	0,241	0,001164
Vps29	7,41E-08	-0,1628	0,752	0,85	0,001168
Minos1	7,48E-08	-0,15061	0,794	0,876	0,00118
Coq7	7,54E-08	-0,11747	0,349	0,553	0,001189
Nlk	7,72E-08	-0,14084	0,283	0,468	0,001219
Zfp637	7,75E-08	-0,14317	0,369	0,555	0,001223
Mpv17l2	7,76E-08	-0,13031	0,272	0,445	0,001225
Mpst	7,92E-08	-0,16747	0,622	0,787	0,001249
Urod	7,98E-08	-0,14473	0,319	0,485	0,001259
Lhfpl2	8,17E-08	-0,0882	0,077	0,194	0,00129
Rps13	8,2E-08	-0,11717	0,993	0,994	0,001294
Naxd	8,24E-08	-0,17048	0,604	0,768	0,0013
Rpl7l1	8,32E-08	-0,13058	0,301	0,481	0,001312
Ppa2	8,42E-08	-0,11086	0,281	0,462	0,001328
Rps27a	8,44E-08	-0,11324	1	0,998	0,001331
Capn6	8,47E-08	0,07764	0,059	0	0,001337
Rpl15	8,48E-08	-0,13343	0,987	0,983	0,001337
Nxt1	8,5E-08	-0,11451	0,094	0,215	0,001341
Smim12	8,51E-08	-0,11887	0,264	0,439	0,001343
Gm10031	8,6E-08	-0,09388	0,061	0,167	0,001356
Rcn2	8,66E-08	-0,12741	0,325	0,511	0,001366
Ccdc191	8,84E-08	-0,1189	0,178	0,331	0,001395
Bckdhb	9,01E-08	-0,11996	0,182	0,331	0,001422
Syngr2	9,18E-08	-0,14068	0,424	0,614	0,001448
4833420G	9,27E-08	-0,12894	0,261	0,441	0,001462
H2-Ke6	9,3E-08	-0,13958	0,301	0,479	0,001467
Araf	9,4E-08	-0,15883	0,629	0,789	0,001484
Fancf	9,54E-08	-0,11582	0,141	0,281	0,001505
Ppp1cc	9,65E-08	-0,18477	0,673	0,781	0,001522
Nkx2-9	9,74E-08	-0,32877	0,16	0,289	0,001537
Rtn1	9,75E-08	-0,06364	0,004	0,061	0,001538
E2f3	9,77E-08	-0,08263	0,026	0,108	0,001541
Mn1	9,77E-08	-0,12169	0,163	0,308	0,001541
Rab2b	9,87E-08	-0,15531	0,321	0,5	0,001557
Rab3ip	9,87E-08	-0,11509	0,112	0,241	0,001557
9330182L	9,97E-08	-0,17258	0,4	0,578	0,001572
Bbip1	9,98E-08	-0,16747	0,552	0,715	0,001574
Arglu1	1,02E-07	-0,14083	0,916	0,958	0,00161
Bad	1,02E-07	-0,11519	0,169	0,314	0,001614
Cry1	1,03E-07	-0,10493	0,141	0,283	0,001619
2610001J	1,04E-07	-0,16822	0,587	0,738	0,001639
Nhlrc1	1,04E-07	-0,13459	0,095	0,213	0,00164
Trmt10a	1,04E-07	-0,10167	0,156	0,306	0,001646
Mrps23	1,04E-07	-0,12898	0,292	0,462	0,001648

Tmem181a	1,05E-07	-0,14085	0,275	0,451	0,001662
Sra1	1,05E-07	-0,16837	0,556	0,705	0,001664
Ier5	1,05E-07	0,161202	0,134	0,04	0,001664
Tmem250-p	1,1E-07	-0,13281	0,261	0,43	0,001728
Lgals8	1,1E-07	-0,13003	0,356	0,538	0,00173
Ufl1	1,11E-07	-0,14063	0,433	0,627	0,001744
Apc	1,11E-07	0,201649	0,963	0,939	0,001746
Clip2	1,14E-07	0,220379	0,761	0,673	0,001799
Anapc16	1,14E-07	-0,1562	0,628	0,795	0,001803
Lgr4	1,15E-07	-0,11343	0,105	0,23	0,001815
Timm9	1,15E-07	-0,12654	0,29	0,464	0,001819
Xxylt1	1,16E-07	-0,12393	0,143	0,281	0,001824
Ramp1	1,16E-07	-0,06196	0,006	0,065	0,001832
Nadk2	1,16E-07	-0,14346	0,288	0,46	0,001835
Tcaim	1,16E-07	-0,1195	0,152	0,295	0,001836
Trim7	1,16E-07	-0,1212	0,088	0,205	0,001838
Prdx2	1,17E-07	-0,16378	0,822	0,884	0,001851
Arfgap3	1,17E-07	-0,13035	0,259	0,426	0,001851
Coq8a	1,18E-07	-0,10835	0,11	0,238	0,001858
Mat2b	1,18E-07	-0,12958	0,426	0,599	0,001865
Cpa2	1,18E-07	-0,09274	0,062	0,169	0,001868
Samhd1	1,23E-07	0,238016	0,451	0,319	0,001942
Rad23a	1,23E-07	-0,16245	0,578	0,749	0,001946
Snrpe	1,25E-07	-0,1386	0,517	0,694	0,001966
Acsf2	1,26E-07	-0,09986	0,035	0,122	0,001982
Sars	1,27E-07	-0,18995	0,789	0,863	0,002002
Setd5	1,27E-07	0,236532	0,71	0,599	0,002009
Jund	1,29E-07	0,285498	1	0,998	0,002028
Alg6	1,29E-07	-0,12526	0,13	0,264	0,002035
Smarcd3	1,29E-07	-0,10695	0,11	0,236	0,002041
Pank3	1,31E-07	0,247285	0,596	0,494	0,002069
Nfx1	1,32E-07	-0,13462	0,316	0,5	0,002079
Arhgef2	1,32E-07	0,202379	0,952	0,914	0,002084
Tmed4	1,33E-07	-0,13428	0,677	0,854	0,002099
Zfp580	1,33E-07	-0,12781	0,193	0,338	0,0021
Arpc1b	1,33E-07	0,237833	0,703	0,589	0,002106
Hipk2	1,34E-07	0,178787	0,956	0,943	0,002113
Aamdc	1,35E-07	-0,17844	0,67	0,783	0,002131
Nfia	1,35E-07	0,209394	0,927	0,863	0,002133
Pnisr	1,38E-07	-0,11485	0,943	0,977	0,002176
Cnih4	1,41E-07	-0,14367	0,457	0,646	0,002226
Caap1	1,42E-07	-0,11978	0,171	0,316	0,002235
Ubb	1,43E-07	-0,12202	1	0,998	0,002256
Arpc5l	1,45E-07	-0,14635	0,534	0,703	0,00229
Tmed2	1,46E-07	-0,14103	0,947	0,956	0,002304
Srsf2	1,47E-07	0,20207	0,974	0,964	0,002323
Sec61b	1,5E-07	-0,14257	0,525	0,679	0,002364
Specc1	1,5E-07	-0,1734	0,793	0,871	0,002365
Pde4d	1,5E-07	-0,12832	0,112	0,236	0,002371
Sptan1	1,51E-07	0,261916	0,547	0,437	0,002386
H2afx	1,52E-07	-0,13208	0,171	0,314	0,002395
Zfp758	1,53E-07	-0,10131	0,062	0,167	0,002419

Parl	1,54E-07	-0,13958	0,433	0,62	0,002429
Tmem110	1,56E-07	-0,12757	0,29	0,46	0,002458
Abhd4	1,58E-07	-0,15682	0,606	0,768	0,002486
Zfp101	1,58E-07	-0,15111	0,297	0,458	0,00249
Ptcd2	1,58E-07	-0,12589	0,171	0,314	0,002494
Haghl	1,58E-07	-0,12435	0,561	0,751	0,002499
Acad11	1,59E-07	-0,11489	0,099	0,219	0,002509
Yif1a	1,59E-07	-0,15191	0,484	0,658	0,002514
Mlh3	1,61E-07	-0,12968	0,305	0,483	0,002535
Lrrc28	1,61E-07	-0,12107	0,31	0,487	0,002541
Pgls	1,61E-07	-0,13845	0,396	0,576	0,002541
Cyb5r3	1,61E-07	0,215822	0,38	0,247	0,002543
Gstk1	1,64E-07	-0,13958	0,226	0,378	0,002584
Commd3	1,64E-07	-0,15769	0,62	0,776	0,002593
Nudt5	1,65E-07	-0,12492	0,251	0,42	0,002597
Rnaseh2b	1,65E-07	-0,11843	0,174	0,321	0,002602
Mrps7	1,66E-07	-0,13655	0,503	0,677	0,002625
Ndufaf8	1,68E-07	-0,13591	0,321	0,504	0,00265
Gng5	1,68E-07	-0,15152	0,892	0,911	0,002651
Samm50	1,69E-07	-0,13408	0,42	0,618	0,00267
Hmgcl	1,7E-07	-0,14424	0,569	0,726	0,002675
Ppp1r11	1,7E-07	-0,14159	0,508	0,679	0,002679
Mta1	1,7E-07	-0,12519	0,222	0,378	0,002686
Psd3	1,7E-07	-0,11111	0,411	0,601	0,002689
Nsmce4a	1,71E-07	-0,14826	0,543	0,711	0,002691
Zfp560	1,71E-07	-0,12206	0,202	0,357	0,002705
Apba2	1,76E-07	0,149548	0,178	0,07	0,002775
Plekhg1	1,76E-07	0,252485	0,822	0,787	0,00278
1110019D1	1,77E-07	-0,09609	0,029	0,112	0,002788
Thumpd1	1,77E-07	-0,13606	0,35	0,525	0,002794
Snx32	1,77E-07	-0,13958	0,514	0,698	0,002799
1110008F1	1,77E-07	-0,13772	0,55	0,738	0,0028
Grm3	1,78E-07	-0,16998	0,481	0,652	0,002813
Mrpl28	1,79E-07	-0,12299	0,444	0,62	0,002818
Ganc	1,79E-07	-0,12803	0,196	0,344	0,002824
Ddx1	1,79E-07	-0,1571	0,778	0,888	0,002827
Scnm1	1,8E-07	-0,13529	0,451	0,639	0,002837
Mphosph6	1,82E-07	-0,10976	0,334	0,523	0,002867
Meaf6	1,82E-07	-0,152	0,4	0,578	0,00287
Ccpg1os	1,82E-07	-0,10104	0,062	0,165	0,002873
Tmem222	1,82E-07	-0,13133	0,391	0,58	0,002878
Kmt2a	1,83E-07	0,249856	0,725	0,662	0,002884
Smox	1,84E-07	-0,11785	0,281	0,46	0,0029
Rpp21	1,86E-07	-0,13713	0,338	0,517	0,002939
Wdr18	1,88E-07	-0,11827	0,268	0,437	0,00296
Csad	1,88E-07	-0,14405	0,174	0,316	0,002967
Smco3	1,92E-07	-0,19191	0,503	0,637	0,003027
Dram2	1,92E-07	-0,14432	0,363	0,544	0,003031
Fkbp3	1,93E-07	-0,15514	0,58	0,728	0,003042
Dld	1,93E-07	-0,12114	0,4	0,586	0,003046
Tmem135	1,93E-07	-0,13143	0,255	0,424	0,003049
Senp8	1,96E-07	-0,12227	0,136	0,268	0,003091

Hoxd3os1	1,98E-07	-0,1408	0,303	0,487	0,003117
Bcar1	1,99E-07	0,236487	0,723	0,633	0,003136
Rnf5	2,02E-07	-0,15821	0,798	0,886	0,003192
Dnajc30	2,07E-07	-0,14093	0,273	0,43	0,003271
Srp9	2,15E-07	-0,16972	0,651	0,791	0,003387
1110004F1	2,16E-07	-0,14296	0,787	0,88	0,003408
Dcxr	2,2E-07	-0,11899	0,139	0,272	0,003463
Yae1d1	2,2E-07	-0,10433	0,394	0,593	0,003471
Ndufs2	2,2E-07	-0,13296	0,813	0,899	0,003475
Maob	2,21E-07	-0,114	0,079	0,188	0,00349
Vps4a	2,22E-07	-0,13451	0,499	0,688	0,003497
Dtd1	2,25E-07	-0,13846	0,389	0,572	0,003544
Glrx5	2,25E-07	-0,12869	0,35	0,54	0,003545
Ufm1	2,25E-07	-0,13184	0,422	0,616	0,003555
Spg7	2,29E-07	-0,12654	0,288	0,456	0,003619
Uckl1	2,31E-07	-0,13823	0,264	0,424	0,003648
Mapt	2,32E-07	0,157515	1	0,998	0,003661
Rsl24d1	2,34E-07	-0,13958	0,393	0,568	0,003699
Anapc13	2,37E-07	-0,15713	0,639	0,781	0,003737
Gm44421	2,39E-07	-0,08717	0,039	0,127	0,003764
Srp68	2,39E-07	-0,11108	0,295	0,47	0,003769
Tnfaip6	2,5E-07	-0,12671	0,936	0,943	0,003944
Serp1	2,51E-07	-0,16961	0,497	0,658	0,003961
Trim36	2,51E-07	-0,15291	0,369	0,553	0,003963
4930430F(	2,51E-07	-0,13836	0,323	0,504	0,003965
Pdcd6	2,53E-07	-0,14823	0,613	0,759	0,003997
Oxld1	2,56E-07	-0,09165	0,053	0,15	0,004035
Rer1	2,57E-07	-0,14429	0,873	0,916	0,004057
Psmd10	2,58E-07	-0,11865	0,185	0,333	0,004068
Chrac1	2,58E-07	-0,12992	0,235	0,399	0,004069
Bcap29	2,6E-07	-0,12731	0,24	0,401	0,004102
H3f3b	2,61E-07	0,2126	1	0,996	0,004125
Hsdl2	2,62E-07	-0,12113	0,27	0,445	0,004128
Rps7	2,63E-07	-0,11685	0,982	0,983	0,004155
D10Jhu81e	2,64E-07	-0,13462	0,338	0,513	0,004167
Cmtm3	2,66E-07	0,156687	0,193	0,082	0,004195
Fdx1	2,7E-07	-0,12808	0,218	0,371	0,004252
Cox19	2,71E-07	-0,11291	0,299	0,479	0,004275
Ndufs3	2,71E-07	-0,15464	0,567	0,732	0,004277
Stx18	2,72E-07	-0,12883	0,273	0,441	0,004292
Actl6a	2,73E-07	-0,11081	0,328	0,511	0,004301
Rnf138	2,74E-07	-0,11214	0,259	0,424	0,004322
Adgrg1	2,77E-07	0,180835	0,233	0,112	0,004371
Akirin2	2,79E-07	-0,14578	0,589	0,762	0,004397
Ift57	2,79E-07	-0,12405	0,156	0,291	0,004401
Pecr	2,86E-07	-0,09649	0,156	0,3	0,004504
Gpi1	2,88E-07	-0,15446	0,87	0,914	0,004538
Tab1	2,88E-07	-0,11474	0,196	0,35	0,004539
Ankrd49	2,91E-07	-0,11144	0,279	0,454	0,004597
Cenpq	2,92E-07	-0,10425	0,092	0,207	0,004605
Slc24a5	2,93E-07	-0,10082	0,347	0,534	0,004628
Trappc3	2,94E-07	-0,13958	0,538	0,694	0,004631

Timmdc1	2,94E-07	-0,10357	0,121	0,247	0,004636
Ube2q2	2,95E-07	-0,12444	0,259	0,418	0,004659
Uxs1	2,99E-07	-0,12468	0,185	0,329	0,004722
Tmem33	3E-07	-0,13838	0,862	0,928	0,004725
Cuta	3E-07	-0,13752	0,78	0,88	0,004733
Hnrnpa0	3,02E-07	0,186219	0,877	0,861	0,004767
Fam161b	3,03E-07	-0,10127	0,09	0,205	0,004786
Tiprl	3,05E-07	-0,12364	0,352	0,534	0,004809
Zfp979	3,06E-07	-0,07651	0,055	0,154	0,004826
Sez6l2	3,07E-07	-0,1343	0,954	0,981	0,004842
Saraf	3,08E-07	-0,13129	0,972	0,968	0,004862
Zfp950	3,09E-07	-0,12975	0,338	0,519	0,00488
Zfp87	3,12E-07	-0,11476	0,108	0,228	0,004918
Lsm2	3,12E-07	-0,12793	0,202	0,346	0,00493
Pter	3,13E-07	-0,10813	0,136	0,268	0,004934
Laptm4b	3,16E-07	-0,15791	0,725	0,857	0,004978
Ahsa2	3,18E-07	-0,14314	0,358	0,525	0,005016
Fgl2	3,21E-07	0,276801	0,083	0,013	0,005057
Ndufa12	3,21E-07	-0,15278	0,662	0,789	0,005071
Selenos	3,23E-07	-0,16325	0,897	0,939	0,00509
Rsu1	3,32E-07	-0,14186	0,716	0,844	0,005231
Ift43	3,32E-07	-0,16563	0,512	0,688	0,005232
Gm44386	3,42E-07	-0,09282	0,064	0,167	0,005391
Zmiz1	3,42E-07	0,267384	0,633	0,58	0,005401
Glrx3	3,44E-07	-0,16086	0,655	0,772	0,00542
Dnm3	3,54E-07	-0,151	0,945	0,979	0,005583
Coq5	3,6E-07	-0,106	0,272	0,445	0,005685
Cdc42ep2	3,61E-07	-0,15098	0,949	0,979	0,005687
Srsf3	3,62E-07	0,193775	0,965	0,92	0,005716
Rassf2	3,63E-07	0,18144	0,91	0,886	0,005719
Matr3	3,63E-07	0,184991	0,965	0,935	0,005723
Clk1	3,65E-07	0,196742	0,93	0,905	0,005755
AC133505.	3,66E-07	-0,06825	0,011	0,074	0,005774
Cdan1	3,68E-07	-0,11616	0,156	0,291	0,005813
Cyb5b	3,7E-07	-0,12619	0,332	0,506	0,00584
Rint1	3,73E-07	-0,11495	0,279	0,454	0,005887
Psmd9	3,74E-07	-0,12176	0,422	0,616	0,005893
Ubald2	3,79E-07	-0,10867	0,418	0,612	0,005978
Lzts2	3,8E-07	0,177107	0,967	0,947	0,005999
Bche	3,8E-07	-0,11412	0,215	0,367	0,006001
Dhdds	3,84E-07	-0,13023	0,283	0,443	0,006062
Fmc1	3,85E-07	-0,13468	0,521	0,703	0,00607
Eif2s1	3,85E-07	-0,11062	0,383	0,582	0,006077
Ccdc85b	3,86E-07	-0,13741	0,442	0,635	0,006087
A330049N	3,88E-07	-0,14233	0,228	0,378	0,006117
Mtx1	3,89E-07	-0,12664	0,224	0,378	0,00613
March2	3,9E-07	-0,13675	0,56	0,736	0,006148
Ppp1r10	3,94E-07	0,37892	0,561	0,479	0,006217
Dync1h1	3,97E-07	0,193866	0,974	0,941	0,006259
Gm26890	3,97E-07	-0,14328	0,294	0,46	0,006265
Got1	3,98E-07	-0,13958	0,319	0,481	0,006274
Ttc12	4,08E-07	-0,09274	0,066	0,169	0,00644

Arpc1a	4,1E-07	-0,17951	0,974	0,985	0,006475
Mrpl13	4,12E-07	-0,14317	0,354	0,523	0,0065
Cyb5d2	4,12E-07	-0,14105	0,191	0,331	0,0065
Rad51d	4,13E-07	-0,1286	0,147	0,276	0,006517
Gpd2	4,24E-07	-0,23032	0,433	0,57	0,006691
Fhit	4,24E-07	-0,08875	0,037	0,12	0,006692
Mrpl16	4,24E-07	-0,09187	0,139	0,272	0,006692
Coq10a	4,29E-07	-0,13048	0,303	0,466	0,006771
Gclc	4,34E-07	-0,13008	0,347	0,517	0,006848
Tubb2b	4,35E-07	-0,45088	0,094	0,207	0,00686
Trpm7	4,35E-07	0,218388	0,694	0,61	0,006861
Taf6l	4,38E-07	-0,11001	0,268	0,435	0,006917
Ppm1m	4,47E-07	-0,11059	0,084	0,192	0,00705
Rnase4	4,47E-07	0,205769	0,196	0,084	0,007052
H2afj	4,47E-07	-0,15518	0,934	0,977	0,007053
Nfu1	4,51E-07	-0,12377	0,29	0,449	0,007108
Ptpra	4,51E-07	-0,12557	0,947	0,964	0,007112
Ndufb2	4,57E-07	-0,15446	0,824	0,892	0,007204
Polr2h	4,61E-07	-0,09684	0,222	0,384	0,007275
Faim	4,68E-07	-0,15028	0,532	0,696	0,007376
Sem1	4,68E-07	-0,13622	0,886	0,939	0,007387
Ktn1	4,71E-07	-0,12548	0,989	0,996	0,007435
Gal3st1	4,75E-07	-0,18899	0,936	0,964	0,00749
Rtraf	4,78E-07	-0,16511	0,745	0,859	0,007534
Nat9	4,81E-07	-0,12482	0,279	0,441	0,007589
Mut	4,88E-07	-0,16267	0,361	0,527	0,007693
Tor1a	4,92E-07	-0,10609	0,294	0,46	0,007754
Slc6a6	4,98E-07	0,206962	0,945	0,935	0,007851
Nkx6-2	4,98E-07	-0,13188	0,983	0,994	0,007859
Mff	5,08E-07	-0,13578	0,703	0,827	0,008016
Pgp	5,09E-07	-0,14302	0,883	0,909	0,008038
Zc3h6	5,2E-07	-0,10939	0,117	0,243	0,008198
Abhd5	5,21E-07	-0,14141	0,547	0,715	0,008226
Uros	5,21E-07	-0,08786	0,099	0,215	0,008226
Erg28	5,22E-07	-0,13619	0,594	0,745	0,008242
Fan1	5,23E-07	-0,11757	0,141	0,27	0,008251
Zfp428	5,24E-07	-0,07347	0,064	0,167	0,008272
Mrpl2	5,26E-07	-0,11218	0,268	0,43	0,008293
Rab39b	5,26E-07	-0,10458	0,295	0,468	0,008293
Vapa	5,28E-07	-0,11928	0,954	0,96	0,008324
Etfb	5,28E-07	-0,14546	0,543	0,696	0,008327
Zfp141	5,29E-07	-0,08089	0,05	0,141	0,008353
Tprgl	5,3E-07	-0,14309	0,855	0,924	0,008358
Fopnl	5,31E-07	-0,1249	0,422	0,599	0,008381
Alkbh1	5,37E-07	-0,13086	0,211	0,357	0,00847
Lpin1	5,38E-07	0,291942	0,499	0,399	0,00848
Ripk1	5,39E-07	0,197686	0,231	0,118	0,008501
Fam53a	5,4E-07	-0,125	0,218	0,363	0,008524
Ahcyl1	5,48E-07	-0,13047	0,754	0,876	0,008643
Gstp2	5,48E-07	0,184965	0,138	0,046	0,008646
Cyb561d2	5,5E-07	-0,10459	0,235	0,395	0,00868
Erp44	5,51E-07	-0,11749	0,352	0,527	0,008685

Cep104	5,54E-07	-0,10416	0,283	0,447	0,008733
B430010I2	5,55E-07	-0,08409	0,187	0,333	0,008763
Sqor	5,59E-07	-0,07575	0,029	0,108	0,008818
C1qbp	5,64E-07	-0,14929	0,528	0,675	0,008895
Zfp511	5,66E-07	-0,1095	0,167	0,308	0,00893
Pdia4	5,67E-07	-0,15194	0,527	0,69	0,008948
Cep290	5,69E-07	-0,11975	0,207	0,367	0,008974
Tsr3	5,7E-07	-0,12102	0,228	0,378	0,008996
Srsf5	5,87E-07	0,180029	0,98	0,966	0,009268
Scd2	5,93E-07	-0,14603	1	0,998	0,009348
Tmem70	5,96E-07	-0,12061	0,215	0,361	0,009409
Gm26917	5,98E-07	0,260633	0,543	0,441	0,009434
Uchl5	6E-07	-0,13227	0,209	0,35	0,009463
Trmt6	6,04E-07	-0,1125	0,264	0,435	0,00953
Olfr671	6,09E-07	-0,10859	0,062	0,16	0,009606
Fam171a1	6,09E-07	-0,1301	0,246	0,403	0,009615
Ext2	6,11E-07	-0,11335	0,262	0,418	0,009642
Chmp5	6,12E-07	-0,14078	0,84	0,905	0,009661
Vdac3	6,13E-07	-0,14611	0,82	0,903	0,009668
Mtmr2	6,16E-07	0,209294	0,809	0,751	0,009719
Tssc4	6,17E-07	-0,08676	0,257	0,422	0,00973
Manbal	6,17E-07	-0,13019	0,472	0,641	0,009736
Pdcd10	6,26E-07	-0,13447	0,756	0,859	0,009873
Sass6	6,29E-07	-0,13066	0,27	0,432	0,009925
Mcts2	6,36E-07	-0,09543	0,088	0,198	0,010036
Actr6	6,38E-07	-0,10931	0,235	0,395	0,010067
Sdhd	6,45E-07	-0,12051	0,367	0,546	0,010178
Hsd17b11	6,53E-07	-0,16207	0,554	0,707	0,010296
1110012L1	6,54E-07	-0,09298	0,134	0,264	0,010313
Rpl6	6,55E-07	-0,11632	0,996	0,996	0,010332
Psmd14	6,59E-07	-0,1231	0,461	0,648	0,010402
Zfp771	6,61E-07	-0,19496	0,728	0,819	0,01043
Mgst3	6,64E-07	-0,47858	0,455	0,563	0,01047
Tyw3	6,74E-07	-0,07563	0,035	0,116	0,01064
Rnf157	6,81E-07	-0,12185	0,121	0,241	0,010745
BC017643	6,87E-07	-0,12633	0,213	0,354	0,010836
Praf2	6,96E-07	-0,11545	0,407	0,601	0,010987
Tac2	6,97E-07	-0,04537	0	0,044	0,010989
Trim59	6,98E-07	0,211458	0,978	0,964	0,011006
Gnpda2	7,03E-07	-0,13094	0,321	0,487	0,011098
Hmgn5	7,05E-07	-0,11616	0,141	0,268	0,011125
Poc1a	7,08E-07	0,259329	0,514	0,407	0,011169
Mvb12a	7,13E-07	-0,1501	0,391	0,555	0,011248
Gm20515	7,14E-07	-0,09322	0,044	0,131	0,011268
Edem2	7,15E-07	-0,13032	0,497	0,667	0,011287
Cpne2	7,2E-07	-0,0754	0,028	0,103	0,01136
Hist1h4h	7,26E-07	-0,15013	0,143	0,266	0,011457
Tst	7,27E-07	-0,14079	0,321	0,481	0,01147
Meg3	7,28E-07	-0,05025	0,006	0,059	0,011481
Strn	7,28E-07	-0,14417	0,633	0,781	0,011483
Ndufv3	7,34E-07	-0,14178	0,743	0,85	0,011578
Gm39469	7,34E-07	-0,10098	0,055	0,148	0,011581

Gid8	7,38E-07	-0,09226	0,228	0,382	0,01164
Tmem219	7,42E-07	-0,11818	0,308	0,483	0,011705
2310039H(	7,58E-07	-0,10529	0,114	0,232	0,011953
Tsacc	7,61E-07	-0,07469	0,042	0,129	0,012005
Dusp8	7,64E-07	-0,10907	0,325	0,5	0,012048
Wnt3	7,66E-07	-0,12079	0,286	0,451	0,012087
Btc	7,82E-07	-0,0683	0,075	0,179	0,01234
Trap1	7,86E-07	-0,11799	0,27	0,42	0,012397
Zmat5	7,88E-07	-0,11739	0,47	0,648	0,012426
1110038F1	7,89E-07	-0,1107	0,327	0,502	0,012442
Tctex1d2	7,96E-07	-0,10471	0,138	0,266	0,012563
Ranbp9	7,99E-07	-0,13042	0,284	0,439	0,0126
Mrps26	8,02E-07	-0,1462	0,429	0,586	0,012653
Scly	8,02E-07	-0,13958	0,257	0,405	0,012653
Prelid1	8,05E-07	-0,1439	0,446	0,62	0,012706
Gpatch11	8,09E-07	-0,10085	0,316	0,5	0,012763
Srsf6	8,09E-07	0,182679	0,734	0,662	0,012765
Synj2	8,17E-07	-0,12334	0,407	0,591	0,012896
Prickle1	8,2E-07	0,331182	0,508	0,401	0,01293
Fam213a	8,28E-07	-0,16943	0,598	0,736	0,013062
Upp2	8,33E-07	-0,10443	0,103	0,215	0,013139
Gstz1	8,33E-07	-0,12731	0,261	0,414	0,013144
Rps27l	8,35E-07	-0,11603	0,697	0,812	0,013169
Agk	8,35E-07	-0,08534	0,152	0,287	0,013178
Mkln1	8,43E-07	0,217371	0,754	0,703	0,013295
Gm5914	8,48E-07	-0,08619	0,05	0,139	0,013383
Slc35a5	8,66E-07	-0,14562	0,598	0,757	0,013664
B230209E1	8,67E-07	-0,10043	0,037	0,118	0,013673
Tbck	8,8E-07	-0,11108	0,29	0,458	0,013891
Tmem205	8,81E-07	-0,14561	0,611	0,751	0,013901
Nptxr	8,87E-07	0,079372	0,07	0,008	0,013993
Vcp	8,87E-07	0,148767	0,991	0,981	0,014
Cluap1	8,97E-07	-0,12296	0,255	0,407	0,014154
Timp3	8,97E-07	-0,10156	0,02	0,089	0,014157
Sdr42e1	8,98E-07	-0,07819	0,04	0,124	0,014171
Plpp1	9,21E-07	-0,09029	0,084	0,192	0,014524
Usp9x	9,22E-07	0,208936	0,873	0,833	0,014539
Gatb	9,22E-07	-0,07106	0,079	0,186	0,01454
Rcbtb1	9,26E-07	-0,17374	0,901	0,937	0,014605
Mpp2	9,38E-07	-0,14154	0,49	0,665	0,014797
Eif1b	9,44E-07	-0,15025	0,815	0,884	0,014885
A330015K(	9,44E-07	-0,14534	0,339	0,508	0,014892
Wdr83	9,55E-07	-0,10821	0,193	0,333	0,015073
Lrrc51	9,57E-07	-0,08159	0,039	0,12	0,0151
Borcs8	9,68E-07	-0,11928	0,152	0,276	0,015275
1500015A(	9,75E-07	-0,07033	0,033	0,112	0,015381
Dnaja3	9,78E-07	-0,1125	0,301	0,466	0,015428
Srsf9	9,81E-07	-0,12981	0,539	0,7	0,015468
Dpm1	9,87E-07	-0,14053	0,541	0,709	0,015568
Atp6v0e	9,89E-07	-0,13692	0,613	0,768	0,015609
Csdc2	9,99E-07	-0,08561	0,062	0,158	0,015764
Vps72	1E-06	-0,1307	0,33	0,489	0,015773

Cnih3	1,02E-06	0,105097	0,101	0,025	0,016113
Efemp1	1,02E-06	-0,12872	0,143	0,266	0,016153
Kifap3	1,05E-06	-0,12126	0,651	0,785	0,016573
2610044O	1,06E-06	-0,09528	0,084	0,19	0,016674
Dynll1	1,06E-06	0,228305	0,998	0,992	0,016682
Prkd3	1,06E-06	0,210712	0,719	0,673	0,016696
Amer2	1,06E-06	-0,17119	0,528	0,681	0,01675
Kif5a	1,06E-06	0,226813	0,374	0,251	0,016765
Elac2	1,06E-06	-0,10153	0,114	0,23	0,016793
Tmed1	1,07E-06	-0,1176	0,27	0,422	0,01689
Rtkn2	1,07E-06	-0,0957	0,273	0,441	0,016921
Zfp442	1,08E-06	-0,07504	0,046	0,133	0,017065
Tmem11	1,08E-06	-0,1007	0,224	0,378	0,017108
Nrbf2	1,09E-06	-0,10415	0,257	0,414	0,017148
Zfp945	1,09E-06	-0,09006	0,099	0,213	0,017167
1190005I0	1,09E-06	-0,15403	0,508	0,675	0,017172
Ndufb7	1,1E-06	-0,1185	0,939	0,977	0,017313
Plp2	1,11E-06	0,123547	0,141	0,051	0,017443
Nudt14	1,11E-06	-0,1063	0,103	0,213	0,017497
Cryzl1	1,11E-06	-0,13632	0,453	0,62	0,017582
Pdcd7	1,12E-06	-0,10189	0,239	0,392	0,017591
Borcs7	1,12E-06	-0,13064	0,295	0,451	0,017645
Elovl1	1,14E-06	-0,17376	0,835	0,884	0,017957
Ndp	1,14E-06	-0,1046	0,094	0,203	0,018029
Thoc3	1,15E-06	-0,12121	0,288	0,449	0,018207
Alad	1,15E-06	-0,10963	0,154	0,281	0,018215
Slc11a2	1,17E-06	-0,11072	0,163	0,295	0,018457
Med21	1,17E-06	-0,16948	0,6	0,751	0,018532
Tmem208	1,18E-06	-0,10745	0,479	0,673	0,018634
Saysd1	1,18E-06	-0,12593	0,18	0,31	0,018648
Spr	1,18E-06	-0,11675	0,393	0,572	0,018677
Abce1	1,19E-06	-0,0934	0,334	0,521	0,018714
Ric8a	1,19E-06	-0,12084	0,341	0,511	0,018792
Dcaf8	1,19E-06	-0,16041	0,659	0,77	0,018835
Elp4	1,2E-06	-0,12378	0,229	0,371	0,018889
Astn2	1,22E-06	0,114906	0,114	0,034	0,019213
Gatsl2	1,22E-06	-0,10923	0,161	0,297	0,019249
Mccc1	1,24E-06	-0,13411	0,25	0,395	0,019595
Exosc9	1,25E-06	-0,11247	0,189	0,323	0,019704
Hdac11	1,25E-06	0,205565	0,912	0,869	0,019785
Cetn2	1,26E-06	-0,1086	0,273	0,432	0,019896
Vps28	1,26E-06	-0,14569	0,683	0,804	0,019911
Exosc4	1,27E-06	-0,12692	0,321	0,483	0,020071
Romo1	1,28E-06	-0,14566	0,62	0,762	0,020172
Thop1	1,28E-06	-0,09796	0,266	0,426	0,02022
Sept1	1,28E-06	-0,12584	0,305	0,462	0,020268
Scand1	1,29E-06	-0,14566	0,789	0,871	0,02028
B9d1	1,29E-06	-0,10245	0,127	0,247	0,020355
Pbx3	1,29E-06	-0,1269	0,288	0,449	0,020376
Rab10os	1,3E-06	-0,1139	0,294	0,458	0,020472
Nfe2l2	1,31E-06	-0,14708	0,439	0,597	0,020717
Ndufs6	1,32E-06	-0,13364	0,868	0,916	0,020805

Mrpl1	1,32E-06	-0,09988	0,174	0,31	0,020822
Smap2	1,32E-06	-0,13486	0,534	0,679	0,02089
Tacc1	1,33E-06	-0,14971	0,571	0,728	0,020948
Med28	1,33E-06	-0,14524	0,514	0,7	0,02103
Hibch	1,34E-06	-0,12351	0,206	0,342	0,021108
Copa	1,36E-06	0,176677	0,749	0,673	0,021525
Tmem56	1,37E-06	-0,11381	0,117	0,234	0,02163
2010315B(	1,37E-06	-0,08628	0,05	0,137	0,021639
Yipf1	1,37E-06	-0,13129	0,382	0,553	0,021679
Slc34a3	1,39E-06	-0,17595	0,134	0,247	0,021879
B3galt5	1,39E-06	-0,18344	0,679	0,791	0,021913
Tmem189	1,4E-06	-0,1426	0,93	0,928	0,022008
Cbr4	1,4E-06	-0,08653	0,083	0,186	0,022058
Bloc1s3	1,41E-06	-0,09681	0,119	0,234	0,022308
Rwdd1	1,42E-06	-0,14229	0,596	0,749	0,022338
Dlk2	1,42E-06	-0,10015	0,039	0,118	0,022389
Cox18	1,42E-06	-0,08889	0,094	0,203	0,022437
Rbck1	1,43E-06	-0,11082	0,393	0,576	0,022631
Snap25	1,44E-06	-0,05438	0,006	0,057	0,022684
Cnbp	1,47E-06	-0,11883	0,952	0,979	0,023126
Rab37	1,47E-06	-0,2508	0,185	0,31	0,023171
Akt1s1	1,49E-06	-0,1132	0,44	0,61	0,023503
Rnh1	1,49E-06	-0,13133	0,495	0,65	0,023549
Ube2a	1,5E-06	-0,14639	0,275	0,418	0,023635
Poldip2	1,5E-06	-0,12439	0,33	0,483	0,023733
Tbca	1,54E-06	-0,11093	0,472	0,639	0,02435
Cops9	1,55E-06	-0,13695	0,91	0,92	0,024396
Mrpl32	1,55E-06	-0,10662	0,294	0,456	0,024483
Amotl1	1,55E-06	0,137805	0,092	0,021	0,024483
1190007I0	1,56E-06	-0,09922	0,132	0,253	0,024652
Mrpl30	1,57E-06	-0,12624	0,587	0,757	0,024701
Cldn34c1	1,58E-06	-0,12396	0,22	0,361	0,024886
Cops4	1,58E-06	-0,12709	0,492	0,662	0,024889
Pdhx	1,58E-06	-0,10909	0,174	0,308	0,024981
Kcnu1	1,6E-06	-0,06766	0,026	0,097	0,025286
Apool	1,61E-06	-0,10579	0,128	0,247	0,025391
Zfp942	1,62E-06	-0,09876	0,084	0,188	0,025618
Ptprk	1,63E-06	-0,12047	0,448	0,629	0,025683
Sgk2	1,63E-06	-0,11034	0,198	0,338	0,025708
Tsg101	1,65E-06	-0,1378	0,606	0,747	0,026089
Polr3k	1,66E-06	-0,10553	0,231	0,38	0,026123
Vipas39	1,67E-06	-0,10679	0,233	0,38	0,026272
Smyd4	1,67E-06	-0,07184	0,101	0,215	0,026379
Ndufa10	1,67E-06	-0,15261	0,763	0,84	0,026412
Mrpl23	1,68E-06	-0,16211	0,648	0,759	0,026546
Psmg3	1,68E-06	-0,11253	0,251	0,397	0,026579
Sorcs3	1,69E-06	-0,10491	0,062	0,154	0,026646
BC003331	1,7E-06	-0,09503	0,389	0,589	0,026761
Tmem245	1,7E-06	0,20693	0,714	0,639	0,02679
Rnf170	1,72E-06	-0,09388	0,18	0,319	0,027072
Mrpl27	1,72E-06	-0,12401	0,404	0,591	0,027129
Pcyox1l	1,73E-06	-0,14614	0,283	0,424	0,02725

Nudt7	1,74E-06	-0,09459	0,072	0,169	0,027468
Zfp322a	1,75E-06	-0,10773	0,149	0,276	0,02755
Caml	1,77E-06	-0,12541	0,389	0,561	0,027906
Alg1	1,77E-06	-0,10386	0,198	0,34	0,027936
Slpi	1,79E-06	-0,12507	0,112	0,224	0,028239
Gulp1	1,79E-06	0,100426	0,103	0,027	0,028249
Chchd7	1,81E-06	-0,11886	0,328	0,489	0,028548
Fam49b	1,82E-06	-0,15549	0,578	0,698	0,028647
0610012G	1,82E-06	-0,11639	0,422	0,593	0,028656
Irf9	1,82E-06	0,200321	0,207	0,101	0,028786
Mrto4	1,85E-06	-0,08188	0,389	0,58	0,029195
Nipsnap3b	1,85E-06	-0,13142	0,481	0,641	0,029215
A830010M	1,85E-06	-0,11728	0,393	0,572	0,029242
Snrnp27	1,87E-06	-0,13801	0,673	0,814	0,029462
Timm10	1,87E-06	-0,09721	0,198	0,335	0,029527
Polr2g	1,92E-06	-0,12397	0,574	0,73	0,030317
Rap1a	1,92E-06	-0,15541	0,994	0,996	0,030319
Clpb	1,93E-06	-0,12662	0,303	0,46	0,030377
Ubap2l	1,93E-06	0,210289	0,719	0,658	0,030439
Sssca1	1,93E-06	-0,1091	0,371	0,549	0,030514
Esd	1,95E-06	-0,13772	0,547	0,717	0,030705
Pdlim1	1,95E-06	-0,15	0,444	0,597	0,030743
Tmem175	1,95E-06	-0,09541	0,139	0,264	0,030749
Tax1bp1	2E-06	-0,1585	0,804	0,873	0,031533
Tnfsf9	2E-06	0,163729	0,147	0,057	0,031549
Cdc34	2,01E-06	-0,10981	0,202	0,34	0,031774
Smap1	2,04E-06	-0,15416	0,556	0,662	0,032127
Rnpep	2,06E-06	-0,12623	0,268	0,418	0,032479
Rbms1	2,06E-06	0,270615	0,345	0,226	0,032536
Plrg1	2,07E-06	-0,12433	0,259	0,405	0,032586
Aldh7a1	2,08E-06	-0,11257	0,229	0,376	0,032806
0610037L1	2,1E-06	-0,10761	0,385	0,561	0,033122
Ncl	2,11E-06	0,201902	0,976	0,97	0,033211
Adamts20	2,13E-06	-0,08373	0,106	0,219	0,033579
Tmod1	2,13E-06	0,218278	0,961	0,861	0,033617
Pcsk1n	2,13E-06	-0,17337	0,939	0,945	0,033622
Plxna1	2,13E-06	0,133174	0,2	0,097	0,033627
Isoc2a	2,14E-06	-0,09954	0,145	0,268	0,033801
Atp5d	2,15E-06	-0,11787	0,956	0,962	0,033911
Creld2	2,16E-06	-0,08775	0,189	0,331	0,034148
Vps16	2,19E-06	-0,08771	0,246	0,401	0,03458
Rgs3	2,2E-06	0,199956	0,771	0,726	0,03464
Mtm1	2,21E-06	-0,1286	0,248	0,39	0,03482
Gpr89	2,24E-06	-0,11978	0,231	0,369	0,0353
Snhg6	2,24E-06	-0,13636	0,464	0,622	0,03537
Tmed3	2,25E-06	-0,14647	0,512	0,679	0,035437
Atp8a1	2,25E-06	0,153038	0,987	0,975	0,0355
Yif1b	2,26E-06	-0,12114	0,539	0,694	0,035645
Dusp3	2,28E-06	-0,17677	0,85	0,878	0,035951
Hpf1	2,29E-06	-0,12734	0,426	0,584	0,036088
Dync1i1	2,29E-06	-0,08846	0,088	0,192	0,036131
Etfa	2,32E-06	-0,09745	0,215	0,361	0,036595

Parp8	2,38E-06	-0,09903	0,095	0,2	0,037484
Gm26724	2,38E-06	-0,07404	0,077	0,177	0,037489
Ccndbp1	2,38E-06	-0,13684	0,565	0,715	0,037497
Polr2m	2,4E-06	-0,10436	0,354	0,53	0,037875
Tbce	2,44E-06	-0,09998	0,242	0,401	0,038463
Wdr77	2,44E-06	-0,13385	0,217	0,346	0,038492
Uri1	2,45E-06	-0,09358	0,316	0,485	0,038716
Ankrd13a	2,46E-06	0,19877	0,804	0,757	0,038828
Fam122a	2,51E-06	-0,10332	0,187	0,321	0,039532
Cbx5	2,51E-06	0,210162	0,692	0,637	0,039648
Ap5s1	2,53E-06	-0,08739	0,073	0,169	0,039972
Msn	2,58E-06	0,204286	0,655	0,559	0,040625
Rpp25l	2,58E-06	-0,10646	0,228	0,369	0,040628
Wnk1	2,59E-06	0,147042	1	0,998	0,040789
Ndufaf4	2,62E-06	-0,11893	0,182	0,31	0,041262
Slc35b3	2,62E-06	-0,09743	0,127	0,243	0,041373
Coa6	2,64E-06	-0,10521	0,262	0,411	0,041676
Sv2a	2,66E-06	-0,13757	0,996	0,994	0,041984
Fam104a	2,68E-06	-0,122	0,446	0,616	0,042356
Fibp	2,69E-06	-0,12277	0,24	0,382	0,042407
Trappc1	2,71E-06	-0,13134	0,255	0,395	0,042703
Cenpv	2,72E-06	-0,1121	0,169	0,295	0,042887
Efna2	2,74E-06	-0,10139	0,207	0,344	0,043253
Bet1l	2,74E-06	-0,10427	0,31	0,475	0,043282
Smim14	2,76E-06	-0,14538	0,811	0,867	0,043574
Gps1	2,78E-06	-0,11833	0,554	0,719	0,043808
Ctsf	2,78E-06	-0,10309	0,396	0,572	0,043849
Cdkl5	2,86E-06	-0,14065	0,426	0,595	0,045063
Cnpy3	2,88E-06	-0,10804	0,437	0,612	0,045432
Gpr137b	2,88E-06	-0,14632	0,343	0,511	0,045477
Mlycd	2,91E-06	-0,10783	0,183	0,314	0,045832
Klhdc2	2,92E-06	-0,11395	0,49	0,654	0,046085
Ube2g2	2,93E-06	-0,09715	0,182	0,316	0,046194
Gm45356	2,94E-06	-0,07122	0,028	0,097	0,046405
Cep41	2,95E-06	-0,08551	0,081	0,181	0,046476
Rnf181	3E-06	-0,13958	0,732	0,827	0,047329
Rbm18	3,02E-06	-0,09809	0,332	0,494	0,047646
Cdkn1a	3,02E-06	0,330219	0,189	0,089	0,047697
Sephs2	3,06E-06	-0,11226	0,183	0,31	0,048335
Sh3gl2	3,08E-06	-0,10437	0,084	0,184	0,04864
Pmf1	3,09E-06	-0,07046	0,039	0,116	0,048739
Emc9	3,11E-06	-0,10459	0,217	0,354	0,049089
Emd	3,11E-06	-0,1116	0,288	0,439	0,049123
Enpp6	3,16E-06	0,148616	0,938	0,751	0,049807
Chm	3,16E-06	-0,09334	0,325	0,496	0,049841
Rnf214	3,16E-06	-0,09673	0,264	0,42	0,04986
Lss	3,17E-06	0,30301	0,402	0,3	0,05008
Paip1	3,18E-06	-0,10393	0,622	0,785	0,050125
Tada2a	3,2E-06	-0,10085	0,244	0,388	0,050444
Nbdy	3,2E-06	-0,09835	0,108	0,217	0,050507
Kif1bp	3,2E-06	-0,13126	0,866	0,922	0,050525
Tmem129	3,23E-06	-0,1009	0,167	0,293	0,051022

Nme1	3,27E-06	-0,1467	0,602	0,734	0,051551
Ppie	3,28E-06	-0,08363	0,18	0,31	0,05174
Dzip3	3,29E-06	-0,07949	0,18	0,314	0,051922
Acy1	3,3E-06	-0,11899	0,273	0,428	0,051992
Grpel1	3,3E-06	-0,12845	0,528	0,677	0,052109
Supt4a	3,3E-06	-0,14037	0,694	0,812	0,052111
Adgrv1	3,33E-06	-0,11711	0,202	0,335	0,052461
Fbxo22	3,33E-06	-0,12367	0,228	0,359	0,052487
Rfesd	3,34E-06	-0,09103	0,11	0,219	0,052645
Tap1	3,35E-06	0,132755	0,055	0,004	0,052841
Gm36356	3,38E-06	-0,0901	0,051	0,135	0,05333
Itpa	3,39E-06	-0,12084	0,294	0,443	0,053494
Gse1	3,43E-06	0,243979	0,484	0,399	0,054125
Dnajb12	3,46E-06	-0,11666	0,473	0,641	0,054571
Frs3	3,48E-06	-0,08714	0,145	0,268	0,054825
Gpr62	3,49E-06	-0,1454	0,859	0,914	0,055133
Zfp958	3,5E-06	-0,07711	0,05	0,133	0,055288
Scg5	3,51E-06	-0,21703	0,622	0,734	0,055337
Dscr3	3,53E-06	-0,10634	0,182	0,314	0,0557
Ephb2	3,54E-06	0,097137	0,081	0,017	0,055829
Casq1	3,54E-06	-0,0873	0,068	0,16	0,055861
Sccpdh	3,55E-06	-0,2509	0,945	0,943	0,056021
Clstn1	3,58E-06	0,201147	0,763	0,711	0,056462
Ptma	3,58E-06	0,126424	1	0,998	0,056472
Nudc	3,6E-06	-0,12101	0,835	0,901	0,056728
Gapdh	3,6E-06	-0,14521	0,998	0,998	0,056829
Polr2f	3,63E-06	-0,11696	0,965	0,97	0,057244
Gm13293	3,65E-06	-0,12411	0,292	0,445	0,057513
Pitpna	3,66E-06	-0,13415	0,578	0,717	0,057672
Tubgcp5	3,68E-06	-0,11357	0,198	0,331	0,058081
Stt3a	3,69E-06	0,192555	0,714	0,65	0,058168
Map6d1	3,7E-06	-0,15496	0,717	0,806	0,058335
Tmem234	3,7E-06	-0,10976	0,927	0,937	0,058349
Leprot	3,72E-06	-0,13869	0,591	0,709	0,058723
Cnot1	3,73E-06	0,202962	0,569	0,485	0,058826
Thap3	3,74E-06	-0,12135	0,303	0,451	0,059053
Vwa1	3,78E-06	0,309974	0,36	0,253	0,059687
Dexi	3,81E-06	-0,10096	0,167	0,293	0,060153
Zfp800	3,86E-06	-0,11411	0,367	0,536	0,060824
Paqr7	3,87E-06	-0,09534	0,136	0,253	0,060977
Plec	3,87E-06	0,08931	0,088	0,021	0,060992
Trim2	3,87E-06	-0,13759	0,967	0,981	0,0611
Tmem63a	3,88E-06	-0,12786	0,956	0,981	0,061239
Mlf2	3,9E-06	-0,11199	0,976	0,973	0,061507
Acaa1a	3,9E-06	-0,12999	0,879	0,935	0,061531
Tle3	3,91E-06	0,314697	0,525	0,449	0,06171
Spag7	3,93E-06	-0,12051	0,437	0,603	0,062045
Gpr37l1	4E-06	-0,07059	0,017	0,076	0,063139
Sil1	4,09E-06	-0,12895	0,295	0,437	0,064563
Hint2	4,09E-06	-0,10911	0,244	0,388	0,064597
Srd5a3	4,15E-06	-0,12035	0,261	0,405	0,065425
2700062C(	4,15E-06	-0,11145	0,202	0,331	0,065469

AU041133	4,18E-06	-0,07046	0,037	0,112	0,065872
Snx18	4,23E-06	-0,14838	0,4	0,557	0,066715
H1fx	4,25E-06	-0,08653	0,068	0,16	0,067056
Klf9	4,26E-06	-0,14079	0,749	0,842	0,067261
Tmem163	4,31E-06	-0,14844	0,16	0,281	0,068009
Fabp5	4,33E-06	-0,15978	0,994	0,998	0,068285
Nop16	4,36E-06	-0,09774	0,167	0,291	0,068765
Naca	4,37E-06	-0,12081	0,976	0,981	0,068892
Tubb2a	4,37E-06	-0,14796	0,165	0,289	0,068937
Wsb1	4,43E-06	0,237937	0,51	0,409	0,069919
Ubl7	4,58E-06	-0,09838	0,299	0,449	0,072179
Zfp930	4,58E-06	-0,06883	0,042	0,12	0,072223
Mrps30	4,64E-06	-0,09824	0,15	0,27	0,07315
9330159F1	4,64E-06	-0,13489	0,319	0,468	0,073247
Tomm40	4,67E-06	-0,09733	0,371	0,538	0,073611
Etfrf1	4,72E-06	-0,09788	0,273	0,424	0,074387
Snx21	4,76E-06	-0,09235	0,174	0,302	0,075029
Akirin1	4,81E-06	-0,15069	0,609	0,738	0,075922
Ccl25	4,82E-06	-0,08766	0,073	0,167	0,075974
Zfp617	4,82E-06	-0,10207	0,156	0,276	0,07603
mt-Co3	4,85E-06	-0,09657	1	1	0,076526
Tcta	4,94E-06	-0,10627	0,213	0,348	0,077931
Ppme1	4,94E-06	-0,12275	0,354	0,523	0,077937
Rmi1	4,94E-06	-0,08551	0,084	0,184	0,077955
Lpcat3	4,96E-06	-0,0993	0,189	0,321	0,078191
Lrrc42	4,97E-06	-0,10196	0,15	0,27	0,078415
Cdk5	5,02E-06	-0,14577	0,648	0,762	0,079166
Rpl39	5,03E-06	-0,09553	0,993	0,985	0,079281
Slc50a1	5,04E-06	-0,13651	0,679	0,814	0,079491
Orc4	5,07E-06	-0,11787	0,275	0,418	0,079926
Spcs2	5,08E-06	-0,11681	0,936	0,973	0,080214
Cog6	5,12E-06	-0,07942	0,16	0,289	0,080817
Src	5,13E-06	0,114337	0,156	0,065	0,080939
Rnf149	5,17E-06	-0,11287	0,283	0,426	0,081619
Hebp1	5,19E-06	0,232886	0,747	0,671	0,081807
Pnmal1	5,27E-06	-0,08675	0,057	0,141	0,083114
Dmac2	5,3E-06	-0,0952	0,121	0,232	0,083611
Cops3	5,36E-06	-0,13538	0,479	0,631	0,084488
Ap3m1	5,37E-06	-0,10786	0,442	0,624	0,084717
Fnip1	5,37E-06	0,207581	0,727	0,7	0,084749
Clip3	5,4E-06	0,145376	0,969	0,932	0,085195
Sorcs1	5,42E-06	-0,09603	0,228	0,365	0,085472
Gm14326	5,42E-06	-0,08865	0,064	0,152	0,085514
Plpp6	5,43E-06	-0,09113	0,081	0,175	0,085667
Tfam	5,46E-06	-0,0976	0,286	0,441	0,086074
Wdr60	5,5E-06	-0,1076	0,193	0,323	0,086698
Kdm5d	5,6E-06	-0,08626	0,068	0,158	0,088347
Tusc2	5,65E-06	-0,11646	0,273	0,416	0,089058
Tmem161a	5,66E-06	-0,1113	0,35	0,515	0,089295
Cr1l	5,75E-06	-0,12969	0,323	0,477	0,090716
Tceal8	5,78E-06	-0,08018	0,257	0,407	0,091145
Stard13	5,78E-06	-0,09519	0,183	0,314	0,091188

Pxk	5,79E-06	-0,10252	0,339	0,498	0,091392
Kctd3	5,8E-06	-0,15391	0,763	0,861	0,091523
Selenom	5,8E-06	-0,14181	0,963	0,973	0,091567
Zdhhc4	5,83E-06	-0,10288	0,176	0,302	0,091934
Letm2	5,83E-06	-0,10911	0,235	0,382	0,091998
Gm11808	5,89E-06	0,206725	0,644	0,584	0,092978
Daam1	5,91E-06	0,173416	0,95	0,873	0,093184
Psmb7	5,92E-06	-0,12019	0,818	0,888	0,093428
Fgf7	5,99E-06	0,141363	0,163	0,072	0,094517
Tmem184c	6E-06	-0,13543	0,462	0,622	0,094655
Adamtsl4	6,02E-06	-0,13796	0,606	0,759	0,094993
Acot8	6,11E-06	-0,09939	0,161	0,283	0,09633
Lrrn3	6,11E-06	-0,07234	0,037	0,11	0,096464
Ube2d1	6,12E-06	-0,12226	0,932	0,93	0,096535
Rheb	6,17E-06	-0,12579	0,534	0,688	0,097387
Nubp2	6,19E-06	-0,08593	0,321	0,485	0,097576
Gpatch2	6,19E-06	-0,09112	0,257	0,401	0,097713
Zfp593	6,2E-06	-0,08845	0,141	0,257	0,097778
Adam11	6,23E-06	-0,1471	0,13	0,241	0,098346
Nfic	6,25E-06	0,188318	0,941	0,907	0,098672
Sec22b	6,26E-06	-0,09574	0,31	0,466	0,098744
Pafah1b1	6,38E-06	-0,10921	0,998	0,998	0,100658
Mthfd1	6,41E-06	-0,09645	0,202	0,335	0,10109
Caskin2	6,49E-06	-0,0969	0,231	0,373	0,102347
Sbno1	6,5E-06	0,194861	0,796	0,757	0,102549
Taf12	6,51E-06	-0,10548	0,235	0,371	0,102778
Rfc4	6,56E-06	-0,08802	0,081	0,175	0,103507
Rabl3	6,58E-06	-0,09856	0,114	0,219	0,10384
Wdr61	6,61E-06	-0,10835	0,323	0,475	0,104262
Osgep	6,61E-06	-0,12311	0,391	0,54	0,104335
Tmem147	6,64E-06	-0,11027	0,692	0,825	0,104772
Slc25a39	6,66E-06	-0,10912	0,316	0,468	0,105137
Hist1h1c	6,67E-06	-0,12689	0,13	0,245	0,10528
Tmem98	6,7E-06	-0,14475	0,655	0,797	0,105646
Zfp27	6,7E-06	-0,07059	0,042	0,118	0,105659
Msantd4	6,73E-06	-0,10586	0,277	0,422	0,106101
Atf7ip	6,75E-06	0,210765	0,752	0,698	0,106418
Tubd1	6,78E-06	-0,06466	0,037	0,11	0,106958
Btbd10	6,78E-06	-0,10081	0,229	0,365	0,106977
Eci1	6,79E-06	-0,11377	0,45	0,624	0,10707
Hspa1a	6,84E-06	0,310782	0,486	0,361	0,107837
Smad7	6,89E-06	0,16719	0,98	0,979	0,108694
Adrm1	6,9E-06	-0,12154	0,479	0,635	0,108903
Akr1e1	6,91E-06	-0,0832	0,083	0,177	0,108982
Gcfc2	6,92E-06	-0,09259	0,086	0,184	0,109248
Zfp523	6,95E-06	-0,08101	0,167	0,295	0,109712
Prex1	6,98E-06	-0,13422	0,418	0,576	0,110174
Gpr137	7E-06	-0,09031	0,248	0,395	0,110446
Fgfr1	7,03E-06	-0,08214	0,103	0,209	0,110971
Pigs	7,05E-06	-0,09876	0,305	0,456	0,111163
Crybg3	7,06E-06	-0,16171	0,385	0,523	0,111418
Imp4	7,13E-06	-0,10568	0,462	0,627	0,112544

Hilpda	7,21E-06	-0,07222	0,039	0,112	0,113667
Rgcc	7,26E-06	-0,04372	0,004	0,046	0,114534
Nosip	7,28E-06	-0,10198	0,314	0,462	0,114859
Gorab	7,36E-06	-0,09004	0,083	0,177	0,116046
Zfp992	7,36E-06	-0,0629	0,033	0,103	0,116155
Coq9	7,41E-06	-0,07649	0,185	0,316	0,116931
6530409C:	7,43E-06	-0,06682	0,033	0,103	0,117291
Rnps1	7,44E-06	-0,09468	0,323	0,487	0,117408
Aqp4	7,45E-06	-0,0519	0,004	0,046	0,11751
Hsd17b4	7,47E-06	-0,13875	0,673	0,778	0,117858
Slc24a4	7,47E-06	0,07769	0,057	0,006	0,117925
Ano4	7,5E-06	-0,16847	0,574	0,694	0,118375
Wipf1	7,53E-06	0,193913	0,613	0,517	0,118802
Rabl6	7,54E-06	-0,11611	0,338	0,483	0,118904
Usp31	7,54E-06	-0,1323	0,783	0,854	0,118975
Fam107b	7,55E-06	-0,11928	0,149	0,259	0,119154
Desi1	7,59E-06	-0,1774	0,963	0,979	0,119732
Commd8	7,59E-06	-0,12804	0,488	0,635	0,119813
Snapc5	7,65E-06	-0,11294	0,501	0,648	0,120608
Zfp605	7,65E-06	-0,08334	0,134	0,249	0,120671
Cbs	7,66E-06	-0,214	0,161	0,272	0,120888
Id2	7,67E-06	0,231644	0,141	0,059	0,120988
Tmem29	7,7E-06	-0,09603	0,156	0,274	0,121427
Dlg1	7,74E-06	0,160172	0,872	0,774	0,12214
Rab28	7,75E-06	-0,07477	0,244	0,39	0,122269
Pus3	7,78E-06	-0,08497	0,106	0,211	0,122772
Faap24	7,91E-06	-0,08802	0,086	0,181	0,124851
Csnk1g3	7,92E-06	-0,11853	0,334	0,487	0,124978
Ppp2cb	7,96E-06	-0,10117	0,204	0,331	0,125514
Efl1	7,97E-06	-0,09094	0,156	0,274	0,125739
Ptdss2	8,04E-06	-0,09612	0,306	0,458	0,126879
Opalin	8,08E-06	-0,36893	0,802	0,759	0,12748
Morf4l1	8,1E-06	-0,09604	0,998	0,996	0,127751
Rc3h1	8,11E-06	0,260022	0,624	0,58	0,127952
Gm13889	8,17E-06	-0,0796	0,051	0,131	0,128896
Psmb1	8,18E-06	-0,11389	0,941	0,973	0,129085
Prpsap1	8,2E-06	-0,12835	0,607	0,736	0,129399
Tub	8,36E-06	-0,03727	0	0,036	0,131872
Tmem167	8,38E-06	-0,13391	0,55	0,717	0,132243
Rilpl2	8,43E-06	-0,09837	0,152	0,268	0,133056
Rtkn	8,44E-06	0,173421	0,881	0,806	0,133103
Nfs1	8,5E-06	-0,09988	0,178	0,302	0,134109
Dnajb11	8,52E-06	-0,11404	0,541	0,681	0,134353
Msrb2	8,53E-06	-0,05968	0,039	0,112	0,134549
1110046JC	8,53E-06	-0,10132	0,16	0,281	0,134573
Zfp449	8,53E-06	-0,06054	0,046	0,124	0,13464
Cdpf1	8,59E-06	-0,10245	0,13	0,241	0,13556
Tprn	8,6E-06	0,18324	0,903	0,863	0,135629
Ehd2	8,62E-06	0,080662	0,081	0,019	0,13606
Plppr5	8,67E-06	-0,04697	0,007	0,055	0,136705
Ppp2r5a	8,8E-06	0,171093	0,73	0,65	0,138819
Dhx9	8,83E-06	0,201529	0,598	0,534	0,139234

Orc6	8,83E-06	-0,11924	0,27	0,407	0,139297
Zfp512	8,83E-06	-0,10616	0,312	0,464	0,139352
Aurkaip1	8,89E-06	-0,13873	0,651	0,776	0,140247
Sec61a1	8,92E-06	0,202567	0,666	0,601	0,140726
Bag5	9,04E-06	-0,09779	0,248	0,386	0,142563
Cd109	9,08E-06	0,082312	0,061	0,008	0,143243
Akr1a1	9,1E-06	-0,11998	0,921	0,939	0,143493
Pcbp2	9,13E-06	0,152219	0,987	0,966	0,144064
Cyp46a1	9,14E-06	-0,0652	0,039	0,112	0,144118
Smim7	9,17E-06	-0,1349	0,73	0,81	0,144623
Mri1	9,27E-06	-0,08184	0,094	0,192	0,146309
Phax	9,28E-06	-0,10266	0,446	0,627	0,146459
Dbt	9,4E-06	-0,10775	0,262	0,407	0,148244
Exosc5	9,42E-06	-0,0976	0,283	0,428	0,148598
2810004N1	9,45E-06	-0,11695	0,378	0,536	0,149053
Serbp1	9,55E-06	0,171684	0,976	0,956	0,150594
Mast3	9,57E-06	0,187839	0,853	0,797	0,150943
Brcc3	9,67E-06	-0,10653	0,284	0,426	0,152531
Acp1	9,74E-06	-0,12913	0,556	0,698	0,1536
Hmgxb4	9,78E-06	-0,10549	0,253	0,388	0,154252
Rpa1	9,8E-06	-0,11492	0,354	0,5	0,154579
Nudt18	9,83E-06	-0,10138	0,176	0,297	0,155048
Dcun1d5	9,88E-06	-0,09798	0,36	0,527	0,155814
Letmd1	9,88E-06	-0,0944	0,264	0,407	0,155922
Tomm22	9,9E-06	-0,12683	0,661	0,791	0,156165
Pet100	9,94E-06	-0,13524	0,763	0,844	0,156817
N6amt1	9,99E-06	-0,10991	0,139	0,249	0,157588
Tcp11l1	1,01E-05	-0,08888	0,213	0,346	0,15858
Ube2m	1,01E-05	-0,11627	0,701	0,814	0,158877
Pfdn6	1,01E-05	-0,11529	0,791	0,892	0,159307
Egln1	1,02E-05	-0,08059	0,479	0,658	0,160435
Ccdc94	1,02E-05	-0,06494	0,055	0,137	0,161512
Ggh	1,03E-05	-0,12037	0,462	0,622	0,161826
Fastkd1	1,03E-05	-0,08461	0,103	0,205	0,162691
Hras	1,03E-05	-0,11288	0,47	0,629	0,162862
Eya4	1,04E-05	0,211496	0,259	0,156	0,16383
AC129328.	1,05E-05	-0,06233	0,028	0,093	0,164872
Rps15	1,05E-05	0,151004	0,996	0,996	0,165047
Ciao1	1,05E-05	-0,10936	0,262	0,399	0,165232
3110009E1	1,05E-05	-0,08662	0,09	0,186	0,1654
Wdr20	1,05E-05	-0,14875	0,479	0,61	0,165722
Tcp11l2	1,05E-05	-0,11412	0,235	0,365	0,165783
BC051226	1,05E-05	-0,07845	0,116	0,224	0,166282
Kif3c	1,06E-05	-0,10119	0,464	0,616	0,166855
Srsf4	1,06E-05	-0,12142	0,297	0,437	0,16689
Bod1	1,06E-05	-0,1044	0,396	0,555	0,167141
Cox7a2l	1,06E-05	-0,13448	0,749	0,827	0,16741
Cd47	1,07E-05	-0,10398	0,982	0,989	0,168514
6430573F1	1,07E-05	-0,05729	0,018	0,076	0,169191
Nr2c2ap	1,07E-05	-0,09259	0,328	0,483	0,169305
Gatd1	1,07E-05	-0,1003	0,25	0,384	0,16946
Ubl4a	1,08E-05	-0,10071	0,202	0,329	0,169838

Sf3a3	1,08E-05	-0,09996	0,334	0,479	0,170889
Arpin	1,09E-05	-0,08698	0,092	0,188	0,171425
Spred2	1,09E-05	-0,06625	0,068	0,156	0,17178
Polr2j	1,1E-05	-0,11284	0,589	0,747	0,173942
Wdr45	1,12E-05	-0,09662	0,286	0,432	0,176206
Crebrf	1,12E-05	0,183649	0,774	0,69	0,176875
Rasa3	1,13E-05	0,171858	0,782	0,736	0,177646
Ldah	1,13E-05	-0,14717	0,648	0,759	0,178029
Ptpn14	1,13E-05	0,119755	0,068	0,013	0,178159
Slk	1,15E-05	0,214505	0,772	0,698	0,180702
Prr18	1,16E-05	0,18413	0,897	0,838	0,182728
Ywhag	1,16E-05	0,177517	0,745	0,66	0,183203
Arfip1	1,17E-05	-0,11083	0,264	0,399	0,184433
Asphd2	1,17E-05	-0,09338	0,079	0,169	0,184698
Nabp2	1,18E-05	-0,11079	0,65	0,766	0,18591
Alkbh6	1,19E-05	-0,10012	0,194	0,316	0,187051
Ctu2	1,19E-05	-0,08242	0,128	0,236	0,187726
Retsat	1,19E-05	-0,0764	0,07	0,156	0,18792
Fam114a2	1,2E-05	-0,09824	0,406	0,563	0,189032
Sh3bgrl	1,2E-05	-0,10679	0,253	0,392	0,189722
Lmbr1	1,21E-05	-0,10048	0,161	0,276	0,190205
Rgmb	1,21E-05	-0,12937	0,257	0,392	0,190284
Phlda3	1,21E-05	-0,13119	0,901	0,924	0,190329
Mrps15	1,21E-05	-0,10076	0,402	0,574	0,191244
Tbcb	1,22E-05	-0,10793	0,817	0,909	0,192468
Dusp28	1,23E-05	-0,07999	0,09	0,186	0,194465
Taok3	1,24E-05	-0,09639	0,426	0,599	0,194974
Rpf2	1,24E-05	-0,10367	0,163	0,278	0,195154
Tdrd6	1,24E-05	-0,07874	0,051	0,129	0,195295
Hltf	1,24E-05	-0,10238	0,334	0,489	0,196317
Zkscan14	1,25E-05	-0,06726	0,077	0,169	0,197093
Scg3	1,25E-05	-0,07993	0,116	0,222	0,197954
Ccdc90b	1,26E-05	-0,09653	0,22	0,354	0,198287
Zfp579	1,26E-05	-0,12654	0,328	0,481	0,198667
1600014C:	1,26E-05	-0,0843	0,079	0,169	0,198674
Gm17018	1,26E-05	-0,08814	0,262	0,405	0,198911
Ctnnbl1	1,26E-05	-0,09349	0,178	0,3	0,199316
Gna12	1,26E-05	0,140303	0,998	0,996	0,199473
Marc2	1,27E-05	-0,10483	0,459	0,61	0,200968
Gclm	1,29E-05	-0,13625	0,785	0,878	0,20391
Gm16740	1,3E-05	-0,06298	0,02	0,078	0,205671
Lrrfip2	1,31E-05	-0,10251	0,316	0,47	0,206076
Wrap53	1,31E-05	-0,10848	0,36	0,525	0,206519
Hadh	1,32E-05	-0,09621	0,728	0,844	0,208328
AC161376.	1,33E-05	-0,12214	0,204	0,325	0,209243
Scn1b	1,33E-05	0,139524	0,217	0,116	0,209366
Dhx35	1,33E-05	-0,0508	0,022	0,082	0,210546
Rnf219	1,34E-05	-0,07009	0,055	0,135	0,211986
Rhbdl2	1,35E-05	-0,06129	0,035	0,103	0,212382
Gtf3a	1,35E-05	-0,09935	0,417	0,582	0,212624
Svip	1,35E-05	-0,13711	0,631	0,755	0,212649
Rpl7a	1,35E-05	-0,11441	0,972	0,97	0,212769

Hist3h2a	1,35E-05	-0,12053	0,295	0,439	0,212995
Il1rapl1	1,36E-05	-0,12316	0,108	0,205	0,214278
Echdc1	1,38E-05	-0,10721	0,305	0,449	0,217011
Nedd9	1,38E-05	-0,0685	0,053	0,133	0,218074
Tmem246	1,39E-05	-0,08036	0,305	0,466	0,218581
Defb47	1,39E-05	-0,04991	0,006	0,049	0,218947
Rbmxl1	1,4E-05	-0,09812	0,317	0,464	0,221381
Cwf19l2	1,41E-05	-0,10506	0,406	0,561	0,222432
Ptprm	1,41E-05	-0,08988	0,143	0,257	0,222451
Mpzl1	1,43E-05	0,246593	0,728	0,671	0,225478
Adprhl2	1,43E-05	-0,08498	0,261	0,405	0,225496
Ash2l	1,44E-05	-0,10189	0,323	0,47	0,227043
Gnaq	1,45E-05	0,184351	0,804	0,768	0,22863
Fbxo8	1,45E-05	-0,10589	0,321	0,483	0,228965
Notch1	1,45E-05	0,110845	0,11	0,038	0,229113
Pex11b	1,45E-05	-0,09834	0,218	0,346	0,229189
Gls	1,45E-05	0,24146	0,556	0,489	0,229243
Dtd2	1,46E-05	-0,0915	0,218	0,348	0,230135
Yeats4	1,49E-05	-0,09375	0,193	0,316	0,234398
Rdh14	1,5E-05	-0,09838	0,174	0,293	0,236982
Nubp1	1,52E-05	-0,07996	0,119	0,224	0,240013
Mfsd2a	1,52E-05	-0,08783	0,05	0,124	0,240087
Scoc	1,53E-05	-0,13132	0,771	0,861	0,240839
Ccdc32	1,57E-05	-0,10627	0,218	0,338	0,246924
Bola2	1,58E-05	-0,10181	0,648	0,781	0,248496
Nsun6	1,58E-05	-0,08798	0,196	0,325	0,249851
Phf5a	1,58E-05	-0,12131	0,609	0,732	0,249853
Gatad2b	1,59E-05	0,223291	0,798	0,745	0,250816
Nfkbib	1,59E-05	-0,09185	0,235	0,367	0,251438
Mtfr1l	1,61E-05	-0,13433	0,499	0,624	0,253372
Bcat1	1,61E-05	-0,1176	0,281	0,424	0,25361
Setd6	1,61E-05	-0,08712	0,07	0,154	0,254335
Zgpat	1,61E-05	-0,09412	0,134	0,241	0,254394
Gtf2f2	1,62E-05	-0,09609	0,224	0,35	0,255345
Ntpcr	1,63E-05	-0,08985	0,143	0,253	0,257051
Arl6ip1	1,65E-05	-0,10937	0,985	0,998	0,25959
Ccdc124	1,65E-05	-0,11062	0,528	0,681	0,259923
Mrps11	1,65E-05	-0,10306	0,261	0,39	0,259952
Eral1	1,65E-05	-0,08811	0,086	0,177	0,260482
Tusc3	1,65E-05	-0,09374	0,128	0,232	0,261016
Camk2d	1,66E-05	0,179959	0,281	0,175	0,261588
Rabepk	1,66E-05	-0,09144	0,106	0,205	0,261871
Kiz	1,68E-05	-0,1	0,189	0,308	0,264963
Acox1	1,69E-05	0,208788	0,675	0,639	0,266431
Eif2s2	1,69E-05	-0,10944	0,972	0,981	0,267215
Mark1	1,7E-05	-0,10315	0,105	0,2	0,267845
Gcdh	1,7E-05	-0,09774	0,176	0,293	0,267957
Mcrip2	1,7E-05	-0,06653	0,057	0,137	0,268348
Pdik1l	1,7E-05	-0,09103	0,125	0,228	0,268427
Dlat	1,7E-05	-0,09978	0,206	0,331	0,268458
Gamt	1,71E-05	-0,16727	0,833	0,888	0,269989
Tjap1	1,74E-05	-0,11812	0,376	0,519	0,273876

Tmem106b	1,74E-05	-0,12203	0,727	0,827	0,273963
Gm9945	1,75E-05	-0,10103	0,207	0,333	0,275517
Cic	1,75E-05	0,201208	0,554	0,487	0,276019
Bcap31	1,76E-05	-0,11937	0,914	0,947	0,277586
Tex2	1,77E-05	0,187907	0,716	0,669	0,278877
Acot11	1,78E-05	0,133174	0,183	0,093	0,280443
Ptprd	1,78E-05	-0,0869	1	0,996	0,28049
Vrk1	1,78E-05	-0,08215	0,139	0,249	0,281224
Phyh	1,79E-05	-0,11597	0,393	0,546	0,28296
Slc43a2	1,8E-05	-0,09785	0,552	0,726	0,283656
Limch1	1,8E-05	0,12571	0,987	0,964	0,283771
Tirap	1,8E-05	-0,09708	0,196	0,321	0,284462
Dennd1c	1,81E-05	0,088167	0,053	0,006	0,284838
Adpgk	1,81E-05	-0,08627	0,163	0,278	0,286048
Snrpa1	1,84E-05	-0,10444	0,25	0,382	0,289572
C77080	1,84E-05	0,061092	0,053	0,006	0,290121
4732471J0	1,85E-05	-0,08313	0,064	0,146	0,291573
Me2	1,85E-05	-0,10073	0,284	0,422	0,292423
Elp5	1,85E-05	-0,0754	0,248	0,388	0,292573
St7l	1,86E-05	-0,10203	0,51	0,677	0,29395
Mrpl12	1,88E-05	-0,09666	0,501	0,671	0,296357
Foxn2	1,91E-05	-0,11056	0,327	0,475	0,300744
Asb3	1,92E-05	-0,08804	0,141	0,249	0,303685
1700019D0	1,93E-05	-0,07854	0,088	0,181	0,304253
Rps19bp1	1,93E-05	-0,10052	0,283	0,424	0,304479
Zfp780b	1,94E-05	-0,09722	0,154	0,264	0,30528
Sox4	1,94E-05	0,22776	0,172	0,082	0,306799
Ttll5	1,95E-05	-0,12011	0,336	0,477	0,308011
Adprm	1,95E-05	-0,08667	0,125	0,228	0,308023
Spsb1	1,96E-05	0,190416	0,607	0,555	0,309001
Chid1	1,96E-05	-0,09048	0,152	0,264	0,309033
Spg11	1,97E-05	-0,09663	0,163	0,278	0,310252
Pgbd5	1,97E-05	-0,07302	0,272	0,422	0,31155
Rapgef5	1,98E-05	-0,2043	0,378	0,502	0,312561
Magohb	1,98E-05	-0,08653	0,094	0,186	0,312982
G6pdx	1,99E-05	-0,10908	0,312	0,449	0,313982
Orc2	1,99E-05	-0,11016	0,213	0,333	0,314029
Eif3h	1,99E-05	-0,0964	0,756	0,857	0,314662
Zbtb11os1	2,01E-05	-0,14111	0,149	0,251	0,31721
Dnajc4	2,01E-05	-0,09683	0,213	0,338	0,317379
Slc25a10	2,02E-05	-0,11577	0,187	0,3	0,318027
Fbxl12	2,03E-05	-0,06638	0,167	0,291	0,319645
Gm4221	2,03E-05	-0,05734	0,033	0,099	0,319652
Sox2	2,03E-05	0,378299	0,778	0,722	0,320833
Abcb8	2,05E-05	-0,16286	0,347	0,487	0,323481
Ldb3	2,06E-05	-0,12163	0,215	0,338	0,324287
Tyrobp	2,06E-05	-0,04644	0,011	0,059	0,324405
Prorsd1	2,06E-05	-0,09235	0,095	0,188	0,325428
Spcs3	2,07E-05	-0,10951	0,244	0,369	0,326179
Snx24	2,07E-05	-0,10016	0,319	0,466	0,32714
Rbmx	2,1E-05	-0,0876	0,422	0,58	0,331233
Dnajb9	2,1E-05	-0,08281	0,411	0,578	0,331563

Vsnl1	2,12E-05	-0,0395	0,002	0,038	0,334004
Sh2b2	2,12E-05	-0,03747	0,002	0,038	0,334635
Cbll1	2,13E-05	-0,11599	0,295	0,43	0,335727
Gpld1	2,14E-05	-0,07615	0,075	0,162	0,337521
Pdgfc	2,15E-05	-0,05502	0,017	0,07	0,33882
Taf9b	2,15E-05	-0,10188	0,42	0,576	0,339751
Mrps18b	2,16E-05	-0,09525	0,279	0,422	0,340074
Mynn	2,18E-05	-0,08505	0,22	0,352	0,343285
Alkbh7	2,18E-05	-0,0905	0,209	0,333	0,344347
Erf	2,19E-05	0,223584	0,417	0,323	0,345541
Hgsnat	2,2E-05	-0,08449	0,218	0,35	0,346341
Tubg2	2,2E-05	-0,05704	0,035	0,101	0,346803
Elp6	2,2E-05	-0,09784	0,141	0,247	0,346918
Mpped2	2,2E-05	-0,09157	0,048	0,12	0,347744
Rps19	2,21E-05	0,180589	0,985	0,97	0,348327
Phf3	2,21E-05	0,165454	0,851	0,819	0,349091
Otud7a	2,22E-05	-0,10257	0,136	0,238	0,350351
Lrp11	2,22E-05	-0,04481	0,011	0,059	0,350529
Rbfa	2,23E-05	-0,08352	0,206	0,333	0,351345
Lrrfip1	2,25E-05	0,07343	0,048	0,004	0,354415
Dirc2	2,25E-05	-0,09854	0,444	0,62	0,35521
Utp11	2,28E-05	-0,08199	0,437	0,612	0,359499
Hsph1	2,28E-05	-0,07856	0,473	0,608	0,360289
Gmnn	2,3E-05	-0,07644	0,04	0,11	0,362134
Gpr37	2,3E-05	-0,09726	1	0,998	0,36243
Cox11	2,32E-05	-0,0717	0,059	0,137	0,366759
Bclaf1	2,35E-05	0,200632	0,815	0,793	0,370394
Gtf2h5	2,35E-05	-0,09603	0,71	0,823	0,370412
Tmsb15b1	2,38E-05	-0,06304	0,035	0,101	0,374857
Vezf1	2,39E-05	0,197657	0,772	0,719	0,37762
Cfap36	2,4E-05	-0,12021	0,563	0,696	0,379129
Gm49359	2,42E-05	-0,07107	0,066	0,148	0,38255
Rnf208	2,44E-05	-0,13377	0,341	0,479	0,384665
Ypel2	2,45E-05	0,161612	0,985	0,956	0,387246
Slc25a45	2,46E-05	-0,07012	0,094	0,188	0,387713
Sel1l3	2,47E-05	-0,09385	0,084	0,173	0,389448
Zfp825	2,5E-05	-0,06888	0,057	0,135	0,394713
Tfpt	2,52E-05	-0,10275	0,255	0,382	0,398037
Tsnax	2,53E-05	-0,09117	0,417	0,595	0,398799
Msh2	2,53E-05	-0,0844	0,154	0,268	0,399449
Smarcc2	2,54E-05	0,194682	0,771	0,717	0,399967
Gm13546	2,54E-05	-0,04591	0,006	0,046	0,400314
Ttll3	2,54E-05	-0,06111	0,05	0,124	0,40073
Kctd21	2,54E-05	-0,06855	0,079	0,167	0,400733
Mrpl9	2,55E-05	-0,07368	0,343	0,502	0,402288
Nit1	2,56E-05	-0,09656	0,343	0,494	0,404203
Mterf1a	2,56E-05	-0,07426	0,081	0,169	0,404267
Maoa	2,57E-05	-0,10158	0,394	0,555	0,40496
Ccm2	2,57E-05	-0,09093	0,22	0,344	0,40597
Fam32a	2,59E-05	-0,1194	0,376	0,519	0,407885
Zfp386	2,59E-05	-0,09616	0,161	0,272	0,408963
Aldh5a1	2,62E-05	-0,09056	0,143	0,253	0,412947

Mrps18a	2,65E-05	-0,09791	0,466	0,631	0,418406
Ift22	2,7E-05	-0,11472	0,312	0,445	0,425293
Txn2	2,72E-05	-0,11228	0,62	0,747	0,428853
Pusl1	2,74E-05	-0,08314	0,161	0,274	0,432593
Clasrp	2,74E-05	-0,09653	0,468	0,635	0,4329
Ebpl	2,75E-05	-0,12302	0,517	0,667	0,433677
Tmem126b	2,75E-05	-0,08172	0,18	0,297	0,434276
Stx3	2,76E-05	0,134485	0,2	0,108	0,434735
Irgm2	2,77E-05	0,151204	0,072	0,017	0,437123
Apip	2,79E-05	-0,09946	0,224	0,35	0,440603
2810001G:	2,83E-05	-0,07526	0,077	0,162	0,447122
Pmpcb	2,85E-05	-0,08803	0,36	0,519	0,449644
Adamts4	2,86E-05	0,18192	0,936	0,895	0,450695
Metap2	2,86E-05	-0,09662	0,919	0,947	0,451504
Aaed1	2,88E-05	-0,08455	0,211	0,335	0,453841
Cyp4f13	2,88E-05	-0,08479	0,218	0,34	0,453977
Tmem101	2,88E-05	-0,10184	0,422	0,586	0,454065
Ube2f	2,9E-05	-0,10294	0,218	0,34	0,457191
Sst	2,9E-05	-0,03116	0	0,032	0,457315
AI987944	2,91E-05	-0,0833	0,224	0,354	0,458529
Nlrc5	2,91E-05	0,082735	0,072	0,017	0,459045
Trappc6b	2,91E-05	-0,07611	0,328	0,481	0,459549
Timm22	2,92E-05	-0,07427	0,35	0,517	0,459976
Ralgds	2,93E-05	0,142776	0,961	0,909	0,46247
Reep5	2,94E-05	-0,13344	0,976	0,956	0,464065
Plagl1	2,94E-05	-0,08586	0,119	0,219	0,464242
Itgad	2,95E-05	-0,0783	0,039	0,105	0,464839
Cnpy2	2,96E-05	-0,10231	0,538	0,69	0,466625
Cd151	2,96E-05	0,151714	0,235	0,139	0,46679
Uty	2,97E-05	-0,08948	0,095	0,188	0,468088
Stag1	3,02E-05	0,203078	0,516	0,428	0,47632
Ranbp2	3,02E-05	0,191662	0,618	0,54	0,477124
2900093K:	3,03E-05	-0,08464	0,132	0,234	0,477359
Aar2	3,04E-05	-0,08528	0,239	0,369	0,479728
Slc25a29	3,06E-05	-0,10245	0,136	0,236	0,483402
Rbp1	3,07E-05	0,061265	0,101	0,034	0,484354
Timm50	3,08E-05	-0,10293	0,312	0,449	0,48606
Aimp1	3,12E-05	-0,1036	0,683	0,797	0,491913
Napepld	3,12E-05	-0,07049	0,246	0,388	0,492116
Carf	3,12E-05	-0,08037	0,139	0,247	0,492482
2210408I2	3,13E-05	-0,06875	0,061	0,139	0,493275
Iars2	3,13E-05	-0,0866	0,215	0,342	0,494297
Ddx59	3,14E-05	-0,08252	0,084	0,171	0,494777
Lbr	3,15E-05	-0,09953	0,462	0,614	0,497285
Msantd3	3,16E-05	-0,07885	0,083	0,169	0,49861
Fosl2	3,17E-05	0,151457	0,165	0,082	0,499342
BC028528	3,17E-05	-0,09004	0,086	0,173	0,49975
Snrpg	3,22E-05	-0,10951	0,686	0,77	0,508429
Pmm1	3,23E-05	-0,12885	0,583	0,705	0,509283
Clcn6	3,26E-05	0,208292	0,567	0,506	0,514555
Haus1	3,29E-05	-0,07428	0,11	0,207	0,518352
Zfc3h1	3,29E-05	-0,09306	0,292	0,424	0,519568

Purg	3,29E-05	-0,07675	0,123	0,224	0,519685
Snrnp35	3,33E-05	-0,08473	0,134	0,238	0,525222
Ino80b	3,36E-05	-0,09508	0,472	0,629	0,530424
Ier3ip1	3,37E-05	-0,1112	0,569	0,724	0,53096
Entpd5	3,38E-05	-0,12838	0,479	0,622	0,532528
Psmg1	3,39E-05	-0,10719	0,174	0,283	0,534489
Ino80c	3,4E-05	-0,09363	0,174	0,287	0,535844
Stat1	3,4E-05	0,451123	0,371	0,289	0,536324
Abhd14a	3,41E-05	-0,10362	0,415	0,572	0,537527
Eif2b1	3,43E-05	-0,08242	0,22	0,348	0,540746
Gria4	3,44E-05	-0,09791	0,224	0,348	0,542312
Snapin	3,44E-05	-0,1084	0,82	0,892	0,542438
Ece1	3,44E-05	-0,06071	0,026	0,084	0,542633
Llph	3,46E-05	-0,10582	0,501	0,675	0,545968
Ptpdc1	3,46E-05	-0,10776	0,429	0,568	0,546269
Ubxn8	3,47E-05	-0,0924	0,152	0,259	0,547343
Mgat5	3,47E-05	-0,07943	0,136	0,243	0,547429
Ndufaf7	3,47E-05	-0,06558	0,215	0,346	0,548212
Nppc	3,49E-05	-0,10622	0,116	0,213	0,550588
Dus2	3,51E-05	-0,07319	0,191	0,314	0,554107
Cope	3,51E-05	-0,12791	0,754	0,835	0,554301
Nuak1	3,52E-05	0,229246	0,494	0,405	0,555786
Lyrm1	3,53E-05	-0,07615	0,088	0,177	0,556881
Ascc1	3,55E-05	-0,08487	0,202	0,325	0,559472
Emg1	3,55E-05	-0,08658	0,358	0,513	0,56003
Gm816	3,55E-05	-0,0524	0,011	0,057	0,56017
Exosc8	3,56E-05	-0,0959	0,237	0,359	0,561208
Prrg2	3,58E-05	-0,08614	0,11	0,205	0,565226
Fam98a	3,61E-05	-0,12527	0,411	0,565	0,569463
Hepacam	3,64E-05	-0,12101	0,934	0,964	0,573515
Nipal4	3,65E-05	-0,12469	0,521	0,662	0,575937
Pdcd6ip	3,69E-05	0,178875	0,765	0,724	0,581993
Faf2	3,69E-05	-0,07446	0,409	0,597	0,582877
Vps45	3,7E-05	-0,09334	0,196	0,314	0,583274
Mmp15	3,7E-05	-0,09329	0,094	0,184	0,583559
Ccdc130	3,7E-05	-0,07865	0,103	0,196	0,584101
Hsp90aa1	3,73E-05	-0,0748	1	0,998	0,587773
Tnik	3,73E-05	-0,09638	0,172	0,287	0,587827
Eepd1	3,74E-05	-0,06345	0,04	0,108	0,589302
Adi1	3,75E-05	-0,13214	0,767	0,819	0,591776
Sox8	3,75E-05	0,206935	0,895	0,869	0,592061
Tada3	3,76E-05	-0,09554	0,288	0,426	0,592465
Crtac1	3,76E-05	0,260899	0,371	0,278	0,593543
Dach2	3,76E-05	-0,06312	0,02	0,074	0,593815
Polr3d	3,77E-05	-0,09371	0,306	0,441	0,595151
Fbxl6	3,78E-05	-0,06635	0,095	0,188	0,596697
Sema6d	3,81E-05	0,243658	0,703	0,603	0,601407
Cadm2	3,82E-05	-0,11649	0,525	0,665	0,602393
Dennd6b	3,82E-05	-0,09453	0,108	0,2	0,603307
Arfip2	3,82E-05	-0,09906	0,354	0,498	0,603421
Alg5	3,84E-05	-0,07903	0,294	0,441	0,605664
1500015L2	3,84E-05	-0,07413	0,061	0,137	0,606547

Fuz	3,89E-05	-0,08116	0,152	0,259	0,613086
BC002059	3,92E-05	-0,05165	0,061	0,139	0,617864
Prrg1	3,92E-05	-0,10727	0,486	0,637	0,618049
Plppr3	3,92E-05	0,088804	0,105	0,038	0,618712
4632415L(	3,92E-05	-0,10055	0,127	0,224	0,619151
Gm29825	3,93E-05	-0,03747	0,002	0,036	0,6198
Gm19705	3,96E-05	-0,07988	0,088	0,175	0,624153
Med29	3,96E-05	-0,08608	0,314	0,46	0,625106
Lnpep	3,98E-05	0,157763	0,664	0,582	0,628433
Nagpa	3,99E-05	-0,07895	0,222	0,35	0,628828
Cdr2l	3,99E-05	-0,08321	0,365	0,521	0,629932
Trmt5	4E-05	-0,06332	0,048	0,118	0,630285
Fabp7	4E-05	-0,03422	0,011	0,057	0,630792
2210011C:	4E-05	-0,06845	0,031	0,093	0,631818
Myof	4,01E-05	0,141625	0,194	0,103	0,632663
Tubg1	4,02E-05	-0,10012	0,196	0,308	0,633705
Rad17	4,09E-05	-0,07192	0,237	0,371	0,645095
Ube2g1	4,09E-05	-0,11614	0,558	0,711	0,645168
Dusp23	4,1E-05	-0,10476	0,134	0,234	0,646716
Idh3b	4,1E-05	-0,09688	0,433	0,591	0,646914
Hspg2	4,11E-05	0,064834	0,059	0,011	0,647608
Mtg1	4,12E-05	-0,07472	0,116	0,213	0,649837
E4f1	4,13E-05	-0,08384	0,163	0,274	0,650976
Rrnad1	4,14E-05	-0,08742	0,248	0,373	0,653349
Mrpl21	4,15E-05	-0,08601	0,299	0,443	0,654117
Clgn	4,15E-05	-0,16604	0,095	0,181	0,655093
Zfp994	4,15E-05	-0,07121	0,048	0,118	0,655121
Fancl	4,16E-05	-0,06913	0,066	0,146	0,656562
Gm12258	4,17E-05	-0,07046	0,061	0,137	0,657146
Kcnk1	4,17E-05	-0,05159	0,02	0,074	0,65747
Midn	4,18E-05	0,108308	0,084	0,025	0,659499
Mis12	4,28E-05	-0,08251	0,143	0,249	0,674645
Nasp	4,28E-05	0,245211	0,473	0,388	0,675843
Ell2	4,32E-05	-0,07235	0,358	0,513	0,681638
Aacs	4,33E-05	0,248412	0,365	0,274	0,682576
Adam9	4,33E-05	0,175503	0,585	0,515	0,683345
Rmnd1	4,37E-05	-0,11241	0,226	0,344	0,69014
Amacr	4,38E-05	-0,06387	0,048	0,118	0,690433
Slc25a26	4,39E-05	-0,08043	0,073	0,154	0,691827
Pcgf5	4,4E-05	-0,07716	0,105	0,198	0,694131
Rad51b	4,4E-05	-0,04878	0,015	0,063	0,694721
Nim1k	4,42E-05	0,098013	0,066	0,015	0,696858
Pole4	4,42E-05	-0,09631	0,407	0,565	0,697679
Lrrc4c	4,45E-05	-0,08752	0,189	0,304	0,701387
Csmd2	4,45E-05	-0,08401	0,064	0,141	0,701706
Naf1	4,45E-05	-0,09032	0,261	0,388	0,702044
Fh1	4,46E-05	-0,09243	0,297	0,426	0,703246
Mtf1	4,47E-05	-0,10464	0,444	0,591	0,704489
Rad1	4,5E-05	-0,07932	0,196	0,314	0,710572
Map4	4,51E-05	0,169366	0,824	0,776	0,71126
Rhou	4,51E-05	-0,12435	0,96	0,954	0,711738
1110059E:	4,53E-05	-0,07965	0,233	0,363	0,715228

Stam2	4,53E-05	-0,07301	0,321	0,473	0,71528
Dcaf17	4,54E-05	-0,19957	0,763	0,842	0,716704
Tgs1	4,56E-05	-0,08863	0,383	0,536	0,719703
Fam192a	4,56E-05	-0,08154	0,389	0,549	0,720104
Rev3l	4,57E-05	-0,08744	0,237	0,367	0,721637
Smc2	4,59E-05	-0,08225	0,169	0,281	0,72337
Kcnq1ot1	4,59E-05	0,322497	0,818	0,728	0,724058
0610009O:	4,6E-05	-0,10749	0,29	0,418	0,725533
Trmt13	4,61E-05	-0,07472	0,317	0,46	0,727594
Gtf3c3	4,63E-05	-0,10354	0,18	0,293	0,730239
Dnph1	4,63E-05	-0,07539	0,059	0,133	0,731059
As3mt	4,64E-05	-0,09519	0,272	0,399	0,731251
9130401M	4,66E-05	-0,09986	0,24	0,365	0,734532
Mroh7	4,72E-05	0,077335	0,073	0,019	0,743949
Klf11	4,72E-05	0,136675	0,24	0,143	0,744184
Pfkl	4,73E-05	-0,07484	0,143	0,249	0,746209
Tmem106c	4,75E-05	-0,10539	0,343	0,479	0,74874
Gabra3	4,77E-05	-0,04427	0,006	0,044	0,752823
Dnajb4	4,79E-05	-0,07059	0,352	0,511	0,755731
Bptf	4,81E-05	0,144122	0,912	0,842	0,758686
Denr	4,82E-05	-0,10973	0,565	0,705	0,760263
Hsd17b7	4,84E-05	0,25758	0,396	0,316	0,763292
Mkrn3	4,86E-05	-0,06414	0,05	0,12	0,76611
Drg2	4,86E-05	-0,09276	0,345	0,494	0,767466
Cpe	4,89E-05	0,215986	0,635	0,551	0,77192
Kdr	4,91E-05	-0,0741	0,035	0,097	0,774178
Rwdd4a	4,92E-05	-0,08575	0,22	0,342	0,775472
Sirt1	4,94E-05	0,160066	0,38	0,276	0,779875
Abhd18	4,95E-05	-0,04482	0,174	0,3	0,781357
Tom1l2	4,97E-05	0,200081	0,629	0,58	0,783405
Malsu1	4,97E-05	-0,08671	0,257	0,388	0,783926
Arsb	4,97E-05	-0,07643	0,264	0,403	0,784509
Tnks2	5,01E-05	0,153409	0,84	0,793	0,790586
Dnajc9	5,01E-05	-0,0987	0,264	0,395	0,790835
Srprb	5,07E-05	-0,06986	0,297	0,445	0,799362
Ube3a	5,07E-05	0,164207	0,906	0,878	0,800364
Gls2	5,09E-05	-0,07734	0,061	0,135	0,802383
Tomm40l	5,12E-05	-0,098	0,178	0,287	0,807101
Ift27	5,12E-05	-0,10025	0,303	0,437	0,807718
Cwc25	5,14E-05	-0,0774	0,255	0,395	0,810231
Epb41l4b	5,14E-05	-0,09438	0,295	0,428	0,810802
Amph	5,15E-05	-0,08108	0,279	0,424	0,812453
Unc5c	5,18E-05	0,202633	0,659	0,597	0,816599
Oxnad1	5,19E-05	-0,0899	0,119	0,213	0,819115
Rnf152	5,21E-05	0,216015	0,209	0,122	0,821868
Dguok	5,24E-05	-0,09157	0,402	0,542	0,826164
Sap30bp	5,27E-05	-0,0881	0,363	0,511	0,831579
Mif4gd	5,28E-05	-0,11463	0,554	0,684	0,833253
Rsrc2	5,28E-05	0,167294	0,912	0,861	0,833264
Calr	5,29E-05	-0,1226	0,983	0,977	0,834375
Fam229b	5,31E-05	-0,08837	0,116	0,209	0,837961
Hspa2	5,31E-05	0,19298	0,475	0,371	0,83827

Kctd6	5,33E-05	-0,08103	0,134	0,234	0,840291
Vdac1	5,33E-05	-0,12699	0,617	0,741	0,841221
Atg14	5,34E-05	-0,06964	0,182	0,3	0,842757
Ficd	5,37E-05	-0,0665	0,077	0,16	0,846765
4930522L1	5,37E-05	-0,06107	0,072	0,152	0,84722
Hjurp	5,37E-05	-0,10062	0,273	0,403	0,84774
Akr7a5	5,4E-05	-0,09123	0,404	0,565	0,851905
Smim4	5,45E-05	-0,07993	0,134	0,234	0,859498
Hip1	5,47E-05	-0,13713	0,624	0,73	0,862843
Gar1	5,48E-05	-0,07377	0,248	0,38	0,864135
Zfyve16	5,48E-05	-0,08892	0,248	0,373	0,864771
Sulf2	5,49E-05	0,174518	0,919	0,897	0,865682
Snapc1	5,49E-05	-0,11338	0,336	0,477	0,866267
Lsg1	5,53E-05	-0,06169	0,268	0,416	0,872646
Syncrip	5,56E-05	0,216283	0,602	0,551	0,87693
Stradb	5,58E-05	-0,08069	0,103	0,192	0,880989
Ufc1	5,59E-05	-0,11421	0,578	0,713	0,881929
Hdac3	5,62E-05	-0,09192	0,233	0,354	0,886626
Apln	5,7E-05	-0,10869	0,312	0,447	0,899962
Nip7	5,72E-05	-0,07995	0,215	0,333	0,902117
Usp25	5,76E-05	-0,12976	0,51	0,643	0,908032
Gcsh	5,76E-05	-0,10036	0,246	0,367	0,908185
Rnf114	5,79E-05	-0,08603	0,27	0,399	0,913291
Lmbrd1	5,79E-05	-0,107	0,967	0,983	0,913406
Riox1	5,83E-05	-0,06282	0,059	0,133	0,920152
1810058I2	5,84E-05	-0,11582	0,708	0,814	0,921912
Ostm1	5,87E-05	-0,08608	0,312	0,449	0,926103
Rpl36al	5,89E-05	-0,11047	0,839	0,876	0,928703
Serinc1	5,9E-05	0,104423	0,998	0,996	0,930224
Phkg2	5,91E-05	-0,07293	0,272	0,407	0,93218
Stk25	5,92E-05	-0,11366	0,44	0,574	0,934303
Mcfd2	5,93E-05	-0,08558	0,332	0,483	0,935686
Zcwpw1	5,95E-05	-0,07825	0,211	0,331	0,938182
Cct5	5,95E-05	-0,11063	0,817	0,897	0,938918
Arid5a	5,96E-05	0,238928	0,286	0,192	0,939799
Macf1	5,96E-05	-0,13364	0,717	0,823	0,940696
Wdr92	5,97E-05	-0,07167	0,079	0,16	0,941247
Dmxl1	6,01E-05	0,211861	0,598	0,549	0,94856
Slc37a4	6,03E-05	-0,08098	0,116	0,209	0,950961
Snd1	6,03E-05	-0,06682	0,31	0,458	0,951296
Gtf2h2	6,06E-05	-0,06095	0,139	0,247	0,956183
Tdrkh	6,08E-05	-0,06937	0,211	0,331	0,959614
Dhrs7	6,11E-05	-0,09419	0,462	0,618	0,963469
Kin	6,11E-05	-0,08218	0,229	0,357	0,96448
Gm14295	6,16E-05	-0,06925	0,068	0,146	0,971297
Mum1	6,16E-05	-0,08949	0,428	0,586	0,972528
Exosc1	6,17E-05	-0,07755	0,224	0,35	0,973531
Npm3	6,19E-05	-0,08397	0,189	0,302	0,976005
Snrpd2	6,19E-05	-0,10057	0,87	0,92	0,976843
Tmem159	6,22E-05	-0,08729	0,394	0,557	0,981861
Fsd1	6,25E-05	-0,07952	0,294	0,435	0,985519
Gorasp2	6,26E-05	-0,09129	0,4	0,546	0,987603

Zfp369	6,29E-05	-0,08698	0,094	0,179	0,991973
Zcchc18	6,32E-05	-0,12502	0,229	0,35	0,997172
Zfp975	6,32E-05	-0,0555	0,024	0,078	0,997184
Fuom	6,36E-05	-0,10225	0,163	0,264	1
Zfp799	6,37E-05	-0,06572	0,105	0,198	1
Enox2	6,37E-05	-0,08307	0,134	0,232	1
Acot7	6,4E-05	-0,09424	0,93	0,956	1
Deaf1	6,47E-05	-0,10665	0,233	0,348	1
C030029H(	6,5E-05	-0,27706	0,594	0,641	1
Nrap	6,5E-05	-0,08059	0,095	0,184	1
Abcg1	6,51E-05	-0,13558	0,844	0,88	1
Slc8a1	6,52E-05	-0,08039	0,101	0,19	1
2610301B:	6,57E-05	-0,08097	0,145	0,249	1
Crebl2	6,6E-05	-0,10158	0,27	0,395	1
Ermp1	6,61E-05	0,160949	0,914	0,888	1
Adgrg6	6,62E-05	0,046603	0,033	0	1
Hist1h2be	6,63E-05	-0,09321	0,106	0,196	1
Slc27a4	6,64E-05	-0,08493	0,44	0,595	1
Ssr2	6,7E-05	-0,10158	0,67	0,795	1
Rwdd3	6,74E-05	-0,04946	0,02	0,072	1
Dnajc14	6,74E-05	-0,09421	0,272	0,392	1
H2-Q7	6,75E-05	0,07851	0,039	0,002	1
Lrmda	6,78E-05	-0,05352	0,037	0,099	1
Catsper2	6,79E-05	-0,0688	0,084	0,169	1
Nelfcd	6,8E-05	-0,09435	0,222	0,338	1
Ngrn	6,84E-05	-0,06633	0,117	0,213	1
Galnt6	6,84E-05	-0,1676	0,839	0,892	1
Trp53bp2	6,85E-05	0,150084	0,844	0,768	1
Dnlz	6,9E-05	-0,09468	0,439	0,591	1
Zfhx3	6,9E-05	0,287866	0,354	0,27	1
Bmf	6,92E-05	0,084801	0,075	0,021	1
Sirt3	6,94E-05	-0,08057	0,251	0,38	1
Pex16	6,97E-05	-0,08161	0,25	0,373	1
Gm26720	7,02E-05	0,083969	0,108	0,042	1
Snrpd3	7,03E-05	-0,10579	0,69	0,795	1
Ptrhd1	7,04E-05	-0,10563	0,202	0,312	1
Rft1	7,11E-05	-0,07716	0,163	0,272	1
Upf3a	7,16E-05	-0,07848	0,297	0,439	1
Rabac1	7,21E-05	-0,08951	0,987	0,987	1
Dock7	7,22E-05	0,191252	0,248	0,156	1
Cog8	7,27E-05	-0,08119	0,189	0,302	1
Lsm6	7,27E-05	-0,10727	0,552	0,692	1
Sfi1	7,29E-05	-0,09079	0,16	0,262	1
D3Ertd751	7,3E-05	-0,06162	0,158	0,27	1
Nbas	7,35E-05	0,189725	0,752	0,692	1
Zfp688	7,36E-05	-0,09616	0,167	0,268	1
Dph3	7,38E-05	-0,09063	0,457	0,595	1
Cog4	7,39E-05	-0,08919	0,345	0,485	1
Gosr2	7,41E-05	-0,08089	0,33	0,47	1
Fam58b	7,47E-05	-0,08331	0,167	0,272	1
Mipol1	7,48E-05	-0,09986	0,149	0,247	1
Mrps35	7,55E-05	-0,08125	0,167	0,272	1

Ndfip1	7,57E-05	-0,0871	0,305	0,445	1
Rrn3	7,59E-05	-0,06221	0,279	0,418	1
Xrcc2	7,59E-05	-0,07023	0,073	0,152	1
Sae1	7,61E-05	-0,08293	0,207	0,323	1
Cox6b2	7,65E-05	-0,07131	0,057	0,129	1
Mta3	7,66E-05	-0,09499	0,484	0,635	1
Cdc42ep1	7,67E-05	-0,13426	0,679	0,785	1
Nutf2	7,71E-05	-0,07787	0,139	0,238	1
Lamtor1	7,81E-05	-0,11619	0,818	0,865	1
Tnfrsf1a	7,84E-05	0,120379	0,128	0,057	1
Zfp672	7,89E-05	-0,0932	0,191	0,3	1
Gbp7	7,93E-05	0,136396	0,075	0,021	1
Fam102b	7,96E-05	0,095082	0,145	0,068	1
Pir	7,97E-05	-0,07658	0,094	0,179	1
Zcchc9	7,99E-05	-0,09918	0,303	0,437	1
Plxna4	8E-05	0,070752	0,068	0,017	1
Gm6710	8,01E-05	-0,07717	0,077	0,156	1
Nav1	8,04E-05	0,169901	0,631	0,536	1
Tarbp1	8,05E-05	-0,05434	0,029	0,086	1
Zpr1	8,07E-05	-0,07804	0,277	0,405	1
Aasdh	8,09E-05	-0,06726	0,083	0,165	1
Dpm2	8,13E-05	-0,12656	0,459	0,591	1
Nrip1	8,13E-05	-0,11279	0,336	0,473	1
Anln	8,16E-05	-0,17389	0,972	0,945	1
Mks1	8,19E-05	-0,07238	0,187	0,302	1
Alyref	8,19E-05	-0,11618	0,574	0,711	1
Rnf34	8,21E-05	-0,08558	0,33	0,466	1
Zdhhc24	8,3E-05	-0,07864	0,083	0,162	1
Plxdc2	8,37E-05	0,133215	0,954	0,914	1
Grn	8,42E-05	0,20537	0,525	0,46	1
Sharpin	8,48E-05	-0,0838	0,196	0,306	1
Ttyh2	8,53E-05	-0,12215	0,985	0,987	1
Acad8	8,54E-05	-0,06945	0,207	0,327	1
Stx6	8,57E-05	0,163014	0,739	0,686	1
Cyp4f16	8,59E-05	-0,07221	0,105	0,194	1
Erh	8,69E-05	-0,09884	0,499	0,652	1
Zc3h8	8,69E-05	-0,0882	0,075	0,152	1
Dera	8,69E-05	-0,08074	0,083	0,162	1
Pfkm	8,77E-05	-0,03402	0,006	0,042	1
Jkamp	8,82E-05	-0,10651	0,556	0,705	1
Wdcp	8,85E-05	-0,08551	0,167	0,272	1
Hddc3	8,86E-05	-0,0814	0,143	0,241	1
Itgb5	8,91E-05	-0,09971	0,361	0,502	1
Wrb	8,96E-05	-0,08142	0,24	0,357	1
Col9a1	8,96E-05	-0,04623	0,022	0,074	1
Fahd1	8,98E-05	-0,08726	0,171	0,274	1
Birc6	8,99E-05	0,16646	0,793	0,751	1
Gm5113	9,06E-05	-0,07727	0,083	0,162	1
Wscd1	9,07E-05	-0,08979	0,998	0,998	1
Dalrd3	9,1E-05	-0,08098	0,242	0,365	1
Gopc	9,1E-05	-0,09719	0,262	0,388	1
Clcn4	9,13E-05	-0,11093	0,864	0,903	1

Fam136a	9,19E-05	-0,06448	0,11	0,203	1
Cntn3	9,31E-05	-0,08379	0,125	0,219	1
Phc1	9,33E-05	-0,08938	0,424	0,578	1
Zdhhc3	9,35E-05	-0,09542	0,273	0,403	1
D130040H	9,41E-05	-0,05493	0,09	0,175	1
Rps9	9,46E-05	-0,06763	0,996	0,996	1
Mrpl46	9,51E-05	-0,08016	0,198	0,31	1
Ccnc	9,56E-05	-0,07078	0,226	0,35	1
4930453N	9,58E-05	-0,09192	0,272	0,397	1
Rnaset2a	9,63E-05	-0,07522	0,228	0,352	1
Plxnb1	9,64E-05	0,190146	0,516	0,441	1
Smim5	9,68E-05	-0,10145	0,05	0,116	1
Svbp	9,91E-05	-0,05877	0,273	0,405	1
Eefsec	9,92E-05	-0,08498	0,251	0,371	1
Pigf	9,96E-05	-0,06926	0,147	0,249	1
Cep85l	0,0001	-0,07273	0,156	0,262	1
Rpl30	0,0001	-0,07441	0,996	0,996	1
1300002E1	0,0001	-0,08242	0,171	0,276	1
Slf1	0,000101	-0,07988	0,176	0,283	1
Gm40578	0,000101	-0,02911	0	0,027	1
Mcm3	0,000102	-0,06937	0,07	0,146	1
Tmem243	0,000103	-0,0857	0,38	0,525	1
Lrif1	0,000103	-0,10656	0,349	0,481	1
Ltn1	0,000104	-0,09953	0,363	0,508	1
Zfp788	0,000104	-0,07981	0,161	0,264	1
Kcnrg	0,000104	0,077739	0,066	0,017	1
Mon1b	0,000104	-0,08054	0,127	0,219	1
Inpp5k	0,000105	-0,0959	0,231	0,344	1
Fam228b	0,000106	-0,12002	0,244	0,352	1
Fam96b	0,000106	-0,09556	0,637	0,774	1
Atg4d	0,000106	-0,09426	0,327	0,454	1
Zswim9	0,000106	-0,05967	0,094	0,179	1
Eif1a	0,000106	0,190663	0,317	0,228	1
Fyco1	0,000106	-0,08412	0,279	0,401	1
Vim	0,000106	0,190843	0,125	0,055	1
Fggy	0,000106	-0,07953	0,149	0,249	1
Acad12	0,000106	-0,06586	0,068	0,143	1
Zfhx4	0,000106	-0,10367	0,321	0,454	1
Ethe1	0,000106	-0,0984	0,224	0,331	1
Dnmt3a	0,000106	0,168951	0,672	0,584	1
Ubash3b	0,000107	-0,05105	0,273	0,414	1
Tmem121	0,000107	-0,08714	0,158	0,257	1
Tsen15	0,000107	-0,06753	0,123	0,217	1
Rps6	0,000107	-0,09009	0,993	0,985	1
Zfp14	0,000108	-0,06789	0,128	0,224	1
Tmx2	0,000108	-0,10843	0,642	0,764	1
B3gat3	0,000109	-0,07784	0,466	0,62	1
Slc22a18	0,000109	-0,05142	0,02	0,07	1
Grasp	0,000109	0,16099	0,215	0,129	1
Brd4	0,00011	0,15938	0,67	0,597	1
Ranbp1	0,00011	-0,11099	0,62	0,736	1
Znrd1as	0,00011	-0,06125	0,062	0,135	1

Tmem184b	0,000111	-0,09073	0,383	0,527	1
Pqlc3	0,000111	0,101446	0,16	0,08	1
Chd3os	0,000111	-0,04969	0,031	0,089	1
Gm46218	0,000112	-0,06414	0,05	0,116	1
Cd2ap	0,000112	-0,06303	0,418	0,589	1
Lgmn	0,000112	0,122234	0,936	0,871	1
Gphn	0,000113	-0,11053	0,499	0,641	1
Rabggta	0,000114	-0,07434	0,172	0,278	1
Atg9a	0,000114	-0,09805	0,294	0,42	1
2810006K2	0,000114	-0,06104	0,088	0,171	1
Rsrc1	0,000114	-0,09821	0,683	0,783	1
Bmp4	0,000114	0,088091	0,062	0,015	1
Sema6a	0,000114	-0,16929	0,505	0,616	1
Fibin	0,000115	-0,08655	0,105	0,192	1
Hdac7	0,000115	0,20327	0,406	0,321	1
P2rx7	0,000115	-0,0629	0,215	0,338	1
Abt1	0,000116	-0,07741	0,156	0,257	1
Ccdc115	0,000116	-0,07748	0,191	0,3	1
L1cam	0,000116	0,084801	0,066	0,017	1
Aplp2	0,000116	-0,08322	0,998	0,998	1
Fam206a	0,000117	-0,07546	0,125	0,217	1
Zfp738	0,000117	-0,08268	0,183	0,293	1
Ssr4	0,000118	-0,12028	0,721	0,819	1
Gpn1	0,000119	-0,07383	0,169	0,274	1
Cep295	0,000119	-0,09267	0,239	0,361	1
Top1mt	0,00012	-0,07669	0,101	0,186	1
Ccdc152	0,00012	-0,08885	0,127	0,219	1
Srfbp1	0,000121	-0,06189	0,086	0,167	1
Abhd17a	0,000121	-0,1208	0,815	0,876	1
Zfp157	0,000121	-0,06195	0,218	0,34	1
Wdyhv1	0,000123	-0,05167	0,084	0,167	1
Nit2	0,000123	-0,08069	0,101	0,186	1
Ccdc127	0,000124	-0,08716	0,369	0,508	1
Leprotl1	0,000124	-0,09536	0,486	0,631	1
9030025P2	0,000124	-0,12417	0,152	0,249	1
Stk19	0,000125	-0,06937	0,286	0,418	1
Otud7b	0,000125	0,155276	0,983	0,97	1
Stxbp3	0,000126	-0,09443	0,903	0,937	1
Krcc1	0,000127	-0,07331	0,35	0,498	1
6330562C2	0,000127	-0,05954	0,059	0,129	1
Hmgb3	0,000128	-0,0723	0,062	0,133	1
Tmem242	0,000128	-0,08397	0,237	0,357	1
Zyx	0,000128	0,192019	0,525	0,437	1
H2afv	0,000129	-0,09106	0,589	0,717	1
Mettl16	0,00013	-0,08642	0,301	0,432	1
Ccdc12	0,00013	-0,11184	0,571	0,711	1
Zfp40	0,00013	-0,08088	0,141	0,238	1
Scamp1	0,00013	-0,09474	0,334	0,468	1
Tdrd3	0,00013	-0,06924	0,189	0,302	1
Timm17a	0,000131	-0,11102	0,594	0,728	1
Ndufa2	0,000131	-0,0865	0,958	0,981	1
Wdr19	0,000132	-0,06937	0,068	0,141	1

Aebp1	0,000132	0,271735	0,361	0,274	1
Saxo2	0,000132	-0,06755	0,068	0,141	1
Porcn	0,000132	-0,07876	0,163	0,268	1
Ankrd54	0,000133	-0,07037	0,16	0,262	1
Cdkl3	0,000134	-0,0719	0,077	0,154	1
Tkfc	0,000134	-0,07906	0,083	0,16	1
Mia2	0,000134	-0,10754	0,497	0,65	1
Drap1	0,000135	-0,10709	0,815	0,884	1
Usp18	0,000135	0,083983	0,05	0,008	1
Lcat	0,000135	-0,09314	0,077	0,152	1
Dcun1d4	0,000135	-0,09147	0,167	0,268	1
Mtfr1	0,000135	-0,07092	0,121	0,213	1
Gm32633	0,000136	-0,03799	0,015	0,059	1
Celsr2	0,000137	-0,10249	0,253	0,371	1
Rpl27-ps3	0,000138	-0,06837	0,062	0,133	1
Eif3k	0,000138	-0,10378	0,873	0,914	1
Ythdc1	0,000138	0,127357	0,749	0,665	1
Klhdc1	0,000138	-0,07601	0,105	0,19	1
Mto1	0,00014	-0,07462	0,139	0,234	1
Gemin8	0,00014	-0,07406	0,108	0,194	1
Eif4e2	0,00014	-0,08684	0,358	0,502	1
Slc25a18	0,00014	-0,02749	0,002	0,032	1
Ap3s2	0,00014	-0,08919	0,292	0,416	1
Bloc1s4	0,00014	-0,07764	0,237	0,354	1
Ctsh	0,000141	-0,04208	0,007	0,044	1
Gm4279	0,000142	-0,04007	0,007	0,044	1
Acly	0,000143	0,206644	0,6	0,536	1
Pcdhga7	0,000143	-0,06748	0,048	0,112	1
Zfp1	0,000144	-0,08844	0,202	0,308	1
Klhl28	0,000145	-0,06874	0,275	0,403	1
Gm38335	0,000145	0,115314	0,075	0,023	1
Taf11	0,000146	-0,07671	0,369	0,5	1
Ephx1	0,000146	-0,10885	0,143	0,238	1
Arhgef28	0,000147	-0,11627	0,45	0,591	1
Nudt6	0,000147	-0,05983	0,048	0,112	1
Pdxp	0,000149	-0,04588	0,022	0,072	1
Tigd2	0,000149	-0,09888	0,213	0,321	1
Smtnl2	0,000149	-0,09029	0,094	0,173	1
4732440D(	0,00015	-0,06781	0,073	0,148	1
Neb	0,000151	-0,04171	0,004	0,036	1
Lix1	0,000151	-0,04189	0,006	0,04	1
Atox1	0,000151	-0,10237	0,75	0,835	1
Ids	0,000152	0,15944	0,817	0,778	1
Lmf1	0,000152	-0,0608	0,352	0,502	1
Txndc9	0,000153	-0,09618	0,558	0,698	1
Cdc42bpa	0,000154	-0,1056	0,956	0,96	1
Sirt4	0,000154	-0,07092	0,13	0,224	1
Lysmd2	0,000155	-0,03756	0,059	0,129	1
Slc30a2	0,000155	-0,03786	0,004	0,036	1
2700097O(	0,000156	-0,0562	0,215	0,333	1
Litaf	0,000157	-0,09483	0,991	0,992	1
Ash1l	0,000158	0,165688	0,844	0,787	1

Vkorc1	0,000158	-0,11787	0,574	0,688	1
Pomk	0,000159	-0,05642	0,061	0,131	1
Mef2a	0,000159	0,180976	0,76	0,728	1
Rhobtb3	0,000159	-0,11005	0,666	0,8	1
Gm45495	0,00016	-0,06373	0,05	0,114	1
Tomm6	0,00016	-0,042	0,389	0,563	1
0610010K1	0,000161	-0,08657	0,266	0,384	1
Eif2d	0,000162	-0,06407	0,158	0,262	1
Mrps9	0,000162	-0,09319	0,273	0,392	1
Ercc6l2	0,000163	-0,09497	0,35	0,487	1
Jrkl	0,000163	-0,0711	0,187	0,297	1
Zcchc10	0,000164	-0,08125	0,145	0,241	1
Fam214a	0,000164	-0,09784	0,18	0,283	1
Bcas2	0,000164	-0,10437	0,694	0,812	1
Fzd7	0,000164	-0,07116	0,106	0,192	1
Mien1	0,000165	-0,08992	0,818	0,861	1
Rmnd5b	0,000165	-0,07962	0,158	0,255	1
Slc35b2	0,000165	-0,0833	0,305	0,439	1
Ercc5	0,000166	-0,07886	0,152	0,249	1
Sspo	0,000167	0,117491	0,198	0,114	1
Gm26782	0,000167	-0,09876	0,15	0,243	1
Rpain	0,000168	-0,06193	0,2	0,314	1
Trp53rka	0,000169	-0,05907	0,217	0,333	1
Zfpl1	0,000169	-0,0797	0,292	0,416	1
Abcb10	0,000169	-0,122	0,36	0,492	1
Apoa1	0,00017	0,054949	0,035	0,002	1
Senp5	0,00017	-0,0779	0,277	0,399	1
Rdh12	0,00017	-0,06166	0,062	0,133	1
Rit1	0,00017	-0,06715	0,196	0,308	1
Terf2ip	0,00017	-0,07746	0,306	0,443	1
Degs1	0,000171	-0,0907	0,978	0,992	1
Zfp346	0,000171	-0,07895	0,152	0,249	1
Carnmt1	0,000171	-0,07248	0,182	0,289	1
Plekhg5	0,000172	0,039574	0,029	0	1
Gm5617	0,000172	-0,07949	0,198	0,308	1
Tkt	0,000172	-0,09104	0,994	0,994	1
Vamp8	0,000172	0,181968	0,383	0,291	1
Derl1	0,000173	-0,10369	0,714	0,804	1
Rpe	0,000174	-0,0784	0,306	0,439	1
Pdcd2	0,000174	-0,07691	0,187	0,291	1
Eid2b	0,000174	-0,06648	0,092	0,173	1
Rab11b	0,000174	-0,10391	0,789	0,854	1
Vps25	0,000174	-0,05897	0,073	0,148	1
Fam207a	0,000174	-0,07089	0,215	0,325	1
Arf1	0,000176	-0,09723	0,95	0,968	1
Arhgef40	0,000176	0,107159	0,141	0,07	1
Rbmx2	0,000176	-0,07574	0,18	0,283	1
Dnajb5	0,000177	-0,06454	0,059	0,127	1
Creb3	0,000177	-0,07819	0,341	0,481	1
Rps10	0,00018	-0,07423	0,998	0,996	1
Rab24	0,000181	-0,09628	0,35	0,479	1
Mmaa	0,000182	-0,06053	0,174	0,281	1

Casp3	0,000182	0,060527	0,061	0,015	1
C1qa	0,000183	-0,04267	0,024	0,074	1
Negr1	0,000184	-0,07132	0,167	0,272	1
Gpn2	0,000186	-0,05613	0,16	0,264	1
Zfp330	0,000186	-0,05644	0,218	0,338	1
Rell1	0,000188	0,18355	0,488	0,39	1
1010001B2	0,000188	-0,025	0	0,025	1
Gm266	0,000188	-0,02706	0	0,025	1
Gm29642	0,000188	-0,02911	0	0,025	1
Naaa	0,000189	-0,06521	0,073	0,148	1
Slc25a19	0,000189	-0,0603	0,279	0,409	1
Zdhhc16	0,00019	-0,07777	0,141	0,234	1
Eef1akmt1	0,000191	-0,0721	0,103	0,186	1
Dnaja2	0,000191	-0,08353	0,695	0,789	1
Bub3	0,000191	-0,10174	0,818	0,907	1
Nudt9	0,000192	-0,08144	0,462	0,618	1
Cmss1	0,000192	-0,08242	0,161	0,257	1
Gsto2	0,000192	-0,0661	0,088	0,167	1
Hacd2	0,000193	-0,08636	0,352	0,494	1
Cbfb	0,000193	-0,06359	0,352	0,494	1
Pmepa1	0,000194	-0,10168	0,22	0,325	1
Nxt2	0,000194	-0,12095	0,613	0,728	1
Copg2	0,000194	-0,0911	0,339	0,466	1
Pcf11	0,000195	0,202136	0,505	0,432	1
Arhgef9	0,000195	-0,09708	0,191	0,291	1
Sap30l	0,000196	-0,0862	0,328	0,462	1
Srp19	0,000197	-0,10449	0,662	0,774	1
Slc4a1ap	0,000197	-0,08183	0,2	0,306	1
Nrip2	0,000198	-0,03663	0,011	0,051	1
Aggf1	0,000199	-0,05328	0,371	0,534	1
Mrpl35	0,0002	-0,08184	0,231	0,348	1
Ywhab	0,0002	-0,09732	0,965	0,96	1
2410004B1	0,000201	-0,07956	0,437	0,584	1
Frg1	0,000202	-0,1026	0,481	0,614	1
Rbm34	0,000203	-0,06628	0,264	0,392	1
Nedd4l	0,000204	0,163206	0,4	0,302	1
Pik3r3	0,000205	0,260565	0,325	0,238	1
Maea	0,000205	-0,06407	0,292	0,42	1
Med19	0,000205	-0,09681	0,367	0,481	1
Map2k1	0,000205	-0,08613	0,477	0,614	1
Rad18	0,000207	-0,07071	0,064	0,133	1
Azi2	0,000208	-0,0869	0,317	0,439	1
Csde1	0,000209	0,121683	0,98	0,962	1
Cotl1	0,000209	-0,08368	0,16	0,259	1
Gfer	0,00021	-0,07121	0,191	0,297	1
Necap2	0,000211	-0,0939	0,55	0,684	1
Mettl4	0,000212	-0,05497	0,035	0,091	1
Mesd	0,000214	-0,0771	0,319	0,447	1
Pnpt1	0,000216	-0,06149	0,136	0,23	1
Crbn	0,000216	-0,09579	0,336	0,464	1
Rps6ka1	0,000216	-0,07609	0,211	0,319	1
Vamp4	0,000217	-0,09133	0,354	0,477	1

Ppip5k2	0,000217	-0,08593	0,284	0,414	1
Pclo	0,000217	-0,08947	0,244	0,359	1
Nudt1	0,000218	-0,06663	0,081	0,156	1
Ndufs1	0,000218	-0,08902	0,527	0,675	1
Mfn1	0,000219	-0,10639	0,532	0,635	1
Usp39	0,000219	-0,07643	0,174	0,276	1
Gm13403	0,000219	-0,05754	0,053	0,118	1
Gas8	0,000221	-0,06646	0,121	0,209	1
2810429I0	0,000221	-0,0679	0,171	0,272	1
Slitrk5	0,000222	-0,05518	0,018	0,063	1
Lamtor3	0,000225	-0,07673	0,295	0,422	1
Ruvbl1	0,000225	-0,08537	0,301	0,426	1
Ppil1	0,000227	-0,07473	0,145	0,238	1
Smug1	0,000229	-0,07384	0,092	0,171	1
Timm44	0,000229	-0,07642	0,369	0,515	1
Hdgfl2	0,00023	-0,07504	0,332	0,466	1
Edc3	0,00023	-0,07164	0,097	0,177	1
Fam124a	0,000231	0,052846	0,039	0,004	1
Tecpr2	0,000231	-0,07961	0,332	0,47	1
Sgpl1	0,000232	0,127835	0,347	0,251	1
Dync1i2	0,000232	-0,08907	0,982	0,992	1
Pigu	0,000232	-0,08412	0,319	0,447	1
Mttp	0,000233	-0,05468	0,077	0,152	1
Mtmr14	0,000233	-0,0696	0,145	0,241	1
Dnajc3	0,000234	-0,09641	0,774	0,863	1
Plekha1	0,000234	0,184051	0,844	0,793	1
F8a	0,000235	-0,06667	0,139	0,232	1
Trappc4	0,000235	-0,10709	0,666	0,793	1
Rtcb	0,000235	-0,08372	0,492	0,65	1
Cpped1	0,000235	-0,09064	0,187	0,287	1
Rock2	0,000235	0,171353	0,717	0,69	1
Eif3i	0,000235	-0,09876	0,659	0,753	1
Med15	0,000236	-0,0641	0,286	0,422	1
Nudt16l1	0,000236	-0,09768	0,385	0,502	1
Lyrm9	0,000236	-0,07991	0,154	0,249	1
Zfand2a	0,000238	-0,07504	0,182	0,285	1
Alg2	0,000239	-0,07522	0,257	0,378	1
Fyb	0,00024	-0,03261	0,009	0,046	1
Ppm1k	0,000241	-0,0734	0,226	0,338	1
Stat2	0,000242	0,216114	0,312	0,224	1
Tram2	0,000242	0,099485	0,139	0,07	1
Them4	0,000243	-0,08005	0,242	0,359	1
Pmpca	0,000243	-0,07504	0,345	0,479	1
Celf1	0,000247	0,187737	0,624	0,582	1
Slc9b2	0,000248	-0,12257	0,055	0,118	1
Xrcc1	0,000248	-0,05257	0,213	0,329	1
2610037D	0,000248	-0,07333	0,117	0,205	1
Trim27	0,000249	-0,06438	0,13	0,222	1
Blcap	0,000249	-0,07375	0,244	0,359	1
Ahnak	0,00025	0,123731	0,059	0,015	1
Ankrd39	0,00025	-0,08607	0,196	0,3	1
Zfp65	0,00025	-0,06342	0,086	0,162	1

Nxf1	0,00025	-0,10293	0,694	0,787	1
Sdhaf2	0,00025	-0,06817	0,226	0,338	1
A930006I0	0,000251	-0,03747	0,002	0,03	1
Gria2	0,000252	-0,03136	0,002	0,03	1
Pla2g7	0,000252	-0,02727	0,002	0,03	1
Rrp36	0,000253	-0,0576	0,295	0,43	1
1110020A:	0,000254	-0,05337	0,031	0,084	1
Srp54a	0,000254	-0,03478	0,259	0,39	1
Sc5d	0,000254	0,152199	0,853	0,812	1
Hnrnpl	0,000255	0,155324	0,883	0,871	1
Nifk	0,000255	-0,05385	0,206	0,31	1
Ston2	0,000257	-0,11024	0,277	0,384	1
Insig2	0,000258	-0,07151	0,29	0,418	1
Asb8	0,000259	-0,06456	0,281	0,409	1
2610002M	0,00026	-0,06978	0,123	0,209	1
Surf2	0,00026	-0,07852	0,242	0,357	1
Zfp521	0,00026	-0,03624	0,007	0,042	1
Kif1a	0,00026	0,097477	0,989	0,981	1
Asna1	0,000261	-0,07461	0,44	0,601	1
Pcca	0,000262	-0,07715	0,215	0,319	1
Map9	0,000265	-0,07237	0,077	0,15	1
Farsb	0,000265	-0,07869	0,242	0,354	1
Snrpa	0,000265	-0,08937	0,451	0,595	1
Zfp458	0,000265	-0,0766	0,127	0,215	1
Fam98b	0,000267	-0,07271	0,29	0,418	1
Fnip2	0,000269	0,129297	0,279	0,188	1
Pura	0,000269	0,123204	0,974	0,962	1
Tbcc	0,000269	-0,07327	0,233	0,346	1
Xaf1	0,00027	-0,10029	0,554	0,681	1
Mecr	0,000273	-0,07	0,097	0,175	1
Kndc1	0,000274	0,182868	0,837	0,806	1
Psmb9	0,000277	0,057056	0,028	0	1
6720427I0	0,000277	-0,07658	0,088	0,165	1
Fam173b	0,000278	-0,09541	0,158	0,249	1
Kctd15	0,000279	0,096499	0,13	0,063	1
Surf6	0,000281	-0,08602	0,161	0,253	1
Tmem123	0,000281	-0,08168	0,451	0,603	1
Fbxl14	0,000282	-0,06464	0,15	0,245	1
Ggnbp2	0,000283	0,167014	0,67	0,624	1
Nsrp1	0,000285	-0,07301	0,314	0,441	1
Ankmy2	0,000286	-0,07255	0,103	0,184	1
Stoml1	0,000287	-0,07754	0,189	0,289	1
Noa1	0,000287	-0,07981	0,163	0,257	1
Orai1	0,000292	-0,08074	0,128	0,215	1
C2cd2	0,000295	-0,06375	0,16	0,259	1
Hscb	0,000295	-0,08375	0,176	0,272	1
Ebna1bp2	0,000296	-0,07168	0,422	0,565	1
Anxa4	0,000296	0,136048	0,312	0,219	1
Sub1	0,000297	-0,09889	0,672	0,789	1
Psmb6	0,000297	-0,10236	0,945	0,962	1
Glmn	0,000297	-0,06609	0,105	0,188	1
Ak1	0,000297	-0,09228	0,189	0,285	1

Dhfr	0,000298	-0,04801	0,117	0,207	1
Rdm1	0,000299	-0,06632	0,13	0,219	1
Mzt2	0,000303	-0,08586	0,138	0,224	1
Hacl1	0,000303	-0,0666	0,103	0,184	1
Nipsnap1	0,000305	-0,05592	0,11	0,196	1
Cox10	0,000307	-0,06688	0,083	0,156	1
Rnf6	0,000307	-0,08732	0,295	0,409	1
Mylpf	0,000308	-0,07326	0,119	0,205	1
Gm45871	0,000308	-0,05399	0,046	0,105	1
Oxct1	0,000308	-0,10244	0,582	0,705	1
Vma21	0,000309	-0,08801	0,596	0,722	1
Zfp729a	0,00031	-0,08193	0,217	0,323	1
Tmem238	0,000311	-0,09681	0,125	0,207	1
Garem1	0,000311	-0,0745	0,112	0,194	1
Lzic	0,000312	-0,07974	0,141	0,23	1
Tmbim6	0,000312	-0,07364	1	0,998	1
1810043G(	0,000313	-0,13533	0,194	0,287	1
Kxd1	0,000313	-0,08564	0,396	0,519	1
Tagln2	0,000314	0,125948	0,339	0,232	1
Slx4ip	0,000314	-0,06048	0,095	0,175	1
Dph7	0,000314	-0,07047	0,134	0,222	1
Pms1	0,000316	-0,06758	0,105	0,186	1
Rps15a	0,000317	-0,07718	0,976	0,989	1
Parp3	0,000317	0,143645	0,207	0,127	1
Retreg3	0,000319	0,167786	0,694	0,648	1
Tgfbrap1	0,000319	-0,06687	0,288	0,409	1
Polr3c	0,000319	-0,06048	0,106	0,188	1
Nt5c3	0,00032	-0,05752	0,424	0,58	1
Rpp40	0,000321	-0,05954	0,062	0,129	1
Ten1	0,000321	-0,09257	0,538	0,66	1
Bmpr2	0,000322	0,166463	0,604	0,553	1
Wdr91	0,000323	-0,08425	0,398	0,527	1
Pak1ip1	0,000323	-0,08426	0,339	0,464	1
Mpp1	0,000324	-0,07855	0,128	0,213	1
Leo1	0,000324	-0,08401	0,262	0,378	1
Abtb2	0,000325	-0,05406	0,161	0,259	1
Eif4b	0,000326	0,151409	0,736	0,703	1
Rnf135	0,000329	-0,07093	0,117	0,2	1
Kpna4	0,000329	0,156567	0,602	0,538	1
Snrnp40	0,00033	-0,08328	0,284	0,397	1
Atp5s	0,00033	-0,08779	0,141	0,228	1
Nsfl1c	0,000332	-0,09965	0,6	0,732	1
AW146154	0,000333	-0,06612	0,073	0,143	1
Snrpd1	0,000334	-0,07751	0,517	0,648	1
Nkap	0,000334	-0,07991	0,481	0,622	1
Zmat1	0,000334	-0,08632	0,295	0,416	1
Rdh5	0,000335	-0,06024	0,072	0,141	1
Cops2	0,000335	-0,07079	0,519	0,667	1
Fam129b	0,000336	0,096202	0,161	0,089	1
Oraov1	0,000338	-0,08501	0,169	0,262	1
Hdgf	0,000338	-0,09405	0,844	0,901	1
Golt1b	0,00034	-0,0858	0,312	0,43	1

Zfp639	0,000341	-0,06622	0,321	0,454	1
Utp23	0,000341	-0,0633	0,134	0,222	1
Capn10	0,000341	-0,06829	0,163	0,259	1
Zfp81	0,000341	-0,08045	0,117	0,198	1
Xbp1	0,000342	-0,08947	0,505	0,633	1
Hip1r	0,000346	0,188832	0,552	0,489	1
Cbwd1	0,000348	-0,06943	0,114	0,196	1
Plcd4	0,000348	-0,03322	0,011	0,049	1
Actn4	0,000348	0,152809	0,848	0,8	1
Akr1b3	0,000349	-0,09479	0,543	0,681	1
Fam45a	0,000349	-0,07402	0,316	0,441	1
St13	0,000349	-0,06552	0,982	0,998	1
mt-Co2	0,00035	-0,0681	1	1	1
Arhgdig	0,000352	-0,02294	0	0,023	1
Apoc1	0,000352	-0,02706	0	0,023	1
Acsl6	0,000352	-0,02706	0	0,023	1
Uba2	0,000352	-0,09008	0,572	0,7	1
Pofut2	0,000353	-0,07531	0,259	0,376	1
Zfp952	0,000354	-0,05899	0,094	0,171	1
6430503K(	0,000357	-0,07283	0,134	0,219	1
Yjefn3	0,000357	-0,06289	0,075	0,146	1
2810402E2	0,000357	-0,04476	0,072	0,143	1
Gdap10	0,000358	0,15934	0,178	0,105	1
Zmpste24	0,000358	-0,04565	0,352	0,504	1
Cul4b	0,000358	-0,06848	0,099	0,177	1
Szt2	0,000359	-0,07	0,103	0,181	1
Etnk1	0,000359	0,135976	0,939	0,911	1
Dnaja4	0,00036	-0,07395	0,105	0,184	1
Wipf2	0,00036	-0,07357	0,352	0,494	1
Gon4l	0,000362	-0,06566	0,323	0,454	1
Erbb4	0,000363	-0,08697	0,244	0,354	1
Dgat2	0,000364	0,117822	0,319	0,222	1
Acot6	0,000366	-0,05497	0,084	0,158	1
Bmp2k	0,000367	0,126417	0,921	0,888	1
Ypel5	0,00037	-0,07107	0,385	0,536	1
Arhgap29	0,00037	0,045889	0,037	0,004	1
Hnrnpul2	0,00037	0,168837	0,73	0,671	1
Lactb	0,00037	-0,07586	0,141	0,232	1
Cntfr	0,000371	0,188356	0,459	0,378	1
Fastk	0,000372	-0,06312	0,244	0,357	1
Tpgs1	0,000375	-0,07835	0,505	0,658	1
Phc3	0,000376	0,16932	0,635	0,591	1
Ccdc66	0,00038	-0,07438	0,308	0,435	1
5330434G(	0,000381	-0,05015	0,308	0,437	1
2310009B:	0,000382	-0,06459	0,086	0,16	1
Ccdc34	0,000382	-0,0633	0,411	0,557	1
Immp2l	0,000383	-0,05226	0,064	0,131	1
Amz2	0,000388	-0,07012	0,294	0,411	1
C330018D:	0,000388	-0,05669	0,106	0,188	1
Gemin2	0,00039	-0,07162	0,125	0,209	1
Uba5	0,00039	-0,06354	0,44	0,595	1
Utp3	0,000391	-0,06387	0,266	0,384	1

A830052D1	0,000392	-0,05013	0,022	0,068	1
Mterf2	0,000394	-0,05823	0,051	0,112	1
Sh3glb1	0,000396	-0,08344	0,983	0,996	1
Rilpl1	0,000396	-0,08401	0,332	0,458	1
Idua	0,000397	-0,05926	0,077	0,148	1
Mrpl3	0,000398	-0,07986	0,288	0,401	1
Ergic2	0,000399	-0,08582	0,569	0,696	1
1110034G1	0,000403	-0,06801	0,176	0,272	1
Zfp2	0,000403	-0,06767	0,161	0,259	1
Mtrr	0,000404	-0,06697	0,105	0,184	1
Gm34590	0,000404	-0,05226	0,055	0,118	1
Nudt22	0,000405	-0,0641	0,11	0,19	1
Abcf2	0,000406	-0,07523	0,215	0,319	1
Creg1	0,000407	-0,06577	0,264	0,386	1
Zfp182	0,000407	-0,06451	0,217	0,331	1
Acadvl	0,00041	-0,0896	0,429	0,57	1
Stau1	0,000411	-0,06538	0,336	0,462	1
Pfn4	0,000411	-0,06323	0,061	0,124	1
Msi2	0,000411	0,174502	0,67	0,616	1
Sept8	0,000412	-0,08551	0,993	0,989	1
Syp	0,000413	0,134166	0,259	0,173	1
Slc25a35	0,000416	-0,04216	0,037	0,091	1
Fundc1	0,000417	-0,08803	0,341	0,466	1
C87436	0,000417	-0,06873	0,233	0,346	1
Mettl25	0,000418	-0,07278	0,11	0,19	1
Sfmbt1	0,000419	-0,059	0,11	0,192	1
Mdh2	0,00042	-0,09707	0,82	0,899	1
Per3	0,000421	-0,0444	0,187	0,291	1
Chpt1	0,000421	-0,12642	0,743	0,819	1
Cdip1	0,000423	0,126496	0,848	0,812	1
Gle1	0,000424	-0,07322	0,354	0,483	1
Slc25a25	0,000424	-0,04479	0,042	0,099	1
Syt4	0,000426	0,334087	0,04	0,006	1
Thoc5	0,000426	-0,06982	0,16	0,253	1
Akap6	0,000427	-0,04569	0,376	0,53	1
Crebzf	0,00043	-0,09785	0,578	0,719	1
Rnf26	0,000431	-0,07619	0,143	0,23	1
Gm16168	0,000434	-0,05103	0,119	0,205	1
Ccdc77	0,000435	-0,05063	0,13	0,219	1
Gm36839	0,000436	0,081938	0,086	0,034	1
Pggt1b	0,000437	-0,07162	0,121	0,205	1
Sh3gl1	0,000437	-0,07162	0,299	0,418	1
Ctsd	0,000437	0,116018	0,989	0,981	1
Atp2c1	0,000438	-0,09843	0,89	0,918	1
Gm47101	0,000439	0,042743	0,031	0,002	1
Gm38534	0,00044	0,037466	0,031	0,002	1
Sh3rf1	0,000441	-0,05384	0,046	0,103	1
Ckb	0,000443	-0,13195	0,996	0,998	1
Luzp2	0,000444	-0,1325	0,314	0,42	1
Gm45518	0,000446	0,057056	0,026	0	1
Slc6a7	0,000446	0,057056	0,026	0	1
Pa2g4	0,000447	-0,10118	0,637	0,747	1

Syne3	0,00045	-0,05855	0,07	0,137	1
Rab30	0,000452	-0,0757	0,211	0,314	1
Psmd4	0,000453	-0,09823	0,824	0,905	1
Jph1	0,000454	-0,09264	0,483	0,616	1
Cops8	0,000455	-0,09715	0,523	0,643	1
Pld3	0,000456	-0,06323	0,303	0,432	1
Foxred1	0,000457	-0,0708	0,172	0,266	1
Slc37a3	0,000457	-0,06131	0,204	0,312	1
Csnk2b	0,000458	-0,09914	0,714	0,816	1
Dstyk	0,000459	0,169759	0,76	0,762	1
Rpap3	0,000459	-0,06673	0,172	0,266	1
Rtca	0,000459	-0,06321	0,18	0,278	1
Zfp62	0,000459	-0,05974	0,317	0,451	1
Nap1l1	0,00046	0,174827	0,752	0,7	1
Gm15972	0,000462	-0,04391	0,024	0,07	1
Ubxn2a	0,000463	-0,06802	0,284	0,407	1
B230307C2	0,000463	-0,08074	0,136	0,219	1
Phospho2	0,000464	-0,07494	0,141	0,228	1
Gm16141	0,000465	-0,04997	0,024	0,07	1
Ndufaf1	0,000466	-0,07	0,105	0,181	1
Slc4a4	0,000467	-0,04152	0,002	0,027	1
Hnmt	0,000468	-0,02727	0,002	0,027	1
Ermard	0,000468	-0,10381	0,191	0,287	1
Gm42205	0,000468	-0,02522	0,002	0,027	1
Zmat3	0,000469	-0,05203	0,174	0,276	1
Patz1	0,00047	-0,06807	0,251	0,365	1
E130102H2	0,00047	-0,06679	0,2	0,304	1
Ccdc58	0,000473	-0,06268	0,136	0,224	1
Casc4	0,000473	-0,10591	0,217	0,321	1
Pole3	0,000473	-0,0676	0,317	0,437	1
Slc5a11	0,000475	-0,04126	0,028	0,076	1
Hars2	0,000477	-0,0715	0,128	0,211	1
Tnfsf12	0,000477	0,078792	0,119	0,057	1
Tmem251	0,000477	-0,06017	0,158	0,251	1
Unc5b	0,000478	0,123641	0,965	0,943	1
Cwc15	0,000478	-0,09744	0,888	0,924	1
Rpgr	0,000478	-0,0665	0,077	0,146	1
Ankrd26	0,000478	-0,08517	0,273	0,384	1
Lin37	0,000478	-0,06676	0,158	0,249	1
Cntrl	0,00048	-0,06987	0,295	0,414	1
Tvp23a	0,00048	-0,04949	0,07	0,137	1
Dhps	0,00048	-0,08262	0,367	0,494	1
9430065F1	0,000481	-0,04811	0,04	0,095	1
Amd2	0,000482	0,196894	0,833	0,816	1
Prmt9	0,000485	-0,04823	0,112	0,194	1
Nrtn	0,000486	-0,10616	0,108	0,186	1
Tmem8b	0,000487	-0,07393	0,161	0,253	1
Relb	0,00049	-0,0707	0,106	0,184	1
Prickle3	0,00049	0,047586	0,112	0,051	1
Snf8	0,000492	-0,0851	0,56	0,688	1
Trib2	0,000493	-0,0457	0,017	0,057	1
Sall1	0,000495	-0,10346	0,356	0,47	1

Oma1	0,000498	-0,06632	0,13	0,215	1
Prmt2	0,0005	-0,08389	0,44	0,572	1
Tsix	0,000503	0,179612	0,29	0,205	1
Mphosph1(	0,000505	-0,04637	0,334	0,47	1
Birc2	0,000505	-0,06155	0,325	0,46	1
Baalc	0,000506	-0,03157	0,004	0,032	1
Larp1	0,000506	0,172281	0,604	0,551	1
Pign	0,000508	-0,05442	0,183	0,285	1
Tmem27	0,000509	-0,02814	0,006	0,036	1
Zfp37	0,000509	-0,05411	0,215	0,321	1
Spock2	0,00051	-0,02749	0,004	0,032	1
Sod2	0,000511	-0,09724	0,505	0,618	1
Tmf1	0,000511	-0,10531	0,725	0,8	1
Rnf32	0,000512	-0,05635	0,05	0,108	1
Nhlrc3	0,000518	-0,07546	0,123	0,205	1
Pigx	0,000519	-0,04584	0,125	0,211	1
Mul1	0,00052	-0,06547	0,217	0,321	1
Ptpn11	0,00052	0,127192	0,894	0,861	1
Zfp438	0,000521	-0,05752	0,103	0,181	1
Synj2bp	0,000522	-0,08412	0,277	0,388	1
Cep19	0,000522	-0,04794	0,04	0,095	1
Armt1	0,000522	-0,04891	0,101	0,179	1
Gtf3c6	0,000525	-0,08933	0,371	0,489	1
Gabbr1	0,000525	-0,08106	0,969	0,979	1
Pes1	0,000526	-0,06245	0,231	0,335	1
Otud6b	0,000526	-0,08091	0,378	0,504	1
Jmjd8	0,000528	-0,06498	0,117	0,198	1
Yars2	0,00053	-0,05598	0,094	0,169	1
Pms2	0,000532	-0,0496	0,169	0,266	1
Tmub2	0,000535	-0,0799	0,332	0,447	1
Atxn10	0,00054	-0,09696	0,758	0,821	1
Aamp	0,000544	-0,09729	0,894	0,897	1
Mccc2	0,000544	-0,07344	0,123	0,203	1
1700066M	0,000544	-0,07956	0,235	0,34	1
Mkrn2	0,000545	-0,08608	0,312	0,424	1
AI413582	0,000546	-0,08178	0,494	0,637	1
Zfp770	0,000546	-0,07534	0,158	0,247	1
Mrpl33	0,000547	-0,09937	0,655	0,749	1
Ppm1g	0,000552	-0,05215	0,385	0,534	1
Pwp1	0,000553	-0,06292	0,172	0,268	1
Bcl7b	0,000554	-0,08723	0,481	0,624	1
Hus1	0,000558	-0,05809	0,198	0,3	1
Mrpl45	0,000558	-0,08949	0,226	0,323	1
Zfp612	0,000558	-0,06722	0,2	0,302	1
Gpx1	0,000559	-0,16621	0,165	0,253	1
Mrpl44	0,000562	-0,05995	0,092	0,165	1
Psma4	0,000562	-0,10318	0,723	0,814	1
Dbp	0,000567	-0,08119	0,163	0,253	1
AU040972	0,000568	-0,03442	0,011	0,046	1
Pim1	0,000568	0,086448	0,062	0,019	1
Olfr718-ps:	0,00057	0,055797	0,059	0,017	1
Dgcr14	0,00057	-0,0671	0,112	0,19	1

Clint1	0,00057	-0,05677	0,262	0,38	1
Sav1	0,000571	-0,08822	0,277	0,382	1
Hnrnpu	0,000572	0,110499	0,983	0,97	1
Pus1	0,000574	-0,06127	0,117	0,198	1
Pik3r4	0,000574	-0,05865	0,16	0,253	1
Lysmd4	0,000577	-0,07141	0,101	0,175	1
Hmgb1	0,000578	-0,05245	0,998	0,996	1
Decr1	0,000578	-0,06924	0,206	0,306	1
Nsmaf	0,000584	-0,05035	0,138	0,226	1
Smu1	0,000585	-0,08657	0,428	0,553	1
Zfp384	0,000585	-0,05498	0,141	0,23	1
Atg16l1	0,000587	-0,0885	0,264	0,371	1
Larp4	0,000589	0,174394	0,572	0,525	1
Dusp12	0,000589	-0,06479	0,191	0,289	1
Txnrd2	0,00059	-0,06523	0,128	0,211	1
Casp9	0,000594	-0,06195	0,165	0,257	1
Fam241a	0,000605	-0,06775	0,228	0,331	1
Tmem170	0,000605	-0,06108	0,145	0,232	1
Maged1	0,000606	0,151551	0,646	0,599	1
Rnf14	0,000607	-0,09163	0,512	0,631	1
Cxcl14	0,000607	-0,03601	0,011	0,046	1
Plekhn1	0,000607	-0,05457	0,237	0,348	1
Pepd	0,000608	-0,09513	0,486	0,618	1
Ormdl1	0,000611	-0,08215	0,183	0,276	1
Pgm3	0,000611	-0,06105	0,231	0,34	1
Fam43a	0,000611	-0,06547	0,039	0,091	1
Dnpep	0,000612	-0,06206	0,312	0,432	1
Exoc8	0,000614	-0,0651	0,123	0,205	1
Magi2	0,000614	0,128878	0,936	0,899	1
Pfn1	0,000615	-0,06281	0,983	0,985	1
Tysnd1	0,000615	-0,06662	0,193	0,287	1
Thap2	0,000615	-0,06699	0,472	0,599	1
Ptk2	0,000616	-0,09271	0,712	0,806	1
BC022687	0,000616	-0,03978	0,022	0,065	1
Ahcy	0,000619	-0,0412	0,018	0,059	1
Ubap1	0,000623	-0,08249	0,352	0,475	1
Zfp160	0,000626	-0,07201	0,211	0,312	1
Afg3l1	0,000626	-0,06108	0,415	0,549	1
Srsf10	0,000628	0,155454	0,653	0,603	1
Get4	0,000634	-0,07901	0,536	0,662	1
Fam172a	0,000635	-0,07603	0,536	0,684	1
Gtf2e2	0,000638	-0,09042	0,22	0,312	1
Inip	0,00064	-0,07578	0,13	0,211	1
Rab12	0,000641	-0,06887	0,339	0,464	1
Pdia3	0,000641	-0,09996	0,958	0,954	1
Rnf168	0,000642	-0,05069	0,306	0,432	1
Mat2a	0,000643	-0,10781	0,98	0,968	1
Thap12	0,000648	-0,07879	0,312	0,424	1
Azin1	0,000649	0,173455	0,451	0,384	1
Lman2l	0,000653	-0,06882	0,193	0,289	1
Numb	0,000655	-0,06302	0,204	0,304	1
Rnaseh2c	0,000655	-0,0943	0,49	0,61	1

4921504A2	0,000655	-0,08157	0,114	0,19	1
Abcd3	0,000657	-0,09213	0,42	0,546	1
Snta1	0,000661	-0,06615	0,18	0,276	1
Tshz2	0,000661	-0,02294	0	0,021	1
Prkce	0,000661	0,06959	0,075	0,027	1
Rab3gap1	0,000669	-0,04585	0,402	0,553	1
Wdr35	0,000671	-0,07823	0,099	0,171	1
A430046D1	0,000673	-0,05381	0,062	0,124	1
Trim46	0,000673	0,080308	0,09	0,038	1
Zfp973	0,000677	-0,04692	0,033	0,082	1
Polr3a	0,00068	-0,06016	0,176	0,27	1
Fam219b	0,000681	-0,06839	0,204	0,304	1
Cyp39a1	0,000682	-0,06794	0,116	0,194	1
Ceacam1	0,000686	-0,05272	0,044	0,099	1
Grid2	0,00069	-0,09254	0,253	0,35	1
Mrpl22	0,000691	-0,05645	0,183	0,281	1
Ogt	0,000691	-0,08771	0,789	0,867	1
Usp30	0,000691	-0,06384	0,394	0,534	1
Dnajc10	0,000692	-0,08005	0,301	0,416	1
Pigc	0,000696	-0,06706	0,127	0,209	1
Trpv2	0,000698	0,055664	0,072	0,025	1
Ap5z1	0,000698	-0,06802	0,244	0,352	1
Mrpl17	0,000699	-0,09084	0,53	0,656	1
Arf2	0,000701	-0,06611	0,237	0,344	1
Parn	0,000702	-0,05911	0,29	0,405	1
Cul2	0,000708	-0,05527	0,213	0,319	1
Mybphl	0,000708	0,037466	0,029	0,002	1
Trappc9	0,000716	-0,0576	0,284	0,407	1
Slc45a3	0,000717	-0,09022	0,198	0,291	1
Mettl3	0,000719	-0,0806	0,202	0,295	1
Pafah1b2	0,000728	-0,09883	0,853	0,886	1
Rhbdd1	0,000728	-0,05446	0,11	0,188	1
Pigk	0,000728	-0,07043	0,424	0,563	1
Diaph2	0,000729	-0,08623	0,187	0,281	1
Zmat2	0,000731	-0,08677	0,855	0,884	1
Irf2bp1	0,000732	-0,0633	0,325	0,443	1
Chmp7	0,000736	-0,08014	0,749	0,871	1
Zbtb42	0,000738	0,039607	0,039	0,006	1
Mrpl4	0,000742	-0,08587	0,303	0,411	1
Acrbp	0,000743	-0,06663	0,077	0,143	1
Myc	0,000747	0,115568	0,081	0,032	1
Hnrnph2	0,000747	-0,09394	0,721	0,829	1
Polr2a	0,000747	0,17844	0,547	0,511	1
Lsm4	0,000749	-0,09199	0,624	0,732	1
Srr	0,00075	-0,06696	0,18	0,272	1
Ubl3	0,000751	-0,10334	0,941	0,97	1
Fpgt	0,000755	-0,07563	0,191	0,283	1
Vps8	0,000756	-0,0726	0,347	0,464	1
Ap1s1	0,000757	-0,08938	0,327	0,437	1
Nr2f6	0,000758	-0,06473	0,121	0,2	1
Hist2h3c1	0,000759	-0,0441	0,053	0,112	1
Peg3	0,00076	-0,08933	0,292	0,407	1

Tmem18	0,000761	-0,07092	0,152	0,238	1
Zfp74	0,000767	-0,05811	0,191	0,287	1
Adam23	0,000768	-0,10143	0,33	0,449	1
Babam1	0,000769	-0,06108	0,378	0,515	1
Ap2s1	0,000771	-0,09688	0,837	0,886	1
Stard5	0,000771	-0,06438	0,121	0,2	1
Pcbd2	0,000776	-0,08199	0,196	0,287	1
Dip2b	0,000781	-0,08028	0,961	0,968	1
5730403I0	0,000783	-0,03462	0,013	0,049	1
Ctps2	0,00079	-0,06293	0,141	0,226	1
Dynlrb1	0,00079	-0,09843	0,903	0,911	1
Zfp35	0,000792	-0,06275	0,081	0,148	1
Hars	0,000795	-0,07126	0,462	0,601	1
Dpysl5	0,000798	-0,06536	0,147	0,234	1
Coro1b	0,000799	-0,06582	0,393	0,525	1
Chek2	0,000799	0,07806	0,13	0,068	1
Ttc38	0,0008	-0,06496	0,081	0,148	1
Ndufa9	0,000801	-0,07483	0,477	0,618	1
Ddx21	0,000801	-0,06015	0,306	0,42	1
Arf4	0,000803	-0,08926	0,703	0,768	1
Manea	0,000804	-0,05779	0,251	0,361	1
Lyar	0,000805	-0,06371	0,158	0,247	1
Psma6	0,000812	-0,09379	0,739	0,812	1
Gk	0,000816	-0,05165	0,062	0,124	1
Crk	0,000816	0,147839	0,668	0,616	1
Kpna3	0,000819	-0,07367	0,391	0,523	1
Mfsd13b	0,000819	-0,03741	0,017	0,055	1
Yipf5	0,00082	-0,06017	0,527	0,679	1
Eny2	0,000822	-0,07327	0,611	0,745	1
Ppp1r3f	0,000828	-0,04813	0,072	0,137	1
Wdr26	0,000829	0,147842	0,655	0,633	1
Khdrbs1	0,000833	0,120908	0,826	0,783	1
Lpar6	0,000837	-0,04684	0,095	0,167	1
Rfxap	0,000839	-0,06134	0,306	0,42	1
Mpdz	0,000839	-0,06574	0,363	0,492	1
Mterf3	0,000842	-0,05355	0,248	0,359	1
Nr4a3	0,000844	0,086893	0,05	0,013	1
Ldlrad3	0,000844	0,175241	0,558	0,519	1
Spopl	0,000846	-0,05742	0,206	0,306	1
Pstk	0,000847	-0,07437	0,213	0,31	1
Ptprs	0,000848	0,172254	0,385	0,312	1
Dkc1	0,000851	-0,05738	0,27	0,384	1
Ggact	0,000852	-0,07024	0,108	0,181	1
Pdcd4	0,000858	-0,08816	0,327	0,439	1
Mospd2	0,000861	-0,04681	0,319	0,443	1
Macrod2	0,000861	-0,05781	0,172	0,266	1
Ngly1	0,000862	-0,05554	0,251	0,363	1
Ccdc51	0,000865	-0,04409	0,026	0,07	1
Fam181b	0,000867	-0,02727	0,002	0,025	1
Socs2	0,000876	0,059579	0,046	0,011	1
Frmd4b	0,000876	0,144982	0,886	0,871	1
Dusp16	0,000876	-0,06236	0,374	0,5	1

U2surp	0,000882	0,1361	0,82	0,795	1
Stard4	0,000887	0,198939	0,325	0,249	1
Wdr11	0,000889	-0,06197	0,187	0,283	1
2900076A(	0,000892	-0,05428	0,13	0,211	1
Gsta3	0,000892	-0,04462	0,026	0,07	1
Pnmal2	0,000893	-0,04462	0,035	0,084	1
Pfdn2	0,000894	-0,0778	0,813	0,886	1
Ube2e2	0,000899	-0,07419	0,684	0,814	1
Slc29a1	0,0009	0,047228	0,05	0,013	1
Zfand1	0,000901	-0,0572	0,094	0,165	1
Cfap97	0,000901	-0,05552	0,191	0,291	1
Gm21988	0,000904	-0,05446	0,053	0,11	1
Ppfia2	0,000909	-0,05337	0,039	0,089	1
Itpk1	0,00091	0,150542	0,899	0,876	1
Sirpa	0,000924	0,087081	0,128	0,068	1
Smim15	0,000924	-0,07604	0,754	0,823	1
Rcc1	0,000924	-0,06417	0,077	0,141	1
Ralyl	0,000927	-0,04052	0,026	0,07	1
Atp1b1	0,000927	-0,02749	0,004	0,03	1
Esrp1	0,000931	-0,02545	0,004	0,03	1
mt-Nd6	0,000934	-0,08501	0,385	0,517	1
Mgme1	0,000935	-0,05299	0,158	0,247	1
Rab3gap2	0,000938	-0,06825	0,363	0,489	1
Klhl22	0,00094	-0,06008	0,105	0,177	1
Rpl41	0,00094	-0,05011	0,998	0,998	1
Napg	0,000943	-0,07459	0,398	0,532	1
Cep57	0,000944	-0,06212	0,136	0,217	1
Arrdc2	0,000948	-0,07197	0,338	0,466	1
Mindy2	0,000948	0,160594	0,732	0,688	1
Psmb8	0,000949	0,072627	0,033	0,004	1
Timm29	0,00095	-0,06139	0,244	0,344	1
Cep192	0,00095	-0,06413	0,119	0,196	1
Sf1	0,00095	0,133242	0,681	0,629	1
Clk4	0,000952	0,149615	0,75	0,736	1
Pithd1	0,000952	-0,09698	0,653	0,757	1
Tpr	0,000953	0,145379	0,888	0,85	1
Plekha3	0,000953	-0,06615	0,194	0,289	1
Fars2	0,000954	-0,05839	0,163	0,249	1
C920006O:	0,000955	-0,0647	0,073	0,137	1
Ptpn1	0,000958	-0,06836	0,284	0,395	1
Irx5	0,000963	0,11017	0,099	0,046	1
Ttc32	0,000963	-0,05271	0,099	0,173	1
Gm28172	0,000963	-0,03818	0,022	0,063	1
Nsmce2	0,000963	-0,06446	0,407	0,544	1
Ddx47	0,000964	-0,07008	0,22	0,316	1
Galk1	0,000964	-0,04899	0,361	0,496	1
Sun2	0,000965	0,14285	0,391	0,312	1
Lonp2	0,000966	-0,06266	0,387	0,517	1
2610008E1	0,000967	-0,07459	0,24	0,34	1
Usp12	0,000967	-0,06749	0,15	0,234	1
Tmem62	0,000968	-0,07786	0,321	0,445	1
Fkbp14	0,000971	-0,0471	0,079	0,146	1

Cfl2	0,000971	-0,04799	0,987	0,985	1
C1ql3	0,000972	-0,05026	0,103	0,177	1
Dcp1a	0,000972	-0,05323	0,193	0,289	1
Ccdc22	0,000974	-0,07437	0,22	0,316	1
Adat2	0,000974	-0,05002	0,046	0,099	1
Ing4	0,000974	-0,05436	0,49	0,643	1
Enpp2	0,000977	-0,06573	1	0,998	1
Nudt13	0,000977	-0,06296	0,33	0,458	1
Zdhhc6	0,000979	-0,05511	0,18	0,274	1
Dgkz	0,000979	0,154207	0,461	0,392	1
Tmem220	0,000979	-0,05412	0,062	0,122	1
Dcun1d1	0,000982	-0,05236	0,369	0,504	1
Uxt	0,000984	-0,05455	0,193	0,289	1
5830454E(	0,000984	-0,03462	0,011	0,044	1
Eif2b2	0,000984	-0,06636	0,294	0,407	1
Acin1	0,000985	0,153173	0,807	0,787	1
Asap1	0,000987	-0,0734	0,306	0,424	1
Trim3	0,000988	-0,08101	0,51	0,637	1
Slc7a1	0,00099	0,057204	0,073	0,027	1
Vwa8	0,000992	-0,06193	0,194	0,291	1
Map2k2	0,000992	-0,06846	0,822	0,88	1
Tcf25	0,000998	0,112434	0,971	0,954	1
6330403K(	0,001	-0,02878	0,011	0,044	1
Rbks	0,001	-0,0734	0,101	0,171	1
Txnip	0,001003	0,199947	0,479	0,411	1
Naaladl2	0,001007	-0,08089	0,106	0,177	1
Hps4	0,001019	-0,05515	0,167	0,255	1
Nat8f4	0,001021	-0,0308	0,011	0,044	1
Gabra2	0,001022	-0,02899	0,011	0,044	1
D8Ertd738	0,001027	-0,08143	0,903	0,903	1
Zc2hc1a	0,001028	-0,05839	0,149	0,234	1
Mid2	0,001034	0,091815	0,119	0,061	1
Nlgn1	0,001035	-0,05352	0,033	0,08	1
Nmrk1	0,001035	-0,07126	0,134	0,213	1
Rpp38	0,001035	-0,06483	0,081	0,146	1
Pcnx4	0,001036	-0,0475	0,147	0,232	1
Eci2	0,001043	-0,0705	0,343	0,466	1
Nipal3	0,001044	-0,09245	0,428	0,54	1
Isoc2b	0,00105	-0,03522	0,015	0,051	1
Snx25	0,001051	-0,03481	0,022	0,063	1
Proser1	0,001053	-0,04861	0,193	0,289	1
Polr3gl	0,001053	-0,0717	0,152	0,234	1
Rnf103	0,001055	-0,08452	0,308	0,418	1
Tada1	0,001056	-0,08641	0,486	0,61	1
Ccdc86	0,001057	-0,06658	0,211	0,306	1
Tex9	0,001058	-0,06534	0,081	0,146	1
Celf2	0,001058	-0,05384	0,028	0,072	1
Samd4b	0,001065	0,128134	0,664	0,616	1
5430403G:	0,001069	-0,05083	0,046	0,099	1
Wdr33	0,00107	-0,05088	0,253	0,363	1
Cd99l2	0,001074	-0,05664	0,286	0,397	1
Calu	0,001075	0,151052	0,514	0,458	1

Slc25a46	0,001077	-0,07413	0,33	0,443	1
Tmem9	0,001083	-0,05686	0,079	0,143	1
Mylk	0,001083	0,071331	0,037	0,006	1
Iba57	0,00109	-0,03322	0,015	0,051	1
9530068E(	0,001097	-0,07383	0,585	0,717	1
Usp34	0,001098	0,152002	0,758	0,724	1
Lctl	0,001101	-0,03121	0,015	0,051	1
Cdc23	0,001115	-0,07039	0,248	0,35	1
Irgq	0,001116	0,090095	0,154	0,089	1
Npr2	0,001116	0,038541	0,037	0,006	1
Gm17056	0,001116	0,040294	0,037	0,006	1
Fbxo9	0,00112	-0,05823	0,411	0,549	1
Isg20l2	0,00112	-0,06385	0,112	0,186	1
Gm49207	0,00112	-0,04869	0,064	0,124	1
Csgalnact2	0,001122	-0,05173	0,15	0,236	1
Ube2w	0,001123	-0,07359	0,31	0,42	1
Nae1	0,001123	-0,04777	0,36	0,489	1
Pygo2	0,001123	-0,05001	0,187	0,283	1
Sac3d1	0,001127	-0,05821	0,119	0,196	1
Arrb1	0,00113	0,079508	0,084	0,036	1
Ppp6c	0,001131	-0,05417	0,483	0,622	1
Rnf144a	0,001132	-0,07031	0,275	0,384	1
2310061I0	0,001134	-0,0639	0,193	0,285	1
Nr4a2	0,001137	0,063577	0,028	0,002	1
Ppp1r37	0,001138	-0,08995	0,483	0,605	1
Ccdc25	0,001139	-0,06923	0,24	0,342	1
Myo9a	0,001141	0,186831	0,62	0,591	1
Hoxa4	0,001149	-0,0669	0,217	0,31	1
Cmc2	0,001156	-0,05823	0,141	0,224	1
Hecw2	0,001159	-0,07807	0,472	0,608	1
Wdr55	0,00116	-0,05109	0,105	0,177	1
Nipsnap2	0,00116	-0,07953	0,472	0,614	1
Nop53	0,001163	0,144407	0,747	0,713	1
Gm21847	0,001164	0,030716	0,022	0	1
Clec2d	0,001164	0,048353	0,022	0	1
Dmwd	0,001164	-0,08984	0,539	0,654	1
Dynlt1a	0,001165	-0,05613	0,094	0,162	1
Cdk2	0,001165	0,068823	0,084	0,036	1
Kmt5b	0,001169	0,178239	0,578	0,538	1
Eaf1	0,001171	-0,04671	0,145	0,23	1
Rnmt	0,001174	-0,04687	0,264	0,378	1
Sypl	0,001175	-0,15802	0,994	0,994	1
Rbm7	0,001175	-0,05694	0,44	0,584	1
3110002H:	0,001176	-0,05941	0,154	0,238	1
Palm2	0,001179	-0,05753	0,237	0,346	1
Tns1	0,001179	0,145849	0,376	0,295	1
Gpank1	0,00118	-0,05524	0,18	0,272	1
Rabgap1	0,001188	-0,05662	0,497	0,652	1
Cdk7	0,001192	-0,06834	0,183	0,27	1
Hax1	0,001192	-0,05705	0,374	0,502	1
Sugp1	0,001193	-0,07809	0,246	0,346	1
Mrpl19	0,001194	-0,07118	0,237	0,338	1

Gm8186	0,001196	-0,05415	0,051	0,105	1
Top1	0,001204	0,16661	0,868	0,842	1
Gm6297	0,001207	-0,03639	0,024	0,065	1
D130017N	0,00121	-0,06338	0,121	0,196	1
Dctn5	0,001211	-0,06158	0,325	0,449	1
4930539J0	0,001215	-0,04267	0,029	0,074	1
Irak1	0,001222	-0,0602	0,251	0,354	1
Gm43713	0,001224	-0,05783	0,139	0,219	1
Pex19	0,001224	-0,07043	0,437	0,574	1
Iqcc	0,001229	-0,05356	0,141	0,224	1
Pqbp1	0,001233	-0,08737	0,435	0,561	1
B930036N1	0,001242	0,055301	0,075	0,03	1
Scg2	0,001243	-0,01881	0	0,019	1
Hoxb8	0,001243	-0,01881	0	0,019	1
Hbb-bt	0,001243	-1,6069	0	0,019	1
Fgd3	0,001244	-0,05178	0,187	0,281	1
Psmf1	0,001245	-0,07069	0,207	0,3	1
Tmed7	0,001246	0,122609	0,956	0,939	1
Gm28875	0,001252	-0,06729	0,169	0,257	1
Tfdp1	0,00126	-0,07059	0,158	0,241	1
Thumpd3	0,001261	-0,06291	0,207	0,302	1
Tmem167b	0,001262	-0,0623	0,283	0,39	1
Zbtb43	0,001262	-0,05542	0,165	0,251	1
Zfp277	0,001263	-0,05826	0,224	0,325	1
Lin7c	0,001264	-0,06944	0,372	0,489	1
Vcl	0,001267	-0,05768	0,061	0,118	1
Cd46	0,001268	-0,03678	0,024	0,065	1
Tmem160	0,001269	-0,07214	0,341	0,451	1
Mcrs1	0,001269	-0,06954	0,407	0,538	1
Ptpn13	0,00127	-0,04064	0,013	0,046	1
Rpp14	0,001273	-0,07294	0,231	0,325	1
Zfp959	0,001275	-0,04943	0,05	0,103	1
Twf1	0,001277	-0,08681	0,758	0,848	1
Leng1	0,001279	-0,06777	0,253	0,354	1
Nod1	0,00128	0,127348	0,259	0,184	1
Apba3	0,001282	-0,06138	0,18	0,268	1
Tmc6	0,001283	-0,06945	0,268	0,373	1
Gm26909	0,001284	-0,08497	0,116	0,188	1
Ints6	0,001284	0,228784	0,435	0,38	1
Mterf4	0,001285	-0,06535	0,108	0,179	1
Clpx	0,001286	-0,05468	0,301	0,414	1
Cog1	0,001291	-0,06005	0,163	0,249	1
Bdkrb2	0,001293	-0,0562	0,079	0,143	1
Gm46447	0,001305	-0,03848	0,035	0,082	1
Glmp	0,001305	-0,08713	0,583	0,7	1
Dimt1	0,001307	-0,06092	0,16	0,247	1
Zranb2	0,001312	-0,09046	0,745	0,823	1
Hes6	0,001317	-0,05391	0,097	0,167	1
Gm12089	0,001318	0,04135	0,044	0,011	1
Nucks1	0,00132	0,101282	0,985	0,962	1
Ttf1	0,001323	-0,05092	0,209	0,31	1
Fam118b	0,001325	-0,05762	0,103	0,173	1

Xrcc5	0,001327	-0,05077	0,152	0,236	1
Arel1	0,001328	-0,07904	0,255	0,357	1
Coil	0,001331	-0,05227	0,094	0,162	1
Nsun2	0,001333	-0,08013	0,369	0,483	1
Epb41l2	0,001336	0,135562	0,85	0,812	1
Pxmp4	0,001341	-0,02907	0,294	0,405	1
Tmcc2	0,001345	0,124931	0,905	0,886	1
Kcnq3	0,001345	-0,08207	0,629	0,749	1
Scfd2	0,001345	-0,07139	0,121	0,194	1
Zfp444	0,001349	-0,06535	0,11	0,181	1
Clpp	0,001352	-0,10016	0,532	0,637	1
Ccnjl	0,001354	-0,02719	0,013	0,046	1
Trappc12	0,001354	-0,04837	0,314	0,439	1
1700113A1	0,001358	-0,04986	0,094	0,162	1
Alg14	0,001358	-0,06236	0,283	0,392	1
Setd3	0,001362	-0,08615	0,727	0,812	1
Med20	0,001363	-0,05626	0,224	0,321	1
Psma1	0,001366	-0,07652	0,585	0,698	1
Kpna1	0,001366	-0,04355	0,33	0,454	1
Pank1	0,001369	-0,0448	0,031	0,076	1
Gm4876	0,001382	-0,03199	0,007	0,036	1
Csrnp1	0,001385	0,255511	0,198	0,133	1
Fam120a	0,001387	0,162413	0,622	0,601	1
Nr3c1	0,001393	0,114184	0,949	0,918	1
E130307A1	0,001395	-0,05334	0,2	0,295	1
Zbtb22	0,001402	-0,05928	0,169	0,253	1
Rpl18a	0,001402	0,119142	1	0,994	1
Gm7967	0,001405	-0,12724	0,022	0,061	1
Cdh6	0,001406	-0,0508	0,123	0,203	1
Gm996	0,001408	-0,04411	0,099	0,169	1
Med1	0,001411	-0,03552	0,413	0,568	1
Brd8	0,001411	0,14464	0,635	0,601	1
Sema7a	0,001413	0,173872	0,613	0,572	1
Acat1	0,001413	-0,0914	0,556	0,667	1
Fahd2a	0,001417	-0,06596	0,132	0,209	1
Harbi1	0,001417	-0,05783	0,066	0,124	1
Jakmip2	0,001417	-0,07679	0,231	0,329	1
Cstf3	0,001422	-0,04847	0,198	0,293	1
Agap3	0,001429	-0,07529	0,334	0,445	1
Oxr1	0,001429	-0,09399	0,683	0,787	1
Sec14l5	0,001435	0,157719	0,76	0,755	1
Gm12184	0,001443	-0,0412	0,062	0,12	1
Pmm2	0,001445	-0,04414	0,297	0,42	1
Ttc5	0,001448	-0,04026	0,336	0,468	1
Fam118a	0,001448	-0,05468	0,09	0,156	1
Psma7	0,001448	-0,08166	0,963	0,956	1
Gpn3	0,001451	-0,04645	0,251	0,359	1
Zfp58	0,001452	-0,05241	0,046	0,097	1
Poc5	0,001454	-0,0644	0,222	0,319	1
Hyal1	0,001455	-0,05497	0,092	0,158	1
Spock1	0,001457	0,155983	0,772	0,703	1
Trp53i13	0,001464	-0,06538	0,349	0,464	1

Slc25a3	0,001465	-0,09372	0,928	0,935	1
Adra1b	0,001472	-0,057	0,066	0,124	1
9330111N(	0,00148	-0,06377	0,048	0,099	1
Ddx54	0,001483	-0,0408	0,316	0,439	1
Adck1	0,001483	-0,0644	0,16	0,241	1
Gab2	0,001485	-0,04602	0,106	0,179	1
Chtf8	0,001488	-0,05942	0,218	0,312	1
Ddx52	0,001495	-0,06176	0,255	0,359	1
Shkbp1	0,001499	-0,06422	0,194	0,285	1
Hlcs	0,001507	-0,0663	0,152	0,234	1
Tbc1d12	0,001519	-0,05714	0,372	0,502	1
Igdcc4	0,00152	0,045181	0,031	0,004	1
Cyp3a13	0,00152	-0,0673	0,068	0,127	1
Smarce1	0,001523	-0,07097	0,459	0,572	1
D430042O	0,001525	-0,06407	0,2	0,289	1
Fam19a2	0,001527	0,042044	0,031	0,004	1
Mkks	0,00153	-0,05848	0,121	0,194	1
Emc3	0,001533	-0,07619	0,681	0,789	1
Arl10	0,001539	-0,05012	0,077	0,139	1
Alg11	0,001545	-0,05906	0,206	0,297	1
Ppp4r3b	0,001564	-0,05811	0,462	0,584	1
Mettl26	0,001565	-0,04971	0,354	0,489	1
Timm21	0,001567	-0,04887	0,224	0,323	1
Dusp22	0,001567	-0,05738	0,161	0,245	1
Zfr2	0,001568	-0,05995	0,081	0,143	1
Ctnna1	0,001569	0,128236	0,837	0,81	1
Hspb6	0,00158	-0,05177	0,04	0,089	1
Cnot11	0,00158	-0,06343	0,152	0,232	1
Mapkapk5	0,001583	-0,05663	0,095	0,162	1
Ubald1	0,001589	-0,06933	0,439	0,553	1
Bag4	0,001589	-0,05221	0,261	0,369	1
Commd2	0,001592	-0,06323	0,246	0,344	1
Cyc1	0,001594	-0,09766	0,692	0,759	1
Lgalsl	0,001596	-0,04548	0,055	0,11	1
Chil1	0,001597	-0,04034	0,028	0,07	1
Chtop	0,001598	0,120012	0,754	0,715	1
Fbxo18	0,001601	-0,08577	0,165	0,243	1
Sco2	0,001602	-0,06064	0,101	0,169	1
Ncald	0,001603	-0,06268	0,826	0,861	1
AW209491	0,001606	-0,04009	0,11	0,184	1
1500009C(	0,001614	-0,02522	0,002	0,023	1
Slbp	0,001615	0,175413	0,294	0,222	1
Impdh2	0,001615	-0,04085	0,349	0,481	1
Nrxn3	0,001617	-0,02317	0,002	0,023	1
Gm42413	0,00162	-0,02111	0,002	0,023	1
Lama2	0,00162	-0,02111	0,002	0,023	1
Cyba	0,00162	-0,02111	0,002	0,023	1
Nsg2	0,00162	-0,02111	0,002	0,023	1
Bbs2	0,001627	-0,05873	0,108	0,179	1
Prr14l	0,001631	-0,04592	0,233	0,335	1
Zfp397	0,001632	-0,04713	0,382	0,5	1
Slc9a3r2	0,001632	0,253019	0,352	0,285	1

Tango2	0,001636	-0,05785	0,283	0,397	1
Ncf2	0,001638	-0,1804	0,011	0,042	1
Gpr61	0,001638	-0,04777	0,04	0,089	1
3300002I0	0,00165	-0,03038	0,011	0,042	1
Wdr47	0,00165	-0,06257	0,288	0,392	1
C1ql1	0,001652	-0,02134	0,002	0,023	1
Ubn1	0,001656	0,15666	0,628	0,608	1
Cd2bp2	0,001659	-0,05586	0,299	0,414	1
Mob3b	0,001662	0,128473	0,629	0,555	1
Prkdc	0,001664	-0,07153	0,191	0,278	1
Siah1a	0,001673	-0,06613	0,336	0,454	1
Hacd3	0,001675	-0,05533	0,264	0,369	1
Mplkip	0,001679	-0,046	0,242	0,348	1
Iglon5	0,001681	0,059375	0,035	0,006	1
Btbd2	0,001687	-0,05928	0,172	0,255	1
Adsl	0,001688	-0,06077	0,189	0,274	1
Abhd8	0,001691	-0,02545	0,004	0,027	1
Rpl35	0,001692	-0,04674	0,965	0,958	1
Hexim1	0,001693	0,192028	0,31	0,241	1
Coasy	0,001695	-0,07408	0,358	0,466	1
Cbx6	0,001696	0,149899	0,657	0,608	1
Rgs7	0,001697	-0,02339	0,004	0,027	1
Gm35850	0,001697	-0,02339	0,004	0,027	1
Msrb1	0,001697	-0,14966	0,738	0,791	1
Ncoa7	0,001699	-0,07223	0,277	0,38	1
Mindy3	0,001709	-0,06631	0,424	0,555	1
Gm26759	0,001709	-0,02794	0,028	0,07	1
Snx3	0,00171	-0,07757	0,93	0,951	1
Gm16894	0,001715	-0,03561	0,018	0,055	1
Tsr2	0,001726	-0,06052	0,288	0,397	1
Akap17b	0,001729	-0,05492	0,051	0,103	1
Cend1	0,00173	-0,05474	0,356	0,475	1
Gm10371	0,00173	-0,03502	0,015	0,049	1
Nelfa	0,001733	-0,06559	0,15	0,23	1
Pus7	0,001734	-0,07596	0,31	0,42	1
Mrm3	0,001738	-0,05709	0,062	0,118	1
Samd1	0,00174	-0,03872	0,387	0,523	1
Trappc10	0,001747	-0,08947	0,807	0,884	1
Nelfe	0,001749	-0,04165	0,323	0,449	1
Iqgap1	0,001751	0,149224	0,367	0,289	1
Mtmr11	0,001755	0,035026	0,035	0,006	1
Trim24	0,001758	-0,05712	0,308	0,416	1
Zfp932	0,00176	-0,07514	0,183	0,268	1
Sema3e	0,001764	-0,03542	0,015	0,049	1
Gm15417	0,001764	-0,07819	0,233	0,323	1
Ccdc174	0,001767	-0,03884	0,356	0,479	1
Esyt2	0,001776	0,158209	0,36	0,304	1
Nkain2	0,001779	0,120604	0,868	0,846	1
Lpgat1	0,001779	0,128718	0,93	0,869	1
Gm1673	0,001786	-0,03837	0,018	0,055	1
Tada2b	0,001787	-0,05673	0,16	0,243	1
Gart	0,00179	-0,05471	0,36	0,485	1

Rexo5	0,001799	-0,02941	0,015	0,049	1
Cfap20	0,001802	-0,04584	0,339	0,464	1
Slc33a1	0,001803	-0,06966	0,295	0,401	1
Nob1	0,001804	-0,0535	0,172	0,259	1
Gm13594	0,001805	-0,04249	0,029	0,072	1
Zfand3	0,001806	-0,06353	0,374	0,494	1
Parp9	0,001807	0,044496	0,026	0,002	1
Ercc8	0,001808	-0,05821	0,119	0,192	1
Map2k4	0,001814	-0,07984	0,239	0,329	1
Dnajc2	0,001818	-0,05723	0,277	0,382	1
Tulp3	0,001823	-0,32935	0,303	0,403	1
Kptn	0,001824	-0,06968	0,222	0,314	1
Cdk2ap2	0,001824	-0,0695	0,372	0,492	1
Rpf1	0,001826	-0,05816	0,171	0,253	1
Arl3	0,001829	-0,07479	0,538	0,662	1
Coq3	0,00183	-0,06663	0,086	0,148	1
Fgfr1op	0,00183	-0,04103	0,097	0,167	1
Ppp1r7	0,001833	-0,06767	0,428	0,538	1
Wscd2	0,001834	-0,03442	0,024	0,063	1
Pik3ip1	0,001839	-0,08139	0,626	0,736	1
Nisch	0,001843	0,097766	0,939	0,914	1
Chfr	0,001844	-0,04081	0,336	0,458	1
Rbm17	0,001844	-0,06333	0,396	0,517	1
Actg1	0,001857	0,138963	0,998	0,996	1
Skp1a	0,001864	-0,08158	0,817	0,873	1
Gatad1	0,001865	-0,06894	0,646	0,747	1
Acot9	0,00187	-0,06007	0,128	0,203	1
Mfsd13a	0,001871	-0,06926	0,158	0,236	1
Txnl1	0,001881	-0,08307	0,774	0,869	1
Col13a1	0,001883	0,076042	0,092	0,042	1
Mre11a	0,001883	-0,06287	0,114	0,186	1
Rgs14	0,001885	0,030716	0,02	0	1
Fbxo31	0,001886	-0,06942	0,316	0,422	1
Slc30a9	0,001887	-0,09788	0,527	0,637	1
Fam222b	0,001888	-0,07691	0,145	0,219	1
Fiz1	0,001888	-0,06719	0,281	0,382	1
Oaz2	0,001891	-0,06434	0,339	0,451	1
Sod1	0,001892	0,145693	0,734	0,703	1
Tmem132c	0,001894	0,048653	0,039	0,008	1
Alg3	0,001895	-0,05605	0,083	0,143	1
Arl6ip4	0,001898	-0,05839	0,382	0,506	1
Mfsd14a	0,001902	-0,07867	0,772	0,833	1
Tnxb	0,001913	0,058622	0,042	0,011	1
Mpg	0,00192	-0,06872	0,103	0,169	1
Slc39a13	0,001927	-0,06154	0,222	0,314	1
Zswim7	0,001928	-0,05229	0,156	0,238	1
Sgip1	0,001931	-0,07595	0,235	0,329	1
2510002D:	0,001932	-0,0494	0,081	0,143	1
Add3	0,001941	-0,13065	0,818	0,854	1
Nle1	0,001943	-0,04078	0,042	0,091	1
Pstpip2	0,001947	-0,06524	0,158	0,236	1
Klf10	0,001948	0,143833	0,149	0,089	1

Tbl1x	0,001949	-0,05247	0,385	0,511	1
B230344G:	0,001957	-0,05139	0,108	0,177	1
Nek3	0,001957	-0,05115	0,053	0,105	1
Hbs1l	0,001963	-0,06326	0,323	0,437	1
Chst2	0,001963	-0,1173	0,345	0,449	1
Upf3b	0,001967	-0,06506	0,556	0,667	1
Mapkap1	0,001972	-0,05537	0,343	0,466	1
Gm12166	0,001976	-0,04986	0,046	0,095	1
Zfp809	0,001977	-0,05141	0,116	0,188	1
Tmem151a	0,001978	-0,07289	0,998	0,998	1
Yme1l1	0,001981	0,156547	0,62	0,57	1
Gstm4	0,001985	-0,05768	0,066	0,122	1
Nat10	0,001994	-0,04215	0,094	0,16	1
Csnk2a2	0,001994	-0,06774	0,38	0,511	1
Nrg2	0,001999	-0,04943	0,051	0,103	1
Lig4	0,002001	-0,05136	0,086	0,15	1
Chic2	0,002003	-0,04305	0,042	0,091	1
Ttc33	0,002014	-0,04085	0,356	0,481	1
Atp6v0a2	0,00202	-0,06292	0,437	0,57	1
Zwint	0,002031	-0,05374	0,349	0,487	1
Rpl38	0,00204	-0,03331	0,996	0,998	1
2310033P(	0,002042	-0,06826	0,266	0,369	1
Zfp260	0,00205	-0,03575	0,343	0,479	1
Zkscan7	0,002056	-0,0478	0,073	0,133	1
Nme6	0,002064	-0,05333	0,081	0,141	1
Xpa	0,002067	-0,04902	0,31	0,426	1
Pnn	0,002072	-0,08324	0,811	0,873	1
Ptp4a1	0,002076	-0,08068	0,998	0,992	1
Ptges2	0,00208	-0,04807	0,226	0,321	1
Crls1	0,002081	-0,07116	0,273	0,371	1
Tmem108	0,002081	-0,06675	0,294	0,397	1
Acat2	0,002082	-0,07216	0,341	0,454	1
Serpine2	0,002085	-0,02632	0,009	0,038	1
Zfyve9	0,002085	-0,04773	0,35	0,468	1
9330198N:	0,002093	-0,08307	0,239	0,329	1
Golga5	0,002098	-0,05477	0,217	0,31	1
Rpl13	0,0021	0,112809	1	0,996	1
Fip1l1	0,002107	-0,08452	0,606	0,707	1
Ptbp3	0,002114	0,177091	0,472	0,43	1
Tns3	0,002116	0,146986	0,615	0,57	1
Slc31a2	0,002119	-0,07032	0,332	0,445	1
Aen	0,002121	-0,05056	0,128	0,203	1
Actr8	0,002128	-0,05127	0,292	0,399	1
Relt	0,002136	-0,03818	0,026	0,065	1
Psmc1	0,002144	-0,06691	0,749	0,821	1
Serac1	0,002147	-0,03617	0,037	0,082	1
Tfg	0,00215	-0,08922	0,552	0,643	1
Reep4	0,002154	-0,04325	0,062	0,118	1
Tmem259	0,002155	-0,06702	0,541	0,66	1
Rbbp5	0,002157	-0,04413	0,213	0,306	1
Coq6	0,00216	-0,05566	0,143	0,222	1
2810414N(	0,002162	-0,03301	0,017	0,051	1

Slc30a6	0,002163	-0,06884	0,114	0,181	1
Bloc1s6	0,002167	-0,04971	0,088	0,152	1
Fam53c	0,002173	-0,0444	0,273	0,382	1
Mpv17l	0,002181	-0,07029	0,228	0,319	1
Ergic1	0,002181	-0,05762	0,101	0,167	1
Ube2d3	0,002186	0,145845	0,961	0,966	1
Ext1	0,00219	-0,04364	0,117	0,192	1
Gba	0,002198	-0,05525	0,292	0,401	1
Gm14399	0,002201	-0,03322	0,017	0,051	1
Zrsr2	0,002202	-0,05775	0,334	0,443	1
Cdc42ep4	0,002206	-0,05319	0,057	0,11	1
Ing2	0,00221	-0,04287	0,209	0,302	1
Rabif	0,002213	-0,06524	0,187	0,27	1
Megf8	0,002223	0,107712	0,255	0,179	1
Ap1s2	0,002225	-0,04792	0,116	0,186	1
Gpr176	0,002236	-0,0308	0,013	0,044	1
Cspg5	0,002241	-0,05512	0,084	0,148	1
N4bp2l1	0,002242	-0,04507	0,095	0,162	1
Vps39	0,002249	-0,05906	0,213	0,306	1
Gpalpp1	0,002252	-0,05755	0,183	0,268	1
Hnrnpa3	0,002252	0,122646	0,978	0,964	1
Spred1	0,002255	-0,08708	0,71	0,793	1
Gm17655	0,00226	-0,03697	0,026	0,065	1
Lgi4	0,002261	0,179469	0,272	0,203	1
Prpf4	0,002262	-0,04938	0,244	0,344	1
Golph3l	0,002272	-0,05339	0,172	0,255	1
Alas1	0,002275	-0,05445	0,185	0,274	1
1810010H	0,002276	-0,031	0,013	0,044	1
Sox10	0,002285	0,107253	1	0,994	1
Pef1	0,002293	-0,07248	0,297	0,395	1
Cpm	0,002293	-0,07689	0,672	0,774	1
H13	0,002297	-0,06139	0,382	0,492	1
Mtcp1	0,002298	-0,0456	0,081	0,141	1
Myo6	0,002305	-0,0854	0,983	0,987	1
Zfp518a	0,002307	-0,04454	0,239	0,333	1
2700016F	0,002309	-0,05376	0,095	0,16	1
Ccdc62	0,002311	-0,04359	0,064	0,12	1
Vps37b	0,002311	0,155376	0,209	0,146	1
Gsk3b	0,00232	0,124764	0,921	0,897	1
Zfp94	0,002321	-0,06009	0,086	0,148	1
Sh3d19	0,002324	0,151247	0,49	0,443	1
Fxyd7	0,002326	-0,28021	0,088	0,148	1
Chp1	0,002329	-0,04536	0,338	0,458	1
Homez	0,002335	-0,0467	0,048	0,097	1
Sort1	0,002336	0,109838	0,989	0,985	1
Ift88	0,00234	-0,05845	0,103	0,169	1
Gm32250	0,002345	-0,01674	0	0,017	1
Chgb	0,002345	-0,01674	0	0,017	1
Nkain4	0,002345	-0,01674	0	0,017	1
Sncb	0,002345	-0,01674	0	0,017	1
Gm29502	0,002345	-0,01674	0	0,017	1
Prkcz	0,002348	-0,0818	0,875	0,903	1

Mak16	0,002349	-0,04152	0,237	0,338	1
Slc3a2	0,00235	0,139574	0,822	0,764	1
Gng12	0,002356	-0,08879	0,789	0,852	1
Pard3b	0,002357	0,035363	0,029	0,004	1
Ift140	0,002368	-0,05391	0,097	0,162	1
Nadk	0,00237	-0,064	0,314	0,426	1
Lyplal1	0,002372	-0,04303	0,033	0,076	1
Tatdn3	0,002378	-0,05564	0,116	0,186	1
Nck1	0,002391	-0,05039	0,169	0,253	1
Rhbdd3	0,002395	-0,06035	0,11	0,177	1
Dr1	0,0024	-0,04183	0,207	0,3	1
Kat6a	0,002401	0,152282	0,672	0,656	1
Aph1b	0,002403	-0,05485	0,178	0,264	1
Acd	0,002408	-0,05739	0,123	0,194	1
Vps37a	0,002408	-0,05728	0,406	0,536	1
Ttc7b	0,002413	-0,07014	0,147	0,222	1
Gm9856	0,002414	-0,03422	0,044	0,093	1
Mrpl49	0,002416	-0,07494	0,22	0,304	1
Farsa	0,002418	-0,05306	0,244	0,338	1
Sned1	0,00242	0,144657	0,323	0,243	1
Igf1r	0,002427	-0,06798	0,512	0,635	1
Ccdc73	0,002439	-0,0415	0,048	0,097	1
Letm1	0,002439	-0,04404	0,371	0,496	1
Bag6	0,002448	0,126442	0,679	0,633	1
Tk2	0,002456	-0,05511	0,134	0,207	1
Eif3m	0,002457	-0,0633	0,683	0,776	1
Arl16	0,002459	-0,05667	0,145	0,222	1
Wls	0,002459	0,136398	0,8	0,757	1
Zfp422	0,002466	-0,04103	0,136	0,209	1
Selenoo	0,002469	-0,0428	0,264	0,371	1
Slc1a5	0,002476	0,054699	0,059	0,021	1
Tbl3	0,00248	-0,05764	0,167	0,249	1
Gigyf1	0,002483	0,173991	0,585	0,568	1
Gm42756	0,002497	-0,10491	0,321	0,418	1
2310057M	0,0025	-0,05339	0,163	0,243	1
Usp54	0,002504	0,115885	0,861	0,838	1
Rae1	0,002509	-0,07743	0,244	0,329	1
Mxra7	0,002524	0,074291	0,141	0,082	1
Slc35a1	0,00253	-0,0644	0,165	0,243	1
Pacsin2	0,002533	-0,05515	0,206	0,295	1
Prepl	0,002536	-0,0662	0,139	0,213	1
9630009A(	0,00254	-0,04172	0,066	0,122	1
Rrm2b	0,002545	-0,04724	0,413	0,54	1
9530077C(	0,00255	-0,03834	0,066	0,122	1
Memo1	0,002559	-0,0573	0,128	0,2	1
Psmg2	0,002564	-0,05308	0,224	0,316	1
Flad1	0,002577	-0,04093	0,163	0,247	1
Sspn	0,002577	-0,0425	0,411	0,555	1
Fam98c	0,002578	-0,04932	0,172	0,255	1
Pex1	0,002582	-0,06454	0,253	0,35	1
Scap	0,002585	-0,06186	0,4	0,532	1
Plxna2	0,002591	0,090888	0,13	0,074	1

Trip12	0,002591	0,136298	0,633	0,593	1
Dlst	0,002592	-0,04691	0,464	0,601	1
Ggta1	0,002597	-0,03921	0,018	0,053	1
Ncor1	0,002613	0,108494	0,919	0,897	1
Mfap1b	0,00262	-0,06894	0,47	0,593	1
Bud23	0,002625	-0,0514	0,224	0,316	1
Gpat4	0,002627	-0,0358	0,406	0,549	1
Dnaic2	0,002638	-0,04533	0,035	0,078	1
Clasp2	0,002641	0,068628	0,998	0,983	1
Arl6ip6	0,002644	-0,037	0,172	0,259	1
Cptp	0,002648	-0,04443	0,092	0,156	1
Eif2a	0,002649	-0,04963	0,422	0,561	1
Sergef	0,00265	-0,06035	0,117	0,186	1
Tmem132a	0,002652	-0,05707	0,143	0,217	1
Cdc27	0,002656	-0,06712	0,217	0,304	1
Txlng	0,00266	-0,05854	0,171	0,253	1
Spidr	0,002664	-0,06734	0,11	0,175	1
Tti1	0,00267	-0,04806	0,086	0,148	1
Tcf12	0,002672	0,126385	0,895	0,854	1
Phpt1	0,002673	-0,07046	0,55	0,677	1
Ruvbl2	0,002675	-0,04685	0,25	0,35	1
Abhd14b	0,002676	-0,04888	0,182	0,266	1
Mettl21a	0,002678	-0,0509	0,086	0,148	1
Eipr1	0,002686	-0,05671	0,202	0,289	1
Lpcat4	0,002688	-0,03756	0,075	0,135	1
Riox2	0,00269	-0,05134	0,077	0,135	1
Supt6	0,002696	0,134254	0,62	0,601	1
Eno2	0,002697	0,097454	0,218	0,146	1
Zfp995	0,0027	-0,03342	0,018	0,053	1
Adgrl1	0,0027	-0,05302	0,083	0,143	1
Zfp629	0,002703	-0,04293	0,07	0,127	1
Srf	0,00272	0,113952	0,218	0,152	1
Mkl2	0,002733	-0,0615	0,182	0,266	1
Pigv	0,002735	-0,05374	0,117	0,186	1
Stard6	0,002736	-0,03883	0,015	0,046	1
Gm42726	0,002738	0,098058	0,211	0,143	1
Raly	0,002747	0,101531	0,89	0,863	1
Sec14l1	0,002747	0,165058	0,532	0,487	1
P3h4	0,002748	-0,09882	0,615	0,717	1
Bbs7	0,002749	-0,04894	0,053	0,103	1
Dhx8	0,002751	-0,04634	0,172	0,255	1
Cradd	0,002761	-0,04686	0,158	0,238	1
Ubqln1	0,002763	0,122983	0,793	0,741	1
Slc12a2	0,002765	-0,05919	0,998	0,998	1
Nfasc	0,002777	0,097165	0,985	0,989	1
Wdr5b	0,002782	-0,03288	0,05	0,099	1
Cyp4f17	0,002784	-0,02835	0,011	0,04	1
6430548M	0,002789	-0,06387	0,171	0,251	1
Ptgds	0,00279	-0,26824	0,941	0,835	1
Dido1	0,002793	-0,04848	0,283	0,39	1
Taco1os	0,002794	-0,0535	0,055	0,105	1
Tor1aip2	0,002798	-0,04958	0,459	0,593	1

Jmjd7	0,0028	-0,05134	0,077	0,135	1
Maz	0,002801	0,111252	0,639	0,603	1
Flrt1	0,002801	0,126442	0,15	0,093	1
Ei24	0,002816	-0,07439	0,633	0,728	1
Rab5a	0,002834	-0,07769	0,818	0,869	1
Spata24	0,002842	-0,05979	0,158	0,236	1
Snx9	0,002848	-0,06126	0,316	0,424	1
G6pc3	0,00285	-0,04823	0,372	0,494	1
Tsen34	0,002853	-0,07696	0,534	0,643	1
Gm32856	0,002862	-0,03142	0,015	0,046	1
Med22	0,00287	-0,06668	0,229	0,316	1
Pmp22	0,002872	0,148542	0,989	0,954	1
Slc25a1	0,002877	-0,04055	0,442	0,57	1
Extl2	0,002879	-0,04813	0,317	0,43	1
Man1a	0,00288	0,112574	0,139	0,082	1
Wasf1	0,002881	-0,05931	0,272	0,369	1
Rtl5	0,002881	-0,02384	0,006	0,03	1
Socs1	0,002887	0,048653	0,037	0,008	1
Kdelr1	0,002907	-0,06617	0,565	0,709	1
0610030E2	0,002907	-0,05536	0,288	0,388	1
Tradd	0,002908	-0,05076	0,226	0,321	1
Tmem104	0,002918	-0,05912	0,106	0,171	1
Gm16133	0,002918	0,03995	0,037	0,008	1
Aktip	0,002926	-0,08706	0,71	0,793	1
Slc25a14	0,002939	-0,05863	0,15	0,226	1
Lemd2	0,002939	-0,05116	0,361	0,47	1
Scarb1	0,002944	-0,06237	0,145	0,219	1
Cep57l1	0,002947	-0,06673	0,172	0,251	1
Wnt4	0,002948	0,025044	0,024	0,002	1
Med27	0,00296	-0,04716	0,165	0,247	1
Mars2	0,002962	-0,05154	0,112	0,179	1
Rps6ka5	0,002963	-0,08696	0,543	0,66	1
A430005L1	0,002965	-0,05282	0,255	0,348	1
Ddb1	0,002965	0,129377	0,728	0,703	1
Selenon	0,002965	-0,05536	0,303	0,403	1
Dym	0,002971	-0,06174	0,194	0,278	1
Dnajb14	0,002974	-0,07883	0,739	0,829	1
Cdc42	0,002974	-0,06594	0,993	0,992	1
R3hcc1	0,002975	-0,08041	0,255	0,342	1
Med4	0,002976	-0,03595	0,35	0,475	1
Zscan26	0,002978	-0,07127	0,347	0,449	1
6030458C1	0,002978	-0,04735	0,143	0,217	1
Rpl36a	0,002979	-0,05807	0,95	0,958	1
Naa60	0,003003	-0,04625	0,228	0,319	1
Gm43567	0,003007	-0,02317	0,002	0,021	1
Gja1	0,003018	-0,01904	0,002	0,021	1
Gm29650	0,003018	-0,01904	0,002	0,021	1
Pkm	0,003025	0,099534	0,901	0,871	1
Jade1	0,003027	0,168248	0,541	0,496	1
Adipor1	0,003027	-0,06812	0,778	0,833	1
Pigh	0,003028	-0,04125	0,121	0,192	1
Lipt2	0,003029	-0,05226	0,084	0,143	1

Mal	0,003032	-0,07244	1	0,996	1
Dnajc8	0,003033	-0,07447	0,745	0,833	1
Mthfd2	0,003052	-0,05488	0,308	0,414	1
Fut8	0,003054	-0,07235	0,558	0,69	1
Flrt3	0,003058	-0,04861	0,046	0,093	1
Hbq1b	0,00306	-0,02954	0,002	0,021	1
Wdr66	0,003061	0,023573	0,018	0	1
C1qtnf4	0,003067	-0,02749	0,004	0,025	1
Nsun3	0,003068	-0,03045	0,024	0,061	1
Ntan1	0,003069	-0,0516	0,572	0,707	1
Mpdu1	0,003073	-0,08236	0,464	0,565	1
Kcnj2	0,003074	-0,02898	0,138	0,215	1
Prkrip1	0,003076	-0,06558	0,239	0,329	1
Ndst4	0,003077	-0,02339	0,004	0,025	1
Slc46a1	0,003077	-0,01721	0,002	0,021	1
Pou3f3	0,003085	0,133081	0,848	0,819	1
Psen1	0,003092	-0,07391	0,461	0,574	1
Dscam	0,003096	0,034025	0,044	0,013	1
Prkcq	0,003101	-0,08614	0,38	0,485	1
Sgk1	0,003104	-0,12247	0,49	0,584	1
Kif21b	0,003105	-0,06427	0,163	0,243	1
Akap7	0,003106	-0,04658	0,277	0,38	1
Dolpp1	0,003121	-0,04891	0,117	0,186	1
Tlnrd1	0,003124	-0,04495	0,066	0,12	1
Bbc3	0,003157	0,081687	0,156	0,095	1
Bcl10	0,003161	-0,07171	0,363	0,456	1
Rnf121	0,003169	-0,05769	0,248	0,344	1
Rexo4	0,003171	-0,0625	0,215	0,3	1
Mtmr10	0,003177	0,135315	0,644	0,61	1
Pnrc1	0,003178	0,145847	0,771	0,724	1
Slc22a15	0,003179	-0,06129	0,161	0,238	1
Zmynd19	0,003186	-0,04069	0,105	0,171	1
Cops7a	0,003201	-0,05189	0,325	0,443	1
Fam120b	0,003207	-0,05144	0,301	0,405	1
Tpd52l2	0,003211	-0,05807	0,602	0,719	1
Rhoj	0,003214	0,14671	0,332	0,251	1
Cep44	0,003216	-0,04099	0,196	0,285	1
Ppp1r15b	0,003226	0,153449	0,517	0,475	1
Gm14296	0,003231	-0,03829	0,138	0,213	1
Mrps22	0,003232	-0,05691	0,183	0,266	1
Tcea2	0,003237	-0,04562	0,068	0,122	1
Lingo2	0,003242	-0,03601	0,026	0,063	1
Thap7	0,003243	-0,05498	0,2	0,285	1
Usp7	0,003252	0,171576	0,523	0,489	1
Zfp563	0,003252	-0,0395	0,061	0,114	1
Zfp773	0,003256	-0,05391	0,081	0,139	1
Ormdl3	0,00326	-0,04017	0,292	0,401	1
Rgl1	0,003263	0,075035	0,139	0,082	1
Lrfn4	0,003264	0,0749	0,123	0,07	1
BC029722	0,003278	-0,074	0,38	0,487	1
Brd7	0,003279	-0,06928	0,615	0,711	1
Erlin2	0,003281	-0,06547	0,323	0,424	1

Pcdhga10	0,003294	-0,0362	0,026	0,063	1
Rbm10	0,003296	-0,05614	0,295	0,397	1
Efcab2	0,003313	-0,07805	0,11	0,173	1
Uqcc1	0,003319	-0,06082	0,161	0,236	1
Mapk1ip1	0,003329	-0,05076	0,211	0,295	1
9330020H(	0,00333	-0,03183	0,02	0,055	1
Tomm34	0,003331	-0,04882	0,383	0,502	1
Pgap1	0,003334	-0,05543	0,347	0,466	1
Cyb561d1	0,003335	-0,03651	0,053	0,103	1
1110051M	0,003338	-0,0637	0,264	0,357	1
B230217C:	0,003349	-0,05876	0,147	0,219	1
Naa10	0,003365	-0,04035	0,266	0,365	1
Tiparp	0,003368	0,260866	0,272	0,213	1
Thap11	0,003372	-0,0527	0,229	0,321	1
Tmem206	0,003378	-0,04009	0,112	0,179	1
Cyp20a1	0,003387	-0,0657	0,506	0,633	1
Cep95	0,003399	-0,0344	0,097	0,162	1
Zbtb26	0,003401	-0,05121	0,081	0,139	1
Ap4b1	0,003409	-0,05048	0,099	0,162	1
Gnpnat1	0,003417	-0,06433	0,204	0,285	1
Dtwd2	0,003427	-0,04288	0,048	0,095	1
Skap2	0,003438	-0,04645	0,576	0,694	1
Gm29394	0,003439	-0,02114	0,029	0,07	1
Bin3	0,00344	-0,05627	0,138	0,211	1
Ptprn	0,003441	-0,03378	0,231	0,323	1
Rcc1l	0,003447	-0,05257	0,121	0,19	1
1110002L(	0,00345	-0,04981	0,075	0,131	1
Klhl26	0,003453	-0,05715	0,075	0,131	1
Atxn3	0,00346	-0,05851	0,259	0,35	1
Zkscan5	0,003482	-0,04891	0,116	0,184	1
Nthl1	0,003482	-0,04515	0,178	0,262	1
Trmt112	0,003482	-0,06033	0,618	0,738	1
D16Ertd47	0,003492	-0,06494	0,82	0,88	1
Ntsr2	0,003511	-0,02835	0,009	0,036	1
Hoxd1	0,00352	-0,05766	0,116	0,181	1
Sbf2	0,003523	0,188588	0,543	0,519	1
Dpp3	0,003523	-0,06102	0,261	0,352	1
Egln2	0,003527	-0,04601	0,411	0,538	1
Gmds	0,003536	-0,05873	0,119	0,186	1
Wdr70	0,003537	-0,05769	0,341	0,445	1
1700096K:	0,003538	-0,04868	0,154	0,23	1
Metap1	0,003555	-0,05415	0,279	0,371	1
Ebag9	0,003555	-0,05721	0,196	0,278	1
2810455O(	0,003558	-0,02429	0,009	0,036	1
Ifnar2	0,003565	-0,06771	0,328	0,426	1
Fuca1	0,003578	-0,07921	0,741	0,829	1
Trp53cor1	0,003583	-0,03261	0,009	0,036	1
Mettl18	0,003588	-0,0292	0,017	0,049	1
Gm14410	0,003588	-0,0292	0,017	0,049	1
Stk39	0,003597	-0,07749	0,73	0,81	1
Thap1	0,003598	-0,06162	0,176	0,255	1
B230303O:	0,003601	-0,03342	0,017	0,049	1

Gm49042	0,003608	-0,03059	0,013	0,042	1
Copg1	0,003612	-0,06257	0,325	0,437	1
Riok3	0,003616	-0,04997	0,481	0,599	1
Atp5g3	0,003619	-0,06999	0,806	0,863	1
Mvp	0,003625	0,1792	0,521	0,492	1
Nol8	0,003626	-0,04491	0,275	0,373	1
Atp1b2	0,003628	-0,04901	0,057	0,108	1
Pex3	0,003637	-0,06025	0,334	0,443	1
Spef2	0,003645	-0,07153	0,079	0,135	1
Fam198b	0,003646	-0,04194	0,022	0,057	1
Mocs2	0,003651	-0,04479	0,464	0,584	1
Nup93	0,00366	-0,05149	0,073	0,129	1
Skiv2l2	0,003664	-0,04805	0,418	0,549	1
Ran	0,003664	-0,0694	0,857	0,903	1
Tmem256	0,003672	-0,05646	0,945	0,951	1
Ttc9c	0,003684	-0,03696	0,229	0,321	1
Dopey2	0,003692	-0,06828	0,429	0,553	1
Triobp	0,0037	-0,0534	0,167	0,245	1
Ltbp1	0,003701	0,04064	0,028	0,004	1
BC024139	0,003704	-0,04479	0,051	0,099	1
Uso1	0,003709	-0,04924	0,332	0,449	1
Cdc40	0,00371	-0,0378	0,378	0,508	1
Slc25a24	0,003712	0,037125	0,028	0,004	1
Ddit3	0,003721	0,2346	0,684	0,667	1
Kmt2d	0,003725	0,166023	0,453	0,405	1
Lcmt1	0,003729	-0,05466	0,341	0,449	1
Shisa5	0,003736	-0,05748	0,099	0,16	1
Katnbl1	0,003741	-0,04932	0,172	0,253	1
Ngdn	0,003742	-0,04253	0,383	0,502	1
Platr25	0,003748	-0,05584	0,097	0,158	1
Mtif3	0,003748	-0,0527	0,165	0,241	1
Rpl7	0,003753	-0,07644	0,989	0,989	1
Ecd	0,003758	-0,0452	0,251	0,352	1
Dtx3	0,003781	-0,0281	0,418	0,542	1
Maged2	0,003781	-0,05498	0,231	0,319	1
C1ql2	0,003793	0,040294	0,028	0,004	1
Zfp931	0,003793	-0,0344	0,079	0,137	1
Itgav	0,003799	-0,093	0,76	0,812	1
Ago4	0,003799	-0,04754	0,051	0,099	1
D130020L(	0,003801	-0,03959	0,05	0,097	1
Slc25a16	0,003813	-0,04502	0,152	0,228	1
Pnpla2	0,00382	-0,05414	0,312	0,411	1
Med6	0,003859	-0,0562	0,176	0,255	1
Zfp850	0,003861	-0,02539	0,013	0,042	1
Tagap1	0,003869	-0,04813	0,079	0,135	1
Slco3a1	0,003872	-0,10693	0,969	0,958	1
Wfikkn2	0,003872	-0,03402	0,022	0,057	1
Clk3	0,003878	-0,0562	0,264	0,361	1
Cntn1	0,003887	-0,09321	0,062	0,114	1
Srgap2	0,003888	-0,05842	0,253	0,346	1
Hypk	0,003892	-0,07562	0,523	0,646	1
BC052040	0,003902	-0,05333	0,084	0,141	1

Slc25a17	0,003918	-0,03645	0,244	0,34	1
Zfp119b	0,003919	-0,03203	0,022	0,057	1
6230400D1	0,003925	-0,04144	0,035	0,076	1
Ccni	0,003936	0,139618	0,699	0,656	1
Vamp2	0,003942	-0,06272	0,829	0,895	1
Zfp668	0,003951	-0,04342	0,061	0,112	1
Kdm6a	0,003957	0,151797	0,604	0,574	1
Mrrf	0,003957	-0,05914	0,139	0,209	1
Sh3glb2	0,003958	-0,05525	0,505	0,641	1
Elp2	0,003967	-0,06143	0,231	0,316	1
Agpat5	0,003978	-0,0591	0,45	0,565	1
Lrig3	0,003981	-0,03111	0,286	0,397	1
Zdhhc20	0,00399	-0,09036	0,969	0,964	1
Riok2	0,004012	-0,0395	0,161	0,241	1
Ttll1	0,004026	-0,05196	0,084	0,141	1
Klf15	0,004033	-0,05388	0,198	0,281	1
Wwp1	0,004046	-0,06751	0,538	0,648	1
Tfdp2	0,004059	-0,06989	0,547	0,658	1
Tbpl1	0,004061	-0,06547	0,194	0,274	1
Rap2c	0,004062	-0,0639	0,2	0,281	1
Bckdk	0,004064	-0,05454	0,262	0,354	1
Kmt2c	0,004073	0,138647	0,787	0,736	1
Eif4a1	0,004078	0,104396	0,993	0,983	1
Retreg2	0,004081	0,117873	0,758	0,7	1
Fam210a	0,004092	-0,06193	0,2	0,283	1
Afap1l2	0,004103	0,052017	0,05	0,017	1
Pnkp	0,004111	-0,04305	0,261	0,354	1
Dsel	0,004135	-0,08481	0,138	0,205	1
Cpsf4	0,00416	-0,04971	0,099	0,16	1
Tipin	0,004172	-0,06422	0,193	0,272	1
A230057D0	0,004179	-0,03908	0,116	0,184	1
Snx5	0,004182	-0,07852	0,613	0,732	1
Sp7	0,004199	-0,05358	0,121	0,188	1
Kmt5c	0,004206	-0,05861	0,13	0,198	1
Nxpe3	0,004226	-0,05904	0,182	0,259	1
Abtb1	0,004234	-0,05852	0,167	0,243	1
Prrc2c	0,004235	0,098239	0,965	0,951	1
Epb41l4a	0,004238	-0,04262	0,088	0,148	1
Prox2	0,004241	-0,03692	0,042	0,086	1
Rcl1	0,004244	-0,05103	0,156	0,23	1
Gm26825	0,004245	0,148586	0,123	0,072	1
Cdadc1	0,004248	-0,05625	0,363	0,464	1
Scrn2	0,004253	-0,04901	0,068	0,12	1
Fbxl2	0,004258	-0,04754	0,057	0,105	1
Adap1	0,004262	0,12232	0,956	0,956	1
Slc52a2	0,004264	-0,06061	0,123	0,188	1
Mgll	0,004269	-0,07173	0,206	0,291	1
Zfp51	0,004278	-0,04619	0,046	0,091	1
Fbxo28	0,004282	-0,04899	0,49	0,616	1
Tdg	0,004287	-0,05282	0,222	0,31	1
Pdss2	0,00429	-0,05197	0,101	0,162	1
Osgepl1	0,004304	-0,04555	0,112	0,177	1

Dpf2	0,004311	-0,03422	0,4	0,525	1
Arf6	0,004314	-0,03092	0,213	0,304	1
Gtf2h3	0,004315	-0,04267	0,117	0,184	1
Ppp2r2a	0,004324	0,109876	0,974	0,956	1
Nmi	0,004339	-0,04476	0,092	0,152	1
Actr2	0,004342	0,120056	0,767	0,743	1
Odf2	0,00435	-0,03343	0,332	0,445	1
Thsd1	0,00435	-0,05303	0,07	0,122	1
Tsen2	0,004369	-0,04682	0,178	0,257	1
Phyhipl	0,00437	0,118764	0,978	0,956	1
S100b	0,004375	0,094903	0,905	0,791	1
Pars2	0,004379	-0,03167	0,029	0,068	1
Dnm3os	0,004392	-0,02856	0,035	0,076	1
Mrpl52	0,004395	-0,08771	0,6	0,679	1
Tpcn1	0,0044	-0,06568	0,231	0,319	1
Ccar1	0,004401	0,150765	0,615	0,586	1
Hba-a1	0,004406	-2,45088	0,024	0,059	1
Igf2bp2	0,004412	0,057281	0,035	0,008	1
2610021A(	0,004413	-0,069	0,128	0,194	1
Kctd14	0,004419	0,04135	0,031	0,006	1
Bms1	0,004433	-0,06105	0,228	0,31	1
Slc38a1	0,004445	-0,03902	0,015	0,044	1
Gm7173	0,004446	-0,01466	0	0,015	1
Stmn2	0,004446	-0,01466	0	0,015	1
Tac1	0,004446	-0,01466	0	0,015	1
Gm39318	0,004446	-0,01466	0	0,015	1
Gm17399	0,004446	-0,01466	0	0,015	1
Ppp1r3c	0,004446	-0,01466	0	0,015	1
Nrgn	0,004446	-0,01881	0	0,015	1
Mlc1	0,004446	-0,01881	0	0,015	1
Ccp110	0,004454	-0,13958	0,862	0,835	1
Tmem115	0,004461	-0,06272	0,338	0,439	1
Cbx4	0,004464	0,13747	0,686	0,662	1
Ube2e1	0,004465	-0,03987	0,483	0,612	1
Ssh1	0,004466	0,100004	0,189	0,127	1
Thsd4	0,00447	-0,03384	0,062	0,114	1
Manba	0,004472	0,106586	0,257	0,19	1
2510046G1	0,00448	-0,03422	0,024	0,059	1
Snupn	0,004495	-0,05476	0,171	0,247	1
Aaas	0,004498	-0,04509	0,099	0,16	1
Rps29	0,004499	-0,04292	1	1	1
Ss18	0,004499	-0,0488	0,461	0,593	1
Snu13	0,004501	-0,0675	0,916	0,93	1
Pacrg	0,004525	-0,02783	0,018	0,051	1
Tmbim1	0,004528	-0,06876	0,983	0,996	1
Rps17	0,004536	-0,06115	0,987	0,983	1
Zfp866	0,004539	-0,06904	0,145	0,215	1
Nup43	0,004551	-0,04754	0,059	0,108	1
Ppp1r1b	0,004583	-0,02878	0,015	0,044	1
Brf2	0,004588	-0,05683	0,114	0,177	1
Sumo3	0,004595	-0,04847	0,479	0,614	1
Mafa	0,004601	0,035774	0,042	0,013	1

Sco1	0,004608	-0,03589	0,081	0,137	1
Tbc1d20	0,004615	-0,07307	0,268	0,357	1
Cep250	0,004617	-0,04427	0,163	0,241	1
Syf2	0,004619	-0,06652	0,908	0,928	1
App	0,004629	-0,05714	1	0,998	1
Mlx	0,00463	-0,05931	0,261	0,348	1
Ikzf2	0,004631	-0,06006	0,301	0,399	1
Rftn2	0,004634	-0,05772	0,319	0,426	1
Zfp869	0,004637	-0,04462	0,048	0,093	1
Ankmy1	0,004638	0,060548	0,097	0,051	1
Rundc3a	0,004649	-0,05624	0,409	0,519	1
Tcof1	0,00465	-0,03666	0,229	0,321	1
Cc2d1b	0,004655	-0,04204	0,316	0,424	1
Ncs1	0,004664	-0,07238	0,139	0,207	1
Ap3m2	0,004666	-0,05489	0,171	0,247	1
Tbc1d22a	0,004668	-0,06136	0,15	0,219	1
Mgat4b	0,004669	-0,04861	0,283	0,376	1
Gbe1	0,004673	-0,03941	0,081	0,137	1
Mbtps2	0,004685	-0,04495	0,229	0,319	1
Snx19	0,004691	-0,06096	0,215	0,295	1
Cct3	0,004693	-0,071	0,822	0,878	1
Maff	0,004694	0,031943	0,035	0,008	1
Tpra1	0,004699	-0,04298	0,112	0,177	1
Ilf2	0,004702	-0,05991	0,675	0,778	1
Lamp2	0,004707	0,103987	0,996	0,977	1
Tmem268	0,004711	-0,03198	0,264	0,361	1
Mzf1	0,004713	-0,02676	0,011	0,038	1
Cnot8	0,004731	-0,04981	0,301	0,401	1
Rabep2	0,004733	-0,04762	0,121	0,188	1
Dusp5	0,00474	0,026828	0,022	0,002	1
Ikbkap	0,004758	-0,06053	0,172	0,249	1
Triap1	0,004784	-0,05459	0,189	0,268	1
E2f4	0,004801	-0,05681	0,139	0,209	1
Tceanc2	0,004802	-0,04937	0,194	0,274	1
Dmap1	0,004808	-0,04608	0,242	0,329	1
Tspan15	0,004821	-0,05477	0,958	0,956	1
Brix1	0,004829	-0,04325	0,301	0,403	1
Magee1	0,004838	-0,04708	0,259	0,352	1
Pofut1	0,004842	-0,04803	0,167	0,243	1
Zfp748	0,004851	-0,04442	0,149	0,222	1
Ergic3	0,004857	-0,08083	0,692	0,757	1
Actn1	0,004877	-0,03478	0,068	0,12	1
Gm26935	0,004907	-0,03818	0,026	0,061	1
Ptcd3	0,004908	-0,04241	0,207	0,293	1
L3mbtl2	0,004913	-0,07015	0,176	0,247	1
Susd6	0,004917	-0,03943	0,299	0,407	1
Tmem218	0,004924	-0,03537	0,048	0,093	1
Tbcel	0,004926	-0,06843	0,341	0,439	1
Tinf2	0,004935	-0,05967	0,103	0,162	1
Usp14	0,004938	-0,05564	0,361	0,468	1
Ift80	0,004965	-0,04541	0,2	0,283	1
Katnal1	0,004966	0,148104	0,589	0,549	1

Ccdc187	0,004968	-0,05257	0,11	0,173	1
Zfp933	0,004973	-0,05212	0,099	0,158	1
Asb11	0,004983	0,021779	0,017	0	1
Zfp949	0,004994	-0,065	0,167	0,238	1
Pak1	0,004999	0,116701	0,989	0,962	1
Atrn	0,005007	0,126934	0,802	0,772	1
Idh3g	0,005016	-0,07706	0,521	0,629	1
Luzp1	0,005032	-0,05039	0,061	0,11	1
Ube2i	0,005057	-0,07311	0,576	0,694	1
Mthfd1l	0,00506	-0,04102	0,061	0,11	1
Calm3	0,005065	-0,06896	0,967	0,97	1
Usp8	0,005069	-0,06585	0,497	0,61	1
Hdac8	0,005072	-0,04762	0,125	0,192	1
Mfge8	0,005072	0,138017	0,47	0,405	1
Syap1	0,005092	-0,05001	0,305	0,407	1
Klhl7	0,005092	-0,04954	0,163	0,238	1
Bap1	0,005094	-0,05271	0,158	0,23	1
Otulin	0,005094	-0,07549	0,275	0,359	1
Uvrag	0,005103	-0,05308	0,218	0,302	1
Mars	0,005125	-0,04346	0,264	0,359	1
Lin52	0,005142	-0,05747	0,154	0,226	1
Zfp251	0,005143	-0,05566	0,147	0,215	1
Fkrp	0,005152	-0,05678	0,169	0,243	1
Rpap1	0,005153	-0,02807	0,117	0,181	1
Gemin6	0,005153	-0,04132	0,051	0,097	1
Pogz	0,005156	0,112636	0,305	0,238	1
Zfp354a	0,005162	-0,04278	0,094	0,152	1
2310011J0	0,005168	-0,06754	0,499	0,61	1
Dazap2	0,005171	0,12126	0,927	0,878	1
Dynlt1b	0,005186	-0,02202	0,006	0,027	1
Rbm25	0,005188	0,0894	0,956	0,956	1
Bcas1os1	0,00519	0,047884	0,055	0,021	1
Tenm2	0,005207	-0,04295	0,086	0,143	1
Rhno1	0,005212	-0,05145	0,152	0,224	1
Ttc14	0,005213	-0,05348	0,695	0,831	1
Arpc5	0,005215	0,10435	0,936	0,903	1
Acsbg1	0,005225	-0,01611	0,006	0,027	1
Bcas3	0,005229	-0,06479	0,314	0,403	1
Gm4673	0,005235	-0,04839	0,15	0,222	1
Unc119	0,005255	-0,03264	0,026	0,061	1
Msh6	0,005264	-0,04474	0,123	0,188	1
Mettl17	0,005268	-0,04073	0,075	0,129	1
Wdr74	0,005274	-0,04441	0,196	0,276	1
Nectin3	0,005286	-0,03457	0,108	0,173	1
B3galt6	0,005286	-0,0617	0,112	0,173	1
Grpel2	0,005297	-0,04847	0,237	0,327	1
Gtf2a1	0,00531	0,131712	0,739	0,711	1
Zfp111	0,005317	-0,04276	0,077	0,131	1
Aptx	0,005317	-0,07474	0,191	0,262	1
Polr2e	0,005352	-0,07172	0,6	0,7	1
Commd10	0,005376	-0,05283	0,204	0,285	1
Btbd19	0,005384	-0,03031	0,033	0,072	1

Rnf24	0,0054	-0,07217	0,128	0,192	1
Rbm28	0,005402	-0,04494	0,477	0,61	1
Lias	0,005403	-0,04843	0,308	0,405	1
Ppp2r5d	0,005406	-0,04946	0,185	0,264	1
Dnal1	0,005418	-0,05525	0,138	0,205	1
Pgam5	0,005429	-0,03422	0,136	0,205	1
Atg13	0,005431	-0,05589	0,222	0,306	1
Stambp	0,005436	-0,08649	0,492	0,603	1
Rprd1a	0,00549	-0,02231	0,407	0,538	1
Surf4	0,005493	-0,04403	0,369	0,479	1
Tmem50b	0,005494	-0,04764	0,378	0,498	1
4930452G:	0,005494	-0,02625	0,02	0,053	1
Fam122b	0,005497	-0,04801	0,134	0,203	1
Rab11fip2	0,005501	-0,0914	0,543	0,652	1
Dhx29	0,005511	-0,05809	0,202	0,278	1
Ankrd52	0,005514	-0,06485	0,125	0,188	1
Skil	0,005516	0,188568	0,383	0,327	1
Cntd1	0,005519	-0,0321	0,048	0,093	1
Emc1	0,005523	0,117261	0,743	0,743	1
Sertad2	0,005544	0,151133	0,516	0,477	1
Pomgnt1	0,005572	-0,05884	0,457	0,578	1
Haus5	0,005573	-0,03242	0,101	0,162	1
Prpf8	0,005582	0,146092	0,587	0,565	1
Gm17949	0,005589	-0,02134	0,004	0,023	1
Kcna2	0,005594	0,168001	0,246	0,184	1
Hspa1b	0,005598	0,525008	0,424	0,363	1
Capn9	0,005607	-0,01928	0,004	0,023	1
Tmem38a	0,005611	-0,04736	0,101	0,16	1
Prrc2b	0,005617	0,131587	0,661	0,635	1
Hfe	0,005621	-0,01904	0,002	0,019	1
Gpm6a	0,005621	-0,02111	0,002	0,019	1
Gripap1	0,005623	-0,0458	0,417	0,536	1
Cspp1	0,005629	-0,0401	0,398	0,53	1
Tmeff2	0,00563	-0,04938	0,96	0,979	1
Neu4	0,005631	-0,01698	0,002	0,019	1
Pm20d2	0,005631	-0,01698	0,002	0,019	1
Tmem100	0,005631	-0,01698	0,002	0,019	1
Aard	0,005631	-0,01698	0,002	0,019	1
Taf6	0,005633	-0,03973	0,161	0,236	1
Sdhaf3	0,005637	-0,0408	0,092	0,15	1
H2afz	0,005645	-0,04974	0,82	0,888	1
Map3k4	0,005659	-0,05098	0,325	0,428	1
Sp1	0,005667	0,151565	0,497	0,466	1
AC110241.	0,005674	-0,05345	0,103	0,162	1
A830009L(	0,005675	-0,02157	0,004	0,023	1
Tmem150c	0,005687	-0,06783	0,369	0,468	1
Zfp658	0,005688	-0,03167	0,033	0,072	1
Zcchc3	0,005698	-0,04427	0,149	0,219	1
Adhfe1	0,005699	-0,03304	0,028	0,063	1
Zfp414	0,005699	-0,01685	0,211	0,302	1
Pcp4	0,005708	-0,01928	0,002	0,019	1
Rpgrip1l	0,005711	-0,0564	0,138	0,205	1

Gm26699	0,00576	-0,02571	0,16	0,234	1
Hmg20a	0,005781	-0,06185	0,341	0,435	1
Ptrh2	0,005791	-0,04498	0,206	0,283	1
Capza1	0,005811	-0,03792	0,367	0,481	1
Bag1	0,005822	-0,05094	0,361	0,464	1
Cdc45	0,005827	-0,0322	0,009	0,034	1
Dusp26	0,00584	-0,07556	0,914	0,949	1
Ide	0,005862	-0,07094	0,468	0,565	1
Utp14b	0,005869	0,247121	0,431	0,39	1
Shd	0,005881	-0,02539	0,017	0,046	1
Cenpj	0,005888	-0,05193	0,046	0,089	1
Strap	0,005889	-0,05144	0,628	0,743	1
Trem2	0,005904	-0,02407	0,009	0,034	1
Kcnk5	0,005912	-0,04102	0,064	0,114	1
Rrp8	0,005915	-0,0233	0,147	0,219	1
Sec23b	0,005915	-0,04934	0,376	0,489	1
Arfgap1	0,005929	-0,04911	0,272	0,367	1
Afdn	0,005929	0,1074	0,692	0,671	1
Aatf	0,005949	-0,03736	0,251	0,342	1
Gm45847	0,005973	-0,02876	0,033	0,072	1
Gm17135	0,006047	0,027959	0,026	0,004	1
Prcp	0,006054	-0,05105	0,292	0,386	1
Pycrl	0,006054	-0,05368	0,174	0,249	1
Gm26910	0,006057	-0,03422	0,053	0,099	1
Dcdc2b	0,006058	-0,03024	0,022	0,055	1
Dars	0,006078	-0,06337	0,545	0,662	1
Tarsl2	0,00608	-0,04051	0,277	0,367	1
Tmem263	0,006084	-0,05116	0,222	0,302	1
Tnni1	0,006092	-0,07481	0,606	0,717	1
Rplp0	0,006111	-0,02279	0,996	0,992	1
Bfar	0,006113	-0,0678	0,341	0,439	1
Psmb3	0,006115	-0,06992	0,897	0,945	1
Yod1	0,006118	-0,04236	0,231	0,321	1
Car11	0,006119	-0,0476	0,161	0,232	1
Zmym6	0,006139	-0,05111	0,189	0,268	1
Sec22a	0,006166	-0,05377	0,2	0,278	1
Usp24	0,006198	-0,01466	0,288	0,401	1
Milr1	0,006209	0,061281	0,044	0,015	1
Hoxd8	0,006219	-0,08101	0,648	0,745	1
Gtf3c1	0,00622	-0,06209	0,413	0,525	1
Fam60a	0,006226	-0,03632	0,053	0,099	1
Gm3435	0,006248	-0,03659	0,029	0,065	1
Zdhhc9	0,006278	0,131866	0,699	0,694	1
Mdm2	0,006291	0,170576	0,626	0,605	1
Ttc3	0,006296	0,113477	0,829	0,772	1
Prr3	0,006316	-0,05311	0,182	0,255	1
Ehd4	0,006317	-0,03251	0,13	0,198	1
Xrcc6	0,006336	-0,04759	0,268	0,357	1
Alkbh5	0,006344	0,132186	0,732	0,698	1
Snx6	0,006398	-0,0629	0,752	0,827	1
Gorasp1	0,006398	-0,04831	0,145	0,213	1
Fscn1	0,006399	-0,07335	0,945	0,922	1

Pced1a	0,00641	-0,0395	0,092	0,15	1
Atf7	0,006419	0,134235	0,552	0,53	1
6030407O(	0,006422	-0,05391	0,099	0,156	1
Tldc1	0,006424	-0,03481	0,029	0,065	1
Utp18	0,006446	-0,04034	0,198	0,276	1
Vat1l	0,006447	0,045857	0,044	0,015	1
Eif4h	0,006457	-0,04548	0,985	0,985	1
Ubp1	0,006472	-0,03784	0,275	0,369	1
Ice1	0,006479	-0,05299	0,369	0,481	1
Arpc3	0,006489	-0,0698	0,679	0,783	1
Wdr53	0,006494	-0,04908	0,09	0,146	1
Ccng2	0,006495	-0,0096	0,206	0,295	1
Babam2	0,0065	-0,08202	0,4	0,494	1
Rere	0,006501	0,147553	0,622	0,605	1
Bcdin3d	0,006514	-0,0594	0,139	0,205	1
Snx14	0,006519	-0,0451	0,235	0,323	1
Spata7	0,006523	-0,0924	0,139	0,207	1
Smyd2	0,00654	-0,05419	0,185	0,257	1
Rab11fip4	0,006542	0,12832	0,517	0,475	1
Rbak	0,006555	-0,04217	0,163	0,238	1
Cap1	0,006597	-0,0536	0,539	0,665	1
Pcif1	0,006606	-0,04914	0,477	0,599	1
Ranbp3	0,006611	-0,04965	0,314	0,409	1
Smndc1	0,00662	-0,04011	0,497	0,618	1
Hivep3	0,006642	0,187913	0,424	0,378	1
Armc10	0,006666	-0,05927	0,136	0,2	1
Plcb3	0,006673	0,039265	0,04	0,013	1
B230219D:	0,006676	-0,07071	0,829	0,865	1
Atp8b5	0,006677	-0,05007	0,061	0,108	1
Nt5c2	0,006679	-0,06274	0,194	0,268	1
Dcaf13	0,006688	-0,03238	0,226	0,316	1
Rnf11	0,006691	-0,05992	0,561	0,688	1
Kif3a	0,006722	0,113758	0,892	0,882	1
Fbxl17	0,006724	-0,04968	0,18	0,253	1
Thnsl1	0,006729	-0,0594	0,123	0,184	1
Zrsr1	0,006731	-0,06682	0,345	0,435	1
Fign	0,006736	-0,03268	0,299	0,401	1
Ubr2	0,006768	0,107282	0,796	0,768	1
Lcp1	0,006778	-0,03702	0,068	0,118	1
Kbtbd3	0,006812	-0,056	0,165	0,236	1
Zc3h14	0,006923	-0,03639	0,33	0,437	1
Tfe3	0,006925	-0,03604	0,169	0,245	1
Shtn1	0,006937	-0,06409	0,897	0,937	1
Chchd6	0,006959	-0,04094	0,204	0,283	1
Oat	0,006974	-0,07003	0,49	0,605	1
S1pr5	0,006976	-0,08677	0,853	0,882	1
Fam180a	0,006988	0,032601	0,037	0,011	1
Gm26532	0,006993	0,077881	0,072	0,034	1
Tor1b	0,007004	-0,05235	0,349	0,454	1
Phf7	0,007016	-0,03597	0,039	0,078	1
Ehd1	0,007044	-0,0885	0,628	0,713	1
Topbp1	0,00705	-0,05751	0,158	0,228	1

Ankrd10	0,007064	-0,0324	0,301	0,405	1
Pex12	0,007075	-0,06121	0,088	0,141	1
Prkar2a	0,007082	-0,0494	0,152	0,222	1
Gm14391	0,007092	-0,02794	0,029	0,065	1
Gm26526	0,007099	-0,05398	0,229	0,31	1
Rfc3	0,007111	-0,04059	0,112	0,173	1
Nucb2	0,007128	-0,02668	0,024	0,057	1
Trappc11	0,007133	-0,06344	0,189	0,259	1
Maip1	0,007135	-0,04649	0,193	0,27	1
Ybey	0,007137	-0,05663	0,081	0,133	1
Chmp6	0,007153	-0,04474	0,128	0,192	1
Pex14	0,007159	-0,05954	0,233	0,312	1
Kdm5c	0,007168	0,121358	0,429	0,371	1
Synj1	0,007173	-0,04558	0,4	0,519	1
Appbp2	0,007195	0,116074	0,842	0,808	1
2310022A1	0,007207	-0,05406	0,101	0,158	1
Supt7l	0,007214	-0,04875	0,172	0,247	1
Wbp11	0,007223	-0,06322	0,615	0,717	1
Herpud2	0,007232	-0,04694	0,462	0,584	1
Ndufaf6	0,007249	-0,03286	0,039	0,078	1
Cwc22	0,007252	-0,04721	0,152	0,222	1
Tbc1d10a	0,007252	-0,03308	0,057	0,103	1
Hps3	0,007281	-0,05257	0,167	0,238	1
Ppp1r15a	0,007288	0,220029	0,332	0,283	1
A930028N(	0,007292	-0,04005	0,04	0,08	1
Rfk	0,007294	-0,03014	0,303	0,399	1
Gpr17	0,007297	-0,03402	0,007	0,03	1
Rapgef4	0,007319	-0,02814	0,007	0,03	1
Cnep1r1	0,007326	-0,0543	0,393	0,5	1
Gm15834	0,007338	-0,0218	0,007	0,03	1
Mettl2	0,007391	-0,03573	0,149	0,219	1
Phf14	0,007414	-0,0767	0,523	0,624	1
Calr3	0,007442	-0,02202	0,007	0,03	1
Dedd	0,007452	-0,05491	0,297	0,386	1
Itm2c	0,007463	0,087785	0,963	0,916	1
Tpm4	0,007473	0,103261	0,213	0,152	1
Gm10282	0,007483	-0,01998	0,007	0,03	1
Trmt11	0,007499	-0,04194	0,143	0,211	1
Tfb1m	0,007501	-0,03915	0,09	0,146	1
Sacm1l	0,007501	-0,03881	0,363	0,466	1
Ero1lb	0,007502	-0,03767	0,051	0,095	1
Rars2	0,007506	-0,05669	0,112	0,171	1
Vac14	0,007513	-0,03957	0,167	0,241	1
Chd4	0,007522	0,13074	0,916	0,876	1
Derl3	0,007539	-0,03422	0,026	0,059	1
Eml6	0,007549	-0,04949	0,154	0,222	1
Golga1	0,007553	-0,0218	0,281	0,384	1
Klhl32	0,007556	0,043749	0,081	0,04	1
Zfp358	0,007563	-0,05111	0,209	0,287	1
Rab8a	0,007573	-0,04938	0,336	0,432	1
Nedd4	0,007576	0,098818	0,98	0,949	1
Pum3	0,007582	-0,03838	0,325	0,422	1

Rfc2	0,007594	0,140482	0,622	0,605	1
Tuba1b	0,007595	0,167259	0,831	0,764	1
Psme2	0,00763	0,00139	0,316	0,43	1
Pigw	0,007631	-0,01816	0,007	0,03	1
A930024E(	0,007631	-0,01635	0,007	0,03	1
Patj	0,007632	0,026828	0,02	0,002	1
Gfod2	0,007637	0,028132	0,086	0,141	1
Pard6a	0,00764	-0,0589	0,172	0,243	1
Sgpp1	0,007644	-0,04239	0,301	0,399	1
Camk4	0,007644	0,028609	0,02	0,002	1
Ttll4	0,007654	-0,05954	0,103	0,158	1
Pds5b	0,007659	0,164104	0,483	0,451	1
Cyp4f14	0,00766	-0,0415	0,051	0,095	1
Ftsj1	0,007664	-0,01744	0,248	0,338	1
Tsc22d1	0,007667	0,173133	0,332	0,276	1
Tspyl2	0,007678	-0,02601	0,319	0,432	1
Tbccd1	0,007694	-0,05108	0,182	0,253	1
Map2	0,007694	-0,0366	0,226	0,312	1
Atp13a2	0,007698	-0,04119	0,187	0,264	1
Scyl3	0,007706	-0,02297	0,123	0,188	1
4930419G:	0,007749	0,026506	0,02	0,002	1
Cryl1	0,007762	0,030058	0,02	0,002	1
Nup54	0,007765	-0,04903	0,187	0,262	1
Rnf10	0,007787	0,104494	0,794	0,789	1
Gm10073	0,007787	-0,03343	0,033	0,07	1
Vps26b	0,007802	-0,06012	0,239	0,316	1
Mllt11	0,007814	-0,06072	0,138	0,2	1
Elavl3	0,007824	-0,04954	1	0,996	1
Abca8b	0,007838	-0,08016	0,246	0,323	1
Hist1h1e	0,007852	-0,05229	0,143	0,209	1
Rangrf	0,007898	-0,03422	0,108	0,169	1
Cmas	0,007905	-0,03608	0,283	0,38	1
B3gat2	0,007915	-0,02247	0,011	0,036	1
Ncoa4	0,007976	0,119964	0,639	0,597	1
Wdr41	0,00799	-0,05473	0,231	0,31	1
9430038I0	0,007992	-0,03422	0,077	0,129	1
Nfix	0,007997	0,082234	0,998	0,996	1
Tmem216	0,007998	-0,03306	0,04	0,08	1
Prss36	0,008008	-0,03171	0,05	0,093	1
Trmt2a	0,008008	-0,03928	0,237	0,321	1
Psmc5	0,008034	-0,06305	0,756	0,838	1
Atxn7l1	0,008041	-0,04694	0,088	0,141	1
Cd44	0,008051	0,101584	0,117	0,07	1
Tmem183a	0,008052	-0,04772	0,272	0,357	1
Sri	0,008065	-0,06722	0,69	0,789	1
Fem1b	0,008084	0,111736	0,339	0,278	1
Med24	0,008105	-0,04968	0,171	0,243	1
Mrps5	0,00812	-0,0279	0,406	0,525	1
Parp12	0,008128	0,072941	0,125	0,076	1
Gm4950	0,008141	0,018182	0,015	0	1
Lama5	0,008141	0,016379	0,015	0	1
Mgp	0,008141	0,016379	0,015	0	1

Ajuba	0,008141	0,016379	0,015	0	1
Tmem95	0,008141	0,016379	0,015	0	1
Pappa	0,008141	0,021779	0,015	0	1
Tmem88	0,008141	0,021779	0,015	0	1
Gm26545	0,008141	0,019982	0,015	0	1
Podxl	0,008141	0,019982	0,015	0	1
S100a8	0,008141	0,021779	0,015	0	1
Tfap2a	0,008141	0,023573	0,015	0	1
Ppm1n	0,008141	0,048353	0,015	0	1
Polr3b	0,008158	-0,0594	0,101	0,156	1
Tmem185a	0,008167	-0,03768	0,101	0,158	1
1700021F(	0,008172	-0,04706	0,15	0,217	1
Alcam	0,008175	-0,05998	0,628	0,726	1
Vps51	0,008179	-0,06107	0,09	0,141	1
Ikbkb	0,008195	-0,04989	0,328	0,42	1
Slc25a12	0,008202	-0,05865	0,345	0,447	1
Rnf146	0,008222	-0,03592	0,435	0,549	1
Rnf4	0,008225	-0,05579	0,371	0,466	1
Zfp707	0,00823	-0,0378	0,066	0,114	1
Ociad1	0,00824	-0,05341	0,873	0,892	1
Vps33b	0,008246	-0,04081	0,134	0,198	1
Cenpa	0,008251	-0,02689	0,026	0,059	1
Nhsl1	0,008262	0,050584	0,059	0,025	1
Tceanc	0,008266	-0,03725	0,064	0,112	1
Tmem97	0,008272	-0,05233	0,275	0,363	1
Setd7	0,008275	0,112518	0,646	0,605	1
Ccdc181	0,008301	-0,04726	0,084	0,137	1
Tor2a	0,008314	-0,03742	0,163	0,236	1
Pfdn1	0,008316	-0,05842	0,593	0,69	1
Uck1	0,008317	-0,0495	0,281	0,369	1
Fabp3	0,008324	-0,03735	0,035	0,072	1
Bcs1l	0,008349	-0,03875	0,127	0,19	1
Cyb5r4	0,008361	-0,04441	0,189	0,264	1
Snapc3	0,008382	-0,05396	0,235	0,314	1
Nicn1	0,008404	-0,03978	0,255	0,342	1
Preb	0,008424	-0,01791	0,349	0,464	1
Hemk1	0,008428	-0,04102	0,07	0,118	1
Gm7598	0,00846	-0,01736	0,02	0,051	1
Trak2	0,008469	0,099108	0,194	0,137	1
Flot1	0,008481	-0,07208	0,29	0,369	1
Arxes2	0,008483	-0,01258	0	0,013	1
Ect2	0,008483	-0,01258	0	0,013	1
Postn	0,008483	-0,01258	0	0,013	1
Gm43653	0,008483	-0,01258	0	0,013	1
Gpr162	0,008483	-0,01258	0	0,013	1
4932443L1	0,008483	-0,01258	0	0,013	1
4933405L1	0,008483	-0,01258	0	0,013	1
Serpinb1b	0,008483	-0,01258	0	0,013	1
Ly6h	0,008483	-0,01258	0	0,013	1
Atp2b4	0,008483	-0,01466	0	0,013	1
Rlbp1	0,008483	-0,01466	0	0,013	1
Trim9	0,008483	-0,01466	0	0,013	1

Cabp1	0,008483	-0,01881	0	0,013	1
Lrrc24	0,008483	-0,01674	0	0,013	1
Doc2g	0,008483	-0,02088	0	0,013	1
Ip6k2	0,008499	-0,01639	0,308	0,411	1
H2-T23	0,008513	0,107663	0,064	0,03	1
Tap2	0,008513	0,060904	0,046	0,017	1
Haus3	0,00853	-0,03704	0,202	0,281	1
Ncapd3	0,008534	-0,0252	0,334	0,441	1
Alg9	0,008543	-0,03306	0,171	0,243	1
Rab22a	0,008545	0,141423	0,541	0,513	1
Gm12324	0,00856	-0,03559	0,035	0,072	1
Gm49375	0,008573	-0,05642	0,048	0,089	1
Ints10	0,008579	-0,05337	0,191	0,262	1
Fzr1	0,008582	-0,04854	0,233	0,312	1
Man2b1	0,008584	-0,0231	0,262	0,354	1
Nif3l1	0,008594	-0,0436	0,092	0,146	1
Zfp488	0,008619	0,05784	0,094	0,051	1
Anapc10	0,008621	-0,03604	0,172	0,247	1
Afg3l2	0,008635	-0,05395	0,167	0,234	1
Pla2g6	0,008639	-0,04491	0,108	0,167	1
Kif2a	0,00866	-0,02939	0,433	0,557	1
Ado	0,008669	-0,05533	0,983	0,977	1
Cdk5rap2	0,008674	-0,0794	0,561	0,65	1
Txndc15	0,008689	-0,06073	0,64	0,749	1
Ube2b	0,00869	-0,04256	0,798	0,873	1
Ftl1	0,008694	0,134231	1	1	1
Clic1	0,008712	0,043769	0,059	0,025	1
Snx30	0,008713	0,1276	0,818	0,793	1
Zcchc24	0,008725	0,133194	0,839	0,793	1
Lypd6	0,00873	0,112542	0,183	0,129	1
Rpap2	0,008736	-0,04937	0,127	0,188	1
Rnls	0,008737	-0,03151	0,044	0,084	1
Osbp	0,008776	-0,06089	0,431	0,542	1
Rps3a1	0,008778	-0,03833	0,998	0,996	1
2010320M	0,008783	-0,03136	0,053	0,097	1
Rcan1	0,008814	-0,02818	0,165	0,238	1
Zfp703	0,008824	0,104359	0,121	0,074	1
Hectd4	0,008825	-0,02964	0,284	0,382	1
Renbp	0,008831	0,061274	0,09	0,049	1
Hhatl	0,008832	0,22406	0,49	0,481	1
Lrrc57	0,008843	-0,0425	0,194	0,27	1
Dnah8	0,008899	-0,02384	0,006	0,025	1
Nceh1	0,008932	0,166024	0,624	0,572	1
Ppp1r13b	0,008949	0,126562	0,303	0,247	1
Ccdc50	0,00895	0,151273	0,611	0,593	1
Thbs3	0,008973	-0,05849	0,217	0,293	1
Ptprz1	0,008978	-0,01974	0,006	0,025	1
Urgcp	0,008979	-0,04167	0,16	0,228	1
Sestd1	0,00899	-0,0441	0,095	0,15	1
Cstad	0,008992	-0,01248	0,006	0,025	1
Gm49086	0,009018	-0,01405	0,006	0,025	1
Gm27017	0,009018	-0,01769	0,006	0,025	1

Psmd12	0,009042	-0,04766	0,536	0,652	1
Tarbp2	0,009055	-0,04706	0,149	0,215	1
Fam160b2	0,009062	-0,04007	0,125	0,186	1
Fam131a	0,009087	-0,05165	0,081	0,131	1
Sars2	0,009095	-0,0456	0,088	0,141	1
Enpp3	0,009095	-0,02334	0,028	0,061	1
Sap18b	0,0091	-0,02517	0,017	0,044	1
Atad5	0,009116	-0,02632	0,075	0,127	1
Fbxl20	0,00912	-0,03678	0,246	0,335	1
Tmc3	0,009136	-0,03458	0,084	0,137	1
Man2c1	0,009154	-0,06	0,242	0,319	1
Acap2	0,00917	-0,05628	0,771	0,846	1
Igip	0,00919	0,149195	0,4	0,367	1
Nmb	0,009192	-0,03004	0,022	0,053	1
Inpp5e	0,009194	-0,03202	0,088	0,141	1
Nalcn	0,009197	-0,04325	0,057	0,101	1
Gm13404	0,009201	-0,02835	0,028	0,061	1
Mavs	0,009213	-0,05168	0,11	0,167	1
Tia1	0,009218	-0,07579	0,604	0,694	1
Nfatc3	0,009232	0,131437	0,442	0,395	1
Tlk1	0,009278	-0,01578	0,367	0,487	1
Mfsd3	0,009365	-0,0572	0,099	0,154	1
Tmem68	0,009379	-0,03486	0,204	0,283	1
Cabin1	0,009388	-0,05191	0,209	0,285	1
Fam149b	0,009396	-0,0506	0,202	0,274	1
Fam160a1	0,009404	0,024725	0,024	0,004	1
Dcp1b	0,009408	-0,04338	0,183	0,257	1
Rmdn2	0,009413	-0,04288	0,048	0,089	1
Cnnm3	0,009419	-0,04118	0,268	0,354	1
Eri3	0,009446	-0,04704	0,499	0,616	1
Fmnl3	0,009458	0,038924	0,042	0,015	1
Kctd18	0,009461	-0,029	0,235	0,319	1
Serpine1	0,009461	0,040294	0,024	0,004	1
Csnk1e	0,009462	-0,05647	0,653	0,753	1
Dus4l	0,009504	-0,0408	0,088	0,141	1
Hoxa7	0,00952	-0,02046	0,017	0,044	1
Ctbs	0,009522	-0,04571	0,117	0,177	1
Dhx30	0,009536	-0,03054	0,426	0,551	1
Zfp418	0,009548	-0,03266	0,037	0,074	1
Nol10	0,00956	-0,03031	0,077	0,129	1
Hist2h2aa1	0,009561	-0,02926	0,055	0,099	1
Lmnb2	0,009575	-0,0572	0,101	0,154	1
Mpv17	0,00959	-0,04022	0,431	0,551	1
Wfikkn1	0,009603	0,02409	0,024	0,004	1
Noc3l	0,009656	-0,0426	0,158	0,226	1
Ppat	0,009658	-0,04412	0,161	0,232	1
Cdk8	0,009694	-0,03555	0,169	0,241	1
Zfp93	0,0097	-0,02805	0,046	0,086	1
Tm9sf3	0,009761	0,092785	0,971	0,958	1
Asph	0,009806	-0,03	0,083	0,135	1
Gtf2ird2	0,009816	-0,02835	0,037	0,074	1
Lactb2	0,009837	-0,05439	0,202	0,272	1

Rad23b	0,009841	0,115198	0,58	0,551	1
Gpr108	0,009854	-0,04591	0,376	0,483	1
Gm31812	0,009868	-0,02473	0,013	0,038	1
Tmem120b	0,009871	-0,02835	0,116	0,175	1
Llgl1	0,009874	-0,06389	0,661	0,776	1
Fkbp4	0,009875	-0,08053	0,771	0,829	1
D930016D	0,009882	-0,0494	0,161	0,228	1
Shroom3	0,009887	-0,03507	0,128	0,192	1
Bach2	0,009889	-0,05202	0,152	0,217	1
Pdzd8	0,009903	-0,04491	0,422	0,532	1
Cask	0,009905	-0,044	0,244	0,325	1
Aspdh	0,009907	0,028114	0,042	0,015	1
Iqcb1	0,009943	-0,04248	0,178	0,251	1
Nabp1	0,009947	0,108494	0,125	0,078	1
Ppil4	0,009949	-0,03918	0,347	0,445	1
Gm48371	0,009949	-0,02202	0,009	0,032	1
Gtpbp2	0,009954	-0,03215	0,317	0,42	1
Neu1	0,00996	-0,03033	0,365	0,477	1
Sdccag8	0,00997	-0,04868	0,193	0,266	1
1600002K(	0,009977	-0,04276	0,081	0,131	1
Mrpl39	0,009984	-0,05216	0,191	0,262	1
Shroom2	0,010008	0,14397	0,596	0,572	1
Nvl	0,010038	-0,04902	0,301	0,392	1
Atxn2l	0,010049	0,125756	0,62	0,586	1
Pisd	0,010072	-0,04584	0,301	0,397	1
Aimp2	0,010089	-0,04645	0,253	0,335	1
Ears2	0,010095	-0,03325	0,048	0,089	1
Crebbp	0,010098	0,124922	0,635	0,591	1
AU022252	0,010112	-0,05419	0,176	0,245	1
Pik3cb	0,010114	-0,04457	0,165	0,234	1
Ppcs	0,010114	-0,03922	0,051	0,093	1
Abhd17c	0,010119	-0,05852	0,167	0,232	1
Mkln1os	0,010127	-0,01928	0,004	0,021	1
Man1c1	0,010132	-0,03497	0,077	0,127	1
S1pr2	0,010132	0,05505	0,092	0,051	1
Zfp526	0,010157	-0,02089	0,013	0,038	1
Gm8013	0,010157	-0,01909	0,013	0,038	1
Wfdc3	0,01016	-0,01721	0,004	0,021	1
Nhsl2	0,01016	-0,01721	0,004	0,021	1
Gm39129	0,01016	-0,01721	0,004	0,021	1
Atp8a2	0,01016	-0,01721	0,004	0,021	1
Tsn	0,010179	-0,08087	0,813	0,876	1
Dock4	0,010186	-0,03447	0,352	0,462	1
Hmces	0,010195	-0,03892	0,138	0,2	1
Mfn2	0,010199	-0,05478	0,266	0,348	1
Suds3	0,010214	-0,0429	0,328	0,426	1
Ddah2	0,01022	0,147775	0,42	0,365	1
Rps25	0,010241	-0,0206	0,982	0,992	1
Gm47469	0,010265	-0,03004	0,024	0,055	1
Syn2	0,010279	-0,01951	0,004	0,021	1
Ogfod2	0,010327	-0,03306	0,174	0,245	1
AC174678.	0,010329	-0,01356	0,004	0,021	1

Ankrd24	0,01033	-0,0556	0,171	0,236	1
Phkb	0,010347	-0,07242	0,255	0,335	1
Ift74	0,010354	-0,0534	0,15	0,215	1
Nacc1	0,010394	-0,05686	0,246	0,323	1
Ep300	0,010423	0,145469	0,565	0,546	1
Greb1l	0,010426	0,022626	0,028	0,006	1
Ppwd1	0,010434	-0,00973	0,294	0,395	1
Abraxas2	0,010441	-0,03422	0,218	0,297	1
Wdtc1	0,010452	-0,05439	0,323	0,407	1
Gbp3	0,010455	0,03995	0,031	0,008	1
Tenm4	0,010483	-0,05001	0,116	0,173	1
Wwtr1	0,010484	0,03117	0,028	0,006	1
Pbx2	0,01049	-0,04569	0,211	0,289	1
Irf7	0,010493	0,032601	0,035	0,011	1
Gm47512	0,010497	-0,02403	0,018	0,046	1
Mamld1	0,010507	0,04344	0,031	0,008	1
Adarb2	0,010509	-0,01698	0,002	0,017	1
Fam49a	0,010509	-0,01698	0,002	0,017	1
Fam227b	0,010528	-0,0149	0,002	0,017	1
Mmd	0,010528	-0,0149	0,002	0,017	1
Gm39473	0,010528	-0,0149	0,002	0,017	1
Capn12	0,010541	-0,01478	0,009	0,032	1
Gcc2	0,010543	-0,07089	0,539	0,641	1
Jmy	0,010545	-0,05685	0,468	0,574	1
Gm43674	0,010554	0,02908	0,035	0,011	1
Trappc13	0,010583	-0,04147	0,345	0,447	1
Mbnl1	0,010586	0,125916	0,927	0,899	1
Ctdsp2	0,010593	0,120662	0,661	0,633	1
Cers4	0,010604	0,134796	0,303	0,238	1
Rpl18	0,010606	-0,02887	0,994	0,992	1
Tmem63b	0,010639	0,109389	0,637	0,599	1
Zfp266	0,010658	-0,05632	0,528	0,633	1
Tug1	0,010677	-0,03354	0,462	0,605	1
Sf3b1	0,010687	0,115116	0,93	0,918	1
Fbxo47	0,010689	-0,01307	0,002	0,017	1
Ccdc137	0,010703	-0,05498	0,141	0,203	1
Armc1	0,010727	-0,05171	0,607	0,743	1
Sec61a2	0,010733	-0,03592	0,499	0,627	1
Zfp212	0,010743	-0,04103	0,112	0,169	1
Qars	0,010818	-0,05221	0,294	0,382	1
Zfp235	0,010843	-0,0357	0,077	0,127	1
Pitpnb	0,010858	-0,03976	0,484	0,595	1
Hist1h4d	0,010871	-0,04136	0,108	0,165	1
Eif3a	0,010884	0,089399	0,939	0,918	1
Dnajc11	0,010913	-0,01134	0,288	0,39	1
Bcor	0,010924	0,110251	0,275	0,217	1
3110043O	0,010932	-0,02625	0,136	0,2	1
Foxk2	0,010943	-0,03452	0,253	0,338	1
Zdhhc23	0,010953	-0,0491	0,057	0,099	1
Slain1	0,010957	0,075505	1	0,998	1
Ufsp1	0,010981	-0,03403	0,066	0,112	1
Eif3j2	0,010988	-0,06022	0,2	0,27	1

Spsb2	0,010996	-0,04194	0,092	0,143	1
Car13	0,011048	-0,03501	0,033	0,068	1
Gm31105	0,011048	-0,03501	0,033	0,068	1
Brwd3	0,011057	-0,02566	0,167	0,238	1
Ypel4	0,011073	-0,04608	0,092	0,143	1
Mtbp	0,011087	-0,03403	0,051	0,093	1
Cep120	0,011097	-0,0477	0,371	0,466	1
E130308A1	0,011102	-0,03803	0,385	0,5	1
Uba3	0,011179	-0,03334	0,383	0,483	1
Cwc27	0,011215	-0,04156	0,246	0,327	1
Matn2	0,011218	-0,04306	0,158	0,226	1
Ccnd3	0,011264	-0,06771	0,239	0,312	1
Rapgef2	0,011269	-0,04766	0,213	0,291	1
Ggt1	0,011309	-0,04234	0,044	0,082	1
Swsap1	0,011324	-0,03223	0,026	0,057	1
Nufip1	0,011337	-0,04596	0,149	0,213	1
Gm14419	0,011344	-0,03045	0,026	0,057	1
Ier3	0,011389	0,064075	0,048	0,019	1
Hspbp1	0,011409	-0,02997	0,273	0,363	1
Cox16	0,011464	-0,04868	0,11	0,165	1
Zfp318	0,011483	-0,04742	0,261	0,342	1
Tha1	0,011503	-0,03167	0,033	0,068	1
Plpbp	0,011508	-0,0894	0,532	0,618	1
Rev1	0,011567	-0,03361	0,228	0,308	1
Srrm1	0,011575	0,099832	0,947	0,939	1
Ampd2	0,011576	-0,0383	0,044	0,082	1
Gtpbp10	0,011601	-0,04011	0,088	0,139	1
Ykt6	0,011613	-0,05608	0,277	0,359	1
Paox	0,011623	-0,04194	0,09	0,141	1
Rbm26	0,011633	-0,04814	0,558	0,677	1
Haus7	0,011633	-0,02674	0,154	0,222	1
Cgrrf1	0,011657	-0,04293	0,183	0,253	1
Ehd3	0,011694	-0,03176	0,226	0,306	1
Sult5a1	0,011765	-0,03402	0,015	0,04	1
Wdr48	0,011802	-0,05198	0,305	0,388	1
Las1l	0,011806	-0,01883	0,356	0,473	1
Kcnj10	0,011901	-0,07451	0,972	0,981	1
Rex1bd	0,011936	-0,06112	0,749	0,84	1
Hat1	0,011936	-0,02737	0,237	0,321	1
Kdm5a	0,011959	0,140562	0,626	0,616	1
Peli2	0,011968	-0,02282	0,339	0,454	1
Picalm	0,011968	-0,07195	0,934	0,956	1
Prss41	0,01197	-0,03121	0,015	0,04	1
Map4k2	0,011978	-0,05231	0,213	0,287	1
Dnajb3	0,01198	-0,02495	0,015	0,04	1
Gm43242	0,011993	-0,05345	0,106	0,16	1
Ube2j2	0,011994	-0,04415	0,402	0,5	1
Slc1a3	0,011997	-0,01179	0,45	0,576	1
Ercc3	0,012031	-0,04005	0,176	0,245	1
Zfp365	0,012056	-0,08556	0,56	0,652	1
Mus81	0,012079	-0,04332	0,125	0,184	1
K230015D(	0,012084	-0,02292	0,015	0,04	1

Galnt4	0,012106	-0,04965	0,075	0,122	1
Eif4g3	0,012109	-0,10841	0,571	0,624	1
Med11	0,012113	-0,03276	0,101	0,156	1
Plekhm3	0,012122	-0,05932	0,295	0,371	1
Gm13483	0,01214	-0,03617	0,046	0,084	1
Dcp2	0,012141	-0,04922	0,117	0,173	1
Slc26a11	0,012149	-0,0438	0,139	0,2	1
Lrrc14	0,012191	-0,0373	0,15	0,215	1
Chd7	0,012197	0,14724	0,701	0,694	1
Tmem201	0,012228	-0,03684	0,18	0,253	1
Comtd1	0,012235	-0,05001	0,191	0,259	1
Slc41a1	0,01225	-0,01891	0,136	0,2	1
Hmgxb3	0,012253	-0,0617	0,123	0,177	1
Nfatc2ip	0,012271	-0,04595	0,081	0,129	1
Rasl11a	0,012307	0,026828	0,018	0,002	1
Ubxn6	0,012316	-0,05734	0,613	0,713	1
Dnajc16	0,012321	-0,05049	0,169	0,234	1
Tmc7	0,012322	-0,00498	0,273	0,376	1
Ssb	0,012359	-0,06501	0,793	0,842	1
Lmo7	0,012361	-0,03678	0,028	0,059	1
Sfn	0,012369	0,019672	0,018	0,002	1
Gm45353	0,012369	0,019672	0,018	0,002	1
BC030499	0,012389	0,019672	0,018	0,002	1
Dis3l	0,012397	-0,03676	0,161	0,228	1
Gabarap	0,012411	-0,02799	0,996	0,994	1
Dtnbp1	0,01244	-0,04308	0,189	0,259	1
Mfsd14b	0,012446	-0,04678	0,499	0,599	1
Fam57a	0,012477	-0,03233	0,382	0,489	1
Dtx3l	0,012488	0,061467	0,044	0,017	1
2310016G	0,0125	-0,01776	0,015	0,04	1
Cactin	0,012505	-0,03852	0,143	0,207	1
Eef1b2	0,012515	-0,04489	0,923	0,937	1
Pros1	0,012557	0,09288	0,209	0,152	1
Cdh1	0,012558	0,03995	0,018	0,002	1
Fgf12	0,012576	-0,0406	0,048	0,086	1
Myrip	0,012577	0,083565	0,178	0,124	1
4930572G	0,012584	-0,02554	0,026	0,057	1
Kcp	0,012584	-0,01974	0,007	0,027	1
Aldh3b2	0,012584	-0,01974	0,007	0,027	1
Nt5c	0,012601	-0,06822	0,283	0,354	1
Thrb	0,01262	-0,02632	0,007	0,027	1
Glt28d2	0,012621	0,019055	0,018	0,002	1
Gm10827	0,012631	-0,05179	0,046	0,084	1
Gm38414	0,012637	-0,02157	0,015	0,04	1
Vps50	0,012643	-0,03969	0,352	0,447	1
Iars	0,012647	-0,04985	0,514	0,637	1
Mfap3	0,012652	-0,04983	0,174	0,241	1
Tnrc18	0,012658	-0,03552	0,27	0,359	1
Zfp68	0,012678	-0,02341	0,29	0,388	1
Mettl13	0,012693	-0,03791	0,121	0,179	1
Toe1	0,012698	-0,03663	0,15	0,215	1
Mtg2	0,012735	-0,05607	0,213	0,285	1

Tlk2	0,012739	-0,05716	0,336	0,42	1
Pan2	0,012781	-0,03796	0,149	0,213	1
Cavin2	0,012803	-0,03574	0,193	0,266	1
Megf11	0,012808	-0,01792	0,007	0,027	1
Commd7	0,012811	-0,05257	0,36	0,449	1
Trim41	0,012825	-0,03311	0,209	0,289	1
BC005624	0,012835	-0,06608	0,679	0,774	1
Tyw1	0,012843	-0,05231	0,237	0,314	1
Sart1	0,012851	-0,05132	0,328	0,416	1
Otud4	0,012852	0,120015	0,391	0,342	1
Gpr155	0,012854	0,108432	0,664	0,62	1
Slc39a3	0,012908	-0,0298	0,433	0,559	1
1110002O	0,012922	-0,02247	0,011	0,034	1
Rbm6	0,012923	0,122237	0,543	0,504	1
Tnfrsf19	0,012932	-0,01322	0,007	0,027	1
Smtn	0,012932	0,081246	0,106	0,063	1
Arhgef18	0,01296	-0,03899	0,119	0,177	1
Wdr54	0,012962	-0,03838	0,097	0,15	1
Slc15a4	0,012976	0,14062	0,683	0,686	1
Glcci1	0,012992	-0,03916	0,123	0,181	1
Sdr39u1	0,013003	-0,03517	0,057	0,099	1
Dcakd	0,013013	-0,04442	0,138	0,198	1
Eif3j1	0,013032	0,115334	0,783	0,743	1
Tpi1	0,013039	0,134694	0,622	0,599	1
Ctr9	0,013064	-0,04557	0,583	0,703	1
Tox	0,013096	-0,03617	0,037	0,072	1
Acsl3	0,013119	0,19798	0,765	0,732	1
Gm14403	0,013142	-0,03932	0,059	0,101	1
Col27a1	0,013156	0,102925	0,136	0,089	1
Ttc4	0,013176	-0,01724	0,323	0,43	1
Znrf1	0,013186	-0,05245	0,297	0,388	1
Dcaf12	0,013193	-0,03493	0,284	0,376	1
Glg1	0,013227	0,106734	0,802	0,789	1
4932438A	0,013237	0,137603	0,697	0,669	1
Cpq	0,013244	-0,03834	0,061	0,103	1
Mgat2	0,01326	-0,02114	0,262	0,352	1
Akip1	0,01326	-0,0375	0,094	0,146	1
Gm11423	0,013281	-0,02421	0,035	0,07	1
Usf1	0,013318	-0,05495	0,327	0,416	1
4930481A	0,013329	-0,02089	0,011	0,034	1
Mosmo	0,01336	-0,0387	0,367	0,468	1
D5Ertd615	0,013362	0,012762	0,013	0	1
Tbc1d1	0,013363	0,021779	0,013	0	1
BC049987	0,013363	0,019982	0,013	0	1
Itga2	0,013363	0,019982	0,013	0	1
Irx3	0,013363	0,025364	0,013	0	1
Fam117a	0,013369	-0,03924	0,097	0,15	1
Snx11	0,013373	-0,01739	0,239	0,323	1
Zdhhc2	0,013429	0,040035	0,062	0,03	1
Cul9	0,013464	-0,07652	0,237	0,304	1
Arfgef2	0,013494	-0,05005	0,327	0,407	1
Zbtb6	0,013552	-0,03185	0,211	0,287	1

Ppp2r1b	0,013563	-0,04475	0,117	0,173	1
Agpat3	0,013614	-0,05709	0,875	0,892	1
Ccdc107	0,013637	-0,05212	0,101	0,152	1
Srbd1	0,013639	-0,03882	0,202	0,274	1
Dusp11	0,013641	0,102264	0,747	0,732	1
Dennd5b	0,013654	-0,04247	0,472	0,595	1
Bud31	0,013685	-0,0375	0,484	0,61	1
Gm15991	0,013698	-0,01525	0,011	0,034	1
Utp20	0,01372	-0,0378	0,105	0,158	1
AC122413.	0,013739	-0,02899	0,017	0,042	1
Mnat1	0,013744	-0,06547	0,268	0,342	1
Dis3l2	0,013768	-0,04219	0,33	0,428	1
Uchl1	0,013791	-0,1893	0,321	0,395	1
Dhodh	0,013817	-0,03544	0,138	0,198	1
Wdr89	0,013828	-0,0438	0,152	0,215	1
Gtpbp8	0,013848	-0,0562	0,125	0,179	1
Rdx	0,01386	0,100325	1	0,996	1
Sap18	0,013872	-0,05155	0,668	0,745	1
Fzd8	0,013876	0,055763	0,073	0,038	1
Ddx56	0,013896	-0,02365	0,25	0,333	1
Rassf3	0,013897	0,071084	0,128	0,082	1
Eno3	0,013901	0,036847	0,04	0,015	1
Ice2	0,013926	-0,0462	0,169	0,236	1
Cln8	0,013926	-0,04989	0,593	0,719	1
Pls3	0,013928	-0,01421	0,426	0,559	1
Hexb	0,013963	-0,03155	0,303	0,39	1
Ippk	0,01399	-0,03485	0,38	0,481	1
Banf1	0,014023	-0,02354	0,426	0,542	1
Zfp120	0,014039	-0,03667	0,119	0,177	1
Zfp983	0,014044	-0,03518	0,05	0,089	1
Abca3	0,014072	-0,03406	0,224	0,302	1
Eif2b5	0,014073	-0,05036	0,321	0,405	1
Dph5	0,014109	-0,04297	0,211	0,281	1
Ldlrap1	0,014122	-0,07902	0,345	0,432	1
Ranbp6	0,014138	-0,04257	0,15	0,213	1
Zc3h15	0,01415	-0,05423	0,661	0,749	1
Tnip1	0,014159	-0,04608	0,244	0,321	1
Nprl2	0,014164	-0,04412	0,202	0,272	1
Atg10	0,014166	-0,04488	0,156	0,217	1
Hnrnpd	0,014171	0,12214	0,717	0,709	1
Umad1	0,014177	-0,05085	0,141	0,2	1
Poli	0,014196	-0,02835	0,037	0,072	1
Fam81a	0,014198	0,026676	0,04	0,015	1
Gtf3c5	0,014199	-0,04911	0,167	0,232	1
Lsm10	0,014211	-0,0476	0,178	0,243	1
Efr3a	0,014222	-0,04629	0,261	0,342	1
Dcun1d3	0,014234	-0,03184	0,149	0,213	1
Alkbh8	0,014238	-0,04582	0,255	0,335	1
Nans	0,014269	-0,04696	0,18	0,247	1
Zfp775	0,014273	-0,0456	0,094	0,143	1
Gramd1c	0,014286	-0,02315	0,017	0,042	1
Slc39a14	0,014288	0,112119	0,235	0,179	1

Gm29083	0,01429	-0,03659	0,031	0,063	1
Hif1a	0,014328	0,115682	0,545	0,513	1
Apbb1ip	0,01434	-0,04304	0,139	0,2	1
Nnt	0,014349	-0,02359	0,017	0,042	1
Ankrd12	0,014465	0,117709	0,89	0,859	1
Dbi	0,014478	-0,05233	1	0,998	1
Rps21	0,014483	-0,04013	1	0,998	1
Siah2	0,014493	-0,04574	0,327	0,418	1
Trim26	0,014509	-0,05486	0,281	0,359	1
Grb2	0,014536	-0,056	0,708	0,776	1
Ercc1	0,014591	-0,03167	0,251	0,333	1
Dus3l	0,014593	-0,05466	0,273	0,35	1
Dnttip1	0,014595	-0,04335	0,295	0,382	1
Zfp647	0,014596	-0,02224	0,022	0,051	1
Abhd10	0,014597	-0,04861	0,161	0,224	1
Lrtm2	0,014606	0,037521	0,037	0,013	1
Parp4	0,014631	-0,03685	0,229	0,306	1
Cacna2d4	0,014631	0,082768	0,081	0,044	1
Acox3	0,014659	0,107712	0,242	0,184	1
Tnfrsf13c	0,014672	-0,03403	0,064	0,108	1
Wwc2	0,014717	0,151224	0,323	0,274	1
Rpl10	0,014728	-0,01508	0,982	0,981	1
Hexa	0,014763	0,102167	0,651	0,62	1
Fbxo34	0,014769	-0,03625	0,086	0,135	1
Samd9l	0,014795	0,032272	0,037	0,013	1
Man2b2	0,014806	-0,03295	0,101	0,154	1
Rcbtb2	0,01484	-0,03383	0,051	0,091	1
1700019L1	0,014857	0,039639	0,05	0,021	1
Ddx19b	0,014883	-0,04359	0,075	0,12	1
Aldh1b1	0,014891	0,032272	0,037	0,013	1
Iws1	0,014902	-0,02678	0,394	0,5	1
Asb13	0,014938	-0,03647	0,066	0,11	1
Grwd1	0,014946	-0,04235	0,123	0,179	1
Rars	0,014947	-0,04471	0,185	0,253	1
Stip1	0,014974	-0,04526	0,587	0,686	1
Flnc	0,014979	0,097551	0,176	0,122	1
Brsk1	0,014984	-0,04371	0,229	0,304	1
Zfp710	0,014996	-0,05386	0,134	0,192	1
Nbeal1	0,015008	0,110799	0,6	0,563	1
Nr2c1	0,015013	-0,05425	0,163	0,224	1
Hmgb2	0,015014	-0,03493	0,101	0,154	1
Vps26a	0,015024	-0,03897	0,42	0,53	1
Ddb2	0,015083	-0,03365	0,066	0,11	1
Gm3448	0,015085	-0,01059	0,099	0,152	1
Natd1	0,015086	-0,0481	0,158	0,219	1
Gatc	0,015122	-0,04124	0,165	0,232	1
Satb1	0,015123	-0,04262	0,092	0,141	1
E130311K1	0,015189	-0,0395	0,07	0,114	1
Il11	0,015208	-0,03092	0,04	0,076	1
Gm17259	0,015212	-0,02886	0,05	0,089	1
Col9a3	0,015249	-0,05267	0,376	0,468	1
Adck2	0,015268	-0,05257	0,149	0,207	1

Cdkl2	0,015289	0,096618	0,193	0,141	1
Zfp644	0,015328	0,13299	0,71	0,709	1
Gm26916	0,015329	0,051749	0,033	0,011	1
Slc19a1	0,015368	-0,03171	0,051	0,091	1
Zcchc6	0,015437	0,129168	0,541	0,519	1
Ano8	0,015458	-0,05613	0,141	0,198	1
Timp4	0,01547	-0,01951	0,006	0,023	1
A730017C2	0,01547	-0,01951	0,006	0,023	1
Atg12	0,015488	-0,03151	0,288	0,373	1
Apmap	0,015502	-0,029	0,257	0,344	1
Igsf9b	0,015519	0,11348	0,228	0,173	1
Gna13	0,015528	0,16063	0,417	0,38	1
Tspan12	0,015537	-0,01745	0,006	0,023	1
Nek11	0,015537	-0,01745	0,006	0,023	1
Krt28	0,015537	-0,01745	0,006	0,023	1
Hnrnpa2b1	0,015546	0,077037	0,998	0,998	1
Gm15893	0,015592	0,032601	0,033	0,011	1
Mpi	0,015594	-0,03697	0,09	0,139	1
Pygb	0,015604	-0,04129	0,182	0,249	1
Ino80dos	0,015669	-0,02119	0,097	0,15	1
Micall2	0,01567	0,039671	0,064	0,032	1
Kbtbd4	0,015715	-0,03643	0,088	0,137	1
A430105J0	0,01572	-0,01769	0,006	0,023	1
B630019K0	0,015725	-0,05605	0,09	0,137	1
Nek4	0,015727	-0,023	0,215	0,293	1
Lrpprc	0,015743	-0,04356	0,36	0,451	1
Spice1	0,015749	-0,03839	0,165	0,232	1
Spata2	0,015779	-0,03855	0,422	0,538	1
Eri1	0,015779	-0,05457	0,24	0,316	1
Adam17	0,015806	-0,0393	0,33	0,43	1
Mettl5	0,015839	-0,04326	0,213	0,285	1
Tapbpl	0,015853	-0,01805	0,029	0,061	1
Gtf3c4	0,015861	-0,0421	0,149	0,209	1
Cdkn2a	0,015863	0,027959	0,026	0,006	1
Zfp846	0,015888	-0,04104	0,174	0,241	1
Gm3604	0,015895	-0,0227	0,013	0,036	1
Btbd9	0,015912	-0,0408	0,336	0,42	1
Tuba4a	0,015928	0,05987	0,123	0,078	1
Cldn10	0,015974	-0,01587	0,006	0,023	1
C2cd4c	0,015988	0,024407	0,026	0,006	1
Fam46a	0,016007	-0,04941	0,233	0,306	1
Ctsz	0,016086	-0,05671	0,712	0,795	1
Atp11a	0,016091	0,124878	0,848	0,81	1
Eid3	0,016104	0,034691	0,029	0,008	1
Taco1	0,016106	-0,03247	0,042	0,078	1
Ddx58	0,01611	0,051829	0,07	0,036	1
Vti1a	0,016121	-0,02361	0,27	0,357	1
Cep128	0,016121	-0,04477	0,081	0,127	1
6330418K0	0,016136	-0,02447	0,024	0,053	1
Pold2	0,016138	-0,03673	0,224	0,297	1
Rab6a	0,016173	-0,03789	0,954	0,958	1
Mex3a	0,01618	0,034691	0,026	0,006	1

Arhgef7	0,016218	0,076548	0,239	0,179	1
Mrpl47	0,016237	-0,05139	0,114	0,167	1
Oser1	0,016249	-0,02692	0,459	0,574	1
Atp6v1d	0,016273	-0,05177	0,585	0,688	1
Ddx41	0,016285	-0,03864	0,191	0,259	1
Synpr	0,016326	-0,03442	0,018	0,044	1
Pfkfb1	0,016326	-0,01049	0	0,011	1
Gm30173	0,016326	-0,01049	0	0,011	1
Etnppl	0,016326	-0,01049	0	0,011	1
Gm43689	0,016326	-0,01049	0	0,011	1
Glipr2	0,016326	-0,01049	0	0,011	1
2900089D:	0,016326	-0,01049	0	0,011	1
Gm20387	0,016326	-0,01049	0	0,011	1
Ntn5	0,016326	-0,01049	0	0,011	1
Asb5	0,016326	-0,01049	0	0,011	1
Nwd1	0,016326	-0,01049	0	0,011	1
Gm16573	0,016326	-0,01049	0	0,011	1
Camkv	0,016326	-0,01049	0	0,011	1
Tmie	0,016326	-0,01049	0	0,011	1
Tdgf1	0,016326	-0,01049	0	0,011	1
Rasl10b	0,016326	-0,01049	0	0,011	1
Gm11437	0,016326	-0,01049	0	0,011	1
Rbfox3	0,016326	-0,01049	0	0,011	1
Gm5678	0,016326	-0,01049	0	0,011	1
Pcdhga1	0,016326	-0,01049	0	0,011	1
Nudt8	0,016326	-0,01049	0	0,011	1
Cit	0,016326	-0,01258	0	0,011	1
Gm15915	0,016326	-0,01258	0	0,011	1
2900052N(	0,016326	-0,01258	0	0,011	1
Bex1	0,016326	-0,01466	0	0,011	1
Gm16233	0,016327	-0,01881	0	0,011	1
Zfp871	0,016338	-0,02666	0,372	0,483	1
Ppp2r5b	0,016396	-0,05762	0,506	0,605	1
Pxdn	0,016501	-0,03386	0,103	0,156	1
Eftud2	0,016502	-0,01788	0,272	0,361	1
Ranbp17	0,016517	-0,02202	0,009	0,03	1
Pld6	0,016517	-0,02202	0,009	0,03	1
Mettl1	0,016559	-0,01004	0,231	0,312	1
Lgi3	0,016565	0,11387	0,938	0,907	1
Ccdc18	0,016581	-0,02451	0,009	0,03	1
A930017K:	0,016588	0,077899	0,145	0,097	1
Blzf1	0,016609	-0,03214	0,202	0,274	1
Chml	0,01663	0,057897	0,11	0,068	1
Ppm1h	0,016676	-0,03404	0,13	0,188	1
Zfp383	0,016721	-0,0427	0,138	0,196	1
Orai3	0,016774	-0,04001	0,106	0,158	1
AI597479	0,016778	-0,05314	0,149	0,207	1
Gm34006	0,016796	0,033034	0,046	0,019	1
Eif2ak1	0,016802	-0,02646	0,292	0,382	1
Ptrh1	0,016807	-0,02021	0,009	0,03	1
Slc5a3	0,016811	0,156478	0,602	0,563	1
Iqck	0,016828	-0,01839	0,009	0,03	1

Ppp3cc	0,016849	-0,03258	0,189	0,259	1
Crlf3	0,01689	-0,05608	0,235	0,304	1
Eif6	0,016922	-0,05884	0,727	0,802	1
Bzw1	0,016981	-0,04213	0,917	0,945	1
Gas6	0,016985	0,062681	0,284	0,222	1
Trappc5	0,016989	-0,06839	0,483	0,568	1
Rom1	0,017009	-0,03598	0,128	0,186	1
Psmc4	0,017035	-0,04873	0,628	0,715	1
Dcc	0,017045	-0,02825	0,026	0,055	1
Tra2a	0,01707	0,100392	0,932	0,916	1
Xpo5	0,01709	-0,03651	0,059	0,099	1
2810457G	0,017219	-0,01978	0,018	0,044	1
Hgh1	0,017268	-0,02421	0,033	0,065	1
Dlgap4	0,017308	-0,03796	0,152	0,213	1
Gm4419	0,017332	-0,03657	0,101	0,152	1
Ppp1r12c	0,017384	-0,03367	0,33	0,424	1
BC004004	0,017413	-0,05761	0,47	0,559	1
Simc1	0,017432	-0,03127	0,088	0,137	1
Gas2l1	0,017438	-0,04923	0,193	0,257	1
Tsfm	0,017444	-0,04564	0,176	0,238	1
Pdxdc1	0,017481	-0,05257	0,637	0,73	1
Zfp664	0,017495	0,098655	0,699	0,662	1
Nfrkb	0,017517	-0,02843	0,239	0,319	1
Slc31a1	0,017531	-0,04031	0,064	0,105	1
Eif3g	0,017556	-0,05826	0,727	0,8	1
Hist2h4	0,017575	-0,01799	0,018	0,044	1
Tfb2m	0,017617	-0,03571	0,169	0,234	1
Sirt5	0,017634	-0,03013	0,11	0,165	1
Ccdc97	0,017666	-0,03656	0,176	0,243	1
Glb1	0,017678	-0,02135	0,026	0,055	1
Ing3	0,017683	-0,01542	0,218	0,297	1
Nrde2	0,017706	-0,03456	0,138	0,196	1
Cpt2	0,017752	-0,03527	0,138	0,196	1
Aifm1	0,017758	-0,05124	0,2	0,266	1
3110062M	0,017768	-0,05661	0,132	0,186	1
Copz1	0,017843	-0,02946	0,453	0,574	1
2900055J2	0,017853	0,073847	0,128	0,084	1
Zfp146	0,01792	-0,03076	0,095	0,146	1
Nelfb	0,017945	-0,03249	0,349	0,441	1
Spns1	0,017988	-0,04383	0,439	0,532	1
Kpna2	0,01804	-0,03323	0,037	0,07	1
Strbp	0,018043	-0,04413	0,209	0,281	1
Qrsl1	0,018053	-0,01772	0,051	0,091	1
Aagab	0,018165	-0,04919	0,158	0,215	1
Rfx3	0,018166	-0,03248	0,13	0,188	1
Cnpy4	0,018183	-0,03829	0,161	0,224	1
Xpnpep1	0,018186	-0,03551	0,198	0,266	1
Hook2	0,018205	0,045793	0,066	0,034	1
Dmpk	0,01821	-0,04214	0,165	0,228	1
Dbndd2	0,018239	0,097578	0,993	0,983	1
Pex11a	0,018245	-0,03554	0,062	0,103	1
Ddah1	0,018296	-0,01721	0,004	0,019	1

Gm12204	0,018296	-0,01721	0,004	0,019	1
Slc9a3r1	0,018298	-0,05352	0,222	0,295	1
Mturn	0,01832	0,101847	0,165	0,116	1
1810044D(	0,018336	-0,03498	0,061	0,101	1
Serpini1	0,018345	-0,03018	0,044	0,08	1
Mapk8ip2	0,018346	0,041707	0,042	0,017	1
Rab20	0,018354	-0,01515	0,004	0,019	1
Dcaf7	0,018363	-0,03254	0,242	0,321	1
Gm28809	0,018394	0,04378	0,042	0,017	1
Fig4	0,018412	-0,04459	0,123	0,177	1
Rfxank	0,018441	-0,04882	0,165	0,226	1
Plekha8	0,018489	-0,0533	0,114	0,165	1
Cnbd2	0,018509	-0,03922	0,05	0,086	1
Mtrf1	0,018509	-0,05087	0,073	0,116	1
Lpo	0,018523	-0,02563	0,055	0,095	1
Papola	0,018578	-0,02497	0,639	0,747	1
Herc3	0,018591	-0,03914	0,064	0,105	1
Mettl14	0,018594	-0,03927	0,22	0,285	1
Unkl	0,018607	-0,04063	0,094	0,141	1
Naga	0,018637	-0,05645	0,2	0,264	1
Thg1l	0,018658	-0,03743	0,064	0,105	1
Keap1	0,018687	-0,03168	0,417	0,521	1
2700038G:	0,018741	-0,03136	0,061	0,101	1
Pcyt1a	0,018746	-0,03926	0,398	0,506	1
Lpar1	0,018758	-0,05861	0,974	0,981	1
St8sia1	0,018772	-0,01563	0,004	0,019	1
Cd300e	0,018779	-0,02495	0,015	0,038	1
Fcf1	0,018827	-0,02442	0,325	0,414	1
Stx1b	0,018836	0,093882	0,161	0,114	1
Morn4	0,018877	-0,03875	0,128	0,184	1
Miga2	0,018927	-0,03583	0,112	0,165	1
Zfp951	0,018936	-0,02292	0,015	0,038	1
Pskh1	0,018964	-0,03671	0,117	0,171	1
Fbxl3	0,018971	0,111544	0,75	0,762	1
Nup160	0,018978	-0,03371	0,154	0,215	1
Myo5a	0,01903	0,139265	0,305	0,259	1
Dffb	0,019091	-0,02396	0,046	0,082	1
mt-Nd4l	0,019094	0,086079	0,993	0,992	1
Tspan32	0,019098	-0,02719	0,015	0,038	1
Slc39a10	0,019117	0,076185	0,172	0,122	1
Ankrd16	0,019214	-0,0291	0,119	0,175	1
Tmem19	0,019217	-0,04147	0,44	0,546	1
Banp	0,019232	-0,03021	0,206	0,276	1
Nampt	0,019298	0,129128	0,611	0,586	1
Cep63	0,019317	-0,03488	0,167	0,232	1
Gmeb2	0,019354	-0,03865	0,116	0,169	1
Abcb7	0,019363	-0,03405	0,147	0,205	1
Jrk	0,019415	-0,02764	0,048	0,084	1
Aip	0,019423	-0,04436	0,538	0,643	1
Stk4	0,019458	-0,06466	0,286	0,357	1
Tmem74	0,019519	-0,03345	0,05	0,086	1
Nup88	0,019534	-0,04376	0,31	0,39	1

Rbbp4	0,019553	-0,06041	0,846	0,882	1
Slc19a2	0,019584	-0,00305	0,02	0,046	1
Clu	0,019639	-0,02075	0,182	0,247	1
Rpl34	0,019669	-0,03758	0,993	0,994	1
Trim68	0,019676	-0,03132	0,05	0,086	1
Ndor1	0,019679	-0,03711	0,053	0,091	1
Nefl	0,019697	-0,0149	0,002	0,015	1
Ypel1	0,019702	-0,01855	0,044	0,08	1
Gm47283	0,019704	0,14495	0,727	0,7	1
8430432A(	0,019734	-0,01283	0,002	0,015	1
Dnah7b	0,019734	-0,01283	0,002	0,015	1
Ccdc160	0,019734	-0,01283	0,002	0,015	1
Kcna3	0,019734	-0,01283	0,002	0,015	1
Spata18	0,019734	-0,01283	0,002	0,015	1
B230322F(	0,019734	-0,01283	0,002	0,015	1
Gm16845	0,019734	-0,01283	0,002	0,015	1
Thy1	0,019734	-0,01283	0,002	0,015	1
Ankrd11	0,019747	-0,0602	0,831	0,863	1
Plcb1	0,019771	-0,01799	0,015	0,038	1
Vrk3	0,019775	-0,0397	0,292	0,369	1
Prmt3	0,019799	-0,05322	0,226	0,293	1
4933406C1	0,019802	-0,0415	0,055	0,093	1
Wdr43	0,019806	-0,01836	0,372	0,475	1
Dnajc21	0,019869	-0,03843	0,341	0,424	1
Gm33819	0,019882	-0,01721	0,002	0,015	1
Oprk1	0,019951	0,025044	0,017	0,002	1
Nab2	0,019951	0,028609	0,017	0,002	1
Daglb	0,019964	-0,03407	0,288	0,367	1
Gm32122	0,019979	-0,11638	0,097	0,146	1
Nup210l	0,019984	0,019672	0,017	0,002	1
Hes7	0,019984	0,019672	0,017	0,002	1
Tcf3	0,019986	-0,03713	0,321	0,405	1
Rab23	0,019993	-0,03171	0,262	0,344	1
Elavl4	0,019993	-0,011	0,002	0,015	1
Gm4724	0,019993	-0,00942	0,002	0,015	1
Cadm4	0,020022	-0,0355	1	0,992	1
2210016F1	0,020046	-0,02125	0,187	0,255	1
Ube2t	0,02005	0,016075	0,017	0,002	1
Gm11491	0,02005	0,016075	0,017	0,002	1
Ap3b1	0,020067	-0,04101	0,336	0,42	1
Necap1	0,020077	-0,02234	0,18	0,247	1
A3galt2	0,020083	0,014271	0,017	0,002	1
Lhfpl3	0,020092	-0,0218	0,037	0,07	1
Nos1ap	0,0201	-0,0333	0,083	0,129	1
Eri2	0,020172	-0,03404	0,121	0,175	1
4921524J1	0,02018	-0,03387	0,145	0,205	1
Prrc2a	0,020203	0,118656	0,679	0,694	1
Zfp420	0,020204	-0,0408	0,095	0,143	1
Pink1	0,020219	0,108712	0,824	0,8	1
Mrpl38	0,020236	0,000845	0,314	0,411	1
Mrpl24	0,020271	-0,05862	0,602	0,686	1
Cdk9	0,020371	-0,02821	0,36	0,458	1

Sin3b	0,020383	-0,05657	0,554	0,65	1
Ptpn3	0,020388	0,017264	0,017	0,002	1
Mipep	0,020388	-0,03403	0,068	0,11	1
Ccdc59	0,020423	-0,05372	0,646	0,745	1
Ppp2r3a	0,020457	-0,06986	0,917	0,922	1
Oxsm	0,020526	-0,01734	0,059	0,099	1
Cxxc5	0,020556	0,110155	0,85	0,85	1
Sash1	0,020572	-0,06914	0,717	0,789	1
Fadd	0,02061	-0,04814	0,11	0,16	1
Slc30a1	0,020643	0,130396	0,248	0,2	1
Palb2	0,020679	-0,02225	0,011	0,032	1
Trim39	0,020706	-0,03705	0,171	0,232	1
Rnf139	0,020771	-0,02858	0,29	0,373	1
Pycard	0,020792	0,049523	0,062	0,032	1
Ifi27l2a	0,020845	0,345406	0,105	0,065	1
Rnf115	0,020849	-0,01857	0,339	0,437	1
Cdc14b	0,020871	-0,04091	0,072	0,114	1
Apobr	0,020897	-0,02247	0,011	0,032	1
Pphln1	0,020914	-0,04455	0,229	0,297	1
Itpkb	0,020945	-0,02066	0,011	0,032	1
Strip2	0,020988	0,045477	0,048	0,021	1
Mtf2	0,021003	-0,01409	0,334	0,422	1
Alyref2	0,021003	-0,04119	0,116	0,167	1
Ss18l1	0,021059	-0,03683	0,077	0,12	1
P3h3	0,021073	-0,03199	0,007	0,025	1
Washc1	0,021141	-0,04247	0,31	0,39	1
Tars2	0,021156	-0,04104	0,2	0,268	1
B3gntl1	0,021225	-0,0248	0,053	0,091	1
Rbm15	0,021248	-0,01058	0,312	0,405	1
Ppih	0,021252	-0,03038	0,134	0,19	1
A230107N(	0,021293	-0,01974	0,007	0,025	1
Actr5	0,021323	-0,02413	0,13	0,188	1
Alg13	0,021334	-0,04589	0,108	0,158	1
Psma3	0,021338	-0,04521	0,89	0,899	1
Nap1l4	0,02134	-0,04107	0,824	0,886	1
Elovl4	0,021362	0,068859	0,176	0,127	1
Plagl2	0,021369	-0,02915	0,066	0,108	1
Crot	0,021411	-0,04319	0,261	0,335	1
Med17	0,021423	-0,04782	0,27	0,34	1
Myo1d	0,021486	-0,04204	0,776	0,838	1
Ncbp1	0,021504	-0,04172	0,222	0,291	1
Sorbs1	0,021531	-0,03299	0,128	0,184	1
Rest	0,021556	-0,02102	0,286	0,376	1
Atg4b	0,021587	-0,05408	0,323	0,403	1
Grsf1	0,021591	-0,03473	0,143	0,2	1
Psmd7	0,021605	-0,04427	0,857	0,914	1
Eif3c	0,021615	0,084527	0,947	0,945	1
Gabrr2	0,021633	-0,04212	0,024	0,051	1
Trmt10c	0,021634	-0,0389	0,108	0,158	1
Rngtt	0,021646	-0,03775	0,273	0,348	1
Fmn1	0,021658	0,115064	0,27	0,217	1
3110082I1	0,02167	-0,03106	0,031	0,061	1

Ribc1	0,021673	-0,02539	0,017	0,04	1
Nbl1	0,021744	-0,01405	0,007	0,025	1
Fhl1	0,021752	-0,03836	0,396	0,496	1
Prr11	0,021778	-0,01682	0,011	0,032	1
Gpr107	0,021795	-0,03525	0,349	0,447	1
Gm46430	0,021797	-0,03403	0,077	0,12	1
Mga	0,021834	0,155676	0,536	0,506	1
Kat5	0,021856	-0,02779	0,244	0,321	1
Eif1ad	0,021899	-0,03708	0,211	0,276	1
2510009E(	0,021944	0,138148	0,598	0,576	1
Clptm1l	0,021955	-0,06258	0,508	0,599	1
Gm17484	0,021958	-0,02908	0,031	0,061	1
Shmt1	0,021998	-0,03092	0,04	0,074	1
Gnb5	0,022	-0,02714	0,119	0,173	1
Gfm1	0,022002	-0,02447	0,147	0,207	1
Mcm3ap	0,022061	-0,04696	0,18	0,241	1
Tnc	0,022068	0,010949	0,011	0	1
Gm26745	0,022068	0,010949	0,011	0	1
Gm44700	0,022068	0,010949	0,011	0	1
Unc5a	0,022068	0,014572	0,011	0	1
Pigr	0,022068	0,012762	0,011	0	1
Gm37634	0,022068	0,012762	0,011	0	1
Olfr1238	0,022068	0,012762	0,011	0	1
Gm26784	0,022068	0,014572	0,011	0	1
Olfr328	0,022068	0,014572	0,011	0	1
Mocos	0,022069	0,019982	0,011	0	1
AC125141.	0,022069	0,018182	0,011	0	1
Olfr267	0,022069	0,016379	0,011	0	1
Selplg	0,022069	0,016379	0,011	0	1
Ppp1r18os	0,022069	0,016379	0,011	0	1
Aqp5	0,022069	0,021779	0,011	0	1
Lsmem1	0,022069	0,019982	0,011	0	1
Iigp1	0,022069	0,039574	0,011	0	1
Galnt5	0,022069	0,021779	0,011	0	1
Supt5	0,022092	0,132585	0,53	0,513	1
Ilf3	0,022131	0,118476	0,516	0,489	1
Sp2	0,022179	-0,03532	0,222	0,293	1
Zfp64	0,022211	-0,03646	0,147	0,205	1
C920021L1	0,022273	-0,02576	0,031	0,061	1
Rgs17	0,022339	0,059976	0,053	0,025	1
B3gat1	0,022342	-0,04058	0,332	0,418	1
Esyt1	0,022356	0,054103	0,081	0,046	1
Npepps	0,022363	-0,05351	0,853	0,884	1
Nudcd3	0,022401	0,114832	0,699	0,696	1
Cstf2t	0,022403	-0,02261	0,284	0,369	1
Secisbp2	0,022456	-0,02042	0,323	0,411	1
Apex1	0,022493	-0,03184	0,099	0,148	1
Slc25a53	0,022535	-0,03441	0,044	0,078	1
Exosc10	0,022569	-0,01766	0,272	0,357	1
Tle6	0,02257	-0,03441	0,057	0,095	1
Tstd3	0,02259	-0,0321	0,057	0,095	1
Fech	0,022634	-0,07128	0,602	0,673	1

Hist4h4	0,022634	0,053725	0,044	0,019	1
Wdr5	0,022659	-0,02561	0,106	0,156	1
Rragd	0,022698	0,029544	0,035	0,013	1
Cyb5rl	0,022733	-0,0194	0,039	0,072	1
Bmp2	0,022829	-0,01556	0,042	0,076	1
Gm15853	0,022903	-0,03247	0,044	0,078	1
Fxn	0,022907	-0,02598	0,068	0,11	1
Wdr36	0,022934	-0,03924	0,11	0,16	1
Stim1	0,022935	-0,04331	0,525	0,629	1
Nom1	0,022976	-0,02424	0,218	0,289	1
1700037C:	0,022992	-0,04235	0,127	0,179	1
AC127341.	0,023002	0,045131	0,059	0,03	1
Mfsd8	0,023007	-0,04052	0,226	0,295	1
Prkra	0,023054	-0,04052	0,182	0,243	1
Tedc2	0,023068	-0,02332	0,04	0,074	1
Mtpn	0,023101	-0,016	0,341	0,437	1
Tspo	0,023155	0,068413	0,147	0,101	1
Lrrc4	0,02316	0,071143	0,209	0,156	1
Dtx1	0,023169	0,052895	0,059	0,03	1
Zfp790	0,023256	-0,02694	0,057	0,095	1
Nt5c3b	0,023268	-0,03655	0,248	0,319	1
Tubb6	0,023288	0,024725	0,02	0,004	1
Clptm1	0,023333	0,090873	0,809	0,795	1
Lfng	0,023355	0,028283	0,02	0,004	1
Gm26884	0,023421	0,026506	0,02	0,004	1
Arfrp1	0,023437	-0,02935	0,347	0,435	1
Malat1	0,023443	0,09722	0,998	0,998	1
Spink10	0,023499	-0,02752	0,033	0,063	1
Aco2	0,023533	-0,05571	0,804	0,867	1
Ikzf5	0,023535	-0,00492	0,376	0,492	1
Nlrc3	0,023555	0,019363	0,02	0,004	1
Ptprf	0,023565	0,138183	0,549	0,53	1
Rc3h2	0,02357	0,137324	0,606	0,603	1
Tdrd7	0,023619	-0,04399	0,224	0,291	1
Gm49077	0,023621	0,015772	0,02	0,004	1
Creb1	0,023652	0,129321	0,517	0,498	1
Sec14l2	0,023656	-0,05462	0,178	0,236	1
Ptpn21	0,023706	-0,0318	0,07	0,112	1
Gm26840	0,023745	0,02523	0,031	0,011	1
Hoxaas3	0,023886	-0,02468	0,024	0,051	1
1190002N:	0,023947	-0,02879	0,299	0,382	1
Rad50	0,023967	-0,01064	0,211	0,285	1
Prdx5	0,02398	-0,03182	0,646	0,77	1
Dnm2	0,024011	0,082142	0,901	0,867	1
Herpud1	0,024026	-0,03983	0,426	0,523	1
Plekhh3	0,02406	0,064918	0,083	0,049	1
Arl4c	0,024091	0,053503	0,064	0,034	1
Pi4k2a	0,024141	-0,01771	0,283	0,363	1
Rfng	0,02417	-0,02826	0,152	0,213	1
Prox1	0,024195	0,106774	0,914	0,89	1
Rhog	0,024212	-0,04293	0,982	0,983	1
Fbln7	0,024221	-0,03223	0,026	0,053	1

Tex261	0,024327	-0,05687	0,585	0,673	1
Emp2	0,024345	0,03995	0,024	0,006	1
Rlf	0,024357	-0,01442	0,349	0,445	1
Mpnd	0,024361	-0,04367	0,36	0,456	1
Tial1	0,024422	-0,0513	0,602	0,677	1
Fam184b	0,024438	0,02409	0,028	0,008	1
Ang	0,024448	0,055763	0,059	0,03	1
Lars	0,024467	-0,0225	0,382	0,473	1
Ssr3	0,024504	-0,04476	0,886	0,93	1
Dcbld2	0,024513	0,120026	0,299	0,249	1
Zfp655	0,024578	-0,03207	0,248	0,319	1
Mitd1	0,024608	-0,04734	0,218	0,285	1
Rpusd3	0,024628	-0,03674	0,05	0,084	1
A430033K(	0,024692	-0,03827	0,088	0,133	1
Gm14443	0,024713	-0,02654	0,013	0,034	1
Sec11a	0,024716	-0,01703	0,325	0,418	1
Csrnp2	0,024746	0,039762	0,092	0,055	1
Ddx55	0,024784	-0,02646	0,279	0,357	1
6720489N:	0,024786	-0,03867	0,051	0,086	1
Frzb	0,024818	0,027637	0,024	0,006	1
Usp48	0,024825	-0,15789	0,488	0,563	1
Mb21d2	0,024829	-0,04328	0,106	0,154	1
4930579G:	0,024832	-0,0395	0,101	0,148	1
Ctps	0,02485	-0,06499	0,128	0,184	1
Smc2os	0,024898	-0,02247	0,013	0,034	1
Rnf31	0,024904	0,096161	0,228	0,177	1
Trim62	0,02494	0,050079	0,081	0,046	1
Zdhhc21	0,024941	-0,02921	0,268	0,348	1
Cacul1	0,024956	0,108505	0,407	0,367	1
Slc25a42	0,024971	-0,04705	0,103	0,15	1
Atad3aos	0,025014	-0,03517	0,064	0,103	1
Phb2	0,025027	-0,04139	0,516	0,614	1
Atp6v0b	0,025106	-0,01839	0,993	0,989	1
Kcna4	0,025116	-0,02473	0,013	0,034	1
Usp11	0,025127	-0,03985	0,257	0,329	1
AC158554.	0,025144	0,031087	0,059	0,03	1
Rabggtb	0,025195	-0,06267	0,272	0,335	1
Gm43062	0,025201	0,018748	0,024	0,006	1
1810021B:	0,025268	-0,02135	0,018	0,042	1
Ttc19	0,025292	-0,02517	0,283	0,363	1
Rcan2	0,025295	0,107221	0,943	0,949	1
Cep112	0,025314	0,071245	0,061	0,032	1
Lypd6b	0,025337	-0,02242	0,072	0,114	1
Mast2	0,025426	-0,02938	0,204	0,27	1
Rcan3	0,025464	-0,03236	0,158	0,217	1
Qtrt2	0,025466	-0,04069	0,099	0,146	1
Rps28	0,025468	0,098289	0,987	0,989	1
Terf1	0,025468	-0,02042	0,244	0,323	1
Slc39a6	0,0255	-0,03144	0,218	0,287	1
Nars2	0,02551	-0,03546	0,123	0,175	1
C1qc	0,025524	-0,02292	0,013	0,034	1
Prim1	0,025525	-0,03078	0,182	0,245	1

Mthfsd	0,025537	-0,02545	0,141	0,198	1
Bcl7a	0,025574	-0,01743	0,539	0,665	1
4930570G:	0,025579	-0,02089	0,013	0,034	1
Lrp1b	0,02562	-0,0407	0,908	0,926	1
Dcaf4	0,025653	-0,05149	0,191	0,249	1
Gmip	0,025654	0,038964	0,05	0,023	1
Acbd3	0,02568	-0,02166	0,422	0,523	1
Mkrn1	0,025704	-0,06666	0,76	0,816	1
Pgap2	0,025732	-0,04843	0,53	0,624	1
Cdc7	0,025738	-0,03346	0,064	0,103	1
Afap1	0,025756	0,144946	0,495	0,483	1
Mcph1	0,02577	-0,01886	0,013	0,034	1
Wdr76	0,025774	-0,02098	0,095	0,143	1
Col9a2	0,025807	-0,02605	0,061	0,099	1
Slc35e4	0,025821	-0,05413	0,435	0,53	1
Gars	0,025858	-0,02012	0,411	0,517	1
Tubb3	0,025871	0,109658	0,692	0,652	1
Prdx4	0,025918	-0,02581	0,4	0,502	1
Rnf25	0,025939	-0,03334	0,136	0,19	1
Pcdhga8	0,025973	-0,01822	0,018	0,042	1
G2e3	0,025978	-0,03755	0,237	0,308	1
Chn1	0,025994	0,069478	0,143	0,099	1
Pou6f1	0,026027	0,081332	0,169	0,122	1
Trps1	0,026032	-0,03537	0,433	0,527	1
Stk11	0,026053	0,126415	0,519	0,506	1
Sumf2	0,026069	-0,03013	0,125	0,177	1
Pbrm1	0,026147	0,127242	0,758	0,766	1
Zfp84	0,02615	-0,0218	0,171	0,232	1
Psip1	0,026154	-0,05419	0,916	0,937	1
Etaa1	0,02618	-0,03455	0,156	0,213	1
Gm27029	0,02624	-0,01776	0,018	0,042	1
Camkk2	0,026278	-0,01606	0,026	0,053	1
Spata13	0,026285	-0,03333	0,108	0,158	1
Pdcd2l	0,026304	-0,03697	0,16	0,217	1
Rragb	0,026347	-0,03983	0,13	0,181	1
Gtpbp6	0,026368	-0,03352	0,321	0,407	1
Acbd6	0,026377	-0,035	0,506	0,618	1
Slmap	0,026415	-0,03484	0,226	0,293	1
Aars2	0,026448	-0,03743	0,075	0,116	1
Adss	0,026528	-0,02707	0,231	0,302	1
Fam151b	0,026564	-0,05852	0,165	0,217	1
Tspan31	0,02666	-0,03969	0,611	0,707	1
Tspan5	0,026664	-0,05367	0,743	0,821	1
Slc20a1	0,026679	0,090089	0,191	0,143	1
Gtf3c2	0,026681	0,09936	0,517	0,487	1
Rasip1	0,026698	0,060035	0,095	0,059	1
Foxj1	0,026717	-0,04721	0,13	0,181	1
Rnpc3	0,026742	-0,02204	0,453	0,551	1
Ikbip	0,026792	-0,06297	0,196	0,255	1
Elk4	0,026816	0,124942	0,382	0,34	1
Fbxw16	0,026839	-0,03017	0,009	0,027	1
Cdkn1c	0,026905	0,040307	0,473	0,407	1

Vldlr	0,02693	0,110307	0,868	0,909	1
Sox13	0,02707	-0,02611	0,048	0,082	1
Spg21	0,027071	-0,0345	0,306	0,386	1
Srek1ip1	0,027088	-0,03555	0,156	0,213	1
Anapc15	0,027105	-0,03842	0,128	0,181	1
Gins3	0,027158	-0,03018	0,05	0,084	1
Nmral1	0,02717	-0,08005	0,883	0,905	1
Slx4	0,027176	-0,04293	0,086	0,129	1
CAAA0111(	0,027216	-0,02958	0,231	0,302	1
Anks1	0,027256	-0,01582	0,202	0,274	1
Ttc30b	0,027274	-0,03958	0,086	0,129	1
Stk38	0,027316	-0,04854	0,283	0,357	1
Erich3	0,027354	-0,04694	0,083	0,124	1
Uprt	0,027376	-0,01199	0,006	0,021	1
Selenot	0,027429	-0,04578	0,93	0,937	1
Eif3e	0,027465	-0,06357	0,65	0,698	1
6720483E2	0,027485	-0,01792	0,009	0,027	1
Ly6g6e	0,027543	-0,05768	0,061	0,097	1
Sirt7	0,027545	-0,00466	0,334	0,432	1
Gm4631	0,027577	-0,04018	0,108	0,156	1
Ubr5	0,027587	0,123139	0,583	0,576	1
Erp29	0,027606	-0,0386	0,683	0,757	1
Mypop	0,027664	-0,01283	0,11	0,162	1
Stx17	0,027694	-0,01196	0,343	0,435	1
Fam120aos	0,027733	-0,02223	0,037	0,068	1
Spen	0,027744	0,125529	0,525	0,502	1
2310022B(	0,02775	-0,09094	0,774	0,806	1
Smco4	0,027763	-0,01199	0,006	0,021	1
Mtmr1	0,027764	-0,04571	0,206	0,266	1
Cldn11	0,027765	-0,05864	1	0,998	1
Ehmt1	0,02785	-0,01776	0,327	0,414	1
Zfp30	0,02785	-0,02593	0,114	0,165	1
Zfp407	0,027888	-0,04151	0,152	0,207	1
Eif1ax	0,027891	-0,0299	0,64	0,736	1
5730409E(	0,027892	-0,03219	0,149	0,205	1
Golgb1	0,027912	-0,05455	0,543	0,652	1
Emb	0,027916	-0,01611	0,009	0,027	1
Ildr1	0,027916	-0,01611	0,009	0,027	1
Cwf19l1	0,028022	-0,02139	0,106	0,156	1
Apoo	0,028073	-0,03659	0,165	0,224	1
Fat1	0,028103	-0,0263	0,338	0,426	1
Mospd3	0,028178	-0,04108	0,128	0,179	1
Cep152	0,028226	-0,02442	0,037	0,068	1
Atg4a	0,028238	-0,03494	0,108	0,156	1
Dopey1	0,028245	-0,0186	0,394	0,498	1
Fam160b1	0,02825	-0,03704	0,213	0,278	1
Lgals3	0,028283	0,205024	0,079	0,046	1
Emp1	0,028304	0,146035	0,037	0,015	1
Zfp609	0,028415	-0,01064	0,233	0,306	1
Msl2	0,028436	0,126316	0,396	0,352	1
Zfp426	0,028439	-0,0271	0,16	0,219	1
Ints8	0,028453	-0,03163	0,145	0,2	1

Pgd	0,028487	-0,04075	0,226	0,291	1
Htra2	0,028528	-0,03079	0,141	0,196	1
Szrd1	0,028548	-0,01312	0,338	0,426	1
Fastkd2	0,028576	-0,03404	0,099	0,146	1
Dus1l	0,028616	-0,03623	0,174	0,234	1
Arrdc3	0,028642	0,115983	0,943	0,937	1
Ube4a	0,028676	-0,02919	0,394	0,489	1
Hcfc2	0,028715	-0,03614	0,204	0,268	1
Snx12	0,028737	-0,03015	0,202	0,268	1
Pbld1	0,028825	0,030191	0,037	0,015	1
Trpt1	0,028836	-0,03279	0,114	0,162	1
Nup37	0,028838	-0,01978	0,02	0,044	1
Ltv1	0,028854	-0,03979	0,231	0,295	1
Prps2	0,028891	-0,04728	0,327	0,405	1
Dennd4b	0,029003	0,119253	0,266	0,222	1
Gpr27	0,029021	0,031943	0,037	0,015	1
Fchsd2	0,029025	0,035917	0,061	0,032	1
Acadm	0,029084	-0,01949	0,319	0,409	1
Slc12a8	0,029093	-0,02676	0,015	0,036	1
Nhp2	0,02912	-0,03129	0,286	0,363	1
Rnf40	0,029137	-0,03957	0,174	0,232	1
Slc9a9	0,029252	-0,04649	0,178	0,234	1
Mapk8ip3	0,029279	0,095303	0,743	0,73	1
Pinx1	0,029286	-0,04838	0,121	0,169	1
Gm15050	0,029301	-0,03461	0,031	0,059	1
Gm26610	0,029327	-0,02066	0,015	0,036	1
Tspoap1	0,029327	-0,02066	0,015	0,036	1
Cd55	0,029362	-0,02292	0,015	0,036	1
Hspd1	0,02938	-0,05187	0,848	0,89	1
Micu2	0,029392	-0,0218	0,444	0,568	1
Phf20	0,029499	0,107135	0,701	0,684	1
Ppard	0,029515	-0,0379	0,213	0,274	1
Brca2	0,029656	-0,04495	0,062	0,099	1
Bud13	0,029702	-0,0248	0,053	0,089	1
Armc6	0,029776	-0,03899	0,121	0,171	1
Cxxc1	0,029805	-0,03943	0,286	0,361	1
Utp14a	0,02986	-0,02097	0,262	0,342	1
Bbs9	0,029908	-0,02487	0,092	0,137	1
Cks1b	0,02991	-0,03481	0,031	0,059	1
Chd6	0,029945	0,107914	0,719	0,709	1
Tbc1d8b	0,029964	-0,02856	0,04	0,072	1
Cdc5l	0,029995	-0,03639	0,576	0,681	1
Ogfod3	0,030085	-0,04308	0,176	0,234	1
Lancl2	0,030098	-0,06117	0,185	0,241	1
Zfp966	0,030141	-0,01729	0,015	0,036	1
Cds2	0,030261	0,094836	0,738	0,711	1
Brms1l	0,030285	-0,0205	0,286	0,367	1
Drg1	0,03033	-0,03651	0,45	0,553	1
Ncdn	0,030378	-0,0414	0,521	0,637	1
Atrip	0,030435	-0,02237	0,145	0,2	1
Spa17	0,030493	-0,03636	0,044	0,076	1
Hdhd5	0,030502	-0,03366	0,086	0,129	1

Map3k13	0,030539	-0,02658	0,077	0,118	1
Tpst2	0,03067	-0,03696	0,202	0,264	1
Trib1	0,030694	0,114268	0,105	0,068	1
Cyp2d22	0,030729	0,081989	0,099	0,063	1
Gipr	0,030735	-0,03474	0,103	0,15	1
Pdhb	0,030741	-0,02263	0,523	0,633	1
March8	0,030788	-0,06301	0,851	0,903	1
Tmem267	0,030824	-0,02658	0,083	0,124	1
Rxrb	0,03088	-0,03666	0,424	0,515	1
Plin3	0,030954	-0,251	0,719	0,736	1
Rchy1	0,030967	-0,04073	0,572	0,679	1
Insm1	0,030972	-0,01215	0,015	0,036	1
Ifrd2	0,031001	-0,03293	0,088	0,131	1
Atp6v1a	0,031033	0,065611	0,866	0,814	1
Ankhd1	0,031056	0,138016	0,517	0,523	1
Herc2	0,031062	-0,05778	0,633	0,724	1
Trmo	0,03107	-0,02866	0,059	0,095	1
Gne	0,031136	-0,02451	0,139	0,192	1
Pigm	0,031168	-0,02275	0,145	0,2	1
5031425E2	0,031199	-0,02996	0,237	0,306	1
Otub1	0,031213	-0,04474	0,565	0,654	1
Mbip	0,03135	-0,044	0,29	0,365	1
Paqr8	0,031359	-0,08151	0,514	0,601	1
Pald1	0,031394	0,065284	0,172	0,127	1
Psrc1	0,031423	0,039265	0,033	0,013	1
Ginm1	0,031473	-0,0051	0,262	0,34	1
Usp1	0,031525	-0,02318	0,312	0,397	1
Ofd1	0,031579	-0,029	0,127	0,179	1
Ivd	0,031637	-0,04277	0,321	0,399	1
Mcm8	0,03165	-0,02954	0,077	0,118	1
Adcy5	0,0317	0,103579	0,615	0,595	1
Emc8	0,031773	-0,03432	0,58	0,696	1
Id4	0,031805	0,009542	0,086	0,131	1
Igfbp2	0,031814	-0,0084	0	0,008	1
Resp18	0,031814	-0,0084	0	0,008	1
4930447M	0,031814	-0,0084	0	0,008	1
Ckmt1	0,031814	-0,0084	0	0,008	1
Ctxn2	0,031814	-0,0084	0	0,008	1
Bpifb4	0,031814	-0,0084	0	0,008	1
Pltp	0,031814	-0,0084	0	0,008	1
Btk	0,031814	-0,0084	0	0,008	1
Scml2	0,031814	-0,0084	0	0,008	1
1110032F0	0,031814	-0,0084	0	0,008	1
Gm42812	0,031814	-0,0084	0	0,008	1
Gm43240	0,031814	-0,0084	0	0,008	1
Hes3	0,031814	-0,0084	0	0,008	1
Adcyap1r1	0,031814	-0,0084	0	0,008	1
Oscar	0,031814	-0,0084	0	0,008	1
Pglyrp1	0,031814	-0,0084	0	0,008	1
Axl	0,031814	-0,0084	0	0,008	1
Upk1a	0,031814	-0,0084	0	0,008	1
Gm17102	0,031814	-0,0084	0	0,008	1

Olfr691	0,031814	-0,0084	0	0,008	1
Cpxm2	0,031814	-0,0084	0	0,008	1
Stk32c	0,031814	-0,0084	0	0,008	1
Gm31793	0,031814	-0,0084	0	0,008	1
Zfp791	0,031814	-0,0084	0	0,008	1
Gm45639	0,031814	-0,0084	0	0,008	1
Calb2	0,031814	-0,0084	0	0,008	1
Pstpip1	0,031814	-0,0084	0	0,008	1
Gm36278	0,031814	-0,0084	0	0,008	1
Arpp21	0,031814	-0,0084	0	0,008	1
Vstm2a	0,031814	-0,0084	0	0,008	1
Rab11fip4c	0,031814	-0,0084	0	0,008	1
Zfp652os	0,031814	-0,0084	0	0,008	1
Hoxb6	0,031814	-0,0084	0	0,008	1
Igfbp4	0,031814	-0,0084	0	0,008	1
Hist1h3e	0,031814	-0,0084	0	0,008	1
Gm2762	0,031814	-0,0084	0	0,008	1
Cartpt	0,031814	-0,0084	0	0,008	1
AC133868.	0,031814	-0,0084	0	0,008	1
Rit2	0,031814	-0,0084	0	0,008	1
Fads2	0,031814	-0,0084	0	0,008	1
Bdnf	0,031814	-0,01049	0	0,008	1
Slc32a1	0,031814	-0,01049	0	0,008	1
Ankrd6	0,031814	-0,01049	0	0,008	1
Slc6a11	0,031814	-0,01049	0	0,008	1
Pla2g4c	0,031814	-0,01049	0	0,008	1
Cdiptos	0,031814	-0,01049	0	0,008	1
Cnmd	0,031814	-0,01049	0	0,008	1
Gm48393	0,031814	-0,01049	0	0,008	1
Gria1	0,031814	-0,01049	0	0,008	1
Retn	0,031814	-0,01258	0	0,008	1
Gm15938	0,031814	-0,01258	0	0,008	1
Svep1	0,031814	-0,01258	0	0,008	1
Map2k7	0,031841	-0,02218	0,459	0,572	1
Gm32051	0,031878	0,055797	0,033	0,013	1
C530008M	0,031949	-0,07158	0,193	0,251	1
Sarnp	0,031966	-0,04629	0,561	0,654	1
Dph6	0,031972	-0,03595	0,132	0,184	1
Amt	0,032131	-0,03384	0,061	0,097	1
Rap1b	0,032155	-0,02594	0,519	0,624	1
Cc2d2a	0,03217	-0,03044	0,171	0,228	1
Spop	0,032193	0,082756	0,802	0,743	1
Lipo3	0,032277	-0,01962	0,042	0,074	1
Paxbp1	0,032278	-0,02065	0,332	0,416	1
Gzf1	0,032281	-0,01865	0,171	0,23	1
Setd2	0,032295	0,134858	0,578	0,551	1
Gps2	0,032308	-0,03109	0,439	0,536	1
C330011M	0,032314	0,025044	0,015	0,002	1
Ppp2r1a	0,032328	-0,07278	0,844	0,865	1
Cav2	0,032355	-0,10251	0,182	0,232	1
Kif7	0,032362	0,028435	0,033	0,013	1
Gm31763	0,032367	0,019672	0,015	0,002	1

Sertad4	0,032367	0,028609	0,015	0,002	1
4933404O1	0,032368	-0,0218	0,072	0,112	1
Plpp7	0,032382	-0,03334	0,143	0,196	1
5730480H0	0,032397	-0,03245	0,125	0,175	1
Gm15582	0,03242	0,023256	0,015	0,002	1
Gm43738	0,032473	0,016075	0,015	0,002	1
Mettl27	0,032473	0,016075	0,015	0,002	1
Klf5	0,032473	0,016075	0,015	0,002	1
Zfp473	0,032473	0,026828	0,015	0,002	1
Ctla2a	0,032473	0,023256	0,015	0,002	1
St14	0,032473	0,017875	0,015	0,002	1
Ogg1	0,032499	-0,03514	0,095	0,139	1
Gm7276	0,032526	0,014271	0,015	0,002	1
Trrap	0,032544	0,086052	0,424	0,363	1
Recql	0,032706	-0,03796	0,161	0,217	1
Hcfc1r1	0,032751	-0,04517	0,776	0,848	1
Thumpd2	0,032764	-0,0218	0,031	0,059	1
Ccsap	0,03282	-0,03136	0,064	0,101	1
Pum1	0,032835	0,105082	0,782	0,766	1
Rep15	0,032865	0,036246	0,057	0,03	1
Rbbp6	0,032886	0,153322	0,703	0,686	1
Krtcap2	0,032921	-0,04373	0,796	0,85	1
Uqcrc1	0,032923	-0,04211	0,793	0,842	1
Kdm1a	0,032926	-0,03271	0,457	0,559	1
Gsta4	0,032931	-0,02134	0,004	0,017	1
Gm16240	0,032935	0,041023	0,042	0,019	1
Ahnak2	0,032957	0,02138	0,015	0,002	1
Cebpb	0,032958	0,07288	0,217	0,165	1
Sik1	0,032971	0,035917	0,057	0,03	1
B3gnt4	0,033033	-0,01307	0,004	0,017	1
Dyx1c1	0,033033	-0,01307	0,004	0,017	1
AU019823	0,033079	-0,04603	0,253	0,321	1
Mtpap	0,033115	-0,04356	0,24	0,306	1
Gm26843	0,033207	-0,02292	0,017	0,038	1
Mbd4	0,033218	-0,02369	0,059	0,095	1
Atf6b	0,03327	-0,02209	0,363	0,456	1
Adnp	0,033309	-0,02942	0,09	0,133	1
Tmem17	0,033334	-0,03384	0,066	0,103	1
Traf3ip2	0,033344	-0,01332	0,004	0,017	1
Usp20	0,033384	-0,04067	0,178	0,236	1
Tdo2	0,033409	-0,0218	0,031	0,059	1
Insc	0,033434	0,122671	0,844	0,859	1
Gm26668	0,033447	-0,01125	0,004	0,017	1
Rictor	0,033462	0,122114	0,58	0,57	1
Samd8	0,033467	0,099702	0,769	0,755	1
Fntb	0,033477	-0,09068	0,339	0,407	1
Ddx23	0,033492	-0,01459	0,246	0,323	1
Kctd2	0,033507	-0,06006	0,583	0,66	1
Ift52	0,033564	-0,01326	0,194	0,262	1
Gm17705	0,033582	-0,01816	0,011	0,03	1
Cutc	0,033664	-0,03136	0,064	0,101	1
Eef1g	0,033714	0,106542	0,888	0,869	1

Rpl11	0,033717	-0,023	0,994	0,989	1
Epha4	0,033738	-0,03224	0,213	0,278	1
Nup98	0,033774	0,140174	0,446	0,416	1
Rnf38	0,033798	0,120836	0,328	0,289	1
Tmem39a	0,033831	-0,03343	0,211	0,276	1
Tigd3	0,033866	-0,00942	0,004	0,017	1
4930513D1	0,033866	-0,00397	0,004	0,017	1
Sowaha	0,033871	-0,04288	0,25	0,319	1
Mbd1	0,033885	-0,04187	0,226	0,289	1
Pi4k2b	0,033939	-0,02039	0,103	0,15	1
Plekhg2	0,034012	0,058089	0,048	0,023	1
Twf2	0,034084	-0,04008	0,24	0,302	1
Mcm4	0,034091	-0,02923	0,178	0,238	1
Wdr95	0,034106	-0,01635	0,011	0,03	1
B230317F2	0,034185	-0,01863	0,011	0,03	1
Vapb	0,034194	-0,03134	0,538	0,65	1
B4galt6	0,034206	-0,04771	0,125	0,173	1
Tmem196	0,034276	-0,02359	0,017	0,038	1
Prkaca	0,034326	-0,04724	0,516	0,61	1
Stard7	0,034345	-0,01929	0,402	0,504	1
Golga3	0,034401	-0,01892	0,284	0,363	1
Rcor3	0,034425	0,131465	0,514	0,5	1
Rpl28	0,034458	-0,03668	1	0,998	1
4932422M	0,034461	-0,00411	0,051	0,086	1
Atp6v0e2	0,034566	-0,0554	0,864	0,876	1
Rab14	0,034643	-0,03196	0,989	0,987	1
Aspscr1	0,034648	-0,03422	0,371	0,451	1
Capza2	0,034698	-0,03876	0,828	0,88	1
Gm15545	0,034701	-0,02246	0,024	0,049	1
Kcnk2	0,034719	0,03544	0,029	0,011	1
Baz1b	0,03478	0,125409	0,662	0,66	1
Zbtb24	0,034787	-0,03458	0,117	0,165	1
Zfp597	0,034833	-0,02319	0,11	0,158	1
Slc25a44	0,034916	-0,02253	0,341	0,426	1
Zmynd8	0,034936	-0,05906	0,716	0,793	1
Nsd1	0,034955	0,089201	0,69	0,688	1
Homer3	0,034972	-0,03332	0,268	0,338	1
Pdcl	0,035017	-0,03494	0,286	0,359	1
Metap1d	0,035035	-0,05123	0,382	0,464	1
Brox	0,035038	0,095442	0,481	0,454	1
Unc79	0,035174	-0,01273	0,011	0,03	1
Rpl26	0,03521	0,095549	0,994	0,996	1
Lpcat1	0,035225	0,077166	0,154	0,112	1
Tamm41	0,035287	-0,02835	0,07	0,108	1
Zeb2os	0,035332	-0,038	0,211	0,272	1
Exoc7	0,035388	-0,03559	0,411	0,506	1
Gm7467	0,03544	-0,02645	0,062	0,099	1
Ccdc134	0,035454	-0,02743	0,132	0,184	1
Ccdc148	0,035484	0,090145	0,117	0,08	1
Atg5	0,035487	-0,02762	0,237	0,304	1
Tfap4	0,035504	-0,031	0,114	0,16	1
Srrt	0,035509	-0,02208	0,38	0,475	1

Rps6kl1	0,035659	-0,02671	0,094	0,137	1
Hdac10	0,035666	-0,03583	0,114	0,16	1
Heatr6	0,035723	-0,01139	0,259	0,335	1
Syngr3	0,035744	-0,01868	0,024	0,049	1
Btbd3	0,035818	-0,04022	0,319	0,395	1
Sfpq	0,03585	0,110098	0,965	0,954	1
Abl2	0,035861	0,10004	0,275	0,224	1
Mettl5os	0,035879	-0,01769	0,007	0,023	1
Gm21671	0,035879	-0,01769	0,007	0,023	1
Gm21814	0,035879	-0,01769	0,007	0,023	1
Fam169a	0,035879	-0,01974	0,007	0,023	1
Ik	0,036068	-0,03893	0,756	0,827	1
Klf2	0,036082	0,003913	0,029	0,011	1
Appl1	0,036094	0,10172	0,688	0,681	1
Slc35c1	0,036155	-0,02455	0,064	0,101	1
Kbtbd8	0,03617	-0,02266	0,048	0,08	1
Bhlhb9	0,036198	-0,01792	0,007	0,023	1
Cd274	0,036208	0,057281	0,026	0,008	1
Tmem25	0,036246	-0,02805	0,051	0,084	1
Abcc5	0,036308	-0,02589	0,694	0,789	1
Ccdc71	0,036315	-0,03076	0,103	0,148	1
Zufsp	0,036331	-0,02298	0,235	0,302	1
Ggnbp1	0,036492	-0,02592	0,088	0,131	1
Epop	0,03654	0,025865	0,026	0,008	1
Hist1h3d	0,036562	-0,01381	0,007	0,023	1
Zfp41	0,036606	-0,04488	0,154	0,207	1
Gm37756	0,036747	0,009132	0,009	0	1
Gssos2	0,036747	0,009132	0,009	0	1
Cfap46	0,036747	0,009132	0,009	0	1
Gm31805	0,036747	0,009132	0,009	0	1
Gramd2	0,036747	0,009132	0,009	0	1
Gm34923	0,036747	0,009132	0,009	0	1
Ckap4	0,036747	0,012762	0,009	0	1
1110017D	0,036747	0,010949	0,009	0	1
Ticrr	0,036747	0,010949	0,009	0	1
Utf1	0,036747	0,010949	0,009	0	1
Tshr	0,036747	0,010949	0,009	0	1
Msh5	0,036747	0,010949	0,009	0	1
Hrasls5	0,036747	0,010949	0,009	0	1
Gm28609	0,036748	0,012762	0,009	0	1
Hvcn1	0,036748	0,012762	0,009	0	1
Vmn1r43	0,036748	0,012762	0,009	0	1
Ephb3	0,036748	0,012762	0,009	0	1
Gm47316	0,036748	0,014572	0,009	0	1
Nanos1	0,036748	0,016379	0,009	0	1
Gfra2	0,036748	0,021779	0,009	0	1
Parp14	0,036748	0,028935	0,009	0	1
4930589L	0,036768	0,031829	0,018	0,004	1
Mettl9	0,036768	-0,03807	0,549	0,65	1
Gmppb	0,036769	-0,02826	0,112	0,158	1
Brd3os	0,036772	-0,02003	0,035	0,063	1
Fancc	0,036784	-0,04232	0,182	0,236	1

Acvr2b	0,036812	-0,03551	0,204	0,264	1
Unc45a	0,036899	0,090129	0,233	0,188	1
Zbed5	0,036908	-0,02351	0,051	0,084	1
Tle1	0,036923	-0,02299	0,481	0,586	1
Ssr1	0,036957	0,090308	0,965	0,939	1
Itga9	0,036969	-0,0149	0,002	0,013	1
Gm597	0,036969	-0,01698	0,002	0,013	1
Gm16033	0,036973	0,017569	0,018	0,004	1
Galnt13	0,037036	-0,01283	0,002	0,013	1
Gm19265	0,037036	-0,01283	0,002	0,013	1
Pdzrn3	0,037036	-0,01283	0,002	0,013	1
Zpbp	0,037036	-0,01283	0,002	0,013	1
Gm26620	0,037036	-0,0149	0,002	0,013	1
Pgm2l1	0,037047	0,077196	0,09	0,057	1
Lnx2	0,037074	0,033264	0,022	0,006	1
R3hdm1	0,037101	-0,06652	0,774	0,835	1
3110079O	0,037103	-0,01075	0,002	0,013	1
Gm13373	0,037103	-0,01075	0,002	0,013	1
Cp	0,037103	-0,01075	0,002	0,013	1
Kcnd2	0,037103	-0,01075	0,002	0,013	1
Gm21284	0,037103	-0,01075	0,002	0,013	1
Trhde	0,037103	-0,01075	0,002	0,013	1
Gm10647	0,037103	-0,01075	0,002	0,013	1
Ahrr	0,037103	-0,01075	0,002	0,013	1
Vcpip1	0,037157	-0,03554	0,341	0,416	1
Olfr259	0,037179	0,013972	0,018	0,004	1
Pcmtd2	0,037189	0,014195	0,345	0,456	1
Hist2h3c2	0,037199	-0,02315	0,018	0,04	1
Prdm4	0,037204	-0,02566	0,105	0,15	1
Lrp4	0,037219	0,050974	0,235	0,179	1
Tyk2	0,03723	-0,02883	0,105	0,15	1
Jmjd6	0,037325	-0,01605	0,389	0,489	1
Ptn	0,037332	0,074283	0,98	0,947	1
Rbm33	0,037407	-0,01596	0,352	0,437	1
Rnf126	0,037438	-0,02843	0,332	0,409	1
Ttll9	0,037444	-0,011	0,002	0,013	1
Capn7	0,037516	-0,04299	0,222	0,285	1
Pde7a	0,037523	-0,0501	0,207	0,264	1
Zfr	0,037557	0,102294	0,85	0,825	1
4930403P	0,037633	0,022626	0,022	0,006	1
Gm14698	0,037656	0,039002	0,064	0,036	1
Sowahc	0,037736	0,089304	0,202	0,158	1
Prpf38a	0,037801	-0,03125	0,279	0,35	1
Zscan21	0,037814	-0,02883	0,11	0,156	1
Lyrm7	0,037861	-0,02026	0,035	0,063	1
Casp7	0,037915	0,019055	0,022	0,006	1
Hspbap1	0,037977	-0,03309	0,077	0,116	1
Rsf1	0,038005	0,103774	0,76	0,738	1
Arid1b	0,038024	0,120597	0,473	0,454	1
Pfas	0,038089	-0,03672	0,174	0,23	1
Rps27	0,038124	-0,01825	0,996	0,996	1
Rnase1	0,03818	0,009489	0,018	0,004	1

Prpf38b	0,03832	0,115641	0,657	0,639	1
Dhx36	0,038323	-0,04548	0,734	0,821	1
Ddx39	0,038339	-0,03002	0,141	0,192	1
Eps8	0,038445	-0,02857	0,013	0,032	1
Aff4	0,038496	0,118614	0,706	0,738	1
Spns2	0,038525	-0,01208	0,538	0,652	1
Utp6	0,038588	-0,02779	0,248	0,314	1
Rpia	0,038589	-0,00868	0,255	0,335	1
Znhit6	0,038591	-0,02057	0,24	0,306	1
Dgcr8	0,038601	-0,0349	0,16	0,213	1
Fgd6	0,038628	-0,02476	0,068	0,105	1
Gm21781	0,038671	-0,01713	0,026	0,051	1
Acad10	0,038722	-0,02451	0,013	0,032	1
Gm16861	0,038722	-0,02451	0,013	0,032	1
Rinl	0,038742	0,076216	0,117	0,08	1
Zbtb45	0,038785	-0,03661	0,094	0,135	1
B3galt1	0,038808	-0,03587	0,169	0,224	1
Fbrsl1	0,038874	-0,04025	0,218	0,276	1
Anp32a	0,038922	-0,03617	0,717	0,793	1
Rab11a	0,038955	-0,0524	0,582	0,656	1
Grk4	0,038997	-0,02865	0,073	0,112	1
Gfm2	0,039	-0,03916	0,125	0,171	1
Ankib1	0,039016	0,076517	0,811	0,781	1
Tspyl4	0,039047	-0,05782	0,679	0,755	1
Msl1	0,039248	0,123654	0,499	0,485	1
Eef2kmt	0,039248	-0,02375	0,134	0,184	1
Huwe1	0,039267	0,093825	0,831	0,804	1
Srsf1	0,039276	0,104431	0,635	0,624	1
Abi2	0,039396	0,122954	0,594	0,593	1
4930503L1	0,039533	-0,03709	0,163	0,217	1
Fam212a	0,03955	-0,02068	0,028	0,053	1
Mef2b	0,039568	-0,02878	0,013	0,032	1
5730455P1	0,039655	-0,01555	0,411	0,513	1
Dgki	0,039695	-0,04762	0,266	0,331	1
Atat1	0,03972	-0,0435	0,578	0,656	1
Tasp1	0,039729	-0,02114	0,039	0,068	1
Sclt1	0,039764	-0,0198	0,185	0,245	1
Fsd1l	0,039805	-0,01883	0,517	0,614	1
Srp72	0,039831	-0,03042	0,503	0,599	1
Stk10	0,039965	-0,0218	0,072	0,11	1
Fbxo25	0,040073	-0,0263	0,327	0,401	1
Taf8	0,040108	-0,02733	0,132	0,181	1
Cct7	0,040142	-0,05958	0,901	0,924	1
Dolk	0,040167	-0,02372	0,24	0,306	1
Gm26670	0,040178	-0,01658	0,013	0,032	1
Fam205a1	0,040178	-0,01658	0,013	0,032	1
Lrrc40	0,040193	-0,03532	0,099	0,141	1
Gm47689	0,040205	-0,01037	0,018	0,04	1
Abcb9	0,040235	-0,02855	0,097	0,139	1
Gm47059	0,04039	-0,01525	0,013	0,032	1
Aarsd1	0,040417	-0,03108	0,092	0,133	1
Lrba	0,04044	0,101202	0,215	0,173	1

Calcoco1	0,040448	-0,02011	0,338	0,424	1
Banf2os	0,040523	-0,0376	0,02	0,042	1
Entpd6	0,04054	-0,02845	0,057	0,091	1
A930015D(	0,040554	-0,01322	0,013	0,032	1
Pdap1	0,040638	-0,03576	0,87	0,903	1
Mxd4	0,04064	0,10372	0,818	0,81	1
Upf1	0,04064	-0,03071	0,428	0,525	1
Nxpe4	0,0407	-0,03495	0,088	0,129	1
Taf3	0,040703	-0,03106	0,339	0,42	1
Gnl1	0,040743	-0,01859	0,209	0,272	1
Txnrd1	0,040755	-0,01103	0,336	0,422	1
Msh3	0,040772	-0,02053	0,29	0,363	1
Zfand2b	0,040779	-0,02874	0,288	0,357	1
Tmem42	0,040791	-0,05161	0,182	0,234	1
Mmgt2	0,040794	0,037575	0,044	0,021	1
Lhpp	0,04088	-0,02369	0,072	0,11	1
Gm10277	0,040891	-0,03031	0,042	0,072	1
Gm45768	0,040983	0,132205	0,525	0,515	1
Larp1b	0,041072	-0,0527	0,217	0,272	1
Ell	0,041108	-0,02746	0,167	0,222	1
Dynlt1f	0,041113	-0,03247	0,042	0,072	1
Smurf2	0,041191	-0,02148	0,283	0,357	1
Ogdh	0,041194	0,096865	0,657	0,66	1
Prkag1	0,041234	-0,03618	0,336	0,416	1
4930480K2	0,041271	-0,0231	0,04	0,07	1
Dclk2	0,041294	0,057201	0,117	0,08	1
4833439L1	0,041402	-0,05961	0,635	0,7	1
Irf3	0,041456	-0,00994	0,308	0,399	1
Trim47	0,041561	0,053396	0,072	0,042	1
Tceal3	0,041569	0,001909	0,145	0,2	1
Iqce	0,041668	-0,03221	0,097	0,139	1
Slc35e2	0,04168	-0,02216	0,191	0,251	1
Zfp763	0,041689	-0,02304	0,079	0,118	1
Vangl1	0,041692	-0,02116	0,053	0,086	1
Ubqln2	0,041738	-0,0391	0,695	0,778	1
Ciart	0,041772	-0,01972	0,079	0,118	1
Fktn	0,041817	-0,04741	0,716	0,77	1
Ppm1d	0,04182	-0,01207	0,224	0,289	1
Plpp5	0,041921	-0,0249	0,029	0,055	1
Ppl	0,041956	0,05742	0,061	0,034	1
Sema5a	0,04196	0,191179	0,31	0,266	1
Tomm70a	0,042222	-0,01216	0,382	0,485	1
Mfsd4a	0,042322	-0,03762	0,114	0,158	1
A830019P(	0,042372	0,027794	0,035	0,015	1
Aph1a	0,04251	-0,04233	0,65	0,759	1
Rnaseh2a	0,042521	-0,02756	0,178	0,236	1
Gm14412	0,042554	-0,01759	0,028	0,053	1
Map3k10	0,042624	-0,04217	0,185	0,238	1
Zfp131	0,042651	-0,01087	0,328	0,418	1
Ak6	0,042658	-0,0197	0,22	0,283	1
Gm30122	0,04272	-0,03106	0,031	0,057	1
Med13	0,042768	0,111968	0,622	0,601	1

Ddx51	0,042926	-0,02755	0,061	0,095	1
Nfe2l1	0,042991	0,093044	0,558	0,549	1
Depdc5	0,042998	-0,02662	0,262	0,333	1
Amigo1	0,043043	-0,02638	0,083	0,122	1
BC048403	0,043077	-0,02289	0,042	0,072	1
Fam50a	0,043182	-0,01519	0,35	0,437	1
Mtor	0,043228	-0,02737	0,24	0,304	1
Flii	0,043265	-0,03272	0,45	0,557	1
Tango6	0,043308	-0,02506	0,042	0,072	1
Vps37c	0,043452	-0,02559	0,253	0,321	1
2310035C:	0,04347	-0,02166	0,437	0,54	1
Strada	0,043477	-0,03492	0,139	0,188	1
Soga1	0,043582	-0,04253	0,207	0,264	1
Hsp90b1	0,043653	-0,04528	0,991	0,994	1
Dnajc7	0,04371	-0,03973	0,723	0,819	1
Smarcad1	0,043716	-0,00893	0,29	0,373	1
Gm32036	0,043749	-0,02777	0,09	0,131	1
Guf1	0,043773	-0,05271	0,152	0,2	1
Gm37494	0,043776	-0,04746	0,464	0,561	1
Ints14	0,043887	-0,02101	0,128	0,177	1
Lonrf1	0,043909	-0,02927	0,266	0,333	1
D730003I1	0,043941	-0,03169	0,209	0,268	1
Cstf1	0,043996	-0,00628	0,105	0,148	1
Trmu	0,044218	-0,03295	0,112	0,156	1
Gm26737	0,044234	-0,05059	0,009	0,025	1
Ythdf1	0,044258	-0,02678	0,35	0,432	1
Gm38394	0,04434	0,036968	0,061	0,034	1
BC051077	0,044362	0,054459	0,073	0,044	1
Rps23	0,044364	-0,01199	0,991	0,996	1
Gm6563	0,04439	-0,01239	0,02	0,042	1
mt-Atp6	0,044442	-0,03807	1	1	1
Kctd7	0,044461	-0,01906	0,193	0,253	1
Slc35c2	0,044587	-0,02311	0,169	0,224	1
Usp53	0,044636	0,111736	0,268	0,224	1
Klhl40	0,0447	0,05139	0,079	0,049	1
Luc7l2	0,044734	0,062269	0,963	0,951	1
Rsph9	0,044784	0,052441	0,073	0,044	1
Ntm	0,044792	-0,02021	0,009	0,025	1
Fbxl15	0,044808	-0,02487	0,095	0,137	1
Itpr1	0,044838	-0,03151	0,046	0,076	1
Zfp712	0,044923	-0,02332	0,044	0,074	1
Epc1	0,044931	0,111857	0,58	0,58	1
Kirrel2	0,044941	-0,01587	0,009	0,025	1
Rsph3b	0,044941	-0,01587	0,009	0,025	1
2900011O(	0,044994	-0,02066	0,015	0,034	1
Podxl2	0,045001	0,045015	0,095	0,061	1
Zfp704	0,045092	0,118153	0,583	0,574	1
Pcdh9	0,045135	-0,05872	0,993	0,998	1
Ddx20	0,045139	-0,03738	0,081	0,118	1
Cdca4	0,045157	-0,00883	0,02	0,042	1
E330034G:	0,045253	-0,01816	0,009	0,025	1
Atxn1l	0,045314	-0,02921	0,2	0,259	1

Gm45051	0,045543	-0,02	0,022	0,044	1
Aqr	0,045554	-0,01392	0,308	0,39	1
Gm6598	0,045563	-0,01405	0,009	0,025	1
Spout1	0,045574	-0,03612	0,233	0,293	1
Thoc6	0,045582	-0,01729	0,2	0,257	1
Anapc2	0,045622	0,120328	0,62	0,618	1
Srpk1	0,045628	-0,02659	0,334	0,407	1
Eif3f	0,045639	-0,03356	0,833	0,888	1
Ddx10	0,045647	-0,01748	0,196	0,257	1
Ccz1	0,045716	0,029044	0,488	0,582	1
Samd4	0,045725	-0,0322	0,345	0,416	1
Ccar2	0,045729	-0,03438	0,224	0,287	1
Dpp9	0,045734	-0,04025	0,215	0,272	1
Ehbp1	0,045757	0,076397	0,2	0,156	1
St3gal5	0,04583	-0,03251	0,4	0,485	1
Bace2	0,045999	-0,0607	0,123	0,167	1
Ehbp1l1	0,046008	0,028757	0,031	0,013	1
Naif1	0,046047	-0,01682	0,015	0,034	1
Gm32926	0,046047	-0,01682	0,015	0,034	1
Tnfrsf21	0,046053	-0,01909	0,015	0,034	1
Dnaja1	0,046128	0,02033	0,958	0,958	1
Faah	0,046203	-0,07051	0,305	0,373	1
Zfp362	0,046211	-0,03032	0,121	0,167	1
Rybp	0,046273	-0,0509	0,523	0,603	1
Kifc5b	0,046278	0,023461	0,031	0,013	1
Tmem260	0,046298	-0,02373	0,328	0,407	1
Gm5602	0,0463	-0,01454	0,009	0,025	1
Pwwp2a	0,046319	-0,04122	0,374	0,447	1
Mafk	0,046322	0,10586	0,183	0,143	1
Gfpt1	0,046331	-0,01833	0,358	0,447	1
Tyms	0,046407	-0,01705	0,015	0,034	1
Tbc1d14	0,046423	-0,04483	0,631	0,747	1
3110001I2	0,046525	0,030321	0,04	0,019	1
Dsn1	0,046577	-0,01539	0,006	0,019	1
Xrra1	0,046577	-0,01539	0,006	0,019	1
Atp6ap1	0,046629	0,061435	0,98	0,96	1
Rbm3os	0,046764	-0,01332	0,006	0,019	1
Gm13205	0,046764	-0,01332	0,006	0,019	1
Lrrc46	0,046764	-0,01332	0,006	0,019	1
2810049E(	0,046764	-0,01332	0,006	0,019	1
Gm17111	0,046764	-0,01332	0,006	0,019	1
Txn1	0,046839	0,087479	0,683	0,658	1
Rtn4ip1	0,046903	-0,02393	0,064	0,099	1
Asxl3	0,046912	0,012905	0,27	0,348	1
Vhl	0,046947	-0,02786	0,25	0,312	1
Rbm38	0,046967	0,006653	0,031	0,013	1
Ccdc24	0,047058	-0,02396	0,046	0,076	1
1700001O:	0,047146	-0,01356	0,006	0,019	1
Rpl37a	0,047155	-0,01727	0,998	0,994	1
Syne1	0,047188	-0,0125	0,138	0,19	1
Galnt17	0,04721	-0,01759	0,031	0,057	1
Tmem120a	0,047235	-0,01637	0,141	0,192	1

Atad2	0,04729	0,047212	0,11	0,074	1
Shpk	0,047336	-0,0115	0,006	0,019	1
Pgf	0,047336	-0,0115	0,006	0,019	1
Dgke	0,047339	-0,03195	0,134	0,181	1
Cnnm2	0,047508	-0,02522	0,112	0,156	1
Nkrf	0,047622	-0,01543	0,059	0,093	1
Utp15	0,047623	-0,02908	0,156	0,207	1
Klhdc8a	0,047667	0,157762	0,29	0,253	1
Rplp1	0,04782	-0,01825	1	0,998	1
Fkbpl	0,047837	-0,02625	0,07	0,105	1
Mllt6	0,047969	0,133858	0,453	0,449	1
Eya3	0,04801	-0,02915	0,075	0,112	1
Fbxl18	0,048068	-0,0178	0,194	0,253	1
Erlin1	0,048115	-0,01637	0,046	0,076	1
Rimkla	0,04812	-0,02157	0,033	0,059	1
Snx7	0,048309	-0,03915	0,101	0,141	1
Rab4b	0,048331	-0,02772	0,156	0,209	1
Lgals2	0,048381	-0,03127	0,084	0,122	1
Bcr	0,048385	-0,03688	0,178	0,23	1
Dync1li1	0,048418	-0,03783	0,415	0,502	1
Ccdc138	0,048456	0,044425	0,068	0,04	1
Ddhd2	0,048459	-0,03367	0,481	0,572	1
Ngfr	0,048572	0,109101	0,174	0,135	1
Srd5a1	0,048725	0,132238	0,451	0,411	1
Oscp1	0,048842	-0,02028	0,048	0,078	1
Eed	0,048844	-0,01507	0,218	0,283	1
Heg1	0,048849	-0,0338	0,29	0,363	1
Ring1	0,048886	-0,00509	0,497	0,618	1
Gpaa1	0,048923	-0,01878	0,268	0,342	1
Elf2	0,04906	0,098137	0,565	0,557	1
Slc45a1	0,049147	-0,01637	0,048	0,078	1
Hmgcll1	0,049176	0,045422	0,068	0,04	1
Zfp974	0,049179	-0,02116	0,064	0,099	1
Pcdhb14	0,049214	0,067823	0,114	0,078	1
Rbm3	0,049276	0,101039	0,576	0,561	1
Gm26881	0,049295	-0,02978	0,053	0,084	1
Tbc1d19	0,049323	-0,01787	0,246	0,316	1
Acsf3	0,049338	-0,02243	0,068	0,103	1
Adat1	0,049364	-0,02527	0,099	0,141	1
Adamtsl1	0,049524	-0,01343	0,541	0,662	1
Ogfrl1	0,049535	-0,01509	0,185	0,243	1
Klhl15	0,04963	-0,01921	0,193	0,251	1
Camk2g	0,049651	-0,03527	0,127	0,171	1
Borcs6	0,049695	-0,02466	0,317	0,39	1
Plag1	0,04986	-0,03255	0,086	0,124	1
Sec23ip	0,04994	-0,01227	0,29	0,365	1
Mr1	0,049971	0,027315	0,028	0,011	1
Alg8	0,05023	-0,02266	0,053	0,084	1
Tspan2os	0,050382	-0,01868	0,024	0,046	1
Ddx19a	0,050408	-0,01857	0,246	0,31	1
Unc119b	0,050432	-0,02865	0,084	0,122	1
Tmem132b	0,050489	-0,04906	0,473	0,559	1

Chd1l	0,050554	-0,02003	0,13	0,179	1
Dynlt3	0,050595	-0,04219	0,569	0,669	1
Fam213b	0,050597	-0,02857	0,339	0,414	1
Edrf1	0,050637	-0,01452	0,141	0,192	1
Nmd3	0,050695	-0,00796	0,171	0,228	1
Zfp467	0,050738	0,074801	0,213	0,167	1
Rsl1d1	0,050773	-0,03519	0,53	0,618	1
Fignl2	0,050824	-0,02112	0,017	0,036	1
Morc3	0,050825	-0,02072	0,206	0,266	1
Fam161a	0,050839	-0,02201	0,05	0,08	1
Tcn2	0,050851	-0,01078	0,25	0,319	1
Prkca	0,050875	-0,03909	0,651	0,734	1
Wdr75	0,050902	-0,02082	0,138	0,186	1
Gins1	0,050917	-0,01932	0,017	0,036	1
Sema4b	0,051024	0,033134	0,051	0,027	1
A030001D:	0,051252	-0,03012	0,083	0,12	1
Rfwd3	0,051254	-0,03906	0,253	0,314	1
Ahi1	0,05137	-0,00287	0,492	0,601	1
Zfp553	0,051417	-0,02923	0,442	0,536	1
Zik1	0,051418	-0,01827	0,035	0,061	1
Mtdh	0,051501	0,092922	0,972	0,951	1
Spag9	0,051571	0,06893	0,969	0,964	1
Dnase2a	0,051627	0,071823	0,112	0,078	1
Nr1d1	0,051658	-0,01705	0,017	0,036	1
Tmem168	0,051705	-0,01759	0,279	0,352	1
Btrc	0,051718	-0,04662	0,297	0,357	1
Tmem240	0,051791	-0,00253	0,024	0,046	1
Vasp	0,051819	-0,0344	0,11	0,152	1
Mafb	0,051988	0,048238	0,037	0,017	1
Kat8	0,052016	-0,01914	0,165	0,219	1
Stat5a	0,052156	0,027633	0,051	0,027	1
Cog7	0,052249	-0,00786	0,29	0,369	1
Gm37885	0,05225	0,028114	0,037	0,017	1
Gm16223	0,052269	-0,02447	0,026	0,049	1
Gzmm	0,052349	0,028902	0,037	0,017	1
Mrm2	0,052405	-0,02121	0,13	0,177	1
Fastkd5	0,052464	-0,01525	0,017	0,036	1
Pcdh10	0,052582	0,026154	0,47	0,574	1
Gm26582	0,052748	0,021466	0,013	0,002	1
Fam126b	0,052824	-0,00306	0,325	0,403	1
Cacna2d1	0,052833	0,014271	0,013	0,002	1
Kcnk7	0,052833	0,014271	0,013	0,002	1
Gm5608	0,052833	0,016075	0,013	0,002	1
Ascc3	0,052853	0,153838	0,429	0,418	1
Mcl1	0,052873	0,127373	0,723	0,709	1
Sv2c	0,052884	0,082728	0,024	0,008	1
Rdh13	0,052905	-0,03914	0,068	0,101	1
Uhrf1bp1	0,052924	-0,02694	0,057	0,089	1
Rpl9	0,052998	-0,01165	0,993	0,996	1
Gm53	0,053002	0,010655	0,013	0,002	1
Cd300a	0,053002	0,010655	0,013	0,002	1
Ucn2	0,053005	0,12101	0,013	0,002	1

2310074N1	0,053152	-0,02021	0,011	0,027	1
Itpr2	0,053192	0,107809	0,277	0,232	1
Cuedc1	0,053278	-0,02613	0,67	0,747	1
Prpf3	0,053378	-0,01224	0,134	0,184	1
Bricd5	0,053437	-0,02543	0,057	0,089	1
S100a11	0,053457	0,027158	0,037	0,017	1
Taf2	0,053474	-0,03681	0,235	0,295	1
Bzw2	0,053529	-0,03615	0,127	0,171	1
Fkbp15	0,053534	-0,03198	0,319	0,39	1
Vps52	0,053615	-0,0251	0,35	0,432	1
Ubr4	0,053659	0,092688	0,44	0,405	1
Glrx	0,053719	-0,01191	0,017	0,036	1
Nup153	0,053737	0,079557	0,277	0,234	1
Arhgap33	0,05382	-0,01365	0,198	0,255	1
Suox	0,053932	-0,02263	0,077	0,114	1
Fam199x	0,053945	0,093968	0,275	0,234	1
Psme4	0,053946	0,093097	0,398	0,363	1
Tmem88b	0,054053	-0,04316	1	0,998	1
Zfp113	0,054081	-0,03216	0,139	0,186	1
Slc13a3	0,054317	-0,05052	0,106	0,146	1
Gtf2f1	0,054427	0,002799	0,426	0,54	1
Zswim1	0,0545	-0,03103	0,072	0,105	1
Cgn	0,054551	0,03117	0,024	0,008	1
Scaper	0,054606	0,133838	0,383	0,357	1
Dlg3	0,054637	-0,01476	0,055	0,086	1
Lin54	0,054646	-0,02053	0,303	0,373	1
Eif5a	0,054647	-0,03483	0,95	0,956	1
Upf2	0,054687	-0,00427	0,413	0,513	1
Pcdhb4	0,054736	0,030842	0,024	0,008	1
Ggct	0,05477	0,006743	0,382	0,479	1
Pdk4	0,054775	-0,02027	0,15	0,2	1
Zeb1	0,054792	0,029405	0,024	0,008	1
Tmem131l	0,054792	0,029405	0,024	0,008	1
Ncor2	0,054824	0,126333	0,484	0,462	1
Tbx6	0,054894	0,037241	0,042	0,021	1
Zfp85	0,05491	-0,02245	0,039	0,065	1
Itfg1	0,054975	-0,036	0,721	0,789	1
Gxylt2	0,055034	0,018748	0,024	0,008	1
Snapc2	0,055038	-0,02754	0,163	0,213	1
Map7	0,055135	0,070104	0,991	0,996	1
Cstb	0,055135	0,118287	0,802	0,781	1
Gdi2	0,055175	-0,04494	0,908	0,909	1
Larp6	0,055228	-0,04785	0,778	0,838	1
4732491K2	0,055327	-0,02421	0,04	0,068	1
Bicc1	0,055375	-0,0162	0,266	0,338	1
Ube2j1	0,055399	-0,03204	0,233	0,291	1
Uimc1	0,055405	-0,03216	0,262	0,329	1
Tnrc6a	0,055536	0,095345	0,901	0,897	1
Dhx32	0,055569	-0,02403	0,103	0,143	1
Cebpg	0,055601	0,019597	0,382	0,483	1
2310034G0	0,055727	-0,00348	0,011	0,027	1
Dnajc12	0,055735	-0,02651	0,09	0,129	1

Supt16	0,055878	0,102097	0,618	0,61	1
Zfyve1	0,055888	-0,01053	0,339	0,42	1
Prpf40a	0,055899	0,110945	0,721	0,738	1
Nol7	0,055899	-0,03694	0,783	0,825	1
Ap3b2	0,055925	-0,03285	0,295	0,363	1
Tfcp2	0,055993	-0,01758	0,215	0,276	1
Clta	0,056232	-0,02862	0,967	0,956	1
Bdh1	0,05627	-0,04091	0,079	0,114	1
Edem1	0,056278	-0,00718	0,255	0,329	1
Frmd6	0,056439	-0,03051	0,042	0,07	1
Gm48742	0,056461	0,041393	0,064	0,038	1
Nubpl	0,056484	-0,02563	0,062	0,095	1
Filip1l	0,056485	-0,0295	0,029	0,053	1
Abhd2	0,056617	0,089745	0,283	0,241	1
Gap43	0,056698	-0,01713	0,028	0,051	1
Slc4a10	0,056794	-0,02157	0,018	0,038	1
Pibf1	0,056799	-0,01409	0,33	0,411	1
Mmadhc	0,056813	-0,02617	0,484	0,584	1
Tapt1	0,056816	-0,02279	0,383	0,468	1
Rrp15	0,056894	-0,02976	0,119	0,162	1
Eloa	0,056963	-0,0152	0,268	0,338	1
Slc10a3	0,056981	-0,01729	0,018	0,038	1
Celf5	0,057035	0,027959	0,02	0,006	1
Sptlc1	0,057066	-0,00448	0,409	0,508	1
Pafah2	0,057117	-0,0218	0,062	0,095	1
Snrnp70	0,057203	0,087332	0,978	0,975	1
Sgsm2	0,057226	0,049729	0,123	0,086	1
Ppil2	0,05726	-0,01219	0,508	0,622	1
Vps36	0,057272	0,003805	0,292	0,369	1
Angptl6	0,057302	-0,02258	0,128	0,175	1
Zfp626	0,057338	-0,01718	0,139	0,186	1
Mbd3	0,057428	-0,02536	0,6	0,703	1
Mc1r	0,057449	0,020842	0,02	0,006	1
Yap1	0,057449	0,020842	0,02	0,006	1
Phf11c	0,057449	0,024407	0,02	0,006	1
Tpk1	0,057454	-0,02464	0,042	0,07	1
Tceal9	0,05758	0,115367	0,593	0,565	1
H2-M5	0,0576	0,021153	0,017	0,004	1
Kctd12	0,0576	0,024725	0,017	0,004	1
Rbm8a	0,057616	-0,054	0,703	0,755	1
Pdzd4	0,057757	0,019363	0,017	0,004	1
Ccl4	0,057757	0,019363	0,017	0,004	1
Impdh1	0,057757	0,02294	0,017	0,004	1
Swap70	0,057757	0,024725	0,017	0,004	1
Cct8	0,057796	-0,04857	0,789	0,833	1
Zfp944	0,057807	-0,02343	0,097	0,137	1
Pitpnm1	0,057865	0,017264	0,02	0,006	1
Mxd3	0,057865	0,020842	0,02	0,006	1
Shroom4	0,057914	0,017569	0,017	0,004	1
Pank2	0,057978	-0,03287	0,178	0,228	1
Stom	0,058071	0,02294	0,017	0,004	1
Olfr639	0,058071	0,015772	0,017	0,004	1

Gm44759	0,058071	0,019363	0,017	0,004	1
Cntln	0,058121	-0,02819	0,198	0,255	1
Ifit1	0,058152	0,042397	0,017	0,004	1
Grik2	0,058152	0,097436	0,338	0,302	1
Lrrc75aos2	0,058228	0,019363	0,017	0,004	1
A330084C1	0,058228	0,015772	0,017	0,004	1
1810062O1	0,058228	0,013972	0,017	0,004	1
Pick1.1	0,058228	0,013972	0,017	0,004	1
Kidins220	0,05823	0,112947	0,708	0,736	1
Ak2	0,05825	-0,01519	0,367	0,458	1
Ccdc61	0,058309	-0,02702	0,119	0,162	1
Kmt2b	0,058321	-0,01982	0,281	0,35	1
A330102I1	0,058358	0,018748	0,02	0,006	1
Inafm2	0,05838	-0,02163	0,273	0,335	1
1700007G1	0,058385	0,012168	0,017	0,004	1
Rasd2	0,058385	0,012168	0,017	0,004	1
Abcg4	0,058385	0,012168	0,017	0,004	1
Trim45	0,058666	-0,02625	0,068	0,101	1
Sema3c	0,058804	-0,01515	0,004	0,015	1
Gm45159	0,058804	-0,01515	0,004	0,015	1
Hdx	0,058846	-0,04427	0,088	0,124	1
C030047K2	0,058979	-0,01307	0,004	0,015	1
Gm26873	0,058979	-0,01307	0,004	0,015	1
Frat1	0,059019	-0,02414	0,062	0,095	1
Myh7b	0,059063	0,108483	0,262	0,219	1
Gm10457	0,059069	-0,02157	0,004	0,015	1
Parm1	0,059083	-0,02063	0,323	0,399	1
Hprt	0,059129	-0,03141	0,228	0,285	1
Mfap1a	0,05914	-0,02474	0,435	0,515	1
Wdr38	0,059155	-0,011	0,004	0,015	1
Kcnq4	0,059155	-0,011	0,004	0,015	1
Gm42495	0,059155	-0,011	0,004	0,015	1
Gm10602	0,059155	-0,011	0,004	0,015	1
Acsm3	0,059155	-0,011	0,004	0,015	1
Pxdc1	0,059155	-0,011	0,004	0,015	1
Slc15a2	0,059183	-0,01606	0,028	0,051	1
Ppp6r3	0,059232	0,105701	0,514	0,5	1
Pigt	0,059236	0,1041	0,644	0,656	1
Sf3b4	0,05925	0,000794	0,406	0,511	1
Ube2q1	0,059275	-0,01783	0,424	0,496	1
Sgsh	0,059289	-0,03659	0,16	0,209	1
Mfhas1	0,059327	-0,02511	0,029	0,053	1
Siva1	0,059391	-0,01572	0,483	0,58	1
Cdc42bpg	0,059422	0,01308	0,02	0,006	1
Piezo2	0,059476	0,086507	0,222	0,175	1
Apba1	0,059481	0,110001	0,475	0,462	1
Jag1	0,059491	-0,01769	0,007	0,021	1
Fam186a	0,059491	-0,01769	0,007	0,021	1
E130114P1	0,059491	-0,01974	0,007	0,021	1
Rnasel	0,059491	-0,02589	0,007	0,021	1
Mex3d	0,059563	-0,02077	0,246	0,312	1
Adgra3	0,059581	-0,02135	0,029	0,053	1

Mb21d1	0,059601	-0,01125	0,004	0,015	1
Pot1a	0,059634	-0,0468	0,229	0,285	1
1700010I1	0,059777	-0,01563	0,007	0,021	1
Ddx25	0,059779	-0,00553	0,004	0,015	1
Srgn	0,059779	-0,00735	0,004	0,015	1
Hdgfl3	0,059784	-0,02129	0,615	0,717	1
Nup210	0,059882	0,044376	0,105	0,072	1
C130071C(	0,059886	0,00853	0,253	0,325	1
Bckdha	0,059998	-0,02771	0,51	0,603	1
Aplf	0,060045	-0,02686	0,07	0,103	1
Pabpc5	0,060063	-0,01356	0,007	0,021	1
Cnksr3	0,060095	-0,04657	0,101	0,139	1
En2	0,060121	-0,01155	0,196	0,253	1
mt-Nd2	0,060223	0,063397	1	0,998	1
Adamts1	0,060344	0,362513	0,369	0,346	1
Tsc22d2	0,06035	0,150339	0,47	0,458	1
Nradd	0,060385	0,05329	0,092	0,061	1
Hexdc	0,060424	-0,02656	0,161	0,211	1
Hoxa1	0,060427	-0,01998	0,007	0,021	1
A330069E1	0,060449	-0,03774	0,088	0,127	1
Sdha	0,060528	-0,0425	0,894	0,922	1
Rpa2	0,060705	-0,02907	0,277	0,342	1
Gpatch8	0,060728	-0,03904	0,866	0,916	1
Gnpat	0,06076	-0,03233	0,292	0,359	1
Gm2464	0,060789	-0,01175	0,007	0,021	1
Gm44658	0,060789	-0,01175	0,007	0,021	1
Gm15743	0,060789	-0,01175	0,007	0,021	1
Trim56	0,060906	0,066364	0,099	0,068	1
Pigl	0,060964	-0,02625	0,068	0,101	1
Nbea	0,060978	-0,05428	0,611	0,681	1
Clcn7	0,060993	-0,02573	0,209	0,266	1
Sqstm1	0,061045	0,068875	0,95	0,941	1
Tmem55a	0,061099	-0,02588	0,147	0,194	1
Mtmr6	0,061173	0,110832	0,572	0,582	1
Fzd5	0,061516	-0,03108	0,088	0,124	1
Taf7	0,061576	-0,00771	0,424	0,513	1
Arhgap24	0,061582	-0,01863	0,013	0,03	1
Traf2	0,061623	-0,02772	0,163	0,213	1
Ccdc92	0,061639	0,068286	0,194	0,154	1
Slc4a8	0,061705	0,084799	0,295	0,247	1
Trip4	0,061809	-0,03176	0,363	0,439	1
Ccdc47	0,061901	-0,02872	0,897	0,937	1
Stkld1	0,061909	0,007313	0,007	0	1
Olfml3	0,061909	0,007313	0,007	0	1
Cand2	0,061909	0,007313	0,007	0	1
Pirb	0,061909	0,007313	0,007	0	1
D030034A	0,061909	0,007313	0,007	0	1
Cyp4v3	0,061909	0,007313	0,007	0	1
Disc1	0,061909	0,007313	0,007	0	1
Zmiz1os1	0,061909	0,007313	0,007	0	1
Haus4	0,061909	0,007313	0,007	0	1
Tcap	0,061909	0,007313	0,007	0	1

Nckap5los	0,061909	0,007313	0,007	0	1
Gm49227	0,061909	0,007313	0,007	0	1
Acsl5	0,061909	0,007313	0,007	0	1
Timp1	0,061909	0,019982	0,007	0	1
2410004P(	0,061909	0,016379	0,007	0	1
Clvs1	0,061909	0,012762	0,007	0	1
Gm47117	0,061909	0,012762	0,007	0	1
Gm48342	0,061909	0,010949	0,007	0	1
Olfr366	0,061909	0,009132	0,007	0	1
Stk32b	0,061909	0,009132	0,007	0	1
Wipf3	0,061909	0,009132	0,007	0	1
Prrt3	0,061909	0,009132	0,007	0	1
1700067K(	0,061909	0,009132	0,007	0	1
Gm26816	0,061909	0,009132	0,007	0	1
Gm35166	0,061909	0,009132	0,007	0	1
Kbtbd6	0,061909	0,009132	0,007	0	1
Gm26767	0,061909	0,009132	0,007	0	1
Gpr22	0,061909	0,009132	0,007	0	1
Fkbp11	0,061909	0,009132	0,007	0	1
Mirt1	0,061909	0,009132	0,007	0	1
Rapgef6	0,061909	0,133289	0,402	0,384	1
Stab1	0,061909	0,010949	0,007	0	1
I830134H(	0,061909	0,010949	0,007	0	1
Tgtp2	0,061909	0,025364	0,007	0	1
Ccl2	0,061909	0,019982	0,007	0	1
9930111J2	0,061909	0,016379	0,007	0	1
D030040B:	0,061909	0,012762	0,007	0	1
Tmem82	0,061909	0,012762	0,007	0	1
AI854703	0,061909	0,012762	0,007	0	1
Parp10	0,061909	0,012762	0,007	0	1
Scn7a	0,061909	0,018182	0,007	0	1
Zfp61	0,06192	-0,03199	0,086	0,122	1
Nop9	0,061988	-0,02081	0,125	0,169	1
Wdfy1	0,062084	0,13829	0,451	0,439	1
A230009B:	0,062091	-0,02939	0,275	0,342	1
Slc4a7	0,062128	0,05326	0,141	0,103	1
Angel2	0,062201	-0,01128	0,448	0,557	1
Aldh2	0,062269	-0,06588	0,246	0,304	1
Zfp341	0,062269	-0,03086	0,033	0,057	1
Kirrel3	0,062426	0,090599	0,189	0,148	1
Txnl4b	0,062458	-0,022	0,105	0,146	1
Nupl2	0,062481	-0,0194	0,046	0,074	1
Zfp787	0,062496	-0,03504	0,191	0,241	1
Mbd2	0,062514	-0,04375	0,587	0,675	1
C030034I2	0,062533	-0,0063	0,028	0,051	1
Cep131	0,06254	-0,02537	0,077	0,112	1
Irf2bp2	0,062585	0,125703	0,774	0,814	1
Arhgef1	0,062681	-0,02364	0,325	0,392	1
Zfp513	0,062731	-0,03999	0,141	0,184	1
Soga3	0,062752	-0,04097	0,596	0,667	1
Itpkc	0,062917	0,069772	0,11	0,078	1
Klhl11	0,062949	-0,0333	0,095	0,133	1

Trpc4ap	0,06299	0,082546	0,824	0,848	1
Ctsl	0,063035	-0,04682	0,679	0,743	1
Tanc2	0,063074	-0,01548	0,33	0,411	1
Msra	0,063139	0,087666	0,25	0,209	1
Chd8	0,06314	0,100797	0,585	0,572	1
Marcks	0,06315	-0,05983	0,02	0,04	1
Zfp317	0,063158	-0,02601	0,193	0,247	1
Il1r2	0,063178	-0,00631	0	0,006	1
Gm15675	0,063178	-0,00631	0	0,006	1
Gm29514	0,063178	-0,00631	0	0,006	1
Cd48	0,063178	-0,00631	0	0,006	1
Lcn2	0,063178	-0,00631	0	0,006	1
Tgm2	0,063178	-0,00631	0	0,006	1
Wisp2	0,063178	-0,00631	0	0,006	1
Gdpd2	0,063178	-0,00631	0	0,006	1
Arxes1	0,063178	-0,00631	0	0,006	1
Htr2c	0,063178	-0,00631	0	0,006	1
Ace2	0,063178	-0,00631	0	0,006	1
Rbm46	0,063178	-0,00631	0	0,006	1
Vcam1	0,063178	-0,00631	0	0,006	1
Bmpr1b	0,063178	-0,00631	0	0,006	1
Gm34866	0,063178	-0,00631	0	0,006	1
Gm11831	0,063178	-0,00631	0	0,006	1
Gm12394	0,063178	-0,00631	0	0,006	1
Echdc2	0,063178	-0,00631	0	0,006	1
Laptm5	0,063178	-0,00631	0	0,006	1
Extl1	0,063178	-0,00631	0	0,006	1
Alpl	0,063178	-0,00631	0	0,006	1
Poln	0,063178	-0,00631	0	0,006	1
Gm16054	0,063178	-0,00631	0	0,006	1
Tacr1	0,063178	-0,00631	0	0,006	1
Gm44210	0,063178	-0,00631	0	0,006	1
Clec4d	0,063178	-0,00631	0	0,006	1
Zfp296	0,063178	-0,00631	0	0,006	1
B3gnt8	0,063178	-0,00631	0	0,006	1
Gm9885	0,063178	-0,00631	0	0,006	1
Gm44899	0,063178	-0,00631	0	0,006	1
Tsku	0,063178	-0,00631	0	0,006	1
Gm26705	0,063178	-0,00631	0	0,006	1
Fam57b	0,063178	-0,00631	0	0,006	1
Plpp4	0,063178	-0,00631	0	0,006	1
Tacc2	0,063178	-0,00631	0	0,006	1
Matk	0,063178	-0,00631	0	0,006	1
Gm15319	0,063178	-0,00631	0	0,006	1
Npy1r	0,063178	-0,00631	0	0,006	1
Cx3cl1	0,063178	-0,00631	0	0,006	1
Chst5	0,063178	-0,00631	0	0,006	1
Pnoc	0,063178	-0,00631	0	0,006	1
Gm41183	0,063178	-0,00631	0	0,006	1
Gpc5	0,063178	-0,00631	0	0,006	1
Pdgfd	0,063178	-0,00631	0	0,006	1
4930524O(	0,063178	-0,00631	0	0,006	1

Gm47043	0,063178	-0,00631	0	0,006	1
Cabp7	0,063178	-0,00631	0	0,006	1
Nefh	0,063178	-0,00631	0	0,006	1
4930505A(	0,063178	-0,00631	0	0,006	1
Tlx3	0,063178	-0,00631	0	0,006	1
4930412M	0,063178	-0,00631	0	0,006	1
Abcc3	0,063178	-0,00631	0	0,006	1
Hoxb9	0,063178	-0,00631	0	0,006	1
Etv4	0,063178	-0,00631	0	0,006	1
Kcnj16	0,063178	-0,00631	0	0,006	1
Hist1h4i	0,063178	-0,00631	0	0,006	1
A330076C(	0,063178	-0,00631	0	0,006	1
Cap2	0,063178	-0,00631	0	0,006	1
Gm35725	0,063178	-0,00631	0	0,006	1
Efcab10	0,063178	-0,00631	0	0,006	1
Ngb	0,063178	-0,00631	0	0,006	1
Mchr1	0,063178	-0,00631	0	0,006	1
Wnt7b	0,063178	-0,00631	0	0,006	1
AU022754	0,063178	-0,00631	0	0,006	1
Igfbp6	0,063178	-0,00631	0	0,006	1
Hoxc9	0,063178	-0,00631	0	0,006	1
Fstl1	0,063178	-0,00631	0	0,006	1
2810407A:	0,063178	-0,00631	0	0,006	1
Rpl7a-ps5	0,063178	-0,00631	0	0,006	1
Pcdha12	0,063178	-0,00631	0	0,006	1
Pcdhga2	0,063178	-0,00631	0	0,006	1
1700011I0	0,063178	-0,00631	0	0,006	1
Golga7b	0,063178	-0,00631	0	0,006	1
Ina	0,063178	-0,00631	0	0,006	1
A930001A:	0,063178	-0,0084	0	0,006	1
Arhgef26	0,063178	-0,0084	0	0,006	1
F3	0,063178	-0,0084	0	0,006	1
Zfp69	0,063178	-0,0084	0	0,006	1
1700001J(	0,063178	-0,0084	0	0,006	1
Add2	0,063178	-0,0084	0	0,006	1
Ccdc38	0,063178	-0,0084	0	0,006	1
Unc5d	0,063178	-0,0084	0	0,006	1
Unc13c	0,063178	-0,0084	0	0,006	1
Zmynd15	0,063178	-0,0084	0	0,006	1
Sult4a1	0,063178	-0,0084	0	0,006	1
Grp	0,063178	-0,0084	0	0,006	1
Gm16299	0,063178	-0,0084	0	0,006	1
Fndc9	0,063178	-0,01049	0	0,006	1
Tmem178	0,063178	-0,01258	0	0,006	1
Hoxb5	0,063178	-0,01466	0	0,006	1
Kif13b	0,063209	0,070399	0,91	0,901	1
Pdzd7	0,063293	-0,01502	0,013	0,03	1
Nsmf	0,063321	-0,02687	0,512	0,62	1
Zc4h2	0,063482	-0,01352	0,167	0,219	1
Atg101	0,063701	-0,01558	0,261	0,327	1
Fxyd3	0,063788	-0,02764	0,051	0,08	1
Slc2a3	0,063922	0,031291	0,039	0,019	1

Mtmr12	0,064117	-0,02816	0,092	0,129	1
Khk	0,064189	-0,01916	0,103	0,143	1
Eif2b4	0,064365	-0,0338	0,336	0,407	1
Dand5	0,064377	-0,03458	0,108	0,148	1
Dmd	0,064441	-0,03872	0,097	0,133	1
9430015G1	0,064456	-0,02757	0,092	0,129	1
Shoc2	0,064799	-0,01046	0,231	0,297	1
Cpsf3	0,064956	-0,0167	0,319	0,39	1
Zfp709	0,064962	-0,02113	0,033	0,057	1
Gm10701	0,065077	0,047182	0,05	0,027	1
Acvr1	0,065145	-0,02008	0,161	0,211	1
Zbtb25	0,065234	-0,03276	0,101	0,139	1
Cux1	0,065354	0,119565	0,574	0,586	1
Gm16124	0,065373	-0,02983	0,022	0,042	1
Slc13a5	0,065389	-0,02378	0,033	0,057	1
Sdad1	0,065435	-0,00541	0,165	0,219	1
Tmem109	0,065857	-0,01066	0,433	0,519	1
Mief1	0,065866	-0,01869	0,194	0,249	1
Pcdh7	0,065888	-0,04615	0,039	0,063	1
Fam131b	0,06608	-0,06238	0,571	0,627	1
Dtna	0,066112	0,125193	0,71	0,715	1
Wbp4	0,066229	-0,01325	0,521	0,603	1
Chchd10	0,066237	-0,18329	0,2	0,247	1
Slc8b1	0,066258	0,05211	0,167	0,127	1
Cdyl	0,06639	-0,02826	0,128	0,171	1
9330175E1	0,066401	0,02169	0,029	0,013	1
Spc24	0,066401	0,023461	0,029	0,013	1
Ankrd17	0,066469	0,123673	0,585	0,593	1
Armcx6	0,066585	-0,02266	0,053	0,082	1
1810009A1	0,066607	-0,01782	0,033	0,057	1
Sorbs3	0,066912	-0,02818	0,481	0,574	1
2010111I0	0,066941	-0,00989	0,505	0,603	1
Dpysl2	0,066954	-0,03779	1	0,998	1
Pik3c3	0,067063	-0,01058	0,339	0,418	1
Fbxo21	0,067077	0,093515	0,679	0,698	1
Phf2	0,067106	0,074623	0,361	0,316	1
Lysmd3	0,067136	0,012366	0,303	0,382	1
Papolg	0,067181	-0,02651	0,09	0,127	1
Gtf2e1	0,067219	-0,03002	0,136	0,179	1
Cul1	0,067268	-0,03384	0,633	0,707	1
Gm8797	0,067272	-0,03038	0,061	0,091	1
Snrnp200	0,067483	-0,02616	0,398	0,483	1
Gm15594	0,067508	-0,0112	0,046	0,074	1
Zfp53	0,067618	-0,02325	0,086	0,122	1
Plod3	0,067677	-0,00909	0,396	0,481	1
Rab13	0,067688	-0,0205	0,051	0,08	1
Tcp1	0,067698	-0,03124	0,75	0,821	1
Cox20	0,067927	-0,01861	0,396	0,481	1
Trim23	0,067936	-0,02905	0,288	0,344	1
Fmr1	0,067972	0,110124	0,633	0,631	1
Wrap73	0,068026	-0,01859	0,213	0,27	1
Gm26673	0,068049	0,044442	0,05	0,027	1

Tacc3	0,068064	-0,01691	0,094	0,131	1
Gon7	0,068097	-0,02021	0,114	0,156	1
Zkscan3	0,06814	-0,03455	0,477	0,565	1
Mob2	0,068163	-0,01037	0,343	0,43	1
Gm42715	0,06823	0,041406	0,073	0,046	1
Gsr	0,06832	-0,04019	0,369	0,443	1
Cbx7	0,068379	-0,01469	0,248	0,312	1
Shank1	0,068398	-0,02604	0,024	0,044	1
Zfp948	0,06841	-0,0332	0,152	0,198	1
Rnf8	0,068417	-0,03032	0,167	0,215	1
Mon2	0,068505	0,097295	0,686	0,7	1
Nt5e	0,068572	-0,01886	0,073	0,108	1
Stx7	0,068745	-0,04174	0,782	0,846	1
Ccdc166	0,068762	-0,01958	0,035	0,059	1
Ndrg3	0,068831	-0,03411	0,516	0,614	1
Gm26733	0,068852	-0,01239	0,022	0,042	1
Cln5	0,068889	-0,01357	0,382	0,464	1
Agl	0,068944	-0,02401	0,172	0,224	1
Park2	0,06896	-0,01153	0,141	0,188	1
Zfp870	0,068981	-0,02752	0,037	0,061	1
Atp6v1g2	0,069243	-0,0151	0,05	0,078	1
Abraxas1	0,069282	-0,00505	0,031	0,055	1
Zbtb17	0,069482	0,053916	0,158	0,12	1
Gucy1b1	0,069715	-0,03117	0,061	0,091	1
Ntrk2	0,069761	-0,00705	0,031	0,055	1
Dpf3	0,069784	-0,01283	0,002	0,011	1
Pde5a	0,069784	-0,0149	0,002	0,011	1
Ppox	0,069828	-0,02064	0,147	0,194	1
Gm17281	0,069907	-0,01075	0,002	0,011	1
Gm30097	0,069907	-0,01075	0,002	0,011	1
Gm45708	0,069907	-0,01075	0,002	0,011	1
Slc39a2	0,069907	-0,01075	0,002	0,011	1
9230116L(	0,069907	-0,01075	0,002	0,011	1
Pcdha11.1	0,069907	-0,01075	0,002	0,011	1
Zfp768	0,069909	-0,03014	0,169	0,217	1
Lrrc59	0,069938	-0,00336	0,257	0,323	1
Col5a3	0,069951	0,04378	0,035	0,017	1
Oxa1l	0,06997	-0,00255	0,367	0,451	1
Got2	0,069993	-0,02903	0,596	0,692	1
Gm13563	0,070031	-0,00866	0,002	0,011	1
Gria3	0,070031	-0,00866	0,002	0,011	1
Gm15477	0,070031	-0,00866	0,002	0,011	1
Gbp4	0,070031	-0,00866	0,002	0,011	1
Tmem116	0,070031	-0,00866	0,002	0,011	1
Gm44264	0,070031	-0,00866	0,002	0,011	1
Gm45799	0,070031	-0,00866	0,002	0,011	1
Gm48045	0,070031	-0,00866	0,002	0,011	1
Btbd11	0,070031	-0,00866	0,002	0,011	1
Trim43a	0,070031	-0,00866	0,002	0,011	1
Adora2b	0,070031	-0,00866	0,002	0,011	1
Stmnd1	0,070031	-0,00866	0,002	0,011	1
C030010L1	0,070031	-0,00866	0,002	0,011	1

Gm3417	0,070031	-0,00866	0,002	0,011	1
Crtc1	0,070045	-0,02722	0,112	0,152	1
Wdr12	0,070053	-0,00356	0,253	0,321	1
Klhl5	0,070127	-0,02835	0,167	0,215	1
Plekhh1	0,070296	0,065427	0,978	0,96	1
Rpgrip1	0,070331	-0,00775	0,255	0,319	1
Zfp367	0,070439	-0,02255	0,158	0,207	1
Ccdc126	0,070552	-0,03365	0,068	0,099	1
Pex6	0,070572	-0,03592	0,314	0,382	1
Chpf2	0,070616	-0,04507	0,114	0,152	1
Clybl	0,070627	-0,01322	0,015	0,032	1
1810030O(	0,070628	-0,03957	0,242	0,302	1
Cd302	0,070656	-0,00683	0,002	0,011	1
Cplx2	0,070656	-0,00683	0,002	0,011	1
Rab3c	0,070656	-0,00683	0,002	0,011	1
Gm29773	0,07066	-0,10072	0,009	0,023	1
Ankfy1	0,070693	-0,0275	0,347	0,42	1
Ehmt2	0,070792	0,092888	0,417	0,384	1
Zfp72	0,071028	-0,02501	0,059	0,089	1
Mlec	0,071038	-0,00715	0,505	0,61	1
2410080I0	0,071078	-0,00938	0,015	0,032	1
Cpeb3	0,071191	-0,01913	0,338	0,414	1
Dnajc27	0,07125	-0,01712	0,099	0,137	1
Alg12	0,071268	-0,02755	0,068	0,099	1
Itgb1	0,071366	0,08224	0,761	0,749	1
Ago1	0,071392	-0,01118	0,376	0,449	1
Rab35	0,071425	-0,01739	0,262	0,327	1
Dars2	0,071504	-0,01806	0,086	0,122	1
Mlst8	0,071525	-0,02751	0,139	0,184	1
Bbs10	0,071538	-0,01322	0,015	0,032	1
mt-Atp8	0,071552	0,082302	0,961	0,956	1
Brinp3	0,071571	-0,01822	0,024	0,044	1
Gm26546	0,071603	-0,01142	0,015	0,032	1
Laptm4a	0,071677	-0,03758	0,864	0,884	1
Zcrb1	0,071834	-0,03966	0,646	0,73	1
Gtf2h1	0,07197	-0,02668	0,261	0,316	1
Rbm5	0,072066	0,071423	0,839	0,808	1
9930012K:	0,072119	-0,01943	0,055	0,084	1
Dnm1	0,072131	-0,01587	0,009	0,023	1
Etaa1os	0,072228	-0,00957	0,024	0,044	1
Spryd4	0,072385	-0,02115	0,061	0,091	1
Zc3hc1	0,072526	-0,02762	0,15	0,196	1
Gm28424	0,072528	-0,01816	0,009	0,023	1
Plk3	0,072686	0,000365	0,312	0,386	1
Lrat	0,072921	-0,01611	0,009	0,023	1
Gm44053	0,073	-0,01405	0,009	0,023	1
C330027C(	0,073	-0,01405	0,009	0,023	1
4932438H:	0,07308	-0,01248	0,009	0,023	1
Rpl17	0,073094	0,001746	0,987	0,985	1
Kansl1	0,073155	0,121468	0,523	0,521	1
Usf3	0,0732	-0,01161	0,185	0,238	1
Ccdc71l	0,073225	-0,03802	0,149	0,19	1

Basp1	0,073286	-0,08703	0,044	0,07	1
Neil2	0,073396	-0,01199	0,009	0,023	1
Rhpn1	0,073396	-0,01199	0,009	0,023	1
Gm17552	0,073396	-0,01199	0,009	0,023	1
Tpm3-rs7	0,073425	-0,01442	0,024	0,044	1
Nck2	0,073457	-0,01626	0,22	0,281	1
Gm7347	0,073496	0,025547	0,026	0,011	1
Diras1	0,073673	0,093966	0,242	0,205	1
Amfr	0,073766	0,07742	0,75	0,747	1
Snx27	0,073854	0,085953	0,662	0,648	1
Pkdcc	0,073861	0,020223	0,026	0,011	1
Id1	0,07388	0,026041	0,035	0,017	1
Slitrk3	0,073958	-0,00887	0,009	0,023	1
Tmem198b	0,073986	-0,0111	0,024	0,044	1
Gm16794	0,074055	-0,00222	0,015	0,032	1
Sh3bp5l	0,074064	-0,03183	0,332	0,395	1
Mospd1	0,074115	-0,02699	0,139	0,184	1
Kif9	0,074278	-0,00837	0,009	0,023	1
Lrrc20	0,074335	-0,02897	0,044	0,07	1
Vps53	0,074349	-0,04078	0,402	0,473	1
Slc25a32	0,074426	-0,0226	0,114	0,154	1
Ccdc171	0,074435	-0,01873	0,039	0,063	1
Ube2d2a	0,074479	-0,04061	0,923	0,937	1
Spry1	0,074525	0,011462	0,218	0,285	1
Txndc11	0,074529	-0,01592	0,149	0,194	1
Zfpm2	0,074569	0,023973	0,035	0,017	1
Itpripl1	0,074595	0,018442	0,026	0,011	1
Gramd1b	0,074634	-0,01871	0,424	0,496	1
Mrps27	0,074656	-0,02414	0,068	0,099	1
Zfp961	0,074737	-0,02095	0,064	0,095	1
Tmed5	0,074769	-0,01025	0,736	0,816	1
Ddit4	0,074948	0,199983	0,47	0,468	1
Hps6	0,075045	-0,01546	0,039	0,063	1
Emp3	0,075112	0,097224	0,084	0,057	1
Tmem41b	0,075273	0,148104	0,598	0,612	1
Ppp1r9a	0,075299	-0,02292	0,433	0,519	1
Taf1	0,075323	0,102328	0,69	0,705	1
Rnf166	0,075334	-0,01625	0,15	0,196	1
Sdc2	0,075436	-0,01913	0,026	0,046	1
Kctd17	0,075452	0,10975	0,495	0,496	1
Aff1	0,075538	-0,01788	0,169	0,219	1
Npat	0,075724	-0,02584	0,538	0,618	1
Mcoln1	0,075732	-0,01842	0,305	0,371	1
Smad3	0,075781	0,054465	0,127	0,093	1
Zfp112	0,075792	-0,02973	0,079	0,112	1
Cep78	0,075813	-0,00765	0,108	0,148	1
Cachd1	0,076007	-0,02958	0,187	0,236	1
Ankrd46	0,076185	-0,03509	0,642	0,741	1
2210016L2	0,076279	-0,03796	0,697	0,759	1
Sgta	0,076293	-0,03397	0,872	0,907	1
Zfp507	0,076353	-0,01836	0,16	0,207	1
Arvcf	0,076398	-0,02243	0,068	0,099	1

Rab34	0,076402	0,037241	0,046	0,025	1
Gnai3	0,076494	0,073193	0,609	0,597	1
Gm14418	0,076598	-0,0189	0,026	0,046	1
Scaf4	0,076617	-0,02288	0,193	0,245	1
Pim3	0,076658	0,044714	0,802	0,85	1
Lamc1	0,076724	-0,00752	0,38	0,464	1
Anxa11	0,076786	0,02968	0,051	0,03	1
Zdhhc18	0,076865	0,064848	0,261	0,217	1
Coro7	0,076893	-0,04142	0,264	0,321	1
Kdm6b	0,076924	0,191347	0,217	0,181	1
Bnip1	0,076966	-0,00922	0,116	0,156	1
Gm7324	0,07699	-0,01932	0,017	0,034	1
Zfp472	0,077054	-0,02686	0,075	0,108	1
Lekr1	0,077159	-0,02947	0,128	0,169	1
A830005F2	0,077248	-0,01502	0,017	0,034	1
H2afy2	0,07726	-0,02285	0,068	0,099	1
Zfp398	0,077361	-0,01393	0,176	0,226	1
Fto	0,077488	0,104239	0,646	0,656	1
B4galt7	0,077538	-0,01975	0,099	0,137	1
Hnrnpul1	0,077656	0,108438	0,617	0,614	1
Ist1	0,077726	-0,0224	0,527	0,637	1
Znfx1	0,077751	-0,01457	0,363	0,443	1
Exog	0,077902	-0,02578	0,077	0,11	1
Tbc1d2b	0,078248	0,027633	0,051	0,03	1
Pard6g	0,078314	0,055858	0,086	0,059	1
Uggt1	0,078318	-0,01822	0,281	0,346	1
Skida1	0,078325	-0,01322	0,017	0,034	1
Ccnk	0,078606	-0,03422	0,306	0,367	1
Ipo11	0,07869	-0,02595	0,152	0,196	1
2900060B1	0,078953	0,040393	0,07	0,044	1
Ip6k1	0,079041	-0,01619	0,45	0,546	1
Gm20300	0,079043	-0,01515	0,323	0,388	1
Cstf2	0,079173	-0,03628	0,272	0,327	1
Pcdhgb4	0,079187	-0,02369	0,07	0,101	1
Zfp207	0,079233	-0,04019	0,628	0,709	1
Capns1	0,079436	-0,02122	0,978	0,981	1
Phactr4	0,079478	-0,03061	0,483	0,568	1
Gpr158	0,079598	-0,0169	0,028	0,049	1
Pdzd9	0,079655	-0,01539	0,006	0,017	1
Nckap1	0,079745	0,076096	0,82	0,814	1
Vps11	0,079828	-0,01975	0,33	0,401	1
Grm4	0,079863	-0,01563	0,006	0,017	1
2810442N1	0,079961	-0,01125	0,006	0,017	1
Cenpu	0,079961	-0,01125	0,006	0,017	1
Lrrc73	0,079961	-0,01125	0,006	0,017	1
Celf4	0,079961	-0,01125	0,006	0,017	1
Zfp39	0,079999	-0,0189	0,028	0,049	1
Tesk1	0,080111	-0,03405	0,189	0,234	1
Blm	0,080168	-0,02592	0,088	0,122	1
Osbpl11	0,08017	-0,03725	0,174	0,219	1
Trim11	0,080215	-0,03958	0,154	0,196	1
Sesn1	0,080222	-0,02756	0,18	0,226	1

Tbl1xr1	0,080251	0,000474	0,462	0,568	1
Ncaph2	0,080278	-0,03634	0,363	0,432	1
6030443JC	0,080378	-0,01667	0,028	0,049	1
Gak	0,080385	0,101242	0,31	0,281	1
Neo1	0,080458	0,091081	0,78	0,783	1
Pcdhb5	0,080514	0,02409	0,022	0,008	1
Rgs16	0,080517	0,054803	0,088	0,059	1
Arap1	0,080547	0,083882	0,31	0,272	1
Clhc1	0,080582	-0,00786	0,006	0,017	1
Gas1	0,080582	-0,00786	0,006	0,017	1
Efhd2	0,080611	0,071903	0,171	0,135	1
E430018J2	0,080624	-0,01698	0,042	0,068	1
Atp6v0a1	0,080686	0,098865	0,615	0,612	1
Spsb3	0,08078	-0,02027	0,277	0,335	1
Tmem217	0,080788	-0,00579	0,006	0,017	1
Gm16192	0,080788	-0,00942	0,006	0,017	1
Gm39214	0,080788	-0,00942	0,006	0,017	1
Gm46440	0,080788	-0,00942	0,006	0,017	1
1700105P(	0,080788	-0,00942	0,006	0,017	1
Gm10655	0,080857	0,022313	0,022	0,008	1
Stard10	0,080929	-0,02147	0,268	0,329	1
Nova1	0,080968	-0,00625	0,226	0,283	1
Pcnp	0,081033	0,073693	0,967	0,954	1
Sigmar1	0,081203	-0,02835	0,185	0,232	1
Exoc6	0,081325	-0,01711	0,136	0,179	1
Cnot2	0,081331	0,009838	0,336	0,407	1
Zscan29	0,081478	-0,03227	0,125	0,165	1
Zranb3	0,081502	-0,00977	0,04	0,065	1
Prmt7	0,081543	-0,03275	0,196	0,245	1
Sephs1	0,081668	-0,0299	0,152	0,196	1
Nudt21	0,081673	-0,01619	0,455	0,549	1
Gm48619	0,081709	-0,02268	0,029	0,051	1
Gdi1	0,081786	0,066015	0,914	0,924	1
Brwd1	0,081803	0,012307	0,466	0,572	1
Irf2bpl	0,081825	0,130308	0,301	0,272	1
Yipf2	0,081847	-0,02355	0,24	0,297	1
Bnip2	0,081894	0,000122	0,308	0,384	1
Wbp2	0,082085	-0,03771	0,967	0,983	1
Nagk	0,082202	0,000442	0,272	0,342	1
Tcaf1	0,082216	-0,01754	0,572	0,656	1
Wasl	0,082363	0,084194	0,626	0,608	1
Tubb4b	0,082409	0,004658	0,587	0,671	1
Acad9	0,08243	-0,03306	0,202	0,249	1
E430024I0	0,082562	-0,02028	0,05	0,076	1
Phykpl	0,082584	0,075353	0,156	0,12	1
Zfp446	0,082586	-0,03169	0,105	0,141	1
Kras	0,082604	0,103976	0,508	0,511	1
Lonp1	0,082696	0,010308	0,319	0,405	1
Slc25a36	0,08271	-0,01982	0,418	0,517	1
Efcc1	0,082787	0,075816	0,204	0,167	1
Lrrc61	0,082903	-0,03236	0,165	0,209	1
Agbl5	0,082989	-0,01816	0,011	0,025	1

Sfxn5	0,083252	-0,00978	0,136	0,179	1
Rgs2	0,083257	0,018367	0,095	0,065	1
Stx4a	0,083308	0,090319	0,826	0,804	1
Actr10	0,083387	-0,03325	0,683	0,783	1
Jdp2	0,083394	-0,02997	0,347	0,411	1
Rita1	0,083409	-0,02346	0,084	0,118	1
Fam220a	0,083419	0,011007	0,442	0,557	1
Spry3	0,083486	-0,01611	0,011	0,025	1
Gtf2h4	0,083497	-0,02835	0,101	0,137	1
Psmc2	0,083553	-0,04223	0,813	0,873	1
Vps9d1	0,083591	-0,01504	0,248	0,306	1
Cyfip2	0,083597	0,083565	0,328	0,285	1
Fbxo3	0,083665	-0,01847	0,281	0,348	1
9530082P2	0,083684	-0,02158	0,048	0,074	1
Brf1	0,083711	-0,00066	0,29	0,354	1
Prpf18	0,083757	-0,01921	0,295	0,365	1
Nmnat1	0,083777	-0,01984	0,048	0,074	1
C030037D(	0,083985	-0,01405	0,011	0,025	1
Gm47551	0,084059	0,021071	0,031	0,015	1
Supv3l1	0,084103	-0,01553	0,079	0,112	1
Nin	0,084153	0,073266	0,134	0,101	1
Gm36266	0,084247	-0,05682	0,011	0,025	1
N4bp2l2	0,084362	0,113976	0,628	0,635	1
Nol12	0,084413	-0,00284	0,277	0,346	1
Slc35a3	0,084529	-0,02935	0,231	0,283	1
Narf	0,084622	-0,01946	0,306	0,373	1
Cep126	0,084732	-0,01606	0,029	0,051	1
Bysl	0,084737	-0,02936	0,15	0,192	1
Cdk10	0,08479	-0,0279	0,158	0,203	1
Acat3	0,084999	-0,01224	0,011	0,025	1
Galnt10	0,085025	-0,01325	0,127	0,169	1
Txlna	0,085214	-0,03349	0,539	0,633	1
Mtrf1l	0,085285	-0,02522	0,117	0,156	1
Zfp956	0,085395	-0,01335	0,029	0,051	1
Tubgcp3	0,085571	-0,01317	0,206	0,259	1
Mtfmt	0,08569	-0,02366	0,09	0,124	1
Ltbp4	0,085753	0,051566	0,048	0,027	1
Tusc1	0,085769	-0,02006	0,048	0,074	1
Eif4enif1	0,085816	-0,00794	0,262	0,327	1
Ppif	0,085836	-0,022	0,09	0,124	1
Ank2	0,085838	-0,03855	0,998	0,994	1
Galk2	0,085875	-0,02487	0,099	0,135	1
Fuk	0,085986	-0,02438	0,055	0,082	1
Zbed4	0,086005	-0,03459	0,079	0,11	1
BC065403	0,086022	-0,00862	0,011	0,025	1
Gm43666	0,08613	0,014271	0,011	0,002	1
Gm47319	0,08613	0,014271	0,011	0,002	1
Cacna1b	0,08613	0,021466	0,011	0,002	1
Lag3	0,08613	0,017875	0,011	0,002	1
Tmem51os	0,08613	0,016075	0,011	0,002	1
Ung	0,08613	0,019672	0,011	0,002	1
Tagln3	0,086162	-0,01215	0,018	0,036	1

Gm12925	0,086266	0,016075	0,011	0,002	1
Tagln	0,086266	0,012465	0,011	0,002	1
Ttll12	0,086333	-0,01811	0,099	0,135	1
2310030G(	0,086403	0,014271	0,011	0,002	1
Ackr1	0,086403	0,010655	0,011	0,002	1
Gm14762	0,086403	0,010655	0,011	0,002	1
Gm15728	0,086403	0,010655	0,011	0,002	1
Gm26847	0,086403	0,010655	0,011	0,002	1
1810028F(	0,086403	0,010655	0,011	0,002	1
Sphk1	0,086403	0,010655	0,011	0,002	1
G630018N	0,086403	0,010655	0,011	0,002	1
Dpysl3	0,086403	0,010655	0,011	0,002	1
Pcgf1	0,086476	-0,02117	0,088	0,122	1
Ap1b1	0,086518	-0,01519	0,194	0,247	1
Olfr212	0,086539	0,008842	0,011	0,002	1
Gm34776	0,086547	-0,02201	0,051	0,078	1
Tpx2	0,086626	-0,00887	0,011	0,025	1
Mlh1	0,086668	-0,02257	0,139	0,181	1
Plekha2	0,086684	0,11469	0,251	0,219	1
Sugp2	0,086761	-0,01037	0,194	0,245	1
Srrd	0,086766	-0,03752	0,156	0,196	1
Hdac4	0,086781	0,114123	0,325	0,297	1
Ocrl	0,086799	-0,014	0,143	0,188	1
Blmh	0,086807	-0,00914	0,49	0,58	1
D10Wsu10	0,086821	0,051525	0,431	0,521	1
Ociad2	0,086883	0,024407	0,018	0,006	1
Rrp7a	0,086906	-0,00295	0,446	0,544	1
Tmem117	0,086931	-0,01226	0,44	0,536	1
Rnf113a1	0,087055	-0,00862	0,011	0,025	1
Nol6	0,087177	-0,0099	0,167	0,215	1
Scx	0,087186	0,020842	0,018	0,006	1
Psmb4	0,087187	-0,02578	0,947	0,96	1
P4ha1	0,087208	0,005055	0,332	0,409	1
Arf3	0,087254	0,107663	0,433	0,409	1
S100pbp	0,087295	-0,01647	0,189	0,241	1
Chl1	0,08749	0,019055	0,018	0,006	1
4831440D:	0,08749	0,019055	0,018	0,006	1
Rarg	0,08749	0,022626	0,018	0,006	1
Ggps1	0,087535	-0,02354	0,417	0,492	1
Traip	0,087577	-0,00374	0,011	0,025	1
Riok1	0,087663	0,011751	0,301	0,376	1
Lasp1	0,087665	0,024285	0,037	0,019	1
Atg7	0,087793	-0,00368	0,224	0,283	1
Dusp6	0,087794	0,01547	0,018	0,006	1
Olfr920	0,087794	0,01547	0,018	0,006	1
Spef1	0,087794	0,017264	0,018	0,006	1
Ezr	0,087794	0,017264	0,018	0,006	1
Mtch1	0,087868	0,052034	0,978	0,964	1
Crcp	0,087907	-0,03067	0,174	0,219	1
Slc36a4	0,087926	-0,01903	0,185	0,232	1
Tmem94	0,088079	-0,02261	0,11	0,148	1
Sike1	0,088353	-0,01822	0,356	0,443	1

Sorcs2	0,088513	0,01517	0,018	0,006	1
Cacfd1	0,08854	-0,03038	0,257	0,31	1
Zfp874b	0,088613	-0,01411	0,149	0,192	1
Gm44777	0,088676	-0,0166	0,05	0,076	1
Nudt3	0,088859	0,075018	0,837	0,812	1
Lims2	0,088973	-0,03006	0,231	0,283	1
Ube2l6	0,089027	-0,00809	0,018	0,006	1
Vmn1r12	0,089128	0,011286	0,018	0,006	1
Sltm	0,089271	0,096457	0,787	0,774	1
Plk4	0,08936	-0,05671	0,068	0,097	1
Atp8b1	0,089361	-0,01469	0,218	0,274	1
Rnf123	0,089382	-0,0288	0,169	0,213	1
Dzip1	0,089426	0,040506	0,121	0,089	1
Rnd3	0,089467	0,0015	0,16	0,205	1
Smad1	0,089484	-0,01236	0,253	0,312	1
Rasl11b	0,089624	-0,00053	0,358	0,43	1
Tesk2	0,089761	-0,02904	0,299	0,357	1
Ccser2	0,089764	0,120825	0,514	0,519	1
Vps13a	0,089777	-0,00143	0,29	0,357	1
Kif6	0,089821	-0,02794	0,218	0,27	1
Ephb1	0,089882	-0,04699	0,829	0,852	1
Dnajc28	0,089915	-0,01406	0,031	0,053	1
Snhg18	0,089944	-0,02137	0,055	0,082	1
Ccl27a	0,089968	-0,01958	0,033	0,055	1
Inpp5b	0,089991	-0,01841	0,167	0,213	1
Slc46a2	0,089995	0,024787	0,042	0,023	1
Fkbp9	0,089998	0,053851	0,207	0,169	1
Hinfp	0,09002	-0,03742	0,161	0,203	1
Fitm2	0,090142	-0,01691	0,169	0,215	1
Os9	0,090176	0,068926	0,752	0,741	1
Fstl3	0,09024	0,041023	0,042	0,023	1
Aldh9a1	0,090288	-0,02981	0,391	0,468	1
Aida	0,090351	-0,01128	0,31	0,369	1
Ikbkg	0,090406	-0,02493	0,136	0,177	1
Otub2	0,090509	-0,0166	0,053	0,08	1
Cacna1a	0,09055	0,067194	0,086	0,059	1
Zfp395	0,090611	0,089225	0,261	0,224	1
Btf3l4	0,090631	-0,02447	0,165	0,209	1
Kdm6bos	0,090702	0,015772	0,015	0,004	1
Slc22a17	0,090843	-0,03339	0,905	0,935	1
Zwilch	0,090847	-0,01958	0,035	0,057	1
Fzd1	0,09094	0,013972	0,015	0,004	1
Gm27253	0,09094	0,013972	0,015	0,004	1
Egln3	0,09094	0,015772	0,015	0,004	1
Gm37768	0,091178	0,012168	0,015	0,004	1
9030612E(	0,091178	0,012168	0,015	0,004	1
Map3k20	0,091416	0,010362	0,015	0,004	1
Odf3l2	0,091416	0,010362	0,015	0,004	1
Lgals9	0,091416	0,010362	0,015	0,004	1
Vmn2r124	0,091416	0,010362	0,015	0,004	1
Fcho2	0,091421	0,083248	0,78	0,797	1
Il17b	0,091451	-0,01667	0,02	0,038	1

Cnot9	0,091456	-0,02064	0,149	0,192	1
Zfp24	0,091502	0,100226	0,633	0,639	1
Pcdhga11	0,091556	-0,02775	0,07	0,099	1
Trpm3	0,091798	-0,01037	0,02	0,038	1
Atic	0,09185	-0,00304	0,266	0,333	1
Zfp646	0,092019	-0,01046	0,25	0,312	1
Gprasp1	0,092082	0,010441	0,382	0,466	1
Ints11	0,092534	-0,02085	0,176	0,224	1
Gpr157	0,092631	0,027165	0,066	0,042	1
B230354K1	0,092696	-0,02692	0,138	0,179	1
Nptn	0,092743	0,081121	0,714	0,715	1
U2af1	0,092775	-0,05366	0,894	0,905	1
Pcdhga5	0,092784	-0,01683	0,053	0,08	1
Mob3c	0,092793	-0,02137	0,057	0,084	1
Ric1	0,092962	-0,02283	0,244	0,295	1
Zfp60	0,093032	0,006527	0,327	0,397	1
Zfp946	0,093036	-0,03311	0,09	0,122	1
Cyth3	0,093043	-0,02362	0,198	0,249	1
Pgs1	0,093214	-0,01796	0,279	0,338	1
Lmbrd2	0,093288	0,087578	0,594	0,589	1
Mcrip1	0,093294	-0,04018	0,828	0,848	1
Pias4	0,093317	-0,0038	0,387	0,47	1
Synpo	0,093404	0,071092	0,246	0,209	1
Gabpb1	0,093416	-0,01691	0,103	0,139	1
Gm33979	0,093422	0,023461	0,028	0,013	1
Esf1	0,093422	-0,01351	0,431	0,515	1
Prmt5	0,093541	-0,0218	0,193	0,238	1
Mgat3	0,093679	-0,00941	0,442	0,538	1
Zfp9	0,093752	0,001366	0,158	0,205	1
Bex2	0,093822	-0,00883	0,02	0,038	1
Gm20219	0,093855	-0,01978	0,022	0,04	1
Ndrg4	0,093968	-0,00257	0,09	0,124	1
2410002F2	0,093985	-0,00588	0,358	0,439	1
Zfp874a	0,094039	-0,01705	0,095	0,131	1
Deptor	0,094092	-0,03482	0,22	0,268	1
Srrm2	0,094232	0,065987	0,996	0,994	1
Trim35	0,094361	0,067868	0,978	0,96	1
Crem	0,094457	-0,03002	0,138	0,177	1
Cux2	0,094469	-0,0123	0,15	0,196	1
Dhrs13	0,094484	-0,01958	0,037	0,059	1
Notch2	0,094791	0,0443	0,149	0,114	1
Tonsl	0,094949	0,02169	0,028	0,013	1
Megf6	0,094958	0,022837	0,028	0,013	1
Clcn5	0,094993	-0,01766	0,251	0,308	1
Rwdd2b	0,095008	-0,01055	0,035	0,057	1
D5Ertd579	0,095173	0,08922	0,653	0,677	1
Ghitm	0,095352	-0,03746	0,813	0,831	1
Stxbp2	0,095389	-0,12868	0,228	0,276	1
Tnrc6b	0,095509	0,067392	0,846	0,84	1
Tcirg1	0,09556	0,084188	0,316	0,276	1
Chmp4b	0,095811	-0,03392	0,894	0,918	1
Ubtf	0,09599	0,099455	0,633	0,631	1

Fbxw11	0,095993	0,11789	0,439	0,432	1
Lcmt2	0,096058	-0,0169	0,064	0,093	1
Mtap	0,09608	0,041058	0,068	0,044	1
AI837181	0,096147	-0,02463	0,334	0,401	1
Taf1b	0,096189	-0,02398	0,2	0,247	1
Cyb5r2	0,096196	-0,0186	0,064	0,093	1
Zfp410	0,096218	-0,01406	0,262	0,319	1
Bsdc1	0,096224	-0,02249	0,424	0,506	1
Rrp1b	0,096293	-0,00495	0,141	0,186	1
Slc25a15	0,09632	-0,03019	0,099	0,133	1
Prkar1b	0,096351	-0,02224	0,022	0,04	1
Gm14966	0,096413	-0,02202	0,037	0,059	1
Kit	0,096568	0,020561	0,084	0,057	1
Setd4	0,0966	-0,02421	0,037	0,059	1
Pgrmc2	0,096786	0,073822	0,587	0,553	1
Srxn1	0,096798	-0,03649	0,183	0,228	1
Ppp3cb	0,096858	0,022558	0,38	0,479	1
Urb2	0,096889	-0,01543	0,066	0,095	1
Pnpla8	0,09689	-0,0109	0,488	0,589	1
Tigar	0,097109	-0,02421	0,04	0,063	1
Rhob	0,097215	0,076524	1	0,998	1
Rprd1b	0,097264	-0,01156	0,233	0,289	1
Ttc41	0,097274	-0,00987	0,013	0,027	1
Tmem170b	0,097352	-0,01764	0,233	0,287	1
Ube2v1	0,097376	-0,02003	0,039	0,061	1
Ankzf1	0,097443	-0,01047	0,134	0,175	1
Mboat7	0,097603	0,05624	0,253	0,211	1
Gprasp2	0,097725	-0,01675	0,037	0,059	1
Polh	0,097894	-0,00887	0,013	0,027	1
Tm7sf2	0,097964	-0,0243	0,084	0,116	1
Il33	0,098107	-0,06816	0,93	0,833	1
Map3k21	0,098257	-0,01356	0,007	0,019	1
Gm19412	0,098257	-0,01356	0,007	0,019	1
BC024063	0,098278	-0,02625	0,024	0,042	1
Ccny	0,098511	-0,01982	0,275	0,333	1
Rab6b	0,098771	0,116241	0,648	0,637	1
Plekhm1	0,098858	0,050901	0,138	0,105	1
Phtf1	0,098912	-0,03933	0,125	0,16	1
Ski	0,098961	0,106685	0,536	0,54	1
Lat2	0,098988	-0,01868	0,024	0,042	1
Pagr1a	0,099186	0,030695	0,081	0,055	1
Rspry1	0,099253	-0,00853	0,363	0,437	1
Hddc2	0,099268	-0,01175	0,007	0,019	1
Paf1	0,099292	-0,00555	0,356	0,428	1
Nup85	0,099315	-0,02494	0,191	0,236	1
Spdl1	0,09938	-0,00993	0,007	0,019	1
Fam208b	0,099414	-0,0453	0,29	0,342	1
Bmi1	0,099453	0,11416	0,512	0,515	1
Zfp651	0,099484	0,102481	0,473	0,464	1
Zcchc7	0,099548	0,080081	0,787	0,816	1
Fgfr3	0,099717	-0,00786	0,007	0,019	1
A330035P1	0,099717	-0,00968	0,007	0,019	1

1700047I1	0,099717	-0,00968	0,007	0,019	1
Asb6	0,099736	-0,00957	0,204	0,253	1
Phrf1	0,099793	-0,01503	0,281	0,342	1
B3galnt2	0,09991	-0,02512	0,081	0,112	1
Akap13	0,099978	-0,02485	0,042	0,065	1
Rad9b	0,100054	-0,0222	0,095	0,129	1
Tmco3	0,100236	-0,02308	0,558	0,648	1
Lxn	0,100286	-0,00993	0,007	0,019	1
Gm43137	0,100286	-0,00993	0,007	0,019	1
Klc3	0,100286	-0,00993	0,007	0,019	1
Znhit2	0,100304	-0,01847	0,143	0,184	1
Nt5dc3	0,100305	-0,00993	0,108	0,146	1
Zfp26	0,10031	-0,00852	0,193	0,243	1
Alkbh3	0,100423	-0,0127	0,11	0,148	1
Stk24	0,100457	0,069907	0,217	0,181	1
Srp54c	0,100465	-0,02289	0,044	0,068	1
Ndc1	0,100504	-0,01442	0,18	0,228	1
Slc20a2	0,100541	-0,03806	0,75	0,795	1
Idnk	0,10061	-0,01207	0,211	0,262	1
Dynlt1c	0,10074	-0,00786	0,007	0,019	1
Zgrf1	0,100786	-0,02158	0,042	0,065	1
Tmem47	0,101004	-0,02068	0,024	0,042	1
Baiap2	0,101204	0,044786	0,05	0,03	1
4930432K2	0,101215	-0,01489	0,024	0,042	1
Ppp6r2	0,101304	-0,0266	0,339	0,405	1
Tti2	0,101366	-0,01196	0,114	0,152	1
Lockd	0,101414	0,022526	0,033	0,017	1
Rhot1	0,101454	-0,02312	0,365	0,432	1
Ncoa3	0,101493	0,005318	0,459	0,563	1
Btbd1	0,101632	-0,02775	0,554	0,624	1
Ric3	0,101638	-0,02619	0,106	0,141	1
Dpy19l4	0,101769	0,004316	0,349	0,428	1
Trp53inp2	0,101797	0,115197	0,306	0,281	1
Prkag3	0,102184	-0,02495	0,015	0,03	1
Usp38	0,1022	-0,04487	0,176	0,217	1
Cdr2	0,102229	-0,02656	0,16	0,2	1
Sdf4	0,102365	-0,02437	0,864	0,903	1
Rnf125	0,102511	-0,01477	0,039	0,061	1
Mpp6	0,102543	-0,02114	0,042	0,065	1
Gm16638	0,102627	-0,01993	0,083	0,114	1
Dnaaf5	0,102762	-0,03404	0,127	0,162	1
Adgrb3	0,102802	-0,0247	0,117	0,154	1
Slc38a2	0,102819	0,143458	0,98	0,964	1
Fancm	0,10284	-0,03653	0,112	0,146	1
Sept11	0,102843	0,055142	0,105	0,076	1
Colgalt2	0,102882	0,032693	0,068	0,044	1
Frmd4a	0,103181	0,030076	0,343	0,42	1
Zmiz2	0,10356	0,104758	0,426	0,403	1
Med23	0,103716	0,066606	0,194	0,16	1
Atp5o.1	0,103727	-0,04074	0,934	0,928	1
Dcaf11	0,103776	-0,01298	0,407	0,485	1
Usp15	0,103823	0,103224	0,615	0,624	1

Tspan6	0,103854	0,024846	0,068	0,044	1
Fance	0,103879	-0,03113	0,261	0,312	1
Sfxn3	0,103886	0,044632	0,13	0,099	1
Sesn3	0,103976	0,012581	0,305	0,369	1
Rab43	0,104095	0,024787	0,044	0,025	1
Zfp551	0,104113	-0,01868	0,026	0,044	1
Tmem214	0,104151	0,02736	0,281	0,352	1
Mllt10	0,104228	-0,01409	0,343	0,409	1
Fam13a	0,104391	-0,00953	0,039	0,061	1
Grik1	0,104596	-0,00605	0,217	0,268	1
Cep55	0,104615	-0,01307	0,004	0,013	1
Atp11c	0,104626	-0,03542	0,139	0,177	1
Tnfrsf22	0,104731	0,020458	0,039	0,021	1
Foxo6	0,104836	0,039437	0,083	0,057	1
Snw1	0,104864	-0,0236	0,787	0,829	1
Zmym5	0,104881	0,132817	0,506	0,519	1
Atm	0,104931	-0,01913	0,215	0,266	1
Pgrmc1	0,105007	-0,02305	0,694	0,762	1
Rcor1	0,10501	-0,01566	0,198	0,247	1
Fbxl5	0,105105	0,082649	0,794	0,795	1
Gm20186	0,105106	-0,00883	0,024	0,042	1
Rgp1	0,105115	-0,02695	0,132	0,169	1
Acbd7	0,105211	-0,00892	0,004	0,013	1
Chp2	0,105211	-0,00892	0,004	0,013	1
Gm45846	0,105211	-0,00892	0,004	0,013	1
Gm20687	0,105211	-0,00892	0,004	0,013	1
Fbxo48	0,105211	-0,00892	0,004	0,013	1
5430425K1	0,105211	-0,00892	0,004	0,013	1
Lgi1	0,105211	-0,00892	0,004	0,013	1
Nup214	0,105255	-0,00138	0,312	0,382	1
Trmt10b	0,105285	-0,03087	0,222	0,27	1
Zswim8	0,105388	-0,02094	0,383	0,456	1
Gm15860	0,105396	-0,0162	0,026	0,044	1
Nop14	0,105397	0,021035	0,286	0,354	1
Ovca2	0,105437	-0,01372	0,044	0,068	1
Tspan14	0,105544	0,09005	0,631	0,652	1
Rcor2	0,105548	0,02908	0,024	0,011	1
Lrrtm3	0,105583	-0,01683	0,046	0,07	1
C1galt1	0,105689	-0,0207	0,189	0,234	1
Syne4	0,105814	-0,00917	0,004	0,013	1
Klhl10	0,105814	-0,00917	0,004	0,013	1
Proser3	0,105945	-0,01592	0,046	0,07	1
Lrp8	0,105955	0,030516	0,024	0,011	1
Csf2ra	0,105958	-0,01258	0,152	0,194	1
Pfkfb2	0,105977	-0,01296	0,07	0,099	1
Luc7l3	0,105994	-0,0487	0,925	0,932	1
Selenoi	0,106016	-0,01778	0,323	0,386	1
1700029I1	0,106051	0,022001	0,024	0,011	1
Gm27003	0,106115	-0,00709	0,004	0,013	1
Gjb2	0,106115	-0,00709	0,004	0,013	1
Itga3	0,106115	-0,00709	0,004	0,013	1
Ccdc150	0,106149	0,005489	0,006	0	1

Gm38037	0,106149	0,005489	0,006	0	1
Spaca9	0,106149	0,005489	0,006	0	1
9530027J(	0,106149	0,005489	0,006	0	1
P2ry14	0,106149	0,005489	0,006	0	1
Riiad1	0,106149	0,005489	0,006	0	1
Cd72	0,106149	0,005489	0,006	0	1
Spag8	0,106149	0,005489	0,006	0	1
Gm26566	0,106149	0,005489	0,006	0	1
Cdkn2b	0,106149	0,005489	0,006	0	1
Dnali1	0,106149	0,005489	0,006	0	1
Slc25a34	0,106149	0,005489	0,006	0	1
Gm42902	0,106149	0,005489	0,006	0	1
Met	0,106149	0,005489	0,006	0	1
Akr1b8	0,106149	0,005489	0,006	0	1
D7Ertd128	0,106149	0,005489	0,006	0	1
4930558N	0,106149	0,005489	0,006	0	1
Shank2	0,106149	0,005489	0,006	0	1
Ces2b	0,106149	0,005489	0,006	0	1
Cacna1d	0,106149	0,005489	0,006	0	1
Dhrs2	0,106149	0,005489	0,006	0	1
Tcf7	0,106149	0,005489	0,006	0	1
Apoh	0,106149	0,005489	0,006	0	1
Hist1h2ak	0,106149	0,005489	0,006	0	1
1700099I0	0,106149	0,005489	0,006	0	1
Efcab11	0,106149	0,005489	0,006	0	1
9330161L(	0,106149	0,005489	0,006	0	1
Hoxc4	0,106149	0,005489	0,006	0	1
Itga5	0,106149	0,005489	0,006	0	1
AC166832.	0,106149	0,005489	0,006	0	1
Sept5	0,106149	0,005489	0,006	0	1
Crb3	0,106149	0,005489	0,006	0	1
Map3k8	0,106149	0,005489	0,006	0	1
Ppp1r32	0,106149	0,005489	0,006	0	1
Cfap43	0,106149	0,005489	0,006	0	1
Angptl3	0,10615	0,012762	0,006	0	1
Lacc1	0,10615	0,010949	0,006	0	1
Col4a4	0,10615	0,009132	0,006	0	1
Palmd	0,10615	0,009132	0,006	0	1
P2ry2	0,10615	0,009132	0,006	0	1
Stx11	0,10615	0,009132	0,006	0	1
Tgfbr3l	0,10615	0,009132	0,006	0	1
Gm15222	0,10615	0,009132	0,006	0	1
Kif23	0,10615	0,009132	0,006	0	1
AC153140.	0,10615	0,009132	0,006	0	1
Ly6g5b	0,10615	0,009132	0,006	0	1
Catip	0,10615	0,007313	0,006	0	1
Gm26683	0,10615	0,007313	0,006	0	1
Gli2	0,10615	0,007313	0,006	0	1
Fcgr2b	0,10615	0,007313	0,006	0	1
Nr6a1os	0,10615	0,007313	0,006	0	1
Hfm1	0,10615	0,007313	0,006	0	1
Vmn1r47	0,10615	0,007313	0,006	0	1

Apoc4	0,10615	0,007313	0,006	0	1
Omp	0,10615	0,007313	0,006	0	1
Ctrl	0,10615	0,007313	0,006	0	1
Olfr18	0,10615	0,007313	0,006	0	1
Ccl28	0,10615	0,007313	0,006	0	1
D630044L2	0,10615	0,007313	0,006	0	1
Arhgap28	0,10615	0,007313	0,006	0	1
Gldc	0,10615	0,007313	0,006	0	1
Eno4	0,10615	0,007313	0,006	0	1
Gm42047	0,10615	0,034268	0,006	0	1
Hcn3	0,10615	0,010949	0,006	0	1
9930021J0	0,106235	0,012662	0,534	0,635	1
Sdc3	0,106426	-0,01093	0,015	0,03	1
Arhgap19	0,106426	-0,00733	0,015	0,03	1
Nipa2	0,106429	0,003522	0,218	0,274	1
Ttc37	0,10646	-0,0038	0,193	0,243	1
Gm12840	0,106556	0,020223	0,024	0,011	1
Parp11	0,106601	-0,02375	0,051	0,076	1
Ticam1	0,106654	0,036701	0,07	0,046	1
Rfx4	0,106722	-0,00735	0,004	0,013	1
Rbbp7	0,106788	0,095829	0,549	0,544	1
Gaa	0,106836	0,09021	0,422	0,399	1
Camk1	0,106879	-0,03709	0,36	0,416	1
Rpl12	0,107013	0,105395	0,956	0,935	1
Hdac2	0,107036	-0,02661	0,308	0,369	1
Lrrn1	0,107043	-0,04248	0,906	0,932	1
Max	0,107125	0,002411	0,387	0,468	1
Skp2	0,107146	-0,01843	0,07	0,099	1
Ubxn2b	0,107161	-0,01401	0,158	0,2	1
Tmpo	0,107168	0,103123	0,563	0,561	1
Bcl2l1	0,107235	-0,0218	0,71	0,793	1
Arhgef37	0,107511	-0,01788	0,048	0,072	1
Zfp108	0,107547	0,019245	0,044	0,025	1
Mid1	0,107569	0,01487	0,024	0,011	1
A930024N1	0,107678	0,02523	0,024	0,011	1
D3Ertd254	0,107699	-0,02101	0,121	0,158	1
Gm27010	0,107953	-0,01475	0,079	0,11	1
Nup188	0,108208	0,042423	0,121	0,091	1
Tmed9	0,108241	-0,02094	0,89	0,918	1
Traf3ip1	0,108365	-0,00763	0,116	0,152	1
Prkar2b	0,108377	-0,00758	0,015	0,03	1
Yars	0,108444	-0,0087	0,325	0,39	1
Arhgap17	0,108591	-0,01302	0,378	0,456	1
Mecp2	0,108778	0,10011	0,683	0,711	1
Kdelc1	0,108817	-0,01719	0,086	0,118	1
Kcnh2	0,109028	-0,00733	0,015	0,03	1
Slc16a7	0,109145	0,107553	0,369	0,348	1
Wwox	0,109272	-0,01777	0,231	0,283	1
Ulk1	0,109424	0,073019	0,25	0,213	1
Smpd4	0,109665	0,066663	0,15	0,118	1
Gm39121	0,109693	-0,02886	0,062	0,089	1
Idh2	0,110056	-0,02249	0,25	0,3	1

Lats2	0,110076	-0,02559	0,235	0,285	1
Ubn2	0,110093	0,085331	0,754	0,743	1
Ttc39c	0,110104	-0,00206	0,132	0,173	1
Eif4e	0,110114	0,0015	0,519	0,608	1
Mcm7	0,110161	-0,05588	0,217	0,255	1
Tmem87b	0,110427	-0,00605	0,457	0,542	1
Scd1	0,110714	0,116935	0,739	0,732	1
Taf1a	0,110827	-0,01354	0,092	0,124	1
Zfp524	0,110938	-0,02398	0,207	0,253	1
Zc3h7a	0,110991	-0,0089	0,299	0,359	1
Pde8a	0,111074	-0,02651	0,91	0,89	1
C2cd2l	0,111129	-0,0186	0,182	0,228	1
Cd68	0,11115	0,02528	0,057	0,036	1
Spag1	0,111162	-0,01222	0,046	0,07	1
Pam	0,11127	-0,00565	0,508	0,593	1
Pdk1	0,111537	-0,01592	0,046	0,07	1
Atp6v0d1	0,111697	-0,03422	0,861	0,888	1
Slc7a11	0,112127	-0,01705	0,017	0,032	1
Trit1	0,112326	-0,01984	0,053	0,078	1
Lmnb1	0,112381	0,065007	0,143	0,112	1
Impact	0,112423	-0,03702	0,578	0,654	1
Smpd3	0,112429	0,077812	0,114	0,086	1
Id3	0,112451	0,064548	0,064	0,042	1
Mnt	0,112527	0,080281	0,284	0,251	1
Dnase1l1	0,112538	-0,01335	0,028	0,046	1
Iffo1	0,112607	0,114503	0,477	0,473	1
Zfp882	0,112677	-0,02826	0,112	0,146	1
D830025C(	0,112696	0,043406	0,077	0,053	1
Ahr	0,112755	0,031943	0,029	0,015	1
A630072M	0,113017	-0,0205	0,051	0,076	1
Thrsp	0,11308	-0,02696	0,136	0,175	1
Ints1	0,113181	0,08384	0,429	0,411	1
Ireb2	0,113312	-0,00953	0,321	0,388	1
2310068J1	0,113495	-0,02072	0,051	0,076	1
Helb	0,113547	0,049797	0,119	0,091	1
Hsp90ab1	0,113557	0,044438	1	0,998	1
Vav2	0,113581	-0,00957	0,028	0,046	1
Elmsan1	0,113629	-0,02407	0,22	0,268	1
Akr1c13	0,113642	-0,01667	0,029	0,049	1
Gm16062	0,113698	-0,01346	0,017	0,032	1
Rplp2	0,113712	-0,00038	0,993	0,992	1
Mex3c	0,113773	-0,00384	0,356	0,424	1
Mblac1	0,113781	-0,01297	0,017	0,032	1
Dgkq	0,114023	-0,01224	0,136	0,175	1
Zswim4	0,114026	0,027319	0,051	0,032	1
Cpsf6	0,114166	0,096917	0,613	0,618	1
Aak1	0,11417	0,014769	0,325	0,397	1
Slc39a9	0,114222	-0,01091	0,281	0,34	1
Rbm11	0,114489	-0,01877	0,055	0,08	1
Cavin3	0,11451	-0,07448	0,833	0,81	1
Rsbn1l	0,114558	0,002028	0,442	0,534	1
Fbxw2	0,11466	-0,01934	0,363	0,437	1

A430073D:	0,114667	-0,01381	0,009	0,021	1
Vps18	0,114678	-0,02001	0,218	0,266	1
Rbm45	0,114721	-0,01801	0,119	0,156	1
Gm32005	0,114737	-0,03199	0,084	0,114	1
Tmem55b	0,114764	-0,01926	0,688	0,793	1
Recql5	0,114784	-0,01609	0,127	0,165	1
Mkl1	0,114949	-0,01465	0,05	0,074	1
Cadps2	0,114961	0,05013	0,11	0,082	1
5430402O:	0,115254	-0,01175	0,009	0,021	1
AC151836.	0,115254	-0,01175	0,009	0,021	1
Tmem186	0,115318	-0,0097	0,112	0,148	1
Sept2	0,115343	-0,028	0,171	0,211	1
Bicral	0,115594	0,098833	0,349	0,319	1
Smc4	0,115678	-0,02164	0,277	0,335	1
Yipf6	0,115706	-0,01121	0,356	0,43	1
Fndc5	0,11585	-0,01199	0,009	0,021	1
Fam107a	0,11585	-0,01199	0,009	0,021	1
Man1a2	0,11585	0,001099	0,439	0,527	1
Armcx4	0,116327	0,044114	0,04	0,023	1
Ccrl2	0,116442	-0,00993	0,009	0,021	1
Zfp174	0,116816	-0,01383	0,031	0,051	1
Ssfa2	0,116889	0,140857	0,503	0,517	1
Bloc1s5	0,116893	-0,00844	0,226	0,278	1
Sf3b2	0,116912	-0,02912	0,958	0,966	1
Cenpb	0,116945	0,123613	0,521	0,536	1
Jpx	0,116985	-0,02669	0,136	0,173	1
Gm45716	0,117281	-0,00476	0,009	0,021	1
Eif4a3	0,117477	-0,02789	0,686	0,764	1
Cmbl	0,117479	-0,03884	0,163	0,2	1
Ddx49	0,117522	-0,02619	0,239	0,287	1
Cct4	0,117525	-0,04661	0,741	0,778	1
Pim2	0,117639	-0,00812	0,009	0,021	1
Swt1	0,117769	0,017607	0,488	0,597	1
Rad21	0,117956	0,074698	0,815	0,804	1
Pigg	0,11817	-0,02826	0,116	0,15	1
Atp23	0,118208	0,017532	0,029	0,015	1
Rbm12b1	0,11825	-0,02912	0,106	0,139	1
Ppp4r4	0,118297	0,024285	0,035	0,019	1
Mcur1	0,118329	-0,04608	0,174	0,211	1
Sar1a	0,118375	-0,01893	0,565	0,652	1
Wdr46	0,118391	-0,02913	0,196	0,241	1
Shb	0,118545	-0,02415	0,209	0,255	1
Gm43672	0,118571	-0,02554	0,035	0,055	1
Rbms2	0,11877	-0,01772	0,057	0,082	1
Gm12207	0,118847	0,022313	0,02	0,008	1
Ocel1	0,118918	-0,00948	0,11	0,146	1
Fjx1	0,118931	0,089442	0,083	0,059	1
Slc25a40	0,118967	-0,01175	0,105	0,139	1
Osbp2	0,119073	0,072011	0,343	0,306	1
Nat14	0,119079	-0,03103	0,073	0,101	1
Pcolce2	0,119173	-0,07219	0,659	0,698	1
Nectin2	0,119193	0,015459	0,029	0,015	1

Timp2	0,119281	0,052488	0,877	0,812	1
Gm14168	0,11933	0,01696	0,02	0,008	1
Plin4	0,119349	0,038103	0,136	0,103	1
Fa2h	0,119386	0,041445	0,998	0,994	1
Dffa	0,119587	-0,01556	0,138	0,177	1
Atg2a	0,119602	0,071893	0,24	0,207	1
Zfp119a	0,119621	-0,01359	0,031	0,051	1
Gm15952	0,119792	-0,01752	0,018	0,034	1
Fam184a	0,11986	-0,03333	0,106	0,139	1
Sumf1	0,119903	-0,02451	0,143	0,181	1
Vamp3	0,119995	-0,03791	0,763	0,814	1
Klhl20	0,120051	-0,01451	0,167	0,211	1
Tmem79	0,120058	-0,01502	0,018	0,034	1
Polrmt	0,120204	-0,0242	0,127	0,162	1
Gm26766	0,1203	0,013376	0,02	0,008	1
Arl1	0,120323	-0,02901	0,717	0,774	1
Cd27	0,120404	0,033514	0,044	0,068	1
Elmo1	0,120699	0,070277	0,873	0,878	1
Med14	0,120725	0,045742	0,174	0,139	1
Xlr3b	0,12121	-0,01845	0,018	0,034	1
Rp9	0,121708	-0,00277	0,525	0,597	1
Nr3c2	0,12203	-0,01571	0,099	0,131	1
Cars	0,122077	-0,02696	0,183	0,224	1
Ctdnep1	0,12223	-0,01234	0,363	0,435	1
Rtf2	0,122364	-0,02089	0,585	0,677	1
Fnbp4	0,122483	-0,01926	0,336	0,397	1
Mgat4a	0,122531	0,018394	0,035	0,019	1
Tchp	0,122806	0,002529	0,092	0,124	1
Fem1a	0,122825	-0,00739	0,259	0,312	1
Ubr7	0,12289	-0,02162	0,233	0,281	1
Fbxo5	0,123099	0,044392	0,088	0,063	1
Mvd	0,12317	0,125657	0,286	0,257	1
Atp6v1c1	0,123265	-0,01925	0,473	0,546	1
Adamts2	0,123319	-0,02091	0,218	0,266	1
Fam135a	0,12361	-0,01938	0,226	0,274	1
Nsun7	0,12379	0,025328	0,064	0,042	1
Ncstn	0,123898	-0,02424	0,242	0,291	1
Slc39a11	0,124132	-0,02221	0,081	0,11	1
Pcdh1	0,124301	-0,02443	0,106	0,139	1
1700030K(	0,124318	-0,0218	0,075	0,103	1
Rps12	0,12445	0,096748	0,989	0,994	1
Kpnb1	0,124453	0,082974	0,648	0,667	1
Tepsin	0,124483	-0,0126	0,062	0,089	1
Nrbp1	0,124605	-0,01089	0,325	0,386	1
Igf2bp3	0,124737	-0,00858	0,101	0,135	1
Shroom1	0,124869	-0,0285	0,27	0,321	1
Bcl2l12	0,125066	-0,01886	0,079	0,108	1
Rbpms	0,125382	-0,00452	0,018	0,034	1
Mns1	0,125478	-0,00097	0,018	0,034	1
Tefm	0,125499	-0,01407	0,059	0,084	1
Epm2aip1	0,125541	0,017082	0,404	0,485	1
Fam178b	0,125846	-0,0164	0,084	0,114	1

Prkd2	0,125876	0,032487	0,053	0,034	1
Fam114a1	0,126052	0,022637	0,059	0,038	1
Gga2	0,126186	0,049133	0,114	0,086	1
Limk2	0,126434	-0,04413	0,255	0,302	1
Stx12	0,126515	-0,03823	0,745	0,8	1
Wdfy3	0,126704	0,089095	0,681	0,671	1
Pgap3	0,126775	-0,01254	0,035	0,055	1
Rbm42	0,126779	0,073188	0,741	0,759	1
Lrp12	0,126797	-0,01918	0,172	0,215	1
Dlg2	0,126864	-0,00605	0,371	0,445	1
Rpl21	0,126995	-0,00848	0,998	0,996	1
Stn1	0,127005	-0,0114	0,167	0,211	1
B3galt2	0,127072	-0,01822	0,02	0,036	1
Fam133b	0,127107	-0,02826	0,615	0,648	1
Parvb	0,127109	0,039527	0,174	0,139	1
Ap5m1	0,127332	0,002133	0,217	0,27	1
Uck2	0,127334	-0,0222	0,119	0,154	1
Ppp1r14b	0,127398	-0,02003	0,136	0,173	1
Plekho1	0,127427	-0,01752	0,02	0,036	1
Vars2	0,127457	-0,01043	0,059	0,084	1
Polr1a	0,12752	-0,02161	0,196	0,241	1
Arl14ep	0,127792	-0,00758	0,257	0,312	1
Gm15859	0,12781	0,042079	0,083	0,059	1
Coq10b	0,127813	0,116193	0,253	0,23	1
Ift46	0,127844	-0,014	0,323	0,388	1
Mical3	0,128206	0,095334	0,585	0,591	1
Mef2c	0,1283	-0,01845	0,02	0,036	1
Dazap1	0,128344	-0,00953	0,527	0,61	1
Nfyb	0,128365	0,011357	0,323	0,39	1
Tanc1	0,128373	0,080423	0,651	0,656	1
Gpatch2l	0,128427	-0,0049	0,261	0,316	1
Gm527	0,128717	-0,01606	0,037	0,057	1
Slc10a7	0,12891	-0,03563	0,209	0,251	1
Dmtf1	0,128922	-0,01134	0,367	0,439	1
Cbx1	0,128985	-0,00773	0,655	0,741	1
Gm26810	0,129023	-0,01405	0,011	0,023	1
Vmac	0,129075	-0,03301	0,141	0,175	1
Agtrap	0,129079	-0,01654	0,077	0,105	1
Armc9	0,129187	-0,01505	0,127	0,162	1
Nf1	0,12924	0,073413	0,752	0,743	1
Magi3	0,129316	-0,0033	0,035	0,055	1
Eya1	0,129573	-0,00421	0	0,004	1
Gm37233	0,129573	-0,00421	0	0,004	1
Sgpp2	0,129573	-0,00421	0	0,004	1
Otos	0,129573	-0,00421	0	0,004	1
Entpd2	0,129573	-0,00421	0	0,004	1
Egfl7	0,129573	-0,00421	0	0,004	1
Chst1	0,129573	-0,00421	0	0,004	1
A930018P2	0,129573	-0,00421	0	0,004	1
Cst7	0,129573	-0,00421	0	0,004	1
1700003F1	0,129573	-0,00421	0	0,004	1
Vstm2l	0,129573	-0,00421	0	0,004	1

Gm14455	0,129573	-0,00421	0	0,004	1
Gm14401	0,129573	-0,00421	0	0,004	1
Glod5	0,129573	-0,00421	0	0,004	1
Gm15247	0,129573	-0,00421	0	0,004	1
Gm43241	0,129573	-0,00421	0	0,004	1
Bcl2l15	0,129573	-0,00421	0	0,004	1
Ddit4l	0,129573	-0,00421	0	0,004	1
Lrrc7	0,129573	-0,00421	0	0,004	1
Rad54b	0,129573	-0,00421	0	0,004	1
Phf24	0,129573	-0,00421	0	0,004	1
Plppr1	0,129573	-0,00421	0	0,004	1
Cfap57	0,129573	-0,00421	0	0,004	1
Zmynd12	0,129573	-0,00421	0	0,004	1
Hpca	0,129573	-0,00421	0	0,004	1
Ptafr	0,129573	-0,00421	0	0,004	1
Arhgef10l	0,129573	-0,00421	0	0,004	1
Prdm16	0,129573	-0,00421	0	0,004	1
Lgi2	0,129573	-0,00421	0	0,004	1
Tlr1	0,129573	-0,00421	0	0,004	1
Shisa3	0,129573	-0,00421	0	0,004	1
Gabra4	0,129573	-0,00421	0	0,004	1
Lnx1	0,129573	-0,00421	0	0,004	1
Crybb3	0,129573	-0,00421	0	0,004	1
Elfn1	0,129573	-0,00421	0	0,004	1
5730422E(	0,129573	-0,00421	0	0,004	1
Nxph1	0,129573	-0,00421	0	0,004	1
Aass	0,129573	-0,00421	0	0,004	1
Lrrtm4	0,129573	-0,00421	0	0,004	1
Cfap100	0,129573	-0,00421	0	0,004	1
Gm15687	0,129573	-0,00421	0	0,004	1
Hif3a	0,129573	-0,00421	0	0,004	1
Slc7a10	0,129573	-0,00421	0	0,004	1
Kcnc1	0,129573	-0,00421	0	0,004	1
Il16	0,129573	-0,00421	0	0,004	1
Trim34a	0,129573	-0,00421	0	0,004	1
Trim12a	0,129573	-0,00421	0	0,004	1
Itgax	0,129573	-0,00421	0	0,004	1
Ebf3	0,129573	-0,00421	0	0,004	1
Gm48021	0,129573	-0,00421	0	0,004	1
Aire	0,129573	-0,00421	0	0,004	1
Kitl	0,129573	-0,00421	0	0,004	1
Lyz1	0,129573	-0,00421	0	0,004	1
Rdh16	0,129573	-0,00421	0	0,004	1
Efnb2	0,129573	-0,00421	0	0,004	1
Adprhl1	0,129573	-0,00421	0	0,004	1
Atp7b	0,129573	-0,00421	0	0,004	1
Dctd	0,129573	-0,00421	0	0,004	1
Gm45848	0,129573	-0,00421	0	0,004	1
Pbx4	0,129573	-0,00421	0	0,004	1
Gm26586	0,129573	-0,00421	0	0,004	1
Gm42031	0,129573	-0,00421	0	0,004	1
Kcng4	0,129573	-0,00421	0	0,004	1

Car5a	0,129573	-0,00421	0	0,004	1
Trim67	0,129573	-0,00421	0	0,004	1
Gm31718	0,129573	-0,00421	0	0,004	1
Mir124a-1l	0,129573	-0,00421	0	0,004	1
Sox21	0,129573	-0,00421	0	0,004	1
Olfm2	0,129573	-0,00421	0	0,004	1
AB124611	0,129573	-0,00421	0	0,004	1
Epor	0,129573	-0,00421	0	0,004	1
Glb1l2	0,129573	-0,00421	0	0,004	1
Olfr981	0,129573	-0,00421	0	0,004	1
C030014I2	0,129573	-0,00421	0	0,004	1
Bco2	0,129573	-0,00421	0	0,004	1
Cck	0,129573	-0,00421	0	0,004	1
Tgm4	0,129573	-0,00421	0	0,004	1
Gm11963	0,129573	-0,00421	0	0,004	1
Upp1	0,129573	-0,00421	0	0,004	1
Slc22a21	0,129573	-0,00421	0	0,004	1
Slc16a11	0,129573	-0,00421	0	0,004	1
Proca1	0,129573	-0,00421	0	0,004	1
Tmem132e	0,129573	-0,00421	0	0,004	1
Pctp	0,129573	-0,00421	0	0,004	1
Meox1	0,129573	-0,00421	0	0,004	1
Mpp3	0,129573	-0,00421	0	0,004	1
Gm11739	0,129573	-0,00421	0	0,004	1
Gm48708	0,129573	-0,00421	0	0,004	1
Nrn1	0,129573	-0,00421	0	0,004	1
Gm26877	0,129573	-0,00421	0	0,004	1
Dbn1	0,129573	-0,00421	0	0,004	1
Gm47467	0,129573	-0,00421	0	0,004	1
Cys1	0,129573	-0,00421	0	0,004	1
Zdhhc22	0,129573	-0,00421	0	0,004	1
Chga	0,129573	-0,00421	0	0,004	1
Fam181a	0,129573	-0,00421	0	0,004	1
Ighm	0,129573	-0,00421	0	0,004	1
Gm48079	0,129573	-0,00421	0	0,004	1
Scrt1	0,129573	-0,00421	0	0,004	1
Mpped1	0,129573	-0,00421	0	0,004	1
Bin2	0,129573	-0,00421	0	0,004	1
Shisa9	0,129573	-0,00421	0	0,004	1
Cd86	0,129573	-0,00421	0	0,004	1
Boc	0,129573	-0,00421	0	0,004	1
Olfr204	0,129573	-0,00421	0	0,004	1
Igsf5	0,129573	-0,00421	0	0,004	1
Cpne5	0,129573	-0,00421	0	0,004	1
Adgre1	0,129573	-0,00421	0	0,004	1
Vit	0,129573	-0,00421	0	0,004	1
Arhgef33	0,129573	-0,00421	0	0,004	1
Gm6594	0,129573	-0,00421	0	0,004	1
Gm6277	0,129573	-0,00421	0	0,004	1
Nol4	0,129573	-0,00421	0	0,004	1
Pcdha9	0,129573	-0,00421	0	0,004	1
Kcnn2	0,129573	-0,00421	0	0,004	1

Camk2a	0,129573	-0,00421	0	0,004	1
Mro	0,129573	-0,00421	0	0,004	1
Gm6133	0,129573	-0,00421	0	0,004	1
Frmd8os	0,129573	-0,00421	0	0,004	1
Aldh1a7	0,129573	-0,00421	0	0,004	1
Pip5k1b	0,129573	-0,00421	0	0,004	1
Gm15491	0,129573	-0,00421	0	0,004	1
Adrb1	0,129573	-0,00421	0	0,004	1
Inpp4b	0,129573	-0,0084	0	0,004	1
Nyap2	0,129573	-0,00631	0	0,004	1
Hmcn1	0,129573	-0,00631	0	0,004	1
Gad1	0,129573	-0,00631	0	0,004	1
Fsbp	0,129573	-0,00631	0	0,004	1
Col15a1	0,129573	-0,00631	0	0,004	1
Gm11266	0,129573	-0,00631	0	0,004	1
Prom1	0,129573	-0,00631	0	0,004	1
Gm33370	0,129573	-0,00631	0	0,004	1
Gm43118	0,129573	-0,00631	0	0,004	1
Npy	0,129573	-0,00631	0	0,004	1
Sspnos	0,129573	-0,00631	0	0,004	1
Fam174b	0,129573	-0,00631	0	0,004	1
Ascl2	0,129573	-0,00631	0	0,004	1
Cideb	0,129573	-0,00631	0	0,004	1
Gm40513	0,129573	-0,00631	0	0,004	1
Bcl2a1b	0,129573	-0,00631	0	0,004	1
Hoxb4	0,129573	-0,00631	0	0,004	1
3110006O(	0,129573	-0,00631	0	0,004	1
Myt1l	0,129573	-0,00631	0	0,004	1
Prox2os	0,129573	-0,00631	0	0,004	1
Sncg	0,129573	-0,0084	0	0,004	1
Ptgr1	0,129573	-0,01049	0	0,004	1
Arhgap42	0,129573	-0,01049	0	0,004	1
6030419C:	0,129573	-0,01258	0	0,004	1
Emsy	0,129575	0,091799	0,56	0,561	1
Snx22	0,129748	-0,01719	0,075	0,103	1
4930426I2	0,129811	-0,01419	0,062	0,089	1
Prkaa1	0,129888	-0,01728	0,239	0,289	1
Prpsap2	0,129898	-0,03259	0,349	0,399	1
Ehhadh	0,130008	-0,00532	0,059	0,084	1
Il17ra	0,130193	-0,01683	0,088	0,118	1
Ldha	0,130221	0,074968	0,101	0,076	1
Nsd2	0,130294	0,072526	0,501	0,498	1
Ddx46	0,130361	-0,02146	0,545	0,616	1
Hey1	0,130365	-0,01224	0,011	0,023	1
Gm12279	0,130365	-0,01224	0,011	0,023	1
Stxbp1	0,130451	0,07032	0,272	0,241	1
AC130815.	0,130476	0,161626	0,229	0,2	1
C1qb	0,130762	-0,01191	0,02	0,036	1
Slc39a7	0,130781	0,077229	0,65	0,652	1
Bcan	0,130859	-0,01886	0,011	0,023	1
Ubxn7	0,130905	0,000303	0,343	0,411	1
Pm20d1	0,130935	0,031499	0,017	0,006	1

Chmp1a	0,130944	-0,02053	0,523	0,61	1
Sfr1	0,130964	-0,0148	0,954	0,949	1
Cflar	0,130969	-0,01135	0,198	0,245	1
Bicd2	0,131059	0,096446	0,714	0,715	1
Gm28557	0,131097	-0,00656	0,011	0,023	1
Ifih1	0,131097	-0,01018	0,011	0,023	1
Thap8	0,131097	-0,01018	0,011	0,023	1
Tlcd1	0,131097	-0,01018	0,011	0,023	1
Gm48899	0,131097	-0,01018	0,011	0,023	1
Fen1	0,131098	0,020638	0,161	0,203	1
Txndc5	0,131228	-0,01386	0,086	0,116	1
Gm43263	0,131236	-0,01629	0,039	0,059	1
Tk1	0,131372	0,017264	0,017	0,006	1
Lyrm4	0,131395	-0,02258	0,132	0,167	1
Gm9803	0,131402	-0,01827	0,04	0,061	1
Umps	0,131447	-0,01912	0,097	0,129	1
Nol3	0,131842	0,061671	0,398	0,369	1
St8sia5	0,131973	0,069488	0,316	0,276	1
Foxj2	0,131987	0,079944	0,35	0,323	1
Sdc1	0,132014	0,07704	0,048	0,03	1
Stx16	0,132123	0,010323	0,574	0,671	1
Sh3kbp1	0,132182	-0,02454	0,725	0,772	1
Zfp105	0,132227	-0,0123	0,037	0,057	1
Il10rb	0,132248	0,017264	0,017	0,006	1
Ckap2	0,132248	0,01547	0,017	0,006	1
Ddx18	0,132273	0,006537	0,442	0,54	1
Halr1	0,132343	-0,01075	0,002	0,008	1
Calhm2	0,132343	-0,01075	0,002	0,008	1
Lmtk2	0,132419	0,047405	0,15	0,12	1
Dock1	0,132449	0,099056	0,765	0,755	1
Glyctk	0,132456	-0,00837	0,011	0,023	1
Ccnf	0,132456	-0,00837	0,011	0,023	1
Tmub1	0,132488	-0,02019	0,239	0,287	1
Tceal5	0,13257	-0,00866	0,002	0,008	1
Tnfaip3	0,13257	-0,00866	0,002	0,008	1
Nrsn1	0,13257	-0,00866	0,002	0,008	1
Radil	0,13257	-0,01283	0,002	0,008	1
Cavin1	0,13257	-0,01283	0,002	0,008	1
Mical1	0,13257	-0,02111	0,002	0,008	1
Ap1m1	0,132635	-0,00508	0,25	0,3	1
Kantr	0,132652	-0,01754	0,11	0,143	1
Pram1	0,132687	0,013673	0,017	0,006	1
Klhl23	0,132687	0,011873	0,017	0,006	1
Net1	0,132687	0,011873	0,017	0,006	1
Gm20045	0,132777	-0,00257	0,075	0,103	1
3110035E1	0,132796	-0,00657	0,002	0,008	1
Gm42417	0,132796	-0,00657	0,002	0,008	1
5730559C1	0,132796	-0,00657	0,002	0,008	1
Ccdc190	0,132796	-0,00657	0,002	0,008	1
Kcnj9	0,132796	-0,00657	0,002	0,008	1
Spag6	0,132796	-0,00657	0,002	0,008	1
Mamdc4	0,132796	-0,00657	0,002	0,008	1

BC016548	0,132796	-0,00657	0,002	0,008	1
Rtl8c	0,132796	-0,00657	0,002	0,008	1
Fgf2	0,132796	-0,00657	0,002	0,008	1
Gm3764	0,132796	-0,00657	0,002	0,008	1
Pou3f2	0,132796	-0,00657	0,002	0,008	1
AI427809	0,132796	-0,00657	0,002	0,008	1
Dmrta2	0,132796	-0,00657	0,002	0,008	1
Abcb4	0,132796	-0,00657	0,002	0,008	1
Gm9970	0,132796	-0,00657	0,002	0,008	1
Gm43660	0,132796	-0,00657	0,002	0,008	1
Anxa3	0,132796	-0,00657	0,002	0,008	1
Nos1	0,132796	-0,00657	0,002	0,008	1
Gm15627	0,132796	-0,00657	0,002	0,008	1
Ache	0,132796	-0,00657	0,002	0,008	1
Fam71f2	0,132796	-0,00657	0,002	0,008	1
Tril	0,132796	-0,00657	0,002	0,008	1
Ccdc142	0,132796	-0,00657	0,002	0,008	1
Gm45441	0,132796	-0,00657	0,002	0,008	1
Grm5	0,132796	-0,00657	0,002	0,008	1
Gm32687	0,132796	-0,00657	0,002	0,008	1
Lrp1	0,132796	-0,00657	0,002	0,008	1
Gm15350	0,132796	-0,00657	0,002	0,008	1
Gm49083	0,132796	-0,00657	0,002	0,008	1
Gm21750	0,132796	-0,00657	0,002	0,008	1
Ednrb	0,132796	-0,00657	0,002	0,008	1
1810041H	0,132796	-0,00657	0,002	0,008	1
Rasl10a	0,132796	-0,00657	0,002	0,008	1
BC049762	0,132796	-0,00657	0,002	0,008	1
Hoxb7	0,132796	-0,00657	0,002	0,008	1
Hoxb2	0,132796	-0,00657	0,002	0,008	1
Gsdma	0,132796	-0,00657	0,002	0,008	1
Vmn1r218	0,132796	-0,00657	0,002	0,008	1
Gm29675	0,132796	-0,00657	0,002	0,008	1
Sfrp5	0,132796	-0,00657	0,002	0,008	1
Kcnip2	0,132796	-0,00657	0,002	0,008	1
Pcdh19	0,132838	0,025865	0,017	0,006	1
Ctu1	0,132885	-0,01001	0,04	0,061	1
Slc35a4	0,133048	-0,01898	0,411	0,483	1
Pold1	0,133183	-0,01322	0,103	0,135	1
Tmlhe	0,133314	-0,00855	0,035	0,055	1
D17H6S53	0,133405	-0,03723	0,134	0,167	1
Pcdhb13	0,133575	0,011579	0,017	0,006	1
Zc3h7b	0,133652	-0,01871	0,374	0,441	1
Rrp12	0,13368	-0,01376	0,088	0,118	1
Smim6	0,133709	0,000729	0,002	0,008	1
Lcn11	0,133709	-0,00109	0,002	0,008	1
Slitrk2	0,133709	-0,00291	0,002	0,008	1
S100a3	0,133709	-0,00291	0,002	0,008	1
Igsf21	0,133709	-0,00291	0,002	0,008	1
Tmem150b	0,133709	-0,00291	0,002	0,008	1
1700102H	0,133709	-0,00291	0,002	0,008	1
Igfbp5	0,133709	-0,00474	0,002	0,008	1

C130013H(	0,133709	-0,00474	0,002	0,008	1
Gm9754	0,133709	-0,00474	0,002	0,008	1
Sftpc	0,133709	-0,00474	0,002	0,008	1
Slitrk1	0,133709	-0,00474	0,002	0,008	1
Spag5	0,133709	-0,00474	0,002	0,008	1
Dgkg	0,133709	-0,00474	0,002	0,008	1
Gm15829	0,133709	-0,00474	0,002	0,008	1
Fam171a2	0,133709	-0,00318	0,002	0,008	1
Ilkap	0,133792	-0,00632	0,543	0,648	1
1700066B:	0,133825	-0,00656	0,011	0,023	1
Ppp2r2b	0,133825	-0,00656	0,011	0,023	1
Lix1l	0,134442	0,040822	0,112	0,084	1
Nuf2	0,134463	-0,01125	0,006	0,015	1
Plcxd2	0,134614	0,016056	0,026	0,013	1
Fxr1	0,134668	-0,03494	0,883	0,899	1
Mcmdc2	0,134912	0,009489	0,017	0,006	1
Atpaf2	0,134924	-0,01618	0,088	0,118	1
Gm14308	0,13495	-0,00917	0,006	0,015	1
Med9os	0,13495	-0,00917	0,006	0,015	1
Gm26964	0,13495	-0,00917	0,006	0,015	1
Pik3r5	0,13495	-0,00917	0,006	0,015	1
C2	0,13495	-0,00917	0,006	0,015	1
Arsk	0,135057	0,00244	0,207	0,257	1
Mfsd5	0,135082	-0,02064	0,279	0,331	1
Bhlhe41	0,135125	0,083565	0,857	0,819	1
Bend3	0,135204	-0,00476	0,011	0,023	1
Nfat5	0,13534	0,10223	0,673	0,654	1
Spaca6	0,13536	-0,0069	0,154	0,194	1
Paqr6	0,135417	0,1424	0,439	0,416	1
Prss53	0,135422	-0,01394	0,022	0,038	1
Kank1	0,135443	-0,01974	0,006	0,015	1
Fdxr	0,135486	0,002495	0,334	0,401	1
Noct	0,135496	-0,01103	0,039	0,059	1
Dlg5	0,135529	-0,00835	0,18	0,226	1
Tmco6	0,135586	-0,02394	0,141	0,177	1
Adcy9	0,135593	-0,02826	0,116	0,148	1
Amdhd2	0,135606	0,105854	0,398	0,388	1
Clrn1	0,135606	-0,00942	0,006	0,015	1
AC161165.	0,135606	-0,00942	0,006	0,015	1
Aph1c	0,135626	-0,02202	0,024	0,04	1
Xndc1	0,135766	-0,01914	0,163	0,203	1
Fbxo32	0,135808	0,077862	0,734	0,728	1
Zfp953	0,135825	-0,01698	0,04	0,061	1
Gmfb	0,135923	0,078283	0,717	0,724	1
Osbpl9	0,136069	-0,01612	0,161	0,203	1
G630016G	0,136097	-0,00735	0,006	0,015	1
Msi1	0,136097	-0,00735	0,006	0,015	1
Gm16049	0,136097	-0,00735	0,006	0,015	1
Phlpp1	0,13614	0,05712	0,983	0,996	1
Fzd3	0,136152	-0,0216	0,136	0,171	1
Epg5	0,136271	-0,02388	0,16	0,196	1
Kat2a	0,136281	-0,02466	0,156	0,194	1

Cecr2	0,136317	-0,01274	0,064	0,091	1
Gm2000	0,136512	-0,00842	0,084	0,114	1
Pou3f1	0,136549	0,180729	0,75	0,734	1
Meis1	0,136553	-0,0013	0,334	0,397	1
Tube1	0,136566	-0,01191	0,022	0,038	1
Rabgap1l	0,136715	0,066739	0,55	0,538	1
Serinc5	0,136869	0,075279	0,796	0,719	1
Phf10	0,136914	-0,02955	0,398	0,456	1
Zfp619	0,136954	-0,01349	0,04	0,061	1
Avpi1	0,13701	-0,00953	0,037	0,057	1
Trpv3	0,137148	0,02932	0,068	0,046	1
Tmem67	0,137157	-0,01152	0,079	0,108	1
Phf2os1	0,137252	-0,00371	0,006	0,015	1
Bcl2l11	0,137319	0,086282	0,224	0,194	1
Slc23a2	0,137794	-0,02835	0,187	0,226	1
Top3a	0,137858	-0,01151	0,042	0,063	1
Dok7	0,137918	0,057198	0,149	0,12	1
Cep72	0,137937	-0,00975	0,095	0,127	1
Epb41l4ao:	0,137945	-0,00877	0,233	0,283	1
Rpl5	0,138039	0,08278	0,993	0,987	1
Cdkn2aipnl	0,138224	-0,00143	0,226	0,276	1
Rbm12b2	0,138239	-0,00833	0,116	0,15	1
Lrrc56	0,138391	-0,01198	0,042	0,063	1
Mapk1ip1l	0,138464	-0,0031	0,27	0,323	1
Cul4a	0,138637	-0,01574	0,328	0,388	1
Smyd3	0,138757	0,000183	0,132	0,171	1
Plk2	0,138816	0,04802	0,07	0,049	1
Gpr75	0,138909	-0,01349	0,04	0,061	1
Ccnj	0,138925	-0,01766	0,048	0,07	1
Csnk2a1	0,138995	-0,02403	0,954	0,954	1
Nrxn1	0,139076	-0,00324	0,066	0,093	1
Jade2	0,139197	0,06752	0,226	0,192	1
Papd4	0,139222	-0,00416	0,275	0,333	1
Gm17690	0,139258	-0,01596	0,024	0,04	1
Mdm1	0,139281	-0,0218	0,051	0,074	1
Uevld	0,139352	-0,00438	0,077	0,105	1
Rtp4	0,13957	0,002334	0,039	0,059	1
Prkcb	0,139637	0,101986	0,389	0,371	1
Ppp1r14c	0,139703	-0,01523	0,044	0,065	1
Mrs2	0,139714	-0,02401	0,251	0,302	1
Tmtc4	0,139758	-0,03156	0,064	0,089	1
Rad54l2	0,139762	-0,01822	0,215	0,259	1
Ankle2	0,139928	-0,0152	0,288	0,34	1
Gm5141	0,140089	-0,01151	0,042	0,063	1
Zfand6	0,140174	-0,02772	0,628	0,69	1
Cnot10	0,140336	-0,00209	0,347	0,416	1
Dner	0,140366	0,034775	0,075	0,053	1
Ppp1r16a	0,140421	0,002614	0,273	0,333	1
Ssrp1	0,140473	-0,0065	0,444	0,532	1
Hoxa2	0,140598	0,037731	0,077	0,055	1
Tbc1d32	0,140625	-0,02733	0,117	0,15	1
Stk3	0,140742	-0,00647	0,171	0,215	1

Rrad	0,140785	0,028283	0,013	0,004	1
Stk17b	0,140916	0,030072	0,083	0,059	1
Lpin2	0,140918	-0,02308	0,048	0,07	1
Fxyd4	0,140961	0,013979	0,031	0,017	1
Clip1	0,14097	-0,02239	0,123	0,156	1
Smad9	0,141082	0,015459	0,031	0,017	1
Nuak2	0,14114	0,015772	0,013	0,004	1
Gmfg	0,141264	0,012465	0,009	0,002	1
Ccne1	0,141264	0,012465	0,009	0,002	1
Rnd1	0,141264	0,012465	0,009	0,002	1
Nhs	0,141264	0,016075	0,009	0,002	1
Cox15	0,141412	-0,01047	0,139	0,175	1
Brinp2	0,141481	0,010655	0,009	0,002	1
Oacyl	0,141481	0,010655	0,009	0,002	1
Lrrn2	0,141481	0,012465	0,009	0,002	1
Lck	0,141481	0,012465	0,009	0,002	1
Pgbd1	0,141496	0,013972	0,013	0,004	1
Gm30074	0,141496	0,017569	0,013	0,004	1
Hist2h2bb	0,141496	0,015772	0,013	0,004	1
Gm49201	0,141542	-0,01429	0,013	0,025	1
Arid4b	0,141553	0,070517	0,905	0,884	1
Det1	0,141587	-0,01418	0,044	0,065	1
Gm12092	0,141605	-0,02158	0,057	0,08	1
Wsb2	0,141635	-0,02246	0,604	0,688	1
Gm26615	0,141699	0,008842	0,009	0,002	1
Zfp82	0,141699	0,008842	0,009	0,002	1
Cdca2	0,141699	0,008842	0,009	0,002	1
B430319H:	0,141699	0,008842	0,009	0,002	1
Dio2	0,141699	0,008842	0,009	0,002	1
Gm11027	0,141852	0,019363	0,013	0,004	1
Scn3b	0,141852	0,015772	0,013	0,004	1
Cish	0,141852	0,013972	0,013	0,004	1
Ngef	0,141916	0,007025	0,009	0,002	1
Mov10	0,141916	0,007025	0,009	0,002	1
4930599N:	0,141916	0,007025	0,009	0,002	1
Gm26765	0,141916	0,007025	0,009	0,002	1
Smad6	0,141916	0,007025	0,009	0,002	1
Gm35818	0,141916	0,007025	0,009	0,002	1
Grhl2	0,141916	0,007025	0,009	0,002	1
AC098883.	0,141916	0,007025	0,009	0,002	1
Gm26749	0,141916	0,007025	0,009	0,002	1
Slc15a3	0,141916	0,007025	0,009	0,002	1
Nlrc4	0,142165	-0,01454	0,013	0,025	1
Gm15265	0,142208	0,015772	0,013	0,004	1
Il23a	0,142208	0,010362	0,013	0,004	1
Gm10644	0,142208	0,010362	0,013	0,004	1
9430037G(	0,142208	0,010362	0,013	0,004	1
2810408A:	0,142208	0,010362	0,013	0,004	1
Serpina9	0,142208	0,010362	0,013	0,004	1
Rap2b	0,142312	0,003638	0,15	0,192	1
Setd1a	0,142401	-0,03032	0,136	0,169	1
Myl6	0,142436	-0,02563	0,993	0,987	1

Cdk2ap1	0,142447	-0,02307	0,624	0,694	1
Pxylp1	0,142556	-0,00988	0,119	0,154	1
Gm26624	0,142565	0,008552	0,013	0,004	1
Gm36757	0,142684	-0,01943	0,053	0,076	1
Oasl2	0,142748	0,013673	0,013	0,004	1
Trip13	0,142749	0,01517	0,013	0,004	1
Mtch2	0,14277	-0,02854	0,67	0,741	1
Vmn1r81	0,142794	0,006738	0,009	0,002	1
Eif2s3x	0,142811	0,078629	0,528	0,519	1
Ppp1r9b	0,142837	-0,02315	0,417	0,477	1
Cmpk2	0,142927	0,011873	0,013	0,004	1
Larp7	0,142969	0,033412	0,229	0,291	1
Cyhr1	0,142997	-0,01957	0,85	0,907	1
Gm26617	0,143013	0,004922	0,009	0,002	1
Ptges3	0,143049	-0,02881	0,985	0,985	1
Pdgfra	0,143155	-0,01093	0,013	0,025	1
Atad2b	0,143163	0,098528	0,459	0,443	1
Hexim2	0,143244	-0,01175	0,046	0,068	1
Cdk4	0,143253	-0,02166	0,457	0,538	1
Gm10160	0,143286	0,01007	0,013	0,004	1
Ccdc186	0,143502	0,01406	0,431	0,523	1
Tmem231	0,143675	-0,01086	0,024	0,04	1
Zbtb37	0,143769	0,020386	0,272	0,331	1
Zfp503	0,143803	0,039533	0,106	0,08	1
Agtpbp1	0,143824	-0,0252	0,49	0,557	1
Gm9972	0,1439	-0,00682	0,013	0,025	1
Cacng7	0,14391	-0,01354	0,297	0,352	1
Brap	0,14393	-0,01778	0,343	0,403	1
Psd2	0,143962	-0,01067	0,174	0,217	1
Hlf	0,144062	-0,00554	0,024	0,04	1
Zfp142	0,144145	0,066848	0,198	0,169	1
Ulk4	0,144163	-0,0112	0,046	0,068	1
Srsf12	0,144229	-0,0162	0,026	0,042	1
Tnpo1	0,144267	-0,00826	0,394	0,464	1
Ralgps2	0,144293	0,054809	0,07	0,049	1
Hs6st1	0,144346	-0,01652	0,283	0,34	1
Cntn4	0,144365	-0,02308	0,048	0,07	1
Atrx	0,144508	0,074537	0,903	0,899	1
Pla2g3	0,144531	-0,01068	0,013	0,025	1
Gm10501	0,144531	-0,01068	0,013	0,025	1
Ddx24	0,14457	-0,03899	0,78	0,81	1
Rabgef1	0,144586	-0,01215	0,182	0,226	1
Faf1	0,14469	-0,01293	0,29	0,344	1
Noxo1	0,144738	-0,01061	0,024	0,04	1
Ccdc117	0,144768	-0,01337	0,213	0,257	1
Apbb2	0,144905	-0,05391	0,888	0,89	1
Mapk9	0,144917	-0,02111	0,251	0,297	1
Ptbp1	0,144977	0,081922	0,272	0,247	1
Gpatch4	0,144992	-0,02644	0,141	0,177	1
Crlf2	0,144996	-0,01287	0,026	0,042	1
Mmgt1	0,145161	0,009187	0,398	0,487	1
Slc6a8	0,145213	0,089996	0,648	0,639	1

Tnfrsf12a	0,145291	0,219902	0,127	0,101	1
Lrch1	0,145308	0,044034	0,108	0,082	1
Ints7	0,14533	-0,03281	0,387	0,447	1
Gm13031	0,145415	-0,00733	0,013	0,025	1
Zfp810	0,145415	-0,00913	0,013	0,025	1
Kyat3	0,145547	-0,02413	0,139	0,175	1
Mettl8	0,145552	0,001317	0,15	0,19	1
Tmem57	0,14557	0,001897	0,473	0,549	1
Rpl23a	0,145651	-0,00859	0,956	0,962	1
Ubfd1	0,145749	-0,01452	0,134	0,169	1
Lrch2	0,146115	-0,02777	0,204	0,245	1
Rbm41	0,146184	-0,02243	0,073	0,099	1
Nol9	0,146353	0,005909	0,086	0,116	1
Cyth1	0,146405	-0,03611	0,859	0,867	1
Usp46	0,146497	0,06365	0,305	0,272	1
Atxn7	0,146574	-0,01302	0,222	0,266	1
Dhcr7	0,146616	-0,00519	0,554	0,643	1
Dock5	0,146693	-0,02074	0,525	0,605	1
Prob1	0,14696	0,068172	0,198	0,169	1
Jcad	0,146963	0,022647	0,081	0,057	1
Pias1	0,147149	-0,00316	0,281	0,344	1
Sh3yl1	0,147207	-0,0166	0,053	0,076	1
Apaf1	0,147498	0,042743	0,094	0,07	1
Saal1	0,147719	-0,00651	0,101	0,133	1
Cables2	0,147802	0,042743	0,086	0,063	1
Erp27	0,147856	-0,01287	0,026	0,042	1
2810021J2	0,147903	-0,01965	0,059	0,082	1
Sec24a	0,147908	-0,00207	0,251	0,304	1
Arl13b	0,14811	0,031271	0,415	0,513	1
Glud1	0,148129	0,076564	0,679	0,69	1
Elmod3	0,14814	-0,01524	0,163	0,203	1
Frmd5	0,148315	-0,02144	0,683	0,77	1
Plgrkt	0,148328	-0,01199	0,264	0,316	1
Snx33	0,148527	0,096239	0,428	0,411	1
Brat1	0,148621	-0,01632	0,174	0,215	1
Mgrn1	0,148716	-0,01476	0,464	0,544	1
Kdm7a	0,148824	0,005032	0,255	0,304	1
Znrf3	0,14891	-0,01704	0,167	0,205	1
Ssx2ip	0,148943	-0,01668	0,059	0,082	1
Dock3	0,148986	-0,00779	0,31	0,367	1
Ssh3	0,14928	-0,00835	0,193	0,236	1
Rlim	0,14934	0,138126	0,505	0,494	1
Spin1	0,149352	-0,0218	0,217	0,259	1
Htra3	0,149357	-0,01652	0,172	0,211	1
Dyrk1b	0,149359	-0,01796	0,116	0,148	1
Zfp219	0,149424	0,031548	0,092	0,068	1
Lemd3	0,149477	-0,0147	0,22	0,266	1
Acot1	0,149489	0,00891	0,545	0,61	1
Rbm48	0,149495	-0,01061	0,235	0,283	1
Ankrd9	0,149706	-0,01727	0,059	0,082	1
D930048G	0,14971	0,032693	0,07	0,049	1
Bst2	0,149726	0,079508	0,051	0,034	1

Plxna3	0,149929	0,027629	0,07	0,049	1
Zfp692	0,150014	0,065216	0,211	0,181	1
Kif16b	0,15004	0,062145	0,25	0,217	1
Zkscan4	0,150085	-0,01263	0,028	0,044	1
Map10	0,150145	-0,01882	0,068	0,093	1
Adam10	0,150161	-0,04587	0,771	0,823	1
Fam76b	0,15026	5,44E-05	0,279	0,333	1
Fbxo4	0,150378	-0,00695	0,125	0,16	1
Rbl2	0,15041	-0,00517	0,472	0,546	1
Pkia	0,150578	-0,02341	0,101	0,131	1
Senp2	0,150593	0,08635	0,481	0,479	1
Ilvbl	0,150825	-0,05299	0,664	0,7	1
Exd2	0,151034	-0,01882	0,323	0,382	1
Ahcyl2	0,151048	0,092924	0,483	0,481	1
Ppp1r12a	0,151065	0,073965	0,76	0,804	1
Safb	0,151101	0,098543	0,739	0,759	1
Cdc42se1	0,151174	0,096569	0,561	0,565	1
Jph4	0,151195	-0,00988	0,67	0,709	1
Nacc2	0,151269	-0,01227	0,846	0,873	1
Dtnb	0,15132	-0,01475	0,09	0,118	1
Phf21a	0,151468	0,003609	0,361	0,426	1
Fxyd1	0,151723	-0,26893	0,194	0,222	1
Tmed10	0,15184	-0,02015	1	0,998	1
Ap3s1	0,151952	-0,01763	0,086	0,114	1
Tm9sf2	0,152065	0,061762	0,71	0,717	1
Nrm	0,152186	0,018442	0,022	0,011	1
Pcdhb22	0,152221	-0,02826	0,079	0,105	1
Gtf2i	0,152347	0,07991	0,769	0,797	1
Pdss1	0,152359	-0,01315	0,053	0,076	1
Casp8ap2	0,15236	0,022186	0,31	0,378	1
Dscaml1	0,152416	0,09023	0,622	0,654	1
Rnf220	0,152566	0,083082	0,776	0,785	1
Syn3	0,152673	-0,01287	0,028	0,044	1
Hsf5	0,152909	-0,01533	0,055	0,078	1
Afg1l	0,152959	-0,01143	0,055	0,078	1
Pus7l	0,152994	-0,00187	0,048	0,07	1
Abcc1	0,153037	-0,00731	0,18	0,222	1
Rpn2	0,153044	-0,02287	0,734	0,8	1
Cdkn2d	0,153427	-0,01503	0,563	0,633	1
Phf6	0,153436	0,003412	0,2	0,249	1
9030624JC	0,153522	0,006306	0,506	0,601	1
Nmnat3	0,153599	-0,01601	0,062	0,086	1
Zfp740	0,153601	-0,00307	0,55	0,646	1
Rexo1	0,15378	-0,01665	0,33	0,384	1
Atp8b2	0,153813	0,022736	0,044	0,027	1
Dgkh	0,153921	0,030321	0,039	0,023	1
Hdac1	0,154051	-0,0088	0,38	0,445	1
Duxbl3	0,154073	-0,01273	0,015	0,027	1
B4galt4	0,154108	-0,00908	0,028	0,044	1
Ints9	0,154124	0,063469	0,211	0,181	1
Bend6	0,154199	-0,01093	0,015	0,027	1
Mllt1	0,154254	-0,01642	0,128	0,162	1

Sf3b3	0,154312	0,0164	0,453	0,53	1
Pomt2	0,154497	-0,04168	0,229	0,27	1
Pdlim3	0,154707	-0,01297	0,015	0,027	1
Tmtc3	0,154727	-0,01629	0,138	0,173	1
Sdcbp	0,154778	-0,00747	0,428	0,498	1
Aldh3a2	0,15497	-0,0291	0,391	0,456	1
Ccdc17	0,155075	-0,01068	0,015	0,027	1
Raver1	0,155075	-0,01068	0,015	0,027	1
Nbeal2	0,155075	-0,01068	0,015	0,027	1
Tfip11	0,155166	-0,00945	0,239	0,285	1
Ube2s	0,15517	-0,02189	0,741	0,808	1
Brip1os	0,155217	0,063216	0,185	0,156	1
Mink1	0,155439	0,093095	0,686	0,694	1
Naa35	0,155518	0,003163	0,352	0,428	1
Ubqln4	0,155581	-0,00025	0,198	0,243	1
Gm16316	0,155712	-0,01093	0,015	0,027	1
Bbof1	0,155919	-0,01419	0,073	0,099	1
4632404H1	0,156227	-0,01588	0,072	0,097	1
Gm14322	0,156234	-0,01287	0,029	0,046	1
Zfp677	0,156308	-0,01969	0,068	0,093	1
Pcdhgb1	0,15635	-0,01117	0,015	0,027	1
Hps1	0,156414	-0,01319	0,077	0,103	1
Rnf111	0,156423	-0,01731	0,314	0,371	1
Wdr37	0,156765	-0,0185	0,323	0,376	1
Scube3	0,156948	0,030085	0,108	0,082	1
Med18	0,156991	0,03644	0,079	0,057	1
Rusc2	0,158196	0,095415	0,554	0,574	1
Zfp574	0,158205	-0,0155	0,11	0,141	1
Gdpd5	0,158341	0,077349	0,398	0,376	1
Odf2l	0,158375	-0,00605	0,183	0,226	1
Senp1	0,158379	-0,0037	0,235	0,281	1
Peli1	0,158514	0,025093	0,497	0,61	1
Mreg	0,158549	0,021602	0,039	0,023	1
Zranb1	0,158602	0,089671	0,585	0,591	1
Pgpep1	0,158605	0,033147	0,149	0,118	1
Map2k3	0,158888	0,059242	0,156	0,129	1
4930426L0	0,158896	-0,0115	0,007	0,017	1
Gnb1l	0,158896	-0,0115	0,007	0,017	1
Iqch	0,158896	-0,01356	0,007	0,017	1
Gas2l3	0,158896	-0,02384	0,007	0,017	1
Spin4	0,158934	-0,01536	0,031	0,049	1
Cant1	0,158948	-0,00106	0,317	0,378	1
Wtap	0,159083	-0,00822	0,369	0,432	1
Mknk2	0,159092	0,054797	0,154	0,127	1
Usp4	0,159106	-0,01936	0,363	0,418	1
Pde11a	0,159117	0,014286	0,062	0,042	1
Gnl3l	0,159124	-0,01698	0,497	0,589	1
Ctsa	0,159159	0,051124	0,903	0,888	1
Cmip	0,159188	0,113104	0,606	0,614	1
Xpo6	0,15922	0,102545	0,352	0,34	1
Tmem254a	0,159375	-0,01713	0,033	0,051	1
Elmo2	0,159553	-0,02589	0,187	0,224	1

Pcdhb18	0,159738	-0,02343	0,084	0,112	1
Ino80e	0,159849	-0,01381	0,294	0,346	1
Baz2b	0,159869	-0,04215	0,793	0,819	1
Col20a1	0,159915	-0,00812	0,007	0,017	1
Phb	0,160053	0,000741	0,552	0,658	1
Dvl3	0,160186	0,003618	0,316	0,373	1
Clec2l	0,160253	-0,00968	0,007	0,017	1
Gm15510	0,160253	-0,00968	0,007	0,017	1
2610507B1	0,160474	-0,03905	0,615	0,673	1
Hspa13	0,160872	-0,01115	0,365	0,424	1
Camkmt	0,160873	-0,01351	0,07	0,095	1
6430573P(	0,160932	-0,0076	0,007	0,017	1
Pik3c2a	0,161001	-0,01604	0,512	0,582	1
Fbxo10	0,16108	0,024014	0,094	0,07	1
Meis3	0,161171	0,020764	0,033	0,019	1
Pkd2	0,161212	-0,00347	0,328	0,388	1
Gm20342	0,161216	0,026089	0,231	0,285	1
Dbnl	0,161598	-0,01272	0,488	0,557	1
Kcnb1	0,161619	-0,00786	0,007	0,017	1
Zfp608	0,161716	0,000125	0,35	0,409	1
Sart3	0,161844	-0,01577	0,209	0,249	1
Grk2	0,162292	-0,00035	0,272	0,329	1
Zfp433	0,162303	-0,00036	0,007	0,017	1
Gm5784	0,162303	-0,00036	0,007	0,017	1
Zfand4	0,162342	-0,01936	0,037	0,055	1
Npepl1	0,16243	-0,01009	0,25	0,297	1
Ndn	0,162439	-0,02955	0,222	0,262	1
Usp16	0,162491	0,008926	0,396	0,473	1
Ccdc43	0,16256	-0,01255	0,182	0,222	1
Nkx2-2	0,162848	0,010911	0,475	0,568	1
Gin1	0,1629	-0,0022	0,149	0,184	1
Map3k5	0,162954	0,032473	0,108	0,082	1
Cd34	0,163011	0,036255	0,182	0,15	1
Senp7	0,163011	0,026079	0,431	0,527	1
Foxj3	0,163019	0,085179	0,277	0,251	1
Pcdhgc4	0,163095	0,034695	0,073	0,053	1
Pik3ca	0,163207	0,090945	0,433	0,426	1
Hspa9	0,163217	0,07561	0,802	0,814	1
Plvap	0,163385	-0,00765	0,103	0,076	1
1810013L2	0,163416	0,000183	0,27	0,321	1
Abhd17b	0,163444	-0,03442	0,822	0,842	1
Cyfip1	0,163486	0,086934	0,541	0,534	1
Myo1c	0,163603	0,028413	0,051	0,034	1
Elmod2	0,163624	-0,02044	0,251	0,295	1
Tmem169	0,163761	-0,00821	0,068	0,093	1
Invs	0,163793	-0,02274	0,171	0,207	1
Scpep1	0,163903	0,07884	0,257	0,23	1
E2f6	0,16417	-0,01665	0,16	0,196	1
Smim3	0,164219	-0,03038	0,059	0,08	1
Fbxo27	0,1648	-0,0033	0,033	0,051	1
A230072E1	0,164832	-0,01093	0,017	0,03	1
Cmtr1	0,165067	0,033605	0,103	0,078	1

Akap11	0,165208	0,057323	0,787	0,816	1
Prdm9	0,165218	-0,01525	0,017	0,03	1
Rtn2	0,165242	-0,01805	0,037	0,055	1
Slf2	0,165295	0,105311	0,433	0,424	1
Lrrc47	0,165323	-0,01909	0,262	0,31	1
Ift81	0,165364	-0,00763	0,123	0,156	1
Zmym4	0,166346	0,016238	0,387	0,46	1
Wisp1	0,166576	-0,00429	0,031	0,049	1
Rmnd5a	0,16698	0,085017	0,499	0,487	1
Nav3	0,167092	-0,01431	0,125	0,158	1
Psmd5	0,167277	-0,01648	0,229	0,272	1
Syngr1	0,167448	-0,00922	0,117	0,15	1
Il11ra1	0,167587	-0,03163	0,15	0,184	1
Ltbr	0,167697	0,052428	0,161	0,133	1
Atp11b	0,16778	0,101763	0,483	0,494	1
Scyl1	0,167816	-0,01784	0,292	0,344	1
Snx16	0,167853	-0,00566	0,253	0,3	1
Fyn	0,167986	0,101248	0,536	0,553	1
Zbtb5	0,167987	-0,01081	0,119	0,152	1
Rundc3b	0,168107	0,027633	0,046	0,03	1
Dse	0,16853	-0,0207	0,04	0,059	1
Endov	0,1686	-0,022	0,11	0,139	1
Btaf1	0,1687	-0,01326	0,255	0,3	1
Mad2l1	0,168724	-0,02506	0,051	0,072	1
Cnot6	0,168839	0,129754	0,4	0,414	1
Rbsn	0,169432	-0,00134	0,327	0,384	1
Gpr137c	0,169517	0,027364	0,292	0,357	1
Cse1l	0,169617	-0,01975	0,25	0,295	1
Zw10	0,169818	-0,01812	0,15	0,186	1
Pspc1	0,169824	0,006764	0,316	0,376	1
Eya2	0,170012	-0,03025	0,066	0,089	1
Gdpgp1	0,170022	-0,02289	0,048	0,068	1
Ctnnd2	0,170116	-0,0207	0,04	0,059	1
Btg3	0,170133	0,036968	0,053	0,036	1
Chst8	0,170708	0,07324	0,332	0,302	1
Mettl15	0,170994	-0,01053	0,128	0,162	1
Lrrtm2	0,170999	0,143129	0,262	0,238	1
Zdhhc17	0,171531	-0,01287	0,694	0,774	1
Shmt2	0,171609	-0,01258	0,158	0,194	1
0610010F(	0,171774	-0,01637	0,226	0,268	1
Zfp784	0,172041	-0,00705	0,037	0,055	1
Cog5	0,172064	-0,0066	0,295	0,352	1
Gm17518	0,172107	-0,01322	0,018	0,032	1
Ncoa1	0,172137	0,09103	0,38	0,361	1
Pcsk2os1	0,17249	-0,01752	0,018	0,032	1
Atp6v1h	0,17271	-0,00881	0,549	0,627	1
Uba1	0,172811	0,074227	0,749	0,762	1
Cyb5r1	0,172862	0,097532	0,233	0,211	1
Gm11032	0,172969	0,01696	0,018	0,008	1
Map3k14	0,172969	0,01696	0,018	0,008	1
Cks2	0,172969	0,01696	0,018	0,008	1
Zfyve28	0,172969	0,018748	0,018	0,008	1

Pmvk	0,173055	0,013936	0,47	0,568	1
Trim25	0,173162	0,049599	0,134	0,108	1
Klhl24	0,173197	0,084831	0,706	0,7	1
Gm11508	0,173243	-0,00809	0,018	0,032	1
Zc3h13	0,173281	8,83E-05	0,472	0,553	1
Dpp7	0,173407	0,084247	0,239	0,215	1
Osbpl6	0,173469	0,028393	0,066	0,046	1
Gm43254	0,173636	0,01517	0,018	0,008	1
St6galnac3	0,17365	-0,02029	0,714	0,804	1
1110008L1	0,173716	-0,00855	0,037	0,055	1
Tox3	0,173753	-0,01191	0,018	0,032	1
Wdfy2	0,173767	0,086122	0,378	0,361	1
Papd7	0,173912	-0,00282	0,169	0,207	1
Ska2	0,174002	-0,02375	0,138	0,169	1
Il12rb1	0,174098	0,02449	0,103	0,078	1
Tmem150a	0,174189	-0,01258	0,273	0,323	1
4933411E(	0,174305	0,01696	0,018	0,008	1
Fez2	0,174313	-0,02131	0,538	0,612	1
Nr2f1	0,174477	0,016658	0,018	0,008	1
Slamf1	0,174486	0,045825	0,04	0,025	1
Cdk5rap1	0,174504	-0,02569	0,051	0,072	1
Chuk	0,174515	-0,01072	0,483	0,549	1
Ythdc2	0,174518	0,006357	0,295	0,35	1
Tpm1	0,1748	-0,01683	0,615	0,662	1
Slc25a47	0,174887	-0,00987	0,018	0,032	1
Tnnt2	0,174974	0,011579	0,018	0,008	1
Tm4sf1	0,174974	0,011579	0,018	0,008	1
Chst3	0,174999	-0,0176	0,116	0,146	1
Ganab	0,174999	0,109165	0,47	0,468	1
3100002H(	0,175149	0,016658	0,018	0,008	1
Tbc1d13	0,175161	-0,01283	0,172	0,209	1
Phf1	0,175419	-0,01326	0,202	0,241	1
Cdc16	0,175602	-0,00148	0,305	0,357	1
Gla	0,175819	-0,00852	0,123	0,156	1
Rorb	0,175822	0,01308	0,018	0,008	1
Siah1b	0,175827	-0,01254	0,04	0,059	1
Btbd16	0,175994	0,041749	0,068	0,049	1
Cebpz	0,176041	0,007358	0,534	0,62	1
Ptov1	0,176121	-0,0325	0,833	0,869	1
Rb1cc1	0,176159	0,077918	0,765	0,741	1
Wdhd1	0,176179	0,053503	0,055	0,038	1
B9d2	0,176183	-0,0218	0,066	0,089	1
Gm5535	0,176486	-0,02266	0,057	0,078	1
Ppp4r1	0,176605	0,054172	0,22	0,19	1
Topors	0,176656	0,120379	0,382	0,367	1
Shc4	0,176784	-0,01465	0,044	0,063	1
Nudt10	0,176838	-0,01962	0,046	0,065	1
Socs6	0,177055	0,10467	0,299	0,274	1
Opa3	0,177085	0,001237	0,303	0,357	1
Rfx1	0,177282	0,037877	0,099	0,076	1
Htt	0,177354	0,088971	0,372	0,354	1
Maneal	0,177525	-0,02025	0,141	0,175	1

Utp4	0,177558	-0,01442	0,125	0,156	1
Rab21	0,17778	0,060355	0,921	0,916	1
Dnmt1	0,178037	0,020128	0,242	0,3	1
Prmt1	0,178056	-0,00031	0,657	0,749	1
Zfp729b	0,178146	0,001639	0,145	0,181	1
AW554918	0,178892	-0,01802	0,171	0,207	1
Lmtk3	0,179025	-0,0058	0,039	0,057	1
Gucy1a1	0,179048	-0,01175	0,009	0,019	1
Gm20457	0,179048	-0,01175	0,009	0,019	1
Lppos	0,179048	-0,01175	0,009	0,019	1
B230217O	0,179048	-0,01175	0,009	0,019	1
Gm45844	0,179061	-0,00754	0,04	0,059	1
Thbs2	0,179182	0,033514	0,075	0,055	1
Trabd	0,179198	-0,01272	0,277	0,329	1
Etfdh	0,179247	-0,01041	0,239	0,287	1
Krba1	0,179429	-0,01216	0,152	0,188	1
Il6st	0,179509	0,115116	0,532	0,551	1
Filip1	0,179567	-0,02429	0,009	0,019	1
Fhl3	0,179903	-0,00968	0,009	0,019	1
Spint2	0,179903	-0,00968	0,009	0,019	1
Usp45	0,180031	-0,00238	0,149	0,184	1
Ppp1ca	0,180113	-0,03928	0,872	0,882	1
Ercc6	0,18034	-0,01669	0,105	0,133	1
Snapc4	0,180353	-0,02052	0,072	0,095	1
Eid2	0,180433	-0,02052	0,066	0,089	1
Tmem140	0,180503	0,044807	0,035	0,021	1
Col4a3bp	0,180815	0,08192	0,411	0,399	1
Hyou1	0,180873	-0,01238	0,229	0,27	1
Arhgef10	0,181093	-0,03131	0,954	0,96	1
Cav1	0,181138	-0,01712	0,108	0,137	1
5033421B(	0,181167	0,031322	0,061	0,042	1
Ccser1	0,181193	-0,0137	0,02	0,034	1
Olfr655	0,18129	-0,01158	0,009	0,019	1
AC163040.	0,181457	-0,00605	0,009	0,019	1
Ccnyl1	0,181468	-0,01869	0,194	0,232	1
St3gal3	0,181543	0,08418	0,325	0,304	1
Zfp189	0,181777	-0,01418	0,051	0,072	1
Pcdhb16	0,181793	-0,02447	0,083	0,108	1
Cd59b	0,181932	-0,01882	0,059	0,08	1
1700008J(	0,181969	-0,01855	0,051	0,072	1
Mgea5	0,182151	-0,00909	0,646	0,719	1
AC161165.	0,182154	-0,00812	0,009	0,019	1
Clasp1	0,182219	0,057571	0,204	0,175	1
Ptcd1	0,182247	0,001595	0,13	0,165	1
Usp49	0,182315	0,032062	0,075	0,055	1
Hk2	0,182331	-0,00656	0,02	0,034	1
Repin1	0,182454	-0,01528	0,106	0,135	1
Tmem132c	0,182577	-0,01397	0,066	0,089	1
Ighmbp2	0,182613	-0,01372	0,048	0,068	1
Polr2b	0,182679	0,02413	0,336	0,401	1
Nop58	0,182766	0,119888	0,294	0,272	1
Iqcg	0,182821	-0,00962	0,02	0,034	1

Eva1c	0,182821	-0,00962	0,02	0,034	1
Mogat1	0,182918	0,023973	0,035	0,021	1
Gm45890	0,183021	-0,00605	0,009	0,019	1
Tvp23bos	0,183021	-0,00605	0,009	0,019	1
Trpv4	0,183024	0,02294	0,048	0,032	1
Stk40	0,18305	0,074134	0,165	0,139	1
Cct6b	0,1832	-0,00656	0,009	0,019	1
Dennd6a	0,183255	-0,01568	0,261	0,304	1
Ankrd27	0,183269	0,06938	0,206	0,179	1
Zcchc17	0,183445	-0,03626	0,684	0,713	1
Zdhhc14	0,183584	0,071909	0,69	0,694	1
Osbpl8	0,183618	-0,00605	0,231	0,276	1
Sp3	0,183846	0,082594	0,492	0,494	1
Rfc1	0,18423	-0,01199	0,635	0,713	1
Zfp719	0,184291	-0,01816	0,068	0,091	1
Zbtb10	0,184304	0,0527	0,143	0,118	1
Bhlhe22	0,184312	-0,011	0,004	0,011	1
Kcna6	0,184377	-0,06003	0,763	0,825	1
Tcte2	0,184494	-0,00824	0,055	0,076	1
Gm45869	0,184594	-0,00424	0,009	0,019	1
Pidd1	0,184805	-0,00892	0,004	0,011	1
Lingo3	0,184805	-0,00892	0,004	0,011	1
Gm20522	0,184805	-0,00892	0,004	0,011	1
Xkrx	0,184805	-0,011	0,004	0,011	1
Tmem147c	0,184851	-0,00783	0,02	0,034	1
Me1	0,18515	-0,02937	0,428	0,496	1
Pex5	0,18518	-0,02324	0,22	0,259	1
Rpl31	0,185234	-0,02022	0,961	0,96	1
Galnt3	0,185298	-0,00683	0,004	0,011	1
Tfpi2	0,185298	-0,00683	0,004	0,011	1
Lmntd1	0,185298	-0,00683	0,004	0,011	1
Mtag2	0,185298	-0,00683	0,004	0,011	1
Igf1	0,185298	-0,00683	0,004	0,011	1
Palld	0,185298	-0,00683	0,004	0,011	1
Ncan	0,185298	-0,00683	0,004	0,011	1
Gm9725	0,185298	-0,00683	0,004	0,011	1
Opn4	0,185298	-0,00683	0,004	0,011	1
Mlip	0,185298	-0,00683	0,004	0,011	1
Mgat5b	0,185298	-0,00683	0,004	0,011	1
Ccdc40	0,185298	-0,00683	0,004	0,011	1
Hist1h3f	0,185298	-0,00683	0,004	0,011	1
D630036H	0,185298	-0,00683	0,004	0,011	1
Bcl11b	0,185298	-0,00683	0,004	0,011	1
Smco1	0,185298	-0,00683	0,004	0,011	1
Cdo1	0,185298	-0,00683	0,004	0,011	1
Epn2	0,185315	0,068205	0,982	0,985	1
Hs2st1	0,185494	0,03132	0,33	0,395	1
Wfdc12	0,185796	-0,00942	0,004	0,011	1
Cdh11	0,185844	0,003351	0,611	0,696	1
Ano1	0,186044	-0,00709	0,004	0,011	1
Gm20463	0,186044	-0,00709	0,004	0,011	1
Adam28	0,186095	0,019303	0,029	0,017	1

Ccdc57	0,18615	0,016356	0,024	0,013	1
Fam210b	0,186189	-0,02299	0,125	0,154	1
Gm14424	0,186227	0,021909	0,035	0,021	1
Prkd1	0,186293	-0,00345	0,004	0,011	1
Micu3	0,186306	0,003877	0,367	0,437	1
Mlf1	0,186351	0,021909	0,035	0,021	1
Saa3	0,186351	0,018394	0,035	0,021	1
Cand1	0,186522	0,073591	0,352	0,333	1
Sec16b	0,18654	-0,005	0,004	0,011	1
2310040G:	0,18654	-0,005	0,004	0,011	1
Gm12227	0,18654	-0,005	0,004	0,011	1
Myh3	0,18654	-0,005	0,004	0,011	1
Afmid	0,18654	-0,005	0,004	0,011	1
Gm47167	0,18654	-0,005	0,004	0,011	1
Gstcd	0,186745	-0,01278	0,095	0,122	1
Tbc1d23	0,186764	0,002776	0,406	0,473	1
Dot1l	0,186841	0,002158	0,468	0,542	1
Trmt1l	0,186874	0,015974	0,317	0,376	1
Spryd3	0,186981	-0,01953	0,183	0,219	1
Akap1	0,187362	0,065866	0,206	0,179	1
Bmt2	0,187381	-0,00412	0,255	0,302	1
Braf	0,187438	0,013281	0,382	0,454	1
Atcayos	0,187457	0,007313	0,004	0	1
Gm47604	0,187457	0,007313	0,004	0	1
Fam83h	0,187457	0,007313	0,004	0	1
Slc7a4	0,187457	0,007313	0,004	0	1
Rsph1	0,187457	0,007313	0,004	0	1
Gm38033	0,187457	0,003663	0,004	0	1
March4	0,187457	0,003663	0,004	0	1
Cd247	0,187457	0,003663	0,004	0	1
Esrrg	0,187457	0,003663	0,004	0	1
Camk1d	0,187457	0,003663	0,004	0	1
Sfmbt2	0,187457	0,003663	0,004	0	1
Tor4a	0,187457	0,003663	0,004	0	1
Col5a1	0,187457	0,003663	0,004	0	1
Ak8	0,187457	0,003663	0,004	0	1
Kcnj3	0,187457	0,003663	0,004	0	1
Cers6	0,187457	0,003663	0,004	0	1
Dlx1	0,187457	0,003663	0,004	0	1
Pde1a	0,187457	0,003663	0,004	0	1
Ano3	0,187457	0,003663	0,004	0	1
Oip5	0,187457	0,003663	0,004	0	1
2010308F(	0,187457	0,003663	0,004	0	1
Sox3	0,187457	0,003663	0,004	0	1
C030034L1	0,187457	0,003663	0,004	0	1
Ccna2	0,187457	0,003663	0,004	0	1
Crabp2	0,187457	0,003663	0,004	0	1
Lrrc39	0,187457	0,003663	0,004	0	1
Gm42457	0,187457	0,003663	0,004	0	1
Akna	0,187457	0,003663	0,004	0	1
Matn1	0,187457	0,003663	0,004	0	1
Gm26648	0,187457	0,003663	0,004	0	1

Gm10419	0,187457	0,003663	0,004	0	1
Lhx5	0,187457	0,003663	0,004	0	1
Cped1	0,187457	0,003663	0,004	0	1
Fbxo41	0,187457	0,003663	0,004	0	1
Cxcl12	0,187457	0,003663	0,004	0	1
9430041J1	0,187457	0,003663	0,004	0	1
C5ar2	0,187457	0,003663	0,004	0	1
Gm45187	0,187457	0,003663	0,004	0	1
Gm10605	0,187457	0,003663	0,004	0	1
Trim6	0,187457	0,003663	0,004	0	1
Olfr678	0,187457	0,003663	0,004	0	1
Mical2	0,187457	0,003663	0,004	0	1
Ifitm2	0,187457	0,003663	0,004	0	1
Mtfr2	0,187457	0,003663	0,004	0	1
Sh3rf3	0,187457	0,003663	0,004	0	1
Adora2a	0,187457	0,003663	0,004	0	1
Dyrk2	0,187457	0,003663	0,004	0	1
Gm44956	0,187457	0,003663	0,004	0	1
Csmd1	0,187457	0,003663	0,004	0	1
Smim18	0,187457	0,003663	0,004	0	1
Pdgfrl	0,187457	0,003663	0,004	0	1
4932416K2	0,187457	0,003663	0,004	0	1
Dnaaf1	0,187457	0,003663	0,004	0	1
Gm20735	0,187457	0,003663	0,004	0	1
Piwil2	0,187457	0,003663	0,004	0	1
Mmp12	0,187457	0,003663	0,004	0	1
Slc37a2	0,187457	0,003663	0,004	0	1
Bmp5	0,187457	0,003663	0,004	0	1
Tpbg	0,187457	0,003663	0,004	0	1
Gm47950	0,187457	0,003663	0,004	0	1
Osbpl10	0,187457	0,003663	0,004	0	1
Fam183b	0,187457	0,003663	0,004	0	1
Rasd1	0,187457	0,003663	0,004	0	1
Rilp	0,187457	0,003663	0,004	0	1
Ush1g	0,187457	0,003663	0,004	0	1
Plekhd1	0,187457	0,003663	0,004	0	1
Gm15941	0,187457	0,003663	0,004	0	1
4930544F0	0,187457	0,003663	0,004	0	1
Gm29019	0,187457	0,003663	0,004	0	1
Prr5	0,187457	0,003663	0,004	0	1
Gm30085	0,187457	0,003663	0,004	0	1
Ccdc184	0,187457	0,003663	0,004	0	1
Rtl10	0,187457	0,003663	0,004	0	1
D930030I0	0,187457	0,003663	0,004	0	1
Lca5l	0,187457	0,003663	0,004	0	1
Ager	0,187457	0,003663	0,004	0	1
B430306N0	0,187457	0,003663	0,004	0	1
Vav1	0,187457	0,003663	0,004	0	1
Best1	0,187457	0,003663	0,004	0	1
Kalrn	0,187458	0,021779	0,004	0	1
Frmd3	0,187458	0,012762	0,004	0	1
H2-M3	0,187458	0,012762	0,004	0	1

Nrip3	0,187458	0,009132	0,004	0	1
Hes1	0,187458	0,009132	0,004	0	1
Nmbr	0,187458	0,007313	0,004	0	1
Ccdc162	0,187458	0,007313	0,004	0	1
Cdkl1	0,187458	0,007313	0,004	0	1
Arg2	0,187458	0,007313	0,004	0	1
Cfh	0,187458	0,005489	0,004	0	1
Dusp9	0,187458	0,005489	0,004	0	1
Arhgap4	0,187458	0,005489	0,004	0	1
Vtcn1	0,187458	0,005489	0,004	0	1
Atg4a-ps	0,187458	0,005489	0,004	0	1
Adora3	0,187458	0,005489	0,004	0	1
B4galt1	0,187458	0,005489	0,004	0	1
0610043K1	0,187458	0,005489	0,004	0	1
Tctex1d1	0,187458	0,005489	0,004	0	1
Artn	0,187458	0,005489	0,004	0	1
Hes5	0,187458	0,005489	0,004	0	1
Nptx2	0,187458	0,005489	0,004	0	1
Dysf	0,187458	0,005489	0,004	0	1
Gabrb3	0,187458	0,005489	0,004	0	1
Ctsc	0,187458	0,005489	0,004	0	1
Pde2a	0,187458	0,005489	0,004	0	1
Cracr2b	0,187458	0,005489	0,004	0	1
Lrriq1	0,187458	0,005489	0,004	0	1
Dusp4	0,187458	0,005489	0,004	0	1
Olfr374	0,187458	0,005489	0,004	0	1
Snx20	0,187458	0,005489	0,004	0	1
Cpne6	0,187458	0,005489	0,004	0	1
Ifi47	0,187458	0,005489	0,004	0	1
Arl5c	0,187458	0,005489	0,004	0	1
Fzd2	0,187458	0,005489	0,004	0	1
Fmnl1	0,187458	0,005489	0,004	0	1
Lrr1	0,187458	0,005489	0,004	0	1
Gm28370	0,187458	0,005489	0,004	0	1
Efcab1	0,187458	0,005489	0,004	0	1
March3	0,187458	0,005489	0,004	0	1
Neurl4	0,187528	0,073934	0,292	0,268	1
Mfsd4b1	0,187686	-0,01776	0,024	0,038	1
Tmem198	0,187788	-0,00318	0,004	0,011	1
Gm12905	0,187788	-0,00318	0,004	0,011	1
Cftr	0,187788	-0,00318	0,004	0,011	1
Angpt1	0,187788	-0,00318	0,004	0,011	1
Zfp109	0,187788	-0,00136	0,004	0,011	1
Gins2	0,187806	-0,01453	0,088	0,114	1
Tars	0,187909	-0,01965	0,224	0,264	1
Sema4c	0,188008	0,071731	0,31	0,285	1
Zfp52	0,188132	0,012491	0,024	0,013	1
Ppp6r1	0,188135	-0,01509	0,29	0,338	1
Sntb2	0,1884	-0,0143	0,055	0,076	1
Wdr24	0,188485	-0,01453	0,064	0,086	1
Camta2	0,188669	-0,00477	0,18	0,217	1
Tcf20	0,188693	-0,01536	0,222	0,262	1

Lrpap1	0,18886	-0,00931	0,646	0,717	1
Snx4	0,189029	-0,01484	0,305	0,357	1
Stxbp4	0,189091	-0,01109	0,106	0,135	1
Slc7a6	0,18913	-0,01453	0,086	0,112	1
Irak2	0,189398	-0,01315	0,059	0,08	1
Fam91a1	0,18942	0,087436	0,411	0,399	1
Gm12802	0,189446	-0,00987	0,022	0,036	1
Slc12a9	0,189456	0,084362	0,459	0,454	1
Vps13c	0,189466	0,016308	0,264	0,319	1
Ak3	0,189469	0,041313	0,921	0,878	1
Rps16	0,189694	0,005843	0,993	0,992	1
Socs7	0,189862	0,068734	0,182	0,156	1
Klc2	0,190117	0,031125	0,084	0,063	1
Galc	0,190492	0,096354	0,477	0,473	1
Tsc1	0,190506	0,067993	0,558	0,574	1
1700017B(	0,190856	0,026694	0,055	0,038	1
St6galnac6	0,191062	-0,00848	0,057	0,078	1
Lsm14b	0,191157	-0,00824	0,314	0,371	1
Eif5a2	0,191226	0,003192	0,207	0,253	1
Slc35d1	0,191317	-0,01662	0,086	0,112	1
Exo5	0,19137	-0,00752	0,051	0,072	1
Asic1	0,191439	-0,01394	0,024	0,038	1
Axin2	0,191516	-0,01734	0,068	0,091	1
B930082K(	0,191795	0,035986	0,055	0,038	1
Ago3	0,191982	0,074676	0,659	0,66	1
Crmp1	0,192088	0,030932	0,084	0,063	1
Wrnip1	0,192191	-0,01347	0,229	0,272	1
Klhl9	0,192465	0,009953	0,404	0,481	1
Slc30a7	0,192799	-0,00779	0,15	0,184	1
Zfp691	0,192931	-0,008	0,053	0,074	1
Zfp110	0,192952	0,016545	0,262	0,316	1
Kank2	0,193477	-0,0116	0,101	0,129	1
Abca7	0,193513	-0,01009	0,121	0,152	1
Enkd1	0,193835	0,031731	0,042	0,027	1
Gpt2	0,193865	-0,01138	0,255	0,297	1
Smarcb1	0,193998	0,001392	0,582	0,673	1
Fam185a	0,19403	-0,01596	0,026	0,04	1
Slc16a6	0,194071	0,001639	0,132	0,165	1
Ctif	0,194277	-0,01685	0,237	0,278	1
Clk2	0,19433	0,000681	0,215	0,257	1
Nudt11	0,194342	-0,00674	0,101	0,129	1
Spryd7	0,194756	-0,0049	0,378	0,439	1
Angptl2	0,194968	0,022626	0,015	0,006	1
Adgrb2	0,195246	-0,00788	0,108	0,137	1
Spdya	0,195271	-0,01086	0,024	0,038	1
Rps20	0,195313	-0,01104	0,976	0,989	1
Aup1	0,195502	-0,01643	0,536	0,603	1
Ano10	0,19551	-0,01191	0,38	0,437	1
Ssna1	0,195553	0,081074	0,655	0,677	1
Polr1b	0,195763	0,036186	0,095	0,074	1
Dennd2a	0,195813	-0,02809	0,174	0,207	1
Scamp2	0,195897	0,075732	0,64	0,654	1

Trp53rkb	0,196237	-0,0099	0,073	0,097	1
Tmsb15b2	0,196747	-0,00993	0,011	0,021	1
Plcg2	0,196747	-0,00993	0,011	0,021	1
Vcan	0,196747	-0,00993	0,011	0,021	1
Cenpk	0,196747	-0,00993	0,011	0,021	1
AC132444.	0,196747	-0,00993	0,011	0,021	1
Trim12c	0,196821	0,013673	0,015	0,006	1
Lgr5	0,196821	0,01547	0,015	0,006	1
Vav3	0,196823	0,020532	0,015	0,006	1
Serinc3	0,196884	0,057261	0,912	0,914	1
Trim28	0,196926	-0,01428	0,495	0,576	1
1700025G(	0,197205	-0,00545	0,435	0,517	1
Zdhhc1	0,197348	-0,03159	0,143	0,173	1
Smad4	0,197374	0,091408	0,453	0,46	1
Gm48914	0,197441	0,011873	0,015	0,006	1
Vwa3b	0,197442	-0,01018	0,011	0,021	1
Phyhd1	0,197442	-0,01018	0,011	0,021	1
Fzd9	0,197488	-0,00656	0,024	0,038	1
Dusp10	0,197797	0,165082	0,417	0,418	1
Fam13b	0,197865	-0,00781	0,239	0,285	1
Ick	0,197896	-0,00605	0,536	0,612	1
Anxa9	0,198061	0,01007	0,015	0,006	1
Sh3d21	0,198061	0,01007	0,015	0,006	1
Gm26809	0,198061	0,01007	0,015	0,006	1
Olfr539	0,198061	0,01007	0,015	0,006	1
Gm15965	0,198061	0,01007	0,015	0,006	1
Togaram1	0,198083	-0,0241	0,16	0,192	1
Anxa7	0,19812	0,068734	0,182	0,156	1
Slc16a10	0,198273	0,01517	0,015	0,006	1
Tatdn1	0,198288	-0,01793	0,189	0,226	1
Gm36445	0,198475	-0,00631	0,011	0,021	1
Ripk4	0,198475	-0,00812	0,011	0,021	1
Hcn1	0,198482	0,014572	0,015	0,006	1
Acsl1	0,198549	-0,02787	0,681	0,734	1
Gm10791	0,198896	0,040527	0,112	0,089	1
Xkr4	0,198925	0,004725	0,121	0,152	1
Ramp2	0,199	-0,00862	0,011	0,021	1
Gm48022	0,199173	-0,00837	0,011	0,021	1
Abr	0,199346	-0,00656	0,011	0,021	1
Appl2	0,199431	0,021089	0,561	0,643	1
Pck2	0,199521	0,009489	0,015	0,006	1
Myadm	0,199702	-0,00698	0,095	0,122	1
Gm34788	0,19973	0,007975	0,015	0,006	1
Kat6b	0,199751	0,004153	0,215	0,257	1
Zfp354c	0,199797	-0,01863	0,13	0,16	1
E130309D(	0,199881	-0,02001	0,132	0,162	1
Fuca2	0,200124	-0,01209	0,158	0,192	1
Kif20b	0,200135	-0,00352	0,024	0,038	1
Gm26682	0,200142	0,030643	0,037	0,023	1
Gm42421	0,200213	-0,00631	0,011	0,021	1
Pcdhgc3	0,200213	-0,00631	0,011	0,021	1
H3f3aos	0,200213	-0,0027	0,011	0,021	1

Gm1976	0,200213	-0,0027	0,011	0,021	1
Pdxk	0,200299	0,093215	0,317	0,295	1
Gm49130	0,200322	-0,01037	0,026	0,04	1
Fam193b	0,200556	0,091399	0,376	0,354	1
Dclre1a	0,200688	-0,00559	0,106	0,135	1
Gm29325	0,200706	0,035556	0,088	0,068	1
Cldn12	0,200913	-0,00414	0,072	0,095	1
Ppp2r2d	0,201081	0,000517	0,497	0,58	1
Cfdp1	0,201309	-0,02456	0,75	0,791	1
Slc9a8	0,20142	-0,00841	0,244	0,289	1
Git2	0,201582	0,094252	0,587	0,589	1
Lig1	0,201598	-0,00123	0,248	0,293	1
Rps8	0,201714	0,076106	1	1	1
Mcu	0,201743	-0,0226	0,119	0,148	1
Fam3c	0,201952	0,038259	0,222	0,19	1
Rhpn2	0,20226	-7,7E-05	0,294	0,348	1
Bcl2l13	0,202286	0,042743	0,143	0,118	1
Pi4ka	0,202446	-0,01017	0,402	0,462	1
Parp1	0,202694	-0,00481	0,317	0,376	1
Crybg2	0,203106	0,029944	0,064	0,046	1
Naprt	0,203241	0,017716	0,062	0,044	1
Tpmt	0,203319	-0,01093	0,165	0,2	1
Prkacb	0,203323	0,055085	0,991	0,979	1
Vkorc1l1	0,20336	0,087432	0,354	0,344	1
Haus2	0,203423	-0,00157	0,314	0,369	1
Gab3	0,203548	-0,00938	0,011	0,021	1
Agfg1	0,203605	0,012762	0,499	0,574	1
Ube4b	0,203816	0,09127	0,424	0,418	1
Qpct	0,203926	0,116277	0,495	0,508	1
Armc5	0,203994	-0,0218	0,088	0,112	1
Nat2	0,204186	-0,01311	0,028	0,042	1
Lrig1	0,204241	-0,00629	0,07	0,093	1
AC123724.	0,204318	0,023662	0,037	0,023	1
Cep162	0,204355	0,002515	0,176	0,215	1
Pcsk2	0,204503	0,006632	0,119	0,15	1
Nkx2-2os	0,20476	-0,00175	0,028	0,042	1
Def8	0,204911	-0,00812	0,2	0,241	1
Plcg1	0,205201	-0,01943	0,211	0,249	1
Pfkfb4	0,205338	0,070717	0,191	0,167	1
Slc41a3	0,205617	0,023551	0,05	0,034	1
Uckl1os	0,205723	0,023662	0,037	0,023	1
Ascc2	0,205832	-0,02362	0,198	0,234	1
Mms19	0,206328	0,047763	0,193	0,165	1
Cep83	0,206469	-0,01375	0,218	0,257	1
Mrps2	0,206824	-0,01954	0,106	0,133	1
Aldh4a1	0,207098	-0,00312	0,125	0,156	1
Lig3	0,207117	0,005668	0,461	0,538	1
5430431A:	0,207157	0,035093	0,081	0,061	1
Rps26	0,207337	0,032916	0,985	0,992	1
Chic1	0,207476	-0,00168	0,222	0,264	1
Trove2	0,207511	0,118205	0,613	0,639	1
Gpr173	0,207814	-0,00908	0,028	0,042	1

Suz12	0,207849	0,021018	0,339	0,405	1
Nadsyn1	0,208404	-0,01536	0,033	0,049	1
Rab5b	0,208671	-0,01816	0,618	0,705	1
Ccdc173	0,208677	-0,00248	0,07	0,093	1
Strn4	0,208778	0,091839	0,631	0,652	1
Cad	0,208821	0,032168	0,088	0,068	1
Cdc73	0,209358	0,086803	0,404	0,399	1
Lzts3	0,209386	-0,02101	0,132	0,16	1
Gm10561	0,209645	-0,00529	0,028	0,042	1
Pnpla7	0,209753	-0,01677	0,112	0,139	1
Rnf41	0,210394	-0,01619	0,248	0,289	1
Tln1	0,210436	0,073861	0,716	0,73	1
Synm	0,210742	0,092223	0,791	0,8	1
Sox12	0,210901	0,003177	0,158	0,194	1
Cnot6l	0,211052	0,025328	0,361	0,43	1
Ufd1	0,211554	-0,01524	0,552	0,624	1
Tnfaip8	0,211683	0,020764	0,031	0,019	1
Glyr1	0,211841	0,088289	0,558	0,582	1
Mbd6	0,211902	0,064873	0,323	0,304	1
Mast4	0,211975	0,046603	0,991	0,996	1
Lcor	0,211995	-0,00545	0,228	0,268	1
Rps11	0,212221	0,003544	0,993	0,989	1
Efcab7	0,212608	-0,01134	0,031	0,046	1
Hhip	0,212705	0,016457	0,545	0,622	1
Epha5	0,212844	-0,00429	0,031	0,046	1
Gm26735	0,21304	0,018442	0,02	0,011	1
Fnbp1l	0,21304	0,022001	0,02	0,011	1
Rap1gds1	0,213094	0,044814	0,822	0,806	1
Dnajc25	0,213123	-0,01452	0,105	0,131	1
Dnajc1	0,213221	-0,00131	0,378	0,432	1
Phlpp2	0,213317	0,035525	0,125	0,101	1
Zfp960	0,213343	-0,00862	0,013	0,023	1
Zfp512b	0,21349	-0,01537	0,117	0,146	1
A430106G	0,21366	0,064068	0,128	0,105	1
Sf3a1	0,213838	-0,01769	0,316	0,365	1
Hectd1	0,213886	0,084058	0,776	0,81	1
Rtf1	0,213922	0,074636	0,705	0,715	1
Ccdc122	0,21395	0,016658	0,02	0,011	1
Aurka	0,21395	0,018442	0,02	0,011	1
Fbxl21	0,214989	0,023973	0,031	0,019	1
Gsto1	0,215077	-0,0216	0,149	0,177	1
Cds1	0,215128	0,001682	0,15	0,186	1
Asb7	0,21542	0,002673	0,222	0,264	1
Slc16a3	0,215659	-0,01606	0,035	0,051	1
Adgra2	0,215778	0,01487	0,02	0,011	1
Syk	0,215784	0,016356	0,02	0,011	1
Nr1h2	0,216079	-0,00101	0,534	0,62	1
Slc25a51	0,216234	0,079074	0,622	0,637	1
Wtip	0,216261	-0,00026	0,029	0,044	1
Gm16958	0,216288	-0,02244	0,051	0,07	1
Nsg1	0,216327	-0,0185	0,037	0,053	1
Smg6	0,216383	0,049757	0,336	0,409	1

Etohd2	0,216479	-0,0185	0,04	0,057	1
Gm17354	0,216579	-0,01419	0,075	0,097	1
Npm1	0,216589	-0,00923	0,932	0,935	1
Mybl2	0,216695	0,01487	0,02	0,011	1
Ccdc63	0,216695	0,011286	0,02	0,011	1
Zfp592	0,216724	-0,0218	0,178	0,209	1
Hey2	0,216862	0,028435	0,026	0,015	1
Fam76a	0,217134	-0,00572	0,673	0,738	1
Megf9	0,217457	-0,0048	0,481	0,563	1
Gm26901	0,217509	-0,01254	0,035	0,051	1
Rttn	0,21791	0,043393	0,101	0,08	1
Pclaf	0,217991	0,019609	0,026	0,015	1
Cdc14a	0,217997	0,007888	0,139	0,171	1
Dgkd	0,218112	0,068706	0,35	0,327	1
Gldn	0,218475	0,021153	0,011	0,004	1
Supt20	0,218653	0,019991	0,417	0,494	1
Lifr	0,218789	0,024269	0,466	0,559	1
Astn1	0,218918	0,007119	0,02	0,011	1
Arhgap32	0,218926	0,093916	0,367	0,365	1
Junos	0,218998	0,013972	0,011	0,004	1
Cdkn2aip	0,219116	0,019005	0,22	0,264	1
Nrep	0,219133	0,015757	0,026	0,015	1
BC034090	0,219464	0,068389	0,2	0,177	1
Tmem138	0,219488	-0,01635	0,114	0,141	1
Gsap	0,219521	0,015772	0,011	0,004	1
Tnfsf11	0,219521	0,015772	0,011	0,004	1
Zc3hav1l	0,219521	0,013972	0,011	0,004	1
Npff	0,219521	0,021153	0,011	0,004	1
Pid1	0,219521	0,017569	0,011	0,004	1
Terf2	0,219557	-0,00107	0,231	0,274	1
Lclat1	0,219563	-0,02009	0,068	0,089	1
Zfp955a	0,219626	-0,01006	0,037	0,053	1
Gnaz	0,219823	-0,015	0,04	0,057	1
D6Wsu163	0,219826	-0,02423	0,114	0,139	1
Thoc1	0,219829	-0,00357	0,207	0,247	1
Pcdhgb5	0,22002	-0,01103	0,037	0,053	1
Tmem59l	0,220042	-0,01679	0,204	0,236	1
Spp2	0,220045	0,010362	0,011	0,004	1
Birc5	0,220045	0,010362	0,011	0,004	1
Arhgap26	0,220045	0,010362	0,011	0,004	1
Smc1b	0,220045	0,017569	0,011	0,004	1
Plekha7	0,220045	0,012168	0,011	0,004	1
Tram1	0,220248	-0,00688	0,367	0,426	1
Setdb2	0,220504	-0,01822	0,081	0,103	1
Plxnd1	0,220569	0,008552	0,011	0,004	1
Cebpa	0,220569	0,008552	0,011	0,004	1
Slc10a1	0,220569	0,008552	0,011	0,004	1
Osmr	0,220569	0,008552	0,011	0,004	1
Gm28285	0,220569	0,008552	0,011	0,004	1
Rab8b	0,220657	-0,00644	0,257	0,302	1
Grid1	0,220835	0,01547	0,011	0,004	1
Ranbp10	0,221049	-0,00687	0,202	0,238	1

Zfp354b	0,22106	-0,01079	0,035	0,051	1
Olfr742	0,221094	0,006738	0,011	0,004	1
Gm9837	0,221094	0,006738	0,011	0,004	1
Erbb2	0,221094	0,006738	0,011	0,004	1
Msx2	0,221094	0,006738	0,011	0,004	1
Cnih2	0,221094	0,006738	0,011	0,004	1
Xylt2	0,221148	-0,01719	0,086	0,11	1
BC005561	0,22124	0,005078	0,538	0,62	1
Arl4a	0,221439	-0,00605	0,167	0,2	1
Gm32296	0,221817	-0,01273	0,015	0,025	1
2810474O	0,22199	0,008425	0,321	0,38	1
Naa30	0,222309	0,065843	0,314	0,291	1
Ctnna2	0,222393	-0,05075	0,428	0,475	1
Fam46c	0,222417	-0,0019	0,011	0,004	1
Dnajc13	0,22254	-0,00936	0,323	0,376	1
Gatad2a	0,222576	0,003273	0,42	0,489	1
Kars	0,222604	-0,0027	0,356	0,414	1
Ccdc84	0,222671	-0,02743	0,147	0,175	1
Fam83f	0,222681	0,000456	0,011	0,004	1
Mis18a	0,223009	-0,00806	0,035	0,051	1
Gm21994	0,223195	-0,00917	0,006	0,013	1
Gm26846	0,223195	-0,00917	0,006	0,013	1
Ajap1	0,223233	0,028248	0,051	0,036	1
Ripk2	0,223516	-0,01349	0,044	0,061	1
Pld1	0,223638	-0,00747	0,325	0,371	1
Gm20404	0,223641	-0,015	0,046	0,063	1
Fam83a	0,223698	-0,0115	0,006	0,013	1
Psmb10	0,223718	0,008964	0,138	0,169	1
Serpinh1	0,223774	0,022637	0,051	0,036	1
Smchd1	0,22385	0,130712	0,495	0,504	1
Rims1	0,223945	-0,00709	0,006	0,013	1
Gm26801	0,223945	-0,00709	0,006	0,013	1
Nebl	0,223945	-0,00709	0,006	0,013	1
Gm14091	0,223945	-0,00709	0,006	0,013	1
Cfap74	0,223945	-0,00709	0,006	0,013	1
Gm14508	0,223945	-0,00709	0,006	0,013	1
Smyd1	0,223945	-0,00709	0,006	0,013	1
Olfr552	0,223945	-0,00709	0,006	0,013	1
Esco2	0,223945	-0,00709	0,006	0,013	1
Slc28a3	0,223945	-0,00709	0,006	0,013	1
2810032G	0,223945	-0,00709	0,006	0,013	1
AI463170	0,223945	-0,00709	0,006	0,013	1
Gm5093	0,223945	-0,00709	0,006	0,013	1
Nme5	0,223945	-0,00709	0,006	0,013	1
Ccdc74a	0,223949	-0,00942	0,006	0,013	1
Esrra	0,224165	-0,0093	0,106	0,133	1
Spata2l	0,224442	0,024356	0,051	0,036	1
Lpp	0,224451	-0,0076	0,006	0,013	1
Diaph3	0,224509	-0,00502	0,015	0,025	1
Micu1	0,224668	-0,02946	0,631	0,684	1
4930515G	0,224701	-0,00735	0,006	0,013	1
0610005C	0,224701	-0,00735	0,006	0,013	1

Pex11g	0,224902	-0,01292	0,057	0,076	1
Fxyd6	0,224953	0,015757	0,006	0,013	1
Gm13375	0,225155	-0,01727	0,061	0,08	1
Ube2l3	0,225237	-0,02725	0,936	0,949	1
Unc13d	0,225239	0,026842	0,039	0,025	1
Ap3d1	0,225255	0,092085	0,428	0,422	1
Gm43518	0,225455	-0,00163	0,006	0,013	1
Arhgef4	0,225455	-0,00527	0,006	0,013	1
Itga10	0,225455	-0,00527	0,006	0,013	1
Krtcap3	0,225455	-0,00527	0,006	0,013	1
Gm17092	0,225455	-0,00527	0,006	0,013	1
Gm15637	0,225455	-0,00527	0,006	0,013	1
Gm48740	0,225455	-0,00527	0,006	0,013	1
Nprl3	0,22546	-0,02796	0,103	0,127	1
Timm23	0,225764	-0,00904	0,039	0,055	1
Tspan17	0,226197	-0,01532	0,49	0,551	1
Enox1	0,226249	0,000938	0,358	0,42	1
Adal	0,226353	-0,00697	0,11	0,137	1
Tns2	0,226518	0,029498	0,134	0,11	1
Foxo3	0,226552	0,003942	0,246	0,291	1
5330413P1	0,226553	-0,00682	0,015	0,025	1
Fam135b	0,226553	-0,00682	0,015	0,025	1
Fam234b	0,226606	0,08277	0,448	0,439	1
Pcsk6	0,226881	0,081682	0,596	0,608	1
Gm49289	0,226972	-0,00345	0,006	0,013	1
Arhgap21	0,227399	0,091415	0,567	0,574	1
Rufy1	0,22741	-0,00399	0,508	0,582	1
Tbc1d22b	0,227451	-0,01193	0,25	0,287	1
Ssbp3	0,227578	0,10077	0,367	0,363	1
P4htm	0,227961	0,067881	0,374	0,359	1
Htatip2	0,227969	0,032591	0,061	0,044	1
Mgat1	0,228039	-0,01817	0,253	0,293	1
Epn1	0,228088	-0,01466	0,591	0,665	1
Ppm1f	0,22817	-0,01287	0,206	0,243	1
Exosc2	0,228609	-0,02478	0,198	0,232	1
4831440E1	0,22861	-0,00502	0,015	0,025	1
Rsbn1	0,228728	0,018266	0,387	0,456	1
B3galt4	0,228786	-0,01407	0,068	0,089	1
Vrk2	0,229039	-0,01533	0,059	0,078	1
Rnpepl1	0,229268	0,071681	0,633	0,662	1
Zfp839	0,229491	-0,0143	0,086	0,11	1
Trmt61a	0,229673	-0,01287	0,088	0,112	1
Hyal2	0,229742	-0,00911	0,092	0,116	1
Esco1	0,229874	-0,01524	0,25	0,289	1
2700033N1	0,23001	-0,01465	0,05	0,068	1
Nrcam	0,230076	0,042404	0,046	0,032	1
Commd5	0,230106	-0,01151	0,048	0,065	1
Armc7	0,23028	-0,01556	0,057	0,076	1
Gm46404	0,230388	-0,00754	0,04	0,057	1
Mrnip	0,230695	-0,01143	0,053	0,072	1
Ttc7	0,230915	0,048146	0,123	0,101	1
Ank	0,230941	-0,04081	0,971	0,932	1

Aldh3b1	0,230949	-0,00496	0,301	0,348	1
Ccdc6	0,23107	0,046459	0,218	0,192	1
Gm48855	0,231231	-0,01096	0,057	0,076	1
Tgfb3	0,231379	-0,01316	0,134	0,162	1
Champ1	0,231737	-0,01871	0,158	0,188	1
Slc36a1	0,231804	-0,00972	0,051	0,07	1
Leng8	0,231949	0,001194	0,598	0,694	1
Tmem241	0,231965	0,003656	0,037	0,053	1
Trp53inp1	0,232118	0,103535	0,218	0,196	1
Rfc5	0,23229	-0,01384	0,061	0,08	1
Pak4	0,232363	-0,02139	0,112	0,137	1
Erap1	0,232767	-0,01338	0,057	0,076	1
Casp12	0,232936	0,012465	0,007	0,002	1
Smarca2	0,233076	-0,03148	0,593	0,643	1
Smarcd2	0,233172	-0,02588	0,16	0,188	1
Pabpc4	0,233225	-0,01442	0,145	0,175	1
Xpo4	0,23324	-0,00661	0,266	0,31	1
Tcaf2	0,233281	0,008842	0,007	0,002	1
Ffar1	0,233281	0,008842	0,007	0,002	1
Wee1	0,233281	0,008842	0,007	0,002	1
Il17rd	0,233281	0,008842	0,007	0,002	1
Tert	0,233281	0,008842	0,007	0,002	1
Cndp1	0,233281	0,014271	0,007	0,002	1
Slc7a5	0,233281	0,012465	0,007	0,002	1
A230050P2	0,233281	0,010655	0,007	0,002	1
Icam1	0,233281	0,010655	0,007	0,002	1
Ctns	0,23344	0,002207	0,128	0,158	1
Rif1	0,23357	0,090996	0,677	0,694	1
Srrm4	0,233626	0,016075	0,007	0,002	1
3632451O0	0,233626	0,008842	0,007	0,002	1
Gm26511	0,233626	0,008842	0,007	0,002	1
Gm26698	0,233626	0,008842	0,007	0,002	1
Dtl	0,233626	0,007025	0,007	0,002	1
Nexn	0,233626	0,007025	0,007	0,002	1
Raver2	0,233626	0,007025	0,007	0,002	1
Gm17300	0,233626	0,007025	0,007	0,002	1
Rsg1	0,233626	0,007025	0,007	0,002	1
Olfr635	0,233626	0,007025	0,007	0,002	1
Parva	0,233626	0,007025	0,007	0,002	1
Slc6a15	0,233626	0,007025	0,007	0,002	1
Gm34934	0,233626	0,007025	0,007	0,002	1
Ets1	0,233626	0,007025	0,007	0,002	1
Ticam2	0,233626	0,007025	0,007	0,002	1
Tesmin	0,233626	0,007025	0,007	0,002	1
Kif11	0,233626	0,007025	0,007	0,002	1
Zfp706	0,233671	-0,01445	0,994	0,994	1
Gm28551	0,233972	0,005205	0,007	0,002	1
Rbl1	0,233972	0,005205	0,007	0,002	1
A530013C2	0,233972	0,005205	0,007	0,002	1
Samd10	0,233972	0,005205	0,007	0,002	1
Nudt17	0,233972	0,005205	0,007	0,002	1
Gpx7	0,233972	0,005205	0,007	0,002	1

Uchl1os	0,233972	0,005205	0,007	0,002	1
Cldn15	0,233972	0,005205	0,007	0,002	1
Gm45437	0,233972	0,005205	0,007	0,002	1
1700120K(	0,233972	0,005205	0,007	0,002	1
5930422O:	0,233972	0,005205	0,007	0,002	1
A930006L(	0,233972	0,005205	0,007	0,002	1
Amigo3	0,233972	0,005205	0,007	0,002	1
Gm6566	0,233972	0,005205	0,007	0,002	1
Gm17058	0,233972	0,005205	0,007	0,002	1
Ciita	0,233972	0,005205	0,007	0,002	1
D430001F:	0,233972	0,005205	0,007	0,002	1
Kifc1	0,233972	0,005205	0,007	0,002	1
Atxn1	0,23402	0,158082	0,328	0,325	1
Rps18	0,234131	0,018681	0,971	0,973	1
Asap2	0,234257	-0,00901	0,389	0,447	1
Nme7	0,234347	-0,00948	0,05	0,068	1
Arntl2	0,234468	-0,00887	0,017	0,027	1
Fastkd3	0,234628	-0,01049	0,053	0,072	1
Map4k3	0,234681	-0,01652	0,171	0,203	1
Stx5a	0,234786	-0,00518	0,495	0,572	1
Zfp746	0,234848	0,006105	0,187	0,226	1
Paxx	0,234851	0,06183	0,602	0,608	1
Bicra	0,234997	-0,00648	0,163	0,196	1
Klhl12	0,235064	-0,00217	0,202	0,238	1
Cpeb1	0,235095	0,079076	0,615	0,616	1
AW549877	0,235116	0,067552	0,618	0,643	1
Casc1	0,235144	-0,00913	0,017	0,027	1
Acaa2	0,235432	-0,01072	0,051	0,07	1
Chd1	0,235699	0,008889	0,345	0,403	1
Vwa5a	0,235763	-0,01032	0,292	0,331	1
Cep170b	0,235842	0,051399	0,18	0,156	1
Ncapd2	0,236	-0,01113	0,064	0,084	1
Fbxl16	0,236181	0,012517	0,039	0,025	1
Akt2	0,23636	0,01335	0,336	0,397	1
Kcmf1	0,236554	0,003859	0,415	0,479	1
Rpl36a-ps1	0,236648	-0,00707	0,017	0,027	1
Apex2	0,236791	-0,01013	0,072	0,093	1
Prpf39	0,236952	-0,00896	0,547	0,635	1
Fbxo42	0,237114	-0,01592	0,112	0,137	1
Eif4ebp2	0,237224	0,007545	0,215	0,255	1
Maml1	0,237408	-0,01916	0,114	0,139	1
Plpp2	0,237518	-0,02609	0,631	0,675	1
Zfp598	0,237742	-0,0048	0,191	0,226	1
Ldhd	0,238009	-5,8E-05	0,108	0,135	1
Golph3	0,238036	0,005787	0,367	0,426	1
Ptpn4	0,238074	0,066901	0,231	0,209	1
Gbf1	0,23833	0,006204	0,343	0,403	1
Mapkbp1	0,238504	-0,01037	0,075	0,097	1
Bbs5	0,238758	-0,00653	0,061	0,08	1
A430105I1	0,238811	-0,00842	0,084	0,108	1
Fam168b	0,238859	0,071927	0,415	0,403	1
Hoxc6	0,23894	-0,01297	0,018	0,03	1

Zfp652	0,239155	0,01805	0,349	0,411	1
Rnf187	0,239233	-0,00974	0,897	0,892	1
Rtl6	0,239448	-0,01748	0,106	0,131	1
Dclre1c	0,24007	0,051477	0,095	0,076	1
Nudt4	0,240199	-0,05097	0,655	0,686	1
Gm45345	0,240223	0,035207	0,178	0,152	1
Acadsb	0,240243	-0,00275	0,369	0,424	1
Mcm9	0,24044	-0,00737	0,136	0,165	1
Prcc	0,240445	0,001814	0,189	0,226	1
Rps6ka2	0,240603	-0,005	0,163	0,196	1
Spindoc	0,240879	-0,00369	0,088	0,112	1
Tmem9b	0,240945	-0,00329	0,831	0,869	1
Gm26541	0,24106	0,016931	0,033	0,021	1
Zcchc8	0,241164	-0,00016	0,229	0,27	1
Zfp13	0,241198	-0,00679	0,051	0,07	1
Gm4707	0,241552	0,020615	0,061	0,044	1
Slc25a23	0,241659	0,002392	0,572	0,65	1
Mau2	0,241783	0,085464	0,617	0,658	1
Mindy4	0,241847	-0,00531	0,051	0,07	1
Eef2k	0,241918	0,047819	0,116	0,095	1
Ahdc1	0,241987	-0,0121	0,139	0,169	1
Etv5	0,24211	-0,00434	0,046	0,063	1
Trmt12	0,242129	0,00094	0,066	0,086	1
Slc38a3	0,242324	0,052313	0,103	0,082	1
Mfsd7a	0,242483	0,02294	0,053	0,038	1
Klhl29	0,24257	0,015162	0,033	0,021	1
Ap4m1	0,242623	0,01505	0,174	0,211	1
Psme3	0,242674	-0,00705	0,231	0,27	1
Tufm	0,242789	-0,00994	0,27	0,312	1
Zfp628	0,242903	0,020316	0,061	0,044	1
Sgcb	0,243042	0,000988	0,626	0,707	1
Med25	0,243052	-0,00926	0,36	0,411	1
Zdhhc13	0,243069	-0,00438	0,083	0,105	1
Kdm3a	0,243393	0,108649	0,358	0,357	1
Smarcal1	0,243881	0,060772	0,2	0,175	1
Heatr3	0,244233	0,017229	0,49	0,568	1
Mrgbp	0,244283	0,003746	0,196	0,234	1
AI197445	0,244349	-0,01955	0,02	0,032	1
Nedd1	0,244418	-0,0056	0,141	0,171	1
Pttg1	0,24458	-0,0112	0,297	0,338	1
Usp10	0,244686	0,00991	0,257	0,306	1
Card10	0,244982	0,04406	0,081	0,063	1
Gm9493	0,245158	-0,00733	0,018	0,03	1
Cs	0,245367	-0,00038	0,539	0,622	1
Rpl23	0,245524	-0,01119	0,998	0,998	1
Wdr44	0,246145	0,005066	0,2	0,238	1
A630089N(	0,246157	0,001339	0,018	0,03	1
Mbtd1	0,246377	0,008842	0,358	0,418	1
Epha10	0,246408	-0,01346	0,02	0,032	1
Dstn	0,246693	0,054427	1	0,987	1
4930455G(	0,246845	0,029044	0,04	0,027	1
Whamm	0,24702	0,046321	0,167	0,143	1

Lncpint	0,247063	0,052499	0,24	0,215	1
Foxk1	0,24745	-0,00477	0,185	0,219	1
Dagla	0,247898	0,018748	0,017	0,008	1
Hdac6	0,24798	0,046203	0,127	0,105	1
Zfp287	0,248088	-0,01509	0,196	0,23	1
Kdelr2	0,248107	-0,00653	0,402	0,468	1
Csnk1a1	0,248245	0,05339	0,987	0,998	1
Vangl2	0,248284	0,010624	0,167	0,2	1
Dgat1	0,24829	-0,00099	0,189	0,226	1
Dact1	0,248749	0,017532	0,028	0,017	1
Arpc2	0,248959	0,061854	0,837	0,859	1
Xab2	0,249002	0,018354	0,394	0,464	1
Hnrnpll	0,249017	0,004272	0,325	0,376	1
Tax1bp3	0,249082	0,000332	0,178	0,211	1
Cpt1c	0,249093	0,007313	0,237	0,276	1
Polr1c	0,249232	-0,00992	0,228	0,266	1
H2-Q10	0,249249	0,022001	0,017	0,008	1
Ythdf2	0,249306	-0,01692	0,378	0,428	1
Wars	0,249384	-0,00418	0,297	0,346	1
Hsd3b7	0,249697	0,018748	0,017	0,008	1
Polg2	0,249757	0,039012	0,139	0,116	1
Crybb1	0,249918	-0,00938	0,02	0,032	1
Efcab12	0,250109	0,015757	0,028	0,017	1
Ttc39b	0,250109	0,019303	0,028	0,017	1
Abi1	0,250502	0,110204	0,417	0,426	1
Gm12981	0,250599	0,011579	0,017	0,008	1
1700040D	0,250892	-0,0115	0,007	0,015	1
S100a1	0,25095	0,037971	0,963	0,916	1
G0s2	0,251109	0,032748	0,112	0,137	1
Tecpr1	0,251188	-0,00436	0,358	0,422	1
Smc5	0,251466	0,106758	0,387	0,39	1
Gtf2ird1	0,251596	-0,00926	0,116	0,141	1
Fam35a	0,251597	-0,02159	0,09	0,112	1
Plekhm2	0,252132	-0,00643	0,283	0,323	1
Gm3550	0,252343	-0,00758	0,02	0,032	1
G730003C	0,25241	0,007975	0,017	0,008	1
Orai2	0,25241	0,007975	0,017	0,008	1
Tsc2	0,252517	0,074273	0,378	0,369	1
Tm9sf1	0,252686	0,002504	0,081	0,103	1
Tmem182	0,252701	-0,00657	0,002	0,006	1
Cacna1e	0,252701	-0,00657	0,002	0,006	1
Smim10l2a	0,252701	-0,00657	0,002	0,006	1
Cth	0,252701	-0,00657	0,002	0,006	1
Tspan33	0,252701	-0,00657	0,002	0,006	1
1700040L(	0,252701	-0,00657	0,002	0,006	1
Gm44662	0,252701	-0,00657	0,002	0,006	1
Gm19325	0,252701	-0,00657	0,002	0,006	1
Ppm1e	0,252701	-0,00657	0,002	0,006	1
Pdzd2	0,252701	-0,00657	0,002	0,006	1
Dbx2	0,252701	-0,00657	0,002	0,006	1
P3h2	0,252701	-0,00657	0,002	0,006	1
C130060C(	0,252701	-0,00657	0,002	0,006	1

Kcnc3	0,252701	-0,00866	0,002	0,006	1
Ifitm3	0,252701	-0,00866	0,002	0,006	1
Ptp4a3	0,252701	-0,00866	0,002	0,006	1
Hoxb3	0,252701	-0,01075	0,002	0,006	1
Isg20	0,252701	-0,01698	0,002	0,006	1
Phkg1	0,252824	0,00326	0,048	0,065	1
Agbl2	0,252861	-0,00735	0,007	0,015	1
Mms22l	0,252861	-0,00735	0,007	0,015	1
Mansc1	0,252861	-0,00735	0,007	0,015	1
Pdf	0,252861	-0,00735	0,007	0,015	1
Drc3	0,252861	-0,00735	0,007	0,015	1
Adgrb1	0,25305	0,054278	0,231	0,207	1
Pten	0,25307	0,058507	0,64	0,646	1
9930104L(	0,253087	-0,00696	0,123	0,15	1
Tmem185b	0,253097	0,005252	0,106	0,133	1
Ndnf	0,253101	0,030573	0,055	0,04	1
Speg	0,253109	-0,00448	0,002	0,006	1
Opn3	0,253109	-0,00448	0,002	0,006	1
Armc3	0,253109	-0,00448	0,002	0,006	1
Ntng2	0,253109	-0,00448	0,002	0,006	1
Olfr1310	0,253109	-0,00448	0,002	0,006	1
Gm14435	0,253109	-0,00448	0,002	0,006	1
Mageb16	0,253109	-0,00448	0,002	0,006	1
Bex4	0,253109	-0,00448	0,002	0,006	1
Actrt3	0,253109	-0,00448	0,002	0,006	1
Sis	0,253109	-0,00448	0,002	0,006	1
Zkscan16	0,253109	-0,00448	0,002	0,006	1
Gm13212	0,253109	-0,00448	0,002	0,006	1
Pik3cd	0,253109	-0,00448	0,002	0,006	1
Chd5	0,253109	-0,00448	0,002	0,006	1
Kctd8	0,253109	-0,00448	0,002	0,006	1
Gm35911	0,253109	-0,00448	0,002	0,006	1
Gm42918	0,253109	-0,00448	0,002	0,006	1
Dnah10	0,253109	-0,00448	0,002	0,006	1
Fbxo24	0,253109	-0,00448	0,002	0,006	1
Cpa5	0,253109	-0,00448	0,002	0,006	1
1700026J1	0,253109	-0,00448	0,002	0,006	1
Map4k1	0,253109	-0,00448	0,002	0,006	1
Zfp977	0,253109	-0,00448	0,002	0,006	1
Rsf1os1	0,253109	-0,00448	0,002	0,006	1
Rsf1os2	0,253109	-0,00448	0,002	0,006	1
Chrdl2	0,253109	-0,00448	0,002	0,006	1
Gm20597	0,253109	-0,00448	0,002	0,006	1
Mypn	0,253109	-0,00448	0,002	0,006	1
Reep6	0,253109	-0,00448	0,002	0,006	1
Syt1	0,253109	-0,00448	0,002	0,006	1
C130073E2	0,253109	-0,00448	0,002	0,006	1
Ptger1	0,253109	-0,00448	0,002	0,006	1
Gm10248	0,253109	-0,00448	0,002	0,006	1
Gm48940	0,253109	-0,00448	0,002	0,006	1
Rnf17	0,253109	-0,00448	0,002	0,006	1
Npm2	0,253109	-0,00448	0,002	0,006	1

Gm38431	0,253109	-0,00448	0,002	0,006	1
Ttc36	0,253109	-0,00448	0,002	0,006	1
Gm47409	0,253109	-0,00448	0,002	0,006	1
Gm47112	0,253109	-0,00448	0,002	0,006	1
Pdlim4	0,253109	-0,00448	0,002	0,006	1
1700001P(	0,253109	-0,00448	0,002	0,006	1
Dhx58	0,253109	-0,00448	0,002	0,006	1
Zfp750	0,253109	-0,00448	0,002	0,006	1
Gm48116	0,253109	-0,00448	0,002	0,006	1
Acot12	0,253109	-0,00448	0,002	0,006	1
2410018L1	0,253109	-0,00448	0,002	0,006	1
Six4	0,253109	-0,00448	0,002	0,006	1
Gm15943	0,253109	-0,00448	0,002	0,006	1
Pvalb	0,253109	-0,00448	0,002	0,006	1
Ppp1r1a	0,253109	-0,00448	0,002	0,006	1
Aifm3	0,253109	-0,00448	0,002	0,006	1
Zdhhc19	0,253109	-0,00448	0,002	0,006	1
AC110166.	0,253109	-0,00448	0,002	0,006	1
Mrap	0,253109	-0,00448	0,002	0,006	1
Zfp947	0,253109	-0,00448	0,002	0,006	1
Def6	0,253109	-0,00448	0,002	0,006	1
Slc3a1	0,253109	-0,00448	0,002	0,006	1
Kif20a	0,253109	-0,00448	0,002	0,006	1
Pcdha5	0,253109	-0,00448	0,002	0,006	1
Ms4a4c	0,253109	-0,00448	0,002	0,006	1
Plce1	0,253109	-0,00448	0,002	0,006	1
Trafd1	0,253302	-0,00791	0,2	0,236	1
Dnaaf2	0,253355	-0,01142	0,022	0,034	1
Gm21887	0,253581	0,026711	0,079	0,061	1
Cdk5r2	0,253608	-0,0076	0,007	0,015	1
Gm5577	0,253608	-0,0076	0,007	0,015	1
Malt1	0,253887	0,019849	0,145	0,177	1
Mzb1	0,253929	-0,00474	0,002	0,006	1
Hoxb3os	0,253929	-0,00527	0,002	0,006	1
Tnfaip1	0,254046	-0,0094	0,191	0,224	1
Lman2	0,254177	0,08581	0,55	0,582	1
Dlgap1	0,25434	0,006453	0,002	0,006	1
Gm7697	0,25434	0,001003	0,002	0,006	1
Gm37850	0,25434	-0,00082	0,002	0,006	1
Gm15537	0,25434	-0,00082	0,002	0,006	1
0610039K1	0,25434	-0,00265	0,002	0,006	1
Flicr	0,25434	-0,00265	0,002	0,006	1
Cd52	0,25434	-0,00265	0,002	0,006	1
Pon3	0,25434	-0,00265	0,002	0,006	1
Fcgrt	0,25434	-0,00265	0,002	0,006	1
Hectd2	0,25434	-0,00265	0,002	0,006	1
Ppfia4	0,25434	-0,00291	0,002	0,006	1
Zfpm1	0,254582	0,087789	0,446	0,456	1
Rpusd2	0,2546	-0,00371	0,007	0,015	1
Morn5	0,2546	-0,00553	0,007	0,015	1
Zscan25	0,2546	-0,00553	0,007	0,015	1
Tshb	0,254814	0,014572	0,022	0,013	1

2010109A1	0,254814	0,014572	0,022	0,013	1
Serhl	0,254814	0,016356	0,022	0,013	1
3010003L2	0,254947	-0,00656	0,022	0,034	1
Rbpjl	0,255002	0,037352	0,114	0,093	1
Pcx	0,255141	0,073488	0,439	0,432	1
Pou2f1	0,255202	0,095569	0,347	0,34	1
Alms1	0,255225	0,048643	0,073	0,057	1
Spata5	0,25543	-0,01605	0,097	0,12	1
Cdkn1b	0,255514	0,069391	0,688	0,686	1
Adgre5	0,255599	-0,00397	0,007	0,015	1
Mfsd10	0,255606	-0,01031	0,132	0,16	1
Ptcra	0,25562	0,008627	0,017	0,008	1
Cpsf7	0,255733	0,019335	0,396	0,464	1
Etl4	0,255983	0,012785	0,022	0,013	1
Tgfb1	0,255983	0,018137	0,022	0,013	1
Osbpl2	0,25623	0,000883	0,314	0,363	1
Rps27rt	0,256325	-0,0129	0,237	0,272	1
Eaf2	0,256539	-0,01061	0,022	0,034	1
Hbegf	0,256661	0,0494	0,095	0,076	1
Plekhf2	0,256816	0,036377	0,064	0,049	1
Gys1	0,257098	-0,00397	0,007	0,015	1
Tmem65	0,257381	0,007404	0,475	0,549	1
Rsad2	0,257956	0,029222	0,022	0,013	1
Grik5	0,25817	-0,02827	0,273	0,31	1
Gm10762	0,258413	-0,00783	0,022	0,034	1
Gm16702	0,259059	-0,00809	0,022	0,034	1
Dmrtc1a	0,25925	-0,01822	0,028	0,04	1
BC025920	0,259335	0,020615	0,055	0,04	1
Sat1	0,259406	0,134247	0,644	0,643	1
Snrpf	0,259564	-0,00858	0,75	0,8	1
Zfp3	0,259675	-0,01061	0,024	0,036	1
Tmx3	0,259835	-0,00775	0,624	0,686	1
Oxsr1	0,259877	0,081209	0,229	0,209	1
Mpp7	0,260035	-0,01086	0,022	0,034	1
Ppm1a	0,260192	-0,00712	0,754	0,821	1
Bivm	0,260413	-0,01553	0,086	0,108	1
Cmtm6	0,2607	0,008401	0,251	0,295	1
Carm1	0,26077	-0,0094	0,198	0,232	1
Mocs3	0,261072	-0,01998	0,123	0,148	1
Csrp2	0,261156	0,022334	0,055	0,04	1
Dph2	0,261357	-0,01242	0,09	0,112	1
Cenpc1	0,261431	0,016728	0,275	0,325	1
Puf60	0,26147	0,052749	0,895	0,867	1
B4galt3	0,261538	0,077486	0,301	0,285	1
Ctdspl2	0,261572	0,007225	0,297	0,348	1
Sobp	0,261661	0,016426	0,127	0,103	1
Nhlrc4	0,261678	-0,003	0,024	0,036	1
Gba2	0,261852	-0,01756	0,075	0,095	1
Osbpl5	0,262091	-0,01863	0,125	0,15	1
Hmbox1	0,262158	0,006024	0,394	0,449	1
Asphd1	0,26217	0,000847	0,077	0,099	1
Arpp19	0,26223	-0,00886	0,945	0,935	1

Ralb	0,262349	-0,00899	0,183	0,215	1
Iqsec2	0,262587	-0,00478	0,022	0,034	1
Mapk3	0,26267	-0,01875	0,983	0,975	1
Atp9a	0,262718	-0,03614	0,901	0,901	1
Polr3g	0,262964	-0,00558	0,099	0,122	1
Ubtd2	0,262985	-0,01374	0,077	0,097	1
Pyroxd1	0,263148	-0,02827	0,207	0,236	1
Gm43598	0,263253	0,026842	0,035	0,023	1
Mylip	0,263571	-0,0143	0,084	0,105	1
Snrpb2	0,263593	0,025663	0,431	0,517	1
Cenpw	0,26361	-0,01239	0,026	0,038	1
Ier5l	0,263861	0,064428	0,084	0,068	1
Foxo1	0,264033	0,072891	0,369	0,354	1
Parg	0,264103	0,021092	0,305	0,352	1
Slc5a5	0,264225	-0,01287	0,026	0,038	1
Traf3	0,264409	-0,0169	0,075	0,095	1
Plekho2	0,264705	0,06223	0,187	0,167	1
Ube2z	0,264789	0,007564	0,297	0,344	1
Mex3b	0,264928	0,103712	0,206	0,19	1
Wdr45b	0,265253	0,019267	0,55	0,633	1
Col16a1	0,265314	0,064141	0,283	0,257	1
Gtpbp3	0,265457	-0,00988	0,134	0,16	1
Knstrn	0,265722	-0,01037	0,026	0,038	1
Fndc3b	0,265979	0,030833	0,141	0,118	1
Fcho1	0,266304	0,015438	0,055	0,04	1
Prune2	0,266368	0,09681	0,288	0,272	1
Mafg	0,266623	0,095923	0,572	0,599	1
Tgfb2	0,266769	0,044425	0,05	0,036	1
Ncoa2	0,267197	0,081538	0,55	0,563	1
Rps4x	0,267543	0,002631	0,976	0,989	1
Srsf11	0,268468	0,042843	0,886	0,888	1
Armcx1	0,268814	-0,02098	0,299	0,333	1
Gtpbp4	0,268938	0,038103	0,448	0,523	1
Wdr7	0,268983	-0,00871	0,262	0,302	1
Ttc27	0,269236	-0,01197	0,09	0,112	1
Pea15a	0,269563	0,001762	0,983	0,981	1
Miga1	0,269568	0,035855	0,358	0,432	1
Abhd13	0,26969	-0,01526	0,13	0,156	1
Setd1b	0,269869	0,100995	0,389	0,388	1
Acads	0,269925	0,032272	0,156	0,133	1
Pds5a	0,270016	0,093625	0,47	0,477	1
Abcd1	0,270048	0,050397	0,196	0,175	1
Msto1	0,270451	-0,00421	0,116	0,141	1
Tspan2	0,27052	-0,01673	0,989	0,992	1
Spats1	0,270607	-0,00656	0,026	0,038	1
Uggt2	0,270706	0,027061	0,117	0,146	1
S100a13	0,270831	0,062977	0,947	0,922	1
Sfxn2	0,27095	0,046017	0,158	0,135	1
Mad2l1bp	0,271098	-0,00748	0,083	0,103	1
Ttc8	0,271154	-0,01491	0,185	0,215	1
Mmab	0,271341	-0,00907	0,103	0,127	1
Gusb	0,271737	-0,01064	0,257	0,295	1

Tshz1	0,272048	0,076899	0,534	0,536	1
Pip4k2a	0,272059	0,060768	0,985	0,994	1
Kdm1b	0,272095	0,032075	0,136	0,114	1
Nme3	0,272188	-0,01498	0,072	0,091	1
Rce1	0,272431	-0,00643	0,268	0,308	1
Tnpo3	0,272936	0,091844	0,481	0,494	1
Slc39a1	0,27312	0,046085	0,578	0,578	1
Rasa1	0,273222	-0,0045	0,352	0,399	1
Shbg	0,273235	-0,01199	0,009	0,017	1
Amot	0,273711	-0,00968	0,009	0,017	1
Gm26799	0,273711	-0,00968	0,009	0,017	1
Itga2b	0,273711	-0,00968	0,009	0,017	1
Zscan2	0,273944	-0,01335	0,033	0,046	1
Kdm4c	0,274051	-0,00482	0,202	0,234	1
4933427D1	0,274071	-0,00013	0,112	0,137	1
Inafm1	0,274117	0,039533	0,11	0,091	1
Dusp18	0,274138	0,032062	0,059	0,044	1
Ints13	0,274342	0,006107	0,259	0,304	1
Odc1	0,274491	0,035709	0,283	0,259	1
Sugt1	0,27477	-0,02896	0,651	0,703	1
1700034P1	0,274913	-0,0076	0,009	0,017	1
Ankrd23	0,274913	-0,0076	0,009	0,017	1
Catspere2	0,274913	-0,0076	0,009	0,017	1
Gm14426	0,274913	-0,0076	0,009	0,017	1
Gm21269	0,274913	-0,0076	0,009	0,017	1
Gm6712	0,274913	-0,0076	0,009	0,017	1
Tubgcp2	0,274978	0,005964	0,189	0,222	1
Ovgp1	0,275067	-0,00908	0,029	0,042	1
Snrpc	0,275246	-0,01082	0,606	0,679	1
Fbxo45	0,27529	0,012829	0,292	0,34	1
Tenm3	0,275445	0,027006	0,042	0,03	1
Kcnma1	0,275453	0,058165	0,206	0,179	1
4930488L2	0,275856	-0,02006	0,053	0,07	1
AI464131	0,275921	-0,01383	0,037	0,051	1
Zfp963	0,276188	0,002302	0,026	0,038	1
4932435O1	0,276851	-0,00579	0,009	0,017	1
Mok	0,276851	-0,00579	0,009	0,017	1
Gm36899	0,276851	-0,00579	0,009	0,017	1
Add1	0,277026	0,008427	0,916	0,882	1
Cep76	0,277099	-0,01546	0,05	0,065	1
Racgap1	0,277134	0,002034	0,026	0,038	1
Gm2415	0,27719	-0,00723	0,088	0,11	1
Gadd45a	0,277395	-0,02799	0,325	0,363	1
Zbtb34	0,277433	0,026548	0,066	0,051	1
Cenpm	0,277583	-0,00605	0,009	0,017	1
2210008F0	0,277826	-0,00424	0,009	0,017	1
Zfp759	0,277912	-0,015	0,046	0,061	1
Pik3c2b	0,277961	0,00439	0,747	0,793	1
Zfp867	0,277975	-0,01158	0,035	0,049	1
Ydjc	0,278021	-0,01453	0,042	0,057	1
Zfp971	0,278113	-0,00806	0,035	0,049	1
Khsrp	0,278668	0,097016	0,45	0,464	1

Parp16	0,278798	-0,00216	0,009	0,017	1
Zfp937	0,279149	-0,01465	0,055	0,072	1
Pola2	0,279191	-0,02037	0,108	0,131	1
Sec22c	0,279247	-0,01635	0,117	0,141	1
Cdk16	0,279279	0,004981	0,512	0,586	1
Taf4b	0,279299	0,020458	0,029	0,019	1
Scaf11	0,279497	0,045437	0,895	0,89	1
Gtpbp1	0,279534	-0,00419	0,101	0,124	1
Eef1d	0,279706	0,042743	0,945	0,945	1
Traf5	0,279777	-0,00063	0,009	0,017	1
D730045B(	0,279777	-0,00243	0,009	0,017	1
Sfxn1	0,27982	0,018999	0,029	0,019	1
Tmem28	0,28039	-0,00078	0,105	0,129	1
Pkd1	0,28053	0,071658	0,33	0,319	1
Tbc1d9b	0,280547	0,0657	0,367	0,346	1
B130006D(	0,280738	-0,01183	0,035	0,049	1
Aldh6a1	0,280741	-0,01516	0,659	0,698	1
Gm45447	0,280753	-0,00036	0,009	0,017	1
Becn1	0,280814	-0,00221	0,385	0,443	1
Wdr73	0,281358	0,002529	0,105	0,129	1
Cenpe	0,281498	-0,00078	0,028	0,04	1
Ago2	0,28184	0,005897	0,248	0,289	1
Rps14	0,282074	0,049906	0,998	0,996	1
Man1b1	0,282204	-0,00466	0,354	0,403	1
Tspyl1	0,282314	0,002346	0,58	0,671	1
Asxl2	0,282397	0,06724	0,536	0,54	1
AC146911.	0,282963	-0,00654	0,051	0,068	1
Xcr1	0,283012	0,013685	0,029	0,019	1
Gm16152	0,283012	0,017231	0,029	0,019	1
Atcay	0,283457	-0,00485	0,07	0,089	1
Rpl36	0,283507	-0,00361	0,996	0,998	1
Prkcsh	0,283528	0,063996	0,683	0,698	1
Zadh2	0,283642	-0,00984	0,222	0,253	1
Trim44	0,283683	0,063707	0,633	0,639	1
Hsd3b2	0,283747	0,025099	0,037	0,025	1
Npr1	0,283942	0,001766	0,028	0,04	1
Htatsf1	0,284058	0,003834	0,45	0,523	1
Reps1	0,284234	-0,01769	0,264	0,3	1
Rnf216	0,284294	0,014053	0,453	0,515	1
Nmt2	0,284394	0,078738	0,272	0,253	1
Sulf1	0,284478	-0,00211	0	0,002	1
Dnah7a	0,284478	-0,00211	0	0,002	1
Casp8	0,284478	-0,00211	0	0,002	1
Ihh	0,284478	-0,00211	0	0,002	1
Sp110	0,284478	-0,00211	0	0,002	1
2810459M	0,284478	-0,00211	0	0,002	1
Inpp5d	0,284478	-0,00211	0	0,002	1
Tnfrsf11a	0,284478	-0,00211	0	0,002	1
Tmem37	0,284478	-0,00211	0	0,002	1
Celrr	0,284478	-0,00211	0	0,002	1
Map3k19	0,284478	-0,00211	0	0,002	1
Ctse	0,284478	-0,00211	0	0,002	1

Fam163a	0,284478	-0,00211	0	0,002	1
Rxrg	0,284478	-0,00211	0	0,002	1
F11r	0,284478	-0,00211	0	0,002	1
Gm38251	0,284478	-0,00211	0	0,002	1
Kcnh1	0,284478	-0,00211	0	0,002	1
Traf3ip3	0,284478	-0,00211	0	0,002	1
Sapcd2	0,284478	-0,00211	0	0,002	1
Ptges	0,284478	-0,00211	0	0,002	1
Ass1	0,284478	-0,00211	0	0,002	1
C130021I2	0,284478	-0,00211	0	0,002	1
Traf1	0,284478	-0,00211	0	0,002	1
Ptgs1	0,284478	-0,00211	0	0,002	1
Sp5	0,284478	-0,00211	0	0,002	1
Gm26558	0,284478	-0,00211	0	0,002	1
Ttn	0,284478	-0,00211	0	0,002	1
Calcrl	0,284478	-0,00211	0	0,002	1
Pamr1	0,284478	-0,00211	0	0,002	1
Rhov	0,284478	-0,00211	0	0,002	1
Dusp2	0,284478	-0,00211	0	0,002	1
Gm14005	0,284478	-0,00211	0	0,002	1
Pdyn	0,284478	-0,00211	0	0,002	1
Adam33	0,284478	-0,00211	0	0,002	1
Siglec1	0,284478	-0,00211	0	0,002	1
Syndig1	0,284478	-0,00211	0	0,002	1
Nrsn2	0,284478	-0,00211	0	0,002	1
Necab3	0,284478	-0,00211	0	0,002	1
2310005A(	0,284478	-0,00211	0	0,002	1
Mmp24	0,284478	-0,00211	0	0,002	1
Kcns1	0,284478	-0,00211	0	0,002	1
Cdh22	0,284478	-0,00211	0	0,002	1
Dok5	0,284478	-0,00211	0	0,002	1
Chrna4	0,284478	-0,00211	0	0,002	1
Gpr82	0,284478	-0,00211	0	0,002	1
Chst7	0,284478	-0,00211	0	0,002	1
Slc9a7	0,284478	-0,00211	0	0,002	1
Dcaf12l1	0,284478	-0,00211	0	0,002	1
Igsf1	0,284478	-0,00211	0	0,002	1
Gabre	0,284478	-0,00211	0	0,002	1
Bgn	0,284478	-0,00211	0	0,002	1
Itm2a	0,284478	-0,00211	0	0,002	1
Pou3f4	0,284478	-0,00211	0	0,002	1
Rps6ka6	0,284478	-0,00211	0	0,002	1
Tceal6	0,284478	-0,00211	0	0,002	1
Kctd12b	0,284478	-0,00211	0	0,002	1
Rnf138rt1	0,284478	-0,00211	0	0,002	1
Tlr7	0,284478	-0,00211	0	0,002	1
Arhgap6	0,284478	-0,00211	0	0,002	1
Gm37350	0,284478	-0,00211	0	0,002	1
Fabp4	0,284478	-0,00211	0	0,002	1
Mir124-2h	0,284478	-0,00211	0	0,002	1
A830092H	0,284478	-0,00211	0	0,002	1
P2ry1	0,284478	-0,00211	0	0,002	1

9330121J0	0,284478	-0,00211	0	0,002	1
Cd1d1	0,284478	-0,00211	0	0,002	1
Cd5l	0,284478	-0,00211	0	0,002	1
Iqgap3	0,284478	-0,00211	0	0,002	1
Sema4a	0,284478	-0,00211	0	0,002	1
Efna3	0,284478	-0,00211	0	0,002	1
Tnfaip8l2	0,284478	-0,00211	0	0,002	1
Hmgcs2	0,284478	-0,00211	0	0,002	1
Spag17	0,284478	-0,00211	0	0,002	1
Kcnc4	0,284478	-0,00211	0	0,002	1
5330417C2	0,284478	-0,00211	0	0,002	1
Plppr4	0,284478	-0,00211	0	0,002	1
Ndst3	0,284478	-0,00211	0	0,002	1
Bdh2	0,284478	-0,00211	0	0,002	1
Adgrl2	0,284478	-0,00211	0	0,002	1
Car8	0,284478	-0,00211	0	0,002	1
Gm11837	0,284478	-0,00211	0	0,002	1
Bach2os	0,284478	-0,00211	0	0,002	1
Pdzk1ip1	0,284478	-0,00211	0	0,002	1
Kif2c	0,284478	-0,00211	0	0,002	1
Ccdc30	0,284478	-0,00211	0	0,002	1
Dlgap3	0,284478	-0,00211	0	0,002	1
Fgr	0,284478	-0,00211	0	0,002	1
Cnr2	0,284478	-0,00211	0	0,002	1
Mfap2	0,284478	-0,00211	0	0,002	1
Gm13052	0,284478	-0,00211	0	0,002	1
Lrrc38	0,284478	-0,00211	0	0,002	1
Zfp981	0,284478	-0,00211	0	0,002	1
Disp3	0,284478	-0,00211	0	0,002	1
Ube4bos1	0,284478	-0,00211	0	0,002	1
Steap2	0,284478	-0,00211	0	0,002	1
Otof	0,284478	-0,00211	0	0,002	1
Nat8l	0,284478	-0,00211	0	0,002	1
9630001P1	0,284478	-0,00211	0	0,002	1
Ppargc1a	0,284478	-0,00211	0	0,002	1
Sod3	0,284478	-0,00211	0	0,002	1
8030423F2	0,284478	-0,00211	0	0,002	1
Antxr2	0,284478	-0,00211	0	0,002	1
Prkg2	0,284478	-0,00211	0	0,002	1
Galnt9	0,284478	-0,00211	0	0,002	1
Miat	0,284478	-0,00211	0	0,002	1
Sdsl	0,284478	-0,00211	0	0,002	1
Morn3	0,284478	-0,00211	0	0,002	1
5930412G1	0,284478	-0,00211	0	0,002	1
Vgf	0,284478	-0,00211	0	0,002	1
Flt1	0,284478	-0,00211	0	0,002	1
Medag	0,284478	-0,00211	0	0,002	1
Mdfic	0,284478	-0,00211	0	0,002	1
Hoxa10	0,284478	-0,00211	0	0,002	1
Gm16499	0,284478	-0,00211	0	0,002	1
Prr15	0,284478	-0,00211	0	0,002	1
Tnip3	0,284478	-0,00211	0	0,002	1

Lrrtm1	0,284478	-0,00211	0	0,002	1
1700003E1	0,284478	-0,00211	0	0,002	1
Wnt7a	0,284478	-0,00211	0	0,002	1
Fam19a1	0,284478	-0,00211	0	0,002	1
Fam19a4	0,284478	-0,00211	0	0,002	1
Hrh1	0,284478	-0,00211	0	0,002	1
Pianp	0,284478	-0,00211	0	0,002	1
9330179D1	0,284478	-0,00211	0	0,002	1
Prmt8	0,284478	-0,00211	0	0,002	1
Arhgdib	0,284478	-0,00211	0	0,002	1
Rerg	0,284478	-0,00211	0	0,002	1
Lmo3	0,284478	-0,00211	0	0,002	1
Lrmp	0,284478	-0,00211	0	0,002	1
Gm17216	0,284478	-0,00211	0	0,002	1
Pira2	0,284478	-0,00211	0	0,002	1
Lair1	0,284478	-0,00211	0	0,002	1
Syt5	0,284478	-0,00211	0	0,002	1
Shisa7	0,284478	-0,00211	0	0,002	1
Bcam	0,284478	-0,00211	0	0,002	1
Sptbn4	0,284478	-0,00211	0	0,002	1
Hcst	0,284478	-0,00211	0	0,002	1
Dmkn	0,284478	-0,00211	0	0,002	1
BC046251	0,284478	-0,00211	0	0,002	1
A730056A(	0,284478	-0,00211	0	0,002	1
Acan	0,284478	-0,00211	0	0,002	1
Gm35040	0,284478	-0,00211	0	0,002	1
Cfap161	0,284478	-0,00211	0	0,002	1
A230065N1	0,284478	-0,00211	0	0,002	1
Me3	0,284478	-0,00211	0	0,002	1
P4ha3	0,284478	-0,00211	0	0,002	1
Gm4070	0,284478	-0,00211	0	0,002	1
Calca	0,284478	-0,00211	0	0,002	1
Dnah3	0,284478	-0,00211	0	0,002	1
Hs3st4	0,284478	-0,00211	0	0,002	1
Gsg1l	0,284478	-0,00211	0	0,002	1
Doc2a	0,284478	-0,00211	0	0,002	1
Chst15	0,284478	-0,00211	0	0,002	1
Fank1	0,284478	-0,00211	0	0,002	1
Sprn	0,284478	-0,00211	0	0,002	1
Cyp2e1	0,284478	-0,00211	0	0,002	1
Esr1	0,284478	-0,00211	0	0,002	1
E030030I0	0,284478	-0,00211	0	0,002	1
Tpd52l1	0,284478	-0,00211	0	0,002	1
Hs3st5	0,284478	-0,00211	0	0,002	1
Pcbd1	0,284478	-0,00211	0	0,002	1
Gstt1	0,284478	-0,00211	0	0,002	1
Gm867	0,284478	-0,00211	0	0,002	1
Grin3b	0,284478	-0,00211	0	0,002	1
Plk5	0,284478	-0,00211	0	0,002	1
Smim24	0,284478	-0,00211	0	0,002	1
Ntn4	0,284478	-0,00211	0	0,002	1
Plxnc1	0,284478	-0,00211	0	0,002	1

Ptprr	0,284478	-0,00211	0	0,002	1
Grip1	0,284478	-0,00211	0	0,002	1
Fam155a	0,284478	-0,00211	0	0,002	1
Myo16	0,284478	-0,00211	0	0,002	1
Angpt2	0,284478	-0,00211	0	0,002	1
Adam5	0,284478	-0,00211	0	0,002	1
Gm45411	0,284478	-0,00211	0	0,002	1
Gm26632	0,284478	-0,00211	0	0,002	1
Hapln4	0,284478	-0,00211	0	0,002	1
Mast1	0,284478	-0,00211	0	0,002	1
Irx3os	0,284478	-0,00211	0	0,002	1
Bean1	0,284478	-0,00211	0	0,002	1
Slc9a5	0,284478	-0,00211	0	0,002	1
Osgin1	0,284478	-0,00211	0	0,002	1
Necab2	0,284478	-0,00211	0	0,002	1
Cbfa2t3	0,284478	-0,00211	0	0,002	1
Spire2	0,284478	-0,00211	0	0,002	1
Dbndd1	0,284478	-0,00211	0	0,002	1
Fam89a	0,284478	-0,00211	0	0,002	1
Nrp1	0,284478	-0,00211	0	0,002	1
D830030K:	0,284478	-0,00211	0	0,002	1
Wdfy4	0,284478	-0,00211	0	0,002	1
Gprin2	0,284478	-0,00211	0	0,002	1
Slc35f4	0,284478	-0,00211	0	0,002	1
4930579G:	0,284478	-0,00211	0	0,002	1
Ebf2	0,284478	-0,00211	0	0,002	1
Gm4675	0,284478	-0,00211	0	0,002	1
Pgr	0,284478	-0,00211	0	0,002	1
Scn2b	0,284478	-0,00211	0	0,002	1
Layn	0,284478	-0,00211	0	0,002	1
AI593442	0,284478	-0,00211	0	0,002	1
Celf6	0,284478	-0,00211	0	0,002	1
Skor1	0,284478	-0,00211	0	0,002	1
Gm47270	0,284478	-0,00211	0	0,002	1
Rbpms2	0,284478	-0,00211	0	0,002	1
Cgnl1	0,284478	-0,00211	0	0,002	1
Ddx43	0,284478	-0,00211	0	0,002	1
Plscr1	0,284478	-0,00211	0	0,002	1
Plscr4	0,284478	-0,00211	0	0,002	1
Sema3f	0,284478	-0,00211	0	0,002	1
Gm26614	0,284478	-0,00211	0	0,002	1
Osm	0,284478	-0,00211	0	0,002	1
Igfbp3	0,284478	-0,00211	0	0,002	1
Ikzf1	0,284478	-0,00211	0	0,002	1
Grb10	0,284478	-0,00211	0	0,002	1
Bcl11a	0,284478	-0,00211	0	0,002	1
Fbll1	0,284478	-0,00211	0	0,002	1
C1qtnf2	0,284478	-0,00211	0	0,002	1
Slc22a4	0,284478	-0,00211	0	0,002	1
P4ha2	0,284478	-0,00211	0	0,002	1
Shisa6	0,284478	-0,00211	0	0,002	1
Kcnab3	0,284478	-0,00211	0	0,002	1

Tekt1	0,284478	-0,00211	0	0,002	1
Itgae	0,284478	-0,00211	0	0,002	1
Adap2	0,284478	-0,00211	0	0,002	1
Ccl9	0,284478	-0,00211	0	0,002	1
Abi3	0,284478	-0,00211	0	0,002	1
Skap1	0,284478	-0,00211	0	0,002	1
Tbkbp1	0,284478	-0,00211	0	0,002	1
Tns4	0,284478	-0,00211	0	0,002	1
Ptges3l	0,284478	-0,00211	0	0,002	1
2810433D(	0,284478	-0,00211	0	0,002	1
Cd300lb	0,284478	-0,00211	0	0,002	1
Gng4	0,284478	-0,00211	0	0,002	1
Hist1h4j	0,284478	-0,00211	0	0,002	1
Hist1h1b	0,284478	-0,00211	0	0,002	1
Gcnt2	0,284478	-0,00211	0	0,002	1
Trpc7	0,284478	-0,00211	0	0,002	1
Dapk1	0,284478	-0,00211	0	0,002	1
1700001L1	0,284478	-0,00211	0	0,002	1
Pcsk1	0,284478	-0,00211	0	0,002	1
Hapln1	0,284478	-0,00211	0	0,002	1
Atp6ap1l	0,284478	-0,00211	0	0,002	1
Bhmt	0,284478	-0,00211	0	0,002	1
AW495222	0,284478	-0,00211	0	0,002	1
F2rl2	0,284478	-0,00211	0	0,002	1
Gm5086	0,284478	-0,00211	0	0,002	1
Gm807	0,284478	-0,00211	0	0,002	1
Cd180	0,284478	-0,00211	0	0,002	1
Rnf180	0,284478	-0,00211	0	0,002	1
Gm47997	0,284478	-0,00211	0	0,002	1
Gm33680	0,284478	-0,00211	0	0,002	1
Lrfn5	0,284478	-0,00211	0	0,002	1
Gpr135	0,284478	-0,00211	0	0,002	1
Prkch	0,284478	-0,00211	0	0,002	1
Dbpht2	0,284478	-0,00211	0	0,002	1
Rab15	0,284478	-0,00211	0	0,002	1
Syndig1l	0,284478	-0,00211	0	0,002	1
Flrt2	0,284478	-0,00211	0	0,002	1
Gpr65	0,284478	-0,00211	0	0,002	1
Kcnk10	0,284478	-0,00211	0	0,002	1
Pld4	0,284478	-0,00211	0	0,002	1
Fam105a	0,284478	-0,00211	0	0,002	1
Rgs22	0,284478	-0,00211	0	0,002	1
Gm30159	0,284478	-0,00211	0	0,002	1
Ly6e	0,284478	-0,00211	0	0,002	1
Gm48952	0,284478	-0,00211	0	0,002	1
Csf2rb2	0,284478	-0,00211	0	0,002	1
Tex33	0,284478	-0,00211	0	0,002	1
Mfng	0,284478	-0,00211	0	0,002	1
Apobec3	0,284478	-0,00211	0	0,002	1
Cacna1i	0,284478	-0,00211	0	0,002	1
Panx2	0,284478	-0,00211	0	0,002	1
AC101921.	0,284478	-0,00211	0	0,002	1

Nell2	0,284478	-0,00211	0	0,002	1
Itgb7	0,284478	-0,00211	0	0,002	1
Rmi2	0,284478	-0,00211	0	0,002	1
Pkp2	0,284478	-0,00211	0	0,002	1
Nrros	0,284478	-0,00211	0	0,002	1
Hcls1	0,284478	-0,00211	0	0,002	1
Ccdc80	0,284478	-0,00211	0	0,002	1
Chodl	0,284478	-0,00211	0	0,002	1
Gm1604a	0,284478	-0,00211	0	0,002	1
Baiap3	0,284478	-0,00211	0	0,002	1
Fgd2	0,284478	-0,00211	0	0,002	1
Olfr63	0,284478	-0,00211	0	0,002	1
H2-Aa	0,284478	-0,00211	0	0,002	1
H2-Eb1	0,284478	-0,00211	0	0,002	1
Lrfn2	0,284478	-0,00211	0	0,002	1
Tnfaip8l1	0,284478	-0,00211	0	0,002	1
Xdh	0,284478	-0,00211	0	0,002	1
Kcnk12	0,284478	-0,00211	0	0,002	1
Gm15328	0,284478	-0,00211	0	0,002	1
Pcdhac2	0,284478	-0,00211	0	0,002	1
Rab27b	0,284478	-0,00211	0	0,002	1
Gal	0,284478	-0,00211	0	0,002	1
Lrp5	0,284478	-0,00211	0	0,002	1
Npas4	0,284478	-0,00211	0	0,002	1
Cdca5	0,284478	-0,00211	0	0,002	1
Fam111a	0,284478	-0,00211	0	0,002	1
Olfr1459	0,284478	-0,00211	0	0,002	1
Trpm6	0,284478	-0,00211	0	0,002	1
Prkg1	0,284478	-0,00211	0	0,002	1
Rbp4	0,284478	-0,00211	0	0,002	1
Pnlip	0,284478	-0,00211	0	0,002	1
Ptgs2	0,284478	-0,00421	0	0,002	1
Zcchc12	0,284478	-0,00421	0	0,002	1
Rai2	0,284478	-0,00421	0	0,002	1
Gabrg1	0,284478	-0,00421	0	0,002	1
Smim17	0,284478	-0,00421	0	0,002	1
Lilr4b	0,284478	-0,00421	0	0,002	1
Tsnaxip1	0,284478	-0,00421	0	0,002	1
5033426O	0,284478	-0,00421	0	0,002	1
Vwc2	0,284478	-0,00421	0	0,002	1
Rap1gap2	0,284478	-0,00421	0	0,002	1
Rad51ap2	0,284478	-0,00421	0	0,002	1
Cdh18	0,284478	-0,00421	0	0,002	1
Gda	0,284478	-0,00421	0	0,002	1
Prrg4	0,284478	-0,00631	0	0,002	1
Atg9b	0,284478	-0,00631	0	0,002	1
Mir670hg	0,284478	-0,0084	0	0,002	1
Lmo2	0,284478	-0,0084	0	0,002	1
Pde7b	0,284478	-0,0084	0	0,002	1
Lamb1	0,284478	-0,0084	0	0,002	1
Sfrp1	0,284487	-0,00754	0,037	0,051	1
Cdon	0,284639	0,034391	0,112	0,093	1

Gm14325	0,285116	-0,00454	0,037	0,051	1
Gmppa	0,285183	-0,01906	0,207	0,238	1
Dtwd1	0,285232	-0,00393	0,084	0,105	1
Zfp280b	0,285263	-0,01143	0,057	0,074	1
Dhx40	0,285395	0,084353	0,435	0,435	1
Zhx2	0,285432	-0,00846	0,158	0,186	1
Nfyc	0,286152	0,062288	0,259	0,241	1
Abcd4	0,286373	0,004855	0,266	0,308	1
Tut1	0,286453	-0,00506	0,048	0,063	1
Gm26518	0,286534	0,08991	0,235	0,217	1
Ptdss1	0,286743	0,066453	0,462	0,462	1
Acbd4	0,28689	-0,00359	0,053	0,07	1
Pde4a	0,286972	-0,00385	0,222	0,255	1
Cntrob	0,287407	-0,0088	0,04	0,055	1
Apeh	0,287469	-0,00533	0,075	0,095	1
Rsph3a	0,287587	-0,00904	0,044	0,059	1
Smarca1	0,287593	0,034203	0,037	0,025	1
Jakmip3	0,287597	0,015648	0,523	0,601	1
Cpne3	0,287651	0,067968	0,334	0,319	1
Zfp91	0,287944	-0,01864	0,84	0,876	1
Cep68	0,287988	-0,014	0,147	0,173	1
Fam189b	0,288568	0,004816	0,066	0,084	1
Gm2a	0,288727	0,070919	0,6	0,595	1
Cab39	0,288933	0,011534	0,609	0,684	1
Tmem43	0,28894	-0,01267	0,202	0,234	1
Ubap2	0,28917	0,024635	0,301	0,354	1
Fam117b	0,289802	0,094457	0,356	0,352	1
Hspa5	0,289874	-0,03237	0,93	0,941	1
Ezh2	0,290044	0,060164	0,213	0,192	1
Mmp14	0,290049	0,025865	0,013	0,006	1
Fancb	0,290593	-0,00993	0,011	0,019	1
Txndc2	0,290617	0,01547	0,013	0,006	1
Insl6	0,290617	0,013673	0,013	0,006	1
Ifit3b	0,290617	0,017264	0,013	0,006	1
2610035D1	0,290911	0,03947	0,218	0,262	1
Ssc4d	0,291473	0,01007	0,013	0,006	1
Azin2	0,291473	0,011873	0,013	0,006	1
Gm9903	0,291473	0,011873	0,013	0,006	1
Itgb3	0,291545	-0,00263	0,055	0,072	1
Ints5.1	0,291644	0,000756	0,097	0,12	1
1700066B1	0,291755	0,016056	0,024	0,015	1
Tardbp	0,291832	0,054223	0,611	0,622	1
Gm15420	0,291993	-0,00786	0,011	0,019	1
Bend4	0,292329	0,01007	0,013	0,006	1
Pilrb2	0,292329	0,01007	0,013	0,006	1
Grm8	0,292329	0,008263	0,013	0,006	1
Stac3	0,292329	0,008263	0,013	0,006	1
Gm47428	0,292329	0,008263	0,013	0,006	1
Rai14	0,292329	0,008263	0,013	0,006	1
Slc35e3	0,2924	0,045051	0,217	0,194	1
Arid4a	0,292482	0,002776	0,4	0,464	1
Matn4	0,292518	0,010446	0,503	0,574	1

Otud5	0,292587	0,06911	0,33	0,319	1
Zfp821	0,292611	-0,01914	0,156	0,184	1
Snx29	0,293005	0,005252	0,218	0,255	1
Epc2	0,293109	0,002412	0,327	0,373	1
Klhdc8b	0,293177	-0,01068	0,011	0,019	1
Eogt	0,293177	0,016056	0,024	0,015	1
Actr3	0,293179	-0,00868	0,728	0,77	1
9430002A1	0,293187	0,006453	0,013	0,006	1
Runx2os1	0,293192	0,01517	0,013	0,006	1
Gm45605	0,293217	-0,00531	0,05	0,065	1
Rnf19b	0,293283	0,047047	0,149	0,129	1
Zscan12	0,293332	-0,00317	0,072	0,091	1
Rapgef3	0,294087	0,027207	0,182	0,158	1
Slc25a48	0,29411	-0,00424	0,011	0,019	1
Hgs	0,29411	-0,00605	0,011	0,019	1
Ctbp2	0,29434	0,006169	0,013	0,006	1
Dock6	0,294603	0,012491	0,024	0,015	1
Aff2	0,294603	0,014275	0,024	0,015	1
Cenpt	0,294882	-0,00024	0,064	0,082	1
Arid3b	0,294898	-0,00605	0,07	0,089	1
Mob1a	0,29515	0,066426	0,266	0,249	1
Itfg2	0,295194	0,016023	0,31	0,363	1
Fbxw8	0,295514	-0,00676	0,338	0,38	1
Rps6ka4	0,295579	0,014572	0,207	0,245	1
Ccdc89	0,29558	-0,00456	0,048	0,063	1
Supt3	0,29562	0,058995	0,319	0,304	1
Cep97	0,295623	0,068735	0,615	0,61	1
Snn	0,295664	0,083249	0,299	0,289	1
Mtmr9	0,295856	-8,1E-06	0,31	0,357	1
Oplah	0,295917	-0,00238	0,059	0,076	1
Gm10273	0,296034	0,010704	0,024	0,015	1
Cbx2	0,296034	0,010704	0,024	0,015	1
Zfp456	0,296034	0,010704	0,024	0,015	1
Gm48420	0,296034	0,010704	0,024	0,015	1
Arfgap2	0,296217	0,052222	0,694	0,696	1
1700008O0	0,296237	-0,00424	0,011	0,019	1
Kazald1	0,296237	-0,00424	0,011	0,019	1
Sec24c	0,296479	0,059863	0,336	0,321	1
Zfp263	0,296658	0,000257	0,185	0,215	1
Zfat	0,296988	0,021517	0,051	0,038	1
Gm48682	0,297247	0,014572	0,037	0,025	1
Gmcl1	0,297747	0,017842	0,306	0,359	1
Gpr153	0,297903	0,018137	0,018	0,011	1
Gpr35	0,298137	0,023775	0,018	0,011	1
Mfap3l	0,298183	0,03187	0,103	0,084	1
Pak3	0,298235	-0,00568	0,266	0,297	1
Faap20	0,298426	0,001833	0,13	0,156	1
Gdf11	0,298808	0,016902	0,044	0,032	1
Gpsm1	0,298879	0,001817	0,294	0,338	1
Ap1ar	0,298886	0,030519	0,248	0,3	1
Qsox2	0,299279	0,004166	0,233	0,27	1
Gm15879	0,299325	0,01308	0,018	0,011	1

Phf21b	0,299325	0,018442	0,018	0,011	1
Poll	0,299392	-0,01531	0,086	0,105	1
Isca1	0,299611	0,009355	0,218	0,255	1
Bcl2l2	0,299793	-0,0084	0,319	0,361	1
Snap91	0,30013	-0,00993	0,112	0,135	1
Syt9	0,300433	-0,00975	0,097	0,118	1
Jak1	0,300489	-0,01	0,461	0,517	1
Gm48529	0,300515	0,011286	0,018	0,011	1
Adarb1	0,300515	0,01487	0,018	0,011	1
Zfp462	0,300573	0,01176	0,402	0,462	1
Ddx27	0,300611	-0,00143	0,284	0,323	1
Dctn1	0,300656	-0,02462	0,82	0,827	1
Rbm19	0,300734	-0,01356	0,128	0,152	1
Fmn2	0,300917	0,008493	0,044	0,032	1
Coq8b	0,301133	0,071568	0,228	0,209	1
Art3	0,301665	-0,11525	0,149	0,169	1
Tle2	0,301754	0,057375	0,226	0,207	1
Xrn2	0,301923	0,029825	0,484	0,551	1
Nfkbil1	0,302198	-0,02507	0,165	0,19	1
Gnb1	0,302639	0,039178	0,972	0,975	1
Per2	0,302671	0,014572	0,018	0,011	1
Ptpn18	0,302911	0,009201	0,018	0,011	1
Ogfr	0,302973	0,011329	0,347	0,405	1
Miip	0,303002	0,029242	0,114	0,095	1
Usp5	0,30313	0,011839	0,558	0,635	1
Wac	0,303186	0,08517	0,635	0,662	1
Zbtb7b	0,303308	0,057895	0,325	0,312	1
Lrguk	0,304111	0,007404	0,018	0,011	1
Kif13a	0,304112	-0,0139	0,655	0,696	1
Atr	0,304374	-0,00663	0,239	0,276	1
Zfp941	0,30448	-0,01043	0,013	0,021	1
Pde10a	0,304536	0,025726	0,031	0,021	1
Dmxl2	0,304566	0,07181	0,239	0,222	1
Cuedc2	0,304615	-0,01201	0,8	0,857	1
Il17rb	0,304855	-0,01386	0,086	0,105	1
Ndel1	0,305109	0,003251	0,378	0,43	1
Trub1	0,305228	0,00341	0,189	0,222	1
Lnpk	0,305342	0,001411	0,492	0,565	1
Prex2	0,305377	-0,00812	0,013	0,021	1
Dtx4	0,305426	-0,01778	0,081	0,099	1
Hif1an	0,305522	-0,00196	0,226	0,262	1
Rpl27	0,30566	-0,00284	0,969	0,958	1
AC149090.	0,305728	0,081824	0,776	0,768	1
Nipbl	0,305774	0,077315	0,782	0,77	1
Pak2	0,305862	0,039103	0,923	0,924	1
E2f2	0,306293	-0,00656	0,013	0,021	1
Ddost	0,306314	-0,00898	0,723	0,764	1
Dag1	0,306514	0,002842	0,156	0,184	1
Olfml1	0,306848	0,01904	0,563	0,637	1
Nucb1	0,306873	0,021067	0,481	0,57	1
Lrp6	0,30693	0,077679	0,552	0,57	1
Etf1	0,306948	0,011873	0,42	0,487	1

Ppp4r2	0,307001	0,06929	0,661	0,671	1
Pld2	0,307204	0,05326	0,143	0,124	1
Pml	0,307522	0,051559	0,125	0,108	1
Zbtb18	0,307561	-0,01827	0,139	0,165	1
Mapkapk3	0,307656	-0,0027	0,013	0,021	1
Cage1	0,307656	-0,00631	0,013	0,021	1
Eef1akmt4	0,307656	-0,00631	0,013	0,021	1
Zfp772	0,308325	0,02294	0,053	0,04	1
Zbtb4	0,30843	0,101168	0,339	0,333	1
Kpna6	0,308516	-0,01099	0,316	0,354	1
Lrrc41	0,308632	-0,00327	0,253	0,291	1
Mtif2	0,308912	-0,01616	0,194	0,224	1
Vezt	0,309011	0,005604	0,358	0,407	1
Exoc2	0,309082	-0,00424	0,229	0,264	1
Rrp9	0,309114	0,02735	0,222	0,262	1
Mrm1	0,309436	-0,01908	0,084	0,103	1
Fgfr4	0,309447	0,026073	0,053	0,04	1
Gm20721	0,309945	-0,0045	0,013	0,021	1
Pcgf6	0,310128	-0,01996	0,108	0,129	1
Gm26609	0,310143	0,03346	0,039	0,027	1
Zbtb49	0,310159	0,005497	0,051	0,068	1
Rnf44	0,310333	-0,01419	0,378	0,426	1
Zfp954	0,310655	0,033735	0,143	0,122	1
Eml4	0,310715	-0,00398	0,11	0,133	1
Fn3krp	0,310723	0,039527	0,189	0,167	1
H2afy	0,310879	-0,03848	0,602	0,627	1
Flvcr1	0,31112	0,036739	0,125	0,105	1
Wdr17	0,312189	0,018093	0,031	0,021	1
Gm15559	0,312246	0,008058	0,013	0,021	1
Nnat	0,312354	0,020914	0,053	0,04	1
Mcm6	0,312691	0,023828	0,077	0,061	1
Akap10	0,31309	0,004225	0,158	0,188	1
Slc27a1	0,313121	0,04333	0,422	0,485	1
Napb	0,31317	0,050605	0,15	0,131	1
Rbpj	0,313381	-0,00306	0,391	0,443	1
Nol11	0,313441	-0,00723	0,255	0,285	1
Gpr21	0,313635	0,013391	0,031	0,021	1
Cadm3	0,31377	0,026641	0,152	0,131	1
Urm1	0,314199	-0,01307	0,758	0,814	1
Ppp3ca	0,31454	-0,00289	0,916	0,911	1
Cmtr2	0,315128	0,018302	0,061	0,046	1
Polg	0,315192	0,010624	0,174	0,205	1
Tmem127	0,315538	0,015132	0,398	0,464	1
Aebp2	0,315817	0,07356	0,53	0,553	1
Caly	0,315943	-0,00837	0,015	0,023	1
Zfp54	0,315943	-0,00837	0,015	0,023	1
Nfe2l3	0,31652	-0,00497	0,593	0,665	1
Ccnb1ip1	0,316874	0,018646	0,046	0,034	1
Trappc8	0,317003	0,002046	0,354	0,401	1
Sh3tc2	0,317357	4,18E-05	0,235	0,268	1
Slc8a3	0,317443	-0,00627	0,138	0,162	1
Nid1	0,317461	0,051755	0,218	0,196	1

Tmem176b	0,317616	0,04726	0,139	0,12	1
Epha1	0,317658	0,01746	0,053	0,04	1
Ipo13	0,317817	-0,02237	0,683	0,728	1
Mapk8ip1	0,318183	-0,02257	1	0,998	1
Pcdhb11	0,318229	0,024167	0,039	0,027	1
Cd3eap	0,318404	0,008281	0,218	0,253	1
Arrb2	0,318522	-0,0003	0,112	0,135	1
Crnkl1	0,318648	0,006375	0,251	0,289	1
Lrrcc1	0,318813	-0,0218	0,259	0,291	1
1810032O	0,320026	-0,00462	0,084	0,103	1
Gxylt1	0,320151	0,091372	0,352	0,346	1
Brpf1	0,320368	-0,00985	0,189	0,215	1
Furin	0,320415	-0,00625	0,211	0,243	1
Gm44037	0,3208	-0,00683	0,004	0,008	1
Pcolce	0,3208	-0,00683	0,004	0,008	1
Gm26540	0,3208	-0,00683	0,004	0,008	1
Llgl2	0,3208	-0,00683	0,004	0,008	1
Serpina3m	0,3208	-0,00683	0,004	0,008	1
Gm38399	0,320811	-0,00476	0,015	0,023	1
Acsl4	0,321101	0,062194	0,207	0,188	1
Sap130	0,321541	0,047212	0,11	0,093	1
2900009J0	0,321585	-0,00474	0,004	0,008	1
Aspm	0,321585	-0,00474	0,004	0,008	1
Rgs1	0,321585	-0,00474	0,004	0,008	1
Gm37640	0,321585	-0,00474	0,004	0,008	1
4933430I1	0,321585	-0,00474	0,004	0,008	1
Tes	0,321585	-0,00474	0,004	0,008	1
Vmn1r4	0,321585	-0,00474	0,004	0,008	1
Ccdc8	0,321585	-0,00474	0,004	0,008	1
Gm45359	0,321585	-0,00474	0,004	0,008	1
4933417D	0,321585	-0,00474	0,004	0,008	1
Gm47794	0,321585	-0,00474	0,004	0,008	1
AC061963.	0,321585	-0,00474	0,004	0,008	1
Gm10642	0,321585	-0,00474	0,004	0,008	1
A730071L1	0,321585	-0,00474	0,004	0,008	1
Olfr318	0,321585	-0,00474	0,004	0,008	1
Gm12278	0,321585	-0,00474	0,004	0,008	1
Gm48610	0,321585	-0,00474	0,004	0,008	1
Cthrc1	0,321585	-0,00474	0,004	0,008	1
AC125141.	0,321585	-0,00474	0,004	0,008	1
G430049J0	0,321585	-0,00474	0,004	0,008	1
Rgs7bp	0,321587	-0,00709	0,004	0,008	1
Gm16212	0,321706	-0,00322	0,015	0,023	1
Haus6	0,321747	-0,01237	0,156	0,181	1
Pja1	0,321938	0,027135	0,53	0,601	1
Drp2	0,32198	-0,0322	0,004	0,008	1
Slc12a6	0,322332	0,020557	0,33	0,378	1
Hspa1l	0,322373	-0,005	0,004	0,008	1
Krr1	0,322416	0,024769	0,305	0,35	1
9330104G	0,322596	-0,01008	0,081	0,099	1
1700021N	0,322767	-0,00318	0,004	0,008	1
Gm26625	0,323161	-0,00109	0,004	0,008	1

Unc80	0,323161	-0,00291	0,004	0,008	1
A830008E2	0,323161	-0,00291	0,004	0,008	1
Olfr1124	0,323161	-0,00291	0,004	0,008	1
Draxin	0,323161	-0,00291	0,004	0,008	1
Styxl1	0,323161	-0,00291	0,004	0,008	1
Sap25	0,323161	-0,00291	0,004	0,008	1
Rasgef1a	0,323161	-0,00291	0,004	0,008	1
Wdr93	0,323161	-0,00291	0,004	0,008	1
Agpat2	0,323164	0,022837	0,026	0,017	1
Gm14302	0,323262	0,000632	0,015	0,023	1
Pqlc1	0,323262	-0,00116	0,015	0,023	1
Ablim2	0,323874	0,095633	0,49	0,5	1
H2-T22	0,323902	0,036878	0,099	0,082	1
Pip4k2b	0,324155	0,034981	0,22	0,198	1
Trmt44	0,324422	-0,01601	0,066	0,082	1
Smg8	0,324497	-0,01159	0,073	0,091	1
Acap3	0,324646	0,002469	0,152	0,179	1
Masp2	0,324741	0,002546	0,004	0,008	1
Slc35f1	0,324741	-0,00109	0,004	0,008	1
Chrne	0,324741	-0,00109	0,004	0,008	1
Ralgapa1	0,325012	0,00516	0,466	0,523	1
Bop1	0,325043	0,015737	0,229	0,268	1
Cdc25a	0,32509	-0,00818	0,081	0,099	1
Caprin2	0,32517	0,060567	0,094	0,078	1
Taok2	0,325886	0,030702	0,356	0,426	1
Smc6	0,326521	0,004218	0,609	0,69	1
Igsf8	0,326618	0,046437	0,756	0,724	1
Qk	0,326684	0,032236	1	0,998	1
Tsr1	0,32675	-0,00821	0,075	0,093	1
Dync1li2	0,326802	0,054189	0,897	0,873	1
Mroh1	0,326834	0,00234	0,143	0,169	1
D2hgdh	0,326917	0,010056	0,11	0,133	1
Rnf141	0,326956	0,063127	0,888	0,878	1
Rufy2	0,326984	0,011019	0,475	0,563	1
Sfswap	0,32701	0,071519	0,563	0,578	1
Htra1	0,327105	0,145125	0,754	0,785	1
Inppl1	0,327121	0,0453	0,218	0,196	1
Phactr3	0,327157	0,053643	0,483	0,468	1
Klhl36	0,327534	-0,00707	0,017	0,025	1
Olfm1	0,327792	0,005472	0,372	0,428	1
Styx	0,328146	0,040803	0,2	0,236	1
Gm17275	0,328163	0,022837	0,026	0,017	1
Tpm3	0,328207	0,071011	0,862	0,882	1
Pkn1	0,328257	0,006764	0,341	0,386	1
Kat14	0,328738	0,004773	0,18	0,209	1
Nup155	0,329093	0,033651	0,13	0,112	1
Unk	0,329286	0,025218	0,228	0,266	1
Fam219a	0,329324	0,013121	0,418	0,485	1
Tmem30a	0,32942	0,052822	0,983	0,987	1
Fam173a	0,32983	-0,01117	0,631	0,7	1
Gm10471	0,329839	0,015757	0,026	0,017	1
Fam227a	0,329839	0,015757	0,026	0,017	1

Fbxo33	0,329906	0,070493	0,222	0,268	1
Usp42	0,330171	0,076645	0,213	0,198	1
E330009J0	0,330221	0,094389	0,495	0,506	1
Dhdh	0,330388	-0,01083	0,073	0,091	1
Prdm5	0,330688	0,002596	0,158	0,186	1
Nup205	0,331317	0,034191	0,161	0,141	1
Unc93b1	0,331357	-0,01316	0,145	0,169	1
Wnk3	0,331479	-0,01578	0,061	0,076	1
Myo10	0,331586	-0,00913	0,018	0,027	1
Zfp276	0,331775	-0,00013	0,334	0,378	1
Mybl1	0,331816	-0,01113	0,068	0,084	1
E430024P1	0,332053	-0,00322	0,017	0,025	1
Shcbp1	0,332053	0,000362	0,017	0,025	1
Gm10382	0,332053	-0,00143	0,017	0,025	1
Klhl18	0,332316	0,011794	0,224	0,259	1
Cenpf	0,332519	0,058143	0,048	0,036	1
Scn8a	0,332773	0,036121	0,073	0,059	1
Abhd11	0,332887	-0,00403	0,233	0,266	1
Nemf	0,333004	0,007459	0,624	0,679	1
Bloc1s1	0,33309	0,018106	0,65	0,749	1
Prpf19	0,333093	-0,03312	0,736	0,747	1
1700037H(	0,333421	-0,00967	0,073	0,091	1
Rgma	0,333645	0,000484	0,598	0,662	1
Zbtb3	0,333651	-0,00707	0,018	0,027	1
Fkbp5	0,333977	0,025099	0,033	0,023	1
Rack1	0,334064	-0,00575	0,947	0,939	1
1700047M	0,334112	-0,00387	0,928	0,939	1
Ccnd1	0,334231	0,163729	0,05	0,065	1
Tnks	0,334492	0,065317	0,668	0,679	1
Atp13a1	0,334546	-0,01056	0,255	0,287	1
Isy1	0,334567	0,003635	0,191	0,222	1
Grik3	0,33492	0,020458	0,026	0,017	1
Hdlbp	0,335437	-0,01338	0,639	0,69	1
Dhx33	0,335499	-0,00896	0,068	0,084	1
Mki67	0,336359	-0,00348	0,018	0,027	1
Zfp868	0,336545	0,002207	0,139	0,165	1
Gm26664	0,336842	-0,01191	0,022	0,032	1
Tspan4	0,336984	-0,02268	0,026	0,036	1
Gm1979	0,337109	0,012168	0,009	0,004	1
Anapc5	0,337111	0,048144	0,866	0,854	1
Rasl12	0,337116	-0,01053	0,138	0,16	1
Pcdhgb2	0,337201	-0,00374	0,018	0,027	1
Car3	0,337273	0,007493	0,017	0,025	1
Rnf213	0,33758	0,025457	0,055	0,042	1
Yes1	0,337617	0,039499	0,176	0,156	1
Bahd1	0,337822	-0,0092	0,07	0,086	1
Colgalt1	0,337834	0,010806	0,306	0,35	1
Fam83e	0,33786	0,010362	0,009	0,004	1
Dpf1	0,337877	0,026842	0,033	0,023	1
Ctxn3	0,338224	-0,00605	0,02	0,03	1
Ppp3r1	0,338231	0,001536	0,372	0,42	1
Vamp1	0,338403	0,002641	0,084	0,103	1

Myl6b	0,338517	0,017135	0,072	0,057	1
Gm26601	0,338537	0,028373	0,09	0,074	1
Nr1h3	0,338612	0,012168	0,009	0,004	1
Etnk2	0,338612	0,008552	0,009	0,004	1
Klk7	0,338612	0,008552	0,009	0,004	1
Clstn2	0,338612	0,008552	0,009	0,004	1
Cgref1	0,338612	0,013972	0,009	0,004	1
Spata19	0,338612	0,010362	0,009	0,004	1
Csnk1d	0,338772	0,024911	0,442	0,513	1
Rab33a	0,339026	-0,00342	0,209	0,238	1
Tdp2	0,339108	-0,00479	0,204	0,234	1
Pitpnm2	0,339365	0,008552	0,009	0,004	1
A330074K2	0,339365	0,008552	0,009	0,004	1
Vsig10l	0,339365	0,006738	0,009	0,004	1
Gm11149	0,339365	0,006738	0,009	0,004	1
Jag2	0,339365	0,006738	0,009	0,004	1
Rab3il1	0,339403	-0,0099	0,072	0,089	1
Cdk14	0,339405	-0,01175	0,103	0,122	1
Kansl3	0,339591	0,023935	0,284	0,329	1
4930558K0	0,340118	0,004922	0,009	0,004	1
Gm26691	0,340118	0,004922	0,009	0,004	1
Map3k6	0,340118	0,004922	0,009	0,004	1
Triml2	0,340118	0,004922	0,009	0,004	1
Ces2f	0,340118	0,004922	0,009	0,004	1
C230035I1	0,340118	0,004922	0,009	0,004	1
Gm47071	0,340118	0,004922	0,009	0,004	1
Gm26694	0,340118	0,004922	0,009	0,004	1
Slc38a9	0,340119	0,012916	0,396	0,451	1
Habp4	0,340145	-0,00649	0,141	0,165	1
Top2b	0,340462	-0,00536	0,631	0,703	1
Klhl2	0,340679	0,046569	0,978	0,979	1
Gpsm2	0,340717	0,025433	0,262	0,306	1
Tmem86b	0,341102	0,020992	0,04	0,03	1
Mapre1	0,341267	0,078238	0,725	0,768	1
Trpc1	0,341496	-0,0006	0,393	0,441	1
Arhgef3	0,341507	-0,01643	0,026	0,036	1
A230083G1	0,34201	0,002823	0,009	0,004	1
Mfsd12	0,342467	0,056643	0,088	0,074	1
Ikzf4	0,342647	0,016356	0,02	0,013	1
Hap1	0,342746	-0,01037	0,024	0,034	1
Nup62	0,342788	0,029981	0,174	0,154	1
Ccdc93	0,343213	-0,01115	0,105	0,124	1
Fchsd1	0,343296	0,014867	0,033	0,023	1
Zfp873	0,343302	-0,01191	0,026	0,036	1
Ncmap	0,343439	-0,01191	0,022	0,032	1
Zhx3	0,343968	0,005093	0,255	0,291	1
9130023H1	0,343983	-0,01175	0,051	0,065	1
Gm17477	0,344221	-0,00426	0,022	0,032	1
Pcdhb9	0,344246	-0,00806	0,022	0,032	1
8030462N1	0,344461	0,004555	0,068	0,084	1
Rangap1	0,344482	0,007676	0,569	0,643	1
Gga1	0,344569	0,016653	0,33	0,371	1

Svil	0,34478	-0,01726	0,09	0,108	1
Wasf2	0,345049	0,067256	0,677	0,7	1
Lypla2	0,345301	0,00494	0,222	0,251	1
Gpt	0,345611	0,088702	0,521	0,527	1
Chd9	0,345626	0,004435	0,534	0,597	1
Phf20l1	0,345787	0,074628	0,633	0,654	1
Prpf6	0,345869	0,006826	0,31	0,35	1
C2cd5	0,345883	0,071129	0,365	0,352	1
Spg20	0,345895	0,058856	0,972	0,96	1
Tmem107	0,345955	0,01602	0,048	0,036	1
Tgfbr3	0,34607	0,056166	0,174	0,158	1
Efna1	0,346123	0,081223	0,349	0,34	1
Mmp11	0,346193	-0,01917	0,04	0,053	1
Hoxa5	0,346222	0,033232	0,05	0,038	1
Tet3	0,346289	0,131605	0,477	0,513	1
Dtx2	0,346353	-0,0093	0,103	0,122	1
Gm45250	0,346382	-0,0063	0,022	0,032	1
Fgd1	0,346506	-0,00783	0,024	0,034	1
Gng10	0,346506	-0,00783	0,024	0,034	1
Sgo2a	0,34654	-0,00883	0,024	0,034	1
Psmc6	0,346792	0,003078	0,802	0,85	1
Kif3b	0,346806	0,005652	0,134	0,158	1
H2-DMa	0,347061	0,010994	0,02	0,013	1
Arid3a	0,347371	-0,00678	0,055	0,07	1
Cbx8	0,347399	-0,00384	0,057	0,072	1
Wdr27	0,347411	-0,00703	0,053	0,068	1
Ralgapa2	0,347413	0,009862	0,204	0,236	1
Ipo7	0,347488	0,030121	0,396	0,47	1
Atn1	0,347513	0,089291	0,488	0,506	1
Mpp5	0,347642	0,042743	0,846	0,835	1
Cnppd1	0,347845	0,092318	0,4	0,416	1
Rubcn	0,347892	0,065561	0,428	0,424	1
Cers5	0,34822	0,003713	0,345	0,388	1
Grk5	0,348247	-0,00859	0,024	0,034	1
Prkci	0,348314	-0,0116	0,143	0,165	1
Trim65	0,348521	-0,01113	0,064	0,08	1
Cdc42bpb	0,348865	-0,0086	0,33	0,369	1
1700052K:	0,348974	-0,00481	0,048	0,061	1
Pi4kb	0,349183	0,034159	0,226	0,205	1
Gdap2	0,349309	-0,01096	0,138	0,16	1
Gm42695	0,349736	-0,00426	0,022	0,032	1
D830024N	0,349811	-0,00977	0,05	0,063	1
Cdca8	0,350141	-0,0063	0,024	0,034	1
Mapk8	0,350176	0,07137	0,317	0,31	1
Zfp454	0,350188	-0,00554	0,029	0,04	1
Luc7l	0,35032	0,071919	0,433	0,437	1
Tmem177	0,35072	-0,01158	0,035	0,046	1
Nova2	0,35127	0,007119	0,02	0,013	1
Wdr13	0,351443	0,002349	0,314	0,352	1
Aco1	0,351587	0,005692	0,218	0,253	1
Dnttip2	0,351965	0,037261	0,499	0,586	1
Slc35b4	0,35203	-0,00906	0,237	0,268	1

Col3a1	0,352049	0,016379	0,002	0	1
Pax3	0,352049	0,016379	0,002	0	1
Rab11fip1	0,352049	0,010949	0,002	0	1
Oxtr	0,352049	0,009132	0,002	0	1
Eln	0,352049	0,007313	0,002	0	1
Rcn3	0,352049	0,007313	0,002	0	1
Lamb2	0,352049	0,007313	0,002	0	1
Tmprss7	0,352049	0,007313	0,002	0	1
Chrm1	0,352049	0,007313	0,002	0	1
Ccdc120	0,352049	0,005489	0,002	0	1
Necab1	0,352049	0,005489	0,002	0	1
Reep1	0,352049	0,005489	0,002	0	1
Adm	0,352049	0,005489	0,002	0	1
Adamts10	0,352049	0,005489	0,002	0	1
4933407L2	0,352049	0,003663	0,002	0	1
Ildr2	0,352049	0,003663	0,002	0	1
Cdca7	0,352049	0,003663	0,002	0	1
4933423P2	0,352049	0,003663	0,002	0	1
Ncaph	0,352049	0,003663	0,002	0	1
Stoml3	0,352049	0,003663	0,002	0	1
Cd53	0,352049	0,003663	0,002	0	1
Tctex1d4	0,352049	0,003663	0,002	0	1
Ptpru	0,352049	0,003663	0,002	0	1
4930553P1	0,352049	0,003663	0,002	0	1
Gm44148	0,352049	0,003663	0,002	0	1
Dll3	0,352049	0,003663	0,002	0	1
Kcnmb4os2	0,352049	0,003663	0,002	0	1
Mmp19	0,352049	0,003663	0,002	0	1
Hpgd	0,352049	0,003663	0,002	0	1
Adcy7	0,352049	0,003663	0,002	0	1
Adgrg3	0,352049	0,003663	0,002	0	1
Nid2	0,352049	0,003663	0,002	0	1
Dleu7	0,352049	0,003663	0,002	0	1
Gpc6	0,352049	0,003663	0,002	0	1
Naalad2	0,352049	0,003663	0,002	0	1
Havcr2	0,352049	0,003663	0,002	0	1
Sgcd	0,352049	0,003663	0,002	0	1
N4bp3	0,352049	0,003663	0,002	0	1
Cntnap1	0,352049	0,003663	0,002	0	1
Evpl	0,352049	0,003663	0,002	0	1
AI463229	0,352049	0,003663	0,002	0	1
Hk3	0,352049	0,003663	0,002	0	1
Gm31946	0,352049	0,003663	0,002	0	1
Ankrd34b	0,352049	0,003663	0,002	0	1
K230010J2	0,352049	0,003663	0,002	0	1
Cacng2	0,352049	0,003663	0,002	0	1
Myo1f	0,352049	0,003663	0,002	0	1
C230072F1	0,352049	0,003663	0,002	0	1
Cbln2	0,352049	0,003663	0,002	0	1
Sptbn2	0,352049	0,003663	0,002	0	1
Blnk	0,352049	0,003663	0,002	0	1
Pi15	0,352049	0,001833	0,002	0	1

Gm973	0,352049	0,001833	0,002	0	1
Sp140	0,352049	0,001833	0,002	0	1
Bok	0,352049	0,001833	0,002	0	1
Cntnap5a	0,352049	0,001833	0,002	0	1
Dyrk3	0,352049	0,001833	0,002	0	1
Prelp	0,352049	0,001833	0,002	0	1
Rcsd1	0,352049	0,001833	0,002	0	1
Fcgr3	0,352049	0,001833	0,002	0	1
Klhdc9	0,352049	0,001833	0,002	0	1
Gm34342	0,352049	0,001833	0,002	0	1
Proser2	0,352049	0,001833	0,002	0	1
Card9	0,352049	0,001833	0,002	0	1
1700007K:	0,352049	0,001833	0,002	0	1
Lmx1b	0,352049	0,001833	0,002	0	1
Scn3a	0,352049	0,001833	0,002	0	1
Spi1	0,352049	0,001833	0,002	0	1
Pak6	0,352049	0,001833	0,002	0	1
Plcb2	0,352049	0,001833	0,002	0	1
Mertk	0,352049	0,001833	0,002	0	1
Lbp	0,352049	0,001833	0,002	0	1
Sycp2	0,352049	0,001833	0,002	0	1
Was	0,352049	0,001833	0,002	0	1
Slc25a43	0,352049	0,001833	0,002	0	1
Hs6st2	0,352049	0,001833	0,002	0	1
Pnma3	0,352049	0,001833	0,002	0	1
Tmem164	0,352049	0,001833	0,002	0	1
Chrdl1	0,352049	0,001833	0,002	0	1
Gm16337	0,352049	0,001833	0,002	0	1
Wdr49	0,352049	0,001833	0,002	0	1
Kirrel	0,352049	0,001833	0,002	0	1
Lrrc71	0,352049	0,001833	0,002	0	1
Dennd2c	0,352049	0,001833	0,002	0	1
Slc44a3	0,352049	0,001833	0,002	0	1
Dapp1	0,352049	0,001833	0,002	0	1
Col24a1	0,352049	0,001833	0,002	0	1
Slc44a5	0,352049	0,001833	0,002	0	1
Penk	0,352049	0,001833	0,002	0	1
Gem	0,352049	0,001833	0,002	0	1
Elavl2	0,352049	0,001833	0,002	0	1
Podn	0,352049	0,001833	0,002	0	1
Gm12968	0,352049	0,001833	0,002	0	1
Pdpn	0,352049	0,001833	0,002	0	1
Tnfrsf1b	0,352049	0,001833	0,002	0	1
Igfbp7	0,352049	0,001833	0,002	0	1
Stbd1	0,352049	0,001833	0,002	0	1
Prdm8	0,352049	0,001833	0,002	0	1
Cryba4	0,352049	0,001833	0,002	0	1
Sez6l	0,352049	0,001833	0,002	0	1
Ksr2	0,352049	0,001833	0,002	0	1
Tfr2	0,352049	0,001833	0,002	0	1
Gm15410	0,352049	0,001833	0,002	0	1
Gm3294	0,352049	0,001833	0,002	0	1

Prrt4	0,352049	0,001833	0,002	0	1
Gprin3	0,352049	0,001833	0,002	0	1
Prokr1	0,352049	0,001833	0,002	0	1
0610040F(	0,352049	0,001833	0,002	0	1
4930540M	0,352049	0,001833	0,002	0	1
Mfap5	0,352049	0,001833	0,002	0	1
Apobec1	0,352049	0,001833	0,002	0	1
Vwf	0,352049	0,001833	0,002	0	1
Cracr2a	0,352049	0,001833	0,002	0	1
Pde3a	0,352049	0,001833	0,002	0	1
Usp29	0,352049	0,001833	0,002	0	1
Pou2f2	0,352049	0,001833	0,002	0	1
Grin2d	0,352049	0,001833	0,002	0	1
E2f8	0,352049	0,001833	0,002	0	1
Fam189a1	0,352049	0,001833	0,002	0	1
Hapln3	0,352049	0,001833	0,002	0	1
P2ry6	0,352049	0,001833	0,002	0	1
Folr1	0,352049	0,001833	0,002	0	1
Igsf6	0,352049	0,001833	0,002	0	1
Il21r	0,352049	0,001833	0,002	0	1
Gm44672	0,352049	0,001833	0,002	0	1
6430531B:	0,352049	0,001833	0,002	0	1
Lsp1	0,352049	0,001833	0,002	0	1
Samd5	0,352049	0,001833	0,002	0	1
Myb	0,352049	0,001833	0,002	0	1
Vsir	0,352049	0,001833	0,002	0	1
Cabcoco1	0,352049	0,001833	0,002	0	1
Rhobtb1	0,352049	0,001833	0,002	0	1
Trpm2	0,352049	0,001833	0,002	0	1
Slc1a6	0,352049	0,001833	0,002	0	1
Shc2	0,352049	0,001833	0,002	0	1
Nmrk2	0,352049	0,001833	0,002	0	1
Ascl1	0,352049	0,001833	0,002	0	1
Cfap54	0,352049	0,001833	0,002	0	1
Lin7a	0,352049	0,001833	0,002	0	1
E2f7	0,352049	0,001833	0,002	0	1
Myo1a	0,352049	0,001833	0,002	0	1
Vegfc	0,352049	0,001833	0,002	0	1
Cyp4f18	0,352049	0,001833	0,002	0	1
Nod2	0,352049	0,001833	0,002	0	1
Hydin	0,352049	0,001833	0,002	0	1
Gm2237	0,352049	0,001833	0,002	0	1
Dnah12	0,352049	0,001833	0,002	0	1
1810011H:	0,352049	0,001833	0,002	0	1
Gch1	0,352049	0,001833	0,002	0	1
Phf11b	0,352049	0,001833	0,002	0	1
Arl11	0,352049	0,001833	0,002	0	1
Fam167a	0,352049	0,001833	0,002	0	1
Pbk	0,352049	0,001833	0,002	0	1
Rubcnl	0,352049	0,001833	0,002	0	1
Fam216b	0,352049	0,001833	0,002	0	1
Fat3	0,352049	0,001833	0,002	0	1

Zfp558	0,352049	0,001833	0,002	0	1
Gm26592	0,352049	0,001833	0,002	0	1
Rab27a	0,352049	0,001833	0,002	0	1
Nme9	0,352049	0,001833	0,002	0	1
Cx3cr1	0,352049	0,001833	0,002	0	1
Ttc21a	0,352049	0,001833	0,002	0	1
Myo1g	0,352049	0,001833	0,002	0	1
Gm16170	0,352049	0,001833	0,002	0	1
Gm12185	0,352049	0,001833	0,002	0	1
Obscn	0,352049	0,001833	0,002	0	1
Dnah2	0,352049	0,001833	0,002	0	1
Bcl6b	0,352049	0,001833	0,002	0	1
Cxcl16	0,352049	0,001833	0,002	0	1
Haspin	0,352049	0,001833	0,002	0	1
Tmigd1	0,352049	0,001833	0,002	0	1
Coro6	0,352049	0,001833	0,002	0	1
Ccl5	0,352049	0,001833	0,002	0	1
Nog	0,352049	0,001833	0,002	0	1
Top2a	0,352049	0,001833	0,002	0	1
Aoc2	0,352049	0,001833	0,002	0	1
Gjc1	0,352049	0,001833	0,002	0	1
Nptx1	0,352049	0,001833	0,002	0	1
Pycr1	0,352049	0,001833	0,002	0	1
Zfp184	0,352049	0,001833	0,002	0	1
Hist1h2bg	0,352049	0,001833	0,002	0	1
S1pr3	0,352049	0,001833	0,002	0	1
Diras2	0,352049	0,001833	0,002	0	1
Gm26555	0,352049	0,001833	0,002	0	1
Slc6a19	0,352049	0,001833	0,002	0	1
Iqgap2	0,352049	0,001833	0,002	0	1
Gpx8	0,352049	0,001833	0,002	0	1
Itga1	0,352049	0,001833	0,002	0	1
L3hypdh	0,352049	0,001833	0,002	0	1
Ppp1r36	0,352049	0,001833	0,002	0	1
Ccdc177	0,352049	0,001833	0,002	0	1
Acot4	0,352049	0,001833	0,002	0	1
4732463B(	0,352049	0,001833	0,002	0	1
Gpr68	0,352049	0,001833	0,002	0	1
Tmem179	0,352049	0,001833	0,002	0	1
Cdca7l	0,352049	0,001833	0,002	0	1
Plcxd3	0,352049	0,001833	0,002	0	1
Dnah5	0,352049	0,001833	0,002	0	1
Adcy8	0,352049	0,001833	0,002	0	1
Gsdmd	0,352049	0,001833	0,002	0	1
Rac2	0,352049	0,001833	0,002	0	1
Kdelr3	0,352049	0,001833	0,002	0	1
Pnpla3	0,352049	0,001833	0,002	0	1
Celsr1	0,352049	0,001833	0,002	0	1
Mapk11	0,352049	0,001833	0,002	0	1
Odf3b	0,352049	0,001833	0,002	0	1
Syt10	0,352049	0,001833	0,002	0	1
Lrrk2	0,352049	0,001833	0,002	0	1

Ccdc65	0,352049	0,001833	0,002	0	1
Dhh	0,352049	0,001833	0,002	0	1
Espl1	0,352049	0,001833	0,002	0	1
AC118542.	0,352049	0,001833	0,002	0	1
Gp1bb	0,352049	0,001833	0,002	0	1
Ece2	0,352049	0,001833	0,002	0	1
Gm26569	0,352049	0,001833	0,002	0	1
Cfap44	0,352049	0,001833	0,002	0	1
Cd200r4	0,352049	0,001833	0,002	0	1
Epha3	0,352049	0,001833	0,002	0	1
Cxadr	0,352049	0,001833	0,002	0	1
1700110C:	0,352049	0,001833	0,002	0	1
Airn	0,352049	0,001833	0,002	0	1
Rab44	0,352049	0,001833	0,002	0	1
Tbc1d22bc	0,352049	0,001833	0,002	0	1
H2-DMb1	0,352049	0,001833	0,002	0	1
Gm11131	0,352049	0,001833	0,002	0	1
Plin5	0,352049	0,001833	0,002	0	1
Gm4951	0,352049	0,001833	0,002	0	1
Rnf165	0,352049	0,001833	0,002	0	1
Slc14a1	0,352049	0,001833	0,002	0	1
Fermt3	0,352049	0,001833	0,002	0	1
Ms4a6d	0,352049	0,001833	0,002	0	1
Mamdc2	0,352049	0,001833	0,002	0	1
Fas	0,352049	0,001833	0,002	0	1
Ifit1bl1	0,352049	0,001833	0,002	0	1
Hhex	0,352049	0,001833	0,002	0	1
Entpd1	0,352049	0,001833	0,002	0	1
Klk9	0,352284	0,03116	0,064	0,051	1
Donson	0,352479	-0,00143	0,059	0,074	1
Diexf	0,352536	0,004591	0,139	0,165	1
Ifit3	0,352544	0,050023	0,015	0,008	1
Pcdhgb6	0,352625	-0,00426	0,024	0,034	1
Nsun5	0,352686	-0,00172	0,119	0,141	1
Fgf11	0,352712	-0,00248	0,022	0,032	1
Tbc1d10b	0,352786	0,06102	0,75	0,787	1
BC049715	0,352838	0,022313	0,015	0,008	1
Pag1	0,35287	-0,00233	0,211	0,241	1
Ceacam2	0,352897	-0,01175	0,042	0,055	1
Rab26	0,353211	-0,00452	0,024	0,034	1
Daam2	0,353539	0,070537	0,921	0,924	1
Rab40b	0,353777	-0,00933	0,033	0,044	1
Tmem209	0,353786	-0,00128	0,261	0,291	1
Tmem2	0,354022	0,013376	0,015	0,008	1
Rasgef1b	0,354161	0,014033	0,081	0,099	1
Ccdc85c	0,354223	-0,0058	0,059	0,074	1
Inpp4a	0,354673	-0,01269	0,046	0,059	1
Nsf	0,35477	0,022626	0,251	0,289	1
Col2a1	0,354828	-0,00933	0,028	0,038	1
Casp2	0,354931	0,000823	0,117	0,139	1
Stox2	0,355089	0,048311	0,839	0,873	1
Ccdc3	0,355207	0,009779	0,015	0,008	1

Gm15892	0,355207	0,013376	0,015	0,008	1
Gm26514	0,355207	0,011579	0,015	0,008	1
Rab11fip5	0,3555	0,009897	0,04	0,03	1
5033406O(	0,355543	-0,00529	0,028	0,038	1
Ccng1	0,356086	0,004497	0,303	0,344	1
Map1s	0,356147	0,002128	0,292	0,325	1
Gm49396	0,356395	0,009779	0,015	0,008	1
Atg16l2	0,356466	-0,00754	0,042	0,055	1
4931428F(	0,356709	-0,00409	0,057	0,072	1
Rrs1	0,356795	0,003212	0,45	0,515	1
Arf5	0,356864	0,048151	0,905	0,895	1
Tmod3	0,35704	-0,00529	0,262	0,295	1
L2hgdh	0,357082	-0,00605	0,042	0,055	1
Ccdc88a	0,357182	0,059152	0,974	0,979	1
Ero1l	0,357623	0,077451	0,369	0,361	1
Trp53bp1	0,357675	0,04554	0,231	0,211	1
St5	0,357853	0,083901	0,228	0,215	1
Madd	0,357968	-0,00729	0,044	0,057	1
2810405F1	0,358038	-0,0058	0,04	0,053	1
Yeats2	0,358046	0,076373	0,257	0,249	1
Eif3b	0,358076	0,008015	0,549	0,618	1
Zfp607a	0,358135	-0,00187	0,044	0,057	1
Dbf4	0,358149	-0,00605	0,033	0,044	1
St6galnac4	0,358507	-0,00274	0,026	0,036	1
Seh1l	0,358774	0,003951	0,237	0,272	1
Bcat2	0,358902	-0,00627	0,136	0,158	1
Zbtb33	0,359011	-0,00667	0,2	0,228	1
Decr2	0,359012	0,04705	0,07	0,057	1
Mpz	0,359084	-0,11764	0,015	0,008	1
Fam110a	0,35998	0,004079	0,015	0,008	1
Paqr3	0,360014	-0,00085	0,048	0,061	1
Zfp940	0,360033	-0,00629	0,048	0,061	1
Smad5	0,360165	0,072596	0,376	0,378	1
Kif18a	0,360178	0,02294	0,05	0,038	1
Kif1b	0,36027	0,004458	1	0,998	1
Zfp968	0,360506	-0,00403	0,035	0,046	1
Stx2	0,360508	-0,0039	0,248	0,283	1
Irs1	0,360803	0,033915	0,088	0,074	1
Zbed3	0,361016	-0,00605	0,035	0,046	1
Slc38a6	0,361308	-0,02488	0,244	0,268	1
Sidt2	0,361622	0,072778	0,42	0,416	1
Taf1c	0,361895	0,019954	0,075	0,061	1
Pcdhb6	0,362199	-0,0058	0,029	0,04	1
Eapp	0,362247	0,001822	0,567	0,641	1
Ccne2	0,362343	-0,00709	0,006	0,011	1
Cd300lf	0,362343	-0,00709	0,006	0,011	1
Gm10373	0,362343	-0,00709	0,006	0,011	1
Srpr	0,362885	0,02922	0,516	0,612	1
Gm13610	0,363446	-0,005	0,006	0,011	1
Ankrd35	0,363446	-0,005	0,006	0,011	1
Dao	0,363446	-0,005	0,006	0,011	1
Gm43154	0,363446	-0,005	0,006	0,011	1

Gm44739	0,363446	-0,005	0,006	0,011	1
Rgs10	0,363446	-0,005	0,006	0,011	1
Gm49353	0,363446	-0,005	0,006	0,011	1
Gm27030	0,363446	-0,005	0,006	0,011	1
4732419C:	0,363446	-0,005	0,006	0,011	1
Gng2	0,363446	-0,005	0,006	0,011	1
Duxbl1	0,363446	-0,005	0,006	0,011	1
Gm45606	0,363446	-0,005	0,006	0,011	1
Hmga1b	0,363446	-0,005	0,006	0,011	1
Dnmt3aos	0,363446	-0,005	0,006	0,011	1
Gm26513	0,363446	-0,005	0,006	0,011	1
9330136K:	0,363446	-0,005	0,006	0,011	1
Tll2	0,363446	-0,005	0,006	0,011	1
Pola1	0,363553	0,018646	0,141	0,167	1
Pygm	0,363818	-0,00735	0,006	0,011	1
Fam20c	0,364044	0,001131	0,253	0,285	1
Wwc1	0,364187	-0,00527	0,006	0,011	1
Syt16	0,364187	-0,00527	0,006	0,011	1
Rapgef3os2	0,364187	-0,00527	0,006	0,011	1
Stac2	0,364556	0,000183	0,006	0,011	1
Gm13830	0,364799	0,016232	0,092	0,076	1
Ankrd61	0,365061	0,008138	0,024	0,034	1
Actr3b	0,365064	0,017198	0,042	0,032	1
Txnrd3	0,365156	0,033328	0,061	0,049	1
Slc6a20b	0,365293	0,002269	0,006	0,011	1
Mgmt	0,365293	-0,00136	0,006	0,011	1
Gm38250	0,365293	-0,00136	0,006	0,011	1
Bag2	0,365293	-0,00318	0,006	0,011	1
St8sia4	0,365293	-0,00318	0,006	0,011	1
Gchfr	0,365293	-0,00318	0,006	0,011	1
Cdyl2	0,365293	-0,00318	0,006	0,011	1
Eno1b	0,365293	-0,00318	0,006	0,011	1
Tfpi	0,365295	-0,00553	0,006	0,011	1
Golga2	0,365362	0,006293	0,387	0,432	1
Immt	0,365538	0,008475	0,761	0,825	1
Ryk	0,36554	0,010347	0,49	0,551	1
Sh2d6	0,366096	-0,0033	0,037	0,049	1
Arhgef11	0,366258	0,001909	0,169	0,196	1
Amd1	0,366593	0,05521	0,85	0,84	1
Ubxn11	0,366729	0,013685	0,028	0,019	1
Rpl27a	0,367013	0,013686	1	0,994	1
Casd1	0,367104	0,003417	0,24	0,272	1
Myh10	0,367146	-0,00136	0,006	0,011	1
Ttf2	0,367146	0,002269	0,006	0,011	1
Gm48678	0,367444	0,04705	0,035	0,025	1
Ugdh	0,367988	0,119253	0,224	0,213	1
Sft2d2	0,36801	0,018818	0,336	0,388	1
Amz1	0,368668	0,010126	0,028	0,019	1
Capzb	0,368943	0,060624	0,727	0,753	1
D230017M	0,369	0,005549	0,029	0,04	1
Wdr1	0,369086	0,021217	0,521	0,58	1
Fads6	0,369168	0,056145	0,661	0,669	1

Top3b	0,36998	0,01501	0,275	0,312	1
Klhdc3	0,369999	-0,00696	0,321	0,359	1
Tor1aip1	0,370184	0,005439	0,653	0,724	1
Ddx50	0,370203	0,076429	0,561	0,589	1
Fads1	0,370393	0,064517	0,235	0,222	1
Fbxl4	0,370559	0,000183	0,143	0,167	1
Cbarp	0,371038	0,014279	0,035	0,025	1
Psmd11	0,371093	0,035453	0,783	0,795	1
Ttyh1	0,371166	-0,07778	0,306	0,333	1
AA467197	0,371287	0,009552	0,028	0,019	1
Rabl2	0,371297	2,06E-05	0,099	0,118	1
Xiap	0,371378	0,077119	0,626	0,66	1
Ldlrad4	0,371575	0,073634	0,477	0,487	1
Scamp5	0,371768	0,069024	0,765	0,776	1
3110045C2	0,3721	0,006475	0,033	0,044	1
Slc7a14	0,3727	0,014863	0,042	0,032	1
Cercam	0,372769	0,04133	0,22	0,203	1
Zfp11	0,372795	0,001395	0,046	0,059	1
Epb41l1	0,372878	0,085432	0,497	0,506	1
Ppp2r5c	0,372886	-0,00891	0,782	0,829	1
Rock1	0,373462	-0,01779	0,831	0,865	1
Gan	0,373917	0,004407	0,273	0,31	1
Tmem131	0,374455	0,059991	0,323	0,31	1
Ttbk1	0,374601	-0,00937	0,253	0,281	1
Lyst	0,374606	0,01752	0,233	0,268	1
Ccdc88c	0,374885	0,001812	0,05	0,063	1
Loxl2	0,376035	0,0087	0,035	0,025	1
Ap2a2	0,37654	-0,00394	0,565	0,629	1
Tmem176a	0,376581	0,017067	0,119	0,101	1
Ly6g6f	0,376793	-0,02373	0,389	0,435	1
Gm2694	0,377003	-0,02671	0,094	0,11	1
Smurf1	0,377197	0,00444	0,171	0,198	1
Sbf1	0,377675	-0,01844	0,85	0,88	1
Cd59a	0,377729	-0,08506	0,505	0,449	1
Gng7	0,377966	0,006102	0,609	0,694	1
Tspyl5	0,378311	0,007746	0,057	0,044	1
Spats2l	0,379129	-0,01343	0,182	0,205	1
Zfp595	0,379288	-0,02241	0,094	0,11	1
Lrrc45	0,380444	-0,00154	0,174	0,198	1
Stim2	0,38105	0,004522	0,198	0,228	1
Aldh16a1	0,381075	-0,00696	0,127	0,148	1
Trim37	0,381161	-0,00914	0,139	0,16	1
Ppp1r12b	0,381382	0,018646	0,198	0,23	1
Rab33b	0,38144	0,008036	0,514	0,568	1
Rpl14	0,381732	0,00747	0,994	0,996	1
Klf3	0,381879	0,075835	0,589	0,608	1
A930003A1	0,38272	-0,00746	0,086	0,103	1
Ipo9	0,382832	0,052362	0,299	0,287	1
Rps6ka3	0,382857	0,039623	0,132	0,116	1
Map1b	0,383587	0,02889	0,87	0,808	1
Cenpo	0,383613	-0,00943	0,072	0,086	1
Ppp4c	0,383655	0,084634	0,429	0,428	1

Pcdhga12	0,383675	-0,01026	0,171	0,194	1
Rabep1	0,383765	0,024211	0,44	0,508	1
Chmp3	0,383774	0,014138	0,569	0,652	1
Cpsf2	0,383805	0,007769	0,207	0,238	1
Clp1	0,383984	0,009652	0,138	0,162	1
Mboat1	0,384774	0,013682	0,411	0,477	1
Snrpb	0,385277	0,043509	0,894	0,895	1
St3gal1	0,386089	0,033949	0,114	0,097	1
Tsen54	0,38616	-0,0126	0,07	0,084	1
Slc38a10	0,386317	0,05966	0,558	0,58	1
Med13l	0,386346	0,019077	0,406	0,447	1
Foxp4	0,38649	0,03699	0,112	0,097	1
Ttyh3	0,386818	-0,0056	0,134	0,154	1
Olfml2b	0,386995	0,018605	0,231	0,209	1
Pex10	0,387006	-0,0093	0,105	0,122	1
Vash2	0,387038	0,007025	0,006	0,002	1
Irs3	0,387038	0,007025	0,006	0,002	1
Zic3	0,387038	0,012465	0,006	0,002	1
Gm42851	0,387038	0,008842	0,006	0,002	1
Adra1a	0,387038	0,008842	0,006	0,002	1
Pomgnt2	0,387042	-0,0195	0,09	0,105	1
Sec16a	0,387126	0,020052	0,242	0,276	1
U2af2	0,387373	0,092035	0,591	0,635	1
Dpagt1	0,387555	0,013783	0,117	0,139	1
Syt6	0,387578	0,013972	0,006	0,002	1
Cited1	0,387578	0,008842	0,006	0,002	1
Gm26762	0,387578	0,007025	0,006	0,002	1
Gm26583	0,387578	0,007025	0,006	0,002	1
Gm5475	0,387578	0,007025	0,006	0,002	1
Cldn8	0,387578	0,007025	0,006	0,002	1
Gm38336	0,387578	0,005205	0,006	0,002	1
Sardh	0,387578	0,005205	0,006	0,002	1
4930502E1	0,387578	0,005205	0,006	0,002	1
Fgf13	0,387578	0,005205	0,006	0,002	1
Ptgfrn	0,387578	0,005205	0,006	0,002	1
Lpar3	0,387578	0,005205	0,006	0,002	1
Slc8a2	0,387578	0,005205	0,006	0,002	1
AU020206	0,387578	0,005205	0,006	0,002	1
Tnfrsf23	0,387578	0,005205	0,006	0,002	1
Phxr2	0,387578	0,005205	0,006	0,002	1
Gm39228	0,387578	0,005205	0,006	0,002	1
Gm26521	0,387578	0,005205	0,006	0,002	1
Gm47232	0,387578	0,005205	0,006	0,002	1
Lrrc2	0,387578	0,005205	0,006	0,002	1
Gm12264	0,387578	0,005205	0,006	0,002	1
Gm43951	0,387578	0,005205	0,006	0,002	1
Serpinf2	0,387578	0,005205	0,006	0,002	1
Wfdc17	0,387578	0,005205	0,006	0,002	1
Gpx2	0,387578	0,005205	0,006	0,002	1
Fam71d	0,387578	0,005205	0,006	0,002	1
Fam69c	0,387578	0,005205	0,006	0,002	1
Chmp2a	0,38801	-0,01171	0,963	0,956	1

Mybph	0,388119	0,003382	0,006	0,002	1
Grin1	0,388119	0,003382	0,006	0,002	1
Olfr1122	0,388119	0,003382	0,006	0,002	1
Olfr1260	0,388119	0,003382	0,006	0,002	1
Grem1	0,388119	0,003382	0,006	0,002	1
Gm17096	0,388119	0,003382	0,006	0,002	1
Xlr3a	0,388119	0,003382	0,006	0,002	1
Tmem35a	0,388119	0,003382	0,006	0,002	1
Gm15298	0,388119	0,003382	0,006	0,002	1
Dcst2	0,388119	0,003382	0,006	0,002	1
Gm42681	0,388119	0,003382	0,006	0,002	1
I830077J0	0,388119	0,003382	0,006	0,002	1
Dennd2d	0,388119	0,003382	0,006	0,002	1
Aqp7	0,388119	0,003382	0,006	0,002	1
Mycl	0,388119	0,003382	0,006	0,002	1
Angptl7	0,388119	0,003382	0,006	0,002	1
Cort	0,388119	0,003382	0,006	0,002	1
Gbp6	0,388119	0,003382	0,006	0,002	1
Rph3a	0,388119	0,003382	0,006	0,002	1
A430078I0	0,388119	0,003382	0,006	0,002	1
Vmn1r76	0,388119	0,003382	0,006	0,002	1
Gm9844	0,388119	0,003382	0,006	0,002	1
Nphs1	0,388119	0,003382	0,006	0,002	1
Igflr1	0,388119	0,003382	0,006	0,002	1
Olfr571	0,388119	0,003382	0,006	0,002	1
1810010D(	0,388119	0,003382	0,006	0,002	1
B230208H	0,388119	0,003382	0,006	0,002	1
Ppil6	0,388119	0,003382	0,006	0,002	1
Gm10778	0,388119	0,003382	0,006	0,002	1
Gm31182	0,388119	0,003382	0,006	0,002	1
Itga7	0,388119	0,003382	0,006	0,002	1
Isyna1	0,388119	0,003382	0,006	0,002	1
Ankle1	0,388119	0,003382	0,006	0,002	1
D030051J2	0,388119	0,003382	0,006	0,002	1
Mmrn2	0,388119	0,003382	0,006	0,002	1
Olfr905	0,388119	0,003382	0,006	0,002	1
Fxyd2	0,388119	0,003382	0,006	0,002	1
Plet1os	0,388119	0,003382	0,006	0,002	1
Gm10658	0,388119	0,003382	0,006	0,002	1
9630041A(	0,388119	0,003382	0,006	0,002	1
Gm33054	0,388119	0,003382	0,006	0,002	1
A730085K(	0,388119	0,003382	0,006	0,002	1
Nxn	0,388119	0,003382	0,006	0,002	1
Eme1	0,388119	0,003382	0,006	0,002	1
Gm9796	0,388119	0,003382	0,006	0,002	1
AK157302	0,388119	0,003382	0,006	0,002	1
Gm38604	0,388119	0,003382	0,006	0,002	1
4931403G	0,388119	0,003382	0,006	0,002	1
Gdnf	0,388119	0,003382	0,006	0,002	1
Car15	0,388119	0,003382	0,006	0,002	1
AC126280.	0,388119	0,003382	0,006	0,002	1
Gm9968	0,388119	0,003382	0,006	0,002	1

Angptl4	0,388119	0,003382	0,006	0,002	1
Colec12	0,388119	0,003382	0,006	0,002	1
Chsy3	0,388119	0,003382	0,006	0,002	1
Gm16541	0,388119	0,003382	0,006	0,002	1
Col17a1	0,388119	0,003382	0,006	0,002	1
Slc25a13	0,388251	0,076831	0,42	0,418	1
Ppp2r2c	0,388421	0,069576	0,901	0,895	1
Pcgf2	0,388662	0,004922	0,006	0,002	1
Slc25a2	0,388662	0,004922	0,006	0,002	1
Uba52	0,388768	0,071389	0,765	0,764	1
Bcl9	0,388915	0,035765	0,402	0,47	1
Ythdf3	0,388917	-0,00445	0,394	0,435	1
Pdpk1	0,388952	0,028606	0,385	0,445	1
Dhx37	0,389149	0,005173	0,149	0,173	1
Nusap1	0,389204	0,003102	0,006	0,002	1
Reln	0,389204	0,006169	0,006	0,002	1
Ccdc141	0,389489	-0,00735	0,007	0,013	1
Cetn4	0,389489	-0,00735	0,007	0,013	1
Mpeg1	0,389489	-0,0115	0,007	0,013	1
Rasgrp3	0,389688	0,069122	0,807	0,789	1
A930003O	0,389747	0,001279	0,006	0,002	1
Tex15	0,389747	0,001279	0,006	0,002	1
Slc5a10	0,389747	0,001279	0,006	0,002	1
AC159200.	0,389747	0,001279	0,006	0,002	1
Tgfbr2	0,389747	-0,00291	0,006	0,002	1
Adam22	0,389887	0,021352	0,433	0,492	1
Rab1b	0,39011	0,060548	0,648	0,681	1
Samd14	0,390394	0,012491	0,022	0,015	1
Sh2b1	0,390607	0,046277	0,266	0,249	1
Plxnb3	0,39062	0,048163	0,694	0,7	1
D330050G	0,390866	-0,00527	0,007	0,013	1
Cenpi	0,390866	-0,00527	0,007	0,013	1
1700015F1	0,390866	-0,00527	0,007	0,013	1
Rarres2	0,390866	-0,00527	0,007	0,013	1
1700069B(	0,390866	-0,00527	0,007	0,013	1
Gm20163	0,390866	-0,00527	0,007	0,013	1
Pelo	0,390866	-0,00527	0,007	0,013	1
Gm48796	0,391215	-0,00968	0,007	0,013	1
Nsa2	0,391401	0,002738	0,699	0,73	1
Gm17146	0,391601	0,020458	0,029	0,021	1
Tcf7l1	0,39191	0,011616	0,022	0,015	1
Prr7	0,392152	0,012491	0,022	0,015	1
Tet2	0,392439	0,016048	0,29	0,335	1
Kctd9	0,392782	0,027185	0,121	0,105	1
Rhoq	0,392922	-0,00029	0,25	0,285	1
Fcer1g	0,392942	0,001993	0,007	0,013	1
AC154782.	0,392942	0,001993	0,007	0,013	1
BC030343	0,392942	-0,00163	0,007	0,013	1
Nespas	0,392942	-0,00345	0,007	0,013	1
Fam196b	0,392942	-0,00345	0,007	0,013	1
Gm30198	0,392942	-0,00345	0,007	0,013	1
Mkx	0,392942	-0,00345	0,007	0,013	1

Otud1	0,393566	-0,01453	0,097	0,114	1
Cxcr4	0,393672	0,008342	0,022	0,015	1
Slc17a9	0,39376	0,016931	0,029	0,021	1
Grtp1	0,393914	0,007119	0,022	0,015	1
Gt(ROSA)26	0,393925	0,004209	0,022	0,015	1
Gm29237	0,393984	-8,8E-05	0,007	0,013	1
Acp6	0,394004	0,004128	0,128	0,15	1
Sipa1l1	0,394554	0,019602	0,079	0,065	1
Nos3	0,395024	-0,00163	0,007	0,013	1
Gm26852	0,395024	-0,00163	0,007	0,013	1
Mettl7a3	0,395024	0,000183	0,007	0,013	1
Mief2	0,395314	-0,01242	0,099	0,116	1
Sppl3	0,395438	0,088458	0,415	0,428	1
5033417F2	0,395447	0,013116	0,044	0,034	1
Zfp622	0,395572	0,017549	0,251	0,287	1
Crat	0,395594	-0,00086	0,25	0,281	1
Zbtb14	0,396522	-0,00238	0,116	0,135	1
Ccdc39	0,396523	-0,00106	0,079	0,095	1
Rpusd1	0,396737	-0,02075	0,09	0,105	1
Lmf2	0,396775	0,005562	0,281	0,316	1
Brd9	0,396803	0,048785	0,708	0,711	1
Hook1	0,397112	0,000183	0,007	0,013	1
Mier3	0,397365	0,045257	0,24	0,226	1
AC163032.	0,397523	0,011032	0,138	0,16	1
Ispd	0,397536	-0,00246	0,182	0,207	1
Eif2ak2	0,397582	0,002717	0,044	0,034	1
Cmah	0,397801	0,020615	0,053	0,042	1
Anapc7	0,397993	-0,00884	0,305	0,335	1
Vopp1	0,3981	0,013391	0,029	0,021	1
2700012I2	0,3981	0,013391	0,029	0,021	1
Fer	0,398187	0,003185	0,2	0,228	1
Ube2k	0,398374	0,005681	0,648	0,711	1
Morc2a	0,398414	0,043335	0,183	0,167	1
Arl8b	0,398436	0,040741	0,787	0,797	1
Scaf1	0,39848	0,021738	0,519	0,595	1
Nbn	0,398573	0,000568	0,147	0,169	1
Nus1	0,398844	0,035236	0,4	0,475	1
Bmp1	0,398902	0,050255	0,27	0,253	1
Abcb1b	0,398981	0,016632	0,029	0,021	1
Ank3	0,399118	-0,00365	0,987	0,987	1
Trir	0,39922	-0,01111	0,732	0,787	1
Pip4k2c	0,399711	0,047601	0,429	0,418	1
Mtss1l	0,400897	0,049785	0,18	0,165	1
Nphp1	0,400988	-0,00124	0,073	0,089	1
Ptar1	0,401019	-0,00855	0,127	0,146	1
Prkag2	0,401078	0,012228	0,037	0,027	1
Sh3pxd2b	0,4011	0,009614	0,044	0,034	1
Pcyox1	0,401832	0,063388	0,464	0,466	1
G3bp2	0,402203	0,054396	0,794	0,812	1
Phc2	0,402236	-0,00754	0,837	0,848	1
Ankrd13b	0,402308	0,054405	0,35	0,34	1
Usb1	0,402857	-0,00726	0,066	0,08	1

Hmox1	0,403067	0,034406	0,134	0,118	1
9130019O:	0,403148	0,021732	0,053	0,042	1
Exoc4	0,403213	0,020934	0,481	0,555	1
Ctcf	0,403282	0,058491	0,717	0,724	1
Pcmtd1	0,403302	0,038382	0,813	0,816	1
Plbd2	0,403398	0,018836	0,402	0,462	1
Tiam1	0,404396	-0,02095	0,055	0,068	1
Hsf2	0,404481	0,012459	0,215	0,243	1
Sord	0,404493	0,014572	0,125	0,108	1
Rgs20	0,40456	0,025547	0,017	0,011	1
Fam109a	0,404609	-0,01531	0,092	0,108	1
March6	0,404795	0,067368	0,402	0,397	1
Sept10	0,404979	-0,00428	0,253	0,283	1
Pigb	0,405156	-0,0032	0,083	0,099	1
Atp6v0c	0,405307	-0,01072	0,059	0,072	1
Olig2	0,405315	0,007053	0,793	0,728	1
Antxr1	0,405521	0,0015	0,086	0,103	1
Tgfbr1	0,405555	0,061399	0,224	0,211	1
Enpp4	0,40584	0,039359	0,943	0,918	1
Eif4g1	0,405944	0,054046	0,739	0,77	1
Ckap5	0,405945	0,001202	0,787	0,812	1
Cbfa2t2	0,405965	0,006952	0,239	0,27	1
Sytl5	0,405994	0,007774	0,029	0,021	1
Zc3h11a	0,406221	0,023135	0,09	0,076	1
Lrrc49	0,406254	0,041715	0,281	0,264	1
Tbxas1	0,406362	-0,01587	0,009	0,015	1
Zfp292	0,406438	0,060991	0,521	0,519	1
Lrwd1	0,406874	-0,0216	0,136	0,154	1
Stub1	0,406936	-0,00132	0,712	0,764	1
Cryz	0,407066	0,024087	0,112	0,097	1
Atl2	0,407479	0,033322	0,161	0,146	1
Gspt1	0,407877	-0,01694	0,732	0,755	1
Ift172	0,407926	-0,008	0,158	0,179	1
Rad52	0,407945	-0,00652	0,097	0,114	1
Rsad1	0,408116	0,031857	0,084	0,072	1
Cdc37	0,408332	0,039196	0,848	0,857	1
Crip1	0,408351	-0,06244	0,139	0,158	1
Gnl2	0,408605	0,025106	0,356	0,409	1
Mvb12b	0,409308	-0,01351	0,657	0,7	1
Kif24	0,409593	-0,00553	0,009	0,015	1
Lrrd1	0,409593	-0,00553	0,009	0,015	1
Zfp459	0,409593	-0,00553	0,009	0,015	1
Zfp97	0,410247	-0,00579	0,009	0,015	1
Pigo	0,410276	-0,0046	0,068	0,082	1
Trip10	0,410548	0,01487	0,017	0,011	1
Sepsecs	0,410732	0,005892	0,145	0,167	1
Rxra	0,411242	0,012033	0,079	0,065	1
Myo19	0,411454	0,012785	0,017	0,011	1
Shcbp1l	0,411558	-0,00957	0,092	0,108	1
Armc2	0,41187	-8,8E-05	0,009	0,015	1
Vmn2r97	0,41187	-0,0019	0,009	0,015	1
Gm28836	0,41187	-0,00371	0,009	0,015	1

Gm20632	0,41187	-0,00371	0,009	0,015	1
Gm13166	0,41187	-0,00371	0,009	0,015	1
Gm11696	0,41187	-0,00371	0,009	0,015	1
Gm26836	0,412052	0,009489	0,017	0,011	1
1700056N	0,412052	0,01487	0,017	0,011	1
Entpd7	0,412417	0,028073	0,092	0,078	1
Ciz1	0,412512	0,020214	0,222	0,253	1
Birc3	0,412525	-0,00397	0,009	0,015	1
Bicd1	0,412542	0,035807	0,655	0,66	1
Copb1	0,412857	0,014876	0,545	0,633	1
Extl3	0,412906	-0,00549	0,295	0,321	1
Sox6	0,413178	-0,00424	0,009	0,015	1
Fam221b	0,41356	0,007689	0,017	0,011	1
Usp40	0,413753	0,02323	0,317	0,361	1
BC037039	0,414153	-0,0019	0,009	0,015	1
Dnmbp	0,414153	-8,8E-05	0,009	0,015	1
Acvr2a	0,414419	-0,00605	0,099	0,116	1
Mpzl3	0,414472	0,018137	0,017	0,011	1
Slc4a11	0,414774	0,007404	0,017	0,011	1
Pcbp4	0,414927	0,030725	0,994	0,989	1
Rpl4	0,415035	0,047133	0,983	0,983	1
Txk	0,41507	0,005886	0,017	0,011	1
Zfp423	0,41507	0,005886	0,017	0,011	1
Appbp2os	0,41507	0,005886	0,017	0,011	1
Trim32	0,415313	0,001817	0,286	0,321	1
AC162302.	0,415353	0,011688	0,053	0,042	1
Usp13	0,415571	0,019245	0,039	0,03	1
Ptpn9	0,415581	-0,01109	0,119	0,137	1
Slc35e1	0,415761	-0,00143	0,202	0,228	1
Atf5	0,41599	0,020877	0,24	0,276	1
Sema4d	0,41625	-0,0244	0,989	0,966	1
Rptor	0,416507	0,048042	0,242	0,228	1
Tbrg4	0,416732	-0,00274	0,152	0,173	1
Sacs	0,417287	0,009377	0,158	0,181	1
Gk5	0,417388	0,000425	0,103	0,12	1
Rhot2	0,418017	0,048132	0,226	0,213	1
Zfp275	0,418226	0,007475	0,22	0,249	1
Rnf185	0,418302	-0,01283	0,141	0,16	1
Grcc10	0,418353	0,01885	0,473	0,542	1
Bax	0,41838	0,005292	0,697	0,755	1
Ppfibp1	0,418632	0,02884	0,073	0,061	1
Adgrg2	0,418702	0,031322	0,055	0,044	1
Pcid2	0,418843	0,007392	0,246	0,278	1
Cald1	0,418954	-0,00212	0,512	0,568	1
2210406O	0,419462	0,020688	0,039	0,03	1
Nat6	0,419988	-0,00019	0,171	0,194	1
Ctdsp1	0,420073	0,058077	0,422	0,424	1
Serpinc1	0,420228	0,023662	0,031	0,023	1
Rbm4b	0,420624	0,090227	0,448	0,458	1
Slc7a6os	0,420642	0,035257	0,2	0,184	1
Gm26870	0,420681	0,475268	0,011	0,006	1
Xntrpc	0,420852	-0,00786	0,011	0,017	1

Pcdhga3	0,420852	-0,00786	0,011	0,017	1
Rpl10-ps3	0,420912	0,002537	0,228	0,253	1
Ppm1b	0,421069	0,017979	0,488	0,568	1
Acp2	0,421693	0,008842	0,288	0,319	1
Frs2	0,421739	-0,00491	0,286	0,316	1
Vps13d	0,422502	0,056273	0,268	0,259	1
Zfp335os	0,422684	-0,00579	0,011	0,017	1
Gm15283	0,422684	-0,00579	0,011	0,017	1
Gm26789	0,422709	0,022837	0,024	0,017	1
A230083N:	0,422962	0,01007	0,011	0,006	1
Gm35339	0,422962	0,01007	0,011	0,006	1
Gm35315	0,422995	-0,01018	0,011	0,017	1
Abl1	0,423037	0,053325	0,622	0,635	1
P3h1	0,423123	-0,00827	0,051	0,063	1
Lgals1	0,423214	0,024285	0,024	0,017	1
Bclaf3	0,423336	0,08576	0,294	0,289	1
Fgd4	0,423353	0,058709	0,226	0,209	1
Ccdc106	0,424105	0,008263	0,011	0,006	1
Gm20075	0,424105	0,008263	0,011	0,006	1
Gdf15	0,424105	0,013673	0,011	0,006	1
Gm26538	0,424105	0,013673	0,011	0,006	1
Magee2	0,424105	0,01007	0,011	0,006	1
Gabrd	0,424105	0,01007	0,011	0,006	1
Lmln	0,424231	-0,00022	0,095	0,112	1
Helq	0,424288	-0,01341	0,088	0,103	1
Sox9	0,424489	0,01517	0,011	0,006	1
Mfsd11	0,424689	0,027694	0,347	0,401	1
Elf1	0,42489	0,027588	0,29	0,331	1
Clec11a	0,42514	-0,00216	0,011	0,017	1
Gm14167	0,42514	-0,00397	0,011	0,017	1
Pdpr	0,425168	0,027909	0,125	0,11	1
Hist2h2ac	0,42525	0,008263	0,011	0,006	1
Capg	0,42525	0,008263	0,011	0,006	1
Gm21917	0,42525	0,008263	0,011	0,006	1
Hpgds	0,42525	0,006453	0,011	0,006	1
Plaur	0,42525	0,006453	0,011	0,006	1
4732496C(	0,42525	0,006453	0,011	0,006	1
Emid1	0,42525	0,006453	0,011	0,006	1
Pnma1	0,42525	0,006453	0,011	0,006	1
Gm16279	0,42525	0,006453	0,011	0,006	1
Helz	0,425562	0,040262	0,128	0,114	1
Sufu	0,425563	0,029667	0,116	0,101	1
St3gal2	0,425678	0,040646	0,169	0,154	1
Cacnb3	0,425899	0,023352	0,031	0,023	1
Pcna	0,42598	0,060045	0,624	0,641	1
Pls1	0,426377	0,0009	0,932	0,928	1
Gm12530	0,426382	-0,00876	0,05	0,061	1
Helz2	0,426396	0,00464	0,011	0,006	1
H6pd	0,426396	0,00464	0,011	0,006	1
Sez6	0,426396	0,00464	0,011	0,006	1
BC055324	0,4264	0,006169	0,011	0,006	1
Mphosph9	0,426721	0,01856	0,154	0,179	1

Pdcd11	0,426754	0,017312	0,301	0,342	1
AL732506.	0,427165	0,007975	0,011	0,006	1
Tctn3	0,427301	-0,00679	0,051	0,063	1
Hist1h4c	0,427605	0,005043	0,011	0,017	1
1700028K(	0,427605	-0,00036	0,011	0,017	1
Pdzrn4	0,427605	-0,00036	0,011	0,017	1
Ahctf1	0,427939	0,06096	0,347	0,331	1
Dpy19l1	0,428007	0,068611	0,998	0,994	1
Snhg10	0,428281	0,011405	0,055	0,044	1
Fmo5	0,428395	0,0047	0,053	0,065	1
Hspa4	0,428433	0,001312	0,673	0,738	1
Cul5	0,428552	0,068564	0,628	0,648	1
Haus8	0,42864	0,024674	0,224	0,255	1
Heatr5a	0,428676	0,021624	0,259	0,293	1
Bcas1os2	0,428746	0,012198	0,024	0,017	1
Thada	0,428946	0,002663	0,112	0,131	1
Rbm14	0,428959	0,005196	0,2	0,228	1
Plekhb2	0,428961	0,058469	0,365	0,363	1
Chkb	0,429033	0,04714	0,303	0,291	1
Lrsam1	0,429436	0,000736	0,13	0,15	1
Slc35f5	0,42949	0,002366	0,176	0,2	1
Etv6	0,429759	-0,00531	0,053	0,065	1
Zfp879	0,429887	-0,00812	0,013	0,019	1
Sgsm1	0,429887	-0,01224	0,013	0,019	1
9330175M	0,430077	-0,00036	0,011	0,017	1
Olfr775	0,430077	-0,00036	0,011	0,017	1
Card6	0,430077	0,001446	0,011	0,017	1
Zfp106	0,43025	0,010956	0,44	0,496	1
Optn	0,430293	0,022456	0,347	0,399	1
Hadha	0,430624	0,013662	0,8	0,768	1
Asap3	0,430629	0,01018	0,039	0,03	1
Zfp382	0,430677	-0,00187	0,053	0,065	1
Morc4	0,43084	0,014853	0,073	0,061	1
Nav2	0,431067	0,03465	0,229	0,264	1
Carns1	0,431396	-0,02983	0,695	0,711	1
Zfp667	0,431705	-0,00263	0,055	0,068	1
Fbxw15	0,431913	-0,00605	0,013	0,019	1
Camk2b	0,431913	-0,00605	0,013	0,019	1
Mdk	0,43221	-0,01043	0,013	0,019	1
Ctnnd1	0,432362	0,032955	0,407	0,475	1
Dip2c	0,432522	0,109918	0,394	0,411	1
Gm10643	0,432557	0,003246	0,011	0,017	1
Egfem1	0,432797	0,008627	0,024	0,017	1
Dna2	0,432797	0,008627	0,024	0,017	1
Vgll4	0,433054	0,05805	0,738	0,726	1
Mmachc	0,43313	-0,0088	0,04	0,051	1
Rrm1	0,433437	-0,00297	0,264	0,291	1
Olfr1369-p	0,433717	-0,04715	0,015	0,021	1
Spred3	0,433823	0,011907	0,024	0,017	1
Rusc1	0,434077	0,008342	0,024	0,017	1
Pptc7	0,43409	0,034432	0,215	0,196	1
Prpf40b	0,434375	0,01721	0,198	0,226	1

Cat	0,434648	0,001997	0,565	0,627	1
Bfsp1	0,434655	-0,01453	0,039	0,049	1
Zfp429	0,434683	0,013098	0,031	0,023	1
Fbxl19	0,435081	-0,00375	0,123	0,141	1
Nsdhl	0,435093	0,034401	0,264	0,3	1
Dnmt3b	0,435123	-0,0045	0,013	0,019	1
Lsm11	0,435198	0,018596	0,057	0,046	1
Atxn7l2	0,435216	-0,00258	0,05	0,061	1
Rpl8	0,435273	0,023056	0,994	0,992	1
Clmn	0,435395	-0,03613	0,921	0,865	1
Pikfyve	0,435506	0,00966	0,383	0,439	1
Gm15445	0,435546	0,015728	0,048	0,038	1
Pnp	0,435686	0,022617	0,213	0,241	1
Neto2	0,435859	0,008342	0,024	0,017	1
Ms4a6c	0,436124	0,005994	0,024	0,017	1
Gnl3	0,436227	0,104074	0,53	0,553	1
Col1a2	0,436417	0,001833	0,095	0,112	1
Rab26os	0,436688	0,018302	0,057	0,046	1
Tle4	0,43676	0,086228	0,391	0,401	1
Ninj1	0,436927	-0,03732	0,099	0,114	1
Nup107	0,436965	0,009486	0,105	0,122	1
Slc25a37	0,437035	0,020764	0,075	0,063	1
Sgsm3	0,437115	-0,00214	0,119	0,137	1
Gm5464	0,437164	0,004764	0,013	0,019	1
9130604C2	0,437164	0,001173	0,013	0,019	1
Map3k7	0,437438	0,031538	0,338	0,392	1
Grik4	0,437546	-0,00108	0,132	0,152	1
Zscan18	0,437765	0,002072	0,051	0,063	1
Lims1	0,438041	0,077988	0,606	0,652	1
Tmem35b	0,438088	-0,01086	0,033	0,042	1
Pnpo	0,438104	-0,01068	0,017	0,023	1
Tmem161b	0,438299	-0,00428	0,251	0,276	1
Ccdc157	0,438435	0,030626	0,086	0,074	1
AL591952.	0,438488	-0,0111	0,033	0,042	1
Fxr2	0,438492	0,066057	0,642	0,671	1
Ghr	0,438688	-0,00044	0,024	0,017	1
Mxd1	0,438753	0,040895	0,25	0,289	1
Naa40	0,438771	0,011685	0,127	0,148	1
Gm2885	0,438948	-0,00656	0,015	0,021	1
Anks6	0,438999	0,017716	0,057	0,046	1
Cpt1a	0,439115	-0,0083	0,039	0,049	1
Ern1	0,439629	-0,0106	0,079	0,093	1
Rnft2	0,439676	-0,01601	0,07	0,082	1
Bnip3l	0,439699	0,045863	1	1	1
Gm45620	0,439802	-0,00063	0,013	0,019	1
Sgce	0,440044	0,052267	0,609	0,713	1
Ccpg1	0,440082	0,028413	0,288	0,329	1
Spon1	0,440101	0,005994	0,013	0,019	1
Dctn4	0,440203	0,03193	0,956	0,922	1
Arfgef1	0,440212	0,072754	0,576	0,616	1
Mitf	0,440543	0,018945	0,259	0,289	1
Ttc9	0,440685	-0,00482	0,196	0,219	1

Ppm1l	0,441118	0,071387	0,336	0,333	1
Stau2	0,441157	-0,00088	0,095	0,112	1
Chsy1	0,441161	-0,0027	0,015	0,021	1
Gm28221	0,441161	-0,0027	0,015	0,021	1
Ube2o	0,441197	0,055559	0,316	0,306	1
5430416N(	0,441777	-0,01226	0,11	0,127	1
Mysm1	0,442	0,083565	0,574	0,608	1
Anxa6	0,442356	0,013994	0,048	0,038	1
Gm20498	0,442503	-0,00605	0,039	0,049	1
Impad1	0,443328	-0,00483	0,699	0,772	1
Kcna1	0,443738	0,056696	0,923	0,88	1
Gm20939	0,443943	-0,0009	0,015	0,021	1
Bicdl1	0,443982	-0,00883	0,031	0,04	1
Agpat1	0,444285	0,010037	0,31	0,35	1
Akap8l	0,444396	0,013157	0,523	0,578	1
Gm17435	0,444725	0,029631	0,059	0,049	1
Tmem39b	0,444825	-0,00366	0,084	0,099	1
Sdk2	0,444858	-0,02033	0,07	0,082	1
Tmem51	0,445075	0,016292	0,057	0,046	1
A130010J1	0,445143	-0,01083	0,079	0,093	1
Rab29	0,445166	-0,01055	0,037	0,046	1
2900026A(	0,445298	-0,00938	0,02	0,027	1
Samd12	0,445824	-0,00731	0,033	0,042	1
Ripor1	0,445907	0,015701	0,075	0,063	1
Shc1	0,445966	0,028215	0,099	0,086	1
Gnptab	0,4461	-0,00271	0,587	0,648	1
Rgl3	0,446168	-0,00883	0,029	0,038	1
Zbtb21	0,446435	0,072873	0,217	0,207	1
Bcl6	0,446498	0,053088	0,211	0,198	1
Gm10687	0,446511	-0,00846	0,141	0,16	1
Slc46a3	0,446734	-0,0009	0,015	0,021	1
Cnot7	0,446783	0,030193	0,369	0,43	1
Gm31152	0,446814	-0,00143	0,017	0,023	1
Frg2f1	0,447359	-0,00681	0,031	0,04	1
Strip1	0,447404	0,0535	0,251	0,241	1
Rps6kc1	0,447413	-0,00103	0,163	0,184	1
Slc9a6	0,447893	-0,01998	0,371	0,392	1
Proscos	0,448261	-0,00733	0,022	0,03	1
Trim13	0,448616	0,107056	0,492	0,544	1
Khdrbs2	0,44874	-0,00758	0,022	0,03	1
Gm5547	0,448991	-0,00883	0,022	0,03	1
1700088E(	0,449047	0,000873	0,05	0,061	1
Gm28041	0,449208	-0,00322	0,017	0,023	1
4933415A(	0,449535	0,006273	0,015	0,021	1
Ube3c	0,449647	0,026506	0,343	0,388	1
Gm12525	0,449915	-0,00378	0,033	0,042	1
Suclg2	0,450392	0,016902	0,04	0,032	1
Fut11	0,450765	0,006463	0,152	0,173	1
Vps54	0,451043	0,000327	0,204	0,228	1
Nlgn3	0,451064	-0,00706	0,024	0,032	1
Dok1	0,451604	0,004209	0,017	0,023	1
Txndc16	0,45166	0,061612	0,864	0,903	1

Mycn	0,452009	-0,00052	0,035	0,044	1
Ubxn1	0,452054	-0,00858	0,961	0,97	1
Agrn	0,45233	0,05053	0,228	0,213	1
Tpp1	0,452593	0,051477	0,657	0,686	1
Qtrt1	0,452684	-0,00692	0,16	0,179	1
Eml3	0,452767	0,04217	0,207	0,192	1
Lcorl	0,452932	0,074286	0,626	0,643	1
Smarcc1	0,453168	0,028627	0,486	0,553	1
Vamp5	0,453853	0,089479	0,464	0,481	1
Neat1	0,453922	-0,02637	0,996	0,992	1
Gm45184	0,453967	-0,00274	0,028	0,036	1
Aldh1l2	0,454226	-0,01366	0,394	0,424	1
Zbtb44	0,454301	0,063567	0,36	0,357	1
Atad3a	0,454325	0,018605	0,213	0,241	1
Edc4	0,454413	0,045851	0,224	0,213	1
Fbxw9	0,454498	0,004225	0,161	0,184	1
Hk1	0,454716	0,075329	0,29	0,287	1
Cbx3	0,454875	-0,01618	0,936	0,93	1
Sec31a	0,454887	0,065406	0,446	0,468	1
Scaf8	0,455107	0,023589	0,321	0,363	1
Prep	0,455293	-0,00436	0,077	0,091	1
Siae	0,455427	0,030377	0,167	0,152	1
Gm16099	0,455588	-0,00426	0,024	0,032	1
Cdc42se2	0,455752	0,047946	0,767	0,772	1
Ddi2	0,456565	0,077563	0,29	0,285	1
6330403L(	0,457252	0,034471	0,112	0,099	1
Tmem8	0,457258	0,002985	0,037	0,046	1
Aldh1l1	0,457339	0,014863	0,04	0,032	1
Meis2	0,457752	-0,01813	0,22	0,245	1
Pole	0,457755	0,001608	0,02	0,027	1
Rsph10b	0,457835	0,00097	0,037	0,046	1
Zfp334	0,457884	0,008203	0,114	0,131	1
Gm26660	0,458251	0,008318	0,088	0,103	1
Pogk	0,458259	0,059059	0,661	0,69	1
Ctdspl	0,45835	0,074506	0,417	0,428	1
Zmym1	0,458392	-0,00667	0,198	0,219	1
Asb1	0,458501	-0,00544	0,233	0,255	1
Taf4	0,458713	0,047947	0,149	0,137	1
Egf	0,458996	0,011367	0,02	0,027	1
Gm26808	0,458997	-0,00097	0,026	0,034	1
Gm29562	0,45941	0,016356	0,018	0,013	1
Yrdc	0,459574	0,0015	0,251	0,278	1
Cop1	0,459934	0,065949	0,521	0,563	1
Ints6l	0,459973	0,019597	0,382	0,435	1
Slit2	0,460115	0,013987	0,033	0,025	1
Tmem86a	0,460214	-0,00123	0,029	0,038	1
Ttpal	0,460475	0,043052	0,145	0,133	1
Cavin4	0,460543	0,008842	0,086	0,101	1
Hp1bp3	0,46081	0,044823	0,868	0,861	1
Ttc17	0,460869	-0,01128	0,2	0,222	1
Cars2	0,460987	-0,01258	0,16	0,179	1
Hoxd3	0,461214	0,010994	0,018	0,013	1

Tmem53	0,461214	0,012785	0,018	0,013	1
T2	0,461214	0,016356	0,018	0,013	1
Gramd1a	0,461304	0,020824	0,228	0,257	1
Ttc13	0,461361	0,053419	0,172	0,162	1
Asl	0,461671	0,023411	0,121	0,108	1
Tstd2	0,461683	0,01752	0,257	0,289	1
Mbtps1	0,462186	0,006642	0,448	0,506	1
Mbnl2	0,462322	0,02449	1	0,998	1
Fam84b	0,462424	0,010704	0,018	0,013	1
Gm37305	0,462447	-0,0007	0,022	0,03	1
Pgm1	0,46257	0,041075	0,062	0,053	1
Akap8	0,462586	0,068231	0,407	0,42	1
Gnptg	0,462637	0,005547	0,572	0,627	1
Stt3b	0,462765	0,01058	0,367	0,411	1
Fau	0,463391	0,004474	0,998	0,996	1
Itsn1	0,463674	-0,00331	0,121	0,137	1
Ddx28	0,463728	-0,01384	0,064	0,076	1
Snap29	0,463743	0,05108	0,29	0,335	1
Kcnip3	0,46387	0,012517	0,033	0,025	1
Xkr8	0,463917	0,021352	0,07	0,059	1
Rab1a	0,463978	0,011432	0,574	0,641	1
Zkscan2	0,463979	0,000537	0,029	0,038	1
Hr	0,463987	0,050402	0,255	0,245	1
Eml5	0,464099	0,011142	0,176	0,2	1
Zdhhc5	0,464139	0,000716	0,165	0,186	1
St6galnac5	0,46456	0,009614	0,033	0,025	1
Mdm4	0,464752	0,033294	0,672	0,681	1
4833418N(	0,464821	0,003386	0,022	0,03	1
Zbtb8a	0,464833	0,009201	0,018	0,013	1
Stam	0,464845	0,088808	0,484	0,521	1
Cep70	0,465498	0,010203	0,143	0,165	1
Tex30	0,465653	-0,00675	0,101	0,116	1
Ccdc114	0,46605	0,007119	0,018	0,013	1
Hells	0,46605	0,007119	0,018	0,013	1
Ppfia1	0,466369	0,016991	0,273	0,304	1
Lonrf2	0,46676	0,008983	0,033	0,025	1
Ifrd1	0,467077	0,026154	0,534	0,612	1
F8	0,467163	-0,00556	0,068	0,08	1
Rhbdf1	0,467868	0,005323	0,018	0,013	1
Kti12	0,468054	0,019205	0,108	0,095	1
Acaca	0,468077	0,057132	0,806	0,819	1
Camk2n2	0,468107	-0,01772	0,061	0,072	1
R3hcc1l	0,46813	0,025218	0,224	0,255	1
Gns	0,46816	0,017677	0,407	0,451	1
Irak3	0,468842	-0,00741	0,119	0,135	1
Elfn2	0,469089	0,005043	0,018	0,013	1
Lrrc8a	0,469476	0,009857	0,171	0,194	1
Rnf2	0,469559	0,011462	0,132	0,152	1
Lypla1	0,469649	-0,00251	0,204	0,226	1
Tpp2	0,469726	0,01669	0,294	0,331	1
Bscl2	0,469942	0,020662	0,363	0,403	1
Cul7	0,470124	0,022755	0,143	0,129	1

Nbr1	0,47043	0,027721	0,763	0,808	1
Tbc1d24	0,470778	0,011945	0,193	0,217	1
Gm48551	0,47102	0,015155	0,042	0,034	1
Chn2	0,471516	-0,0117	0,881	0,882	1
Zfp697	0,471528	0,013391	0,026	0,019	1
Rnaset2b	0,471557	0,000262	0,136	0,154	1
B4galt5	0,471783	0,009838	0,026	0,019	1
Zfyve27	0,471893	0,008425	0,391	0,432	1
Arl2bp	0,472007	-0,00682	0,842	0,859	1
Usp37	0,472092	0,063546	0,328	0,321	1
Vars	0,472539	-0,00283	0,239	0,259	1
Ptpn12	0,472607	0,041885	0,209	0,194	1
Ccdc9	0,472955	0,001358	0,136	0,154	1
Fam168a	0,472981	-0,01667	0,971	0,968	1
Gnb2	0,473413	0,012942	0,982	0,983	1
Hivep1	0,473818	-0,01205	0,066	0,078	1
Gnao1	0,474624	-0,01283	0,998	0,998	1
Gm26853	0,474958	0,019533	0,235	0,264	1
Golga4	0,475059	0,048774	0,615	0,614	1
Slc22a23	0,475527	0,092819	0,563	0,603	1
Zc3h12c	0,475908	0,006286	0,138	0,156	1
Tab2	0,476056	0,038211	0,655	0,673	1
Gm26882	0,476556	0,031047	0,094	0,082	1
Camta1	0,477025	0,029972	0,576	0,639	1
Dvl2	0,477791	0,016208	0,229	0,257	1
Crtc3	0,477882	0,079784	0,352	0,359	1
Zfp46	0,478163	0,008904	0,139	0,158	1
Pcdhb19	0,478888	0,025202	0,07	0,059	1
Pcdh17	0,479109	-0,00211	0,424	0,487	1
Ppt1	0,479562	-0,00346	0,738	0,785	1
Pcdhgc5	0,47977	0,012829	0,042	0,034	1
Rpn1	0,480164	0,000643	0,758	0,793	1
Elac1	0,480347	0,036312	0,149	0,135	1
Rb1	0,480401	0,073413	0,426	0,439	1
Twsg1	0,48049	0,007025	0,233	0,257	1
Rffl	0,480528	0,007726	0,78	0,833	1
Arap2	0,480603	0,046456	0,936	0,905	1
Akap9	0,480649	0,004588	0,774	0,783	1
Prdm15	0,481026	0,017295	0,088	0,103	1
Itgb4	0,481258	-0,00978	0,772	0,757	1
Flot2	0,481937	0,060669	0,303	0,293	1
Rab7b	0,481962	-0,02161	0,152	0,169	1
Lztr1	0,482796	0,017897	0,45	0,496	1
Katnb1	0,483368	-0,00852	0,143	0,16	1
Tom1	0,48362	0,025913	0,38	0,424	1
Cnot3	0,483644	-0,00044	0,532	0,591	1
Apoa2	0,483664	-0,00448	0,002	0,004	1
Meig1	0,483664	-0,00448	0,002	0,004	1
Efhc2	0,483664	-0,00448	0,002	0,004	1
Eva1b	0,483664	-0,00448	0,002	0,004	1
Arhgap18	0,483664	-0,00448	0,002	0,004	1
Gm45894	0,483664	-0,00448	0,002	0,004	1

M5C1000I:	0,483664	-0,00448	0,002	0,004	1
Spsb4	0,483664	-0,00448	0,002	0,004	1
Arhgap44	0,483664	-0,00448	0,002	0,004	1
Ebi3	0,483664	-0,00448	0,002	0,004	1
Rell2	0,483664	-0,00448	0,002	0,004	1
Gjb6	0,483664	-0,00657	0,002	0,004	1
Spry4	0,483664	-0,00866	0,002	0,004	1
Mpp4	0,484374	-0,00238	0,002	0,004	1
Zdbf2	0,484374	-0,00238	0,002	0,004	1
Myl1	0,484374	-0,00238	0,002	0,004	1
Gm28535	0,484374	-0,00238	0,002	0,004	1
Plekha6	0,484374	-0,00238	0,002	0,004	1
Platr23	0,484374	-0,00238	0,002	0,004	1
Cd84	0,484374	-0,00238	0,002	0,004	1
4930551O:	0,484374	-0,00238	0,002	0,004	1
Fcnaos	0,484374	-0,00238	0,002	0,004	1
Ttc16	0,484374	-0,00238	0,002	0,004	1
Trp53i11	0,484374	-0,00238	0,002	0,004	1
Elf5	0,484374	-0,00238	0,002	0,004	1
Lamp5	0,484374	-0,00238	0,002	0,004	1
6820408C:	0,484374	-0,00238	0,002	0,004	1
Ptprt	0,484374	-0,00238	0,002	0,004	1
Bmp7	0,484374	-0,00238	0,002	0,004	1
Gm14862	0,484374	-0,00238	0,002	0,004	1
Cybb	0,484374	-0,00238	0,002	0,004	1
Gpc4	0,484374	-0,00238	0,002	0,004	1
Xlr4b	0,484374	-0,00238	0,002	0,004	1
Kcne1l	0,484374	-0,00238	0,002	0,004	1
Dcx	0,484374	-0,00238	0,002	0,004	1
Gm20754	0,484374	-0,00238	0,002	0,004	1
Tlr2	0,484374	-0,00238	0,002	0,004	1
Gm43714	0,484374	-0,00238	0,002	0,004	1
She	0,484374	-0,00238	0,002	0,004	1
Gm31305	0,484374	-0,00238	0,002	0,004	1
Casq2	0,484374	-0,00238	0,002	0,004	1
Gm21962	0,484374	-0,00238	0,002	0,004	1
Epha7	0,484374	-0,00238	0,002	0,004	1
Ak4	0,484374	-0,00238	0,002	0,004	1
Skint8	0,484374	-0,00238	0,002	0,004	1
Csf3r	0,484374	-0,00238	0,002	0,004	1
Trnp1	0,484374	-0,00238	0,002	0,004	1
Nphp4	0,484374	-0,00238	0,002	0,004	1
Gm10062	0,484374	-0,00238	0,002	0,004	1
Ppp2r2cos	0,484374	-0,00238	0,002	0,004	1
Ncapg	0,484374	-0,00238	0,002	0,004	1
0610040J0	0,484374	-0,00238	0,002	0,004	1
Rhoh	0,484374	-0,00238	0,002	0,004	1
9130230L2	0,484374	-0,00238	0,002	0,004	1
Hcar2	0,484374	-0,00238	0,002	0,004	1
Tmem130	0,484374	-0,00238	0,002	0,004	1
Hoxa9	0,484374	-0,00238	0,002	0,004	1
A2m	0,484374	-0,00238	0,002	0,004	1

Cdca3	0,484374	-0,00238	0,002	0,004	1
Gm15704	0,484374	-0,00238	0,002	0,004	1
Vmn2r28	0,484374	-0,00238	0,002	0,004	1
Vmn2r29	0,484374	-0,00238	0,002	0,004	1
Cd22	0,484374	-0,00238	0,002	0,004	1
Prss23	0,484374	-0,00238	0,002	0,004	1
Ddias	0,484374	-0,00238	0,002	0,004	1
Plk1	0,484374	-0,00238	0,002	0,004	1
Coro1a	0,484374	-0,00238	0,002	0,004	1
Nps	0,484374	-0,00238	0,002	0,004	1
Pbld2	0,484374	-0,00238	0,002	0,004	1
Itgb2	0,484374	-0,00238	0,002	0,004	1
Chst11	0,484374	-0,00238	0,002	0,004	1
Parpbp	0,484374	-0,00238	0,002	0,004	1
Gm47578	0,484374	-0,00238	0,002	0,004	1
Pawr	0,484374	-0,00238	0,002	0,004	1
Gm32552	0,484374	-0,00238	0,002	0,004	1
Gm16230	0,484374	-0,00238	0,002	0,004	1
Mcf2l	0,484374	-0,00238	0,002	0,004	1
Slc7a2	0,484374	-0,00238	0,002	0,004	1
Galntl6	0,484374	-0,00238	0,002	0,004	1
Csgalnact1	0,484374	-0,00238	0,002	0,004	1
Jak3	0,484374	-0,00238	0,002	0,004	1
Gm35572	0,484374	-0,00238	0,002	0,004	1
4933406B:	0,484374	-0,00238	0,002	0,004	1
Elmo3	0,484374	-0,00238	0,002	0,004	1
Fhod1	0,484374	-0,00238	0,002	0,004	1
Gm17344	0,484374	-0,00238	0,002	0,004	1
A430057M	0,484374	-0,00238	0,002	0,004	1
Nefm	0,484374	-0,00238	0,002	0,004	1
AC114585.	0,484374	-0,00238	0,002	0,004	1
Fli1	0,484374	-0,00238	0,002	0,004	1
Olfr976	0,484374	-0,00238	0,002	0,004	1
1700110K:	0,484374	-0,00238	0,002	0,004	1
Rbms3	0,484374	-0,00238	0,002	0,004	1
4732414G(	0,484374	-0,00238	0,002	0,004	1
Dph1	0,484374	-0,00238	0,002	0,004	1
Abhd15	0,484374	-0,00238	0,002	0,004	1
Plxdc1	0,484374	-0,00238	0,002	0,004	1
Hsd17b1	0,484374	-0,00238	0,002	0,004	1
Golm1	0,484374	-0,00238	0,002	0,004	1
4930525G:	0,484374	-0,00238	0,002	0,004	1
Ckmt2	0,484374	-0,00238	0,002	0,004	1
Gm26527	0,484374	-0,00238	0,002	0,004	1
Gm48780	0,484374	-0,00238	0,002	0,004	1
Batf	0,484374	-0,00238	0,002	0,004	1
Gm26531	0,484374	-0,00238	0,002	0,004	1
Gm32618	0,484374	-0,00238	0,002	0,004	1
Ppara	0,484374	-0,00238	0,002	0,004	1
Cpt1b	0,484374	-0,00238	0,002	0,004	1
Cpne8	0,484374	-0,00238	0,002	0,004	1
AC125199.	0,484374	-0,00238	0,002	0,004	1

Gm16196	0,484374	-0,00238	0,002	0,004	1
Gpsm3	0,484374	-0,00238	0,002	0,004	1
H2-Q6	0,484374	-0,00238	0,002	0,004	1
Gm20443	0,484374	-0,00238	0,002	0,004	1
Capn11	0,484374	-0,00238	0,002	0,004	1
Gnmt	0,484374	-0,00238	0,002	0,004	1
Ddx11	0,484374	-0,00238	0,002	0,004	1
Gm26734	0,484374	-0,00238	0,002	0,004	1
Ankrd29	0,484374	-0,00238	0,002	0,004	1
Gm26823	0,484374	-0,00238	0,002	0,004	1
Mcc	0,484374	-0,00238	0,002	0,004	1
Gm17669	0,484374	-0,00238	0,002	0,004	1
A330032B1	0,484374	-0,00238	0,002	0,004	1
Gm15402	0,484375	-0,00683	0,002	0,004	1
Zfp169	0,484745	0,004012	0,106	0,122	1
Gm26879	0,484747	0,02182	0,044	0,036	1
Stk11ip	0,484951	-0,01475	0,105	0,118	1
Itpr3	0,485085	-0,00318	0,002	0,004	1
Fbxl8	0,485326	0,022334	0,053	0,044	1
Ncbp3	0,485647	0,014572	0,272	0,304	1
Pcdhb17	0,485683	-0,0165	0,114	0,129	1
Pot1b	0,485775	0,037756	0,138	0,127	1
Lpl	0,485797	0,003102	0,002	0,004	1
Gm19345	0,485797	0,001279	0,002	0,004	1
Piwil4	0,485797	0,001279	0,002	0,004	1
Mdh1b	0,485797	-0,00055	0,002	0,004	1
Gm26936	0,485797	-0,00055	0,002	0,004	1
Phf19	0,485797	-0,00055	0,002	0,004	1
Snph	0,485797	-0,00055	0,002	0,004	1
Gm26952	0,485797	-0,00055	0,002	0,004	1
Tsx	0,485797	-0,00055	0,002	0,004	1
Dmrta1	0,485797	-0,00055	0,002	0,004	1
Gm29707	0,485797	-0,00055	0,002	0,004	1
Gm15612	0,485797	-0,00055	0,002	0,004	1
Vmn1r65	0,485797	-0,00055	0,002	0,004	1
Glipr1	0,485797	-0,00055	0,002	0,004	1
Shisa2	0,485797	-0,00055	0,002	0,004	1
Ephx2	0,485797	-0,00055	0,002	0,004	1
Col22a1	0,485797	-0,00055	0,002	0,004	1
Spats2	0,485797	-0,00055	0,002	0,004	1
Krt83	0,485797	-0,00055	0,002	0,004	1
Vasn	0,485797	-0,00055	0,002	0,004	1
Cd74	0,485797	-0,00055	0,002	0,004	1
Prx	0,485797	0,009779	0,002	0,004	1
Ak9	0,485797	0,001003	0,002	0,004	1
Arpc4	0,486035	0,000768	0,71	0,77	1
Vegfa	0,48604	0,04642	0,103	0,118	1
Galns	0,486519	0,023531	0,072	0,061	1
4933434E2	0,487094	-0,00332	0,699	0,77	1
Rras2	0,487628	0,012033	0,061	0,051	1
Pias3	0,487711	0,014821	0,171	0,194	1
Ppp1r3e	0,487844	0,038522	0,169	0,156	1

Fam71e1	0,487921	-0,01198	0,053	0,063	1
Cherp	0,487931	0,048062	0,279	0,268	1
Hnrnpk	0,488153	0,048294	1	0,992	1
Plcb4	0,488262	-0,00837	0,161	0,179	1
Klhl42	0,488672	-0,01009	0,138	0,154	1
Cdkn2c	0,488726	-0,00605	0,061	0,072	1
Cdk12	0,488729	0,057357	0,578	0,61	1
Dyrk1a	0,488891	0,074735	0,406	0,43	1
Mier1	0,489267	0,00484	0,639	0,696	1
Mocs1	0,48932	0,016194	0,119	0,105	1
Abcg2	0,489469	-0,01096	0,053	0,063	1
AA986860	0,489661	-0,00213	0,062	0,074	1
Nrp2	0,490198	0,032645	0,16	0,146	1
Fam160a2	0,490634	-0,00914	0,152	0,169	1
Rala	0,49118	-0,01917	0,817	0,812	1
Prrc1	0,492058	0,041648	0,242	0,232	1
Idh1	0,492244	0,057381	0,53	0,544	1
Focad	0,492256	0,00175	0,095	0,11	1
Nsd3	0,493224	0,060567	0,648	0,673	1
Cep164	0,494041	0,012094	0,253	0,281	1
Gm4593	0,494107	0,102381	0,532	0,574	1
Tgfa	0,494483	0,044894	0,824	0,823	1
Nmnat2	0,494864	0,007975	0,013	0,008	1
Bid	0,494864	0,007975	0,013	0,008	1
Nek8	0,494864	0,01517	0,013	0,008	1
Fam241b	0,495281	-0,00845	0,084	0,097	1
Ubxn4	0,495433	-0,01454	0,846	0,848	1
Pakap	0,495621	0,011286	0,013	0,008	1
Dhrs13os	0,495621	0,009489	0,013	0,008	1
Pold3	0,496155	-0,00401	0,233	0,253	1
Chaf1b	0,496371	0,009779	0,013	0,008	1
Gm21992	0,496371	0,007975	0,013	0,008	1
Gm44718	0,496371	0,006169	0,013	0,008	1
Gm26804	0,496371	0,006169	0,013	0,008	1
Hyls1	0,496371	0,006169	0,013	0,008	1
Trim33	0,496445	0,022249	0,356	0,407	1
Dcaf5	0,497238	0,026239	0,171	0,156	1
Gcn1l1	0,497687	0,031151	0,391	0,445	1
Hoxd4	0,49788	0,004359	0,013	0,008	1
Galnt2	0,49788	0,004359	0,013	0,008	1
Utrn	0,498263	-0,0027	0,013	0,008	1
Wdr4	0,498368	-0,00438	0,088	0,101	1
Ccdc163	0,498552	0,027476	0,242	0,272	1
Dhtkd1	0,498595	-0,00238	0,059	0,07	1
Capn5	0,49892	0,011462	0,266	0,293	1
Thoc2	0,498947	0,066186	0,622	0,641	1
Nyap1	0,499018	0,004079	0,013	0,008	1
Pnrc2	0,499733	0,027882	0,404	0,456	1
Pcdhb10	0,500222	0,016334	0,028	0,021	1
Uhmk1	0,500509	0,064517	0,402	0,405	1
Heatr1	0,500725	-0,00939	0,081	0,093	1
Mtmr4	0,500787	0,071731	0,312	0,31	1

Rgs12	0,50122	0,011616	0,028	0,021	1
Evi2	0,50122	0,011616	0,028	0,021	1
Tpd52	0,501643	-0,00418	0,804	0,812	1
Ephb6	0,501673	0,001993	0,013	0,008	1
Rfx7	0,502013	0,002596	0,262	0,289	1
Fam83d	0,502255	0,024476	0,028	0,021	1
Zhx1	0,502906	0,037215	0,404	0,46	1
Zfp335	0,503082	0,019314	0,083	0,072	1
Zbtb12	0,503421	-0,00426	0,145	0,162	1
Dcaf1	0,50351	0,005182	0,261	0,289	1
Plat	0,503794	-0,01434	0,356	0,384	1
Plod1	0,503989	0,049264	0,71	0,728	1
Zfp281	0,503996	0,070918	0,668	0,696	1
Arih2	0,50401	0,019357	0,33	0,361	1
Arhgap5	0,504046	0,039611	0,952	0,941	1
Dicer1	0,504275	0,079557	0,607	0,66	1
Zfp28	0,504311	0,001552	0,057	0,068	1
Prickle2	0,50457	0,024645	0,393	0,445	1
Mib1	0,504725	0,090069	0,369	0,38	1
1810055G(	0,504785	-0,00628	0,11	0,124	1
Ncapg2	0,504806	0,050223	0,158	0,148	1
Gm17382	0,504856	0,017834	0,02	0,015	1
Bbs4	0,505088	-0,00672	0,138	0,154	1
Ubiad1	0,505954	-0,00892	0,083	0,095	1
St3gal6	0,506449	0,069543	0,272	0,266	1
Tmcc1	0,507118	0,071277	0,897	0,901	1
Lrig2	0,507183	0,011275	0,277	0,306	1
Hbp1	0,507188	0,07759	0,593	0,629	1
Mier2	0,507206	-0,00714	0,172	0,19	1
Cdipt	0,507445	0,010994	0,347	0,386	1
Cdk15	0,50755	0,015757	0,02	0,015	1
Slc2a12	0,507576	0,007774	0,028	0,021	1
Poc1b	0,507824	0,048822	0,196	0,186	1
Zbtb2	0,50786	0,003643	0,127	0,143	1
Rps5	0,508193	0,017966	0,993	0,981	1
Syn1	0,508359	-0,00813	0,106	0,12	1
Nr6a1	0,508883	0,003522	0,057	0,068	1
AC154507.	0,509042	0,008913	0,02	0,015	1
Gm26519	0,509308	0,022124	0,037	0,03	1
Ssbp4	0,509803	0,017105	0,073	0,063	1
Zfp984	0,510126	0,005994	0,028	0,021	1
Bex3	0,510322	0,034466	0,281	0,266	1
Arntl	0,510897	-0,00892	0,079	0,091	1
Pde12	0,511059	0,013824	0,171	0,192	1
Sp4	0,511146	0,004779	0,314	0,344	1
Stk35	0,511862	-0,01978	0,121	0,135	1
Calm2	0,512621	-0,00295	0,998	0,992	1
Recql4	0,512683	0,004209	0,028	0,021	1
Slc22a7	0,512707	0,013972	0,007	0,004	1
Pipox	0,513086	-0,00111	0,055	0,065	1
Nfib	0,513186	0,051353	0,888	0,884	1
Tmem200c	0,51356	0,006554	0,02	0,015	1

Rnf150	0,513745	0,010362	0,007	0,004	1
Rbm22	0,514029	0,049172	0,637	0,652	1
Zfp408	0,514131	0,001812	0,055	0,065	1
Fkbp2	0,514697	0,003282	0,815	0,823	1
Ddx60	0,514783	0,006738	0,007	0,004	1
Gm49226	0,514783	0,006738	0,007	0,004	1
Cpne9	0,514783	0,012168	0,007	0,004	1
Tbx2	0,514783	0,012168	0,007	0,004	1
Tnnc1	0,514783	0,010362	0,007	0,004	1
Stat5b	0,514952	0,007198	0,092	0,105	1
Slc35f6	0,515079	0,021195	0,338	0,378	1
Eif4g2	0,515556	0,045368	0,993	0,998	1
Ttl	0,515793	0,043052	0,128	0,118	1
Il6ra	0,515822	0,008552	0,007	0,004	1
Rfx2	0,515822	0,006738	0,007	0,004	1
Hsd11b1	0,515822	0,004922	0,007	0,004	1
Sall4	0,515822	0,004922	0,007	0,004	1
Gja6	0,515822	0,004922	0,007	0,004	1
Slc27a3	0,515822	0,004922	0,007	0,004	1
Phtf1os	0,515822	0,004922	0,007	0,004	1
Dpysl4	0,515822	0,004922	0,007	0,004	1
4930405J1	0,515822	0,004922	0,007	0,004	1
Eif4ebp1	0,515822	0,004922	0,007	0,004	1
Prkcd	0,515822	0,004922	0,007	0,004	1
Uaca	0,515822	0,004922	0,007	0,004	1
Fbxl22	0,515822	0,004922	0,007	0,004	1
Gm37401	0,515822	0,004922	0,007	0,004	1
Hecw1	0,515822	0,004922	0,007	0,004	1
Rnf144b	0,515822	0,004922	0,007	0,004	1
Gm10269	0,515822	0,004922	0,007	0,004	1
Ppp2ca	0,515959	0,042143	0,998	0,992	1
Cblb	0,51598	0,054381	0,239	0,228	1
Cyth2	0,516028	0,066608	0,582	0,616	1
Camsap2	0,516256	0,092175	0,387	0,409	1
Cdc42ep3	0,516344	0,008263	0,007	0,004	1
Gm43597	0,516488	0,004437	0,061	0,072	1
Mlxip	0,51652	0,04187	0,183	0,173	1
Kif28	0,516862	0,003102	0,007	0,004	1
Cybrd1	0,516862	0,003102	0,007	0,004	1
Ankef1	0,516862	0,003102	0,007	0,004	1
Rhox8	0,516862	0,003102	0,007	0,004	1
Gm15577	0,516862	0,003102	0,007	0,004	1
Cdk19os	0,516862	0,003102	0,007	0,004	1
Pcsk4	0,516862	0,003102	0,007	0,004	1
Gm11175	0,516862	0,003102	0,007	0,004	1
Gm35256	0,516862	0,003102	0,007	0,004	1
Slc12a3	0,516862	0,003102	0,007	0,004	1
Gm16741	0,516862	0,003102	0,007	0,004	1
1700102P(	0,516862	0,003102	0,007	0,004	1
Psme2b	0,516862	0,003102	0,007	0,004	1
Cd300lg	0,516862	0,003102	0,007	0,004	1
Arhgap27	0,516862	0,003102	0,007	0,004	1

Gm17529	0,516862	0,003102	0,007	0,004	1
Gm15738	0,516862	0,003102	0,007	0,004	1
Nme4	0,516862	0,003102	0,007	0,004	1
Pcdha8	0,516862	0,003102	0,007	0,004	1
Gm42997	0,516864	0,00464	0,007	0,004	1
Nfatc1	0,516864	0,00464	0,007	0,004	1
Pla2g15	0,516998	0,003124	0,05	0,059	1
Gpbp1	0,517341	0,051068	0,606	0,654	1
Dchs1	0,517385	0,008263	0,007	0,004	1
Gm16105	0,517385	0,006453	0,007	0,004	1
Wnk2	0,517385	0,00464	0,007	0,004	1
Hsd17b12	0,517565	0,049912	0,815	0,829	1
Tm6sf1	0,517695	0,013695	0,037	0,03	1
Ptpn23	0,517852	0,047245	0,277	0,268	1
Stmn3	0,517855	-0,0088	0,044	0,053	1
Limd2	0,517906	0,002823	0,007	0,004	1
Sema4f	0,517906	0,002269	0,007	0,004	1
Galnt1	0,518156	0,066031	0,501	0,527	1
Rpl19	0,518343	0,024859	1	0,994	1
Flywch2	0,518644	0,001549	0,114	0,129	1
D830035M	0,518948	0,001003	0,007	0,004	1
Tdrp	0,518948	-0,00318	0,007	0,004	1
Mcm2	0,519304	0,000349	0,09	0,103	1
Dnajc18	0,519755	0,005118	0,611	0,658	1
Tln2	0,520099	0,086546	0,495	0,506	1
Gdap1	0,520226	0,051073	0,114	0,103	1
Naa50	0,520669	0,028205	0,484	0,546	1
Mrgpre	0,520725	0,002757	0,084	0,097	1
Dcaf15	0,520906	-0,0083	0,139	0,154	1
Il2	0,521957	-0,00207	0,05	0,059	1
Nupr1	0,522153	0,002529	0,094	0,082	1
Ctdp1	0,522236	0,004643	0,112	0,127	1
Use1	0,522281	0,045873	0,618	0,641	1
Gm9801	0,522291	0,001134	0,051	0,061	1
Slc5a6	0,522562	0,043998	0,13	0,12	1
Rufy3	0,522762	0,042654	0,928	0,935	1
Arcn1	0,523092	0,024787	0,583	0,646	1
Alkbh4	0,523378	0,001395	0,046	0,055	1
Gm29666	0,523497	0,013994	0,037	0,03	1
Scfd1	0,52367	0,034462	0,42	0,466	1
Naglu	0,523841	0,008781	0,171	0,19	1
Tnks1bp1	0,523912	-0,00317	0,079	0,091	1
Hnrnpm	0,52436	0,009355	0,732	0,766	1
Pitrm1	0,524394	0,006069	0,088	0,101	1
Xpnpep3	0,525156	0,006463	0,15	0,169	1
Gm340	0,525822	0,017359	0,064	0,076	1
Cdkal1	0,525898	0,006544	0,305	0,338	1
Cops7b	0,526157	0,012529	0,147	0,165	1
Sos2	0,526319	0,055557	0,475	0,5	1
Chst14	0,526667	0,000183	0,046	0,055	1
Klhl21	0,527077	0,03056	0,112	0,101	1
Dek	0,527476	0,038232	0,855	0,844	1

Nudt12	0,527595	-0,00356	0,042	0,051	1
Thra	0,527708	-0,00257	0,989	0,989	1
Nckipsd	0,527729	-0,00726	0,072	0,082	1
Katna1	0,527752	0,011177	0,187	0,209	1
Adamts6	0,527809	0,006572	0,046	0,055	1
Smarca4	0,528303	0,067995	0,602	0,629	1
Senp6	0,528565	0,040734	0,85	0,84	1
Msrb3	0,528579	0,018394	0,029	0,023	1
Cyp26b1	0,528656	0,008772	0,037	0,03	1
Capn15	0,528825	0,016102	0,29	0,316	1
Cd200	0,528923	0,063087	0,174	0,162	1
Trp53	0,528961	0,029674	0,273	0,259	1
Nol4l	0,529235	0,079522	0,358	0,369	1
Ube2r2	0,529804	0,006792	0,594	0,652	1
AI606181	0,529929	0,000265	0,106	0,12	1
Dync2li1	0,530577	-0,01559	0,13	0,143	1
Map2k6	0,530726	-0,00233	0,048	0,057	1
Nono	0,530803	0,046639	0,642	0,671	1
Nphp3	0,530842	0,001349	0,081	0,093	1
Anapc1	0,531173	-0,00143	0,382	0,422	1
Polr3f	0,53121	0,034036	0,196	0,224	1
Rnf20	0,531317	0,001802	0,624	0,662	1
Gm20604	0,531999	0,001395	0,048	0,057	1
Pabpn1	0,532024	0,06787	0,782	0,823	1
Klhdc4	0,532151	-0,00176	0,176	0,196	1
Gm5148	0,532182	0,010009	0,048	0,057	1
Ephx4	0,532379	0,001134	0,048	0,057	1
Slc44a2	0,532435	0,029021	0,387	0,432	1
Rpl10a	0,53264	0,056985	0,912	0,914	1
Glt1d1	0,532665	-0,00454	0,04	0,049	1
AI314180	0,532969	0,036238	0,501	0,572	1
Il12a	0,533004	-7,7E-05	0,042	0,051	1
Gpcpd1	0,533286	0,025846	0,147	0,167	1
Rab3b	0,533558	0,034181	0,209	0,198	1
Ammecr1	0,533873	0,029111	0,088	0,078	1
2510039O	0,5339	0,019143	0,328	0,361	1
Gm12976	0,534197	0,001962	0,073	0,063	1
Scyl2	0,534233	0,036477	0,22	0,209	1
Ap2m1	0,534274	0,041591	0,928	0,947	1
Dcaf6	0,535188	0,019027	0,336	0,367	1
Naa16	0,535328	0,04085	0,24	0,23	1
Knop1	0,536323	0,030643	0,622	0,679	1
Fkbp7	0,536435	-0,0068	0,04	0,049	1
Nlgn2	0,536512	0,019832	0,206	0,192	1
Isoc1	0,536539	0,002108	0,079	0,091	1
Zfp715	0,536771	-0,00975	0,114	0,127	1
C1qtnf12	0,536859	0,001854	0,079	0,091	1
Cpsf1	0,53694	0,003407	0,18	0,198	1
Rela	0,536951	0,013301	0,429	0,473	1
Fam8a1	0,537012	0,018143	0,308	0,342	1
Gm48960	0,537647	0,014572	0,029	0,023	1
Rnft1	0,538018	0,032777	0,103	0,093	1

1700020I1	0,53816	0,067342	0,593	0,629	1
Rassf8	0,538219	0,016343	0,429	0,492	1
Fbf1	0,538417	-0,00229	0,15	0,167	1
Tmem229b	0,538427	-0,01019	0,068	0,078	1
Raf1	0,539568	0,057648	0,569	0,614	1
Chadl	0,53993	-0,01588	0,128	0,143	1
Plscr3	0,539986	-0,01189	0,139	0,154	1
Tbc1d16	0,54004	0,074348	0,418	0,439	1
Atp2a2	0,540135	-0,03214	0,866	0,869	1
Elovl6	0,540387	0,029533	0,679	0,741	1
Bmpr1a	0,54057	0,023685	0,299	0,333	1
Bbs1	0,541081	0,006069	0,084	0,097	1
Bloc1s6os	0,541449	0,019303	0,022	0,017	1
Gpr19	0,541828	0,035138	0,154	0,143	1
Kdelc2	0,542392	0,015438	0,048	0,04	1
2610020C(	0,542655	-0,00279	0,039	0,046	1
Fam208a	0,54323	0,038767	0,71	0,709	1
Oaf	0,543318	-0,00279	0,037	0,044	1
Gen1	0,543476	0,007493	0,029	0,023	1
BC024978	0,543657	-0,00088	0,097	0,11	1
Mapkapk2	0,543682	0,023372	0,347	0,39	1
Tsta3	0,54376	0,039938	0,242	0,234	1
Kansl1l	0,543793	0,087088	0,338	0,348	1
Ets2	0,543802	0,015757	0,022	0,017	1
Gad2	0,544146	-0,00474	0,004	0,006	1
Serinc4	0,544146	-0,00474	0,004	0,006	1
Gm11851	0,544146	-0,00474	0,004	0,006	1
9530046B1	0,544146	-0,00474	0,004	0,006	1
Uba7	0,544146	-0,00474	0,004	0,006	1
Hmmr	0,544146	-0,00474	0,004	0,006	1
Sgo1	0,544146	-0,00474	0,004	0,006	1
Zmynd10	0,544146	-0,00892	0,004	0,006	1
Cd24a	0,544146	-0,011	0,004	0,006	1
1810041L1	0,544146	-0,02134	0,004	0,006	1
Hba-a2	0,544146	-0,11553	0,004	0,006	1
Shq1	0,544835	-0,00776	0,066	0,076	1
Ccdc14	0,544959	0,01746	0,048	0,04	1
Gm26892	0,545317	-0,00265	0,004	0,006	1
Gm36975	0,545317	-0,00265	0,004	0,006	1
Fcrl6	0,545317	-0,00265	0,004	0,006	1
Dlx1as	0,545317	-0,00265	0,004	0,006	1
Olfr1191-p	0,545317	-0,00265	0,004	0,006	1
Slc12a5	0,545317	-0,00265	0,004	0,006	1
Gm14820	0,545317	-0,00265	0,004	0,006	1
Tm4sf4	0,545317	-0,00265	0,004	0,006	1
Kcnn3	0,545317	-0,00265	0,004	0,006	1
Rrh	0,545317	-0,00265	0,004	0,006	1
Gm11261	0,545317	-0,00265	0,004	0,006	1
Stpg1	0,545317	-0,00265	0,004	0,006	1
Fhad1	0,545317	-0,00265	0,004	0,006	1
Gm13091	0,545317	-0,00265	0,004	0,006	1
Cd38	0,545317	-0,00265	0,004	0,006	1

Gm42745	0,545317	-0,00265	0,004	0,006	1
Gm43136	0,545317	-0,00265	0,004	0,006	1
Hepacam2	0,545317	-0,00265	0,004	0,006	1
Irf5	0,545317	-0,00265	0,004	0,006	1
5730596B2	0,545317	-0,00265	0,004	0,006	1
Loxl3	0,545317	-0,00265	0,004	0,006	1
Gm4969	0,545317	-0,00265	0,004	0,006	1
Ttc9b	0,545317	-0,00265	0,004	0,006	1
Fbxo17	0,545317	-0,00265	0,004	0,006	1
Rasgrp4	0,545317	-0,00265	0,004	0,006	1
Lsr	0,545317	-0,00265	0,004	0,006	1
Gm26790	0,545317	-0,00265	0,004	0,006	1
Syngr4	0,545317	-0,00265	0,004	0,006	1
Ttc23	0,545317	-0,00265	0,004	0,006	1
Lmo1	0,545317	-0,00265	0,004	0,006	1
Gm44647	0,545317	-0,00265	0,004	0,006	1
Pgghg	0,545317	-0,00265	0,004	0,006	1
Smpdl3a	0,545317	-0,00265	0,004	0,006	1
Gm16235	0,545317	-0,00265	0,004	0,006	1
Tnfsf13b	0,545317	-0,00265	0,004	0,006	1
Gm21119	0,545317	-0,00265	0,004	0,006	1
Gm16183	0,545317	-0,00265	0,004	0,006	1
Lingo1	0,545317	-0,00265	0,004	0,006	1
Odf3l1	0,545317	-0,00265	0,004	0,006	1
Ttk	0,545317	-0,00265	0,004	0,006	1
Slc35g2	0,545317	-0,00265	0,004	0,006	1
4833445I0	0,545317	-0,00265	0,004	0,006	1
Gm28048	0,545317	-0,00265	0,004	0,006	1
1700125H1	0,545317	-0,00265	0,004	0,006	1
Sp6	0,545317	-0,00265	0,004	0,006	1
C030017D0	0,545317	-0,00265	0,004	0,006	1
Gm31508	0,545317	-0,00265	0,004	0,006	1
Gm17597	0,545317	-0,00265	0,004	0,006	1
Polq	0,545317	-0,00265	0,004	0,006	1
Stx19	0,545317	-0,00265	0,004	0,006	1
Gm20468	0,545317	-0,00265	0,004	0,006	1
Ndc80	0,545317	-0,00265	0,004	0,006	1
Gm10184	0,545317	-0,00265	0,004	0,006	1
Rasgrp2	0,545317	-0,00265	0,004	0,006	1
Elk1	0,545635	1,43E-05	0,073	0,084	1
Rbm4	0,54564	0,024903	0,079	0,07	1
Pmel	0,545904	-0,00291	0,004	0,006	1
Ggt6	0,545904	-0,00291	0,004	0,006	1
Serpinf1	0,545904	-0,00291	0,004	0,006	1
Gm47015	0,545904	-0,00812	0,004	0,006	1
Tbc1d25	0,545948	0,002717	0,04	0,049	1
4833413G1	0,546719	-0,00078	0,039	0,046	1
Kbtbd11	0,546815	0,016535	0,077	0,068	1
4933429H1	0,547076	0,001003	0,004	0,006	1
Rnf223	0,547076	0,001003	0,004	0,006	1
Mtus2	0,547076	0,001003	0,004	0,006	1
Fcgbp	0,547076	0,001003	0,004	0,006	1

Rbfox1	0,547076	0,001003	0,004	0,006	1
Gm26562	0,547076	0,001003	0,004	0,006	1
Npl	0,547076	-0,00082	0,004	0,006	1
Exo1	0,547076	-0,00082	0,004	0,006	1
Disp1	0,547076	-0,00082	0,004	0,006	1
AA543186	0,547076	-0,00082	0,004	0,006	1
Gm26899	0,547076	-0,00082	0,004	0,006	1
Lhfp	0,547076	-0,00082	0,004	0,006	1
Fam151a	0,547076	-0,00082	0,004	0,006	1
Cyp4x1	0,547076	-0,00082	0,004	0,006	1
Pzp	0,547076	-0,00082	0,004	0,006	1
Scamp4	0,547076	-0,00082	0,004	0,006	1
Sh2d4b	0,547076	-0,00082	0,004	0,006	1
Dlgap5	0,547076	-0,00082	0,004	0,006	1
Gm12224	0,547076	-0,00082	0,004	0,006	1
Slfn5	0,547076	-0,00082	0,004	0,006	1
Tbx4	0,547076	-0,00082	0,004	0,006	1
Myl4	0,547076	-0,00082	0,004	0,006	1
Pitpnc1	0,547076	-0,00082	0,004	0,006	1
Cacng4	0,547076	-0,00082	0,004	0,006	1
Gmpr	0,547076	-0,00082	0,004	0,006	1
Rslcan18	0,547076	-0,00082	0,004	0,006	1
Abcd2	0,547076	-0,00082	0,004	0,006	1
Gng13	0,547076	-0,00082	0,004	0,006	1
Gm15956	0,547076	-0,00082	0,004	0,006	1
2010110K1	0,547076	-0,00082	0,004	0,006	1
Fam19a5	0,547728	0,034404	0,483	0,555	1
Grin3a	0,548251	0,000729	0,004	0,006	1
Lrriq3	0,548523	0,013979	0,022	0,017	1
Tbl2	0,548756	-0,00447	0,136	0,15	1
Gm43403	0,548839	0,001003	0,004	0,006	1
Gm45069	0,548839	0,00464	0,004	0,006	1
Plau	0,548839	0,002823	0,004	0,006	1
Nap1l2	0,549416	0,011907	0,022	0,017	1
Tapbp	0,549866	0,077665	0,33	0,333	1
Wdr59	0,549926	0,001945	0,202	0,222	1
Dll1	0,550328	0,065808	0,062	0,055	1
Gm12758	0,55055	0,002717	0,04	0,049	1
Gm40841	0,55089	0,008627	0,022	0,017	1
Epas1	0,551033	-0,00108	0,114	0,127	1
Dzank1	0,551682	-0,00628	0,09	0,101	1
Gm38393	0,551756	0,026133	0,092	0,082	1
Prdm10	0,551923	0,001854	0,075	0,086	1
Gm13905	0,552083	0,008342	0,022	0,017	1
Sos1	0,552669	0,028627	0,486	0,557	1
Gm36423	0,552925	0,009489	0,015	0,011	1
Col23a1	0,552925	0,01308	0,015	0,011	1
Hic2	0,552978	0,028674	0,081	0,072	1
Tmem210	0,553262	0,006836	0,022	0,017	1
Ilk	0,553415	0,016092	0,637	0,696	1
Rin2	0,553988	0,057757	0,259	0,253	1
Wdr81	0,554244	0,042165	0,206	0,196	1

Zfp777	0,554458	0,008389	0,108	0,122	1
Gm13919	0,554761	0,007689	0,015	0,011	1
Cpxm1	0,554761	0,007689	0,015	0,011	1
Ipo4	0,554822	-0,00583	0,161	0,177	1
1700020D(	0,555058	-0,01465	0,029	0,036	1
Cep135	0,55584	0,001735	0,132	0,146	1
Eid1	0,555953	0,003042	0,974	0,981	1
Jam2	0,556455	-0,00049	0,07	0,08	1
Pccb	0,556471	0,001682	0,361	0,395	1
Ankrd13c	0,556544	0,024962	0,409	0,46	1
Gm29361	0,5566	0,009489	0,015	0,011	1
Olfr1322	0,5566	0,005886	0,015	0,011	1
Kcnmb4	0,5566	0,005886	0,015	0,011	1
Lrrc8e	0,556838	0,004764	0,022	0,017	1
Taf15	0,557302	0,008668	0,58	0,646	1
Sh3pxd2a	0,557341	0,007404	0,015	0,011	1
Srsf7	0,557641	0,055193	0,668	0,696	1
Gm43915	0,557709	0,005604	0,015	0,011	1
Pwp2	0,558438	0,021977	0,105	0,095	1
Ifi35	0,558574	0,007906	0,073	0,084	1
Nploc4	0,558644	0,012379	0,279	0,31	1
Zc3h10	0,558774	0,026031	0,128	0,118	1
Kcnn1	0,559128	-0,00175	0,031	0,038	1
Bag3	0,559269	0,06386	0,29	0,283	1
Map3k1	0,55927	-0,00706	0,029	0,036	1
2010015M	0,559513	-0,00731	0,029	0,036	1
Tox2	0,559554	0,0038	0,015	0,011	1
AC160336.	0,559639	-0,08095	0,884	0,861	1
Btbd6	0,55971	0,024394	0,16	0,148	1
Pik3r2	0,559838	0,056395	0,328	0,333	1
Fam193a	0,560072	0,034673	0,415	0,458	1
Ppip5k1	0,560104	0,044629	0,248	0,238	1
Pvt1	0,560153	0,020446	0,176	0,196	1
Axin1	0,560291	0,017846	0,226	0,251	1
G3bp1	0,561076	0,019217	0,594	0,652	1
Nenf	0,561229	0,039221	0,877	0,878	1
Wiz	0,561313	0,012682	0,317	0,348	1
Rac3	0,561867	-0,00378	0,031	0,038	1
Nt5dc1	0,561991	-0,00026	0,033	0,04	1
Nqo2	0,562085	-0,00326	0,031	0,038	1
Ncoa5	0,562282	0,004461	0,158	0,175	1
Ensa	0,562292	0,004359	0,563	0,631	1
Peli3	0,562313	-0,00149	0,031	0,038	1
Slc25a20	0,562878	0,020248	0,048	0,057	1
Zscan20	0,563047	0,00534	0,07	0,08	1
Aff3	0,56306	0,02169	0,149	0,137	1
Slc24a3	0,563348	0,018283	0,105	0,118	1
Spty2d1	0,563591	0,063104	0,272	0,268	1
Tbc1d17	0,563686	0,0192	0,556	0,616	1
Vcpkmt	0,564097	-0,0063	0,028	0,034	1
Sec24b	0,564103	0,048764	0,327	0,376	1
Homer1	0,564242	0,037714	0,206	0,232	1

Man2c1os	0,564349	-0,00656	0,028	0,034	1
Prkar1a	0,565105	-0,01508	0,98	0,966	1
Slco2b1	0,565235	0,012256	0,04	0,034	1
Adgra1	0,565319	-0,00123	0,028	0,034	1
Herc4	0,565642	0,01324	0,371	0,414	1
Mtmr3	0,5658	0,021658	0,29	0,323	1
Zfp827	0,566309	0,049912	0,163	0,154	1
Pip5k1a	0,566497	0,021352	0,262	0,289	1
Marveld1	0,566776	-0,00274	0,029	0,036	1
Dhx16	0,566861	0,027726	0,237	0,264	1
Nr2c2	0,566895	0,046244	0,442	0,508	1
Gm42893	0,567258	-0,00123	0,029	0,036	1
Evi5	0,567474	0,02643	0,083	0,074	1
Tfeb	0,56791	0,062184	0,505	0,527	1
Prps1	0,568122	-0,01099	0,178	0,192	1
Ltbp3	0,568285	0,011688	0,05	0,042	1
Ewsr1	0,568548	0,026725	0,798	0,861	1
Vps4b	0,568711	0,031632	0,538	0,601	1
Slc9a1	0,568874	-0,00518	0,176	0,192	1
Slc30a4	0,569095	0,024291	0,242	0,27	1
Cenpl	0,569183	0,008772	0,035	0,042	1
Mbd5	0,569287	0,017183	0,211	0,234	1
Ncln	0,569681	0,053241	0,514	0,536	1
Npdc1	0,569748	-0,00076	0,802	0,838	1
Pacs1	0,569835	-0,00452	0,026	0,032	1
Ap2b1	0,570328	0,05966	0,655	0,707	1
Rnf215	0,570389	0,012653	0,213	0,234	1
Dbr1	0,570504	-0,00372	0,108	0,12	1
Dmtn	0,570761	-0,00827	0,057	0,065	1
Prkx	0,570777	0,036312	0,222	0,213	1
Uhrf1bp1l	0,570802	0,033693	0,147	0,137	1
Ica1l	0,570824	-0,005	0,006	0,008	1
Dnhd1	0,570824	-0,005	0,006	0,008	1
Lrrc63	0,570824	-0,005	0,006	0,008	1
Gm26829	0,570824	-0,005	0,006	0,008	1
Kif19a	0,570824	-0,00709	0,006	0,008	1
Clic3	0,570824	-0,01125	0,006	0,008	1
Pde4dip	0,570848	0,02698	0,639	0,7	1
Fbln1	0,571332	-0,00527	0,006	0,008	1
Star	0,571633	-0,00248	0,028	0,034	1
Cbln1	0,571633	-0,00248	0,028	0,034	1
Zfp213	0,571913	0,002907	0,123	0,137	1
Fv1	0,572344	-0,00291	0,006	0,008	1
Rhof	0,572344	-0,00291	0,006	0,008	1
Fancd2	0,572344	-0,00291	0,006	0,008	1
Aldh1a3	0,572344	-0,00291	0,006	0,008	1
Cers3	0,572344	-0,00291	0,006	0,008	1
Rassf7	0,572344	-0,00291	0,006	0,008	1
Gm48935	0,572344	-0,00291	0,006	0,008	1
Gm26797	0,572344	-0,00291	0,006	0,008	1
Ccdc85a	0,572344	-0,00291	0,006	0,008	1
Gm26534	0,572344	-0,00291	0,006	0,008	1

Hist1h2bb	0,572344	-0,00291	0,006	0,008	1
Gm48653	0,572344	-0,00291	0,006	0,008	1
AC140186.	0,572344	-0,00291	0,006	0,008	1
Olfr107	0,572344	-0,00291	0,006	0,008	1
Slc1a1	0,572344	-0,00291	0,006	0,008	1
A930007I1	0,572344	-0,00291	0,006	0,008	1
Gm16068	0,572344	-0,00291	0,006	0,008	1
Zan	0,572346	-0,00527	0,006	0,008	1
Pfkfb3	0,572475	0,022208	0,217	0,241	1
Dpp8	0,572505	0,025663	0,417	0,466	1
Hace1	0,572641	0,023023	0,253	0,241	1
Arl8a	0,572717	0,015857	0,974	0,979	1
Gm38944	0,572853	-0,00318	0,006	0,008	1
Alox8	0,572853	-0,00318	0,006	0,008	1
Gm12359	0,572853	-0,00318	0,006	0,008	1
Rgs11	0,572853	-0,00318	0,006	0,008	1
Tro	0,573284	-0,01322	0,02	0,025	1
Wdr25	0,573908	-0,00579	0,024	0,03	1
Far2os1	0,574376	0,002546	0,006	0,008	1
Cdc25b	0,574376	-0,00109	0,006	0,008	1
Msx1	0,574376	-0,00109	0,006	0,008	1
Gabrb1	0,574376	-0,00109	0,006	0,008	1
Akr1b10	0,574376	-0,00109	0,006	0,008	1
Phldb3	0,574376	-0,00109	0,006	0,008	1
A230060F1	0,574376	-0,00109	0,006	0,008	1
Kcnh5	0,574376	-0,00109	0,006	0,008	1
Gm26785	0,574376	-0,00109	0,006	0,008	1
Ablim3	0,574376	-0,00109	0,006	0,008	1
5930430L(	0,574554	0,023531	0,061	0,053	1
Eps8l1	0,574832	0,017425	0,061	0,053	1
Smad2	0,575469	0,044243	0,165	0,158	1
Armc8	0,575534	0,072392	0,295	0,3	1
Rpl37	0,575727	0,005015	0,996	0,998	1
Wdr6	0,576289	0,002571	0,028	0,034	1
Gm960	0,576412	0,000729	0,006	0,008	1
Cables1	0,576412	0,002546	0,006	0,008	1
BC037034	0,577388	-0,00372	0,108	0,12	1
Zfp583	0,577533	-0,00924	0,055	0,063	1
Prmt6	0,578013	-0,00185	0,108	0,12	1
6430550D:	0,5781	-0,00044	0,024	0,03	1
Zcchc11	0,578358	0,045787	0,404	0,449	1
Pcdhb3	0,578451	0,007975	0,006	0,008	1
Mios	0,579164	0,009757	0,158	0,173	1
Gm26649	0,579354	0,004064	0,029	0,036	1
Cenps	0,579434	-0,00478	0,026	0,032	1
Zfp114	0,579942	-0,00876	0,053	0,061	1
Lca5	0,580388	0,023325	0,095	0,086	1
Nfkb2	0,580836	0,013716	0,061	0,053	1
Anp32e	0,581355	0,053106	0,642	0,679	1
Tbc1d2	0,581517	0,001339	0,024	0,03	1
Ppp1r26	0,581523	-0,00248	0,024	0,03	1
Lepr	0,58157	-0,01587	0,007	0,011	1

Gm26982	0,581817	-0,00348	0,022	0,027	1
Cnksr2	0,582105	-0,00426	0,022	0,027	1
Ppp4r3a	0,582286	0,082097	0,466	0,502	1
Nup133	0,582416	0,015714	0,246	0,268	1
Pkn2	0,582721	0,066485	0,622	0,65	1
Maf1	0,582902	0,018007	0,574	0,656	1
Synrg	0,583068	-0,00128	0,635	0,681	1
Rapgef1	0,583541	0,004971	0,2	0,219	1
Gm19710	0,583826	-0,00527	0,007	0,011	1
Dynlrb2	0,583826	-0,00527	0,007	0,011	1
Slc29a2	0,584002	0,001339	0,026	0,032	1
Tbc1d15	0,584033	0,022616	0,666	0,738	1
Zfp455	0,584518	0,002842	0,026	0,032	1
Ric8b	0,584738	0,020638	0,215	0,203	1
Incenp	0,585049	0,018548	0,062	0,055	1
Tab3	0,585053	-0,00092	0,061	0,07	1
Ifngr1	0,585308	0,016896	0,233	0,257	1
Cmtm4	0,585466	0,036374	0,099	0,091	1
Dcbld1	0,585634	-0,00318	0,007	0,011	1
Agap2	0,585634	-0,00318	0,007	0,011	1
Gm45733	0,585634	-0,00318	0,007	0,011	1
Gm11520	0,585634	-0,00318	0,007	0,011	1
Gm4211	0,585634	-0,00318	0,007	0,011	1
Zfp641	0,585634	-0,00318	0,007	0,011	1
Notch4	0,585634	-0,00318	0,007	0,011	1
Gm42669	0,58605	0,000362	0,02	0,025	1
Ebf1	0,58609	-0,00345	0,007	0,011	1
6030408B1	0,58609	-0,00345	0,007	0,011	1
1700020L2	0,586238	0,00326	0,061	0,07	1
Pcdhb21	0,586477	0,026073	0,042	0,036	1
Fgf9	0,586543	-0,00163	0,007	0,011	1
Mapk4	0,586543	-0,00163	0,007	0,011	1
Zfp991	0,586577	0,010126	0,024	0,019	1
Gm48604	0,586577	0,010126	0,024	0,019	1
Ecm1	0,586577	0,011907	0,024	0,019	1
Cox7a1	0,586605	-0,00476	0,018	0,023	1
Pip5k1c	0,586703	0,012414	0,097	0,11	1
Slc4a2	0,586873	0,033546	0,739	0,755	1
Gm10037	0,586911	-0,00502	0,018	0,023	1
Pcdhga4	0,587166	0,013391	0,024	0,019	1
AI429214	0,587231	-0,00656	0,017	0,021	1
Gigyf2	0,587571	0,051981	0,516	0,534	1
Barx2	0,587821	-0,00798	0,055	0,063	1
Muc1	0,587902	0,002269	0,007	0,011	1
4932430I1	0,587902	0,000456	0,007	0,011	1
Katnal2	0,587902	0,000456	0,007	0,011	1
1700084E1	0,587902	-0,00136	0,007	0,011	1
1700112D1	0,587902	-0,00136	0,007	0,011	1
Fam110b	0,587902	-0,00136	0,007	0,011	1
2410021H0	0,587902	-0,00136	0,007	0,011	1
Creb3l4	0,588357	-0,00163	0,007	0,011	1
Tspan32os	0,588357	-0,00163	0,007	0,011	1

Chek1	0,588357	-0,00163	0,007	0,011	1
Gm45352	0,588357	-0,00163	0,007	0,011	1
Nrxn2	0,588392	0,008244	0,673	0,711	1
Psmd2	0,588548	0,037642	0,787	0,793	1
9630013A2	0,588711	-0,04463	0,009	0,013	1
Ccdc96	0,588811	0,000183	0,007	0,011	1
Rnf19a	0,589007	0,07558	0,338	0,346	1
Coq4	0,589411	0,013329	0,172	0,19	1
Sapcd1	0,589774	0,003386	0,022	0,027	1
Qpctl	0,589865	0,009323	0,174	0,192	1
Xk	0,589896	-0,00116	0,018	0,023	1
Rs1	0,589902	-0,00348	0,018	0,023	1
Hgs.1	0,58994	-0,00553	0,009	0,013	1
Gm4787	0,58994	-0,00553	0,009	0,013	1
D11Wsu47	0,59003	-0,00872	0,075	0,084	1
Pdlim7	0,590053	-0,0045	0,017	0,021	1
AC134548.	0,590053	-0,0045	0,017	0,021	1
Kcne4	0,590173	0,01487	0,007	0,011	1
Nfkbie	0,590173	0,002269	0,007	0,011	1
Cdnf	0,590354	-0,00579	0,009	0,013	1
Nepro	0,591593	-0,00605	0,051	0,059	1
Ggt7	0,591629	0,035446	0,237	0,228	1
Adam18	0,591901	-0,00605	0,013	0,017	1
Gm29183	0,591997	-0,00345	0,009	0,013	1
Ankrd42	0,591997	-0,00345	0,009	0,013	1
Gm48882	0,591997	-0,00345	0,009	0,013	1
Gm19935	0,591997	-0,00345	0,009	0,013	1
Gemin4	0,591997	-0,00345	0,009	0,013	1
Hist1h2ac	0,591997	-0,00345	0,009	0,013	1
Dhrs11	0,592	-0,00579	0,009	0,013	1
Lman1	0,592001	0,033412	0,264	0,291	1
Sycp3	0,592131	-0,00579	0,011	0,015	1
Atp2b2	0,592134	-0,00812	0,011	0,015	1
AL589692.	0,592412	-0,00371	0,009	0,013	1
Adipor2	0,592495	-0,01708	0,917	0,914	1
Emilin1	0,592825	-0,0019	0,009	0,013	1
Kifc3	0,592839	0,038486	0,306	0,344	1
Wdr78	0,592923	0,003934	0,02	0,025	1
Ccl22	0,59301	0,006273	0,024	0,019	1
Rbfox2	0,593062	0,016671	0,116	0,129	1
Bcl2	0,593091	-0,0045	0,015	0,019	1
Pgc	0,593091	-0,0045	0,015	0,019	1
Fbxl12os	0,593203	-0,0027	0,017	0,021	1
Smg9	0,593423	0,036107	0,286	0,281	1
Gm26779	0,593524	-0,00296	0,017	0,021	1
Fndc10	0,593668	-0,00283	0,055	0,063	1
Slc41a2	0,593891	0,011082	0,042	0,036	1
Gm10118	0,594375	-0,00397	0,013	0,017	1
Cst6	0,5944	0,007493	0,018	0,023	1
Gm44175	0,594407	-0,00371	0,011	0,015	1
Klk10	0,594407	-0,00371	0,011	0,015	1
Xylb	0,594407	-0,00371	0,011	0,015	1

Dock2	0,594407	-0,00371	0,011	0,015	1
Gm9917	0,594473	0,001993	0,009	0,013	1
Mgst1	0,594473	-0,00163	0,009	0,013	1
Slc1a4	0,594473	-0,00163	0,009	0,013	1
Zfp808	0,594473	-0,00163	0,009	0,013	1
Gm17035	0,594473	-0,00163	0,009	0,013	1
Hoxc8	0,594473	-0,00163	0,009	0,013	1
Mphosph8	0,594653	0,050544	0,558	0,627	1
Fbxw4	0,594713	0,001212	0,134	0,148	1
Gm26786	0,594889	-0,0019	0,009	0,013	1
Wars2	0,594892	-0,00406	0,053	0,061	1
Ate1	0,595115	0,029911	0,539	0,629	1
Bard1	0,595171	-0,00036	0,011	0,015	1
Slc4a3	0,59522	0,010798	0,042	0,036	1
Ylpm1	0,595333	0,025296	0,55	0,612	1
Rpl13a-ps1	0,595355	0,006273	0,024	0,019	1
Hykk	0,595649	0,004486	0,024	0,019	1
Gm15492	0,595753	-0,00063	0,015	0,019	1
Gm4316	0,595753	-0,00063	0,015	0,019	1
Gm9958	0,595753	-0,00243	0,015	0,019	1
Bc1	0,595879	0,010234	0,042	0,036	1
Tbp	0,595962	0,017673	0,165	0,154	1
Gm7008	0,596091	-0,0027	0,015	0,019	1
Syvn1	0,596342	0,000116	0,277	0,302	1
Cep295nl	0,596424	-0,0009	0,015	0,019	1
Gng8	0,596562	0,105914	0,439	0,464	1
Hoxa3	0,59672	0,008713	0,112	0,124	1
Smarca5	0,596777	0,034205	0,895	0,88	1
C230004F1	0,596954	0,000183	0,009	0,013	1
Gstt2	0,596954	0,000183	0,009	0,013	1
Cers1	0,596954	0,000183	0,009	0,013	1
Ropn1l	0,596954	0,000183	0,009	0,013	1
Gm5427	0,596954	0,001993	0,009	0,013	1
Gm15747	0,597072	0,008913	0,011	0,015	1
B230312C(	0,597072	0,003522	0,011	0,015	1
Tmsb15l	0,597072	-0,0019	0,011	0,015	1
2310047D(	0,597072	-0,0019	0,011	0,015	1
Spata33	0,597072	-0,0019	0,011	0,015	1
Zfp965	0,597215	-0,00036	0,013	0,017	1
Gm26590	0,597215	-0,00216	0,013	0,017	1
Phka2	0,597247	0,016366	0,147	0,162	1
Nsun4	0,597397	-0,0046	0,075	0,084	1
Avl9	0,597998	0,018464	0,455	0,498	1
Ptpn2	0,598205	-0,00358	0,138	0,15	1
9230104M	0,598284	0,001173	0,013	0,017	1
Pqlc2	0,598528	0,017575	0,185	0,173	1
Acer3	0,598611	0,012722	0,11	0,124	1
Mthfd2l	0,598686	0,019709	0,095	0,108	1
Stxbp5	0,598783	0,01631	0,172	0,19	1
Cramp1l	0,599013	0,03436	0,206	0,196	1
Nudcd1	0,599093	-0,00355	0,127	0,139	1
Gm26516	0,599097	-0,0009	0,015	0,019	1

Mxi1	0,599389	0,059654	0,662	0,681	1
Speer4b	0,599439	0,001993	0,009	0,013	1
Retreg1	0,599453	0,001065	0,949	0,937	1
4933428G:	0,599525	0,004486	0,017	0,021	1
Gm16907	0,599525	0,002696	0,017	0,021	1
Rin1	0,599743	0,001719	0,011	0,015	1
Psmd3	0,599875	0,036683	0,75	0,759	1
Tnip2	0,599955	0,022761	0,139	0,156	1
Sin3a	0,599957	0,025854	0,233	0,257	1
Pdia5	0,600063	0,000902	0,013	0,017	1
4930590JC	0,600064	0,012785	0,017	0,013	1
Nomo1	0,600087	0,025058	0,259	0,285	1
Tgfb1i1	0,60027	0,003522	0,009	0,013	1
Zfp575	0,600771	-0,00143	0,015	0,019	1
Zc3h4	0,60092	0,025398	0,25	0,274	1
Snx8	0,601086	0,024582	0,283	0,316	1
Lrp10	0,601125	0,019009	0,371	0,407	1
Paip2b	0,601744	-0,01449	0,4	0,445	1
Zfp143	0,601887	0,028451	0,13	0,146	1
Btd	0,601922	0,016886	0,145	0,16	1
Rnf167	0,601954	0,052063	0,56	0,601	1
Gm26772	0,602039	0,021602	0,011	0,015	1
Bik	0,60211	0,000902	0,015	0,019	1
Wdr62	0,602419	0,001719	0,011	0,015	1
1700086O(	0,602502	0,004885	0,033	0,027	1
Tes3-ps	0,602703	0,007302	0,042	0,036	1
Primpol	0,602761	0,01018	0,033	0,027	1
Cd83	0,60291	0,001446	0,013	0,017	1
Rflnb	0,60291	0,003246	0,013	0,017	1
Scn2a	0,602991	-0,01127	0,046	0,053	1
Trip6	0,603511	0,006099	0,033	0,027	1
Gm12355	0,604024	0,008263	0,009	0,006	1
Oaz3	0,604024	0,011873	0,009	0,006	1
D630003M	0,604337	0,012785	0,017	0,013	1
Dnase1l2	0,604791	0,004764	0,015	0,019	1
Mss51	0,605099	0,003522	0,011	0,015	1
Gspt2	0,605268	0,008318	0,086	0,097	1
Flt3l	0,605485	0,006453	0,009	0,006	1
Gm30606	0,605485	0,006453	0,009	0,006	1
Nsl1	0,605485	0,011873	0,009	0,006	1
Lrp3	0,605485	0,01007	0,009	0,006	1
Gm37584	0,605485	0,008263	0,009	0,006	1
Frrs1	0,605485	0,008263	0,009	0,006	1
Gm16350	0,605485	0,008263	0,009	0,006	1
Akt3	0,60588	-0,00266	0,138	0,15	1
Prkab1	0,60589	0,015379	0,119	0,11	1
AC034116.	0,605973	0,007975	0,009	0,006	1
Gale	0,60646	0,005549	0,042	0,036	1
Igfbpl1	0,60646	0,006169	0,009	0,006	1
Zfp970	0,606469	0,011327	0,018	0,023	1
Cr2	0,606478	0,016356	0,017	0,013	1
Zfp781	0,606478	0,009201	0,017	0,013	1

Gnrh1	0,606947	0,008263	0,009	0,006	1
Prdm11	0,606947	0,006453	0,009	0,006	1
Zfp711	0,606947	0,006453	0,009	0,006	1
Gm15478	0,606947	0,006453	0,009	0,006	1
Ampd3	0,606947	0,006453	0,009	0,006	1
Hk1os	0,606947	0,006453	0,009	0,006	1
Gm13528	0,606947	0,00464	0,009	0,006	1
Gm28874	0,606947	0,00464	0,009	0,006	1
1500002C1	0,606947	0,00464	0,009	0,006	1
Fam221a	0,606947	0,00464	0,009	0,006	1
Gm44702	0,606947	0,00464	0,009	0,006	1
Gm44949	0,606947	0,00464	0,009	0,006	1
4930563J1	0,606947	0,00464	0,009	0,006	1
Ppp1r27	0,606947	0,00464	0,009	0,006	1
Ribc2	0,606947	0,00464	0,009	0,006	1
Slc35g1	0,607924	0,004359	0,009	0,006	1
Tmx4	0,608034	0,041015	0,49	0,546	1
Sdc4	0,608145	0,049481	0,169	0,162	1
Irak4	0,608314	-0,00369	0,099	0,11	1
Ipo5	0,608337	0,070113	0,576	0,608	1
Gm9873	0,60841	0,002823	0,009	0,006	1
Vegfd	0,60841	0,002823	0,009	0,006	1
Gm26833	0,60841	0,002823	0,009	0,006	1
Gm45351	0,60841	0,002823	0,009	0,006	1
Lgals7	0,60841	0,002823	0,009	0,006	1
Perp	0,60841	0,002823	0,009	0,006	1
Jph3	0,60841	0,002823	0,009	0,006	1
Mir17hg	0,60841	0,002823	0,009	0,006	1
Tmppe	0,60841	0,002823	0,009	0,006	1
Foxc1	0,60841	0,002823	0,009	0,006	1
4930461C1	0,60841	0,002823	0,009	0,006	1
Galm	0,60841	0,002823	0,009	0,006	1
Ctf1	0,608622	0,009201	0,017	0,013	1
Vstm2b	0,608622	0,005604	0,017	0,013	1
Unc13a	0,608622	0,005604	0,017	0,013	1
Nemp1	0,608658	-0,00381	0,05	0,057	1
Nf2	0,609097	0,049036	0,321	0,321	1
Tbk1	0,609147	0,040317	0,261	0,255	1
Gpx4	0,609215	0,026788	1	0,998	1
Arhgap11a	0,609345	0,008627	0,017	0,013	1
Zfp148	0,609353	0,098271	0,494	0,54	1
E2f1	0,609389	0,002546	0,009	0,006	1
Tollip	0,609964	-0,00159	0,455	0,5	1
Ptpa	0,610197	0,025855	0,672	0,688	1
Prc1	0,610849	0,017231	0,015	0,019	1
Igf2r	0,611748	-0,00293	0,191	0,209	1
Usp28	0,611761	0,026727	0,092	0,084	1
Fanci	0,61185	0,003522	0,017	0,013	1
Lbhd1	0,61185	0,003522	0,017	0,013	1
C730034F0	0,611934	0,007746	0,062	0,055	1
Ercc4	0,612076	-0,00198	0,172	0,188	1
St18	0,612494	0,050584	0,806	0,827	1

Sun1	0,613714	0,061219	0,374	0,388	1
Bach1	0,613822	0,025441	0,345	0,38	1
Zdhhc7	0,613837	-0,00012	0,165	0,179	1
Ccdc15	0,614375	-0,00411	0,075	0,084	1
Megf10	0,614418	0,048238	0,582	0,603	1
Hsdl1	0,61502	0,017397	0,229	0,253	1
Rccd1	0,615487	0,032456	0,176	0,167	1
Fam120c	0,615567	0,040193	0,209	0,203	1
Zc3h3	0,615726	0,02806	0,112	0,103	1
Rpl9-ps6	0,615885	0,011082	0,044	0,038	1
Lima1	0,616939	0,024925	0,191	0,211	1
Kbtbd2	0,616982	0,039901	0,299	0,295	1
Zfp282	0,616988	0,039762	0,172	0,165	1
Heca	0,617219	0,031957	0,297	0,327	1
Gm10516	0,617243	-0,00036	0,017	0,013	1
Ints2	0,617573	0,002552	0,127	0,139	1
Zfhx2	0,618646	0,017493	0,237	0,257	1
Rps12-ps3	0,61876	0,011616	0,026	0,021	1
Ube2h	0,618761	0,040548	0,697	0,713	1
Snx2	0,618913	0,040921	0,464	0,525	1
Sec24d	0,618967	-0,0019	0,121	0,133	1
Large1	0,619557	0,016535	0,077	0,07	1
Usp35	0,619836	0,017742	0,125	0,116	1
Dgcr2	0,620507	0,017733	0,365	0,407	1
Atl1	0,620562	0,036228	0,389	0,437	1
Rbm12	0,620605	0,003522	0,198	0,215	1
Pxn	0,620621	0,012633	0,189	0,207	1
Hsf1	0,620799	0,015704	0,253	0,274	1
Pgm2	0,621002	0,012394	0,196	0,215	1
Cxcr2	0,621344	0,009267	0,026	0,021	1
S100a7a	0,621623	0,011616	0,026	0,021	1
Map3k11	0,621675	0,060712	0,38	0,392	1
Sertad3	0,621847	0,031405	0,143	0,135	1
Gabpb2	0,621875	0,028701	0,367	0,403	1
Serf1	0,62204	0,026031	0,114	0,105	1
Sms	0,622206	0,047586	0,09	0,084	1
Rwdd2a	0,62299	0,002859	0,051	0,059	1
Gpatch1	0,623141	-0,00687	0,2	0,213	1
Xpc	0,623437	0,008964	0,147	0,16	1
Cpeb4	0,623668	0,051659	0,705	0,722	1
Xrn1	0,623883	0,07031	0,435	0,462	1
1110035H1	0,624221	0,010751	0,026	0,021	1
Fam126a	0,624393	0,060236	0,477	0,515	1
B230311B(	0,624492	0,008058	0,026	0,021	1
Klk6	0,625771	-0,32376	0,921	0,802	1
Mark4	0,625877	0,03436	0,204	0,198	1
Speer4a	0,626048	0,021215	0,046	0,04	1
Pbxip1	0,626575	0,046751	0,189	0,184	1
Krit1	0,626673	0,032645	0,761	0,829	1
Cacnb1	0,626682	0,035733	0,059	0,053	1
Orc3	0,627127	0,004334	0,171	0,186	1
Srgap1	0,627193	0,087688	0,411	0,422	1

Xpo1	0,628013	0,08304	0,424	0,439	1
Klf8	0,628222	-0,0063	0,042	0,049	1
Rbm43	0,628238	0,011327	0,026	0,021	1
Zfp566	0,628491	-0,00015	0,092	0,101	1
Ccdc28a	0,628665	0,006232	0,077	0,086	1
Hnrnpf	0,629117	0,039946	0,954	0,93	1
Fubp3	0,629274	0,031136	0,505	0,58	1
Gm7292	0,630073	0,006653	0,035	0,03	1
Fkbp8	0,630283	-0,00056	0,928	0,932	1
Kdm3b	0,630345	0,049559	0,27	0,304	1
Mta2	0,630428	-0,00351	0,193	0,209	1
Erich6	0,630552	0,003659	0,026	0,021	1
Ibtk	0,630776	0,013979	0,328	0,354	1
Mvk	0,631336	0,067896	0,418	0,437	1
Pbx1	0,631683	0,092328	0,294	0,304	1
Cklf	0,632147	0,003522	0,116	0,127	1
Pfdn5	0,632441	0,005839	0,98	0,97	1
Eprs	0,632612	0,017275	0,802	0,829	1
Cnst	0,632786	-0,009	0,127	0,137	1
Thrap3	0,632938	0,041687	0,783	0,783	1
Wdr34	0,632962	-0,00285	0,132	0,143	1
B3gnt9	0,633024	0,039881	0,404	0,445	1
Mtfp1	0,633291	0,000707	0,042	0,049	1
St6gal1	0,63335	0,091722	0,422	0,451	1
Msantd2	0,63347	0,020371	0,152	0,167	1
Shprh	0,633707	0,057002	0,448	0,513	1
Snrk	0,633754	0,000568	0,156	0,169	1
Noc2l	0,634807	0,048605	0,295	0,335	1
Mapk14	0,635155	0,067357	0,264	0,268	1
A730063M	0,635342	-0,00104	0,044	0,051	1
Tjp1	0,635393	0,000916	0,688	0,688	1
Ipo8	0,6359	0,062304	0,345	0,352	1
Kansl2	0,6363	0,024362	0,319	0,35	1
Zbtb40	0,636436	0,000523	0,07	0,078	1
Dennd4a	0,636688	0,051376	0,484	0,561	1
Fmnl2	0,637668	0,014884	0,899	0,897	1
Dhx38	0,637888	0,028396	0,187	0,207	1
Pvr	0,640217	0,009428	0,101	0,112	1
Rtn4r	0,640921	-0,00429	0,04	0,046	1
Cpd	0,641411	0,041336	0,969	0,973	1
Ppcdc	0,64154	0,010781	0,226	0,245	1
Hspa4l	0,641737	0,02711	0,56	0,614	1
Setmar	0,641821	-0,0033	0,04	0,046	1
Pcdhb20	0,642248	0,000349	0,075	0,068	1
Gpkow	0,642323	0,016257	0,206	0,224	1
Slc25a28	0,642607	0,011528	0,349	0,382	1
Fndc3a	0,642764	0,065103	0,297	0,304	1
Ybx1	0,642771	0,016379	0,985	0,981	1
Fam3a	0,643187	0,030562	0,288	0,319	1
Aftph	0,643203	0,047148	0,387	0,441	1
Lipe	0,643231	0,065984	0,394	0,416	1
Zcchc14	0,643397	0,054666	0,31	0,312	1

Dhx34	0,644056	0,003903	0,095	0,105	1
Rdh10	0,644137	0,027325	0,081	0,074	1
Cenpp	0,644184	0,008493	0,046	0,04	1
Ddx39b	0,644436	-0,00201	0,941	0,89	1
Grk6	0,644466	0,025267	0,171	0,162	1
Pemt	0,644538	0,011939	0,037	0,032	1
Pdha1	0,644659	0,034775	0,569	0,572	1
Blvrb	0,645263	0,019486	0,092	0,084	1
Lurap1	0,645411	0,020647	0,259	0,285	1
Morf4l2	0,645632	0,086735	0,723	0,77	1
Api5	0,645645	0,019237	0,484	0,54	1
Thap4	0,645674	-0,00037	0,136	0,148	1
Gm44623	0,645693	-0,00227	0,04	0,046	1
Anp32b	0,645794	0,015361	0,91	0,918	1
Slc2a10	0,645971	0,005205	0,004	0,002	1
Rnf207	0,645971	0,005205	0,004	0,002	1
Lmod3	0,645971	0,005205	0,004	0,002	1
Il4ra	0,645971	0,005205	0,004	0,002	1
Cenph	0,645971	0,005205	0,004	0,002	1
Gm20420	0,645971	0,005205	0,004	0,002	1
Shank3	0,645971	0,005205	0,004	0,002	1
Trank1	0,645971	0,014271	0,004	0,002	1
Prss35	0,645971	0,008842	0,004	0,002	1
Brsk2	0,646009	0,034892	0,321	0,361	1
Ino80	0,64639	0,06297	0,343	0,354	1
Cggbp1	0,646447	0,051388	0,596	0,641	1
Usp47	0,646463	0,022874	0,4	0,437	1
Zfp747	0,646742	0,019027	0,079	0,072	1
H1f0	0,646758	0,062152	0,574	0,639	1
Rln1	0,646787	0,008842	0,004	0,002	1
Atrnl1	0,646787	0,008842	0,004	0,002	1
Mme	0,646787	0,007025	0,004	0,002	1
Kcnip4	0,646787	0,007025	0,004	0,002	1
Adamts9	0,646787	0,007025	0,004	0,002	1
Arhgef17	0,646787	0,007025	0,004	0,002	1
Col5a2	0,646787	0,005205	0,004	0,002	1
Cyr61	0,646787	0,005205	0,004	0,002	1
Aqp1	0,646787	0,005205	0,004	0,002	1
Cox6a2	0,646787	0,005205	0,004	0,002	1
CT025678.	0,646787	0,005205	0,004	0,002	1
Tnk1	0,646787	0,005205	0,004	0,002	1
Copz2	0,646787	0,005205	0,004	0,002	1
Fam84a	0,646787	0,005205	0,004	0,002	1
Rgs6	0,646787	0,005205	0,004	0,002	1
Pld5	0,646787	0,003382	0,004	0,002	1
Capn8	0,646787	0,003382	0,004	0,002	1
Enkur	0,646787	0,003382	0,004	0,002	1
Slc2a6	0,646787	0,003382	0,004	0,002	1
Erv3	0,646787	0,003382	0,004	0,002	1
Gm26869	0,646787	0,003382	0,004	0,002	1
Hist2h2aa2	0,646787	0,003382	0,004	0,002	1
Hspb7	0,646787	0,003382	0,004	0,002	1

Mmp23	0,646787	0,003382	0,004	0,002	1
4930548H:	0,646787	0,003382	0,004	0,002	1
Gm7854	0,646787	0,003382	0,004	0,002	1
1700124L1	0,646787	0,003382	0,004	0,002	1
B430219N:	0,646787	0,003382	0,004	0,002	1
Dram1	0,646787	0,003382	0,004	0,002	1
Nt5dc2	0,646787	0,003382	0,004	0,002	1
Phyhip	0,646787	0,003382	0,004	0,002	1
Plcd1	0,646787	0,003382	0,004	0,002	1
Rcvrn	0,646787	0,003382	0,004	0,002	1
Gm12319	0,646787	0,003382	0,004	0,002	1
Gngt2	0,646787	0,003382	0,004	0,002	1
Tmem106a	0,646787	0,003382	0,004	0,002	1
1810034E1	0,646787	0,003382	0,004	0,002	1
AL591946.	0,646787	0,003382	0,004	0,002	1
Sh3bp1	0,646787	0,003382	0,004	0,002	1
Gm27209	0,646787	0,003382	0,004	0,002	1
Vpreb1	0,646787	0,003382	0,004	0,002	1
Gm10226	0,646787	0,003382	0,004	0,002	1
E230001N(	0,646787	0,003382	0,004	0,002	1
Cfap53	0,646787	0,003382	0,004	0,002	1
Gal3st3	0,646787	0,003382	0,004	0,002	1
Tmem72	0,647037	0,010704	0,018	0,015	1
Gm49164	0,647037	0,012491	0,018	0,015	1
Mob4	0,647145	0,02774	0,517	0,565	1
Nek7	0,647519	0,00929	0,545	0,591	1
4930444P:	0,647604	0,001556	0,004	0,002	1
Rassf5	0,647604	0,001556	0,004	0,002	1
Nek2	0,647604	0,001556	0,004	0,002	1
Gm13479	0,647604	0,001556	0,004	0,002	1
Olfr1156	0,647604	0,001556	0,004	0,002	1
Syt13	0,647604	0,001556	0,004	0,002	1
Pax6	0,647604	0,001556	0,004	0,002	1
Il1b	0,647604	0,001556	0,004	0,002	1
Cfap61	0,647604	0,001556	0,004	0,002	1
Trib3	0,647604	0,001556	0,004	0,002	1
Tspyl3	0,647604	0,001556	0,004	0,002	1
Cpne1	0,647604	0,001556	0,004	0,002	1
Gm27206	0,647604	0,001556	0,004	0,002	1
Pabpc1l	0,647604	0,001556	0,004	0,002	1
Nfatc2	0,647604	0,001556	0,004	0,002	1
Cdh4	0,647604	0,001556	0,004	0,002	1
Srpx	0,647604	0,001556	0,004	0,002	1
Gpr174	0,647604	0,001556	0,004	0,002	1
Tmod4	0,647604	0,001556	0,004	0,002	1
Tifa	0,647604	0,001556	0,004	0,002	1
Gm40155	0,647604	0,001556	0,004	0,002	1
Mpl	0,647604	0,001556	0,004	0,002	1
Heyl	0,647604	0,001556	0,004	0,002	1
Rhd	0,647604	0,001556	0,004	0,002	1
Zfp993	0,647604	0,001556	0,004	0,002	1
Fbxl13	0,647604	0,001556	0,004	0,002	1

Jakmip1	0,647604	0,001556	0,004	0,002	1
Cxcl10	0,647604	0,001556	0,004	0,002	1
Gm26826	0,647604	0,001556	0,004	0,002	1
Scnn1a	0,647604	0,001556	0,004	0,002	1
Tmem145	0,647604	0,001556	0,004	0,002	1
G630030J(	0,647604	0,001556	0,004	0,002	1
Gm15396	0,647604	0,001556	0,004	0,002	1
Gm15635	0,647604	0,001556	0,004	0,002	1
Gm35082	0,647604	0,001556	0,004	0,002	1
Olfr633	0,647604	0,001556	0,004	0,002	1
Olfr675	0,647604	0,001556	0,004	0,002	1
Olfr715b	0,647604	0,001556	0,004	0,002	1
Gm44773	0,647604	0,001556	0,004	0,002	1
Hebp2	0,647604	0,001556	0,004	0,002	1
Gm48249	0,647604	0,001556	0,004	0,002	1
Tmem200a	0,647604	0,001556	0,004	0,002	1
Pkib	0,647604	0,001556	0,004	0,002	1
Cdk1	0,647604	0,001556	0,004	0,002	1
Ptprb	0,647604	0,001556	0,004	0,002	1
Stat6	0,647604	0,001556	0,004	0,002	1
Rdh9	0,647604	0,001556	0,004	0,002	1
AC117232.	0,647604	0,001556	0,004	0,002	1
Vmn2r87	0,647604	0,001556	0,004	0,002	1
Col4a2	0,647604	0,001556	0,004	0,002	1
Ank1	0,647604	0,001556	0,004	0,002	1
Gm26721	0,647604	0,001556	0,004	0,002	1
Gm21817	0,647604	0,001556	0,004	0,002	1
Cdkn3	0,647604	0,001556	0,004	0,002	1
Rec8	0,647604	0,001556	0,004	0,002	1
4930444M	0,647604	0,001556	0,004	0,002	1
Scel	0,647604	0,001556	0,004	0,002	1
Gm35835	0,647604	0,001556	0,004	0,002	1
Exph5	0,647604	0,001556	0,004	0,002	1
Gm38150	0,647604	0,001556	0,004	0,002	1
Gfpt2	0,647604	0,001556	0,004	0,002	1
Olfr30	0,647604	0,001556	0,004	0,002	1
Mycbpap	0,647604	0,001556	0,004	0,002	1
Plcd3	0,647604	0,001556	0,004	0,002	1
Hist1h2bf	0,647604	0,001556	0,004	0,002	1
Gm47061	0,647604	0,001556	0,004	0,002	1
Fbp1	0,647604	0,001556	0,004	0,002	1
Gm4117	0,647604	0,001556	0,004	0,002	1
Dtnbos	0,647604	0,001556	0,004	0,002	1
AC163633.	0,647604	0,001556	0,004	0,002	1
AC164424.	0,647604	0,001556	0,004	0,002	1
Gm35558.	0,647604	0,001556	0,004	0,002	1
Ptger4	0,647604	0,001556	0,004	0,002	1
Baiap2l2	0,647604	0,001556	0,004	0,002	1
Pced1b	0,647604	0,001556	0,004	0,002	1
Hoxc10	0,647604	0,001556	0,004	0,002	1
Hoxc5	0,647604	0,001556	0,004	0,002	1
AC161607.	0,647604	0,001556	0,004	0,002	1

Abi3bp	0,647604	0,001556	0,004	0,002	1
Tmem30c	0,647604	0,001556	0,004	0,002	1
Cbr3	0,647604	0,001556	0,004	0,002	1
2310043M	0,647604	0,001556	0,004	0,002	1
Tcf19	0,647604	0,001556	0,004	0,002	1
Alk	0,647604	0,001556	0,004	0,002	1
Pcdhb2	0,647604	0,001556	0,004	0,002	1
Gm26742	0,647604	0,001556	0,004	0,002	1
Stambpl1	0,647604	0,001556	0,004	0,002	1
AW011738	0,647604	0,004922	0,004	0,002	1
Ntrk3	0,647604	0,003102	0,004	0,002	1
Nudt15	0,647755	0,000445	0,042	0,049	1
Atp5a1	0,647911	0,010372	0,98	0,987	1
Emilin2	0,648005	0,049458	0,461	0,487	1
Epm2a	0,648051	0,016176	0,125	0,116	1
Errfi1	0,648525	0,064169	0,62	0,648	1
Gm20696	0,64916	0,011652	0,037	0,032	1
Gdf9	0,649196	0,006749	0,046	0,04	1
A030005L1	0,649239	-0,00055	0,004	0,002	1
Xlr4a	0,649239	-0,00055	0,004	0,002	1
Bcar3	0,649239	-0,00055	0,004	0,002	1
Mypopos	0,649239	-0,00055	0,004	0,002	1
Gm32913	0,649239	-0,00055	0,004	0,002	1
Gata6	0,649239	-0,00265	0,004	0,002	1
Cyp7b1	0,649239	-0,00474	0,004	0,002	1
Ntng1	0,649239	-0,00474	0,004	0,002	1
Gm47320	0,649456	0,008913	0,018	0,015	1
Nutf2-ps1	0,649456	0,010704	0,018	0,015	1
Zfp280c	0,649527	0,058825	0,461	0,527	1
Ppp1r2	0,649901	0,063727	0,561	0,622	1
Suco	0,650859	0,022438	0,411	0,451	1
Paxip1	0,651156	0,037584	0,202	0,196	1
Unc13b	0,651368	0,013168	0,068	0,061	1
9130221H	0,651825	0,01018	0,037	0,032	1
Alpk2	0,651879	0,014275	0,018	0,015	1
Gm16174	0,652118	0,011039	0,028	0,023	1
Kctd11	0,652458	0,026342	0,136	0,129	1
Arl15	0,65281	0,011652	0,037	0,032	1
Ccdc136	0,653037	0,024346	0,25	0,27	1
Mcmbp	0,653212	0,045344	0,495	0,511	1
Nectin1	0,653266	0,044496	0,543	0,561	1
D7Ertd443	0,653879	0,009711	0,593	0,656	1
Il18bp	0,654082	0,014867	0,028	0,023	1
E530011L2	0,65411	0,00097	0,039	0,044	1
Gfra1	0,654211	-0,00583	0,145	0,156	1
AC135964.	0,654305	0,005323	0,018	0,015	1
Arhgap12	0,654453	0,03803	0,259	0,285	1
Cir1	0,65493	0,011773	0,607	0,673	1
Far1	0,655076	0,045987	0,732	0,738	1
Zfp280d	0,655173	0,075861	0,519	0,563	1
Gm26606	0,655351	0,005043	0,018	0,015	1
AC163720.	0,655643	0,012256	0,048	0,042	1

Nop56	0,65594	0,077259	0,49	0,523	1
Gm26798	0,65622	0,001921	0,04	0,046	1
Hunk	0,65626	0,008842	0,044	0,051	1
Slc23a1	0,656345	0,00516	0,028	0,023	1
Xylt1	0,656468	0,013405	0,037	0,032	1
Eif2ak3	0,656563	0,011177	0,2	0,215	1
Hipk1	0,656746	0,057724	0,426	0,487	1
Suv39h1	0,656895	0,025578	0,16	0,175	1
Nfkbia	0,657019	0,064163	0,349	0,388	1
Atp13a3	0,657316	0,051477	0,317	0,319	1
Gm43796	0,657796	0,007459	0,092	0,101	1
Rasgef1c	0,657838	0,020386	0,039	0,034	1
Ankrd50	0,657951	0,023208	0,163	0,156	1
Jup	0,658339	0,010083	0,837	0,873	1
Nckap5l	0,658349	-0,00026	0,039	0,044	1
2410089E(	0,658441	0,031216	0,323	0,352	1
Bcl3	0,659027	-0,00466	0,086	0,095	1
Lmo4	0,659786	0,037284	0,358	0,388	1
Tshz3	0,66057	0,00297	0,018	0,015	1
Syde2	0,660599	0,018456	0,083	0,076	1
9530034E1	0,661231	0,010234	0,048	0,042	1
Zcchc4	0,661253	0,002907	0,116	0,127	1
Mapre2	0,661346	0,001728	0,91	0,884	1
Casp6	0,661429	0,005715	0,028	0,023	1
Fpgs	0,661811	-0,0044	0,103	0,112	1
Dnajb2	0,661988	0,015757	1	0,994	1
Orc5	0,662526	0,015312	0,176	0,192	1
Usp6nl	0,663223	0,022196	0,294	0,319	1
Bak1	0,663294	-0,00807	0,242	0,257	1
Gm10069	0,663305	0,004731	0,039	0,044	1
Faap100	0,663443	0,047798	0,226	0,224	1
Dync2h1	0,663574	0,028954	0,187	0,179	1
Col11a2	0,663593	0,041715	0,233	0,255	1
Arrdc1	0,663609	0,007939	0,048	0,042	1
Brpf3	0,663736	0,023512	0,108	0,101	1
Mtx3	0,663833	0,015581	0,158	0,171	1
Lins1	0,664185	0,015688	0,083	0,076	1
Ninl	0,664274	0,002842	0,028	0,023	1
Spata31d1	0,664549	0,035911	0,178	0,173	1
Prune1	0,664669	0,001895	0,106	0,116	1
Zfp128	0,66469	0,010844	0,059	0,053	1
Smarcd1	0,665192	0,047549	0,831	0,88	1
Zfp865	0,665441	0,036477	0,194	0,215	1
Cacna1c	0,66547	0,018646	0,039	0,034	1
Zcchc2	0,665496	0,008656	0,147	0,16	1
Naa15	0,665534	0,057169	0,571	0,629	1
Hspa12a	0,665674	-0,00326	0,035	0,04	1
Gdap1l1	0,665727	-0,00052	0,037	0,042	1
Tbc1d31	0,666706	0,007109	0,11	0,12	1
Zkscan17	0,666836	0,016194	0,097	0,108	1
Gabpa	0,6678	0,057013	0,235	0,234	1
Mapk6	0,668379	0,032985	0,371	0,411	1

Bri3bp	0,668717	0,013555	0,149	0,162	1
Rbm27	0,66902	0,031072	0,431	0,481	1
Sh3bgrl2	0,669778	-0,01553	0,09	0,097	1
Zfp661	0,670008	-0,00478	0,033	0,038	1
Abcc4	0,670043	-0,0058	0,033	0,038	1
Gramd4	0,670195	0,023033	0,136	0,129	1
Zfp59	0,670262	-0,00378	0,033	0,038	1
Rap1gap	0,670283	0,015438	0,039	0,034	1
Kif22	0,670667	-0,00026	0,035	0,04	1
Dcaf10	0,671119	0,03442	0,172	0,167	1
Plaa	0,671208	0,020935	0,275	0,3	1
Sh2d3c	0,671324	0,011579	0,011	0,008	1
Med26	0,672018	-0,00267	0,081	0,089	1
Vegfb	0,672515	0,096932	0,47	0,5	1
Fam72a	0,673147	0,007975	0,011	0,008	1
Disp2	0,673147	0,011579	0,011	0,008	1
Pcdh8	0,673147	0,009779	0,011	0,008	1
Leng9	0,673698	0,001934	0,123	0,133	1
Fbxw7	0,673943	0,030874	0,16	0,175	1
Ssbp2	0,67395	0,026848	0,222	0,241	1
Chaf1a	0,674106	0,011367	0,039	0,034	1
Lats1	0,674873	0,054748	0,36	0,365	1
Lyn	0,674972	0,006169	0,011	0,008	1
Lin7b	0,674972	0,006169	0,011	0,008	1
1700048O	0,674972	0,006169	0,011	0,008	1
Gm10785	0,674972	0,006169	0,011	0,008	1
Olfr90	0,674972	0,006169	0,011	0,008	1
St6galnac2	0,674972	0,013376	0,011	0,008	1
Rassf1	0,675061	0,067355	0,349	0,39	1
Gm29417	0,675076	0,011082	0,039	0,034	1
Gna11	0,675262	0,025865	0,758	0,808	1
AY074887	0,67543	0,007689	0,011	0,008	1
Gm49322	0,67547	0,017889	0,095	0,089	1
Ints3	0,675483	0,036586	0,347	0,38	1
Gga3	0,675567	0,03874	0,194	0,213	1
Gm29994	0,675887	0,005886	0,011	0,008	1
Gm27200	0,675897	-0,00581	0,097	0,105	1
Asah1	0,676259	0,01555	0,527	0,589	1
Bcl9l	0,676284	0,004979	0,437	0,464	1
March7	0,676604	0,084031	0,521	0,557	1
Mapre3	0,676685	0,031302	0,561	0,622	1
March9	0,676716	0,0064	0,064	0,072	1
Gm26690	0,676799	0,004359	0,011	0,008	1
AC154760.	0,676799	0,004359	0,011	0,008	1
Gm7628	0,676799	0,004359	0,011	0,008	1
Lrrc14b	0,676799	0,004359	0,011	0,008	1
3110070M	0,676799	0,004359	0,011	0,008	1
Coro2b	0,676986	0,062102	0,448	0,473	1
Rab40c	0,677049	0,01372	0,473	0,523	1
Cdk13	0,677619	0,080483	0,424	0,451	1
Smg5	0,67762	0,039987	0,339	0,342	1
Eps15l1	0,677638	0,019139	0,209	0,23	1

Zmynd11	0,67765	0,041989	0,673	0,692	1
Cxxc4	0,677715	0,004079	0,011	0,008	1
Marf1	0,677958	0,056379	0,644	0,688	1
Ppan	0,67834	0,033122	0,154	0,171	1
Eif4e3	0,678372	0,027217	0,222	0,215	1
Tbc1d8	0,678627	0,002546	0,011	0,008	1
Kcnj13	0,678627	0,002546	0,011	0,008	1
Thsd7b	0,678627	0,002546	0,011	0,008	1
Gm5862	0,678627	0,002546	0,011	0,008	1
Fam196a	0,678627	0,002546	0,011	0,008	1
Gm26674	0,678627	0,002546	0,011	0,008	1
Pate2	0,678627	0,002546	0,011	0,008	1
Tlcd2	0,678627	0,002546	0,011	0,008	1
Rhebl1	0,678679	0,00851	0,084	0,093	1
Dpy19l3	0,679087	0,005886	0,011	0,008	1
Col7a1	0,679087	0,004079	0,011	0,008	1
Fut10	0,679104	0,014826	0,174	0,167	1
Lrrc75b	0,679228	0,004895	0,105	0,114	1
Gm21663	0,679545	0,002269	0,011	0,008	1
Osbpl3	0,679545	0,002269	0,011	0,008	1
Gmeb1	0,679613	-0,01237	0,167	0,175	1
Ndst2	0,680503	0,004816	0,081	0,074	1
Raph1	0,680787	0,015863	0,128	0,12	1
Arhgap25	0,68098	0,012807	0,029	0,025	1
B130011K(	0,681376	-0,00163	0,011	0,008	1
Cib2	0,681575	0,015757	0,02	0,017	1
Socs4	0,681739	0,015906	0,257	0,274	1
Zfp518b	0,682004	0,000614	0,059	0,065	1
Rcc2	0,682542	0,068389	0,189	0,188	1
Zfp229	0,683202	0,002302	0,033	0,038	1
Cep85	0,683256	0,006618	0,152	0,165	1
Rapsn	0,684125	-0,00136	0,057	0,063	1
4931440F1	0,684129	-0,00163	0,011	0,008	1
Schip1	0,68421	0,049953	0,339	0,384	1
Ipmk	0,685146	0,031079	0,229	0,251	1
Slc25a30	0,685487	0,04133	0,198	0,196	1
Fancg	0,685641	-0,00486	0,094	0,101	1
Fyttd1	0,685681	0,051676	0,521	0,578	1
Olfr889	0,685692	0,015448	0,029	0,025	1
Atl3	0,685992	0,036176	0,36	0,397	1
Ift122	0,686361	0,011747	0,161	0,152	1
Kcnh7	0,686924	0,010414	0,02	0,017	1
Fam214b	0,686961	0,053634	0,262	0,266	1
Gm40117	0,686967	-0,00283	0,055	0,061	1
Sall2	0,687004	0,018596	0,051	0,046	1
Rab32	0,687617	0,007493	0,029	0,025	1
Abcf1	0,687781	0,028279	0,699	0,755	1
Slc30a5	0,687846	0,038103	0,301	0,297	1
Mycbp2	0,688001	0,030706	0,894	0,897	1
Elavl1	0,688341	0,003872	0,589	0,631	1
Zfp316	0,688537	0,023554	0,191	0,184	1
Jade3	0,688709	0,0015	0,031	0,036	1

Sipa1l2	0,688738	0,035302	0,242	0,238	1
Slc6a1	0,688948	0,039454	0,626	0,641	1
Dct	0,688965	0,002985	0,031	0,036	1
Sh3bp2	0,689054	0,01917	0,163	0,156	1
Tox4	0,689374	0,042112	0,44	0,492	1
Enpp5	0,689582	0,028975	0,91	0,935	1
Gm37065	0,689605	0,008627	0,02	0,017	1
AI182371	0,689605	0,008627	0,02	0,017	1
Arnt	0,689632	0,027807	0,345	0,371	1
Engase	0,689832	0,014572	0,04	0,036	1
Gm45902	0,690122	0,013706	0,037	0,042	1
Hmgn1	0,690144	0,015941	0,56	0,601	1
Bglap2	0,690282	0,014867	0,02	0,017	1
Herc6	0,690861	0,053028	0,112	0,108	1
Smoc1	0,691289	0,010574	0,108	0,118	1
Pomc	0,692234	0,005823	0,031	0,036	1
Zfp7	0,692234	0,005823	0,031	0,036	1
Trpm4	0,692234	0,002302	0,031	0,036	1
Gm9833	0,692289	0,006836	0,02	0,017	1
Ccdc78	0,692289	0,006836	0,02	0,017	1
Sbk3	0,692289	0,010414	0,02	0,017	1
Srek1	0,692404	0,029735	0,642	0,696	1
Slc17a5	0,69285	0,038574	0,266	0,295	1
Znrf2	0,693093	0,029333	0,125	0,118	1
Pard3	0,693278	0,050105	0,618	0,635	1
Hdac9	0,694269	0,037352	0,108	0,103	1
Ercc2	0,69439	0,005072	0,149	0,16	1
Adcy10	0,694976	0,005043	0,02	0,017	1
Creb5	0,695553	0,048489	0,855	0,859	1
Tnpo2	0,695985	0,038829	0,29	0,291	1
BC043934	0,69599	0,004764	0,02	0,017	1
Cdh2	0,696261	0,047559	0,747	0,791	1
Prelid3a	0,69638	0,020206	0,114	0,108	1
AC152827.	0,696544	0,004064	0,031	0,036	1
M6pr	0,696974	0,01959	0,859	0,869	1
Rnf225	0,697101	0,018302	0,051	0,046	1
Peg10	0,697305	-0,00642	0,239	0,249	1
Cdv3	0,697351	0,025623	0,772	0,774	1
Wnt5b	0,697371	0,004064	0,029	0,034	1
Phf23	0,69739	0,05912	0,283	0,287	1
Tmem64	0,69779	0,009428	0,083	0,091	1
Mon1a	0,698101	0,022223	0,152	0,167	1
Erich1	0,698449	0,030374	0,189	0,184	1
Mthfr	0,698683	0,00297	0,02	0,017	1
Wapl	0,699237	0,056956	0,628	0,669	1
Phactr1	0,699838	0,02025	0,877	0,914	1
A730011C1	0,700374	-0,00143	0,02	0,017	1
mt-Nd5	0,700389	0,035513	0,996	0,996	1
Nudt8.1	0,701023	0,00107	0,029	0,034	1
Rai1	0,701116	0,025341	0,128	0,122	1
R3hdm2	0,701243	0,030652	0,574	0,637	1
Erc1	0,701426	0,03218	0,167	0,162	1

Ankrd37	0,702158	0,007205	0,057	0,063	1
Atp6v1b2	0,703149	0,025658	0,604	0,662	1
Arhgef19	0,703159	0,012092	0,073	0,068	1
Diaph1	0,703577	-0,00329	0,187	0,196	1
Zfp689	0,703691	-0,00181	0,05	0,055	1
Serp2	0,70412	0,045437	0,156	0,152	1
Cited2	0,70414	0,02884	0,066	0,061	1
Naa25	0,704276	0,010051	0,163	0,177	1
Exoc1	0,704427	0,048971	0,396	0,449	1
Zfx	0,705023	0,035268	0,29	0,289	1
Scai	0,705244	0,034503	0,2	0,219	1
Spast	0,705326	0,031071	0,462	0,517	1
Daxx	0,705668	0,032993	0,218	0,215	1
Paqr9	0,705751	0,006099	0,029	0,034	1
Klhl35	0,705823	0,021135	0,055	0,051	1
Sugct	0,706209	0,005823	0,04	0,036	1
Bambi	0,70688	0,021772	0,075	0,07	1
Rps24	0,706965	0,032863	0,998	0,998	1
Sde2	0,706968	0,024849	0,387	0,424	1
Usp33	0,707127	0,049664	0,356	0,369	1
Mtr	0,707348	0,029146	0,068	0,063	1
Irs2	0,70772	0,019446	0,785	0,791	1
Otud3	0,708252	-0,004	0,026	0,03	1
Rab11fip3	0,708413	0,041144	0,275	0,27	1
Mia3	0,708937	0,030502	0,486	0,53	1
B3gnt2	0,709023	0,010751	0,031	0,027	1
Arhgap1	0,709244	0,0051	0,2	0,213	1
Amy1	0,709699	0,01351	0,123	0,116	1
Tef	0,710898	0,042569	0,561	0,599	1
Ttc28	0,71092	0,01959	0,15	0,162	1
Whrn	0,71093	0,002571	0,028	0,032	1
Nfya	0,711744	0,029161	0,2	0,217	1
Exoc6b	0,711748	0,009199	0,739	0,774	1
Pcgf3	0,712276	0,058751	0,317	0,329	1
Cdk20	0,712416	0,016003	0,053	0,049	1
Hnrnpc	0,712473	0,016308	0,938	0,956	1
Zbtb41	0,71252	0,035482	0,259	0,255	1
Pelp1	0,712526	0,013083	0,18	0,194	1
Zswim5	0,712549	0,008983	0,031	0,027	1
Tmem202	0,712675	0,003522	0,04	0,036	1
Gm9938	0,712809	-0,00555	0,048	0,053	1
Tcf7l2	0,713386	0,066205	0,288	0,293	1
Cdk17	0,713427	0,028813	0,378	0,42	1
Trmt2b	0,714066	-0,0125	0,189	0,196	1
Mark2	0,714217	0,019042	0,495	0,544	1
Gm9774	0,714427	0,000183	0,05	0,055	1
Gm26981	0,714454	0,018945	0,028	0,032	1
Nkapl	0,714647	0,016313	0,031	0,036	1
Gtdc1	0,715271	0,031173	0,239	0,234	1
Kctd1	0,716117	-0,00017	0,026	0,03	1
Trim21	0,716553	0,007475	0,05	0,055	1
Adnp2	0,716725	0,009652	0,138	0,148	1

Zbtb1	0,716871	0,049951	0,215	0,213	1
Pan3	0,717098	0,056754	0,385	0,428	1
Zdhhc8	0,717336	0,022301	0,264	0,287	1
Lipa	0,717528	0,02367	0,517	0,57	1
Dennd4c	0,717912	0,019073	0,176	0,169	1
Telo2	0,717998	0,017093	0,165	0,177	1
Zfp324	0,718699	-0,00156	0,048	0,053	1
C330007P(	0,718939	0,032962	0,479	0,53	1
Gnpda1	0,719199	0,004101	0,176	0,188	1
CAAA0114:	0,719618	0,007212	0,031	0,027	1
Gm16287	0,71965	0,009332	0,042	0,038	1
BC017158	0,719847	0,003124	0,05	0,055	1
Gm49085	0,720634	0,004885	0,026	0,03	1
Rnf169	0,720635	0,01917	0,154	0,167	1
Med16	0,72189	0,062306	0,235	0,236	1
Lin9	0,722091	0,007858	0,031	0,027	1
Arc	0,722219	0,117873	0,341	0,382	1
Casz1	0,722505	-0,00222	0,024	0,027	1
Gm7361	0,722505	-0,00222	0,024	0,027	1
Dennd1b	0,722798	-0,0007	0,024	0,027	1
Arl5a	0,722837	0,012914	0,505	0,546	1
Flcn	0,722908	0,035709	0,283	0,31	1
Uhrf2	0,723017	0,065685	0,505	0,549	1
Myzap	0,723058	-0,00654	0,05	0,044	1
Sfxn4	0,723069	0,020692	0,079	0,074	1
Rdh11	0,723373	0,061706	0,393	0,449	1
Baz1a	0,723735	0,005823	0,031	0,027	1
Ralgps1	0,723765	0,066173	0,549	0,591	1
Baz2a	0,724951	0,021659	0,332	0,35	1
Cipc	0,725613	0,007885	0,809	0,861	1
Ugcg	0,725667	0,06938	0,191	0,192	1
4930518I1	0,725761	0,005275	0,042	0,038	1
Trak1	0,726338	0,015877	0,139	0,133	1
Gpd1l	0,726495	0,01624	0,215	0,228	1
Phka1	0,727169	0,007364	0,112	0,12	1
Poglut1	0,727323	0,031644	0,393	0,432	1
Adprh	0,727913	0,015009	0,611	0,665	1
Wipi2	0,728096	0,031654	0,561	0,605	1
Hnrnph3	0,728214	0,034139	0,67	0,709	1
Pias2	0,728259	0,048228	0,352	0,361	1
Cobl	0,728363	0,090366	0,376	0,399	1
Usp36	0,728761	0,024725	0,237	0,253	1
Pdlim5	0,728805	0,018461	0,294	0,319	1
Arhgef6	0,728871	0,007689	0,013	0,011	1
Asf1b	0,728871	0,011286	0,013	0,011	1
Gm4285	0,728885	-7,7E-05	0,048	0,053	1
Zfp654	0,728913	0,017797	0,189	0,181	1
Zfp300	0,729123	0,010888	0,048	0,053	1
Gm15462	0,729301	0,009201	0,013	0,011	1
Rad51c	0,729301	0,009201	0,013	0,011	1
Brca1	0,729329	0,013391	0,022	0,019	1
Rgl2	0,729475	0,017575	0,178	0,171	1

Mapk1	0,72949	0,029954	0,631	0,658	1
AC123870.	0,729579	-0,00374	0,022	0,025	1
Samd15	0,72973	0,007404	0,013	0,011	1
Kdm4a	0,729813	0,029769	0,376	0,405	1
Carmil3	0,729823	-5,8E-05	0,108	0,116	1
Slc29a3	0,730092	0,043382	0,418	0,462	1
9430060I0	0,730123	0,001878	0,024	0,027	1
Gm37459	0,730123	0,001878	0,024	0,027	1
Akap12	0,730128	0,030767	0,024	0,027	1
Tob1	0,730355	0,023631	0,769	0,783	1
Map3k2	0,73047	0,049035	0,35	0,39	1
Washc5	0,730857	0,031333	0,25	0,27	1
Pcdha4	0,731016	0,005886	0,013	0,011	1
A830035O	0,731016	0,009489	0,013	0,011	1
Atp6ap2	0,731143	0,034399	0,961	0,943	1
Bdp1	0,731193	0,01701	0,336	0,371	1
Gm16552	0,731447	0,010994	0,013	0,011	1
Anks3	0,731576	0,026076	0,235	0,251	1
Tor3a	0,731605	0,004764	0,022	0,019	1
Nfkb1	0,731837	0,034503	0,2	0,217	1
Ftx	0,73276	0,017851	0,09	0,084	1
Smcr8	0,732867	0,028886	0,143	0,139	1
Pnp2	0,733163	0,004079	0,013	0,011	1
Olfr93	0,733163	0,004079	0,013	0,011	1
Kcnab2	0,733238	-0,00143	0,022	0,025	1
Asns	0,733238	-0,00143	0,022	0,025	1
Kank3	0,733238	-0,00143	0,022	0,025	1
Cd14	0,733595	0,005604	0,013	0,011	1
Zzz3	0,733619	0,045207	0,556	0,586	1
Rac1	0,73375	0,024124	0,996	0,989	1
Zbtb48	0,734189	0,008172	0,07	0,076	1
Wdsub1	0,734827	0,010976	0,209	0,222	1
Map7d1	0,734957	0,029056	0,987	0,994	1
Cog2	0,735074	0,008783	0,211	0,224	1
Actr1a	0,735215	0,015836	0,833	0,827	1
Ccdc169	0,735313	0,002269	0,013	0,011	1
Stx1a	0,735313	0,002269	0,013	0,011	1
Adam32	0,735313	0,002269	0,013	0,011	1
B4galnt1	0,735503	0,006302	0,053	0,049	1
Mob1b	0,735784	0,012054	0,16	0,171	1
Gm15706	0,735887	0,022429	0,033	0,03	1
Fgf1	0,736025	0,030994	0,631	0,665	1
Hps5	0,736147	0,04105	0,215	0,213	1
Mras	0,73617	0,002552	0,123	0,131	1
4930404N	0,736176	0,001993	0,013	0,011	1
A930029G	0,736176	0,001993	0,013	0,011	1
Ubr1	0,736853	0,064249	0,457	0,485	1
Adam4	0,737531	0,003659	0,022	0,025	1
Tdp1	0,737936	0,016671	0,13	0,141	1
Hnrnpa1	0,738226	0,024551	0,835	0,819	1
Gm6169	0,738328	0,000183	0,013	0,011	1
Fn1	0,738411	0,013706	0,044	0,04	1

Josd1	0,738568	0,001266	0,16	0,169	1
Ppp1r21	0,738654	0,005342	0,679	0,717	1
Chdh	0,738737	0,023747	0,229	0,245	1
Sgtb	0,739051	0,002185	0,046	0,051	1
Sorl1	0,739127	0,002259	0,681	0,711	1
N4bp1	0,739571	0,027829	0,29	0,314	1
Gm43848	0,739898	0,013816	0,154	0,165	1
Zmym2	0,740513	0,059704	0,371	0,382	1
Ucp2	0,74062	-0,01142	0,02	0,023	1
Cog3	0,741065	0,025391	0,217	0,211	1
Fut9	0,741162	0,001449	0,044	0,049	1
Xpo7	0,741236	0,042743	0,372	0,42	1
Fnbp1	0,741597	0,001511	0,996	0,998	1
Anapc4	0,742482	0,031401	0,312	0,34	1
Accs	0,743348	0,011939	0,033	0,03	1
4930524J0	0,744463	-0,00116	0,02	0,023	1
4933433G	0,744467	-0,00143	0,02	0,023	1
Gm20517	0,744467	-0,00143	0,02	0,023	1
Edem3	0,744739	0,044384	0,231	0,232	1
Virma	0,744904	0,024167	0,286	0,308	1
Bora	0,745696	0,002478	0,066	0,072	1
Srgap3	0,745792	0,039332	0,442	0,494	1
Wwp2	0,745896	0,058322	0,349	0,354	1
Ezh1	0,745995	0,037159	0,479	0,534	1
Eef1a2	0,746009	0,025726	0,024	0,021	1
Ano6	0,746009	0,020458	0,024	0,021	1
Ifnar1	0,746115	0,016192	0,235	0,251	1
Nmu	0,747058	0,013695	0,022	0,025	1
Gm4737	0,747111	0,014286	0,057	0,053	1
Med12	0,747716	0,027876	0,185	0,179	1
BC051142	0,747975	0,007493	0,022	0,025	1
Gm10638	0,747996	0,000632	0,02	0,023	1
Gm10244	0,748	0,000362	0,02	0,023	1
Atp7a	0,748046	0,015581	0,165	0,177	1
Dgka	0,748164	0,006375	0,033	0,03	1
Gm16052	0,748321	0,003934	0,02	0,023	1
Gpam	0,74859	0,013994	0,046	0,042	1
Mprip	0,748758	0,05052	0,644	0,703	1
Lynx1	0,74915	0,020458	0,024	0,021	1
Cln6	0,749486	0,00097	0,042	0,046	1
Fignl1	0,749491	-0,00476	0,018	0,021	1
Aifm2	0,749526	0,005909	0,105	0,112	1
Onecut2	0,750314	0,093445	0,15	0,15	1
Zc3h12b	0,750436	-0,00781	0,04	0,044	1
Dab2ip	0,750602	0,040518	0,387	0,422	1
Ssh2	0,751191	0,044614	0,45	0,47	1
Tmem136	0,751539	0,001878	0,02	0,023	1
Sh3bp5	0,751552	0,013024	0,894	0,869	1
Pcbp1	0,752083	0,019226	0,943	0,932	1
Stk38l	0,752252	0,017041	0,198	0,192	1
Armcx2	0,752261	0,02938	0,171	0,186	1
Rasa2	0,752284	0,090993	0,24	0,249	1

4930550C1	0,752863	-0,00296	0,018	0,021	1
Gm26563	0,752863	-0,00296	0,018	0,021	1
Gm15601	0,752866	-0,00322	0,018	0,021	1
Fdxacb1	0,753114	0,011208	0,139	0,15	1
Ppp1r17	0,753203	9,42E-05	0,018	0,021	1
Gm15688	0,753248	0,010612	0,068	0,063	1
Dhx15	0,753305	0,053895	0,514	0,544	1
2810403A(	0,753484	0,025601	0,288	0,31	1
Lhfpl4	0,753643	0,025424	0,167	0,162	1
Tubgcp4	0,753779	0,018017	0,239	0,255	1
Capn2	0,753881	0,040365	0,486	0,551	1
Nub1	0,753893	0,025456	0,594	0,637	1
Pprc1	0,754196	0,010106	0,13	0,139	1
Shisa8	0,754474	0,031602	0,266	0,283	1
2700081O1	0,754513	0,000445	0,042	0,046	1
Acss2	0,754641	0,066674	0,422	0,464	1
Lrrc75a	0,755076	0,007774	0,02	0,023	1
Gm26747	0,755443	0,011616	0,024	0,021	1
Ralbp1	0,755469	0,047878	0,782	0,8	1
Tmed8	0,75553	0,018781	0,136	0,131	1
CT025619.	0,755706	0,029353	0,222	0,238	1
Atp5b	0,755894	-0,00073	0,998	0,996	1
Gucd1	0,755997	0,005447	0,086	0,093	1
Plek	0,756235	-0,0009	0,018	0,021	1
Chtf18	0,756241	-0,00143	0,018	0,021	1
Chd2	0,756312	0,051643	0,394	0,437	1
Tep1	0,756421	0,018646	0,086	0,093	1
Trmt1	0,756504	0,044802	0,273	0,3	1
Farp2	0,756743	0,001003	0,101	0,108	1
Tmem87a	0,756811	0,042056	0,382	0,428	1
Numbl	0,756835	0,021045	0,11	0,105	1
Cyld	0,756886	0,017824	0,541	0,578	1
Bahcc1	0,757707	0,028731	0,13	0,141	1
Psmd1	0,758079	0,028548	0,73	0,741	1
Ccnt1	0,758192	0,066106	0,442	0,513	1
Ccdc159	0,758385	0,000445	0,042	0,046	1
Dlc1	0,759334	-0,00481	0,04	0,044	1
Snhg11	0,759342	0,094948	0,116	0,127	1
Dglucy	0,759616	0,000902	0,018	0,021	1
Zfp964	0,759619	0,000632	0,018	0,021	1
Usp22	0,759682	0,024333	0,853	0,833	1
Cluh	0,759748	0,023383	0,174	0,171	1
4930509G1	0,759805	0,007389	0,046	0,051	1
Fkbp1a	0,759861	0,046826	0,987	0,979	1
Egfl8	0,759957	0,013098	0,018	0,021	1
Gm38642	0,760056	0,01051	0,187	0,198	1
Plekha5	0,7604	0,02364	0,275	0,297	1
Rnf43	0,760473	0,056718	0,213	0,23	1
Plxnb2	0,760496	0,007493	0,024	0,021	1
Cd164	0,760719	0,044888	0,492	0,519	1
Atpif1	0,760784	0,03984	0,708	0,736	1
Il1rap	0,761056	0,063184	0,868	0,876	1

Zfp445	0,762124	0,006632	0,657	0,681	1
Tmem91	0,762176	0,014867	0,02	0,023	1
Ak5	0,762213	-0,0045	0,017	0,019	1
Ttc26	0,762388	0,007774	0,024	0,021	1
Gm47438	0,762388	0,007774	0,024	0,021	1
Mrps6	0,762498	0,060144	0,558	0,608	1
Flna	0,7626	0,013521	0,13	0,124	1
C78859	0,762814	-0,0053	0,04	0,044	1
Usp2	0,762859	0,027709	0,279	0,297	1
Rab9b	0,763001	0,002696	0,018	0,021	1
Nfxl1	0,763243	0,010408	0,139	0,133	1
Gm13920	0,763343	0,004209	0,018	0,021	1
Pcbp3	0,76335	0,006495	0,081	0,076	1
Mdn1	0,763377	0,040924	0,266	0,268	1
Tedc1	0,76354	0,008842	0,057	0,053	1
Lad1	0,763647	-0,00348	0,017	0,019	1
D230025D	0,763865	0,056094	0,262	0,268	1
Trip11	0,764348	0,005338	0,771	0,757	1
Casc3	0,764349	0,056688	0,394	0,414	1
Slc25a22	0,764417	0,018693	0,095	0,091	1
Hist1h2ap	0,764913	0,004486	0,024	0,021	1
Zswim3	0,765421	-0,00243	0,017	0,019	1
Lrrc27	0,765421	-0,00243	0,017	0,019	1
Gm6793	0,766386	0,010362	0,006	0,004	1
Pdp2	0,767066	0,030833	0,132	0,129	1
Fam212b	0,767121	0,026812	0,185	0,181	1
Mcub	0,767732	0,004922	0,006	0,004	1
1810019N	0,767732	0,004922	0,006	0,004	1
Htr1b	0,767732	0,004922	0,006	0,004	1
Prlr	0,767732	0,013972	0,006	0,004	1
Gm14827	0,767732	0,010362	0,006	0,004	1
Hist1h1d	0,767732	0,008552	0,006	0,004	1
Gm16982	0,767732	0,006738	0,006	0,004	1
Fgf18	0,767732	0,006738	0,006	0,004	1
Rad9a	0,767828	0,028858	0,163	0,16	1
Cep170	0,768302	0,032857	0,717	0,736	1
B3glct	0,768395	0,022373	0,094	0,101	1
Nrf1	0,768619	0,046751	0,194	0,196	1
Gm28529	0,768637	0,001173	0,017	0,019	1
Mamstr	0,76864	-0,0009	0,017	0,019	1
Nmt1	0,768982	0,027671	0,71	0,755	1
Mcm10	0,769079	0,012168	0,006	0,004	1
Adam1a	0,769079	0,010362	0,006	0,004	1
Ttll8	0,769079	0,008552	0,006	0,004	1
Rprm	0,769079	0,004922	0,006	0,004	1
Fat4	0,769079	0,004922	0,006	0,004	1
Gm37170	0,769079	0,004922	0,006	0,004	1
Hrk	0,769079	0,004922	0,006	0,004	1
Gprc5d	0,769079	0,004922	0,006	0,004	1
Nynrin	0,769079	0,004922	0,006	0,004	1
Gm33280	0,769079	0,003102	0,006	0,004	1
4933424G	0,769079	0,003102	0,006	0,004	1

Gm17634	0,769079	0,003102	0,006	0,004	1
Tstd1	0,769079	0,003102	0,006	0,004	1
2210411M	0,769079	0,003102	0,006	0,004	1
Nox1	0,769079	0,003102	0,006	0,004	1
Fam166b	0,769079	0,003102	0,006	0,004	1
Fam229a	0,769079	0,003102	0,006	0,004	1
Gm16503	0,769079	0,003102	0,006	0,004	1
Gm38947	0,769079	0,003102	0,006	0,004	1
Creb3l3	0,769079	0,003102	0,006	0,004	1
Fhl4	0,769079	0,003102	0,006	0,004	1
Slmapos2	0,769079	0,003102	0,006	0,004	1
Olfm4	0,769079	0,003102	0,006	0,004	1
Gm36198	0,769079	0,003102	0,006	0,004	1
Pth1r	0,769079	0,003102	0,006	0,004	1
4930556J2	0,769079	0,003102	0,006	0,004	1
Camkk1	0,769079	0,003102	0,006	0,004	1
Rph3al	0,769079	0,003102	0,006	0,004	1
Gm34868	0,769079	0,003102	0,006	0,004	1
Gm16059	0,769079	0,003102	0,006	0,004	1
Amhr2	0,769079	0,003102	0,006	0,004	1
Impg2	0,769079	0,003102	0,006	0,004	1
Mdga1	0,769079	0,003102	0,006	0,004	1
Gm26722	0,769179	0,013168	0,068	0,074	1
Cnnm4	0,769406	0,031857	0,075	0,072	1
Acvr1b	0,769665	-0,00772	0,092	0,097	1
Plcxd1	0,769753	0,002823	0,006	0,004	1
Nrbp2	0,769831	0,043348	0,943	0,945	1
Setdb1	0,769844	0,036764	0,297	0,3	1
Sh3bp4	0,770265	0,00326	0,062	0,068	1
Mastl	0,770281	0,0015	0,04	0,044	1
Gm16897	0,770427	0,001279	0,006	0,004	1
Knl1	0,770427	0,001279	0,006	0,004	1
Kcnq2	0,770427	0,001279	0,006	0,004	1
Xlr4c	0,770427	0,001279	0,006	0,004	1
Gm128	0,770427	0,001279	0,006	0,004	1
Gm42957	0,770427	0,001279	0,006	0,004	1
Gm43064	0,770427	0,001279	0,006	0,004	1
Gm43825	0,770427	0,001279	0,006	0,004	1
Msantd1	0,770427	0,001279	0,006	0,004	1
Fzd10	0,770427	0,001279	0,006	0,004	1
Gm10874	0,770427	0,001279	0,006	0,004	1
Gm9008	0,770427	0,001279	0,006	0,004	1
Eva1a	0,770427	0,001279	0,006	0,004	1
Ccnd2	0,770427	0,001279	0,006	0,004	1
Gm44686	0,770427	0,001279	0,006	0,004	1
Wnt11	0,770427	0,001279	0,006	0,004	1
Gm4353	0,770427	0,001279	0,006	0,004	1
Gm47598	0,770427	0,001279	0,006	0,004	1
Elk3	0,770427	0,001279	0,006	0,004	1
8030455M	0,770427	0,001279	0,006	0,004	1
Il17d	0,770427	0,001279	0,006	0,004	1
4930594M	0,770427	0,001279	0,006	0,004	1

Gm19531	0,770427	0,001279	0,006	0,004	1
Gm5922	0,770427	0,001279	0,006	0,004	1
Gm12212	0,770427	0,001279	0,006	0,004	1
Gm17334	0,770427	0,001279	0,006	0,004	1
Vmo1	0,770427	0,001279	0,006	0,004	1
Ccl3	0,770427	0,001279	0,006	0,004	1
Cdc6	0,770427	0,001279	0,006	0,004	1
Gm11361	0,770427	0,001279	0,006	0,004	1
A330048O(	0,770427	0,001279	0,006	0,004	1
Dok3	0,770427	0,001279	0,006	0,004	1
Gm46332	0,770427	0,001279	0,006	0,004	1
9030624G:	0,770427	0,001279	0,006	0,004	1
Gm47096	0,770427	0,001279	0,006	0,004	1
A230087F1	0,770427	0,001279	0,006	0,004	1
1700001L(	0,770427	0,001279	0,006	0,004	1
Gm4262	0,770427	0,001279	0,006	0,004	1
Slc51a	0,770427	0,001279	0,006	0,004	1
3300005D(	0,770427	0,001279	0,006	0,004	1
Cchcr1	0,770427	0,001279	0,006	0,004	1
Psd	0,770427	0,001279	0,006	0,004	1
Xpot	0,770571	0,017015	0,194	0,205	1
Csnk1g1	0,770604	0,051123	0,233	0,234	1
Gm20449	0,771101	0,001003	0,006	0,004	1
H2-Ab1	0,771101	0,001003	0,006	0,004	1
Rasgrf1	0,771102	0,000729	0,006	0,004	1
Npc2	0,771683	0,03153	0,993	0,983	1
Eif3l	0,772409	0,018631	0,912	0,93	1
2700069I1	0,77245	-0,00082	0,006	0,004	1
Gm42984	0,77245	-0,00082	0,006	0,004	1
Srrm3	0,77245	-0,00082	0,006	0,004	1
Gm17039	0,77245	-0,00082	0,006	0,004	1
Prickle4	0,77245	-0,00082	0,006	0,004	1
Sema6b	0,77245	-0,00082	0,006	0,004	1
Lrtm1	0,77245	-0,00291	0,006	0,004	1
Gm17203	0,772577	0,002422	0,017	0,019	1
Fbxo38	0,772987	0,044867	0,281	0,287	1
Faxc	0,77396	0,011688	0,048	0,044	1
Cttnbp2nl	0,774213	0,029916	0,481	0,523	1
Rnasek	0,774632	0,008216	0,042	0,046	1
Lipt1	0,77508	0,00297	0,017	0,019	1
Ccdc189	0,77508	0,00297	0,017	0,019	1
Pkd2l2	0,77508	0,00297	0,017	0,019	1
Tnr	0,77508	0,006554	0,017	0,019	1
Adamtsl3	0,775128	0,012658	0,086	0,082	1
Zdhhc15	0,775209	0,005003	0,04	0,044	1
Itih5	0,775755	0,003192	0,167	0,175	1
Tmc4	0,775799	0,006273	0,017	0,019	1
Kri1	0,775831	0,023793	0,2	0,196	1
Usp21	0,776025	0,007341	0,178	0,188	1
Exoc5	0,776086	0,029784	0,574	0,593	1
Zfp532	0,776952	0,037322	0,527	0,597	1
Qrich1	0,777377	0,041828	0,372	0,407	1

Lta4h	0,77781	0,03999	0,433	0,449	1
Slco2a1	0,777888	0,009201	0,015	0,013	1
Ap5b1	0,777888	0,005604	0,015	0,013	1
Cbl	0,778061	0,066177	0,567	0,624	1
Abcc10	0,77844	0,006589	0,079	0,084	1
Lrrn4cl	0,778668	0,006273	0,017	0,019	1
Snip1	0,779213	0,015328	0,169	0,179	1
Paqr4	0,779304	0,014572	0,572	0,622	1
Dact3	0,779378	0,054013	0,747	0,804	1
Pop1	0,779481	0,026727	0,077	0,074	1
Myo7a	0,779514	0,005043	0,015	0,013	1
Sel1l	0,780083	0,054442	0,783	0,791	1
9330102E(	0,780322	0,0038	0,015	0,013	1
Hyal3	0,780322	0,0038	0,015	0,013	1
B130046B:	0,780322	0,0038	0,015	0,013	1
Sgms2	0,780672	0,018511	0,136	0,131	1
Arsa	0,780806	0,032496	0,338	0,369	1
Chst10	0,781137	0,003522	0,015	0,013	1
Prps1l3	0,781462	0,003253	0,039	0,042	1
Dusp14	0,781538	0,011907	0,017	0,019	1
Tnfsfm13	0,781778	-0,00036	0,015	0,017	1
Mfsd2b	0,781778	-0,00036	0,015	0,017	1
Kctd20	0,781878	0,032109	0,283	0,308	1
Polr1e	0,781973	0,016632	0,026	0,023	1
Uspl1	0,782326	0,038308	0,319	0,348	1
Rab7	0,782459	0,03112	0,991	0,989	1
Shf	0,782472	0,01346	0,084	0,08	1
1700086P(	0,782759	0,001993	0,015	0,013	1
Olfr1385	0,782759	0,001993	0,015	0,013	1
5033430I1	0,782759	0,001993	0,015	0,013	1
Hectd3	0,782883	0,012416	0,213	0,226	1
Dis3	0,782936	0,010497	0,147	0,154	1
Zfp493	0,783187	0,011688	0,048	0,044	1
Rnf145	0,783378	0,018488	0,246	0,259	1
Ccdc149	0,783574	0,001719	0,015	0,013	1
Morn1	0,783673	-0,00149	0,037	0,04	1
R3hdm4	0,783939	0,060898	0,618	0,669	1
Klhl8	0,784233	0,003522	0,172	0,181	1
Zfp273	0,784241	0,03014	0,094	0,091	1
Rab5c	0,784442	0,004787	0,848	0,844	1
Map3k3	0,784473	0,012782	0,154	0,162	1
Tigd5	0,78464	0,009614	0,037	0,034	1
Fbxo6	0,784685	0,009082	0,176	0,186	1
Aim2	0,784825	0,003246	0,015	0,017	1
Glb1l	0,785337	0,009552	0,026	0,023	1
Rnf217	0,785337	0,011327	0,026	0,023	1
Gm13075	0,785619	-0,00982	0,035	0,038	1
Pcnt	0,785766	0,015662	0,27	0,285	1
Pkmyt1	0,786396	0,007282	0,101	0,108	1
Klk8	0,786721	0,097201	0,059	0,055	1
Slc38a7	0,786917	0,017937	0,149	0,158	1
Rpl32	0,78744	0,039944	0,987	0,987	1

Sema3d	0,787796	0,012338	0,061	0,057	1
Sema6c	0,787875	0,003246	0,015	0,017	1
Pttg1ip	0,788122	0,021868	0,69	0,738	1
Zc3h12a	0,78815	0,000614	0,057	0,061	1
Gm26603	0,788258	0,004764	0,015	0,017	1
Nolc1	0,789138	0,060318	0,354	0,367	1
Stag2	0,789312	0,036054	0,738	0,764	1
Nup50	0,789373	0,072392	0,292	0,312	1
Washc2	0,789465	0,041482	0,908	0,911	1
Itga8	0,789628	0,007493	0,026	0,023	1
Rps2	0,789716	0,0802	0,993	0,992	1
Zfp12	0,78972	0,007949	0,123	0,131	1
Rrp1	0,789828	0,025031	0,844	0,865	1
Cacng5	0,790038	0,01602	0,037	0,034	1
Mark3	0,79024	0,056803	0,598	0,65	1
Evi5l	0,790461	0,040347	0,413	0,46	1
Ifngr2	0,790476	0,017607	0,088	0,084	1
Dpp6	0,790498	-0,00476	0,013	0,015	1
Lrrc29	0,790928	0,008627	0,015	0,017	1
Gcc1	0,791239	0,049216	0,13	0,129	1
Ppp2r5e	0,791371	0,037004	0,358	0,373	1
Dedd2	0,791622	0,037863	0,189	0,2	1
Angel1	0,791739	0,009634	0,099	0,095	1
Slc26a2	0,791841	0,026799	0,161	0,173	1
Brdt	0,792341	0,030089	0,314	0,333	1
Dut	0,792418	0,022505	0,174	0,186	1
Gm3055	0,792534	-0,00424	0,013	0,015	1
Jpt1	0,792739	0,022749	0,09	0,097	1
Synpo2	0,79294	-0,0019	0,013	0,015	1
Traf4	0,79294	-0,0019	0,013	0,015	1
Pafah1b3	0,792942	-0,00216	0,013	0,015	1
Tbc1d30	0,793049	0,023551	0,039	0,036	1
Gm49179	0,793308	0,0087	0,026	0,023	1
Hspb1	0,793353	0,001608	0,013	0,015	1
Mcm5	0,793584	0,006202	0,048	0,044	1
Specc1l	0,794061	0,022715	0,294	0,316	1
Ccdc13	0,794115	0,03924	0,558	0,629	1
Cttn	0,794125	0,019602	0,418	0,449	1
Coa5	0,794263	0,01289	0,207	0,217	1
Tjp2	0,794366	0,04076	0,796	0,827	1
Brd1	0,794561	0,021517	0,594	0,643	1
Dqx1	0,794986	-0,00381	0,013	0,015	1
Phlda1	0,795169	0,138801	0,262	0,27	1
Pih1d1	0,795338	0,027124	0,294	0,316	1
Olfr1160	0,795798	-8,8E-05	0,013	0,015	1
Gm14327	0,795798	-8,8E-05	0,013	0,015	1
Gm49064	0,795798	-8,8E-05	0,013	0,015	1
Gm17227	0,795798	-8,8E-05	0,013	0,015	1
Dnd1	0,7958	-0,00036	0,013	0,015	1
Sec23a	0,795867	0,039051	0,215	0,215	1
Zfp653	0,795898	0,037597	0,16	0,158	1
Gm26830	0,796209	0,001446	0,013	0,015	1

Timeless	0,796377	0,003934	0,026	0,023	1
Csrnp3	0,796594	0,015986	0,218	0,23	1
Plppr2	0,797269	0,022742	0,064	0,061	1
Gdpd3	0,79731	0,018347	0,039	0,036	1
Ubtd1	0,797326	0,013134	0,05	0,046	1
Peak1	0,797745	0,061801	0,266	0,291	1
Smyd5	0,797819	0,003767	0,128	0,135	1
Ptbp2	0,79783	0,020801	0,618	0,675	1
Psmc3	0,797867	-0,00091	0,936	0,935	1
Zkscan8	0,797923	0,038021	0,172	0,171	1
Akt1	0,798271	0,046923	0,429	0,454	1
Hivep2	0,798293	0,005188	0,073	0,078	1
1700109H(	0,798658	0,014856	0,062	0,059	1
Syt14	0,798659	0,001719	0,013	0,015	1
Gm47547	0,798661	0,001446	0,013	0,015	1
Arl5b	0,79891	0,040056	0,147	0,158	1
Card19	0,799107	0,02721	0,714	0,734	1
Ddx42	0,799225	0,053131	0,475	0,511	1
Lrrc8c	0,799544	0,046737	0,341	0,346	1
Nhej1	0,799566	0,001395	0,055	0,059	1
Maml2	0,799675	0,006667	0,057	0,061	1
Gm42670	0,800297	0,004764	0,013	0,015	1
Slx1b	0,800832	0,003522	0,189	0,198	1
Rnd2	0,801152	0,04336	0,578	0,599	1
Plp1	0,801347	-0,01571	1	0,998	1
1700056E2	0,801522	0,005323	0,013	0,015	1
Csk	0,801693	0,039901	0,288	0,293	1
Plekhg3	0,801792	0,055202	0,683	0,751	1
Rprd2	0,802634	0,043607	0,4	0,445	1
Pou5f2	0,80267	0,007291	0,075	0,08	1
Setbp1	0,802914	0,0008	0,15	0,156	1
Numa1	0,802971	0,051571	0,547	0,584	1
Tbc1d4	0,803243	0,011516	0,075	0,08	1
Hnrnpab	0,803292	0,043927	0,767	0,81	1
Pus10	0,803431	0,015937	0,101	0,097	1
Aox1	0,80394	7,43E-06	0,037	0,034	1
Patl1	0,80399	0,039499	0,182	0,181	1
Pcsk7	0,804187	0,033317	0,217	0,234	1
Eno1	0,804411	0,010972	0,591	0,605	1
Gm16973	0,80454	0,01879	0,064	0,061	1
Adam15	0,805151	0,026291	0,345	0,371	1
Zfp180	0,805151	0,030656	0,288	0,308	1
Ifit2	0,805278	0,020948	0,204	0,213	1
Zfp236	0,805312	0,018849	0,231	0,228	1
Dxo	0,805375	0,020896	0,18	0,177	1
Atp2b1	0,80554	0,048497	0,688	0,743	1
Snx13	0,805574	0,019689	0,363	0,386	1
Gpr45	0,805585	-0,00371	0,011	0,013	1
Gm16867	0,805585	-0,00371	0,011	0,013	1
Tmem255a	0,805588	-0,00424	0,011	0,013	1
Ogfod1	0,805886	0,029699	0,154	0,152	1
Myd88	0,806085	0,000687	0,072	0,076	1

Zfp319	0,806514	0,015849	0,156	0,165	1
Klf16	0,806515	0,007982	0,099	0,095	1
Gm42788	0,80756	0,006202	0,05	0,046	1
Mepce	0,80803	0,053839	0,358	0,397	1
Eif5b	0,808163	0,048742	0,774	0,787	1
1110006O:	0,808238	-0,00163	0,011	0,013	1
Tmtc1	0,808238	-0,00163	0,011	0,013	1
Grhl1	0,808238	-0,00163	0,011	0,013	1
6330403N:	0,80824	-0,0019	0,011	0,013	1
4930469K:	0,80824	-0,0019	0,011	0,013	1
Vash1	0,809161	0,002302	0,033	0,036	1
Arhgap39	0,809297	0,047918	0,415	0,46	1
Phtf2	0,80945	0,022151	0,064	0,061	1
Hist1h2ae	0,81001	-0,00216	0,011	0,013	1
Gm10220	0,810075	0,017834	0,017	0,015	1
Pank4	0,810752	0,001402	0,106	0,112	1
Cep89	0,810764	0,004591	0,15	0,158	1
Atmin	0,810856	0,021045	0,106	0,103	1
Cfap126	0,810894	0,000183	0,011	0,013	1
Ccdc142os	0,810894	0,000183	0,011	0,013	1
Sap30	0,810894	0,000183	0,011	0,013	1
Gm6994	0,810894	0,000183	0,011	0,013	1
Fam20b	0,811083	0,024617	0,169	0,167	1
Prtg	0,811088	0,017742	0,103	0,11	1
Ftsj3	0,811201	0,03706	0,281	0,306	1
Elp3	0,811369	0,015672	0,306	0,323	1
Fbxo46	0,811485	0,001735	0,156	0,162	1
Gpatch3	0,811507	0,026407	0,066	0,063	1
Aprt	0,81183	0,023985	0,161	0,171	1
Rhobtb2	0,812013	0,021452	0,123	0,131	1
Cadps	0,812103	0,027767	0,084	0,082	1
Gm11110	0,81223	0,008292	0,051	0,049	1
Syt11	0,812695	0,008842	1	0,998	1
Prr12	0,812772	0,026859	0,272	0,272	1
Slc16a2	0,812872	0,003903	0,088	0,093	1
Rsl1	0,813355	0,006099	0,033	0,036	1
Pcp4l1	0,813553	0,0038	0,011	0,013	1
Wdr31	0,813553	0,0038	0,011	0,013	1
Ecm2	0,813554	0,001719	0,011	0,013	1
Myo18a	0,813699	-0,01094	0,809	0,802	1
Smg1	0,813864	0,068535	0,42	0,449	1
Nrarp	0,81437	0,007579	0,035	0,038	1
Auts2	0,814493	0,015941	0,633	0,658	1
Lsm14a	0,814752	0,055713	0,574	0,641	1
Irak1bp1	0,814799	0,005702	0,132	0,139	1
Ghdc	0,814927	0,005558	0,204	0,213	1
Apc2	0,815041	0,007025	0,141	0,148	1
Tsga10	0,815297	0,031754	0,206	0,219	1
Rab3d	0,81561	0,009897	0,028	0,025	1
Poldip3	0,815621	0,046593	0,503	0,54	1
Lefty1	0,815631	-0,00348	0,094	0,099	1
B4galt2	0,815724	0,007025	0,075	0,08	1

Sh2b3	0,815938	0,011124	0,051	0,049	1
Gm34552	0,816213	0,005604	0,011	0,013	1
Prodh	0,816213	0,012785	0,011	0,013	1
Gsk3a	0,816834	0,01816	0,598	0,665	1
Mtmr7	0,817268	-0,00326	0,031	0,034	1
Itgb8	0,81729	0,033606	0,745	0,764	1
Eea1	0,817325	-0,00068	0,492	0,511	1
Trdmt1	0,817813	0,012389	0,092	0,097	1
Gm21680	0,817828	0,004064	0,033	0,036	1
2510017J1	0,818125	0,02294	0,04	0,038	1
Clcn1	0,818149	0,005323	0,017	0,015	1
Foxm1	0,818149	0,007119	0,017	0,015	1
Zfp607b	0,818902	-0,00054	0,123	0,129	1
Zc3h18	0,819641	0,018583	0,418	0,456	1
Qsox1	0,819851	0,062102	0,435	0,466	1
Gm32369	0,820052	-0,00553	0,009	0,011	1
Sipa1l3	0,820215	0,027854	0,242	0,243	1
Cst3	0,82023	0,017773	0,796	0,804	1
Hspb2	0,820338	0,008439	0,072	0,076	1
Wfs1	0,820385	-0,00143	0,119	0,124	1
P2ry12	0,820425	-0,00406	0,05	0,053	1
Zfp451	0,820532	0,0622	0,369	0,414	1
Ap1g1	0,820697	0,048075	0,35	0,384	1
Slc45a4	0,820764	0,011124	0,051	0,049	1
Mdrl	0,820845	0,003522	0,017	0,015	1
Rps6kb1	0,820907	0,031274	0,558	0,605	1
Frrs1l	0,820994	0,002596	0,051	0,055	1
Ppp2r3d	0,821205	0,038177	0,27	0,276	1
Zfp982	0,821618	0,003246	0,017	0,015	1
Adcy3	0,821865	-0,00123	0,031	0,034	1
Myef2	0,821871	0,033098	0,576	0,631	1
Agps	0,821937	0,022996	0,512	0,565	1
Intu	0,821949	0,001395	0,05	0,053	1
Lurap1l	0,822397	-0,0007	0,031	0,034	1
Ctxn1	0,822397	-0,0007	0,031	0,034	1
Evl	0,822437	0,014572	0,053	0,051	1
Gm12496	0,822482	-0,00345	0,009	0,011	1
Spocd1	0,822482	-0,00345	0,009	0,011	1
Pde3b	0,822482	-0,00345	0,009	0,011	1
Slc18b1	0,822482	-0,00345	0,009	0,011	1
Klf12	0,822482	-0,00345	0,009	0,011	1
Dhrs3	0,822482	-0,00553	0,009	0,011	1
Tnfrsf10b	0,822482	-0,00553	0,009	0,011	1
C8g	0,822482	-0,01175	0,009	0,011	1
Srpk3	0,822618	0,046112	0,262	0,283	1
Itpripl2	0,822986	0,02304	0,051	0,049	1
Epb41l5	0,823303	0,012542	0,04	0,038	1
Hipk3	0,823462	0,048836	0,354	0,388	1
Galnt16	0,823889	0,029044	0,251	0,268	1
Eif2ak4	0,824127	0,021925	0,127	0,135	1
Myh9	0,824239	0,040992	0,305	0,31	1
Gm16286	0,824387	0,010136	0,67	0,7	1

Ddx5	0,824764	0,047539	1	1	1
Trim17	0,824913	-0,00136	0,009	0,011	1
Gm49271	0,824913	-0,00136	0,009	0,011	1
Syngap1	0,824913	-0,00136	0,009	0,011	1
Uchl4	0,824915	-0,00163	0,009	0,011	1
Rora	0,825066	0,062033	0,292	0,3	1
Gm45509	0,825484	0,00523	0,051	0,055	1
Cul3	0,825981	0,019486	0,683	0,741	1
Zfyve26	0,825997	0,014163	0,345	0,365	1
2700049A(	0,826146	0,004393	0,106	0,112	1
Hpcal1	0,826364	0,021619	0,217	0,215	1
Gm13620	0,826375	-0,00397	0,009	0,011	1
Psme1	0,826379	0,041267	0,156	0,167	1
Prkaa2	0,826608	0,011055	0,105	0,101	1
Stxbp5l	0,826743	0,002302	0,031	0,034	1
Gm10010	0,827016	-0,00036	0,017	0,015	1
Polk	0,82702	0,017575	0,167	0,173	1
Myo9b	0,827259	0,036166	0,306	0,323	1
B9d1os	0,827348	0,000456	0,009	0,011	1
Gm10353	0,827348	0,000456	0,009	0,011	1
Slc12a4	0,828075	0,010834	0,112	0,118	1
Mmp2	0,828207	0,016313	0,035	0,038	1
Wbp1l	0,828285	0,021909	0,413	0,445	1
Ska3	0,828323	0,009201	0,009	0,011	1
Pomt1	0,828517	0,020496	0,145	0,154	1
Phldb2	0,828643	0,0015	0,031	0,034	1
Zfp811	0,829186	0,000272	0,031	0,034	1
Lrch3	0,829444	0,061599	0,371	0,392	1
Cplx1	0,829784	0,002269	0,009	0,011	1
Pcnx2	0,829784	0,002269	0,009	0,011	1
Tomm6os	0,829784	0,004079	0,009	0,011	1
Tmem41a	0,829794	0,014289	0,066	0,063	1
Arrdc4	0,829996	0,002302	0,031	0,034	1
Nacad	0,83042	0,037324	0,55	0,618	1
Sbk1	0,830422	0,020896	0,165	0,162	1
Mfsd9	0,831077	0,002842	0,031	0,034	1
Zfp202	0,831259	0,009614	0,033	0,036	1
Sphk2	0,831503	0,023325	0,297	0,314	1
9330160F1	0,831526	0,014572	0,033	0,036	1
Elf4	0,831568	-0,003	0,029	0,032	1
Zfp385a	0,832221	0,004079	0,009	0,011	1
Tlr3	0,832621	-0,00017	0,028	0,025	1
AI504432	0,832765	0,012699	0,268	0,266	1
Pcdhga6	0,832859	0,003124	0,05	0,053	1
Ambra1	0,833271	0,026023	0,196	0,194	1
Gmps	0,833297	0,056257	0,382	0,426	1
Camsap1	0,833694	0,056686	0,272	0,285	1
Fbn2	0,834648	-0,00326	0,029	0,032	1
Sdhc	0,834973	0,041739	0,644	0,688	1
Cdk11b	0,835147	0,027476	0,611	0,635	1
Gm26764	0,835424	0,00461	0,031	0,034	1
Mib2	0,835824	0,027599	0,261	0,266	1

Kif5c	0,835859	0,05423	0,31	0,319	1
Acbd5	0,836593	0,004925	0,725	0,751	1
Etv3	0,836938	0,031998	0,191	0,19	1
Arfgef3	0,837769	0,036701	0,253	0,268	1
Tpt1	0,837773	0,058046	1	1	1
Tcea1	0,837986	0,015431	0,824	0,816	1
Trim8	0,838751	0,050775	0,453	0,477	1
Ammecr1l	0,839585	0,019533	0,261	0,274	1
Adck5	0,840805	0,001339	0,029	0,032	1
4930513N	0,840988	0,006453	0,007	0,006	1
Irf8	0,840988	0,017264	0,007	0,006	1
Taf9	0,840988	0,01007	0,007	0,006	1
Fbrs	0,841128	0,034349	0,316	0,321	1
Snx17	0,841856	0,005728	0,688	0,738	1
Tubgcp6	0,842026	0,023905	0,088	0,086	1
Tpcn2	0,842308	0,008983	0,029	0,027	1
Usp32	0,842318	0,054418	0,38	0,422	1
Ppig	0,842352	0,032751	0,695	0,734	1
Spire1	0,842434	0,063088	0,439	0,479	1
B230398E(	0,842737	0,00464	0,007	0,006	1
Syce2	0,842737	0,00464	0,007	0,006	1
Ccr5	0,842737	0,00464	0,007	0,006	1
Hist1h4a	0,842737	0,00464	0,007	0,006	1
Tmc1	0,842737	0,00464	0,007	0,006	1
Ebf4	0,842737	0,006453	0,007	0,006	1
Slc16a4	0,842737	0,006453	0,007	0,006	1
Kif15	0,842737	0,006453	0,007	0,006	1
Pnck	0,843406	-0,00109	0,007	0,008	1
Gm20707	0,843406	-0,00109	0,007	0,008	1
Fam149a	0,843406	-0,00109	0,007	0,008	1
Esyt3	0,843406	-0,00109	0,007	0,008	1
AC124739.	0,843406	-0,00109	0,007	0,008	1
Srp54b	0,843406	-0,00109	0,007	0,008	1
Tdrd9	0,843406	-0,00109	0,007	0,008	1
Srl	0,843406	-0,00109	0,007	0,008	1
Tead3	0,843406	-0,00109	0,007	0,008	1
Ppp1r18	0,843406	-0,00109	0,007	0,008	1
Kctd16	0,843406	-0,00109	0,007	0,008	1
Tal2	0,843407	-0,00136	0,007	0,008	1
Lrrc8d	0,843643	0,060689	0,539	0,589	1
Rasgrf2	0,843953	0,000456	0,007	0,008	1
Strn3	0,843976	0,029433	0,646	0,677	1
Ulk2	0,843977	0,021439	0,545	0,574	1
Gm6525	0,844488	0,008263	0,007	0,006	1
Exd1	0,844488	0,006453	0,007	0,006	1
Tec	0,844488	0,006453	0,007	0,006	1
Cntnap2	0,844488	0,00464	0,007	0,006	1
Thnsl2	0,844488	0,00464	0,007	0,006	1
Rcn1	0,844488	0,002823	0,007	0,006	1
Ercc6l	0,844488	0,002823	0,007	0,006	1
1810062G	0,844488	0,002823	0,007	0,006	1
Sync	0,844488	0,002823	0,007	0,006	1

Gm15614	0,844488	0,002823	0,007	0,006	1
Fxyd5	0,844488	0,002823	0,007	0,006	1
Clec3b	0,844488	0,002823	0,007	0,006	1
Dscc1	0,844488	0,002823	0,007	0,006	1
Prss12	0,844489	0,0038	0,007	0,006	1
Nars	0,844553	0,032645	0,69	0,751	1
Ncoa6	0,844646	0,065375	0,358	0,38	1
Cdh13	0,845072	0,012491	0,007	0,006	1
Mef2d	0,845113	0,009002	0,261	0,272	1
Pfkp	0,8453	0,009897	0,029	0,032	1
Dclre1b	0,845379	-0,00119	0,114	0,118	1
Gm28322	0,845504	0,269968	0,152	0,156	1
Usp3	0,845531	0,016328	0,178	0,175	1
Atg2b	0,845545	0,008427	0,183	0,19	1
Phf12	0,845556	0,052039	0,367	0,38	1
Kcnd1	0,845592	0,007975	0,007	0,008	1
Gm2447	0,845592	0,004359	0,007	0,008	1
Akap5	0,845592	0,004359	0,007	0,008	1
Rgs9	0,845592	0,002546	0,007	0,008	1
Slc9a3	0,845592	0,002546	0,007	0,008	1
Gm16386	0,845592	0,002546	0,007	0,008	1
Efhc1	0,845592	0,000729	0,007	0,008	1
Mroh3	0,845592	0,000729	0,007	0,008	1
Echdc3	0,845592	0,000729	0,007	0,008	1
Olfr1307	0,845592	0,000729	0,007	0,008	1
Gm16754	0,845592	0,000729	0,007	0,008	1
Sarm1	0,845592	0,000729	0,007	0,008	1
Gm40557	0,845592	0,000729	0,007	0,008	1
Vmn2r95	0,845592	0,000729	0,007	0,008	1
Itga4	0,845959	0,016232	0,073	0,078	1
Slc11a1	0,846239	0,001003	0,007	0,006	1
Chrnb2	0,846239	0,001003	0,007	0,006	1
Gm29561	0,846239	0,001003	0,007	0,006	1
1700057H	0,846239	0,001003	0,007	0,006	1
Tekt2	0,846239	0,001003	0,007	0,006	1
Scml4	0,846239	0,001003	0,007	0,006	1
A330041J2	0,846239	0,001003	0,007	0,006	1
Tmc8	0,846239	0,001003	0,007	0,006	1
Dnah17	0,846239	0,001003	0,007	0,006	1
Gm47123	0,846239	0,001003	0,007	0,006	1
Gm26803	0,846239	0,001003	0,007	0,006	1
Fbxl7	0,846239	0,001003	0,007	0,006	1
Emx2os	0,846239	0,001003	0,007	0,006	1
Tm6sf2	0,84624	0,002546	0,007	0,006	1
Mknk1	0,846326	0,009167	0,105	0,11	1
Ttc30a1	0,846659	0,006932	0,029	0,027	1
Gnb4	0,84676	0,051712	0,508	0,555	1
Arhgap15	0,846777	0,024331	0,088	0,093	1
Ftl1-ps1	0,846987	0,019382	0,161	0,171	1
Tubb5	0,847065	0,00228	0,703	0,717	1
Foxred2	0,847139	-0,00104	0,046	0,049	1
Washc4	0,847233	0,017832	0,624	0,667	1

Gm4793	0,847236	-0,00222	0,028	0,03	1
Iffo2	0,84728	0,020181	0,07	0,068	1
Hmg20b	0,847332	0,083839	0,389	0,42	1
Eif5	0,847576	0,021777	0,952	0,96	1
A930004D:	0,847582	0,008627	0,018	0,017	1
4930532G:	0,847779	0,002546	0,007	0,008	1
Gm47237	0,847779	0,002546	0,007	0,008	1
Adamts5	0,847779	0,002546	0,007	0,008	1
Gm10036	0,847779	0,002546	0,007	0,008	1
Iapp	0,847779	0,004359	0,007	0,008	1
Csnk1g2	0,847819	0,041847	0,394	0,432	1
Usf2	0,847988	0,039938	0,739	0,787	1
Mllt3	0,848514	0,026812	0,183	0,194	1
Kif17	0,848576	-0,00109	0,007	0,006	1
Far2	0,848576	-0,00109	0,007	0,006	1
Dok4	0,848576	-0,00109	0,007	0,006	1
A730098A:	0,848576	-0,00109	0,007	0,006	1
Kcnj12	0,848576	-0,00109	0,007	0,006	1
Sfrp4	0,848576	-0,00109	0,007	0,006	1
Gm15966	0,848576	-0,00109	0,007	0,006	1
Pcdhb8	0,848576	-0,00109	0,007	0,006	1
Snrnp48	0,849154	0,046786	0,672	0,736	1
Cacna2d3	0,849281	0,008292	0,05	0,053	1
Emc10	0,849586	0,017349	0,994	0,992	1
AV099323	0,849846	0,003659	0,029	0,027	1
Zfp516	0,849858	0,011367	0,029	0,027	1
Rad51ap1	0,849967	0,01517	0,007	0,008	1
Kctd10	0,850229	0,012658	0,095	0,093	1
Asic3	0,850513	0,006836	0,018	0,017	1
Agt	0,850513	0,010414	0,018	0,017	1
Gm28151	0,850513	0,008627	0,018	0,017	1
Gm13054	0,850513	0,008627	0,018	0,017	1
Cfp	0,850721	0,003386	0,029	0,027	1
Sipa1	0,850929	-0,00896	0,061	0,063	1
Sf3a2	0,850982	0,042032	0,365	0,395	1
Flywch1	0,851332	0,028861	0,58	0,612	1
Nr1d2	0,851514	0,05324	0,242	0,247	1
Magi1	0,851572	0,065556	0,694	0,73	1
Hspb8	0,851598	0,00461	0,029	0,027	1
C2cd3	0,851835	0,041571	0,18	0,181	1
Fbxo30	0,851879	0,031328	0,165	0,165	1
Xpr1	0,85224	0,047148	0,215	0,23	1
Vsig10	0,852751	0,007025	0,042	0,04	1
Slain2	0,852771	0,062575	0,323	0,361	1
Gm29107	0,853445	0,008627	0,018	0,017	1
S100a4	0,853445	0,017532	0,018	0,017	1
Lmbr1l	0,853659	0,018456	0,086	0,084	1
Arih1	0,85377	0,023883	0,519	0,561	1
Skiv2l	0,854096	0,003811	0,343	0,359	1
Hist2h2be	0,854181	0,004764	0,018	0,017	1
Fem1c	0,854364	0,019849	0,374	0,395	1
Limd1	0,854626	0,069489	0,424	0,454	1

Mrpl53	0,85513	-0,00579	0,026	0,027	1
Erbb3	0,855174	0,034661	0,609	0,648	1
Tctn1	0,855217	0,002619	0,079	0,082	1
Cdc20	0,855374	0,002842	0,029	0,027	1
Zbtb7a	0,855391	0,031116	0,817	0,827	1
Clec16a	0,855638	0,017668	0,229	0,228	1
Mfsd4b4	0,855662	0,003113	0,028	0,03	1
Gm39459	0,855665	0,002842	0,028	0,03	1
Pcdhb15	0,856383	0,011616	0,018	0,017	1
Ing5	0,856781	0,035595	0,193	0,205	1
Rrbp1	0,857159	0,011958	0,958	0,947	1
Tmx1	0,857422	0,03494	0,404	0,435	1
Urb1	0,857744	0,008172	0,083	0,086	1
Serpinb6a	0,858204	0,032055	0,818	0,819	1
Btbd7	0,858397	0,062658	0,4	0,437	1
Actr1b	0,858405	0,044551	0,382	0,42	1
Igsf3	0,858887	0,031047	0,152	0,152	1
Acss1	0,859316	0,001446	0,018	0,017	1
Arid2	0,859688	0,036176	0,347	0,376	1
Gm16536	0,859869	0,002529	0,121	0,118	1
St7	0,861488	-0,00078	0,127	0,131	1
Tgds	0,861782	0,005447	0,083	0,086	1
Mindy1	0,861789	0,032219	0,618	0,65	1
Cnot4	0,861799	0,043517	0,499	0,538	1
Mtcl1	0,862116	0,014829	0,15	0,156	1
1700094M	0,862558	-0,00291	0,006	0,006	1
Cited4	0,862558	-0,00291	0,006	0,006	1
Gpx3	0,862558	-0,00291	0,006	0,006	1
Gm14964	0,862558	-0,00291	0,006	0,006	1
Reep2	0,862558	-0,005	0,006	0,006	1
Cdh8	0,862986	0,027077	0,183	0,181	1
Ddx31	0,863341	0,004461	0,046	0,049	1
Gemin5	0,863401	0,002529	0,116	0,12	1
Ccdc112	0,863412	0,020891	0,18	0,19	1
Gm9484	0,863566	0,003386	0,026	0,027	1
Actb	0,863591	0,006422	1	0,998	1
Tmtc2	0,863688	0,083807	0,435	0,479	1
Eda2r	0,863786	0,00891	0,05	0,053	1
Bend7	0,863824	-0,00318	0,006	0,006	1
Rnf128	0,863824	-0,00553	0,006	0,006	1
Mdc1	0,864185	0,033546	0,244	0,247	1
Acot2	0,864327	0,047694	0,426	0,47	1
Tmem236	0,864457	-0,00082	0,006	0,006	1
Nek6	0,864457	-0,00082	0,006	0,006	1
Gm48908	0,864457	-0,00082	0,006	0,006	1
Mnd1	0,864457	-0,00082	0,006	0,006	1
Dcst1	0,864457	-0,00082	0,006	0,006	1
Gm4349	0,864457	-0,00082	0,006	0,006	1
Gm12827	0,864457	-0,00082	0,006	0,006	1
Cfap69	0,864457	-0,00082	0,006	0,006	1
Gm42556	0,864457	-0,00082	0,006	0,006	1
Ica1	0,864457	-0,00082	0,006	0,006	1

Gm26807	0,864457	-0,00082	0,006	0,006	1
Psg16	0,864457	-0,00082	0,006	0,006	1
Drd4	0,864457	-0,00082	0,006	0,006	1
4932443I1	0,864457	-0,00082	0,006	0,006	1
1700030J2	0,864457	-0,00082	0,006	0,006	1
Gm39302	0,864457	-0,00082	0,006	0,006	1
5830418P1	0,864457	-0,00082	0,006	0,006	1
Gm11992	0,864457	-0,00082	0,006	0,006	1
A730081D(	0,864457	-0,00082	0,006	0,006	1
Gm48250	0,864457	-0,00082	0,006	0,006	1
Cdhr2	0,864457	-0,00082	0,006	0,006	1
Zfp457	0,864457	-0,00082	0,006	0,006	1
Gm47849	0,864457	-0,00082	0,006	0,006	1
Gm28989	0,864457	-0,00082	0,006	0,006	1
Gm40437	0,864457	-0,00082	0,006	0,006	1
4930408O1	0,864457	-0,00082	0,006	0,006	1
AC113595.	0,864457	-0,00082	0,006	0,006	1
Gm21833	0,864457	-0,00082	0,006	0,006	1
1700022N1	0,864457	-0,00082	0,006	0,006	1
Csf1r	0,864457	-0,00082	0,006	0,006	1
Pygo1	0,864458	-0,00109	0,006	0,006	1
Nab1	0,86474	0,058491	0,382	0,418	1
Dck	0,86478	-0,003	0,026	0,027	1
Fry	0,864867	0,00398	0,18	0,186	1
Drosha	0,865051	0,023173	0,288	0,289	1
Clstn3	0,865091	0,000729	0,006	0,006	1
Pnpla6	0,865302	0,023098	0,283	0,297	1
AC116487.	0,865704	0,002717	0,044	0,046	1
Taf5	0,866198	0,011244	0,158	0,165	1
Nxf2	0,866357	0,00464	0,006	0,006	1
4930593A(	0,866357	0,002823	0,006	0,006	1
Gm10399	0,866357	0,002823	0,006	0,006	1
Slc18a2	0,866357	0,002823	0,006	0,006	1
4930537H1	0,866357	0,001003	0,006	0,006	1
Il12rb2	0,866357	0,001003	0,006	0,006	1
Ptpn5	0,866357	0,001003	0,006	0,006	1
Nr2f2	0,866357	0,001003	0,006	0,006	1
Ttll13	0,866357	0,001003	0,006	0,006	1
Adam12	0,866357	0,001003	0,006	0,006	1
Gm33148	0,866357	0,001003	0,006	0,006	1
Kdm4d	0,866357	0,001003	0,006	0,006	1
Pih1d2	0,866357	0,001003	0,006	0,006	1
Gm11940	0,866357	0,001003	0,006	0,006	1
Carmil1	0,866357	0,001003	0,006	0,006	1
Gm30655	0,866357	0,001003	0,006	0,006	1
Tex22	0,866357	0,001003	0,006	0,006	1
Uhrf1	0,866357	0,001003	0,006	0,006	1
Gm38910	0,866358	0,000729	0,006	0,006	1
Car7	0,866358	0,000729	0,006	0,006	1
Limk1	0,867186	0,022847	0,116	0,122	1
Crocc	0,867602	0,006653	0,028	0,03	1
Frat2	0,868027	0,014572	0,068	0,072	1

Eml2	0,868156	0,021992	0,925	0,937	1
Zkscan1	0,868255	0,035402	0,266	0,283	1
Ophn1	0,868258	0,002823	0,006	0,006	1
Gm15910	0,868258	0,002823	0,006	0,006	1
Fam129c	0,868258	0,002823	0,006	0,006	1
Myh6	0,868258	0,002823	0,006	0,006	1
Adgrf4	0,868258	0,002823	0,006	0,006	1
Arhgef15	0,868258	0,00464	0,006	0,006	1
Mucl2	0,868258	0,00464	0,006	0,006	1
Tmem191c	0,868394	0,020181	0,068	0,072	1
Prr14	0,86919	0,061832	0,248	0,257	1
Pdp1	0,87085	0,018012	0,127	0,133	1
Zfp239	0,870881	0,074727	0,453	0,504	1
Dnajc5	0,870993	0,035038	0,528	0,565	1
Wdr3	0,871081	0,006223	0,158	0,162	1
Ndufv1	0,871477	0,015566	0,809	0,812	1
Efnb1	0,871867	0,01485	0,072	0,076	1
Hira	0,87221	0,029275	0,231	0,234	1
Hcfc1	0,87257	0,040457	0,374	0,395	1
Nemp2	0,872992	0,01805	0,116	0,114	1
Plin2	0,873187	0,008458	0,123	0,12	1
Rbm15b	0,874072	0,052069	0,215	0,219	1
Gng3	0,874659	0,027626	0,075	0,074	1
Teddm2	0,874745	0,003793	0,026	0,027	1
Heatr5b	0,875027	0,006018	0,191	0,196	1
Inpp1	0,875368	0,005212	0,132	0,137	1
L3mbtl3	0,875455	0,002034	0,031	0,03	1
Ankrd44	0,87632	0,038421	0,294	0,312	1
Herc1	0,877088	0,06921	0,488	0,534	1
Dnajc6	0,877678	0,041419	0,394	0,426	1
Syde1	0,877865	0,016718	0,114	0,112	1
Fam222a	0,878537	0,033576	0,556	0,595	1
Lonrf3	0,878581	0,010126	0,02	0,019	1
Kat7	0,878603	0,036403	0,369	0,384	1
Rad54l	0,878804	0,005387	0,044	0,046	1
Traf6	0,878966	0,023179	0,253	0,268	1
Prag1	0,879029	0,054578	0,446	0,487	1
Hnrnpr	0,880054	0,012715	0,806	0,827	1
Uba6	0,880135	0,038848	0,294	0,314	1
Cttnbp2	0,880464	-0,00114	0,677	0,696	1
Zfp764	0,880526	0,012033	0,059	0,057	1
Gm3854	0,880903	0,009953	0,046	0,044	1
Ankrd13d	0,881367	0,006475	0,044	0,046	1
Spata1	0,881669	1,43E-05	0,073	0,076	1
Ralgapb	0,882596	0,049761	0,374	0,401	1
Kbtbd7	0,882938	0,023208	0,165	0,173	1
Gm48071	0,88321	0,007212	0,026	0,027	1
Med12l	0,883838	0,00215	0,02	0,019	1
Zfp687	0,883997	0,028597	0,251	0,266	1
Wdr90	0,884124	9,42E-05	0,024	0,025	1
Ppfia3	0,88444	0,001608	0,024	0,025	1
Arhgap20	0,884441	0,003113	0,024	0,025	1

Cacnb4	0,884648	0,05628	0,64	0,679	1
Lgals3bp	0,884887	0,004764	0,02	0,019	1
Nfil3	0,885442	0,020371	0,165	0,171	1
Hoxd4.1	0,88559	0,004486	0,02	0,019	1
Gm20652	0,88559	0,004486	0,02	0,019	1
Maml3	0,885916	0,024224	0,121	0,12	1
Zbtb11	0,885943	0,06566	0,437	0,485	1
Zfp248	0,886155	0,017018	0,103	0,101	1
Ppp1r8	0,886364	0,022301	0,259	0,259	1
Pde8b	0,886406	-0,00474	0,004	0,004	1
Gm11273	0,886975	0,002985	0,044	0,042	1
Klhl13	0,887508	-0,00852	0,053	0,051	1
Atxn7l3	0,887922	0,035733	0,31	0,331	1
Pcdhga9	0,887936	0,002717	0,042	0,044	1
Ubr3	0,887952	0,025971	0,58	0,614	1
Cfap45	0,887962	-0,00265	0,004	0,004	1
Gm2464.1	0,887962	-0,00265	0,004	0,004	1
Gm27022	0,887962	-0,00265	0,004	0,004	1
Gm42864	0,887962	-0,00265	0,004	0,004	1
Asprv1	0,887962	-0,00265	0,004	0,004	1
Gm49380	0,887962	-0,00265	0,004	0,004	1
Crtap	0,887962	-0,00265	0,004	0,004	1
Muc4	0,887962	-0,00265	0,004	0,004	1
Gm35439	0,888043	0,00297	0,02	0,019	1
Wdpcp	0,888264	-0,00092	0,07	0,068	1
Kyat1	0,888461	0,01289	0,073	0,072	1
Abcb6	0,888652	0,017814	0,105	0,11	1
Gm16222	0,888741	-0,005	0,004	0,004	1
Bcorl1	0,88888	0,050868	0,347	0,365	1
Amer1	0,88913	0,03485	0,112	0,112	1
Gm37988	0,889519	-0,00055	0,004	0,004	1
Gm27184	0,889519	-0,00055	0,004	0,004	1
Ikbke	0,889519	-0,00055	0,004	0,004	1
Gm16701.:	0,889519	-0,00055	0,004	0,004	1
Gm31728	0,889519	-0,00055	0,004	0,004	1
Gm14486	0,889519	-0,00055	0,004	0,004	1
Klhl41	0,889519	-0,00055	0,004	0,004	1
Zfp385b	0,889519	-0,00055	0,004	0,004	1
Bbox1	0,889519	-0,00055	0,004	0,004	1
Gm13963	0,889519	-0,00055	0,004	0,004	1
Gm17555	0,889519	-0,00055	0,004	0,004	1
Kif16bos	0,889519	-0,00055	0,004	0,004	1
Gm14286	0,889519	-0,00055	0,004	0,004	1
Gm14597	0,889519	-0,00055	0,004	0,004	1
Bbs12	0,889519	-0,00055	0,004	0,004	1
Gm37933	0,889519	-0,00055	0,004	0,004	1
Triqk	0,889519	-0,00055	0,004	0,004	1
1700022I1	0,889519	-0,00055	0,004	0,004	1
Gm12932	0,889519	-0,00055	0,004	0,004	1
Plch2	0,889519	-0,00055	0,004	0,004	1
4632411P(	0,889519	-0,00055	0,004	0,004	1
Gm29609	0,889519	-0,00055	0,004	0,004	1

Gm49027	0,889519	-0,00055	0,004	0,004	1
Rflna	0,889519	-0,00055	0,004	0,004	1
Gm20594	0,889519	-0,00055	0,004	0,004	1
Gm44287	0,889519	-0,00055	0,004	0,004	1
4930471M	0,889519	-0,00055	0,004	0,004	1
Vmn1r58	0,889519	-0,00055	0,004	0,004	1
Mia	0,889519	-0,00055	0,004	0,004	1
Lrfn3	0,889519	-0,00055	0,004	0,004	1
Olfr624	0,889519	-0,00055	0,004	0,004	1
Itgal	0,889519	-0,00055	0,004	0,004	1
Ifitm10	0,889519	-0,00055	0,004	0,004	1
4933406P(	0,889519	-0,00055	0,004	0,004	1
F420014N2	0,889519	-0,00055	0,004	0,004	1
Gm26768	0,889519	-0,00055	0,004	0,004	1
Gm10131	0,889519	-0,00055	0,004	0,004	1
Gm10873	0,889519	-0,00055	0,004	0,004	1
Panx1	0,889519	-0,00055	0,004	0,004	1
Olfr77	0,889519	-0,00055	0,004	0,004	1
Olfr975	0,889519	-0,00055	0,004	0,004	1
Crtam	0,889519	-0,00055	0,004	0,004	1
Gm36799	0,889519	-0,00055	0,004	0,004	1
1700065D1	0,889519	-0,00055	0,004	0,004	1
AU023762	0,889519	-0,00055	0,004	0,004	1
Cmtm7	0,889519	-0,00055	0,004	0,004	1
Dlec1	0,889519	-0,00055	0,004	0,004	1
Gatsl3	0,889519	-0,00055	0,004	0,004	1
Gm5431	0,889519	-0,00055	0,004	0,004	1
Rhbdl3	0,889519	-0,00055	0,004	0,004	1
Gm11592	0,889519	-0,00055	0,004	0,004	1
Rara	0,889519	-0,00055	0,004	0,004	1
Fkbp10	0,889519	-0,00055	0,004	0,004	1
Gm39397	0,889519	-0,00055	0,004	0,004	1
Cygb	0,889519	-0,00055	0,004	0,004	1
Ripor2	0,889519	-0,00055	0,004	0,004	1
Omd	0,889519	-0,00055	0,004	0,004	1
Mcidas	0,889519	-0,00055	0,004	0,004	1
Gm26520	0,889519	-0,00055	0,004	0,004	1
Dab2	0,889519	-0,00055	0,004	0,004	1
4930592A(	0,889519	-0,00055	0,004	0,004	1
Eppk1	0,889519	-0,00055	0,004	0,004	1
Gm16537	0,889519	-0,00055	0,004	0,004	1
AC120150.	0,889519	-0,00055	0,004	0,004	1
Gm20319	0,889519	-0,00055	0,004	0,004	1
Pnldc1	0,889519	-0,00055	0,004	0,004	1
Has1	0,889519	-0,00055	0,004	0,004	1
H2-T24	0,889519	-0,00055	0,004	0,004	1
Gm10093	0,889519	-0,00055	0,004	0,004	1
St8sia3os	0,889519	-0,00055	0,004	0,004	1
Mppe1	0,889519	-0,00055	0,004	0,004	1
Capn1	0,889519	-0,00055	0,004	0,004	1
Cyb561a3	0,889519	-0,00055	0,004	0,004	1
Gm10053	0,889519	-0,00055	0,004	0,004	1

Pyroxd2	0,889519	-0,00055	0,004	0,004	1
Sox5	0,889519	-0,00082	0,004	0,004	1
Gm45833	0,889519	-0,00082	0,004	0,004	1
Il3ra	0,889519	-0,00082	0,004	0,004	1
Ust	0,890124	0,007858	0,033	0,032	1
Gm16302	0,890298	0,001003	0,004	0,004	1
AC126055.	0,890416	-0,00044	0,024	0,025	1
Dusp7	0,890432	0,009487	0,572	0,601	1
Men1	0,890982	0,026598	0,257	0,259	1
2810454H(	0,891038	0,029816	0,136	0,143	1
Gm31282	0,891076	0,008552	0,004	0,004	1
Gm20659	0,891076	0,004922	0,004	0,004	1
Tmem151b	0,891076	0,004922	0,004	0,004	1
Sdcbp2	0,891076	0,003102	0,004	0,004	1
Fam209	0,891076	0,003102	0,004	0,004	1
Gm10767	0,891076	0,003102	0,004	0,004	1
Gm26792	0,891076	0,003102	0,004	0,004	1
Maf	0,891076	0,002546	0,004	0,004	1
Zbtb46	0,891076	0,001279	0,004	0,004	1
Pcdh18	0,891076	0,001279	0,004	0,004	1
Fcrls	0,891076	0,001279	0,004	0,004	1
Lor	0,891076	0,001279	0,004	0,004	1
Frmpd1	0,891076	0,001279	0,004	0,004	1
Sh2d5	0,891076	0,001279	0,004	0,004	1
Samd11	0,891076	0,001279	0,004	0,004	1
Stap1	0,891076	0,001279	0,004	0,004	1
Vmn1r46	0,891076	0,001279	0,004	0,004	1
1700028J1	0,891076	0,001279	0,004	0,004	1
Gm32849	0,891076	0,001279	0,004	0,004	1
Gm2814	0,891076	0,001279	0,004	0,004	1
Gm47414	0,891076	0,001279	0,004	0,004	1
Gm26536	0,891076	0,001279	0,004	0,004	1
Zglp1	0,891076	0,001279	0,004	0,004	1
Ccdc151	0,891076	0,001279	0,004	0,004	1
Kirrel3os	0,891076	0,001279	0,004	0,004	1
Pknox2	0,891076	0,001279	0,004	0,004	1
Islr	0,891076	0,001279	0,004	0,004	1
Gm47113	0,891076	0,001279	0,004	0,004	1
Rrm2	0,891076	0,001279	0,004	0,004	1
A330009N:	0,891076	0,001279	0,004	0,004	1
Popdc2	0,891076	0,001279	0,004	0,004	1
Tcte3	0,891076	0,001279	0,004	0,004	1
Clip4	0,891076	0,001279	0,004	0,004	1
Zxdc	0,891159	0,02278	0,268	0,27	1
Hhat	0,8912	0,001173	0,02	0,019	1
Clcn3	0,891328	0,034356	0,967	0,954	1
Slain1os	0,891905	0,000902	0,02	0,019	1
Mogs	0,89192	0,021104	0,138	0,143	1
Caskin1	0,891988	0,005715	0,024	0,025	1
Als2	0,892345	0,012175	0,119	0,124	1
Smg7	0,892524	0,043201	0,371	0,388	1
Ckap2l	0,892634	0,003102	0,004	0,004	1

Gm14409	0,892634	0,003102	0,004	0,004	1
Calb1	0,892634	0,003102	0,004	0,004	1
Lat	0,892634	0,003102	0,004	0,004	1
Stpg4	0,892634	0,003102	0,004	0,004	1
Ap4e1	0,893121	0,017901	0,154	0,16	1
Cobll1	0,893389	0,066245	0,475	0,536	1
Ttc25	0,893849	0,017716	0,048	0,046	1
Gm49336	0,894057	0,045974	0,253	0,264	1
Cela1	0,894386	-0,00867	0,459	0,46	1
Fbxo11	0,894441	0,029703	0,407	0,447	1
Fam228a	0,894645	0,007827	0,072	0,07	1
6530437J2	0,894714	0,006169	0,009	0,008	1
Fam217b	0,894714	0,009779	0,009	0,008	1
Ankrd40	0,895045	0,036054	0,782	0,793	1
Vps13b	0,895192	0,037272	0,314	0,331	1
Zfp568	0,895262	0,01346	0,077	0,08	1
Zfp326	0,895319	0,062276	0,55	0,603	1
Dvl1	0,895459	0,03867	0,312	0,338	1
Fam89b	0,895768	0,005715	0,024	0,025	1
Clspn	0,89577	0,007212	0,024	0,025	1
Zmym3	0,896617	0,033189	0,261	0,276	1
S100a6	0,896631	0,105507	0,51	0,515	1
Gm45767	0,896648	0,022665	0,134	0,133	1
Gm47785	0,896797	0,004359	0,009	0,008	1
Gm15441	0,896797	0,007975	0,009	0,008	1
Spc25	0,896797	0,006169	0,009	0,008	1
Apold1	0,896797	0,006169	0,009	0,008	1
Eps8l2	0,896797	0,006169	0,009	0,008	1
Cryaa	0,896798	0,005886	0,009	0,008	1
Inhbb	0,896798	0,005604	0,009	0,008	1
Hdhd3	0,89775	0,01486	0,048	0,046	1
Zfp623	0,897897	0,00676	0,075	0,078	1
Arhgap31	0,898232	0,049457	0,306	0,331	1
Nexmif	0,89836	0,01487	0,009	0,008	1
Sppl2a	0,898839	0,033093	0,461	0,496	1
Tspan13	0,898881	0,004359	0,009	0,008	1
Gm28043	0,898881	0,004359	0,009	0,008	1
A430110L2	0,898881	0,002546	0,009	0,008	1
Hmga2	0,898881	0,002546	0,009	0,008	1
Inca1	0,898881	0,002546	0,009	0,008	1
4933408B1	0,898882	0,004079	0,009	0,008	1
Gm26944	0,89893	-0,00296	0,02	0,019	1
Ly96	0,899035	0,003253	0,039	0,04	1
Mfsd1	0,899317	0,012653	0,211	0,217	1
Efcab5	0,899607	0,012829	0,035	0,034	1
Gpr146	0,9004	0,013018	0,145	0,143	1
Taf5l	0,900413	0,021892	0,095	0,099	1
Agfg2	0,900896	0,017945	0,206	0,215	1
Rgsl1	0,900965	0,000729	0,009	0,008	1
Mmp17	0,900965	0,000729	0,009	0,008	1
Sbk2	0,900965	0,000729	0,009	0,008	1
Arhgef25	0,900965	0,000729	0,009	0,008	1

A830082K1	0,900965	0,000729	0,009	0,008	1
Gm26533	0,900965	0,000729	0,009	0,008	1
Olfr1423	0,900965	0,000729	0,009	0,008	1
Asxl1	0,901181	0,033471	0,235	0,249	1
Olfr961	0,901487	0,000456	0,009	0,008	1
Rpp25	0,901487	0,000456	0,009	0,008	1
Pcdhb12	0,901487	0,000456	0,009	0,008	1
Kdsr	0,901793	0,03758	0,67	0,698	1
Ints12	0,901908	0,01162	0,112	0,116	1
Sesn2	0,902013	0,020316	0,044	0,046	1
Bri3	0,902072	0,018243	0,894	0,886	1
Set	0,902156	0,02173	0,961	0,964	1
Cebpd	0,902391	0,082818	0,284	0,306	1
Nme2	0,902998	0,009167	0,116	0,114	1
1700041G1	0,903052	-0,0019	0,009	0,008	1
Tnk2	0,903122	0,034709	0,242	0,249	1
Lzts1	0,903334	0,011039	0,024	0,025	1
Pcna-ps2	0,903565	0,005275	0,04	0,042	1
5730507C0	0,903573	-0,00136	0,009	0,008	1
Rbbp8	0,903573	-0,00136	0,009	0,008	1
Gm44763	0,904242	0,009953	0,042	0,044	1
Hmga1	0,904307	0,001194	0,075	0,078	1
Rel	0,90481	0,028977	0,066	0,07	1
Dnm1l	0,907064	0,026089	0,521	0,551	1
Bod1l	0,907855	0,023718	0,754	0,776	1
Rhoa	0,907892	0,020373	1	0,998	1
Phf13	0,908111	0,028037	0,143	0,15	1
Safb2	0,908179	0,045131	0,569	0,622	1
Rpl35a	0,908894	0,049063	0,998	0,994	1
Tgif2	0,908901	0,004671	0,088	0,091	1
Mboat2	0,908973	0,057495	0,512	0,561	1
Fubp1	0,909292	0,060504	0,717	0,734	1
Exoc3	0,909354	0,031081	0,714	0,745	1
Mettl6	0,91021	0,022423	0,189	0,19	1
Hook3	0,910577	0,033168	0,848	0,878	1
Tmem248	0,910902	0,037103	0,308	0,319	1
Kdm4b	0,911101	0,030058	0,213	0,224	1
Map1a	0,911161	0,026112	0,996	0,989	1
Nek9	0,911168	0,032066	0,396	0,42	1
Gfod1	0,911493	0,002529	0,116	0,118	1
Afap1l1	0,912539	0,056754	0,4	0,445	1
Srpk2	0,913088	0,01712	0,662	0,743	1
Ptk2b	0,913111	0,010515	0,039	0,04	1
Nln	0,913239	0,030728	0,228	0,23	1
Galt	0,913257	0,004486	0,022	0,021	1
Nupr1l	0,913257	0,006273	0,022	0,021	1
Mutyh	0,913471	0,007774	0,022	0,023	1
Pcdhgb7	0,913472	0,007493	0,022	0,023	1
Pdgfa	0,913901	0,014912	0,956	0,937	1
Gm28959	0,913931	0,004209	0,022	0,021	1
Armcx3	0,914001	0,033109	0,44	0,47	1
Ino80d	0,914309	0,068199	0,519	0,576	1

Polm	0,914379	0,004437	0,073	0,072	1
Akap2	0,914593	0,01212	0,095	0,099	1
Eme2	0,91461	0,00461	0,035	0,034	1
Vps33a	0,914631	0,008671	0,226	0,234	1
Caprin1	0,915069	0,033932	0,706	0,738	1
Prdm2	0,915766	0,038784	0,352	0,365	1
Setx	0,915821	0,062015	0,402	0,445	1
Pde1c	0,916238	0,032722	0,297	0,302	1
Nhlrc2	0,916596	0,037352	0,288	0,308	1
Zfp469	0,916619	0,008058	0,022	0,021	1
Sept3	0,916957	0,004209	0,022	0,021	1
Psmc3ip	0,917909	0,011082	0,037	0,036	1
Tex10	0,91792	0,026065	0,286	0,297	1
Jmjd4	0,918143	0,005549	0,039	0,04	1
Kdm8	0,918143	0,009051	0,039	0,04	1
Ttbk2	0,918749	0,065166	0,374	0,403	1
Larp4b	0,918761	0,063384	0,547	0,591	1
Twnk	0,919022	0,023917	0,16	0,167	1
Tram1l1	0,919784	0,006133	0,07	0,072	1
Rragc	0,920353	0,048925	0,534	0,584	1
Fgfr1op2	0,920842	0,017505	0,659	0,703	1
Itpka	0,921277	-0,00238	0,002	0,002	1
Tmem212	0,921277	-0,00238	0,002	0,002	1
Orc1	0,921277	-0,00238	0,002	0,002	1
Rbakdn	0,921277	-0,00238	0,002	0,002	1
Slc16a13	0,921277	-0,00238	0,002	0,002	1
Dock11	0,921277	-0,00291	0,002	0,002	1
St8sia3	0,921277	-0,00448	0,002	0,002	1
Tmem158	0,921277	-0,00474	0,002	0,002	1
Col4a1	0,921277	-0,00657	0,002	0,002	1
Prkab2	0,921519	0,001194	0,07	0,072	1
Ube3b	0,922021	0,0204	0,246	0,251	1
Mterf1b	0,922278	0,000902	0,02	0,021	1
A830018L1	0,922382	-0,00027	0,002	0,002	1
Gm28981	0,922382	-0,00027	0,002	0,002	1
Wnt6	0,922382	-0,00027	0,002	0,002	1
Sphkap	0,922382	-0,00027	0,002	0,002	1
Ackr3	0,922382	-0,00027	0,002	0,002	1
Syt2	0,922382	-0,00027	0,002	0,002	1
Ptpn7	0,922382	-0,00027	0,002	0,002	1
Pkp1	0,922382	-0,00027	0,002	0,002	1
Ptprc	0,922382	-0,00027	0,002	0,002	1
Prrx1	0,922382	-0,00027	0,002	0,002	1
Arhgap30	0,922382	-0,00027	0,002	0,002	1
Ccdc121	0,922382	-0,00027	0,002	0,002	1
Slc39a12	0,922382	-0,00027	0,002	0,002	1
Scn1a	0,922382	-0,00027	0,002	0,002	1
Chrna1os	0,922382	-0,00027	0,002	0,002	1
Olfr1284	0,922382	-0,00027	0,002	0,002	1
Ryr3	0,922382	-0,00027	0,002	0,002	1
Bub1b	0,922382	-0,00027	0,002	0,002	1
Gm14978	0,922382	-0,00027	0,002	0,002	1

Il1a	0,922382	-0,00027	0,002	0,002	1
Pax1	0,922382	-0,00027	0,002	0,002	1
Lpin3	0,922382	-0,00027	0,002	0,002	1
Ube2c	0,922382	-0,00027	0,002	0,002	1
Zfp217	0,922382	-0,00027	0,002	0,002	1
Gm14393	0,922382	-0,00027	0,002	0,002	1
Xlr	0,922382	-0,00027	0,002	0,002	1
5430427O	0,922382	-0,00027	0,002	0,002	1
Eda	0,922382	-0,00027	0,002	0,002	1
Gpr143	0,922382	-0,00027	0,002	0,002	1
Gm15726	0,922382	-0,00027	0,002	0,002	1
Gpr160	0,922382	-0,00027	0,002	0,002	1
Mccc1os	0,922382	-0,00027	0,002	0,002	1
Sertm1	0,922382	-0,00027	0,002	0,002	1
Igsf10	0,922382	-0,00027	0,002	0,002	1
Celf3	0,922382	-0,00027	0,002	0,002	1
Gm20633	0,922382	-0,00027	0,002	0,002	1
Fcgr1	0,922382	-0,00027	0,002	0,002	1
Ankrd34a	0,922382	-0,00027	0,002	0,002	1
Ngf	0,922382	-0,00027	0,002	0,002	1
Ptpn22	0,922382	-0,00027	0,002	0,002	1
Kcnd3	0,922382	-0,00027	0,002	0,002	1
Gm26530	0,922382	-0,00027	0,002	0,002	1
Alpk1	0,922382	-0,00027	0,002	0,002	1
Gbp5	0,922382	-0,00027	0,002	0,002	1
B230334C	0,922382	-0,00027	0,002	0,002	1
Gm26857	0,922382	-0,00027	0,002	0,002	1
Runx1t1	0,922382	-0,00027	0,002	0,002	1
Gm20878	0,922382	-0,00027	0,002	0,002	1
Brinp1	0,922382	-0,00027	0,002	0,002	1
Ror1	0,922382	-0,00027	0,002	0,002	1
Bend5	0,922382	-0,00027	0,002	0,002	1
Runx3	0,922382	-0,00027	0,002	0,002	1
Mxra8	0,922382	-0,00027	0,002	0,002	1
Isg15	0,922382	-0,00027	0,002	0,002	1
Cdk6	0,922382	-0,00027	0,002	0,002	1
Gbp9	0,922382	-0,00027	0,002	0,002	1
Myo1h	0,922382	-0,00027	0,002	0,002	1
Gm16001	0,922382	-0,00027	0,002	0,002	1
Caln1	0,922382	-0,00027	0,002	0,002	1
Card11	0,922382	-0,00027	0,002	0,002	1
Gm20635	0,922382	-0,00027	0,002	0,002	1
Tex26	0,922382	-0,00027	0,002	0,002	1
Cd8b1	0,922382	-0,00027	0,002	0,002	1
Slc4a5	0,922382	-0,00027	0,002	0,002	1
Gm26588	0,922382	-0,00027	0,002	0,002	1
Alox5	0,922382	-0,00027	0,002	0,002	1
Iqsec3	0,922382	-0,00027	0,002	0,002	1
Ptpn6	0,922382	-0,00027	0,002	0,002	1
Rasl2-9	0,922382	-0,00027	0,002	0,002	1
Catsperg1	0,922382	-0,00027	0,002	0,002	1
Hpn	0,922382	-0,00027	0,002	0,002	1

Gm12781	0,922382	-0,00027	0,002	0,002	1
Tdrd12	0,922382	-0,00027	0,002	0,002	1
Mir9-3hg	0,922382	-0,00027	0,002	0,002	1
Homer2	0,922382	-0,00027	0,002	0,002	1
Folr2	0,922382	-0,00027	0,002	0,002	1
Olfr472	0,922382	-0,00027	0,002	0,002	1
Gm47710	0,922382	-0,00027	0,002	0,002	1
Abracl	0,922382	-0,00027	0,002	0,002	1
Ddo	0,922382	-0,00027	0,002	0,002	1
2810425M	0,922382	-0,00027	0,002	0,002	1
4921516A(	0,922382	-0,00027	0,002	0,002	1
Amh	0,922382	-0,00027	0,002	0,002	1
1500009L1	0,922382	-0,00027	0,002	0,002	1
Ppp1r3b	0,922382	-0,00027	0,002	0,002	1
Sorbs2	0,922382	-0,00027	0,002	0,002	1
Neil3	0,922382	-0,00027	0,002	0,002	1
Lyl1	0,922382	-0,00027	0,002	0,002	1
G430095P	0,922382	-0,00027	0,002	0,002	1
Exoc3l	0,922382	-0,00027	0,002	0,002	1
Il34	0,922382	-0,00027	0,002	0,002	1
Vstm4	0,922382	-0,00027	0,002	0,002	1
Gm48933	0,922382	-0,00027	0,002	0,002	1
Olfr731	0,922382	-0,00027	0,002	0,002	1
Klhl33	0,922382	-0,00027	0,002	0,002	1
Psmb11	0,922382	-0,00027	0,002	0,002	1
Thtpa	0,922382	-0,00027	0,002	0,002	1
Fut4	0,922382	-0,00027	0,002	0,002	1
Zfp872	0,922382	-0,00027	0,002	0,002	1
Olfr986	0,922382	-0,00027	0,002	0,002	1
Tex12	0,922382	-0,00027	0,002	0,002	1
Gm47271	0,922382	-0,00027	0,002	0,002	1
Ccnb2	0,922382	-0,00027	0,002	0,002	1
Cacna2d2	0,922382	-0,00027	0,002	0,002	1
Susd5	0,922382	-0,00027	0,002	0,002	1
Gm12116	0,922382	-0,00027	0,002	0,002	1
Gm10447	0,922382	-0,00027	0,002	0,002	1
Glra1	0,922382	-0,00027	0,002	0,002	1
Ntn1	0,922382	-0,00027	0,002	0,002	1
Olfr392	0,922382	-0,00027	0,002	0,002	1
Mmp28	0,922382	-0,00027	0,002	0,002	1
Tom1l1	0,922382	-0,00027	0,002	0,002	1
Car10	0,922382	-0,00027	0,002	0,002	1
Spata20	0,922382	-0,00027	0,002	0,002	1
Gm11639	0,922382	-0,00027	0,002	0,002	1
Cd300c2	0,922382	-0,00027	0,002	0,002	1
Gm11772	0,922382	-0,00027	0,002	0,002	1
Inhba	0,922382	-0,00027	0,002	0,002	1
Hist1h2br	0,922382	-0,00027	0,002	0,002	1
Hist1h2ad	0,922382	-0,00027	0,002	0,002	1
Hist1h2ab	0,922382	-0,00027	0,002	0,002	1
D130043K	0,922382	-0,00027	0,002	0,002	1
Serpinb9	0,922382	-0,00027	0,002	0,002	1

Gm48707	0,922382	-0,00027	0,002	0,002	1
Ly86	0,922382	-0,00027	0,002	0,002	1
Gm47732	0,922382	-0,00027	0,002	0,002	1
Gm28707	0,922382	-0,00027	0,002	0,002	1
Rnf182	0,922382	-0,00027	0,002	0,002	1
Susd3	0,922382	-0,00027	0,002	0,002	1
Gprin1	0,922382	-0,00027	0,002	0,002	1
Adcy2	0,922382	-0,00027	0,002	0,002	1
Gm17750	0,922382	-0,00027	0,002	0,002	1
4833422C:	0,922382	-0,00027	0,002	0,002	1
Pygl	0,922382	-0,00027	0,002	0,002	1
Sptb	0,922382	-0,00027	0,002	0,002	1
Noxred1	0,922382	-0,00027	0,002	0,002	1
Gm16006	0,922382	-0,00027	0,002	0,002	1
C1qtnf6	0,922382	-0,00027	0,002	0,002	1
AL590144.	0,922382	-0,00027	0,002	0,002	1
Gm20324	0,922382	-0,00027	0,002	0,002	1
Gtse1	0,922382	-0,00027	0,002	0,002	1
Mapk12	0,922382	-0,00027	0,002	0,002	1
Nckap1l	0,922382	-0,00027	0,002	0,002	1
Glis2	0,922382	-0,00027	0,002	0,002	1
Ifitm7	0,922382	-0,00027	0,002	0,002	1
Chrd	0,922382	-0,00027	0,002	0,002	1
B630019A:	0,922382	-0,00027	0,002	0,002	1
Tmem44	0,922382	-0,00027	0,002	0,002	1
Gm15564	0,922382	-0,00027	0,002	0,002	1
Robo2	0,922382	-0,00027	0,002	0,002	1
Prss27	0,922382	-0,00027	0,002	0,002	1
Rhbdl1	0,922382	-0,00027	0,002	0,002	1
Ip6k3	0,922382	-0,00027	0,002	0,002	1
Spdef	0,922382	-0,00027	0,002	0,002	1
Aif1	0,922382	-0,00027	0,002	0,002	1
Lst1	0,922382	-0,00027	0,002	0,002	1
Zfp57	0,922382	-0,00027	0,002	0,002	1
Fhod3	0,922382	-0,00027	0,002	0,002	1
Efemp2	0,922382	-0,00027	0,002	0,002	1
Lpxn	0,922382	-0,00027	0,002	0,002	1
Pcsk5	0,922382	-0,00027	0,002	0,002	1
Cnnm1	0,922382	-0,00027	0,002	0,002	1
Ablim1	0,922382	-0,00027	0,002	0,002	1
Rorc	0,922382	-0,00055	0,002	0,002	1
Ints4	0,922799	0,053983	0,343	0,371	1
Jarid2	0,922938	0,07057	0,457	0,504	1
Cldn19	0,923487	0,013376	0,002	0,002	1
Col14a1	0,923487	0,005205	0,002	0,002	1
Vwa5b2	0,923487	0,005205	0,002	0,002	1
Fgl1	0,923487	0,003382	0,002	0,002	1
Klhl3	0,923487	0,003382	0,002	0,002	1
Efna5	0,923487	0,003382	0,002	0,002	1
1500015O:	0,923487	0,001556	0,002	0,002	1
Gm20753	0,923487	0,001556	0,002	0,002	1
Ar	0,923487	0,001556	0,002	0,002	1

Gbp2	0,923487	0,001556	0,002	0,002	1
Gabbr2	0,923487	0,001556	0,002	0,002	1
Rab42	0,923487	0,001556	0,002	0,002	1
Ncf1	0,923487	0,001556	0,002	0,002	1
Siglech	0,923487	0,001556	0,002	0,002	1
Gm15880	0,923487	0,001556	0,002	0,002	1
Cnn2	0,923487	0,001556	0,002	0,002	1
Rmst	0,923487	0,001556	0,002	0,002	1
Arhgap10	0,923487	0,001556	0,002	0,002	1
Tbc1d9	0,923487	0,001556	0,002	0,002	1
Erc2	0,923487	0,001556	0,002	0,002	1
Lrrc18	0,923487	0,001556	0,002	0,002	1
Zic1	0,923487	0,001556	0,002	0,002	1
Plod2	0,923487	0,001556	0,002	0,002	1
4930500F1	0,923487	0,001556	0,002	0,002	1
6430571L1	0,923487	0,001556	0,002	0,002	1
Gm26542	0,923487	0,001556	0,002	0,002	1
Shc3	0,923487	0,001556	0,002	0,002	1
Spata9	0,923487	0,001556	0,002	0,002	1
Mis18bp1	0,923487	0,001556	0,002	0,002	1
Ak7	0,923487	0,001556	0,002	0,002	1
Rimbp3	0,923487	0,001556	0,002	0,002	1
4632428C(	0,923487	0,001556	0,002	0,002	1
AC150035.	0,923487	0,001556	0,002	0,002	1
Syt7	0,923487	0,001556	0,002	0,002	1
Ms4a7	0,923487	0,001556	0,002	0,002	1
Pja2	0,92356	0,037393	0,778	0,81	1
N4bp2	0,923721	0,014039	0,101	0,103	1
Khnyn	0,923792	0,008273	0,134	0,137	1
Smc1a	0,923915	0,008814	0,732	0,747	1
Zfp90	0,924901	0,040215	0,22	0,234	1
Foxo4	0,924966	0,020698	0,152	0,156	1
Colec11	0,925723	0,006273	0,02	0,021	1
Phf8	0,92594	0,035029	0,266	0,283	1
Atad1	0,926058	0,029753	0,661	0,698	1
Rundc1	0,926386	0,016385	0,149	0,154	1
Rps6kb2	0,926924	0,027604	0,222	0,226	1
Nop2	0,927337	0,029498	0,119	0,12	1
Smc3	0,927596	0,03065	0,727	0,762	1
Sympk	0,927861	0,029655	0,497	0,549	1
Dennd1a	0,927928	0,034993	0,376	0,388	1
Ggcx	0,928077	0,019989	0,099	0,103	1
Socs5	0,928289	0,038508	0,281	0,293	1
Traf7	0,928819	0,03153	0,18	0,19	1
Foxp1	0,928913	0,03389	0,624	0,654	1
Klc4	0,929575	0,010115	0,079	0,078	1
Kat2b	0,929738	0,017446	0,723	0,77	1
Zyg11b	0,930055	0,024106	0,316	0,329	1
Zzef1	0,930397	0,067971	0,347	0,369	1
Zfp36l1	0,93055	0,012228	0,02	0,021	1
Rpl3	0,930822	0,038239	0,998	0,996	1
Ttc21b	0,931039	0,035068	0,13	0,131	1

Creb3l2	0,931745	0,064759	0,519	0,555	1
Ccdc82	0,93224	0,048149	0,222	0,234	1
Atp9b	0,932372	0,0349	0,279	0,291	1
Eif3d	0,932764	0,018552	0,642	0,698	1
Adcy6	0,934559	0,011205	0,072	0,074	1
Pcm1	0,93525	0,012294	0,897	0,892	1
D630045J1	0,935572	0,011327	0,024	0,023	1
Gm26508	0,935572	0,009552	0,024	0,023	1
Syt3	0,935699	0,009732	0,053	0,055	1
Alkbh2	0,935903	0,000803	0,035	0,036	1
Yy1	0,936097	0,014095	0,732	0,764	1
Ctc1	0,936383	0,02294	0,136	0,141	1
Rfx5	0,93664	0,009118	0,051	0,051	1
Xkr6	0,936672	0,040683	0,163	0,167	1
Icmt	0,936776	0,010846	0,284	0,293	1
A730015C1	0,936804	0,009489	0,011	0,011	1
Nek1	0,93751	0,046971	0,206	0,211	1
Wrn	0,937628	0,038421	0,292	0,31	1
Opa1	0,937781	0,023849	0,554	0,608	1
Itch	0,938258	-0,01202	0,912	0,911	1
Gabarapl1	0,938571	0,018941	0,895	0,867	1
Rad51	0,939125	0,004209	0,024	0,023	1
Cyb5d1	0,939125	0,004209	0,024	0,023	1
Cdh24	0,939175	0,004079	0,011	0,011	1
Nes	0,939175	0,018442	0,011	0,011	1
Atp1a3	0,939175	0,01487	0,011	0,011	1
Gm28230	0,939175	0,007689	0,011	0,011	1
Gm16751	0,939175	0,007689	0,011	0,011	1
Slc26a6	0,939175	0,005886	0,011	0,011	1
Pknox1	0,939277	0,058134	0,297	0,312	1
Prpf4b	0,939662	0,043315	0,877	0,863	1
Eif4a2	0,93967	0,021794	0,932	0,932	1
Cirbp	0,939733	0,045333	0,716	0,753	1
Pcdhb7	0,939774	0,006932	0,024	0,023	1
Mhrt	0,940101	-0,0009	0,018	0,019	1
Garnl3	0,940367	0,032368	0,141	0,143	1
Ing1	0,940868	0,080368	0,396	0,428	1
Rbm8a2	0,941546	0,002269	0,011	0,011	1
Gm32568	0,941546	0,002269	0,011	0,011	1
Senp3	0,941661	0,03509	0,284	0,295	1
Rag1	0,942021	0,001993	0,011	0,011	1
Cspg4	0,942021	0,001993	0,011	0,011	1
7630403G1	0,942495	0,001719	0,011	0,011	1
1300017J0	0,942965	0,015162	0,02	0,021	1
Hsf4	0,943001	0,000632	0,018	0,019	1
Parp6	0,943109	0,032365	0,161	0,169	1
Gm28050	0,943363	0,000902	0,018	0,019	1
U2af1l4	0,943725	0,00297	0,018	0,019	1
8030442B0	0,943918	0,000456	0,011	0,011	1
Rimklb	0,943918	0,000456	0,011	0,011	1
1700112J1	0,943918	0,000456	0,011	0,011	1
Ict1os	0,943918	0,000456	0,011	0,011	1

Greb1	0,943918	0,000456	0,011	0,011	1
Tiam2	0,943918	0,000456	0,011	0,011	1
Tulp2	0,943918	0,001993	0,011	0,011	1
Alg10b	0,944087	0,007429	0,156	0,156	1
Ubox5	0,944334	0,001812	0,064	0,065	1
Gm13199	0,944393	0,000183	0,011	0,011	1
Adam8	0,944393	0,000183	0,011	0,011	1
Kiss1r	0,944393	0,000183	0,011	0,011	1
4931414P1	0,944932	0,004337	0,035	0,036	1
Zxdb	0,946101	0,008022	0,136	0,135	1
Hs3st1	0,946144	0,019924	0,776	0,827	1
Cdk5r1	0,946542	0,019602	0,07	0,072	1
Cdt1	0,946626	0,002696	0,018	0,019	1
Enc1	0,946765	-0,00163	0,011	0,011	1
Gnal	0,946765	-0,00163	0,011	0,011	1
Copb2	0,94707	0,070414	0,457	0,504	1
Bbx	0,9472	0,008446	0,563	0,578	1
Coa7	0,947646	0,021045	0,106	0,11	1
Gid4	0,947824	0,056289	0,378	0,416	1
Plekhf1	0,947854	0,030785	0,312	0,329	1
Pcnx	0,947953	0,059399	0,266	0,287	1
Ada	0,948118	0,004816	0,081	0,082	1
Zscan22	0,948983	0,021609	0,103	0,103	1
Atf1	0,949436	0,050734	0,281	0,302	1
Gm34961	0,950252	0,004764	0,018	0,019	1
Adar	0,950297	0,011777	0,165	0,169	1
Ldb1	0,9503	0,030749	0,295	0,302	1
Car5b	0,950442	-0,00143	0,024	0,023	1
Kif1c	0,951045	0,019486	0,183	0,186	1
2410131K1	0,951474	0,016158	0,128	0,129	1
Map4k5	0,95229	0,012086	0,666	0,684	1
Comt	0,952341	0,016768	0,743	0,759	1
Srcap	0,953267	0,033524	0,316	0,327	1
Depdc1b	0,953349	0,003656	0,051	0,051	1
Sprtn	0,953659	0,012551	0,174	0,175	1
Dap	0,953753	0,009845	0,083	0,082	1
Gm10138	0,953794	0,015122	0,084	0,086	1
Chd3	0,953967	0,007858	0,035	0,036	1
Tcerg1	0,954346	0,034183	0,378	0,401	1
Zbtb38	0,954502	0,037032	0,809	0,844	1
Sag	0,954648	0,009576	0,083	0,082	1
Usp27x	0,955193	0,008772	0,039	0,038	1
Gm47802	0,955449	0,011039	0,026	0,025	1
9130011E1	0,955776	0,022637	0,042	0,042	1
Ppp1r13l	0,956419	0,008058	0,018	0,019	1
Lypd1	0,956671	-0,12903	0,037	0,036	1
Fam78b	0,956782	0,008342	0,018	0,019	1
Aqp11	0,956782	0,008342	0,018	0,019	1
Ccnt2	0,956971	0,026462	0,653	0,698	1
Ttll11	0,957423	-0,0045	0,017	0,017	1
Gpbp1l1	0,957473	0,050775	0,505	0,555	1
Usp19	0,957862	0,033285	0,446	0,466	1

Wdr82	0,957945	0,036464	0,272	0,283	1
Gapvd1	0,95806	0,0483	0,615	0,65	1
Zbtb39	0,958503	0,017018	0,103	0,105	1
Rpl22	0,95858	0,03103	0,982	0,964	1
Zkscan6	0,959157	0,020907	0,088	0,089	1
Glce	0,959592	-0,00381	0,051	0,051	1
Atf6	0,959598	0,056517	0,349	0,378	1
Sema3b	0,959731	-0,00372	0,084	0,084	1
Kdm5b	0,960652	0,062095	0,371	0,405	1
Galnt18	0,960872	-0,00243	0,017	0,017	1
Ube2cbp	0,960872	-0,00243	0,017	0,017	1
Rp2	0,961114	0,022674	0,161	0,165	1
Nkain1	0,961284	0,022164	0,624	0,667	1
Pom121	0,961386	0,053446	0,378	0,418	1
Slc26a1	0,961972	0,00461	0,033	0,034	1
Zfhx2os	0,962211	0,01148	0,066	0,068	1
D17Wsu92	0,96231	0,030321	0,545	0,584	1
Cadm1	0,962782	0,084162	0,754	0,783	1
Pex26	0,963387	0,010337	0,066	0,068	1
Por	0,963497	0,063121	0,426	0,466	1
Secisbp2l	0,963654	0,014745	0,987	0,981	1
Tm9sf4	0,963878	0,036915	0,433	0,47	1
Papd5	0,964039	0,048	0,297	0,314	1
Myo1e	0,964246	0,055112	0,565	0,637	1
Mlkl	0,964322	-0,00036	0,017	0,017	1
Noc4l	0,964426	0,012966	0,114	0,116	1
Vps35	0,965806	0,003315	0,895	0,914	1
Jak2	0,965857	0,039113	0,417	0,445	1
Gsdme	0,966192	-0,00097	0,031	0,032	1
Metrnl	0,966286	0,019915	0,013	0,013	1
Kremen1	0,966412	0,031629	0,182	0,188	1
Zim1	0,966623	0,000902	0,017	0,017	1
Aldh18a1	0,967006	0,001173	0,017	0,017	1
Stard9	0,967059	0,022452	0,237	0,243	1
Snhg12	0,967063	0,05089	0,451	0,485	1
Arv1	0,96716	0,005929	0,048	0,049	1
Gm43625	0,967389	0,003246	0,017	0,017	1
Gm37027	0,967389	0,001446	0,017	0,017	1
Atf2	0,96764	0,047895	0,484	0,527	1
Mmp16	0,968838	-0,00044	0,026	0,025	1
Plekha4	0,968912	0,010994	0,013	0,013	1
Rapgefl1	0,968912	0,014572	0,013	0,013	1
Qser1	0,969062	0,01958	0,251	0,255	1
Bcl7c	0,96912	0,042541	0,477	0,53	1
Ptprj	0,969246	0,009953	0,04	0,04	1
Rims2	0,969548	-0,00356	0,046	0,046	1
Mybbp1a	0,969565	0,056855	0,409	0,443	1
Chpf	0,969886	0,01649	0,088	0,089	1
Gm7160	0,970457	0,003246	0,017	0,017	1
Nras	0,970995	0,049098	0,655	0,705	1
Crtc2	0,971114	0,031914	0,202	0,207	1
Atxn2	0,971293	0,033954	0,719	0,766	1

Pcnx3	0,971366	0,018607	0,286	0,289	1
Soat1	0,971414	0,023186	0,218	0,219	1
Bcas3os1	0,971538	0,005604	0,013	0,013	1
Trio	0,971538	0,008913	0,013	0,013	1
B3galnt1	0,972291	0,033375	0,18	0,188	1
Suv39h2	0,972994	0,006749	0,04	0,04	1
Gm26710	0,973142	0,004764	0,017	0,017	1
Fndc4	0,973359	0,012566	0,048	0,049	1
Map2k3os	0,973525	0,005043	0,017	0,017	1
A330008L1	0,973525	0,006836	0,017	0,017	1
1700018L(	0,973526	0,010126	0,017	0,017	1
Smpd2	0,974093	0,020698	0,163	0,167	1
Neurl2	0,974164	0,0038	0,013	0,013	1
Gm10306	0,974164	0,009201	0,013	0,013	1
Gm42067	0,974164	0,005604	0,013	0,013	1
Nrd1	0,974483	0,008401	0,829	0,838	1
Aste1	0,975179	0,017295	0,095	0,097	1
Ep400	0,975362	0,057132	0,468	0,506	1
Fzd4	0,975486	0,00461	0,031	0,032	1
Ulk3	0,975649	0,016468	0,105	0,105	1
Insr	0,975667	0,056536	0,347	0,369	1
Cry2	0,975683	0,059375	0,277	0,295	1
Rtel1	0,9759	0,007205	0,062	0,063	1
Vps41	0,976393	0,048238	0,607	0,665	1
Dlg4	0,976459	0,011722	0,057	0,057	1
Stk36	0,97679	0,0038	0,013	0,013	1
Gm17586	0,97679	0,001993	0,013	0,013	1
Klhl25	0,97683	0,01346	0,081	0,082	1
Mfsd6	0,97876	0,034097	0,712	0,774	1
1700055D:	0,979417	0,000183	0,013	0,013	1
Cnga1	0,979417	0,000183	0,013	0,013	1
Tatdn2	0,979737	0,017764	0,193	0,198	1
Slc35d2	0,979855	-8,8E-05	0,013	0,013	1
Tmem69	0,980163	0,004582	0,055	0,055	1
Slc9b1	0,980293	-0,00216	0,013	0,013	1
C3	0,980293	-0,00216	0,013	0,013	1
Ksr1	0,980798	0,007025	0,231	0,234	1
Sema4g	0,980896	0,018741	0,077	0,078	1
Brd3	0,982049	0,041045	0,508	0,536	1
Epb41	0,982483	-0,01525	0,013	0,013	1
Mob3a	0,982856	0,024051	0,253	0,264	1
Farp1	0,983209	-0,00149	0,086	0,086	1
Parp2	0,983233	0,012054	0,171	0,173	1
Crkl	0,983656	0,046601	0,365	0,397	1
Reps2	0,983799	0,067412	0,327	0,357	1
Map1lc3a	0,983876	0,015247	0,917	0,922	1
Cdk18	0,984397	0,054724	0,666	0,707	1
B230369F2	0,984556	0,027179	0,081	0,082	1
Rras	0,985076	0,002392	0,097	0,097	1
Tmco4	0,985268	0,006932	0,028	0,027	1
A530017D:	0,985519	0,006942	0,132	0,133	1
Klhdc10	0,986157	0,022088	0,196	0,203	1

Tet1	0,987013	0,051908	0,332	0,352	1
Cenpn	0,987348	0,006202	0,046	0,046	1
Cc2d1a	0,987428	0,017176	0,143	0,146	1
Lgals4	0,98805	0,008216	0,046	0,046	1
Scrib	0,988095	0,036312	0,299	0,314	1
Pole2	0,989477	0,003113	0,028	0,027	1
Kifc2	0,989804	0,030045	0,172	0,177	1
Fam83b	0,989824	0,00107	0,029	0,03	1
Dhx57	0,989868	0,02858	0,145	0,148	1
Ywhah	0,990094	0,032722	0,593	0,62	1
Hirip3	0,990205	0,028518	0,178	0,184	1
Rhoc	0,990276	0,029605	0,435	0,46	1
Gm7694	0,990379	0,000803	0,028	0,027	1
Rpusd4	0,990518	0,003913	0,075	0,076	1
Neu3	0,990613	-0,00036	0,015	0,015	1
Hs1bp3	0,990691	0,022208	0,235	0,238	1
Pask	0,991021	-8,8E-05	0,015	0,015	1
Ptprg	0,991021	-8,8E-05	0,015	0,015	1
5033403F(	0,991021	-8,8E-05	0,015	0,015	1
Fermt2	0,991143	0,01188	0,958	0,973	1
Desi2	0,991363	0,029362	0,277	0,287	1
Ctbp1	0,991397	0,011353	0,848	0,854	1
Aars	0,991445	0,067278	0,525	0,58	1
Map3k12	0,991548	0,013824	0,163	0,167	1
Slc22a5	0,991897	0,013808	0,169	0,171	1
Kdm2b	0,992363	0,015531	0,211	0,217	1
Pecam1	0,992391	0,011652	0,031	0,032	1
Rps3	0,992489	0,026373	0,998	0,994	1
4931406P:	0,992576	0,026951	0,804	0,808	1
Ivns1abp	0,993042	0,066216	0,439	0,475	1
Pramef8	0,993248	0,004671	0,088	0,089	1
Cep350	0,993866	0,05657	0,466	0,513	1
Rab36	0,993878	0,001719	0,015	0,015	1
4430402I1	0,993878	0,001719	0,015	0,015	1
AC165953.	0,994473	0,033526	0,138	0,141	1
Arhgap35	0,994847	0,051834	0,389	0,418	1
Sec63	0,995982	0,013863	0,622	0,65	1
Man2a1	0,9963	0,020472	0,061	0,061	1
Vps37d	0,996379	0,001036	0,072	0,072	1
Gm14703	0,996735	0,005323	0,015	0,015	1
Npsr1	0,996735	0,006554	0,015	0,015	1
Nlrx1	0,996954	0,011688	0,046	0,046	1
Rftn1	0,997371	0,023389	0,675	0,679	1
Olfr46	0,997383	0,0087	0,029	0,03	1
Mapk7	0,998003	0,012838	0,189	0,192	1
Mrpl37	0,998261	0,006944	0,16	0,16	1
Palm	0,99869	0,027098	0,211	0,217	1
Ap1g2	0,998804	0,005003	0,044	0,044	1
Reck	0,998837	0,006653	0,029	0,03	1
Gm42595	0,998837	0,006653	0,029	0,03	1
Hid1	0,999034	0,055499	0,389	0,424	1
Etfbkmt	0,999128	0,0087	0,029	0,03	1

Zfp78	0,999128	0,0087	0,029	0,03	1
Sirt6	0,999285	0,010362	0,136	0,137	1
Neil1	0,999391	0,00626	0,117	0,118	1
Pou5f1	0,999592	0,007119	0,015	0,015	1
Spata6	0,99968	0,014302	0,105	0,105	1
Nr4a1	1	0,073319	0,127	0,131	1

	p_val	avg_logFC	pct.1	pct.2	p_val_adj
Xist	3,96E-41	0,9086	0,581	0,262	6,24E-37
Gm8797	2,19E-33	-0,31203	0,075	0,291	3,45E-29
Ddx3y	1,3E-23	-0,30596	0,333	0,589	2,05E-19
Tsix	4,26E-21	0,264717	0,25	0,065	6,72E-17
Gatm	1,69E-20	-0,16709	0,999	1	2,67E-16
Gstp1	1,44E-19	0,47719	0,984	0,937	2,27E-15
Etv1	1,62E-19	-0,27876	0,942	0,974	2,56E-15
Dbndd2	1,93E-18	0,228641	0,988	0,98	3,04E-14
Klf13	2,29E-18	0,272809	0,992	0,989	3,61E-14
Eif2s3y	9,97E-18	-0,24439	0,274	0,491	1,57E-13
Gm42205	4,72E-16	-0,10287	0,014	0,101	7,44E-12
Sparc	1,22E-14	0,318224	0,844	0,732	1,92E-10
Pex5l	4,08E-14	-0,2879	0,968	0,985	6,43E-10
Sept7	7,22E-13	-0,17487	0,998	1	1,14E-08
Gjc3	2,6E-12	-0,1897	0,987	0,997	4,11E-08
Ubc	4,21E-12	0,24384	0,998	0,998	6,64E-08
Hdac11	6,69E-12	0,226236	0,892	0,841	1,06E-07
Mt1	7,08E-12	0,417837	0,932	0,885	1,12E-07
Hsph1	8,63E-12	-0,17232	0,536	0,714	1,36E-07
Trim16	1,01E-11	0,103517	0,09	0,008	1,59E-07
Slc44a1	1,37E-11	0,147228	0,999	1	2,16E-07
Hnrnpa0	1,57E-11	0,194824	0,869	0,802	2,48E-07
Per1	3,84E-11	0,33121	0,34	0,197	6,06E-07
4933431E2	5,68E-11	-0,21362	0,633	0,732	8,97E-07
Uba52	1,51E-10	0,206528	0,764	0,662	2,39E-06
mt-Cytb	2,24E-10	-0,12875	0,999	1	3,53E-06
Hist2h2ac	2,52E-10	-0,05685	0,009	0,063	3,98E-06
Uty	2,65E-10	-0,12463	0,138	0,265	4,19E-06
Egr1	3,34E-10	1,120079	0,345	0,211	5,27E-06
Eml1	3,42E-10	-0,2076	0,677	0,777	5,39E-06
6330403K(	3,6E-10	-0,10851	0,026	0,098	5,68E-06
Gng11	4E-10	-0,16147	0,966	0,982	6,3E-06
Eif3j2	4,34E-10	0,1953	0,233	0,114	6,84E-06
Kdm5d	5,08E-10	-0,13081	0,11	0,223	8,02E-06
Npsr1	5,39E-10	-0,08985	0,015	0,075	8,51E-06
Atf3	5,93E-10	0,442802	0,146	0,049	9,36E-06
Hspa8	1,03E-09	-0,11115	0,999	1	1,62E-05
Gpc1	1,04E-09	0,254758	0,599	0,472	1,65E-05
Nacc2	1,15E-09	-0,18432	0,859	0,894	1,81E-05
mt-Atp6	1,59E-09	-0,1081	1	1	2,5E-05
Gpm6b	2,36E-09	-0,11356	1	1	3,73E-05
Cd63	2,36E-09	0,203117	0,933	0,917	3,73E-05
Atg4c	3,24E-09	-0,13841	0,427	0,577	5,11E-05
Usp31	3,64E-09	-0,20712	0,816	0,881	5,75E-05
Adgrl3	3,98E-09	-0,13934	0,194	0,325	6,27E-05
Gria2	4,04E-09	-0,11577	0,015	0,07	6,37E-05
D1Ertd622	4,35E-09	0,193819	0,912	0,849	6,87E-05
Ugt8a	4,4E-09	-0,13614	0,998	0,998	6,93E-05
Srsf5	4,99E-09	0,156073	0,974	0,945	7,87E-05
Efcab14	6,87E-09	-0,20029	0,982	0,992	0,000108
Aldoc	7,11E-09	-0,21478	0,276	0,42	0,000112

Hip1	7,87E-09	-0,13922	0,673	0,803	0,000124
Psmb8	1,28E-08	-0,57364	0,02	0,076	0,000202
Mt2	1,31E-08	0,384088	0,271	0,159	0,000207
Junb	1,36E-08	0,521429	0,472	0,341	0,000215
Jam3	1,71E-08	-0,12933	0,958	0,982	0,00027
Hebp1	1,99E-08	0,217028	0,711	0,598	0,000314
Fam171b	2,14E-08	-0,1386	0,954	0,964	0,000337
Mbp	2,2E-08	0,105938	0,999	1	0,000347
Cd274	2,33E-08	-0,39329	0,018	0,072	0,000368
Scd2	2,6E-08	-0,13746	0,999	1	0,00041
Psat1	3E-08	-0,13811	0,966	0,987	0,000474
Spock3	4,25E-08	-0,19256	0,843	0,889	0,00067
Crim1	5,47E-08	-0,16133	0,342	0,47	0,000863
Scnm1	6,34E-08	0,153434	0,539	0,421	0,001
mt-Nd4	6,71E-08	-0,10873	0,999	1	0,001058
Jund	6,98E-08	0,171548	0,999	0,998	0,001101
S100a16	7,13E-08	-0,15009	0,969	0,992	0,001125
Rab4a	7,2E-08	-0,16394	0,467	0,597	0,001136
AC149090.	8,36E-08	-0,14902	0,772	0,857	0,001318
Gm43241	8,8E-08	-0,03632	0,002	0,034	0,001389
Ldhb	1,05E-07	-0,20043	0,634	0,746	0,001657
Oxr1	1,1E-07	-0,16562	0,731	0,811	0,001728
Spred1	1,15E-07	-0,15936	0,749	0,816	0,001812
Abca8a	1,43E-07	-0,23451	0,247	0,354	0,002263
Lrrn3	1,47E-07	-0,09539	0,071	0,151	0,002315
Slc25a29	1,57E-07	-0,09949	0,183	0,296	0,002475
Slc6a9	1,62E-07	-0,14098	0,883	0,935	0,002555
Mbnl2	1,79E-07	-0,09478	0,999	1	0,002821
Dixdc1	1,81E-07	-0,13447	0,919	0,969	0,002851
Tmeff1	1,97E-07	-0,11962	0,92	0,969	0,003101
Gpx4	2,06E-07	0,112104	0,999	1	0,003255
Kcnj10	2,24E-07	-0,15975	0,976	0,99	0,00353
H3f3b	2,46E-07	0,154566	0,998	0,993	0,00388
Ahcyl1	2,54E-07	-0,12953	0,811	0,872	0,004006
Ogdhl	2,66E-07	-0,19962	0,205	0,317	0,004199
Sppl2b	2,73E-07	-0,13325	0,28	0,398	0,004307
Inpp5f	2,77E-07	-0,13463	0,882	0,927	0,004373
Fgfr2	3,24E-07	-0,13153	0,97	0,982	0,005109
Hbb-bs	3,42E-07	2,147944	0,041	0	0,005395
Gstm7	4,27E-07	-0,14471	0,877	0,925	0,00674
Ctsk	4,36E-07	-0,13744	0,368	0,486	0,006874
March1	4,51E-07	-0,13825	0,31	0,431	0,007108
Rasal2	4,91E-07	-0,154	0,494	0,589	0,007743
Dbt	6,04E-07	-0,15055	0,33	0,439	0,009527
Asah2	6,6E-07	-0,12625	0,195	0,299	0,010414
Hhatl	6,93E-07	0,204129	0,486	0,385	0,01093
Gm47283	6,96E-07	0,147226	0,714	0,626	0,010979
Pcdh15	7,44E-07	-0,11313	0,09	0,171	0,011737
Slu7	7,56E-07	-0,16104	0,791	0,878	0,011921
Pou3f1	7,65E-07	0,189624	0,743	0,688	0,012068
Tap2	8,3E-07	-0,21447	0,032	0,088	0,013094
1110038F1	8,44E-07	-0,11352	0,408	0,537	0,013313

Ncam2	9,37E-07	-0,13595	0,688	0,789	0,014786
Crtac1	9,75E-07	0,15753	0,328	0,215	0,015376
Mapk8ip1	9,78E-07	0,109853	0,999	1	0,015435
Sgta	1,03E-06	-0,11805	0,888	0,928	0,016277
mt-Co2	1,08E-06	-0,09086	1	1	0,017051
Slc12a2	1,25E-06	-0,11344	0,998	1	0,01968
Dclk1	1,35E-06	-0,13537	0,181	0,281	0,021339
Cdh19	1,47E-06	-0,12518	0,37	0,494	0,023198
Il1rap	1,62E-06	-0,1149	0,871	0,928	0,025501
Zfp445	1,65E-06	-0,13745	0,668	0,758	0,025983
Clic5	1,73E-06	-0,10625	0,101	0,182	0,027242
Tac1	1,82E-06	-0,033	0,007	0,041	0,028679
Asrgl1	1,82E-06	-0,13599	0,687	0,772	0,028772
Elovl5	1,83E-06	-0,15307	0,776	0,842	0,028864
Parp14	2,17E-06	-0,08783	0,005	0,036	0,0342
mt-Co3	2,21E-06	-0,08895	1	1	0,034875
Prex1	2,36E-06	-0,14406	0,492	0,593	0,037292
Ier2	2,38E-06	0,43814	0,19	0,107	0,037571
Kcnk13	2,39E-06	-0,11336	0,725	0,816	0,037736
Psmb9	2,46E-06	-0,21187	0,015	0,055	0,038856
Car2	2,61E-06	-0,11559	1	1	0,041222
Qdpr	2,69E-06	-0,17116	0,99	0,992	0,042484
Trim2	2,73E-06	-0,12373	0,974	0,985	0,043009
Dock9	2,76E-06	-0,15965	0,763	0,824	0,043512
Dock5	2,91E-06	-0,13067	0,562	0,66	0,045949
Kif21a	2,93E-06	-0,11412	0,954	0,963	0,046229
Garem2	2,93E-06	-0,11949	0,115	0,197	0,046238
Bloc1s1	3,16E-06	0,156495	0,696	0,629	0,049917
Acap2	3,46E-06	-0,11219	0,806	0,878	0,054539
Cpox	3,55E-06	-0,18152	0,974	0,979	0,056031
9330159F1	3,65E-06	-0,1146	0,389	0,514	0,057513
Pcdh9	4,18E-06	-0,11753	0,995	0,998	0,065957
Adgrb3	4,61E-06	-0,11373	0,134	0,221	0,072762
Gm11808	4,7E-06	0,140947	0,616	0,52	0,07413
Tacc1	4,83E-06	-0,12696	0,644	0,741	0,076145
Adam19	5,41E-06	-0,12692	0,324	0,433	0,085284
Abcc5	5,62E-06	-0,12137	0,738	0,839	0,088688
Aif1l	5,7E-06	-0,11915	0,426	0,538	0,089988
Hhip	5,75E-06	-0,15552	0,581	0,66	0,090719
Gatsl2	5,85E-06	-0,09531	0,225	0,328	0,092334
Ybx3	6E-06	-0,11176	0,837	0,912	0,094724
Scg2	6,01E-06	-0,03728	0,009	0,042	0,094771
Dnm3	6,07E-06	-0,13421	0,961	0,974	0,095702
Tbc1d5	6,67E-06	-0,12662	0,627	0,72	0,105289
Ccdc91	6,68E-06	-0,10711	0,228	0,327	0,105414
Pak1	6,71E-06	0,117575	0,976	0,974	0,105807
Sil1	6,79E-06	-0,12409	0,361	0,465	0,107184
Atp9a	6,83E-06	-0,11444	0,901	0,932	0,107768
Lgr4	7,13E-06	-0,08467	0,163	0,255	0,112413
Lyz2	7,33E-06	0,091761	0,157	0,081	0,115712
Megf10	7,96E-06	-0,13671	0,592	0,673	0,125534
Lats2	7,97E-06	-0,11002	0,258	0,361	0,12581

Adam10	8,15E-06	-0,10054	0,795	0,868	0,128509
Cdc42ep1	8,69E-06	-0,10725	0,728	0,816	0,13703
Pak7	8,85E-06	-0,14507	0,629	0,714	0,139662
Npepps	8,95E-06	-0,11041	0,868	0,885	0,141178
Lrrc1	9,3E-06	-0,12107	0,462	0,572	0,146717
Fam234a	9,75E-06	-0,11046	0,453	0,576	0,153843
Gm15446	9,93E-06	-0,06824	0,073	0,14	0,156714
Notch1	1E-05	0,07863	0,077	0,024	0,158046
Ndrg2	1,02E-05	-0,15497	0,264	0,363	0,160179
Pgbd5	1,03E-05	-0,10923	0,342	0,45	0,163248
St3gal4	1,04E-05	0,131684	0,789	0,707	0,164109
Ubfd1	1,05E-05	-0,08354	0,15	0,237	0,16544
Add3	1,1E-05	-0,14883	0,835	0,88	0,173588
Sik3	1,1E-05	0,130403	0,911	0,881	0,174043
Glrb	1,12E-05	-0,14366	0,636	0,696	0,177127
Bcat1	1,14E-05	-0,13811	0,347	0,452	0,180098
Armt1	1,17E-05	-0,08214	0,137	0,221	0,185264
Hmgcs1	1,2E-05	0,373447	0,937	0,951	0,189424
Dab1	1,24E-05	-0,0714	0,051	0,109	0,195144
Cldn11	1,24E-05	0,085305	0,999	1	0,195492
Prkca	1,38E-05	-0,12428	0,69	0,774	0,218254
Gpd2	1,43E-05	-0,16838	0,497	0,576	0,224809
Myo6	1,46E-05	-0,09102	0,985	0,995	0,229882
Rpl10-ps3	1,46E-05	0,091566	0,239	0,15	0,231101
Npas3	1,5E-05	-0,11172	0,226	0,32	0,236085
Pigc	1,5E-05	-0,0791	0,165	0,254	0,236323
Cln3	1,53E-05	0,153037	0,725	0,66	0,241992
Csmd3	1,66E-05	-0,12079	0,447	0,538	0,262019
Slc24a4	1,75E-05	0,04908	0,033	0,002	0,276837
Dclk3	1,77E-05	-0,06432	0,081	0,15	0,279863
Gng12	1,84E-05	-0,09312	0,818	0,888	0,290549
Klhl4	1,87E-05	-0,14221	0,674	0,735	0,294918
Dnah14	1,94E-05	-0,08534	0,231	0,333	0,306639
Eif3j1	1,96E-05	-0,09381	0,764	0,847	0,309605
Kat2b	1,96E-05	-0,1311	0,745	0,821	0,309656
Glo1	2,04E-05	-0,14313	0,844	0,865	0,322486
Wasf3	2,1E-05	-0,09712	0,184	0,273	0,330854
Cd82	2,13E-05	0,125372	0,974	0,976	0,335845
Sqle	2,15E-05	0,243762	0,663	0,602	0,339121
Cab39l	2,16E-05	-0,11704	0,278	0,377	0,340927
Atxn7l3b	2,18E-05	-0,08919	0,978	0,99	0,34374
Fam168a	2,3E-05	-0,12678	0,97	0,979	0,363602
Tmsb10	2,4E-05	0,165712	0,614	0,533	0,378795
Bin1	2,46E-05	-0,1052	0,997	0,997	0,38863
Sall1	2,54E-05	-0,11433	0,409	0,52	0,400606
Igsf11	2,58E-05	-0,12694	0,687	0,772	0,407485
Rpsa	2,64E-05	0,115988	0,999	1	0,41647
Sat2	2,65E-05	-0,09845	0,303	0,407	0,417968
Sema3c	2,68E-05	-0,03534	0,009	0,039	0,422084
Osbpl7	2,69E-05	-0,13417	0,395	0,475	0,424546
Fhdc1	2,72E-05	-0,07823	0,145	0,229	0,42858
Erbb4	2,75E-05	-0,11786	0,295	0,398	0,433102

Cdc37l1	2,76E-05	-0,09747	0,997	0,998	0,434774
Btc	2,8E-05	-0,06964	0,124	0,202	0,441548
Nbas	2,99E-05	0,172281	0,724	0,68	0,471668
Itgav	3,04E-05	-0,15064	0,784	0,818	0,479011
Cdk5rap2	3,12E-05	-0,11059	0,603	0,691	0,492277
Eps15	3,18E-05	-0,12335	0,871	0,907	0,501586
Synpo	3,19E-05	0,102056	0,229	0,146	0,503121
Paip1	3,26E-05	-0,08593	0,698	0,772	0,514846
Gbp7	3,38E-05	-0,39229	0,05	0,102	0,53379
Frmd4a	3,39E-05	-0,09351	0,379	0,483	0,535182
Slc38a2	3,49E-05	-0,10414	0,973	0,992	0,550228
Fos	3,49E-05	0,652335	0,671	0,593	0,551219
Tmem229a	3,53E-05	-0,12366	0,915	0,928	0,557293
Tagln2	3,59E-05	0,177301	0,289	0,198	0,566663
Elavl3	3,61E-05	-0,09937	0,998	1	0,569351
Robo1	3,65E-05	-0,11497	0,303	0,403	0,575891
Gstm1	3,75E-05	-0,10452	0,649	0,735	0,591003
Galnt7	3,77E-05	-0,13207	0,557	0,636	0,593994
Arhgap5	3,79E-05	-0,108	0,947	0,943	0,598532
Omg	3,84E-05	-0,12211	0,759	0,813	0,606271
Serpind1	3,89E-05	-0,16424	0,209	0,293	0,61334
Snca	4,06E-05	-0,08103	0,165	0,249	0,640976
Micall1	4,12E-05	0,121042	0,978	0,972	0,649534
Amph	4,26E-05	-0,08377	0,346	0,45	0,671279
Isoc2b	4,26E-05	-0,04194	0,031	0,076	0,671625
Sst	4,45E-05	-0,03766	0,015	0,049	0,701347
Picalm	4,6E-05	-0,11116	0,944	0,974	0,725898
Nap1l3	4,66E-05	-0,10793	0,163	0,244	0,735881
Bcas1	4,85E-05	0,204535	0,973	0,969	0,76475
Cmpk2	5,09E-05	-0,06248	0,009	0,037	0,802267
Slc12a4	5,29E-05	-0,06034	0,115	0,189	0,834347
Tapbpl	5,5E-05	-0,12712	0,044	0,093	0,868035
Epb41l3	5,51E-05	-0,10021	0,946	0,984	0,86906
Kif5a	5,56E-05	0,131177	0,317	0,228	0,877592
Sqor	5,58E-05	-0,06424	0,066	0,124	0,880585
Ogt	5,67E-05	-0,09855	0,825	0,876	0,894901
Tmem56	5,78E-05	-0,09707	0,172	0,252	0,912416
Jakmip2	5,8E-05	-0,09663	0,277	0,371	0,915696
Dpy19l1	5,93E-05	-0,09198	0,996	1	0,935067
Fbxo44	5,97E-05	-0,10457	0,366	0,468	0,94173
Parp12	5,97E-05	-0,10603	0,102	0,167	0,942134
Gm43796	6,18E-05	0,061314	0,096	0,042	0,974862
Rhob	6,36E-05	0,097298	0,999	1	1
Gm43242	6,46E-05	-0,07903	0,132	0,207	1
Dusp15	6,59E-05	-0,11992	0,572	0,646	1
4931406C(	6,6E-05	-0,11997	0,409	0,494	1
Hcn2	6,63E-05	-0,15382	0,901	0,925	1
Fbn2	6,65E-05	-0,05997	0,03	0,073	1
Rnf44	6,71E-05	-0,10162	0,4	0,502	1
Gbp3	6,8E-05	-0,2077	0,021	0,057	1
Mrpl48	6,81E-05	-0,0959	0,811	0,863	1
Nav3	6,81E-05	-0,10839	0,14	0,215	1

Ifit3	7,16E-05	-0,22668	0,012	0,042	1
Bbx	7,27E-05	-0,11053	0,57	0,657	1
S100a10	7,28E-05	0,134048	0,17	0,099	1
Cd9	7,32E-05	0,180962	0,911	0,867	1
Plp1	7,37E-05	-0,06333	0,999	1	1
Tubb4a	7,54E-05	-0,10228	0,999	0,998	1
Cyp2j6	7,56E-05	-0,12726	0,29	0,38	1
Erap1	7,63E-05	-0,0703	0,066	0,122	1
Gadd45g	7,75E-05	0,424726	0,148	0,085	1
Zrsr1	7,81E-05	-0,12817	0,387	0,483	1
Ube2l6	7,81E-05	-0,03362	0,013	0,044	1
Gm42418	7,84E-05	-0,03043	1	1	1
Pkp4	8,03E-05	0,153267	0,836	0,782	1
Egr2	8,1E-05	0,378782	0,16	0,094	1
Acot11	8,18E-05	0,087637	0,141	0,078	1
H2-M3	8,35E-05	-0,03456	0,002	0,021	1
Lhfpl2	8,43E-05	-0,08992	0,132	0,203	1
Ptch1	8,54E-05	-0,09338	0,44	0,54	1
Tmem87a	8,56E-05	0,116497	0,403	0,319	1
Ndufa8	8,92E-05	-0,09411	0,709	0,802	1
Egr3	9,35E-05	0,125624	0,077	0,029	1
Tvp23a	9,43E-05	-0,08437	0,101	0,166	1
Hspa2	9,68E-05	0,139894	0,427	0,345	1
Slc25a12	9,83E-05	-0,08467	0,393	0,507	1
C4b	0,0001	0,159813	0,85	0,802	1
Ddr1	0,000103	0,112262	0,987	0,984	1
Lrp1b	0,000104	-0,10039	0,917	0,938	1
Oasl2	0,000104	-0,09123	0,009	0,036	1
Cdc14a	0,000105	-0,07109	0,154	0,233	1
Abat	0,000106	-0,0785	0,238	0,332	1
Macf1	0,000109	-0,13017	0,766	0,831	1
Tcaim	0,00011	-0,0851	0,219	0,304	1
Kif5b	0,000116	0,132369	0,921	0,894	1
Pptc7	0,000117	-0,0869	0,206	0,288	1
Cotl1	0,000118	-0,08877	0,206	0,293	1
Fbln2	0,000118	0,103502	0,123	0,065	1
Plcl1	0,000118	-0,09988	0,96	0,959	1
Tap1	0,000122	-0,27292	0,031	0,072	1
Sox4	0,000122	0,23664	0,131	0,072	1
Rpl13a	0,000122	0,137566	0,934	0,911	1
Folh1	0,000126	-0,12589	0,398	0,488	1
Aspa	0,000131	-0,09169	0,983	0,997	1
Ptms	0,000135	0,156858	0,831	0,779	1
Metrn	0,000136	-0,08589	0,761	0,824	1
Opcml	0,000137	-0,0878	0,152	0,224	1
Zfp536	0,00014	0,120715	0,935	0,906	1
Igsf8	0,000154	0,141041	0,741	0,691	1
Map4k5	0,000155	-0,09843	0,674	0,746	1
Itgb8	0,000155	-0,1091	0,754	0,803	1
Calm1	0,000157	-0,07454	0,998	1	1
Kif1b	0,00016	-0,0792	0,999	1	1
Ccdc32	0,000164	-0,08564	0,274	0,361	1

Anxa2	0,000166	0,142626	0,342	0,257	1
Slc36a4	0,00017	-0,08095	0,207	0,286	1
Mtus1	0,000172	-0,11218	0,798	0,849	1
Erbin	0,000173	0,118729	0,964	0,953	1
Rap2a	0,000173	-0,1065	0,445	0,528	1
Nap1l5	0,000173	-0,1096	0,843	0,894	1
Tmem140	0,000174	-0,11115	0,028	0,067	1
Ece1	0,000178	-0,05689	0,053	0,102	1
Ppp4r3b	0,000178	-0,08662	0,519	0,615	1
Paics	0,000178	-0,08432	0,846	0,875	1
Chdh	0,000179	-0,08268	0,237	0,322	1
Tmem88b	0,000179	-0,0687	0,999	1	1
March8	0,000182	-0,07684	0,875	0,904	1
Tmod2	0,000184	-0,14944	0,81	0,803	1
Padi2	0,000186	0,083239	0,873	0,759	1
Parp10	0,000186	-0,04233	0,004	0,024	1
Cd59a	0,000187	0,105802	0,479	0,379	1
Zfp952	0,000187	-0,08133	0,13	0,197	1
Spata13	0,000192	-0,07346	0,132	0,202	1
Plekhf1	0,000194	0,106634	0,32	0,241	1
Fam222a	0,000194	0,114084	0,574	0,506	1
Rpl27-ps3	0,000199	-0,06233	0,095	0,156	1
Vwa1	0,000199	0,156252	0,31	0,231	1
Afap1	0,000202	0,120681	0,49	0,407	1
Rnf103	0,000204	-0,10113	0,359	0,444	1
Fosb	0,000205	0,4628	0,242	0,167	1
Ermn	0,000207	-0,06781	0,999	1	1
Socs3	0,000211	0,25106	0,287	0,21	1
Ano10	0,000216	-0,07992	0,406	0,501	1
Tnfsf9	0,000222	0,101739	0,105	0,054	1
Rgmb	0,000222	-0,09534	0,32	0,411	1
Zmiz1	0,000223	0,124272	0,608	0,525	1
Eef1akmt2	0,000224	-0,08198	0,256	0,343	1
Hepacam	0,000231	-0,10217	0,948	0,943	1
Col6a2	0,000234	0,154477	0,139	0,081	1
Hnrnpdl	0,000235	0,104836	0,962	0,938	1
Glul	0,000238	-0,13292	0,998	0,998	1
Ccdc88a	0,000242	-0,09686	0,976	0,987	1
P2rx4	0,000243	-0,08841	0,397	0,489	1
Ankub1	0,000246	-0,1532	0,356	0,426	1
Penk	0,000255	-0,01515	0,001	0,016	1
Mpzl1	0,000256	-0,06712	0,702	0,784	1
Ahnak	0,000263	0,081053	0,038	0,008	1
Gstm5	0,000266	-0,10774	0,948	0,95	1
Btg2	0,000275	0,429354	0,307	0,233	1
Evi2a	0,00028	-0,08204	0,978	0,992	1
Hspe1	0,00028	-0,0981	0,889	0,927	1
Pcdhb7	0,000281	-0,04082	0,024	0,059	1
Ncam1	0,000284	0,099461	0,999	1	1
Ctnnbip1	0,000284	-0,09724	0,73	0,772	1
Rida	0,000287	-0,09739	0,468	0,554	1
Ddx60	0,000288	-0,05519	0,006	0,028	1

Msmo1	0,00029	0,23234	0,852	0,841	1
Shb	0,000295	0,082524	0,231	0,158	1
Psme1	0,000299	-0,13508	0,161	0,229	1
Tmprss5	0,0003	-0,08559	0,309	0,398	1
Cpd	0,000301	-0,13146	0,971	0,976	1
Kctd4	0,000303	-0,11101	0,462	0,551	1
Shc4	0,000304	-0,04336	0,053	0,101	1
Sh3bp2	0,000305	0,070043	0,16	0,098	1
Dhcr24	0,000318	0,230596	0,58	0,535	1
Irf2	0,000327	-0,0862	0,443	0,535	1
Ank2	0,000327	-0,06732	0,996	1	1
Ptprd	0,000332	-0,08142	0,998	1	1
Cyp27a1	0,000334	-0,14035	0,427	0,493	1
Sgk3	0,000336	-0,08887	0,386	0,476	1
Poc1a	0,000339	0,152423	0,464	0,392	1
Sars	0,000343	-0,07941	0,823	0,873	1
Hist2h4	0,000347	-0,03685	0,03	0,068	1
Shisa4	0,00035	-0,08683	0,974	0,989	1
Nfic	0,000356	0,116178	0,925	0,915	1
Dsel	0,000358	-0,08489	0,169	0,241	1
Acer2	0,000361	-0,0544	0,073	0,125	1
Enpp3	0,000373	-0,04316	0,043	0,086	1
Hapln2	0,000377	-0,10039	0,957	0,964	1
Map6d1	0,000379	-0,09472	0,759	0,813	1
Ldah	0,000386	-0,08569	0,7	0,777	1
3830406C:	0,000386	-0,07603	0,373	0,467	1
Spp1	0,000392	0,03804	0,048	0,015	1
Gbp6	0,000394	-0,04835	0,004	0,023	1
Nprl2	0,000411	-0,06991	0,235	0,314	1
Iah1	0,000423	-0,08483	0,298	0,385	1
Ccdc77	0,000435	-0,0532	0,172	0,247	1
Brd2	0,000436	0,167522	0,827	0,78	1
Flrt3	0,000438	-0,05154	0,068	0,119	1
Isg15	0,000442	-0,05799	0,002	0,018	1
Gbp2	0,000442	-0,20876	0,002	0,018	1
Prr5l	0,000448	-0,0955	0,925	0,941	1
Rap2b	0,00045	-0,0837	0,17	0,241	1
Mpp2	0,000454	-0,09606	0,571	0,644	1
Igtp	0,000454	-0,46156	0,051	0,094	1
Arsb	0,000456	-0,10284	0,329	0,41	1
Hspg2	0,000469	0,050042	0,036	0,008	1
Mlst8	0,00047	-0,07539	0,16	0,228	1
Rbm39	0,000477	0,083822	0,995	0,985	1
Cat	0,000488	-0,14133	0,594	0,646	1
Fmnl2	0,000488	-0,11647	0,898	0,899	1
Zfp62	0,0005	-0,07743	0,38	0,473	1
Rasgrp3	0,000502	-0,11542	0,799	0,87	1
Idi1	0,000502	0,222579	0,72	0,681	1
Ube2b	0,000505	-0,07733	0,833	0,888	1
Ywhab	0,000508	-0,08475	0,963	0,963	1
Gnai2	0,000509	0,072908	0,996	0,998	1
A930006I0	0,000513	-0,05421	0,015	0,042	1

Cab39	0,000515	-0,09007	0,644	0,725	1
Pdk2	0,000515	-0,0927	0,396	0,488	1
mt-Nd3	0,000535	-0,05266	0,999	1	1
Fam107a	0,000546	-0,03572	0,015	0,042	1
Zfp942	0,000548	-0,05515	0,132	0,198	1
B3galt5	0,00055	-0,12254	0,731	0,777	1
Ttll5	0,000553	-0,08478	0,401	0,486	1
Meg3	0,000553	-0,03476	0,03	0,067	1
Tubg2	0,000555	-0,05545	0,066	0,114	1
Parp8	0,000562	-0,06293	0,144	0,21	1
Ppwd1	0,000594	-0,07965	0,341	0,428	1
Magt1	0,000594	-0,10534	0,93	0,954	1
Lrrtm1	0,000611	-0,01355	0,001	0,015	1
Snap25	0,000627	-0,03552	0,029	0,065	1
Plp2	0,00063	0,069836	0,099	0,052	1
Adgrl1	0,000631	-0,06158	0,111	0,171	1
Rps19	0,000633	0,089013	0,978	0,985	1
Slc20a2	0,000642	-0,09	0,771	0,837	1
Tmem258	0,000643	-0,0856	0,901	0,925	1
Rpl3	0,000654	0,073577	0,997	0,993	1
Hspa4l	0,000656	-0,09796	0,585	0,663	1
Col27a1	0,000673	0,087637	0,114	0,063	1
Lmna	0,000675	0,155978	0,725	0,68	1
Dnajb1	0,000692	0,298578	0,538	0,462	1
Runx2	0,000693	0,054885	0,059	0,023	1
Bace1	0,000704	-0,09657	0,815	0,865	1
Thsd7a	0,000712	-0,10011	0,309	0,392	1
Lztfl1	0,000718	-0,09328	0,425	0,512	1
Rhbdd1	0,000731	-0,0634	0,146	0,211	1
Rps19bp1	0,000731	-0,0614	0,348	0,444	1
Dusp3	0,000732	-0,10516	0,863	0,894	1
Ubb	0,000741	0,088872	0,999	1	1
Pqlc2	0,000743	0,07446	0,18	0,119	1
St18	0,000748	-0,0878	0,816	0,831	1
Gm29455	0,000748	-0,06393	0,166	0,234	1
Psmd8	0,00076	-0,07783	0,688	0,79	1
Lman2l	0,000761	-0,06621	0,237	0,314	1
Ptprs	0,00077	0,111672	0,351	0,276	1
Ppp1cc	0,000774	-0,12125	0,723	0,764	1
Gmppb	0,000792	-0,06246	0,133	0,195	1
Rpl27	0,000803	0,085194	0,964	0,954	1
Vwa5a	0,000811	-0,08238	0,31	0,385	1
Fam118a	0,000815	-0,06514	0,121	0,179	1
Paqr8	0,000816	-0,15091	0,554	0,613	1
Cntfr	0,00082	0,12623	0,421	0,351	1
Rnf114	0,000828	-0,07833	0,33	0,41	1
Ak2	0,00083	-0,09076	0,409	0,483	1
Gas7	0,000842	0,087154	0,919	0,904	1
Erg28	0,000848	-0,07175	0,664	0,737	1
Nckap5	0,000852	0,074399	0,134	0,081	1
Sel1l3	0,000856	-0,08241	0,126	0,184	1
Wdr7	0,000869	-0,06737	0,281	0,361	1

Idnk	0,000876	-0,12061	0,235	0,306	1
Ttc14	0,000879	-0,09702	0,759	0,828	1
Zc3h3	0,000882	-0,04535	0,108	0,166	1
Ankrd11	0,000893	-0,08809	0,846	0,898	1
Hnrnpl	0,000893	0,108671	0,877	0,846	1
Rasip1	0,000894	0,066732	0,079	0,037	1
Dusp7	0,000898	-0,11196	0,586	0,647	1
Rap1a	0,000909	0,083126	0,995	0,993	1
Aplp1	0,000912	-0,05849	1	1	1
Inpp5j	0,000924	-0,05498	0,149	0,215	1
Ptprn	0,000927	0,117325	0,274	0,208	1
Nlrc5	0,000943	-0,07899	0,046	0,086	1
Leprot	0,000944	-0,07578	0,646	0,724	1
Hspa5	0,000947	-0,09863	0,935	0,964	1
Grhpr	0,000948	-0,08105	0,625	0,715	1
Nol3	0,00095	0,10099	0,385	0,301	1
Lbh	0,000952	0,123224	0,882	0,834	1
Tmem242	0,000962	-0,06652	0,292	0,376	1
Ldlr	0,000964	0,296723	0,436	0,377	1
Tox	0,000969	-0,03863	0,053	0,096	1
Tcn2	0,000978	-0,08094	0,282	0,358	1
Zfp36	0,000984	0,310641	0,312	0,244	1
St8sia1	0,000988	-0,02523	0,011	0,034	1
Cacna1h	0,000998	0,087747	0,09	0,047	1
Pcyt1b	0,001006	-0,09982	0,364	0,436	1
B230118H(	0,001006	-0,09093	0,628	0,686	1
Golph3l	0,00101	-0,07049	0,211	0,281	1
Srsf2	0,001019	0,103699	0,97	0,943	1
Nln	0,00102	0,080896	0,229	0,163	1
Sgms1	0,001022	-0,06608	0,303	0,387	1
Txndc12	0,001038	-0,07174	0,445	0,538	1
Slpi	0,001044	0,080578	0,164	0,107	1
Mecr	0,001044	-0,05127	0,133	0,195	1
Scarb2	0,001045	0,100265	0,992	0,992	1
Lgalsl	0,001046	0,038962	0,08	0,039	1
Mvk	0,00105	0,107105	0,427	0,356	1
Nfkbiz	0,001054	0,088389	0,095	0,05	1
Insc	0,001079	-0,06911	0,851	0,888	1
Bcas1os1	0,001084	0,035913	0,039	0,011	1
Rnf170	0,001085	-0,06843	0,244	0,32	1
9330182L(	0,001086	-0,10612	0,483	0,558	1
Ubald2	0,001086	-0,07264	0,508	0,585	1
Dpyd	0,001117	-0,08163	0,16	0,224	1
Kcnq3	0,001139	-0,10421	0,685	0,738	1
Tspan7	0,001164	-0,08323	0,406	0,494	1
Piga	0,001164	-0,09105	0,508	0,579	1
Nmnat1	0,001174	-0,04174	0,06	0,104	1
Gripap1	0,00118	-0,07987	0,472	0,553	1
Csnk1e	0,001188	-0,07532	0,7	0,774	1
Rnf130	0,001195	-0,07272	0,977	0,984	1
Enpp1	0,001198	-0,09581	0,33	0,405	1
Spock1	0,001198	-0,11528	0,74	0,785	1

Glod4	0,001202	-0,08044	0,793	0,837	1
Qpct	0,001202	0,099692	0,501	0,429	1
Tspan9	0,001205	-0,10354	0,258	0,33	1
Pcyt2	0,001221	0,122798	0,911	0,902	1
Akirin1	0,001222	-0,07985	0,669	0,751	1
Slc46a2	0,001245	-0,03627	0,033	0,068	1
Cacybp	0,001246	-0,08159	0,689	0,771	1
Slc25a27	0,001268	-0,14075	0,411	0,48	1
Jpt2	0,001278	-0,06882	0,367	0,455	1
Tmed10	0,001293	0,059291	0,999	1	1
Zcchc24	0,001297	-0,07373	0,817	0,867	1
Spry2	0,001301	-0,06798	0,28	0,356	1
Tax1bp1	0,001304	-0,10238	0,836	0,875	1
Ralbp1	0,001318	-0,08379	0,79	0,846	1
Pcnx	0,001319	-0,05705	0,276	0,356	1
Snx13	0,001327	-0,07079	0,374	0,455	1
Pantr1	0,001365	-0,05743	0,372	0,46	1
Ephb2	0,001367	0,051015	0,051	0,02	1
Nenf	0,00137	0,094002	0,877	0,855	1
Rtn1	0,001375	-0,04138	0,03	0,063	1
Zfp787	0,001379	-0,0573	0,214	0,286	1
Atg3	0,001384	-0,06424	0,792	0,828	1
Rpl19	0,00139	0,072064	0,997	0,998	1
Myo1d	0,001405	-0,08096	0,805	0,837	1
Aldh3b1	0,001406	0,079579	0,323	0,247	1
Fkbp4	0,001414	-0,07639	0,798	0,852	1
Ncald	0,001421	-0,08976	0,842	0,888	1
Adgrv1	0,001424	-0,10439	0,264	0,33	1
Fsd1l	0,001425	-0,08123	0,562	0,613	1
mt-Co1	0,001431	-0,06376	1	1	1
Col6a1	0,001435	0,113979	0,111	0,063	1
Mtmr2	0,001439	0,096112	0,782	0,74	1
Oaz1	0,001451	-0,07206	0,99	0,995	1
Gm39318	0,001455	-0,02042	0,007	0,026	1
Fpgt	0,001457	-0,07989	0,234	0,302	1
Carnmt1	0,001473	-0,06498	0,232	0,302	1
Copa	0,001474	-0,08057	0,713	0,761	1
Sdr39u1	0,001507	0,042816	0,077	0,037	1
Pgp	0,001507	-0,08475	0,895	0,906	1
Mospd1	0,001509	-0,07097	0,16	0,223	1
Nek9	0,001517	-0,06193	0,407	0,502	1
Cic	0,001517	0,110572	0,523	0,46	1
2010315B(	0,001525	-0,04941	0,09	0,141	1
Dusp26	0,001526	-0,06124	0,93	0,958	1
Socs4	0,001529	-0,07439	0,265	0,338	1
Rapgef5	0,001531	-0,13337	0,436	0,511	1
App	0,001555	-0,05438	0,999	1	1
Gas6	0,001559	0,075426	0,255	0,187	1
Atp2a2	0,001576	-0,07971	0,868	0,898	1
Pon2	0,001578	-0,07813	0,594	0,657	1
Olfr338	0,001592	-0,00971	0	0,01	1
Gm12953	0,001592	-0,00971	0	0,01	1

Gm42743	0,001592	-0,01453	0	0,01	1
Zbp1	0,001592	-0,04297	0	0,01	1
Tgfa	0,001596	-0,06798	0,823	0,867	1
Dusp19	0,001623	-0,0697	0,301	0,376	1
Eif3c	0,001628	0,084971	0,946	0,925	1
Cebpz	0,001636	-0,07466	0,574	0,642	1
Ankmy1	0,001641	0,049955	0,076	0,037	1
mt-Nd4l	0,001641	-0,06305	0,992	0,99	1
Aspdh	0,001642	0,025388	0,029	0,007	1
Ttyh2	0,001643	-0,05874	0,986	0,99	1
Neo1	0,001648	-0,06694	0,781	0,852	1
Ostc	0,001675	-0,07452	0,499	0,576	1
Fnta	0,001704	-0,07268	0,834	0,867	1
Tmem125	0,001716	-0,09234	0,805	0,837	1
Serpinb1a	0,001717	-0,11281	0,904	0,938	1
Hipk2	0,00172	0,087644	0,95	0,946	1
Gm16049	0,001758	-0,02598	0,01	0,031	1
Dgcr8	0,001759	-0,07051	0,184	0,247	1
Mmp11	0,001762	-0,03846	0,046	0,085	1
Kmt2e	0,001769	0,10302	0,908	0,889	1
Ppip5k2	0,00177	-0,08112	0,344	0,418	1
Apoa1	0,001773	0,028625	0,02	0,002	1
Grina	0,001784	0,105757	0,748	0,702	1
Car13	0,001787	-0,04239	0,049	0,088	1
Spg20	0,001791	-0,06304	0,967	0,979	1
Plekhh2	0,001793	-0,10463	0,244	0,311	1
Iqsec1	0,001801	0,132546	0,89	0,852	1
Apc	0,001808	0,096944	0,952	0,946	1
Gm27200	0,001809	-0,05272	0,101	0,153	1
Tank	0,001819	-0,06304	0,249	0,322	1
Pcp4	0,001824	-0,02561	0,01	0,031	1
Ctss	0,001847	0,032065	0,06	0,026	1
Kit	0,001866	0,047955	0,072	0,034	1
Fam20b	0,001879	-0,05471	0,168	0,233	1
Card19	0,001901	0,100257	0,723	0,688	1
Skil	0,001902	0,105135	0,357	0,283	1
S100a1	0,001909	0,072929	0,941	0,919	1
Etaa1os	0,00191	-0,02986	0,033	0,067	1
Adss	0,001923	-0,07948	0,264	0,335	1
Gm31793	0,001927	-0,02018	0,004	0,02	1
Resp18	0,001935	-0,017	0,004	0,02	1
Srsf3	0,001943	0,086204	0,944	0,933	1
Grik2	0,001998	-0,09916	0,321	0,4	1
Jak2	0,001998	-0,09891	0,43	0,504	1
Cant1	0,002001	-0,07662	0,345	0,42	1
Polr1d	0,00201	-0,07711	0,65	0,725	1
Gm5113	0,002013	-0,05888	0,12	0,174	1
Thumpd2	0,002041	-0,03289	0,044	0,081	1
Apoe	0,002047	-0,09201	0,962	0,93	1
Nt5c	0,002055	-0,0692	0,316	0,392	1
Hist1h2ap	0,002058	0,020033	0,023	0,003	1
Tdrd6	0,002065	-0,06213	0,087	0,135	1

Cdh10	0,002073	-0,07983	0,286	0,363	1
Fam133b	0,002077	-0,08659	0,63	0,688	1
Celsr2	0,002078	-0,07672	0,308	0,384	1
Fbxw4	0,002081	-0,05224	0,14	0,198	1
Golim4	0,002082	-0,09217	0,311	0,38	1
Hist1h4h	0,002086	-0,08162	0,2	0,265	1
Crip2	0,002114	0,079179	0,371	0,289	1
Dennd1c	0,00212	0,046331	0,031	0,008	1
Akr1b3	0,002121	-0,06803	0,607	0,701	1
Tmem170	0,002149	-0,05822	0,185	0,25	1
Mepce	0,002166	0,105391	0,376	0,315	1
B230209E1	0,002171	-0,04134	0,075	0,12	1
2810006K2	0,002176	-0,05589	0,127	0,182	1
Rgs2	0,002178	0,054004	0,081	0,042	1
Ppm1k	0,002185	-0,06038	0,278	0,353	1
Dnajc3	0,002197	-0,05143	0,816	0,867	1
Zfand6	0,002205	-0,09331	0,657	0,711	1
Prkci	0,002241	-0,05581	0,153	0,213	1
P2ry12	0,002253	-0,04473	0,051	0,089	1
Gm28172	0,002275	-0,04206	0,041	0,076	1
Ubl3	0,002277	-0,06905	0,955	0,976	1
Loxl3	0,002299	-0,02079	0,005	0,021	1
Lrp1	0,002321	-0,01761	0,005	0,021	1
Pip4k2a	0,002342	-0,07835	0,989	0,995	1
Dmxl1	0,002354	-0,07673	0,575	0,652	1
Kcnab1	0,002368	0,083603	0,165	0,111	1
Cyp2j9	0,002383	-0,05712	0,096	0,145	1
Gm20404	0,002397	0,03126	0,054	0,023	1
Gm6133	0,002412	-0,01417	0,002	0,015	1
Rnpep	0,002419	-0,06416	0,338	0,42	1
Pik3c2b	0,002434	-0,05871	0,768	0,836	1
Psmb10	0,002445	-0,19674	0,152	0,207	1
Kbtbd11	0,002462	-0,04217	0,073	0,117	1
Prrg1	0,002474	-0,1073	0,556	0,613	1
Aven	0,002481	-0,09767	0,223	0,288	1
Inip	0,002502	-0,0603	0,168	0,228	1
Ptk2	0,002534	-0,07768	0,756	0,815	1
Ltv1	0,002537	-0,06556	0,261	0,332	1
Arhgap24	0,002551	-0,03207	0,021	0,047	1
Mmp15	0,002566	-0,06459	0,135	0,19	1
Ppp6r2	0,002569	-0,08661	0,37	0,439	1
Gna11	0,002585	-0,07343	0,781	0,824	1
Nkiras2	0,002653	-0,05661	0,171	0,231	1
Slc25a10	0,002654	-0,07463	0,239	0,306	1
Tprn	0,002671	0,092435	0,884	0,859	1
Ptn	0,002688	0,098108	0,965	0,945	1
Zcchc8	0,002757	-0,06027	0,248	0,319	1
Pmm1	0,002775	-0,07094	0,64	0,72	1
Rexo1	0,002783	-0,06114	0,355	0,433	1
Rogdi	0,002786	-0,04926	0,182	0,246	1
Nipa1	0,002822	-0,06703	0,917	0,943	1
Tpi1	0,002831	0,118138	0,611	0,548	1

Tmem50b	0,002844	-0,07081	0,434	0,519	1
Phf13	0,002846	0,058221	0,146	0,096	1
Dusp11	0,002851	-0,07188	0,74	0,816	1
Csdc2	0,002855	-0,05954	0,107	0,156	1
Uap1l1	0,002864	0,116516	0,345	0,289	1
Usf3	0,002869	-0,05823	0,21	0,273	1
Cd46	0,002873	-0,0448	0,043	0,078	1
Got1	0,00288	-0,07641	0,395	0,468	1
Olfr671	0,002901	-0,05506	0,108	0,159	1
Cp	0,002902	-0,01884	0,007	0,024	1
Fam120b	0,002904	-0,07855	0,349	0,42	1
Sema4c	0,002904	0,085606	0,298	0,234	1
Grcc10	0,002905	0,089821	0,505	0,446	1
Smim10l1	0,002906	-0,07796	0,65	0,719	1
Bcl11b	0,00292	-0,01725	0,007	0,024	1
Fdps	0,002924	0,138912	0,835	0,813	1
Tex52	0,002938	-0,06493	0,25	0,317	1
Tsc22d1	0,002945	0,10064	0,306	0,241	1
A930024E(	0,002946	-0,02568	0,018	0,042	1
Trim7	0,002946	-0,06766	0,142	0,197	1
Rbck1	0,002954	-0,06943	0,478	0,553	1
Vmp1	0,002955	-0,07526	0,979	0,984	1
Thsd4	0,002958	-0,05463	0,086	0,132	1
Rnft1	0,002961	0,053784	0,098	0,057	1
Itgb3	0,003011	-0,03258	0,063	0,104	1
Dmtf1	0,003025	-0,07495	0,4	0,472	1
Ephb1	0,00303	-0,08949	0,84	0,88	1
Chp1	0,00305	-0,07925	0,394	0,46	1
Numbl	0,003066	-0,04259	0,108	0,159	1
Rbm3	0,003071	0,117923	0,569	0,522	1
Jph1	0,003095	-0,0665	0,545	0,608	1
Dnaja2	0,003171	-0,07545	0,739	0,785	1
C2cd3	0,003174	-0,06036	0,181	0,241	1
Msn	0,003184	0,114507	0,61	0,556	1
Ruvbl1	0,003185	-0,05108	0,359	0,442	1
Vps50	0,003202	-0,06642	0,396	0,468	1
Pxdn	0,003211	-0,05952	0,128	0,18	1
Apbb1ip	0,003222	-0,04963	0,168	0,229	1
Acadl	0,003222	-0,0872	0,547	0,61	1
Hnmt	0,003223	-0,02679	0,014	0,036	1
1110032A(	0,003225	-0,07008	0,395	0,481	1
Olfml1	0,00325	0,098314	0,598	0,528	1
Aldh6a1	0,003274	-0,07907	0,677	0,72	1
Ctr9	0,003277	-0,05273	0,639	0,728	1
Enpp2	0,003284	-0,05338	0,999	1	1
Pex12	0,003334	0,040403	0,113	0,068	1
Slc4a4	0,003342	-0,02005	0,014	0,036	1
Rab5a	0,003346	-0,06893	0,842	0,865	1
BC030499	0,003352	-0,02247	0,011	0,031	1
Myh7b	0,00336	0,090005	0,242	0,18	1
Insr	0,003366	-0,05529	0,357	0,437	1
Srebf1	0,00339	-0,08106	0,562	0,639	1

Rab5b	0,003393	-0,07382	0,658	0,711	1
Tmem129	0,003399	-0,05319	0,226	0,293	1
Fam19a5	0,003401	0,092401	0,516	0,455	1
Pla2g16	0,003401	-0,05499	0,998	1	1
Magi1	0,003418	-0,0712	0,711	0,761	1
Cadm2	0,003451	-0,09542	0,59	0,634	1
Dip2b	0,003453	-0,05605	0,965	0,974	1
Trim56	0,003457	-0,06138	0,084	0,13	1
Ppm1l	0,003469	-0,07798	0,335	0,407	1
Rnf213	0,00347	-0,0661	0,049	0,085	1
Barx2	0,003481	-0,06142	0,059	0,098	1
Enpp5	0,003489	0,064297	0,921	0,917	1
Suv39h2	0,003504	0,032438	0,04	0,015	1
Gng8	0,003516	0,138768	0,45	0,398	1
Cdc42bpa	0,003523	-0,06605	0,958	0,969	1
Tpd52l1	0,003536	-0,01034	0,001	0,011	1
Tmigd1	0,003536	-0,01034	0,001	0,011	1
Nell2	0,003536	-0,01034	0,001	0,011	1
Rab44	0,003536	-0,01034	0,001	0,011	1
Enah	0,003542	-0,08321	0,27	0,335	1
Gprc5b	0,003549	-0,06945	0,977	0,989	1
Syt9	0,003552	-0,05802	0,107	0,156	1
Bst2	0,003602	-0,18706	0,043	0,076	1
Stard3nl	0,003603	-0,07967	0,703	0,754	1
Sass6	0,003611	-0,0753	0,345	0,411	1
Rps28	0,003614	0,076948	0,988	0,985	1
Phc1	0,003629	-0,07544	0,496	0,563	1
Ubxn2a	0,003648	-0,07976	0,342	0,41	1
Slc2a1	0,003655	-0,0747	0,653	0,722	1
Ctso	0,003691	-0,07298	0,349	0,418	1
Tbcel	0,0037	-0,08662	0,387	0,447	1
Srp72	0,003714	-0,07844	0,548	0,6	1
Slc25a3	0,003726	0,077396	0,931	0,896	1
Parp11	0,003764	-0,03843	0,063	0,102	1
Fah	0,003771	-0,10753	0,6	0,649	1
Rnls	0,003777	-0,03769	0,063	0,102	1
Tmem63a	0,003783	-0,09869	0,968	0,964	1
Cdc42ep4	0,003797	-0,0464	0,081	0,125	1
Skiv2l	0,003798	-0,05783	0,35	0,421	1
Tanc2	0,003798	-0,07052	0,368	0,444	1
Cirbp	0,003807	0,114403	0,733	0,727	1
Nhlrc3	0,00382	-0,05754	0,161	0,218	1
9330198N	0,003851	0,086227	0,281	0,223	1
Paf1	0,003854	-0,07121	0,39	0,463	1
Dnaja4	0,003862	-0,06003	0,141	0,195	1
Specc1	0,003864	-0,08618	0,829	0,854	1
Pcdh7	0,003878	-0,05451	0,05	0,086	1
Kcnu1	0,003879	-0,03624	0,059	0,098	1
Chpt1	0,003888	-0,09079	0,778	0,816	1
Dido1	0,003908	-0,07304	0,333	0,402	1
Slitrk5	0,003937	-0,04706	0,039	0,072	1
Arrb2	0,003949	-0,04707	0,123	0,174	1

Gamt	0,003954	0,098351	0,859	0,813	1
Ifnk	0,003961	-0,0081	0	0,008	1
Btbd17	0,003961	-0,0081	0	0,008	1
Dgkb	0,003961	-0,00971	0	0,008	1
Daam1	0,003979	0,097037	0,915	0,899	1
Heg1	0,003991	-0,07305	0,324	0,395	1
Natd1	0,004004	-0,05731	0,186	0,246	1
Smurf2	0,00401	-0,06921	0,317	0,385	1
Tgfbi	0,004055	0,124669	0,267	0,208	1
Sybu	0,004062	-0,08474	0,306	0,369	1
Gm4876	0,004066	-0,03149	0,021	0,046	1
Tbl1x	0,004074	-0,09192	0,444	0,511	1
Fbrsl1	0,004076	-0,07359	0,245	0,307	1
Cdkn1c	0,00408	0,026006	0,443	0,354	1
9030025P2	0,004084	0,067845	0,197	0,143	1
Ocrl	0,004104	-0,05908	0,164	0,22	1
Cd68	0,004104	0,027919	0,047	0,02	1
Tgtp2	0,004116	-0,03862	0,004	0,018	1
Pxmp4	0,004121	0,061746	0,345	0,283	1
Gm33594	0,004129	-0,07568	0,29	0,354	1
Ckmt1	0,004146	-0,01541	0,004	0,018	1
Basp1	0,004157	-0,07646	0,056	0,093	1
Yod1	0,004182	-0,05755	0,273	0,343	1
Nudt16l1	0,004188	-0,07679	0,44	0,511	1
Lzic	0,004189	-0,05528	0,183	0,242	1
Begain	0,004206	-0,07172	0,088	0,132	1
Anxa5	0,004208	0,012561	0,745	0,667	1
Psmd10	0,004236	-0,05712	0,254	0,322	1
Sertad2	0,004283	0,115123	0,498	0,449	1
Ttc33	0,004309	-0,06627	0,414	0,481	1
Aen	0,004316	-0,04577	0,163	0,221	1
Kmt5a	0,004323	-0,05651	0,842	0,891	1
Coro1c	0,004324	0,125891	0,703	0,673	1
Klf6	0,004353	0,173433	0,612	0,572	1
Rfx3	0,004392	-0,0579	0,157	0,211	1
Mospd2	0,004405	-0,07344	0,377	0,437	1
Igf1r	0,004409	-0,11003	0,569	0,618	1
Nufip1	0,004442	0,056881	0,179	0,127	1
Zdhhc20	0,004454	-0,07417	0,967	0,979	1
Ctnna2	0,004479	-0,11529	0,449	0,514	1
Degs1	0,004505	0,067314	0,984	0,995	1
Gm5141	0,00451	-0,04473	0,052	0,088	1
Azi2	0,004511	-0,08396	0,374	0,442	1
Fam198b	0,004521	-0,03757	0,038	0,07	1
Abhd12	0,004552	-0,07739	0,843	0,893	1
Coq8a	0,004555	-0,0498	0,17	0,228	1
Prickle3	0,004557	0,057968	0,083	0,047	1
Rftn2	0,004613	-0,07238	0,369	0,437	1
Chadl	0,004618	0,059892	0,135	0,089	1
Wipi2	0,004627	-0,07653	0,582	0,647	1
Gba	0,004635	-0,06513	0,342	0,411	1
Gab2	0,004637	-0,05773	0,14	0,193	1

Lig3	0,004657	-0,08049	0,497	0,554	1
Mrps34	0,004665	-0,05867	0,398	0,472	1
Erbb3	0,004668	-0,0884	0,627	0,681	1
Slc33a1	0,004681	-0,05938	0,344	0,415	1
Dcdc2a	0,004691	-0,05499	0,214	0,278	1
Atp2c1	0,004713	-0,06888	0,903	0,912	1
Tial1	0,004713	-0,08229	0,637	0,689	1
Brwd3	0,004715	-0,06942	0,2	0,259	1
5031439G(	0,004752	0,074864	0,979	0,987	1
Mamdc4	0,004767	-0,02079	0,005	0,02	1
Vta1	0,004769	-0,07722	0,658	0,719	1
Fam102b	0,004788	0,053596	0,109	0,067	1
Alg11	0,004851	-0,06808	0,248	0,309	1
Hsd17b12	0,004851	-0,05111	0,821	0,875	1
Ppp3ca	0,00486	-0,06045	0,914	0,946	1
Lzts2	0,004869	0,082785	0,958	0,93	1
Gpr107	0,004894	-0,06073	0,395	0,475	1
Vps13c	0,004897	-0,06709	0,289	0,354	1
Cops5	0,004906	-0,06752	0,721	0,784	1
C3	0,004938	0,01558	0,013	0	1
Mag	0,004962	-0,04711	0,998	1	1
Apod	0,004967	-0,23242	0,998	1	1
Arhgap23	0,004977	0,09067	0,976	0,992	1
Agbl5	0,00498	-0,02353	0,018	0,041	1
Pabpc1	0,005001	0,102766	0,867	0,833	1
1700063D(	0,005034	-0,11961	0,222	0,28	1
Ptma	0,005059	0,059659	0,999	1	1
A330008L1	0,005063	-0,03341	0,017	0,039	1
Slc35e2	0,005067	-0,05814	0,219	0,28	1
Ap1m1	0,005075	-0,04572	0,273	0,34	1
Hunk	0,0051	-0,03347	0,047	0,081	1
Endod1	0,005113	0,041547	0,986	0,985	1
Pcgf6	0,005114	-0,05297	0,118	0,166	1
Srcap	0,005134	0,085555	0,321	0,26	1
Nprl3	0,005153	-0,0412	0,114	0,163	1
Tprgl	0,005178	-0,05902	0,887	0,915	1
Kat8	0,005205	-0,05731	0,19	0,249	1
Cpsf2	0,005238	-0,05373	0,222	0,288	1
Sgpp1	0,00526	-0,07558	0,346	0,416	1
Myof	0,005277	0,071468	0,152	0,104	1
Rrbp1	0,005277	-0,06542	0,953	0,969	1
Kifc3	0,005278	0,079559	0,324	0,263	1
Ercc8	0,005285	-0,0519	0,153	0,207	1
Plekhb1	0,005324	0,099514	0,998	0,998	1
Tmem116	0,005329	-0,01664	0,006	0,021	1
Gm48045	0,005329	-0,01664	0,006	0,021	1
Dusp1	0,005361	0,268034	0,335	0,28	1
Gm20652	0,005377	0,020033	0,02	0,003	1
Suclg2	0,005392	-0,02986	0,036	0,067	1
Hmgcr	0,005395	0,190298	0,538	0,493	1
Mycbp2	0,005396	-0,1041	0,895	0,889	1
Rhbdl2	0,005408	-0,03513	0,067	0,106	1

Pura	0,005428	0,071702	0,969	0,961	1
Adam23	0,005435	0,07772	0,386	0,322	1
Mrpl37	0,005442	-0,04199	0,16	0,218	1
Heatr3	0,005443	-0,07367	0,526	0,576	1
Ergic1	0,005475	-0,04108	0,132	0,184	1
Rnaset2a	0,005483	0,079264	0,286	0,231	1
Pou3f3	0,005495	-0,06936	0,834	0,881	1
2310022B(	0,005501	-0,10093	0,789	0,803	1
Pbx4	0,005566	-0,01096	0,002	0,013	1
Ppp4r2	0,005588	0,082039	0,665	0,624	1
Kif21b	0,00563	-0,07446	0,2	0,259	1
D5Ertd615	0,005669	-0,01884	0,007	0,023	1
Runx1	0,005696	0,098374	0,093	0,055	1
B4galt5	0,005699	0,019669	0,023	0,005	1
Mllt3	0,0057	-0,07143	0,188	0,242	1
Cyld	0,005706	-0,07313	0,558	0,603	1
Tmeff2	0,005712	-0,05404	0,969	0,977	1
Agfg2	0,005725	0,061885	0,21	0,156	1
Sertad1	0,005746	0,11612	0,206	0,156	1
2900011O(	0,00575	-0,02494	0,024	0,049	1
Renbp	0,00576	0,03546	0,071	0,037	1
Bcar1	0,005824	0,114029	0,681	0,639	1
Adora1	0,005828	-0,06778	0,633	0,678	1
Lama2	0,005833	-0,0297	0,012	0,031	1
Itpr1	0,005876	-0,05792	0,06	0,096	1
Gm6712	0,005902	-0,0209	0,013	0,033	1
Pdzrn4	0,005904	-0,0186	0,014	0,034	1
Enoph1	0,005919	-0,08093	0,67	0,732	1
Bod1l	0,005965	-0,06128	0,764	0,839	1
Cipc	0,005979	-0,06573	0,833	0,873	1
Lhfpl3	0,005991	-0,05821	0,052	0,086	1
Frmd8	0,006005	-0,07616	0,935	0,953	1
Olig1	0,006009	-0,07053	0,976	0,984	1
Ppp1r15a	0,006065	0,121829	0,309	0,25	1
Bzw2	0,006066	-0,06257	0,147	0,198	1
Gm29650	0,006066	-0,02127	0,011	0,029	1
Plat	0,006089	0,129696	0,369	0,312	1
1500011B(	0,006096	-0,05882	0,386	0,457	1
Luc7l2	0,006111	-0,0773	0,958	0,967	1
Prpf8	0,006122	0,102331	0,577	0,535	1
Abi2	0,00616	-0,06497	0,594	0,655	1
Ebf1	0,006199	-0,01811	0,009	0,026	1
Apoc1	0,006243	-0,01617	0,011	0,029	1
Gdf11	0,006251	0,024545	0,038	0,015	1
Serinc5	0,006291	-0,09817	0,761	0,802	1
Pcdhgb2	0,006357	-0,02207	0,023	0,047	1
Ago2	0,006363	-0,0729	0,267	0,328	1
Trim24	0,006444	-0,05912	0,358	0,424	1
Ccdc28a	0,006468	-0,04976	0,081	0,122	1
Mthfsl	0,006522	-0,0628	0,448	0,517	1
Pafah1b2	0,00656	-0,07377	0,868	0,902	1
Ccdc63	0,006611	0,014921	0,016	0,002	1

Spsb1	0,006669	0,082454	0,583	0,511	1
Smim13	0,006678	-0,07853	0,473	0,538	1
Ago3	0,006681	-0,08295	0,659	0,699	1
Ube4a	0,006695	-0,06164	0,439	0,511	1
Pacsin1	0,006717	-0,12313	0,399	0,452	1
Txn1	0,006728	0,040522	0,671	0,61	1
Zfp365	0,006744	-0,07396	0,603	0,663	1
Pank3	0,006777	0,117354	0,549	0,507	1
Zfp668	0,00679	-0,0385	0,084	0,127	1
Bmp4	0,006795	0,04851	0,04	0,016	1
Nedd9	0,006812	-0,04097	0,09	0,133	1
Nsun7	0,00683	-0,02207	0,054	0,089	1
Nptn	0,006831	0,083919	0,714	0,667	1
9530059O:	0,006873	-0,04458	0,182	0,239	1
Fig4	0,00692	-0,04341	0,148	0,202	1
Mpz	0,006936	0,150921	0,012	0	1
Abcb10	0,006946	-0,08444	0,421	0,488	1
Agpat4	0,006952	-0,04964	0,992	0,992	1
Ktn1	0,007009	-0,0493	0,992	0,998	1
Ccp110	0,007041	-0,10796	0,85	0,859	1
Zfp91	0,007056	-0,05781	0,857	0,88	1
Snx1	0,007077	-0,06714	0,846	0,865	1
Map4k4	0,007085	0,070905	0,988	0,985	1
Slc35a5	0,007115	-0,06211	0,672	0,73	1
Zfp944	0,007143	-0,0434	0,116	0,163	1
Arhgap25	0,007145	0,020554	0,027	0,008	1
Efr3b	0,007152	-0,05849	0,268	0,333	1
Fbxl5	0,007163	-0,07266	0,795	0,844	1
Eef1d	0,007167	0,064915	0,945	0,927	1
Stxbp6	0,007186	-0,0896	0,64	0,691	1
Galnt12	0,007195	-0,03855	0,076	0,115	1
Med21	0,00721	-0,08486	0,67	0,724	1
BC005561	0,007231	-0,08837	0,576	0,624	1
Gigyf1	0,007234	0,099337	0,577	0,528	1
Gm31105	0,007239	-0,05417	0,049	0,081	1
Evi5l	0,00724	-0,06503	0,435	0,499	1
Specc1l	0,007244	-0,0675	0,304	0,367	1
Gpr37	0,007329	-0,05454	0,999	0,995	1
Sept5	0,007335	-0,01319	0,003	0,015	1
A230057D(	0,007365	-0,05273	0,147	0,198	1
Pin4	0,007386	-0,06879	0,655	0,714	1
Ano8	0,007454	-0,05133	0,168	0,221	1
Psma4	0,007479	-0,06703	0,765	0,821	1
Prkacb	0,007538	-0,05564	0,985	0,982	1
Mynn	0,007554	-0,05624	0,282	0,343	1
Actg1	0,007557	0,070014	0,997	0,995	1
Ahsa1	0,007564	-0,06223	0,772	0,837	1
Gabpa	0,007586	-0,04374	0,235	0,299	1
Slc13a3	0,00759	-0,07913	0,125	0,169	1
Kat7	0,007606	-0,05361	0,376	0,441	1
Nat8f4	0,007631	-0,02325	0,026	0,052	1
Arntl2	0,007674	0,017454	0,022	0,005	1

Pla2g3	0,007688	-0,03091	0,019	0,041	1
Nsun3	0,00772	-0,03345	0,041	0,072	1
Tmem144	0,007742	-0,09906	0,617	0,672	1
Gm48682	0,007839	-0,0256	0,031	0,059	1
Pnpla8	0,007878	-0,05688	0,535	0,621	1
Mboat2	0,007886	0,083443	0,535	0,47	1
Cept1	0,007899	-0,06881	0,564	0,613	1
Pcdh1	0,007937	-0,05786	0,122	0,167	1
Flna	0,007987	0,047471	0,128	0,085	1
Riok2	0,007994	-0,04242	0,198	0,259	1
Mif	0,008	-0,07891	0,883	0,912	1
Ank	0,008071	-0,059	0,953	0,964	1
Pop5	0,00811	-0,06846	0,53	0,587	1
Pllp	0,008134	0,066778	0,999	0,998	1
Fam189a2	0,008158	-0,0604	0,192	0,247	1
Zfp788	0,008172	-0,06225	0,209	0,265	1
Gm43597	0,008179	-0,03949	0,066	0,102	1
Cep68	0,008179	-0,04611	0,159	0,211	1
Scaf11	0,008206	-0,06291	0,893	0,889	1
Mrpl21	0,008235	-0,06392	0,366	0,431	1
Rgl1	0,008241	0,049931	0,113	0,073	1
Rassf2	0,008255	-0,04121	0,899	0,935	1
Tmem263	0,008269	-0,06312	0,259	0,319	1
Prox1	0,00829	-0,07175	0,903	0,932	1
Zfp444	0,008301	-0,03731	0,143	0,195	1
2900060B1	0,008349	0,034042	0,058	0,029	1
Acsf2	0,008372	-0,03999	0,076	0,114	1
Adam22	0,0084	-0,07015	0,46	0,519	1
Rpia	0,008408	0,069707	0,292	0,236	1
Rnf125	0,008448	-0,04107	0,049	0,081	1
2310005A0	0,008542	-0,00873	0,001	0,01	1
Gm44672	0,008542	-0,00873	0,001	0,01	1
Astn2	0,008542	0,047114	0,077	0,044	1
Alad	0,008583	-0,05229	0,213	0,27	1
Ppp2r2b	0,00859	-0,02664	0,017	0,037	1
Erlec1	0,008611	-0,0546	0,399	0,468	1
Cacna1a	0,008623	-0,04801	0,074	0,111	1
Gm16168	0,008639	-0,0517	0,159	0,21	1
Trove2	0,008643	-0,05872	0,625	0,685	1
Afap1l2	0,00866	0,034042	0,034	0,013	1
Mmd2	0,008681	-0,06515	0,282	0,345	1
Bfsp2	0,008685	-0,11175	0,548	0,61	1
Cnp	0,00869	0,039132	1	1	1
Pqlc3	0,008758	0,029227	0,123	0,08	1
Gbf1	0,008826	-0,05371	0,371	0,442	1
Cdip1	0,008855	-0,07119	0,831	0,878	1
Eml5	0,008859	-0,07667	0,187	0,237	1
Prkar1b	0,008877	-0,02393	0,03	0,057	1
Clvs1	0,008886	-0,00928	0,004	0,016	1
Abca2	0,008888	0,0766	0,998	0,998	1
Appl1	0,008898	0,092959	0,685	0,647	1
Iqgap1	0,008938	0,074544	0,331	0,272	1

Smap1	0,008949	-0,05427	0,605	0,683	1
Atp6v1e1	0,008967	-0,05683	0,92	0,938	1
Dnajc16	0,009007	-0,05229	0,199	0,255	1
Wdr41	0,009024	-0,05464	0,268	0,328	1
Fscn1	0,009024	-0,06774	0,934	0,958	1
Clcn3	0,009077	-0,05493	0,961	0,982	1
Rbpjl	0,00912	0,054012	0,104	0,067	1
Zswim1	0,009153	-0,03192	0,087	0,128	1
Gpr153	0,009179	0,020695	0,015	0,002	1
Serpine1	0,009179	0,026437	0,015	0,002	1
Mdc1	0,0092	-0,03628	0,245	0,312	1
Gm45716	0,009212	0,018774	0,015	0,002	1
Rab3gap2	0,009256	-0,07254	0,422	0,483	1
Fbxo2	0,00927	-0,10601	0,451	0,506	1
Stambp	0,009291	-0,0917	0,544	0,597	1
Rps5	0,009306	0,064929	0,987	0,984	1
Scyl3	0,009313	-0,04258	0,153	0,203	1
Zw10	0,009315	-0,04344	0,167	0,22	1
Bcl3	0,009321	0,054229	0,09	0,055	1
Cdh6	0,009321	-0,05696	0,16	0,213	1
Pcdhga8	0,009325	-0,02429	0,029	0,055	1
Oxct1	0,009345	-0,08345	0,639	0,693	1
Aga	0,009357	-0,07417	0,304	0,363	1
Gm45620	0,009417	-0,01824	0,016	0,036	1
Stk3	0,009467	-0,05872	0,191	0,246	1
Fam131b	0,009493	-0,0778	0,597	0,634	1
Psap	0,009515	0,062573	0,999	1	1
Ctsd	0,009515	0,063975	0,985	0,982	1
Fbxo8	0,009541	-0,04994	0,396	0,47	1
Rnf122	0,009548	0,174117	0,447	0,405	1
Abhd5	0,009653	-0,062	0,625	0,667	1
Emc2	0,009661	-0,0542	0,522	0,597	1
Flnb	0,00968	0,05929	0,524	0,457	1
Tmem106b	0,009681	-0,05349	0,773	0,836	1
Lmbrd1	0,009749	-0,05758	0,974	0,967	1
Nes	0,009762	0,020399	0,011	0	1
Mrpl50	0,009804	-0,05649	0,29	0,353	1
S100b	0,009806	0,036725	0,852	0,795	1
Snrpn	0,009821	-0,045	0,767	0,821	1
mt-Atp8	0,009913	-0,05532	0,959	0,971	1
Ywhaz	0,009918	0,079217	0,974	0,969	1
Rab10os	0,009943	-0,06355	0,37	0,436	1
2610008E1	0,009951	-0,04225	0,287	0,356	1
Chml	0,00999	0,041349	0,09	0,055	1
Ankrd45	0,009991	-0,00648	0	0,007	1
S1pr1	0,009991	-0,00648	0	0,007	1
Rims3	0,009991	-0,00648	0	0,007	1
Arl9	0,009991	-0,00648	0	0,007	1
Actl6b	0,009991	-0,00648	0	0,007	1
Ptpro	0,009991	-0,00648	0	0,007	1
Ubap1l	0,009991	-0,00648	0	0,007	1
Ccdc36	0,009991	-0,00648	0	0,007	1

Hrh2	0,009991	-0,00648	0	0,007	1
3300002A1	0,009991	-0,00648	0	0,007	1
Pde1b	0,009991	-0,00648	0	0,007	1
Gm10532	0,009991	-0,00648	0	0,007	1
D930019O	0,009991	-0,0081	0	0,007	1
1810012K1	0,009991	-0,0081	0	0,007	1
Batf2	0,009991	-0,00971	0	0,007	1
2010300C(	0,009991	-0,01132	0	0,007	1
Ggct	0,009999	-0,08004	0,427	0,489	1
Acot6	0,010008	-0,04951	0,119	0,163	1
Nat10	0,010031	-0,04329	0,125	0,171	1
Vac14	0,01006	-0,05614	0,201	0,257	1
Gm20449	0,010073	-0,01088	0,005	0,018	1
Ksr1	0,010075	-0,0638	0,233	0,288	1
Edem1	0,010084	-0,05968	0,289	0,351	1
Dct	0,010104	-0,10369	0,033	0,06	1
Mettl7a1	0,010117	-0,07818	0,67	0,717	1
Slco3a1	0,010118	-0,08026	0,964	0,945	1
Cdan1	0,010124	-0,06251	0,219	0,275	1
Lrig1	0,010146	-0,04212	0,08	0,119	1
Atg9a	0,010271	-0,06022	0,352	0,415	1
Iigp1	0,010327	-0,11663	0,006	0,02	1
Amd2	0,01034	0,100085	0,825	0,777	1
Dusp23	0,010386	-0,05127	0,181	0,234	1
Tmem220	0,010406	0,036587	0,09	0,055	1
Plgrkt	0,01042	-0,06859	0,289	0,348	1
Zfhx4	0,010457	-0,06383	0,383	0,452	1
Phyhipl	0,010459	0,082798	0,968	0,956	1
Stk19	0,010467	-0,05816	0,347	0,405	1
Arhgap32	0,010489	-0,07427	0,366	0,421	1
Rtp4	0,010513	-0,05913	0,048	0,078	1
Rcbtb1	0,010524	-0,0738	0,918	0,925	1
Zfp938	0,010539	-0,05775	0,167	0,216	1
Ehd3	0,010543	-0,05598	0,263	0,32	1
Ly6h	0,01056	-0,01505	0,006	0,02	1
Mif4gd	0,01059	0,076845	0,614	0,574	1
Sall4	0,010615	-0,01566	0,006	0,02	1
Reep4	0,010619	-0,03744	0,088	0,128	1
Ell2	0,010713	-0,05264	0,43	0,494	1
Ano4	0,010717	-0,07738	0,63	0,681	1
N4bp2l1	0,01073	-0,04268	0,127	0,174	1
Fgfr1op	0,010735	-0,05212	0,13	0,176	1
Riox2	0,010754	-0,04093	0,104	0,146	1
Cops2	0,010772	-0,07764	0,588	0,633	1
Nr1d2	0,010775	-0,04793	0,244	0,304	1
Ncor2	0,010775	0,088264	0,474	0,428	1
Ap3b2	0,01081	-0,05631	0,327	0,389	1
Abtb1	0,010813	-0,05274	0,202	0,255	1
Limd2	0,010836	-0,0115	0,006	0,02	1
Acsl1	0,01087	-0,0787	0,706	0,75	1
Dact3	0,010884	0,078432	0,773	0,741	1
Csnk2b	0,010944	-0,06008	0,762	0,782	1

Hip1r	0,010946	0,10815	0,523	0,481	1
Uqcrc1	0,010989	-0,04716	0,816	0,868	1
Gstp2	0,011004	0,073917	0,095	0,06	1
Ccdc187	0,011034	-0,05809	0,139	0,185	1
6530409C:	0,011065	-0,03422	0,066	0,101	1
Phf3	0,011122	-0,03907	0,836	0,883	1
Sptan1	0,011125	0,077971	0,496	0,426	1
1700020D(	0,011125	-0,0224	0,032	0,059	1
Kif17	0,01113	-0,01469	0,007	0,021	1
Dynll2	0,011132	0,113761	0,812	0,79	1
Hecw2	0,011149	-0,08743	0,535	0,595	1
Ppp1r12b	0,011162	-0,06243	0,213	0,267	1
Chgb	0,011167	-0,01786	0,008	0,023	1
Ifit1	0,011178	-0,05405	0,011	0,028	1
Cacna2d1	0,011185	-0,02224	0,008	0,023	1
Galnt13	0,011193	-0,0131	0,007	0,021	1
Ttll9	0,01123	-0,0153	0,007	0,021	1
Tdrd9	0,011236	-0,01786	0,008	0,023	1
Rfx7	0,011239	-0,0667	0,275	0,333	1
Vps36	0,011246	-0,05278	0,328	0,392	1
Ngfr	0,011252	0,080122	0,156	0,114	1
Agmo	0,011284	-0,07653	0,341	0,403	1
Car3	0,011315	-0,02171	0,021	0,042	1
Atp6ap2	0,01132	0,076537	0,953	0,93	1
Sowaha	0,011323	-0,06378	0,282	0,343	1
Hdlbp	0,011342	-0,0521	0,662	0,725	1
Slc7a1	0,011344	0,03627	0,052	0,026	1
AC160336.	0,011348	0,083836	0,873	0,839	1
Ccng1	0,011354	-0,06463	0,322	0,379	1
Nexmif	0,011362	-0,01981	0,009	0,024	1
Bivm	0,011386	-0,04446	0,096	0,137	1
Qtrt2	0,011463	-0,03665	0,121	0,166	1
Rassf4	0,011478	-0,06386	0,219	0,275	1
Map6	0,011525	0,101181	0,528	0,48	1
Irs2	0,011574	-0,03847	0,788	0,844	1
Yipf4	0,011576	-0,05175	0,707	0,785	1
Mrps14	0,011614	-0,06368	0,41	0,472	1
Nde1	0,011622	-0,047	0,599	0,668	1
Loxl2	0,011631	-0,03987	0,03	0,055	1
Appbp2	0,011658	-0,05338	0,826	0,881	1
Aldh1l1	0,011684	-0,0274	0,036	0,063	1
Tia1	0,011735	-0,07433	0,646	0,702	1
Cct7	0,011735	-0,05634	0,912	0,924	1
Slc35e1	0,01176	-0,07006	0,214	0,263	1
Zeb2	0,011766	0,068723	0,999	1	1
Rtl8a	0,011773	0,080215	0,902	0,868	1
Ext1	0,011873	-0,03726	0,152	0,203	1
Wfdc18	0,011875	-0,10852	0,201	0,252	1
Pbx3	0,01191	-0,05776	0,363	0,428	1
Fbxo18	0,011936	-0,04	0,201	0,257	1
Lyar	0,011953	-0,0472	0,199	0,252	1
Kctd3	0,011974	-0,04835	0,809	0,847	1

Cars	0,011987	-0,04614	0,202	0,254	1
Crbn	0,012009	-0,05832	0,395	0,46	1
Apbb1	0,012011	-0,07972	0,947	0,964	1
Dopey1	0,012029	-0,0801	0,443	0,496	1
2410089E(	0,012078	-0,05801	0,337	0,405	1
Maob	0,012135	-0,05904	0,13	0,174	1
Cdh11	0,012138	-0,03806	0,651	0,732	1
Alcam	0,012139	-0,07638	0,673	0,715	1
Nsa2	0,01214	-0,05825	0,713	0,777	1
Lancl1	0,012175	-0,05444	0,508	0,567	1
Dnajc24	0,012176	-0,08753	0,602	0,629	1
Asb7	0,012226	-0,04085	0,241	0,301	1
2810403A(	0,012233	-0,05814	0,298	0,363	1
Slc25a16	0,012306	-0,04902	0,187	0,239	1
Abca6	0,012313	-0,04851	0,124	0,167	1
Tpp2	0,012343	-0,0558	0,311	0,372	1
Mtf1	0,01235	-0,062	0,512	0,576	1
Klhl7	0,012376	-0,04516	0,198	0,252	1
Csrnp2	0,012439	0,038309	0,075	0,044	1
Mrpl34	0,012463	-0,05957	0,535	0,6	1
Usp25	0,01248	-0,08117	0,572	0,602	1
Atl3	0,012495	-0,05935	0,377	0,436	1
Shisa5	0,012524	-0,04278	0,128	0,174	1
Lsm8	0,012557	-0,05711	0,447	0,512	1
Trim32	0,012575	-0,05117	0,302	0,364	1
Swsap1	0,012581	-0,02834	0,04	0,068	1
Tmem25	0,012604	0,028967	0,067	0,037	1
Hba-a1	0,012611	1,78626	0,04	0,018	1
Efhd1	0,012619	-0,06856	0,88	0,896	1
Cnpy2	0,012623	-0,06572	0,608	0,66	1
Smco3	0,01263	-0,07322	0,565	0,615	1
Esd	0,012636	-0,05337	0,626	0,694	1
Robo2	0,012639	-0,01417	0,002	0,011	1
Malat1	0,012645	0,03176	0,998	1	1
Zranb3	0,012681	-0,03643	0,052	0,083	1
Lpin3	0,012699	-0,01096	0,002	0,011	1
Efemp2	0,012699	-0,01096	0,002	0,011	1
Zfand3	0,012705	-0,04863	0,43	0,507	1
Aak1	0,012706	-0,05024	0,358	0,426	1
Casc4	0,012716	-0,08821	0,265	0,314	1
Aass	0,012729	-0,00936	0,002	0,011	1
Ebf3	0,012729	-0,00936	0,002	0,011	1
Ifitm2	0,012729	-0,00936	0,002	0,011	1
Bco2	0,012729	-0,00936	0,002	0,011	1
Pnmal1	0,012787	-0,05448	0,096	0,135	1
Mad2l2	0,012799	-0,0569	0,236	0,291	1
Selenop	0,012816	-0,10941	0,896	0,92	1
Gnb5	0,012822	-0,03947	0,144	0,192	1
Adgrg1	0,012834	0,076648	0,177	0,132	1
Gkap1	0,012899	-0,06663	0,82	0,854	1
Ptpra	0,012916	-0,05776	0,955	0,948	1
Gm21887	0,012933	0,029235	0,071	0,041	1

Sash1	0,012937	-0,08591	0,751	0,769	1
Ttc32	0,012949	-0,03662	0,133	0,18	1
Dpp9	0,012963	-0,05017	0,241	0,298	1
Oma1	0,013025	-0,04515	0,17	0,22	1
Siah2	0,013043	-0,07016	0,369	0,433	1
Litaf	0,013044	-0,06305	0,991	0,993	1
Ap5m1	0,013154	-0,06849	0,241	0,293	1
Ccdc28b	0,013197	-0,05743	0,328	0,389	1
Rsad2	0,013287	-0,03249	0,018	0,037	1
Slmap	0,013301	-0,05118	0,257	0,315	1
Prkcb	0,013347	0,072477	0,381	0,325	1
Tepsin	0,013352	-0,03495	0,075	0,111	1
Fam46a	0,013378	-0,11315	0,267	0,322	1
Uckl1os	0,013414	0,022742	0,03	0,011	1
Irf7	0,013422	-0,04558	0,024	0,046	1
Cfl2	0,013481	0,07103	0,986	0,982	1
Cep85l	0,013496	-0,05052	0,205	0,26	1
Fam13c	0,013525	0,12097	0,639	0,603	1
Etv5	0,013568	-0,01876	0,054	0,086	1
Gatad2b	0,013606	-0,03166	0,773	0,821	1
Med26	0,013741	0,03507	0,084	0,052	1
Mpeg1	0,013775	0,012677	0,01	0	1
Grm8	0,013775	0,010737	0,01	0	1
Osbpl3	0,013775	0,011707	0,01	0	1
Oprk1	0,013775	0,01558	0,01	0	1
Tox4	0,0138	-0,05133	0,464	0,53	1
Cdkl5	0,013802	-0,07847	0,504	0,548	1
BC004004	0,013827	-0,06051	0,511	0,58	1
Ube2n	0,013832	-0,05963	0,808	0,85	1
Dimt1	0,013861	-0,04496	0,2	0,254	1
Abcc1	0,013893	-0,04869	0,199	0,25	1
Ptcd3	0,013904	-0,06105	0,247	0,302	1
Chn2	0,013905	-0,06027	0,881	0,914	1
March5	0,01394	-0,0704	0,508	0,554	1
Pcnx4	0,013944	-0,05112	0,186	0,236	1
Sept9	0,013965	0,088109	0,296	0,244	1
Ahsa2	0,014038	-0,0645	0,436	0,491	1
Ncstn	0,01408	-0,05802	0,265	0,32	1
Pgk1	0,014116	-0,04799	0,792	0,846	1
Sh2b1	0,01412	-0,05011	0,258	0,315	1
Gm48899	0,014164	0,014263	0,017	0,003	1
Hivep2	0,014183	-0,04965	0,076	0,111	1
Dglucy	0,014188	0,016494	0,02	0,005	1
Btf3l4	0,01433	-0,04926	0,185	0,234	1
Gm12279	0,014337	0,013608	0,017	0,003	1
Dnmt1	0,014337	-0,06145	0,269	0,325	1
Nectin3	0,014388	-0,03278	0,138	0,185	1
Baalc	0,014422	-0,02137	0,017	0,036	1
Zfp976	0,014454	-0,03385	0,055	0,086	1
Mras	0,014594	-0,02271	0,127	0,172	1
Sept4	0,01461	-0,05797	0,998	0,997	1
Smarcd1	0,014633	-0,04185	0,854	0,886	1

Map2k2	0,014653	-0,0568	0,849	0,873	1
Scpep1	0,014659	0,056914	0,244	0,193	1
Ndufa10	0,014662	-0,05204	0,799	0,86	1
Fgf1	0,014717	-0,09144	0,647	0,665	1
Msantd3	0,01474	-0,03962	0,123	0,166	1
Sephs1	0,014755	-0,04876	0,173	0,221	1
Ccdc173	0,014763	-0,03619	0,08	0,117	1
Clic4	0,014789	0,063184	0,986	0,997	1
Usp46	0,014792	0,07401	0,289	0,239	1
Glce	0,014811	0,021781	0,051	0,026	1
Gm49085	0,014812	0,024062	0,027	0,01	1
Klhl24	0,014839	-0,0641	0,704	0,766	1
Col9a1	0,014854	-0,03644	0,046	0,075	1
Anln	0,014869	-0,08876	0,96	0,959	1
Uhmk1	0,014881	-0,06688	0,403	0,468	1
Polr1a	0,015055	-0,05209	0,217	0,27	1
Nrf1	0,015077	-0,03041	0,195	0,25	1
Gm36855	0,015083	-0,05506	0,119	0,161	1
Snrpd3	0,01522	-0,04553	0,739	0,776	1
Zfp7	0,015225	-0,02567	0,033	0,059	1
Rab32	0,015228	0,017698	0,027	0,01	1
Foxn2	0,015235	-0,03616	0,395	0,476	1
Tlk2	0,015258	-0,06512	0,375	0,437	1
Cenpb	0,015282	0,089395	0,528	0,48	1
Rpl10	0,015283	0,073674	0,981	0,984	1
Gpd1	0,015337	-0,07807	0,603	0,66	1
Dubr	0,015358	-0,06495	0,262	0,319	1
Gm44763	0,015393	-0,02294	0,043	0,072	1
Syne3	0,015401	-0,04755	0,101	0,14	1
Paox	0,01543	-0,04535	0,114	0,154	1
Atg14	0,015453	-0,04764	0,237	0,291	1
Crat	0,015459	-0,06849	0,264	0,314	1
Adamts6	0,015527	-0,02772	0,05	0,08	1
Aurka	0,015533	-0,01945	0,016	0,034	1
Tjp1	0,01557	-0,07759	0,688	0,74	1
Tra2a	0,015573	-0,06142	0,924	0,95	1
Fam169a	0,015627	-0,0233	0,015	0,033	1
Lrrc45	0,015665	-0,03495	0,185	0,237	1
Cbl	0,01567	-0,06416	0,594	0,659	1
Rbm34	0,015711	-0,05084	0,324	0,385	1
Slc25a44	0,015716	-0,04584	0,381	0,447	1
Cwf19l2	0,015736	-0,06576	0,478	0,533	1
Ptprz1	0,015748	-0,02486	0,015	0,033	1
Sirt1	0,015838	0,065793	0,332	0,276	1
Ly6g6f	0,015874	0,087636	0,41	0,363	1
Tead1	0,015887	0,075597	0,138	0,099	1
Elmo2	0,015893	-0,06049	0,204	0,252	1
Rbm46	0,015939	-0,01319	0,003	0,013	1
Stip1	0,016001	-0,06887	0,633	0,676	1
Zfp583	0,016018	0,033126	0,059	0,033	1
Tsc22d3	0,016026	-0,08989	0,486	0,532	1
Gm816	0,01603	-0,02582	0,032	0,057	1

Csrp2	0,016041	0,03126	0,048	0,024	1
Plppr3	0,016043	0,038014	0,074	0,044	1
Arvcf	0,016062	-0,03585	0,082	0,119	1
Arhgap44	0,016124	-0,00901	0,003	0,013	1
Tmem30a	0,016125	-0,05171	0,985	0,993	1
Hivep3	0,016139	0,098118	0,402	0,346	1
Rae1	0,016151	-0,05603	0,284	0,338	1
Slc9a9	0,016226	-0,0482	0,204	0,257	1
Gpbp1	0,016256	0,056791	0,628	0,577	1
Grm7	0,016326	-0,05771	0,103	0,141	1
Pdgfc	0,016343	-0,031	0,041	0,068	1
Babam2	0,016377	-0,05506	0,444	0,514	1
Acad12	0,016397	-0,03565	0,103	0,143	1
Ranbp6	0,016445	-0,04556	0,18	0,229	1
Sike1	0,016485	-0,05297	0,396	0,467	1
Sdk2	0,016533	-0,04186	0,076	0,111	1
Cadm1	0,016603	-0,04655	0,767	0,803	1
Gtf2a1	0,01661	0,08254	0,726	0,696	1
Rpl18	0,016614	0,053071	0,993	0,993	1
Nacc1	0,016614	-0,0603	0,282	0,335	1
Tmbim1	0,016724	-0,05118	0,989	0,99	1
Urb2	0,016724	0,035969	0,079	0,049	1
Gucy1a1	0,016736	-0,02053	0,014	0,031	1
Rps11	0,016839	0,057469	0,991	0,985	1
Parp9	0,016876	-0,02303	0,015	0,033	1
Chordc1	0,016884	-0,05778	0,573	0,634	1
Gm46218	0,016907	-0,0446	0,08	0,115	1
Spats1	0,016908	-0,02924	0,031	0,055	1
Cntn1	0,016919	-0,07646	0,086	0,122	1
Zfp526	0,01692	0,016443	0,025	0,008	1
Nqo1	0,016937	-0,06901	0,116	0,154	1
St6gal1	0,016958	0,063614	0,436	0,376	1
Utp20	0,01696	-0,05575	0,13	0,171	1
Strada	0,016961	-0,05581	0,162	0,207	1
Uhrf1bp1	0,016971	-0,03241	0,072	0,106	1
Rusc2	0,017031	0,085467	0,563	0,517	1
Pdpr	0,017079	-0,02994	0,118	0,161	1
Entpd5	0,017093	-0,06848	0,546	0,598	1
Pla2g4a	0,017136	-0,06166	0,356	0,413	1
Psen1	0,017156	-0,05359	0,513	0,572	1
Ncdn	0,017162	-0,06512	0,575	0,618	1
AC129328.	0,017168	-0,0246	0,058	0,089	1
Lap3	0,017182	-0,05983	0,97	0,966	1
Pphln1	0,017278	-0,07051	0,261	0,311	1
Cntrl	0,017322	-0,04445	0,35	0,421	1
Scrn1	0,017323	0,08829	0,471	0,426	1
Fgfrl1	0,017332	-0,07431	0,339	0,4	1
Cby1	0,017356	-0,05362	0,253	0,306	1
Cd47	0,017398	-0,05424	0,985	0,995	1
Itgb5	0,017426	-0,05054	0,427	0,498	1
Ppp2r2a	0,017479	0,068992	0,966	0,956	1
Epb41l4b	0,017483	-0,07531	0,357	0,41	1

Vrk1	0,017588	-0,04163	0,19	0,241	1
Nhlrc2	0,017598	-0,05766	0,297	0,356	1
Rps23	0,017636	0,058001	0,993	0,992	1
Zfp74	0,017646	-0,04159	0,236	0,289	1
Gsn	0,017677	-0,07584	0,952	0,961	1
Trub2	0,017685	-0,07662	0,293	0,343	1
Sorbs3	0,017695	0,076493	0,524	0,483	1
Pick1	0,017747	-0,05611	0,398	0,465	1
Clasp2	0,017804	-0,05994	0,991	0,992	1
Trpv2	0,017815	0,02521	0,05	0,026	1
Rwdd3	0,017846	-0,03006	0,044	0,072	1
Hint3	0,017885	-0,07168	0,557	0,589	1
Nhlrc1	0,017913	-0,03777	0,15	0,197	1
Traf5	0,018002	0,016849	0,013	0,002	1
Olfr920	0,018035	0,012988	0,013	0,002	1
Far1	0,018089	-0,05213	0,735	0,771	1
H2-T24	0,018091	-0,01859	0,004	0,015	1
Zfp991	0,01813	0,017755	0,022	0,007	1
Ccdc15	0,018146	-0,04053	0,079	0,114	1
Calb2	0,01816	-0,01381	0,004	0,015	1
Serp1	0,018162	-0,06045	0,572	0,628	1
Ptpre	0,01819	-0,05485	0,339	0,398	1
Josd2	0,018195	-0,04505	0,996	0,995	1
Btg1	0,018238	0,144462	0,404	0,353	1
Flrt1	0,018294	0,05818	0,124	0,086	1
Fbxo7	0,018308	-0,05921	0,787	0,82	1
Epn2	0,018327	-0,04652	0,983	0,985	1
Gm19325	0,018385	-0,01123	0,004	0,015	1
Gm43951	0,018385	-0,01123	0,004	0,015	1
Arfgap1	0,018443	-0,03659	0,316	0,38	1
Foxp1	0,018452	-0,05228	0,638	0,694	1
Cth	0,018454	-0,00963	0,004	0,015	1
Slc6a11	0,018454	-0,00963	0,004	0,015	1
Rtca	0,018462	-0,03526	0,226	0,281	1
Ppp2ca	0,018478	0,060294	0,995	0,992	1
Gm39129	0,018513	-0,01714	0,012	0,028	1
Ckap2l	0,018542	-0,01026	0,004	0,015	1
Gm10371	0,018588	-0,02713	0,03	0,054	1
Cpped1	0,018609	-0,05013	0,234	0,286	1
Maf	0,01863	-0,01115	0,004	0,015	1
Fdft1	0,018636	0,126374	0,658	0,654	1
Prr13	0,018678	-0,05623	0,413	0,475	1
Sec11c	0,018686	0,062592	0,999	1	1
Aqr	0,018714	-0,05539	0,346	0,403	1
Ccdc3	0,018752	-0,01993	0,012	0,028	1
Prkag1	0,018795	-0,05202	0,373	0,437	1
Mrps27	0,018799	-0,04106	0,082	0,117	1
Itgb1bp1	0,01884	-0,06275	0,371	0,421	1
Zfp943	0,018847	-0,05034	0,154	0,198	1
Atp8a1	0,018851	-0,06265	0,981	0,992	1
Casp6	0,018891	-0,02421	0,026	0,047	1
Vopp1	0,018891	-0,02421	0,026	0,047	1

Zfp72	0,018928	-0,04345	0,073	0,106	1
Map4k3	0,018935	-0,04243	0,185	0,236	1
Wipi1	0,018967	-0,05397	0,862	0,885	1
Rps25	0,019003	0,049288	0,986	0,984	1
Glt8d1	0,019021	-0,03711	0,334	0,397	1
Lrrc28	0,019053	-0,05438	0,393	0,45	1
Mrpl24	0,019078	-0,0619	0,641	0,694	1
Smtnl2	0,019114	-0,04761	0,131	0,172	1
Capn6	0,019126	0,027745	0,031	0,013	1
Tbcd	0,019129	-0,05463	0,357	0,416	1
4933434E2	0,019163	0,060995	0,732	0,688	1
Cpsf7	0,019198	-0,05996	0,428	0,493	1
Sparcl1	0,019218	-0,05051	0,09	0,127	1
2610001J0	0,019278	-0,07328	0,658	0,675	1
Ythdf3	0,019314	-0,04268	0,413	0,481	1
AI429214	0,019321	0,014571	0,019	0,005	1
Stat1	0,01934	-0,3497	0,333	0,384	1
Rpl32	0,019368	0,051113	0,987	0,993	1
Pcyox1l	0,01939	-0,05175	0,348	0,407	1
Gm26603	0,019396	0,018113	0,016	0,003	1
Pdlim5	0,019437	-0,0618	0,305	0,361	1
Phf14	0,019443	-0,0512	0,57	0,633	1
Sdhaf4	0,019469	-0,05807	0,203	0,249	1
Nipbl	0,019475	-0,04525	0,776	0,839	1
Ephx1	0,019479	0,053318	0,187	0,143	1
Stard3	0,019487	-0,06925	0,583	0,628	1
Appl2	0,019493	-0,05156	0,6	0,663	1
Gm14091	0,019495	0,008793	0,009	0	1
2810032G0	0,019495	0,008793	0,009	0	1
AI463170	0,019495	0,008793	0,009	0	1
Hbb-bt	0,019495	1,048988	0,009	0	1
Srgn	0,019495	0,010737	0,009	0	1
Klf5	0,019495	0,010737	0,009	0	1
Mb21d1	0,019495	0,010737	0,009	0	1
St14	0,019495	0,011707	0,009	0	1
Cish	0,019495	0,011707	0,009	0	1
Timm17a	0,019523	-0,06266	0,657	0,717	1
5930422O1	0,019533	-0,01602	0,005	0,016	1
Prune2	0,019542	0,074577	0,281	0,229	1
Scg3	0,019578	-0,05711	0,165	0,208	1
Rpusd2	0,019623	-0,01301	0,011	0,026	1
R3hdm1	0,019668	-0,07353	0,803	0,847	1
Herc6	0,019672	-0,09609	0,11	0,148	1
Gm42421	0,01974	0,012644	0,016	0,003	1
Pias4	0,01976	-0,04747	0,426	0,485	1
Gm20387	0,01979	-0,01123	0,005	0,016	1
Mzt2	0,019811	-0,04773	0,178	0,224	1
Fgl2	0,019837	0,053027	0,05	0,026	1
Gpr27	0,01984	0,020554	0,026	0,01	1
Gnaz	0,019927	-0,02281	0,048	0,076	1
Paqr9	0,01999	0,020775	0,031	0,013	1
Snapc3	0,019999	-0,05121	0,272	0,327	1

Gm14418	0,020087	-0,02015	0,035	0,06	1
Gys1	0,020105	-0,01265	0,011	0,026	1
Hnrnph1	0,020147	0,10611	0,84	0,807	1
Cep57	0,020156	-0,04394	0,174	0,221	1
Dcun1d2	0,020169	-0,04128	0,216	0,268	1
Vat1	0,020289	0,051737	0,611	0,541	1
AC154782.	0,02029	-0,01045	0,01	0,024	1
Tmem185b	0,020344	-0,02492	0,119	0,161	1
Hus1	0,020382	-0,05319	0,245	0,294	1
Ckb	0,020396	0,076598	0,997	0,99	1
Me2	0,020422	-0,06439	0,348	0,402	1
Lmo7	0,020425	-0,03588	0,042	0,068	1
Vsnl1	0,020442	-0,01945	0,019	0,037	1
Prkar2a	0,020443	-0,05421	0,184	0,231	1
Sugp2	0,020461	-0,04747	0,218	0,268	1
Lancl2	0,020474	-0,05174	0,211	0,26	1
Gm4951	0,020589	-0,0373	0,001	0,008	1
Gm42372	0,020626	-0,05828	0,253	0,304	1
Klf15	0,020647	-0,0399	0,237	0,291	1
Zfp354c	0,020657	-0,05225	0,144	0,187	1
Tceal6	0,020674	-0,00873	0,001	0,008	1
Tbc1d22bc	0,020674	-0,00873	0,001	0,008	1
Tpgs1	0,020694	-0,05275	0,576	0,626	1
Ncbp3	0,020699	-0,05954	0,287	0,343	1
Nat8l	0,020702	-0,00712	0,001	0,008	1
Aoc2	0,020702	-0,00712	0,001	0,008	1
Tmem179	0,020702	-0,00712	0,001	0,008	1
Rmi2	0,020702	-0,00712	0,001	0,008	1
Fam111a	0,020702	-0,00712	0,001	0,008	1
Ldlrad4	0,020722	0,080626	0,482	0,441	1
Tspyl5	0,020756	-0,03348	0,051	0,08	1
Cnot10	0,020762	-0,05288	0,379	0,433	1
Inca1	0,020812	-0,01274	0,009	0,023	1
Gm44148	0,020816	-0,00775	0,001	0,008	1
AI463229	0,020816	-0,00775	0,001	0,008	1
Zfp810	0,020824	-0,01873	0,019	0,037	1
K230010J2	0,020844	-0,00614	0,001	0,008	1
Plppr5	0,020863	-0,02298	0,029	0,052	1
Abca7	0,020878	-0,02635	0,135	0,18	1
Tubb3	0,020889	0,044253	0,673	0,613	1
A830010M	0,020909	-0,04802	0,476	0,533	1
Phkb	0,020989	-0,04192	0,292	0,351	1
Pyurf	0,02104	-0,04536	0,234	0,285	1
Alg2	0,021057	-0,0639	0,313	0,364	1
Pign	0,021059	-0,056	0,231	0,28	1
Phka1	0,021075	-0,03581	0,116	0,156	1
Asb3	0,021124	-0,04361	0,191	0,241	1
Akap11	0,021132	0,070333	0,801	0,772	1
Iffo1	0,021151	0,077942	0,475	0,424	1
Map7d2	0,021203	-0,08899	0,316	0,361	1
B230398E0	0,021246	-0,01212	0,007	0,02	1
Rnf181	0,021249	-0,05991	0,776	0,823	1

Rpap3	0,02136	-0,05717	0,216	0,263	1
Rabep1	0,021438	-0,054	0,472	0,53	1
Ttc8	0,021465	-0,04108	0,199	0,249	1
Morf4l1	0,021486	-0,0496	0,997	1	1
Srp19	0,021525	-0,04542	0,714	0,776	1
Gm43598	0,021635	-0,02051	0,029	0,052	1
Ptpn14	0,021695	0,055789	0,042	0,021	1
Tubb2b	0,021787	-0,19386	0,146	0,185	1
Zbtb42	0,021812	0,023774	0,024	0,008	1
Slc43a2	0,021833	-0,05776	0,633	0,678	1
Slc39a7	0,0219	0,069679	0,651	0,605	1
Cers4	0,021943	0,060386	0,273	0,223	1
Rps12-ps3	0,021947	0,01901	0,024	0,008	1
Csnk1g3	0,02195	-0,04051	0,405	0,468	1
Ash2l	0,021963	-0,04396	0,392	0,455	1
Rnf187	0,021966	0,0655	0,895	0,894	1
Shank1	0,021971	-0,02545	0,033	0,057	1
Tiam1	0,022114	-0,01427	0,061	0,093	1
Rpp30	0,022177	-0,05742	0,307	0,361	1
Fgf7	0,022178	0,073626	0,121	0,086	1
Mrps21	0,022187	-0,05919	0,695	0,745	1
Rps27rt	0,022224	0,047907	0,253	0,203	1
Crebbp	0,02227	-0,03888	0,614	0,683	1
Sbno2	0,022286	0,246564	0,374	0,338	1
Ctu1	0,022301	-0,02268	0,05	0,078	1
Pcdhgc5	0,022306	-0,02039	0,038	0,063	1
Gatad1	0,022306	-0,04962	0,693	0,753	1
Pmp22	0,022308	0,069534	0,973	0,964	1
Rab26	0,022321	0,021504	0,028	0,011	1
Socs1	0,022322	0,021795	0,024	0,008	1
Sdc4	0,022405	0,066512	0,166	0,127	1
Il1rapl1	0,022451	-0,04508	0,153	0,197	1
Rac1	0,022529	-0,04238	0,993	0,997	1
Ireb2	0,022554	-0,0458	0,352	0,413	1
Gm39459	0,022586	0,021795	0,028	0,011	1
Arid1b	0,022671	0,087681	0,464	0,421	1
Sel1l	0,022718	-0,05247	0,787	0,826	1
Ints6l	0,022862	-0,05139	0,406	0,472	1
Arf1	0,02296	0,050852	0,959	0,946	1
Hs2st1	0,023013	-0,0566	0,36	0,407	1
Gipr	0,023061	-0,04438	0,125	0,166	1
Dctn5	0,023083	-0,04297	0,383	0,447	1
Mgme1	0,023169	-0,05022	0,199	0,246	1
Dgat2	0,023172	0,056888	0,274	0,223	1
Fam32a	0,023381	0,058675	0,443	0,39	1
Ptdss2	0,023404	-0,06363	0,377	0,426	1
Ticam1	0,023405	0,035613	0,059	0,034	1
Sept8	0,023436	-0,04114	0,991	0,997	1
Miga2	0,023446	-0,0341	0,136	0,179	1
Ier5	0,023456	0,064197	0,09	0,06	1
Synj1	0,023501	-0,06006	0,455	0,514	1
Atf7	0,023534	-0,05533	0,542	0,597	1

Numa1	0,023557	-0,05016	0,564	0,623	1
Ccnb1ip1	0,023687	0,015947	0,04	0,02	1
Nfasc	0,02369	-0,05492	0,987	0,985	1
Hnrnpd	0,02374	0,076689	0,713	0,688	1
Tnfrsf12a	0,023836	0,106197	0,115	0,081	1
Src	0,023855	0,0468	0,114	0,08	1
Zbtb40	0,023903	-0,03477	0,074	0,106	1
Pex7	0,024038	-0,04595	0,377	0,436	1
Tmem168	0,024059	-0,03262	0,313	0,377	1
Lrrn1	0,024071	-0,06273	0,919	0,932	1
Tmem39b	0,024104	-0,03066	0,091	0,127	1
Heca	0,024151	-0,03372	0,311	0,371	1
Gopc	0,02416	-0,04542	0,321	0,379	1
Clp1	0,02417	0,045705	0,149	0,111	1
Elmo1	0,024172	-0,06575	0,875	0,917	1
Hnrnpa3	0,024178	0,05937	0,972	0,971	1
Atp13a2	0,024244	-0,05833	0,223	0,27	1
Tatdn1	0,02426	-0,03433	0,206	0,257	1
Fkrp	0,024291	-0,04412	0,203	0,252	1
Ino80c	0,024477	-0,04561	0,227	0,278	1
Trem2	0,024485	0,013916	0,021	0,007	1
Dcbld2	0,024499	0,074357	0,276	0,231	1
Mettl22	0,024535	-0,03887	0,176	0,221	1
Sh3kbp1	0,024539	-0,06666	0,747	0,779	1
Sema6a	0,024549	-0,07346	0,556	0,607	1
Ttc7b	0,024557	-0,03705	0,182	0,229	1
Tspyl4	0,024587	-0,06458	0,714	0,751	1
Tst	0,024597	-0,04205	0,395	0,46	1
Zgrf1	0,024675	-0,02496	0,053	0,081	1
Fam71a	0,02475	0,119472	0,046	0,024	1
Blvrb	0,024784	0,046975	0,088	0,059	1
4632404H1	0,024787	-0,04049	0,083	0,117	1
Nkiras1	0,024803	-0,04293	0,485	0,55	1
Akr7a5	0,02481	0,061146	0,479	0,424	1
Rtn4	0,02482	-0,04447	0,999	1	1
Ercc6l2	0,024929	-0,05889	0,414	0,46	1
Sri	0,024982	-0,04651	0,736	0,798	1
Wbp2	0,024994	-0,0546	0,974	0,99	1
Wdr35	0,02506	-0,04748	0,132	0,172	1
3110009E1	0,0252	-0,03512	0,134	0,176	1
Rbm4b	0,025211	-0,03484	0,452	0,514	1
Cyp2u1	0,025242	-0,04127	0,235	0,283	1
Sfi1	0,02525	-0,07399	0,207	0,25	1
Birc3	0,025253	0,012988	0,012	0,002	1
Hist1h2ae	0,025253	0,013955	0,012	0,002	1
Tbxas1	0,025276	0,014921	0,012	0,002	1
Zfp593	0,025303	-0,03559	0,195	0,244	1
D16Ertd47	0,025323	-0,0593	0,848	0,852	1
Enpp6	0,025411	0,059993	0,851	0,782	1
Rapgef2	0,025461	-0,05751	0,249	0,299	1
Wwp1	0,02564	-0,06354	0,589	0,626	1
Gm35850	0,025665	-0,0221	0,015	0,031	1

4833439L1	0,025676	-0,05985	0,665	0,711	1
Ankrd39	0,025709	-0,03227	0,244	0,299	1
Arfgap3	0,02571	-0,03482	0,337	0,397	1
Zfp786	0,025737	-0,00487	0	0,005	1
Gabra5	0,025737	-0,00487	0	0,005	1
Mgat4c	0,025737	-0,00487	0	0,005	1
Adcy1	0,025737	-0,00487	0	0,005	1
Kcnip1	0,025737	-0,00487	0	0,005	1
D430019H	0,025737	-0,00487	0	0,005	1
Tnfaip2	0,025737	-0,00487	0	0,005	1
Cd80	0,025737	-0,00487	0	0,005	1
Susd4	0,025738	-0,00648	0	0,005	1
Gfra4	0,025738	-0,00648	0	0,005	1
Gm16853	0,025738	-0,00648	0	0,005	1
Col11a1	0,025738	-0,0081	0	0,005	1
Oas1a	0,025738	-0,0081	0	0,005	1
Ccno	0,025738	-0,0081	0	0,005	1
Supt4a	0,025759	-0,0363	0,749	0,818	1
Pigf	0,025786	-0,05206	0,194	0,239	1
Gramd3	0,025893	-0,04145	0,316	0,371	1
Pxylp1	0,025995	-0,04549	0,135	0,176	1
Rb1	0,026071	-0,0336	0,432	0,506	1
Gstz1	0,026072	-0,05239	0,332	0,385	1
Ddx39b	0,026073	0,063765	0,918	0,898	1
Sfpq	0,02608	-0,04234	0,96	0,959	1
Megf9	0,02608	-0,07034	0,519	0,556	1
Tmtc2	0,026109	-0,04851	0,455	0,524	1
Unc13a	0,026162	-0,01957	0,015	0,031	1
Ppp1r14a	0,026166	-0,04745	0,99	0,998	1
Abcb8	0,026197	-0,04537	0,412	0,476	1
Accs	0,026262	-0,01933	0,031	0,054	1
Fubp1	0,026265	-0,04854	0,725	0,784	1
Rasgef1b	0,026271	-0,01738	0,089	0,125	1
Eif4a1	0,026307	0,069606	0,988	0,997	1
Serpinb6a	0,026359	0,074774	0,818	0,795	1
Zfp207	0,026365	-0,05973	0,665	0,72	1
Nfat5	0,026377	-0,07998	0,664	0,709	1
Abhd8	0,026383	-0,018	0,015	0,031	1
Spast	0,026388	-0,04794	0,488	0,551	1
Igsf3	0,026395	0,050781	0,152	0,114	1
Irgm2	0,026432	-0,08656	0,046	0,072	1
Purb	0,026476	0,094879	0,902	0,894	1
Tram1	0,026512	-0,02662	0,395	0,465	1
Cav2	0,026571	-0,08568	0,205	0,249	1
BC028528	0,026584	0,035629	0,127	0,091	1
Zbtb16	0,026599	-0,05195	0,19	0,237	1
Ndufs6	0,026613	-0,05703	0,89	0,912	1
Dpysl5	0,026619	-0,08822	0,187	0,228	1
Mcam	0,026621	0,071032	0,666	0,608	1
H2-T23	0,02671	-0,52087	0,048	0,073	1
Gm2115	0,026898	-0,03855	0,076	0,107	1
Med13l	0,026906	-0,0518	0,425	0,481	1

Psma6	0,026922	-0,04414	0,773	0,813	1
Itih5	0,026982	0,06506	0,171	0,132	1
Zfp523	0,027008	-0,04201	0,227	0,276	1
Irf1	0,027078	0,130805	0,131	0,094	1
Aldh5a1	0,027181	-0,03669	0,194	0,242	1
Tmem42	0,027186	0,047816	0,206	0,163	1
Ndrg3	0,027261	-0,07765	0,561	0,576	1
Znfx1	0,027281	-0,04251	0,4	0,47	1
Tasp1	0,027311	-0,01968	0,052	0,08	1
Unc13d	0,027358	0,024919	0,032	0,015	1
Tac2	0,02736	-0,02005	0,021	0,039	1
Ubr4	0,027366	-0,05033	0,424	0,481	1
Gm17354	0,02742	-0,03531	0,085	0,119	1
Npc2	0,027438	0,063598	0,988	0,989	1
Gin1	0,027475	-0,03527	0,165	0,208	1
Ccdc171	0,027498	0,026509	0,05	0,028	1
Zfp182	0,027529	-0,04614	0,27	0,322	1
Ivd	0,027549	-0,04302	0,357	0,416	1
Ccny	0,027588	-0,033	0,302	0,359	1
Adi1	0,027634	-0,05683	0,791	0,839	1
Fbxo48	0,027693	0,00782	0,008	0	1
Rgs9	0,027693	0,009766	0,008	0	1
Slc9a3	0,027693	0,009766	0,008	0	1
Mgp	0,027693	0,008793	0,008	0	1
Ppm1n	0,027694	0,026152	0,008	0	1
Diexf	0,027806	0,047244	0,151	0,114	1
Ncoa4	0,02789	-0,05249	0,619	0,663	1
Chm	0,027893	-0,0528	0,404	0,46	1
Sf3b5	0,027955	-0,05821	0,639	0,688	1
Cpeb3	0,027963	-0,05333	0,373	0,433	1
Slc25a35	0,027986	-0,02665	0,062	0,091	1
Syf2	0,028025	-0,04839	0,918	0,912	1
Ndst4	0,028082	-0,01739	0,014	0,029	1
Pgm2l1	0,028096	0,041911	0,075	0,047	1
Cenpc1	0,028102	-0,04878	0,298	0,351	1
Zer1	0,028121	-0,06323	0,591	0,616	1
Psmb5	0,028132	-0,05258	0,823	0,849	1
Smyd2	0,02815	-0,05993	0,219	0,262	1
Fam210b	0,028184	-0,04753	0,138	0,177	1
Syt11	0,028184	-0,0448	0,999	1	1
Klhdc4	0,028252	-0,03719	0,185	0,233	1
Snx19	0,028258	-0,03954	0,252	0,304	1
Dhrs7	0,028294	0,052684	0,535	0,485	1
Zfp317	0,02831	-0,03697	0,218	0,267	1
Dtd1	0,02832	-0,07121	0,474	0,519	1
Brcc3	0,028364	-0,04062	0,35	0,405	1
Gm10031	0,028427	-0,03881	0,11	0,146	1
Trim12a	0,028462	-0,02214	0,002	0,01	1
Triobp	0,028481	-0,05297	0,203	0,247	1
Ndc1	0,028487	-0,05964	0,202	0,246	1
Ammecr1	0,028497	-0,02265	0,083	0,117	1
Pcdhga9	0,028512	-0,02329	0,043	0,068	1

Slc45a4	0,028653	-0,0288	0,05	0,076	1
Zfyve26	0,028683	-0,04443	0,354	0,411	1
Ube2j1	0,028715	-0,04155	0,26	0,314	1
Ggnbp1	0,028726	-0,03548	0,108	0,145	1
Hsp90b1	0,028745	-0,05799	0,992	0,997	1
Copg2	0,028758	-0,05915	0,398	0,454	1
Lins1	0,028758	-0,02953	0,079	0,112	1
Cope	0,028831	-0,05015	0,792	0,831	1
Lgi2	0,028853	-0,00775	0,002	0,01	1
2810425M	0,028853	-0,00775	0,002	0,01	1
Cck	0,028853	-0,00775	0,002	0,01	1
Gm15491	0,028853	-0,00775	0,002	0,01	1
Tfrc	0,028853	-0,06608	0,384	0,437	1
Ifit2	0,028925	-0,08334	0,208	0,254	1
Rpl36a	0,028933	0,05094	0,954	0,92	1
Gm47547	0,028967	-0,01449	0,014	0,029	1
Srrd	0,028972	-0,04802	0,175	0,216	1
Dysf	0,028984	-0,00838	0,002	0,01	1
Spata9	0,028984	-0,00838	0,002	0,01	1
Ttc26	0,029031	0,01901	0,023	0,008	1
Cdkl1	0,02905	-0,00579	0,002	0,01	1
Id3	0,029052	-0,03297	0,054	0,081	1
Abhd17c	0,029076	-0,03638	0,197	0,246	1
Wdr78	0,029119	0,01901	0,023	0,008	1
Ccdc58	0,029186	-0,03968	0,177	0,221	1
Dock4	0,029311	-0,04303	0,403	0,459	1
Sorcs1	0,029335	-0,04281	0,291	0,345	1
Foxo1	0,029401	-0,06391	0,362	0,408	1
Lrrc47	0,029459	-0,04124	0,285	0,34	1
Wnk3	0,029511	-0,03568	0,068	0,098	1
Gm37885	0,029518	0,018947	0,027	0,011	1
Cdc14b	0,029522	-0,03354	0,091	0,125	1
Chl1	0,029524	-0,02953	0,013	0,028	1
Cstf2t	0,029548	-0,05199	0,324	0,377	1
Rmdn1	0,029632	0,06938	0,29	0,249	1
Tom1	0,02964	-0,04397	0,4	0,46	1
Csf1	0,029678	0,089799	0,822	0,792	1
Mxra7	0,029799	0,047385	0,114	0,081	1
Apopt1	0,029869	-0,04716	0,492	0,551	1
2700016F2	0,029914	-0,05297	0,126	0,163	1
Gm1673	0,029955	-0,01757	0,035	0,059	1
Dis3	0,029987	-0,03182	0,15	0,193	1
Tsc1	0,030042	-0,07904	0,565	0,61	1
Cad	0,030056	-0,02753	0,079	0,111	1
Asap3	0,030059	0,020484	0,034	0,016	1
Fam13b	0,030114	-0,04542	0,26	0,311	1
Fyco1	0,030141	-0,0623	0,336	0,379	1
Bet1	0,030452	-0,05171	0,684	0,75	1
Tnni1	0,030488	-0,04657	0,658	0,72	1
Agl	0,030505	-0,04458	0,196	0,244	1
Gm26853	0,030527	-0,0383	0,248	0,299	1
Epc2	0,030564	-0,03392	0,348	0,411	1

Med12l	0,030565	-0,01681	0,02	0,037	1
Ppp1r9a	0,030599	-0,05675	0,473	0,527	1
Fosl2	0,03068	0,053225	0,127	0,093	1
Tbc1d14	0,030686	-0,04655	0,685	0,728	1
Ubr5	0,03071	0,058407	0,58	0,514	1
Zfp966	0,030898	-0,01957	0,025	0,044	1
Aldh4a1	0,030947	-0,03074	0,139	0,18	1
Banp	0,031045	-0,03889	0,238	0,288	1
H1f0	0,031054	0,089233	0,605	0,551	1
Dnajc12	0,031072	-0,02885	0,108	0,145	1
Ube2f	0,031097	-0,05108	0,275	0,324	1
Cmtm5	0,031103	0,050818	0,999	1	1
Efna1	0,031202	0,089752	0,344	0,307	1
Fut11	0,03122	-0,04522	0,162	0,203	1
Phf21a	0,031237	-0,03723	0,392	0,45	1
Atp8a2	0,031262	-0,01714	0,012	0,026	1
Hdhd2	0,031279	-0,04569	0,626	0,675	1
Map4k2	0,031499	-0,05254	0,247	0,296	1
Eif3h	0,031522	-0,03663	0,803	0,836	1
Gm34552	0,03174	-0,018	0,012	0,026	1
Atg4a	0,031753	-0,03444	0,131	0,169	1
Cenpx	0,031759	-0,05264	0,448	0,502	1
Arl8a	0,031883	0,042284	0,976	0,974	1
Abraxas1	0,031902	-0,0184	0,042	0,067	1
H2-T22	0,03191	-0,09414	0,091	0,122	1
Prrc2c	0,031969	-0,03922	0,959	0,95	1
Mrps35	0,031979	-0,03857	0,216	0,263	1
Mpv17l2	0,032003	-0,04614	0,352	0,405	1
Gpr45	0,032025	-0,01301	0,012	0,026	1
Ing4	0,032042	-0,04871	0,561	0,608	1
Gm45871	0,032045	-0,03368	0,074	0,104	1
Mthfd2	0,032056	-0,04162	0,357	0,41	1
Ankrd26	0,032087	-0,04389	0,325	0,38	1
Gm29417	0,032185	0,022296	0,036	0,018	1
Gss	0,03224	-0,08935	0,451	0,489	1
Ppp1r10	0,032271	0,16211	0,523	0,494	1
Sqstm1	0,032293	0,056231	0,946	0,932	1
Map3k5	0,032369	-0,02262	0,096	0,132	1
Bdkrb2	0,03241	-0,04325	0,109	0,145	1
Gt(ROSA)26	0,032552	-0,01465	0,019	0,036	1
Prkcz	0,032563	-0,03462	0,888	0,919	1
Hnrnpc	0,032571	-0,04882	0,946	0,967	1
Pfn2	0,032618	-0,04805	0,751	0,805	1
Zc4h2	0,032622	-0,04715	0,191	0,234	1
Pcdhgb6	0,032631	-0,02289	0,028	0,049	1
Alg8	0,032663	0,029879	0,068	0,042	1
Abhd18	0,032722	-0,0383	0,233	0,283	1
Txndc17	0,032819	-0,05391	0,701	0,763	1
Slc25a51	0,032843	-0,05969	0,629	0,672	1
B230219D:	0,032866	-0,0504	0,846	0,878	1
2010107E(	0,032914	-0,05771	0,838	0,865	1
Zfp157	0,032943	-0,03725	0,275	0,33	1

Ccni	0,032947	-0,04517	0,679	0,712	1
Gm45768	0,032989	0,082559	0,52	0,473	1
Tmcc3	0,032996	0,073923	0,994	0,997	1
Gm16638	0,033006	-0,0346	0,097	0,132	1
Rcn2	0,033056	0,074473	0,411	0,369	1
Smc1a	0,033113	-0,03294	0,739	0,779	1
Selenok	0,033125	0,053897	1	1	1
Hhat	0,033155	0,01134	0,02	0,007	1
Gtf2h4	0,033191	-0,03634	0,118	0,154	1
Cfdp1	0,033228	-0,05023	0,769	0,792	1
Phgdh	0,033342	-0,04702	0,984	0,993	1
Ankrd54	0,033364	-0,03872	0,207	0,252	1
Sapcd1	0,033396	0,021212	0,025	0,01	1
Lsm14b	0,033436	-0,04582	0,341	0,392	1
C920021L1	0,03346	-0,02352	0,045	0,07	1
Smim27	0,033481	-0,04686	0,322	0,377	1
Azin1	0,033497	0,102895	0,42	0,377	1
AU040320	0,033527	-0,04537	0,314	0,367	1
Fbl	0,033554	0,105188	0,637	0,598	1
Rbm28	0,033565	-0,06156	0,539	0,59	1
Dscam	0,03357	0,014791	0,029	0,013	1
Smad9	0,033624	0,017398	0,025	0,01	1
Camk2n1	0,033628	-0,07101	0,501	0,54	1
AC162302.	0,033689	-0,03103	0,048	0,073	1
Zfp395	0,033717	0,059295	0,243	0,2	1
Hr	0,033854	0,052426	0,25	0,207	1
Sox2	0,033879	-0,00829	0,752	0,784	1
Hmbox1	0,033886	-0,03243	0,42	0,486	1
Lcn2	0,034091	-0,00998	0,003	0,011	1
Gm14862	0,034091	-0,00998	0,003	0,011	1
Kcnj16	0,034091	-0,00998	0,003	0,011	1
Tmem47	0,034105	-0,02567	0,032	0,054	1
Grm4	0,034153	-0,01424	0,011	0,024	1
Psip1	0,034157	-0,04295	0,925	0,945	1
Rbms3	0,034192	-0,00838	0,003	0,011	1
Acss1	0,034217	-0,01764	0,018	0,034	1
Cct2	0,034255	-0,04542	0,826	0,857	1
Polr2a	0,03429	0,08263	0,53	0,502	1
Tgfb2	0,03432	-0,05924	0,043	0,067	1
Gm12689	0,034389	-0,03946	0,081	0,112	1
Klhl13	0,034427	-0,02885	0,052	0,078	1
Col22a1	0,034431	-0,0074	0,003	0,011	1
Spats2	0,034431	-0,0074	0,003	0,011	1
Atl2	0,034446	0,040459	0,154	0,117	1
Plod1	0,034451	0,071263	0,718	0,694	1
Timm50	0,034508	-0,04847	0,376	0,428	1
Nradd	0,034605	0,026752	0,078	0,05	1
9530077C(	0,034623	-0,03157	0,092	0,125	1
Nck1	0,03463	-0,03948	0,208	0,254	1
Fsd1	0,034634	0,065898	0,359	0,319	1
Taf5l	0,034722	-0,01978	0,097	0,132	1
Ppp2r5c	0,034761	-0,05701	0,804	0,836	1

Dapk2	0,034795	-0,04308	0,133	0,171	1
Gpcpd1	0,034816	-0,0343	0,156	0,197	1
Rps26	0,034824	0,056792	0,988	0,989	1
Micall2	0,034834	0,022142	0,049	0,028	1
Rps16	0,034886	0,056908	0,992	0,99	1
Ubac1	0,034983	-0,03397	0,422	0,483	1
4931440F1	0,035	-0,01714	0,01	0,023	1
Bag3	0,035086	-0,0355	0,287	0,34	1
Dse	0,035169	-0,02037	0,049	0,075	1
Gpat4	0,035171	0,071947	0,472	0,441	1
Notch2	0,035222	0,040124	0,132	0,098	1
Cdh1	0,035263	0,028344	0,011	0,002	1
Flii	0,035291	0,059575	0,5	0,455	1
Cep290	0,035309	-0,04167	0,282	0,337	1
C2cd4c	0,035363	0,014571	0,017	0,005	1
Iars2	0,035381	-0,04166	0,274	0,324	1
Ccl4	0,035422	0,012988	0,011	0,002	1
Gramd1a	0,035433	-0,04374	0,241	0,288	1
Glyctk	0,035437	0,013608	0,017	0,005	1
Ephb6	0,035454	0,012021	0,011	0,002	1
Nmnat2	0,035486	0,011052	0,011	0,002	1
Paqr4	0,035528	-0,05741	0,596	0,633	1
Cyp46a1	0,035537	-0,0315	0,073	0,102	1
Hspd1	0,035537	-0,03495	0,868	0,885	1
Mthfs	0,035557	-0,04217	0,326	0,379	1
Cyb561d1	0,035621	-0,02642	0,077	0,107	1
Tfg	0,035626	-0,0539	0,595	0,631	1
Psen2	0,035779	-0,03671	0,415	0,476	1
Armc1	0,035786	-0,06043	0,67	0,694	1
Eno2	0,035789	0,065709	0,184	0,146	1
Kcna6	0,035804	-0,05394	0,792	0,813	1
Tub	0,035922	-0,0239	0,017	0,033	1
Ifih1	0,035922	-0,03012	0,017	0,033	1
C77080	0,036013	0,020123	0,031	0,015	1
Nat8f1	0,036023	-0,05876	0,271	0,317	1
Sv2c	0,03604	0,034175	0,017	0,005	1
Vps13a	0,036064	-0,04288	0,321	0,374	1
Ryk	0,036082	-0,06815	0,518	0,564	1
Dtwd2	0,036097	-0,02671	0,07	0,099	1
Tmem131l	0,03614	0,014529	0,017	0,005	1
Snrnp70	0,036142	0,075225	0,976	0,979	1
Mrps12	0,03619	-0,06255	0,489	0,528	1
Arcn1	0,036228	-0,0564	0,612	0,66	1
Surf4	0,036273	-0,04929	0,42	0,478	1
Uba2	0,036276	-0,05255	0,632	0,673	1
Lpcat2	0,036324	-0,08693	0,342	0,385	1
Hsp90aa1	0,036332	-0,03403	0,999	1	1
Rpl37	0,036359	0,044488	0,997	0,998	1
Samm50	0,036387	-0,04817	0,512	0,559	1
Cyth2	0,036435	0,058515	0,598	0,543	1
Ppp2r3c	0,036498	-0,03309	0,374	0,434	1
Triap1	0,036499	-0,03325	0,226	0,275	1

Gm6598	0,036575	-0,01921	0,017	0,033	1
Yipf3	0,036649	-0,0434	0,58	0,626	1
Sh3d19	0,03667	0,093666	0,468	0,434	1
Pkm	0,036723	0,065735	0,887	0,893	1
Nkx2-2	0,036754	-0,06495	0,518	0,571	1
Laptm4b	0,036829	-0,04582	0,786	0,805	1
Scoc	0,036863	-0,04468	0,813	0,85	1
Chmp2b	0,036955	-0,05668	0,589	0,626	1
Pitpnm1	0,036979	0,012333	0,014	0,003	1
Gm45518	0,036981	0,026054	0,014	0,003	1
Cpsf6	0,036996	-0,04763	0,615	0,662	1
Fam221b	0,037036	0,011366	0,014	0,003	1
Xntrpc	0,037036	0,011366	0,014	0,003	1
Mitf	0,037039	-0,04058	0,273	0,324	1
Zfp277	0,03708	-0,08781	0,271	0,32	1
Slc5a11	0,037092	-0,04473	0,05	0,075	1
Trps1	0,037138	-0,05266	0,477	0,522	1
Cpxm2	0,037183	-0,01061	0,004	0,013	1
Dab2	0,037183	-0,017	0,004	0,013	1
Otud7b	0,037288	-0,04502	0,977	0,984	1
Ntng2	0,037316	-0,00901	0,004	0,013	1
Hes3	0,037316	-0,00901	0,004	0,013	1
Spryd7	0,037321	-0,05192	0,406	0,455	1
Nlgn3	0,037322	-0,01779	0,027	0,047	1
Nudt8.1	0,037335	-0,02312	0,031	0,052	1
Nlgn1	0,037391	-0,03037	0,055	0,081	1
Rab3ip	0,037422	-0,02758	0,172	0,216	1
Adnp	0,03746	-0,03128	0,11	0,145	1
Mapk3	0,037471	0,056669	0,979	0,977	1
Psmg3	0,037515	-0,05175	0,319	0,366	1
Cyp51	0,037549	0,1358	0,705	0,688	1
Mroh3	0,037576	-0,0153	0,008	0,02	1
Kcnc3	0,037585	-0,00705	0,004	0,013	1
Zfp994	0,037589	-0,03639	0,08	0,111	1
Iglon5	0,037591	0,028794	0,022	0,008	1
Anp32b	0,0376	0,056007	0,914	0,873	1
Faim2	0,037606	-0,04652	0,913	0,932	1
Srsf6	0,037612	-0,05183	0,701	0,738	1
Dck	0,03763	0,021138	0,026	0,011	1
Ubac2	0,037646	-0,04489	0,315	0,367	1
Cpt1a	0,037663	-0,02668	0,043	0,067	1
Perp	0,037679	-0,0131	0,008	0,02	1
Mib1	0,037688	-0,04096	0,374	0,428	1
Kdm3b	0,037778	-0,04468	0,286	0,337	1
Zic3	0,037855	-0,00413	0,004	0,013	1
1700047M	0,03786	0,061938	0,933	0,925	1
Stau1	0,037866	-0,04059	0,395	0,447	1
Akap1	0,037888	-0,03274	0,193	0,239	1
Cnih2	0,037888	-0,0115	0,008	0,02	1
Otud1	0,037911	-0,04528	0,105	0,138	1
Atp6v1g1	0,037917	-0,0484	0,968	0,977	1
Rtl5	0,037932	-0,01353	0,017	0,033	1

Pigz	0,037948	-0,12668	0,775	0,772	1
Sf3b4	0,037967	-0,04488	0,454	0,512	1
Gjb2	0,037995	-0,01212	0,008	0,02	1
Slamf1	0,038079	0,032043	0,033	0,016	1
Slc50a1	0,038079	-0,04836	0,742	0,779	1
Prkrip1	0,038148	-0,04318	0,281	0,33	1
Tmem234	0,038247	-0,04467	0,931	0,953	1
Auh	0,038262	-0,04859	0,695	0,748	1
Ndst1	0,038289	0,058893	0,627	0,584	1
Mtmr11	0,038291	0,017098	0,022	0,008	1
Apeh	0,038312	-0,03441	0,084	0,115	1
Dyrk1b	0,038336	-0,0382	0,131	0,167	1
Tmem74	0,038348	0,028053	0,067	0,042	1
Slc25a1	0,038357	-0,06031	0,501	0,538	1
Sat1	0,038404	-0,06488	0,644	0,694	1
Gm10471	0,038404	0,017098	0,022	0,008	1
Gm38394	0,038563	-0,01945	0,048	0,073	1
Derl1	0,038565	-0,04889	0,756	0,805	1
Tyrobp	0,038574	0,016692	0,033	0,016	1
Nudt7	0,038603	-0,03287	0,117	0,153	1
Gm45250	0,03865	-0,0204	0,026	0,046	1
Fam178b	0,038694	0,033791	0,098	0,068	1
Dnm1	0,038705	-0,02174	0,016	0,031	1
Prkaa1	0,038784	-0,03804	0,262	0,312	1
Mlip	0,03882	-0,01088	0,007	0,018	1
Akap9	0,038824	-0,03088	0,778	0,842	1
Tril	0,038833	-0,00963	0,005	0,015	1
Gm16183	0,038833	-0,00963	0,005	0,015	1
Gramd4	0,038842	-0,03921	0,132	0,169	1
Ccnyl1	0,038842	-0,02936	0,212	0,259	1
Pts	0,038866	-0,04726	0,269	0,314	1
Khdrbs3	0,03897	-0,05328	0,263	0,306	1
Cycs	0,038987	-0,05086	0,738	0,78	1
Arxes2	0,039038	-0,01186	0,006	0,016	1
Dynll1	0,039094	0,063683	0,995	0,993	1
Spag5	0,039122	-0,00866	0,005	0,015	1
Fus	0,03919	0,054205	0,989	0,99	1
Btbd3	0,039203	-0,06687	0,354	0,398	1
Mapre3	0,039214	0,052186	0,59	0,546	1
Serpinb1b	0,039218	-0,01026	0,006	0,016	1
Eef2	0,039259	0,051948	0,999	1	1
B3galnt2	0,039263	-0,02842	0,095	0,128	1
Gm597	0,039276	-0,00956	0,007	0,018	1
Ulk2	0,039277	-0,05747	0,558	0,611	1
Klhdc10	0,039302	-0,03979	0,199	0,246	1
Itm2c	0,039311	0,048222	0,941	0,935	1
Hyou1	0,039336	-0,04757	0,248	0,298	1
Slc27a3	0,03934	-0,01088	0,006	0,016	1
Rgs11	0,039447	-0,00893	0,007	0,018	1
Porcn	0,039468	-0,02382	0,212	0,262	1
Gm21284	0,039521	0,006846	0,007	0	1
Gm47071	0,039521	0,006846	0,007	0	1

Phldb3	0,039521	0,00782	0,007	0	1
Dnhd1	0,039521	0,00782	0,007	0	1
Lrrc63	0,039521	0,00782	0,007	0	1
Spata19	0,039521	0,009766	0,007	0	1
Gm47319	0,039521	0,009766	0,007	0	1
Lag3	0,039521	0,011707	0,007	0	1
Itga2	0,039521	0,010737	0,007	0	1
Gm6793	0,039707	-0,00476	0,005	0,015	1
Ap2m1	0,039777	0,041589	0,937	0,928	1
Pld1	0,039797	-0,06939	0,346	0,387	1
Pcsk6	0,039804	-0,07263	0,602	0,616	1
Lsamp	0,03985	-0,03694	0,071	0,099	1
Coq10a	0,039861	-0,05055	0,379	0,429	1
Tcf7l2	0,039934	-0,0437	0,29	0,346	1
Actr8	0,03994	-0,03969	0,342	0,395	1
Ifnar1	0,039946	-0,03106	0,242	0,289	1
Uqcrfs1	0,039963	-0,04166	0,669	0,707	1
Washc3	0,039977	-0,02811	0,26	0,312	1
Polr2h	0,040003	0,055017	0,297	0,254	1
Limd1	0,040078	-0,03847	0,438	0,494	1
Trp53rkb	0,040081	-0,02773	0,084	0,115	1
Ubl7	0,040106	-0,05325	0,369	0,415	1
Nr2c2	0,040113	-0,03932	0,473	0,528	1
Ndrg1	0,040229	0,04896	0,999	1	1
Fabp5	0,040277	-0,07317	0,996	0,995	1
Ankib1	0,040336	-0,05598	0,797	0,831	1
Eea1	0,040442	-0,03915	0,5	0,572	1
Calu	0,040451	0,064252	0,488	0,454	1
Dcaf11	0,040457	-0,04346	0,444	0,501	1
Cmc1	0,040473	-0,04925	0,349	0,393	1
Ninj2	0,040534	-0,04305	0,612	0,662	1
Rbm26	0,040541	-0,04203	0,613	0,66	1
Srrm2	0,040551	-0,03747	0,995	0,992	1
Slc35a2	0,040586	-0,05055	0,617	0,663	1
Cks1b	0,040614	0,01841	0,044	0,024	1
Rps6ka4	0,040714	0,045092	0,225	0,184	1
Zfp626	0,040784	-0,02595	0,161	0,202	1
Kat2a	0,04087	-0,04252	0,174	0,215	1
Coasy	0,040891	-0,06044	0,408	0,45	1
Gm20300	0,040937	-0,05091	0,353	0,405	1
Gm49322	0,040952	0,026924	0,092	0,063	1
Dennd5b	0,040954	-0,05339	0,529	0,572	1
Ermp1	0,040978	-0,05396	0,902	0,927	1
Arl6ip1	0,041115	-0,03549	0,991	0,984	1
Cdk8	0,041291	-0,04404	0,202	0,244	1
Tnfaip6	0,041303	0,086638	0,939	0,925	1
Osgin2	0,041401	-0,03039	0,235	0,283	1
Nectin1	0,04146	0,069348	0,552	0,499	1
Cnn3	0,041475	-0,04856	0,757	0,793	1
Myl6b	0,041489	0,020077	0,065	0,041	1
Gapdh	0,041507	0,052216	0,998	0,998	1
Brip1os	0,041591	-0,0438	0,172	0,211	1

Rpn1	0,041668	-0,05243	0,774	0,813	1
Mtg1	0,041696	-0,03777	0,161	0,2	1
Slc37a3	0,041732	-0,0268	0,254	0,306	1
Zkscan8	0,041771	-0,03334	0,172	0,213	1
Cdk7	0,041776	0,045608	0,224	0,182	1
Limch1	0,041848	0,049274	0,976	0,974	1
Vamp5	0,041862	0,085884	0,472	0,434	1
1110002L(	0,041904	-0,03688	0,101	0,133	1
Zfp866	0,04197	-0,02464	0,178	0,223	1
Zfp617	0,042026	-0,06572	0,212	0,25	1
Thoc2	0,042033	-0,06116	0,631	0,68	1
Ispd	0,04213	-0,0395	0,193	0,237	1
Ephx4	0,042139	0,027987	0,052	0,031	1
Spag7	0,042199	-0,07335	0,514	0,553	1
Wipf2	0,042235	-0,06346	0,418	0,459	1
Sspo	0,042238	0,052237	0,159	0,124	1
Sorl1	0,042317	-0,04765	0,695	0,727	1
Trafd1	0,042404	-0,03727	0,217	0,262	1
Vcl	0,042493	-0,03281	0,087	0,119	1
Gemin8	0,042516	-0,04115	0,148	0,185	1
Samhd1	0,042528	0,078573	0,39	0,35	1
Spats2l	0,042532	-0,03652	0,192	0,236	1
Nutf2	0,042596	0,039157	0,185	0,146	1
Magohb	0,042666	-0,04228	0,136	0,172	1
Lpcat3	0,042693	-0,04312	0,25	0,298	1
Cmtm3	0,042732	0,034042	0,141	0,106	1
Ostf1	0,042811	-0,05345	0,348	0,395	1
Trim39	0,042832	-0,03857	0,199	0,246	1
Phldb1	0,042871	0,066573	0,98	0,98	1
Manbal	0,042873	-0,0514	0,551	0,6	1
Ddx42	0,042894	0,06653	0,492	0,454	1
Pla2g7	0,042918	-0,01424	0,015	0,029	1
Ykt6	0,042962	-0,04849	0,315	0,363	1
Wwc2	0,043013	-0,03602	0,3	0,351	1
Prpf4b	0,04308	-0,04297	0,87	0,899	1
Atp1a1	0,043088	0,057973	0,993	0,992	1
Rfk	0,043117	-0,03868	0,347	0,397	1
Zbtb24	0,04324	-0,03529	0,139	0,177	1
Rbbp5	0,043262	-0,03525	0,256	0,301	1
Nmu	0,043308	0,027185	0,024	0,01	1
Mtx1	0,043309	-0,04482	0,295	0,345	1
Msl3	0,043363	-0,04213	0,326	0,377	1
Tab2	0,043398	-0,06369	0,663	0,689	1
Lyrm2	0,043409	-0,04631	0,347	0,393	1
Scp2	0,043475	0,047154	0,93	0,917	1
Nudt3	0,043551	-0,03719	0,825	0,878	1
Wdtc1	0,043622	-0,04602	0,362	0,415	1
Fbxl21	0,043797	-0,01231	0,026	0,044	1
Mlycd	0,043798	-0,03389	0,244	0,293	1
Msi2	0,043806	-0,0546	0,645	0,678	1
Prnp	0,043852	0,039116	0,994	1	1
Cuedc1	0,043941	-0,05105	0,706	0,764	1

Atp5f1	0,043965	-0,04049	0,943	0,941	1
Jade1	0,044036	0,06506	0,52	0,473	1
Fam102a	0,044075	0,062098	0,924	0,927	1
Rhoj	0,044097	0,079611	0,294	0,254	1
Kank3	0,044102	0,014529	0,024	0,01	1
Pibf1	0,044172	-0,03034	0,368	0,431	1
Rps6ka1	0,044239	-0,04917	0,261	0,302	1
Acox1	0,044241	0,072572	0,658	0,624	1
Bche	0,04433	-0,03636	0,286	0,337	1
Zcchc7	0,044396	-0,07598	0,801	0,824	1
Brinp3	0,04449	-0,02429	0,033	0,054	1
Lbr	0,044501	-0,06464	0,533	0,584	1
Slc25a17	0,044537	-0,02858	0,289	0,34	1
Banf2os	0,044538	0,024919	0,03	0,015	1
Ptpn1	0,044669	-0,04816	0,336	0,384	1
Slc6a6	0,044726	0,037541	0,94	0,93	1
Exosc2	0,044801	-0,05456	0,214	0,255	1
Tox3	0,044835	-0,03209	0,025	0,042	1
Snu13	0,044853	0,052178	0,922	0,878	1
Ndufb10	0,044911	-0,04719	0,891	0,906	1
Erp44	0,044962	-0,0469	0,434	0,481	1
Stmn3	0,044976	-0,03046	0,048	0,072	1
Ddt	0,044982	-0,04443	0,536	0,579	1
4932422M	0,045041	0,03546	0,068	0,044	1
Rad23b	0,045155	0,071588	0,566	0,537	1
Arglu1	0,045161	-0,04654	0,935	0,951	1
N6amt1	0,045166	-0,03542	0,19	0,234	1
Pabpc5	0,045352	-0,01678	0,014	0,028	1
Traf4	0,045352	-0,01678	0,014	0,028	1
Pum1	0,045365	0,070769	0,774	0,75	1
Tmsb15b1	0,045421	-0,0302	0,066	0,093	1
Pgap1	0,04567	-0,05367	0,402	0,45	1
Tob2	0,045681	0,106744	0,672	0,65	1
Lrp11	0,045684	-0,01968	0,033	0,054	1
Cux1	0,045705	0,085356	0,58	0,566	1
Lrrc20	0,045714	-0,02347	0,056	0,081	1
Osbpl1a	0,045718	-0,04216	0,985	0,989	1
Dcaf10	0,045722	-0,03024	0,17	0,211	1
Knop1	0,045812	0,069457	0,649	0,6	1
Wdr48	0,045814	-0,03374	0,343	0,397	1
AC126055.	0,045914	-0,0198	0,025	0,042	1
Kif16b	0,045986	-0,03331	0,235	0,283	1
Cnep1r1	0,046004	-0,03903	0,443	0,506	1
Marcksl1	0,046026	0,082589	0,807	0,771	1
Vangl1	0,046036	-0,03136	0,069	0,096	1
Tbcb	0,046037	-0,05052	0,86	0,873	1
Katnal1	0,046039	0,069775	0,57	0,535	1
Myl12b	0,046045	-0,04176	0,603	0,647	1
Caskin2	0,046057	-0,04992	0,297	0,341	1
Gpbp1l1	0,046061	-0,04015	0,528	0,582	1
Aff2	0,046346	-0,0144	0,02	0,036	1
Anxa4	0,046365	0,049858	0,269	0,224	1

Vps18	0,046383	-0,0351	0,24	0,286	1
Pcdhga5	0,046419	-0,03037	0,066	0,093	1
2310009B1	0,046443	-0,02708	0,121	0,156	1
Pcdhga3	0,046472	-0,01265	0,014	0,028	1
Def8	0,046496	-0,05164	0,219	0,26	1
Rhoq	0,046501	-0,03624	0,266	0,315	1
Gm15688	0,046587	-0,02651	0,066	0,093	1
Nubp2	0,04676	-0,04233	0,397	0,452	1
Slc2a8	0,046821	-0,03782	0,198	0,239	1
Bax	0,04689	-0,03281	0,724	0,777	1
Tfdp2	0,046903	-0,04535	0,599	0,642	1
Dkc1	0,046916	0,071982	0,323	0,285	1
Psd3	0,047014	-0,05447	0,5	0,546	1
Mtmr10	0,047027	-0,04449	0,628	0,685	1
Stx4a	0,047072	0,053266	0,816	0,798	1
Cstb	0,047145	0,062619	0,792	0,741	1
Dnajc10	0,04716	-0,03573	0,354	0,413	1
Shisa8	0,04721	0,095591	0,274	0,236	1
Gm31152	0,047241	-0,01091	0,02	0,036	1
Tceal9	0,047274	0,073745	0,58	0,55	1
Slc25a47	0,047315	-0,01611	0,025	0,042	1
Prickle1	0,047329	0,140868	0,458	0,433	1
Stub1	0,047419	-0,04294	0,736	0,78	1
Mrps6	0,047518	-0,04968	0,581	0,621	1
Kif3a	0,04756	0,067577	0,887	0,889	1
Shprh	0,047622	-0,05928	0,478	0,527	1
Pcna-ps2	0,047628	0,018469	0,041	0,023	1
Rcc2	0,047759	-0,01689	0,188	0,233	1
Hoxd3	0,047821	0,013608	0,016	0,005	1
Pgd	0,047824	-0,03244	0,256	0,304	1
Atg2b	0,047866	-0,01716	0,186	0,233	1
Jade2	0,047906	-0,0502	0,21	0,247	1
Tacc3	0,047908	-0,0275	0,111	0,145	1
Gab3	0,047987	0,019669	0,016	0,005	1
Dpf2	0,048106	-0,03724	0,458	0,524	1
5430416N0	0,04814	-0,03564	0,118	0,151	1
Abhd13	0,048144	-0,03495	0,142	0,179	1
Rnf38	0,048231	-0,0377	0,31	0,359	1
Sart1	0,048331	-0,04963	0,369	0,418	1
Commd1	0,048357	-0,05117	0,726	0,766	1
Zc3h7a	0,04836	-0,05002	0,327	0,376	1
Stard9	0,048409	-0,0433	0,239	0,283	1
Rbm48	0,04842	-0,04394	0,257	0,302	1
Negr1	0,048441	-0,05015	0,216	0,257	1
Snx6	0,048543	-0,04127	0,787	0,811	1
Crlf3	0,048561	-0,05122	0,267	0,312	1
Myo1e	0,048596	0,063699	0,599	0,566	1
Hmgxb4	0,048694	-0,03835	0,316	0,371	1
Gcat	0,048747	-0,05052	0,289	0,337	1
Nr4a2	0,048749	0,025295	0,016	0,005	1
Derl2	0,048844	-0,05289	0,467	0,512	1
Fdxr	0,048844	-0,03534	0,365	0,418	1

Ddx17	0,049057	-0,05023	0,873	0,878	1
Stard5	0,049157	-0,04276	0,158	0,195	1
Selenoh	0,049165	-0,0394	0,368	0,421	1
Csrp1	0,049169	-0,04586	0,999	1	1
Fam49b	0,049265	-0,0464	0,634	0,688	1
St3gal5	0,049305	-0,05982	0,44	0,483	1
Atp6v1a	0,049335	-0,05105	0,842	0,86	1
Gm42726	0,049431	0,053656	0,18	0,145	1
Akap12	0,049449	0,035243	0,026	0,011	1
Rasgef1c	0,049455	-0,02191	0,036	0,057	1
Slc38a1	0,0495	-0,02063	0,028	0,047	1
Tsga10	0,049501	-0,04361	0,212	0,259	1
Lamtor3	0,049507	-0,04017	0,354	0,407	1
Camk2d	0,049521	0,072562	0,232	0,193	1
Sh2b2	0,04963	-0,01571	0,019	0,034	1
Stt3a	0,049675	0,068255	0,684	0,644	1
Trim25	0,049753	0,04074	0,122	0,091	1
Dcc	0,04976	-0,03023	0,039	0,06	1
Washc4	0,049818	-0,06102	0,644	0,673	1
Fbxl12	0,049823	-0,03251	0,225	0,27	1
Dgkz	0,049827	0,06841	0,429	0,392	1
Hes7	0,049877	0,011052	0,01	0,002	1
Olfr90	0,049921	0,010083	0,01	0,002	1
Azin2	0,049921	0,011052	0,01	0,002	1
Mettl7a3	0,049921	0,011052	0,01	0,002	1
B9d1os	0,049965	0,009112	0,01	0,002	1
Rfwd3	0,049977	-0,0381	0,282	0,328	1
Slc4a10	0,049998	-0,02502	0,027	0,046	1
A3galt2	0,050009	0,008141	0,01	0,002	1
Rarres2	0,050009	0,008141	0,01	0,002	1
Szrd1	0,050013	-0,03907	0,379	0,429	1
Ppt2	0,050068	-0,03913	0,231	0,275	1
Zc3h15	0,050069	-0,05113	0,702	0,751	1
Gm12185	0,050078	-0,0247	0,001	0,007	1
Otud6b	0,050126	-0,06664	0,437	0,476	1
Ttn	0,050211	-0,01194	0,001	0,007	1
Sh2d3c	0,050235	0,011366	0,01	0,002	1
Senp5	0,050245	-0,03334	0,334	0,384	1
Gm45411	0,050277	-0,00712	0,001	0,007	1
Elavl2	0,050277	-0,00873	0,001	0,007	1
1700110C:	0,050277	-0,01034	0,001	0,007	1
Tnfrsf11a	0,050343	-0,0055	0,001	0,007	1
Fam163a	0,050343	-0,0055	0,001	0,007	1
Lbp	0,050343	-0,0055	0,001	0,007	1
Sema4a	0,050343	-0,0055	0,001	0,007	1
Adgrl2	0,050343	-0,0055	0,001	0,007	1
Ube4bos1	0,050343	-0,0055	0,001	0,007	1
4930540M	0,050343	-0,0055	0,001	0,007	1
Lmo3	0,050343	-0,0055	0,001	0,007	1
Me3	0,050343	-0,0055	0,001	0,007	1
Dnah3	0,050343	-0,0055	0,001	0,007	1
Gm26614	0,050343	-0,0055	0,001	0,007	1

Gm16170	0,050343	-0,0055	0,001	0,007	1
Hist1h4j	0,050343	-0,0055	0,001	0,007	1
Gm26555	0,050343	-0,0055	0,001	0,007	1
Gm807	0,050343	-0,0055	0,001	0,007	1
Olfr63	0,050343	-0,0055	0,001	0,007	1
Gm30198	0,050414	0,00749	0,01	0,002	1
2210406O	0,050441	0,022949	0,034	0,018	1
Tubg1	0,050469	-0,0521	0,248	0,288	1
Eif1ax	0,050473	-0,05117	0,685	0,719	1
Gabra3	0,05056	-0,02538	0,024	0,041	1
Plpp3	0,050563	0,120721	0,699	0,694	1
Hint1	0,050603	-0,03826	0,971	0,976	1
Reep1	0,05061	-0,00516	0,001	0,007	1
Tubb6	0,050673	0,014263	0,013	0,003	1
Fbxl19	0,050676	-0,03889	0,132	0,166	1
Mtx2	0,050698	0,060738	0,555	0,528	1
Col2a1	0,050738	-0,01956	0,032	0,052	1
Spef1	0,050904	0,012333	0,013	0,003	1
2610507B	0,050934	-0,06133	0,642	0,667	1
Gm21269	0,051059	0,00943	0,013	0,003	1
Gm29325	0,051131	-0,02887	0,079	0,107	1
Nelfe	0,051154	0,060385	0,382	0,341	1
Grk5	0,051222	-0,01446	0,028	0,047	1
Cdk9	0,05127	0,052451	0,405	0,363	1
Emc1	0,051299	0,056145	0,743	0,715	1
Herc3	0,051303	-0,03567	0,083	0,112	1
Tns1	0,051308	0,06356	0,339	0,296	1
Gna12	0,051313	0,054104	0,997	0,998	1
Stmn1	0,051338	-0,04139	0,998	1	1
Creld1	0,051343	-0,04149	0,293	0,34	1
Phb2	0,051359	-0,06018	0,561	0,593	1
Rpl38	0,051412	0,045086	0,997	0,997	1
Mavs	0,051496	-0,02408	0,136	0,174	1
Tfip11	0,051533	0,052237	0,26	0,22	1
Brca1	0,051534	0,016744	0,021	0,008	1
2310011J0	0,051551	-0,0502	0,551	0,593	1
Abtb2	0,051635	-0,03573	0,207	0,247	1
Atg101	0,051636	-0,0222	0,291	0,346	1
Fam53b	0,051868	0,080511	0,861	0,844	1
Phb	0,05194	0,039378	0,602	0,553	1
Nmb	0,05195	-0,0159	0,036	0,057	1
Strn	0,051996	-0,0465	0,702	0,746	1
Ing3	0,052021	-0,0351	0,255	0,301	1
Peli3	0,052038	0,015994	0,034	0,018	1
Vamp8	0,052044	0,069718	0,341	0,298	1
Eif5a	0,052085	0,048618	0,953	0,94	1
Plxna1	0,052094	0,042341	0,152	0,119	1
Tnrc6b	0,052342	-0,05018	0,843	0,852	1
Traf6	0,052366	-0,04035	0,26	0,304	1
S100a11	0,052633	0,02303	0,027	0,013	1
Epdr1	0,052757	-0,03449	0,423	0,483	1
Rsl24d1	0,052777	-0,04917	0,474	0,528	1

Tab3	0,05278	-0,02286	0,065	0,091	1
Gemin6	0,052788	-0,03277	0,073	0,099	1
Prmt3	0,052996	-0,05615	0,257	0,299	1
Hsd17b4	0,053019	-0,04359	0,722	0,78	1
Herpud2	0,053158	-0,03387	0,519	0,571	1
Gpr37l1	0,053226	-0,01443	0,044	0,067	1
Metap1	0,053237	-0,03817	0,322	0,369	1
Cenpj	0,05326	-0,03495	0,066	0,091	1
Ssrp1	0,053322	0,056935	0,485	0,446	1
Zfp9	0,053387	-0,03289	0,18	0,22	1
Aktip	0,0534	0,054661	0,749	0,735	1
Proser3	0,053408	-0,02439	0,057	0,081	1
Nemp1	0,053455	0,021781	0,053	0,033	1
Nat9	0,053534	-0,03401	0,354	0,407	1
1110059G	0,053555	-0,04144	0,313	0,359	1
Zfp433	0,053655	-0,00755	0,012	0,024	1
Nme1	0,053716	-0,04646	0,663	0,72	1
Sgpl1	0,053748	0,052402	0,302	0,263	1
Hadha	0,053765	-0,05071	0,785	0,818	1
Trmt10a	0,053949	0,048991	0,226	0,187	1
Eef2kmt	0,053958	-0,03594	0,157	0,193	1
Tpra1	0,05397	0,031022	0,142	0,109	1
Samd1	0,054061	0,051554	0,45	0,398	1
Gm26917	0,054096	0,104908	0,496	0,478	1
Fn3k	0,054156	-0,06826	0,441	0,481	1
Rtf2	0,054196	-0,04371	0,628	0,659	1
Pde4dip	0,054197	-0,04109	0,667	0,72	1
Tmem202	0,054265	0,022296	0,038	0,021	1
Rufy3	0,054278	-0,04022	0,931	0,961	1
Mrpl28	0,05432	-0,04496	0,526	0,572	1
Il12a	0,054403	-0,02581	0,046	0,068	1
Dlg1	0,054404	-0,05351	0,826	0,854	1
Skp1a	0,054467	-0,03515	0,843	0,855	1
En2	0,054526	-0,02116	0,223	0,27	1
Cops3	0,054546	-0,04227	0,55	0,59	1
2610021A(	0,054592	-0,03512	0,159	0,197	1
A330049N(	0,054617	0,06797	0,297	0,263	1
Tulp4	0,054624	0,053372	0,999	1	1
Tomm70a	0,054651	-0,04046	0,43	0,486	1
Crybg3	0,054854	-0,10526	0,449	0,483	1
Arf5	0,05493	0,05775	0,9	0,889	1
Cdc42	0,054987	0,049338	0,992	0,99	1
Fbxw17	0,055294	-0,03609	0,278	0,328	1
Adcy5	0,055317	0,062251	0,605	0,566	1
Mdga2	0,055363	-0,05311	0,533	0,563	1
Tomm40	0,055439	-0,0382	0,448	0,496	1
Msantd4	0,055508	0,048108	0,344	0,301	1
Tbc1d13	0,055598	-0,03242	0,189	0,229	1
Stk10	0,055621	-0,02887	0,089	0,119	1
Mcoln1	0,055624	0,06251	0,336	0,298	1
Dcaf8	0,055747	-0,03495	0,711	0,763	1
Hmg20a	0,055759	-0,03745	0,385	0,433	1

Extl3	0,055816	-0,05396	0,307	0,353	1
Kdr	0,055851	-0,02398	0,064	0,089	1
Minos1	0,055858	-0,03399	0,832	0,867	1
Rps10	0,055922	0,038437	0,997	0,998	1
Mapre2	0,055968	-0,06763	0,898	0,902	1
Cep126	0,056015	-0,01722	0,039	0,06	1
Anxa9	0,056017	-0,01398	0,011	0,023	1
Ddah1	0,056017	-0,01398	0,011	0,023	1
Tpst1	0,056135	-0,0361	0,644	0,685	1
Ano6	0,056152	0,022163	0,023	0,01	1
Ocel1	0,056208	0,037711	0,127	0,096	1
Arhgef2	0,056216	0,062393	0,934	0,915	1
Sec11a	0,056242	-0,03599	0,368	0,421	1
Itgb1	0,056272	0,076153	0,756	0,738	1
Slc35b1	0,056357	-0,03836	0,571	0,626	1
Scarb1	0,056373	-0,03944	0,18	0,218	1
Golga5	0,05639	-0,04132	0,26	0,302	1
Rbm38	0,056517	0,02501	0,023	0,01	1
Ddah2	0,056522	0,078779	0,395	0,364	1
Tnfsf12	0,056579	0,027621	0,09	0,063	1
Gm26546	0,0567	0,017398	0,023	0,01	1
4933430I1	0,056735	0,005871	0,006	0	1
Tnc	0,056735	0,005871	0,006	0	1
Tes	0,056735	0,005871	0,006	0	1
Gm3417	0,056735	0,005871	0,006	0	1
Wdr93	0,056735	0,006846	0,006	0	1
Uaca	0,056735	0,006846	0,006	0	1
B430319H:	0,056735	0,006846	0,006	0	1
Tnnc1	0,056735	0,009766	0,006	0	1
Lck	0,056735	0,008793	0,006	0	1
Selplg	0,056735	0,008793	0,006	0	1
Rnd1	0,056735	0,008793	0,006	0	1
Nfatc1	0,056735	0,008793	0,006	0	1
Rnf150	0,056735	0,009766	0,006	0	1
Gm42756	0,056744	-0,07376	0,366	0,41	1
Snapc2	0,056759	-0,03414	0,186	0,226	1
Pola2	0,056905	0,0401	0,119	0,089	1
Tecpr1	0,057209	-0,04437	0,388	0,436	1
Gtpbp8	0,057232	-0,04292	0,15	0,185	1
Ndufb11	0,057267	0,055671	0,949	0,94	1
Eif4g2	0,057274	-0,02898	0,995	0,998	1
Smim3	0,057413	-0,04148	0,069	0,094	1
Pa2g4	0,057415	-0,04571	0,688	0,743	1
Igf2bp2	0,057416	0,023318	0,023	0,01	1
Pdcd5	0,057521	-0,03517	0,793	0,829	1
Arhgef40	0,057522	0,040809	0,108	0,08	1
Gm49359	0,057553	-0,0546	0,104	0,135	1
Rtn2	0,057638	-0,0314	0,045	0,067	1
Zfp467	0,05768	0,046704	0,191	0,154	1
Sf3b2	0,057864	0,050482	0,962	0,963	1
Praf2	0,057908	0,046509	0,498	0,454	1
Dusp14	0,057915	0,017098	0,018	0,007	1

Wdr81	0,057962	-0,03353	0,201	0,242	1
4932438A1	0,057979	-0,05696	0,684	0,725	1
Catsper2	0,05798	-0,04815	0,124	0,154	1
Map1lc3a	0,057985	0,06936	0,92	0,919	1
Rexo5	0,058005	-0,0185	0,03	0,049	1
Cnih1	0,058041	-0,04584	0,694	0,754	1
Cttnbp2nl	0,058055	-0,04726	0,5	0,548	1
Slit2	0,058074	0,017289	0,029	0,015	1
Cmc4	0,058076	-0,03991	0,296	0,341	1
Cdk15	0,058094	0,013877	0,018	0,007	1
Ifit3b	0,058129	-0,05504	0,01	0,021	1
Tnfrsf19	0,058131	-0,01526	0,017	0,031	1
Pex26	0,05828	0,022121	0,067	0,044	1
Mapt	0,058304	0,035598	0,999	1	1
Mbtps1	0,05848	-0,0198	0,475	0,54	1
Fry	0,05851	-0,04887	0,183	0,221	1
Psmc2	0,058527	0,057831	0,841	0,831	1
Dennd4a	0,058548	-0,05319	0,52	0,564	1
Tmem200c	0,058599	0,012611	0,018	0,007	1
Crnkl1	0,058603	-0,04971	0,269	0,311	1
Tcaf1	0,058657	-0,04029	0,611	0,649	1
Grik5	0,058725	0,040146	0,29	0,247	1
Srpk2	0,058844	-0,04364	0,7	0,756	1
Ppp2r3a	0,058872	-0,05161	0,92	0,937	1
Synrg	0,058911	-0,05804	0,657	0,709	1
Ggh	0,058982	0,056397	0,537	0,501	1
Dnajb6	0,059151	-0,04463	0,857	0,88	1
Fign	0,059183	-0,04461	0,346	0,397	1
Acot13	0,059291	-0,03007	0,471	0,532	1
Tecpr2	0,059316	-0,03735	0,396	0,455	1
Apbb2	0,059568	-0,07036	0,889	0,889	1
Smim1	0,059685	-0,0689	0,559	0,582	1
Gpm6a	0,059688	-0,01239	0,01	0,021	1
Lsm1	0,059716	-0,03985	0,322	0,371	1
Mtdh	0,059734	-0,04968	0,963	0,974	1
Samd8	0,059783	-0,04814	0,763	0,807	1
Mat2a	0,059819	0,076381	0,974	0,971	1
Hist2h2aa1	0,059838	0,032108	0,076	0,052	1
Wdfy3	0,059873	-0,04651	0,676	0,724	1
Spg11	0,059918	-0,03126	0,217	0,262	1
Hmgcll1	0,059934	0,010448	0,055	0,034	1
Kantr	0,059942	-0,0334	0,126	0,159	1
Utp18	0,059961	-0,02344	0,235	0,28	1
Ctnnal1	0,059988	-0,04233	0,332	0,38	1
Dgkh	0,060025	0,022008	0,031	0,016	1
Mrpl46	0,060124	-0,04511	0,25	0,291	1
Prkd2	0,060152	0,023437	0,044	0,026	1
Gm19705	0,060249	0,032946	0,129	0,098	1
Zdhhc13	0,060258	-0,03388	0,093	0,122	1
Acp2	0,060307	-0,03577	0,302	0,35	1
Igbp1	0,060383	-0,03629	0,418	0,475	1
Trabd	0,060414	-0,03708	0,301	0,345	1

Lmnb1	0,060513	0,050667	0,129	0,099	1
Ttc4	0,060552	-0,04328	0,373	0,416	1
Mrpl10	0,06068	-0,03061	0,373	0,436	1
Hoxa7	0,060736	-0,02908	0,029	0,047	1
Gm14410	0,060766	0,015742	0,031	0,016	1
Arf3	0,060778	-0,02839	0,422	0,475	1
Eif4g3	0,060837	-0,06333	0,596	0,633	1
Gm32122	0,060895	-0,21572	0,12	0,15	1
Khk	0,060898	-0,03151	0,122	0,154	1
Trim47	0,060947	0,036898	0,058	0,037	1
Dscr3	0,060968	-0,03314	0,243	0,286	1
Zfyve19	0,061052	-0,02943	0,235	0,28	1
Pigyl	0,061186	-0,03635	0,867	0,901	1
Hyal1	0,061201	-0,02472	0,123	0,156	1
Gtf2ird1	0,061206	-0,04449	0,128	0,159	1
Mid2	0,061233	0,042303	0,092	0,067	1
Bmyc	0,061283	-0,02763	0,674	0,746	1
Rps6ka5	0,061292	-0,06371	0,598	0,608	1
BC029722	0,061313	0,037493	0,43	0,38	1
Hs3st1	0,06135	0,081164	0,8	0,79	1
Txndc16	0,061441	-0,03444	0,882	0,881	1
Chd6	0,061674	-0,02915	0,714	0,767	1
Ctnnd2	0,061701	-0,01887	0,049	0,072	1
Rtel1	0,061702	-0,02093	0,063	0,088	1
Ss18l2	0,061742	-0,06058	0,761	0,785	1
Mroh7	0,061819	0,027337	0,048	0,029	1
Esrp1	0,061829	-0,01642	0,016	0,029	1
Srsf12	0,061849	-0,02217	0,033	0,052	1
Mcts2	0,061938	-0,03325	0,139	0,174	1
Rgs16	0,061953	-0,03978	0,075	0,101	1
Tmem41b	0,062073	0,099781	0,605	0,574	1
Lypd6	0,062206	0,051887	0,158	0,125	1
Snhg6	0,062299	0,041492	0,538	0,488	1
Pola1	0,062329	0,040169	0,153	0,12	1
Med1	0,062354	-0,05345	0,485	0,528	1
Supt5	0,062361	0,068728	0,522	0,475	1
Snhg9	0,062444	-0,02582	0,281	0,33	1
Suclg1	0,062478	-0,03404	0,651	0,707	1
Spryd3	0,062528	-0,03255	0,2	0,241	1
Pkd2l1	0,062533	-0,07665	0,145	0,179	1
Josd1	0,062558	-0,04204	0,164	0,2	1
Slc25a14	0,062571	-0,02127	0,185	0,228	1
Trmt11	0,062608	-0,02875	0,175	0,213	1
Armc5	0,062714	-0,01956	0,099	0,13	1
Gm31812	0,062727	0,014834	0,025	0,011	1
Gm17518	0,062727	0,014834	0,025	0,011	1
Rer1	0,062756	-0,02774	0,893	0,922	1
Mkks	0,062762	-0,03147	0,155	0,19	1
9330111N(	0,062809	0,041749	0,072	0,049	1
Sgsm1	0,062905	-0,01195	0,016	0,029	1
Rdh5	0,063033	0,028143	0,104	0,076	1
Cdc27	0,063136	-0,04129	0,257	0,299	1

Nup43	0,063148	-0,02753	0,081	0,109	1
Selenow	0,063438	0,037055	0,995	0,998	1
Gm10602	0,063442	-0,01053	0,009	0,02	1
Ubn2	0,063447	-0,04602	0,749	0,79	1
Pcca	0,063488	-0,04412	0,263	0,302	1
Dnmt3b	0,0635	-0,01353	0,016	0,029	1
Epha10	0,063545	-0,01335	0,026	0,042	1
Lsm14a	0,063571	0,058057	0,605	0,561	1
Spata5	0,063591	-0,02183	0,108	0,14	1
Fkbp1a	0,063591	0,051804	0,983	0,984	1
Ap2a1	0,063626	-0,05668	0,945	0,948	1
Zfp11	0,063685	-0,02003	0,052	0,075	1
Utp4	0,063736	0,040821	0,139	0,109	1
4632415L(	0,063794	-0,04563	0,172	0,207	1
Rbbp8	0,063918	-0,00956	0,009	0,02	1
Rad52	0,063965	-0,02087	0,105	0,137	1
Tmem237	0,064024	-0,03982	0,396	0,447	1
Sgce	0,064216	-0,02844	0,658	0,706	1
Rhoa	0,064223	0,029309	0,999	0,998	1
Carmil3	0,064249	-0,01427	0,112	0,145	1
Cwf19l1	0,064343	-0,03272	0,13	0,163	1
Cdc45	0,064354	-0,01248	0,021	0,036	1
Rbm25	0,06437	0,061215	0,956	0,946	1
Ttc9	0,064398	0,055235	0,207	0,172	1
Usp12	0,064412	-0,03927	0,189	0,226	1
Nxph1	0,064427	-0,00775	0,002	0,008	1
Nrn1	0,064427	-0,00775	0,002	0,008	1
Lst1	0,064427	-0,00775	0,002	0,008	1
Cnnm1	0,064427	-0,00775	0,002	0,008	1
Haus5	0,064446	-0,03001	0,13	0,163	1
Tnik	0,064449	-0,04635	0,226	0,265	1
Gm26890	0,064467	-0,03778	0,371	0,426	1
Zfp398	0,064517	-0,02389	0,199	0,241	1
Tm7sf3	0,064522	-0,0462	0,934	0,954	1
Cd83	0,064533	0,012644	0,015	0,005	1
D630003M	0,064533	0,014571	0,015	0,005	1
Sphkap	0,064565	-0,00614	0,002	0,008	1
Olfr1284	0,064565	-0,00614	0,002	0,008	1
Glod5	0,064565	-0,00614	0,002	0,008	1
Gpr143	0,064565	-0,00614	0,002	0,008	1
Mccc1os	0,064565	-0,00614	0,002	0,008	1
Lnx1	0,064565	-0,00614	0,002	0,008	1
Gm20635	0,064565	-0,00614	0,002	0,008	1
Slc7a10	0,064565	-0,00614	0,002	0,008	1
Cacna2d2	0,064565	-0,00614	0,002	0,008	1
Car10	0,064565	-0,00614	0,002	0,008	1
Gm48708	0,064565	-0,00614	0,002	0,008	1
4833422C:	0,064565	-0,00614	0,002	0,008	1
Gm48079	0,064565	-0,00614	0,002	0,008	1
Gm15941	0,064565	-0,00614	0,002	0,008	1
Scrt1	0,064565	-0,00614	0,002	0,008	1
Lca5l	0,064565	-0,00614	0,002	0,008	1

Aldh1a7	0,064565	-0,00614	0,002	0,008	1
Clta	0,064593	0,041718	0,962	0,958	1
Coprs	0,064625	-0,04582	0,194	0,231	1
Cr2	0,064662	0,016494	0,015	0,005	1
Fam98a	0,064702	-0,0388	0,483	0,537	1
Man2c1	0,064706	-0,04259	0,278	0,32	1
A430033K(	0,064724	-0,0332	0,109	0,14	1
A430105I1	0,064845	-0,04306	0,095	0,124	1
Sp3os	0,064872	-0,05683	0,254	0,293	1
Snx20	0,064912	-0,00516	0,002	0,008	1
Prox2os	0,064912	-0,00516	0,002	0,008	1
4632428C(	0,064912	-0,00516	0,002	0,008	1
Ift20	0,065112	-0,05176	0,698	0,738	1
Tcf12	0,065278	-0,05531	0,876	0,886	1
Mtmr14	0,065328	-0,02312	0,189	0,231	1
Nudt10	0,06533	-0,01991	0,055	0,078	1
Brap	0,065332	-0,04575	0,371	0,415	1
Dot1l	0,065485	0,083994	0,502	0,476	1
Timm21	0,065518	-0,02909	0,27	0,317	1
Pard6g	0,065553	0,027552	0,074	0,05	1
Slc9b2	0,065569	-0,06312	0,084	0,112	1
5033403F(	0,065576	0,00813	0,015	0,005	1
Hopx	0,065606	-0,12865	0,645	0,65	1
Hmgb2	0,065718	-0,03326	0,126	0,158	1
Pcdhgb4	0,065837	-0,02655	0,084	0,112	1
Aurkaip1	0,065866	-0,03571	0,71	0,738	1
Arhgap21	0,066008	-0,04153	0,57	0,616	1
Rdh10	0,066077	0,03034	0,078	0,054	1
Apex1	0,066083	-0,03634	0,122	0,153	1
Rhobtb3	0,066091	-0,04869	0,728	0,754	1
Zfp141	0,066131	-0,03477	0,092	0,12	1
Vrk3	0,066187	-0,03235	0,328	0,374	1
Efnb3	0,066232	-0,03116	0,997	1	1
Eif2b1	0,066235	-0,03715	0,28	0,324	1
Zfp945	0,066321	-0,03825	0,152	0,187	1
Srxn1	0,066353	0,060636	0,204	0,171	1
Tmx2	0,066439	-0,05821	0,699	0,722	1
Gabarapl1	0,066513	-0,04589	0,882	0,915	1
Sv2a	0,066524	-0,04311	0,995	0,992	1
Sncb	0,066548	-0,0115	0,008	0,018	1
Kpna6	0,066551	-0,04474	0,334	0,377	1
Tfeb	0,066578	0,057638	0,515	0,473	1
Rbp1	0,066581	0,036856	0,07	0,047	1
Inpp5k	0,066587	-0,03057	0,284	0,33	1
Dnm3os	0,066594	-0,02567	0,054	0,076	1
Zdhhc17	0,06671	-0,05087	0,731	0,754	1
Tfpi	0,066855	-0,01556	0,008	0,018	1
5430425K:	0,066893	-0,00991	0,008	0,018	1
Mnt	0,067101	0,053202	0,269	0,231	1
Phpt1	0,067148	0,053844	0,609	0,563	1
Cep135	0,067184	-0,03479	0,138	0,172	1
Ptprf	0,067283	-0,06271	0,54	0,571	1

2310033P(	0,067344	-0,03455	0,314	0,361	1
Wdr3	0,067347	-0,02789	0,16	0,197	1
Mcm9	0,06747	-0,02233	0,149	0,185	1
Cntrob	0,067476	-0,02445	0,047	0,068	1
Fgf11	0,067515	0,017343	0,026	0,013	1
Msrb1	0,067619	-0,02922	0,763	0,782	1
Prr18	0,067643	0,080351	0,869	0,847	1
Mrpl53	0,06767	0,015138	0,026	0,013	1
Park2	0,06768	-0,03561	0,163	0,198	1
Tmem189	0,067709	-0,04804	0,929	0,948	1
Lrrc4c	0,067913	-0,0354	0,242	0,281	1
Wasl	0,067963	-0,05517	0,617	0,655	1
Pak4	0,068	-0,03272	0,124	0,156	1
Narf	0,068033	-0,03376	0,338	0,384	1
Pid1	0,068076	-0,00568	0,008	0,018	1
Lims1	0,068093	0,06888	0,627	0,6	1
Acy3	0,068175	-0,03672	0,196	0,237	1
Dis3l2	0,0682	-0,05498	0,376	0,421	1
Serpinh1	0,068358	-0,02014	0,044	0,065	1
Fzd3	0,068358	-0,0405	0,152	0,185	1
Tgif2	0,068495	-0,0305	0,089	0,117	1
Lrba	0,068576	-0,02345	0,195	0,236	1
Agpat3	0,068653	-0,04086	0,883	0,902	1
Asic4	0,068751	-0,00325	0	0,003	1
Rgs4	0,068751	-0,00325	0	0,003	1
Nectin4	0,068751	-0,00325	0	0,003	1
Kcnt1	0,068751	-0,00325	0	0,003	1
Zfp804a	0,068751	-0,00325	0	0,003	1
Il2rg	0,068751	-0,00325	0	0,003	1
Gm6377	0,068751	-0,00325	0	0,003	1
Gpr149	0,068751	-0,00325	0	0,003	1
Fstl5	0,068751	-0,00325	0	0,003	1
Adra2c	0,068751	-0,00325	0	0,003	1
Gm3519	0,068751	-0,00325	0	0,003	1
Mlxipl	0,068751	-0,00325	0	0,003	1
Col26a1	0,068751	-0,00325	0	0,003	1
Iqub	0,068751	-0,00325	0	0,003	1
Smo	0,068751	-0,00325	0	0,003	1
Grip2	0,068751	-0,00325	0	0,003	1
Cntn6	0,068751	-0,00325	0	0,003	1
Mrgprf	0,068751	-0,00325	0	0,003	1
Susd2	0,068751	-0,00325	0	0,003	1
Col18a1	0,068751	-0,00325	0	0,003	1
A930009A:	0,068751	-0,00325	0	0,003	1
Gm3636	0,068751	-0,00325	0	0,003	1
Rem2	0,068751	-0,00325	0	0,003	1
9230110C:	0,068751	-0,00325	0	0,003	1
Bmper	0,068751	-0,00325	0	0,003	1
Elmod1	0,068751	-0,00325	0	0,003	1
Gm46123	0,068751	-0,00325	0	0,003	1
Celsr3	0,068751	-0,00325	0	0,003	1
Cacna1g	0,068751	-0,00325	0	0,003	1

Krt222	0,068751	-0,00325	0	0,003	1
Ryr2	0,068751	-0,00325	0	0,003	1
Gm33195	0,068751	-0,00325	0	0,003	1
Fkbp1b	0,068751	-0,00325	0	0,003	1
Sostdc1	0,068751	-0,00325	0	0,003	1
Tunar	0,068751	-0,00325	0	0,003	1
Dennd3	0,068751	-0,00325	0	0,003	1
Acvrl1	0,068751	-0,00325	0	0,003	1
Krt1	0,068751	-0,00325	0	0,003	1
Neurl1b	0,068751	-0,00325	0	0,003	1
Kcng2	0,068751	-0,00325	0	0,003	1
Sall3	0,068751	-0,00325	0	0,003	1
Neto1	0,068751	-0,00325	0	0,003	1
Fads3	0,068751	-0,00325	0	0,003	1
Lbx1	0,068751	-0,00325	0	0,003	1
Adra2a	0,068751	-0,00325	0	0,003	1
4930523C(	0,068751	-0,00487	0	0,003	1
Serping1	0,068751	-0,00487	0	0,003	1
Atp6v0d2	0,068751	-0,00487	0	0,003	1
Stra6l	0,068751	-0,00487	0	0,003	1
Ccdc180	0,068751	-0,00487	0	0,003	1
Hpcal4	0,068751	-0,00487	0	0,003	1
Kcnk3	0,068751	-0,00487	0	0,003	1
Alox5ap	0,068751	-0,00487	0	0,003	1
Fbxo16	0,068751	-0,00487	0	0,003	1
C2cd4b	0,068751	-0,00487	0	0,003	1
Tekt5	0,068751	-0,00487	0	0,003	1
Plcl2	0,068751	-0,00487	0	0,003	1
Adcyap1	0,068751	-0,00487	0	0,003	1
Rasa4	0,068751	-0,00648	0	0,003	1
Hoxa6	0,068751	-0,00648	0	0,003	1
Rassf10	0,068751	-0,00971	0	0,003	1
Trim30a	0,068751	-0,01132	0	0,003	1
Mrrf	0,06877	-0,04035	0,172	0,207	1
Sf3b6	0,068816	-0,0391	0,745	0,784	1
Gmip	0,068851	0,020346	0,037	0,021	1
Slc8a1	0,068872	0,025504	0,142	0,111	1
Amz2	0,069139	-0,04107	0,348	0,393	1
Fbxl15	0,069207	-0,03634	0,115	0,145	1
Mfsd6	0,069259	-0,04954	0,741	0,777	1
Far2os1	0,069293	-0,0175	0,007	0,016	1
Atg5	0,069323	-0,03539	0,268	0,311	1
Snx21	0,069342	-0,0439	0,234	0,272	1
Cpeb1	0,069448	-0,04006	0,615	0,652	1
Pde4d	0,069652	0,031402	0,17	0,135	1
Xpo5	0,069682	-0,02058	0,078	0,104	1
Galnt3	0,069717	-0,01088	0,007	0,016	1
Ccdc85a	0,069717	-0,01248	0,007	0,016	1
Pttg1	0,06973	-0,06426	0,316	0,356	1
Rrp1	0,069754	0,060306	0,854	0,841	1
Gm26699	0,06981	-0,03842	0,194	0,229	1
Riox1	0,069897	0,014232	0,093	0,067	1

Cd14	0,069924	0,012333	0,012	0,003	1
Tbl3	0,069966	-0,03022	0,205	0,244	1
Cenpp	0,06998	-0,02272	0,043	0,063	1
Abca5	0,069985	0,081715	0,398	0,363	1
Dph5	0,070005	-0,03042	0,243	0,283	1
Gm16033	0,070028	0,010399	0,012	0,003	1
Gm26694	0,070048	-0,00928	0,007	0,016	1
Vmn2r97	0,070133	0,010399	0,012	0,003	1
4930426L(	0,070133	0,00943	0,012	0,003	1
Olfr93	0,070133	0,00943	0,012	0,003	1
Lpin1	0,070183	0,100213	0,452	0,418	1
Gm26785	0,070193	-0,00991	0,007	0,016	1
Uqcrq	0,070229	-0,04307	0,963	0,967	1
Lrrd1	0,070237	0,008461	0,012	0,003	1
A230083N:	0,070334	0,010083	0,009	0,002	1
Gm15582	0,070334	0,012988	0,009	0,002	1
Zfp612	0,070374	-0,04216	0,247	0,289	1
Gabrd	0,070396	0,010083	0,009	0,002	1
Eef1akmt1	0,070436	-0,02784	0,141	0,176	1
Bicc1	0,070455	-0,06575	0,299	0,333	1
Gm21994	0,070457	0,008141	0,009	0,002	1
Slc28a3	0,070519	0,007169	0,009	0,002	1
Olfr1423	0,070519	0,007169	0,009	0,002	1
Manea	0,070637	-0,03767	0,302	0,346	1
Irgm1	0,070673	-0,11003	0,107	0,135	1
Xrra1	0,070765	0,007811	0,012	0,003	1
Rps6kl1	0,070805	-0,02851	0,114	0,145	1
Casq1	0,070809	-0,02726	0,111	0,141	1
Galnt17	0,070816	-0,02155	0,043	0,063	1
Hoxc4	0,070824	-0,01479	0,003	0,01	1
Atn1	0,070851	-0,04288	0,497	0,538	1
Armc8	0,070889	-0,03094	0,297	0,341	1
Rnd2	0,070901	0,094756	0,588	0,559	1
Atf4	0,070944	0,12841	0,745	0,751	1
Wdr54	0,070964	0,030742	0,122	0,093	1
Agk	0,07097	-0,03758	0,215	0,252	1
Etv3	0,07098	-0,04032	0,19	0,226	1
Htra2	0,071087	-0,01579	0,167	0,207	1
Srbd1	0,071101	-0,04467	0,236	0,273	1
Gm26782	0,071123	-0,03086	0,193	0,233	1
Srebf2	0,071157	0,084021	0,843	0,852	1
Alkbh8	0,071249	-0,03427	0,292	0,338	1
Mvp	0,071379	0,06424	0,507	0,462	1
Zfp296	0,071418	-0,00838	0,003	0,01	1
Bcl9	0,071456	-0,05229	0,434	0,467	1
Pcgf5	0,071469	-0,02405	0,148	0,184	1
Fam213b	0,071586	0,056415	0,374	0,332	1
Tacr1	0,071617	-0,00677	0,003	0,01	1
Gm48249	0,071617	-0,00677	0,003	0,01	1
4930524O(	0,071617	-0,00677	0,003	0,01	1
Baiap2l2	0,071617	-0,00677	0,003	0,01	1
Ppara	0,071617	-0,00677	0,003	0,01	1

Gm26734	0,071617	-0,00677	0,003	0,01	1
1700011I0	0,071617	-0,00677	0,003	0,01	1
Gm32913	0,071751	-0,00901	0,003	0,01	1
Sowahc	0,071758	0,04101	0,182	0,148	1
Dusp12	0,071992	0,050854	0,237	0,202	1
Pnkd	0,072014	-0,04168	0,837	0,847	1
Gjb6	0,072017	-0,00481	0,003	0,01	1
S1pr5	0,072178	-0,0436	0,867	0,876	1
Gdap1l1	0,07218	0,017824	0,039	0,023	1
Strip1	0,072204	0,052033	0,246	0,211	1
Gng10	0,072251	0,014487	0,028	0,015	1
Nap1l4	0,072412	0,044088	0,853	0,834	1
Xrcc5	0,072463	-0,03527	0,191	0,229	1
Capn15	0,072479	0,043774	0,302	0,26	1
Rbm41	0,072567	-0,02718	0,085	0,112	1
Chrne	0,072646	-0,0115	0,006	0,015	1
B3galt1	0,072696	-0,0409	0,194	0,231	1
Zfp120	0,072848	-0,03793	0,146	0,179	1
Akap10	0,0729	-0,04113	0,172	0,207	1
Lysmd2	0,072916	-0,02044	0,091	0,119	1
Hdx	0,072979	-0,03235	0,105	0,133	1
Kctd1	0,073018	-0,02016	0,027	0,044	1
Ppp6r1	0,073023	-0,02517	0,312	0,363	1
Bcl7c	0,073073	0,058797	0,501	0,478	1
Rpl11	0,073117	0,03804	0,992	0,995	1
Ppt1	0,073123	-0,03511	0,76	0,808	1
Wdr26	0,073295	-0,04957	0,645	0,67	1
Fam174a	0,073309	-0,03694	0,711	0,746	1
Qk	0,073329	-0,02594	0,999	1	1
Klhl35	0,073368	0,026042	0,053	0,034	1
Cacnb3	0,073398	-0,01933	0,027	0,044	1
Prim1	0,073412	0,049241	0,211	0,177	1
Kifc1	0,073419	-0,01283	0,005	0,013	1
Tti1	0,073435	-0,02192	0,115	0,146	1
Kctd8	0,073537	-0,017	0,004	0,011	1
Arpc1b	0,073556	0,065076	0,65	0,628	1
Jag1	0,073577	-0,01327	0,014	0,026	1
Pygo1	0,073589	-0,00671	0,006	0,015	1
Asb5	0,073701	-0,01123	0,005	0,013	1
Mapk8ip2	0,073712	0,025203	0,03	0,016	1
Eya2	0,073722	0,028812	0,077	0,054	1
Scd3	0,073764	-0,05099	0,262	0,301	1
4933406P(	0,073783	-0,01061	0,004	0,011	1
AC127341.	0,073792	0,021496	0,045	0,028	1
AU040972	0,07384	-0,01742	0,027	0,044	1
Mark3	0,073852	-0,02847	0,622	0,67	1
Arl5a	0,073907	0,053055	0,524	0,489	1
Pld6	0,073915	-0,02041	0,019	0,033	1
Smn1	0,07395	-0,03316	0,25	0,289	1
Abcb4	0,073985	-0,00963	0,005	0,013	1
Tarbp2	0,074001	-0,03544	0,18	0,215	1
Zfp451	0,074014	0,04798	0,39	0,343	1

Stk32c	0,074029	-0,00901	0,004	0,011	1
Rhbdl3	0,074029	-0,00901	0,004	0,011	1
Chsy3	0,074029	-0,01061	0,004	0,011	1
Ddx54	0,07408	-0,03858	0,373	0,418	1
Polr2c	0,074099	-0,05312	0,577	0,605	1
Ubash3b	0,074148	-0,0157	0,339	0,385	1
Gm13091	0,074269	-0,00803	0,005	0,013	1
Smpdl3a	0,074269	-0,00803	0,005	0,013	1
Mtus2	0,074271	-0,00928	0,005	0,013	1
Gm29609	0,074276	-0,0074	0,004	0,011	1
Fbxo24	0,074276	-0,0074	0,004	0,011	1
Plet1os	0,074276	-0,0074	0,004	0,011	1
Gm47112	0,074276	-0,0074	0,004	0,011	1
Eppk1	0,074276	-0,0074	0,004	0,011	1
AC133868.	0,074276	-0,0074	0,004	0,011	1
Tuba4a	0,074294	0,041353	0,102	0,076	1
Rfc5	0,074302	-0,02489	0,07	0,094	1
Mccc1	0,074319	-0,03276	0,317	0,363	1
Rbm12b2	0,074329	0,03465	0,132	0,102	1
Fgfr1	0,074333	-0,02456	0,152	0,187	1
Ogfrl1	0,074383	-0,03203	0,212	0,252	1
Serinc4	0,074441	-0,00866	0,005	0,013	1
Cyp4x1	0,074441	-0,00866	0,005	0,013	1
Atr	0,074467	-0,03119	0,256	0,298	1
Stx8	0,074513	-0,04485	0,423	0,467	1
Ifitm3	0,074526	-0,00705	0,004	0,011	1
Ndufa9	0,074547	-0,04771	0,543	0,59	1
Sorcs3	0,07455	-0,04014	0,105	0,133	1
Ndufaf3	0,074605	-0,03959	0,417	0,47	1
Mfsd14a	0,074638	-0,02707	0,801	0,844	1
Calb1	0,074651	-0,00866	0,004	0,011	1
Gm10638	0,074664	0,011962	0,022	0,01	1
Nox1	0,074727	-0,00705	0,005	0,013	1
Myl4	0,074727	-0,00705	0,005	0,013	1
Nmt1	0,074743	-0,03766	0,731	0,758	1
Lat	0,074775	-0,01026	0,004	0,011	1
Chst3	0,074822	-0,02167	0,13	0,163	1
Zhx2	0,074923	-0,0392	0,171	0,205	1
Ppih	0,075058	-0,02532	0,16	0,195	1
Atf2	0,075069	-0,0316	0,504	0,558	1
Slc24a3	0,075097	-0,01977	0,111	0,141	1
Tubgcp5	0,075128	-0,03841	0,26	0,299	1
Ctsb	0,075164	0,073028	0,995	0,997	1
Caly	0,075173	-0,01353	0,019	0,033	1
Rasa2	0,075223	0,068021	0,244	0,21	1
Map3k12	0,075246	0,054862	0,165	0,135	1
Brd3	0,075352	-0,03382	0,521	0,574	1
Zyg11b	0,075374	-0,03522	0,322	0,367	1
Chid1	0,075429	-0,03067	0,204	0,242	1
Srpk3	0,075483	-0,03113	0,272	0,319	1
Akap6	0,075641	-0,05084	0,447	0,486	1
Pum2	0,075772	-0,03214	0,773	0,8	1

Sh3glb1	0,075844	-0,03725	0,989	0,993	1
Slc11a2	0,075928	-0,02257	0,225	0,267	1
Tollip	0,075947	-0,04341	0,476	0,522	1
Plec	0,075981	0,029235	0,057	0,037	1
Paqr6	0,075991	0,088003	0,428	0,397	1
Slc29a1	0,076337	0,024545	0,032	0,018	1
Xaf1	0,076357	-0,10179	0,613	0,644	1
Pnpla6	0,076378	-0,04591	0,289	0,327	1
Utp3	0,076426	-0,03387	0,321	0,364	1
Platr25	0,076469	-0,03102	0,126	0,158	1
Ube2j2	0,076564	0,047146	0,447	0,4	1
Apool	0,076566	-0,02216	0,184	0,223	1
Eif2ak1	0,07672	-0,04888	0,334	0,369	1
Dnpep	0,07677	-0,02452	0,368	0,418	1
Efhd2	0,076816	-0,03203	0,154	0,189	1
Col4a3bp	0,076838	0,05863	0,405	0,366	1
Yipf1	0,076851	-0,04661	0,461	0,504	1
Sft2d2	0,076917	-0,04444	0,36	0,403	1
Uqcc2	0,076955	-0,03075	0,823	0,883	1
Ccz1	0,077056	0,062191	0,532	0,476	1
Lin7c	0,077092	-0,01491	0,427	0,488	1
Cstf2	0,077126	-0,02795	0,297	0,343	1
Htatip2	0,077409	0,021067	0,053	0,034	1
Pigv	0,077411	-0,03881	0,149	0,18	1
Cgn	0,077457	0,015486	0,017	0,007	1
AC135964.	0,077501	0,009413	0,017	0,007	1
Asb1	0,077508	-0,05329	0,243	0,278	1
Cela1	0,077547	0,094427	0,459	0,428	1
Zfp286	0,077636	0,018407	0,071	0,049	1
Mapk10	0,07777	-0,04837	0,175	0,208	1
Nfs1	0,077791	-0,0279	0,236	0,278	1
Hypk	0,077914	0,059041	0,58	0,561	1
Ndufc2	0,078022	-0,04619	0,904	0,943	1
Srp14	0,078023	0,053784	0,971	0,956	1
Acbd6	0,078111	0,044423	0,558	0,517	1
Pagr1a	0,078113	0,019664	0,069	0,047	1
Asxl2	0,078115	-0,04141	0,538	0,58	1
Sphk2	0,078234	-0,03453	0,305	0,346	1
Adamtsl4	0,078325	-0,05257	0,677	0,712	1
Ccdc167	0,078343	-0,02357	0,196	0,237	1
Sh3rf1	0,078343	-0,02081	0,073	0,098	1
Zmym4	0,078385	0,053018	0,421	0,379	1
Fktn	0,078386	-0,05724	0,741	0,748	1
4930432K2	0,078405	0,020123	0,032	0,018	1
Snx27	0,078406	-0,04077	0,656	0,698	1
Osbpl9	0,078459	-0,04308	0,181	0,216	1
Csnk1g1	0,078485	-0,01576	0,234	0,28	1
Frs3	0,078497	-0,02361	0,202	0,241	1
Mgat4a	0,078502	-0,0117	0,027	0,044	1
Ddrgk1	0,078506	0,061668	0,938	0,946	1
Zswim4	0,078541	-0,01968	0,042	0,062	1
Senp3	0,078588	-0,03826	0,289	0,33	1

Plekhg3	0,078592	-0,04299	0,714	0,728	1
Il18	0,078746	-0,05382	0,586	0,603	1
Zfp131	0,078749	0,039196	0,37	0,322	1
Nudt9	0,078753	0,058166	0,535	0,501	1
Tmem107	0,078755	-0,01816	0,042	0,062	1
Tln1	0,078801	0,058493	0,722	0,689	1
Atcay	0,07897	-0,02213	0,079	0,104	1
Tigd2	0,078992	-0,0419	0,263	0,306	1
Clns1a	0,079081	-0,04741	0,52	0,554	1
Rab8b	0,079089	-0,04816	0,278	0,314	1
Cxcr2	0,079112	0,018651	0,024	0,011	1
Slc5a5	0,079121	-0,01067	0,031	0,049	1
Pknox1	0,07913	-0,01478	0,304	0,354	1
Snap47	0,079234	-0,04523	0,498	0,535	1
Cbx4	0,079274	0,068752	0,675	0,642	1
2410080I0	0,079369	-0,01369	0,023	0,037	1
Peak1	0,079405	-0,02295	0,278	0,322	1
Trim41	0,079468	-0,0345	0,246	0,286	1
Rasal1	0,079488	-0,04839	0,19	0,224	1
Zfp566	0,079524	0,035679	0,096	0,072	1
Dph6	0,079604	-0,03938	0,156	0,19	1
Tmem59	0,079751	-0,02864	0,982	1	1
Atmin	0,079799	0,038032	0,105	0,08	1
Nars2	0,079894	-0,02957	0,147	0,18	1
Slc29a3	0,079895	-0,02126	0,439	0,494	1
Slc7a11	0,079933	0,014834	0,024	0,011	1
Vti1b	0,079958	-0,03608	0,61	0,655	1
Ino80b	0,080043	0,049905	0,545	0,509	1
Borcs5	0,080068	-0,05528	0,349	0,385	1
Thbs3	0,080156	0,033009	0,252	0,213	1
Lrrc49	0,080162	0,05396	0,273	0,236	1
Dnajc6	0,080162	-0,04191	0,409	0,454	1
Epha1	0,080191	0,013988	0,047	0,029	1
Slc24a2	0,080261	0,05552	0,995	0,997	1
Tab1	0,080296	-0,03656	0,268	0,307	1
AA467197	0,080332	0,015138	0,024	0,011	1
Rgcc	0,080483	0,01292	0,024	0,011	1
Ints3	0,080512	-0,03959	0,362	0,408	1
Wdfy2	0,080739	-0,02887	0,37	0,42	1
Hotairm1	0,08074	-0,04941	0,4	0,442	1
Eaf1	0,080874	-0,01915	0,184	0,223	1
Rpusd3	0,080936	-0,01979	0,066	0,089	1
Zbtb8os	0,080938	-0,0382	0,402	0,447	1
Klhl25	0,080946	0,028282	0,081	0,059	1
Ipo9	0,081069	-0,03396	0,293	0,335	1
Arl15	0,081378	-0,02051	0,034	0,052	1
Dnajc30	0,081483	-0,04209	0,346	0,387	1
Ptgr2	0,081487	-0,04298	0,422	0,465	1
Phka2	0,081522	-0,03363	0,154	0,187	1
Wdr61	0,081735	-0,03832	0,394	0,437	1
Kdm4b	0,081883	-0,04414	0,218	0,255	1
Tmem159	0,081903	0,060359	0,47	0,45	1

Acads	0,082031	-0,02312	0,145	0,179	1
Ltn1	0,082044	-0,03566	0,431	0,473	1
Gm36975	0,082086	0,004895	0,005	0	1
Kcnj9	0,082086	0,004895	0,005	0	1
A530013C2	0,082086	0,004895	0,005	0	1
Gm14820	0,082086	0,004895	0,005	0	1
Tm4sf4	0,082086	0,004895	0,005	0	1
Gm43064	0,082086	0,004895	0,005	0	1
Ntn5	0,082086	0,004895	0,005	0	1
Gm44686	0,082086	0,004895	0,005	0	1
1700120K0	0,082086	0,004895	0,005	0	1
Gm16235	0,082086	0,004895	0,005	0	1
Gm15350	0,082086	0,004895	0,005	0	1
Gm31805	0,082086	0,004895	0,005	0	1
Gm16573	0,082086	0,004895	0,005	0	1
Il17d	0,082086	0,004895	0,005	0	1
Gramd2	0,082086	0,004895	0,005	0	1
Ttk	0,082086	0,004895	0,005	0	1
Gm5922	0,082086	0,004895	0,005	0	1
Rasl10a	0,082086	0,004895	0,005	0	1
Rasl10b	0,082086	0,004895	0,005	0	1
Ccl3	0,082086	0,004895	0,005	0	1
Dok3	0,082086	0,004895	0,005	0	1
Gm34923	0,082086	0,004895	0,005	0	1
Gm47096	0,082086	0,004895	0,005	0	1
Gm6566	0,082086	0,004895	0,005	0	1
1700001L0	0,082086	0,004895	0,005	0	1
Gm20468	0,082086	0,004895	0,005	0	1
Hba-a2	0,082086	0,059065	0,005	0	1
Srrm4	0,082086	0,010737	0,005	0	1
Slitrk2	0,082086	0,006846	0,005	0	1
Fcgbp	0,082086	0,006846	0,005	0	1
Lrtm1	0,082086	0,006846	0,005	0	1
Gm26698	0,082086	0,006846	0,005	0	1
Nexn	0,082086	0,005871	0,005	0	1
1110017D1	0,082086	0,005871	0,005	0	1
Fam229a	0,082086	0,005871	0,005	0	1
Utf1	0,082086	0,005871	0,005	0	1
Gm15915	0,082086	0,005871	0,005	0	1
Sftpc	0,082086	0,005871	0,005	0	1
Olfm4	0,082086	0,005871	0,005	0	1
Hmmr	0,082086	0,005871	0,005	0	1
Gm12224	0,082086	0,005871	0,005	0	1
Rph3al	0,082086	0,005871	0,005	0	1
Gm17039	0,082086	0,005871	0,005	0	1
1810019N1	0,082086	0,006846	0,005	0	1
Ggt6	0,082086	0,006846	0,005	0	1
Tert	0,082086	0,006846	0,005	0	1
Fam171a2	0,082086	0,008793	0,005	0	1
Hist1h1d	0,082086	0,008793	0,005	0	1
Gm47316	0,082086	0,00782	0,005	0	1
Slc35d1	0,082141	0,032539	0,098	0,073	1

Rmdn3	0,082241	-0,03424	0,299	0,341	1
Dusp16	0,082341	-0,04646	0,433	0,472	1
Gpr173	0,082377	-0,01685	0,034	0,052	1
Egln1	0,082407	0,080735	0,562	0,548	1
Zfp595	0,08249	-0,02283	0,101	0,13	1
Rsl1	0,082602	0,017585	0,034	0,02	1
Sdr42e1	0,08271	0,025313	0,079	0,057	1
Impact	0,082721	0,071732	0,613	0,597	1
Gm42695	0,082751	-0,01516	0,026	0,042	1
Cnot1	0,08284	0,0696	0,53	0,496	1
Samd4b	0,082857	-0,05381	0,642	0,673	1
Atad1	0,082867	-0,04601	0,678	0,719	1
3110045C2	0,083033	0,026328	0,038	0,023	1
Gucy1b1	0,083039	-0,02698	0,075	0,099	1
Rabac1	0,083102	0,049068	0,987	0,995	1
Ctsl	0,083103	0,066503	0,709	0,683	1
Os9	0,083153	0,060979	0,747	0,745	1
Dab2ip	0,083216	0,054358	0,403	0,369	1
Acvr1b	0,083269	0,028971	0,094	0,07	1
Gse1	0,083372	0,071905	0,445	0,416	1
Tle3	0,083429	0,104615	0,49	0,459	1
Gm4221	0,083457	-0,03732	0,064	0,086	1
Aff4	0,083501	-0,03789	0,721	0,756	1
Noct	0,083551	-0,02467	0,048	0,068	1
Mrpl33	0,083591	-0,03462	0,699	0,727	1
Map3k13	0,083732	-0,02823	0,096	0,124	1
Dph3	0,083863	0,062404	0,521	0,496	1
Tdrkh	0,083878	-0,02319	0,267	0,311	1
Exoc6b	0,083882	-0,0555	0,756	0,78	1
Parm1	0,083949	-0,024	0,358	0,418	1
Pcdhb6	0,083958	-0,01921	0,034	0,052	1
Gm28322	0,08399	0,138063	0,154	0,125	1
Naglu	0,084167	-0,03349	0,18	0,213	1
Tagap1	0,084207	-0,02896	0,105	0,133	1
Pcif1	0,0843	-0,03782	0,534	0,571	1
Zfp871	0,08431	-0,03822	0,424	0,476	1
Tmed3	0,08434	-0,04485	0,59	0,626	1
Npm3	0,084368	-0,0399	0,241	0,281	1
Gm16174	0,084408	0,017996	0,026	0,013	1
Gm5547	0,084408	0,023689	0,026	0,013	1
Nipa2	0,084473	-0,03553	0,244	0,283	1
Emc3	0,084478	-0,03251	0,731	0,785	1
Ppcdc	0,084548	0,027283	0,235	0,197	1
Nmral1	0,084579	0,064934	0,893	0,868	1
Rapgef4	0,08459	-0,00968	0,018	0,031	1
Gm17203	0,084707	-0,00934	0,018	0,031	1
Gars	0,084818	-0,03199	0,46	0,506	1
Nrarp	0,084875	0,017235	0,036	0,021	1
Zfp275	0,084948	-0,03922	0,234	0,27	1
Prpf19	0,085005	-0,04379	0,741	0,776	1
Aldh1b1	0,085037	0,017044	0,026	0,013	1
Sec24a	0,085051	-0,0382	0,276	0,319	1

AC116487.	0,085073	-0,01418	0,045	0,065	1
Kctd18	0,085189	-0,0419	0,274	0,311	1
Cavin4	0,085312	-0,0211	0,093	0,12	1
Adam17	0,085485	-0,03837	0,377	0,426	1
Ddx10	0,085546	-0,03617	0,225	0,262	1
Gsk3b	0,085654	0,050376	0,91	0,881	1
Gm3550	0,08567	0,014183	0,026	0,013	1
Gm266	0,085732	-0,01142	0,012	0,023	1
Dbi	0,085839	0,026733	0,999	1	1
Pcdhb18	0,085908	-0,00132	0,097	0,127	1
Arrdc3	0,085915	-0,03233	0,94	0,943	1
Eif2d	0,08601	-0,02864	0,206	0,244	1
Tcf7l1	0,086117	0,01901	0,019	0,008	1
Zfp688	0,086238	-0,02842	0,214	0,252	1
Pkdcc	0,086354	0,013263	0,019	0,008	1
Myo7a	0,086364	0,014571	0,014	0,005	1
1700001O:	0,086372	-0,01045	0,012	0,023	1
Ndp	0,086441	-0,01799	0,144	0,179	1
Shtn1	0,086456	-0,0401	0,916	0,928	1
Trp53cor1	0,086479	-0,01335	0,022	0,036	1
Ercc6	0,086682	-0,03892	0,118	0,146	1
Ankrd10	0,086726	-0,04795	0,349	0,39	1
6330418K(	0,08676	-0,01803	0,037	0,055	1
C78859	0,086787	0,025301	0,042	0,026	1
BC049715	0,086835	-0,00687	0,012	0,023	1
Snrpc	0,086836	-0,03354	0,64	0,668	1
Ndufs1	0,086839	-0,03447	0,596	0,654	1
Mettl3	0,086885	-0,04113	0,245	0,281	1
Tmbim6	0,086899	-0,03274	0,999	1	1
Igdcc4	0,086933	0,016692	0,019	0,008	1
Lamtor4	0,086992	-0,03892	0,677	0,719	1
B3glct	0,087034	-0,01335	0,097	0,125	1
Gm16861	0,087113	-0,01404	0,022	0,036	1
Gm14167	0,087211	0,009747	0,014	0,005	1
Nub1	0,08723	0,03949	0,614	0,567	1
Rnf14	0,087238	-0,03851	0,567	0,595	1
Shmt1	0,087272	0,026318	0,056	0,037	1
Ndufa6	0,087324	-0,03	0,855	0,893	1
Cd109	0,08735	0,02021	0,036	0,021	1
Hdgfl2	0,087411	-0,03685	0,395	0,441	1
Dctn1	0,087447	0,052868	0,823	0,818	1
Trim46	0,087452	0,025673	0,066	0,046	1
Pomc	0,087473	-0,01803	0,033	0,05	1
Mpp7	0,087474	0,02303	0,027	0,015	1
Trip10	0,087612	0,011991	0,014	0,005	1
Foxk2	0,087679	-0,03567	0,292	0,33	1
Pstk	0,087748	-0,02811	0,258	0,299	1
Adgra3	0,087897	0,018762	0,04	0,024	1
Aatk	0,087983	0,044475	0,996	0,998	1
Zfp653	0,088086	0,034632	0,159	0,128	1
Elovl1	0,088091	-0,03765	0,858	0,873	1
Cd34	0,088095	0,025915	0,167	0,135	1

Aimp1	0,088098	-0,03304	0,736	0,8	1
Cdc42se2	0,088132	-0,04126	0,769	0,789	1
Zfp758	0,088137	-0,02552	0,111	0,14	1
Cnksr3	0,088189	0,026448	0,119	0,091	1
Rnf13	0,088247	-0,03609	0,998	1	1
Polr2f	0,088436	0,037277	0,968	0,964	1
Atp10b	0,088498	-0,03241	0,236	0,275	1
Fam19a2	0,08862	0,012886	0,019	0,008	1
Gm48551	0,088726	0,012484	0,038	0,023	1
Gpnmb	0,088782	0,020923	0,044	0,028	1
Ogdh	0,08887	-0,06317	0,658	0,668	1
Nmi	0,088878	-0,03822	0,12	0,148	1
Rps21	0,088924	0,038433	0,999	1	1
Gm34776	0,088951	0,021279	0,064	0,044	1
Wdr82	0,089005	-0,02505	0,277	0,32	1
Tubd1	0,089021	-0,02116	0,071	0,094	1
Zfp143	0,089036	0,052588	0,137	0,111	1
Ier5l	0,089137	0,048166	0,077	0,055	1
Cav1	0,089158	-0,04096	0,122	0,15	1
Akirin2	0,089302	-0,03663	0,669	0,719	1
Trappc1	0,089344	-0,02723	0,32	0,366	1
Pdzd11	0,08936	-0,03964	0,332	0,372	1
Cebpb	0,089435	0,079813	0,192	0,163	1
Styx	0,08945	-0,05308	0,217	0,247	1
Zcchc9	0,089545	-0,05622	0,365	0,407	1
Pacrg	0,089553	-0,01566	0,033	0,05	1
Esrra	0,089561	-0,02711	0,119	0,148	1
Parn	0,089599	-0,03763	0,343	0,385	1
Defb47	0,089775	-0,01706	0,026	0,041	1
Insig1	0,089859	0,116297	0,796	0,789	1
Fam122b	0,08987	-0,04147	0,166	0,197	1
Sec24c	0,089889	-0,02038	0,329	0,376	1
Xrcc3	0,090018	0,045007	0,378	0,335	1
Dmac2	0,090165	-0,03807	0,173	0,205	1
Ints8	0,090261	-0,02839	0,171	0,205	1
Ebp	0,090349	0,054318	0,619	0,593	1
Tmem186	0,090491	-0,02328	0,129	0,159	1
Smox	0,090491	-0,04398	0,364	0,41	1
Ago4	0,090548	-0,02024	0,074	0,098	1
Gnai1	0,090554	-0,05039	0,978	0,974	1
Ralgps1	0,090698	-0,07104	0,568	0,595	1
Zfp760	0,090798	-0,0356	0,18	0,213	1
Ehbp1	0,090804	-0,0332	0,18	0,215	1
Efnb1	0,090834	0,024867	0,074	0,052	1
Mfap3	0,090861	-0,03574	0,205	0,241	1
Ogfod2	0,090891	-0,03025	0,207	0,242	1
Map2k3	0,090913	-0,02743	0,143	0,176	1
Gng7	0,090928	-0,03751	0,649	0,696	1
Qrsl1	0,091018	-0,02601	0,07	0,093	1
Ube2g1	0,091061	-0,0386	0,629	0,685	1
3110021N	0,091161	-0,05287	0,338	0,377	1
Bcas2	0,09123	-0,04351	0,749	0,785	1

Lrif1	0,091248	0,086024	0,41	0,39	1
Arhgdig	0,091271	-0,01495	0,011	0,021	1
Zkscan2	0,091532	-0,01317	0,033	0,05	1
Akt3	0,091557	-0,03004	0,143	0,174	1
D830024N	0,09164	0,019857	0,056	0,037	1
Trp53i13	0,091677	0,049333	0,402	0,366	1
Gm26737	0,091711	-0,01874	0,017	0,029	1
Greb1	0,091819	-0,01336	0,011	0,021	1
Tmem50a	0,091844	-0,0369	0,955	0,959	1
Sept11	0,091855	0,046248	0,091	0,068	1
Lmo4	0,092002	-0,02088	0,372	0,423	1
Acrbp	0,092054	-0,02237	0,108	0,137	1
Ddx19b	0,092078	0,020182	0,096	0,072	1
Ntmt1	0,09217	-0,03495	0,29	0,33	1
Wdr77	0,092461	-0,02863	0,277	0,319	1
Rit1	0,092473	-0,03163	0,248	0,286	1
Pnkp	0,092478	-0,03239	0,304	0,343	1
Zbed3	0,092547	-0,01733	0,04	0,059	1
Mkrn3	0,092593	-0,02158	0,082	0,107	1
BC003965	0,092594	-0,04157	0,434	0,472	1
Rpa2	0,092623	0,037427	0,307	0,267	1
Wdr83os	0,092682	-0,04015	0,553	0,597	1
A830019P(	0,092712	-0,01075	0,026	0,041	1
Pcdhgb5	0,092724	-0,01408	0,044	0,063	1
Zfp119a	0,092739	-0,0158	0,04	0,059	1
Apip	0,092766	-0,03667	0,283	0,322	1
Ccdc134	0,092817	-0,02068	0,156	0,189	1
Tmem80	0,092823	-0,04717	0,54	0,585	1
Sumf1	0,092917	-0,02139	0,161	0,195	1
S100a6	0,092951	0,002778	0,512	0,566	1
Bckdha	0,093006	0,060422	0,553	0,517	1
Med25	0,093023	0,063066	0,384	0,353	1
Mob3a	0,093034	-0,03203	0,258	0,298	1
Il33	0,093181	0,054301	0,885	0,821	1
Nrip1	0,093353	-0,04964	0,399	0,447	1
Rnf112	0,093357	-0,04579	0,353	0,39	1
Gna13	0,093481	-0,02272	0,399	0,449	1
Zfp251	0,093528	-0,0401	0,179	0,211	1
Prps2	0,093535	-0,0295	0,363	0,407	1
Naf1	0,093548	-0,03741	0,32	0,361	1
Nup98	0,093614	0,072712	0,432	0,397	1
Nup214	0,093772	-0,03803	0,344	0,38	1
Anapc11	0,093801	-0,0239	0,909	0,94	1
Rabgap1l	0,093805	0,04436	0,545	0,502	1
Myh14	0,093815	-0,05075	0,508	0,556	1
Naprt	0,093855	0,019506	0,054	0,036	1
Megf6	0,093892	0,019307	0,021	0,01	1
Gmppa	0,093896	-0,02503	0,222	0,262	1
Mier1	0,093917	-0,04495	0,665	0,693	1
Prpf38b	0,09395	-0,05435	0,649	0,665	1
Snhg10	0,094018	-0,02165	0,05	0,07	1
Tyro3	0,094123	0,076671	0,892	0,891	1

Rpl10a	0,094231	0,057438	0,913	0,883	1
Cluh	0,094293	-0,02613	0,173	0,207	1
Pcdhgc4	0,094382	-0,01853	0,064	0,086	1
Deaf1	0,09439	-0,03137	0,287	0,327	1
Gpn3	0,094564	-0,04166	0,301	0,338	1
Tcf4	0,094632	0,045195	0,995	0,998	1
Zbtb7a	0,094642	-0,06524	0,821	0,836	1
Tm7sf2	0,09475	0,025851	0,099	0,075	1
Cpm	0,094759	-0,043	0,719	0,766	1
Dedd2	0,094897	0,059764	0,194	0,164	1
Gnl1	0,09499	-0,02218	0,238	0,278	1
Otulin	0,095018	-0,04768	0,314	0,348	1
Akt1s1	0,095164	-0,03187	0,519	0,569	1
Naxd	0,095248	-0,02789	0,68	0,73	1
Ckap5	0,095434	-0,03058	0,799	0,815	1
Stard13	0,095508	-0,02065	0,244	0,286	1
Erlin1	0,095538	-0,01922	0,06	0,081	1
Wdr11	0,095619	-0,04238	0,232	0,268	1
Cptp	0,095708	-0,02923	0,122	0,151	1
Ublcp1	0,095715	-0,02686	0,422	0,472	1
Tmod1	0,095724	0,09119	0,915	0,912	1
Gm10382	0,095731	0,01292	0,021	0,01	1
Pclaf	0,095732	-0,00995	0,021	0,034	1
Mrpl58	0,095765	-0,04987	0,615	0,647	1
BC034090	0,095776	0,056036	0,189	0,161	1
Bcas1os2	0,09578	0,011962	0,021	0,01	1
Gm15853	0,09581	-0,02083	0,06	0,081	1
Wbp11	0,095827	-0,02572	0,662	0,719	1
Rasl11a	0,095837	0,013299	0,011	0,003	1
Zfp954	0,095892	0,033074	0,133	0,106	1
Rgs14	0,095977	0,013299	0,011	0,003	1
Armc6	0,096031	-0,01567	0,144	0,177	1
Tet1	0,096034	-0,04857	0,342	0,377	1
Ech1	0,096041	-0,03229	0,81	0,826	1
Wdr92	0,096117	-0,02919	0,117	0,145	1
Gm26786	0,096117	0,010399	0,011	0,003	1
Zmym1	0,096268	-0,03916	0,208	0,242	1
Snhg20	0,096322	-0,03093	0,412	0,459	1
Chaf1b	0,096397	0,010399	0,011	0,003	1
A330084C:	0,096397	0,00943	0,011	0,003	1
Pick1.1	0,096397	0,008461	0,011	0,003	1
Tmem126k	0,096459	-0,03099	0,235	0,273	1
Mvb12b	0,096523	-0,05079	0,677	0,691	1
Rps3a1	0,096571	0,038083	0,997	1	1
Polr2k	0,096579	-0,04484	0,528	0,567	1
Cuta	0,096775	-0,03392	0,826	0,85	1
Sap30bp	0,09703	-0,04858	0,432	0,467	1
Car14	0,097227	-0,02728	0,91	0,924	1
Itgb3bp	0,097253	-0,03406	0,25	0,288	1
Lta4h	0,097279	-0,01829	0,441	0,493	1
Gm36445	0,097349	-0,00976	0,016	0,028	1
Dock3	0,097376	-0,02197	0,337	0,384	1

Noxo1	0,097428	0,016342	0,031	0,018	1
Lmnb2	0,097494	-0,02565	0,126	0,154	1
Elfn2	0,097494	-0,01257	0,016	0,028	1
Fgd1	0,097503	-0,01395	0,028	0,044	1
Mnat1	0,097554	-0,05117	0,302	0,337	1
Mrps7	0,09768	-0,0453	0,584	0,62	1
Borcs7	0,097757	-0,04977	0,368	0,407	1
Zfp568	0,097822	-0,02102	0,079	0,102	1
Emc8	0,09789	-0,04372	0,634	0,675	1
Epha4	0,097909	-0,0419	0,243	0,283	1
Trim23	0,09794	-0,04344	0,314	0,35	1
Fgd4	0,098274	0,060949	0,218	0,187	1
Ube2v2	0,098294	-0,03168	0,532	0,589	1
Mafg	0,098589	-0,01256	0,585	0,66	1
Slc41a1	0,098702	0,040109	0,166	0,137	1
Mfap3l	0,098767	0,039291	0,094	0,072	1
Vps4a	0,098837	-0,0389	0,587	0,623	1
Mettl13	0,098898	-0,02339	0,148	0,18	1
Fance	0,098914	0,031326	0,285	0,246	1
Ttc12	0,09894	-0,03547	0,114	0,141	1
Chmp6	0,098944	-0,02442	0,158	0,19	1
D330050G	0,098949	-0,01115	0,01	0,02	1
Synj2	0,09895	-0,03202	0,493	0,537	1
Pcsk2os1	0,098957	-0,00988	0,025	0,039	1
Nit2	0,099006	0,040672	0,14	0,114	1
Rapgef6	0,099027	-0,0149	0,394	0,444	1
Scamp1	0,099033	-0,04215	0,396	0,436	1
Poll	0,099076	-0,01634	0,095	0,122	1
Tmem135	0,099301	-0,03059	0,334	0,379	1
Ralyl	0,09931	-0,02085	0,046	0,065	1
Helq	0,09935	-0,03442	0,095	0,12	1
S100a8	0,099375	0,010083	0,008	0,002	1
Tfap2a	0,099375	0,011052	0,008	0,002	1
Ppp2r5e	0,099376	-0,04106	0,365	0,408	1
Tex261	0,099503	-0,04205	0,626	0,67	1
E2f1	0,099548	0,008141	0,008	0,002	1
Kcnk7	0,099548	0,008141	0,008	0,002	1
Nsl1	0,099548	0,011052	0,008	0,002	1
Gm5608	0,099548	0,009112	0,008	0,002	1
Ube2t	0,099617	-0,01018	0,01	0,02	1
Gm7628	0,099617	-0,01018	0,01	0,02	1
Hfe	0,099617	-0,01018	0,01	0,02	1
Echdc3	0,099634	0,007169	0,008	0,002	1
Gchfr	0,099634	0,007169	0,008	0,002	1
Gm28874	0,099634	0,007169	0,008	0,002	1
Gm44949	0,099634	0,007169	0,008	0,002	1
Gm28285	0,099634	0,007169	0,008	0,002	1
Eno1b	0,099634	0,007169	0,008	0,002	1
Wdr76	0,099635	-0,02373	0,118	0,146	1
Tspan17	0,09968	0,071214	0,518	0,493	1
Gm26833	0,099721	0,006195	0,008	0,002	1
Gm45351	0,099721	0,006195	0,008	0,002	1

Gm9837	0,099721	0,006195	0,008	0,002	1
Dnmt3aos	0,099721	0,006195	0,008	0,002	1
Kctd16	0,099721	0,006195	0,008	0,002	1
Prpf39	0,099726	0,050652	0,588	0,545	1
Tada2b	0,099732	-0,03368	0,198	0,234	1
Fbxl17	0,099758	-0,03605	0,214	0,247	1
Pcmt1	0,099875	-0,04324	0,725	0,766	1
Qsox1	0,099881	0,079395	0,449	0,431	1
AC034116.	0,099983	0,00943	0,008	0,002	1
Fads1	0,100059	-0,02815	0,229	0,268	1
Zfp516	0,100064	-0,01248	0,028	0,044	1
Gm26916	0,100079	0,027185	0,023	0,011	1
Gldn	0,100157	0,010713	0,008	0,002	1
Tnks1bp1	0,100176	0,014269	0,084	0,062	1
Gm46430	0,100239	-0,02186	0,097	0,124	1
Ctbs	0,100245	-0,02924	0,145	0,176	1
D2hgdh	0,100258	-0,01816	0,121	0,15	1
Ino80e	0,100365	-0,03205	0,318	0,364	1
Kpna3	0,100367	-0,03918	0,452	0,489	1
Gm26735	0,100374	0,013916	0,016	0,007	1
Gm13610	0,100418	0,004573	0,008	0,002	1
Gclm	0,100436	-0,04072	0,828	0,841	1
Zcwpw1	0,100496	-0,03583	0,267	0,304	1
Cacnb4	0,100583	-0,04581	0,658	0,709	1
Mtif2	0,10068	-0,01848	0,208	0,247	1
Anp32e	0,100819	0,051515	0,659	0,634	1
Ankrd16	0,100888	-0,0208	0,145	0,177	1
Fut10	0,10093	-0,03953	0,171	0,202	1
Hook1	0,100964	-0,00824	0,01	0,02	1
Adra1b	0,101067	-0,02383	0,093	0,119	1
4932438H:	0,101079	0,013916	0,016	0,007	1
Slc25a36	0,101126	-0,04374	0,464	0,507	1
Asb6	0,101176	0,039133	0,227	0,192	1
Dgka	0,101211	-0,02181	0,031	0,047	1
Klhdc1	0,101256	-0,02487	0,144	0,176	1
Rnf6	0,101334	0,040859	0,348	0,311	1
Zfp2	0,101381	-0,02364	0,207	0,246	1
Kbtbd7	0,10139	-0,02589	0,169	0,202	1
Zmpste24	0,1014	-0,01967	0,423	0,475	1
C1qc	0,101426	0,015789	0,023	0,011	1
Asxl1	0,101641	-0,02898	0,241	0,281	1
Lrrc29	0,101674	0,011651	0,016	0,007	1
Mrm2	0,10171	-0,02339	0,152	0,184	1
Trim36	0,101721	-0,04907	0,454	0,502	1
Nrbf2	0,101961	-0,02894	0,33	0,374	1
Rbm27	0,101978	0,050472	0,454	0,416	1
Slc12a6	0,10203	-0,02041	0,352	0,4	1
H13	0,102042	-0,03715	0,433	0,476	1
Ankrd13b	0,102051	0,056816	0,345	0,311	1
Abcf2	0,102079	-0,03779	0,263	0,298	1
Arntl	0,102161	-0,02146	0,084	0,109	1
Mfsd14b	0,102214	-0,0423	0,546	0,572	1

Pcdhb21	0,102221	-0,01374	0,039	0,057	1
Syngr3	0,102262	0,016938	0,035	0,021	1
Snrnp40	0,102334	-0,03206	0,337	0,376	1
Wsb2	0,102425	-0,0412	0,643	0,672	1
Ptbp1	0,102483	0,048653	0,26	0,228	1
Rbsn	0,102496	-0,03182	0,353	0,39	1
Sh3gl1	0,1025	-0,03495	0,354	0,395	1
Vcan	0,102504	0,007482	0,016	0,007	1
Mrpl43	0,102509	-0,03803	0,65	0,675	1
Tbck	0,102547	-0,03806	0,368	0,403	1
Flywch1	0,102588	-0,05961	0,595	0,607	1
Pcdhga7	0,102695	-0,02517	0,078	0,101	1
Ube2d2a	0,102712	0,050814	0,929	0,925	1
Lzts3	0,102718	-0,02101	0,145	0,177	1
Akap17b	0,102746	-0,0199	0,076	0,099	1
Gramd1b	0,102793	-0,057	0,457	0,494	1
Nmd3	0,102821	0,039145	0,197	0,166	1
Pex13	0,102898	-0,0259	0,42	0,475	1
Wdr34	0,102907	0,023372	0,137	0,109	1
Cstf1	0,102978	-0,01496	0,125	0,153	1
Zc3h12b	0,103012	-0,01674	0,042	0,06	1
Cse1l	0,103164	-0,02466	0,271	0,311	1
Cdpf1	0,103169	-0,02799	0,182	0,215	1
Cox14	0,103301	-0,03728	0,682	0,727	1
Nfix	0,103308	0,039616	0,997	1	1
Fads6	0,103579	-0,03744	0,664	0,702	1
Utp23	0,103585	-0,03284	0,175	0,207	1
Gm10701	0,103608	0,026885	0,039	0,024	1
Scap	0,103613	-0,03669	0,461	0,512	1
Usp28	0,103693	-0,01822	0,088	0,114	1
Il17rb	0,103739	-0,02945	0,095	0,12	1
Slc25a48	0,103795	-0,01388	0,015	0,026	1
Kif2a	0,10382	0,052348	0,491	0,452	1
Arhgap1	0,103876	-0,03086	0,206	0,241	1
Gadd45b	0,103943	0,179231	0,351	0,324	1
Gm47469	0,104126	-0,02051	0,038	0,055	1
Gm32296	0,104138	-0,01126	0,02	0,033	1
Hsd17b10	0,104146	-0,03822	0,595	0,636	1
Zfp110	0,104171	-0,02527	0,288	0,328	1
Chmp2a	0,104198	0,046712	0,96	0,961	1
Pigx	0,104291	-0,02222	0,165	0,198	1
Gm15893	0,104328	0,011932	0,023	0,011	1
Hist1h3d	0,104379	-0,01327	0,015	0,026	1
Jun	0,104391	0,319034	0,885	0,891	1
Alkbh3	0,104418	-0,03221	0,128	0,156	1
Ube2h	0,104513	-0,04219	0,705	0,748	1
Eif4b	0,104577	0,058919	0,72	0,715	1
Slc26a11	0,104605	-0,02501	0,168	0,2	1
Sox8	0,104728	0,050319	0,883	0,872	1
Asap2	0,104806	0,048279	0,416	0,376	1
Eogt	0,105	-0,00872	0,02	0,033	1
Gas2l1	0,105189	-0,03877	0,223	0,259	1

Camta1	0,105221	-0,04604	0,605	0,642	1
Mycn	0,105301	0,019408	0,039	0,024	1
Abcc4	0,105315	-0,01461	0,035	0,052	1
C2cd2l	0,105321	-0,02569	0,203	0,239	1
Pygb	0,1054	-0,0475	0,213	0,246	1
Gpr137c	0,105452	-0,04013	0,322	0,363	1
Nckap1	0,105479	-0,05408	0,817	0,828	1
Mrpl47	0,105527	-0,02747	0,138	0,167	1
Pold4	0,105551	-0,03451	0,27	0,311	1
Olfr718-ps1	0,105607	0,019699	0,039	0,024	1
Clip3	0,105632	0,049793	0,952	0,964	1
Ino80	0,105651	0,053002	0,348	0,315	1
Slc16a1	0,105657	-0,05025	0,736	0,782	1
1700025G0	0,105683	-0,04519	0,473	0,511	1
Stim2	0,105759	-0,0261	0,212	0,249	1
Mef2c	0,105796	-0,01779	0,027	0,042	1
L1cam	0,105843	0,032473	0,043	0,028	1
Srd5a3	0,105934	-0,04043	0,328	0,364	1
Bad	0,105974	-0,02638	0,237	0,275	1
Hmox2	0,106167	-0,03808	0,845	0,885	1
Tiparp	0,106188	0,110264	0,244	0,216	1
Amotl1	0,106264	0,039102	0,059	0,041	1
Gulp1	0,106486	0,042481	0,068	0,049	1
Ube2e3	0,106528	-0,02129	0,771	0,816	1
9330199G1	0,106601	-0,02608	0,077	0,099	1
Rufy2	0,106829	0,067398	0,516	0,491	1
Phax	0,106857	-0,04338	0,53	0,569	1
Tmem176a	0,106878	0,059103	0,111	0,086	1
Mrpl52	0,106884	-0,04527	0,637	0,683	1
Ppp2r2d	0,106994	0,067634	0,536	0,509	1
Olfr552	0,107003	-0,00893	0,009	0,018	1
4933427D1	0,107014	-0,02093	0,124	0,153	1
Rmi1	0,107069	-0,02953	0,131	0,159	1
Btbd6	0,107072	0,030946	0,154	0,125	1
Fam89b	0,107189	0,015138	0,025	0,013	1
Gm49179	0,107189	0,020846	0,025	0,013	1
Csnk2a2	0,107193	-0,03035	0,441	0,488	1
Mfsd13a	0,107237	-0,03303	0,194	0,228	1
Phactr1	0,107273	-0,04055	0,894	0,883	1
Zfp979	0,107325	-0,02545	0,101	0,127	1
Ndst2	0,107354	-0,02226	0,078	0,101	1
Pcf11	0,107369	0,067019	0,471	0,439	1
Magee2	0,107688	-0,0108	0,009	0,018	1
Jmy	0,107744	-0,04131	0,517	0,558	1
Lrrc61	0,107817	-0,01313	0,185	0,223	1
Fam53a	0,107819	-0,04597	0,286	0,32	1
Papss1	0,107848	-0,02579	0,942	0,967	1
Mfap1b	0,107854	-0,04479	0,527	0,569	1
2810402E2	0,107881	-0,0047	0,105	0,133	1
Ube2s	0,1079	-0,03746	0,772	0,802	1
Crkl	0,107905	-0,023	0,38	0,429	1
Daam2	0,107911	-0,03794	0,922	0,935	1

Zfp984	0,107962	0,013227	0,025	0,013	1
Anks6	0,10798	-0,02025	0,052	0,072	1
Pitpnb	0,108054	-0,03527	0,536	0,585	1
Zbtb26	0,108301	-0,03235	0,108	0,135	1
Taf4b	0,108363	0,015742	0,025	0,013	1
Art3	0,108424	-0,13467	0,158	0,184	1
Psma2	0,108433	-0,04587	0,885	0,901	1
Ccdc34	0,108494	-0,02835	0,479	0,527	1
Msra	0,108519	0,026585	0,231	0,193	1
Clcn6	0,108571	0,055811	0,539	0,507	1
Ttc41	0,108614	-0,00361	0,02	0,033	1
Nucb1	0,108617	0,042763	0,522	0,498	1
1810055G	0,10864	-0,0325	0,117	0,143	1
Pstpip2	0,108665	-0,03543	0,194	0,226	1
Nosip	0,108738	-0,03145	0,383	0,424	1
Gm26610	0,108739	0,011313	0,025	0,013	1
Slc2a12	0,108796	0,012579	0,025	0,013	1
Bach2	0,108827	-0,02237	0,183	0,218	1
Pea15a	0,108965	0,042009	0,982	0,982	1
Sco1	0,10916	-0,02098	0,107	0,133	1
Chchd2	0,10916	-0,02426	0,998	0,995	1
Taf6	0,109708	-0,04205	0,196	0,228	1
Gm4524	0,109783	-0,03892	0,114	0,14	1
Hid1	0,109859	-0,02869	0,405	0,459	1
P3h4	0,109974	-0,03087	0,662	0,706	1
Setdb1	0,110024	0,035867	0,298	0,26	1
Cdc25a	0,110071	0,021957	0,089	0,067	1
Api5	0,110183	-0,04295	0,51	0,554	1
Pik3r2	0,110291	0,048864	0,331	0,299	1
Atg13	0,110313	-0,01614	0,261	0,304	1
Fam57a	0,110375	-0,04726	0,432	0,462	1
Lsm5	0,110597	-0,03715	0,596	0,623	1
Hyal2	0,110601	0,029024	0,103	0,08	1
Zfp101	0,110655	-0,0676	0,372	0,4	1
Anapc16	0,110663	-0,04108	0,706	0,735	1
Rnf225	0,110666	0,018931	0,049	0,033	1
Tmem260	0,110713	-0,04422	0,365	0,403	1
Srgap2	0,110805	-0,03635	0,296	0,335	1
Gm28875	0,110826	-0,02863	0,21	0,244	1
Pcdhb22	0,111087	-0,01891	0,091	0,115	1
Rab33a	0,111136	-0,066	0,223	0,254	1
Fmn2	0,11115	-0,01052	0,038	0,055	1
Cpe	0,111309	0,064562	0,596	0,55	1
Gm43263	0,111417	-0,0184	0,048	0,067	1
Ring1	0,111503	0,060831	0,553	0,52	1
Dmtn	0,111546	0,013285	0,061	0,042	1
Mest	0,111615	-0,04855	0,149	0,177	1
Xpo1	0,111703	-0,02156	0,431	0,478	1
Mmgt1	0,11174	-0,03542	0,44	0,488	1
Adgrg6	0,111826	0,015486	0,018	0,008	1
Ptp4a1	0,111913	-0,03569	0,995	0,997	1
A930004D	0,111942	0,013263	0,018	0,008	1

Epop	0,111942	0,014223	0,018	0,008	1
Daxx	0,112012	0,028024	0,217	0,184	1
Rbm10	0,112067	-0,03455	0,342	0,385	1
Gm4419	0,112226	-0,02148	0,125	0,153	1
2310057M	0,112278	-0,01837	0,2	0,237	1
Srcin1	0,112353	0,030815	0,966	0,969	1
Myo9a	0,112627	-0,04707	0,606	0,616	1
Rhoc	0,112736	0,049846	0,447	0,408	1
Gmfb	0,112805	-0,02731	0,72	0,759	1
Ift88	0,11284	-0,02408	0,133	0,163	1
Elk1	0,112911	-0,02677	0,079	0,101	1
Ints1	0,113096	0,034391	0,421	0,38	1
Gm29502	0,113148	-0,0131	0,008	0,016	1
Coa4	0,113213	-0,02295	0,107	0,133	1
Tshb	0,113325	0,01069	0,018	0,008	1
Slc8a3	0,113425	-0,01955	0,149	0,18	1
Lsm12	0,11344	-0,04431	0,54	0,576	1
Ythdf2	0,113518	0,031189	0,401	0,364	1
Tnnt2	0,113608	-0,01265	0,014	0,024	1
Nudt13	0,113614	-0,02219	0,39	0,434	1
Rhbdd3	0,113633	-0,01744	0,141	0,172	1
Plekha3	0,113781	-0,03434	0,238	0,272	1
Arid4b	0,113817	-0,03184	0,895	0,896	1
Rps27	0,11391	0,046452	0,996	0,997	1
Slc27a4	0,113973	-0,05205	0,512	0,538	1
Zfp706	0,114037	0,033922	0,994	0,995	1
Pbld1	0,114113	0,013192	0,026	0,015	1
Gm9873	0,11424	-0,00991	0,008	0,016	1
Aqp11	0,114335	-0,0103	0,019	0,031	1
Dusp18	0,114363	-0,01248	0,052	0,072	1
Smc1b	0,114725	-0,00727	0,008	0,016	1
Gfm1	0,114813	-0,01549	0,175	0,208	1
Zdhhc18	0,114851	0,042468	0,24	0,21	1
Cbfb	0,114954	-0,03217	0,418	0,46	1
Rab10	0,114974	-0,02749	0,98	0,974	1
Tm4sf1	0,115124	-0,00948	0,014	0,024	1
Ambra1	0,115167	-0,01155	0,195	0,233	1
B9d2	0,115258	-0,01945	0,077	0,099	1
Eef1a2	0,115308	-0,01136	0,023	0,036	1
Mrpl23	0,115417	-0,02281	0,7	0,764	1
Flt3l	0,115621	-0,00796	0,008	0,016	1
Gm10036	0,115621	-0,00796	0,008	0,016	1
Polr2j	0,115653	-0,03523	0,662	0,686	1
Serp2	0,115682	0,041886	0,154	0,127	1
Plekha7	0,11569	-0,00699	0,008	0,016	1
Tmem138	0,115784	-0,01667	0,127	0,156	1
Qtrt1	0,115833	-0,02128	0,169	0,202	1
Zfp809	0,115902	-0,02308	0,149	0,18	1
Erf	0,116008	0,071489	0,373	0,345	1
Tut1	0,116091	-0,01055	0,055	0,075	1
Zfp26	0,116153	-0,02022	0,216	0,252	1
Ik	0,116159	-0,0253	0,789	0,82	1

Rp2	0,116163	-0,02789	0,163	0,193	1
Clybl	0,116263	-0,01056	0,023	0,036	1
Ccdc71l	0,116324	-0,03789	0,168	0,198	1
5430431A:	0,116324	0,022955	0,072	0,052	1
Tmem248	0,116394	0,041462	0,313	0,28	1
Tex2	0,116533	0,049143	0,694	0,659	1
Tmed1	0,116575	-0,03605	0,341	0,376	1
Hsd3b7	0,116608	0,013608	0,013	0,005	1
Gen1	0,116617	0,009691	0,026	0,015	1
Aig1	0,116649	-0,04045	0,933	0,927	1
Ccdc130	0,116661	-0,04472	0,146	0,174	1
Ifrd1	0,116715	0,043649	0,57	0,541	1
Vmn1r12	0,116829	0,011679	0,013	0,005	1
Parp16	0,116829	0,010713	0,013	0,005	1
Hnrnpm	0,116859	-0,03418	0,748	0,792	1
Tk2	0,116899	-0,01924	0,168	0,2	1
Atp5g3	0,117018	-0,02757	0,832	0,865	1
Ppme1	0,117033	-0,04357	0,433	0,47	1
Dync1li2	0,117076	0,039716	0,886	0,885	1
Dpysl2	0,117127	0,017263	0,999	1	1
Cldn14	0,117127	-0,02155	0,28	0,324	1
Dennd4b	0,117211	0,055401	0,245	0,215	1
Wdr6	0,117241	-0,00946	0,03	0,046	1
Imp3	0,117259	-0,0319	0,741	0,782	1
1700055D:	0,117272	0,007811	0,013	0,005	1
Scx	0,117276	0,011027	0,013	0,005	1
Spdya	0,117397	0,01983	0,03	0,018	1
Gtf2b	0,117604	-0,04091	0,491	0,533	1
H2-Ke6	0,117622	-0,02937	0,384	0,429	1
Gtf3c1	0,11764	-0,02257	0,465	0,52	1
Mtmr6	0,117663	-0,03708	0,577	0,605	1
Rpusd1	0,117706	-0,02265	0,097	0,122	1
Gm10306	0,117721	0,011027	0,013	0,005	1
Mettl4	0,117743	-0,01715	0,061	0,081	1
Zfp397	0,117785	-0,02707	0,437	0,472	1
Trappc11	0,117979	-0,00998	0,222	0,262	1
Spata33	0,118018	0,007162	0,013	0,005	1
Fntb	0,118075	-0,0928	0,371	0,405	1
Ncapd2	0,118113	-0,01764	0,074	0,096	1
Flvcr1	0,118165	-0,03219	0,116	0,141	1
Gm49077	0,118241	0,006194	0,013	0,005	1
Herpud1	0,118263	0,065377	0,471	0,437	1
Rwdd1	0,11828	-0,04603	0,667	0,678	1
Lrrcc1	0,118425	-0,03215	0,274	0,312	1
Actr1a	0,118459	-0,03522	0,83	0,847	1
Rpl34	0,118461	0,038337	0,993	0,993	1
Cdc42se1	0,11853	-0,04483	0,563	0,587	1
Nf1	0,118621	-0,02512	0,748	0,779	1
Emp3	0,118823	0,031322	0,072	0,052	1
Pigp	0,118858	0,055094	0,645	0,631	1
Phkg1	0,118972	0,028053	0,056	0,039	1
Cisd1	0,119026	-0,01388	0,881	0,907	1

Gga1	0,119102	0,047963	0,349	0,314	1
Ube2a	0,119144	-0,01945	0,342	0,384	1
Lcor	0,119152	-0,02778	0,246	0,283	1
Gpr89	0,119264	-0,02386	0,295	0,337	1
Zfp518a	0,119358	-0,03736	0,283	0,319	1
Elmod2	0,119398	-0,04536	0,272	0,306	1
Lmln	0,119436	0,029332	0,103	0,08	1
Oard1	0,119445	-0,0212	0,402	0,455	1
Ids	0,119485	-0,03523	0,799	0,823	1
Pabpc4	0,119505	0,037337	0,159	0,132	1
Mybl1	0,119662	-0,02496	0,076	0,098	1
Ctnna3	0,119958	-0,03786	0,387	0,42	1
Ikbke	0,120055	0,003918	0,004	0	1
Gm16701.1	0,120055	0,003918	0,004	0	1
Armc3	0,120055	0,003918	0,004	0	1
Olfr1122	0,120055	0,003918	0,004	0	1
Grem1	0,120055	0,003918	0,004	0	1
Xlr3a	0,120055	0,003918	0,004	0	1
Pik3cd	0,120055	0,003918	0,004	0	1
Gm49027	0,120055	0,003918	0,004	0	1
A430078I0	0,120055	0,003918	0,004	0	1
Olfr571	0,120055	0,003918	0,004	0	1
Itgal	0,120055	0,003918	0,004	0	1
D030034A1	0,120055	0,003918	0,004	0	1
Itga7	0,120055	0,003918	0,004	0	1
Isyna1	0,120055	0,003918	0,004	0	1
Ankle1	0,120055	0,003918	0,004	0	1
Olfr975	0,120055	0,003918	0,004	0	1
Fxyd2	0,120055	0,003918	0,004	0	1
Gatsl3	0,120055	0,003918	0,004	0	1
Vstm2a	0,120055	0,003918	0,004	0	1
Igfbp4	0,120055	0,003918	0,004	0	1
Cartpt	0,120055	0,003918	0,004	0	1
4931403G1	0,120055	0,003918	0,004	0	1
Gdnf	0,120055	0,003918	0,004	0	1
Nckap5los	0,120055	0,003918	0,004	0	1
Gm16537	0,120055	0,003918	0,004	0	1
AC110166.	0,120055	0,003918	0,004	0	1
Has1	0,120055	0,003918	0,004	0	1
Fads2	0,120055	0,003918	0,004	0	1
Ms4a4c	0,120055	0,003918	0,004	0	1
Gm16541	0,120055	0,003918	0,004	0	1
Timp1	0,120055	0,010737	0,004	0	1
Isg20	0,120055	0,009766	0,004	0	1
2410004P(	0,120055	0,008793	0,004	0	1
Gm7697	0,120055	0,006846	0,004	0	1
Fam209	0,120055	0,005871	0,004	0	1
Gm26762	0,120055	0,005871	0,004	0	1
Cfap45	0,120055	0,004895	0,004	0	1
Slc32a1	0,120055	0,004895	0,004	0	1
Lor	0,120055	0,004895	0,004	0	1
Cd52	0,120055	0,004895	0,004	0	1

A930003O1	0,120055	0,004895	0,004	0	1
Pon3	0,120055	0,004895	0,004	0	1
Tex15	0,120055	0,004895	0,004	0	1
Zglp1	0,120055	0,004895	0,004	0	1
Wfdc17	0,120055	0,004895	0,004	0	1
Gpx2	0,120055	0,004895	0,004	0	1
Clip4	0,120055	0,004895	0,004	0	1
Fam69c	0,120055	0,004895	0,004	0	1
Stab1	0,120055	0,005871	0,004	0	1
Adra1a	0,120055	0,006846	0,004	0	1
Scn7a	0,120055	0,009766	0,004	0	1
Smim4	0,120081	-0,02897	0,181	0,211	1
Gpr137	0,120105	-0,0393	0,316	0,353	1
Haus3	0,120167	-0,03391	0,238	0,275	1
Cyc1	0,120196	-0,0408	0,723	0,764	1
Lat2	0,120315	0,020123	0,032	0,02	1
Bloc1s6os	0,120408	0,017398	0,02	0,01	1
Mrs2	0,12044	-0,02188	0,275	0,315	1
Nipal3	0,120501	-0,03001	0,48	0,54	1
Emc7	0,120629	0,04061	0,944	0,928	1
Pla2g6	0,120812	-0,01447	0,135	0,166	1
Tmem250-p	0,120819	-0,0359	0,34	0,377	1
Nedd8	0,120831	0,041378	0,899	0,876	1
Slf1	0,121051	-0,02403	0,226	0,26	1
Egfl8	0,121123	0,02026	0,02	0,01	1
Pkd2	0,12114	-0,03905	0,356	0,39	1
Lonrf2	0,121153	-0,01885	0,029	0,044	1
Surf2	0,121177	-0,03764	0,295	0,33	1
Cntn3	0,121185	-0,03192	0,169	0,2	1
3300002I0	0,121251	-0,02088	0,026	0,039	1
Ap3m1	0,121251	-0,05648	0,527	0,561	1
Calcoco1	0,121272	-0,04245	0,378	0,416	1
Yif1b	0,12134	0,04765	0,611	0,576	1
Pgap2	0,121341	0,054177	0,574	0,553	1
Trip4	0,121365	-0,02881	0,398	0,441	1
4930455G(	0,121484	0,024739	0,034	0,021	1
Adk	0,121539	-0,00653	0,626	0,675	1
Gmnn	0,121573	-0,0173	0,073	0,094	1
Rgs17	0,121589	0,034831	0,04	0,026	1
Ehd4	0,121675	0,042178	0,162	0,135	1
Cachd1	0,121701	-0,04065	0,21	0,242	1
Ak5	0,121754	-0,01195	0,018	0,029	1
Gpr146	0,121823	-0,02975	0,144	0,172	1
Pisd	0,121882	-0,02324	0,345	0,39	1
Cox19	0,121981	-0,03761	0,383	0,42	1
5031425E2	0,121987	-0,03553	0,269	0,302	1
C1galt1c1	0,122077	-0,02564	0,343	0,384	1
Cdo1	0,122177	-0,00928	0,007	0,015	1
Tmem210	0,122201	0,01069	0,02	0,01	1
Zfp456	0,122201	0,01069	0,02	0,01	1
6230400D1	0,122247	0,022349	0,054	0,037	1
Zmynd8	0,122262	-0,039	0,752	0,772	1

Dpysl3	0,122332	-0,00991	0,007	0,015	1
Fndc3a	0,122441	-0,03481	0,3	0,338	1
BC037034	0,122465	0,023415	0,114	0,089	1
Apobec3	0,122485	-0,00712	0,001	0,005	1
Slc25a43	0,122485	-0,01034	0,001	0,005	1
Slc6a19	0,122485	-0,01034	0,001	0,005	1
Gm6525	0,122486	-0,00699	0,007	0,015	1
Kifc2	0,122496	0,036693	0,175	0,146	1
Nme7	0,122586	-0,01106	0,058	0,078	1
Tmem110	0,122625	0,036809	0,369	0,33	1
Dnah7a	0,122638	-0,0055	0,001	0,005	1
Ihh	0,122638	-0,0055	0,001	0,005	1
Gm38251	0,122638	-0,0055	0,001	0,005	1
Scn3a	0,122638	-0,0055	0,001	0,005	1
Pdyn	0,122638	-0,0055	0,001	0,005	1
Hs6st2	0,122638	-0,0055	0,001	0,005	1
Ppargc1a	0,122638	-0,0055	0,001	0,005	1
Pde3a	0,122638	-0,0055	0,001	0,005	1
Cyp2e1	0,122638	-0,0055	0,001	0,005	1
Fat3	0,122638	-0,0055	0,001	0,005	1
L3hypdh	0,122638	-0,0055	0,001	0,005	1
Gm30159	0,122638	-0,0055	0,001	0,005	1
Gm47123	0,122713	-0,00768	0,007	0,015	1
Slc1a1	0,122713	-0,00768	0,007	0,015	1
Rxrg	0,122791	-0,00389	0,001	0,005	1
Chrna4	0,122791	-0,00389	0,001	0,005	1
Was	0,122791	-0,00389	0,001	0,005	1
Igsf1	0,122791	-0,00389	0,001	0,005	1
Cd1d1	0,122791	-0,00389	0,001	0,005	1
Zfp981	0,122791	-0,00389	0,001	0,005	1
Disp3	0,122791	-0,00389	0,001	0,005	1
Gm17216	0,122791	-0,00389	0,001	0,005	1
Usp29	0,122791	-0,00389	0,001	0,005	1
Pcbd1	0,122791	-0,00389	0,001	0,005	1
Necab2	0,122791	-0,00389	0,001	0,005	1
Gm2237	0,122791	-0,00389	0,001	0,005	1
Zfp558	0,122791	-0,00389	0,001	0,005	1
Obscn	0,122791	-0,00389	0,001	0,005	1
Ptges3l	0,122791	-0,00389	0,001	0,005	1
1700001L1	0,122791	-0,00389	0,001	0,005	1
Rnf180	0,122791	-0,00389	0,001	0,005	1
Gm47997	0,122791	-0,00389	0,001	0,005	1
Dbpht2	0,122791	-0,00389	0,001	0,005	1
Tex33	0,122791	-0,00389	0,001	0,005	1
Espl1	0,122791	-0,00389	0,001	0,005	1
Gm15328	0,122791	-0,00389	0,001	0,005	1
Rab27b	0,122791	-0,00389	0,001	0,005	1
Gal	0,122791	-0,00389	0,001	0,005	1
Olfr1459	0,122791	-0,00389	0,001	0,005	1
Map3k1	0,122839	0,016641	0,032	0,02	1
Cmpk1	0,122979	-0,04913	0,525	0,556	1
Tmem57	0,123043	-0,02857	0,508	0,54	1

Cbln2	0,123098	-0,00452	0,001	0,005	1
Fuz	0,123145	-0,02623	0,202	0,234	1
Amacr	0,123182	0,024053	0,08	0,06	1
Diaph2	0,123232	-0,03998	0,231	0,267	1
Pax3	0,123252	0,003927	0,001	0,005	1
Ncaph	0,123252	-0,00291	0,001	0,005	1
Gabrg1	0,123252	-0,00291	0,001	0,005	1
Adcy7	0,123252	-0,00291	0,001	0,005	1
Naalad2	0,123252	-0,00291	0,001	0,005	1
Cntnap1	0,123252	-0,00291	0,001	0,005	1
Snx33	0,123431	-0,02557	0,42	0,465	1
Plxna2	0,123439	-0,02116	0,104	0,13	1
Hccs	0,123487	-0,03916	0,484	0,525	1
Arnt	0,12349	-0,0364	0,357	0,39	1
Proscos	0,123526	-0,01465	0,026	0,039	1
Zmynd19	0,123541	-0,02983	0,135	0,163	1
Fmc1	0,123561	0,027832	0,605	0,566	1
Smad7	0,123625	0,046035	0,979	0,993	1
Orai2	0,123627	-0,01142	0,013	0,023	1
Nfx1	0,123696	-0,04237	0,401	0,439	1
Tox2	0,123753	-0,01265	0,013	0,023	1
Mydgf	0,12385	-0,0305	0,646	0,699	1
Irf8	0,123953	-0,00374	0,007	0,015	1
Nbn	0,123964	-0,02917	0,157	0,187	1
Tnpo1	0,123964	-0,03105	0,427	0,473	1
Tmem206	0,12398	0,031758	0,143	0,117	1
Pikfyve	0,123996	-0,0352	0,409	0,455	1
1500015A(	0,124192	0,014714	0,07	0,05	1
Tmem55a	0,124197	-0,02253	0,169	0,2	1
Actl6a	0,124348	-0,04652	0,413	0,446	1
Lypd1	0,124382	0,083183	0,036	0,023	1
Ggt1	0,124437	0,019096	0,062	0,044	1
Trappc12	0,124565	-0,03703	0,372	0,411	1
Poldip2	0,124624	-0,02835	0,401	0,446	1
Trak2	0,124664	0,033812	0,168	0,14	1
Cpsf3	0,124672	-0,03184	0,352	0,39	1
Rps6kb1	0,124686	0,052016	0,58	0,561	1
Ddi2	0,124792	-0,02024	0,288	0,327	1
Lamp1	0,124888	0,028669	0,999	1	1
Tcf20	0,124897	-0,03101	0,24	0,275	1
Dennd1b	0,124928	-0,01325	0,026	0,039	1
Topbp1	0,124955	-0,01777	0,19	0,226	1
Ap3s2	0,12515	-0,03699	0,349	0,387	1
Fez1	0,125291	-0,02595	0,998	1	1
Nfib	0,12557	-0,03984	0,886	0,893	1
Ppm1a	0,125602	-0,01926	0,785	0,821	1
Ehmt1	0,125653	-0,02757	0,367	0,41	1
Asxl3	0,125682	-0,0305	0,306	0,343	1
Slc30a6	0,125871	-0,0166	0,145	0,176	1
Manba	0,125873	0,038452	0,226	0,197	1
Tram2	0,125943	0,035027	0,107	0,085	1
G3bp2	0,125944	-0,02844	0,803	0,849	1

Shroom1	0,125968	-0,04062	0,293	0,332	1
Zswim9	0,125997	-0,0204	0,133	0,161	1
Unc119b	0,1261	-0,02416	0,102	0,127	1
Papola	0,126144	-0,02361	0,689	0,728	1
Ntpcr	0,126192	-0,02932	0,194	0,226	1
Ndfip2	0,126226	-0,02665	0,81	0,842	1
Tti2	0,126239	-0,02178	0,132	0,159	1
Dcaf15	0,126302	-0,02176	0,146	0,176	1
2610002M	0,126319	-0,03029	0,163	0,192	1
Pcnt	0,126346	-0,01848	0,277	0,319	1
Ncs1	0,126449	-0,03281	0,171	0,2	1
Gk	0,126457	-0,01901	0,091	0,115	1
Gm9938	0,126528	-0,01619	0,05	0,068	1
Atp5k	0,126642	-0,03802	0,978	0,976	1
Snx15	0,126732	0,047148	0,814	0,818	1
Olfm1	0,126757	-0,04754	0,398	0,433	1
Stxbp5l	0,126886	-0,01992	0,032	0,047	1
Lxn	0,126951	-0,00693	0,013	0,023	1
Adgrb2	0,12702	-0,02689	0,122	0,148	1
Sar1b	0,127064	-0,03814	0,666	0,701	1
Tdp2	0,127074	-0,04275	0,218	0,249	1
Ccdc88c	0,127123	-0,02185	0,056	0,075	1
Mrpl18	0,127184	-0,03673	0,586	0,616	1
Nrbp1	0,127199	0,035764	0,353	0,317	1
Hoxa5	0,12721	0,023637	0,044	0,029	1
Trim59	0,12722	0,058986	0,972	0,969	1
Tomm22	0,127356	-0,02289	0,721	0,764	1
Lgmn	0,127472	-0,02087	0,906	0,922	1
Mpp6	0,127558	-0,01818	0,053	0,072	1
Csk	0,127644	-0,01804	0,29	0,333	1
Zfp282	0,127654	-0,01823	0,169	0,2	1
Usb1	0,127691	-0,01503	0,073	0,094	1
Spcs2	0,12791	0,047774	0,953	0,953	1
Efs	0,127928	0,058854	0,263	0,231	1
Slirp	0,127958	-0,03069	0,647	0,685	1
Adamts20	0,128129	-0,02405	0,159	0,189	1
Cpeb2	0,128139	-0,03835	0,818	0,829	1
Cacna1c	0,128193	-0,01214	0,036	0,052	1
Snrpg	0,128216	0,052023	0,725	0,725	1
Khnyn	0,128278	0,032226	0,135	0,111	1
Cry1	0,128413	-0,03527	0,207	0,239	1
Ddx6	0,128483	0,052719	0,901	0,87	1
Aptx	0,128559	-0,03495	0,224	0,257	1
Gm8186	0,128568	-0,02748	0,077	0,098	1
Vangl2	0,128569	-0,02759	0,183	0,213	1
Tmem161b	0,12858	0,029646	0,263	0,229	1
Slc16a6	0,128658	-0,02241	0,147	0,176	1
Bahcc1	0,128671	-0,00816	0,135	0,166	1
Leprotl1	0,128687	-0,03031	0,553	0,587	1
Slc31a2	0,128697	-0,02267	0,385	0,433	1
E130308A1	0,128773	-0,04158	0,439	0,483	1
Ttc37	0,128802	-0,02315	0,216	0,25	1

Mafk	0,129114	0,056059	0,165	0,14	1
Ski	0,129239	0,053222	0,538	0,507	1
Gtf3a	0,129242	-0,03428	0,494	0,532	1
Sh3pxd2b	0,129295	-0,01803	0,039	0,055	1
Rps2	0,129329	0,051942	0,992	0,992	1
Apba1	0,129378	0,061088	0,469	0,455	1
Nagpa	0,129398	-0,03119	0,282	0,317	1
Sirt7	0,129404	-0,0308	0,38	0,413	1
Fer	0,129415	-0,03103	0,213	0,244	1
Kctd6	0,129434	0,02779	0,181	0,151	1
Atp2b4	0,129567	-0,01248	0,006	0,013	1
Mfsd1	0,129617	-0,02717	0,214	0,247	1
Pgrmc2	0,129716	-0,03445	0,571	0,605	1
Ehhadh	0,12972	0,024768	0,071	0,052	1
Zfp330	0,129756	-0,01352	0,274	0,315	1
Akap13	0,129786	-0,02268	0,053	0,072	1
Vps33a	0,129796	-0,03815	0,23	0,26	1
Smim14	0,12991	-0,02791	0,837	0,865	1
Mapk6	0,130165	-0,0316	0,39	0,428	1
4930537H:	0,130168	-0,00928	0,006	0,013	1
Lclat1	0,130314	-0,02148	0,078	0,099	1
Gm43653	0,13051	-0,00705	0,006	0,013	1
Psg16	0,13051	-0,00705	0,006	0,013	1
Gm4707	0,130519	-0,01467	0,053	0,072	1
Ly96	0,130566	-0,01639	0,039	0,055	1
Svbp	0,130632	0,043769	0,335	0,299	1
Zfp276	0,130736	0,032322	0,354	0,319	1
Trappc8	0,130745	-0,03031	0,376	0,416	1
Kdm6b	0,130784	0,116903	0,2	0,176	1
Mut	0,130881	-0,02707	0,439	0,494	1
Ppdpf	0,130898	-0,04097	0,633	0,675	1
Efcc1	0,130988	-0,05465	0,186	0,213	1
Zfp260	0,131023	-0,02793	0,406	0,447	1
Cnnm3	0,131034	-0,03944	0,308	0,341	1
Dgki	0,131063	-0,0186	0,296	0,338	1
Rnf11	0,131079	-0,03792	0,62	0,644	1
Zfp58	0,131234	-0,0133	0,07	0,091	1
St3gal2	0,131253	-0,02444	0,162	0,192	1
Edem3	0,131338	-0,01065	0,232	0,27	1
Fastkd1	0,131494	0,040605	0,15	0,125	1
Zfp36l2	0,131559	0,08911	0,577	0,543	1
Galnt10	0,131663	-0,02938	0,146	0,174	1
E330009J0	0,131701	-0,03238	0,5	0,54	1
Sh2b3	0,131714	-0,0107	0,05	0,068	1
Nupl2	0,13175	-0,01875	0,059	0,078	1
Trim21	0,131769	-0,02425	0,052	0,07	1
Eci1	0,131811	-0,0465	0,531	0,561	1
Pde5a	0,131897	-0,00413	0,006	0,013	1
C1qtnf12	0,131973	0,012446	0,084	0,063	1
Ankrd17	0,132131	-0,00411	0,589	0,639	1
Fbf1	0,132152	-0,02716	0,158	0,187	1
Lyn	0,13216	0,008461	0,01	0,003	1

Cox6a1	0,13221	0,029791	0,971	0,974	1
Ormdl3	0,132228	-0,01844	0,342	0,387	1
Dxo	0,132272	-0,03233	0,179	0,208	1
Srp54c	0,132325	-0,0206	0,055	0,073	1
Pilrb2	0,132348	0,008461	0,01	0,003	1
B130011K(	0,132348	0,008461	0,01	0,003	1
Rpp40	0,132362	-0,01756	0,093	0,117	1
Rexo2	0,132379	-0,03347	0,851	0,878	1
Fam241a	0,132398	0,028094	0,276	0,241	1
Gm14308	0,132535	0,006519	0,01	0,003	1
Fam196a	0,132535	0,006519	0,01	0,003	1
Tgds	0,132566	-0,01564	0,084	0,107	1
Psme2	0,132785	-0,11709	0,369	0,403	1
Plppr2	0,132908	-0,01306	0,063	0,083	1
Gm10516	0,132967	0,010062	0,015	0,007	1
Usp38	0,132979	-0,01905	0,195	0,231	1
E2f2	0,132981	-0,01099	0,017	0,028	1
Mia3	0,133085	-0,04445	0,506	0,541	1
Fzd1	0,133103	0,006841	0,01	0,003	1
Gm26852	0,133103	0,006841	0,01	0,003	1
Dpm3	0,133479	-0,03266	0,805	0,829	1
Thsd7b	0,13348	0,0049	0,01	0,003	1
Odf3l2	0,13348	0,001668	0,01	0,003	1
Rbm5	0,133496	-0,03989	0,824	0,842	1
Fam228a	0,1335	-0,02307	0,071	0,091	1
Sez6l2	0,133514	-0,03307	0,967	0,963	1
Gmpr2	0,133649	-0,02222	0,323	0,363	1
Trim45	0,13367	-0,02102	0,083	0,106	1
Paip2	0,133731	-0,0331	0,961	0,977	1
Ube2q2	0,133776	-0,03921	0,333	0,359	1
Tma16	0,133779	-0,02416	0,276	0,307	1
Cep295nl	0,133946	-0,01003	0,017	0,028	1
Slc3a2	0,134101	-0,02575	0,795	0,829	1
Timeless	0,134243	-0,01742	0,025	0,037	1
Rapsn	0,134388	0,00925	0,06	0,042	1
Hacd1	0,134472	0,04574	0,421	0,387	1
Pfkfb3	0,134497	0,032122	0,228	0,197	1
Bola1	0,134515	-0,03872	0,328	0,364	1
Slc22a17	0,134558	0,040915	0,919	0,943	1
Galnt16	0,134606	-0,02295	0,259	0,298	1
Ddit4	0,134943	0,072971	0,469	0,447	1
Uqcr10	0,134957	-0,02643	0,954	0,971	1
Mfap1a	0,135064	-0,03347	0,472	0,519	1
Lrig2	0,135073	-0,02582	0,29	0,33	1
Cmbl	0,135148	0,026648	0,181	0,151	1
Amigo1	0,135332	-0,02286	0,101	0,125	1
Ywhaq	0,135444	-0,02475	0,999	1	1
Gm28836	0,135505	-0,00983	0,012	0,021	1
Ccdc142os	0,135505	-0,00983	0,012	0,021	1
Gm29642	0,135574	-0,01045	0,012	0,021	1
Opa1	0,135655	-0,04083	0,579	0,62	1
Dgkd	0,13568	-0,03957	0,34	0,377	1

Mtch1	0,135768	0,042819	0,972	0,958	1
Zbed5	0,135817	0,024768	0,067	0,049	1
Myadm	0,135903	-0,03425	0,108	0,132	1
Coro2b	0,136206	0,041286	0,459	0,429	1
Fam177a	0,136233	-0,04427	0,222	0,254	1
Tfdp1	0,136277	0,027761	0,196	0,166	1
Unc119	0,136292	-0,01698	0,042	0,059	1
Exoc7	0,1363	-0,04459	0,455	0,483	1
Dnm1l	0,136379	0,064002	0,535	0,524	1
Cops7a	0,136403	-0,03392	0,38	0,418	1
Stox2	0,136469	-0,04504	0,855	0,862	1
Slc9a3r2	0,136494	0,034436	0,321	0,285	1
Klhl22	0,136516	-0,02219	0,138	0,166	1
Zfp830	0,136527	-0,02119	0,269	0,307	1
Mgat1	0,136687	-0,03539	0,272	0,306	1
Gm9008	0,136731	-0,00963	0,005	0,011	1
Apobr	0,136955	-0,01187	0,021	0,033	1
Klf3	0,137076	0,057335	0,598	0,566	1
Amn1	0,137118	-0,0201	0,404	0,452	1
Stpg1	0,137213	-0,00803	0,005	0,011	1
Gm37170	0,137215	-0,00928	0,005	0,011	1
Cnrip1	0,137462	-0,00868	0,204	0,241	1
Mrto4	0,137573	-0,03695	0,478	0,506	1
Cpq	0,137609	-0,04008	0,08	0,101	1
Anxa7	0,137636	0,038303	0,17	0,143	1
Etnppl	0,137697	-0,00642	0,005	0,011	1
Uchl1os	0,137697	-0,00642	0,005	0,011	1
Anxa3	0,137697	-0,00642	0,005	0,011	1
Gm21119	0,137697	-0,00642	0,005	0,011	1
Psd	0,137697	-0,00642	0,005	0,011	1
Sema4d	0,137856	0,057035	0,978	0,976	1
Cit	0,137893	-0,00705	0,005	0,011	1
Camkk1	0,137893	-0,00705	0,005	0,011	1
Gmpr	0,137893	-0,00705	0,005	0,011	1
Cdk5rap1	0,137989	-0,02277	0,061	0,08	1
Rbfox1	0,13799	-0,00608	0,005	0,011	1
Anapc4	0,138124	0,044908	0,325	0,294	1
Usp7	0,138261	-0,0209	0,507	0,548	1
Ndufaf6	0,1383	0,025673	0,057	0,041	1
Tha1	0,138351	-0,01315	0,049	0,067	1
Bex1	0,138378	-0,00447	0,005	0,011	1
Npl	0,138378	-0,00545	0,005	0,011	1
Rslcan18	0,138378	-0,00545	0,005	0,011	1
Msh5	0,138378	-0,00545	0,005	0,011	1
Mtpn	0,138438	-0,01511	0,386	0,431	1
Cdkn1b	0,138447	-0,02904	0,687	0,712	1
Coa7	0,138479	-0,02585	0,108	0,132	1
Pigl	0,138653	-0,01664	0,083	0,106	1
Cenpf	0,138957	-0,00532	0,042	0,059	1
Gm6710	0,138979	-0,02064	0,114	0,14	1
Spata24	0,13898	-0,0263	0,194	0,226	1
E2f4	0,138983	-0,00915	0,172	0,205	1

Ccdc127	0,139001	-0,04502	0,434	0,462	1
Coq6	0,13902	-0,01935	0,18	0,211	1
Ffar1	0,139062	-0,00447	0,005	0,011	1
Tmem117	0,139111	0,04476	0,485	0,455	1
Mtap	0,139244	0,025673	0,057	0,041	1
Prrc2b	0,139528	-0,03067	0,649	0,691	1
3110082J2	0,139615	-0,01707	0,071	0,091	1
Stxbp1	0,139662	-0,03642	0,257	0,289	1
9130023H1	0,139678	-0,0206	0,058	0,076	1
Mbd5	0,139753	-0,02509	0,222	0,255	1
Tnxb	0,139844	0,024545	0,027	0,016	1
Dnajb2	0,139951	0,027366	0,997	0,997	1
Rpl13	0,139968	0,042147	0,998	1	1
Fam35a	0,140027	-0,02274	0,1	0,124	1
Usp14	0,140233	-0,02702	0,411	0,454	1
Kcnq1ot1	0,140288	0,116536	0,776	0,741	1
Sfxn3	0,140291	0,027564	0,116	0,093	1
Napg	0,140385	-0,03819	0,46	0,498	1
Rsbn1l	0,140434	-0,03703	0,485	0,53	1
Prkd3	0,140504	-0,03296	0,698	0,722	1
Hectd1	0,140564	-0,02563	0,792	0,795	1
Avpi1	0,140578	-0,00825	0,046	0,063	1
Polr3f	0,140652	-0,01561	0,209	0,242	1
Pbdc1	0,140831	-0,01211	0,33	0,376	1
Gbp9	0,14088	-0,01577	0,002	0,007	1
Pycr2	0,140986	-0,03662	0,588	0,6	1
Rnps1	0,141016	-0,03345	0,399	0,439	1
Clgn	0,141038	-0,0533	0,135	0,161	1
Zfc3h1	0,141067	-0,02717	0,353	0,389	1
Banf1	0,141103	-0,0467	0,48	0,512	1
Kif6	0,141155	0,034786	0,242	0,211	1
Gm26648	0,141159	-0,00775	0,002	0,007	1
Slc22a21	0,141159	-0,00775	0,002	0,007	1
Atp7b	0,141159	-0,01417	0,002	0,007	1
Acot2	0,141163	0,068536	0,447	0,428	1
Gpatch1	0,141225	-0,01346	0,206	0,242	1
Gm12925	0,141246	0,009112	0,007	0,002	1
Slc16a4	0,141246	0,008141	0,007	0,002	1
Tm6sf2	0,141246	0,008141	0,007	0,002	1
Cfap97	0,14127	-0,01816	0,237	0,275	1
Desi2	0,141342	0,053082	0,282	0,254	1
Ica1l	0,141367	0,006195	0,007	0,002	1
Sec16b	0,141367	0,006195	0,007	0,002	1
Gm15728	0,141367	0,006195	0,007	0,002	1
Gm19265	0,141367	0,006195	0,007	0,002	1
1810028F0	0,141367	0,006195	0,007	0,002	1
Clec3b	0,141367	0,006195	0,007	0,002	1
Gm47167	0,141367	0,006195	0,007	0,002	1
Gm15966	0,141367	0,006195	0,007	0,002	1
Gm31718	0,141438	-0,00614	0,002	0,007	1
Gm47271	0,141438	-0,00614	0,002	0,007	1
Efnb2	0,141438	-0,00775	0,002	0,007	1

1810058I2	0,141463	0,05624	0,758	0,746	1
Map3k6	0,141487	0,005221	0,007	0,002	1
Cers3	0,141487	0,005221	0,007	0,002	1
Igf1	0,141487	0,005221	0,007	0,002	1
Hist1h2bb	0,141487	0,005221	0,007	0,002	1
Ahrr	0,141487	0,005221	0,007	0,002	1
Ppp6r3	0,141492	-0,04075	0,507	0,54	1
Arfip1	0,141497	-0,04679	0,327	0,356	1
Rint1	0,141518	-0,02623	0,36	0,402	1
Epha5	0,141614	-0,01333	0,038	0,054	1
Chst1	0,141717	-0,00452	0,002	0,007	1
Gm14393	0,141717	-0,00452	0,002	0,007	1
Mxra8	0,141717	-0,00452	0,002	0,007	1
Gm45187	0,141717	-0,00452	0,002	0,007	1
Olfr678	0,141717	-0,00452	0,002	0,007	1
Amh	0,141717	-0,00452	0,002	0,007	1
Gm44956	0,141717	-0,00452	0,002	0,007	1
Adprhl1	0,141717	-0,00452	0,002	0,007	1
Pdgfrl	0,141717	-0,00452	0,002	0,007	1
Olfr731	0,141717	-0,00452	0,002	0,007	1
Psmb11	0,141717	-0,00452	0,002	0,007	1
Piwil2	0,141717	-0,00452	0,002	0,007	1
Tex12	0,141717	-0,00452	0,002	0,007	1
Proca1	0,141717	-0,00452	0,002	0,007	1
Serpinb9	0,141717	-0,00452	0,002	0,007	1
Dbn1	0,141717	-0,00452	0,002	0,007	1
Zdhhc22	0,141717	-0,00452	0,002	0,007	1
Cd86	0,141717	-0,00452	0,002	0,007	1
Ip6k3	0,141717	-0,00452	0,002	0,007	1
Spdef	0,141717	-0,00452	0,002	0,007	1
Pcdha9	0,141717	-0,00452	0,002	0,007	1
Best1	0,141717	-0,00452	0,002	0,007	1
Rnmt	0,14184	0,05211	0,317	0,285	1
Tcea1	0,141848	-0,03113	0,82	0,828	1
Mgat4b	0,141867	0,039962	0,326	0,291	1
Gm16702	0,141877	-0,02289	0,027	0,041	1
Got2	0,141946	-0,04318	0,641	0,673	1
Cep19	0,14197	-0,03128	0,066	0,085	1
Col15a1	0,141998	-0,00516	0,002	0,007	1
Ift81	0,142071	-0,02953	0,138	0,164	1
Mrps28	0,142076	-0,03062	0,33	0,367	1
Commd6	0,142113	-0,02367	0,716	0,758	1
Trpc4ap	0,14215	-0,02481	0,835	0,842	1
Nufip2	0,142172	0,073072	0,677	0,668	1
Ndufa2	0,142193	-0,02869	0,969	0,972	1
Ercc6l	0,142217	0,004573	0,007	0,002	1
Pus10	0,142245	-0,02396	0,099	0,122	1
Gm40513	0,142278	-0,00354	0,002	0,007	1
Gm28370	0,142278	-0,00354	0,002	0,007	1
Ipp	0,142298	-0,0275	0,125	0,15	1
Tpmt	0,142306	0,047156	0,182	0,156	1
Slc20a1	0,14235	0,040272	0,169	0,143	1

Tbp	0,142361	-0,03395	0,16	0,187	1
Arpp21	0,14252	-0,01061	0,004	0,01	1
Rab2b	0,142539	-0,02803	0,404	0,452	1
Tmem230	0,142556	-0,03227	0,396	0,439	1
Vezf1	0,142646	-0,01712	0,748	0,797	1
Psmc3	0,142653	0,042037	0,935	0,928	1
Rgs3	0,142674	0,064475	0,75	0,759	1
Sumf2	0,142775	-0,02803	0,149	0,176	1
Flywch2	0,142851	0,029633	0,121	0,098	1
Inpp1	0,142881	0,035724	0,134	0,111	1
Gm14286	0,142956	-0,0074	0,004	0,01	1
1700026J1	0,142956	-0,0074	0,004	0,01	1
Mrap	0,142956	-0,0074	0,004	0,01	1
Dbx2	0,143066	-0,00963	0,004	0,01	1
Dtx3l	0,143106	-0,01433	0,031	0,046	1
Eif4enif1	0,143117	-0,03679	0,292	0,325	1
Kirrel3os	0,143176	-0,00803	0,004	0,01	1
Sox5	0,143176	-0,01026	0,004	0,01	1
Usp4	0,143177	-0,03895	0,389	0,423	1
Fam84b	0,143181	-0,02101	0,016	0,026	1
Zbtb37	0,14327	-0,03723	0,299	0,332	1
Gm16240	0,143367	0,025767	0,031	0,02	1
Opn3	0,143393	-0,00579	0,004	0,01	1
Gm17096	0,143393	-0,00579	0,004	0,01	1
Scml2	0,143393	-0,00579	0,004	0,01	1
Plch2	0,143393	-0,00579	0,004	0,01	1
Ppp1r1a	0,143393	-0,00579	0,004	0,01	1
Ggta1	0,143437	-0,01874	0,034	0,049	1
Bicd2	0,143479	-0,04179	0,714	0,746	1
Ttc39c	0,143516	0,041514	0,151	0,127	1
Matn4	0,143589	0,033602	0,536	0,501	1
Zfp827	0,143606	0,040109	0,159	0,133	1
Fcgrt	0,143613	-0,00642	0,004	0,01	1
AU020206	0,143613	-0,00642	0,004	0,01	1
Rnf214	0,143616	-0,03308	0,337	0,374	1
Slc25a24	0,143714	0,016141	0,017	0,008	1
Brd1	0,143724	0,052094	0,617	0,572	1
Pole4	0,1438	-0,04478	0,481	0,509	1
Fam135b	0,143826	-0,01849	0,02	0,031	1
Plekho1	0,143986	0,013839	0,027	0,016	1
Slc10a3	0,144021	-0,0149	0,027	0,041	1
Gm37850	0,144052	-0,00384	0,004	0,01	1
Gm38336	0,144052	-0,00481	0,004	0,01	1
Cdiptos	0,144052	-0,00481	0,004	0,01	1
Muc4	0,144052	-0,00481	0,004	0,01	1
Rad51	0,14406	-0,01933	0,024	0,036	1
Rbl2	0,144133	-0,03266	0,506	0,53	1
Ppp1r16b	0,144152	0,065437	0,982	0,985	1
Aph1a	0,144228	-0,02723	0,701	0,737	1
Sept10	0,144299	0,032156	0,267	0,234	1
Prkaa2	0,14436	-0,02652	0,103	0,127	1
Ccdc73	0,144363	-0,01479	0,071	0,091	1

Tyw5	0,144371	-0,02144	0,19	0,221	1
1700018L(	0,144457	0,016141	0,017	0,008	1
Smarcc1	0,144474	-0,04221	0,517	0,546	1
Jup	0,144524	-0,02503	0,854	0,857	1
Sdc2	0,144551	-0,00596	0,035	0,05	1
Vamp7	0,144581	-0,03262	0,48	0,527	1
Alg13	0,144583	-0,02348	0,132	0,158	1
Mdm2	0,144671	0,057871	0,616	0,593	1
Mzb1	0,144714	-0,00384	0,004	0,01	1
Lmbr1	0,144863	-0,02946	0,215	0,244	1
Slain1	0,144914	0,024676	0,999	1	1
Tpm3	0,144932	0,052899	0,871	0,865	1
Rpl12	0,144938	0,036259	0,946	0,928	1
Ribc1	0,144959	-0,01576	0,027	0,041	1
Ttc38	0,14497	-0,02291	0,112	0,137	1
Cntn4	0,14497	-0,01363	0,058	0,076	1
Hlf	0,144988	-0,01033	0,031	0,046	1
Foxm1	0,145029	-0,01353	0,016	0,026	1
Smc5	0,145096	-0,03789	0,389	0,421	1
Cct5	0,145124	-0,02978	0,854	0,872	1
Gm47061	0,145159	-0,00838	0,003	0,008	1
Cpne8	0,145159	-0,00838	0,003	0,008	1
Gm7008	0,145202	0,01134	0,017	0,008	1
Selenoi	0,145225	-0,03952	0,352	0,389	1
Spire1	0,145299	-0,00215	0,457	0,524	1
Mapk7	0,145319	-0,01995	0,19	0,223	1
Cxcl14	0,145459	-0,02683	0,027	0,041	1
Epha7	0,14553	-0,00677	0,003	0,008	1
Gm44210	0,14553	-0,00677	0,003	0,008	1
Gm17344	0,14553	-0,00677	0,003	0,008	1
Hoxb9	0,14553	-0,00677	0,003	0,008	1
Hoxc9	0,14553	-0,00677	0,003	0,008	1
Alpk2	0,145575	0,014223	0,017	0,008	1
Mcrip1	0,145702	-0,02153	0,837	0,865	1
Elf5	0,145901	-0,00516	0,003	0,008	1
Gm31305	0,145901	-0,00516	0,003	0,008	1
Casq2	0,145901	-0,00516	0,003	0,008	1
Gm11831	0,145901	-0,00516	0,003	0,008	1
Laptm5	0,145901	-0,00516	0,003	0,008	1
A2m	0,145901	-0,00516	0,003	0,008	1
Olfr30	0,145901	-0,00516	0,003	0,008	1
Abhd15	0,145901	-0,00516	0,003	0,008	1
Dtnbos	0,145901	-0,00516	0,003	0,008	1
AC163633.	0,145901	-0,00516	0,003	0,008	1
Gm35558.:	0,145901	-0,00516	0,003	0,008	1
Capn11	0,145901	-0,00516	0,003	0,008	1
Rpl7a-ps5	0,145901	-0,00516	0,003	0,008	1
Gm26823	0,145901	-0,00516	0,003	0,008	1
Gm17669	0,145901	-0,00516	0,003	0,008	1
Mink1	0,146191	-0,00578	0,69	0,764	1
Atp6v1d	0,1462	-0,03897	0,633	0,683	1
Atxn7l2	0,146323	-0,01214	0,055	0,073	1

Gm19345	0,146523	-0,0032	0,003	0,008	1
Phf19	0,146523	-0,00418	0,003	0,008	1
4930548H:	0,146523	-0,00418	0,003	0,008	1
1700124L1	0,146523	-0,00418	0,003	0,008	1
Gngt2	0,146523	-0,00418	0,003	0,008	1
Ift122	0,146548	-0,03346	0,157	0,184	1
Tspo	0,14657	0,008965	0,126	0,101	1
B230317F2	0,146655	-0,01813	0,02	0,031	1
Fbxw9	0,146705	0,032877	0,172	0,145	1
Pvt1	0,146714	-0,02142	0,185	0,216	1
Tnks2	0,146742	-0,02996	0,818	0,842	1
Dgcr6	0,146743	-0,02051	0,378	0,423	1
Rrs1	0,146798	-0,02584	0,48	0,533	1
Hibadh	0,146841	-0,03163	0,642	0,673	1
Mtfr1	0,146841	-0,01914	0,164	0,193	1
Prrt1	0,146861	-0,03362	0,617	0,642	1
Hacd2	0,14698	-0,0429	0,418	0,459	1
Trnt1	0,146997	-0,0486	0,556	0,572	1
Pik3c2a	0,147012	-0,03316	0,545	0,592	1
Trim12c	0,14705	-0,02307	0,011	0,02	1
Tmem209	0,147053	-0,01758	0,275	0,312	1
Pcdhb4	0,147088	0,014183	0,017	0,008	1
Sema6c	0,147093	-0,00879	0,016	0,026	1
Sun1	0,147102	0,069082	0,381	0,363	1
Rassf1	0,147217	0,047413	0,368	0,335	1
Mea1	0,147332	-0,03712	0,386	0,423	1
St8sia5	0,147445	0,024711	0,297	0,262	1
Frmd4b	0,147475	-0,02159	0,879	0,881	1
Drc3	0,147487	-0,00859	0,011	0,02	1
1810021B:	0,147529	0,01224	0,029	0,018	1
Scn1b	0,147529	0,035481	0,17	0,141	1
Apmap	0,147563	0,020021	0,297	0,26	1
Spopl	0,147611	-0,02493	0,252	0,288	1
Pbx1	0,147623	-0,01297	0,298	0,34	1
Cebpzos	0,147723	0,04462	0,296	0,27	1
Csrnp1	0,147726	0,103873	0,168	0,143	1
Spry1	0,148066	-0,02847	0,249	0,283	1
Bmp2k	0,148115	-0,03272	0,906	0,911	1
Gdap10	0,14813	0,006138	0,144	0,176	1
A130010J1	0,148141	0,01484	0,085	0,065	1
Slc39a6	0,148226	-0,04396	0,25	0,278	1
Nifk	0,148272	-0,03584	0,254	0,283	1
Pclo	0,148303	-0,03698	0,297	0,328	1
Bhlhe40	0,14836	0,108253	0,246	0,221	1
Letm2	0,148432	-0,03661	0,303	0,332	1
Tspan15	0,148531	0,042503	0,957	0,951	1
Snx25	0,148828	-0,01418	0,041	0,057	1
Copb1	0,148943	0,042705	0,586	0,554	1
Trim26	0,148951	-0,03092	0,317	0,353	1
Ak1	0,148994	-0,03571	0,234	0,265	1
Acp1	0,149123	0,030722	0,622	0,595	1
Tusc3	0,149194	-0,0431	0,177	0,202	1

Ube2v1	0,149204	0,017128	0,049	0,034	1
Ubqln2	0,149339	-0,02807	0,734	0,766	1
Sltm	0,14936	-0,02576	0,781	0,816	1
Gm29773	0,149442	0,005254	0,016	0,026	1
Ccdc71	0,149527	0,026796	0,124	0,101	1
Lifr	0,14954	-0,04383	0,509	0,551	1
Eef1a1	0,149612	-0,01952	0,999	1	1
Zfp65	0,149892	-0,03426	0,122	0,145	1
Zcchc17	0,149919	-0,01803	0,698	0,766	1
1700066B1	0,150097	-0,01813	0,02	0,031	1
Camk1	0,150143	-0,04591	0,386	0,415	1
Smarcb1	0,150193	-0,02214	0,624	0,654	1
Smug1	0,150234	-0,02155	0,129	0,154	1
Gm30122	0,150261	0,01841	0,043	0,029	1
Aqp4	0,150285	-0,01144	0,024	0,036	1
Matn2	0,150328	-0,03036	0,189	0,22	1
Zfyve27	0,150417	-0,03706	0,41	0,437	1
4931406P1	0,150439	-0,03932	0,806	0,813	1
Cyb5d1	0,150477	-0,00995	0,024	0,036	1
Unc5b	0,150614	-0,04077	0,955	0,958	1
Lig1	0,150661	0,03507	0,269	0,237	1
Brd7	0,150719	-0,02953	0,659	0,683	1
Morc2a	0,150864	-0,01884	0,176	0,207	1
Gm17690	0,150883	0,011904	0,031	0,02	1
Olfr889	0,150957	0,0043	0,027	0,016	1
Spice1	0,151136	-0,02829	0,196	0,226	1
Zfp592	0,151196	0,033815	0,192	0,164	1
1110046J0	0,15133	-0,01657	0,216	0,249	1
Prdx4	0,151417	0,056127	0,447	0,416	1
Nkx6-2	0,151587	0,03797	0,988	0,985	1
Serbp1	0,151644	-0,0236	0,967	0,972	1
Rangrf	0,151942	-0,02231	0,136	0,163	1
Plcb4	0,152008	0,050526	0,17	0,145	1
Sumo3	0,152054	0,057478	0,542	0,527	1
Zc2hc1a	0,152099	-0,02391	0,188	0,22	1
AC158554.	0,152381	0,00962	0,045	0,031	1
Mief2	0,152389	-0,02396	0,107	0,13	1
Mcm3	0,152492	0,019116	0,105	0,083	1
Tnpo2	0,152519	-0,02335	0,29	0,327	1
Sag	0,152532	0,019415	0,082	0,063	1
Scrn2	0,152635	-0,01143	0,092	0,115	1
Eif5a2	0,152661	-0,03296	0,229	0,259	1
Hexdc	0,152663	0,030362	0,184	0,158	1
Ammecr1l	0,152732	0,043975	0,267	0,239	1
Dhrs13	0,152743	-0,01757	0,047	0,063	1
Mfge8	0,152854	0,045668	0,44	0,398	1
Vps8	0,152924	-0,02627	0,401	0,449	1
2700046G(	0,152996	-0,0215	0,116	0,14	1
Dexi	0,153174	-0,03819	0,226	0,255	1
Syde2	0,153195	-0,01541	0,079	0,101	1
Slc25a5	0,153209	-0,03764	0,761	0,779	1
Rbfa	0,153245	-0,01889	0,265	0,301	1

Pprc1	0,153307	0,027033	0,134	0,111	1
Ptpdc1	0,153389	-0,05203	0,494	0,512	1
Dhodh	0,153423	-0,02849	0,166	0,193	1
Mboat7	0,153453	-0,01616	0,234	0,27	1
Paqr3	0,153477	-0,01273	0,054	0,072	1
Smim12	0,153492	-0,03757	0,345	0,379	1
Ppp1r21	0,153596	-0,02946	0,697	0,737	1
F8a	0,153807	-0,03382	0,183	0,21	1
Rab11a	0,153832	-0,0262	0,616	0,655	1
Stxbp4	0,15384	0,032454	0,12	0,098	1
Gm15417	0,153852	0,062537	0,275	0,249	1
Rbms2	0,153875	-0,02454	0,069	0,088	1
Pdrg1	0,15389	-0,02807	0,407	0,447	1
Cdkl3	0,153993	-0,02851	0,113	0,137	1
Wdr5	0,154029	-0,02178	0,13	0,154	1
Ftx	0,154079	-0,02055	0,087	0,109	1
Mast3	0,154483	0,057585	0,827	0,821	1
Gm38393	0,154707	-0,019	0,087	0,109	1
Cdk5	0,154782	-0,02499	0,701	0,725	1
Dgke	0,154817	-0,01945	0,156	0,184	1
Rab11fip5	0,154918	0,011236	0,035	0,023	1
Mks1	0,155033	-0,04134	0,24	0,268	1
Myo1c	0,155059	0,017421	0,043	0,029	1
Bc1	0,15506	0,01369	0,039	0,026	1
Pou2f1	0,155161	0,059059	0,343	0,317	1
Dtnbp1	0,155183	-0,02526	0,222	0,254	1
Cnih3	0,15521	0,020098	0,066	0,049	1
Seh1l	0,155216	-0,02233	0,253	0,289	1
Tk1	0,155706	0,010713	0,012	0,005	1
Ecm2	0,155706	0,010713	0,012	0,005	1
Afap1l1	0,155774	0,060645	0,421	0,393	1
Bsdc1	0,155807	-0,05004	0,462	0,494	1
Kcnh7	0,155923	0,011002	0,019	0,01	1
Qars	0,155997	-0,0237	0,335	0,374	1
Ovgp1	0,156032	0,011236	0,035	0,023	1
Oxnad1	0,156032	-0,02564	0,163	0,19	1
Hps5	0,156061	-0,01395	0,214	0,247	1
Tprkb	0,156107	-0,04764	0,589	0,615	1
Ckap2	0,156278	0,009747	0,012	0,005	1
Pm20d1	0,156281	0,016796	0,012	0,005	1
Fzd9	0,15633	-0,01075	0,03	0,044	1
1700086O	0,15633	-0,01075	0,03	0,044	1
Wdr91	0,15633	-0,00986	0,458	0,506	1
Alkbh2	0,156337	0,009983	0,035	0,023	1
Mlh3	0,156391	-0,02099	0,388	0,426	1
Gm2a	0,156491	0,029829	0,598	0,559	1
Sdf2l1	0,156526	-0,04718	0,378	0,413	1
Pnp2	0,156564	0,007811	0,012	0,005	1
Net1	0,156564	0,007811	0,012	0,005	1
Gm6169	0,156564	0,007811	0,012	0,005	1
Nrxn3	0,156564	0,007811	0,012	0,005	1
Tlr3	0,156575	-0,01551	0,026	0,039	1

Cxcr4	0,156591	0,008751	0,019	0,01	1
Plekhg1	0,156621	-0,01274	0,806	0,834	1
1810024B(	0,156637	-0,00689	0,097	0,12	1
Gm13205	0,156851	0,006841	0,012	0,005	1
Cyba	0,156851	0,006841	0,012	0,005	1
Mrps2	0,156869	-0,02324	0,119	0,143	1
Prpsap1	0,156928	0,049276	0,667	0,641	1
Gm12326	0,156965	-0,02904	0,201	0,231	1
4930503L1	0,156966	0,0329	0,188	0,163	1
Pcdhb13	0,157142	0,00813	0,012	0,005	1
Rpl5	0,157251	0,036161	0,99	0,989	1
Sgk1	0,157291	0,086603	0,534	0,507	1
Phactr2	0,157474	-0,04892	0,247	0,273	1
Vps72	0,15753	-0,02514	0,404	0,439	1
Prelid3b	0,157832	-0,03197	0,382	0,42	1
Suz12	0,157893	-0,02926	0,37	0,405	1
Nabp1	0,157931	0,052323	0,103	0,083	1
Rpl35	0,157994	0,04725	0,962	0,964	1
Srek1	0,158144	-0,03045	0,667	0,719	1
Taf12	0,158715	-0,0166	0,298	0,337	1
Lrp4	0,158745	-0,06487	0,209	0,236	1
Mgst3	0,159072	-0,04183	0,505	0,532	1
Baz2b	0,159089	-0,05814	0,805	0,82	1
2610044O	0,159135	-0,02019	0,133	0,159	1
Adgra1	0,15916	-0,00635	0,03	0,044	1
Them4	0,159162	-0,01919	0,296	0,335	1
Nipsnap3b	0,159207	0,040616	0,555	0,517	1
Kank1	0,159258	-0,01703	0,01	0,018	1
Arhgef28	0,159332	-0,02729	0,515	0,553	1
Zfand2b	0,159333	0,042991	0,32	0,288	1
Smcr8	0,159355	-0,02176	0,141	0,167	1
Fbxw5	0,159358	-0,0488	0,318	0,343	1
Bbs2	0,159535	-0,02207	0,141	0,167	1
Drg2	0,159695	-0,02873	0,414	0,452	1
Cdc34	0,159742	-0,02177	0,266	0,301	1
Igf2r	0,159802	-0,01586	0,199	0,231	1
Smchd1	0,15982	-0,01991	0,5	0,528	1
Rock2	0,159884	-0,00143	0,705	0,772	1
Tbc1d23	0,159986	-0,04335	0,437	0,475	1
Map2	0,160098	-0,04295	0,266	0,298	1
Fam96b	0,160351	-0,04057	0,701	0,709	1
Psma3	0,160439	-0,03142	0,894	0,901	1
Snrpe	0,160624	-0,03544	0,6	0,626	1
Ccnd3	0,160711	0,037743	0,273	0,246	1
AC154760.	0,160738	-0,00859	0,01	0,018	1
Mterf2	0,16075	-0,02642	0,079	0,099	1
Fbxl12os	0,160877	-0,01414	0,019	0,029	1
Pdlim7	0,160997	-0,01195	0,019	0,029	1
Med18	0,161087	-0,0165	0,069	0,088	1
Enox2	0,161138	-0,01753	0,18	0,21	1
Irf2bp2	0,161274	-0,04207	0,793	0,792	1
Lyplal1	0,161434	-0,01585	0,053	0,07	1

Nsmf	0,161461	0,036531	0,562	0,533	1
Gm33979	0,16151	0,013877	0,021	0,011	1
Nrep	0,16151	0,013877	0,021	0,011	1
Gsta4	0,161566	-0,00408	0,01	0,018	1
Vdac2	0,161608	-0,02929	0,932	0,945	1
Zfp420	0,161678	-0,02538	0,118	0,141	1
Klc1	0,161709	0,065378	0,694	0,685	1
Kdm4c	0,161866	-0,0245	0,217	0,247	1
Ttf1	0,16187	-0,03655	0,256	0,285	1
Kbtbd3	0,161902	-0,02593	0,198	0,228	1
Celf2	0,161948	-0,0029	0,048	0,065	1
Hey2	0,162014	0,018651	0,021	0,011	1
Gm4593	0,162037	-0,04132	0,552	0,58	1
AA986860	0,162043	-0,0215	0,068	0,086	1
Ccdc78	0,162074	-0,0116	0,019	0,029	1
Mb21d2	0,162171	0,009313	0,129	0,104	1
Tbc1d10a	0,162185	-0,01289	0,079	0,099	1
Fbxl18	0,162234	0,039003	0,222	0,195	1
Pxmp2	0,162258	-0,02283	0,138	0,164	1
Hspa9	0,162276	0,047523	0,808	0,81	1
Gm26789	0,162307	0,016091	0,021	0,011	1
Aftph	0,16232	-0,02663	0,412	0,454	1
Sec13	0,16244	-0,02876	0,586	0,611	1
4930513D	0,162483	-0,00312	0,01	0,018	1
Epc1	0,162576	0,064457	0,58	0,554	1
Kifc5b	0,162579	-0,01152	0,023	0,034	1
Sav1	0,16258	-0,0175	0,326	0,367	1
Pcdh8	0,162653	-0,00568	0,01	0,018	1
Psma5	0,16276	0,042754	0,715	0,709	1
Wdr47	0,162785	-0,03967	0,337	0,366	1
Rest	0,162832	-0,0339	0,328	0,356	1
Gm5914	0,162885	-0,02996	0,091	0,112	1
Prmt5	0,162953	-0,01051	0,214	0,249	1
Cd200	0,16298	0,009929	0,169	0,202	1
Large1	0,162985	-0,02475	0,074	0,093	1
Pdzd7	0,16303	0,012271	0,021	0,011	1
Kcnk2	0,163109	0,017641	0,021	0,011	1
Gm9833	0,163273	-0,01003	0,019	0,029	1
Fbxw7	0,163362	-0,01681	0,167	0,195	1
Mpv17	0,163394	-0,03261	0,487	0,519	1
Fam72a	0,163404	-0,00505	0,01	0,018	1
Cenpv	0,163426	0,036881	0,228	0,2	1
Gm48960	0,163618	-0,00546	0,026	0,039	1
Nans	0,163698	-0,02121	0,211	0,244	1
Supt20	0,16378	0,063594	0,452	0,444	1
Map7d1	0,163925	0,03504	0,99	0,995	1
Fam229b	0,16406	-0,02716	0,159	0,185	1
Atad2	0,164193	0,01813	0,093	0,073	1
Dnajc27	0,164232	-0,01177	0,117	0,141	1
Fam160b1	0,164274	-0,02795	0,243	0,275	1
Gria4	0,164357	-0,06667	0,282	0,306	1
Pdcd11	0,164438	0,036281	0,32	0,289	1

Champ1	0,164811	-0,0307	0,172	0,2	1
Cep95	0,164851	-0,01509	0,128	0,153	1
Rtn3	0,164958	-0,01916	0,997	0,997	1
Fastkd3	0,164966	0,014502	0,062	0,046	1
Jdp2	0,165046	0,045889	0,377	0,35	1
Gins3	0,165065	-0,01545	0,066	0,085	1
Tmem132a	0,165069	-0,01914	0,178	0,208	1
Zfx	0,165122	-0,02733	0,289	0,327	1
Ddhd2	0,165176	0,056218	0,523	0,498	1
Hacd3	0,165188	-0,02427	0,313	0,35	1
9330104G(	0,16552	-0,01978	0,089	0,111	1
Cib2	0,165643	-0,00838	0,019	0,029	1
Elac2	0,165649	0,031563	0,168	0,143	1
Poc1b	0,165656	-0,03005	0,191	0,22	1
Gipc1	0,16569	-0,03336	0,48	0,509	1
Gtf2e1	0,165787	-0,01789	0,156	0,184	1
Tarbp1	0,165799	-0,0155	0,056	0,073	1
Rusc1	0,165805	0,00715	0,021	0,011	1
Rxra	0,166117	-0,02818	0,073	0,091	1
Ralgapa1	0,166126	-0,0318	0,493	0,541	1
Hbs1l	0,166156	-0,0299	0,376	0,41	1
Tbccd1	0,16622	-0,03169	0,215	0,244	1
Ttll12	0,166408	-0,01641	0,116	0,14	1
Eefsec	0,166409	-0,02468	0,307	0,341	1
Aacs	0,166458	0,080289	0,323	0,299	1
Golga2	0,166489	0,045216	0,408	0,377	1
Iqce	0,166505	-0,0287	0,117	0,14	1
Gm12258	0,166547	-0,02552	0,096	0,117	1
AC133505.	0,166576	-0,01026	0,04	0,055	1
Traf7	0,166631	-0,02296	0,184	0,213	1
Atp1b2	0,166748	-0,01723	0,08	0,101	1
Amy1	0,166764	0,024644	0,12	0,098	1
Kmt2d	0,167033	-0,00974	0,431	0,475	1
Psmd4	0,167072	-0,02613	0,862	0,889	1
2700049A(	0,167088	-0,01271	0,109	0,133	1
Bglap2	0,167189	-0,00423	0,019	0,029	1
Pds5a	0,167443	0,038176	0,473	0,452	1
Suds3	0,167484	-0,01331	0,374	0,426	1
Phrf1	0,167553	-0,03453	0,309	0,343	1
Uxs1	0,167921	-0,02567	0,252	0,283	1
Sugt1	0,167958	-0,05433	0,675	0,683	1
Cpt2	0,168117	-0,02213	0,165	0,192	1
Stat5a	0,168142	-0,00932	0,04	0,055	1
D130040H	0,168354	-0,02167	0,13	0,154	1
Pms1	0,168534	-0,01347	0,142	0,169	1
Vapa	0,168559	0,038272	0,957	0,963	1
St13	0,168576	-0,022	0,989	0,987	1
Nlrx1	0,168723	-0,01595	0,046	0,062	1
Scly	0,168899	-0,02543	0,326	0,366	1
Phip	0,168912	-0,02541	0,775	0,784	1
Kdm2b	0,168934	-0,02687	0,214	0,242	1
Ndel1	0,169	-0,03027	0,402	0,433	1

Spcs3	0,169112	-0,04279	0,302	0,333	1
Abcg2	0,169178	0,016632	0,058	0,042	1
Uqcc3	0,16919	-0,02724	0,424	0,459	1
Sdhaf1	0,169232	-0,02176	0,364	0,405	1
Mrpl51	0,16924	-0,03937	0,58	0,603	1
Yipf2	0,169309	0,031709	0,267	0,237	1
Eif2s3x	0,16931	0,045301	0,524	0,506	1
2700038G:	0,16933	0,025583	0,079	0,062	1
Fam214b	0,169425	0,046009	0,264	0,236	1
Cercam	0,169434	-0,02051	0,212	0,244	1
Zbtb3	0,169459	0,010355	0,023	0,013	1
Nudt12	0,169748	-0,01104	0,046	0,062	1
Ncl	0,170094	0,033857	0,974	0,958	1
Dpp3	0,170111	-0,02966	0,303	0,338	1
Tfb1m	0,170112	-0,01609	0,116	0,14	1
Ubp1	0,170255	-0,04588	0,319	0,345	1
Mterf4	0,170286	-0,01691	0,141	0,167	1
Arhgap17	0,170387	-0,02724	0,414	0,454	1
Acat2	0,17049	0,067072	0,394	0,379	1
Phlda1	0,170547	0,035055	0,266	0,231	1
Pomt2	0,170677	0,035655	0,248	0,22	1
Dnajc18	0,170809	-0,02378	0,633	0,654	1
Commd8	0,170833	-0,04341	0,556	0,585	1
Dnaja1	0,171011	-0,0285	0,958	0,979	1
Hook3	0,171134	-0,04038	0,862	0,885	1
Spag9	0,171222	-0,02748	0,967	0,98	1
Uqcc1	0,17138	-0,0412	0,196	0,221	1
Sema5a	0,171534	-0,05149	0,289	0,317	1
Etfb	0,171564	-0,02866	0,614	0,637	1
Hibch	0,171813	-0,02953	0,269	0,301	1
Usp30	0,171961	-0,01422	0,459	0,511	1
Pbxip1	0,17206	-0,02286	0,186	0,215	1
Rnf26	0,172085	-0,01874	0,184	0,213	1
Eri3	0,172145	-0,03982	0,553	0,574	1
Ctcf	0,172151	0,053004	0,72	0,714	1
Khdrbs2	0,172157	-0,0143	0,026	0,037	1
Traf3	0,172201	-0,02953	0,084	0,104	1
Cd320	0,172334	-0,03055	0,181	0,208	1
Tenm3	0,172605	-0,00712	0,036	0,05	1
Tsc2	0,172745	-0,05426	0,374	0,402	1
Vamp1	0,172779	-0,00921	0,093	0,115	1
9930012K:	0,173021	0,021138	0,069	0,052	1
Gcsh	0,173065	-0,01171	0,302	0,341	1
Polr2m	0,173326	-0,02384	0,436	0,473	1
Magi2	0,173343	0,038294	0,919	0,909	1
Gm44658	0,173428	-0,00948	0,014	0,023	1
Gm30025	0,173456	-0,03021	0,18	0,207	1
Hmga2	0,173462	-0,01115	0,009	0,016	1
Pfn4	0,173535	0,025585	0,09	0,072	1
Ucp2	0,173546	-0,01586	0,022	0,033	1
Card6	0,173728	0,011027	0,014	0,007	1
Pir	0,173787	0,023039	0,133	0,111	1

Dcakd	0,173791	0,029369	0,166	0,141	1
Ppp1r26	0,173995	0,015742	0,026	0,016	1
Psmb3	0,174039	0,036981	0,92	0,914	1
Crem	0,174045	-0,02327	0,156	0,182	1
Tvp23bos	0,174106	0,009097	0,014	0,007	1
4933411E(	0,174106	0,011027	0,014	0,007	1
Gm3055	0,174106	0,010062	0,014	0,007	1
Gm42495	0,174196	-0,00893	0,009	0,016	1
Dqx1	0,174205	0,015512	0,014	0,007	1
Necap1	0,174346	-0,01848	0,211	0,241	1
Gm48604	0,174424	-0,01561	0,022	0,033	1
Sypl	0,174449	0,025904	0,994	0,992	1
9330102E(	0,174484	0,00813	0,014	0,007	1
Gm49064	0,174484	0,00813	0,014	0,007	1
Zcchc14	0,174547	-0,00958	0,311	0,354	1
2610524H(	0,174577	-0,02358	0,177	0,205	1
Gm48071	0,174659	0,014791	0,026	0,016	1
Eif2b2	0,174808	-0,01302	0,346	0,389	1
Nek11	0,174863	0,007162	0,014	0,007	1
1110019D	0,174944	-0,01445	0,068	0,086	1
Katnbl1	0,174965	-0,02149	0,21	0,239	1
Ints6	0,175028	0,092265	0,409	0,39	1
Sytl2	0,175037	0,063163	0,817	0,813	1
Map3k14	0,175059	0,007801	0,014	0,007	1
Xcr1	0,175086	0,010022	0,025	0,015	1
Arl6ip5	0,175087	-0,01291	0,723	0,772	1
Adarb2	0,175123	-0,00796	0,009	0,016	1
Dnd1	0,17534	-0,00914	0,014	0,023	1
Gnb1	0,175488	0,041828	0,974	0,967	1
Mrps26	0,175521	0,049295	0,502	0,488	1
Sdccag3	0,175527	-0,02596	0,479	0,517	1
Zfp438	0,175591	0,034161	0,139	0,117	1
Irgq	0,175659	0,033554	0,124	0,102	1
Utp14b	0,175774	0,017834	0,412	0,457	1
Ip6k1	0,175795	-0,0398	0,495	0,522	1
E4f1	0,175814	-0,01683	0,215	0,249	1
Tns3	0,175847	-0,03313	0,594	0,629	1
Anks1	0,175857	-0,04988	0,236	0,262	1
Lepr	0,175871	-0,00755	0,009	0,016	1
Plaur	0,175958	-0,00636	0,009	0,016	1
AC110241.	0,175971	0,030071	0,131	0,109	1
Bcs1l	0,175978	-0,01375	0,156	0,184	1
Imp4	0,176158	0,039809	0,539	0,502	1
Ninj1	0,176188	-0,00703	0,106	0,128	1
Slc25a46	0,176199	-0,02632	0,383	0,424	1
Gm36266	0,176236	-0,00286	0,018	0,028	1
Ptgds	0,176308	-0,06369	0,892	0,863	1
Prss41	0,176334	0,015093	0,026	0,016	1
Gm47059	0,176375	-0,01118	0,022	0,033	1
Exosc4	0,176386	-0,02631	0,396	0,431	1
Ttl	0,176442	0,036695	0,124	0,102	1
Mospd3	0,176473	-0,0291	0,152	0,177	1

Ppfia3	0,176493	0,010644	0,025	0,015	1
Cdk11b	0,176693	0,02668	0,622	0,577	1
Myrf	0,176693	0,03321	0,989	0,998	1
Mboat1	0,176875	0,050357	0,442	0,411	1
Sc5d	0,176894	0,053398	0,834	0,844	1
Smc3	0,176953	-0,02803	0,743	0,784	1
Rnpepl1	0,176966	0,031859	0,647	0,608	1
Syncrip	0,177035	0,059938	0,578	0,554	1
Alg3	0,177064	-0,01988	0,111	0,133	1
Gm15991	0,177073	-0,01274	0,022	0,033	1
Smurf1	0,17708	0,030397	0,184	0,158	1
Pias2	0,177149	0,043186	0,356	0,325	1
Gm29825	0,177192	-0,01099	0,018	0,028	1
Gm9774	0,177198	0,012092	0,052	0,037	1
Tpx2	0,177338	-0,01126	0,018	0,028	1
Pycard	0,177401	0,020144	0,048	0,034	1
Lysmd3	0,177545	-0,00829	0,34	0,379	1
Cacng7	0,177629	-0,03299	0,323	0,356	1
Depdc5	0,177701	-0,03467	0,295	0,325	1
Gjc2	0,177814	-0,02206	0,97	0,984	1
L3mbtl2	0,177844	-0,03306	0,209	0,237	1
Gdpd1	0,177889	-0,05276	0,569	0,584	1
Ncoa3	0,178061	0,042232	0,507	0,475	1
Col13a1	0,178096	0,011167	0,069	0,052	1
Med15	0,178219	-0,0239	0,349	0,387	1
Gm28151	0,178305	-0,01064	0,018	0,028	1
Mafa	0,178319	0,014143	0,028	0,018	1
Ppl	0,178336	0,021138	0,048	0,034	1
Inpp4a	0,178396	-0,01623	0,052	0,068	1
Zdbf2	0,178406	0,00294	0,003	0	1
Cd48	0,178406	0,00294	0,003	0	1
Cd84	0,178406	0,00294	0,003	0	1
Spaca9	0,178406	0,00294	0,003	0	1
Ttc16	0,178406	0,00294	0,003	0	1
Olfr1156	0,178406	0,00294	0,003	0	1
Il1b	0,178406	0,00294	0,003	0	1
Cfap61	0,178406	0,00294	0,003	0	1
Gpc4	0,178406	0,00294	0,003	0	1
Kcne1l	0,178406	0,00294	0,003	0	1
Htr2c	0,178406	0,00294	0,003	0	1
Tlr2	0,178406	0,00294	0,003	0	1
Gm40155	0,178406	0,00294	0,003	0	1
Bmpr1b	0,178406	0,00294	0,003	0	1
Ak4	0,178406	0,00294	0,003	0	1
Echdc2	0,178406	0,00294	0,003	0	1
Skint8	0,178406	0,00294	0,003	0	1
Nphp4	0,178406	0,00294	0,003	0	1
Fbxl13	0,178406	0,00294	0,003	0	1
Rhoh	0,178406	0,00294	0,003	0	1
Hcar2	0,178406	0,00294	0,003	0	1
Gm26826	0,178406	0,00294	0,003	0	1
Cd22	0,178406	0,00294	0,003	0	1

G630030J(	0,178406	0,00294	0,003	0	1
Gm9885	0,178406	0,00294	0,003	0	1
Tsku	0,178406	0,00294	0,003	0	1
Coro1a	0,178406	0,00294	0,003	0	1
Plpp4	0,178406	0,00294	0,003	0	1
Hebp2	0,178406	0,00294	0,003	0	1
Pkib	0,178406	0,00294	0,003	0	1
Pawr	0,178406	0,00294	0,003	0	1
Ptprb	0,178406	0,00294	0,003	0	1
Stat6	0,178406	0,00294	0,003	0	1
Gm16230	0,178406	0,00294	0,003	0	1
Col4a2	0,178406	0,00294	0,003	0	1
Jak3	0,178406	0,00294	0,003	0	1
Cdkn3	0,178406	0,00294	0,003	0	1
Rec8	0,178406	0,00294	0,003	0	1
Pdgfd	0,178406	0,00294	0,003	0	1
Fli1	0,178406	0,00294	0,003	0	1
1700110K:	0,178406	0,00294	0,003	0	1
Tcf7	0,178406	0,00294	0,003	0	1
Mycbpap	0,178406	0,00294	0,003	0	1
Plxdc1	0,178406	0,00294	0,003	0	1
Plcd3	0,178406	0,00294	0,003	0	1
Cap2	0,178406	0,00294	0,003	0	1
Golm1	0,178406	0,00294	0,003	0	1
Fbp1	0,178406	0,00294	0,003	0	1
Gm26527	0,178406	0,00294	0,003	0	1
Efcab10	0,178406	0,00294	0,003	0	1
Batf	0,178406	0,00294	0,003	0	1
Pced1b	0,178406	0,00294	0,003	0	1
Gpsm3	0,178406	0,00294	0,003	0	1
H2-Q6	0,178406	0,00294	0,003	0	1
Alk	0,178406	0,00294	0,003	0	1
Ankrd29	0,178406	0,00294	0,003	0	1
Itpr3	0,178406	0,008793	0,003	0	1
Angptl3	0,178406	0,006846	0,003	0	1
Ntng1	0,178406	0,005871	0,003	0	1
Kcnip4	0,178406	0,005871	0,003	0	1
Spry4	0,178406	0,005871	0,003	0	1
Col5a2	0,178406	0,004895	0,003	0	1
P2ry2	0,178406	0,004895	0,003	0	1
Stx11	0,178406	0,004895	0,003	0	1
Gm15222	0,178406	0,004895	0,003	0	1
Kif23	0,178406	0,004895	0,003	0	1
AC153140.	0,178406	0,004895	0,003	0	1
Rgs6	0,178406	0,004895	0,003	0	1
Shank3	0,178406	0,004895	0,003	0	1
Fcgr2b	0,178406	0,003918	0,003	0	1
Meig1	0,178406	0,003918	0,003	0	1
Xlr4a	0,178406	0,003918	0,003	0	1
Hspb7	0,178406	0,003918	0,003	0	1
Gm45894	0,178406	0,003918	0,003	0	1
Shisa2	0,178406	0,003918	0,003	0	1

Zmynd15	0,178406	0,003918	0,003	0	1
1810034E1	0,178406	0,003918	0,003	0	1
Sh3bp1	0,178406	0,003918	0,003	0	1
Vpreb1	0,178406	0,003918	0,003	0	1
Gal3st3	0,178406	0,003918	0,003	0	1
Gldc	0,178406	0,003918	0,003	0	1
Prx	0,178406	0,013645	0,003	0	1
Trank1	0,178406	0,009766	0,003	0	1
Hcn3	0,178406	0,005871	0,003	0	1
Pgam1	0,178645	-0,0222	0,878	0,898	1
Tfcp2	0,178678	-0,02389	0,243	0,273	1
Taf4	0,178742	-0,0232	0,143	0,167	1
Vps37b	0,178761	0,038055	0,18	0,153	1
Scmh1	0,178875	-0,01078	0,294	0,335	1
Rfc2	0,178908	-0,01705	0,614	0,655	1
Tnrc6c	0,178947	0,072955	0,789	0,774	1
Urb1	0,178971	-0,019	0,084	0,104	1
Crk	0,179012	-0,03613	0,644	0,662	1
Paxip1	0,179036	0,043581	0,199	0,176	1
B2m	0,179126	-0,13418	0,722	0,665	1
K230015D(	0,179162	0,009379	0,026	0,016	1
Taf11	0,179174	0,039072	0,43	0,398	1
Coq9	0,179242	-0,00879	0,246	0,281	1
Phf6	0,179257	0,030212	0,223	0,195	1
3110040N1	0,17936	0,0245	0,15	0,127	1
Ttc5	0,179367	-0,0324	0,397	0,429	1
Nr4a3	0,179414	0,034306	0,032	0,021	1
Atg7	0,17945	-0,01167	0,251	0,286	1
Chst12	0,179548	-0,02424	0,261	0,293	1
Prr12	0,179551	-0,03657	0,272	0,299	1
Tapt1	0,179587	-0,03073	0,423	0,462	1
Maf1	0,179595	0,045896	0,612	0,621	1
Aasdhppt	0,179652	-0,03509	0,32	0,35	1
4933415A(	0,179702	-0,00619	0,018	0,028	1
4930481A1	0,179936	-0,009	0,022	0,033	1
Acyp1	0,179974	-0,0197	0,322	0,356	1
Gm20186	0,180002	0,015695	0,032	0,021	1
Prickle2	0,180003	-0,0412	0,417	0,444	1
Slc4a1ap	0,180022	-0,02019	0,249	0,283	1
Msh6	0,180072	-0,02442	0,153	0,179	1
Rev3l	0,180154	-0,03248	0,297	0,328	1
Inhbb	0,18018	0,012333	0,009	0,003	1
Dus1l	0,180184	-0,02874	0,202	0,229	1
Tnfaip1	0,180216	-0,03229	0,206	0,233	1
Sertad4	0,180677	0,014263	0,009	0,003	1
Nlk	0,180723	-0,04411	0,369	0,402	1
Nit1	0,180778	0,046925	0,413	0,395	1
mt-Nd2	0,180812	-0,03015	0,999	1	1
Hist1h1e	0,180857	0,023253	0,174	0,148	1
Tada2a	0,180945	-0,02074	0,311	0,346	1
Cox5b	0,181013	-0,03155	0,973	0,989	1
Psmf1	0,181048	-0,03255	0,25	0,28	1

Fbxl20	0,18115	-0,02664	0,288	0,32	1
Ap1g1	0,181155	-0,0044	0,366	0,407	1
Mettl27	0,181174	0,00749	0,009	0,003	1
Gdf15	0,181174	0,010399	0,009	0,003	1
Tmpo	0,181278	0,040048	0,562	0,537	1
Gm21917	0,181423	0,00749	0,009	0,003	1
Arhgef4	0,181423	0,006519	0,009	0,003	1
A430110L2	0,181423	0,006519	0,009	0,003	1
Gm19710	0,181423	0,006519	0,009	0,003	1
Gm15637	0,181423	0,006519	0,009	0,003	1
Serpina9	0,181423	0,006519	0,009	0,003	1
Gm7276	0,181423	0,006519	0,009	0,003	1
Wdr33	0,181428	-0,00743	0,304	0,341	1
Alkbh1	0,181443	-0,04881	0,279	0,302	1
Dnajb11	0,181455	-0,03848	0,606	0,624	1
Chd3os	0,181464	-0,01589	0,058	0,075	1
Rpl41	0,181536	0,027754	0,998	0,998	1
Esco2	0,181672	0,005547	0,009	0,003	1
Ppp1r14b	0,181722	-0,02462	0,153	0,179	1
1190002N1	0,181841	-0,02993	0,338	0,371	1
Cobll1	0,181846	0,042527	0,503	0,475	1
Vat1l	0,181924	0,016938	0,03	0,02	1
Cep170b	0,181951	-0,03609	0,169	0,193	1
Taok3	0,18204	-0,03842	0,506	0,541	1
Pgbd1	0,18205	0,006841	0,009	0,003	1
Rab28	0,182123	-0,02533	0,312	0,345	1
Gm15441	0,182174	0,007811	0,009	0,003	1
Aifm1	0,182183	-0,02116	0,231	0,262	1
Iws1	0,182282	0,021048	0,444	0,402	1
Gm45159	0,1823	0,005871	0,009	0,003	1
Pcbp3	0,182331	-0,01843	0,079	0,098	1
Camkk2	0,182354	-0,01875	0,038	0,052	1
Tspan32os	0,182425	0,005224	0,009	0,003	1
Ctnnbl1	0,182433	-0,02786	0,235	0,263	1
Rab37	0,182437	-0,08268	0,243	0,268	1
Tsnax	0,182543	-0,01621	0,5	0,554	1
Fem1b	0,182633	0,029291	0,311	0,278	1
Polr3h	0,182646	-0,01733	0,333	0,371	1
1700109H0	0,182694	0,021279	0,061	0,046	1
Cfp	0,182701	-0,0136	0,028	0,041	1
Uck1	0,182963	-0,02164	0,322	0,356	1
Cox7a2l	0,183033	-0,02227	0,785	0,826	1
Tbc1d32	0,183039	0,031296	0,132	0,111	1
Ilkap	0,183041	0,039272	0,592	0,558	1
Zfp428	0,183064	-0,01564	0,112	0,135	1
Chd9	0,183302	-0,05576	0,563	0,572	1
Asb11	0,18343	0,005224	0,009	0,003	1
Adcy9	0,183651	-0,01891	0,131	0,154	1
Bcl2l13	0,183679	0,036932	0,132	0,111	1
Ceacam1	0,183695	0,026578	0,07	0,054	1
Cog8	0,183792	-0,04504	0,241	0,268	1
Iars	0,184045	0,032372	0,571	0,527	1

Mtss1	0,18422	0,038678	0,787	0,758	1
Plcd4	0,184432	-0,0092	0,028	0,041	1
Aff1	0,184482	-0,01727	0,192	0,223	1
Tsn	0,184544	-0,04041	0,842	0,865	1
Arsg	0,184581	-0,03358	0,926	0,941	1
Shd	0,184613	0,009362	0,03	0,02	1
Rab34	0,184661	0,016243	0,036	0,024	1
Kbtbd2	0,184677	-0,01113	0,297	0,335	1
Srsf9	0,184695	-0,02237	0,614	0,66	1
Ralgapb	0,184757	-0,03597	0,387	0,42	1
Tmed7	0,184845	0,043775	0,948	0,938	1
Isca2	0,185083	-0,02059	0,364	0,407	1
Kdm2a	0,185191	0,077441	0,653	0,66	1
Slc30a4	0,18544	0,028012	0,255	0,226	1
Rpp25l	0,185481	-0,03134	0,293	0,324	1
Igsf9b	0,185507	0,046873	0,202	0,177	1
Taf10	0,185569	-0,0333	0,645	0,676	1
Cep76	0,185822	-0,0191	0,057	0,073	1
Phf1	0,185838	-0,01694	0,22	0,249	1
Stam2	0,186084	-0,02219	0,392	0,428	1
Gemin5	0,186473	-0,02731	0,118	0,14	1
Dhx33	0,186523	-0,01593	0,076	0,094	1
Cyp4f17	0,186542	-0,01248	0,025	0,036	1
Kat14	0,186783	0,033702	0,193	0,169	1
Tcirg1	0,186785	0,049484	0,297	0,275	1
Mmachc	0,186788	-0,01078	0,045	0,06	1
Pde4b	0,186842	-0,02134	0,964	0,974	1
Erich1	0,187044	0,038312	0,186	0,163	1
Dnajc15	0,187079	-0,03988	0,487	0,515	1
Parp4	0,187085	-0,03671	0,265	0,294	1
Kcna1	0,187138	0,042602	0,903	0,878	1
1810022K(	0,187265	-0,03655	0,625	0,654	1
Nup50	0,187266	-0,00965	0,301	0,34	1
Mdk	0,187319	0,01134	0,016	0,008	1
Adgra2	0,187319	0,010377	0,016	0,008	1
Aldoa	0,187445	0,033809	0,996	1	1
Uchl5	0,187449	-0,01533	0,275	0,307	1
Depdc7	0,18751	-0,00987	0,288	0,325	1
Gdi2	0,187602	-0,03437	0,909	0,911	1
Ankrd23	0,1877	-0,01142	0,013	0,021	1
Gm45605	0,187745	0,020362	0,057	0,042	1
Mybl2	0,187781	0,010377	0,016	0,008	1
Mapk1	0,187821	-0,01771	0,644	0,678	1
Ttll3	0,187851	-0,01878	0,084	0,104	1
Galnt11	0,187858	-0,02791	0,316	0,346	1
Gfod2	0,187865	0,022161	0,112	0,135	1
Elp2	0,187902	0,027222	0,271	0,239	1
Grasp	0,187939	0,041243	0,175	0,151	1
Med28	0,188054	-0,01992	0,601	0,647	1
Terf2ip	0,188196	-0,01377	0,37	0,4	1
Baz1b	0,188235	-0,04397	0,661	0,683	1
Rbm11	0,188237	-0,01314	0,067	0,085	1

Bcat2	0,188276	-0,02292	0,146	0,171	1
Trim28	0,188308	0,04754	0,533	0,511	1
Pdk4	0,188421	-0,01116	0,174	0,202	1
Nop2	0,188502	0,019154	0,12	0,098	1
H3f3aos	0,188715	0,009728	0,016	0,008	1
Gm17135	0,188719	0,010042	0,016	0,008	1
Zfp677	0,188724	-0,01272	0,079	0,099	1
Tubb5	0,188824	-0,02308	0,71	0,743	1
Gm42788	0,188906	0,003076	0,048	0,034	1
9430065F1	0,189147	-0,01762	0,066	0,083	1
Sord	0,189256	-0,02923	0,117	0,138	1
Btbd2	0,189329	-0,01541	0,211	0,241	1
Rab40b	0,189387	-0,01188	0,038	0,052	1
Bex3	0,189619	0,026309	0,274	0,244	1
Osgep	0,189636	-0,02407	0,46	0,506	1
Carhsp1	0,18975	-0,04909	0,97	0,985	1
Rs1	0,18981	-0,01465	0,021	0,031	1
Mok	0,189884	-0,00886	0,013	0,021	1
Ubr2	0,189912	0,049942	0,783	0,769	1
St3gal1	0,190092	0,030742	0,106	0,086	1
Trp53bp2	0,190375	0,040411	0,809	0,785	1
Actn1	0,190478	-0,02155	0,092	0,112	1
Dcaf17	0,190517	-0,07947	0,8	0,803	1
Osbpl5	0,190587	-0,01735	0,136	0,161	1
Actr5	0,190685	-0,01666	0,157	0,184	1
Vcpip1	0,190693	-0,01048	0,376	0,418	1
Rgs10	0,1907	-0,00671	0,008	0,015	1
Srp54b	0,1907	-0,00671	0,008	0,015	1
Gm10373	0,190702	-0,00893	0,008	0,015	1
Ribc2	0,190702	-0,00893	0,008	0,015	1
Cenpm	0,190734	-0,01327	0,013	0,021	1
Cd300lf	0,190806	-0,00734	0,008	0,015	1
Snx30	0,19085	-0,01785	0,807	0,844	1
Rnaseh2b	0,190867	-0,02707	0,242	0,27	1
Ppp2r5d	0,190876	-0,01488	0,222	0,252	1
Adamts5	0,190913	-0,00796	0,008	0,015	1
Mterf1b	0,190961	-0,01379	0,021	0,031	1
Dvl1	0,191062	0,038815	0,324	0,299	1
Phkg2	0,1911	-0,0378	0,335	0,359	1
Sdhd	0,191339	-0,01497	0,45	0,499	1
Ints10	0,191468	-0,01066	0,224	0,255	1
Map1lc3b	0,191492	-0,02321	0,971	0,984	1
Caprin1	0,191572	-0,02464	0,721	0,746	1
Cdk20	0,191574	0,023273	0,051	0,037	1
Nefl	0,191642	-0,00573	0,008	0,015	1
Cntn2	0,191747	0,045967	0,995	0,998	1
Chkb	0,191814	-0,00952	0,297	0,338	1
Atf5	0,191886	0,021679	0,257	0,233	1
Aamdc	0,191913	-0,02168	0,722	0,779	1
4930426I2	0,192035	0,031235	0,075	0,059	1
Hmgcl	0,192053	-0,02868	0,642	0,676	1
Zfp606	0,192062	-0,04026	0,216	0,241	1

Fam181b	0,192087	-0,0063	0,013	0,021	1
Cep112	0,192132	0,03365	0,047	0,034	1
Rpl4	0,192142	0,035856	0,983	0,98	1
Ctsh	0,192286	-0,00804	0,025	0,036	1
Rnf166	0,192347	0,0352	0,172	0,148	1
Pmepa1	0,192363	0,031939	0,269	0,239	1
Gm47512	0,192544	-0,00791	0,031	0,044	1
Rdh12	0,192582	-0,01889	0,095	0,115	1
Thap8	0,192671	-0,01134	0,017	0,026	1
Lrrtm3	0,192678	-0,01406	0,057	0,073	1
P3h1	0,192699	0,013285	0,057	0,042	1
Ppid	0,192873	-0,04643	0,617	0,639	1
Gtf3c6	0,192873	-0,02437	0,426	0,46	1
Ppp1r13b	0,192891	0,044049	0,277	0,252	1
8030462N	0,193327	0,0228	0,076	0,059	1
Gpt2	0,193349	-0,01918	0,275	0,306	1
Gclc	0,193374	-0,03067	0,426	0,459	1
Rpap2	0,193381	-0,02049	0,155	0,18	1
Tubgcp2	0,193434	0,047154	0,204	0,18	1
Med8	0,193595	-0,03015	0,428	0,462	1
Peli1	0,193679	0,037816	0,55	0,511	1
Trf	0,193849	-0,03373	1	1	1
Ap1b1	0,194018	-0,01867	0,219	0,25	1
Mrpl49	0,194232	-0,03028	0,259	0,286	1
Fn3krp	0,194305	-0,01855	0,179	0,207	1
Srsf7	0,194432	0,036253	0,681	0,646	1
Fundc1	0,194501	-0,02633	0,399	0,439	1
Tor1aip2	0,194599	-0,02419	0,521	0,551	1
Naa20	0,194601	-0,03636	0,407	0,429	1
Slc1a3	0,194638	-0,05166	0,508	0,53	1
Osbpl11	0,194647	-0,02661	0,195	0,223	1
Exoc6	0,194703	-0,01593	0,156	0,182	1
AC146911.	0,194723	0,012659	0,059	0,044	1
Ranbp10	0,194751	-0,03341	0,219	0,244	1
Oxsm	0,194811	-0,01427	0,078	0,096	1
Gm996	0,194875	0,030883	0,132	0,111	1
P4hb	0,194896	0,057988	0,847	0,862	1
Ntm	0,195041	-0,01003	0,017	0,026	1
Lgals1	0,195061	-0,01421	0,021	0,031	1
Klhl36	0,195112	-0,00872	0,021	0,031	1
E530011L2	0,195146	-0,01052	0,041	0,055	1
Plk3	0,195204	0,061964	0,346	0,325	1
Nktr	0,195285	-0,03964	0,912	0,937	1
Soga1	0,19535	-0,02153	0,234	0,263	1
Pcyox1	0,195429	-0,0166	0,465	0,511	1
Sap18	0,195438	0,036772	0,704	0,689	1
Epm2aip1	0,19552	-0,04199	0,442	0,462	1
Sesn1	0,196075	-0,02847	0,201	0,228	1
Sorbs1	0,196107	-0,02794	0,154	0,177	1
Prep	0,196128	-0,01697	0,083	0,102	1
Tcp1	0,196187	0,04287	0,783	0,784	1
Dusp28	0,196264	-0,02348	0,134	0,158	1

Sema3e	0,196313	-0,03097	0,03	0,042	1
Desi1	0,19633	-0,04005	0,971	0,966	1
Zscan12	0,196381	-0,01687	0,08	0,099	1
Zfp335	0,196703	-0,01933	0,078	0,096	1
Atp7a	0,196782	-0,02067	0,171	0,197	1
Mocs1	0,19686	-0,02565	0,113	0,133	1
Narfl	0,197029	-0,03775	0,226	0,25	1
S100a4	0,197102	0,016443	0,018	0,01	1
BC065403	0,197102	0,01069	0,018	0,01	1
Haus1	0,197132	0,024168	0,155	0,132	1
Btg3	0,197134	0,02521	0,045	0,033	1
Lnpep	0,197149	-0,04173	0,626	0,646	1
Zfp715	0,197203	0,017753	0,12	0,099	1
Wwtr1	0,197288	0,012271	0,018	0,01	1
Emb	0,197641	0,008765	0,018	0,01	1
Gphn	0,197665	-0,04363	0,565	0,587	1
Angptl6	0,197814	-0,01913	0,15	0,176	1
Cers5	0,197855	0,036125	0,365	0,335	1
Serpini1	0,197876	-0,005	0,061	0,078	1
Eif4a3	0,197982	0,042043	0,722	0,706	1
Rab7b	0,19806	0,041589	0,16	0,138	1
Gm20515	0,198173	-0,03174	0,084	0,102	1
Adal	0,198238	-0,03375	0,123	0,145	1
Elavl1	0,198319	-0,04184	0,608	0,628	1
Pgrmc1	0,198393	0,042036	0,725	0,706	1
Cdc73	0,198398	-0,0258	0,401	0,442	1
Sntg1	0,198461	-0,00162	0	0,002	1
Kcnb2	0,198461	-0,00162	0	0,002	1
Sbspon	0,198461	-0,00162	0	0,002	1
Fhl2	0,198461	-0,00162	0	0,002	1
Dpp10	0,198461	-0,00162	0	0,002	1
Kcnt2	0,198461	-0,00162	0	0,002	1
Hlx	0,198461	-0,00162	0	0,002	1
Batf3	0,198461	-0,00162	0	0,002	1
Camk1g	0,198461	-0,00162	0	0,002	1
Fam69b	0,198461	-0,00162	0	0,002	1
Fam163b	0,198461	-0,00162	0	0,002	1
Slc43a3	0,198461	-0,00162	0	0,002	1
D430041D	0,198461	-0,00162	0	0,002	1
Cd93	0,198461	-0,00162	0	0,002	1
Scrt2	0,198461	-0,00162	0	0,002	1
Hck	0,198461	-0,00162	0	0,002	1
Procr	0,198461	-0,00162	0	0,002	1
Myl9	0,198461	-0,00162	0	0,002	1
Rims4	0,198461	-0,00162	0	0,002	1
Gm27032	0,198461	-0,00162	0	0,002	1
Gpr34	0,198461	-0,00162	0	0,002	1
Akap14	0,198461	-0,00162	0	0,002	1
Tenm1	0,198461	-0,00162	0	0,002	1
Slitrk4	0,198461	-0,00162	0	0,002	1
Atp2b3	0,198461	-0,00162	0	0,002	1
Slc7a3	0,198461	-0,00162	0	0,002	1

Mum1l1	0,198461	-0,00162	0	0,002	1
Usp51	0,198461	-0,00162	0	0,002	1
Gm15261	0,198461	-0,00162	0	0,002	1
AI849053	0,198461	-0,00162	0	0,002	1
Slc6a17	0,198461	-0,00162	0	0,002	1
Npnt	0,198461	-0,00162	0	0,002	1
Dnaic1	0,198461	-0,00162	0	0,002	1
Zfp618	0,198461	-0,00162	0	0,002	1
Hacd4	0,198461	-0,00162	0	0,002	1
Kank4	0,198461	-0,00162	0	0,002	1
AB041806	0,198461	-0,00162	0	0,002	1
Vwa5b1	0,198461	-0,00162	0	0,002	1
Drc1	0,198461	-0,00162	0	0,002	1
Evc2	0,198461	-0,00162	0	0,002	1
Rbm47	0,198461	-0,00162	0	0,002	1
Adamts3	0,198461	-0,00162	0	0,002	1
Grk3	0,198461	-0,00162	0	0,002	1
Iqcd	0,198461	-0,00162	0	0,002	1
Rimbp2	0,198461	-0,00162	0	0,002	1
Lrch4	0,198461	-0,00162	0	0,002	1
Gpr12	0,198461	-0,00162	0	0,002	1
Clec5a	0,198461	-0,00162	0	0,002	1
Jazf1	0,198461	-0,00162	0	0,002	1
Atoh8	0,198461	-0,00162	0	0,002	1
Nat8	0,198461	-0,00162	0	0,002	1
Tmem40	0,198461	-0,00162	0	0,002	1
2310001H	0,198461	-0,00162	0	0,002	1
Grin2b	0,198461	-0,00162	0	0,002	1
Mill2	0,198461	-0,00162	0	0,002	1
Lrfn1	0,198461	-0,00162	0	0,002	1
Cd33	0,198461	-0,00162	0	0,002	1
Cd37	0,198461	-0,00162	0	0,002	1
Fes	0,198461	-0,00162	0	0,002	1
Trim5	0,198461	-0,00162	0	0,002	1
Dkk3	0,198461	-0,00162	0	0,002	1
Sox6os	0,198461	-0,00162	0	0,002	1
B4galnt4	0,198461	-0,00162	0	0,002	1
Tnfrsf26	0,198461	-0,00162	0	0,002	1
Clvs2	0,198461	-0,00162	0	0,002	1
Fam26f	0,198461	-0,00162	0	0,002	1
Prdm1	0,198461	-0,00162	0	0,002	1
Lrrc3	0,198461	-0,00162	0	0,002	1
Icosl	0,198461	-0,00162	0	0,002	1
Mybpc1	0,198461	-0,00162	0	0,002	1
Kcnc2	0,198461	-0,00162	0	0,002	1
Gm36325	0,198461	-0,00162	0	0,002	1
Cpne7	0,198461	-0,00162	0	0,002	1
Gm3468	0,198461	-0,00162	0	0,002	1
Rarb	0,198461	-0,00162	0	0,002	1
Cfap70	0,198461	-0,00162	0	0,002	1
1700024G	0,198461	-0,00162	0	0,002	1
Nrg3	0,198461	-0,00162	0	0,002	1

Dydc2	0,198461	-0,00162	0	0,002	1
Slitrk6	0,198461	-0,00162	0	0,002	1
Gpr183	0,198461	-0,00162	0	0,002	1
Il10ra	0,198461	-0,00162	0	0,002	1
Nrg4	0,198461	-0,00162	0	0,002	1
Calml4	0,198461	-0,00162	0	0,002	1
Car12	0,198461	-0,00162	0	0,002	1
Ripply2	0,198461	-0,00162	0	0,002	1
AF529169	0,198461	-0,00162	0	0,002	1
Cpne4	0,198461	-0,00162	0	0,002	1
Bsn	0,198461	-0,00162	0	0,002	1
Prss50	0,198461	-0,00162	0	0,002	1
Slit3	0,198461	-0,00162	0	0,002	1
Nlrp3	0,198461	-0,00162	0	0,002	1
Cfap52	0,198461	-0,00162	0	0,002	1
Pik3r6	0,198461	-0,00162	0	0,002	1
Pitpnm3	0,198461	-0,00162	0	0,002	1
Asic2	0,198461	-0,00162	0	0,002	1
Slfn8	0,198461	-0,00162	0	0,002	1
1700023F(	0,198461	-0,00162	0	0,002	1
Arhgap27o	0,198461	-0,00162	0	0,002	1
Gm20554	0,198461	-0,00162	0	0,002	1
Naip5	0,198461	-0,00162	0	0,002	1
Gm9866	0,198461	-0,00162	0	0,002	1
Mirg	0,198461	-0,00162	0	0,002	1
Kif26a	0,198461	-0,00162	0	0,002	1
Mal2	0,198461	-0,00162	0	0,002	1
Lrrc6	0,198461	-0,00162	0	0,002	1
Ncf4	0,198461	-0,00162	0	0,002	1
Nfam1	0,198461	-0,00162	0	0,002	1
Tuba1c	0,198461	-0,00162	0	0,002	1
Snai2	0,198461	-0,00162	0	0,002	1
Hrasls	0,198461	-0,00162	0	0,002	1
Maats1	0,198461	-0,00162	0	0,002	1
Cd200r1	0,198461	-0,00162	0	0,002	1
Mir155hg	0,198461	-0,00162	0	0,002	1
Sh3bgr	0,198461	-0,00162	0	0,002	1
Pde9a	0,198461	-0,00162	0	0,002	1
Ptk7	0,198461	-0,00162	0	0,002	1
Gm27217	0,198461	-0,00162	0	0,002	1
Kcnh8	0,198461	-0,00162	0	0,002	1
Tgif1	0,198461	-0,00162	0	0,002	1
Galnt14	0,198461	-0,00162	0	0,002	1
Epcam	0,198461	-0,00162	0	0,002	1
Gm10308	0,198461	-0,00162	0	0,002	1
Dsg2	0,198461	-0,00162	0	0,002	1
Pcdha11	0,198461	-0,00162	0	0,002	1
Prelid2	0,198461	-0,00162	0	0,002	1
Ppargc1b	0,198461	-0,00162	0	0,002	1
Syt12	0,198461	-0,00162	0	0,002	1
Slit1	0,198461	-0,00162	0	0,002	1
Neurl3	0,198461	-0,00325	0	0,002	1

Itga6	0,198461	-0,00325	0	0,002	1
Trpc5	0,198461	-0,00325	0	0,002	1
Gm42517	0,198461	-0,00325	0	0,002	1
Tpbgl	0,198461	-0,00325	0	0,002	1
Fam26e	0,198461	-0,00325	0	0,002	1
Gm45472	0,198461	-0,00325	0	0,002	1
Slc7a7	0,198461	-0,00325	0	0,002	1
Klhl1	0,198461	-0,00325	0	0,002	1
Acod1	0,198461	-0,00325	0	0,002	1
Cyb561	0,198461	-0,00325	0	0,002	1
Clec14a	0,198461	-0,00325	0	0,002	1
Tnf	0,198461	-0,00325	0	0,002	1
St6gal2	0,198461	-0,00325	0	0,002	1
Car6	0,198461	-0,00487	0	0,002	1
Foxp2	0,198461	-0,0081	0	0,002	1
Hikeshi	0,198498	-0,02851	0,435	0,465	1
Mapre1	0,1988	0,047335	0,745	0,717	1
Grm3	0,198826	-0,05763	0,56	0,577	1
Mbd3	0,198845	0,026994	0,648	0,61	1
Ufd1	0,198853	0,034547	0,586	0,567	1
H2afv	0,198984	-0,04016	0,649	0,683	1
Mfsd8	0,199213	-0,01881	0,258	0,289	1
Nxt2	0,19922	-0,02899	0,666	0,706	1
Spop	0,199313	-0,02527	0,774	0,8	1
Gm10069	0,199361	-0,00745	0,041	0,055	1
Prepl	0,199388	-0,01802	0,174	0,2	1
Rps14	0,199397	0,024835	0,997	0,998	1
Hes6	0,199447	-0,02808	0,13	0,151	1
Ciapin1	0,199688	-0,0233	0,367	0,402	1
9930021J(	0,199705	-0,01636	0,581	0,611	1
Rhobtb2	0,199782	-0,0236	0,127	0,148	1
Tmem28	0,200061	-0,01613	0,116	0,138	1
Gas2	0,200237	-0,02525	0,151	0,174	1
Mob1a	0,200271	-0,0166	0,258	0,293	1
Fam83d	0,200272	0,000866	0,025	0,036	1
Klhdc2	0,200341	-0,02888	0,566	0,593	1
3010003L2	0,200503	-0,00764	0,027	0,039	1
Cpa2	0,200514	0,028975	0,112	0,093	1
Cinp	0,200521	0,045139	0,295	0,272	1
Ddx39	0,200557	-0,02996	0,165	0,187	1
Cep44	0,200613	-0,03076	0,237	0,265	1
Rbm14	0,200694	-0,01692	0,213	0,242	1
Setd5	0,200854	0,084922	0,658	0,655	1
Slc22a7	0,200881	0,010083	0,006	0,002	1
Gpr17	0,200917	0,00077	0,018	0,01	1
Tmem43	0,200954	-0,01654	0,217	0,247	1
Gm42997	0,201049	0,007169	0,006	0,002	1
Car7	0,201049	0,007169	0,006	0,002	1
Nhs	0,20105	0,009112	0,006	0,002	1
Mbnl1	0,201117	-0,03559	0,914	0,915	1
Ophn1	0,201218	0,006195	0,006	0,002	1
Gm26784	0,201218	0,006195	0,006	0,002	1

Oacyl	0,201218	0,006195	0,006	0,002	1
Mocos	0,201218	0,009112	0,006	0,002	1
Wnk2	0,201218	0,007169	0,006	0,002	1
Tbc1d24	0,201306	0,046018	0,204	0,182	1
Nxpe3	0,20134	0,02699	0,218	0,192	1
Tmem19	0,201344	-0,02282	0,49	0,525	1
Pigr	0,201386	0,005221	0,006	0,002	1
Cd302	0,201386	0,005221	0,006	0,002	1
Gm26615	0,201386	0,005221	0,006	0,002	1
Draxin	0,201386	0,005221	0,006	0,002	1
Styxl1	0,201386	0,005221	0,006	0,002	1
Nr2f2	0,201386	0,005221	0,006	0,002	1
Ttll13	0,201386	0,005221	0,006	0,002	1
4930405J1	0,201386	0,005221	0,006	0,002	1
Cdca2	0,201386	0,005221	0,006	0,002	1
Gm37401	0,201386	0,005221	0,006	0,002	1
9230116L(	0,201386	0,005221	0,006	0,002	1
Gm10269	0,201386	0,005221	0,006	0,002	1
Pnisr	0,201512	-0,01763	0,959	0,966	1
Ccdc66	0,20155	-0,02455	0,367	0,403	1
Rgs1	0,201554	0,004246	0,006	0,002	1
Nek6	0,201554	0,004246	0,006	0,002	1
Gm37640	0,201554	0,004246	0,006	0,002	1
Gm26745	0,201554	0,004246	0,006	0,002	1
Ica1	0,201554	0,004246	0,006	0,002	1
Gpr162	0,201554	0,004246	0,006	0,002	1
Ccdc8	0,201554	0,004246	0,006	0,002	1
4932443L1	0,201554	0,004246	0,006	0,002	1
Gm45359	0,201554	0,004246	0,006	0,002	1
4933405L1	0,201554	0,004246	0,006	0,002	1
Gm39302	0,201554	0,004246	0,006	0,002	1
Trim43a	0,201554	0,004246	0,006	0,002	1
1700102P(	0,201554	0,004246	0,006	0,002	1
Psme2b	0,201554	0,004246	0,006	0,002	1
Cd300lg	0,201554	0,004246	0,006	0,002	1
Gm48250	0,201554	0,004246	0,006	0,002	1
Gm47849	0,201554	0,004246	0,006	0,002	1
Gm28989	0,201554	0,004246	0,006	0,002	1
Cthrc1	0,201554	0,004246	0,006	0,002	1
C030010L1	0,201554	0,004246	0,006	0,002	1
Nme4	0,201554	0,004246	0,006	0,002	1
Naif1	0,20158	-0,01561	0,024	0,034	1
Icmt	0,201762	-0,01377	0,289	0,324	1
Cyb5r3	0,201812	0,055646	0,318	0,298	1
AW209491	0,201876	0,024579	0,144	0,122	1
Cep57l1	0,201882	0,040815	0,209	0,185	1
Smarca5	0,201937	-0,01061	0,888	0,925	1
Aqp5	0,202232	0,006841	0,006	0,002	1
Uhrf1	0,202401	0,003599	0,006	0,002	1
Casp8ap2	0,202532	-0,02252	0,342	0,377	1
Aspm	0,20257	0,002624	0,006	0,002	1
Sigmar1	0,202662	-0,02794	0,207	0,233	1

1810032O(	0,202696	0,018958	0,093	0,075	1
Kctd14	0,202735	0,018293	0,02	0,011	1
Kif3c	0,20279	-0,03374	0,535	0,556	1
Hprt	0,202819	0,017785	0,254	0,224	1
Pdia3	0,203048	0,038599	0,956	0,954	1
Hps1	0,203064	-0,01466	0,089	0,109	1
Slc6a8	0,203156	0,062041	0,644	0,633	1
Zfp959	0,203176	-0,01493	0,075	0,093	1
Car5b	0,203266	-0,01248	0,024	0,034	1
Rpf2	0,203465	-0,03167	0,217	0,244	1
Mtrr	0,203537	-0,02187	0,141	0,164	1
Mllt6	0,203557	0,033149	0,451	0,42	1
Supt6	0,203647	0,039515	0,611	0,58	1
Paxx	0,20368	0,027546	0,605	0,566	1
Rlim	0,20378	-0,02494	0,5	0,528	1
Micu2	0,203783	0,040883	0,501	0,48	1
Blcap	0,203875	-0,02489	0,297	0,328	1
Csgalnact2	0,204077	-0,01545	0,19	0,218	1
Slc37a4	0,204079	-0,0284	0,159	0,182	1
Scfd1	0,204133	0,045488	0,442	0,416	1
Ltbr	0,204134	-0,01293	0,148	0,174	1
Atp8b1	0,204207	0,021646	0,244	0,218	1
Recql	0,20421	-0,03027	0,187	0,211	1
Gm10277	0,204236	-0,01609	0,056	0,072	1
Akt1	0,204315	0,0355	0,441	0,408	1
Edil3	0,204546	-0,01064	0,998	1	1
Trappc6a	0,204567	-0,01559	0,304	0,34	1
Nedd4l	0,20463	0,028078	0,354	0,317	1
Fam13a	0,204814	-0,02488	0,049	0,063	1
Zfp964	0,204822	0,010355	0,02	0,011	1
Atp11a	0,20487	-0,02722	0,83	0,855	1
Kcnrg	0,204883	-0,0206	0,043	0,057	1
Gm32926	0,204895	-0,01091	0,024	0,034	1
Dnmt3a	0,205	0,042058	0,631	0,611	1
Pomgnt1	0,205046	0,041391	0,513	0,489	1
Ptpn4	0,205116	-0,03586	0,221	0,246	1
Ppp2r1a	0,205126	-0,03971	0,854	0,859	1
Pcdhb14	0,205251	0,042987	0,097	0,08	1
Dohh	0,205252	-0,02662	0,416	0,455	1
Pdxk	0,205288	0,046656	0,307	0,281	1
Capn12	0,205346	0,010355	0,02	0,011	1
Arid3b	0,205426	0,017956	0,079	0,062	1
Mri1	0,205477	0,018399	0,139	0,117	1
Rpap1	0,20555	-0,00953	0,147	0,171	1
Wdfy1	0,205654	-0,00788	0,446	0,491	1
Tspan4	0,205731	-0,01556	0,03	0,042	1
Washc2	0,20583	0,044538	0,91	0,872	1
Gm26944	0,206047	0,008436	0,02	0,011	1
AC174678.	0,206181	-0,01045	0,012	0,02	1
Ube2k	0,206182	-0,03866	0,677	0,688	1
Rabep2	0,206421	-0,01861	0,152	0,177	1
Gtf2h3	0,206501	-0,01642	0,148	0,172	1

Tmem108	0,206509	-0,03243	0,342	0,374	1
Camsap1	0,206529	-0,0212	0,278	0,309	1
Sun2	0,206537	0,061441	0,354	0,335	1
Krtcap2	0,206657	-0,03495	0,821	0,821	1
Atp1b3	0,206671	0,057911	0,994	0,997	1
Pcdhga10	0,206721	-0,02039	0,043	0,057	1
Tusc1	0,206747	0,020362	0,06	0,046	1
Mcm8	0,206925	-0,0264	0,096	0,115	1
7630403G1	0,207095	0,010713	0,011	0,005	1
Kctd12	0,207095	0,012644	0,011	0,005	1
Ifnar2	0,207162	-0,02495	0,374	0,413	1
Plscr3	0,207232	0,020235	0,146	0,124	1
Gm17705	0,207276	0,006513	0,02	0,011	1
Grid2	0,207402	-0,02074	0,298	0,333	1
Dhx30	0,207409	-0,01671	0,484	0,524	1
Slc16a10	0,207461	0,011679	0,011	0,005	1
Ythdc2	0,207556	-0,03808	0,321	0,348	1
Ube4b	0,207676	-0,0137	0,421	0,463	1
C1qa	0,207769	0,015852	0,047	0,034	1
Gm26563	0,207812	0,006832	0,02	0,011	1
Speer4b	0,207827	0,008779	0,011	0,005	1
Lgr5	0,207827	0,009747	0,011	0,005	1
Angptl2	0,207829	0,011991	0,011	0,005	1
Rcn1	0,207855	-0,00671	0,007	0,013	1
Mblac2	0,207915	-0,04167	0,295	0,324	1
Gm13403	0,208109	-0,01296	0,083	0,102	1
Clasp1	0,208128	-0,02508	0,19	0,218	1
Sft2d3	0,208163	-0,03251	0,371	0,402	1
Rps8	0,208175	0,034812	1	1	1
Ropn1l	0,208194	0,007811	0,011	0,005	1
Gas1	0,208194	0,009747	0,011	0,005	1
Vsig10	0,208199	0,016243	0,041	0,029	1
Pskh1	0,208309	-0,02292	0,142	0,166	1
H2-M5	0,208441	0,009097	0,011	0,005	1
Rfxap	0,208506	-0,02201	0,359	0,392	1
Hdac5	0,208541	0,046267	0,724	0,704	1
Rbm8a2	0,20856	0,006841	0,011	0,005	1
Morn5	0,20856	0,006841	0,011	0,005	1
Olfr539	0,20856	0,006841	0,011	0,005	1
Gabrb1	0,208682	-0,0051	0,007	0,013	1
Fmr1	0,208712	0,049574	0,632	0,603	1
Cbll1	0,208789	0,028627	0,358	0,327	1
Ano1	0,208803	-0,00573	0,007	0,013	1
Mcm4	0,208902	0,032468	0,206	0,182	1
Agbl2	0,208927	0,005871	0,011	0,005	1
Mansc1	0,208927	0,005871	0,011	0,005	1
Gm12324	0,20895	-0,02188	0,052	0,067	1
Lrrc8a	0,209171	0,03324	0,182	0,159	1
2510017J1	0,209211	0,022789	0,039	0,028	1
Cox15	0,209259	-0,01593	0,156	0,18	1
Fgf22	0,209322	-0,01997	0,132	0,154	1
Sptssa	0,209524	-0,01679	0,852	0,872	1

Olfr639	0,209545	0,006194	0,011	0,005	1
Cgref1	0,209634	-0,00122	0,007	0,013	1
Pias3	0,209644	-0,02495	0,182	0,208	1
Prkar2b	0,209668	0,010978	0,022	0,013	1
Cry2	0,209705	0,045847	0,286	0,263	1
Rpl22	0,20971	0,037409	0,974	0,969	1
Plekhb2	0,209925	0,021138	0,364	0,33	1
Zfp703	0,210053	0,037711	0,099	0,081	1
Zfp469	0,210166	0,012271	0,022	0,013	1
Fam205a1	0,210166	0,009396	0,022	0,013	1
Ints11	0,210179	0,019045	0,198	0,172	1
Atp6v0a2	0,210187	-0,04236	0,499	0,525	1
Atxn1	0,210289	0,064323	0,327	0,301	1
Guk1	0,210299	-0,02375	0,636	0,676	1
Ppp1r9b	0,21033	-0,04446	0,445	0,467	1
Diaph3	0,210433	-0,00811	0,02	0,029	1
Chchd7	0,21055	0,059701	0,403	0,39	1
Spc24	0,210596	0,010667	0,022	0,013	1
Ccl22	0,210681	0,00971	0,022	0,013	1
Slc9a3r1	0,210854	-0,0473	0,256	0,281	1
Msl2	0,210881	-0,01937	0,376	0,403	1
Arhgef18	0,210947	-0,02383	0,146	0,169	1
Hist1h1c	0,210997	0,002822	0,184	0,211	1
Pcdhgc3	0,211035	-0,01134	0,016	0,024	1
Wars	0,211161	-0,03062	0,32	0,351	1
Hace1	0,211281	-0,04175	0,247	0,27	1
Rnf31	0,211406	0,021796	0,204	0,177	1
Nupr1l	0,211443	0,008108	0,022	0,013	1
2510039O	0,211529	-0,00525	0,343	0,384	1
Crmp1	0,211826	-0,01158	0,075	0,093	1
4931428F(	0,21185	0,015669	0,064	0,049	1
Cdc42bpb	0,211867	-0,04525	0,348	0,377	1
Dstyk	0,211948	-0,04307	0,761	0,774	1
Psmc1	0,212028	-0,00616	0,782	0,818	1
Fasn	0,212046	0,036432	0,874	0,873	1
Ubap1	0,212095	-0,03445	0,409	0,434	1
Trim65	0,212166	-0,01174	0,072	0,089	1
Fam213a	0,212254	0,05197	0,662	0,67	1
Gxylt1	0,212313	0,053217	0,349	0,324	1
Ppil2	0,212447	-0,02003	0,561	0,593	1
Tbc1d8b	0,212508	-0,01968	0,055	0,07	1
Crybg2	0,212841	0,020997	0,056	0,042	1
Zfp446	0,21303	-0,01053	0,122	0,145	1
Gde1	0,213034	-0,04586	0,918	0,924	1
1110002O(	0,213237	0,00715	0,022	0,013	1
Usp9x	0,213537	-0,00864	0,855	0,87	1
Mrps5	0,213609	0,031498	0,461	0,436	1
Nhsl1	0,213676	-0,01467	0,043	0,057	1
Kndc1	0,213968	-0,04559	0,822	0,815	1
Cdk2ap2	0,214066	-0,03205	0,428	0,463	1
Kazn	0,214107	0,05331	0,92	0,907	1
Tmem199	0,214131	-0,02217	0,37	0,403	1

Nudt4	0,214194	-0,04256	0,669	0,699	1
Speer4a	0,214277	-0,01383	0,043	0,057	1
Gm26609	0,214479	0,020994	0,033	0,023	1
H2-Q4	0,214567	-0,14444	0,081	0,098	1
Slc1a5	0,214602	0,012722	0,041	0,029	1
Atox1	0,214648	-0,01814	0,79	0,824	1
Morc4	0,214785	-0,01388	0,068	0,085	1
Ccdc47	0,214887	-0,01277	0,916	0,911	1
Klhl20	0,214934	-0,00982	0,187	0,216	1
Hemk1	0,214948	-0,02324	0,092	0,111	1
Mto1	0,215069	-0,02319	0,184	0,208	1
Gm26682	0,215202	-0,00508	0,03	0,042	1
A930028N(	0,215297	-0,01147	0,059	0,075	1
Spata6	0,215404	-0,01628	0,105	0,125	1
Ramp1	0,215405	-0,01102	0,033	0,046	1
Rpl9-ps6	0,215493	0,011211	0,041	0,029	1
Stx16	0,215538	0,035666	0,619	0,587	1
Cbr1	0,215552	-0,02787	0,269	0,298	1
Gfod1	0,215648	-0,0301	0,117	0,137	1
Ccnl2	0,215942	-0,0003	0,772	0,803	1
Malt1	0,216001	-0,02319	0,16	0,182	1
Creg2	0,21605	-0,02127	0,166	0,19	1
Sp2	0,216142	0,035318	0,255	0,231	1
Fam53c	0,216163	-0,02841	0,324	0,354	1
B3gnt9	0,216246	0,037394	0,423	0,397	1
Map2k4	0,216429	0,024	0,281	0,252	1
Srsf10	0,216449	0,024229	0,63	0,585	1
Xlr3b	0,216527	0,01505	0,026	0,016	1
Ivns1abp	0,216535	-0,01017	0,455	0,501	1
Gid4	0,216738	0,02887	0,395	0,359	1
Mthfd2l	0,217025	0,027947	0,101	0,083	1
Ipo4	0,217229	-0,01883	0,169	0,193	1
Ppard	0,217234	-0,02719	0,241	0,268	1
Satb1	0,217346	-0,0287	0,115	0,135	1
Zfp113	0,217384	-0,01558	0,161	0,185	1
Rab8a	0,217447	-0,02774	0,381	0,413	1
Zfp644	0,217516	-0,02027	0,71	0,715	1
Slco2b1	0,217518	0,014326	0,037	0,026	1
Galk2	0,21754	-0,02096	0,116	0,137	1
Fbxo6	0,217565	0,027611	0,181	0,158	1
Gabbr1	0,217566	0,03141	0,974	0,971	1
Gtf2f2	0,217628	-0,02739	0,283	0,307	1
Pank1	0,217649	-0,01526	0,052	0,067	1
Golga4	0,217719	-0,01015	0,614	0,646	1
Ago1	0,217796	-0,03003	0,41	0,437	1
Ctnnb1	0,217821	0,064381	0,831	0,802	1
Vps52	0,21785	-0,02807	0,389	0,42	1
Scrg1	0,21807	-0,02026	0,045	0,059	1
Baz1a	0,218264	0,014707	0,029	0,02	1
D3Ertd254	0,218401	-0,01694	0,138	0,163	1
Zfyve16	0,218534	-0,03481	0,306	0,337	1
Myrip	0,218855	-0,01315	0,153	0,177	1

Med19	0,218902	-0,02006	0,42	0,455	1
Knstrn	0,219016	0,011262	0,031	0,021	1
Cblb	0,219029	-0,03071	0,234	0,259	1
Zfp81	0,219092	-0,03331	0,155	0,177	1
Eri1	0,219242	-0,01737	0,276	0,306	1
Sfxn5	0,219365	-0,01523	0,156	0,18	1
Mmab	0,219503	0,019869	0,114	0,094	1
Ube2o	0,219556	-0,02338	0,311	0,34	1
5730403I0	0,2198	-0,01395	0,029	0,041	1
Slbp	0,219911	0,035837	0,26	0,234	1
Tmem203	0,220017	-0,02526	0,204	0,231	1
Acad11	0,220205	-0,00233	0,155	0,182	1
Zfp488	0,220244	-0,01508	0,074	0,091	1
Etfrf1	0,22033	0,032218	0,343	0,314	1
Tjap1	0,220337	0,037966	0,443	0,415	1
Snx18	0,220343	-0,03209	0,473	0,511	1
Rufy1	0,220463	0,024853	0,543	0,509	1
Pde1c	0,220517	-0,06572	0,299	0,325	1
Commd3	0,22061	-0,02851	0,693	0,74	1
Ntrk2	0,220849	-0,02502	0,042	0,055	1
Gpr180	0,22091	-0,01023	0,194	0,223	1
Spata2	0,220966	0,038568	0,476	0,455	1
Usp39	0,22098	-0,03017	0,222	0,247	1
Txnrd2	0,220988	-0,03247	0,167	0,189	1
Grk4	0,22104	-0,00984	0,091	0,111	1
Nup88	0,221069	-0,01995	0,347	0,38	1
Pigq	0,221118	-0,01601	0,716	0,761	1
Mtmr4	0,221218	-0,01505	0,311	0,346	1
Ctns	0,221305	-0,00435	0,142	0,167	1
Eef1g	0,221398	0,050091	0,879	0,842	1
Pnrc2	0,221821	-0,02277	0,428	0,462	1
Lyrm4	0,221826	-0,02184	0,148	0,171	1
Gtf2e2	0,221902	0,034574	0,263	0,239	1
Rtraf	0,222152	-0,03012	0,798	0,839	1
Zbtb21	0,222202	0,03229	0,212	0,187	1
Krit1	0,222214	-0,01286	0,793	0,828	1
Rps29	0,222337	0,02763	1	1	1
Ube2z	0,222424	-0,02765	0,319	0,348	1
Zfp672	0,222648	-0,00873	0,241	0,273	1
Nat14	0,222748	0,025318	0,086	0,07	1
Ppp1r16a	0,222853	0,047484	0,301	0,281	1
4930452G	0,22289	0,00902	0,035	0,024	1
Gm38414	0,222902	-0,00641	0,026	0,037	1
Ehd2	0,222998	0,019792	0,052	0,039	1
Angel1	0,223025	0,021957	0,097	0,08	1
Maip1	0,223216	-0,03091	0,229	0,254	1
Dcun1d5	0,223481	-0,03031	0,438	0,478	1
Thap7	0,223564	-0,02615	0,239	0,267	1
Nbdy	0,223672	-0,02418	0,159	0,182	1
BC022687	0,223731	-0,01615	0,042	0,055	1
Cdk16	0,223815	0,024392	0,547	0,504	1
Ado	0,223892	-0,02358	0,98	0,99	1

Zfp704	0,224031	0,022218	0,579	0,53	1
Zfp973	0,224078	0,012063	0,056	0,042	1
Ndufa11	0,22408	-0,02774	0,844	0,881	1
Rbmx	0,224237	-0,02014	0,496	0,528	1
Med12	0,224339	-0,01875	0,183	0,208	1
Tax1bp3	0,22446	-0,01507	0,193	0,22	1
Klhl11	0,22451	-0,01651	0,113	0,133	1
Klhl28	0,224542	-0,02245	0,335	0,363	1
Hoxa3	0,224692	0,014803	0,118	0,098	1
Glrx5	0,224787	-0,02217	0,439	0,47	1
Izumo4	0,22479	0,043741	0,317	0,294	1
Ttc39b	0,224945	-0,00804	0,023	0,033	1
Chd1l	0,225053	-0,02596	0,153	0,176	1
Cdc37	0,22508	0,038296	0,852	0,837	1
Naa35	0,225127	-0,0249	0,388	0,418	1
4930509G:	0,225293	0,02021	0,048	0,036	1
Pmf1	0,225297	-0,02251	0,075	0,091	1
Lonrf1	0,225328	-0,01884	0,297	0,328	1
Cybrd1	0,225332	-0,00705	0,006	0,011	1
Gm48908	0,225332	-0,01026	0,006	0,011	1
Trip11	0,225352	-0,03495	0,764	0,753	1
Zfp410	0,225561	-0,01364	0,289	0,322	1
Arpin	0,225574	-0,01594	0,136	0,159	1
Rapgefl1	0,225815	0,013916	0,013	0,007	1
Aars2	0,226056	0,01366	0,094	0,076	1
Ndufb2	0,226093	-0,02513	0,856	0,873	1
Gm40437	0,226139	-0,00545	0,006	0,011	1
Hoxc8	0,226167	-0,0108	0,011	0,018	1
Hdac4	0,226224	-0,02822	0,312	0,34	1
Spef2	0,226278	0,001064	0,105	0,127	1
Gm26625	0,226411	-0,0051	0,006	0,011	1
Kif13a	0,226611	-0,04226	0,674	0,68	1
Rcbtb2	0,226709	-0,01397	0,07	0,086	1
Gm13919	0,226759	0,00813	0,013	0,007	1
Slc35d2	0,226759	0,00813	0,013	0,007	1
Ppp2r1b	0,226786	-0,02986	0,143	0,164	1
Zfp825	0,226796	-0,01569	0,093	0,112	1
Slk	0,226828	0,058282	0,738	0,735	1
Pcdha11.1	0,227086	-0,00447	0,006	0,011	1
Lfng	0,227117	0,01134	0,013	0,007	1
Gm26935	0,22714	-0,01333	0,042	0,055	1
Slc35f1	0,227223	-0,0051	0,006	0,011	1
Epb41	0,227237	0,017398	0,013	0,007	1
Gja1	0,227308	-0,00859	0,011	0,018	1
1700037H(	0,227392	-0,01493	0,081	0,099	1
Kdm6a	0,227441	0,063402	0,59	0,574	1
Rab36	0,227466	-0,01485	0,015	0,023	1
Rnf128	0,227493	-0,00122	0,006	0,011	1
Hsf5	0,227537	-0,02173	0,066	0,081	1
Repin1	0,227696	-0,00981	0,12	0,141	1
Clec16a	0,227699	-0,02319	0,229	0,254	1
Gm26884	0,227709	0,010377	0,013	0,007	1

Smco4	0,227709	0,008448	0,013	0,007	1
Tbc1d10b	0,227713	0,069733	0,767	0,793	1
Cyb5rl	0,22773	0,014837	0,054	0,041	1
Gm38910	0,227765	-0,00573	0,006	0,011	1
Smarcd3	0,227906	-0,00683	0,169	0,195	1
Megf8	0,227935	0,05353	0,22	0,2	1
Chuk	0,228036	-0,02861	0,513	0,545	1
Sh3pxd2a	0,228065	0,006837	0,013	0,007	1
Gm26532	0,228079	0,02378	0,054	0,041	1
Rps12	0,228147	0,037837	0,991	0,99	1
Bptf	0,228208	0,042582	0,879	0,885	1
Klf7	0,228246	0,058038	0,873	0,865	1
Gm12981	0,228302	0,006516	0,013	0,007	1
Atxn2	0,228325	-0,02099	0,741	0,763	1
Serf2	0,228327	-0,01966	0,994	0,998	1
Tsr3	0,228377	0,031903	0,297	0,273	1
Abhd10	0,22846	-0,01924	0,19	0,216	1
Pdzd9	0,22846	-0,00824	0,011	0,018	1
Tomm20	0,228473	-0,01996	0,924	0,925	1
Pqlc1	0,228538	-0,01126	0,019	0,028	1
Ubl5	0,22856	-0,02751	0,951	0,95	1
Mlxip	0,228778	-0,01507	0,179	0,205	1
Nt5dc1	0,228779	-0,00596	0,036	0,049	1
Dhrs7b	0,228842	-0,03774	0,369	0,393	1
Tmem59l	0,228916	0,0374	0,219	0,197	1
Gm42500	0,228974	-0,03716	0,184	0,207	1
Snta1	0,229191	-0,0183	0,225	0,254	1
Cdk14	0,229213	-0,00541	0,112	0,133	1
Srgap1	0,229454	0,054257	0,416	0,395	1
Zfp661	0,22949	-0,01601	0,035	0,047	1
Hsp90ab1	0,229493	0,014583	0,999	1	1
Tatdn2	0,229599	-0,01298	0,195	0,223	1
Slc46a1	0,229612	-0,00602	0,011	0,018	1
Ddc	0,22973	0,062618	0,391	0,366	1
Ssh2	0,22994	-0,04083	0,459	0,488	1
Mdn1	0,229978	-0,00867	0,267	0,298	1
Impdh2	0,230278	-0,02529	0,41	0,447	1
Fan1	0,230293	-0,04745	0,201	0,224	1
H3f3a	0,230419	0,029408	1	0,995	1
Eif3d	0,230572	-0,01634	0,668	0,709	1
Pcdhgb7	0,230593	-0,0077	0,023	0,033	1
Fam43a	0,230892	-0,02642	0,063	0,078	1
Ermard	0,231009	-0,02084	0,236	0,263	1
Dusp5	0,23105	0,000416	0,013	0,007	1
Limk2	0,231079	-0,0222	0,277	0,311	1
Midn	0,231295	0,040362	0,057	0,044	1
Tyk2	0,231386	0,025929	0,126	0,106	1
Klf16	0,231424	0,018072	0,097	0,08	1
Mhrt	0,231503	-0,00845	0,019	0,028	1
Peli2	0,231508	-0,03147	0,393	0,421	1
Pcbp1	0,231612	0,024299	0,938	0,933	1
Gm39121	0,231897	-0,01684	0,075	0,091	1

Gm16141	0,232101	-0,01231	0,045	0,059	1
Pja2	0,232115	-0,03063	0,793	0,798	1
Zfp821	0,232139	-0,00714	0,169	0,195	1
Alms1	0,232431	0,035191	0,066	0,052	1
Zfp729a	0,232562	-0,0181	0,266	0,299	1
Sp3	0,232649	-0,0253	0,493	0,535	1
Ubqln4	0,232671	0,035146	0,219	0,197	1
Tbc1d22b	0,23274	-0,02775	0,267	0,293	1
Zfpl1	0,232833	-0,02098	0,349	0,379	1
Zfp386	0,232953	-0,02341	0,213	0,239	1
Snapin	0,232964	0,022878	0,854	0,852	1
Whrn	0,232987	-0,00946	0,029	0,041	1
Dpp7	0,233068	0,043728	0,228	0,207	1
Zranb2	0,233188	-0,01642	0,781	0,831	1
Prob1	0,233189	0,033587	0,184	0,163	1
Oxa1l	0,233241	0,050162	0,406	0,389	1
Simc1	0,233254	-0,0304	0,111	0,13	1
Gm12216	0,233293	0,00818	0,062	0,047	1
Chd2	0,233561	-0,03968	0,414	0,437	1
Kctd7	0,233724	-0,03	0,221	0,244	1
Stau2	0,233724	-0,02349	0,103	0,122	1
Rpl9	0,233852	0,034854	0,994	0,987	1
Cnnm4	0,23392	0,024954	0,074	0,059	1
mt-Nd6	0,234036	-0,05073	0,447	0,476	1
Zfp1	0,234048	-0,02254	0,251	0,28	1
Mrpl1	0,234074	0,026437	0,237	0,213	1
Prdm10	0,234318	-0,01564	0,08	0,098	1
Zfp781	0,234368	-0,00659	0,015	0,023	1
Rpl28	0,234487	0,031909	0,999	0,998	1
Pik3cb	0,234665	-0,02987	0,197	0,22	1
Cystm1	0,234722	-0,02186	0,965	0,977	1
Senp6	0,234734	0,048557	0,845	0,837	1
Psmd9	0,234942	0,028015	0,512	0,481	1
Trp53	0,235242	0,035915	0,267	0,242	1
Gmcl1	0,235254	-0,01482	0,331	0,364	1
Pacsin2	0,235261	-0,01657	0,247	0,276	1
Rpl8	0,235267	0,028146	0,993	0,997	1
Cep89	0,235268	-0,01676	0,154	0,177	1
Osbpl8	0,235336	-0,01732	0,252	0,281	1
Zyx	0,235389	0,053926	0,484	0,449	1
Fgfr1op2	0,235534	0,029989	0,679	0,668	1
Prune1	0,235638	0,022279	0,111	0,093	1
Adrm1	0,235675	-0,02718	0,552	0,579	1
Pou5f1	0,235676	-0,00562	0,015	0,023	1
Pus7	0,235711	-0,02753	0,361	0,395	1
Anks3	0,235809	-0,01736	0,242	0,268	1
Gne	0,236047	-0,00664	0,164	0,189	1
Ecsit	0,236175	-0,01563	0,289	0,317	1
Lnx2	0,236226	0,016141	0,015	0,008	1
Timm9	0,23623	-0,0088	0,371	0,403	1
Pdk1	0,236519	-0,01691	0,057	0,072	1
Uckl1	0,236521	-0,02071	0,339	0,371	1

Gdpgp1	0,236772	-0,01458	0,057	0,072	1
Usp10	0,236875	-0,02312	0,28	0,307	1
Jmjd1c	0,236919	0,126462	0,691	0,691	1
Srsf4	0,236919	-0,01953	0,362	0,4	1
Snx24	0,237046	-0,02151	0,388	0,421	1
Ecd	0,237057	-0,02351	0,298	0,328	1
Atad3a	0,237057	-0,02432	0,226	0,25	1
Ints9	0,237136	-0,01483	0,197	0,224	1
Scyl2	0,237173	0,021796	0,215	0,19	1
C030034I2	0,237434	-0,01698	0,038	0,05	1
Ndufaf4	0,237484	-0,02606	0,241	0,267	1
Derl3	0,237716	-0,01827	0,041	0,054	1
Rad51d	0,237822	-0,02629	0,207	0,233	1
4930590JC	0,237914	0,01134	0,015	0,008	1
Nrg2	0,238093	-0,01051	0,076	0,093	1
Gpr35	0,238303	0,00077	0,015	0,023	1
Zfp992	0,238362	-0,0143	0,066	0,081	1
Ddx56	0,238427	0,041524	0,289	0,267	1
Wrap53	0,238557	-0,04068	0,437	0,46	1
Plagl1	0,23876	-0,02957	0,166	0,187	1
Pkia	0,238763	-0,01496	0,115	0,135	1
Emc10	0,238777	0,028734	0,993	0,995	1
Cgrrf1	0,238819	-0,02178	0,216	0,241	1
Fam212b	0,238871	0,035285	0,184	0,163	1
Slc12a9	0,23902	0,032778	0,456	0,423	1
Tmem86b	0,239187	-0,00879	0,035	0,047	1
Adam11	0,239213	-0,04855	0,182	0,203	1
Nfyc	0,239449	0,034887	0,25	0,226	1
Ppm1g	0,239463	-0,02109	0,454	0,486	1
Glcci1	0,239584	-0,01126	0,15	0,174	1
Ccdc136	0,239597	0,026894	0,259	0,231	1
Gm48529	0,239609	0,008448	0,015	0,008	1
Cdk18	0,239637	-0,02165	0,685	0,735	1
Impa1	0,239726	-0,0258	0,442	0,472	1
Mettl17	0,239734	-0,01823	0,1	0,119	1
Polr2d	0,239748	-0,02286	0,251	0,278	1
Rcan1	0,239882	-0,01536	0,199	0,224	1
Rpl36	0,240139	0,030815	0,997	0,997	1
Ccrl2	0,240176	0,007482	0,015	0,008	1
Hgs	0,240176	0,007482	0,015	0,008	1
Rnf10	0,240318	0,037077	0,792	0,792	1
9230104M	0,240521	0,009728	0,015	0,008	1
Zfp35	0,240622	-0,0177	0,112	0,132	1
5430402O	0,240743	0,006516	0,015	0,008	1
Gm14168	0,240749	0,007801	0,015	0,008	1
Smarcal1	0,240752	-0,01219	0,188	0,213	1
Capzb	0,24111	0,043031	0,739	0,743	1
Arpc4	0,241121	0,031781	0,738	0,715	1
Zdhhc24	0,241151	-0,02779	0,12	0,138	1
Sdhaf2	0,241214	-0,01835	0,278	0,309	1
Ccpg1os	0,241217	-0,02545	0,11	0,128	1
Slc30a1	0,241368	0,05318	0,226	0,207	1

Ipo11	0,241518	-0,0092	0,173	0,198	1
Ntan1	0,241629	-0,01504	0,635	0,663	1
Rdh13	0,241863	-0,01394	0,083	0,101	1
Adam18	0,241888	0,005871	0,015	0,008	1
Secisbp2l	0,242094	-0,02822	0,984	0,99	1
B4galt6	0,242321	-0,01575	0,147	0,169	1
Rab22a	0,242369	-0,02068	0,528	0,567	1
BC003331	0,242389	-0,02195	0,482	0,514	1
Emc4	0,242491	0,031949	0,6	0,58	1
Grb14	0,242524	-0,03012	0,949	0,941	1
Fam126b	0,242536	0,035636	0,361	0,337	1
Mmp16	0,24254	-0,00423	0,026	0,036	1
Tmem143	0,242553	-0,01766	0,148	0,171	1
Mfsd5	0,242726	-0,01875	0,303	0,338	1
Oaz2	0,242759	-0,01992	0,392	0,423	1
1700052K:	0,242772	-0,00786	0,054	0,068	1
Snupn	0,242901	-0,0258	0,206	0,231	1
Atad2b	0,242905	-0,02717	0,451	0,499	1
Bccip	0,242909	-0,0216	0,365	0,398	1
Rnf41	0,242914	-0,03034	0,267	0,294	1
Ufc1	0,242918	-0,0292	0,641	0,655	1
Cxxc5	0,242979	0,038924	0,85	0,85	1
Trpv4	0,243057	0,019634	0,04	0,029	1
AC163032.	0,243074	-0,0098	0,148	0,172	1
Nkx2-9	0,243084	-0,00593	0,22	0,247	1
Aco1	0,243236	-0,00365	0,235	0,267	1
Rraga	0,243324	-0,02624	0,62	0,665	1
Exosc1	0,243337	0,035382	0,283	0,257	1
Ccdc166	0,243692	0,01025	0,046	0,034	1
Tpgs2	0,243715	-0,01953	0,31	0,34	1
Baiap2	0,24372	0,024931	0,04	0,029	1
Angel2	0,243725	-0,03473	0,499	0,522	1
Mkl2	0,243918	-0,01782	0,221	0,247	1
Hspa1b	0,244048	0,145016	0,395	0,363	1
Gcn1l1	0,244135	0,039269	0,416	0,395	1
Rrh	0,244236	-0,00803	0,005	0,01	1
Slc35g2	0,244236	-0,00803	0,005	0,01	1
Rpl26	0,244278	-0,03181	0,995	0,993	1
Hexim2	0,244347	-0,0221	0,056	0,07	1
Zscan21	0,244416	-0,01518	0,132	0,153	1
Rpa1	0,244417	-0,02529	0,422	0,455	1
Timm8b	0,244449	-0,02332	0,72	0,758	1
Dennd6a	0,24446	-0,03063	0,281	0,307	1
Dync1h1	0,24451	-0,01195	0,959	0,971	1
Osbpl2	0,24451	-0,01167	0,337	0,369	1
Psmd6	0,24464	-0,03238	0,623	0,655	1
Tmem201	0,244705	-0,03027	0,214	0,237	1
Zfp40	0,244782	-0,02843	0,186	0,21	1
Arpc5l	0,244799	-0,02415	0,612	0,636	1
Ankrd28	0,244808	0,011009	0,89	0,919	1
Abcb6	0,245005	-0,012	0,107	0,127	1
Grm5	0,24501	-0,00642	0,005	0,01	1

Ednrb	0,24501	-0,00642	0,005	0,01	1
Gm17597	0,24501	-0,00642	0,005	0,01	1
Kpnb1	0,245051	-0,02942	0,657	0,686	1
Nudt1	0,245195	-0,02237	0,116	0,135	1
Selenon	0,245222	-0,03107	0,349	0,376	1
Zfp709	0,245281	0,010577	0,044	0,033	1
Bex2	0,245448	-0,00946	0,028	0,039	1
Gm49012	0,245574	0,007347	0,097	0,08	1
Trpc1	0,245583	-0,01367	0,415	0,449	1
Akap7	0,245661	0,033176	0,325	0,298	1
Fam129b	0,245685	0,023889	0,128	0,109	1
Sik2	0,245766	-0,03066	0,327	0,356	1
4930524J(	0,245768	-0,00811	0,022	0,031	1
Gm42417	0,245785	-0,00481	0,005	0,01	1
Eva1a	0,245785	-0,00481	0,005	0,01	1
Amigo3	0,245785	-0,00481	0,005	0,01	1
Slc51a	0,245785	-0,00481	0,005	0,01	1
Dlgap5	0,245787	-0,00705	0,005	0,01	1
Cdca8	0,245787	-0,00954	0,028	0,039	1
Ttc30a1	0,245931	-0,00859	0,028	0,039	1
Rev1	0,246058	0,035883	0,265	0,241	1
Ccnh	0,246133	-0,01709	0,393	0,424	1
Ltbp1	0,246193	0,014834	0,017	0,01	1
Slc22a15	0,246208	-0,03776	0,197	0,221	1
Mrpl36	0,246413	-0,02076	0,395	0,434	1
Setd6	0,246565	0,01671	0,109	0,091	1
Radil	0,24672	-0,00189	0,005	0,01	1
2210411M	0,24672	-0,00384	0,005	0,01	1
C130013H(	0,24672	-0,00384	0,005	0,01	1
Sde2	0,246743	-0,03583	0,404	0,433	1
Thra	0,246764	-0,01905	0,989	0,984	1
Sh3bp5	0,246788	0,054175	0,882	0,885	1
Twistnb	0,247084	-0,00415	0,348	0,385	1
Tle4	0,247115	-0,00394	0,395	0,436	1
Grid1	0,247177	0,011366	0,008	0,003	1
Dvl2	0,24738	0,035228	0,242	0,22	1
Dnajc4	0,247402	-0,01576	0,271	0,301	1
Zscan18	0,24743	0,017261	0,057	0,044	1
Gm33819	0,247504	0,008461	0,008	0,003	1
Zc3hav1l	0,247504	0,008461	0,008	0,003	1
Peg3	0,24763	-0,04353	0,345	0,372	1
Lypd6b	0,247751	0,018958	0,091	0,075	1
Nrm	0,247811	0,01069	0,017	0,01	1
Mybphl	0,247811	0,012611	0,017	0,01	1
Syne4	0,247831	0,006519	0,008	0,003	1
Gm47237	0,247831	0,006519	0,008	0,003	1
Klhl10	0,247831	0,006519	0,008	0,003	1
Arhgap26	0,247831	0,006519	0,008	0,003	1
Rasgrf2	0,247831	0,00749	0,008	0,003	1
Urm1	0,247843	-0,02254	0,784	0,807	1
Dnph1	0,247867	-0,02167	0,093	0,111	1
Gm47689	0,248014	-0,00608	0,028	0,039	1

Zmym6	0,248053	-0,03739	0,226	0,249	1
Zmym3	0,248089	-0,01491	0,268	0,299	1
Ampd3	0,248159	0,006519	0,008	0,003	1
Bag2	0,248159	0,005547	0,008	0,003	1
Lama5	0,248159	0,005547	0,008	0,003	1
Sarm1	0,248159	0,005547	0,008	0,003	1
Peg10	0,24816	-0,03369	0,243	0,268	1
D8Ertd738	0,248205	-0,02178	0,903	0,911	1
Tbx6	0,248245	0,020994	0,032	0,023	1
Ormdl1	0,248342	-0,02293	0,227	0,252	1
Pcbp4	0,248454	0,034248	0,992	0,993	1
Gxylt2	0,248461	0,008765	0,017	0,01	1
Snap23	0,24847	-0,03276	0,331	0,358	1
Zfp746	0,248484	-0,02189	0,205	0,231	1
Pnck	0,248486	0,004573	0,008	0,003	1
Chp2	0,248486	0,004573	0,008	0,003	1
Gm45846	0,248486	0,004573	0,008	0,003	1
Slc44a2	0,248591	0,040641	0,408	0,382	1
Alg12	0,248654	-0,01526	0,082	0,099	1
Gm16052	0,248718	-0,0049	0,022	0,031	1
Gm17275	0,2488	-0,00299	0,022	0,031	1
G630016G	0,248817	-0,01018	0,01	0,016	1
Fth1	0,248851	-0,02524	1	1	1
Zim1	0,248901	0,008436	0,017	0,01	1
Stac2	0,248981	0,007811	0,008	0,003	1
Tmem88	0,248981	0,006841	0,008	0,003	1
Atg2a	0,248987	-0,02544	0,225	0,249	1
Dars	0,249111	-0,03339	0,6	0,61	1
Mapkapk3	0,249112	0,009728	0,017	0,01	1
Dtwd1	0,24915	0,023916	0,094	0,078	1
AU019823	0,249244	-0,02527	0,285	0,314	1
Sgsh	0,24936	-0,0135	0,183	0,208	1
Naca	0,249498	0,036548	0,978	0,985	1
Hdac10	0,249507	-0,02316	0,135	0,156	1
Kirrel2	0,249764	0,006837	0,017	0,01	1
Acbd7	0,249803	0,002954	0,008	0,003	1
Gm53	0,249803	0,002954	0,008	0,003	1
D130017N	0,250024	-0,02162	0,156	0,177	1
Smpd4	0,250039	-0,00493	0,135	0,158	1
Ppig	0,250128	-0,01127	0,713	0,748	1
Tmem178b	0,250179	0,042524	0,86	0,844	1
Gpsm1	0,250227	0,014762	0,314	0,283	1
Zfp800	0,250236	-0,0172	0,446	0,478	1
Tgfbr1	0,25024	-0,00819	0,218	0,246	1
Ankrd40	0,250399	0,039898	0,787	0,769	1
Zfp711	0,250795	5,69E-05	0,008	0,003	1
Phospho2	0,25081	-0,00909	0,182	0,207	1
Ppp1r13l	0,250812	0,013877	0,019	0,011	1
Snx5	0,250818	-0,03557	0,668	0,693	1
Rpl15	0,250823	-0,02382	0,985	0,976	1
Gm43625	0,25086	0,007156	0,017	0,01	1
Nos1ap	0,250906	-0,01509	0,104	0,124	1

Mpdz	0,250995	-0,02442	0,423	0,447	1
9430002A1	0,25115	-0,00636	0,01	0,016	1
Magoh	0,251297	-0,02643	0,383	0,411	1
Itpa	0,251377	-0,01248	0,363	0,395	1
Usp40	0,251467	-0,03628	0,338	0,367	1
Zfand2a	0,251674	0,028134	0,23	0,208	1
Nagk	0,251676	0,036318	0,304	0,28	1
Prima1	0,251821	0,058804	0,537	0,52	1
Crtc2	0,251938	-0,01135	0,204	0,231	1
Zswim3	0,252084	-0,00817	0,018	0,026	1
Zfp995	0,252343	-0,01351	0,034	0,046	1
U2af1	0,252408	0,033421	0,899	0,888	1
Dpy19l3	0,252448	-0,00408	0,01	0,016	1
Col7a1	0,252448	-0,00505	0,01	0,016	1
Mid1ip1	0,252675	-0,03142	0,954	0,963	1
Plpp7	0,252732	0,019227	0,168	0,146	1
Gm16152	0,252882	-0,01178	0,025	0,034	1
Chic2	0,252949	-0,01899	0,065	0,08	1
Mmaa	0,253018	0,023584	0,224	0,2	1
Nrxn1	0,253279	-0,0318	0,079	0,094	1
Fabp7	0,253315	0,020705	0,032	0,023	1
Gm28221	0,25354	-0,00625	0,018	0,026	1
Ighmbp2	0,253553	0,012361	0,057	0,044	1
Mdh2	0,253557	-0,01295	0,857	0,88	1
Tpst2	0,25361	0,041172	0,231	0,211	1
Polr3b	0,253673	-0,01132	0,127	0,148	1
Nlrc4	0,253702	0,010042	0,019	0,011	1
Slc17a5	0,253737	-0,03906	0,28	0,302	1
Sik1	0,253788	-0,01349	0,044	0,057	1
Slc39a13	0,25379	0,018707	0,265	0,237	1
Spsb3	0,25383	0,021304	0,304	0,275	1
Gpi1	0,253985	-0,01378	0,89	0,906	1
Zfp354a	0,254036	0,023	0,121	0,102	1
Fbxo10	0,254072	-0,01337	0,082	0,099	1
Zfp213	0,254082	0,019097	0,13	0,111	1
Bmt2	0,254097	-0,02947	0,277	0,301	1
Kdelr2	0,254148	-0,01347	0,433	0,472	1
Bloc1s4	0,254279	-0,02671	0,291	0,317	1
Sbk3	0,254428	0,011002	0,019	0,011	1
AI597479	0,254482	-0,01403	0,176	0,2	1
Zc3h8	0,254504	-0,01977	0,111	0,13	1
Nr6a1	0,254595	-0,01497	0,062	0,076	1
Nop9	0,254768	-0,01179	0,145	0,167	1
Tppp3	0,255029	-0,03813	0,942	0,935	1
Adamtsl1	0,255223	-0,02457	0,598	0,618	1
Rab30	0,255237	-0,02767	0,259	0,285	1
Zfyve28	0,25541	-0,01195	0,014	0,021	1
Hsf4	0,255473	0,010355	0,019	0,011	1
Eif2ak3	0,255545	-0,02424	0,207	0,229	1
Abcd3	0,255561	-0,02419	0,479	0,515	1
1700056E2	0,255613	-0,00976	0,014	0,021	1
Lpo	0,25565	-0,01469	0,074	0,089	1

Dclre1a	0,255708	-0,0159	0,12	0,14	1
Gm16958	0,25586	-0,00715	0,06	0,075	1
Lncpint	0,255968	-0,03313	0,229	0,25	1
Ppib	0,256396	-0,01776	0,956	0,964	1
Kat6a	0,256597	-0,02155	0,664	0,68	1
Clasrp	0,256699	0,025306	0,546	0,524	1
Gm26724	0,256707	-0,02106	0,124	0,143	1
Eid2b	0,256779	-0,01209	0,13	0,151	1
Rexo4	0,256789	0,029607	0,254	0,231	1
Setd7	0,256797	-0,03169	0,627	0,655	1
Nrd1	0,25692	-0,02512	0,833	0,842	1
Ide	0,256967	-0,04034	0,513	0,533	1
Itpripl1	0,257038	0,007475	0,019	0,011	1
Tbl2	0,257041	-0,02103	0,142	0,163	1
Chst10	0,257048	-0,00755	0,014	0,021	1
Ndufs8	0,257053	0,027496	0,641	0,623	1
Timp4	0,257247	-0,0063	0,014	0,021	1
C1qtnf4	0,257251	-0,00596	0,014	0,021	1
Nova1	0,257661	-0,01704	0,252	0,278	1
Cep131	0,257757	-0,01588	0,093	0,111	1
Rnf121	0,257888	-0,02383	0,292	0,32	1
Pmvk	0,257914	-0,01951	0,515	0,554	1
Sdhaf3	0,258055	-0,02678	0,119	0,137	1
Slc22a18	0,258226	-0,02003	0,043	0,055	1
Gm20696	0,258254	-0,00973	0,034	0,046	1
Gm10561	0,258382	-0,01067	0,034	0,046	1
Cks2	0,25839	-0,00659	0,014	0,021	1
Mylk	0,258412	0,024174	0,023	0,015	1
Sgf29	0,258518	-0,02189	0,206	0,229	1
Zfp865	0,25884	0,036375	0,204	0,184	1
Mad2l1	0,258915	-0,02083	0,061	0,075	1
Ppp1r15b	0,259014	-0,02716	0,498	0,533	1
Dcun1d4	0,259066	-0,01916	0,214	0,239	1
Oaf	0,25909	-0,01358	0,04	0,052	1
Mettl6	0,25912	-0,00699	0,189	0,216	1
Zfp646	0,25913	-0,02516	0,279	0,306	1
Tyw3	0,25924	-0,01968	0,073	0,088	1
Phactr3	0,259251	0,045228	0,476	0,455	1
Mindy1	0,259419	-0,03112	0,633	0,659	1
Micu3	0,259491	-0,0482	0,399	0,424	1
Cenpt	0,259565	0,022877	0,073	0,059	1
Ormdl2	0,259566	-0,0113	0,414	0,45	1
Nt5m	0,259625	-0,02997	0,225	0,247	1
Lcorl	0,25972	-0,02862	0,634	0,659	1
Eral1	0,259819	-0,02148	0,129	0,148	1
Dand5	0,259831	0,008454	0,127	0,107	1
Mtmr9	0,259852	-0,02947	0,332	0,359	1
Wwox	0,260034	-0,02361	0,255	0,281	1
Rps9	0,260132	0,016311	0,996	0,998	1
BC051077	0,260179	0,025217	0,06	0,047	1
Zfp619	0,260185	-0,00944	0,05	0,063	1
Gm14403	0,260738	0,017567	0,079	0,063	1

Taf7	0,260806	-0,02352	0,465	0,499	1
Klf2	0,260879	0,036246	0,021	0,013	1
Stoml1	0,260934	0,018875	0,236	0,211	1
Odf2	0,260945	-0,01999	0,385	0,42	1
Csnk2a1	0,261022	0,035567	0,954	0,948	1
Pacs1	0,261137	0,011904	0,028	0,02	1
Gpr176	0,261216	-0,01239	0,027	0,037	1
Smim5	0,261411	0,002123	0,08	0,098	1
Eml6	0,261459	-0,02396	0,185	0,208	1
Dalrd3	0,261822	-0,00637	0,299	0,335	1
Ptpn21	0,261825	0,015924	0,089	0,073	1
Gm7324	0,261853	0,011287	0,025	0,016	1
Gm26664	0,261872	0,011596	0,026	0,018	1
Bdh1	0,261997	-0,01791	0,095	0,112	1
Mogs	0,262009	-0,01363	0,14	0,161	1
Dag1	0,262059	-0,01935	0,169	0,192	1
Sgip1	0,262094	-0,01395	0,279	0,309	1
C1galt1	0,262113	-0,03294	0,21	0,231	1
A230001M	0,262132	-0,01705	0,39	0,42	1
Elp3	0,262153	0,022835	0,314	0,286	1
Ncoa5	0,262202	-0,02312	0,166	0,187	1
E2f5	0,262204	-0,02841	0,236	0,259	1
Zfhx3	0,262224	0,051741	0,315	0,291	1
Mob3c	0,262304	-0,02013	0,07	0,085	1
Gm44623	0,262415	-0,01427	0,043	0,055	1
Ifngr1	0,262422	-0,01248	0,244	0,273	1
Prrc1	0,262434	-0,01214	0,237	0,265	1
Rpgr	0,262535	-0,01988	0,109	0,127	1
Cnst	0,262725	-0,02114	0,132	0,151	1
Trim8	0,262767	0,004562	0,464	0,512	1
Dph7	0,262847	-0,01904	0,175	0,197	1
Uba3	0,262937	-0,01556	0,43	0,465	1
Magee1	0,26305	-0,01743	0,302	0,332	1
H2-K1	0,263076	-0,12712	0,505	0,473	1
Zhx3	0,263207	-0,03743	0,272	0,291	1
C530008M	0,263398	-0,03336	0,22	0,244	1
Trmt10c	0,263408	-0,01827	0,132	0,151	1
Ankrd44	0,263498	0,027131	0,302	0,278	1
Cc2d1a	0,263527	-0,01576	0,144	0,166	1
Lrig3	0,26354	-0,01945	0,338	0,364	1
Hspb1	0,263592	0,004285	0,014	0,021	1
Usp32	0,263636	-0,02881	0,399	0,428	1
Churc1	0,263713	-0,02079	0,703	0,746	1
Kmt2a	0,263869	-0,02439	0,696	0,735	1
Timp2	0,263875	0,036223	0,847	0,831	1
Trmt5	0,263968	-0,02217	0,08	0,096	1
Cast	0,263986	0,037874	0,156	0,137	1
Krcc1	0,263998	0,05408	0,419	0,397	1
Zfp28	0,264002	0,017836	0,062	0,049	1
Prmt7	0,264179	-0,01858	0,219	0,242	1
A330069E1	0,264294	-0,12502	0,106	0,124	1
Cul2	0,264308	-0,02111	0,262	0,288	1

Bend6	0,264496	-0,01187	0,021	0,029	1
Egfem1	0,264665	-0,01064	0,021	0,029	1
Klk9	0,264676	0,054442	0,058	0,046	1
D030051J2	0,264765	-0,0074	0,004	0,008	1
Crtam	0,264765	-0,0074	0,004	0,008	1
Gm10658	0,264765	-0,00901	0,004	0,008	1
Polk	0,265039	-0,02177	0,17	0,19	1
Plekhh3	0,265059	0,02775	0,067	0,054	1
Rex1bd	0,265179	-0,01862	0,791	0,808	1
Tmem11	0,26522	-0,02205	0,295	0,325	1
Mad2l1bp	0,265231	0,019631	0,092	0,076	1
Mfsd12	0,265281	0,004404	0,081	0,099	1
Zfand1	0,265383	-0,0145	0,127	0,146	1
Dact1	0,265401	0,006827	0,023	0,015	1
Nfkbia	0,265436	0,02945	0,367	0,343	1
Chrdl2	0,265489	-0,00579	0,004	0,008	1
1810010D0	0,265489	-0,00579	0,004	0,008	1
Reep6	0,265489	-0,00579	0,004	0,008	1
1700001P0	0,265489	-0,00579	0,004	0,008	1
Rflna	0,265489	-0,0074	0,004	0,008	1
Tnfrsf23	0,26549	-0,01761	0,004	0,008	1
Rrnad1	0,265498	-0,03283	0,306	0,333	1
Hmgn2	0,265638	-0,01649	0,844	0,863	1
Pdzd2	0,265671	-0,00642	0,004	0,008	1
Gm42669	0,265683	0,004277	0,023	0,015	1
Kansl2	0,265746	-0,0257	0,334	0,358	1
Armcx1	0,265867	-0,01323	0,315	0,348	1
Uso1	0,26588	-0,02254	0,387	0,415	1
Dgcr2	0,265899	-0,02945	0,385	0,411	1
Dync1i2	0,265952	-0,02389	0,986	0,993	1
Prox1os	0,266122	0,043768	0,402	0,377	1
Gps2	0,266206	0,026815	0,484	0,463	1
Gm27184	0,266214	-0,00418	0,004	0,008	1
Mybph	0,266214	-0,00418	0,004	0,008	1
Grin1	0,266214	-0,00418	0,004	0,008	1
1110032F0	0,266214	-0,00418	0,004	0,008	1
Chd5	0,266214	-0,00418	0,004	0,008	1
Rph3a	0,266214	-0,00418	0,004	0,008	1
Adcyap1r1	0,266214	-0,00418	0,004	0,008	1
Pglyrp1	0,266214	-0,00418	0,004	0,008	1
Hoxb6	0,266214	-0,00418	0,004	0,008	1
Cygb	0,266214	-0,00418	0,004	0,008	1
Hist1h3e	0,266214	-0,00418	0,004	0,008	1
Pvalb	0,266214	-0,00418	0,004	0,008	1
Zfp947	0,266214	-0,00418	0,004	0,008	1
Kif20a	0,266214	-0,00418	0,004	0,008	1
Acsl5	0,266214	-0,00418	0,004	0,008	1
Thsd1	0,26622	-0,00902	0,094	0,112	1
Zfp455	0,266269	0,009035	0,028	0,02	1
Spred2	0,266295	-0,0177	0,109	0,127	1
Adnp2	0,266337	-0,0162	0,142	0,163	1
Flicr	0,266396	-0,00481	0,004	0,008	1

Nr2c1	0,266422	-0,02866	0,191	0,211	1
Clip1	0,266544	-0,03294	0,138	0,156	1
Chpf2	0,266545	0,029475	0,132	0,114	1
Ginm1	0,266722	-0,01626	0,298	0,324	1
Srfbp1	0,266804	-0,01209	0,124	0,143	1
Telo2	0,266932	-0,01588	0,171	0,193	1
Eif2s2	0,267085	0,030658	0,976	0,969	1
Atp5e	0,267109	-0,02285	0,977	0,987	1
Dlgap1	0,267123	0,001668	0,004	0,008	1
Gm20659	0,267123	-0,00125	0,004	0,008	1
Retn	0,267123	-0,00223	0,004	0,008	1
Zbtb46	0,267123	-0,0032	0,004	0,008	1
Gm32849	0,267123	-0,0032	0,004	0,008	1
Kbtbd6	0,267123	-0,0032	0,004	0,008	1
Cnmd	0,267123	-0,0032	0,004	0,008	1
Gm47232	0,267123	-0,0032	0,004	0,008	1
Sem1	0,267145	-0,0212	0,911	0,927	1
Usp16	0,267186	-0,02832	0,432	0,459	1
Ss18	0,267203	0,031576	0,522	0,502	1
Zfp202	0,267233	-0,00413	0,034	0,046	1
B430010I2	0,267248	-0,03467	0,255	0,28	1
Gm26660	0,267309	-0,02328	0,095	0,112	1
Zfp41	0,267337	-0,01503	0,179	0,203	1
Pgm2	0,267346	-0,01298	0,205	0,231	1
Pdgfa	0,267406	0,029526	0,947	0,935	1
Edem2	0,267482	0,032794	0,576	0,548	1
Habp4	0,26751	-0,01685	0,152	0,174	1
E430024P1	0,267575	-0,01187	0,021	0,029	1
Ppp1r1b	0,267797	0,003964	0,028	0,02	1
Hltf	0,267986	-0,01177	0,406	0,436	1
Il3ra	0,268034	-0,00223	0,004	0,008	1
Cstf3	0,268069	-0,03647	0,242	0,262	1
Mpc1	0,268178	-0,02962	0,982	0,979	1
Ada	0,26818	0,017956	0,081	0,067	1
Acad8	0,268401	-0,02159	0,263	0,288	1
Asap1	0,26846	-0,00865	0,361	0,395	1
Cir1	0,268566	-0,0212	0,638	0,654	1
Purg	0,268629	-0,02665	0,17	0,19	1
Tardbp	0,268634	-0,03631	0,616	0,65	1
Kmt2c	0,268692	-0,00956	0,763	0,807	1
Zmym5	0,268768	0,033892	0,512	0,478	1
Dlst	0,268829	-0,03888	0,528	0,558	1
Tmem216	0,268858	-0,0078	0,059	0,073	1
Ankrd37	0,268861	0,018465	0,06	0,047	1
Nfil3	0,268927	0,022016	0,168	0,148	1
Psme4	0,269016	0,048918	0,382	0,367	1
Stat5b	0,269075	-0,01624	0,098	0,115	1
Ankhd1	0,269112	0,028141	0,52	0,502	1
Ccdc51	0,269365	-0,01205	0,046	0,059	1
Hist3h2ba	0,269585	0,05545	0,449	0,431	1
Orc6	0,269716	-0,02418	0,334	0,367	1
Plekhm2	0,269827	0,024189	0,301	0,273	1

Rhou	0,269876	0,043879	0,957	0,959	1
Ppp1cb	0,269901	-0,02043	0,891	0,902	1
Cenpo	0,269909	-0,01821	0,079	0,094	1
Asic1	0,270214	-0,01109	0,03	0,041	1
Pan2	0,270271	-0,02897	0,179	0,198	1
Bms1	0,270461	-0,02795	0,266	0,289	1
Scamp5	0,27049	-0,02502	0,77	0,803	1
Gnai3	0,270532	0,033902	0,604	0,582	1
Arid2	0,270613	-0,02168	0,36	0,389	1
Klhl32	0,27078	0,015669	0,062	0,049	1
Hdac3	0,270917	-0,02494	0,289	0,317	1
Wdr37	0,270924	-0,03002	0,347	0,371	1
Slc38a9	0,271073	0,045488	0,422	0,4	1
Pam16	0,271176	-0,01698	0,532	0,567	1
Ralgps2	0,271279	0,025318	0,06	0,047	1
Cldnd1	0,271385	-0,01699	0,98	0,99	1
Zfp775	0,271435	-0,0227	0,117	0,135	1
Smyd3	0,271651	-0,00733	0,15	0,172	1
Pfkp	0,271804	-0,00757	0,03	0,041	1
Abhd4	0,271887	-0,03908	0,681	0,694	1
Fam20c	0,271982	0,029809	0,268	0,244	1
Zc3h4	0,272049	0,041457	0,261	0,239	1
Inpp4b	0,272157	0,003918	0,002	0	1
March4	0,272157	0,001961	0,002	0	1
Wnt6	0,272157	0,001961	0,002	0	1
Sgpp2	0,272157	0,001961	0,002	0	1
Ackr3	0,272157	0,001961	0,002	0	1
Syt2	0,272157	0,001961	0,002	0	1
Ptprc	0,272157	0,001961	0,002	0	1
Ccdc121	0,272157	0,001961	0,002	0	1
Tor4a	0,272157	0,001961	0,002	0	1
Col5a1	0,272157	0,001961	0,002	0	1
Ak8	0,272157	0,001961	0,002	0	1
Cers6	0,272157	0,001961	0,002	0	1
Ryr3	0,272157	0,001961	0,002	0	1
Bub1b	0,272157	0,001961	0,002	0	1
Il1a	0,272157	0,001961	0,002	0	1
Xlr	0,272157	0,001961	0,002	0	1
Gm15726	0,272157	0,001961	0,002	0	1
Sertm1	0,272157	0,001961	0,002	0	1
Igsf10	0,272157	0,001961	0,002	0	1
Fcgr1	0,272157	0,001961	0,002	0	1
Kcnd3	0,272157	0,001961	0,002	0	1
Lrrc39	0,272157	0,001961	0,002	0	1
Ddit4l	0,272157	0,001961	0,002	0	1
Runx1t1	0,272157	0,001961	0,002	0	1
Gm20878	0,272157	0,001961	0,002	0	1
Akna	0,272157	0,001961	0,002	0	1
Cfap57	0,272157	0,001961	0,002	0	1
Zmynd12	0,272157	0,001961	0,002	0	1
Runx3	0,272157	0,001961	0,002	0	1
Arhgef10l	0,272157	0,001961	0,002	0	1

Cdk6	0,272157	0,001961	0,002	0	1
Caln1	0,272157	0,001961	0,002	0	1
Cped1	0,272157	0,001961	0,002	0	1
Cfap100	0,272157	0,001961	0,002	0	1
Iqsec3	0,272157	0,001961	0,002	0	1
Ptpn6	0,272157	0,001961	0,002	0	1
Rasl2-9	0,272157	0,001961	0,002	0	1
Hpn	0,272157	0,001961	0,002	0	1
Gm10605	0,272157	0,001961	0,002	0	1
Folr2	0,272157	0,001961	0,002	0	1
Itgax	0,272157	0,001961	0,002	0	1
Gm47710	0,272157	0,001961	0,002	0	1
Ddo	0,272157	0,001961	0,002	0	1
Adora2a	0,272157	0,001961	0,002	0	1
1500009L1	0,272157	0,001961	0,002	0	1
Kitl	0,272157	0,001961	0,002	0	1
Csmd1	0,272157	0,001961	0,002	0	1
Ppp1r3b	0,272157	0,001961	0,002	0	1
Sorbs2	0,272157	0,001961	0,002	0	1
Dctd	0,272157	0,001961	0,002	0	1
Lyl1	0,272157	0,001961	0,002	0	1
Gm42031	0,272157	0,001961	0,002	0	1
Dnaaf1	0,272157	0,001961	0,002	0	1
Mir124a-1l	0,272157	0,001961	0,002	0	1
Mmp12	0,272157	0,001961	0,002	0	1
Fut4	0,272157	0,001961	0,002	0	1
Epor	0,272157	0,001961	0,002	0	1
Slc37a2	0,272157	0,001961	0,002	0	1
C030014I2	0,272157	0,001961	0,002	0	1
Tpbg	0,272157	0,001961	0,002	0	1
Gm47950	0,272157	0,001961	0,002	0	1
Gm12116	0,272157	0,001961	0,002	0	1
Glra1	0,272157	0,001961	0,002	0	1
Fam183b	0,272157	0,001961	0,002	0	1
Tmem132e	0,272157	0,001961	0,002	0	1
Mmp28	0,272157	0,001961	0,002	0	1
Pctp	0,272157	0,001961	0,002	0	1
Meox1	0,272157	0,001961	0,002	0	1
Mpp3	0,272157	0,001961	0,002	0	1
Cd300c2	0,272157	0,001961	0,002	0	1
Ush1g	0,272157	0,001961	0,002	0	1
Inhba	0,272157	0,001961	0,002	0	1
Hist1h2br	0,272157	0,001961	0,002	0	1
Hist1h2ab	0,272157	0,001961	0,002	0	1
D130043K1	0,272157	0,001961	0,002	0	1
Ly86	0,272157	0,001961	0,002	0	1
Pygl	0,272157	0,001961	0,002	0	1
Plekhd1	0,272157	0,001961	0,002	0	1
Fam181a	0,272157	0,001961	0,002	0	1
Gm16006	0,272157	0,001961	0,002	0	1
AL590144.	0,272157	0,001961	0,002	0	1
Prr5	0,272157	0,001961	0,002	0	1

Bin2	0,272157	0,001961	0,002	0	1
Shisa9	0,272157	0,001961	0,002	0	1
Ifitm7	0,272157	0,001961	0,002	0	1
Boc	0,272157	0,001961	0,002	0	1
Ager	0,272157	0,001961	0,002	0	1
B430306N(	0,272157	0,001961	0,002	0	1
Adgre1	0,272157	0,001961	0,002	0	1
Gm6277	0,272157	0,001961	0,002	0	1
Adrb1	0,272157	0,001961	0,002	0	1
Cldn19	0,272157	0,01558	0,002	0	1
Dock11	0,272157	0,006846	0,002	0	1
Ptgr1	0,272157	0,004895	0,002	0	1
Col4a1	0,272157	0,004895	0,002	0	1
Ccdc162	0,272157	0,003918	0,002	0	1
Klhl3	0,272157	0,003918	0,002	0	1
1500015O:	0,272157	0,00294	0,002	0	1
Cfh	0,272157	0,00294	0,002	0	1
Gad1	0,272157	0,00294	0,002	0	1
Itpka	0,272157	0,00294	0,002	0	1
Dusp9	0,272157	0,00294	0,002	0	1
Ar	0,272157	0,00294	0,002	0	1
Tmem212	0,272157	0,00294	0,002	0	1
Adora3	0,272157	0,00294	0,002	0	1
Tctex1d1	0,272157	0,00294	0,002	0	1
Siglech	0,272157	0,00294	0,002	0	1
Gm15880	0,272157	0,00294	0,002	0	1
Pde2a	0,272157	0,00294	0,002	0	1
Cracr2b	0,272157	0,00294	0,002	0	1
Dusp4	0,272157	0,00294	0,002	0	1
Erc2	0,272157	0,00294	0,002	0	1
Lrrc18	0,272157	0,00294	0,002	0	1
Bcl2a1b	0,272157	0,00294	0,002	0	1
Hoxb4	0,272157	0,00294	0,002	0	1
Arl5c	0,272157	0,00294	0,002	0	1
Fmnl1	0,272157	0,00294	0,002	0	1
Ak7	0,272157	0,00294	0,002	0	1
Rimbp3	0,272157	0,00294	0,002	0	1
Tmem184c	0,272186	-0,02474	0,537	0,559	1
Xrn1	0,272207	-0,02534	0,447	0,488	1
Atp2b1	0,272898	-0,00515	0,713	0,746	1
Ppp1ca	0,272986	-0,01892	0,876	0,914	1
Gm40117	0,273059	-0,01482	0,058	0,072	1
Nsd3	0,273097	-0,02711	0,659	0,68	1
Acly	0,27333	-0,01813	0,57	0,585	1
Fabp3	0,27344	-0,01223	0,052	0,065	1
Gm48855	0,273594	0,008934	0,066	0,052	1
Hpcal1	0,273674	0,019622	0,216	0,192	1
Snx10	0,273837	-0,04515	0,328	0,356	1
Zfp511	0,273909	0,035116	0,233	0,213	1
Ppp1r12c	0,273946	-0,01751	0,374	0,407	1
Rubcn	0,274032	0,033956	0,426	0,407	1
Mfsd7a	0,274094	-0,00832	0,046	0,059	1

Dctn2	0,274215	0,029444	0,959	0,959	1
Parp3	0,274216	0,004343	0,17	0,15	1
2810474O:	0,274257	-0,00381	0,348	0,385	1
B3galt2	0,274261	-0,00418	0,027	0,037	1
Psmc4	0,274403	-0,0222	0,668	0,694	1
Tshz3	0,274405	-0,01003	0,017	0,024	1
Gm26825	0,274413	0,013343	0,099	0,119	1
Jmjd7	0,274507	-0,01392	0,104	0,122	1
Rpl14	0,274589	0,031846	0,995	0,99	1
Rps6	0,274696	-0,0301	0,989	0,992	1
Agpat5	0,274702	-0,03059	0,503	0,519	1
Gm45844	0,274926	-0,00918	0,049	0,062	1
Gm29994	0,275053	0,008779	0,01	0,005	1
AY074887	0,275053	0,009747	0,01	0,005	1
Nrp2	0,275078	0,029652	0,153	0,135	1
Hsdl2	0,275184	0,041409	0,351	0,337	1
Pitpna	0,275357	-0,01871	0,643	0,683	1
A630089N(	0,27539	-0,00954	0,024	0,033	1
Golph3	0,275391	-0,01597	0,395	0,424	1
Atp11b	0,27542	-0,0284	0,488	0,527	1
Zfp641	0,275424	-0,00734	0,009	0,015	1
Rims1	0,275424	-0,00893	0,009	0,015	1
Fau	0,275425	0,030356	0,997	0,997	1
Enpp4	0,275444	-0,0403	0,931	0,941	1
Cxxc4	0,275516	0,007811	0,01	0,005	1
Wdr66	0,275516	0,007811	0,01	0,005	1
Ska3	0,275516	0,012644	0,01	0,005	1
Gm34590	0,275652	-0,01061	0,084	0,101	1
Mrpl41	0,275797	-0,02882	0,53	0,553	1
Nhp2	0,27584	0,023595	0,322	0,294	1
Rbm18	0,275892	-0,01425	0,407	0,439	1
Pcdh17	0,2759	-0,06508	0,453	0,465	1
Cplx1	0,27598	0,006841	0,01	0,005	1
Gm27253	0,27598	0,006841	0,01	0,005	1
St6galnac2	0,27598	0,010713	0,01	0,005	1
Cage1	0,27608	-0,00817	0,017	0,024	1
Atp6v1h	0,276094	-0,01582	0,585	0,616	1
Cenpn	0,276103	-0,00925	0,046	0,059	1
Rprd1a	0,276117	-0,01739	0,468	0,493	1
Tubb4b	0,276118	0,056223	0,626	0,611	1
Nab2	0,276137	0,011027	0,01	0,005	1
Chek1	0,27622	-0,00921	0,009	0,015	1
Tnip2	0,276386	0,026748	0,147	0,128	1
Dhrs3	0,276445	0,006841	0,01	0,005	1
Bend4	0,276445	0,006841	0,01	0,005	1
Gm37768	0,276445	0,005871	0,01	0,005	1
Pde3b	0,276445	0,005871	0,01	0,005	1
Stac3	0,276445	0,005871	0,01	0,005	1
Rai14	0,276445	0,005871	0,01	0,005	1
Eprs	0,276485	-0,02898	0,815	0,849	1
Srd5a1	0,276502	0,03366	0,433	0,411	1
Dhdh	0,276509	-0,01183	0,081	0,098	1

Sema3b	0,276581	0,039772	0,084	0,07	1
1700112D:	0,276612	-0,00636	0,009	0,015	1
Lrrc4	0,276741	0,035509	0,184	0,166	1
B3gnt4	0,276909	0,0049	0,01	0,005	1
Gm49271	0,276909	0,0049	0,01	0,005	1
Zfp930	0,276914	0,004059	0,079	0,063	1
Vps16	0,276973	-0,03621	0,318	0,335	1
Nup210l	0,277068	0,006194	0,01	0,005	1
Kdm6bos	0,277068	0,006194	0,01	0,005	1
Txndc2	0,277224	0,006516	0,01	0,005	1
Fbxo9	0,27739	0,031556	0,475	0,446	1
Fahd1	0,277506	-0,01569	0,219	0,244	1
Gm13404	0,277614	-0,00335	0,043	0,055	1
Fchsd2	0,277624	0,008046	0,047	0,036	1
Rcan3	0,277632	-0,01444	0,185	0,208	1
Ghitm	0,277663	-0,01875	0,821	0,837	1
Hcfc1	0,277665	-0,00096	0,384	0,424	1
Ralgapa2	0,277744	-0,01745	0,219	0,244	1
Ndufb5	0,277992	-0,01597	0,815	0,834	1
Nuf2	0,278	0,004254	0,01	0,005	1
Rasl12	0,278125	0,010064	0,148	0,128	1
Pcolce2	0,278269	-0,03162	0,677	0,699	1
Ncapd3	0,278394	0,034311	0,384	0,363	1
Ift22	0,278515	-0,00454	0,374	0,413	1
Tnfrsf21	0,278539	-0,01118	0,024	0,033	1
Exog	0,27875	-0,01232	0,092	0,109	1
Lyrm7	0,278778	-0,01956	0,048	0,06	1
Gpr137b	0,278984	-0,01176	0,421	0,459	1
Scn3b	0,278998	-0,00185	0,009	0,015	1
Gm14391	0,279023	-0,01011	0,046	0,059	1
Maged2	0,279049	-0,02027	0,272	0,298	1
2810429I0	0,279052	-0,03433	0,218	0,237	1
Spata1	0,279255	-0,0165	0,075	0,089	1
Gm10785	0,279406	0,001999	0,01	0,005	1
Thrap3	0,279419	-0,01362	0,783	0,808	1
Trappc2	0,279422	-0,03213	0,479	0,498	1
Pttg1ip	0,279424	0,024903	0,712	0,688	1
Tmed9	0,279501	0,031799	0,903	0,881	1
Commd10	0,279674	-0,01421	0,241	0,268	1
Cars2	0,279737	0,023643	0,169	0,148	1
Ifngr2	0,279782	0,021138	0,086	0,072	1
Clptm1l	0,279792	-0,02254	0,551	0,582	1
Amotl2	0,279896	-0,00827	0,688	0,724	1
G730003C:	0,279951	-0,00824	0,013	0,02	1
Pdcl	0,280136	0,034238	0,32	0,298	1
9530068E(	0,280208	0,033639	0,647	0,62	1
Fam149b	0,280271	-0,00139	0,236	0,265	1
Kdm5c	0,280486	0,03165	0,402	0,374	1
Cyp2j12	0,280588	-0,01416	0,389	0,423	1
Zfp869	0,280718	0,014423	0,069	0,055	1
Asna1	0,280772	0,031007	0,515	0,496	1
Dlk2	0,28082	0,021828	0,076	0,062	1

Sipa1l3	0,280863	-0,00981	0,242	0,27	1
Elmsan1	0,281034	-0,01704	0,242	0,268	1
Map2k6	0,281078	-0,00944	0,052	0,065	1
Eif2b3	0,281159	-0,0121	0,303	0,333	1
Mfn1	0,281205	-0,03243	0,58	0,605	1
Tyms	0,281279	-0,00742	0,024	0,033	1
Atp6v1c1	0,281317	-0,0345	0,507	0,524	1
Atf6b	0,281349	0,0282	0,406	0,377	1
Ddx47	0,281383	-0,02111	0,265	0,289	1
Pvr	0,281586	0,01731	0,106	0,089	1
Urod	0,281927	-0,02017	0,396	0,429	1
Dgcr14	0,28202	-0,0147	0,148	0,169	1
Xpa	0,282214	-0,03377	0,364	0,385	1
Wdr36	0,282339	0,018984	0,133	0,115	1
Snx9	0,282398	0,046018	0,366	0,35	1
Cd3eap	0,282423	-0,01549	0,235	0,26	1
Taf1c	0,282514	0,013217	0,069	0,055	1
Gm43915	0,282572	-0,00693	0,013	0,02	1
Slc15a4	0,282585	0,055062	0,684	0,675	1
Dopey2	0,282641	-0,03451	0,487	0,509	1
Arf2	0,28268	-0,01503	0,287	0,317	1
Wdr70	0,282774	-0,02828	0,39	0,416	1
Atp2b2	0,2828	-0,00596	0,013	0,02	1
Ddx27	0,282836	-0,00556	0,302	0,332	1
Ifi27	0,283158	-0,06826	0,818	0,821	1
Psmd11	0,283168	-0,0303	0,789	0,802	1
Ahi1	0,283265	0,025179	0,543	0,502	1
Rpl23	0,283314	0,016189	0,998	0,993	1
4833420G	0,283315	-0,03495	0,344	0,366	1
Hinfp	0,28348	0,038838	0,181	0,163	1
Pcdh10	0,283495	-0,03731	0,518	0,525	1
B3galt4	0,283519	-0,01421	0,078	0,093	1
Clic1	0,283536	0,009297	0,043	0,033	1
Tmem254a	0,283666	0,011847	0,041	0,031	1
Rab23	0,283707	-0,02242	0,3	0,33	1
Cables2	0,283883	0,018856	0,076	0,062	1
Col16a1	0,283974	-0,03358	0,271	0,296	1
Cct8	0,284005	-0,0214	0,81	0,829	1
Smim20	0,284015	-0,03213	0,455	0,48	1
Borcs6	0,284122	-0,01902	0,351	0,384	1
Riok1	0,284185	-0,01496	0,336	0,364	1
Git2	0,284192	-0,03759	0,588	0,598	1
Ccdc13	0,284262	0,045152	0,591	0,564	1
Plpp5	0,284274	0,010577	0,041	0,031	1
Yme1l1	0,284282	0,049425	0,597	0,579	1
Fermt2	0,284415	-0,02358	0,965	0,963	1
Retreg2	0,284444	0,043054	0,731	0,722	1
Brk1	0,284459	-0,01446	0,905	0,909	1
Nfu1	0,284504	-0,02781	0,364	0,389	1
Cramp1l	0,284609	-0,01777	0,201	0,224	1
E430018J2	0,284672	0,014879	0,054	0,042	1
Dmap1	0,284679	-0,01579	0,283	0,309	1

Zfr2	0,284701	0,025673	0,11	0,094	1
Ficd	0,284774	-0,02129	0,116	0,133	1
Heatr6	0,284914	-0,01927	0,294	0,322	1
Zfp974	0,285406	-0,01541	0,08	0,096	1
Wisp2	0,285473	-0,00516	0,003	0,007	1
Gpr174	0,285473	-0,00516	0,003	0,007	1
Apoh	0,285473	-0,00516	0,003	0,007	1
Pcdhga2	0,285473	-0,00516	0,003	0,007	1
Unc5d	0,28569	-0,00579	0,003	0,007	1
Epb41l2	0,285696	-0,02528	0,832	0,842	1
Dnajc28	0,28578	0,012754	0,041	0,031	1
Hs6st1	0,285825	-0,03271	0,309	0,33	1
Alg9	0,285843	-0,00606	0,204	0,229	1
Gm48371	0,285887	-0,01414	0,02	0,028	1
Gata6	0,285906	-0,00642	0,003	0,007	1
Ccm2	0,286001	0,008577	0,278	0,249	1
Rnf123	0,286014	-0,02285	0,189	0,21	1
Ccdc150	0,28612	-0,00354	0,003	0,007	1
6820408C1	0,28612	-0,00354	0,003	0,007	1
Cpne1	0,28612	-0,00354	0,003	0,007	1
Tmod4	0,28612	-0,00354	0,003	0,007	1
Extl1	0,28612	-0,00354	0,003	0,007	1
Ppp2r2cos	0,28612	-0,00354	0,003	0,007	1
0610040J0	0,28612	-0,00354	0,003	0,007	1
Tmem130	0,28612	-0,00354	0,003	0,007	1
Vmn2r29	0,28612	-0,00354	0,003	0,007	1
Olfr715b	0,28612	-0,00354	0,003	0,007	1
Fam57b	0,28612	-0,00354	0,003	0,007	1
Tmem200a	0,28612	-0,00354	0,003	0,007	1
Pbld2	0,28612	-0,00354	0,003	0,007	1
Gm15319	0,28612	-0,00354	0,003	0,007	1
Pnoc	0,28612	-0,00354	0,003	0,007	1
Gm41183	0,28612	-0,00354	0,003	0,007	1
Scel	0,28612	-0,00354	0,003	0,007	1
4930525G1	0,28612	-0,00354	0,003	0,007	1
Ckmt2	0,28612	-0,00354	0,003	0,007	1
Abi3bp	0,28612	-0,00354	0,003	0,007	1
Pcdhb2	0,28612	-0,00354	0,003	0,007	1
Wdcp	0,286163	-0,0208	0,216	0,239	1
Ttc21b	0,28626	0,036198	0,131	0,114	1
Arhgef26	0,286337	-0,00418	0,003	0,007	1
Ephx2	0,286337	-0,00418	0,003	0,007	1
Gm27209	0,286337	-0,00418	0,003	0,007	1
Sox10	0,286382	-0,00617	0,997	1	1
Rps3	0,286391	0,026311	0,996	0,993	1
Tmem178	0,286554	-0,00223	0,003	0,007	1
Tuba1b	0,286585	0,076713	0,8	0,813	1
Spcs1	0,286612	-0,00331	0,897	0,896	1
Tubgcp4	0,286643	-0,00544	0,246	0,275	1
Ptk2b	0,286652	-0,00838	0,039	0,05	1
Plin3	0,286766	-0,09357	0,727	0,715	1
Ccnk	0,286973	-0,03661	0,335	0,351	1

Ly6g5b	0,286986	-0,00159	0,003	0,007	1
Snph	0,286986	-0,00257	0,003	0,007	1
Tmem106a	0,286986	-0,00257	0,003	0,007	1
Bloc1s2	0,287012	-0,02773	0,43	0,452	1
Gm49375	0,287098	0,021615	0,067	0,054	1
Sars2	0,287148	0,017533	0,113	0,096	1
Pdia4	0,287288	-0,04225	0,603	0,613	1
Tmem101	0,287384	0,028422	0,499	0,478	1
Kdm5a	0,287397	0,040819	0,621	0,6	1
Prss35	0,28742	-0,00125	0,003	0,007	1
Ep300	0,287693	0,045292	0,556	0,54	1
Nop14	0,287866	0,037801	0,318	0,291	1
Tbc1d2	0,28792	-0,00797	0,026	0,036	1
L3mbtl3	0,28793	-0,00102	0,03	0,041	1
Wee1	0,288042	0,005221	0,005	0,002	1
Il17rd	0,288042	0,005221	0,005	0,002	1
Ephb3	0,288042	0,005221	0,005	0,002	1
H2-Ab1	0,288042	0,005221	0,005	0,002	1
Gm16233	0,288042	0,007169	0,005	0,002	1
Slc7a5	0,288042	0,007169	0,005	0,002	1
Fgf18	0,288042	0,006195	0,005	0,002	1
Dcp2	0,288049	-0,01945	0,143	0,163	1
Rpl24	0,288246	-0,01464	0,994	1	1
Tbc1d30	0,288268	0,019054	0,037	0,028	1
Mical1	0,288276	0,010083	0,005	0,002	1
Cd24a	0,288276	0,007169	0,005	0,002	1
Ttll8	0,288276	0,007169	0,005	0,002	1
Lcn11	0,288276	0,006195	0,005	0,002	1
Zmynd10	0,288276	0,006195	0,005	0,002	1
Hrk	0,288276	0,005221	0,005	0,002	1
Nynrin	0,288276	0,005221	0,005	0,002	1
Gm26511	0,288276	0,005221	0,005	0,002	1
Exo1	0,288276	0,004246	0,005	0,002	1
Disp1	0,288276	0,004246	0,005	0,002	1
Fam166b	0,288276	0,004246	0,005	0,002	1
Gm9754	0,288276	0,004246	0,005	0,002	1
Slitrk1	0,288276	0,004246	0,005	0,002	1
Gm36198	0,288276	0,004246	0,005	0,002	1
4930556J2	0,288276	0,004246	0,005	0,002	1
Tbx4	0,288276	0,004246	0,005	0,002	1
Nrsn1	0,288276	0,004246	0,005	0,002	1
Tshr	0,288276	0,004246	0,005	0,002	1
Gng13	0,288276	0,004246	0,005	0,002	1
Sgo1	0,288276	0,004246	0,005	0,002	1
2010110K1	0,288276	0,004246	0,005	0,002	1
Ticam2	0,288276	0,004246	0,005	0,002	1
Hrasls5	0,288276	0,004246	0,005	0,002	1
Nab1	0,288324	-0,01876	0,398	0,429	1
Tango2	0,288402	-0,01607	0,336	0,364	1
Crls1	0,288454	-0,01962	0,319	0,346	1
Mrpl20	0,288499	-0,02416	0,816	0,846	1
Tbc1d22a	0,288505	-0,01721	0,183	0,205	1

Spag6	0,28851	0,00327	0,005	0,002	1
Dlx1as	0,28851	0,00327	0,005	0,002	1
Olfr1191-p	0,28851	0,00327	0,005	0,002	1
BC016548	0,28851	0,00327	0,005	0,002	1
Samd10	0,28851	0,00327	0,005	0,002	1
Pfkfb1	0,28851	0,00327	0,005	0,002	1
Gpx7	0,28851	0,00327	0,005	0,002	1
Dmrta2	0,28851	0,00327	0,005	0,002	1
Gm9970	0,28851	0,00327	0,005	0,002	1
Gm43136	0,28851	0,00327	0,005	0,002	1
Hepacam2	0,28851	0,00327	0,005	0,002	1
Irf5	0,28851	0,00327	0,005	0,002	1
Lsr	0,28851	0,00327	0,005	0,002	1
Gm44647	0,28851	0,00327	0,005	0,002	1
Cfap46	0,28851	0,00327	0,005	0,002	1
Elk3	0,28851	0,00327	0,005	0,002	1
Tnfsf13b	0,28851	0,00327	0,005	0,002	1
4930594M	0,28851	0,00327	0,005	0,002	1
A930006L(	0,28851	0,00327	0,005	0,002	1
Tdgf1	0,28851	0,00327	0,005	0,002	1
Gm28048	0,28851	0,00327	0,005	0,002	1
Vmo1	0,28851	0,00327	0,005	0,002	1
Gm11437	0,28851	0,00327	0,005	0,002	1
1700125H:	0,28851	0,00327	0,005	0,002	1
Hoxb7	0,28851	0,00327	0,005	0,002	1
Hoxb2	0,28851	0,00327	0,005	0,002	1
Cdc6	0,28851	0,00327	0,005	0,002	1
Gm11361	0,28851	0,00327	0,005	0,002	1
A330048O(	0,28851	0,00327	0,005	0,002	1
Gm31508	0,28851	0,00327	0,005	0,002	1
Nudt8	0,28851	0,00327	0,005	0,002	1
Clip2	0,288556	0,044774	0,72	0,72	1
Fech	0,288639	-0,01871	0,635	0,652	1
Cox10	0,288646	-0,01326	0,117	0,135	1
Cndp1	0,288746	0,006519	0,005	0,002	1
Pop4	0,288795	-0,02398	0,604	0,636	1
Hmox1	0,288922	0,037538	0,127	0,111	1
Hoxa4	0,288945	-0,02527	0,26	0,283	1
Ubxn1	0,289106	0,030761	0,966	0,954	1
Smim6	0,289215	0,005547	0,005	0,002	1
Gm43403	0,289215	0,003599	0,005	0,002	1
Snrpb	0,289237	0,030638	0,894	0,873	1
Gad2	0,28945	0,002624	0,005	0,002	1
2700069I1	0,28945	0,002624	0,005	0,002	1
Ets1	0,28945	0,002624	0,005	0,002	1
Pth1r	0,28945	0,002624	0,005	0,002	1
Kctd2	0,289524	0,037052	0,619	0,595	1
Kctd20	0,289616	0,037993	0,294	0,275	1
Pou3f2	0,289685	0,001648	0,005	0,002	1
Gm47598	0,289685	0,001648	0,005	0,002	1
Ciita	0,289685	0,001648	0,005	0,002	1
Wnt11	0,289685	-0,00159	0,005	0,002	1

Nop10	0,289724	0,039173	0,551	0,528	1
Ston2	0,28986	-0,02799	0,327	0,346	1
Rps24	0,290015	0,021194	0,998	0,998	1
Rplp2	0,290134	0,025806	0,992	0,997	1
Zdhhc7	0,290209	0,017679	0,172	0,151	1
Arfgef1	0,290254	-0,02878	0,595	0,608	1
Nsfl1c	0,290262	0,026808	0,661	0,646	1
Agap3	0,290392	0,030376	0,386	0,364	1
Copg1	0,290486	-0,03586	0,377	0,4	1
Nfatc2ip	0,290648	-0,02911	0,103	0,119	1
Rdh14	0,290706	-0,00726	0,23	0,257	1
Apln	0,290729	-0,02614	0,375	0,407	1
Hist1h2bc	0,29077	0,080803	0,861	0,873	1
Gm4285	0,290859	0,017075	0,05	0,039	1
D430042O	0,290867	-0,02533	0,241	0,267	1
Gspt1	0,290934	-0,02103	0,743	0,774	1
Ufm1	0,290995	0,033331	0,512	0,502	1
Zcrb1	0,291057	-0,02098	0,685	0,722	1
Nup205	0,291187	-0,01611	0,152	0,172	1
Smim15	0,291342	-0,01312	0,786	0,829	1
Iscu	0,291458	-0,03391	0,461	0,486	1
Gm46447	0,291668	-0,01022	0,057	0,07	1
Optn	0,29177	0,042168	0,371	0,354	1
Ypel4	0,291796	-0,0175	0,116	0,133	1
Pla2g15	0,291798	-0,00852	0,054	0,067	1
Epm2a	0,29182	-0,02279	0,121	0,138	1
Sp1	0,291825	0,04754	0,483	0,475	1
Fcho1	0,291922	-0,0129	0,048	0,06	1
Abhd16a	0,292051	0,035885	0,542	0,53	1
Gon4l	0,292092	-0,03163	0,384	0,405	1
Rnf40	0,292109	-0,01039	0,201	0,226	1
Itm2b	0,292238	-0,01346	0,999	1	1
Szt2	0,292638	-0,02008	0,139	0,158	1
Kidins220	0,292663	-0,02118	0,721	0,745	1
Csnk1a1	0,292754	-0,0185	0,992	1	1
Ypel3	0,292901	-0,017	0,908	0,93	1
Bmi1	0,292904	0,043326	0,513	0,496	1
BC024978	0,292922	-0,01208	0,103	0,12	1
Col20a1	0,292929	0,010062	0,012	0,007	1
Prodh	0,292929	0,012954	0,012	0,007	1
Gm40841	0,29296	-0,00749	0,02	0,028	1
Cbx5	0,293029	0,031404	0,666	0,655	1
Rassf3	0,293087	0,020138	0,107	0,091	1
Ogfr	0,293196	-0,03979	0,374	0,393	1
Slc9a8	0,293327	0,024938	0,265	0,242	1
Ints12	0,293463	-0,01586	0,114	0,132	1
4930404N	0,293511	0,007162	0,012	0,007	1
Pcdha4	0,293511	0,007162	0,012	0,007	1
Rpl29	0,293811	-0,02625	0,966	0,971	1
Abcf1	0,293837	-0,02114	0,725	0,771	1
Gjb1	0,294006	-0,00431	0,977	0,974	1
C2cd2	0,294084	-0,02734	0,206	0,228	1

Gm49396	0,294093	0,007162	0,012	0,007	1
Gm16867	0,294093	0,006194	0,012	0,007	1
Strap	0,294106	-0,02628	0,681	0,699	1
Tbc1d15	0,294266	-0,02433	0,7	0,717	1
Clec2d	0,29439	0,011624	0,012	0,007	1
Ctnnd1	0,29446	-0,01178	0,439	0,473	1
Ncoa2	0,294491	-0,01656	0,556	0,587	1
Pym1	0,294507	-0,0205	0,151	0,171	1
Sod2	0,294524	-0,01861	0,557	0,58	1
Ndufs3	0,294664	-0,02321	0,644	0,685	1
Map9	0,294826	0,00961	0,111	0,094	1
Tmem27	0,294889	-0,00495	0,02	0,028	1
Btbd1	0,294925	-0,02111	0,587	0,621	1
Prpsap2	0,294965	-0,00608	0,372	0,41	1
6720489N	0,295175	-0,01738	0,068	0,081	1
Mbd4	0,295264	-0,00329	0,076	0,091	1
Gm15545	0,295436	0,01218	0,035	0,026	1
Nedd4	0,29546	-0,02144	0,966	0,967	1
Pi4k2b	0,295701	-0,01849	0,125	0,143	1
Gorab	0,295732	-0,01581	0,127	0,145	1
Dhrs4	0,295775	-0,02394	0,215	0,236	1
P2rx7	0,295846	-0,02288	0,272	0,294	1
Ptdss1	0,295859	-0,0109	0,462	0,506	1
Erp29	0,296306	-0,01136	0,717	0,746	1
Unc79	0,296541	-0,00495	0,02	0,028	1
Mgrn1	0,296581	-0,01115	0,501	0,538	1
Saal1	0,296632	-0,01772	0,116	0,133	1
Slc5a3	0,296648	0,050323	0,584	0,546	1
Actr6	0,296765	-0,01859	0,309	0,335	1
Kmt5b	0,296819	0,045255	0,559	0,545	1
Mzt1	0,29697	-0,03043	0,405	0,434	1
Clpb	0,297305	-0,02976	0,376	0,398	1
Zfp770	0,297689	0,025908	0,199	0,179	1
Psmc5	0,297697	-0,01992	0,794	0,802	1
Nicn1	0,297742	-0,01597	0,295	0,322	1
Tmem86a	0,297827	0,012515	0,033	0,024	1
Pno1	0,297841	-0,02318	0,323	0,346	1
Bbc3	0,297987	0,02101	0,128	0,111	1
Hoxd4.1	0,298199	-0,00399	0,02	0,028	1
Zfp780b	0,298251	-0,0326	0,205	0,224	1
Zfp281	0,298256	0,050651	0,681	0,672	1
Tbca	0,298269	-0,02718	0,55	0,574	1
Galnt6	0,298386	-0,03195	0,864	0,885	1
Pigs	0,298448	-0,0229	0,375	0,4	1
AC152827.	0,298603	0,01218	0,033	0,024	1
Celf6	0,298721	-0,0055	0,001	0,003	1
Gm48952	0,298721	-0,0055	0,001	0,003	1
Hsd17b11	0,298726	-0,00398	0,625	0,672	1
Ddx24	0,298804	-0,02267	0,794	0,821	1
Rassf8	0,298877	-0,02806	0,458	0,489	1
Mcrip2	0,298913	-0,01207	0,094	0,111	1
D930016D	0,298965	-0,0227	0,192	0,215	1

Siglec1	0,299061	-0,00389	0,001	0,003	1
Medag	0,299061	-0,00389	0,001	0,003	1
Lsp1	0,299061	-0,00389	0,001	0,003	1
Itgae	0,299061	-0,00389	0,001	0,003	1
Nptx1	0,299061	-0,00389	0,001	0,003	1
Flrt2	0,299061	-0,00389	0,001	0,003	1
Rab15	0,299061	-0,0055	0,001	0,003	1
Ccdc30	0,299061	-0,00712	0,001	0,003	1
Twf2	0,299189	-0,01725	0,269	0,296	1
Timm8a1	0,299238	0,039319	0,368	0,351	1
G6pc3	0,299269	-0,01059	0,429	0,462	1
Pja1	0,299324	-0,019	0,563	0,58	1
Slc25a22	0,299325	0,016576	0,093	0,078	1
Sp140	0,2994	-0,00227	0,001	0,003	1
Map3k19	0,2994	-0,00227	0,001	0,003	1
Klhdc9	0,2994	-0,00227	0,001	0,003	1
Sapcd2	0,2994	-0,00227	0,001	0,003	1
Card9	0,2994	-0,00227	0,001	0,003	1
Traf1	0,2994	-0,00227	0,001	0,003	1
Ptgs1	0,2994	-0,00227	0,001	0,003	1
Gm26558	0,2994	-0,00227	0,001	0,003	1
Calcrl	0,2994	-0,00227	0,001	0,003	1
Adam33	0,2994	-0,00227	0,001	0,003	1
Nrsn2	0,2994	-0,00227	0,001	0,003	1
Cdh22	0,2994	-0,00227	0,001	0,003	1
Gpr82	0,2994	-0,00227	0,001	0,003	1
Dcaf12l1	0,2994	-0,00227	0,001	0,003	1
Gabre	0,2994	-0,00227	0,001	0,003	1
Arhgap6	0,2994	-0,00227	0,001	0,003	1
Gm16337	0,2994	-0,00227	0,001	0,003	1
Mir124-2hg	0,2994	-0,00227	0,001	0,003	1
Efna3	0,2994	-0,00227	0,001	0,003	1
Kcnc4	0,2994	-0,00227	0,001	0,003	1
Car8	0,2994	-0,00227	0,001	0,003	1
Bach2os	0,2994	-0,00227	0,001	0,003	1
Kif2c	0,2994	-0,00227	0,001	0,003	1
Dlgap3	0,2994	-0,00227	0,001	0,003	1
Miat	0,2994	-0,00227	0,001	0,003	1
Mdfic	0,2994	-0,00227	0,001	0,003	1
Prr15	0,2994	-0,00227	0,001	0,003	1
0610040F(	0,2994	-0,00227	0,001	0,003	1
Mfap5	0,2994	-0,00227	0,001	0,003	1
Apobec1	0,2994	-0,00227	0,001	0,003	1
Pianp	0,2994	-0,00227	0,001	0,003	1
Grin2d	0,2994	-0,00227	0,001	0,003	1
BC046251	0,2994	-0,00227	0,001	0,003	1
Calca	0,2994	-0,00227	0,001	0,003	1
Samd5	0,2994	-0,00227	0,001	0,003	1
Gstt1	0,2994	-0,00227	0,001	0,003	1
Smim24	0,2994	-0,00227	0,001	0,003	1
Ptprr	0,2994	-0,00227	0,001	0,003	1
Fam155a	0,2994	-0,00227	0,001	0,003	1

Gm26632	0,2994	-0,00227	0,001	0,003	1
Nrp1	0,2994	-0,00227	0,001	0,003	1
4930579G	0,2994	-0,00227	0,001	0,003	1
Ebf2	0,2994	-0,00227	0,001	0,003	1
Scn2b	0,2994	-0,00227	0,001	0,003	1
Skor1	0,2994	-0,00227	0,001	0,003	1
Gm47270	0,2994	-0,00227	0,001	0,003	1
Rbpms2	0,2994	-0,00227	0,001	0,003	1
Rab27a	0,2994	-0,00227	0,001	0,003	1
Tbkbp1	0,2994	-0,00227	0,001	0,003	1
Hist1h2bg	0,2994	-0,00227	0,001	0,003	1
Ly6e	0,2994	-0,00227	0,001	0,003	1
Itgb7	0,2994	-0,00227	0,001	0,003	1
Nrros	0,2994	-0,00227	0,001	0,003	1
Cxadr	0,2994	-0,00227	0,001	0,003	1
Gm11131	0,2994	-0,00227	0,001	0,003	1
Rgp1	0,299423	0,025086	0,149	0,132	1
Zfp318	0,29951	-0,02348	0,298	0,322	1
Zfp114	0,299701	-0,01055	0,057	0,07	1
Zcchc12	0,29974	-0,00291	0,001	0,003	1
Evpl	0,29974	-0,00291	0,001	0,003	1
Adat2	0,300015	-0,01521	0,071	0,085	1
Slc23a2	0,300043	-0,03559	0,205	0,223	1
Tmprss7	0,300081	0,000671	0,001	0,003	1
Atg9b	0,300081	-0,00031	0,001	0,003	1
Adm	0,300081	-0,00031	0,001	0,003	1
Ptpru	0,300081	-0,00129	0,001	0,003	1
Hpgd	0,300081	-0,00129	0,001	0,003	1
5033426O	0,300081	-0,00129	0,001	0,003	1
Gpc6	0,300081	-0,00129	0,001	0,003	1
Ankzf1	0,300324	0,021138	0,153	0,135	1
Man1a	0,300416	-0,01158	0,113	0,094	1
9430015G	0,300429	-0,00802	0,109	0,127	1
Gbp5	0,300632	-0,00775	0,002	0,005	1
Ifi47	0,300632	-0,01957	0,002	0,005	1
Cebpg	0,300897	-0,00183	0,429	0,463	1
Strip2	0,300925	0,013355	0,035	0,026	1
Thoc7	0,30095	-0,0165	0,458	0,483	1
Cfap20	0,300962	0,031513	0,397	0,374	1
Fmnl3	0,301055	-0,00574	0,029	0,039	1
Gm28981	0,30116	-0,00452	0,002	0,005	1
Gm14401	0,30116	-0,00452	0,002	0,005	1
Crybb3	0,30116	-0,00452	0,002	0,005	1
5730422E	0,30116	-0,00452	0,002	0,005	1
Klhl33	0,30116	-0,00452	0,002	0,005	1
Gm11963	0,30116	-0,00452	0,002	0,005	1
Gm10447	0,30116	-0,00452	0,002	0,005	1
Gm20324	0,30116	-0,00452	0,002	0,005	1
Gm29019	0,30116	-0,00452	0,002	0,005	1
Zfp57	0,30116	-0,00452	0,002	0,005	1
Kcnj3	0,30116	-0,00936	0,002	0,005	1
Gab1	0,3012	0,031414	0,966	0,95	1

Ddx3x	0,301233	0,090967	0,818	0,816	1
Bpnt1	0,301261	-0,01616	0,626	0,657	1
Yy1	0,30129	-0,02012	0,747	0,777	1
Tceanc	0,301326	0,010365	0,086	0,072	1
0610043K1	0,301425	-0,00516	0,002	0,005	1
Gm38033	0,301689	-0,00291	0,002	0,005	1
Pkp1	0,301689	-0,00291	0,002	0,005	1
Cd247	0,301689	-0,00291	0,002	0,005	1
Esrrg	0,301689	-0,00291	0,002	0,005	1
Dlx1	0,301689	-0,00291	0,002	0,005	1
Pde1a	0,301689	-0,00291	0,002	0,005	1
Oip5	0,301689	-0,00291	0,002	0,005	1
Gm14978	0,301689	-0,00291	0,002	0,005	1
Pax1	0,301689	-0,00291	0,002	0,005	1
Vstm2l	0,301689	-0,00291	0,002	0,005	1
Eda	0,301689	-0,00291	0,002	0,005	1
C030034L1	0,301689	-0,00291	0,002	0,005	1
Ccna2	0,301689	-0,00291	0,002	0,005	1
Gm20633	0,301689	-0,00291	0,002	0,005	1
Bcl2l15	0,301689	-0,00291	0,002	0,005	1
Matn1	0,301689	-0,00291	0,002	0,005	1
Lhx5	0,301689	-0,00291	0,002	0,005	1
Slc4a5	0,301689	-0,00291	0,002	0,005	1
Cxcl12	0,301689	-0,00291	0,002	0,005	1
Gm15687	0,301689	-0,00291	0,002	0,005	1
C5ar2	0,301689	-0,00291	0,002	0,005	1
Hif3a	0,301689	-0,00291	0,002	0,005	1
Il16	0,301689	-0,00291	0,002	0,005	1
Aire	0,301689	-0,00291	0,002	0,005	1
Rdh16	0,301689	-0,00291	0,002	0,005	1
Car5a	0,301689	-0,00291	0,002	0,005	1
Gm20735	0,301689	-0,00291	0,002	0,005	1
Thtpa	0,301689	-0,00291	0,002	0,005	1
Tgm4	0,301689	-0,00291	0,002	0,005	1
Olfr392	0,301689	-0,00291	0,002	0,005	1
Spata20	0,301689	-0,00291	0,002	0,005	1
Gm11739	0,301689	-0,00291	0,002	0,005	1
Hist1h2ad	0,301689	-0,00291	0,002	0,005	1
Gm48707	0,301689	-0,00291	0,002	0,005	1
Gm47467	0,301689	-0,00291	0,002	0,005	1
C1qtnf6	0,301689	-0,00291	0,002	0,005	1
Gtse1	0,301689	-0,00291	0,002	0,005	1
D930030I0	0,301689	-0,00291	0,002	0,005	1
Olfr204	0,301689	-0,00291	0,002	0,005	1
Igsf5	0,301689	-0,00291	0,002	0,005	1
Gm6594	0,301689	-0,00291	0,002	0,005	1
Camk2a	0,301689	-0,00291	0,002	0,005	1
Lpxn	0,301689	-0,00291	0,002	0,005	1
Gabbr2	0,301955	-0,00354	0,002	0,005	1
Rbakdn	0,301955	-0,00354	0,002	0,005	1
Gm26542	0,301955	-0,00354	0,002	0,005	1
Ms4a7	0,301955	-0,00354	0,002	0,005	1

Kctd10	0,301962	0,019509	0,094	0,08	1
Dmrtc1a	0,302011	0,009983	0,033	0,024	1
Mreg	0,302014	0,012515	0,031	0,023	1
Acyp2	0,302048	-0,0011	0,608	0,65	1
Ssx2ip	0,30217	0,019196	0,07	0,057	1
Tm6sf1	0,302193	0,011236	0,033	0,024	1
Tef	0,302212	0,037921	0,579	0,571	1
Dctpp1	0,302264	-0,01911	0,326	0,351	1
Hbegf	0,302371	0,044343	0,086	0,073	1
Vtcn1	0,302485	-0,00193	0,002	0,005	1
Atg4a-ps	0,302485	-0,00193	0,002	0,005	1
Gm11266	0,302485	-0,00193	0,002	0,005	1
Sspnos	0,302485	-0,00193	0,002	0,005	1
Gabrb3	0,302485	-0,00193	0,002	0,005	1
Zic1	0,302485	-0,00193	0,002	0,005	1
4930500F1	0,302485	-0,00193	0,002	0,005	1
3110006O(	0,302485	-0,00193	0,002	0,005	1
AC150035.	0,302485	-0,00193	0,002	0,005	1
March3	0,302485	-0,00193	0,002	0,005	1
Atad3aos	0,302538	-0,01313	0,082	0,098	1
Ptbp3	0,302594	-0,01052	0,452	0,488	1
Kmt2b	0,302775	-0,03189	0,313	0,333	1
Twf1	0,302903	-0,03446	0,8	0,813	1
Hars	0,30292	-0,01026	0,527	0,564	1
Foxp4	0,302927	0,020803	0,105	0,089	1
Acsbg1	0,302933	-0,00721	0,016	0,023	1
Glyr1	0,302979	-0,01425	0,569	0,598	1
Errfi1	0,30318	-0,04539	0,633	0,644	1
Cdk5rap3	0,303192	-0,02189	0,342	0,369	1
Fam83h	0,303281	-0,00095	0,002	0,005	1
Slc7a4	0,303281	-0,00095	0,002	0,005	1
Prkdc	0,303296	-0,02294	0,232	0,255	1
Gm45051	0,303402	-0,00669	0,032	0,042	1
Klf4	0,303417	0,222458	0,18	0,163	1
Birc2	0,303425	-0,0087	0,388	0,429	1
Etohd2	0,303541	0,013653	0,048	0,037	1
Ppp1r35	0,30355	-0,03495	0,311	0,33	1
Fto	0,303599	0,034756	0,651	0,629	1
Eaf2	0,303792	0,016342	0,027	0,02	1
Zfp971	0,303804	-0,0118	0,041	0,052	1
Cyb5a	0,303838	-0,00791	0,873	0,902	1
Ipo13	0,303897	-0,01292	0,704	0,737	1
Dcaf12	0,30391	0,04369	0,327	0,314	1
Uba1	0,304064	0,036847	0,755	0,748	1
Dpagt1	0,304079	-0,01155	0,128	0,146	1
Dnah7b	0,304133	-0,00671	0,008	0,013	1
Itpripl2	0,30428	0,019947	0,05	0,039	1
Gm14305	0,304414	-0,01974	0,229	0,25	1
Gm15706	0,304501	0,01753	0,031	0,023	1
Astn1	0,304572	-0,00562	0,016	0,023	1
Vti1a	0,304649	-0,01962	0,31	0,335	1
A330102I1	0,304871	0,008765	0,014	0,008	1

Yap1	0,305009	0,007801	0,014	0,008	1
AU041133	0,305063	-0,00767	0,072	0,086	1
Cops6	0,305078	-0,02102	0,725	0,774	1
Nup210	0,305118	0,016014	0,089	0,075	1
Clec11a	0,305141	0,007482	0,014	0,008	1
Gm15743	0,305141	0,006516	0,014	0,008	1
1700010I1	0,305141	0,006516	0,014	0,008	1
Cnnm2	0,305261	-0,01271	0,132	0,151	1
4732419C:	0,305327	-0,0051	0,008	0,013	1
Erbb2	0,305327	-0,0051	0,008	0,013	1
Zfp773	0,305345	-0,01419	0,108	0,125	1
Paxbp1	0,305381	0,002845	0,371	0,411	1
Elof1	0,305495	-0,0127	0,443	0,476	1
Frrs1	0,305631	-0,00539	0,008	0,013	1
AC161165.	0,305695	0,006837	0,014	0,008	1
Csnk1g2	0,305817	0,03649	0,412	0,393	1
Txk	0,305827	0,005548	0,014	0,008	1
5033430I1	0,305827	0,005548	0,014	0,008	1
Zbtb14	0,305856	-0,02393	0,125	0,141	1
Vim	0,305898	0,037948	0,092	0,078	1
Abr	0,306111	-0,00466	0,016	0,023	1
Cyth3	0,306161	-0,03696	0,222	0,241	1
1700028K(	0,306245	0,006837	0,014	0,008	1
Gm26836	0,306383	0,005871	0,014	0,008	1
Ap5b1	0,306383	0,005871	0,014	0,008	1
Zkscan4	0,306489	0,006186	0,035	0,026	1
Gm10076	0,306556	-0,01466	0,167	0,187	1
Nsun4	0,306649	-0,01223	0,079	0,094	1
Fam46c	0,30668	-0,00088	0,008	0,013	1
Pygm	0,30668	-0,00379	0,008	0,013	1
Blzf1	0,307008	-0,01186	0,236	0,26	1
Ccdc169	0,307016	-0,0108	0,012	0,018	1
Kti12	0,307031	-0,01216	0,102	0,119	1
Mcts1	0,307293	0,021138	0,391	0,359	1
Ift27	0,307316	-0,00973	0,365	0,403	1
Cul7	0,307506	0,012714	0,136	0,119	1
Farp1	0,307612	-0,02469	0,086	0,101	1
Rrm1	0,307684	0,038461	0,277	0,259	1
Fam206a	0,307953	-0,01554	0,168	0,189	1
Usp35	0,308155	-0,00375	0,121	0,14	1
Gm43672	0,30822	0,016195	0,044	0,034	1
Tdo2	0,30822	0,016195	0,044	0,034	1
Arl6ip4	0,308359	-0,01907	0,44	0,472	1
Abca8b	0,308458	0,009886	0,282	0,254	1
Bcl7a	0,308485	-0,00379	0,598	0,636	1
Ifrd2	0,308534	0,022012	0,108	0,093	1
Morn1	0,308634	-0,00912	0,038	0,049	1
Pald1	0,30865	0,018397	0,151	0,133	1
Vps51	0,308749	0,014921	0,114	0,098	1
Hsd3b2	0,3088	0,008077	0,031	0,023	1
Il18bp	0,308837	-0,01205	0,026	0,034	1
Map3k10	0,308866	-0,02072	0,21	0,231	1

Lgals2	0,308924	0,016985	0,102	0,086	1
Hnrnpf	0,309001	0,035642	0,943	0,935	1
Fkbp7	0,309005	-0,01171	0,044	0,055	1
Grik1	0,309089	0,029047	0,24	0,216	1
Phldb2	0,309293	-0,00037	0,032	0,042	1
Zfp719	0,309432	-0,0165	0,079	0,093	1
Fam162a	0,309592	-0,01266	0,356	0,392	1
Sestd1	0,309611	-0,02572	0,121	0,137	1
Clec2l	0,30979	-0,00727	0,012	0,018	1
Slc17a9	0,309958	0,01224	0,026	0,018	1
Afdn	0,310072	0,020274	0,682	0,662	1
Gpaa1	0,310142	-0,01241	0,302	0,328	1
Jam2	0,310174	0,020098	0,075	0,062	1
Slc6a7	0,310402	0,010002	0,014	0,008	1
4930579G:	0,310638	0,011635	0,123	0,106	1
Ppp1r3e	0,310832	-0,01367	0,163	0,184	1
Usp53	0,310926	-0,00665	0,247	0,272	1
Arl4c	0,310942	0,009851	0,05	0,039	1
Gm16740	0,310946	-0,01171	0,047	0,059	1
Gtf2h1	0,311066	-0,02043	0,287	0,312	1
Polr1b	0,311097	0,017567	0,085	0,072	1
Smyd4	0,311109	-0,01779	0,154	0,172	1
Dnmbp	0,31118	-0,0063	0,012	0,018	1
Atp6v1b2	0,311205	-0,02596	0,631	0,646	1
Armc7	0,311206	0,017781	0,066	0,054	1
Lrrfip1	0,311273	0,020055	0,027	0,02	1
BC037039	0,311307	-0,00568	0,012	0,018	1
Clcn4	0,311355	-0,02201	0,882	0,872	1
Mkln1os	0,31143	-0,00505	0,012	0,018	1
Gnb1l	0,31143	-0,00505	0,012	0,018	1
Slc35a1	0,311611	-0,02612	0,201	0,221	1
Wdr46	0,31171	0,028397	0,217	0,198	1
Usp48	0,31179	-0,05812	0,523	0,537	1
Nbl1	0,311853	0,007801	0,016	0,01	1
Ndufs4	0,311872	-0,0281	0,68	0,691	1
Mpp1	0,312161	-0,02684	0,168	0,187	1
Ppil3	0,312185	-0,01862	0,268	0,293	1
Acy1	0,312315	-0,01283	0,345	0,379	1
Zfp691	0,312389	-0,00381	0,063	0,076	1
Snrpa1	0,312792	0,015048	0,311	0,285	1
Entpd7	0,313013	-0,0068	0,085	0,101	1
Gm17382	0,313024	0,01292	0,018	0,011	1
Rpp21	0,313033	-0,01868	0,421	0,446	1
Tmem184b	0,31309	-0,03269	0,45	0,47	1
Coq5	0,31311	0,02212	0,352	0,325	1
Vwa3b	0,313155	0,006192	0,016	0,01	1
Chchd10	0,313172	-0,14901	0,222	0,236	1
Otud3	0,313383	0,008098	0,027	0,02	1
Klhl29	0,313383	0,008098	0,027	0,02	1
Cklf	0,313449	-0,00175	0,121	0,14	1
Cnpy4	0,313562	-0,01794	0,19	0,211	1
Bpgm	0,313598	-0,02662	0,974	0,98	1

Ccnjl	0,313614	-0,01049	0,028	0,037	1
Dnase2a	0,313742	0,037404	0,096	0,083	1
Phlda3	0,313925	0,037764	0,912	0,932	1
Csad	0,314107	-0,02973	0,24	0,26	1
Cdc7	0,314125	0,00857	0,082	0,068	1
Gm3448	0,314171	0,030175	0,124	0,107	1
Camk4	0,314231	-0,00022	0,012	0,018	1
Polr3c	0,314326	-0,0075	0,144	0,164	1
Stk11ip	0,31446	-0,02241	0,111	0,127	1
Tmcc1	0,314464	-0,01995	0,899	0,896	1
Kif7	0,314486	0,01224	0,024	0,016	1
Ten1	0,314526	-0,0256	0,595	0,626	1
Samd9l	0,31456	-0,00831	0,026	0,034	1
Tusc2	0,314607	-0,0258	0,34	0,363	1
Crtc3	0,314889	0,040062	0,355	0,335	1
AI413582	0,314911	-0,02834	0,56	0,584	1
Rbmxl1	0,31523	-0,02349	0,386	0,408	1
Mef2b	0,315323	0,013534	0,022	0,015	1
Tmem255a	0,315512	-0,00278	0,012	0,018	1
Gins1	0,315538	-0,00613	0,026	0,034	1
Gm28529	0,315589	0,008119	0,018	0,011	1
Spry3	0,315589	0,007156	0,018	0,011	1
Rpe	0,31577	-0,01855	0,368	0,392	1
Gcdh	0,315906	-0,03511	0,231	0,25	1
Adgrg2	0,315941	-0,00688	0,05	0,062	1
Eipr1	0,315988	-0,02261	0,242	0,263	1
Pkd2l2	0,316085	0,007475	0,018	0,011	1
Gm47101	0,316089	0,010667	0,018	0,011	1
Gatc	0,316326	-0,01607	0,196	0,216	1
Brms1l	0,316349	0,035703	0,324	0,306	1
Idh3b	0,316417	0,047027	0,506	0,498	1
Cdk4	0,316455	0,023581	0,495	0,468	1
Lasp1	0,316458	-0,01265	0,028	0,037	1
Jpx	0,316459	-0,0084	0,153	0,174	1
Ddost	0,31657	0,037694	0,742	0,74	1
4921504A1	0,316573	-0,00621	0,149	0,169	1
Lrp8	0,31683	0,009691	0,018	0,011	1
Nploc4	0,316838	-0,01332	0,293	0,322	1
Tmem94	0,31685	-0,01902	0,128	0,145	1
Zbtb11	0,316861	0,053451	0,459	0,449	1
Adsl	0,316875	-0,01703	0,229	0,25	1
Tbk1	0,31692	-0,02193	0,258	0,281	1
Gm26670	0,317309	0,007792	0,022	0,015	1
Dnajb5	0,317317	-0,01183	0,09	0,106	1
Etv6	0,317401	-0,01323	0,059	0,072	1
Tspan32	0,317438	-0,00423	0,026	0,034	1
Tuba1a	0,317468	0,041461	0,999	1	1
Gm16133	0,317556	0,009051	0,024	0,016	1
Psmd5	0,317661	-0,00975	0,249	0,275	1
Smg1	0,317672	-0,01838	0,434	0,455	1
Gm20517	0,317759	0,008108	0,022	0,015	1
Mid1	0,317805	0,00555	0,018	0,011	1

Rpl23a	0,317901	-0,02887	0,959	0,946	1
Herc4	0,317942	-0,02083	0,391	0,42	1
Glt1d1	0,317947	0,011819	0,044	0,034	1
Dlat	0,318024	-0,01094	0,264	0,291	1
Drosha	0,318348	-0,01829	0,289	0,315	1
Rmnd5a	0,318446	0,066832	0,494	0,499	1
Rab12	0,318483	0,037146	0,397	0,376	1
Phc2	0,31865	0,025969	0,842	0,833	1
Mrps22	0,318669	-0,01876	0,222	0,244	1
Nod1	0,318932	-0,02106	0,224	0,246	1
Polr3k	0,318981	-0,01518	0,3	0,327	1
Zfp629	0,319006	-0,00952	0,096	0,112	1
Plcxd2	0,319092	0,00715	0,02	0,013	1
Rab6b	0,31914	0,059588	0,643	0,624	1
Cox8a	0,319168	0,032205	0,992	0,995	1
Eif4ebp2	0,319309	-0,02811	0,234	0,252	1
Fignl1	0,319553	0,005871	0,02	0,013	1
Exoc8	0,31959	-0,00999	0,161	0,182	1
Lctl	0,320089	-0,0117	0,031	0,041	1
Ets2	0,320148	0,007467	0,02	0,013	1
Ttc27	0,320226	-0,01745	0,1	0,115	1
Psmg4	0,320236	-0,03457	0,367	0,389	1
Nhej1	0,320249	0,010511	0,057	0,046	1
Man2b2	0,320553	-0,01482	0,126	0,143	1
Myo5a	0,320725	0,00771	0,284	0,317	1
Aldh9a1	0,320749	-0,00911	0,427	0,457	1
Ctsf	0,320889	-0,02648	0,478	0,504	1
Acsl3	0,320967	-0,00682	0,75	0,789	1
Fbxo28	0,321083	0,030739	0,549	0,528	1
Pter	0,32114	-0,01462	0,197	0,22	1
Nfkbil1	0,321176	-0,00846	0,177	0,198	1
Pcmtd1	0,321262	-0,01376	0,815	0,844	1
Smc6	0,321319	-0,02548	0,647	0,675	1
Tmem60	0,321323	-0,02885	0,539	0,556	1
1700037C:	0,321499	0,017665	0,151	0,133	1
Erc1	0,321829	-0,01211	0,165	0,185	1
Arel1	0,322171	-0,03256	0,302	0,32	1
Prkce	0,322225	0,021842	0,053	0,042	1
Ttc1	0,322319	-0,02104	0,553	0,597	1
Smad4	0,322569	0,036534	0,456	0,439	1
Zfp933	0,322572	-0,00803	0,127	0,145	1
Prelid1	0,322588	0,018564	0,527	0,501	1
Gm16124	0,322669	-0,00696	0,031	0,041	1
Acaca	0,322898	-0,01565	0,812	0,82	1
Stk35	0,32298	-0,01527	0,128	0,145	1
Lims2	0,323062	-0,02572	0,255	0,276	1
Plpp1	0,323193	-0,01209	0,134	0,153	1
Matr3	0,323257	0,041608	0,951	0,961	1
Sirpa	0,323306	0,025153	0,1	0,086	1
9430060I0	0,323358	-0,00079	0,026	0,018	1
Tbc1d17	0,323597	-0,03385	0,584	0,6	1
Psmd2	0,323618	0,026388	0,79	0,779	1

Saxo2	0,323856	-0,02241	0,102	0,117	1
Rmnd5b	0,323879	-0,01133	0,203	0,224	1
Pim3	0,323912	0,067995	0,824	0,826	1
Ddx19a	0,324224	0,030144	0,276	0,257	1
Gm7598	0,324446	-0,0112	0,034	0,044	1
Bola3	0,324485	-0,00843	0,358	0,387	1
Pkn2	0,324646	-0,01752	0,635	0,66	1
Scamp3	0,324654	-0,02895	0,523	0,538	1
Lage3	0,324701	-0,0313	0,416	0,436	1
Zdhhc5	0,32472	-0,01985	0,175	0,193	1
Vps26b	0,324779	-0,03142	0,275	0,294	1
H2-Q7	0,325103	-0,0327	0,022	0,029	1
Vhl	0,325166	0,019671	0,279	0,255	1
Psme3	0,325211	-0,01275	0,249	0,276	1
Higd1a	0,325268	-0,01278	0,679	0,714	1
Mrpl38	0,325347	0,054179	0,359	0,346	1
AI464131	0,32548	-0,01393	0,043	0,054	1
Fam107b	0,325525	-0,01013	0,2	0,223	1
Arhgap39	0,325541	-0,01392	0,436	0,467	1
Tstd3	0,325612	0,012566	0,075	0,062	1
Otub2	0,325636	-0,02185	0,066	0,078	1
Cep295	0,325867	-0,02134	0,295	0,322	1
Bcl2l1	0,326112	-0,00712	0,749	0,797	1
Tubb2a	0,326119	-0,00662	0,223	0,25	1
Jrkl	0,32622	0,032767	0,238	0,22	1
Prdx5	0,326228	0,041398	0,704	0,717	1
2700097O(	0,326297	-0,0199	0,27	0,293	1
Pepd	0,326347	0,034706	0,548	0,527	1
Adck1	0,326365	-0,01182	0,197	0,22	1
Mien1	0,32644	0,016185	0,838	0,854	1
Gm26879	0,326619	0,01339	0,04	0,031	1
Alg6	0,326698	-0,02777	0,192	0,211	1
Klk6	0,326809	-0,11225	0,866	0,789	1
Zfp963	0,32686	-0,00602	0,031	0,041	1
Zfp266	0,327065	-0,02781	0,577	0,598	1
Trp53inp2	0,327145	0,004509	0,294	0,327	1
Actr3b	0,327174	-0,01342	0,037	0,047	1
Mcm7	0,327192	0,025065	0,235	0,215	1
Togaram1	0,327204	-0,01477	0,175	0,195	1
Col11a2	0,327237	0,04178	0,243	0,223	1
Golga1	0,327237	0,046106	0,329	0,314	1
Mettl5os	0,327344	-0,01327	0,015	0,021	1
A930003A:	0,327586	0,010573	0,094	0,08	1
Eif1ad	0,327611	-0,01179	0,241	0,265	1
Gm3435	0,327612	-0,01358	0,046	0,057	1
Tex30	0,327717	0,015516	0,108	0,093	1
Diablo	0,327803	-0,02117	0,637	0,665	1
Tesk2	0,327861	0,027926	0,326	0,302	1
Wdr1	0,328027	-0,00244	0,549	0,582	1
Ankrd12	0,328058	0,035846	0,875	0,86	1
Zbtb20	0,328121	0,043383	0,991	0,993	1
Fam172a	0,328143	-0,02586	0,605	0,6	1

Ccng2	0,32829	0,002925	0,247	0,275	1
Gak	0,328419	-0,00204	0,296	0,327	1
Ice2	0,328436	-0,00367	0,2	0,224	1
Rnaseh2a	0,328638	-0,01312	0,205	0,228	1
Lypla2	0,328668	-0,0027	0,236	0,26	1
Tmem177	0,329032	-0,01214	0,04	0,05	1
Casc1	0,329271	-0,01091	0,022	0,029	1
Tars	0,329292	-0,02841	0,242	0,26	1
Hps3	0,3293	-0,01614	0,2	0,221	1
Rgs7	0,329482	-0,00948	0,015	0,021	1
Pask	0,329482	-0,01107	0,015	0,021	1
Gm16287	0,329637	0,010271	0,04	0,031	1
1700021F(	0,329666	0,006953	0,182	0,161	1
Fa2h	0,329895	0,015811	0,996	0,997	1
Hdac8	0,329899	-0,01861	0,156	0,174	1
U2af2	0,329947	0,020285	0,611	0,577	1
Emilin2	0,329957	0,036121	0,473	0,454	1
Ebna1bp2	0,330014	0,037279	0,489	0,48	1
Nubpl	0,330143	-0,01764	0,078	0,091	1
Gzf1	0,330146	-0,01611	0,198	0,22	1
Rchy1	0,330154	0,024724	0,622	0,589	1
Ate1	0,330161	0,024723	0,581	0,554	1
Pold2	0,330276	-0,00793	0,258	0,285	1
Pcdh11x	0,33029	-0,02998	0,156	0,172	1
Srf	0,330292	0,030762	0,187	0,171	1
Cr1l	0,330477	-0,02176	0,395	0,423	1
Ndn	0,330551	-0,02274	0,24	0,262	1
Zbtb25	0,330621	0,027914	0,119	0,104	1
Cdk10	0,330745	-0,01651	0,179	0,198	1
Mphosph6	0,330856	0,015231	0,422	0,397	1
Akip1	0,330898	0,017753	0,118	0,102	1
Whamm	0,330939	0,020888	0,156	0,14	1
Bicra	0,331106	-0,01762	0,179	0,198	1
Dyrk1a	0,331138	0,032815	0,417	0,402	1
Rab24	0,331199	-0,01736	0,41	0,442	1
Ints5.1	0,331259	0,013489	0,108	0,093	1
Sae1	0,331394	-0,00745	0,261	0,288	1
Gtpbp10	0,331425	-0,01113	0,112	0,128	1
6330403L(	0,331693	-0,0139	0,106	0,122	1
Ints14	0,331716	0,026091	0,151	0,135	1
Stt3b	0,331871	-0,00957	0,388	0,421	1
Ank3	0,331909	-0,01976	0,987	0,995	1
Tmem121	0,331945	-0,01327	0,204	0,226	1
Pcid2	0,331965	0,030626	0,261	0,242	1
Sept3	0,331981	-0,00681	0,022	0,029	1
Larp4b	0,332022	0,049829	0,567	0,553	1
Tmem91	0,332094	-0,01205	0,022	0,029	1
Arhgap19	0,332278	-0,00523	0,022	0,029	1
2810455O(	0,332573	-0,00653	0,022	0,029	1
Adgrb1	0,332776	0,023146	0,22	0,2	1
Dlg3	0,332891	-0,0058	0,07	0,083	1
Fis1	0,332894	-0,01665	0,986	0,998	1

Cadm3	0,332921	-0,01924	0,142	0,159	1
Cd55	0,332956	-0,0143	0,025	0,033	1
Piezo2	0,333002	-0,02839	0,2	0,218	1
Gm14419	0,333028	-0,00905	0,04	0,05	1
Lrch2	0,333069	-0,00056	0,223	0,249	1
Gemin7	0,333224	0,030825	0,423	0,407	1
Ctps	0,333398	-0,00967	0,154	0,172	1
Grik3	0,333502	-0,00484	0,022	0,029	1
Cyp4f16	0,333569	-0,0166	0,146	0,164	1
Fitm2	0,333777	-0,00965	0,19	0,211	1
Mphosph1(	0,333802	-0,03004	0,397	0,421	1
Etfdh	0,333804	0,030131	0,261	0,242	1
Slc30a5	0,333825	0,009628	0,299	0,281	1
Sgsm2	0,333829	-0,00561	0,106	0,122	1
Ercc3	0,333888	0,022049	0,208	0,189	1
Tatdn3	0,333894	-0,00912	0,148	0,167	1
Ubtf	0,333977	0,036176	0,632	0,607	1
Ywhae	0,334014	0,012873	0,999	1	1
Ap3m2	0,334057	-0,0217	0,206	0,226	1
Pkn1	0,334206	-0,01754	0,362	0,387	1
Tra2b	0,334208	0,083224	0,825	0,857	1
Cstad	0,334523	-0,00211	0,015	0,021	1
Sec61b	0,334617	-0,02219	0,597	0,624	1
Gm27017	0,33464	-0,00534	0,015	0,021	1
Esyt2	0,334654	-0,01695	0,334	0,366	1
Ajap1	0,334765	-0,00602	0,044	0,034	1
Pdcd6	0,334781	-0,01114	0,681	0,712	1
Snx32	0,335	0,03581	0,6	0,572	1
Hectd4	0,335135	-0,00871	0,33	0,356	1
Acer3	0,335151	-0,01302	0,117	0,133	1
Mylip	0,335165	-0,01879	0,094	0,109	1
Tbc1d4	0,335222	-0,01574	0,078	0,091	1
Gm7173	0,335559	-0,00768	0,007	0,011	1
Rassf7	0,335559	-0,00768	0,007	0,011	1
Eci2	0,335666	0,026804	0,4	0,385	1
Gm48508	0,33573	-0,01934	0,142	0,159	1
Sec61g	0,335873	-0,04146	0,892	0,901	1
Ak3	0,335894	-0,01759	0,901	0,894	1
Arfip2	0,336146	-0,01699	0,421	0,447	1
Acad10	0,336224	-0,00461	0,022	0,029	1
Slc36a1	0,336283	0,016583	0,06	0,049	1
D11Wsu47	0,336294	-0,01922	0,079	0,093	1
Rab3il1	0,336517	0,018237	0,079	0,067	1
Fibp	0,33658	-0,00481	0,306	0,337	1
Sart3	0,336615	-0,01906	0,228	0,247	1
Tug1	0,336652	0,032438	0,529	0,509	1
Zgpat	0,336683	0,023889	0,184	0,166	1
Ndufs5	0,336702	0,031522	0,973	0,969	1
Ncan	0,33675	-0,00608	0,007	0,011	1
Sbds	0,336824	-0,02955	0,921	0,924	1
Cnksr2	0,336884	-0,0136	0,025	0,033	1
Higd2a	0,336895	-0,02244	0,789	0,807	1

Uchl3	0,336981	-0,0135	0,502	0,543	1
Smim7	0,337057	-0,02319	0,767	0,789	1
Prss12	0,337124	0,011366	0,007	0,003	1
Phyh	0,337257	-0,02965	0,464	0,485	1
Bbof1	0,337271	-0,01799	0,085	0,099	1
Acadvl	0,337428	0,053828	0,495	0,491	1
Brd9	0,337542	0,035691	0,71	0,696	1
Fbln1	0,337549	0,006519	0,007	0,003	1
Dclre1c	0,337577	0,022768	0,086	0,073	1
Rnf216	0,337582	-0,00784	0,482	0,514	1
Sccpdh	0,337765	0,029796	0,944	0,93	1
Dok7	0,337783	0,014007	0,135	0,119	1
Ndufaf5	0,337804	-0,02231	0,141	0,158	1
Rhof	0,337943	-0,00447	0,007	0,011	1
Mtag2	0,337943	-0,00447	0,007	0,011	1
Gm48653	0,337943	-0,00447	0,007	0,011	1
Ubn1	0,337944	0,028104	0,618	0,593	1
Gm960	0,337974	0,005547	0,007	0,003	1
Tmc1	0,337974	0,005547	0,007	0,003	1
Evi2	0,338027	-0,00961	0,025	0,033	1
Scaf4	0,338037	0,027055	0,217	0,198	1
D930048G	0,338059	0,017209	0,06	0,049	1
Gdpd3	0,33818	-0,00409	0,037	0,047	1
Fen1	0,338192	0,011897	0,181	0,202	1
Exd1	0,338399	0,006519	0,007	0,003	1
2310030G(	0,338399	0,006519	0,007	0,003	1
Sync	0,338399	0,004573	0,007	0,003	1
Dscc1	0,338399	0,004573	0,007	0,003	1
Snx22	0,338429	-0,01256	0,088	0,102	1
Msto1	0,338438	-0,01053	0,128	0,145	1
Cacna1b	0,338461	-0,00248	0,007	0,011	1
Skida1	0,338502	-0,009	0,025	0,033	1
Klhl15	0,338529	0,021078	0,22	0,2	1
Dhx36	0,338545	-0,00956	0,774	0,793	1
Grik4	0,338606	-0,01966	0,141	0,158	1
3110079O	0,338825	0,003599	0,007	0,003	1
Chrnb2	0,338825	0,003599	0,007	0,003	1
Gm29561	0,338825	0,003599	0,007	0,003	1
1700057H	0,338825	0,003599	0,007	0,003	1
Aldh1a3	0,338825	0,003599	0,007	0,003	1
Scml4	0,338825	0,003599	0,007	0,003	1
Triml2	0,338825	0,003599	0,007	0,003	1
Gm9725	0,338825	0,003599	0,007	0,003	1
Opn4	0,338825	0,003599	0,007	0,003	1
Gm17399	0,338825	0,003599	0,007	0,003	1
Mgat5b	0,338825	0,003599	0,007	0,003	1
Olfr107	0,338825	0,003599	0,007	0,003	1
Gm16068	0,338825	0,003599	0,007	0,003	1
Emx2os	0,338825	0,003599	0,007	0,003	1
Rabepk	0,338902	-0,01331	0,152	0,171	1
Eif4a2	0,338934	0,03738	0,932	0,94	1
Smg9	0,338958	-0,02549	0,284	0,306	1

4930513N1	0,33904	0,0049	0,007	0,003	1
Gm46440	0,339051	-0,00921	0,011	0,016	1
Gm15614	0,339142	-0,0035	0,007	0,011	1
Far2	0,339142	-0,0035	0,007	0,011	1
Celf4	0,339188	-0,01018	0,011	0,016	1
Cdkn2aipnl	0,339203	-0,04004	0,249	0,267	1
Cdnf	0,339474	-0,01204	0,011	0,016	1
Nr1h3	0,339487	-0,00219	0,007	0,011	1
Ppfibp1	0,339591	0,004667	0,068	0,055	1
Gm43666	0,33968	0,003283	0,007	0,003	1
Reps2	0,339685	-0,00647	0,341	0,366	1
Cacfd1	0,339753	-0,02516	0,282	0,301	1
Tmem192	0,339803	-0,01646	0,397	0,426	1
Sfxn1	0,339862	-0,00927	0,025	0,033	1
Gsta3	0,339877	-0,00951	0,046	0,057	1
A230060F1	0,339893	0,002954	0,007	0,003	1
Sphk1	0,339893	0,002954	0,007	0,003	1
Pcdhb8	0,340107	-0,00028	0,007	0,003	1
Dclre1b	0,340198	-0,01999	0,116	0,132	1
Tmc8	0,34032	0,00198	0,007	0,003	1
Hddc3	0,340405	-0,02619	0,188	0,205	1
Zfp768	0,340436	0,022623	0,191	0,174	1
Usf1	0,340688	-0,01218	0,368	0,397	1
Rhot1	0,340715	-0,02855	0,396	0,416	1
Plcg1	0,340755	-0,01443	0,229	0,252	1
Cwc15	0,341005	-0,02201	0,905	0,917	1
Wdr24	0,341114	-0,01207	0,075	0,088	1
Prpf18	0,341166	-0,02396	0,328	0,351	1
Phf12	0,341289	0,020489	0,373	0,353	1
2900076A(	0,341329	-0,02108	0,168	0,185	1
Gm1979	0,341548	-0,00219	0,007	0,011	1
Sdf2	0,341671	0,025673	0,559	0,541	1
4732440D(	0,341737	-0,03022	0,108	0,122	1
Tcp11l1	0,34177	-0,00302	0,275	0,302	1
Haus2	0,34207	-0,04095	0,34	0,354	1
Wapl	0,342256	-0,01134	0,647	0,672	1
Ankrd42	0,342277	-0,00539	0,011	0,016	1
Tspan14	0,342287	0,020852	0,641	0,62	1
Dazap2	0,342415	-0,01705	0,904	0,924	1
Lcat	0,342487	0,022926	0,112	0,098	1
Pck2	0,342565	-0,00374	0,011	0,016	1
Canx	0,342658	0,032079	0,991	0,993	1
Bcl2l11	0,342663	0,036525	0,21	0,195	1
Rab7	0,342734	0,016054	0,99	0,985	1
Mtfmt	0,342813	-0,01152	0,106	0,122	1
Prox2	0,342824	-0,01497	0,063	0,075	1
Rbks	0,342897	-0,01039	0,133	0,151	1
Zfp429	0,342902	-0,00988	0,027	0,036	1
Zadh2	0,342964	-0,02492	0,237	0,257	1
Cox17	0,343174	-0,01561	0,935	0,928	1
Pdzd4	0,343274	-0,0063	0,011	0,016	1
C1d	0,343503	0,028368	0,545	0,517	1

Hacl1	0,343653	-0,02695	0,14	0,156	1
Gm26804	0,34369	-0,00442	0,011	0,016	1
Enc1	0,34369	-0,00442	0,011	0,016	1
Klc2	0,343772	-0,01	0,075	0,088	1
Gm45356	0,343787	-0,01424	0,06	0,072	1
Ppif	0,344049	0,011143	0,106	0,091	1
Homez	0,344291	0,01104	0,071	0,059	1
Lamp2	0,344401	0,024148	0,987	0,995	1
Erich3	0,344497	-0,01573	0,102	0,117	1
Fam207a	0,344698	0,027242	0,266	0,246	1
Rbm12	0,344745	0,030499	0,206	0,19	1
Dlgap4	0,344777	0,020209	0,181	0,163	1
Atp1a3	0,345107	0,002345	0,011	0,016	1
Pogz	0,345189	0,046368	0,274	0,262	1
Dnajc8	0,345235	-0,01523	0,786	0,811	1
Nelfcd	0,345718	-0,01506	0,276	0,298	1
Clpx	0,345732	-0,02616	0,353	0,374	1
Jarid2	0,345947	0,028294	0,479	0,455	1
Ftsj1	0,345983	-0,01749	0,289	0,311	1
Taf5	0,346232	0,009185	0,161	0,143	1
Taf1	0,346463	0,031806	0,697	0,675	1
Vcpkmt	0,3465	-0,01109	0,03	0,039	1
Suox	0,346524	0,022228	0,094	0,081	1
Ifi27l2a	0,346543	0,08777	0,086	0,101	1
Fnip1	0,346643	-0,02901	0,714	0,743	1
Zfp180	0,346776	0,02357	0,297	0,278	1
Vwa8	0,346804	-0,00526	0,239	0,263	1
Atg10	0,346976	-0,00978	0,184	0,205	1
Vcp	0,346992	-0,0079	0,986	0,989	1
Tspyl1	0,347142	-0,01716	0,622	0,654	1
Gm10762	0,347351	-0,0077	0,027	0,036	1
Eif2ak4	0,347379	0,019248	0,131	0,115	1
Tmod3	0,347391	-0,01139	0,278	0,301	1
Ccdc61	0,347461	0,019305	0,139	0,124	1
2010109A1	0,347648	-0,01318	0,018	0,024	1
Tmem141	0,347725	0,036705	0,316	0,294	1
Dtx1	0,347864	0,020429	0,045	0,036	1
Spsb2	0,347895	-0,01311	0,116	0,132	1
Swap70	0,347952	0,001381	0,011	0,016	1
Mfsd2a	0,348032	-0,01967	0,084	0,098	1
Hoxd3os1	0,348099	0,036566	0,389	0,371	1
Lztr1	0,348165	0,028164	0,471	0,449	1
Ylpm1	0,348221	-0,02227	0,579	0,582	1
Snrnp48	0,348274	-0,01427	0,702	0,722	1
Pcdhb15	0,348339	-0,00647	0,018	0,024	1
0610009B2	0,348362	0,044405	0,423	0,411	1
Smarca4	0,348401	0,021649	0,614	0,574	1
Pigw	0,348442	-0,00941	0,018	0,024	1
Nme2	0,348486	0,010247	0,115	0,099	1
Fam189b	0,348488	-0,0031	0,075	0,088	1
Sft2d1	0,348609	-0,01768	0,773	0,803	1
Cox6b2	0,348628	-0,01748	0,09	0,104	1

Tmem175	0,34864	0,030892	0,197	0,18	1
Rab9	0,348661	-0,03505	0,563	0,595	1
Gdap2	0,348896	-0,01558	0,148	0,166	1
Mfsd4b1	0,349075	-0,01109	0,03	0,039	1
Tob1	0,349116	-0,01264	0,775	0,798	1
Nup160	0,349167	-0,01029	0,183	0,203	1
Micu1	0,349183	-0,00889	0,656	0,698	1
Pbrm1	0,34924	-0,00296	0,762	0,767	1
Ankra2	0,349266	0,021347	0,5	0,483	1
Sdhb	0,349644	-0,0132	0,695	0,73	1
Cox11	0,349738	0,011841	0,095	0,081	1
Tor2a	0,349897	0,018443	0,197	0,179	1
Vash1	0,35003	0,009655	0,034	0,026	1
Ppm1m	0,350047	-0,01689	0,134	0,151	1
Pmpcb	0,35006	-0,01837	0,434	0,455	1
1700017B(	0,350124	0,01023	0,047	0,037	1
Faah	0,350179	-0,03775	0,337	0,356	1
Gm26649	0,350181	0,01218	0,032	0,024	1
Ccdc84	0,350337	-0,01128	0,16	0,179	1
Ddx5	0,350363	0,043438	1	1	1
Rtkn2	0,350452	-0,03418	0,351	0,371	1
Cst3	0,350513	-0,01544	0,8	0,831	1
Med31	0,350539	-0,01094	0,51	0,545	1
Btbd7	0,350614	-0,01264	0,417	0,439	1
Kctd21	0,350782	-0,00703	0,12	0,137	1
Fam8a1	0,3508	-0,02266	0,324	0,345	1
Aggf1	0,350852	0,039049	0,447	0,434	1
Snx2	0,351028	0,037281	0,493	0,481	1
Gfap	0,351179	0,038826	0,213	0,197	1
Fastkd2	0,351226	-0,01663	0,121	0,137	1
Amer1	0,351234	0,021205	0,112	0,098	1
Fgfr4	0,351414	0,012392	0,047	0,037	1
Gm13830	0,351515	0,015449	0,084	0,072	1
Rnf185	0,351561	-0,02597	0,15	0,166	1
Nr3c2	0,351625	-0,00495	0,114	0,13	1
Rapgef1	0,351694	-0,02544	0,209	0,229	1
Gnl3	0,351746	0,044201	0,541	0,514	1
Akap8l	0,352008	0,030563	0,549	0,538	1
Kin	0,352189	0,048035	0,289	0,275	1
Dbil5	0,352205	-0,01743	0,084	0,098	1
Vps26a	0,352271	-0,02391	0,471	0,491	1
Slc35a3	0,352294	0,025153	0,255	0,236	1
Ubtd2	0,352428	0,012476	0,086	0,073	1
Armc10	0,352481	-0,01893	0,166	0,184	1
Dhx9	0,35257	0,032939	0,568	0,556	1
B3gntl1	0,352595	0,01104	0,071	0,059	1
Patl1	0,35293	-0,01167	0,182	0,2	1
Colgalt1	0,352943	-0,01487	0,327	0,351	1
Lmbrd2	0,35313	-0,03053	0,592	0,602	1
Cep63	0,353205	-0,01779	0,197	0,216	1
Dnajc7	0,353266	-0,01527	0,767	0,785	1
Plekha2	0,353294	0,053753	0,237	0,223	1

Zfp64	0,353345	0,016557	0,174	0,156	1
Gm7694	0,353727	-0,00105	0,027	0,036	1
Rras2	0,353874	0,018407	0,056	0,046	1
Nvl	0,353904	-0,01361	0,343	0,371	1
Zmynd11	0,354005	-0,00233	0,682	0,724	1
Tmem38a	0,35409	0,01904	0,129	0,114	1
Ccdc22	0,354168	-0,00396	0,265	0,293	1
Endog	0,354195	0,024038	0,173	0,156	1
Haghl	0,354396	-0,02514	0,65	0,675	1
Dcaf6	0,354462	0,026131	0,35	0,33	1
Tmem104	0,354623	-0,02722	0,136	0,151	1
Dffb	0,354813	-0,00839	0,063	0,075	1
Mt3	0,354932	-0,00841	0,183	0,203	1
Exoc3	0,35499	-0,01746	0,728	0,756	1
Sh3glb2	0,354992	-0,02649	0,568	0,58	1
Vps33b	0,355034	0,019116	0,164	0,146	1
Mgat3	0,35512	-0,01699	0,487	0,52	1
Arl16	0,355247	-0,02137	0,181	0,198	1
Spink10	0,355284	0,009297	0,047	0,037	1
Ddx23	0,355642	-0,00354	0,282	0,311	1
BC005624	0,355734	-0,02741	0,723	0,743	1
Gm32005	0,355803	-0,02022	0,098	0,112	1
Lurap1l	0,355814	0,007138	0,032	0,024	1
Vamp3	0,355914	0,036095	0,787	0,793	1
Ppa1	0,356034	-0,01292	0,186	0,207	1
Atp6ap1	0,356066	0,021988	0,971	0,966	1
Wscd2	0,356098	-0,00872	0,042	0,052	1
Raph1	0,356155	0,024673	0,125	0,111	1
Nomo1	0,356178	0,023802	0,271	0,249	1
Lyrm9	0,356473	0,036961	0,198	0,184	1
Alyref2	0,356743	0,017235	0,139	0,124	1
Uap1	0,356756	-0,01547	0,407	0,439	1
Katna1	0,356904	-0,00245	0,197	0,22	1
Actb	0,357037	0,026699	0,999	1	1
Ubr7	0,357087	-0,01795	0,255	0,276	1
Uevld	0,357169	-0,01522	0,09	0,104	1
Iffo2	0,357233	-0,00703	0,069	0,081	1
Patz1	0,35739	-0,02495	0,304	0,325	1
Cdkal1	0,357457	-0,01883	0,32	0,343	1
Mef2d	0,35756	-0,00202	0,266	0,291	1
Ethe1	0,357765	-0,02073	0,274	0,293	1
Mrpl4	0,357949	-0,02822	0,353	0,376	1
Mindy4	0,357969	-0,01173	0,06	0,072	1
Aldh1a1	0,358109	-0,01871	0,134	0,151	1
Pet100	0,35812	0,036936	0,801	0,808	1
Rab39b	0,358169	-0,01993	0,376	0,402	1
Atxn3	0,358242	-0,01847	0,301	0,324	1
Gm13075	0,358415	-0,02343	0,036	0,046	1
1700113A:	0,358599	-0,02525	0,126	0,14	1
Wbp1l	0,358759	-0,01097	0,428	0,46	1
Ctxn1	0,359008	0,005554	0,032	0,024	1
St7	0,359114	0,020809	0,129	0,114	1

Pcbp2	0,359168	0,024553	0,977	0,982	1
Spin4	0,359178	-0,0106	0,039	0,049	1
Cln8	0,359216	0,034495	0,652	0,631	1
Sec24d	0,35922	0,009313	0,127	0,111	1
Ankrd61	0,359491	0,010291	0,028	0,021	1
Synj2bp	0,359519	-0,02038	0,329	0,353	1
Tomm5	0,359627	-0,0264	0,488	0,509	1
Timm22	0,359645	-0,00983	0,428	0,454	1
Eef1b2	0,359705	0,03736	0,929	0,928	1
Mapk8ip3	0,359926	0,032013	0,737	0,724	1
Erp27	0,359996	-0,00446	0,033	0,042	1
Glb1	0,360068	-0,00624	0,039	0,049	1
Defb42	0,360163	-0,0158	0,139	0,156	1
Cacna2d4	0,360359	0,042556	0,064	0,054	1
Rilpl2	0,360369	-0,01865	0,206	0,224	1
Ccdc17	0,360556	-0,00749	0,021	0,028	1
Smim11	0,360621	-0,01035	0,504	0,54	1
Hspbap1	0,360833	-0,01778	0,095	0,109	1
Hexim1	0,36089	0,071537	0,278	0,267	1
Gm17484	0,360925	-0,00899	0,045	0,055	1
Zdhhc3	0,361128	-0,01836	0,334	0,358	1
Fibin	0,361262	-0,02055	0,145	0,163	1
Gtf2a2	0,361417	-0,01924	0,518	0,55	1
Qpctl	0,361435	0,021884	0,183	0,166	1
Mal	0,361609	0,027917	0,998	1	1
Mccc2	0,361614	-0,01458	0,16	0,177	1
H2-D1	0,361627	-0,18903	0,741	0,714	1
Ahcyl2	0,361677	-0,01727	0,482	0,507	1
AY036118	0,361687	0,088121	0,953	0,969	1
Gm26870	0,361877	0,283649	0,009	0,005	1
Mtfp1	0,361902	-0,00558	0,045	0,055	1
Elovl6	0,361943	0,034763	0,708	0,704	1
Etfa	0,361951	-0,01781	0,283	0,302	1
Aplp2	0,361966	-0,01343	0,998	0,993	1
Letm1	0,362206	-0,02585	0,429	0,45	1
Gm26673	0,36222	-0,00885	0,039	0,049	1
Ctu2	0,36237	-0,01566	0,179	0,197	1
Gm11110	0,362453	0,020429	0,05	0,041	1
Pxk	0,362489	-0,00916	0,413	0,447	1
Armcx3	0,362537	0,058303	0,454	0,455	1
Nasp	0,362553	0,063679	0,434	0,424	1
Adipor2	0,362586	-0,0258	0,916	0,917	1
Dner	0,362676	0,012536	0,065	0,054	1
Mfsd4b4	0,3627	0,010312	0,028	0,021	1
Fhl3	0,362833	-0,01204	0,014	0,02	1
Map1a	0,362879	0,015872	0,993	0,992	1
Trip13	0,363034	0,010062	0,009	0,005	1
Gls2	0,363042	-0,01466	0,095	0,109	1
Rrp12	0,363145	-0,00914	0,102	0,117	1
Hspa4	0,36321	-0,01282	0,704	0,715	1
Tpd52	0,363294	-0,01993	0,808	0,826	1
Atp1a2	0,363358	-0,02538	0,113	0,128	1

BC024139	0,363379	-0,01099	0,074	0,086	1
Gm43738	0,363611	0,005871	0,009	0,005	1
0610005C:	0,363611	0,005871	0,009	0,005	1
Gm49289	0,363611	0,005871	0,009	0,005	1
Gm47785	0,363611	0,005871	0,009	0,005	1
Olfr961	0,363611	0,005871	0,009	0,005	1
6030408B:	0,363611	0,005871	0,009	0,005	1
Gm11027	0,363611	0,009747	0,009	0,005	1
Spc25	0,363611	0,006841	0,009	0,005	1
Hoxc6	0,363841	-0,01091	0,024	0,031	1
Ccdc43	0,363876	0,000924	0,2	0,223	1
Gm29666	0,364045	0,00276	0,033	0,042	1
Tufm	0,364064	-0,03335	0,289	0,304	1
Pcx	0,364142	-0,02547	0,436	0,452	1
4732496C(	0,36419	0,0049	0,009	0,005	1
9430037G(	0,36419	0,0049	0,009	0,005	1
Emid1	0,36419	0,0049	0,009	0,005	1
2810408A:	0,36419	0,0049	0,009	0,005	1
Gm26873	0,36419	0,0049	0,009	0,005	1
Mdm1	0,364265	-0,01415	0,062	0,073	1
Acadm	0,364266	-0,0373	0,361	0,38	1
Rybp	0,364557	-0,03233	0,56	0,579	1
Plekho2	0,364574	0,024548	0,178	0,161	1
Idh3g	0,364579	0,048255	0,571	0,584	1
Cpne2	0,364629	-0,00688	0,063	0,075	1
Pex2	0,364633	-0,0318	0,662	0,686	1
Flnc	0,364634	0,026528	0,151	0,135	1
Fam227b	0,36477	0,003927	0,009	0,005	1
Helz2	0,36477	0,003927	0,009	0,005	1
A830082K:	0,36477	0,003927	0,009	0,005	1
Notch4	0,36477	0,003927	0,009	0,005	1
Nme5	0,36477	0,003927	0,009	0,005	1
Numb	0,364825	-0,01541	0,25	0,27	1
Exosc9	0,364945	-0,02161	0,251	0,272	1
Copz1	0,364974	0,032628	0,509	0,501	1
Nsmce2	0,365141	0,038439	0,471	0,459	1
Nfkbie	0,36516	0,005224	0,009	0,005	1
Abce1	0,365275	-0,0096	0,421	0,447	1
Sptlc2	0,365468	-0,02752	0,473	0,489	1
Ypel5	0,365518	-0,036	0,455	0,475	1
Ptpn23	0,365831	0,020417	0,273	0,252	1
Cdyl	0,365969	-0,01872	0,148	0,164	1
Rcor3	0,366057	0,026374	0,507	0,488	1
Gm26830	0,36614	-0,00659	0,014	0,02	1
Hyal3	0,366266	-0,0063	0,014	0,02	1
Sec22b	0,366276	-0,0043	0,383	0,421	1
Atp5s	0,366285	-0,00672	0,182	0,202	1
Hmgn5	0,3663	-0,01906	0,2	0,218	1
Uqcrh	0,366414	-0,01947	0,961	0,963	1
Zufsp	0,366458	-0,0077	0,266	0,289	1
Cbln1	0,366472	-0,00013	0,03	0,023	1
Gm45733	0,366516	0,00231	0,009	0,005	1

Nol9	0,366519	0,014364	0,1	0,086	1
Zfp335os	0,366526	-0,00568	0,014	0,02	1
Gm17477	0,366532	0,011596	0,026	0,02	1
Psma1	0,366595	-0,01975	0,638	0,662	1
Gnb4	0,366765	0,03565	0,53	0,509	1
Rps7	0,366926	0,037231	0,982	0,976	1
Hic2	0,366929	0,022375	0,077	0,065	1
Rnf113a2	0,366992	-0,01909	0,295	0,312	1
Ikzf2	0,367045	0,011315	0,346	0,319	1
Klhl40	0,367094	0,01315	0,065	0,054	1
Zbtb39	0,367107	-0,01096	0,104	0,119	1
Zcchc18	0,36726	0,028855	0,286	0,267	1
Bcr	0,367369	-0,0275	0,202	0,22	1
Cnih4	0,367597	-0,02321	0,545	0,567	1
Creld2	0,367643	-0,01898	0,255	0,276	1
Itga10	0,367684	0,001668	0,009	0,005	1
Pgls	0,367725	-0,02781	0,48	0,507	1
Tmem8	0,367736	0,01339	0,041	0,033	1
Tfap4	0,367803	-0,01232	0,135	0,151	1
Eva1c	0,367834	0,008738	0,026	0,02	1
Syt14	0,367859	-0,00534	0,014	0,02	1
Gart	0,367922	0,028875	0,418	0,4	1
2210016F1	0,368116	0,032836	0,219	0,202	1
Masp1	0,368375	-0,01995	0,26	0,285	1
Slc25a11	0,368402	0,026119	0,519	0,498	1
Nbeal1	0,368546	-0,00575	0,583	0,605	1
Cmah	0,368596	-0,00553	0,048	0,059	1
Pom121	0,36861	-0,0261	0,396	0,423	1
Mc1r	0,368661	-0,00659	0,014	0,02	1
Plxdc2	0,368796	0,014265	0,935	0,924	1
Tmed5	0,368858	-0,01251	0,773	0,8	1
Ccser1	0,369366	0,009051	0,026	0,02	1
Kdm7a	0,369414	-0,02834	0,278	0,296	1
Marf1	0,369417	-0,01945	0,664	0,686	1
Bet1l	0,369703	-0,01225	0,387	0,416	1
Mrpl32	0,369775	0,032818	0,369	0,353	1
Zbtb41	0,369794	-0,01374	0,257	0,281	1
Cln6	0,370071	-0,01511	0,044	0,054	1
Capn2	0,370213	0,030496	0,516	0,504	1
Ppp2r5b	0,37023	0,031601	0,553	0,55	1
Gm43674	0,370272	-0,00742	0,024	0,031	1
Herc2	0,370369	0,031336	0,675	0,673	1
Tmem126a	0,370461	0,021338	0,382	0,359	1
Zkscan5	0,370462	0,018818	0,147	0,132	1
Clspn	0,370609	0,011596	0,025	0,018	1
Fkbp15	0,370702	0,026204	0,352	0,328	1
Fam160b2	0,37072	0,03509	0,153	0,138	1
Nudt15	0,370771	-0,00679	0,045	0,055	1
Nipal4	0,370824	-0,03066	0,587	0,597	1
Dync1li1	0,370855	-0,02543	0,455	0,483	1
Polr3d	0,371007	0,044837	0,369	0,354	1
Mapk14	0,371072	0,041589	0,266	0,252	1

Ggnbp2	0,371086	-0,00735	0,649	0,663	1
Ube2e2	0,371109	-0,01911	0,745	0,767	1
Rragb	0,371173	-0,01685	0,154	0,171	1
Dnttip2	0,371193	-0,0276	0,54	0,553	1
Fam222b	0,371498	-0,01138	0,18	0,198	1
Nras	0,371774	-0,00616	0,678	0,712	1
Ugp2	0,371911	-0,0138	0,615	0,637	1
Mgat5	0,37235	-0,01707	0,185	0,203	1
Twsg1	0,372456	-0,00905	0,244	0,265	1
Nabp2	0,372548	-0,01726	0,704	0,724	1
Pcmtd2	0,372649	-0,02393	0,396	0,423	1
Vdac3	0,372861	-0,01728	0,859	0,863	1
Mnd1	0,37296	-0,00705	0,006	0,01	1
4930599N:	0,37296	-0,00866	0,006	0,01	1
Gbp4	0,37296	-0,01981	0,006	0,01	1
Dock1	0,372973	-0,02857	0,761	0,776	1
Dbp	0,373	0,029444	0,205	0,187	1
Hist1h4d	0,373127	-0,00924	0,134	0,151	1
CAAA0114:	0,373191	-0,01049	0,029	0,037	1
Mkl1	0,373212	0,013545	0,061	0,05	1
Atp11c	0,373233	-0,00491	0,157	0,176	1
Rabggtb	0,37347	0,032545	0,301	0,283	1
1500015L2	0,37372	-0,02088	0,096	0,109	1
Gcc2	0,373767	-0,00168	0,587	0,623	1
Spns1	0,373808	-0,00113	0,482	0,515	1
1190007I0	0,373884	0,024061	0,188	0,172	1
A930017K:	0,373996	0,032244	0,123	0,109	1
March9	0,37403	0,016246	0,068	0,057	1
Ssbp1	0,374101	0,001802	0,411	0,446	1
Gm11992	0,374138	-0,00545	0,006	0,01	1
Ppp1r2	0,37425	0,016289	0,59	0,546	1
Gpr75	0,374616	0,013617	0,05	0,041	1
Cndp2	0,374619	0,033459	0,34	0,322	1
Noa1	0,374715	0,014028	0,207	0,189	1
Thap1	0,374764	-0,05895	0,213	0,229	1
Gm9801	0,374774	-0,01214	0,056	0,067	1
B3galnt1	0,374776	0,033129	0,184	0,169	1
Gm13546	0,374825	0,00301	0,025	0,018	1
Rnd3	0,37489	0,000408	0,181	0,2	1
Sec23a	0,374993	-0,01382	0,215	0,234	1
Inpp5a	0,375025	0,044068	0,537	0,527	1
Tgfb1i1	0,375057	0,010062	0,011	0,007	1
Nckap5l	0,375081	0,008066	0,041	0,033	1
4930570G:	0,375089	0,010022	0,023	0,016	1
Dlc1	0,375093	-0,0033	0,042	0,052	1
Bphl	0,375107	0,018095	0,21	0,192	1
Wsb1	0,375129	0,054136	0,463	0,452	1
Ccdc186	0,375312	0,029874	0,474	0,454	1
Gm12827	0,375318	-0,00384	0,006	0,01	1
Slc12a3	0,375318	-0,00384	0,006	0,01	1
Unc80	0,37532	-0,00447	0,006	0,01	1
Gm17281	0,37532	-0,00447	0,006	0,01	1

Gpx3	0,37532	-0,00447	0,006	0,01	1
Gm30655	0,37532	-0,00447	0,006	0,01	1
Slc39a10	0,375349	0,03135	0,149	0,135	1
5830454E(	0,375405	0,004918	0,026	0,02	1
Gabra2	0,375515	0,002701	0,026	0,02	1
Cog1	0,375648	0,025673	0,203	0,187	1
Zdhhc16	0,375672	-0,02219	0,184	0,202	1
Mapk8	0,375813	-0,03292	0,314	0,335	1
Mob3b	0,375823	-0,03244	0,595	0,566	1
Zfp263	0,375846	-0,00583	0,199	0,22	1
Rnf217	0,375862	0,008098	0,025	0,018	1
Osbpl6	0,375947	0,020858	0,057	0,047	1
Ggps1	0,375965	-0,01289	0,451	0,481	1
Bcdin3d	0,37614	-0,02261	0,17	0,187	1
Irx5	0,376175	0,025145	0,075	0,063	1
0610012G(	0,376178	-0,01544	0,501	0,519	1
4930539J(	0,376345	-0,01197	0,05	0,06	1
Brd8	0,37646	-0,0179	0,619	0,644	1
Hbq1b	0,376468	0,011027	0,011	0,007	1
Cited4	0,376503	-0,00286	0,006	0,01	1
Mucl2	0,376702	-0,00252	0,006	0,01	1
Fam69a	0,376731	-0,02443	0,221	0,239	1
Luc7l	0,376978	-0,00058	0,435	0,472	1
Kdm8	0,376991	0,01215	0,039	0,031	1
Hcn1	0,377	0,01069	0,011	0,007	1
Hdgfl3	0,377097	-0,02221	0,662	0,691	1
Clhc1	0,377174	0,00813	0,011	0,007	1
Cdkn2d	0,377182	-0,00476	0,596	0,62	1
Slc8b1	0,377198	0,02724	0,148	0,133	1
Ddb2	0,377217	-0,01484	0,086	0,099	1
Rpl39	0,377244	0,022717	0,989	0,984	1
Dyx1c1	0,377252	-0,00636	0,01	0,015	1
Tanc1	0,377438	-0,03224	0,654	0,66	1
Zbtb18	0,377473	-0,00787	0,151	0,169	1
Cnot4	0,377527	0,041685	0,517	0,501	1
Rcl1	0,37757	-0,02346	0,19	0,208	1
Cops8	0,377655	-0,01989	0,579	0,6	1
Fam129c	0,37769	-0,00189	0,006	0,01	1
Lrrn2	0,37769	-0,00092	0,006	0,01	1
Snap29	0,377819	-0,00985	0,311	0,333	1
Gm9917	0,377881	0,007162	0,011	0,007	1
Acsl6	0,377881	0,006194	0,011	0,007	1
Hgs.1	0,377881	0,005224	0,011	0,007	1
Vamp4	0,377947	-0,01332	0,411	0,434	1
Mul1	0,378008	-0,01611	0,265	0,286	1
Sulf2	0,378056	-0,03298	0,909	0,917	1
Fam185a	0,378145	-0,01014	0,032	0,041	1
Mum1	0,37822	-0,00015	0,501	0,543	1
Gpatch11	0,37828	-0,01208	0,401	0,431	1
Pld3	0,378426	0,028045	0,363	0,345	1
Yjefn3	0,37843	0,01726	0,108	0,094	1
Traf3ip2	0,378517	-0,00602	0,01	0,015	1

Pcdhb10	0,378518	0,008087	0,025	0,018	1
Tmem147c	0,378524	-0,0077	0,026	0,034	1
Maml3	0,378587	-0,01194	0,121	0,137	1
Rab20	0,378589	0,004254	0,011	0,007	1
Pdf	0,378589	0,004254	0,011	0,007	1
Nespas	0,378672	-0,00539	0,01	0,015	1
2810414N	0,378867	-0,00954	0,032	0,041	1
Gmfg	0,378878	-0,00092	0,006	0,01	1
Mast4	0,3789	0,027577	0,993	0,998	1
Nek8	0,378947	0,008448	0,011	0,007	1
4732471J0	0,379326	-0,0168	0,102	0,115	1
Retreg1	0,379546	0,021645	0,943	0,943	1
Adssl1	0,379598	0,058377	0,912	0,901	1
Ust	0,37974	-0,01128	0,032	0,041	1
Cggbp1	0,379906	0,031141	0,617	0,602	1
Tvp23b	0,379969	-0,01056	0,447	0,478	1
Aifm2	0,379984	0,01731	0,108	0,094	1
R3hcc1	0,380027	-0,01273	0,295	0,32	1
Galnt5	0,380069	0,001999	0,006	0,01	1
Nos3	0,380095	-0,00442	0,01	0,015	1
Pcbd2	0,380189	-0,02276	0,238	0,255	1
Taz	0,380262	-0,04098	0,486	0,501	1
Prpf4	0,380326	-0,0058	0,29	0,315	1
Rtkn	0,380386	0,047019	0,846	0,826	1
Snrpa	0,38053	-0,01819	0,518	0,541	1
Rundc1	0,380564	-0,0066	0,151	0,169	1
Zkscan1	0,380697	-0,02141	0,274	0,293	1
Zscan2	0,380805	0,008697	0,039	0,031	1
Prex2	0,380841	-0,00755	0,017	0,023	1
Cox7b	0,380861	-0,01526	0,796	0,808	1
Ccdc86	0,380976	-0,02131	0,255	0,275	1
Paqr7	0,381202	-0,01549	0,19	0,208	1
Ghr	0,381228	0,011596	0,021	0,015	1
Selenot	0,381228	0,024384	0,933	0,925	1
Duxbl3	0,381468	0,007792	0,021	0,015	1
Ociad1	0,381472	-0,01473	0,882	0,925	1
Larp1b	0,381894	0,001466	0,242	0,268	1
Dst	0,382041	0,034291	0,995	0,99	1
Zfpm2	0,382159	-0,00451	0,026	0,034	1
Slc1a2	0,382284	-0,01738	0,089	0,102	1
D730045B	0,382305	0,009413	0,013	0,008	1
Setd1a	0,382346	-0,01545	0,151	0,167	1
Acin1	0,382355	-0,00035	0,798	0,828	1
Poglut1	0,382391	0,003451	0,411	0,442	1
Tlcd1	0,382588	-0,00659	0,017	0,023	1
2010320M	0,38267	-0,00161	0,074	0,086	1
Cd27	0,382757	0,03971	0,055	0,046	1
Steap3	0,382795	0,04098	0,155	0,14	1
Dtd2	0,382802	-0,03013	0,279	0,298	1
Dctn3	0,383133	-0,00789	0,857	0,886	1
Stim1	0,383138	-0,00855	0,573	0,593	1
Nr2f6	0,383215	-0,01497	0,158	0,174	1

Adpgk	0,383272	-0,0102	0,217	0,237	1
Rnf135	0,38329	-0,01756	0,156	0,172	1
Zbtb4	0,383324	-0,02917	0,337	0,361	1
Adar	0,383329	-0,00799	0,167	0,185	1
Dagla	0,383449	0,008765	0,013	0,008	1
Rcor1	0,38345	0,019201	0,221	0,202	1
Zfp52	0,383517	0,008436	0,019	0,013	1
Nt5dc3	0,383518	0,026526	0,126	0,112	1
Dusp6	0,383935	0,006516	0,013	0,008	1
Spdl1	0,383935	0,007482	0,013	0,008	1
Ezr	0,383935	0,007482	0,013	0,008	1
Ttll4	0,383968	-0,01657	0,129	0,143	1
Gm48619	0,384081	0,007756	0,039	0,031	1
Slc38a7	0,3841	0,015111	0,153	0,137	1
Top2b	0,384144	0,038258	0,664	0,642	1
Arhgdia	0,384195	0,033807	0,983	0,99	1
Mog	0,384237	-0,02403	0,997	1	1
Papd5	0,384248	-0,01217	0,305	0,328	1
Ubap2	0,384387	-0,0118	0,326	0,351	1
Oraov1	0,384482	0,00043	0,212	0,236	1
Trmt112	0,384567	0,024954	0,674	0,676	1
Sh3gl2	0,384584	-0,01514	0,131	0,146	1
Tagln3	0,384674	-0,00389	0,026	0,034	1
Mark1	0,38468	0,013905	0,149	0,133	1
4930403P2	0,384721	0,008765	0,015	0,01	1
Pdia5	0,384721	0,011651	0,015	0,01	1
Adam9	0,38474	0,027091	0,553	0,548	1
Denr	0,384743	-0,02067	0,63	0,65	1
Gm26799	0,384752	0,005548	0,013	0,008	1
Sycp3	0,384752	0,005548	0,013	0,008	1
Kcnmb4	0,384752	0,005548	0,013	0,008	1
Itga2b	0,384752	0,005548	0,013	0,008	1
Gm17586	0,384752	0,005548	0,013	0,008	1
Gm40578	0,384752	0,005548	0,013	0,008	1
Zrsr2	0,38476	0,030331	0,385	0,369	1
Ccar2	0,384878	-0,02104	0,253	0,272	1
1110035H1	0,384949	0,000819	0,024	0,031	1
Wfikkn1	0,385027	0,010042	0,015	0,01	1
AW146154	0,385119	-0,01232	0,106	0,12	1
Mrps9	0,385225	-0,0126	0,329	0,354	1
C1ql2	0,385477	0,013534	0,017	0,011	1
D5Ertd579	0,385482	-0,01499	0,664	0,685	1
Fam160a1	0,38563	0,007801	0,015	0,01	1
Ehbp1l1	0,38571	0,005553	0,023	0,016	1
Gm14302	0,385931	0,008751	0,019	0,013	1
Rnf20	0,385967	-0,00823	0,642	0,662	1
BC043934	0,386066	0,006832	0,019	0,013	1
Fbxo4	0,386183	-0,01103	0,141	0,158	1
Chchd3	0,386242	-0,01394	0,493	0,517	1
Ttll11	0,386464	0,007156	0,017	0,011	1
Usp6nl	0,386725	-0,01533	0,305	0,328	1
A330035P1	0,386885	0,003937	0,013	0,008	1

Tmem5	0,386949	-0,01894	0,694	0,728	1
Frg1	0,387013	0,029749	0,543	0,535	1
Galk1	0,387079	0,040876	0,424	0,418	1
Rpl18a	0,387086	0,025179	0,997	1	1
Ptrh1	0,387139	0,005871	0,019	0,013	1
Vezt	0,387142	0,038101	0,381	0,366	1
Agfg1	0,387281	-0,03015	0,534	0,538	1
Gm46404	0,387284	0,010835	0,048	0,039	1
Kpna4	0,387313	0,023526	0,572	0,558	1
Gm4316	0,387459	0,007156	0,017	0,011	1
Gm26810	0,387459	0,006192	0,017	0,011	1
Hdac9	0,387497	0,015432	0,106	0,093	1
Txnrd3	0,38756	0,018179	0,055	0,046	1
Fxr2	0,387595	0,022387	0,656	0,649	1
D10Jhu81e	0,387667	-0,01821	0,419	0,444	1
Akr1e1	0,387682	0,016747	0,127	0,112	1
Gm14426	0,387705	0,002968	0,013	0,008	1
Ppp4c	0,387851	-0,0075	0,429	0,457	1
Tnrc6a	0,387876	0,004804	0,899	0,896	1
Gtf3c2	0,387918	-0,02648	0,503	0,522	1
Hspa1a	0,388032	-0,02316	0,428	0,402	1
Per2	0,388067	0,007475	0,015	0,01	1
2310035C2	0,388103	0,007382	0,485	0,447	1
Dhx38	0,388154	0,035273	0,196	0,182	1
Gm49201	0,388214	0,004909	0,019	0,013	1
Gm15559	0,388231	0,002686	0,017	0,023	1
Ddx28	0,388246	-0,01008	0,07	0,081	1
Ctdp1	0,388264	-0,01161	0,119	0,133	1
Zbtb43	0,388316	-0,01799	0,205	0,224	1
Rplp0	0,388409	0,032094	0,994	0,993	1
Klhdc3	0,388415	-0,01996	0,339	0,361	1
Araf	0,388533	-0,00977	0,704	0,728	1
Mbip	0,388621	0,020979	0,325	0,302	1
Rere	0,388643	0,014406	0,614	0,574	1
Terf2	0,388715	-0,02103	0,251	0,27	1
Nudt22	0,388759	-0,00828	0,147	0,164	1
Fndc5	0,388828	0,005228	0,015	0,01	1
Ccnf	0,389177	0,003946	0,017	0,011	1
Grtp1	0,389291	0,003946	0,019	0,013	1
Aasdh	0,389322	-0,01654	0,121	0,135	1
Abcg1	0,389485	0,014102	0,861	0,862	1
Tomm34	0,389671	0,046047	0,439	0,436	1
Crebrf	0,389881	0,051719	0,735	0,753	1
Dync2h1	0,389975	0,018494	0,184	0,167	1
Zdhhc4	0,389986	-0,02078	0,235	0,254	1
Nutf2-ps1	0,390038	0,005871	0,017	0,011	1
Hspa13	0,390273	-0,02641	0,393	0,41	1
Taok2	0,390591	0,04142	0,389	0,376	1
Hbp1	0,3906	0,037028	0,609	0,595	1
Eif2b5	0,390642	0,028072	0,36	0,341	1
Spryd4	0,390717	-0,01454	0,075	0,086	1
4931414P1	0,390755	0,010291	0,035	0,028	1

Yipf6	0,390805	-0,00527	0,391	0,42	1
Gorasp2	0,390894	0,001093	0,468	0,506	1
Helz	0,39092	0,014652	0,122	0,107	1
Lss	0,390996	0,088053	0,354	0,348	1
Msh2	0,391107	-0,02593	0,207	0,223	1
Cox6b1	0,391147	-0,01619	0,978	0,977	1
Maged1	0,39123	-0,02838	0,624	0,628	1
Rad23a	0,391234	-0,01416	0,658	0,686	1
Naaa	0,39154	0,010491	0,108	0,094	1
Susd6	0,391823	-0,02003	0,349	0,366	1
Pik3ca	0,391883	0,007191	0,43	0,465	1
Kif13b	0,391892	-0,0399	0,906	0,92	1
Metap1d	0,392097	-0,00738	0,42	0,454	1
A230009B1	0,392146	-0,02037	0,306	0,325	1
Tctex1d2	0,392605	0,015425	0,197	0,18	1
Rnf4	0,39272	0,030179	0,415	0,407	1
Tigd5	0,392822	0,009963	0,035	0,028	1
Rab2a	0,392825	0,028465	0,964	0,956	1
Foxj2	0,392885	-0,009	0,338	0,366	1
Ftl1	0,393175	0,047384	1	0,998	1
Sec24b	0,39346	-0,00631	0,349	0,379	1
1110065P2	0,393951	-0,01464	0,576	0,597	1
Nxpe4	0,394165	-0,00309	0,107	0,122	1
Ehmt2	0,394169	0,052836	0,401	0,397	1
Psmb6	0,394285	-0,01776	0,953	0,958	1
Cyp39a1	0,394333	-0,01663	0,152	0,167	1
Tmem9b	0,394458	-0,0199	0,849	0,85	1
Zdhhc1	0,394464	-0,00239	0,157	0,176	1
Pnpt1	0,394464	-0,03006	0,18	0,193	1
Fxyd3	0,394807	0,00069	0,065	0,054	1
Pars2	0,394933	-0,00246	0,047	0,057	1
Engase	0,395006	-0,00409	0,038	0,047	1
Skap2	0,395014	-0,01876	0,631	0,659	1
Col23a1	0,395219	-0,0239	0,013	0,018	1
Sfswap	0,395229	0,014673	0,57	0,533	1
Ncln	0,395261	0,0325	0,524	0,512	1
Comt	0,395475	0,042234	0,751	0,769	1
Fbxo27	0,396125	0,000913	0,041	0,05	1
Usp36	0,396353	0,030005	0,244	0,228	1
Swt1	0,396368	-0,03965	0,539	0,564	1
Smarca1	0,396379	0,022789	0,031	0,024	1
Chmp4b	0,396753	0,033796	0,905	0,912	1
Thyn1	0,396824	-0,00359	0,316	0,34	1
Tsta3	0,396857	-0,01782	0,238	0,257	1
Pcdhb11	0,396888	0,015305	0,033	0,026	1
Gnaq	0,396894	-0,01676	0,787	0,793	1
Letmd1	0,397033	-0,01602	0,331	0,348	1
Tapbp	0,397072	-0,1346	0,332	0,343	1
Pex11g	0,397113	0,011386	0,066	0,055	1
Mvb12a	0,397163	-0,01094	0,467	0,506	1
Kdelr1	0,397235	0,027745	0,632	0,633	1
Stam	0,397277	-0,02794	0,501	0,517	1

Sesn3	0,397324	-0,00331	0,335	0,359	1
Gm20045	0,397345	-0,01271	0,088	0,101	1
Zdhhc21	0,397558	-0,03202	0,305	0,322	1
Ptpn2	0,397897	-0,01377	0,143	0,159	1
Fopnl	0,397927	-0,02325	0,504	0,537	1
Chd3	0,398018	0,007444	0,035	0,028	1
Wdr5b	0,398058	0,013731	0,073	0,062	1
Plxna4	0,398081	0,017891	0,044	0,036	1
Crcp	0,398101	-0,01654	0,195	0,213	1
Gnas	0,398165	-0,0165	0,995	0,992	1
Cd151	0,398207	0,008194	0,19	0,172	1
Ppp3r1	0,398459	-0,00144	0,395	0,428	1
Smpd1	0,398904	-0,02069	0,64	0,685	1
Gm14326	0,398944	-0,0124	0,105	0,119	1
Zdhhc6	0,399016	-0,02833	0,224	0,239	1
Zfp59	0,399087	0,010905	0,035	0,028	1
Arhgef11	0,399106	-0,01321	0,182	0,198	1
Asnsd1	0,399204	-0,00925	0,205	0,224	1
Pde6d	0,399284	-0,01337	0,732	0,764	1
Tmem243	0,399787	-0,01461	0,447	0,473	1
Diras1	0,399882	-0,03763	0,225	0,241	1
Lrriq3	0,399945	-0,00145	0,02	0,026	1
Hist2h3c1	0,400136	-0,00813	0,08	0,093	1
Gm44175	0,400282	-0,01142	0,013	0,018	1
Crlf2	0,400419	0,011236	0,033	0,026	1
Sin3a	0,400471	-0,01619	0,244	0,262	1
Capns1	0,400601	0,0198	0,979	0,977	1
Dnaic2	0,400951	0,011736	0,055	0,046	1
Stk11	0,400998	0,036744	0,513	0,506	1
Gm4737	0,401012	0,011437	0,055	0,046	1
Gm14443	0,401082	-0,00681	0,023	0,029	1
Sdf4	0,401204	0,027917	0,882	0,883	1
Sh3bgrl2	0,401231	0,01476	0,093	0,081	1
Ttyh1	0,401366	0,016629	0,319	0,301	1
Zmat3	0,40142	0,027594	0,222	0,207	1
Mrpl13	0,401478	-0,01134	0,433	0,468	1
Dhrs1	0,401571	-0,02826	0,586	0,59	1
A330015K(	0,401718	-0,0279	0,418	0,441	1
Mysm1	0,401757	-0,00752	0,59	0,629	1
Bri3	0,401774	-0,01648	0,89	0,888	1
Galns	0,402235	0,023506	0,067	0,057	1
Tbce	0,402338	-0,00206	0,316	0,346	1
Hax1	0,402469	-0,00818	0,434	0,462	1
Rbpms	0,40248	-0,00859	0,026	0,033	1
Irak2	0,402545	-0,01256	0,069	0,08	1
Frs2	0,402701	0,019887	0,3	0,283	1
Atp23	0,402768	-0,00742	0,023	0,029	1
Capn5	0,402899	-0,01684	0,279	0,302	1
Thap11	0,403229	-0,00462	0,272	0,294	1
Nom1	0,403273	-0,02515	0,251	0,27	1
Rab40c	0,403664	0,019911	0,497	0,478	1
Gm43713	0,403764	0,015998	0,177	0,161	1

Gpr158	0,403774	-0,01188	0,037	0,046	1
Atp13a3	0,4038	0,011556	0,318	0,296	1
Trmt13	0,40388	-0,02623	0,384	0,411	1
Stk36	0,403928	-0,00471	0,013	0,018	1
Gpam	0,403954	0,010555	0,044	0,036	1
Col9a2	0,403989	-0,0022	0,079	0,091	1
1700034P1	0,40407	-0,00505	0,013	0,018	1
Dazap1	0,404092	0,007604	0,565	0,525	1
Odf2l	0,404122	-0,01459	0,203	0,221	1
Echdc1	0,404608	0,021089	0,372	0,353	1
Nsrp1	0,404694	0,046827	0,373	0,363	1
Cep104	0,404697	-0,00824	0,359	0,382	1
Aspscr1	0,404728	-0,00791	0,408	0,439	1
Klhl21	0,404865	-0,01176	0,107	0,12	1
Polr3g	0,404988	-0,00908	0,11	0,124	1
Txnrd1	0,405253	-0,03122	0,376	0,398	1
Cited2	0,405334	0,013731	0,064	0,054	1
Tro	0,405578	-0,00428	0,023	0,029	1
Fastkd5	0,405652	-0,00961	0,026	0,033	1
Nadsyn1	0,40567	-0,01214	0,04	0,049	1
Med23	0,405676	-0,01714	0,179	0,195	1
2310047D0	0,405684	-0,00408	0,013	0,018	1
Bola2	0,405842	-0,00609	0,71	0,722	1
Jmjd6	0,406236	0,032518	0,436	0,423	1
Fchsd1	0,406349	-0,00893	0,028	0,036	1
Cpeb4	0,406532	-0,01678	0,712	0,748	1
Car11	0,406586	-0,01478	0,194	0,211	1
Xpo6	0,406609	-0,02821	0,346	0,363	1
Gm49336	0,406676	-0,00363	0,258	0,281	1
Zfp934	0,406783	-0,02256	0,156	0,171	1
Wdr62	0,40701	-0,00374	0,013	0,018	1
Rnaset2b	0,407178	0,019135	0,144	0,13	1
Cyth1	0,407258	-0,01571	0,863	0,865	1
Cltc	0,407381	0,022337	0,94	0,943	1
Zbtb12	0,407396	0,023258	0,153	0,14	1
Dda1	0,40751	0,020055	0,589	0,571	1
Snrpf	0,4076	0,028816	0,773	0,79	1
Rnf34	0,407638	0,007756	0,394	0,429	1
Gsto2	0,407802	0,025417	0,125	0,112	1
Dpp8	0,408034	-0,00318	0,44	0,478	1
Lsm2	0,408054	-0,00331	0,269	0,293	1
Nim1k	0,408166	0,025307	0,042	0,034	1
Rab11fip4	0,408298	0,030429	0,498	0,486	1
Hnrnpr	0,408703	0,031759	0,816	0,782	1
Scaf1	0,408722	0,032165	0,554	0,537	1
Tex264	0,409152	0,024804	0,575	0,554	1
Klhl5	0,409311	0,013839	0,189	0,172	1
Irak3	0,409634	0,021789	0,127	0,114	1
Rep15	0,409669	0,009297	0,044	0,036	1
Kifap3	0,409686	0,03729	0,713	0,709	1
Clpp	0,40996	-0,01133	0,581	0,598	1
Nme3	0,409965	-0,00697	0,08	0,093	1

Cenpq	0,410014	-0,02532	0,145	0,159	1
Nsun5	0,410465	-0,00791	0,13	0,145	1
Edf1	0,410556	0,025883	0,874	0,854	1
Nmnat3	0,410628	-0,01627	0,074	0,085	1
Sra1	0,410675	-0,0144	0,625	0,628	1
Xbp1	0,41069	-0,02449	0,564	0,582	1
Rbm6	0,410712	0,0308	0,525	0,504	1
Bag6	0,410728	0,021138	0,658	0,652	1
Gm45184	0,410887	-0,00764	0,031	0,039	1
Atp6v0e2	0,410901	-0,00795	0,869	0,902	1
Rpf1	0,410977	-0,02013	0,209	0,224	1
Arf4	0,411077	-0,00462	0,733	0,764	1
Tssc4	0,411125	-0,00513	0,334	0,359	1
Mettl18	0,411366	-0,00954	0,031	0,039	1
Hoxd8	0,411572	0,026705	0,693	0,681	1
Epg5	0,411584	0,002666	0,177	0,197	1
Emg1	0,41171	-0,01667	0,43	0,455	1
Gcc1	0,411921	0,010081	0,13	0,146	1
Ccdc24	0,411953	0,014463	0,06	0,05	1
Tmem62	0,4121	-0,01365	0,379	0,407	1
Reep5	0,41221	-0,00828	0,967	0,956	1
Sirt5	0,412295	0,02198	0,135	0,122	1
Mtx3	0,412398	-0,00735	0,164	0,18	1
Mocs3	0,412498	-0,01077	0,134	0,15	1
Dgat1	0,412525	0,024652	0,206	0,192	1
Pik3ip1	0,412551	-0,01333	0,677	0,704	1
Dennd2a	0,413085	-0,01098	0,189	0,207	1
G6pdx	0,413095	-0,04733	0,376	0,389	1
1500011K1	0,413149	-0,02332	0,498	0,509	1
Nelfb	0,413234	-0,00415	0,392	0,42	1
Alkbh7	0,413268	-0,02103	0,267	0,285	1
Rpl37a	0,413322	0,028371	0,996	0,998	1
Pdia6	0,413478	-0,01859	0,75	0,763	1
Slc29a2	0,413498	-0,00641	0,028	0,036	1
Gm12758	0,413609	0,00743	0,044	0,036	1
Efcab2	0,413642	-0,01497	0,139	0,154	1
Dhx15	0,413665	0,034042	0,528	0,517	1
Camk2b	0,413793	-0,0101	0,016	0,021	1
Ap2s1	0,413837	0,024656	0,86	0,833	1
Syt6	0,414071	0,010083	0,004	0,002	1
Rrn3	0,414145	0,005871	0,343	0,369	1
Sipa1l1	0,414186	0,007075	0,073	0,062	1
Gtf2f1	0,414286	-0,01973	0,479	0,498	1
Sbno1	0,414378	0,048142	0,778	0,793	1
Reln	0,414394	0,010083	0,004	0,002	1
BC052040	0,414422	-0,01703	0,111	0,124	1
Nr4a1	0,414521	0,064926	0,129	0,117	1
Bnip1	0,414607	-0,00562	0,134	0,15	1
Vash2	0,414718	0,004246	0,004	0,002	1
Stpg4	0,414718	0,004246	0,004	0,002	1
D030040B1	0,414718	0,005221	0,004	0,002	1
Tmem82	0,414718	0,005221	0,004	0,002	1

Slc25a2	0,414718	0,005221	0,004	0,002	1
Xrcc2	0,414891	-0,00932	0,11	0,124	1
Pde7a	0,41499	0,028733	0,234	0,218	1
Cited1	0,415041	0,005221	0,004	0,002	1
Tgfbr2	0,415041	0,005221	0,004	0,002	1
Gm15938	0,415041	0,004246	0,004	0,002	1
Sardh	0,415041	0,00327	0,004	0,002	1
4930502E1	0,415041	0,00327	0,004	0,002	1
Smim10l2a	0,415041	0,00327	0,004	0,002	1
Fgf13	0,415041	0,00327	0,004	0,002	1
Gm2464.1	0,415041	0,00327	0,004	0,002	1
Pcdh18	0,415041	0,00327	0,004	0,002	1
Gm27022	0,415041	0,00327	0,004	0,002	1
Sh2d5	0,415041	0,00327	0,004	0,002	1
Tspan33	0,415041	0,00327	0,004	0,002	1
1700040L0	0,415041	0,00327	0,004	0,002	1
Vmn1r46	0,415041	0,00327	0,004	0,002	1
Prrt3	0,415041	0,00327	0,004	0,002	1
Slc8a2	0,415041	0,00327	0,004	0,002	1
Gm39228	0,415041	0,00327	0,004	0,002	1
Gm26536	0,415041	0,00327	0,004	0,002	1
Lrrc2	0,415041	0,00327	0,004	0,002	1
Tcte3	0,415041	0,00327	0,004	0,002	1
Ikbkb	0,415106	-0,01504	0,371	0,389	1
Fzd10	0,415215	-0,00963	0,005	0,008	1
5730559C1	0,415215	-0,01283	0,005	0,008	1
Nrtn	0,415271	-0,02262	0,144	0,159	1
Rad51b	0,415361	-0,00696	0,037	0,046	1
Gm37988	0,415365	0,002293	0,004	0,002	1
Speg	0,415365	0,002293	0,004	0,002	1
Stkld1	0,415365	0,002293	0,004	0,002	1
Klhl41	0,415365	0,002293	0,004	0,002	1
Olfr1310	0,415365	0,002293	0,004	0,002	1
Gm13963	0,415365	0,002293	0,004	0,002	1
Kif16bos	0,415365	0,002293	0,004	0,002	1
Mageb16	0,415365	0,002293	0,004	0,002	1
Gm37933	0,415365	0,002293	0,004	0,002	1
Dcst2	0,415365	0,002293	0,004	0,002	1
Gm42681	0,415365	0,002293	0,004	0,002	1
I830077J0	0,415365	0,002293	0,004	0,002	1
Dennd2d	0,415365	0,002293	0,004	0,002	1
Gm43240	0,415365	0,002293	0,004	0,002	1
Triqk	0,415365	0,002293	0,004	0,002	1
Aqp7	0,415365	0,002293	0,004	0,002	1
Zkscan16	0,415365	0,002293	0,004	0,002	1
Gm12932	0,415365	0,002293	0,004	0,002	1
Angptl7	0,415365	0,002293	0,004	0,002	1
4632411P0	0,415365	0,002293	0,004	0,002	1
Gm35911	0,415365	0,002293	0,004	0,002	1
Gm20594	0,415365	0,002293	0,004	0,002	1
Vmn1r58	0,415365	0,002293	0,004	0,002	1
Igflr1	0,415365	0,002293	0,004	0,002	1

Upk1a	0,415365	0,002293	0,004	0,002	1
Rsf1os2	0,415365	0,002293	0,004	0,002	1
Olfr624	0,415365	0,002293	0,004	0,002	1
Gm10778	0,415365	0,002293	0,004	0,002	1
Gm31182	0,415365	0,002293	0,004	0,002	1
Ptger1	0,415365	0,002293	0,004	0,002	1
Zfp791	0,415365	0,002293	0,004	0,002	1
Gm10248	0,415365	0,002293	0,004	0,002	1
Rnf17	0,415365	0,002293	0,004	0,002	1
Panx1	0,415365	0,002293	0,004	0,002	1
Gm38431	0,415365	0,002293	0,004	0,002	1
Gm36799	0,415365	0,002293	0,004	0,002	1
Pstpip1	0,415365	0,002293	0,004	0,002	1
Gm47409	0,415365	0,002293	0,004	0,002	1
1700065D1	0,415365	0,002293	0,004	0,002	1
9630041A(	0,415365	0,002293	0,004	0,002	1
Eme1	0,415365	0,002293	0,004	0,002	1
Omd	0,415365	0,002293	0,004	0,002	1
2410018L1	0,415365	0,002293	0,004	0,002	1
Gm15943	0,415365	0,002293	0,004	0,002	1
AC126280.	0,415365	0,002293	0,004	0,002	1
Def6	0,415365	0,002293	0,004	0,002	1
St8sia3os	0,415365	0,002293	0,004	0,002	1
Gm10053	0,415365	0,002293	0,004	0,002	1
Plce1	0,415365	0,002293	0,004	0,002	1
Mycbp	0,415392	-0,00572	0,17	0,187	1
Gsdme	0,415404	-0,00669	0,031	0,039	1
Trp53bp1	0,41543	-0,01227	0,222	0,242	1
Ddx50	0,415442	0,021024	0,574	0,561	1
Gm21781	0,415488	-0,0069	0,037	0,046	1
Aph1b	0,415724	-0,02022	0,218	0,236	1
Nr1h2	0,415954	0,022626	0,574	0,567	1
Iqcb1	0,415976	0,013464	0,212	0,195	1
Mllt10	0,416266	-0,02001	0,374	0,395	1
0610039K1	0,416338	0,001648	0,004	0,002	1
Stk32b	0,416338	0,001648	0,004	0,002	1
Eno1	0,416357	0,02776	0,598	0,584	1
Lmo1	0,416362	-0,00642	0,005	0,008	1
Cd59b	0,416546	-0,00665	0,069	0,08	1
Mrpl27	0,416636	0,004759	0,491	0,532	1
Actrt3	0,416662	0,000671	0,004	0,002	1
4930471M	0,416662	0,000671	0,004	0,002	1
Pdlim4	0,416662	0,000671	0,004	0,002	1
Mcidas	0,416662	0,000671	0,004	0,002	1
Pcdha5	0,416662	0,000671	0,004	0,002	1
Pyroxd2	0,416662	0,000671	0,004	0,002	1
Fcrls	0,416662	2,86E-05	0,004	0,002	1
Abhd2	0,416776	-0,00681	0,263	0,285	1
Helb	0,416958	0,01966	0,106	0,094	1
Racgap1	0,416997	-0,00413	0,031	0,039	1
Uba7	0,417282	-0,01026	0,005	0,008	1
Ap3s1	0,417305	-0,00757	0,099	0,112	1

Plk2	0,417355	-0,00871	0,06	0,07	1
Fcrl6	0,417511	-0,00481	0,005	0,008	1
Gm30173	0,417511	-0,00481	0,005	0,008	1
Nudt17	0,417511	-0,00481	0,005	0,008	1
Ttc9b	0,417511	-0,00481	0,005	0,008	1
Polq	0,417511	-0,00481	0,005	0,008	1
Xlr4c	0,417511	-0,00642	0,005	0,008	1
Paip2b	0,417592	0,016433	0,421	0,462	1
Atp1b1	0,417725	-0,00755	0,016	0,021	1
Shoc2	0,417738	0,00182	0,262	0,288	1
Mpi	0,417818	-0,00765	0,113	0,127	1
Sap18b	0,417947	0,007459	0,029	0,023	1
Rsrp1	0,41797	-0,0088	0,998	0,998	1
Mcm10	0,417973	-0,00058	0,005	0,008	1
Fgf12	0,417978	-0,00898	0,066	0,076	1
Usmg5	0,41826	-0,01042	0,858	0,862	1
Gzmm	0,418568	0,015994	0,027	0,021	1
Lsm6	0,418598	-0,02006	0,617	0,636	1
Gm37756	0,418662	-0,0032	0,005	0,008	1
Slc12a5	0,418662	-0,0032	0,005	0,008	1
Gm43825	0,418662	-0,0032	0,005	0,008	1
5730596B1	0,418662	-0,0032	0,005	0,008	1
Ttc23	0,418662	-0,0032	0,005	0,008	1
Pgghg	0,418662	-0,0032	0,005	0,008	1
1810041H1	0,418662	-0,0032	0,005	0,008	1
Tmie	0,418662	-0,0032	0,005	0,008	1
Gm17334	0,418662	-0,0032	0,005	0,008	1
Gm33280	0,418663	-0,00384	0,005	0,008	1
Raver2	0,418663	-0,00384	0,005	0,008	1
Halr1	0,418664	-0,00447	0,005	0,008	1
Zfp764	0,418691	0,018407	0,058	0,049	1
Tspan6	0,418699	-0,0139	0,057	0,067	1
Nf2	0,418826	-0,01876	0,321	0,341	1
Tctn1	0,418848	0,01762	0,08	0,07	1
Bicral	0,418904	0,048487	0,335	0,324	1
Ppfia1	0,418951	0,023545	0,288	0,27	1
Fam83b	0,418965	0,006504	0,029	0,023	1
Flad1	0,41904	-0,00755	0,202	0,221	1
Slc25a42	0,419123	-0,01279	0,125	0,138	1
Wnt3	0,419432	-0,00224	0,363	0,392	1
Foxo6	0,419538	-0,01436	0,071	0,081	1
Rnf169	0,419554	-0,01158	0,16	0,176	1
Slc25a26	0,419758	-0,01705	0,111	0,124	1
Gm17552	0,419765	-0,01072	0,016	0,021	1
Gm43062	0,419769	-0,00562	0,016	0,021	1
4933424G0	0,419816	-0,00223	0,005	0,008	1
Gm28609	0,419817	-0,00286	0,005	0,008	1
Mcub	0,419817	-0,00286	0,005	0,008	1
Gm14827	0,420048	5,69E-05	0,005	0,008	1
Fut9	0,420238	-0,00192	0,046	0,055	1
Xylt1	0,420352	-0,00102	0,034	0,042	1
Fbxo3	0,420353	-0,0205	0,312	0,333	1

1700084E1	0,420398	-0,00476	0,009	0,013	1
Sh3tc2	0,420449	0,023706	0,25	0,234	1
Slc9a6	0,420482	-0,01195	0,381	0,405	1
Gm5093	0,420573	-0,00413	0,009	0,013	1
Gm26533	0,420573	-0,00413	0,009	0,013	1
Ddx25	0,420575	-0,00282	0,009	0,013	1
Rft1	0,420608	0,015275	0,214	0,197	1
A230050P2	0,420971	-0,00028	0,005	0,008	1
Tkfc	0,421015	-0,01381	0,119	0,132	1
Taf3	0,421095	-0,01163	0,377	0,4	1
Tmem176b	0,421525	0,033712	0,131	0,117	1
Gm28424	0,421545	-0,00466	0,016	0,021	1
Exosc7	0,421559	-0,00792	0,319	0,343	1
Agbl3	0,421604	-0,02293	0,184	0,198	1
Ascc3	0,421607	0,000107	0,424	0,455	1
Gm47015	0,421667	-0,00118	0,005	0,008	1
Phyhd1	0,421678	-0,005	0,016	0,021	1
Rhbdf1	0,421678	-0,005	0,016	0,021	1
Ip6k2	0,421682	0,036663	0,356	0,341	1
Cnppd1	0,421698	0,004454	0,407	0,446	1
Camk2n2	0,421718	-0,00527	0,066	0,076	1
Ikbip	0,421874	-0,00605	0,224	0,242	1
Ndufaf8	0,421947	0,009134	0,406	0,449	1
Rad1	0,421956	0,019518	0,251	0,233	1
Reck	0,421973	0,005871	0,029	0,023	1
Tspan13	0,421996	-0,00219	0,009	0,013	1
Krtcap3	0,421996	-0,00316	0,009	0,013	1
Gm48740	0,421996	-0,00316	0,009	0,013	1
E2f6	0,422059	0,025997	0,177	0,163	1
1190005I0	0,42208	-0,01764	0,586	0,613	1
Rasgrf1	0,422129	0,000696	0,005	0,008	1
Ltbp4	0,422129	0,023917	0,038	0,031	1
Coq4	0,422296	-0,00478	0,181	0,198	1
Cpne3	0,422352	-0,00914	0,327	0,354	1
Zhx1	0,422519	0,036697	0,43	0,415	1
Mrpl19	0,422731	-0,00429	0,284	0,307	1
Eif3l	0,422755	0,025116	0,921	0,894	1
2310068J1	0,422835	0,018122	0,063	0,054	1
Ssr4	0,422926	-0,01288	0,766	0,78	1
Znrd1as	0,423007	-0,03139	0,096	0,107	1
Nup85	0,42314	-0,00969	0,212	0,229	1
Ndrg4	0,423287	0,02628	0,106	0,094	1
Scn8a	0,423294	0,018293	0,067	0,057	1
Nrbp2	0,423318	-0,00589	0,944	0,951	1
Hist2h2bb	0,423425	-0,00185	0,009	0,013	1
E130307A1	0,423433	-0,00202	0,244	0,263	1
Synm	0,423434	0,034396	0,795	0,78	1
St6galnac5	0,423775	0,006814	0,029	0,023	1
Sox9	0,423783	0,000416	0,009	0,013	1
Tmem33	0,423835	-0,01231	0,893	0,891	1
Gm15859	0,424453	0,01558	0,072	0,062	1
Tsacc	0,424504	-0,00755	0,082	0,094	1

Rpl7	0,424557	0,01333	0,989	0,985	1
Chtop	0,424696	-0,01849	0,736	0,771	1
Capn7	0,424743	-0,02075	0,251	0,27	1
Rad9b	0,424765	-0,00271	0,111	0,125	1
Gm17435	0,424767	-0,01381	0,054	0,063	1
Phf2	0,424781	0,027531	0,341	0,325	1
C1qb	0,424789	0,008411	0,027	0,021	1
BC055324	0,424852	-0,00122	0,009	0,013	1
6530437J2	0,424852	-0,00122	0,009	0,013	1
Gm45352	0,424852	-0,00122	0,009	0,013	1
1700041G	0,424853	-0,00088	0,009	0,013	1
Ppie	0,42486	-0,00565	0,24	0,259	1
Fcho2	0,424904	0,035301	0,788	0,779	1
Ufsp1	0,425078	-0,00833	0,087	0,099	1
Ttc13	0,425235	0,03208	0,168	0,156	1
Atp13a1	0,425559	0,013956	0,27	0,252	1
Gm10220	0,42566	0,00014	0,016	0,021	1
B4gat1	0,426045	0,028827	0,692	0,685	1
B9d1	0,42614	-0,02015	0,183	0,197	1
Scyl1	0,4262	-0,01305	0,316	0,337	1
Pias1	0,426228	-0,00442	0,31	0,338	1
Prdx1	0,426407	-0,01096	0,999	1	1
Utp6	0,426453	-0,00566	0,279	0,302	1
Kdm4a	0,426641	0,042775	0,39	0,385	1
Sbk1	0,426689	-0,00712	0,164	0,18	1
Mapk9	0,426904	-0,00893	0,273	0,294	1
Gm17146	0,426916	0,012547	0,026	0,02	1
Ccdc107	0,42732	-0,00379	0,125	0,14	1
Med13	0,427363	0,043875	0,612	0,589	1
Gm17259	0,427445	-0,01091	0,068	0,078	1
Ptprj	0,427654	0,010858	0,04	0,033	1
Wrap73	0,428036	0,003859	0,239	0,263	1
Tmed8	0,428147	-0,01487	0,133	0,148	1
Podxl2	0,428237	-0,01022	0,079	0,091	1
Exd2	0,428382	-0,00577	0,35	0,377	1
Vps54	0,428618	-0,01464	0,215	0,233	1
Pspc1	0,428688	0,024413	0,343	0,327	1
Senp2	0,428695	0,020792	0,48	0,463	1
Gm26798	0,428781	-0,00246	0,043	0,052	1
Pi4ka	0,429217	-0,00279	0,43	0,46	1
Mcat	0,42925	-0,02008	0,149	0,163	1
Zbtb33	0,429615	-0,02412	0,213	0,226	1
Efcab5	0,429752	0,001187	0,034	0,042	1
Park7	0,429755	0,023882	0,959	0,925	1
Pik3c3	0,429857	-0,00577	0,376	0,4	1
Htt	0,429881	-0,00336	0,364	0,395	1
Fbxo5	0,429936	0,021277	0,077	0,067	1
Dmwd	0,429967	-0,01297	0,593	0,616	1
Sirt3	0,42997	-0,0191	0,311	0,335	1
Emc6	0,430181	-0,02438	0,55	0,553	1
Lemd3	0,430266	0,023047	0,241	0,226	1
Snhg11	0,430288	0,071153	0,121	0,138	1

Adprm	0,430388	0,012782	0,173	0,158	1
Lin52	0,430423	-0,01693	0,187	0,203	1
U2af1l4	0,430504	-0,00528	0,019	0,024	1
Dcaf13	0,430701	0,008528	0,268	0,294	1
Atpif1	0,430762	0,012889	0,721	0,698	1
Tppp	0,431004	-0,00994	0,987	0,972	1
Slx4	0,43124	-0,01096	0,106	0,119	1
Slc9a1	0,431248	0,019709	0,184	0,169	1
Cd300e	0,431263	0,008738	0,026	0,02	1
4833418N(	0,431393	0,010002	0,026	0,02	1
Hgsnat	0,431414	-0,01564	0,28	0,296	1
Efcab7	0,431425	0,009329	0,038	0,031	1
Timm44	0,431445	-0,01729	0,437	0,463	1
Tlk1	0,431477	-0,01808	0,423	0,444	1
Setmar	0,431614	-0,00063	0,043	0,052	1
Unc45a	0,43164	-0,00583	0,212	0,231	1
Adh5	0,43193	-0,02934	0,842	0,862	1
Gm12184	0,432039	0,009452	0,089	0,078	1
Mthfr	0,432044	-0,00687	0,019	0,024	1
Arrdc2	0,432088	0,027803	0,397	0,384	1
Gm38399	0,432301	-0,00528	0,019	0,024	1
Kars	0,432305	-0,01128	0,383	0,408	1
March6	0,432455	-0,01056	0,399	0,423	1
Prcc	0,432474	0,017303	0,206	0,19	1
Nisch	0,432631	0,031471	0,927	0,93	1
Rars2	0,43278	-0,0084	0,139	0,154	1
Ppp2r3d	0,432793	0,020631	0,273	0,257	1
Rspry1	0,433075	-0,01248	0,397	0,42	1
Ran	0,433453	0,020648	0,878	0,855	1
Lrp10	0,433878	0,028981	0,388	0,376	1
Vipas39	0,433897	0,021193	0,301	0,285	1
Yrdc	0,4339	0,003896	0,264	0,289	1
Shmt2	0,433993	-0,00759	0,175	0,192	1
Slc25a30	0,434046	0,025596	0,197	0,184	1
Ndufc1	0,434055	-0,00334	0,859	0,88	1
Ctps2	0,43408	-0,02331	0,181	0,195	1
Gm37494	0,434173	-0,02992	0,509	0,514	1
Fdxacb1	0,434362	0,003922	0,144	0,13	1
2700012I2	0,434428	0,007144	0,026	0,02	1
Cox6c	0,434474	-0,02356	0,991	0,995	1
Pmpca	0,434568	0,00092	0,407	0,444	1
Siva1	0,434581	0,026331	0,528	0,506	1
Dcaf7	0,434958	-0,00562	0,279	0,299	1
Eif2ak2	0,435106	-0,0157	0,039	0,047	1
Pex10	0,435184	-0,00305	0,113	0,127	1
Tpcn1	0,435211	0,0308	0,272	0,259	1
Arfgap2	0,435294	-0,0188	0,695	0,702	1
Stx1b	0,435455	0,042259	0,139	0,128	1
Usp27x	0,435604	0,009313	0,038	0,031	1
Qrich1	0,43577	0,005663	0,389	0,421	1
Plpp6	0,435844	-0,02031	0,125	0,137	1
Zfp362	0,435881	-0,01682	0,142	0,156	1

Dirc2	0,435886	-0,02671	0,526	0,546	1
Ppox	0,435957	-0,00169	0,169	0,187	1
Naa40	0,436009	-0,00761	0,136	0,151	1
Phc3	0,436081	-0,01401	0,614	0,659	1
Fam192a	0,43619	0,028223	0,463	0,45	1
Maoa	0,436243	0,031331	0,469	0,462	1
Scamp2	0,436254	0,031557	0,647	0,637	1
Smg5	0,436603	-0,02243	0,341	0,358	1
Gm34961	0,436676	-0,00274	0,019	0,024	1
Ctdsp1	0,436843	0,039258	0,423	0,416	1
Smc2	0,43692	0,00906	0,221	0,242	1
Nudt18	0,436932	-0,01504	0,233	0,25	1
Gm7967	0,437084	0,010286	0,04	0,049	1
Zfp639	0,437168	-0,0044	0,383	0,411	1
Tsc22d2	0,437372	0,029128	0,464	0,446	1
Fiz1	0,437444	0,023083	0,328	0,312	1
Sugct	0,437479	0,005243	0,038	0,031	1
Exosc8	0,437609	0,017339	0,293	0,278	1
Alg14	0,437899	-0,01733	0,334	0,353	1
Rab11fip1	0,437979	0,005871	0,001	0	1
Oxtr	0,437979	0,004895	0,001	0	1
Eln	0,437979	0,003918	0,001	0	1
Rcn3	0,437979	0,003918	0,001	0	1
Lamb2	0,437979	0,003918	0,001	0	1
Chrm1	0,437979	0,003918	0,001	0	1
Prrg4	0,437979	0,00294	0,001	0	1
Ccdc120	0,437979	0,00294	0,001	0	1
Adamts10	0,437979	0,00294	0,001	0	1
4933407L2	0,437979	0,001961	0,001	0	1
Ptgs2	0,437979	0,001961	0,001	0	1
Ildr2	0,437979	0,001961	0,001	0	1
Cdca7	0,437979	0,001961	0,001	0	1
4933423P2	0,437979	0,001961	0,001	0	1
Stoml3	0,437979	0,001961	0,001	0	1
Cd53	0,437979	0,001961	0,001	0	1
Smim17	0,437979	0,001961	0,001	0	1
Dll3	0,437979	0,001961	0,001	0	1
Lilr4b	0,437979	0,001961	0,001	0	1
Kcnmb4os2	0,437979	0,001961	0,001	0	1
Adgrg3	0,437979	0,001961	0,001	0	1
Tsnaxip1	0,437979	0,001961	0,001	0	1
Nid2	0,437979	0,001961	0,001	0	1
Dleu7	0,437979	0,001961	0,001	0	1
Vwc2	0,437979	0,001961	0,001	0	1
Havcr2	0,437979	0,001961	0,001	0	1
Rap1gap2	0,437979	0,001961	0,001	0	1
Hk3	0,437979	0,001961	0,001	0	1
Cdh18	0,437979	0,001961	0,001	0	1
Cacng2	0,437979	0,001961	0,001	0	1
Gda	0,437979	0,001961	0,001	0	1
Sulf1	0,437979	0,000981	0,001	0	1
Sp110	0,437979	0,000981	0,001	0	1

2810459M	0,437979	0,000981	0,001	0	1
Inpp5d	0,437979	0,000981	0,001	0	1
Bok	0,437979	0,000981	0,001	0	1
Tmem37	0,437979	0,000981	0,001	0	1
Ctse	0,437979	0,000981	0,001	0	1
Prelp	0,437979	0,000981	0,001	0	1
F11r	0,437979	0,000981	0,001	0	1
Gm34342	0,437979	0,000981	0,001	0	1
Kcnh1	0,437979	0,000981	0,001	0	1
Traf3ip3	0,437979	0,000981	0,001	0	1
Proser2	0,437979	0,000981	0,001	0	1
1700007K1	0,437979	0,000981	0,001	0	1
Ptges	0,437979	0,000981	0,001	0	1
C130021I2	0,437979	0,000981	0,001	0	1
Pamr1	0,437979	0,000981	0,001	0	1
Pak6	0,437979	0,000981	0,001	0	1
Plcb2	0,437979	0,000981	0,001	0	1
Dusp2	0,437979	0,000981	0,001	0	1
Gm14005	0,437979	0,000981	0,001	0	1
Mertk	0,437979	0,000981	0,001	0	1
Necab3	0,437979	0,000981	0,001	0	1
Chst7	0,437979	0,000981	0,001	0	1
Slc9a7	0,437979	0,000981	0,001	0	1
Bgn	0,437979	0,000981	0,001	0	1
Itm2a	0,437979	0,000981	0,001	0	1
Pou3f4	0,437979	0,000981	0,001	0	1
Rps6ka6	0,437979	0,000981	0,001	0	1
Kctd12b	0,437979	0,000981	0,001	0	1
Tlr7	0,437979	0,000981	0,001	0	1
Fabp4	0,437979	0,000981	0,001	0	1
A830092H1	0,437979	0,000981	0,001	0	1
P2ry1	0,437979	0,000981	0,001	0	1
Wdr49	0,437979	0,000981	0,001	0	1
Kirrel	0,437979	0,000981	0,001	0	1
Cd5l	0,437979	0,000981	0,001	0	1
Lrrc71	0,437979	0,000981	0,001	0	1
Tnfaip8l2	0,437979	0,000981	0,001	0	1
Spag17	0,437979	0,000981	0,001	0	1
Dennd2c	0,437979	0,000981	0,001	0	1
5330417C2	0,437979	0,000981	0,001	0	1
Plppr4	0,437979	0,000981	0,001	0	1
Slc44a3	0,437979	0,000981	0,001	0	1
Ndst3	0,437979	0,000981	0,001	0	1
Dapp1	0,437979	0,000981	0,001	0	1
Col24a1	0,437979	0,000981	0,001	0	1
Slc44a5	0,437979	0,000981	0,001	0	1
Gm11837	0,437979	0,000981	0,001	0	1
Podn	0,437979	0,000981	0,001	0	1
Pdzk1ip1	0,437979	0,000981	0,001	0	1
Fgr	0,437979	0,000981	0,001	0	1
Cnr2	0,437979	0,000981	0,001	0	1
Lrrc38	0,437979	0,000981	0,001	0	1

Tnfrsf1b	0,437979	0,000981	0,001	0	1
Otof	0,437979	0,000981	0,001	0	1
Igfbp7	0,437979	0,000981	0,001	0	1
Stbd1	0,437979	0,000981	0,001	0	1
Prdm8	0,437979	0,000981	0,001	0	1
Prkg2	0,437979	0,000981	0,001	0	1
Ksr2	0,437979	0,000981	0,001	0	1
Sdsl	0,437979	0,000981	0,001	0	1
Morn3	0,437979	0,000981	0,001	0	1
Vgf	0,437979	0,000981	0,001	0	1
Tfr2	0,437979	0,000981	0,001	0	1
Gm15410	0,437979	0,000981	0,001	0	1
Gm3294	0,437979	0,000981	0,001	0	1
Prrt4	0,437979	0,000981	0,001	0	1
Hoxa10	0,437979	0,000981	0,001	0	1
Gm16499	0,437979	0,000981	0,001	0	1
Gprin3	0,437979	0,000981	0,001	0	1
Tnip3	0,437979	0,000981	0,001	0	1
Prokr1	0,437979	0,000981	0,001	0	1
Fam19a4	0,437979	0,000981	0,001	0	1
Vwf	0,437979	0,000981	0,001	0	1
9330179D1	0,437979	0,000981	0,001	0	1
Prmt8	0,437979	0,000981	0,001	0	1
Arhgdib	0,437979	0,000981	0,001	0	1
Rerg	0,437979	0,000981	0,001	0	1
Lrmp	0,437979	0,000981	0,001	0	1
Pira2	0,437979	0,000981	0,001	0	1
Lair1	0,437979	0,000981	0,001	0	1
Syt5	0,437979	0,000981	0,001	0	1
Shisa7	0,437979	0,000981	0,001	0	1
Bcam	0,437979	0,000981	0,001	0	1
Hcst	0,437979	0,000981	0,001	0	1
Dmkn	0,437979	0,000981	0,001	0	1
E2f8	0,437979	0,000981	0,001	0	1
A730056A0	0,437979	0,000981	0,001	0	1
Acan	0,437979	0,000981	0,001	0	1
Gm35040	0,437979	0,000981	0,001	0	1
Cfap161	0,437979	0,000981	0,001	0	1
A230065N1	0,437979	0,000981	0,001	0	1
P2ry6	0,437979	0,000981	0,001	0	1
Folr1	0,437979	0,000981	0,001	0	1
Gm4070	0,437979	0,000981	0,001	0	1
Igsf6	0,437979	0,000981	0,001	0	1
Gsg1l	0,437979	0,000981	0,001	0	1
Doc2a	0,437979	0,000981	0,001	0	1
Chst15	0,437979	0,000981	0,001	0	1
Sprn	0,437979	0,000981	0,001	0	1
Myb	0,437979	0,000981	0,001	0	1
Cabcoco1	0,437979	0,000981	0,001	0	1
Trpm2	0,437979	0,000981	0,001	0	1
Slc1a6	0,437979	0,000981	0,001	0	1
Shc2	0,437979	0,000981	0,001	0	1

Grin3b	0,437979	0,000981	0,001	0	1
Ascl1	0,437979	0,000981	0,001	0	1
Cfap54	0,437979	0,000981	0,001	0	1
Ntn4	0,437979	0,000981	0,001	0	1
Lin7a	0,437979	0,000981	0,001	0	1
E2f7	0,437979	0,000981	0,001	0	1
Grip1	0,437979	0,000981	0,001	0	1
Myo16	0,437979	0,000981	0,001	0	1
Angpt2	0,437979	0,000981	0,001	0	1
Adam5	0,437979	0,000981	0,001	0	1
Hapln4	0,437979	0,000981	0,001	0	1
Nod2	0,437979	0,000981	0,001	0	1
Irx3os	0,437979	0,000981	0,001	0	1
Bean1	0,437979	0,000981	0,001	0	1
Slc9a5	0,437979	0,000981	0,001	0	1
Hydin	0,437979	0,000981	0,001	0	1
Spire2	0,437979	0,000981	0,001	0	1
Dnah12	0,437979	0,000981	0,001	0	1
1810011H	0,437979	0,000981	0,001	0	1
Wdfy4	0,437979	0,000981	0,001	0	1
Gch1	0,437979	0,000981	0,001	0	1
Phf11b	0,437979	0,000981	0,001	0	1
Arl11	0,437979	0,000981	0,001	0	1
Fam167a	0,437979	0,000981	0,001	0	1
Pbk	0,437979	0,000981	0,001	0	1
Rubcnl	0,437979	0,000981	0,001	0	1
Fam216b	0,437979	0,000981	0,001	0	1
Gm4675	0,437979	0,000981	0,001	0	1
Pgr	0,437979	0,000981	0,001	0	1
Gm26592	0,437979	0,000981	0,001	0	1
Layn	0,437979	0,000981	0,001	0	1
Cgnl1	0,437979	0,000981	0,001	0	1
Ddx43	0,437979	0,000981	0,001	0	1
Plscr4	0,437979	0,000981	0,001	0	1
Cx3cr1	0,437979	0,000981	0,001	0	1
Osm	0,437979	0,000981	0,001	0	1
Myo1g	0,437979	0,000981	0,001	0	1
Igfbp3	0,437979	0,000981	0,001	0	1
C1qtnf2	0,437979	0,000981	0,001	0	1
Shisa6	0,437979	0,000981	0,001	0	1
Tekt1	0,437979	0,000981	0,001	0	1
Coro6	0,437979	0,000981	0,001	0	1
Adap2	0,437979	0,000981	0,001	0	1
Nog	0,437979	0,000981	0,001	0	1
Abi3	0,437979	0,000981	0,001	0	1
Skap1	0,437979	0,000981	0,001	0	1
2810433D	0,437979	0,000981	0,001	0	1
Gjc1	0,437979	0,000981	0,001	0	1
Cd300lb	0,437979	0,000981	0,001	0	1
Gng4	0,437979	0,000981	0,001	0	1
Hist1h1b	0,437979	0,000981	0,001	0	1
Gcnt2	0,437979	0,000981	0,001	0	1

S1pr3	0,437979	0,000981	0,001	0	1
Diras2	0,437979	0,000981	0,001	0	1
Dapk1	0,437979	0,000981	0,001	0	1
Pcsk1	0,437979	0,000981	0,001	0	1
Hapln1	0,437979	0,000981	0,001	0	1
AW495222	0,437979	0,000981	0,001	0	1
Iqgap2	0,437979	0,000981	0,001	0	1
F2rl2	0,437979	0,000981	0,001	0	1
Gm5086	0,437979	0,000981	0,001	0	1
Gpx8	0,437979	0,000981	0,001	0	1
Gm33680	0,437979	0,000981	0,001	0	1
Prkch	0,437979	0,000981	0,001	0	1
Kcnk10	0,437979	0,000981	0,001	0	1
Gpr68	0,437979	0,000981	0,001	0	1
Pld4	0,437979	0,000981	0,001	0	1
Cdca7l	0,437979	0,000981	0,001	0	1
Fam105a	0,437979	0,000981	0,001	0	1
Dnah5	0,437979	0,000981	0,001	0	1
Rgs22	0,437979	0,000981	0,001	0	1
Adcy8	0,437979	0,000981	0,001	0	1
Gsdmd	0,437979	0,000981	0,001	0	1
Rac2	0,437979	0,000981	0,001	0	1
Mfng	0,437979	0,000981	0,001	0	1
Kdelr3	0,437979	0,000981	0,001	0	1
Cacna1i	0,437979	0,000981	0,001	0	1
Celsr1	0,437979	0,000981	0,001	0	1
Mapk11	0,437979	0,000981	0,001	0	1
Odf3b	0,437979	0,000981	0,001	0	1
Syt10	0,437979	0,000981	0,001	0	1
AC101921.	0,437979	0,000981	0,001	0	1
Ccdc65	0,437979	0,000981	0,001	0	1
Pkp2	0,437979	0,000981	0,001	0	1
AC118542.	0,437979	0,000981	0,001	0	1
Gp1bb	0,437979	0,000981	0,001	0	1
Gm26569	0,437979	0,000981	0,001	0	1
Hcls1	0,437979	0,000981	0,001	0	1
Cfap44	0,437979	0,000981	0,001	0	1
Ccdc80	0,437979	0,000981	0,001	0	1
Epha3	0,437979	0,000981	0,001	0	1
Chodl	0,437979	0,000981	0,001	0	1
Airn	0,437979	0,000981	0,001	0	1
Fgd2	0,437979	0,000981	0,001	0	1
H2-DMb1	0,437979	0,000981	0,001	0	1
H2-Aa	0,437979	0,000981	0,001	0	1
Lrfn2	0,437979	0,000981	0,001	0	1
Plin5	0,437979	0,000981	0,001	0	1
Tnfaip8l1	0,437979	0,000981	0,001	0	1
Xdh	0,437979	0,000981	0,001	0	1
Kcnk12	0,437979	0,000981	0,001	0	1
Rnf165	0,437979	0,000981	0,001	0	1
Slc14a1	0,437979	0,000981	0,001	0	1
Lrp5	0,437979	0,000981	0,001	0	1

Cdca5	0,437979	0,000981	0,001	0	1
Fermt3	0,437979	0,000981	0,001	0	1
Ms4a6d	0,437979	0,000981	0,001	0	1
Trpm6	0,437979	0,000981	0,001	0	1
Mamdc2	0,437979	0,000981	0,001	0	1
Fas	0,437979	0,000981	0,001	0	1
Ifit1bl1	0,437979	0,000981	0,001	0	1
Hhex	0,437979	0,000981	0,001	0	1
Rbp4	0,437979	0,000981	0,001	0	1
Pnlip	0,437979	0,000981	0,001	0	1
Dmpk	0,438207	0,023043	0,194	0,18	1
Zdhhc12	0,438351	-0,01411	0,26	0,278	1
Parl	0,438374	-0,00836	0,52	0,548	1
Ntsr2	0,438544	-0,00619	0,022	0,028	1
Plxnb1	0,438612	0,044205	0,481	0,47	1
Agrn	0,43868	-0,01983	0,221	0,236	1
Rragc	0,438719	0,030691	0,557	0,545	1
A730063M	0,438763	0,008991	0,047	0,039	1
Atf6	0,43883	-0,02598	0,362	0,377	1
Abhd17a	0,438871	-0,01088	0,843	0,863	1
Gm47802	0,438957	0,006823	0,026	0,02	1
Gm32051	0,438957	0,019699	0,024	0,018	1
Avl9	0,43897	-0,01206	0,475	0,499	1
Isoc2a	0,439139	0,01176	0,202	0,185	1
Hykk	0,439144	-0,00557	0,022	0,028	1
Rcan2	0,439182	0,022377	0,946	0,953	1
Tmem14a	0,439219	-0,01952	0,471	0,488	1
Ppp4r4	0,439244	-0,01265	0,027	0,034	1
Syde1	0,439518	-0,01358	0,113	0,125	1
Apex2	0,439751	-0,0128	0,081	0,093	1
Pard6a	0,439837	0,016467	0,205	0,19	1
Mphosph9	0,440169	-0,02687	0,166	0,179	1
Aldh7a1	0,440208	-0,01615	0,297	0,319	1
Zbtb10	0,440238	-0,02008	0,132	0,145	1
Cpt1c	0,440501	-0,01061	0,255	0,272	1
Fjx1	0,440562	0,025499	0,072	0,062	1
2900055J2	0,440621	0,014842	0,108	0,096	1
Sgtb	0,44075	-0,00585	0,048	0,057	1
Smg8	0,440756	-0,00813	0,081	0,093	1
AI314180	0,441091	-0,03119	0,534	0,538	1
Mrpl40	0,441098	-0,01904	0,381	0,4	1
Slc45a1	0,441382	-0,00721	0,062	0,072	1
Gskip	0,441597	-0,01982	0,259	0,275	1
Inafm2	0,441717	-0,00661	0,302	0,322	1
Cep72	0,441733	-0,00388	0,11	0,124	1
Syngr2	0,441745	-0,02351	0,512	0,528	1
Rpl31	0,441762	0,010484	0,961	0,948	1
Galt	0,441809	-0,00399	0,022	0,028	1
Ppp1r12a	0,441841	-0,0253	0,78	0,802	1
Lcp1	0,442113	-0,01569	0,091	0,102	1
Nucks1	0,44218	-0,02698	0,974	0,99	1
Tars2	0,442207	-0,01694	0,232	0,247	1

Amz1	0,442258	0,00651	0,024	0,018	1
Nhsl2	0,442533	-0,00762	0,012	0,016	1
Fam234b	0,442579	-0,00215	0,444	0,47	1
Slc16a3	0,442603	-0,00497	0,042	0,05	1
Safb2	0,44266	-0,00262	0,594	0,628	1
Ggt7	0,442677	-0,00983	0,233	0,25	1
Fundc2	0,442966	-0,0178	0,423	0,447	1
Slc31a1	0,443132	0,014019	0,083	0,073	1
Tln2	0,443164	0,03013	0,5	0,488	1
Gm8013	0,443232	-0,00647	0,025	0,031	1
Slc25a40	0,443239	-0,00401	0,121	0,135	1
Fam214a	0,443263	-0,01721	0,228	0,244	1
3110082I1	0,443413	-0,00651	0,045	0,054	1
Frat2	0,44344	0,014962	0,07	0,06	1
Ypel2	0,443447	-0,00825	0,972	0,967	1
Itih3	0,443549	-0,01346	0,213	0,231	1
Mef2a	0,44371	0,066533	0,745	0,766	1
Heatr5b	0,443793	-0,00513	0,193	0,211	1
Rab1b	0,444004	-0,01883	0,663	0,668	1
Gtf3c4	0,444137	-0,02188	0,177	0,19	1
Zc3h18	0,444203	0,00078	0,436	0,467	1
Hif1a	0,444307	-0,03763	0,53	0,525	1
Gnpnat1	0,444625	-0,02461	0,241	0,257	1
Prdm4	0,444634	0,010191	0,126	0,112	1
Gm3854	0,444657	0,010858	0,045	0,037	1
Prss36	0,444924	-0,01372	0,07	0,08	1
Ppp1r3f	0,445191	0,01736	0,102	0,091	1
Faf1	0,44523	0,020337	0,315	0,301	1
Ergic3	0,445277	-0,00548	0,722	0,75	1
5033417F2	0,44529	-0,0069	0,039	0,047	1
Pole	0,445485	0,007459	0,024	0,018	1
Zbtb45	0,44553	0,011992	0,113	0,101	1
Zfp759	0,445595	-0,01223	0,053	0,062	1
Gadd45gip	0,445775	0,005871	0,503	0,473	1
Plag1	0,445937	0,014084	0,104	0,093	1
Shpk	0,446084	-0,00505	0,012	0,016	1
Tmtc1	0,446404	-0,00442	0,012	0,016	1
Nsg2	0,446404	-0,00442	0,012	0,016	1
Ralb	0,446597	-0,00221	0,198	0,216	1
Arap1	0,446761	1,95E-05	0,292	0,319	1
9330020H0	0,446811	0,006501	0,036	0,029	1
Cpsf1	0,447133	-0,00978	0,188	0,205	1
Ubxn4	0,447174	0,026795	0,847	0,834	1
Il17ra	0,447221	0,019389	0,102	0,091	1
Zfp873	0,447344	-0,0058	0,03	0,037	1
Zfp97	0,447381	-0,00568	0,012	0,016	1
Pemt	0,447401	0,010291	0,034	0,028	1
Scd1	0,447404	0,000881	0,736	0,743	1
Gle1	0,447435	-0,0206	0,414	0,429	1
Mdm4	0,447948	-0,00631	0,676	0,65	1
Dsn1	0,448026	-0,00345	0,012	0,016	1
Acot8	0,448031	-0,01276	0,218	0,236	1

Pcdh19	0,448034	0,001402	0,012	0,016	1
Tmem150c	0,448136	0,000384	0,415	0,447	1
2410004B:	0,448187	-0,0305	0,505	0,512	1
Slc35e4	0,448217	-0,009	0,479	0,499	1
Rab31	0,448509	0,037617	0,932	0,958	1
Gemin2	0,448704	0,013077	0,164	0,15	1
Gap43	0,448835	-0,00069	0,038	0,031	1
Arhgap31	0,448927	-0,0076	0,318	0,345	1
Arhgap33	0,448945	-0,01387	0,225	0,241	1
Larp4	0,448986	-0,01306	0,551	0,571	1
Ppp6c	0,449109	0,011831	0,548	0,515	1
Fstl3	0,449127	0,003684	0,033	0,041	1
Rtcb	0,44926	-0,02435	0,565	0,574	1
Gabpb1	0,449264	0,012218	0,12	0,107	1
Camkmt	0,44957	0,015207	0,081	0,072	1
Gas8	0,449683	-0,01685	0,162	0,176	1
Rfc3	0,449732	2,04E-05	0,14	0,156	1
Bri3bp	0,449853	0,017164	0,155	0,141	1
Krt10	0,4499	-0,00726	0,261	0,283	1
Nfia	0,450044	0,035412	0,897	0,893	1
Gtf2i	0,450209	0,038271	0,782	0,769	1
Ubl4a	0,450424	-0,01776	0,261	0,276	1
Aldh16a1	0,450433	0,015538	0,136	0,124	1
Plk4	0,450559	0,020664	0,081	0,072	1
Zfp426	0,450561	-0,00926	0,187	0,203	1
Sharpin	0,450847	-0,01869	0,247	0,262	1
Usp34	0,450848	0,019826	0,742	0,73	1
Zwilch	0,450944	0,008684	0,045	0,037	1
Snap91	0,450949	0,002187	0,123	0,109	1
Pdhb	0,451061	-0,00199	0,574	0,597	1
Zscan22	0,451157	-0,00802	0,103	0,115	1
Arhgef6	0,45128	-0,00152	0,012	0,016	1
Iqsec2	0,451365	-0,00356	0,027	0,034	1
A430106G:	0,451405	0,018498	0,118	0,133	1
Spg7	0,45149	-0,00451	0,366	0,392	1
Cog6	0,451773	-0,01423	0,22	0,236	1
Kat6b	0,451859	0,005383	0,235	0,255	1
Cep85	0,451878	-0,01248	0,158	0,172	1
D17H6S53I	0,451914	0,011194	0,149	0,135	1
Fuca2	0,451991	-0,01854	0,174	0,187	1
Ubxn7	0,452172	-0,01044	0,375	0,4	1
Drap1	0,452183	0,031158	0,847	0,828	1
Ttbk2	0,452234	-0,02698	0,388	0,405	1
Unc50	0,452265	-0,00953	0,759	0,78	1
Tmem67	0,452284	-0,00795	0,092	0,104	1
Ctif	0,452339	-0,03823	0,256	0,268	1
Eif5	0,452428	0,021364	0,956	0,946	1
Puf60	0,452767	0,017551	0,882	0,888	1
Tspan2os	0,452857	0,00902	0,034	0,028	1
Fam219b	0,452858	-0,02322	0,25	0,265	1
Ddhd1	0,452868	0,085244	0,66	0,657	1
Mbd1	0,452975	-0,01824	0,255	0,272	1

Palm	0,45299	0,035005	0,214	0,203	1
Malsu1	0,453025	-0,02292	0,318	0,332	1
Efna2	0,453105	0,012254	0,271	0,252	1
Dtx2	0,453281	-0,00018	0,112	0,125	1
Fez2	0,453381	0,023334	0,572	0,558	1
Arid4a	0,453467	-0,01358	0,43	0,447	1
Bag1	0,453475	-0,02202	0,409	0,426	1
Txlna	0,45348	-0,03742	0,583	0,598	1
Snw1	0,453559	-0,00631	0,807	0,834	1
Trrap	0,453686	-0,01097	0,395	0,418	1
Ccdc9	0,453687	-0,01294	0,144	0,158	1
Coq2	0,453819	-0,01277	0,391	0,411	1
1700029J0	0,453839	-0,00703	0,114	0,127	1
Rnf19a	0,453858	0,001061	0,342	0,366	1
Mbtps2	0,454065	-0,00411	0,271	0,289	1
Tmem198b	0,454067	-0,00196	0,033	0,041	1
Mrps18b	0,45415	0,045136	0,345	0,345	1
Xkr8	0,454437	-0,00682	0,065	0,075	1
D10Wsu10	0,454481	0,072018	0,473	0,468	1
BC017158	0,454568	0,012988	0,052	0,044	1
Pip4k2c	0,454613	-0,00252	0,424	0,455	1
Nsg1	0,454712	-0,0156	0,044	0,052	1
Heatr5a	0,454736	0,034754	0,275	0,262	1
Ctc1	0,454792	-0,00894	0,138	0,153	1
Sys1	0,454803	-0,02611	0,919	0,937	1
Gm20342	0,454857	0,002662	0,256	0,276	1
Brsk1	0,454884	0,014129	0,264	0,246	1
Map2k7	0,455012	0,011365	0,511	0,493	1
Thap4	0,455195	0,011537	0,141	0,128	1
Dbnl	0,455442	0,023348	0,52	0,517	1
Nr2c2ap	0,455472	0,020797	0,4	0,385	1
Nap1l2	0,455519	0,009066	0,02	0,015	1
Aup1	0,455643	0,016261	0,567	0,548	1
Txndc11	0,455741	0,030497	0,17	0,158	1
Anapc13	0,455806	-0,02106	0,705	0,724	1
Rdm1	0,456095	0,02421	0,172	0,159	1
Onecut2	0,456201	-0,02424	0,15	0,163	1
Cyb561d2	0,45636	0,033542	0,309	0,296	1
Calm3	0,456487	-0,02607	0,969	0,971	1
Iqcc	0,456523	-0,01579	0,18	0,193	1
Pigo	0,456524	0,014594	0,075	0,065	1
Foxk1	0,456584	-0,00489	0,201	0,218	1
Fzd7	0,456654	-0,00326	0,146	0,161	1
Pnpo	0,456655	0,006832	0,02	0,015	1
Ern1	0,456658	-0,01362	0,085	0,096	1
Zfp777	0,456789	0,00819	0,115	0,102	1
Zscan26	0,456978	0,025301	0,395	0,376	1
Cdca4	0,457031	-0,0001	0,03	0,037	1
Bbs7	0,457141	-0,02182	0,077	0,086	1
Setx	0,457228	0,018507	0,422	0,46	1
Xpo4	0,457363	-0,01094	0,287	0,307	1
Mbtd1	0,457384	0,034042	0,386	0,374	1

Lsmem1	0,457772	0,00749	0,006	0,003	1
1810043G(	0,457857	-0,00375	0,237	0,257	1
Gm10273	0,457942	0,005871	0,02	0,015	1
Ttll1	0,458069	-0,00689	0,111	0,124	1
Dok1	0,458092	0,009066	0,02	0,015	1
Fyttd1	0,458266	0,032119	0,548	0,538	1
Psma7	0,45827	0,018564	0,96	0,966	1
Nrxn2	0,458286	-0,03061	0,691	0,701	1
Abcd1	0,458474	0,022868	0,186	0,174	1
Dnajc9	0,458546	0,026287	0,325	0,311	1
Tceanc2	0,458714	-0,01255	0,232	0,249	1
Gm15910	0,458857	0,004573	0,006	0,003	1
AC125141.	0,458857	0,006519	0,006	0,003	1
Olfr267	0,458857	0,005547	0,006	0,003	1
Arhgef15	0,458857	0,005547	0,006	0,003	1
Atl1	0,458895	0,034719	0,411	0,402	1
Wdr18	0,458918	-0,01369	0,346	0,364	1
Klhdc8b	0,458961	-0,00749	0,015	0,02	1
Bahd1	0,459112	-0,01116	0,078	0,088	1
Zbtb44	0,459319	0,022776	0,358	0,343	1
Tdrp	0,4594	0,005547	0,006	0,003	1
Rfx2	0,4594	0,004573	0,006	0,003	1
A830008E2	0,4594	0,003599	0,006	0,003	1
Gm37634	0,4594	0,003599	0,006	0,003	1
Olfr1124	0,4594	0,003599	0,006	0,003	1
Sap25	0,4594	0,003599	0,006	0,003	1
Rasgef1a	0,4594	0,003599	0,006	0,003	1
Gm26540	0,4594	0,003599	0,006	0,003	1
Zfp82	0,4594	0,003599	0,006	0,003	1
Adam12	0,4594	0,003599	0,006	0,003	1
Llgl2	0,4594	0,003599	0,006	0,003	1
Lrrc8e	0,459525	0,005231	0,02	0,015	1
Plekha5	0,459579	0,00112	0,286	0,267	1
Gm13563	0,459942	0,002624	0,006	0,003	1
Dcst1	0,459942	0,002624	0,006	0,003	1
Gm15477	0,459942	0,002624	0,006	0,003	1
Gm42556	0,459942	0,002624	0,006	0,003	1
Pcsk4	0,459942	0,002624	0,006	0,003	1
Btbd11	0,459942	0,002624	0,006	0,003	1
4932443I1	0,459942	0,002624	0,006	0,003	1
Gm47794	0,459942	0,002624	0,006	0,003	1
AC061963.	0,459942	0,002624	0,006	0,003	1
Smad6	0,459942	0,002624	0,006	0,003	1
Adora2b	0,459942	0,002624	0,006	0,003	1
A730081D(	0,459942	0,002624	0,006	0,003	1
Stmnd1	0,459942	0,002624	0,006	0,003	1
Grhl2	0,459942	0,002624	0,006	0,003	1
Gm15738	0,459942	0,002624	0,006	0,003	1
1700022N:	0,459942	0,002624	0,006	0,003	1
G430049J(	0,459942	0,002624	0,006	0,003	1
Pcdha8	0,459942	0,002624	0,006	0,003	1
Gm16105	0,460215	0,0049	0,006	0,003	1

Pigh	0,460269	-0,01392	0,154	0,167	1
Rmnd1	0,460316	-0,01371	0,281	0,299	1
Pomt1	0,460364	0,017306	0,149	0,137	1
Traip	0,460385	0,014183	0,018	0,013	1
Sf3b3	0,460596	-0,0094	0,489	0,499	1
Jmjd8	0,460757	-0,00757	0,155	0,169	1
Ccne1	0,46076	0,00231	0,006	0,003	1
Mr1	0,460971	0,004277	0,02	0,015	1
Txnip	0,460979	0,048129	0,447	0,437	1
Dpysl4	0,461031	0,00198	0,006	0,003	1
Eif4ebp1	0,461031	0,00198	0,006	0,003	1
Colgalt2	0,461083	0,018751	0,057	0,049	1
Tomm7	0,461094	-0,01861	0,868	0,867	1
Ngly1	0,46115	0,007058	0,303	0,283	1
Kyat3	0,461225	-0,00722	0,156	0,171	1
Sap30l	0,461276	-0,00843	0,391	0,408	1
Zik1	0,461401	-0,00985	0,047	0,055	1
Cox18	0,461515	-0,01447	0,144	0,158	1
Ngef	0,461575	0,001005	0,006	0,003	1
Postn	0,461575	0,001005	0,006	0,003	1
Drd4	0,461575	0,001005	0,006	0,003	1
5830418P1	0,461575	0,001005	0,006	0,003	1
Gm12278	0,461575	0,001005	0,006	0,003	1
4930408O1	0,461575	0,001005	0,006	0,003	1
Gm26807	0,461575	-0,00061	0,006	0,003	1
Hist2h2be	0,461598	0,008436	0,018	0,013	1
Pdcd10	0,461703	-0,00886	0,804	0,82	1
Vdac1	0,46175	-0,0168	0,674	0,685	1
Rsph10b	0,461764	0,012754	0,041	0,034	1
Kansl1l	0,461773	0,004567	0,342	0,369	1
mt-Nd1	0,461821	0,031438	0,999	1	1
Gtpbp2	0,461832	-0,01379	0,365	0,382	1
Hif1an	0,461844	0,016101	0,242	0,226	1
Clock	0,46196	0,05202	0,71	0,727	1
Mrm3	0,462107	0,012764	0,088	0,078	1
Slc24a5	0,462152	-0,02135	0,434	0,447	1
Cdc23	0,462153	-0,0137	0,295	0,312	1
Tmem161a	0,462594	-0,01922	0,427	0,444	1
Mpst	0,462619	-0,01657	0,699	0,733	1
Lsm10	0,462642	-0,01865	0,208	0,223	1
Rflnb	0,462681	-0,00562	0,015	0,02	1
Mga	0,462712	0,034797	0,522	0,509	1
Mamstr	0,462813	0,007475	0,018	0,013	1
Gm26910	0,463227	0,012243	0,075	0,065	1
Prkab2	0,463286	0,019477	0,071	0,062	1
Lpcat4	0,463441	-0,00751	0,103	0,115	1
Lactb2	0,463551	-0,00846	0,235	0,252	1
Rcor2	0,463578	0,010022	0,018	0,013	1
Gm13483	0,46358	-0,00878	0,064	0,073	1
Lman1	0,463591	0,036267	0,277	0,263	1
Ap4m1	0,463673	0,007931	0,191	0,21	1
Spa17	0,463842	0,011411	0,059	0,05	1

Fgfbp3	0,463848	-0,00707	0,197	0,215	1
Mmadhc	0,464083	-0,00263	0,531	0,559	1
Lad1	0,464191	0,007467	0,018	0,013	1
Plekhj1	0,464289	0,043422	0,571	0,563	1
Siah1a	0,46447	-0,00671	0,391	0,416	1
Tradd	0,464648	-0,00248	0,27	0,291	1
Smim8	0,464693	-0,01729	0,312	0,33	1
Ndufb3	0,464718	-0,01099	0,836	0,855	1
Gm2885	0,464974	-0,01099	0,018	0,023	1
Tinf2	0,465053	-0,01487	0,131	0,143	1
Cacnb1	0,465201	-0,00199	0,056	0,065	1
Msrb2	0,465358	0,015292	0,073	0,063	1
Rnf113a1	0,465557	0,005871	0,018	0,013	1
Sf3a3	0,465615	0,013354	0,401	0,382	1
Casp9	0,465749	-0,01528	0,208	0,223	1
Cnot3	0,465822	0,030691	0,559	0,543	1
Cobl	0,465827	-0,00223	0,387	0,407	1
Mtmr1	0,465864	-0,02492	0,234	0,247	1
Arrdc4	0,466355	0,008077	0,032	0,026	1
A930024N:	0,466476	0,010022	0,018	0,013	1
Caprin2	0,466564	0,024547	0,086	0,076	1
Mvd	0,466638	0,047755	0,273	0,26	1
Bnip2	0,466708	-0,01904	0,343	0,354	1
Ric1	0,466897	-0,02852	0,268	0,281	1
Prorsd1	0,46697	0,010943	0,138	0,125	1
Parp2	0,467067	-0,00706	0,172	0,187	1
B230322F(	0,467106	-0,0051	0,008	0,011	1
Olfr742	0,467106	-0,0051	0,008	0,011	1
Esyt3	0,467106	-0,0051	0,008	0,011	1
Lgi1	0,467106	-0,0051	0,008	0,011	1
Tead3	0,467106	-0,00671	0,008	0,011	1
Rps27l	0,46724	0,018907	0,751	0,725	1
Gnl2	0,467288	-0,01446	0,381	0,402	1
Usp15	0,467668	0,039503	0,619	0,62	1
St6galnac3	0,467878	0,042373	0,756	0,753	1
Nfrkb	0,467906	0,001315	0,276	0,298	1
Gm10282	0,467999	0,003946	0,018	0,013	1
Siah1b	0,468046	-0,01536	0,049	0,057	1
Cyp4f13	0,468154	-0,02379	0,275	0,286	1
Stmn4	0,468251	0,011873	0,999	1	1
Trappc4	0,468291	-0,01908	0,725	0,748	1
Olfr1369-p	0,468312	-0,05122	0,018	0,013	1
Rnf220	0,468401	0,02732	0,78	0,769	1
Rps15	0,468407	0,03488	0,996	0,997	1
Mirt1	0,468412	-0,00963	0,004	0,007	1
Acp6	0,468462	-0,01087	0,138	0,151	1
Txnl1	0,468489	0,02403	0,818	0,81	1
Pms2	0,468533	0,008458	0,214	0,197	1
Bcl6	0,468624	0,016342	0,205	0,19	1
Tmem35a	0,468686	-0,00418	0,004	0,007	1
Olfr77	0,468686	-0,00418	0,004	0,007	1
Gm9796	0,468686	-0,00418	0,004	0,007	1

Tcap	0,468686	-0,00418	0,004	0,007	1
Pla2g4c	0,468687	-0,00481	0,004	0,007	1
Jph3	0,468712	-0,0035	0,008	0,011	1
Tll2	0,468712	-0,0035	0,008	0,011	1
Hsbp1	0,468751	0,023435	0,984	0,976	1
Fnip2	0,468843	0,023798	0,237	0,223	1
Zfp341	0,468984	-0,0053	0,044	0,052	1
Tmem214	0,469019	0,032372	0,314	0,298	1
Frmd5	0,469072	0,039836	0,723	0,733	1
Dnah8	0,469124	-0,00085	0,015	0,02	1
Fam76a	0,469252	0,02489	0,704	0,702	1
Znrd1	0,469447	0,025473	0,37	0,356	1
Bdnf	0,469511	-0,00481	0,004	0,007	1
Myh10	0,469519	-0,00476	0,008	0,011	1
Ikbkap	0,469622	-0,00619	0,208	0,224	1
Gm36757	0,469706	-0,01215	0,064	0,073	1
Cnot8	0,46973	0,012046	0,347	0,379	1
Ctxn2	0,469785	-0,00257	0,004	0,007	1
Gm14597	0,469785	-0,00257	0,004	0,007	1
Mycl	0,469785	-0,00257	0,004	0,007	1
Map4k1	0,469785	-0,00257	0,004	0,007	1
Nphs1	0,469785	-0,00257	0,004	0,007	1
Zfp977	0,469785	-0,00257	0,004	0,007	1
Rsf1os1	0,469785	-0,00257	0,004	0,007	1
Olfr691	0,469785	-0,00257	0,004	0,007	1
Gm10131	0,469785	-0,00257	0,004	0,007	1
C130073E2	0,469785	-0,00257	0,004	0,007	1
Gm45639	0,469785	-0,00257	0,004	0,007	1
Gm48940	0,469785	-0,00257	0,004	0,007	1
Gm36278	0,469785	-0,00257	0,004	0,007	1
A730085K(	0,469785	-0,00257	0,004	0,007	1
Nxn	0,469785	-0,00257	0,004	0,007	1
Rab11fip4c	0,469785	-0,00257	0,004	0,007	1
Gm20319	0,469785	-0,00257	0,004	0,007	1
Rit2	0,469785	-0,00257	0,004	0,007	1
A330009N1	0,469786	-0,0032	0,004	0,007	1
Hectd2	0,469786	-0,0032	0,004	0,007	1
Gm26792	0,469786	-0,00384	0,004	0,007	1
Ttc25	0,469934	-0,00667	0,047	0,055	1
Tmem245	0,469963	0,039364	0,679	0,673	1
Dcun1d3	0,469966	-0,00213	0,179	0,197	1
E130102H2	0,469989	-0,01106	0,248	0,267	1
Sdc1	0,470114	0,039366	0,039	0,033	1
Slc6a20b	0,470121	0,00039	0,008	0,011	1
Gm13528	0,470121	-0,00252	0,008	0,011	1
Cdyl2	0,470121	-0,00252	0,008	0,011	1
Gm40557	0,470121	-0,00252	0,008	0,011	1
Gusb	0,470288	0,016417	0,275	0,26	1
Frat1	0,470394	0,016437	0,078	0,068	1
1300002E1	0,470401	-0,01199	0,22	0,236	1
Ube2i	0,470577	0,015709	0,631	0,62	1
9930111J2	0,470612	-0,00413	0,004	0,007	1

Zfp948	0,470613	0,026479	0,174	0,163	1
Dcaf1	0,470706	0,013608	0,274	0,257	1
Mrnip	0,470867	0,000401	0,062	0,072	1
Cacna1e	0,470886	-0,00159	0,004	0,007	1
Lpar3	0,470886	-0,00159	0,004	0,007	1
Ankrd6	0,470886	-0,00159	0,004	0,007	1
Gm47113	0,470886	-0,00159	0,004	0,007	1
Gria1	0,470886	-0,00159	0,004	0,007	1
Gm12264	0,470886	-0,00159	0,004	0,007	1
C130060C(	0,470886	-0,00159	0,004	0,007	1
Svep1	0,470887	-0,00223	0,004	0,007	1
I830134HC	0,470887	-0,00223	0,004	0,007	1
AI854703	0,471162	-0,00125	0,004	0,007	1
Hoxd9	0,471189	-0,01748	0,109	0,12	1
Vps9d1	0,471222	0,001591	0,275	0,296	1
Gm26759	0,47127	-0,00092	0,047	0,055	1
Gpatch8	0,471475	-0,00656	0,889	0,906	1
Acd	0,471475	-0,02603	0,156	0,167	1
Cep55	0,471533	-0,00155	0,008	0,011	1
Osgepl1	0,471541	-0,01735	0,142	0,154	1
Idh3a	0,471565	-0,00982	0,435	0,462	1
Nbea	0,471584	-0,0141	0,644	0,66	1
Zdhhc9	0,471676	0,032888	0,697	0,702	1
Eloc	0,47168	-0,0081	0,839	0,859	1
Timp3	0,471705	-0,02823	0,052	0,06	1
Nol6	0,47172	2,6E-05	0,189	0,207	1
Pex11a	0,47182	0,011262	0,081	0,072	1
T2	0,471864	0,008436	0,016	0,011	1
Fnbp1l	0,471867	0,007792	0,016	0,011	1
Gm6563	0,471907	0,009035	0,03	0,024	1
Pcdhb16	0,471912	-0,0074	0,094	0,106	1
Nusap1	0,471989	-0,00061	0,004	0,007	1
Gm16302	0,471989	0,000363	0,004	0,007	1
Gm5617	0,471995	-0,00385	0,249	0,268	1
Tctn3	0,472054	0,012955	0,057	0,049	1
Dusp8	0,472137	0,017531	0,406	0,389	1
Rnf144a	0,472285	-0,01151	0,326	0,345	1
Plekhg5	0,472358	0,003632	0,016	0,011	1
Abcb9	0,472366	0,005005	0,117	0,104	1
BC031181	0,472373	-0,01324	0,913	0,935	1
Dzip3	0,472448	-0,00867	0,242	0,262	1
Uvrag	0,47275	0,000999	0,257	0,276	1
Fam109a	0,472823	-0,00763	0,099	0,111	1
Podxl	0,472946	-0,00058	0,008	0,011	1
Gm12355	0,472946	-0,00058	0,008	0,011	1
Gm20604	0,472956	0,005871	0,052	0,044	1
Calm2	0,473064	0,033927	0,995	0,995	1
Noc3l	0,473098	-0,01067	0,189	0,205	1
Mapkapk2	0,473166	-0,01073	0,367	0,389	1
Acat3	0,473268	-0,00403	0,018	0,023	1
Ucn2	0,473318	0,067979	0,008	0,005	1
Dnajc17	0,473792	-0,01593	0,245	0,26	1

Saysd1	0,473886	-0,01117	0,24	0,257	1
Fmo5	0,474066	0,004656	0,059	0,068	1
Cdc42bpg	0,474183	0,007801	0,014	0,01	1
Gm42670	0,474186	0,010042	0,014	0,01	1
Gm36356	0,474231	-0,01713	0,09	0,101	1
Ebag9	0,474295	-0,00776	0,235	0,252	1
Zbtb8a	0,474315	0,004586	0,016	0,011	1
Tceal1	0,474612	0,001284	0,315	0,34	1
Gsap	0,474733	0,007811	0,008	0,005	1
Gm26582	0,474733	0,008779	0,008	0,005	1
Gm5535	0,474807	-0,00532	0,067	0,076	1
Nek4	0,474955	0,008786	0,251	0,273	1
AC154507.	0,47522	-0,00307	0,018	0,023	1
Gm43254	0,475236	0,006837	0,014	0,01	1
Cct6b	0,475414	0,008119	0,014	0,01	1
Chchd5	0,475439	-0,00623	0,232	0,247	1
Wwc1	0,475441	0,0049	0,008	0,005	1
Syt16	0,475441	0,0049	0,008	0,005	1
Alg5	0,475545	0,034794	0,362	0,353	1
Rab29	0,475718	-0,00712	0,041	0,049	1
Tnfrsf22	0,475748	0,004604	0,03	0,024	1
Rabgef1	0,475946	-0,00869	0,202	0,22	1
Nrip2	0,475984	-0,00831	0,029	0,036	1
Nbeal2	0,476147	-0,00591	0,021	0,026	1
Gm2447	0,476149	0,005871	0,008	0,005	1
Olfr1307	0,476149	0,003927	0,008	0,005	1
Ppp1r27	0,476149	0,003927	0,008	0,005	1
Napa	0,476203	-0,00366	0,892	0,911	1
Cttnbp2	0,476245	-0,03197	0,686	0,689	1
Xk	0,476277	-0,00495	0,021	0,026	1
Slco2a1	0,476289	0,007801	0,014	0,01	1
Ccdc149	0,476289	0,005871	0,014	0,01	1
Vegfd	0,476858	0,002954	0,008	0,005	1
Lgals7	0,476858	0,002954	0,008	0,005	1
Gm27030	0,476858	0,002954	0,008	0,005	1
Gng2	0,476858	0,002954	0,008	0,005	1
Cd300a	0,476858	0,002954	0,008	0,005	1
Foxc1	0,476858	0,002954	0,008	0,005	1
Eif4e3	0,476921	-0,04568	0,219	0,231	1
Caap1	0,476929	-0,00699	0,238	0,255	1
Cenpa	0,476933	-0,00563	0,041	0,049	1
Braf	0,477035	-0,01885	0,415	0,433	1
Gm37584	0,477097	0,004254	0,008	0,005	1
Sox6	0,477147	0,008448	0,012	0,008	1
Lipt1	0,477176	-0,00211	0,018	0,023	1
Tnr	0,477176	-0,00019	0,018	0,023	1
Cbr4	0,477223	-0,00772	0,131	0,143	1
Gm14327	0,477344	0,004904	0,014	0,01	1
Gm17227	0,477344	0,004904	0,014	0,01	1
B930036N	0,47745	-0,0176	0,054	0,046	1
Tnip1	0,477503	-0,01579	0,28	0,294	1
Slc2a3	0,477545	-0,00574	0,029	0,036	1

Dynlt3	0,477556	-0,01886	0,615	0,637	1
Agpat1	0,477641	0,017334	0,329	0,314	1
Orc2	0,477698	0,013251	0,269	0,252	1
Rinl	0,477735	0,03761	0,1	0,091	1
Polb	0,477778	-0,01982	0,596	0,611	1
Gm44702	0,478044	0,00231	0,008	0,005	1
B130006D(	0,478149	-0,00409	0,041	0,049	1
Wrnip1	0,478154	0,020216	0,249	0,236	1
Cebpa	0,478281	0,000696	0,008	0,005	1
Pomk	0,478299	-0,00789	0,093	0,104	1
1700086P(	0,478399	0,003937	0,014	0,01	1
Adck2	0,478436	-0,00986	0,176	0,19	1
Mtmr12	0,47851	-0,01137	0,109	0,12	1
Ttbk1	0,478666	0,003925	0,266	0,288	1
Rxrb	0,478696	-0,01452	0,466	0,489	1
Ankrd35	0,478754	0,001337	0,008	0,005	1
Gm44739	0,478754	0,001337	0,008	0,005	1
Duxbl1	0,478754	0,001337	0,008	0,005	1
Gm45606	0,478754	0,001337	0,008	0,005	1
Sdad1	0,478772	0,023627	0,19	0,177	1
Sipa1	0,47887	0,017105	0,062	0,054	1
Gng5	0,479013	0,037349	0,901	0,898	1
Chmp3	0,479019	0,018264	0,607	0,605	1
Pip5k1a	0,479358	-0,00622	0,275	0,293	1
Elf4	0,479398	0,00524	0,03	0,024	1
Tnfsf11	0,479468	-0,00025	0,008	0,005	1
Ssna1	0,479485	0,025801	0,665	0,657	1
Foxj3	0,479508	0,041654	0,265	0,255	1
Wdr27	0,479511	-0,01265	0,06	0,068	1
Adamtsl3	0,479563	0,018017	0,084	0,075	1
Runx2os1	0,479738	0,010062	0,01	0,007	1
Gm13620	0,479738	0,00813	0,01	0,007	1
9430038I0	0,479773	-0,01152	0,101	0,112	1
B130046B:	0,479814	0,003295	0,014	0,01	1
Akap5	0,479942	-0,00379	0,008	0,005	1
4930469K:	0,480011	0,005548	0,012	0,008	1
Rbx1	0,480067	-0,01853	0,945	0,963	1
Patj	0,480205	0,006837	0,012	0,008	1
Inpp5b	0,480259	-0,00914	0,188	0,203	1
Psenen	0,480367	-0,01506	0,933	0,953	1
Irf2bpl	0,4805	0,032336	0,288	0,276	1
Uchl4	0,48058	0,005224	0,01	0,007	1
Id1	0,480593	-0,00546	0,026	0,033	1
Rbm43	0,480607	-0,00299	0,024	0,029	1
Chac2	0,480702	-0,00055	0,178	0,193	1
4930589L:	0,48078	0,008436	0,012	0,008	1
Spint2	0,480873	0,002328	0,014	0,01	1
Gm11696	0,480967	0,00458	0,012	0,008	1
Pgf	0,480967	0,00458	0,012	0,008	1
F8	0,481002	-0,0156	0,074	0,083	1
Pls3	0,481224	0,021693	0,488	0,476	1
Mxd3	0,481231	0,001381	0,014	0,01	1

Sf3a1	0,48127	-0,00522	0,339	0,358	1
Dstn	0,481408	-0,00664	0,994	0,992	1
BC030343	0,481423	0,005224	0,01	0,007	1
9030612E(	0,481423	0,004254	0,01	0,007	1
Klf12	0,481423	0,004254	0,01	0,007	1
Stradb	0,481468	-0,01713	0,144	0,156	1
Gm29237	0,481636	0,006516	0,01	0,007	1
Insl6	0,481636	0,005548	0,01	0,007	1
Ptpn3	0,481848	0,005871	0,01	0,007	1
Mettl15	0,481884	-0,00935	0,144	0,158	1
Adam32	0,481925	0,00361	0,012	0,008	1
Pik3r3	0,481941	0,026313	0,285	0,309	1
Lactb	0,482049	-0,00653	0,184	0,2	1
Fbxo46	0,482067	0,016998	0,159	0,146	1
Dnajb3	0,482088	-0,00551	0,026	0,033	1
1700015F1	0,482267	0,003283	0,01	0,007	1
Syngap1	0,482267	0,003283	0,01	0,007	1
Brat1	0,482315	0,018743	0,193	0,18	1
Phf23	0,482467	-0,00752	0,285	0,302	1
Cdv3	0,482469	0,013751	0,773	0,759	1
Wdr12	0,482654	0,013525	0,285	0,265	1
Hk2	0,482686	-0,00451	0,026	0,033	1
Myl6	0,482708	0,015373	0,99	0,993	1
Uqcr11	0,48281	-0,00906	0,96	0,976	1
Gm7292	0,482918	-0,00513	0,032	0,039	1
Gm16316	0,483066	-0,0024	0,021	0,026	1
Mettl2	0,483256	0,006936	0,182	0,2	1
Cenpl	0,483305	-0,0016	0,038	0,046	1
Bclaf3	0,483459	0,036534	0,291	0,28	1
Rbm33	0,483528	-0,0064	0,392	0,415	1
Nfkbib	0,483547	-0,02053	0,296	0,311	1
Dym	0,483622	-0,02097	0,234	0,247	1
Kcnb1	0,483658	0,003295	0,012	0,008	1
Ssh1	0,483715	0,002901	0,16	0,176	1
Trpv3	0,483945	-0,00445	0,058	0,067	1
Rnpc3	0,483969	0,04187	0,499	0,501	1
Cops9	0,484022	0,01713	0,915	0,928	1
Selenoo	0,484035	-0,01813	0,314	0,332	1
Rabl2	0,484161	-0,00165	0,108	0,12	1
Rbm3os	0,484231	0,001999	0,012	0,008	1
Slc45a3	0,484249	-0,01116	0,241	0,259	1
Arl8b	0,484256	-0,0174	0,792	0,818	1
Sfmbt1	0,484337	0,015989	0,148	0,137	1
Tarsl2	0,484369	-0,01094	0,319	0,335	1
Cenpi	0,484383	0,001668	0,01	0,007	1
Pm20d2	0,484383	0,001668	0,01	0,007	1
Gm20163	0,484383	0,001668	0,01	0,007	1
Tbc1d8	0,484383	5,69E-05	0,01	0,007	1
Mapkbp1	0,48442	0,000806	0,085	0,075	1
Chd4	0,484456	-0,00301	0,897	0,919	1
Wbp1	0,484702	-0,00615	0,368	0,392	1
Meis2	0,484867	-0,00721	0,232	0,246	1

Prmt6	0,484983	0,010815	0,114	0,102	1
Eml3	0,484989	-0,00062	0,2	0,218	1
Scaper	0,485003	0,042647	0,371	0,363	1
Zbtb17	0,485052	0,016075	0,14	0,128	1
Tbc1d9b	0,485183	-0,0017	0,357	0,379	1
Mtbp	0,485295	-0,01808	0,071	0,08	1
Gm12530	0,485301	0,013876	0,055	0,047	1
2310039H(	0,485343	-0,00495	0,169	0,184	1
Ulk3	0,485708	-0,00168	0,105	0,117	1
Zfhx2os	0,485766	-0,00077	0,067	0,076	1
Aldh2	0,485844	-0,01037	0,273	0,293	1
Polm	0,485937	0,001936	0,073	0,063	1
Selenos	0,486132	-0,00684	0,917	0,935	1
Cadm4	0,486152	0,004041	0,996	0,995	1
Nnt	0,486232	0,007138	0,028	0,023	1
Tmem100	0,486504	5,69E-05	0,01	0,007	1
Nckipsd	0,486556	-0,0091	0,077	0,086	1
Rpain	0,486665	0,027242	0,253	0,241	1
Ccdc97	0,487035	-0,0114	0,207	0,221	1
Hras	0,487175	-0,0267	0,544	0,55	1
E130311K1	0,487293	-0,0089	0,09	0,101	1
Sec14l2	0,487305	-0,00947	0,205	0,221	1
Mlf2	0,4875	-0,01277	0,974	0,982	1
Alas1	0,487933	0,017279	0,227	0,213	1
Ganab	0,488381	0,004747	0,469	0,502	1
C030029H(	0,488524	-0,0383	0,616	0,608	1
Lix1l	0,488684	0,019058	0,099	0,089	1
Ube2g2	0,488703	-0,0166	0,244	0,259	1
Ldlrap1	0,488719	-0,02864	0,386	0,405	1
Ptcd1	0,488828	-0,00636	0,146	0,159	1
Gdi1	0,488878	0,009662	0,919	0,904	1
Cds1	0,489213	0,024472	0,167	0,156	1
Phf8	0,489324	-0,0028	0,274	0,293	1
Saa3	0,489662	0,005554	0,028	0,023	1
Gm12089	0,489662	0,005554	0,028	0,023	1
Supv3l1	0,489815	0,01774	0,094	0,085	1
Stat2	0,489948	0,027226	0,271	0,26	1
Mlf1	0,49003	0,005871	0,028	0,023	1
Fkbp2	0,490144	0,036005	0,818	0,829	1
Ankrd13a	0,490264	0,039136	0,782	0,789	1
Orc3	0,4904	-0,0149	0,178	0,192	1
Mms22l	0,490474	-0,00699	0,011	0,015	1
Rimklb	0,490474	-0,00699	0,011	0,015	1
Jpt1	0,490506	-0,00638	0,093	0,104	1
Gm11273	0,490531	-0,00992	0,043	0,05	1
5730409E(	0,490617	-0,00422	0,175	0,19	1
Secisbp2	0,490661	0,029432	0,364	0,35	1
Ebpl	0,49072	0,037876	0,587	0,582	1
Ldhd	0,490976	0,007018	0,121	0,109	1
Rps20	0,491275	0,02127	0,982	0,977	1
Ggact	0,491332	-0,01394	0,142	0,154	1
Gmeb1	0,491453	-0,01455	0,171	0,184	1

2410015M	0,491562	-0,01454	0,813	0,826	1
Arl4a	0,491868	-0,00072	0,183	0,198	1
Zscan25	0,492086	-0,00602	0,011	0,015	1
Emp1	0,49216	0,047814	0,026	0,021	1
Cd44	0,492322	0,036903	0,095	0,086	1
Snhg12	0,492423	0,028899	0,467	0,47	1
Gm19935	0,492441	-0,00539	0,011	0,015	1
Itpk1	0,492549	0,032411	0,888	0,886	1
Enkd1	0,492581	0,002746	0,035	0,042	1
Prr3	0,492919	-0,01126	0,216	0,231	1
Psrc1	0,492977	0,004923	0,024	0,029	1
Zc3h12a	0,493043	-0,00238	0,059	0,05	1
Mtcl1	0,493236	-0,01165	0,153	0,166	1
Dhx37	0,493347	0,017986	0,16	0,148	1
Donson	0,493439	-0,00436	0,066	0,075	1
Gm26809	0,493877	-0,00602	0,011	0,015	1
Fam199x	0,493896	0,019196	0,256	0,242	1
Gm15965	0,494056	-0,00442	0,011	0,015	1
Brix1	0,494137	-0,00565	0,348	0,369	1
Tmtc3	0,494221	-0,01716	0,154	0,166	1
Smtn	0,494448	0,020874	0,086	0,098	1
Dhx40	0,494522	-0,01073	0,435	0,45	1
Bora	0,494889	-0,00468	0,069	0,078	1
Afg1l	0,494928	-0,00564	0,066	0,075	1
Ppa2	0,495073	0,028092	0,365	0,346	1
Wdr44	0,495111	0,002293	0,218	0,236	1
Trib1	0,495139	0,05031	0,087	0,078	1
Nars	0,495168	-0,00032	0,718	0,751	1
Mdp1	0,495254	-0,00653	0,377	0,4	1
Plpbp	0,495438	0,027249	0,572	0,558	1
Rhog	0,49554	0,019877	0,982	0,995	1
6430550D:	0,49558	0,008411	0,026	0,021	1
Ptprm	0,495622	0,000451	0,196	0,213	1
Kiss1r	0,495673	-0,00345	0,011	0,015	1
Cers1	0,495673	-0,00345	0,011	0,015	1
Gm45353	0,495673	-0,00345	0,011	0,015	1
Meis3	0,495708	0,007459	0,026	0,021	1
Exosc5	0,495765	-0,01512	0,35	0,366	1
Uqcrb	0,496011	-0,0099	0,831	0,844	1
Gm12204	0,49603	-0,00282	0,011	0,015	1
Igip	0,496407	-0,00816	0,385	0,402	1
Txndc5	0,496612	0,006755	0,1	0,089	1
Snapc1	0,496644	0,011919	0,401	0,387	1
Ndufv3	0,496733	-0,00702	0,793	0,785	1
Vav3	0,496757	-0,00307	0,011	0,015	1
Tceal3	0,496816	0,016089	0,171	0,158	1
1700020I1	0,496909	0,033105	0,609	0,602	1
Zfp108	0,496972	0,010599	0,035	0,029	1
R3hdm2	0,497168	-0,01367	0,604	0,61	1
Gnl3l	0,497251	-0,00747	0,54	0,556	1
Ezh1	0,497412	-0,01849	0,504	0,525	1
Dcp1b	0,497511	-0,00951	0,218	0,234	1

Gm13199	0,497651	-0,00185	0,011	0,015	1
Gstt2	0,497651	-0,00185	0,011	0,015	1
Tep1	0,497941	0,010266	0,089	0,101	1
Arl1	0,497975	0,032197	0,744	0,756	1
Pkig	0,498021	-0,00826	0,273	0,291	1
Nop53	0,498369	0,025475	0,731	0,737	1
Tmem267	0,498376	-0,00378	0,102	0,091	1
Trim27	0,498671	0,005054	0,173	0,159	1
Dut	0,498715	0,027998	0,18	0,169	1
Gtf2h2	0,498774	0,008816	0,189	0,176	1
Plpp2	0,499069	0,01252	0,652	0,633	1
Gm12166	0,499326	0,007091	0,069	0,06	1
Cdk2	0,499554	0,004051	0,062	0,054	1
Cep162	0,49958	-0,00344	0,194	0,21	1
Pex16	0,499682	-0,01164	0,307	0,324	1
Gm14325	0,499759	-0,0053	0,043	0,05	1
Gtpbp4	0,499914	-0,03616	0,483	0,462	1
Usp19	0,500215	-0,01541	0,455	0,472	1
Ndufa12	0,500323	-0,00616	0,721	0,753	1
Serpina3n	0,500437	-0,3018	0,659	0,593	1
Spr	0,500704	-0,01466	0,476	0,491	1
Ube2w	0,500815	0,033288	0,361	0,354	1
Fam60a	0,500907	-0,02238	0,075	0,083	1
Tmem38b	0,501132	0,005281	0,452	0,429	1
6030458C1	0,501165	-0,00325	0,178	0,192	1
4930453N1	0,501195	0,000665	0,33	0,353	1
Fgd6	0,501349	0,014269	0,085	0,076	1
Tmed2	0,501471	0,017373	0,951	0,933	1
Mapk1ip1l	0,501518	0,006789	0,294	0,317	1
Trpm7	0,50154	0,037026	0,655	0,654	1
Kbtbd8	0,501647	-0,00381	0,063	0,072	1
Esco1	0,501823	-0,01771	0,268	0,281	1
Rpn2	0,502053	-0,00155	0,764	0,776	1
Hmbs	0,502138	-0,00328	0,249	0,265	1
Ift140	0,50219	-0,00435	0,128	0,14	1
Slc4a8	0,502242	0,025948	0,273	0,26	1
Klhl26	0,502403	-0,00199	0,101	0,112	1
Nfe2l1	0,502526	0,021871	0,553	0,546	1
Zfp53	0,502618	-0,00638	0,103	0,114	1
Rfx5	0,502693	-0,0113	0,051	0,059	1
Zc3hav1	0,502714	-0,04112	0,172	0,182	1
Gm4924	0,502877	-0,01589	0,146	0,158	1
Rc3h2	0,50291	-0,00964	0,605	0,621	1
Sos2	0,503046	-0,0034	0,487	0,517	1
Nudt21	0,50305	0,031058	0,499	0,485	1
Ccar1	0,503323	0,025217	0,602	0,579	1
B630019K0	0,503421	-0,00422	0,112	0,124	1
Zzef1	0,503531	-0,02449	0,357	0,367	1
Ccdc93	0,503595	-0,00647	0,114	0,125	1
Fam117b	0,503648	0,049898	0,354	0,353	1
Coq10b	0,503728	0,036551	0,242	0,231	1
3110062M	0,503737	-0,01654	0,157	0,169	1

Adprh	0,503816	0,01659	0,636	0,634	1
Fam3c	0,503848	-0,002	0,207	0,224	1
Msantd2	0,503914	-0,00251	0,159	0,172	1
Zfp955b	0,503938	-0,01111	0,102	0,112	1
Tor1aip1	0,504028	-0,02479	0,686	0,689	1
Agps	0,504085	-0,0121	0,537	0,556	1
Cabin1	0,50421	-0,02096	0,244	0,259	1
Pkmyt1	0,504212	-0,00339	0,104	0,115	1
Naa16	0,504357	0,031516	0,236	0,226	1
Ldha	0,504584	-0,01618	0,089	0,099	1
Mturn	0,504591	0,033578	0,142	0,132	1
AI480526	0,50462	-0,02604	0,268	0,283	1
Srsf1	0,504916	-0,01753	0,63	0,663	1
Plekhm1	0,504961	-0,00208	0,123	0,135	1
Ubxn6	0,505105	0,02741	0,659	0,663	1
Tom1l2	0,505372	-0,00476	0,606	0,633	1
Arhgef12	0,505408	0,029247	0,765	0,763	1
Srek1ip1	0,505479	-0,00197	0,183	0,197	1
Tfe3	0,505608	-0,0102	0,204	0,22	1
A730017C2	0,505693	-0,0063	0,014	0,018	1
Nudc	0,506015	0,009591	0,866	0,844	1
Ubqln1	0,506042	0,02679	0,768	0,772	1
Kank2	0,506065	-0,02139	0,114	0,124	1
Med27	0,506125	-0,01306	0,203	0,218	1
Klk8	0,506137	-0,0457	0,057	0,049	1
Synpo2	0,506178	-0,00568	0,014	0,018	1
Clmn	0,506228	-0,03045	0,895	0,885	1
Rps18	0,506515	0,006767	0,972	0,969	1
4930522L1	0,506553	0,006162	0,109	0,098	1
Ndufa13	0,506688	0,018693	0,977	0,984	1
Elovl7	0,506961	-0,016	0,962	0,961	1
Aldh1l2	0,506995	-0,04355	0,408	0,415	1
Babam1	0,507013	0,027425	0,442	0,429	1
Tmem132c	0,507242	0,009035	0,025	0,02	1
Gm15050	0,507295	0,00712	0,044	0,037	1
Cdc123	0,507303	0,027404	0,561	0,554	1
Eif6	0,507309	0,023027	0,762	0,759	1
Cspp1	0,507361	0,020173	0,459	0,436	1
Acvr2b	0,507464	-0,03066	0,232	0,241	1
Fam126a	0,507825	-0,00397	0,495	0,515	1
Bcas3	0,507938	-0,00824	0,355	0,374	1
Tmem259	0,507973	0,029157	0,597	0,595	1
Lrpprc	0,508059	0,026166	0,402	0,393	1
Tcea2	0,508838	0,00057	0,093	0,104	1
Upf3b	0,509005	-0,01425	0,607	0,624	1
Laptm4a	0,509347	0,017077	0,873	0,85	1
Taf15	0,509375	-0,02287	0,61	0,626	1
Znhit3	0,509581	0,015509	0,292	0,278	1
Lzts1	0,509664	0,005237	0,025	0,02	1
Tbc1d12	0,509989	-0,006	0,433	0,459	1
Rell1	0,51025	-0,02012	0,443	0,457	1
Gpatch3	0,510291	0,017157	0,065	0,057	1

Klhl2	0,510364	0,022495	0,978	0,979	1
Cdkn1a	0,51044	0,081312	0,142	0,132	1
Frmd6	0,510551	0,002491	0,055	0,047	1
Zmym2	0,510586	0,001301	0,376	0,4	1
Lonp1	0,510651	-0,00349	0,359	0,382	1
Mcfd2	0,510694	-0,02928	0,402	0,408	1
Hspb8	0,510767	-0,01196	0,028	0,034	1
1700123O:	0,510892	-0,00601	0,279	0,296	1
Rad54l2	0,511154	0,007134	0,236	0,221	1
Zfp174	0,511265	-0,0016	0,04	0,047	1
Sntb2	0,511274	0,014673	0,065	0,057	1
Rpl22l1	0,511293	-0,00617	0,759	0,784	1
Filip1	0,511407	0,001402	0,014	0,018	1
Tsfm	0,511567	-0,01875	0,205	0,218	1
Ssb	0,511636	-0,00454	0,816	0,846	1
Phf11c	0,511737	-0,00148	0,014	0,018	1
Pebp1	0,511825	-0,01281	0,98	0,98	1
2010111I0	0,512119	-0,00168	0,551	0,574	1
Timmdc1	0,512459	-0,01678	0,18	0,192	1
Zfand4	0,512609	-0,0118	0,045	0,052	1
Mfsd13b	0,512621	-0,00541	0,034	0,041	1
Invs	0,51264	-0,01574	0,187	0,2	1
Mapkapk5	0,512699	-0,0087	0,127	0,138	1
Cmc2	0,512865	-0,00625	0,18	0,193	1
Ift52	0,512965	-0,01443	0,226	0,239	1
Ptpn13	0,51297	-0,01731	0,028	0,034	1
Tnrc18	0,513019	0,01067	0,311	0,299	1
1600014C:	0,513044	0,012803	0,121	0,111	1
Bcap31	0,513047	0,021717	0,929	0,943	1
Rbm42	0,513064	0,031085	0,75	0,753	1
Tgs1	0,513231	-0,0024	0,454	0,481	1
Rwdd4a	0,513305	0,02422	0,277	0,263	1
Pdcd6ip	0,513345	-0,00255	0,746	0,769	1
Gm11032	0,513374	-0,00181	0,014	0,018	1
Gm26802	0,51351	0,075386	0,049	0,042	1
Mmgt2	0,513523	0,011211	0,033	0,028	1
Tmc7	0,513609	-0,00538	0,321	0,335	1
Sidt2	0,513615	-0,01482	0,418	0,429	1
Cflar	0,513684	-0,0065	0,22	0,237	1
Ssbp4	0,513692	0,007387	0,069	0,06	1
Tmem191c	0,5138	-0,01149	0,07	0,078	1
Slc25a38	0,51389	0,000376	0,245	0,263	1
Ap1g2	0,514131	0,004622	0,044	0,037	1
Wscd1	0,514195	0,023224	0,998	0,998	1
Lin54	0,514277	-0,01375	0,336	0,35	1
Lemd2	0,514278	-0,01038	0,412	0,433	1
Psmb2	0,514325	0,005972	0,859	0,893	1
Saraf	0,514374	0,026316	0,971	0,98	1
Myo19	0,514524	-0,00148	0,014	0,018	1
Prdm15	0,51453	-0,00114	0,095	0,106	1
Shc1	0,514542	-0,0029	0,093	0,104	1
Rars	0,514777	-0,01057	0,217	0,231	1

Smad5	0,515134	-0,01639	0,377	0,392	1
AC130815.	0,515182	0,057472	0,216	0,207	1
Rnf145	0,515204	-0,01814	0,252	0,267	1
Zfp287	0,515324	-0,00456	0,212	0,228	1
Gm9958	0,515432	-0,005	0,017	0,021	1
Dcaf5	0,515471	-0,00823	0,164	0,177	1
Rfc1	0,51549	-0,00517	0,671	0,706	1
Ciart	0,51558	0,014121	0,097	0,088	1
Fancl	0,515613	-0,00638	0,103	0,114	1
Zbtb5	0,515662	-0,00924	0,134	0,146	1
Rel	0,515678	0,023618	0,068	0,06	1
Rab11b	0,515772	-0,01192	0,819	0,847	1
Mrpl9	0,515881	0,033598	0,417	0,41	1
Zfp697	0,515971	0,010334	0,023	0,018	1
Gm16794	0,515977	0,010644	0,023	0,018	1
Rgs20	0,516006	0,00014	0,014	0,018	1
Zfhx2	0,516016	-0,004	0,246	0,263	1
H2afz	0,516024	0,013083	0,852	0,839	1
Atf1	0,51607	0,043827	0,29	0,286	1
Tmem231	0,516229	-0,00696	0,031	0,037	1
Gtf2ird2	0,516304	-0,00487	0,054	0,062	1
Nampt	0,516594	-0,00232	0,6	0,624	1
Mlx	0,516596	-0,00625	0,301	0,322	1
AC163720.	0,516601	-0,01511	0,045	0,052	1
Gaa	0,51661	-0,00777	0,411	0,437	1
Cmip	0,516616	0,019276	0,609	0,613	1
Smc2os	0,516783	-0,00872	0,023	0,028	1
Gm43848	0,517058	-0,00922	0,159	0,172	1
Rps17	0,51709	-0,01053	0,985	0,974	1
Ampd2	0,517126	-0,00904	0,062	0,07	1
Pelp1	0,517138	-0,00582	0,186	0,202	1
Lrrc42	0,517183	-0,00375	0,206	0,223	1
1700030K(	0,51728	0,015123	0,088	0,08	1
Gm20721	0,517389	-0,00403	0,017	0,021	1
Recql4	0,517504	-0,00049	0,025	0,02	1
Man1c1	0,517505	0,00204	0,1	0,089	1
Wdr59	0,517583	-0,01561	0,211	0,224	1
Pla2g12a	0,51765	-0,02058	0,385	0,398	1
Il17b	0,517665	-0,00912	0,028	0,034	1
Anapc5	0,517708	0,031054	0,861	0,837	1
Tmem163	0,517829	-0,00952	0,216	0,229	1
Pramef8	0,517961	-0,01175	0,088	0,098	1
Lynx1	0,517962	0,009362	0,023	0,018	1
Acap3	0,518158	0,021201	0,165	0,154	1
Ipo7	0,518408	-0,00497	0,431	0,459	1
Mrpl55	0,518413	-0,01976	0,421	0,437	1
Eef1e1	0,518673	-0,02559	0,336	0,346	1
Bmp2	0,51877	0,013184	0,058	0,05	1
Eml2	0,518789	-0,01958	0,93	0,919	1
2410002F2	0,519232	0,018333	0,395	0,385	1
Sugp1	0,519297	0,011126	0,292	0,278	1
Zfand5	0,519338	0,030167	0,948	0,948	1

Chd7	0,519362	-0,00162	0,698	0,724	1
Fadd	0,519369	-0,00227	0,133	0,146	1
Nbr1	0,519397	0,029451	0,784	0,784	1
Trim11	0,51964	-0,00957	0,174	0,187	1
Serpine2	0,519923	-0,00557	0,023	0,028	1
Ikzf4	0,519959	-0,00591	0,017	0,021	1
Xpr1	0,520023	0,016184	0,222	0,208	1
Vps37d	0,520179	0,009219	0,072	0,063	1
Enho	0,520272	-0,01745	0,301	0,315	1
Ulk4	0,520389	0,018465	0,056	0,049	1
Fam208b	0,52045	-0,02222	0,314	0,33	1
Pus7l	0,520451	0,011386	0,058	0,05	1
Bckdhb	0,520549	0,014715	0,251	0,237	1
5430403G1	0,520558	-0,00164	0,071	0,08	1
Maff	0,520654	0,007774	0,023	0,018	1
Svip	0,520705	-0,01826	0,689	0,717	1
Mogat1	0,520706	-0,00608	0,028	0,034	1
Ptov1	0,520794	-0,01346	0,85	0,834	1
Ddx51	0,520807	-0,00043	0,077	0,086	1
Thnsl2	0,520808	-0,00734	0,007	0,01	1
Celf1	0,521022	0,032332	0,605	0,587	1
Zfp442	0,521299	-0,0066	0,086	0,096	1
Pmm2	0,521523	-0,0062	0,354	0,374	1
Cbx2	0,521753	-0,0037	0,02	0,024	1
Gm47320	0,521916	-0,00148	0,017	0,021	1
Gba2	0,521983	-0,00512	0,084	0,094	1
AW554918	0,522009	0,006137	0,187	0,174	1
Wfs1	0,522126	0,018886	0,122	0,112	1
Zfp622	0,522419	0,009823	0,268	0,252	1
Itga8	0,522421	-0,00299	0,025	0,02	1
Gm15601	0,522466	-0,00399	0,02	0,024	1
Cwc22	0,522716	-0,01417	0,184	0,197	1
Lamtor1	0,522876	-0,01081	0,84	0,849	1
Nkrf	0,522879	0,01762	0,075	0,067	1
Maz	0,522963	0,009051	0,622	0,595	1
Acbd5	0,523003	-0,00273	0,737	0,782	1
Mfn2	0,523064	-0,02359	0,304	0,314	1
Vps11	0,523106	-0,01236	0,363	0,379	1
Mrpl14	0,5232	-0,00418	0,566	0,592	1
Lrrc8d	0,52322	0,027222	0,562	0,559	1
Gm13373	0,52332	-0,00447	0,007	0,01	1
4930558K0	0,52332	-0,00608	0,007	0,01	1
Mfsd4a	0,523525	-0,00286	0,134	0,146	1
Rsph9	0,523526	0,001626	0,06	0,068	1
Zan	0,523552	-0,00476	0,007	0,01	1
Fignl2	0,523873	-0,00394	0,026	0,031	1
Zmat5	0,523883	0,035174	0,553	0,558	1
Slc39a3	0,523969	-0,00686	0,492	0,515	1
Sephs2	0,524115	0,020905	0,242	0,231	1
Gpalpp1	0,524237	-0,01858	0,223	0,234	1
Rpusd4	0,524311	0,001047	0,076	0,067	1
Map3k3	0,524461	0,018345	0,158	0,146	1

Akr1b10	0,524466	-0,0051	0,007	0,01	1
Rtl6	0,524676	-0,00883	0,118	0,128	1
Lingo3	0,524695	-0,0035	0,007	0,01	1
Zpbp	0,524695	-0,0035	0,007	0,01	1
Cntnap2	0,524695	-0,00413	0,007	0,01	1
Clstn1	0,524702	0,040667	0,739	0,741	1
Ly6g6d	0,524779	0,022743	0,224	0,211	1
Tfpi2	0,524923	-0,00286	0,007	0,01	1
Gm26797	0,524923	-0,00286	0,007	0,01	1
Ccdc40	0,524923	-0,00286	0,007	0,01	1
Prkd1	0,524926	-0,00219	0,007	0,01	1
Irs1	0,52493	0,012733	0,081	0,073	1
Cnot9	0,524935	-0,00035	0,169	0,184	1
Serinc1	0,52517	-0,01705	0,997	1	1
Rhno1	0,525323	-0,00025	0,185	0,2	1
Skiv2l2	0,525325	-0,00327	0,479	0,507	1
Chek2	0,525489	0,022619	0,101	0,093	1
Ap4e1	0,525508	-0,00864	0,157	0,169	1
Nudt11	0,525583	-0,00043	0,114	0,125	1
Etnk2	0,525613	-0,00413	0,007	0,01	1
Senp7	0,525741	-0,01535	0,476	0,489	1
Prps1l3	0,525833	0,009944	0,04	0,034	1
Tpp1	0,525844	0,011698	0,67	0,662	1
Tmem198	0,526072	-0,00252	0,007	0,01	1
Hist1h4a	0,526072	-0,00252	0,007	0,01	1
Slc16a7	0,526079	0,018412	0,359	0,343	1
Dennd1a	0,526092	0,035108	0,382	0,369	1
Atp5d	0,526167	0,021227	0,959	0,959	1
Chmp1b	0,526226	0,011342	0,585	0,572	1
Kif19a	0,5263	-0,00092	0,007	0,01	1
1810062G	0,5263	-0,00189	0,007	0,01	1
Pdzrn3	0,5263	-0,00189	0,007	0,01	1
Gm11149	0,5263	-0,00189	0,007	0,01	1
Mpp5	0,52631	-0,00168	0,841	0,854	1
Ears2	0,526332	-0,01198	0,067	0,075	1
Slc19a1	0,526354	0,010448	0,07	0,062	1
Arfgef3	0,526586	-0,03934	0,26	0,268	1
Spidr	0,526707	-0,00443	0,14	0,153	1
Med14	0,526715	0,008327	0,158	0,174	1
Pbx2	0,526755	-0,00671	0,247	0,265	1
Pdlim3	0,526785	0,008108	0,021	0,016	1
Ap1s1	0,527197	0,002114	0,378	0,398	1
Mcph1	0,527428	-0,00207	0,023	0,028	1
Tstd2	0,527598	-0,01587	0,272	0,285	1
Gm38944	0,527679	-0,00092	0,007	0,01	1
Nrgn	0,527679	-0,00092	0,007	0,01	1
Mlc1	0,527679	-0,00092	0,007	0,01	1
Zfp109	0,527679	5,69E-05	0,007	0,01	1
Taf9	0,52768	0,00039	0,007	0,01	1
Pcsk2	0,527778	0,029128	0,133	0,124	1
Ccdc62	0,527835	-0,01522	0,09	0,099	1
Papolg	0,527883	-0,00908	0,107	0,117	1

Fam204a	0,527897	-0,01337	0,617	0,626	1
Mrpl45	0,527898	-0,01261	0,271	0,285	1
Fbxo33	0,528063	0,007811	0,243	0,259	1
Gm11423	0,528104	-0,00246	0,051	0,059	1
Bmpr2	0,528137	-0,01107	0,58	0,593	1
Xpo7	0,528249	-0,01203	0,395	0,416	1
Rabl3	0,528252	-0,01255	0,163	0,174	1
Tipin	0,528396	-0,02242	0,23	0,239	1
Grk2	0,528397	-0,00471	0,298	0,315	1
Lrrc59	0,528397	-0,01046	0,288	0,301	1
Gfpt2	0,528615	-0,00677	0,003	0,005	1
Pcm1	0,528775	0,024121	0,895	0,872	1
Rttn	0,528803	-0,0082	0,091	0,101	1
Nup107	0,528848	0,009626	0,113	0,102	1
Glg1	0,529034	0,002538	0,796	0,829	1
Cyb5r2	0,529101	-0,00993	0,078	0,086	1
Tubgcp3	0,529174	-0,01172	0,231	0,247	1
Fkbp3	0,52935	-0,01311	0,649	0,681	1
Tnk2	0,529611	0,006879	0,245	0,229	1
Decr1	0,529736	-0,00504	0,252	0,27	1
Zc3h10	0,529746	-0,00667	0,124	0,135	1
Nt5dc2	0,529967	-0,0074	0,003	0,005	1
Heatr1	0,53001	-0,00915	0,086	0,096	1
Trmt10b	0,530033	0,006625	0,244	0,229	1
Use1	0,530182	0,019717	0,629	0,615	1
Pofut1	0,530254	-0,01262	0,202	0,215	1
Tmem170b	0,530295	-0,01134	0,258	0,273	1
Clk4	0,530432	0,020911	0,744	0,75	1
Pex19	0,530479	-0,01582	0,5	0,527	1
Fcnaos	0,530644	-0,00354	0,003	0,005	1
Gm27206	0,530644	-0,00354	0,003	0,005	1
Ncapg	0,530644	-0,00354	0,003	0,005	1
Gm15396	0,530644	-0,00354	0,003	0,005	1
Gm26705	0,530644	-0,00354	0,003	0,005	1
Tacc2	0,530644	-0,00354	0,003	0,005	1
Nefm	0,530644	-0,00354	0,003	0,005	1
Gm4117	0,530644	-0,00354	0,003	0,005	1
Map3k8	0,530644	-0,00354	0,003	0,005	1
Ina	0,530644	-0,00354	0,003	0,005	1
Cfap43	0,530644	-0,00354	0,003	0,005	1
Ptprt	0,530644	-0,00516	0,003	0,005	1
4930444M	0,530644	-0,00516	0,003	0,005	1
Arhgap18	0,530644	-0,00418	0,003	0,005	1
Abhd11	0,530812	0,021023	0,248	0,236	1
Zfp472	0,530837	0,007958	0,09	0,081	1
Sumo1	0,53106	0,029028	0,713	0,725	1
Gli2	0,531321	-0,00418	0,003	0,005	1
Brpf1	0,53138	0,0093	0,201	0,189	1
Miga1	0,531449	0,036477	0,393	0,384	1
Znhit6	0,531532	0,005376	0,271	0,254	1
Plekha6	0,53166	-0,00193	0,003	0,005	1
4930551O	0,53166	-0,00193	0,003	0,005	1

Lamp5	0,53166	-0,00193	0,003	0,005	1
Trib3	0,53166	-0,00193	0,003	0,005	1
Gdpd2	0,53166	-0,00193	0,003	0,005	1
Arxes1	0,53166	-0,00193	0,003	0,005	1
Gm20754	0,53166	-0,00193	0,003	0,005	1
Gm43714	0,53166	-0,00193	0,003	0,005	1
She	0,53166	-0,00193	0,003	0,005	1
Vcam1	0,53166	-0,00193	0,003	0,005	1
Gm21962	0,53166	-0,00193	0,003	0,005	1
Mpl	0,53166	-0,00193	0,003	0,005	1
Gm10062	0,53166	-0,00193	0,003	0,005	1
Hoxa9	0,53166	-0,00193	0,003	0,005	1
Gm15704	0,53166	-0,00193	0,003	0,005	1
Gm44899	0,53166	-0,00193	0,003	0,005	1
Gm15635	0,53166	-0,00193	0,003	0,005	1
Gm44773	0,53166	-0,00193	0,003	0,005	1
Gm47578	0,53166	-0,00193	0,003	0,005	1
Gm32552	0,53166	-0,00193	0,003	0,005	1
Mcf2l	0,53166	-0,00193	0,003	0,005	1
Ank1	0,53166	-0,00193	0,003	0,005	1
Csgalnact1	0,53166	-0,00193	0,003	0,005	1
Elmo3	0,53166	-0,00193	0,003	0,005	1
Cacna1d	0,53166	-0,00193	0,003	0,005	1
AC114585.	0,53166	-0,00193	0,003	0,005	1
4930505A(	0,53166	-0,00193	0,003	0,005	1
Tlx3	0,53166	-0,00193	0,003	0,005	1
4930412M	0,53166	-0,00193	0,003	0,005	1
Abcc3	0,53166	-0,00193	0,003	0,005	1
1700099I0	0,53166	-0,00193	0,003	0,005	1
AC164424.	0,53166	-0,00193	0,003	0,005	1
Mchr1	0,53166	-0,00193	0,003	0,005	1
Hoxc5	0,53166	-0,00193	0,003	0,005	1
2810407A1	0,53166	-0,00193	0,003	0,005	1
Cbr3	0,53166	-0,00193	0,003	0,005	1
Gm20443	0,53166	-0,00193	0,003	0,005	1
Ddx11	0,53166	-0,00193	0,003	0,005	1
Stambpl1	0,53166	-0,00193	0,003	0,005	1
A330032B1	0,53166	-0,00193	0,003	0,005	1
Catip	0,53166	-0,00257	0,003	0,005	1
Dmrta1	0,53166	-0,00257	0,003	0,005	1
Mypopos	0,53166	-0,00257	0,003	0,005	1
Pou6f1	0,531716	-0,01541	0,147	0,158	1
Phtf1	0,531762	0,023839	0,141	0,132	1
Slc23a1	0,531863	0,001445	0,026	0,031	1
Tgfbr3l	0,531999	-0,00159	0,003	0,005	1
Tubgcp6	0,532005	-0,00032	0,087	0,098	1
Nme6	0,532012	0,010573	0,109	0,099	1
Rwdd2b	0,532037	-0,0025	0,045	0,052	1
Med6	0,532092	0,000228	0,213	0,231	1
Stat3	0,532193	0,044868	0,728	0,714	1
Hdhd3	0,532423	0,012392	0,047	0,041	1
Dpf1	0,532435	0,0043	0,028	0,034	1

Mrpl15	0,532585	-0,01326	0,426	0,444	1
Top1mt	0,532676	-0,01487	0,14	0,151	1
Lacc1	0,532677	0,001005	0,003	0,005	1
CT025678.	0,532677	2,86E-05	0,003	0,005	1
A030005L1	0,532677	-0,00095	0,003	0,005	1
Gm26936	0,532677	-0,00095	0,003	0,005	1
F3	0,532677	-0,00095	0,003	0,005	1
Apoc4	0,532677	-0,00095	0,003	0,005	1
Rcvrn	0,532677	-0,00095	0,003	0,005	1
D630044L2	0,532677	-0,00095	0,003	0,005	1
Rell2	0,532677	-0,00095	0,003	0,005	1
Grp	0,532677	-0,00095	0,003	0,005	1
Cfap53	0,532677	-0,00095	0,003	0,005	1
Rnf207	0,532677	-0,00159	0,003	0,005	1
Svil	0,532709	-0,01628	0,098	0,107	1
Wdr43	0,53275	0,033696	0,42	0,411	1
2810021J2	0,532791	-0,00735	0,07	0,078	1
Rngtt	0,533001	0,028185	0,308	0,299	1
Zfp628	0,533013	-0,00978	0,053	0,06	1
Gm42047	0,533017	0,008765	0,003	0,005	1
Zdhhc8	0,533023	0,01472	0,275	0,26	1
Dus2	0,53305	-0,01207	0,248	0,262	1
Arap2	0,53307	-0,04871	0,921	0,88	1
Gale	0,533285	-0,01	0,039	0,046	1
Cog5	0,533385	0,022666	0,322	0,314	1
Hagh	0,533481	0,030037	0,635	0,633	1
Zfp597	0,533557	-0,01332	0,132	0,143	1
Fam76b	0,533739	-0,00738	0,304	0,32	1
Snrpd2	0,533756	0,020377	0,893	0,896	1
Zfp94	0,533855	0,004145	0,115	0,127	1
Wdr19	0,533897	-0,00576	0,102	0,112	1
C1ql3	0,5339	-0,00372	0,137	0,15	1
Lcmt2	0,533904	-0,00903	0,078	0,086	1
Trim68	0,533939	-0,00839	0,067	0,075	1
1110004F1	0,534018	0,010613	0,83	0,831	1
Lpcat1	0,534058	-0,02028	0,134	0,145	1
Snrnp27	0,534714	-0,00762	0,739	0,751	1
Fam184a	0,534719	-0,0078	0,122	0,132	1
Huwe1	0,534787	0,034959	0,818	0,815	1
Pyroxd1	0,534826	-0,00323	0,221	0,236	1
C230037L1	0,535513	-0,0092	0,266	0,283	1
U2surp	0,535638	0,017048	0,809	0,805	1
Neto2	0,535796	0,003955	0,021	0,016	1
5930430L0	0,535863	0,006775	0,057	0,065	1
Sema4b	0,536031	0,00712	0,04	0,034	1
Xpc	0,536044	0,021645	0,153	0,143	1
Trappc5	0,536559	0,005141	0,522	0,506	1
Sall2	0,536624	0,00618	0,049	0,042	1
Mical3	0,536703	0,011696	0,588	0,558	1
AC165953.	0,53677	-0,00275	0,139	0,151	1
Hars2	0,536971	0,007255	0,167	0,154	1
Polr3a	0,536979	-0,0083	0,22	0,233	1

Bbs5	0,536987	0,009517	0,07	0,062	1
Slc34a3	0,537307	0,027569	0,186	0,176	1
Slc39a1	0,537338	0,040021	0,578	0,579	1
Dennd4c	0,537516	0,019152	0,173	0,161	1
Cdk13	0,537644	0,026244	0,437	0,475	1
Cep120	0,537683	-0,01541	0,415	0,431	1
Prkx	0,537745	0,002824	0,218	0,234	1
Zranb1	0,537849	-0,01205	0,588	0,62	1
Tmem160	0,53796	-0,00736	0,393	0,41	1
Trappc2l	0,537969	0,019491	0,646	0,649	1
Zfp462	0,537999	-0,00392	0,43	0,455	1
Wdr45	0,538287	-0,01297	0,354	0,371	1
Ptpn9	0,538454	-0,00772	0,128	0,138	1
Mcm3ap	0,538462	-0,00841	0,208	0,223	1
Pcna	0,538491	0,019797	0,632	0,621	1
Gm12092	0,538823	0,008594	0,068	0,06	1
Zcchc10	0,53924	-0,00015	0,189	0,205	1
Poldip3	0,539311	-0,01599	0,52	0,53	1
Vps37c	0,539342	0,018111	0,285	0,272	1
Coq7	0,53945	-0,00243	0,444	0,468	1
Msl3l2	0,539689	-0,01813	0,255	0,267	1
Kif5c	0,539704	0,031285	0,314	0,307	1
Gm29394	0,540007	0,001567	0,048	0,055	1
Zfp24	0,540038	-0,001	0,636	0,681	1
Znrf3	0,540094	-0,00984	0,184	0,197	1
Tle6	0,540132	-0,01297	0,075	0,083	1
Vma21	0,540201	0,021521	0,655	0,657	1
Gdap1	0,540293	0,00503	0,109	0,12	1
Xkr4	0,54035	0,019079	0,135	0,125	1
Polg2	0,540504	0,017816	0,129	0,119	1
Mis18a	0,540577	-0,00409	0,042	0,049	1
Mrm1	0,540698	0,013273	0,093	0,085	1
Socs2	0,540818	0,01339	0,029	0,024	1
Hist1h2be	0,54086	-0,01082	0,148	0,159	1
Ddx52	0,540869	-0,00792	0,303	0,319	1
Dap	0,540928	-0,00929	0,082	0,091	1
Rbbp9	0,541058	0,021871	0,393	0,38	1
Slc30a2	0,541081	0,00715	0,019	0,015	1
Shkbp1	0,541174	-0,00483	0,237	0,254	1
Gpsm2	0,541221	-0,01748	0,283	0,296	1
Urgcp	0,54125	0,012845	0,191	0,179	1
Fuca1	0,541283	0,004702	0,782	0,834	1
N4bp2l2	0,541389	0,031777	0,631	0,639	1
B3gat3	0,541486	0,021513	0,538	0,525	1
Cdh2	0,541551	0,028823	0,767	0,78	1
Apba3	0,541564	0,002047	0,221	0,237	1
Nav2	0,541771	0,015892	0,245	0,233	1
0610040B1	0,541783	0,014363	0,169	0,158	1
Dnajc25	0,542268	0,016477	0,117	0,107	1
Pradc1	0,542809	-0,00346	0,242	0,259	1
Tm9sf1	0,542872	-0,00471	0,091	0,101	1
Ankrd52	0,542991	0,025425	0,154	0,145	1

Ing5	0,543048	-0,00679	0,198	0,211	1
Arc	0,543118	0,026821	0,36	0,385	1
Eif2b4	0,543232	-0,00623	0,369	0,387	1
Thrsp	0,543422	0,052515	0,154	0,146	1
Ap3b1	0,543443	0,0308	0,375	0,366	1
Msh3	0,543627	0,010558	0,324	0,341	1
Hnrnpll	0,54372	0,025869	0,348	0,34	1
Aar2	0,543884	-0,01533	0,299	0,314	1
Gm10501	0,54395	0,005231	0,019	0,015	1
Gm27029	0,543954	0,006188	0,029	0,024	1
Txlng	0,544042	-0,0129	0,209	0,221	1
Pnpla7	0,544043	-0,01046	0,125	0,135	1
Sort1	0,544109	-0,01435	0,987	0,992	1
Slc38a10	0,544121	0,022386	0,568	0,559	1
Thg1l	0,544186	-0,00422	0,083	0,093	1
Kyat1	0,544219	-0,00255	0,073	0,081	1
Kxd1	0,544347	-0,00133	0,453	0,481	1
Bnip3l	0,544386	-0,00101	1	1	1
Nkain1	0,544408	0,013179	0,644	0,637	1
Cul4b	0,544739	0,011811	0,135	0,125	1
Snx8	0,544843	-0,01404	0,298	0,312	1
Tsr2	0,544955	0,022817	0,339	0,33	1
Lgals9	0,545253	-0,00636	0,01	0,013	1
Rbm15	0,545257	0,006088	0,355	0,374	1
Lmf1	0,5453	0,005467	0,422	0,454	1
Casp2	0,545317	-0,02083	0,128	0,137	1
Dgkq	0,545589	-0,00698	0,154	0,166	1
Eif1b	0,545605	-0,00443	0,847	0,875	1
Fbxw11	0,545992	0,002417	0,436	0,468	1
Rce1	0,546413	-0,00443	0,287	0,304	1
Stx12	0,546508	0,015011	0,77	0,743	1
Rbmx2	0,546643	-0,01212	0,228	0,241	1
Gm9972	0,546666	0,005231	0,019	0,015	1
Ogfod3	0,546846	0,006649	0,203	0,221	1
Fhl1	0,546846	-0,03531	0,443	0,449	1
Psmc3ip	0,546945	0,011211	0,036	0,031	1
Zfp560	0,547006	-0,00622	0,274	0,291	1
Cdh20	0,547034	-0,00265	0,403	0,429	1
Hspb11	0,547136	0,029128	0,349	0,341	1
Rnf111	0,547164	-0,03793	0,341	0,346	1
Zfp383	0,547241	0,01604	0,165	0,154	1
Pate2	0,547243	-0,00476	0,01	0,013	1
Gm26964	0,547243	-0,00636	0,01	0,013	1
1700069B(	0,547243	-0,00796	0,01	0,013	1
5730455P:	0,547446	-0,01677	0,458	0,478	1
Aff3	0,54784	0,019372	0,143	0,133	1
Zfp740	0,547908	-0,01769	0,595	0,597	1
Rfxank	0,548263	-0,00835	0,193	0,207	1
Lima1	0,548286	0,028413	0,2	0,19	1
Akr1c13	0,548294	0,004927	0,038	0,033	1
Manf	0,548353	-0,02104	0,623	0,636	1
Dph2	0,548364	0,01088	0,1	0,091	1

Lin7b	0,548442	-0,00442	0,01	0,013	1
Lrrc14b	0,548639	-0,00539	0,01	0,013	1
Rgma	0,548808	-0,0098	0,628	0,644	1
Morn2	0,548838	-0,0263	0,188	0,197	1
Tkt	0,549034	-0,00765	0,994	0,993	1
Prmt1	0,549235	0,021441	0,7	0,686	1
Txnl4a	0,549238	-0,00624	0,52	0,535	1
Son	0,549416	0,014516	0,996	1	1
Neb	0,549559	0,003955	0,019	0,015	1
Madd	0,549639	-0,00646	0,05	0,057	1
Mtfr1l	0,549788	-0,02768	0,557	0,556	1
Abcf3	0,549821	-0,00286	0,371	0,387	1
Pdcl3	0,549851	0,019266	0,352	0,341	1
Med4	0,550083	-0,00522	0,408	0,428	1
Cbwd1	0,550092	0,012571	0,152	0,141	1
2610037D(	0,550423	-0,01082	0,158	0,169	1
Dolpp1	0,550643	-0,01187	0,149	0,159	1
Cdon	0,5507	0,002371	0,103	0,114	1
C8g	0,550835	0,001689	0,01	0,013	1
Fcer1g	0,550835	0,000721	0,01	0,013	1
Nop16	0,550952	-0,00619	0,225	0,239	1
Pwp1	0,551122	0,014246	0,217	0,205	1
Csrnp3	0,551269	-0,00748	0,224	0,237	1
Nphp3	0,551568	0,011526	0,086	0,078	1
Snx29	0,551574	-0,00685	0,236	0,249	1
Naa15	0,551781	0,02871	0,598	0,595	1
Taco1	0,551812	0,009568	0,059	0,052	1
Ift57	0,551868	0,001739	0,219	0,236	1
Zc3h11a	0,551901	-0,00065	0,083	0,093	1
Ankrd13d	0,55195	0,005871	0,045	0,039	1
Fn1	0,55206	-2E-05	0,042	0,049	1
Gm26981	0,552486	0,010905	0,029	0,024	1
Gm49164	0,552487	0,008436	0,017	0,013	1
Dnase1l2	0,552487	0,008436	0,017	0,013	1
Kif1bp	0,552835	0,002606	0,892	0,902	1
Abl1	0,553082	-0,00442	0,628	0,646	1
March7	0,553088	-0,01582	0,538	0,533	1
Snrnp200	0,553228	-0,00775	0,438	0,454	1
Csf2ra	0,553291	0,01677	0,172	0,161	1
Rsrc1	0,55344	-0,00074	0,729	0,751	1
5330434G(	0,553485	-0,00454	0,368	0,387	1
Plrg1	0,553671	0,012592	0,327	0,312	1
Inf2	0,553792	0,041737	0,968	0,969	1
Vkorc1	0,553851	0,003043	0,627	0,592	1
Acox3	0,554156	0,03135	0,215	0,205	1
Arl2bp	0,554159	-0,01681	0,85	0,859	1
Pafah1b1	0,554303	-0,00542	0,998	1	1
Nus1	0,554411	0,029954	0,435	0,433	1
Sept1	0,554412	-0,00077	0,378	0,397	1
Rap1gap	0,554413	0,00556	0,036	0,031	1
Acbd4	0,554573	0,008934	0,061	0,054	1
Ube2m	0,554699	0,022454	0,754	0,777	1

Mrpl42	0,5547	-0,02134	0,575	0,58	1
Dtna	0,554743	0,038252	0,712	0,722	1
Ppm1h	0,554749	-0,00315	0,157	0,169	1
Tcof1	0,554922	0,019969	0,272	0,26	1
Sssca1	0,555005	-0,0023	0,453	0,475	1
Rplp1	0,555156	0,005324	0,999	0,998	1
Hey1	0,555211	0,00555	0,017	0,013	1
Prc1	0,555218	0,011932	0,017	0,013	1
Bsg	0,55533	0,000233	0,954	0,948	1
Zfp940	0,5556	0,003102	0,054	0,047	1
Ak6	0,555946	0,026783	0,249	0,239	1
Fgfr3	0,556054	-0,0063	0,013	0,016	1
Gfra1	0,556058	0,025673	0,15	0,141	1
Zkscan14	0,55618	0,014803	0,12	0,111	1
Tmem115	0,556192	-0,01812	0,385	0,397	1
Naa30	0,55639	-0,00718	0,303	0,322	1
Ccdc163	0,556605	-0,02082	0,256	0,273	1
Pnn	0,556678	0,023908	0,84	0,831	1
B4galt3	0,556731	0,035465	0,293	0,286	1
Gm16099	0,556752	0,003653	0,027	0,023	1
Retreg3	0,557323	0,011647	0,672	0,646	1
Pole2	0,557329	-0,00702	0,027	0,033	1
Mrfap1	0,557745	0,011421	0,995	0,997	1
D130020L(	0,557935	-0,00527	0,072	0,08	1
Spock2	0,557941	0,003621	0,017	0,013	1
Rsph3b	0,557941	0,003621	0,017	0,013	1
Aldh3b2	0,557941	0,003621	0,017	0,013	1
E330034G:	0,557946	0,004909	0,017	0,013	1
Rap1gds1	0,558034	-0,01422	0,815	0,81	1
Ddx31	0,558058	-0,00558	0,047	0,054	1
Sh3gl3	0,55808	-0,01133	0,456	0,481	1
Gm15445	0,558082	0,011489	0,043	0,037	1
Rsbn1	0,558102	0,031658	0,419	0,411	1
Cep70	0,558111	0,01477	0,153	0,143	1
Vps53	0,558186	-0,00865	0,435	0,454	1
Gca	0,558344	-0,01377	0,342	0,356	1
Rbbp7	0,558387	0,037719	0,547	0,545	1
Tmem70	0,558447	0,011479	0,283	0,27	1
Bcl2	0,558459	0,000746	0,017	0,013	1
Gm26541	0,558474	0,002701	0,027	0,023	1
Dhx57	0,558539	0,021898	0,146	0,137	1
Rala	0,558695	0,024701	0,815	0,844	1
Flot2	0,558697	0,017334	0,298	0,285	1
Mafb	0,558714	-0,00221	0,027	0,033	1
Slc12a8	0,558751	-0,0049	0,025	0,029	1
Impad1	0,558868	-0,00572	0,733	0,754	1
Nol10	0,559014	-0,00783	0,101	0,111	1
Commd4	0,559086	0,021545	0,609	0,598	1
B4galt7	0,55917	-0,00877	0,117	0,127	1
Gm28557	0,559313	0,003308	0,017	0,013	1
Zfp575	0,559492	0,004913	0,017	0,013	1
Hcfc2	0,559505	-0,01593	0,234	0,247	1

Rbms1	0,55961	-0,00955	0,289	0,306	1
Zwint	0,559799	0,036145	0,413	0,421	1
Cox4i1	0,560003	-0,00746	0,999	1	1
Prr14	0,560363	0,028094	0,252	0,241	1
Zfp950	0,560432	-0,01074	0,422	0,442	1
Zbtb2	0,560452	-0,01164	0,134	0,145	1
1110059E2	0,56055	0,024106	0,293	0,285	1
Tsen54	0,560554	-0,00852	0,077	0,085	1
Catspere2	0,560684	-0,00345	0,013	0,016	1
Thumpd3	0,560771	-0,02057	0,251	0,262	1
Pot1a	0,560878	-0,01908	0,255	0,268	1
Pcdhga12	0,560908	-0,00587	0,182	0,195	1
Dnal4	0,561136	0,013839	0,276	0,263	1
Mib2	0,5612	0,025747	0,263	0,255	1
4831440D1	0,561227	-0,00437	0,013	0,016	1
Ubald1	0,561314	-0,01505	0,492	0,512	1
Socs6	0,561642	0,044865	0,288	0,283	1
Sf3a2	0,561693	0,030537	0,379	0,371	1
Glb1l	0,561784	-0,00709	0,025	0,029	1
Gm42067	0,562118	-0,00215	0,013	0,016	1
Fam193b	0,562277	0,004215	0,366	0,384	1
Ube2d1	0,56244	0,022647	0,931	0,927	1
Lyst	0,562489	0,029343	0,249	0,239	1
Zbtb11os1	0,562563	0,024983	0,196	0,215	1
Abca1	0,562655	-0,01321	0,31	0,328	1
Tmx3	0,562696	0,007287	0,653	0,685	1
Plcg2	0,562958	-0,00534	0,016	0,02	1
Zfp458	0,562985	0,019959	0,168	0,158	1
Srpk1	0,562996	0,002916	0,368	0,39	1
Ranbp17	0,563065	-0,00625	0,019	0,023	1
S100pbp	0,563245	0,011072	0,213	0,202	1
Tmem55b	0,563291	-0,00809	0,737	0,732	1
Znhit2	0,56339	0,013077	0,162	0,151	1
Mxd1	0,563419	-0,02322	0,268	0,28	1
Ubap2l	0,563437	0,038932	0,691	0,688	1
Sptbn1	0,563535	-0,00025	0,959	0,964	1
Smad3	0,563718	0,011355	0,111	0,102	1
Tmem240	0,563956	0,017181	0,034	0,029	1
Myd88	0,564014	0,020034	0,074	0,067	1
Npr1	0,564025	0,000866	0,033	0,039	1
Sobp	0,564294	0,006441	0,116	0,106	1
Rcc1l	0,564301	-0,00852	0,153	0,164	1
Tgfbr3	0,564333	0,022265	0,167	0,158	1
Mrpl54	0,564404	-0,01879	0,454	0,467	1
Asphd2	0,564523	0,017919	0,121	0,112	1
Gm9803	0,564536	0,008671	0,05	0,044	1
Actr10	0,564718	0,011884	0,729	0,722	1
Amt	0,564794	0,003189	0,078	0,086	1
Bcl10	0,564821	-0,01772	0,406	0,423	1
Gm5464	0,564915	-0,00115	0,016	0,02	1
Hat1	0,565026	0,005379	0,276	0,26	1
Abcb7	0,565124	-0,00515	0,174	0,185	1

Fam208a	0,565242	-0,02229	0,71	0,712	1
Tmsb15b2	0,565559	-0,00374	0,016	0,02	1
Fbxw15	0,565559	-0,00374	0,016	0,02	1
Stxbp5	0,565559	-0,00714	0,181	0,193	1
Gstm4	0,565574	0,015406	0,092	0,085	1
Abcb1b	0,565635	0,010953	0,026	0,021	1
Usp2	0,565667	-0,03077	0,288	0,294	1
Tmem51	0,565754	0,004329	0,052	0,046	1
Zfp454	0,566099	0,011542	0,034	0,029	1
Ctxn3	0,566132	-0,00203	0,025	0,029	1
Zfp982	0,566219	-0,00403	0,016	0,02	1
Polr2b	0,566277	0,040771	0,366	0,361	1
Prdm2	0,566673	0,007354	0,358	0,39	1
Trir	0,566681	-0,01516	0,758	0,782	1
Pgs1	0,566699	0,0113	0,306	0,291	1
Rbbp4	0,566766	-0,01461	0,863	0,88	1
Rheb	0,566827	-0,00149	0,605	0,634	1
Ptpn18	0,566994	0,006192	0,015	0,011	1
Gm16907	0,567123	-0,00495	0,019	0,023	1
Tcp11l2	0,567348	-0,00071	0,295	0,312	1
Gm10010	0,567516	-0,00278	0,016	0,02	1
Ccdc122	0,567524	-0,00466	0,016	0,02	1
Pin1	0,568004	-0,00602	0,613	0,633	1
Prpf6	0,568127	0,007903	0,329	0,353	1
Pak3	0,568211	-0,00813	0,281	0,294	1
Lbhd1	0,568278	0,005228	0,015	0,011	1
Mrps23	0,568355	0,018086	0,371	0,359	1
Dcxr	0,56844	-0,01657	0,201	0,211	1
Ctnna1	0,568652	-0,00292	0,824	0,831	1
2310074N	0,568771	-0,00211	0,019	0,023	1
Polr1e	0,568779	-0,00013	0,025	0,029	1
Olig2	0,568854	0,007473	0,763	0,779	1
1810010H	0,56893	-0,00108	0,027	0,033	1
Kat5	0,569107	0,008776	0,28	0,301	1
Lrp6	0,569207	-0,00564	0,56	0,576	1
Gm1976	0,569477	0,000113	0,016	0,02	1
Gm49086	0,569564	0,006192	0,015	0,011	1
Cdc5l	0,569678	0,014026	0,625	0,597	1
Pcnp	0,569697	0,024182	0,961	0,951	1
Ccdc159	0,56977	-0,00371	0,044	0,05	1
Tbl1xr1	0,569903	0,009849	0,511	0,543	1
Insm1	0,569973	-0,00075	0,025	0,029	1
Mrps16	0,569984	0,03516	0,493	0,491	1
Nfkb2	0,570104	0,008948	0,057	0,05	1
Vps29	0,570208	0,000202	0,798	0,823	1
2310034G	0,570285	0,001098	0,019	0,023	1
Gm15879	0,570304	-0,00976	0,015	0,011	1
Polr2e	0,570306	0,026866	0,647	0,655	1
Thoc1	0,570377	-0,00741	0,226	0,239	1
Bnip3	0,570539	-0,00549	0,5	0,525	1
BC025920	0,57067	0,011463	0,048	0,042	1
Phf7	0,570742	0,007721	0,057	0,05	1

Gm15420	0,570851	0,003295	0,015	0,011	1
Tmem185a	0,570868	-0,01209	0,128	0,137	1
Bmp1	0,570904	0,01367	0,262	0,249	1
Ndufab1	0,570909	-0,01206	0,836	0,841	1
1700008O(	0,57104	0,003621	0,015	0,011	1
Bfsp1	0,571083	0,008671	0,043	0,037	1
Aplf	0,571103	-0,00096	0,085	0,094	1
Rap1b	0,571113	0,01694	0,568	0,554	1
Ehd1	0,571188	-0,01599	0,667	0,691	1
Eif3b	0,571492	-0,00567	0,581	0,598	1
Ybx1	0,571879	-0,00573	0,983	0,987	1
Actr3	0,572004	0,021744	0,748	0,758	1
Zfp965	0,572144	0,003621	0,015	0,011	1
Acot1	0,572192	0,01938	0,575	0,592	1
Klc4	0,572255	-0,00813	0,079	0,086	1
Zfp369	0,572353	0,002202	0,133	0,145	1
Usp47	0,572624	-0,00865	0,417	0,436	1
Polg	0,572635	0,013074	0,188	0,177	1
Tmem136	0,573047	-0,00551	0,022	0,026	1
B4galt2	0,573222	0,004667	0,078	0,07	1
Slc25a19	0,573346	0,030535	0,34	0,333	1
Atp5c1	0,573753	0,0216	0,939	0,94	1
Hist3h2a	0,573786	0,000594	0,362	0,384	1
Ilvbl	0,573798	0,013647	0,681	0,668	1
Cacng5	0,573874	-0,01205	0,035	0,041	1
Atxn10	0,573927	-0,01142	0,787	0,785	1
Tmem29	0,574271	0,014461	0,211	0,2	1
Nap1l1	0,574286	0,041269	0,728	0,711	1
Usp13	0,574377	0,005871	0,034	0,029	1
Mark4	0,574494	-0,00355	0,201	0,215	1
Ccdc18	0,574519	0,00014	0,019	0,023	1
Por	0,574897	0,023549	0,445	0,429	1
Gm26766	0,575099	0,001052	0,015	0,011	1
Glrx3	0,57512	0,019731	0,71	0,701	1
A730011C:	0,575121	0,001729	0,019	0,023	1
5033421B(	0,575771	-0,00386	0,052	0,059	1
Pop1	0,575819	0,008253	0,076	0,085	1
Stn1	0,575847	0,009879	0,187	0,176	1
CT025619.	0,576224	-0,01795	0,23	0,241	1
Znrf2	0,576318	0,000213	0,122	0,132	1
Gpt	0,576378	0,040379	0,524	0,527	1
Ap4s1	0,57638	-0,01442	0,367	0,382	1
Plaa	0,576384	0,005398	0,287	0,302	1
AC151836.	0,576392	8,49E-05	0,015	0,011	1
Cmss1	0,576414	0,012507	0,206	0,195	1
Stx18	0,576448	-0,00438	0,351	0,369	1
Slc35b2	0,576548	-0,00852	0,367	0,387	1
Olfml2b	0,576611	-0,01686	0,221	0,231	1
Kif3b	0,576829	-0,00451	0,145	0,156	1
Mpped2	0,577066	-0,00737	0,081	0,089	1
Zeb2os	0,577158	0,002146	0,239	0,254	1
Yes1	0,577209	0,004263	0,167	0,156	1

Pex6	0,577252	0,021915	0,345	0,337	1
Mtmr7	0,577272	0,010291	0,032	0,028	1
Il6st	0,577279	0,032846	0,541	0,524	1
Tpr	0,577369	0,041964	0,87	0,873	1
Dnajb12	0,577558	-0,00878	0,552	0,576	1
Dync2li1	0,577584	-0,00761	0,136	0,146	1
Wdr4	0,5776	-0,00941	0,094	0,102	1
Cyfip2	0,577659	-0,02641	0,308	0,32	1
Mettl26	0,577677	0,004658	0,417	0,447	1
Gm17655	0,577751	-0,00063	0,044	0,05	1
Zfp580	0,57786	-0,00389	0,26	0,275	1
Gla	0,57789	-0,00082	0,138	0,15	1
Hnrnpa2b1	0,577976	-0,01649	0,998	1	1
Furin	0,578141	-0,01051	0,226	0,237	1
Plod3	0,578198	-0,00621	0,436	0,452	1
Ltbp3	0,578633	-0,01138	0,046	0,052	1
Tmem106c	0,578796	-0,00244	0,406	0,429	1
Itch	0,578969	-0,00942	0,912	0,906	1
Nacad	0,578999	-0,00974	0,582	0,597	1
Chmp5	0,579061	0,018143	0,87	0,87	1
Ipmk	0,57924	0,032879	0,239	0,231	1
Farp2	0,579252	0,001494	0,104	0,114	1
Fastk	0,579356	0,01456	0,296	0,285	1
Tsr1	0,57937	-0,01076	0,083	0,091	1
Nlgn2	0,579468	0,011032	0,199	0,189	1
Lipt2	0,579763	-0,02054	0,112	0,12	1
Phactr4	0,580003	-0,00067	0,522	0,545	1
Gm45447	0,5802	0,006837	0,013	0,01	1
2210008F(	0,5802	0,006837	0,013	0,01	1
Sned1	0,580232	0,036766	0,286	0,276	1
Grn	0,580415	0,022296	0,495	0,493	1
Rita1	0,580463	0,018843	0,1	0,093	1
Scaf8	0,580508	0,003227	0,341	0,358	1
E430024I0	0,580581	0,011386	0,062	0,055	1
Sclt1	0,580912	-0,00126	0,213	0,228	1
Mrpl3	0,580932	-0,02046	0,341	0,351	1
Jkamp	0,580932	0,025107	0,625	0,633	1
Tspyl2	0,580978	0,01913	0,372	0,361	1
Metrnl	0,581001	0,010355	0,013	0,01	1
Mettl5	0,581065	-0,00286	0,246	0,262	1
Zfp692	0,581231	0,024889	0,197	0,189	1
Ero1l	0,58127	0,014538	0,365	0,348	1
Fam173a	0,581379	0,025807	0,663	0,663	1
1110004E(	0,581498	0,023455	0,293	0,286	1
Pnmal2	0,58156	0,000714	0,058	0,065	1
Snx7	0,581608	0,00818	0,12	0,111	1
Rpl7a	0,5817	0,019641	0,972	0,976	1
Ppia	0,581882	0,011552	1	1	1
Ubr3	0,581984	-0,01791	0,596	0,616	1
Wrb	0,582025	-0,01854	0,294	0,304	1
Aaas	0,582039	0,01305	0,128	0,119	1
Zfyve9	0,582087	0,007482	0,405	0,429	1

Tomm6	0,582189	0,027262	0,47	0,462	1
Fnbp1	0,582269	-0,0203	0,997	0,998	1
Rock1	0,582307	-0,00768	0,847	0,86	1
Garem1	0,582327	-0,01013	0,15	0,161	1
Ttc7	0,582332	-0,00362	0,113	0,122	1
Nhlrc4	0,582396	-0,00886	0,029	0,034	1
Mss51	0,582399	0,002017	0,013	0,01	1
Cog4	0,582407	-0,00892	0,41	0,424	1
Tmx4	0,582536	-0,01827	0,516	0,522	1
Nlrc3	0,582595	0,004904	0,013	0,01	1
4932435O:	0,582595	0,003937	0,013	0,01	1
Ociad2	0,5826	0,006513	0,013	0,01	1
Mtif3	0,582777	-0,00303	0,2	0,213	1
N4bp1	0,583027	-0,01725	0,301	0,314	1
Naaladl2	0,583172	-0,01787	0,139	0,148	1
Zfp105	0,583396	-0,01265	0,046	0,052	1
Rims2	0,583418	-0,00951	0,046	0,052	1
Ipo5	0,583421	0,036079	0,591	0,593	1
1700020L2	0,583488	-0,01165	0,065	0,072	1
Chka	0,583816	0,145801	0,518	0,512	1
Scube3	0,583872	-0,01154	0,096	0,104	1
Aldh3a2	0,583931	-0,00678	0,421	0,444	1
Hddc2	0,584397	0,001689	0,013	0,01	1
Zfp384	0,584463	-0,00892	0,183	0,193	1
Nipsnap1	0,5847	0,003922	0,15	0,14	1
Ift46	0,58475	0,01916	0,353	0,345	1
Tmem167	0,584992	-0,00774	0,628	0,641	1
Sergef	0,585025	-0,00641	0,149	0,159	1
Olfr1322	0,585198	0,002328	0,013	0,01	1
Zfp738	0,585213	-0,00332	0,235	0,249	1
Vps4b	0,585258	0,024916	0,567	0,563	1
Nkap	0,585346	0,00291	0,547	0,558	1
Lcmt1	0,585386	-0,01667	0,392	0,402	1
Rnf219	0,585465	-0,02078	0,092	0,099	1
Gm42893	0,585474	0,007765	0,032	0,028	1
Tenm4	0,585515	-0,03349	0,142	0,15	1
Meaf6	0,585596	0,020661	0,483	0,472	1
Hist4h4	0,585618	0,012754	0,032	0,028	1
Sdhc	0,585652	-0,01298	0,664	0,673	1
Irak4	0,586042	-0,01304	0,104	0,112	1
Uprt	0,586201	0,003621	0,013	0,01	1
Fam114a1	0,586331	-0,00799	0,049	0,055	1
Klk10	0,586399	0,001359	0,013	0,01	1
Gm9493	0,586601	0,005871	0,024	0,02	1
AC123870.	0,586607	0,006189	0,024	0,02	1
B4galnt1	0,586748	0,018236	0,051	0,046	1
Unk	0,58677	0,035965	0,245	0,239	1
Mthfd1l	0,587037	-0,00717	0,083	0,091	1
Gm49130	0,587041	-0,00764	0,032	0,037	1
Coro7	0,587303	-0,01151	0,29	0,304	1
Prmt2	0,587309	0,026423	0,501	0,501	1
Lgals8	0,587437	-0,00638	0,441	0,452	1

Rnf138	0,587508	0,011373	0,336	0,325	1
Mocs2	0,587669	0,016084	0,52	0,498	1
Epb41l1	0,587802	0,029311	0,501	0,489	1
Bace2	0,587806	-0,00639	0,143	0,154	1
Man1a2	0,587964	-0,01727	0,48	0,489	1
Soat1	0,588046	0,004619	0,219	0,234	1
Slc52a2	0,588071	-0,01955	0,153	0,163	1
Nceh1	0,588238	0,038562	0,6	0,595	1
Dnaaf5	0,588248	-0,01155	0,143	0,153	1
D830035M	0,588264	-0,00608	0,006	0,008	1
Acsl4	0,588327	0,026609	0,198	0,189	1
Raf1	0,58836	0,012075	0,59	0,564	1
Polr1c	0,588404	0,010706	0,245	0,234	1
Entpd6	0,58852	-0,01008	0,073	0,08	1
A730071L1	0,588527	-0,00545	0,006	0,008	1
Tsen15	0,588543	0,011616	0,167	0,156	1
Mrps30	0,588668	-0,00362	0,206	0,22	1
Parp6	0,588717	0,00668	0,165	0,177	1
Hadh	0,588843	0,015682	0,782	0,771	1
2010015M	0,588856	0,007444	0,032	0,028	1
Srrt	0,588862	-0,01095	0,424	0,439	1
Arpc3	0,588886	0,02815	0,727	0,751	1
Snapc4	0,588928	0,005871	0,082	0,075	1
Wdr73	0,588934	-0,00188	0,116	0,125	1
Leng9	0,589114	0,01585	0,128	0,119	1
Hnrnph3	0,58914	0,015736	0,688	0,675	1
Tmf1	0,589222	0,00718	0,76	0,784	1
Rnf144b	0,589581	-0,00608	0,006	0,008	1
Snf8	0,589609	0,028257	0,619	0,626	1
Bod1	0,589656	-0,01002	0,47	0,491	1
2410131K1	0,589752	0,005302	0,129	0,14	1
Cplx2	0,589845	-0,00447	0,006	0,008	1
Tmem123	0,589893	0,010885	0,522	0,512	1
Slc25a25	0,590028	0,007086	0,069	0,062	1
1700030J2	0,590107	-0,00384	0,006	0,008	1
Nudt5	0,590325	-0,00231	0,33	0,346	1
Smarcd2	0,590361	-0,0118	0,173	0,182	1
Ptbp2	0,590558	0,019838	0,645	0,637	1
Reep3	0,590581	0,024064	0,991	0,997	1
Afg3l1	0,590636	0,025389	0,477	0,47	1
Atp5a1	0,590874	0,021957	0,983	0,985	1
Btbd16	0,590885	-0,00968	0,059	0,052	1
Sema4f	0,5909	-0,01239	0,006	0,008	1
Dnlz	0,590989	-0,01431	0,509	0,522	1
Sbf2	0,59113	-0,00917	0,532	0,537	1
Kdm4d	0,591427	-0,00286	0,006	0,008	1
BC051142	0,591455	0,004918	0,024	0,02	1
Mfsd3	0,59148	0,01103	0,125	0,115	1
Pdk3	0,591509	0,013172	0,346	0,367	1
Pcdhb19	0,591602	0,018293	0,065	0,059	1
Pcsk7	0,591673	0,007385	0,225	0,241	1
Anks1b	0,591678	0,02269	0,925	0,92	1

Gria3	0,59169	-0,00223	0,006	0,008	1
Vmn1r4	0,59169	-0,00223	0,006	0,008	1
Hexa	0,591726	0,023835	0,637	0,634	1
Nin	0,591872	0,002202	0,119	0,128	1
Rccd1	0,591934	0,002136	0,172	0,185	1
Pam	0,592323	-0,01792	0,548	0,556	1
2700033N	0,59247	0,012006	0,058	0,052	1
Luzp1	0,592476	0,015123	0,083	0,076	1
1700021N	0,592484	-0,00252	0,006	0,008	1
Brpf3	0,592527	0,01731	0,105	0,098	1
Prim2	0,592619	0,001914	0,131	0,141	1
Zfp932	0,592721	-0,01011	0,223	0,234	1
Dchs1	0,592748	-0,00219	0,006	0,008	1
Pipox	0,592835	0,007408	0,06	0,054	1
Nudt6	0,592885	-0,01297	0,078	0,085	1
Phtf1os	0,593011	-0,00125	0,006	0,008	1
Gm33148	0,593011	-0,00125	0,006	0,008	1
Pih1d2	0,593011	-0,00125	0,006	0,008	1
Tex22	0,593011	-0,00125	0,006	0,008	1
Gm14964	0,593011	-0,00125	0,006	0,008	1
Glt28d2	0,593159	0,006837	0,011	0,008	1
2310022A	0,593336	-0,01527	0,128	0,137	1
Dzank1	0,593372	-0,01723	0,095	0,102	1
Fkbp8	0,593452	0,014101	0,93	0,94	1
Gga3	0,593465	0,020538	0,203	0,193	1
Mex3d	0,593626	0,024372	0,277	0,268	1
Gid8	0,593728	-0,01072	0,299	0,311	1
Mageh1	0,593775	0,028103	0,386	0,385	1
Rgs7bp	0,59407	-0,00092	0,006	0,008	1
D630045J1	0,594101	0,004918	0,024	0,02	1
Aida	0,594111	-0,03076	0,338	0,341	1
Stag2	0,594172	-0,00442	0,75	0,766	1
Zfp236	0,594215	0,009459	0,23	0,218	1
Stom	0,594254	0,008448	0,011	0,008	1
Bid	0,594254	0,00458	0,011	0,008	1
Adam8	0,594254	0,00458	0,011	0,008	1
Cdh24	0,594254	0,00458	0,011	0,008	1
AL589692.	0,594254	0,00458	0,011	0,008	1
Pakap	0,594256	0,006837	0,011	0,008	1
Cdc42ep3	0,594334	0,001028	0,006	0,008	1
Shcbp1l	0,59451	0,008799	0,099	0,091	1
Usp37	0,59451	0,033464	0,325	0,317	1
Strn3	0,594758	-0,01484	0,66	0,67	1
Limk1	0,594812	-0,00271	0,119	0,128	1
Lin37	0,595034	0,017009	0,2	0,19	1
Dhrs13os	0,595135	0,004262	0,011	0,008	1
Ap1s2	0,595287	0,010879	0,148	0,138	1
Lrrc75aos2	0,595352	0,006516	0,011	0,008	1
Gm44718	0,595352	0,00361	0,011	0,008	1
Slc1a4	0,595352	0,00361	0,011	0,008	1
Gm17035	0,595352	0,00361	0,011	0,008	1
Gnal	0,595352	0,00361	0,011	0,008	1

Adgre5	0,595354	0,004904	0,011	0,008	1
Krr1	0,595694	0,03174	0,326	0,317	1
Chchd4	0,595748	-0,00576	0,306	0,32	1
Hsd17b7	0,595805	0,060547	0,359	0,353	1
Cds2	0,595863	-0,02904	0,725	0,73	1
Arid5a	0,595998	0,053454	0,242	0,233	1
Rp9	0,596065	0,03186	0,558	0,541	1
Zfpm1	0,596188	-0,02208	0,45	0,454	1
Hdac6	0,596309	0,01535	0,117	0,109	1
Pld2	0,596425	0,023736	0,134	0,127	1
1700007G1	0,596451	0,00264	0,011	0,008	1
1700112J1	0,596451	0,00264	0,011	0,008	1
Zfp882	0,596451	-0,01333	0,128	0,137	1
Golt1b	0,596539	-0,00175	0,367	0,389	1
4930518I1	0,596565	-0,00966	0,04	0,046	1
Gsk3a	0,596618	0,018505	0,629	0,618	1
Fxyd1	0,596664	0,014701	0,207	0,221	1
Prss53	0,59676	-0,00484	0,029	0,034	1
Rab6a	0,596768	0,023182	0,956	0,953	1
Prelid3a	0,596859	0,005005	0,111	0,102	1
Il11ra1	0,596999	-0,00846	0,166	0,176	1
Gm10827	0,597024	-0,0057	0,064	0,057	1
Gstk1	0,597178	0,013443	0,296	0,285	1
Fbxo45	0,597185	-0,01158	0,314	0,325	1
Nfyb	0,59721	-0,0006	0,354	0,372	1
Miip	0,597228	0,014681	0,105	0,098	1
Prdx6	0,597475	-0,00985	0,705	0,717	1
Rps27a	0,597482	0,011109	0,999	1	1
Trdmt1	0,597664	-0,00233	0,094	0,102	1
Agpat2	0,597743	0,009691	0,022	0,018	1
Mios	0,597775	0,001241	0,165	0,176	1
Yif1a	0,597824	-0,00808	0,565	0,574	1
Slf2	0,597853	0,014145	0,429	0,413	1
Lrch3	0,597885	-0,00286	0,381	0,4	1
Mgst1	0,597994	0,00039	0,011	0,008	1
Arpc2	0,598233	0,032721	0,847	0,834	1
Mms19	0,598528	-0,00587	0,18	0,19	1
Ap5z1	0,598805	-0,00974	0,294	0,309	1
Unc13b	0,598865	0,010722	0,065	0,059	1
2810442N1	0,598874	0,001028	0,011	0,008	1
8030442B(	0,598874	0,001028	0,011	0,008	1
Nyap1	0,599097	0,001359	0,011	0,008	1
Las1l	0,599126	0,016876	0,41	0,393	1
Cebpd	0,599212	0,046939	0,294	0,293	1
Gpr108	0,599376	-0,02106	0,426	0,433	1
Plin2	0,599381	-0,01622	0,122	0,13	1
Zfp521	0,599417	-0,00112	0,024	0,02	1
Ndufs7	0,599632	-0,01667	0,959	0,959	1
Glrx2	0,599685	0,005538	0,575	0,611	1
Lsm4	0,599717	-0,01015	0,674	0,68	1
Gm28782	0,599742	-0,00502	0,043	0,049	1
Cyb5r4	0,599814	0,007662	0,224	0,211	1

Dars2	0,599865	0,000316	0,103	0,112	1
Cryzl2	0,600102	-0,02382	0,226	0,233	1
Fam98c	0,600176	0,018391	0,211	0,202	1
Arhgap29	0,60021	0,008098	0,022	0,018	1
Morf4l2	0,600544	0,016303	0,745	0,78	1
Xrcc6	0,600593	0,022961	0,309	0,301	1
Lgals4	0,600886	0,006805	0,046	0,041	1
Sufu	0,601014	0,002107	0,109	0,119	1
Kcnab2	0,601038	-0,00934	0,024	0,028	1
Il4ra	0,601102	0,00327	0,003	0,002	1
Fndc9	0,601102	0,00327	0,003	0,002	1
Cenph	0,601102	0,00327	0,003	0,002	1
AW011738	0,601102	0,005221	0,003	0,002	1
Gm15402	0,601102	0,005221	0,003	0,002	1
Ak9	0,601102	0,005221	0,003	0,002	1
Hoxb5	0,601102	0,005221	0,003	0,002	1
Ntrk3	0,601102	0,004246	0,003	0,002	1
Vldlr	0,601132	0,040566	0,887	0,889	1
Snrpd1	0,601284	-0,00066	0,578	0,585	1
Lrrc73	0,601302	-0,00058	0,011	0,008	1
Prpf40b	0,601354	-0,00108	0,211	0,224	1
Gpatch4	0,601363	-0,00283	0,158	0,169	1
Fdx1	0,601424	-0,01756	0,289	0,301	1
Rln1	0,601541	0,005221	0,003	0,002	1
Adamts9	0,601541	0,004246	0,003	0,002	1
Lpl	0,601541	0,004246	0,003	0,002	1
Cox6a2	0,601541	0,00327	0,003	0,002	1
Piwil4	0,601541	0,00327	0,003	0,002	1
Copz2	0,601541	0,00327	0,003	0,002	1
Fam84a	0,601541	0,00327	0,003	0,002	1
Mdh1b	0,601541	0,002293	0,003	0,002	1
Apoa2	0,601541	0,002293	0,003	0,002	1
Pld5	0,601541	0,002293	0,003	0,002	1
Gm26869	0,601541	0,002293	0,003	0,002	1
Tsx	0,601541	0,002293	0,003	0,002	1
Hfm1	0,601541	0,002293	0,003	0,002	1
1700001J0	0,601541	0,002293	0,003	0,002	1
Vmn1r47	0,601541	0,002293	0,003	0,002	1
Phyhip	0,601541	0,002293	0,003	0,002	1
Spsb4	0,601541	0,002293	0,003	0,002	1
Gm12319	0,601541	0,002293	0,003	0,002	1
Ccl28	0,601541	0,002293	0,003	0,002	1
Krt83	0,601541	0,002293	0,003	0,002	1
Vasn	0,601541	0,002293	0,003	0,002	1
Gm10226	0,601541	0,002293	0,003	0,002	1
E230001N0	0,601541	0,002293	0,003	0,002	1
Ebi3	0,601541	0,002293	0,003	0,002	1
Arhgap28	0,601541	0,002293	0,003	0,002	1
Cd74	0,601541	0,002293	0,003	0,002	1
Eno4	0,601541	0,002293	0,003	0,002	1
4930430F0	0,601555	0,006075	0,407	0,429	1
Srpr	0,601565	0,015795	0,56	0,543	1

Bcorl1	0,60172	0,038028	0,355	0,35	1
Polr2g	0,60178	0,001597	0,647	0,686	1
Ap2b1	0,60195	0,013733	0,679	0,655	1
4930444P1	0,601981	0,001315	0,003	0,002	1
Mpp4	0,601981	0,001315	0,003	0,002	1
Myl1	0,601981	0,001315	0,003	0,002	1
Gm28535	0,601981	0,001315	0,003	0,002	1
Gm29514	0,601981	0,001315	0,003	0,002	1
Gm38037	0,601981	0,001315	0,003	0,002	1
Gm13479	0,601981	0,001315	0,003	0,002	1
Trp53i11	0,601981	0,001315	0,003	0,002	1
Pax6	0,601981	0,001315	0,003	0,002	1
Pabpc1l	0,601981	0,001315	0,003	0,002	1
Cdh4	0,601981	0,001315	0,003	0,002	1
Cybb	0,601981	0,001315	0,003	0,002	1
Srpx	0,601981	0,001315	0,003	0,002	1
Xlr4b	0,601981	0,001315	0,003	0,002	1
Tifa	0,601981	0,001315	0,003	0,002	1
Gm12394	0,601981	0,001315	0,003	0,002	1
Gm26566	0,601981	0,001315	0,003	0,002	1
Dnali1	0,601981	0,001315	0,003	0,002	1
Csf3r	0,601981	0,001315	0,003	0,002	1
Trnp1	0,601981	0,001315	0,003	0,002	1
Rhd	0,601981	0,001315	0,003	0,002	1
9130230L2	0,601981	0,001315	0,003	0,002	1
Gm16054	0,601981	0,001315	0,003	0,002	1
Cxcl10	0,601981	0,001315	0,003	0,002	1
Gm42902	0,601981	0,001315	0,003	0,002	1
Scnn1a	0,601981	0,001315	0,003	0,002	1
Vmn2r28	0,601981	0,001315	0,003	0,002	1
Prss23	0,601981	0,001315	0,003	0,002	1
Gm35082	0,601981	0,001315	0,003	0,002	1
Plk1	0,601981	0,001315	0,003	0,002	1
Chst11	0,601981	0,001315	0,003	0,002	1
Rdh9	0,601981	0,001315	0,003	0,002	1
Vmn2r87	0,601981	0,001315	0,003	0,002	1
Gm35572	0,601981	0,001315	0,003	0,002	1
Gm21817	0,601981	0,001315	0,003	0,002	1
4933406B1	0,601981	0,001315	0,003	0,002	1
Ces2b	0,601981	0,001315	0,003	0,002	1
Fhod1	0,601981	0,001315	0,003	0,002	1
Chst5	0,601981	0,001315	0,003	0,002	1
A430057M	0,601981	0,001315	0,003	0,002	1
Gm35835	0,601981	0,001315	0,003	0,002	1
Exph5	0,601981	0,001315	0,003	0,002	1
Gm38150	0,601981	0,001315	0,003	0,002	1
4732414G	0,601981	0,001315	0,003	0,002	1
Dph1	0,601981	0,001315	0,003	0,002	1
Hist1h2ak	0,601981	0,001315	0,003	0,002	1
Hist1h4i	0,601981	0,001315	0,003	0,002	1
Hist1h2bf	0,601981	0,001315	0,003	0,002	1
Gm35725	0,601981	0,001315	0,003	0,002	1

Gm26531	0,601981	0,001315	0,003	0,002	1
Ngb	0,601981	0,001315	0,003	0,002	1
9330161L(	0,601981	0,001315	0,003	0,002	1
Ptger4	0,601981	0,001315	0,003	0,002	1
Gm32618	0,601981	0,001315	0,003	0,002	1
Cpt1b	0,601981	0,001315	0,003	0,002	1
Igfbp6	0,601981	0,001315	0,003	0,002	1
AC161607.	0,601981	0,001315	0,003	0,002	1
Tmem30c	0,601981	0,001315	0,003	0,002	1
Pcdha12	0,601981	0,001315	0,003	0,002	1
Mcc	0,601981	0,001315	0,003	0,002	1
Gm26742	0,601981	0,001315	0,003	0,002	1
Golga7b	0,601981	0,001315	0,003	0,002	1
Ssh3	0,602035	0,01004	0,213	0,2	1
Vamp2	0,602195	0,024047	0,86	0,852	1
Tnk1	0,602422	0,001648	0,003	0,002	1
Zfp14	0,602479	-0,01055	0,173	0,182	1
Ppp2r2c	0,602489	-0,00314	0,898	0,889	1
A830052D:	0,602511	-0,00432	0,043	0,049	1
Exoc1	0,602546	-0,00078	0,421	0,444	1
Tbc1d16	0,602546	-0,00113	0,428	0,45	1
Uimc1	0,602667	-0,00338	0,293	0,309	1
Ankrd13c	0,602716	0,013288	0,433	0,421	1
Sfxn4	0,602976	0,002582	0,077	0,085	1
Sdc3	0,602985	0,005551	0,022	0,018	1
Gm28959	0,60299	0,005871	0,022	0,018	1
Brd3os	0,603009	-0,01078	0,048	0,054	1
Lsm3	0,603105	-0,02345	0,547	0,556	1
Rrad	0,603253	0,012954	0,009	0,007	1
Gm15675	0,603303	-0,00031	0,003	0,002	1
Tgm2	0,603303	-0,00031	0,003	0,002	1
Alpl	0,603303	-0,00031	0,003	0,002	1
D7Ertd128	0,603303	-0,00031	0,003	0,002	1
Cx3cl1	0,603303	-0,00031	0,003	0,002	1
Cabp7	0,603303	-0,00031	0,003	0,002	1
Hoxc10	0,603303	-0,00031	0,003	0,002	1
Ppp1r32	0,603303	-0,00031	0,003	0,002	1
Riiad1	0,603303	-0,00193	0,003	0,002	1
Gm20420	0,603303	-0,01283	0,003	0,002	1
Cacna2d3	0,603371	-0,00878	0,051	0,057	1
Plekhn1	0,603403	0,012371	0,289	0,275	1
Slc25a37	0,603561	-0,01066	0,07	0,076	1
Gpank1	0,603607	0,026517	0,223	0,216	1
Tmem132b	0,60402	-0,02383	0,513	0,527	1
Sub1	0,604091	0,014685	0,726	0,738	1
Scai	0,604254	-0,01459	0,209	0,22	1
Fyb	0,604285	-0,00394	0,026	0,031	1
Tes3-ps	0,6043	0,003368	0,039	0,034	1
Asns	0,604376	-0,00934	0,024	0,028	1
Mmp17	0,604399	-0,01053	0,009	0,011	1
1700019D(	0,604444	-0,01371	0,132	0,14	1
Tcte2	0,605048	-0,00048	0,065	0,072	1

Tcta	0,605094	0,02734	0,276	0,27	1
Ccdc74a	0,605215	0,005224	0,009	0,007	1
Lpp	0,605215	0,006194	0,009	0,007	1
4933408B1	0,605215	0,006194	0,009	0,007	1
Map2k5	0,605496	-0,00847	0,302	0,314	1
Zfp219	0,60558	-0,00633	0,08	0,088	1
Man2c1os	0,605701	0,004923	0,03	0,026	1
Nle1	0,605734	0,009534	0,065	0,059	1
Ric8b	0,605879	-0,00633	0,209	0,221	1
Gm14698	0,605902	0,010209	0,051	0,046	1
0610030E2	0,605945	-0,00938	0,335	0,348	1
Ppp1r8	0,606075	0,029646	0,259	0,252	1
Lrrc75a	0,606091	0,006189	0,022	0,018	1
Zfp239	0,606121	0,031693	0,477	0,475	1
4930515G0	0,606196	0,004254	0,009	0,007	1
Kcne4	0,606196	0,011991	0,009	0,007	1
Apold1	0,606196	0,005224	0,009	0,007	1
Phf5a	0,606316	0,010448	0,666	0,655	1
Ralgds	0,606712	0,007518	0,937	0,92	1
Exoc5	0,606794	-0,01711	0,583	0,58	1
Psmb7	0,606941	-0,01585	0,851	0,857	1
Smdt1	0,607001	-0,01061	0,981	0,989	1
Tceal8	0,607153	0,030001	0,327	0,319	1
Muc1	0,607179	0,005224	0,009	0,007	1
Katnal2	0,607179	0,004254	0,009	0,007	1
Il23a	0,607179	0,003283	0,009	0,007	1
Gm16279	0,607179	0,003283	0,009	0,007	1
Pde10a	0,607205	-0,002	0,026	0,031	1
Creb3l4	0,607426	0,00361	0,009	0,007	1
Psmd13	0,60767	0,0072	0,656	0,686	1
Fam217b	0,607672	0,003937	0,009	0,007	1
Gm10457	0,607673	0,005871	0,009	0,007	1
Atp6v0e	0,60779	0,005576	0,685	0,717	1
Ddx59	0,608136	-0,00102	0,125	0,115	1
Brinp1	0,60816	-0,00614	0,002	0,003	1
Rgsl1	0,608162	0,00231	0,009	0,007	1
Gm26801	0,608162	0,00231	0,009	0,007	1
Acsm3	0,608162	0,00231	0,009	0,007	1
Gm11520	0,608162	0,00231	0,009	0,007	1
St3gal3	0,608218	-0,02022	0,315	0,322	1
Cpsf4	0,608224	-0,00728	0,128	0,137	1
Ddx20	0,608374	0,017084	0,098	0,091	1
Eif3e	0,608406	-0,00769	0,672	0,698	1
Gtpbp3	0,608493	-0,00334	0,146	0,156	1
Stx2	0,60851	-0,01954	0,264	0,273	1
Zfp553	0,608535	0,028881	0,486	0,481	1
Akt2	0,608833	0,021341	0,364	0,353	1
Zfp658	0,608833	0,008352	0,051	0,046	1
Rbm7	0,608862	-0,0142	0,507	0,524	1
Ubtd1	0,609023	-0,00706	0,048	0,054	1
Prrx1	0,609044	-0,00291	0,002	0,003	1
Gm14455	0,609044	-0,00291	0,002	0,003	1

Ror1	0,609044	-0,00291	0,002	0,003	1
9430041J1	0,609044	-0,00291	0,002	0,003	1
Kcnc1	0,609044	-0,00291	0,002	0,003	1
Kcng4	0,609044	-0,00291	0,002	0,003	1
Gm47732	0,609044	-0,00291	0,002	0,003	1
Rnf182	0,609044	-0,00291	0,002	0,003	1
Gm30085	0,609044	-0,00291	0,002	0,003	1
Slc39a12	0,609044	-0,00452	0,002	0,003	1
Dyrk2	0,609044	-0,00452	0,002	0,003	1
Zfp872	0,609044	-0,00452	0,002	0,003	1
Bak1	0,609095	-0,02143	0,249	0,257	1
Gpld1	0,609105	0,00188	0,116	0,125	1
Luzp2	0,609114	-0,00624	0,363	0,38	1
Capg	0,609148	0,00264	0,009	0,007	1
Fxyd6	0,609278	0,007467	0,009	0,011	1
Acadsb	0,609475	0,009982	0,395	0,42	1
Pcdhb12	0,6095	-0,00539	0,009	0,011	1
Stx6	0,609507	0,025369	0,714	0,719	1
Arnt2	0,609683	0,017518	0,829	0,833	1
Slc4a2	0,609699	-0,00818	0,747	0,769	1
Dock7	0,60989	0,016907	0,205	0,195	1
Eya1	0,60993	-0,00129	0,002	0,003	1
Gm37233	0,60993	-0,00129	0,002	0,003	1
A930018P2	0,60993	-0,00129	0,002	0,003	1
Ano3	0,60993	-0,00129	0,002	0,003	1
Zfp217	0,60993	-0,00129	0,002	0,003	1
Sox3	0,60993	-0,00129	0,002	0,003	1
5430427O	0,60993	-0,00129	0,002	0,003	1
Gm15247	0,60993	-0,00129	0,002	0,003	1
Gm26530	0,60993	-0,00129	0,002	0,003	1
Gm42457	0,60993	-0,00129	0,002	0,003	1
Gm26857	0,60993	-0,00129	0,002	0,003	1
Rad54b	0,60993	-0,00129	0,002	0,003	1
Ptafr	0,60993	-0,00129	0,002	0,003	1
Tlr1	0,60993	-0,00129	0,002	0,003	1
Shisa3	0,60993	-0,00129	0,002	0,003	1
Gm16001	0,60993	-0,00129	0,002	0,003	1
Elfn1	0,60993	-0,00129	0,002	0,003	1
Card11	0,60993	-0,00129	0,002	0,003	1
Tex26	0,60993	-0,00129	0,002	0,003	1
Alox5	0,60993	-0,00129	0,002	0,003	1
Gm12781	0,60993	-0,00129	0,002	0,003	1
Tdrd12	0,60993	-0,00129	0,002	0,003	1
Homer2	0,60993	-0,00129	0,002	0,003	1
Trim6	0,60993	-0,00129	0,002	0,003	1
Olfr472	0,60993	-0,00129	0,002	0,003	1
Sh3rf3	0,60993	-0,00129	0,002	0,003	1
Gm48021	0,60993	-0,00129	0,002	0,003	1
4921516A(	0,60993	-0,00129	0,002	0,003	1
Smim18	0,60993	-0,00129	0,002	0,003	1
Gm45848	0,60993	-0,00129	0,002	0,003	1
Gm26586	0,60993	-0,00129	0,002	0,003	1

G430095P1	0,60993	-0,00129	0,002	0,003	1
4932416K2	0,60993	-0,00129	0,002	0,003	1
Exoc3l	0,60993	-0,00129	0,002	0,003	1
Il34	0,60993	-0,00129	0,002	0,003	1
Gm48933	0,60993	-0,00129	0,002	0,003	1
Olfm2	0,60993	-0,00129	0,002	0,003	1
Olfr981	0,60993	-0,00129	0,002	0,003	1
Bmp5	0,60993	-0,00129	0,002	0,003	1
Susd5	0,60993	-0,00129	0,002	0,003	1
Upp1	0,60993	-0,00129	0,002	0,003	1
Ntn1	0,60993	-0,00129	0,002	0,003	1
Gm11639	0,60993	-0,00129	0,002	0,003	1
Gm11772	0,60993	-0,00129	0,002	0,003	1
Gm26877	0,60993	-0,00129	0,002	0,003	1
Susd3	0,60993	-0,00129	0,002	0,003	1
Gprin1	0,60993	-0,00129	0,002	0,003	1
Cys1	0,60993	-0,00129	0,002	0,003	1
4930544F0	0,60993	-0,00129	0,002	0,003	1
Mpped1	0,60993	-0,00129	0,002	0,003	1
Ccdc184	0,60993	-0,00129	0,002	0,003	1
Rtl10	0,60993	-0,00129	0,002	0,003	1
B630019A1	0,60993	-0,00129	0,002	0,003	1
Tmem44	0,60993	-0,00129	0,002	0,003	1
Gm15564	0,60993	-0,00129	0,002	0,003	1
Prss27	0,60993	-0,00129	0,002	0,003	1
Rhbdl1	0,60993	-0,00129	0,002	0,003	1
Cpne5	0,60993	-0,00129	0,002	0,003	1
Fhod3	0,60993	-0,00129	0,002	0,003	1
Frmd8os	0,60993	-0,00129	0,002	0,003	1
Pip5k1b	0,60993	-0,00129	0,002	0,003	1
Arhgap4	0,60993	-0,00193	0,002	0,003	1
Gm33370	0,60993	-0,00193	0,002	0,003	1
Slc16a13	0,60993	-0,00193	0,002	0,003	1
Nmbr	0,60993	-0,00257	0,002	0,003	1
Rhebl1	0,610024	-0,00329	0,088	0,096	1
Myh9	0,610123	-0,00363	0,307	0,322	1
Adck5	0,610141	-0,00013	0,03	0,026	1
Oscp1	0,610227	-0,00386	0,062	0,068	1
Adap1	0,610373	0,007406	0,956	0,958	1
Vwa5b2	0,610373	2,86E-05	0,002	0,003	1
St8sia3	0,610373	-0,00095	0,002	0,003	1
Wdr38	0,610386	-0,00413	0,009	0,011	1
Gm26624	0,610386	-0,00413	0,009	0,011	1
Gm39473	0,610386	-0,00413	0,009	0,011	1
Trap1	0,610719	0,021969	0,34	0,333	1
Gpd1l	0,610775	0,023213	0,221	0,211	1
Nrip3	0,610816	0,001648	0,002	0,003	1
Arhgap42	0,610816	0,001648	0,002	0,003	1
Hes1	0,610816	0,001648	0,002	0,003	1
Efna5	0,610816	0,000671	0,002	0,003	1
Nyap2	0,610816	-0,00031	0,002	0,003	1
Gm20753	0,610816	-0,00031	0,002	0,003	1

B4galt1	0,610816	-0,00031	0,002	0,003	1
Rab42	0,610816	-0,00031	0,002	0,003	1
Hes5	0,610816	-0,00031	0,002	0,003	1
Gm43118	0,610816	-0,00031	0,002	0,003	1
Nptx2	0,610816	-0,00031	0,002	0,003	1
Npy	0,610816	-0,00031	0,002	0,003	1
Olfr374	0,610816	-0,00031	0,002	0,003	1
Cpne6	0,610816	-0,00031	0,002	0,003	1
Cideb	0,610816	-0,00031	0,002	0,003	1
6430571L1	0,610816	-0,00031	0,002	0,003	1
Fzd2	0,610816	-0,00031	0,002	0,003	1
Shc3	0,610816	-0,00031	0,002	0,003	1
Rsph1	0,610816	-0,00095	0,002	0,003	1
Selenof	0,61082	0,013066	0,971	0,984	1
Fam92a	0,610987	-0,0026	0,343	0,361	1
Tmem196	0,611034	-0,00075	0,026	0,031	1
Nxf1	0,611046	-0,00236	0,737	0,769	1
Trp53rka	0,61123	0,030807	0,271	0,265	1
Tbrg1	0,611281	-0,01452	0,471	0,481	1
Xpnpep1	0,611284	0,024909	0,23	0,223	1
Eml4	0,611476	-0,01505	0,121	0,128	1
Hsf1	0,61158	-0,00044	0,263	0,278	1
Hpgds	0,611612	5,69E-05	0,009	0,007	1
Rorc	0,611703	0,000671	0,002	0,003	1
Gm47604	0,611703	0,000671	0,002	0,003	1
Kalrn	0,611703	0,008461	0,002	0,003	1
Tmem158	0,611703	0,002624	0,002	0,003	1
Mphosph8	0,61179	0,007259	0,59	0,613	1
Ugdh	0,611818	0,037429	0,219	0,208	1
Dock10	0,611889	-0,00885	0,979	0,967	1
Mon1a	0,61202	-0,00315	0,159	0,169	1
Msrb3	0,612089	0,000819	0,026	0,031	1
Sema6d	0,612165	0,002354	0,657	0,67	1
Smarce1	0,612235	-0,01077	0,511	0,525	1
Kcnq4	0,612387	-0,00252	0,009	0,011	1
H6pd	0,612387	-0,00252	0,009	0,011	1
Sbk2	0,612387	-0,00252	0,009	0,011	1
Mmd	0,612387	-0,00252	0,009	0,011	1
Hoxb8	0,612387	-0,00252	0,009	0,011	1
Gm4211	0,612387	-0,00252	0,009	0,011	1
1010001B2	0,61254	-0,0108	0,012	0,015	1
Hint2	0,61258	-0,01424	0,311	0,322	1
Fam83a	0,612836	-0,00345	0,009	0,011	1
Ddx18	0,61293	0,036729	0,488	0,488	1
Lyrm1	0,613072	-0,01084	0,13	0,138	1
Tmem127	0,613247	0,01273	0,429	0,415	1
Pdlim2	0,613248	-0,00541	0,969	0,979	1
Mkln1	0,613249	0,030705	0,73	0,732	1
Zfp958	0,613608	-0,00507	0,088	0,096	1
Mapk4	0,613726	-0,00122	0,009	0,011	1
Nif3l1	0,61381	-0,00684	0,117	0,125	1
Aars	0,613818	-0,00764	0,551	0,559	1

Uqcrc2	0,613824	0,014218	0,848	0,859	1
Gm13375	0,61383	-0,00591	0,07	0,076	1
Ino80d	0,613858	0,011168	0,546	0,567	1
Klf10	0,613906	-0,00438	0,121	0,13	1
4932430I1	0,613948	-0,00058	0,009	0,011	1
Gm10644	0,613948	-0,00155	0,009	0,011	1
Gm26846	0,613948	-0,00155	0,009	0,011	1
Tjp2	0,613992	-0,01487	0,811	0,813	1
Timm29	0,61403	-0,00707	0,29	0,299	1
Kbtbd4	0,614402	-0,01001	0,111	0,119	1
Sema3d	0,614425	-0,01231	0,059	0,065	1
Nfya	0,614645	0,000736	0,208	0,223	1
Nup93	0,614681	-0,00493	0,099	0,107	1
Unkl	0,614794	-0,00877	0,116	0,124	1
Tmem51os	0,614887	0,005871	0,007	0,005	1
BC049987	0,614887	0,005871	0,007	0,005	1
Ung	0,614887	0,007811	0,007	0,005	1
Sar1a	0,615057	0,000862	0,605	0,628	1
Rpa3	0,615081	8,05E-05	0,31	0,327	1
Ccdc106	0,61551	-0,00058	0,009	0,011	1
Fgf9	0,61551	0,00039	0,009	0,011	1
4732491K2	0,615646	0,00216	0,053	0,047	1
Cftr	0,615731	0,003927	0,007	0,005	1
Tagln	0,615731	0,003927	0,007	0,005	1
Ccr5	0,615731	0,003927	0,007	0,005	1
Gm12359	0,615731	0,003927	0,007	0,005	1
Wfdc12	0,615731	0,006841	0,007	0,005	1
Tbc1d1	0,615731	0,006841	0,007	0,005	1
Ebf4	0,615731	0,0049	0,007	0,005	1
Myl12a	0,615837	-0,01651	0,571	0,579	1
Traf3ip1	0,615895	0,013501	0,132	0,124	1
E2f3	0,616024	-0,00871	0,064	0,07	1
Tpm1	0,616106	0,032605	0,637	0,639	1
Trp53inp1	0,616463	0,012908	0,208	0,22	1
Cep170	0,61647	-0,01617	0,726	0,735	1
Tec	0,616576	0,0049	0,007	0,005	1
A330074K2	0,616576	0,003927	0,007	0,005	1
Cdc25b	0,616576	0,002954	0,007	0,005	1
Vsig10l	0,616576	0,002954	0,007	0,005	1
Kcnj12	0,616576	0,002954	0,007	0,005	1
Sfrp4	0,616576	0,002954	0,007	0,005	1
Kcnh5	0,616576	0,002954	0,007	0,005	1
Ablim3	0,616576	0,002954	0,007	0,005	1
Etl4	0,616676	-0,00466	0,018	0,021	1
Nol8	0,616695	0,004745	0,321	0,307	1
Fam83e	0,616859	0,003283	0,007	0,005	1
Isg20l2	0,616866	-0,00611	0,146	0,156	1
Tsen34	0,616933	0,010685	0,585	0,563	1
Pram1	0,617024	-0,00408	0,012	0,015	1
Rela	0,617027	0,027024	0,449	0,446	1
Reps1	0,617216	0,013144	0,281	0,268	1
2810049E0	0,617217	-0,00442	0,012	0,015	1

Gm17111	0,617217	-0,00442	0,012	0,015	1
P4ha1	0,617283	0,028943	0,368	0,395	1
Olfr212	0,617422	0,00198	0,007	0,005	1
Ces2f	0,617422	0,00198	0,007	0,005	1
C230035I1	0,617422	0,00198	0,007	0,005	1
Hist1h3f	0,617422	0,00198	0,007	0,005	1
Gm26803	0,617422	0,00198	0,007	0,005	1
Ppp1r3c	0,617422	0,00198	0,007	0,005	1
St5	0,617628	-0,01092	0,222	0,233	1
Klk7	0,617706	0,00231	0,007	0,005	1
Pex5	0,617819	-0,00452	0,238	0,252	1
Nupr1	0,617838	0,005294	0,088	0,081	1
Dnajb4	0,618131	-0,01259	0,426	0,437	1
Polr3gl	0,618186	0,001572	0,19	0,179	1
Grin3a	0,618191	0,005547	0,005	0,003	1
Hgh1	0,618461	-0,00399	0,048	0,054	1
Kcnj2	0,618513	-0,01211	0,174	0,182	1
Afmid	0,618552	0,001337	0,007	0,005	1
G630018N	0,618552	0,001337	0,007	0,005	1
Colec11	0,618626	0,00014	0,021	0,024	1
Cryaa	0,618639	0,002328	0,009	0,011	1
Fbxl8	0,618723	0,010835	0,049	0,044	1
Ssu72	0,618725	-0,00159	0,635	0,676	1
Gm32633	0,618742	0,012787	0,035	0,031	1
Syn2	0,618784	-0,00312	0,012	0,015	1
Pdss2	0,618803	-0,00551	0,13	0,138	1
Hvcn1	0,618865	0,003599	0,005	0,003	1
Gm45069	0,618865	0,005547	0,005	0,003	1
Gm16982	0,618865	0,004573	0,005	0,003	1
Plau	0,618865	0,004573	0,005	0,003	1
Icam1	0,618865	0,004573	0,005	0,003	1
Klhl23	0,618977	-0,00345	0,012	0,015	1
6430573P(	0,618977	-0,00345	0,012	0,015	1
Gm12840	0,619097	-0,00274	0,018	0,021	1
Ap1ar	0,619111	0,005146	0,272	0,288	1
Parvb	0,619211	0,0104	0,158	0,148	1
Xab2	0,619238	0,020434	0,427	0,418	1
Gm45345	0,619336	-0,00693	0,166	0,176	1
Gm48935	0,619399	0,000363	0,007	0,005	1
Fbxl7	0,619399	0,000363	0,007	0,005	1
Fancd2	0,619399	-0,00125	0,007	0,005	1
Adam1a	0,619539	0,006519	0,005	0,003	1
Cavin1	0,619539	0,004573	0,005	0,003	1
Fat4	0,619539	0,003599	0,005	0,003	1
Igsf21	0,619539	0,003599	0,005	0,003	1
3632451O(	0,619539	0,003599	0,005	0,003	1
Tstd1	0,619539	0,002624	0,005	0,003	1
Gm17300	0,619539	0,002624	0,005	0,003	1
Rsg1	0,619539	0,002624	0,005	0,003	1
Gm42984	0,619539	0,002624	0,005	0,003	1
Pzp	0,619539	0,002624	0,005	0,003	1
Gm38947	0,619539	0,002624	0,005	0,003	1

Tnfaip3	0,619539	0,002624	0,005	0,003	1
Slfn5	0,619539	0,002624	0,005	0,003	1
Gm16059	0,619539	0,002624	0,005	0,003	1
Gm15829	0,619539	0,002624	0,005	0,003	1
Mdga1	0,619539	0,002624	0,005	0,003	1
Prickle4	0,619539	0,002624	0,005	0,003	1
Sema6b	0,619539	0,002624	0,005	0,003	1
Stx1a	0,619562	-0,00282	0,012	0,015	1
Ctf1	0,620029	-0,00181	0,015	0,018	1
Mief1	0,620067	-0,0003	0,22	0,233	1
Thap2	0,620136	-0,00244	0,531	0,553	1
C330011M	0,620205	0,004262	0,009	0,011	1
Gm28551	0,620214	0,001648	0,005	0,003	1
Gm26892	0,620214	0,001648	0,005	0,003	1
Gssos2	0,620214	0,001648	0,005	0,003	1
Kcnq2	0,620214	0,001648	0,005	0,003	1
Rtl8c	0,620214	0,001648	0,005	0,003	1
Kcnn3	0,620214	0,001648	0,005	0,003	1
Gm128	0,620214	0,001648	0,005	0,003	1
AI427809	0,620214	0,001648	0,005	0,003	1
Fhad1	0,620214	0,001648	0,005	0,003	1
Cd38	0,620214	0,001648	0,005	0,003	1
Gm15627	0,620214	0,001648	0,005	0,003	1
Cldn15	0,620214	0,001648	0,005	0,003	1
Gm10874	0,620214	0,001648	0,005	0,003	1
Gm26790	0,620214	0,001648	0,005	0,003	1
Gm45437	0,620214	0,001648	0,005	0,003	1
Gm45441	0,620214	0,001648	0,005	0,003	1
Syngr4	0,620214	0,001648	0,005	0,003	1
Gm32687	0,620214	0,001648	0,005	0,003	1
Nwd1	0,620214	0,001648	0,005	0,003	1
Odf3l1	0,620214	0,001648	0,005	0,003	1
Gm12212	0,620214	0,001648	0,005	0,003	1
Gm4262	0,620214	0,001648	0,005	0,003	1
Gm5678	0,620214	0,001648	0,005	0,003	1
3300005D(	0,620214	0,001648	0,005	0,003	1
Kcnip2	0,620214	0,001648	0,005	0,003	1
Mpc2	0,62028	0,012613	0,889	0,876	1
Tigar	0,620287	-0,00246	0,051	0,057	1
Ildr1	0,620383	-0,00244	0,018	0,021	1
Tmem35b	0,620464	0,002411	0,037	0,033	1
Casp12	0,620552	0,00231	0,005	0,003	1
Gm10118	0,620555	-0,00312	0,015	0,018	1
Ero1lb	0,620778	0,005871	0,072	0,065	1
Wdr83	0,620808	-0,00846	0,258	0,268	1
Stk4	0,620813	-0,00159	0,319	0,335	1
Ckap4	0,62089	0,00198	0,005	0,003	1
Vasp	0,620917	-0,00753	0,13	0,138	1
Samd12	0,620951	-0,0004	0,037	0,033	1
Col9a3	0,620961	0,018692	0,419	0,405	1
Chpf	0,620997	-0,0044	0,088	0,096	1
Ash1l	0,621161	0,023194	0,817	0,811	1

Suv39h1	0,621162	-0,00111	0,167	0,177	1
Slc46a3	0,621197	-0,0037	0,018	0,021	1
Pusl1	0,62121	-0,01148	0,214	0,224	1
Dtl	0,621227	0,001005	0,005	0,003	1
9530046B1	0,621227	0,001005	0,005	0,003	1
Slc6a15	0,621227	0,001005	0,005	0,003	1
Cacng4	0,621227	0,001005	0,005	0,003	1
Rrp1b	0,621304	-0,01307	0,162	0,171	1
Fam110a	0,621325	-0,00185	0,012	0,015	1
Scand1	0,621353	-0,01453	0,827	0,831	1
Pccb	0,621529	-0,01213	0,377	0,387	1
Fancm	0,621551	0,014345	0,128	0,12	1
Rab43	0,621559	0,006814	0,035	0,031	1
Kif22	0,621561	0,003368	0,037	0,033	1
Fhl4	0,621565	-0,00061	0,005	0,003	1
Arhgef7	0,621764	-0,01063	0,211	0,223	1
8030455M	0,621903	2,86E-05	0,005	0,003	1
Gm19531	0,621903	2,86E-05	0,005	0,003	1
BC049762	0,621903	2,86E-05	0,005	0,003	1
Gm17058	0,621903	2,86E-05	0,005	0,003	1
Stx19	0,621903	2,86E-05	0,005	0,003	1
Cchcr1	0,621903	2,86E-05	0,005	0,003	1
Rab9b	0,621948	0,004592	0,02	0,016	1
Npat	0,622132	0,006034	0,575	0,559	1
Dram2	0,622292	-0,02255	0,447	0,462	1
Sco2	0,622431	-0,00748	0,132	0,141	1
Pot1b	0,622431	-0,00596	0,132	0,141	1
Gm13920	0,622445	0,004277	0,02	0,016	1
mt-Nd5	0,622493	-0,02231	0,996	0,993	1
Det1	0,622624	0,000666	0,054	0,06	1
Ppp4r1	0,622781	0,024739	0,206	0,198	1
Dicer1	0,622876	0,029914	0,632	0,634	1
A930029G1	0,62309	-0,00088	0,012	0,015	1
Irak1bp1	0,623231	0,019453	0,135	0,128	1
Cep192	0,623374	0,000421	0,155	0,166	1
Serac1	0,623503	0,004949	0,058	0,052	1
5330413P1	0,623591	0,003632	0,02	0,016	1
Dock6	0,623591	0,003632	0,02	0,016	1
Rnf167	0,62373	0,007396	0,579	0,558	1
Slain1os	0,62392	0,002036	0,02	0,016	1
Phf21b	0,624089	0,001075	0,015	0,018	1
Mcmdc2	0,62427	-0,00055	0,012	0,015	1
Cradd	0,62432	-0,00314	0,195	0,207	1
Zfp212	0,624375	-0,00146	0,138	0,148	1
Ruvbl2	0,62438	0,024819	0,296	0,289	1
Ccdc189	0,624595	-0,00052	0,018	0,021	1
Fam81a	0,624678	0,003332	0,028	0,024	1
Sf3b1	0,625114	0,007433	0,924	0,95	1
Ncoa6	0,625264	0,006085	0,368	0,387	1
Sin3b	0,625623	-0,01425	0,599	0,597	1
Ddb1	0,62581	0,03477	0,716	0,75	1
Tmem183a	0,625866	-0,00588	0,311	0,322	1

Sfxn2	0,625882	0,01293	0,147	0,159	1
Pdhx	0,62592	-0,00776	0,237	0,247	1
Exo5	0,626026	0,012302	0,061	0,055	1
Ilk	0,626038	0,014089	0,664	0,659	1
Uba5	0,626147	0,015143	0,512	0,498	1
Fam50a	0,626366	-0,00647	0,391	0,407	1
Gm16552	0,626626	0,003946	0,012	0,015	1
2610301B2	0,626955	0,020225	0,193	0,185	1
Fancc	0,626997	-0,01404	0,207	0,216	1
Nat2	0,627032	-0,00413	0,034	0,039	1
Syap1	0,627192	0,020676	0,352	0,345	1
Rnf5	0,627193	0,0296	0,839	0,862	1
2610020C0	0,627196	0,008056	0,042	0,037	1
Pag1	0,627271	0,011532	0,225	0,215	1
Pan3	0,627412	0,039855	0,405	0,408	1
C130071C0	0,627804	0,002854	0,287	0,299	1
Gm10687	0,627882	0,00478	0,15	0,161	1
Klhl42	0,627915	-0,0072	0,145	0,154	1
Rpl27a	0,62793	0,00608	0,997	0,998	1
Nedd1	0,627946	0,003946	0,155	0,166	1
Zfp128	0,628038	0,006489	0,056	0,05	1
Pcdhga6	0,628287	-0,00089	0,051	0,057	1
Gm12207	0,628335	0,002671	0,015	0,018	1
Haus7	0,628381	-0,00587	0,185	0,195	1
Map1b	0,628444	-0,0642	0,841	0,844	1
Gm35439	0,628538	0,001075	0,02	0,016	1
Zfp292	0,628564	-0,00528	0,52	0,538	1
Lipa	0,6286	-0,01622	0,542	0,553	1
Ska2	0,628998	0,008629	0,152	0,143	1
Rac3	0,629046	-0,00163	0,034	0,039	1
Mrps31	0,629151	0,008654	0,324	0,315	1
Pde11a	0,629558	-0,007	0,053	0,059	1
Cdk19	0,629575	-0,00785	0,883	0,906	1
1300017J0	0,629931	0,004918	0,021	0,024	1
Nnat	0,629962	0,011161	0,047	0,042	1
Commd5	0,629967	-0,00427	0,056	0,062	1
Abca3	0,630009	0,006603	0,26	0,276	1
Immp2l	0,630087	0,004091	0,095	0,088	1
Foxo3	0,630475	0,00198	0,267	0,254	1
Rad21	0,630495	0,006551	0,81	0,777	1
Zfp850	0,630544	0,009362	0,026	0,023	1
Gyg	0,630567	-0,00738	0,431	0,452	1
Ndufb6	0,630653	-0,01184	0,63	0,646	1
Pqbp1	0,631066	-0,00462	0,494	0,514	1
Wdr55	0,631109	0,010986	0,138	0,13	1
Cop1	0,63118	0,018489	0,541	0,53	1
Chrac1	0,631215	0,002885	0,311	0,298	1
Rab3d	0,631405	0,01282	0,026	0,023	1
Hoxa2	0,631432	-0,0029	0,067	0,073	1
Cyhr1	0,63151	-0,00495	0,876	0,893	1
Rnf168	0,631647	0,004391	0,365	0,384	1
Atp5j2	0,631708	0,01823	0,974	0,963	1

Trim13	0,631814	0,019479	0,516	0,509	1
Med9	0,631903	0,002566	0,242	0,229	1
AU022252	0,632081	0,003804	0,208	0,221	1
Wdyhv1	0,632186	-0,01036	0,123	0,13	1
Morn4	0,632209	0,016386	0,154	0,146	1
Qsox2	0,632259	-0,00182	0,25	0,262	1
Cep152	0,632321	0,000936	0,051	0,057	1
Rfc4	0,632328	0,012191	0,125	0,117	1
Lrrn4cl	0,632399	0,00971	0,018	0,015	1
Rprd1b	0,63254	0,005381	0,259	0,273	1
Fam216a	0,632634	-0,00672	0,184	0,195	1
Nae1	0,632654	0,000787	0,42	0,431	1
Dtx4	0,632721	-0,01215	0,089	0,096	1
Prdx2	0,632761	0,007464	0,851	0,872	1
Tmc3	0,632769	0,004743	0,109	0,117	1
Wnt5b	0,633447	-0,00196	0,031	0,036	1
Afg3l2	0,633454	-0,00307	0,198	0,21	1
Ppfibp2	0,633698	0,031131	0,856	0,836	1
Pard3b	0,633969	0,008751	0,018	0,015	1
Aph1c	0,634189	-0,00418	0,031	0,036	1
Psd2	0,634268	-0,00919	0,194	0,205	1
Cetn3	0,634489	0,004287	0,763	0,785	1
Pih1d1	0,634703	0,00657	0,304	0,324	1
Snrnp25	0,634715	-0,00881	0,187	0,197	1
Wdhd1	0,634819	0,016005	0,047	0,042	1
Amer2	0,634912	-0,00503	0,6	0,608	1
Sipa1l2	0,634966	-0,01643	0,24	0,249	1
Lmtk2	0,634999	0,010117	0,136	0,128	1
Mus81	0,635036	0,00256	0,152	0,163	1
Atp6v0b	0,635122	0,018013	0,991	0,997	1
2510009E(	0,635477	0,011128	0,588	0,564	1
Zbtb7b	0,635567	-0,00803	0,319	0,332	1
6430548M	0,635763	-0,01451	0,208	0,216	1
Mrgpre	0,635884	-0,00209	0,09	0,098	1
Tns2	0,635921	0,011913	0,123	0,115	1
Eif3a	0,635984	0,014386	0,929	0,932	1
Tada1	0,636171	-0,01272	0,544	0,551	1
Atrn	0,636221	0,00327	0,788	0,811	1
2810004N:	0,636472	0,015152	0,451	0,444	1
Asah1	0,636832	0,00974	0,555	0,535	1
Lrch1	0,63684	0,002356	0,096	0,104	1
Plekha1	0,636859	-0,01818	0,82	0,833	1
Rbm17	0,636963	-0,00352	0,452	0,478	1
Cdr2	0,637109	-0,00482	0,179	0,187	1
Decr2	0,637246	0,019819	0,064	0,059	1
Ric8a	0,637257	0,01802	0,42	0,416	1
Myzap	0,637268	0,011112	0,047	0,042	1
Cul3	0,638043	-0,00286	0,71	0,714	1
Ccdc191	0,638218	-0,01797	0,249	0,257	1
Pard3	0,638282	-0,0102	0,626	0,629	1
Cux2	0,638285	-0,00506	0,172	0,18	1
Gm14966	0,638298	0,01023	0,047	0,042	1

Cetn2	0,638381	0,009244	0,347	0,335	1
Idh2	0,638619	-0,01606	0,273	0,281	1
Mfsd11	0,638782	0,012849	0,372	0,361	1
Amfr	0,63887	0,011511	0,749	0,746	1
Crebl2	0,638948	-0,01272	0,328	0,34	1
Rpgrip1l	0,639204	-0,0127	0,169	0,177	1
Ttc3	0,639258	0,022097	0,803	0,777	1
Stxbp2	0,639638	-0,04633	0,25	0,262	1
Kif1c	0,639885	-0,00988	0,184	0,195	1
2310016G:	0,639945	0,006188	0,026	0,023	1
Traf2	0,640007	0,015362	0,186	0,179	1
Fndc3b	0,640034	0,000331	0,131	0,14	1
Tm2d1	0,640121	-0,00279	0,494	0,512	1
Chsy1	0,640261	0,004909	0,018	0,015	1
Wasf2	0,640287	-0,00737	0,688	0,706	1
Trappc3	0,640315	-0,00573	0,61	0,623	1
Tango6	0,640396	0,004949	0,056	0,05	1
Creb1	0,640423	0,00917	0,508	0,491	1
Tmc4	0,640444	0,004913	0,018	0,015	1
Sgo2a	0,640774	-0,00946	0,028	0,024	1
Tpm3-rs7	0,640883	0,006818	0,033	0,029	1
Rhot2	0,640931	0,018583	0,22	0,213	1
Slc35c2	0,640967	0,012533	0,194	0,185	1
Bud23	0,641108	0,000162	0,267	0,281	1
Zc3h7b	0,641503	-0,00527	0,405	0,426	1
Birc6	0,641582	-0,01383	0,773	0,787	1
Gm36839	0,6416	0,020443	0,062	0,057	1
Filip1l	0,641721	0,008671	0,04	0,036	1
Slc41a3	0,641735	-0,00282	0,042	0,047	1
Card10	0,641752	0,010971	0,073	0,067	1
Mterf3	0,641822	-0,00302	0,299	0,311	1
D830025C(	0,641924	0,013731	0,066	0,06	1
Zfp747	0,642009	0,013695	0,076	0,07	1
Gm16286	0,642528	0,022074	0,684	0,691	1
Gm12802	0,642677	-0,00361	0,028	0,033	1
Zfp334	0,642828	-0,0004	0,122	0,13	1
Sh2d6	0,642954	-0,00679	0,042	0,047	1
Tmem246	0,642956	0,030188	0,38	0,376	1
Man1b1	0,643039	0,007383	0,377	0,397	1
Dnajc21	0,643093	0,006703	0,38	0,398	1
Cops4	0,643211	0,017383	0,571	0,561	1
Cand1	0,643269	0,011262	0,343	0,364	1
Apoo	0,643297	0,005067	0,192	0,182	1
Tmem87b	0,643406	-0,01129	0,497	0,511	1
Neil1	0,643465	0,009343	0,118	0,111	1
Isca1	0,643591	-0,0106	0,236	0,244	1
Gm26722	0,643953	0,012243	0,071	0,065	1
Metap2	0,644282	0,026512	0,932	0,922	1
Xndc1	0,644347	-0,00185	0,182	0,192	1
Taldo1	0,644403	-0,00273	0,999	0,998	1
Arl6ip6	0,644715	0,014709	0,213	0,205	1
Ubxn2b	0,644958	0,002394	0,178	0,189	1

6720483E2	0,645002	-0,00022	0,018	0,015	1
Atp6v1f	0,645147	0,016962	0,937	0,946	1
Cnot6	0,645329	-0,00057	0,406	0,423	1
Tpcn2	0,645438	-0,0017	0,028	0,033	1
Lrrc75b	0,645516	0,016937	0,109	0,102	1
Cox7a2	0,645533	-0,01114	0,982	0,972	1
2310061I0	0,645572	0,020092	0,236	0,228	1
B230369F2	0,645655	-0,00732	0,081	0,088	1
Sppl2a	0,645922	-0,01422	0,477	0,483	1
Ctdnep1	0,645944	-0,00319	0,396	0,418	1
Ccnj	0,645967	-0,00575	0,058	0,063	1
Ywhag	0,646243	0,03135	0,706	0,715	1
Ddx21	0,646565	0,002888	0,359	0,341	1
N4bp2	0,646572	-0,0047	0,102	0,109	1
Zfr	0,646701	-0,00892	0,838	0,839	1
Greb1l	0,646764	-0,00403	0,018	0,015	1
Abhd14b	0,64682	-0,00365	0,221	0,231	1
Nop58	0,647234	0,01272	0,284	0,299	1
Syngr1	0,647309	-2,9E-05	0,132	0,141	1
Stx3	0,647375	-0,00402	0,157	0,148	1
Sesn2	0,647382	0,004017	0,045	0,05	1
Rnf141	0,647438	-0,00055	0,883	0,899	1
1700066M	0,647463	-0,00714	0,284	0,296	1
A230072E1	0,647513	-0,00872	0,023	0,026	1
Sdcbp	0,64766	-0,0176	0,46	0,459	1
Mkrn2	0,647711	0,025413	0,364	0,359	1
Slc35b3	0,647729	-0,00701	0,181	0,19	1
Spindoc	0,647852	0,01324	0,099	0,093	1
Ccdc89	0,647855	-0,00819	0,055	0,06	1
Faim	0,648218	-0,00462	0,608	0,624	1
Zfp955a	0,648267	-0,01086	0,044	0,049	1
Gmds	0,648384	-0,0014	0,15	0,14	1
Cald1	0,648584	0,021612	0,538	0,569	1
Ptges3	0,648703	-0,01128	0,985	0,974	1
Pros1	0,648728	-0,00934	0,183	0,19	1
Cd81	0,648755	0,026851	0,999	1	1
Galnt1	0,648764	-0,00035	0,513	0,532	1
Hlcs	0,648932	0,011994	0,19	0,182	1
Cbx6	0,649048	0,027345	0,634	0,636	1
Pogk	0,64909	-0,00981	0,674	0,696	1
Gins4	0,649094	-0,01583	0,548	0,564	1
Nrap	0,649105	-0,00641	0,136	0,145	1
Kremen1	0,649166	0,013616	0,184	0,176	1
Mtrf1	0,649653	0,001154	0,093	0,101	1
Dnajc19	0,649938	0,00452	0,758	0,797	1
Foxred2	0,649948	-0,00651	0,047	0,052	1
Thoc3	0,649964	-0,01319	0,363	0,374	1
Pfdn5	0,650045	0,0089	0,975	0,972	1
Zfp422	0,650074	-0,00961	0,17	0,177	1
Snx4	0,650321	-0,01154	0,329	0,338	1
Lrpap1	0,650325	0,002241	0,679	0,714	1
Ripk1	0,650436	0,039003	0,179	0,176	1

Slc6a1	0,650474	0,007667	0,633	0,628	1
Gm10138	0,650624	0,003212	0,085	0,093	1
Sox12	0,650701	-0,00025	0,175	0,185	1
Pigm	0,650911	0,017281	0,171	0,163	1
Rragd	0,650926	0,01221	0,025	0,021	1
Syne1	0,65107	-0,00875	0,162	0,171	1
Rab14	0,651158	-0,01493	0,988	0,992	1
Gm13889	0,651307	-0,0168	0,088	0,094	1
Tmed4	0,65139	-0,00304	0,76	0,793	1
5730480H(	0,651498	-0,00439	0,148	0,158	1
Hira	0,651671	-0,01859	0,233	0,241	1
Zfp983	0,651694	-0,01256	0,068	0,073	1
Ccser2	0,651872	-0,00996	0,516	0,532	1
Arrdc1	0,651931	0,01023	0,045	0,041	1
Gm48742	0,652139	0,009869	0,052	0,047	1
Cnot7	0,652202	0,027481	0,397	0,392	1
Ccdc115	0,652577	-0,00717	0,241	0,252	1
Lgals3bp	0,652581	-0,01064	0,02	0,023	1
Edc3	0,652633	0,008421	0,134	0,127	1
Sacs	0,652893	0,012018	0,169	0,161	1
Kctd9	0,653585	0,0137	0,114	0,107	1
Txnl4b	0,653882	-0,00301	0,124	0,132	1
Arhgef1	0,654165	0,003921	0,356	0,374	1
Usp49	0,65422	0,007086	0,066	0,06	1
Cenps	0,654406	0,002716	0,028	0,033	1
Tnfaip8	0,654631	-0,00361	0,026	0,029	1
Cep41	0,654817	0,009255	0,128	0,12	1
Bag5	0,654912	0,027695	0,312	0,311	1
Gm10655	0,655312	0,007475	0,016	0,013	1
Mtr	0,655461	0,002859	0,066	0,06	1
Tlnrd1	0,655539	0,003792	0,091	0,085	1
Tada3	0,655796	-0,00804	0,352	0,363	1
Crocc	0,655868	0,001143	0,028	0,033	1
Ccdc85c	0,655879	-0,00505	0,066	0,072	1
Anapc7	0,655974	-0,0022	0,319	0,33	1
Hipk1	0,656183	-0,0118	0,454	0,47	1
Pecam1	0,656269	0,00902	0,031	0,028	1
Casc3	0,656424	-0,00651	0,403	0,416	1
Clcn5	0,656492	0,002843	0,278	0,291	1
Tmsb4x	0,656922	-0,01589	0,999	1	1
Asphd1	0,657137	0,010614	0,087	0,081	1
Mrps24	0,657197	-0,0132	0,639	0,644	1
Pgap3	0,657326	-0,00618	0,044	0,049	1
B3gat2	0,657689	-0,00399	0,023	0,026	1
Fam124a	0,657706	-0,00859	0,023	0,026	1
Zscan29	0,657716	0,012032	0,143	0,137	1
Pinx1	0,657789	0,006147	0,143	0,153	1
Frzb	0,657943	0,00715	0,016	0,013	1
Elp4	0,657946	0,000858	0,295	0,312	1
Palm2	0,658045	-0,03119	0,288	0,291	1
Clcn1	0,658313	0,004586	0,016	0,013	1
Gm44053	0,658313	0,004586	0,016	0,013	1

Zfp953	0,65852	-0,0006	0,05	0,055	1
Tcf25	0,6587	0,012445	0,963	0,971	1
Ugcg	0,658757	0,018836	0,191	0,182	1
Naa38	0,65904	-0,01026	0,573	0,585	1
Pdap1	0,659103	0,000157	0,885	0,907	1
Slitrk3	0,659255	0,007792	0,016	0,013	1
Tspoap1	0,659295	0,00332	0,025	0,021	1
Fam180a	0,659606	-0,00236	0,025	0,021	1
Psmb4	0,659805	0,009352	0,953	0,937	1
9130011E1	0,659824	0,004322	0,042	0,047	1
C330018D:	0,660207	0,009823	0,144	0,137	1
Dhx16	0,660415	0,027463	0,249	0,244	1
Git1	0,660437	-0,01517	0,838	0,852	1
Orc5	0,660456	0,004544	0,184	0,195	1
Cecr2	0,66047	-0,0036	0,077	0,083	1
Elac1	0,660588	0,015947	0,142	0,135	1
Slc25a13	0,660761	-0,01193	0,419	0,426	1
Poc5	0,660955	0,02951	0,267	0,262	1
Atxn7	0,661088	0,008352	0,242	0,231	1
A830005F2	0,661101	0,002686	0,025	0,021	1
Gm7467	0,661349	-9E-05	0,079	0,086	1
Rpl21	0,661364	0,005837	0,997	0,997	1
C87436	0,661386	0,007288	0,286	0,304	1
Arid3a	0,661478	0,015912	0,062	0,057	1
Lars2	0,661669	0,052399	0,887	0,893	1
Ufsp2	0,66173	-0,02054	0,392	0,397	1
Eif3i	0,661918	-0,00077	0,703	0,728	1
Upf3a	0,662116	0,006304	0,363	0,345	1
Cnot6l	0,662153	0,005465	0,394	0,41	1
2900097C:	0,662229	0,035266	0,823	0,826	1
Atp6v0a1	0,662272	0,013074	0,613	0,587	1
Flcn	0,662418	-0,00918	0,295	0,306	1
Lrrc57	0,662449	-0,00764	0,23	0,239	1
Rnaseh1	0,662573	0,004079	0,238	0,228	1
Mex3b	0,662641	0,021672	0,198	0,19	1
Hspb2	0,662772	-0,00012	0,074	0,08	1
Neil2	0,662829	0,002017	0,016	0,013	1
Tnfsfm13	0,662829	0,002017	0,016	0,013	1
Rhpn1	0,662829	0,002017	0,016	0,013	1
Ripk4	0,662829	0,002017	0,016	0,013	1
Chaf1a	0,662919	-0,00163	0,036	0,041	1
Topors	0,663225	0,01332	0,375	0,402	1
Pdlim1	0,663255	0,00154	0,515	0,499	1
Mknk1	0,663349	-0,00714	0,107	0,114	1
Dedd	0,663356	0,010808	0,339	0,358	1
Mre11a	0,663408	-0,00655	0,147	0,156	1
Timm17b	0,663486	-0,00761	0,403	0,415	1
Ripk2	0,663807	0,007415	0,052	0,047	1
Nt5e	0,663874	-0,00617	0,089	0,096	1
Zfp839	0,663909	0,011762	0,097	0,091	1
Plxnb2	0,66408	-0,00299	0,023	0,026	1
Harbi1	0,664115	-0,01667	0,093	0,099	1

Tesk1	0,664244	0,009286	0,21	0,202	1
Ifi35	0,664282	-0,03889	0,079	0,083	1
Mypop	0,664297	0,020035	0,134	0,128	1
Cenpk	0,664336	-0,00215	0,016	0,013	1
Immp1l	0,664621	-0,01342	0,482	0,498	1
Mapk1ip1	0,664661	0,008587	0,25	0,265	1
Brf1	0,66467	0,020072	0,32	0,311	1
Gpkow	0,664912	0,004058	0,214	0,203	1
Fkbp5	0,665026	0,006501	0,028	0,033	1
B230303O:	0,665286	0,00524	0,031	0,028	1
Slc25a32	0,665316	0,001084	0,132	0,141	1
Ube3c	0,665451	-0,00668	0,364	0,371	1
Pold3	0,66559	0,000814	0,242	0,231	1
Rab35	0,665781	-0,00744	0,292	0,302	1
Mau2	0,66619	-0,02657	0,636	0,616	1
Elp5	0,666209	0,004013	0,313	0,327	1
Ints2	0,666338	0,020619	0,132	0,127	1
Mesd	0,666431	-0,00322	0,379	0,39	1
Capza1	0,666436	0,019465	0,42	0,411	1
Tdrd3	0,666438	0,023731	0,241	0,234	1
Pdcd7	0,666598	0,018511	0,31	0,299	1
Il12rb1	0,666812	-0,01989	0,091	0,085	1
Lsm11	0,666841	-0,00766	0,052	0,057	1
Wfikkn2	0,666935	0,004616	0,038	0,034	1
BC017643	0,666955	0,001466	0,279	0,267	1
B230307C:	0,667109	-0,00173	0,175	0,184	1
Soga3	0,667113	-0,00112	0,629	0,637	1
Hp1bp3	0,66716	-0,00551	0,865	0,872	1
Ndufb7	0,667361	0,007751	0,957	0,964	1
Napepld	0,667386	0,004491	0,312	0,327	1
Fpgs	0,667388	0,009676	0,107	0,101	1
Rffl	0,667418	0,009736	0,805	0,813	1
Btrc	0,66745	-0,005	0,325	0,337	1
Polh	0,667477	-0,00336	0,02	0,023	1
Ostm1	0,66756	-0,00838	0,376	0,387	1
Ints7	0,667589	0,021138	0,415	0,411	1
Xkr6	0,667658	0,028325	0,165	0,159	1
Ankrd24	0,667784	0,012225	0,201	0,193	1
Slc25a28	0,668096	0,014652	0,364	0,358	1
Cyp20a1	0,668331	-0,00152	0,565	0,585	1
Pi4kb	0,668553	-0,01269	0,216	0,224	1
Rnase4	0,668959	0,043369	0,144	0,135	1
Pex3	0,668991	0,000981	0,385	0,403	1
Minpp1	0,669347	-0,00564	0,322	0,333	1
Wdr95	0,669382	-0,00211	0,02	0,023	1
Gapvd1	0,669523	-0,01173	0,631	0,642	1
Asb13	0,669629	-0,00356	0,086	0,093	1
R3hcc1l	0,66982	0,011868	0,238	0,255	1
Nectin2	0,66984	0,007783	0,023	0,02	1
Gm43689	0,669871	-0,00481	0,005	0,007	1
Rpgrip1	0,66991	0,002103	0,285	0,298	1
Ndor1	0,670172	-0,00413	0,071	0,076	1

Smoc1	0,670265	-0,00753	0,113	0,12	1
Snrpb2	0,670414	0,024424	0,471	0,467	1
Dcaf4	0,670425	-0,01577	0,218	0,224	1
Myo9b	0,67061	0,000594	0,314	0,327	1
Tcerg1	0,670689	-0,0232	0,389	0,393	1
S100a13	0,670764	-0,01125	0,935	0,948	1
Mindy3	0,670835	0,026744	0,485	0,489	1
Fubp3	0,670925	-0,01333	0,54	0,546	1
Pdp2	0,670943	0,025507	0,131	0,125	1
Ctsz	0,670945	-0,0038	0,751	0,767	1
Hmgxb3	0,671137	-0,01616	0,148	0,154	1
Rbm8a	0,671194	-0,00761	0,727	0,738	1
Gm15972	0,671245	0,001507	0,045	0,041	1
Gm4969	0,671409	-0,0032	0,005	0,007	1
Vmn1r218	0,671409	-0,0032	0,005	0,007	1
A230087F1	0,671409	-0,0032	0,005	0,007	1
Prlr	0,67141	-0,00282	0,005	0,007	1
Ier3ip1	0,671469	0,024347	0,641	0,644	1
Atxn7l3	0,671488	0,018934	0,32	0,311	1
Smim19	0,67152	0,021527	0,48	0,481	1
Gm15492	0,671568	-0,00403	0,017	0,02	1
2210408I2	0,67159	-0,0044	0,097	0,104	1
Ppp5c	0,6716	-0,01146	0,418	0,436	1
Pigg	0,671697	0,01073	0,132	0,125	1
Clcn7	0,671969	0,007641	0,236	0,226	1
Ccdc14	0,672054	-0,00156	0,044	0,049	1
Sirt2	0,672146	0,018304	0,999	0,998	1
Igfbp5	0,672333	-0,00866	0,005	0,007	1
Slc7a6os	0,672389	-0,00351	0,192	0,203	1
Rnf43	0,672436	0,020162	0,221	0,211	1
Ccdc12	0,672554	-0,00947	0,636	0,657	1
Gm17634	0,672641	-0,00223	0,005	0,007	1
Gm26899	0,672641	-0,00223	0,005	0,007	1
Gprc5d	0,672641	-0,00286	0,005	0,007	1
Cntd1	0,672872	0,004961	0,069	0,063	1
Alkbh4	0,6729	0,008962	0,05	0,046	1
Gm16897	0,672948	-0,00159	0,005	0,007	1
Fgf2	0,672948	-0,00159	0,005	0,007	1
Gm3764	0,672948	-0,00159	0,005	0,007	1
Gm42745	0,672948	-0,00159	0,005	0,007	1
Fam71f2	0,672948	-0,00159	0,005	0,007	1
Ccdc142	0,672948	-0,00159	0,005	0,007	1
Ccnd2	0,672948	-0,00159	0,005	0,007	1
Fbxo17	0,672948	-0,00159	0,005	0,007	1
Gm21750	0,672948	-0,00159	0,005	0,007	1
Sp6	0,672948	-0,00159	0,005	0,007	1
Gsdma	0,672948	-0,00159	0,005	0,007	1
Gm10184	0,672948	-0,00159	0,005	0,007	1
Sfrp5	0,672948	-0,00159	0,005	0,007	1
Rnf19b	0,67331	-0,02603	0,139	0,146	1
Adamts2	0,673377	0,008124	0,24	0,231	1
Ppp3cc	0,673388	0,010529	0,222	0,213	1

Cenpe	0,673509	-0,0053	0,033	0,037	1
Gabrr2	0,673525	0,002444	0,036	0,041	1
Eps8l1	0,673538	0,004336	0,057	0,052	1
Chst2	0,673542	-0,03059	0,394	0,397	1
Pi4k2a	0,673664	-0,00262	0,32	0,33	1
Gbe1	0,67368	-0,00519	0,107	0,114	1
Cyp2d22	0,673696	0,007624	0,082	0,089	1
Cdk5r1	0,673759	0,001069	0,071	0,076	1
Rps6kc1	0,673874	0,001001	0,173	0,182	1
Zfp598	0,673905	0,001755	0,207	0,218	1
Megf11	0,674156	-0,00341	0,017	0,02	1
1810041L1	0,674182	0,007162	0,005	0,007	1
4933429H	0,674182	0,000363	0,005	0,007	1
Rnf223	0,674182	0,000363	0,005	0,007	1
Tmem150b	0,674182	0,000363	0,005	0,007	1
Gm26562	0,674182	0,000363	0,005	0,007	1
1700102H	0,674182	0,000363	0,005	0,007	1
AA543186	0,674182	-0,00061	0,005	0,007	1
Lhfp	0,674182	-0,00061	0,005	0,007	1
Gm16503	0,674182	-0,00061	0,005	0,007	1
Srrm3	0,674182	-0,00061	0,005	0,007	1
Ticrr	0,674182	-0,00061	0,005	0,007	1
Olfr635	0,674182	-0,00061	0,005	0,007	1
Creb3l3	0,674182	-0,00061	0,005	0,007	1
Slmapos2	0,674182	-0,00061	0,005	0,007	1
2900052N	0,674182	-0,00061	0,005	0,007	1
Abcd2	0,674182	-0,00061	0,005	0,007	1
Dgkg	0,674182	-0,00061	0,005	0,007	1
Impg2	0,674182	-0,00061	0,005	0,007	1
Tmem39a	0,674221	0,020162	0,241	0,237	1
Add1	0,67439	-0,00458	0,9	0,888	1
Tsc22d4	0,674421	0,01949	0,998	0,997	1
Golgb1	0,67463	0,028613	0,594	0,584	1
Slc22a23	0,674777	0,005234	0,582	0,564	1
Srp9	0,675031	0,000454	0,716	0,737	1
Atrx	0,675188	-0,01408	0,901	0,906	1
Zfp655	0,675203	0,021473	0,281	0,273	1
Vmn1r43	0,675416	0,000363	0,005	0,007	1
Cyp26b1	0,675497	-4,7E-05	0,033	0,037	1
Hdgf	0,67562	0,011959	0,87	0,885	1
Gfra2	0,675725	0,00361	0,005	0,007	1
Man2a1	0,67573	0,007687	0,061	0,067	1
Pank2	0,675943	-0,01226	0,201	0,21	1
3110043O	0,676168	-0,00447	0,166	0,174	1
Lgi3	0,676219	0,002794	0,923	0,904	1
Fam135a	0,676419	0,012727	0,248	0,241	1
Rpl35a	0,676449	0,023941	0,996	0,998	1
Nudt16	0,676465	-0,01115	0,224	0,233	1
Fbxl2	0,67664	-0,01583	0,079	0,085	1
Nanos1	0,67665	0,00231	0,005	0,007	1
Coil	0,676893	0,002171	0,126	0,133	1
Kif1a	0,676917	-0,00668	0,985	0,989	1

Srgap3	0,676965	-0,01203	0,466	0,48	1
Rcc1	0,67707	-0,00343	0,107	0,114	1
Txndc9	0,677162	-0,00973	0,623	0,649	1
Efcab12	0,677243	0,004277	0,023	0,02	1
2810457G(	0,677343	-0,00702	0,03	0,034	1
2510002D:	0,677349	-0,00362	0,11	0,117	1
Ndufa1	0,677383	-0,00114	0,661	0,683	1
Atxn7l1	0,677523	0,004416	0,113	0,106	1
Pgpep1	0,677527	0,017766	0,134	0,128	1
Stk38	0,677597	-0,01118	0,317	0,332	1
Kri1	0,677617	0,014529	0,198	0,213	1
Gm26710	0,677795	-0,01536	0,017	0,02	1
Nadk2	0,677896	-0,01969	0,368	0,376	1
Snrk	0,677988	-0,0118	0,162	0,169	1
Bud31	0,678028	0,023743	0,543	0,54	1
Pls1	0,678113	-0,01164	0,93	0,922	1
Ttll7	0,678235	-0,00774	0,998	1	1
Lppos	0,678351	-0,00471	0,014	0,016	1
Sf1	0,678381	0,012605	0,657	0,67	1
Tm2d2	0,67851	-0,01331	0,764	0,761	1
Orc4	0,679053	-0,01715	0,342	0,35	1
Appbp2os	0,679109	-0,00408	0,014	0,016	1
Mcmbp	0,679204	0,024038	0,502	0,499	1
Pigk	0,679206	0,006816	0,489	0,486	1
Hist1h2ac	0,679235	-0,00699	0,011	0,013	1
Spaca6	0,679297	-0,01153	0,173	0,18	1
Gm26747	0,679299	0,00301	0,023	0,02	1
Ngrn	0,679345	-0,01036	0,162	0,169	1
6720427I0	0,679359	0,001625	0,124	0,132	1
Hist1h4c	0,679415	0,008119	0,014	0,011	1
1700056N:	0,679415	0,007156	0,014	0,011	1
H1fx	0,679657	0,006441	0,111	0,119	1
Pdik1l	0,679708	-0,01071	0,173	0,18	1
Fahd2a	0,679989	0,000164	0,168	0,177	1
Txn2	0,680157	0,022596	0,679	0,685	1
Kcnip3	0,680544	0,006504	0,029	0,026	1
Ptrh2	0,680553	0,008124	0,241	0,254	1
Gfer	0,680616	0,016302	0,24	0,233	1
Olfr655	0,680638	0,028686	0,014	0,011	1
Nob1	0,680816	0,008691	0,213	0,226	1
Mob4	0,680981	0,019081	0,54	0,533	1
AC163040.	0,681013	-0,00374	0,014	0,016	1
Lrwd1	0,681016	0,009807	0,144	0,137	1
Asb8	0,681524	-0,0108	0,341	0,346	1
Caml	0,681535	0,018146	0,469	0,463	1
Zkscan17	0,681544	0,013175	0,102	0,096	1
Dhdds	0,681578	0,017292	0,357	0,35	1
Bik	0,681608	-0,00432	0,017	0,02	1
Gm27010	0,681632	0,013804	0,093	0,088	1
Dera	0,681673	0,009626	0,12	0,114	1
Gm15283	0,681773	-0,00248	0,014	0,016	1
Ppp1r7	0,681849	0,019078	0,479	0,468	1

Mir17hg	0,681953	-0,00671	0,008	0,01	1
Jrk	0,682195	-0,00537	0,065	0,07	1
Fam186a	0,682263	0,002655	0,014	0,011	1
Emilin1	0,682441	-0,00727	0,011	0,013	1
Prkra	0,682621	0,00823	0,21	0,202	1
Armcx6	0,682644	0,008307	0,067	0,062	1
Stard6	0,682758	0,005554	0,029	0,026	1
Fbrs	0,682769	0,008883	0,318	0,306	1
Mki67	0,682777	0,001098	0,023	0,02	1
Nudt2	0,682781	0,015206	0,321	0,312	1
Ap2a2	0,682849	-0,01373	0,595	0,6	1
Pafah1b3	0,68292	-0,00215	0,014	0,016	1
Tex10	0,682987	0,009144	0,291	0,309	1
Edc4	0,683065	0,005614	0,219	0,233	1
Rtl8b	0,683134	-0,00328	0,737	0,754	1
Uxt	0,683258	0,006883	0,237	0,228	1
Taf1a	0,683284	0,007913	0,107	0,101	1
Tgoln1	0,683331	-0,0055	0,894	0,914	1
4930563J1	0,683446	-0,00413	0,008	0,01	1
Vmn2r95	0,683446	-0,00413	0,008	0,01	1
Coa3	0,683555	-0,00644	0,736	0,763	1
Stk39	0,683631	0,006114	0,767	0,792	1
Gm20457	0,68368	-0,00152	0,014	0,016	1
B230217O	0,683687	0,001689	0,014	0,011	1
Atic	0,683772	0,003297	0,297	0,312	1
Gemin4	0,683935	-0,00539	0,011	0,013	1
Tmppe	0,683943	-0,0035	0,008	0,01	1
Ppp1r18	0,683943	-0,0035	0,008	0,01	1
Prdm11	0,683944	-0,00316	0,008	0,01	1
Cnot2	0,68396	0,039794	0,369	0,369	1
Tmbim4	0,683971	0,025053	0,772	0,772	1
Elovl4	0,684036	-0,01196	0,153	0,161	1
Zfp975	0,684094	-0,00404	0,049	0,054	1
Rabl6	0,684139	-0,01411	0,405	0,413	1
Ndufa4	0,684253	0,001618	0,973	0,977	1
Cul1	0,684415	-0,00604	0,667	0,681	1
Ciao1	0,68452	0,01	0,326	0,348	1
Dnal1	0,684741	-0,00568	0,169	0,177	1
Dtx3	0,684751	0,031772	0,476	0,476	1
Snx12	0,684908	-0,0089	0,233	0,241	1
Homer3	0,684988	0,016305	0,3	0,294	1
Psmc6	0,685044	0,007707	0,824	0,834	1
Zfp423	0,685112	0,000721	0,014	0,011	1
Tube1	0,685114	0,003653	0,029	0,026	1
Wnt4	0,685115	0,001052	0,014	0,011	1
Asf1a	0,685197	-0,00687	0,388	0,4	1
Siae	0,68522	-0,01046	0,16	0,167	1
Lnpk	0,685222	-0,01028	0,526	0,53	1
Hnrnpa1	0,685226	0,005104	0,827	0,824	1
Fbxl3	0,685259	0,025749	0,756	0,774	1
Ccl27a	0,685279	0,004	0,043	0,039	1
Noc2l	0,685338	0,021513	0,314	0,307	1

Sp7	0,685381	0,007249	0,152	0,145	1
Gm16751	0,685436	-0,00152	0,011	0,013	1
Dhrs11	0,685436	-0,00248	0,011	0,013	1
1500002C:	0,685438	-0,00252	0,008	0,01	1
Gm16754	0,685438	-0,00252	0,008	0,01	1
Hoxa1	0,685589	0,002345	0,014	0,016	1
Timm10b	0,685599	-0,0042	0,603	0,608	1
Dnajc14	0,685607	-0,01004	0,328	0,333	1
Ercc4	0,685681	0,01014	0,18	0,19	1
Gm16973	0,685773	-0,00077	0,063	0,068	1
Actr2	0,685779	-0,0108	0,756	0,771	1
Dpp6	0,685784	-0,00052	0,014	0,016	1
Srsf11	0,685799	-0,01171	0,887	0,909	1
Sac3d1	0,685799	-0,00376	0,155	0,164	1
AC124739.	0,685935	-0,00189	0,008	0,01	1
Gm26513	0,685935	-0,00189	0,008	0,01	1
Nrn1l	0,686021	0,005571	0,082	0,076	1
Gm48882	0,68629	-0,00219	0,011	0,013	1
Tiam2	0,68629	-0,00219	0,011	0,013	1
Nr2f1	0,686357	0,000113	0,014	0,016	1
Ptges2	0,686435	0,001701	0,27	0,281	1
Gm26545	0,686685	-0,00219	0,008	0,01	1
Cspg4	0,686722	-0,00248	0,011	0,013	1
Ss18l1	0,686823	0,008825	0,097	0,091	1
Usp21	0,686838	0,014774	0,183	0,176	1
Tmem167b	0,686909	0,002429	0,333	0,322	1
3100002H(	0,686929	0,001709	0,014	0,016	1
4930532G:	0,686934	-0,00155	0,008	0,01	1
Tal2	0,686934	-0,00155	0,008	0,01	1
Rnf157	0,68694	-0,008	0,177	0,184	1
Ncor1	0,687072	0,00809	0,909	0,906	1
Fuk	0,687119	-0,00171	0,068	0,073	1
Ttf2	0,687183	0,00039	0,008	0,01	1
Slc39a14	0,68721	0,034688	0,209	0,203	1
Me1	0,687265	0,006405	0,459	0,446	1
Mmp2	0,687352	0,006183	0,036	0,033	1
Gm28230	0,687364	-0,00152	0,011	0,013	1
Socs5	0,687399	-0,00252	0,287	0,301	1
Kcnd1	0,687432	0,002968	0,008	0,01	1
Gm16386	0,687432	5,69E-05	0,008	0,01	1
Itga3	0,687432	-0,00092	0,008	0,01	1
Npff	0,687433	0,002655	0,008	0,01	1
Bcl7b	0,687436	-0,01243	0,548	0,569	1
Zfp623	0,687452	0,010994	0,077	0,072	1
Tulp2	0,687579	-0,00088	0,011	0,013	1
Slc35a4	0,687779	0,003092	0,445	0,465	1
Bscl2	0,687847	0,021431	0,382	0,376	1
Krt28	0,687969	-0,00088	0,014	0,011	1
1810062O:	0,688006	-0,00122	0,011	0,013	1
Hyls1	0,688006	-0,00122	0,011	0,013	1
Zfp808	0,688006	-0,00122	0,011	0,013	1
Gosr1	0,688127	-0,01237	0,431	0,441	1

Gls	0,688281	0,029216	0,525	0,517	1
Ube2r2	0,688315	0,012853	0,621	0,62	1
Clcc1	0,688492	-0,01845	0,368	0,372	1
Acot7	0,688639	-0,00776	0,942	0,933	1
Ints13	0,688751	0,01358	0,28	0,273	1
Lrp3	0,688929	0,001999	0,008	0,01	1
Ubr1	0,689231	0,022943	0,47	0,476	1
Schip1	0,689333	0,01992	0,36	0,38	1
Gm10643	0,689413	0,002345	0,014	0,016	1
Cdk5r2	0,689724	-0,00025	0,011	0,013	1
Slc26a6	0,689724	0,000721	0,011	0,013	1
Cacul1	0,68988	0,022117	0,389	0,384	1
Mad1l1	0,690125	0,004451	0,297	0,309	1
Rad51ap1	0,690428	0,006837	0,008	0,01	1
Serinc3	0,690541	-0,00892	0,913	0,92	1
Snip1	0,690552	-0,01107	0,174	0,18	1
Ankrd49	0,690634	-0,00325	0,36	0,371	1
Sec62	0,690648	-0,00056	0,998	0,997	1
Luc7l3	0,69085	-0,00509	0,928	0,93	1
Sucla2	0,691031	-0,00357	0,425	0,444	1
Fxyd7	0,691146	-0,13779	0,116	0,12	1
Ccdc124	0,69151	-0,02381	0,6	0,592	1
Ms4a6c	0,69152	0,008424	0,021	0,018	1
Slx4ip	0,691706	0,017715	0,132	0,127	1
Cers2	0,691776	-0,01172	0,996	0,998	1
Junos	0,691928	0,001999	0,008	0,01	1
Phykpl	0,691993	0,019799	0,139	0,133	1
Tmem41a	0,69211	0,011064	0,065	0,06	1
Carm1	0,692352	0,011368	0,214	0,207	1
Coro1b	0,692405	0,012033	0,454	0,447	1
Mrpl12	0,692419	0,01957	0,58	0,579	1
Stk38l	0,692463	-0,00812	0,195	0,203	1
Mllt11	0,692503	0,013839	0,167	0,161	1
Tysnd1	0,692596	0,005095	0,237	0,226	1
Dnttip1	0,692917	0,022725	0,336	0,332	1
Crebzf	0,692971	0,013892	0,644	0,676	1
Abcd4	0,693091	-0,00877	0,286	0,296	1
Scrib	0,69312	0,002368	0,306	0,32	1
Ttc19	0,693217	0,01957	0,32	0,314	1
Adipor1	0,693464	0,007658	0,804	0,828	1
Mecp2	0,693696	0,028691	0,696	0,68	1
AI837181	0,69389	0,025874	0,365	0,363	1
Dhx34	0,693954	0,008231	0,1	0,094	1
Zfp605	0,694126	0,005871	0,187	0,179	1
Cog3	0,694237	-0,00596	0,214	0,223	1
Tmem229b	0,694331	-0,00259	0,073	0,078	1
Armc9	0,694372	0,019975	0,143	0,138	1
Tmco1	0,694433	0,021078	0,729	0,74	1
Mettl21a	0,694477	0,011097	0,115	0,109	1
Wls	0,694681	0,021458	0,78	0,79	1
1110051M	0,694822	0,008684	0,307	0,325	1
Trim62	0,694886	0,010427	0,065	0,06	1

Sspn	0,694906	0,015912	0,478	0,47	1
Tmem256	0,694964	0,01435	0,948	0,945	1
Rab3b	0,69504	-0,00884	0,204	0,211	1
Hmces	0,695107	0,004223	0,167	0,159	1
Btbd9	0,69538	-0,0006	0,375	0,385	1
Dtnb	0,695392	0,01233	0,103	0,098	1
Fam96a	0,695399	0,001739	0,408	0,428	1
Fip1l1	0,695563	0,019359	0,653	0,657	1
Ythdc1	0,695827	0,018342	0,71	0,709	1
Pcdhgb1	0,695849	0,002686	0,021	0,018	1
Sec22c	0,696001	-0,01108	0,129	0,135	1
Mrpl39	0,696068	-0,00679	0,224	0,233	1
Nkain2	0,696162	-0,00043	0,858	0,849	1
9130401M	0,696184	-0,00354	0,298	0,309	1
Stxbp3	0,696223	-0,00317	0,919	0,904	1
C030006K:	0,69641	0,012222	0,157	0,151	1
Zfp563	0,696426	-0,00596	0,085	0,091	1
Lrrc4b	0,696527	0,028527	0,818	0,811	1
Zfp229	0,696539	-0,00446	0,035	0,039	1
Dnaja3	0,696704	0,005441	0,378	0,397	1
Rbm12b1	0,696867	0,007309	0,122	0,115	1
Eif1	0,696916	-0,00697	1	1	1
Map2k1	0,697038	0,000994	0,541	0,556	1
Med20	0,697074	0,000286	0,269	0,278	1
Plekhh1	0,697095	-0,022	0,97	0,972	1
Aatf	0,697295	0,022558	0,293	0,291	1
Tcf3	0,697449	0,007403	0,36	0,377	1
Wac	0,697516	-0,00609	0,648	0,665	1
Hk1	0,697519	0,030828	0,289	0,285	1
Zfp551	0,697538	0,007444	0,034	0,031	1
Psmd7	0,697607	0,018058	0,883	0,876	1
Zfp748	0,697656	0,012068	0,183	0,176	1
Tmem165	0,69766	0,010777	0,522	0,514	1
Bfar	0,697756	0,026429	0,387	0,384	1
Rap2c	0,697778	0,001092	0,237	0,247	1
Cep250	0,69787	0,024215	0,199	0,213	1
Brf2	0,697873	0,000261	0,143	0,151	1
Aarsd1	0,698142	-0,00514	0,111	0,117	1
Ndufa3	0,698178	-0,00063	0,984	0,985	1
Pef1	0,698188	-0,00818	0,342	0,353	1
Rfesd	0,698286	-0,00935	0,161	0,167	1
Clk3	0,698406	0,009349	0,309	0,324	1
Llph	0,698661	-0,01689	0,582	0,585	1
Selenbp1	0,698663	-0,00276	0,235	0,244	1
Zkscan6	0,698668	-0,00093	0,088	0,094	1
Pdxdc1	0,698739	-0,00063	0,68	0,691	1
Ppm1d	0,698937	-0,00654	0,254	0,239	1
Ccdc138	0,699069	0,004343	0,055	0,05	1
Rpl17	0,6991	-0,01049	0,986	0,995	1
Tgfbrap1	0,699239	-0,00905	0,344	0,353	1
Zfp30	0,699286	-0,00272	0,137	0,145	1
Cst6	0,699352	0,008098	0,021	0,018	1

Asl	0,699366	0,005294	0,115	0,109	1
Daglb	0,699688	0,002	0,325	0,34	1
Gm4631	0,699964	-0,0001	0,131	0,124	1
Arpp19	0,700022	-0,00894	0,94	0,93	1
Bag4	0,700106	-0,00701	0,311	0,322	1
Fars2	0,700137	0,016803	0,203	0,197	1
Nqo2	0,700642	0,003982	0,034	0,031	1
Kdm1a	0,700696	-0,00308	0,504	0,528	1
Ankrd9	0,700973	-0,00441	0,07	0,075	1
Atg16l1	0,700994	0,001739	0,314	0,332	1
Tex9	0,701154	-0,00642	0,111	0,117	1
Nup35	0,701506	0,006111	0,282	0,294	1
Rnf32	0,701569	-0,01116	0,077	0,081	1
Prpf31	0,701617	-0,00473	0,379	0,389	1
Grb2	0,701732	0,022746	0,74	0,737	1
Zfp532	0,701881	-0,04014	0,559	0,545	1
Fam161a	0,701957	-0,01231	0,064	0,068	1
Pgm1	0,70198	0,014094	0,058	0,063	1
Opalin	0,702034	0,026777	0,782	0,743	1
Gm15510	0,702307	0,003937	0,012	0,01	1
Pcp4l1	0,702307	0,004904	0,012	0,01	1
Dnajc13	0,702342	0,00133	0,347	0,361	1
AI504432	0,702383	-0,01168	0,267	0,272	1
Arid5b	0,702574	0,036796	0,722	0,741	1
Pfdn1	0,702663	0,000447	0,638	0,676	1
Phlpp2	0,702719	-0,00294	0,114	0,12	1
Tdrd7	0,702781	0,004158	0,255	0,267	1
Ythdf1	0,702783	-0,00453	0,389	0,398	1
Tmx1	0,702886	-0,00843	0,418	0,426	1
Rnf146	0,702914	0,016282	0,488	0,488	1
Supt3	0,703336	-0,00788	0,312	0,324	1
Vps13d	0,703634	0,00563	0,264	0,278	1
Sap30	0,703639	0,002968	0,012	0,01	1
2610035D	0,703736	-0,00351	0,238	0,247	1
Slc25a4	0,703874	0,012067	0,996	0,997	1
Cbx1	0,703991	0,00867	0,695	0,685	1
D030056L	0,704174	-0,00647	0,367	0,374	1
Cox7a1	0,704193	0,001075	0,021	0,018	1
Lrrc8b	0,704253	0,003473	0,811	0,821	1
Kcna2	0,704475	0,029769	0,217	0,211	1
Zdhhc23	0,704507	-0,01264	0,077	0,081	1
Il10rb	0,704529	0,004262	0,012	0,01	1
Lgi4	0,704535	0,000612	0,239	0,229	1
Ccl25	0,704606	-0,01468	0,117	0,122	1
Zfp87	0,704732	-0,00182	0,164	0,154	1
Wdr31	0,704751	0,003295	0,012	0,01	1
Prdx3	0,704798	-0,00509	0,533	0,537	1
Kdsr	0,704959	-0,00206	0,683	0,706	1
Grhl1	0,704971	0,001999	0,012	0,01	1
Etnk1	0,705106	0,007606	0,926	0,915	1
Rab13	0,70525	0,005566	0,065	0,06	1
Ccdc152	0,705478	-0,01865	0,17	0,176	1

Uros	0,705678	0,012006	0,153	0,146	1
Erh	0,705779	0,019026	0,57	0,569	1
Sec22a	0,705953	-0,00731	0,237	0,244	1
Gm26764	0,705992	-0,01506	0,032	0,036	1
Cox20	0,706017	0,021635	0,436	0,434	1
Rab3gap1	0,706128	0,019741	0,472	0,465	1
Gm13166	0,706307	0,001359	0,012	0,01	1
Gm6994	0,706307	0,001359	0,012	0,01	1
Sgcb	0,706599	0,027483	0,663	0,649	1
Stard4	0,706682	0,029791	0,289	0,283	1
Arl14ep	0,706958	-0,00765	0,283	0,291	1
Zmat2	0,707066	-0,01013	0,868	0,876	1
Arl10	0,707176	-0,00199	0,106	0,112	1
Zswim7	0,707446	0,014632	0,194	0,189	1
Tldc1	0,707705	-0,00034	0,046	0,05	1
Slc25a20	0,707888	0,009851	0,052	0,057	1
Znrf1	0,707907	0,006531	0,34	0,35	1
Crip1	0,708064	-0,00441	0,148	0,141	1
Tyw1	0,708611	-0,02111	0,273	0,276	1
Nsd2	0,708806	-0,00263	0,5	0,509	1
Chchd6	0,708849	-0,00619	0,24	0,249	1
Frrs1l	0,708854	0,004336	0,053	0,049	1
Cmtm6	0,708879	-0,01194	0,272	0,278	1
Arl2	0,708912	-0,00136	0,966	0,961	1
BC024063	0,708961	-0,00508	0,032	0,036	1
Bcl9l	0,708972	0,012847	0,449	0,476	1
Dnajb9	0,709015	-0,00336	0,489	0,468	1
Rabggta	0,709168	-0,00084	0,222	0,231	1
Samd4	0,70982	0,030812	0,378	0,379	1
Tmem205	0,709911	-0,00922	0,676	0,678	1
Bcap29	0,710044	-0,0007	0,315	0,325	1
Kcnma1	0,710115	0,029085	0,193	0,189	1
Unc93b1	0,710382	-0,01006	0,156	0,163	1
Mta1	0,710448	-0,02017	0,294	0,299	1
Tmco6	0,710462	0,010281	0,158	0,151	1
Gm37305	0,710679	0,010312	0,026	0,023	1
Fbxo21	0,710822	0,004314	0,688	0,701	1
Zfp51	0,711116	-0,00354	0,067	0,072	1
Ints4	0,711118	0,00305	0,356	0,369	1
Marc2	0,711129	0,006957	0,529	0,515	1
Syvn1	0,711223	0,016229	0,289	0,283	1
Rab11fip2	0,711418	0,004748	0,594	0,608	1
Serpinc1	0,711525	0,000519	0,027	0,024	1
Gnao1	0,711594	0,001641	0,998	0,998	1
Rad50	0,71182	0,01161	0,245	0,259	1
Bub3	0,711834	-0,00688	0,86	0,855	1
Creg1	0,711919	-0,00161	0,321	0,33	1
Epb41l5	0,712121	0,007747	0,039	0,036	1
Ift172	0,712175	-0,00782	0,168	0,174	1
Vars	0,712402	0,006872	0,248	0,239	1
Lmf2	0,712452	0,019937	0,297	0,293	1
Hmgb1	0,712639	0,004212	0,997	0,998	1

Gmeb2	0,712736	-0,00111	0,14	0,148	1
Faxc	0,712739	0,000913	0,046	0,05	1
Arhgap12	0,712777	0,022678	0,271	0,286	1
Mxd4	0,712862	0,003133	0,815	0,823	1
Stk16	0,713173	-0,01894	0,473	0,468	1
Fmn1	0,713205	-0,01644	0,245	0,234	1
Mcee	0,713506	0,025221	0,593	0,602	1
6330562C2	0,71356	0,010325	0,091	0,086	1
Abhd17b	0,713693	0,001894	0,831	0,847	1
Tm2d3	0,713721	-0,00222	0,394	0,407	1
Mrpl22	0,71376	-0,00433	0,229	0,237	1
Plvap	0,713782	0,002171	0,09	0,096	1
Fam219a	0,713819	0,025205	0,449	0,455	1
Kdelc1	0,713846	-0,01401	0,101	0,106	1
Zfp189	0,71396	-0,00996	0,061	0,065	1
Smarcc2	0,714169	0,013667	0,746	0,745	1
Zfp280c	0,714263	-0,00687	0,492	0,499	1
Snapc5	0,714445	0,018934	0,569	0,571	1
Fbxl16	0,714623	-0,00295	0,032	0,036	1
Lockd	0,714896	0,008411	0,026	0,023	1
Chd8	0,715268	-0,01805	0,579	0,569	1
Zbtb49	0,715311	-0,00386	0,059	0,063	1
Zc3h6	0,715348	-0,0002	0,176	0,185	1
Smarca2	0,715385	0,0233	0,616	0,647	1
Chchd1	0,715587	-0,0093	0,693	0,704	1
Toe1	0,715659	-0,01566	0,181	0,187	1
Setbp1	0,715706	-0,01075	0,153	0,159	1
Phf20	0,715963	0,016776	0,693	0,694	1
Ppil1	0,715991	-0,00568	0,188	0,197	1
Tmco3	0,716056	0,018108	0,6	0,605	1
Lrrc40	0,716189	-0,00249	0,119	0,125	1
Gltp	0,716222	-0,0039	0,996	1	1
Rpp38	0,716387	0,005292	0,111	0,106	1
Lix1	0,71639	-0,00049	0,022	0,024	1
Zfp493	0,716463	0,00556	0,046	0,042	1
Kansl1	0,716632	-0,01431	0,522	0,527	1
Myc	0,716639	0,018072	0,058	0,054	1
Depdc1b	0,716642	0,00773	0,051	0,047	1
Mipol1	0,716907	-0,00152	0,194	0,203	1
Smg7	0,717142	0,001925	0,379	0,392	1
Armcx4	0,71724	0,01369	0,032	0,029	1
Pcyt1a	0,717251	-0,02417	0,448	0,46	1
Arl5b	0,717283	0,012407	0,152	0,161	1
Cox7c	0,717292	0,002162	0,998	0,998	1
Echs1	0,717515	0,005325	0,824	0,857	1
Klhl18	0,717578	0,002387	0,24	0,25	1
Vps37a	0,717687	0,004932	0,466	0,483	1
Camk2g	0,717725	0,003668	0,147	0,154	1
Gm10244	0,717961	-0,00207	0,022	0,024	1
Spen	0,718074	0,010187	0,514	0,511	1
Lmx1b	0,71812	-0,00227	0,001	0,002	1
Rnf138rt1	0,71812	-0,00227	0,001	0,002	1

Gm12968	0,71812	-0,00227	0,001	0,002	1
Steap2	0,71812	-0,00227	0,001	0,002	1
Wnt7a	0,71812	-0,00227	0,001	0,002	1
Fam19a1	0,71812	-0,00227	0,001	0,002	1
Fam189a1	0,71812	-0,00227	0,001	0,002	1
Rhobtb1	0,71812	-0,00227	0,001	0,002	1
Plxnc1	0,71812	-0,00227	0,001	0,002	1
Cbfa2t3	0,71812	-0,00227	0,001	0,002	1
Slc35f4	0,71812	-0,00227	0,001	0,002	1
Cxcl16	0,71812	-0,00227	0,001	0,002	1
Haspin	0,71812	-0,00227	0,001	0,002	1
Zfp184	0,71812	-0,00227	0,001	0,002	1
Gm1604a	0,71812	-0,00227	0,001	0,002	1
Entpd1	0,71812	-0,00227	0,001	0,002	1
Sycp2	0,71812	-0,00389	0,001	0,002	1
Pi15	0,71812	-0,0055	0,001	0,002	1
Mrps11	0,718143	-0,0053	0,321	0,33	1
Adcy3	0,718166	0,006186	0,032	0,029	1
Cd164	0,718264	0,009955	0,504	0,528	1
Fbxw2	0,718507	0,017375	0,397	0,389	1
Gpn2	0,718574	0,004037	0,208	0,218	1
Ssr3	0,718645	0,009874	0,907	0,894	1
Casp8	0,718788	-0,00064	0,001	0,002	1
Gm973	0,718788	-0,00064	0,001	0,002	1
Cntnap5a	0,718788	-0,00064	0,001	0,002	1
Celrr	0,718788	-0,00064	0,001	0,002	1
Dyrk3	0,718788	-0,00064	0,001	0,002	1
Rcsd1	0,718788	-0,00064	0,001	0,002	1
Fcgr3	0,718788	-0,00064	0,001	0,002	1
Ass1	0,718788	-0,00064	0,001	0,002	1
Sp5	0,718788	-0,00064	0,001	0,002	1
Spi1	0,718788	-0,00064	0,001	0,002	1
Rhov	0,718788	-0,00064	0,001	0,002	1
Syndig1	0,718788	-0,00064	0,001	0,002	1
Mmp24	0,718788	-0,00064	0,001	0,002	1
Kcns1	0,718788	-0,00064	0,001	0,002	1
Dok5	0,718788	-0,00064	0,001	0,002	1
Pnma3	0,718788	-0,00064	0,001	0,002	1
Tmem164	0,718788	-0,00064	0,001	0,002	1
Chrdl1	0,718788	-0,00064	0,001	0,002	1
Gm37350	0,718788	-0,00064	0,001	0,002	1
9330121J(	0,718788	-0,00064	0,001	0,002	1
Iqgap3	0,718788	-0,00064	0,001	0,002	1
Hmgcs2	0,718788	-0,00064	0,001	0,002	1
Bdh2	0,718788	-0,00064	0,001	0,002	1
Gem	0,718788	-0,00064	0,001	0,002	1
Mfap2	0,718788	-0,00064	0,001	0,002	1
Gm13052	0,718788	-0,00064	0,001	0,002	1
Pdpn	0,718788	-0,00064	0,001	0,002	1
9630001P:	0,718788	-0,00064	0,001	0,002	1
Sod3	0,718788	-0,00064	0,001	0,002	1
8030423F2	0,718788	-0,00064	0,001	0,002	1

Antxr2	0,718788	-0,00064	0,001	0,002	1
Galnt9	0,718788	-0,00064	0,001	0,002	1
Cryba4	0,718788	-0,00064	0,001	0,002	1
Sez6l	0,718788	-0,00064	0,001	0,002	1
5930412G1	0,718788	-0,00064	0,001	0,002	1
Flt1	0,718788	-0,00064	0,001	0,002	1
1700003E1	0,718788	-0,00064	0,001	0,002	1
Hrh1	0,718788	-0,00064	0,001	0,002	1
Cracr2a	0,718788	-0,00064	0,001	0,002	1
Pou2f2	0,718788	-0,00064	0,001	0,002	1
Sptbn4	0,718788	-0,00064	0,001	0,002	1
Hapln3	0,718788	-0,00064	0,001	0,002	1
P4ha3	0,718788	-0,00064	0,001	0,002	1
Hs3st4	0,718788	-0,00064	0,001	0,002	1
Il21r	0,718788	-0,00064	0,001	0,002	1
Fank1	0,718788	-0,00064	0,001	0,002	1
6430531B1	0,718788	-0,00064	0,001	0,002	1
Esr1	0,718788	-0,00064	0,001	0,002	1
E030030I0	0,718788	-0,00064	0,001	0,002	1
Hs3st5	0,718788	-0,00064	0,001	0,002	1
Vsir	0,718788	-0,00064	0,001	0,002	1
Gm867	0,718788	-0,00064	0,001	0,002	1
Plk5	0,718788	-0,00064	0,001	0,002	1
Nmrk2	0,718788	-0,00064	0,001	0,002	1
Myo1a	0,718788	-0,00064	0,001	0,002	1
Vegfc	0,718788	-0,00064	0,001	0,002	1
Cyp4f18	0,718788	-0,00064	0,001	0,002	1
Mast1	0,718788	-0,00064	0,001	0,002	1
Osgin1	0,718788	-0,00064	0,001	0,002	1
Dbndd1	0,718788	-0,00064	0,001	0,002	1
Fam89a	0,718788	-0,00064	0,001	0,002	1
D830030K1	0,718788	-0,00064	0,001	0,002	1
Gprin2	0,718788	-0,00064	0,001	0,002	1
AI593442	0,718788	-0,00064	0,001	0,002	1
Plscr1	0,718788	-0,00064	0,001	0,002	1
Nme9	0,718788	-0,00064	0,001	0,002	1
Sema3f	0,718788	-0,00064	0,001	0,002	1
Ttc21a	0,718788	-0,00064	0,001	0,002	1
Ikzf1	0,718788	-0,00064	0,001	0,002	1
Grb10	0,718788	-0,00064	0,001	0,002	1
Bcl11a	0,718788	-0,00064	0,001	0,002	1
Fbll1	0,718788	-0,00064	0,001	0,002	1
Slc22a4	0,718788	-0,00064	0,001	0,002	1
P4ha2	0,718788	-0,00064	0,001	0,002	1
Kcnab3	0,718788	-0,00064	0,001	0,002	1
Dnah2	0,718788	-0,00064	0,001	0,002	1
Bcl6b	0,718788	-0,00064	0,001	0,002	1
Ccl5	0,718788	-0,00064	0,001	0,002	1
Ccl9	0,718788	-0,00064	0,001	0,002	1
Top2a	0,718788	-0,00064	0,001	0,002	1
Tns4	0,718788	-0,00064	0,001	0,002	1
Pycr1	0,718788	-0,00064	0,001	0,002	1

Trpc7	0,718788	-0,00064	0,001	0,002	1
Atp6ap1l	0,718788	-0,00064	0,001	0,002	1
Bhmt	0,718788	-0,00064	0,001	0,002	1
Cd180	0,718788	-0,00064	0,001	0,002	1
Itga1	0,718788	-0,00064	0,001	0,002	1
Lrfn5	0,718788	-0,00064	0,001	0,002	1
Gpr135	0,718788	-0,00064	0,001	0,002	1
Ppp1r36	0,718788	-0,00064	0,001	0,002	1
Ccdc177	0,718788	-0,00064	0,001	0,002	1
Acot4	0,718788	-0,00064	0,001	0,002	1
4732463B(	0,718788	-0,00064	0,001	0,002	1
Syndig1l	0,718788	-0,00064	0,001	0,002	1
Gpr65	0,718788	-0,00064	0,001	0,002	1
Plcxd3	0,718788	-0,00064	0,001	0,002	1
Csf2rb2	0,718788	-0,00064	0,001	0,002	1
Pnpla3	0,718788	-0,00064	0,001	0,002	1
Panx2	0,718788	-0,00064	0,001	0,002	1
Lrrk2	0,718788	-0,00064	0,001	0,002	1
Dhh	0,718788	-0,00064	0,001	0,002	1
Ece2	0,718788	-0,00064	0,001	0,002	1
Cd200r4	0,718788	-0,00064	0,001	0,002	1
Baiap3	0,718788	-0,00064	0,001	0,002	1
H2-Eb1	0,718788	-0,00064	0,001	0,002	1
Pcdhac2	0,718788	-0,00064	0,001	0,002	1
Npas4	0,718788	-0,00064	0,001	0,002	1
Prkg1	0,718788	-0,00064	0,001	0,002	1
Tctex1d4	0,718788	-0,00129	0,001	0,002	1
Rad51ap2	0,718788	-0,00129	0,001	0,002	1
Fam117a	0,718944	0,021857	0,122	0,117	1
Eif4e	0,718981	0,02261	0,56	0,558	1
1110020A	0,719026	0,011386	0,056	0,052	1
Fam78b	0,719119	0,003955	0,019	0,016	1
Socs7	0,719165	-0,00241	0,17	0,177	1
Osbp	0,719417	-0,01176	0,483	0,493	1
Col3a1	0,719456	0,007169	0,001	0,002	1
Mir670hg	0,719456	0,002293	0,001	0,002	1
Lmo2	0,719456	0,002293	0,001	0,002	1
Pde7b	0,719456	0,002293	0,001	0,002	1
Lamb1	0,719456	0,002293	0,001	0,002	1
Rai2	0,719456	0,000336	0,001	0,002	1
4930553P	0,719456	0,000336	0,001	0,002	1
Mmp19	0,719456	0,000336	0,001	0,002	1
Sgcd	0,719456	0,000336	0,001	0,002	1
N4bp3	0,719456	0,000336	0,001	0,002	1
Gm31946	0,719456	0,000336	0,001	0,002	1
Ankrd34b	0,719456	0,000336	0,001	0,002	1
Myo1f	0,719456	0,000336	0,001	0,002	1
C230072F	0,719456	0,000336	0,001	0,002	1
Sptbn2	0,719456	0,000336	0,001	0,002	1
Blnk	0,719456	0,000336	0,001	0,002	1
Necab1	0,719456	-0,00031	0,001	0,002	1
Zfp968	0,719491	-0,00286	0,04	0,044	1

Wdr45b	0,719645	0,008647	0,589	0,579	1
A930015D(	0,719685	0,002054	0,022	0,024	1
Ecm1	0,719685	0,001098	0,022	0,024	1
Zfp664	0,719833	0,017971	0,682	0,67	1
Farsb	0,71984	-0,01564	0,294	0,299	1
Eif3g	0,719858	0,003881	0,761	0,78	1
Kctd5	0,719972	0,014921	0,262	0,257	1
Inppl1	0,720021	0,000207	0,208	0,216	1
Fam71e1	0,720024	0,004026	0,058	0,054	1
Prkar1a	0,720129	-0,01337	0,974	0,972	1
2700046A(	0,720195	-0,02167	0,179	0,185	1
Zbtb6	0,720291	0,001893	0,246	0,255	1
Eif4e2	0,720403	0,024019	0,425	0,434	1
Jmjd4	0,720417	0,003991	0,039	0,036	1
Zfp235	0,7205	-0,0055	0,1	0,094	1
Gm16536	0,720689	0,006736	0,12	0,114	1
Enox1	0,720782	0,001329	0,387	0,405	1
Fxn	0,720938	0,014478	0,087	0,083	1
Cltb	0,721211	0,023592	0,848	0,863	1
Gm13031	0,721244	0,005871	0,019	0,016	1
Focad	0,721295	-0,00814	0,102	0,107	1
Sh3bp5l	0,721307	0,004764	0,361	0,353	1
Aprt	0,721319	0,005871	0,166	0,176	1
Ranbp9	0,721422	-0,00346	0,356	0,366	1
Lrrc51	0,721575	-0,00105	0,077	0,072	1
Tmem65	0,721643	0,001102	0,509	0,496	1
Gm37065	0,721773	0,003632	0,019	0,016	1
AI182371	0,721773	0,003632	0,019	0,016	1
Bbs9	0,721921	-0,00249	0,113	0,119	1
Gfpt1	0,721949	-0,01431	0,399	0,41	1
Creb5	0,721953	-0,017	0,857	0,857	1
Llgl1	0,722294	0,017629	0,714	0,737	1
Mmp14	0,722531	0,014223	0,01	0,008	1
Zscan20	0,722689	0,00076	0,075	0,08	1
Zfp811	0,722879	-0,0004	0,032	0,029	1
Mta2	0,723009	0,017257	0,2	0,195	1
Pus1	0,723019	0,005316	0,155	0,148	1
Atg4d	0,72363	-0,01124	0,386	0,393	1
Zfp667	0,72376	-0,00891	0,061	0,065	1
Bambi	0,723838	0,007064	0,073	0,068	1
Osbp2	0,723862	0,003458	0,326	0,343	1
Zswim8	0,723902	-0,02058	0,417	0,424	1
Tdp1	0,724127	0,00305	0,135	0,143	1
Ubox5	0,724145	0,002211	0,065	0,06	1
Dzip1	0,724258	0,011992	0,106	0,101	1
Gm26808	0,724259	-0,00356	0,029	0,033	1
Pcdhga4	0,724407	0,003964	0,022	0,024	1
Dcdc2b	0,724416	-0,00474	0,037	0,041	1
Ppil4	0,724458	0,005871	0,393	0,405	1
Ndufaf7	0,7247	0,017297	0,276	0,27	1
Slc19a2	0,724874	0,009925	0,032	0,036	1
Hmgn3	0,724924	0,003476	0,27	0,28	1

Cherp	0,724989	0,023428	0,274	0,27	1
5033406O(	0,725032	0,003356	0,032	0,029	1
Ccdc82	0,725304	0,024716	0,228	0,242	1
Arl6	0,725326	0,002306	0,288	0,301	1
Abl2	0,725351	0,02582	0,251	0,246	1
Asph	0,725578	0,005292	0,107	0,114	1
Rnf25	0,725607	-0,00567	0,161	0,167	1
Gm14399	0,725694	0,001768	0,032	0,029	1
Elob	0,725744	-0,00129	0,988	0,987	1
Ccdc59	0,725898	-0,00139	0,692	0,714	1
Timm13	0,726144	0,001218	0,912	0,899	1
Zfp385a	0,726214	0,00458	0,01	0,008	1
Disp2	0,726214	0,006516	0,01	0,008	1
Preb	0,726434	-0,00253	0,402	0,413	1
Trmt1	0,72645	0,011736	0,286	0,28	1
Lipe	0,726503	0,020041	0,404	0,408	1
Trub1	0,726677	0,004565	0,204	0,197	1
Zfp949	0,726796	-0,00517	0,2	0,208	1
Wdsub1	0,727017	-0,01255	0,215	0,221	1
Wdr13	0,727042	-0,01012	0,332	0,337	1
Gga2	0,727053	0,008488	0,101	0,096	1
Papd7	0,727181	0,005871	0,186	0,179	1
Fbxo30	0,727255	0,008607	0,165	0,158	1
Ccdc57	0,727273	-0,00115	0,019	0,016	1
Dld	0,727289	0,003569	0,487	0,472	1
Tspan31	0,727372	-0,02065	0,656	0,642	1
Gm9903	0,727443	0,00458	0,01	0,008	1
Mettl23	0,727448	0,015771	0,433	0,455	1
Aip	0,727594	0,01022	0,587	0,567	1
Ezh2	0,727932	0,001541	0,203	0,211	1
Sertad3	0,728021	0,017928	0,139	0,135	1
Nid1	0,728258	-0,03726	0,208	0,197	1
Dmxl2	0,728311	0,016381	0,231	0,226	1
Psph	0,728518	-0,01047	0,515	0,532	1
Rsph3a	0,728606	0,003391	0,051	0,047	1
Tnfrsf10b	0,728673	0,00361	0,01	0,008	1
Ccdc141	0,728673	0,00264	0,01	0,008	1
Gm26690	0,728673	0,00264	0,01	0,008	1
Slc18b1	0,728673	0,00264	0,01	0,008	1
Fam196b	0,728673	0,00264	0,01	0,008	1
Gm11491	0,728673	0,00264	0,01	0,008	1
Gm47428	0,728673	0,00264	0,01	0,008	1
Gm42715	0,728738	0,010744	0,061	0,057	1
Ccdc90b	0,728771	-0,00672	0,283	0,289	1
Gnb2	0,728789	0,01164	0,982	0,992	1
Eloa	0,728806	-0,00822	0,3	0,307	1
Ensa	0,728806	0,001834	0,595	0,616	1
BC048403	0,728892	0,006796	0,056	0,052	1
Dnm2	0,728947	-0,00991	0,885	0,878	1
Riok3	0,729101	0,015333	0,536	0,538	1
Hdhd5	0,729137	-0,01392	0,106	0,111	1
Iqck	0,729289	0,000442	0,019	0,021	1

Mettl16	0,729356	-0,0008	0,362	0,377	1
Grpel2	0,729622	-0,00967	0,279	0,285	1
Phf2os1	0,729658	0,002968	0,01	0,008	1
Egln3	0,729658	0,002968	0,01	0,008	1
Fut8	0,729687	0,018412	0,619	0,613	1
C330027C(	0,729695	-0,00659	0,016	0,018	1
Zfp3	0,7297	0,000519	0,029	0,033	1
Pus3	0,729751	0,010569	0,155	0,15	1
Wdpcp	0,729768	-0,0035	0,069	0,073	1
Neu4	0,729903	0,001668	0,01	0,008	1
Pelo	0,729903	0,001668	0,01	0,008	1
Vmn2r124	0,729903	0,001668	0,01	0,008	1
C2	0,729903	0,001668	0,01	0,008	1
Lin9	0,729981	-0,0004	0,029	0,033	1
E130309D(	0,729997	-0,00334	0,146	0,153	1
Cdk17	0,730164	0,033429	0,397	0,405	1
Trnau1ap	0,730484	0,011906	0,288	0,281	1
Tecr	0,730488	0,009453	0,997	0,995	1
Mpv17l	0,730513	0,00539	0,27	0,281	1
Dynlt1f	0,730566	0,006486	0,056	0,052	1
9030624J(	0,730626	0,000994	0,551	0,553	1
Cntln	0,730667	-0,00162	0,225	0,216	1
L2hgdh	0,730693	-0,00028	0,048	0,052	1
Actn4	0,730784	-0,00114	0,825	0,813	1
Sarnp	0,730851	-0,00876	0,605	0,623	1
Cdr2l	0,731091	0,003382	0,438	0,457	1
Gm12496	0,731135	0,001028	0,01	0,008	1
3110070M	0,731135	0,001028	0,01	0,008	1
Ndufv2	0,731225	0,010658	0,808	0,829	1
AC132444.	0,731534	-0,00693	0,016	0,018	1
AI606181	0,731548	-0,00339	0,113	0,119	1
Alg1	0,731837	-0,01085	0,264	0,27	1
4933428G:	0,731899	-0,0024	0,019	0,016	1
Gm42595	0,732157	-0,00043	0,029	0,033	1
Gm5862	0,732367	5,69E-05	0,01	0,008	1
Pcnx2	0,732369	0,00039	0,01	0,008	1
1700048O:	0,732369	0,00039	0,01	0,008	1
Gsr	0,73252	-0,0091	0,403	0,411	1
Dcp1a	0,732544	-0,00467	0,237	0,246	1
Rlf	0,732647	-0,00105	0,394	0,408	1
Bmpr1a	0,732778	-0,00592	0,315	0,325	1
Hnrnpu	0,732844	0,000957	0,977	0,98	1
Nip7	0,732964	0,004878	0,27	0,262	1
Ippk	0,732965	-0,00281	0,427	0,434	1
Cyb5d2	0,733115	0,003184	0,256	0,27	1
Mtcp1	0,733298	-0,00566	0,109	0,114	1
Lpar6	0,733386	-0,01332	0,129	0,133	1
Nsmce4a	0,733513	0,009737	0,621	0,608	1
Mark2	0,7336	-0,01965	0,518	0,533	1
Srp54a	0,733626	0,028496	0,32	0,315	1
Gm16212	0,733701	0,002362	0,019	0,021	1
Ptpa	0,733765	-0,01598	0,679	0,676	1

Slc27a1	0,733886	0,02562	0,451	0,447	1
Arv1	0,734131	0,0003	0,048	0,052	1
Rilpl1	0,734487	0,00209	0,391	0,407	1
Hdac1	0,734599	0,016406	0,41	0,41	1
Tedc2	0,734627	-0,00178	0,056	0,06	1
Maml1	0,734717	-0,00353	0,126	0,132	1
Pcdhga11	0,734723	-0,01051	0,083	0,088	1
Gprasp1	0,735397	0,001816	0,421	0,431	1
Mapkap1	0,735512	-0,00582	0,4	0,411	1
Pfdn4	0,735597	0,020882	0,361	0,358	1
Pdha1	0,735819	0,003178	0,57	0,561	1
Gm14296	0,736044	0,005871	0,173	0,166	1
Trappc6b	0,73621	0,013941	0,399	0,389	1
Slc25a18	0,736517	-0,00278	0,016	0,018	1
Zfp879	0,736517	-0,00278	0,016	0,018	1
Mfsd2b	0,736517	-0,00278	0,016	0,018	1
Ctdsp2	0,736698	-0,00342	0,648	0,655	1
Marcks	0,736943	0,01269	0,029	0,033	1
Tirap	0,736992	0,004651	0,254	0,247	1
Mrps15	0,737099	-0,00247	0,482	0,493	1
Fam210a	0,737117	-0,0028	0,238	0,249	1
Nek7	0,737122	-0,01549	0,566	0,569	1
Rab18	0,737142	0,019152	0,839	0,854	1
Tdg	0,737194	0,028278	0,263	0,262	1
Anapc2	0,737619	0,026461	0,619	0,62	1
D3Ertd751	0,738209	-0,00376	0,21	0,218	1
Zfp324	0,738292	-0,00585	0,05	0,054	1
Tet3	0,738418	0,013944	0,494	0,506	1
1110008L1	0,738678	-0,00189	0,045	0,049	1
Kif9	0,738737	-0,00085	0,016	0,018	1
Gm26601	0,738749	0,003201	0,082	0,088	1
Ydjc	0,73889	0,004009	0,049	0,046	1
Bloc1s3	0,738906	0,006694	0,173	0,166	1
Naa60	0,739091	-0,01416	0,27	0,273	1
Ikbkg	0,739161	0,00173	0,155	0,163	1
Lurap1	0,739309	-0,01617	0,271	0,276	1
D230017M	0,739324	-0,0038	0,034	0,037	1
Ripor1	0,739407	-0,0029	0,07	0,065	1
Dnajc1	0,739437	0,004888	0,403	0,418	1
Eya3	0,739506	-0,0024	0,092	0,098	1
Rbm45	0,739813	-0,00029	0,136	0,143	1
Gm48022	0,740033	-0,00244	0,016	0,018	1
Pip5k1c	0,740096	0,019329	0,103	0,099	1
Phf10	0,740218	0,028425	0,425	0,424	1
Ogfod1	0,740291	0,006975	0,153	0,161	1
Slc7a14	0,740367	0,003368	0,037	0,034	1
Cspg5	0,740476	0,00817	0,114	0,109	1
Pik3r4	0,740699	-0,00124	0,203	0,211	1
2700062C(	0,740873	0,019034	0,262	0,259	1
Taco1os	0,740891	-0,0066	0,079	0,083	1
Adam15	0,740909	0,0345	0,357	0,358	1
Tmem109	0,741005	0,010672	0,473	0,489	1

Sec14l5	0,741353	0,013565	0,758	0,769	1
Rad17	0,741434	0,021404	0,299	0,317	1
Milr1	0,741519	0,011186	0,03	0,028	1
Mrpl30	0,741651	0,013608	0,666	0,672	1
Gm28809	0,741785	0,011542	0,03	0,028	1
Cryz	0,741806	-0,00084	0,105	0,111	1
Psmg1	0,741855	0,029536	0,225	0,223	1
Dhfr	0,741923	0,002337	0,159	0,166	1
2210016L2	0,741929	-0,00335	0,726	0,746	1
Slc30a9	0,742042	-0,0116	0,578	0,579	1
Srprb	0,7421	0,010924	0,366	0,359	1
Tle2	0,74238	-0,00253	0,217	0,224	1
Etaa1	0,742399	0,017598	0,183	0,177	1
Gm14295	0,742723	-0,00751	0,104	0,109	1
G2e3	0,742937	0,012835	0,27	0,26	1
Armcx5	0,74298	0,019543	0,21	0,205	1
Tle1	0,743376	0,01861	0,53	0,553	1
Fzd8	0,743578	0,015624	0,057	0,054	1
1700066B1	0,743636	0,004909	0,017	0,015	1
Nck2	0,743723	0,001482	0,248	0,259	1
Acbd3	0,74402	-0,01147	0,469	0,47	1
Elp6	0,744266	-0,00133	0,19	0,198	1
Yeats4	0,74431	0,003164	0,25	0,26	1
Plekhf2	0,744386	0,015669	0,057	0,054	1
Slc30a7	0,744463	0,008039	0,166	0,174	1
Tpk1	0,74461	-0,00944	0,055	0,059	1
Brwd1	0,744673	0,025935	0,515	0,515	1
Pcsk1n	0,74476	0,029292	0,942	0,927	1
Ceacam2	0,744911	0,003715	0,048	0,052	1
Kctd13	0,744997	0,005585	0,979	0,982	1
Nsmaf	0,745092	0,022732	0,179	0,189	1
Opa3	0,745148	0,004545	0,328	0,34	1
Zfp418	0,745231	0,006792	0,054	0,05	1
Zfp868	0,745403	0,015072	0,151	0,146	1
Psmd14	0,745502	0,020742	0,548	0,55	1
Fam103a1	0,74588	-0,00911	0,341	0,341	1
Htatsf1	0,745949	0,018971	0,484	0,485	1
Polrmt	0,746176	-0,01232	0,143	0,148	1
Diaph1	0,746184	-0,00279	0,191	0,198	1
Lrmda	0,746277	0,007091	0,066	0,062	1
Trmo	0,746673	-0,00676	0,076	0,08	1
BC005537	0,746828	0,017375	0,836	0,833	1
Ube3a	0,746831	0,0159	0,893	0,911	1
G3bp1	0,746928	0,005715	0,621	0,649	1
Gm37027	0,747035	0,002982	0,017	0,015	1
Slc10a7	0,747087	0,01058	0,229	0,223	1
Fam91a1	0,747205	0,012857	0,405	0,397	1
Pomp	0,747287	-0,01652	0,907	0,912	1
Zfp512	0,747533	0,004372	0,383	0,379	1
Oat	0,747555	1,06E-05	0,544	0,525	1
Creb3	0,747559	0,022922	0,406	0,408	1
Kctd11	0,74756	0,017766	0,132	0,128	1

Zfp112	0,747569	-0,0044	0,094	0,099	1
Thbs2	0,747615	0,006171	0,066	0,062	1
Trip6	0,74767	0,006501	0,03	0,028	1
Hs1bp3	0,747736	0,010616	0,237	0,246	1
Mex3a	0,747797	0,001424	0,017	0,015	1
Eif3m	0,747902	0,015105	0,726	0,743	1
Exosc3	0,747972	-0,00372	0,164	0,171	1
Shf	0,748022	0,00707	0,082	0,078	1
Zfp449	0,748124	0,004974	0,082	0,078	1
Btbd10	0,748219	0,00205	0,292	0,283	1
Cttn	0,748299	-0,00787	0,433	0,447	1
Gm26508	0,74831	0,005553	0,024	0,021	1
Rps6ka2	0,74838	0,013714	0,179	0,189	1
Dynlt1c	0,748463	-0,00312	0,013	0,015	1
H2-DMa	0,74855	0,002345	0,017	0,015	1
Mrps25	0,748648	-0,00737	0,484	0,494	1
Gtpbp1	0,748696	-0,00362	0,112	0,117	1
Taf2	0,748724	0,014099	0,263	0,257	1
Sult5a1	0,748884	0,00305	0,026	0,029	1
Ofd1	0,749062	-0,01075	0,151	0,156	1
Zbtb22	0,749084	0,010603	0,208	0,218	1
Cbx7	0,749221	-0,00127	0,278	0,286	1
Gm17949	0,749284	-0,00248	0,013	0,015	1
B230312C(	0,749284	-0,00437	0,013	0,015	1
H2afy	0,749284	0,004189	0,613	0,637	1
Tenm2	0,749533	-0,01264	0,113	0,117	1
Capza2	0,750006	0,015526	0,852	0,876	1
Zeb1	0,750066	0,00332	0,017	0,015	1
Pcgf3	0,750147	0,017527	0,323	0,317	1
Zfp111	0,750181	0,009101	0,102	0,098	1
Cmas	0,750354	0,014983	0,328	0,324	1
Gpr85	0,750375	0,001555	0,178	0,185	1
Atpaf1	0,750586	0,011339	0,294	0,288	1
Slc25a39	0,750592	0,000176	0,387	0,397	1
Pcdhb17	0,750894	-0,00185	0,121	0,127	1
Rnf208	0,750981	0,016373	0,405	0,42	1
Ndufv1	0,751081	0,008133	0,811	0,802	1
1600002K(	0,751584	0,006164	0,104	0,099	1
Memo1	0,751735	-0,00693	0,162	0,167	1
Gm7160	0,75177	0,000746	0,017	0,015	1
Cdkn2aip	0,751819	0,018764	0,24	0,234	1
Uchl1	0,75182	-0,07856	0,355	0,354	1
Ttpal	0,752002	0,001104	0,139	0,133	1
9530034E1	0,752148	0,001209	0,045	0,049	1
Tmub1	0,752375	-0,01035	0,261	0,267	1
Tmem120b	0,752535	0,004189	0,143	0,15	1
Polr2l	0,752661	0,006872	0,424	0,436	1
Zdhhc15	0,752715	0,004622	0,042	0,039	1
Chmp1a	0,752726	0,001595	0,563	0,541	1
Cmtr1	0,752881	0,001133	0,091	0,086	1
Arhgef10	0,752911	0,015469	0,957	0,959	1
Carf	0,752937	0,00639	0,189	0,198	1

Map3k2	0,752946	-4,1E-05	0,369	0,359	1
Gm21680	0,752992	-0,0004	0,034	0,037	1
Ptrhd1	0,753381	0,02199	0,253	0,25	1
Rarg	0,753399	0,002345	0,013	0,015	1
Rin1	0,753809	0,001052	0,013	0,015	1
Ppm1f	0,75404	-0,02253	0,223	0,228	1
Atp5g2	0,754189	-0,00369	0,967	0,964	1
Zcchc6	0,75433	-0,01225	0,531	0,532	1
Ubxn11	0,754365	0,000468	0,024	0,021	1
Rfx4	0,754405	0,004254	0,008	0,007	1
Slc35g1	0,754405	0,004254	0,008	0,007	1
Gm3604	0,754684	-0,00049	0,024	0,021	1
Pcdhb9	0,754827	0,009944	0,026	0,029	1
Nup153	0,754854	-0,00618	0,257	0,263	1
1810044D(	0,754921	0,002872	0,079	0,085	1
Sptlc1	0,754951	0,020642	0,455	0,459	1
2900026A(	0,7551	-0,0027	0,024	0,026	1
Tbc1d7	0,755306	0,023286	0,348	0,348	1
Spp2	0,755517	0,003283	0,008	0,007	1
Gm30606	0,755517	0,003283	0,008	0,007	1
Rapgef3os2	0,755517	0,003283	0,008	0,007	1
Gm16350	0,755517	0,004254	0,008	0,007	1
H2afx	0,755528	-0,00309	0,237	0,247	1
Tfb2m	0,755547	0,008758	0,199	0,197	1
Emp2	0,755634	0,010644	0,016	0,018	1
Hoxd1	0,75565	-0,01316	0,146	0,151	1
A830009L(	0,755664	0,001052	0,013	0,015	1
Mtch2	0,755741	0,009378	0,703	0,701	1
Ttc30b	0,755743	-0,00777	0,106	0,111	1
4833413G1	0,755769	-0,00066	0,042	0,046	1
Herc1	0,755916	0,03037	0,509	0,514	1
Dll1	0,755974	0,024597	0,059	0,055	1
Neat1	0,756032	0,03065	0,994	0,995	1
Pde12	0,756124	-0,00402	0,181	0,174	1
Nrde2	0,756349	-0,00365	0,165	0,171	1
Mblac1	0,756439	0,00014	0,024	0,021	1
Zbtb34	0,756556	0,005257	0,059	0,055	1
Stk25	0,756623	0,015305	0,502	0,506	1
Fam83f	0,75663	0,004254	0,008	0,007	1
Gm38250	0,75663	0,003283	0,008	0,007	1
Gm4950	0,75663	0,003283	0,008	0,007	1
Efhc1	0,75663	0,00231	0,008	0,007	1
Fam221a	0,75663	0,00231	0,008	0,007	1
Tmem95	0,75663	0,00231	0,008	0,007	1
Med10	0,756633	0,002994	0,37	0,379	1
Mrpl16	0,75674	0,009587	0,201	0,195	1
Rsrc2	0,756863	0,007386	0,888	0,909	1
Gm16894	0,756868	0,004927	0,035	0,033	1
Gm15594	0,756878	0,007704	0,059	0,055	1
Ppp3cb	0,756979	0,035215	0,426	0,436	1
Galc	0,756997	0,020173	0,475	0,47	1
Atraid	0,757129	0,017358	0,889	0,883	1

Kiz	0,757136	-0,02485	0,244	0,246	1
Ufl1	0,75744	0,004644	0,523	0,515	1
Pim1	0,757545	0,01015	0,042	0,046	1
Ssbp2	0,757676	0,016145	0,231	0,242	1
Spata18	0,757743	0,001337	0,008	0,007	1
Dao	0,757743	0,001337	0,008	0,007	1
Gm43154	0,757743	0,001337	0,008	0,007	1
Thy1	0,757743	0,001337	0,008	0,007	1
Srl	0,757743	0,001337	0,008	0,007	1
4930461C1	0,757743	0,001337	0,008	0,007	1
9330136K2	0,757743	0,001337	0,008	0,007	1
Gm26901	0,757998	-0,00156	0,042	0,046	1
Washc5	0,758032	-0,02287	0,259	0,262	1
B230311B0	0,758198	-0,00049	0,024	0,021	1
Wiz	0,758302	-0,00044	0,332	0,341	1
Rbfox2	0,758306	0,011537	0,122	0,128	1
Rad9a	0,758531	0,01055	0,162	0,158	1
Cul5	0,758642	0,011749	0,637	0,631	1
Gm27003	0,758857	0,000696	0,008	0,007	1
Ccne2	0,758857	0,000696	0,008	0,007	1
Elavl4	0,759136	-0,00092	0,008	0,007	1
Osmr	0,759136	-0,00092	0,008	0,007	1
Senp8	0,759307	-0,00379	0,197	0,205	1
Hexb	0,75935	0,01996	0,343	0,343	1
Nudcd2	0,759407	-0,00724	0,352	0,364	1
Pappa	0,759415	0,001999	0,008	0,007	1
Agtpbp1	0,759434	-0,01635	0,521	0,525	1
Sp4	0,759662	-0,00861	0,328	0,333	1
Mex3c	0,759856	0,022259	0,388	0,387	1
Scfd2	0,759879	-0,00268	0,155	0,161	1
Gm32250	0,759971	-0,00028	0,008	0,007	1
Nkain4	0,759971	-0,00028	0,008	0,007	1
Gm20707	0,759971	-0,00028	0,008	0,007	1
Kcnj13	0,760045	-0,00476	0,01	0,011	1
Nelfa	0,760078	-0,00823	0,187	0,193	1
Prpf40a	0,760236	0,00639	0,729	0,715	1
Lamc1	0,760569	0,014865	0,419	0,413	1
Aes	0,760575	0,013742	0,989	0,993	1
Ola1	0,760687	0,021272	0,46	0,473	1
Wisp1	0,760764	-0,00254	0,039	0,042	1
Ndufb4	0,760898	0,002228	0,888	0,893	1
Nalcn	0,760909	-0,00123	0,078	0,073	1
Prmt9	0,760971	-0,00239	0,15	0,156	1
Hilpda	0,761017	-0,00858	0,073	0,076	1
Adam28	0,761253	-0,00016	0,024	0,026	1
Plxnd1	0,761365	-0,00252	0,008	0,007	1
Ganc	0,761379	-0,00299	0,265	0,27	1
Haus6	0,761427	0,009678	0,168	0,163	1
Fam114a2	0,76144	-0,00156	0,479	0,463	1
Tshz2	0,761453	-0,00539	0,01	0,011	1
Gm48796	0,761454	-0,00088	0,01	0,011	1
Cetn4	0,761688	-0,00219	0,01	0,011	1

Mkx	0,761688	-0,00219	0,01	0,011	1
Pde4a	0,761795	0,010598	0,237	0,229	1
Ccdc39	0,7618	0,002626	0,086	0,091	1
Brox	0,761855	-0,00421	0,468	0,478	1
Dcps	0,761882	-0,009	0,566	0,572	1
Sdccag8	0,762036	0,007669	0,227	0,223	1
Bicdl1	0,762093	0,003037	0,035	0,033	1
Gm26674	0,76239	-0,00155	0,01	0,011	1
Med9os	0,76239	-0,00155	0,01	0,011	1
Pik3r5	0,76239	-0,00155	0,01	0,011	1
Tlcd2	0,76239	-0,00155	0,01	0,011	1
Mkrn1	0,76241	5,69E-05	0,786	0,798	1
Ankle2	0,76248	0,003315	0,312	0,319	1
Zfp354b	0,762773	0,004009	0,042	0,046	1
Igfbpl1	0,763318	-0,03747	0,008	0,007	1
AC161165.	0,763331	-0,00122	0,01	0,011	1
Ankrd27	0,763475	0,009558	0,193	0,202	1
Otud7a	0,763476	-0,00108	0,184	0,19	1
Atp6v1g2	0,763826	-0,00111	0,063	0,067	1
Incenp	0,76393	0,004343	0,059	0,055	1
Msi1	0,764034	-0,00058	0,01	0,011	1
Prpf38a	0,764102	0,005637	0,312	0,324	1
Camsap2	0,764137	0,02833	0,397	0,4	1
Lhpp	0,764221	0,002611	0,089	0,094	1
Fxr1	0,764245	-0,00944	0,89	0,889	1
Lysmd1	0,764304	-0,00052	0,235	0,226	1
Taok1	0,765031	0,001668	0,871	0,891	1
Zfp512b	0,765111	0,006154	0,131	0,125	1
Pop7	0,765225	0,010528	0,277	0,272	1
Golga7	0,765286	0,000102	0,969	0,976	1
Ppat	0,765504	0,013585	0,194	0,19	1
Cc2d1b	0,765549	-0,00554	0,366	0,377	1
Gm4793	0,765568	0,004285	0,028	0,026	1
Nuak1	0,765672	0,035182	0,452	0,457	1
Clrn1	0,765679	0,00039	0,01	0,011	1
Ssc4d	0,765679	0,00039	0,01	0,011	1
Tigd3	0,765679	0,00039	0,01	0,011	1
Trmt12	0,765935	0,000192	0,076	0,08	1
Cct4	0,766022	0,025904	0,759	0,797	1
Tor3a	0,766208	-0,00432	0,021	0,023	1
Dna2	0,766208	-0,00432	0,021	0,023	1
Tspan5	0,766368	0,00306	0,779	0,784	1
Nudcd3	0,766411	0,0224	0,698	0,691	1
Gpatch2	0,766869	0,031545	0,324	0,327	1
Ccnd1	0,766942	0,042197	0,057	0,06	1
Mknk2	0,767013	0,001947	0,141	0,135	1
Zcchc2	0,767046	0,013939	0,153	0,15	1
Npdc1	0,767259	0,016642	0,818	0,829	1
AW549877	0,76752	0,032868	0,63	0,639	1
Tnfrsf1a	0,767698	0,010986	0,095	0,091	1
Ldlrad3	0,768108	-0,00848	0,54	0,553	1
Mlh1	0,768127	-0,0064	0,159	0,164	1

Pdcd2	0,768241	-0,00376	0,236	0,241	1
Setd3	0,768268	-0,00054	0,766	0,798	1
S1pr2	0,768314	-0,00495	0,073	0,076	1
Rdh11	0,768488	0,007899	0,419	0,411	1
Gm10037	0,768516	-0,00336	0,021	0,023	1
Nrcam	0,768772	0,001252	0,039	0,042	1
Tonsl	0,768846	-0,00145	0,021	0,023	1
Slc25a15	0,769293	0,008476	0,115	0,111	1
Snn	0,769298	-0,00761	0,294	0,298	1
Fggy	0,769393	0,007979	0,195	0,19	1
Zfp300	0,76948	0,009234	0,05	0,054	1
Pcdhb20	0,769558	0,012673	0,072	0,068	1
Egf	0,769745	0,010905	0,024	0,026	1
Smndc1	0,769919	0,005195	0,553	0,572	1
Zfp407	0,769929	-0,01663	0,178	0,182	1
Gm26829	0,770022	-0,0051	0,007	0,008	1
Pdcd2l	0,770291	-0,00974	0,186	0,192	1
Lipo3	0,770352	-0,00634	0,057	0,06	1
Slc4a3	0,770575	-0,00563	0,039	0,042	1
Med17	0,770589	-0,00293	0,302	0,311	1
Isy1	0,770605	0,010858	0,205	0,2	1
Ino80dos	0,770624	0,00188	0,122	0,127	1
BC002059	0,770649	0,008511	0,097	0,093	1
Slc11a1	0,770861	-0,00286	0,007	0,008	1
Stmn2	0,770861	-0,00286	0,007	0,008	1
Tekt2	0,770861	-0,00286	0,007	0,008	1
Trhde	0,770861	-0,00286	0,007	0,008	1
AC140186.	0,770861	-0,00447	0,007	0,008	1
3222401L1	0,770941	-0,00503	0,299	0,311	1
Ngdn	0,770946	0,016023	0,439	0,434	1
Dlg2	0,771028	0,021279	0,405	0,403	1
Smg6	0,771523	0,045297	0,37	0,377	1
1110034G:	0,771936	0,004058	0,221	0,213	1
AV099323	0,772132	0,001748	0,028	0,026	1
Fxyd5	0,772261	-0,00189	0,007	0,008	1
A730098A:	0,772261	-0,00189	0,007	0,008	1
Htra3	0,772493	-0,00629	0,19	0,197	1
Twnk	0,772615	-0,00548	0,163	0,169	1
Rrp8	0,772619	-0,00179	0,181	0,187	1
St7l	0,772642	-0,01123	0,588	0,572	1
Rasa1	0,772696	0,000746	0,374	0,361	1
Fv1	0,77282	-0,00125	0,007	0,008	1
Lmntd1	0,77282	-0,00125	0,007	0,008	1
A330041J2	0,77282	-0,00125	0,007	0,008	1
Dnah17	0,77282	-0,00125	0,007	0,008	1
D630036H	0,77282	-0,00125	0,007	0,008	1
A930007I1	0,77282	-0,00125	0,007	0,008	1
Nfkb1	0,772891	-0,00441	0,208	0,213	1
Cyp3a13	0,773362	0,004404	0,095	0,091	1
Ppip5k1	0,7736	0,000497	0,243	0,254	1
Meis1	0,773607	0,013712	0,363	0,359	1
Clstn2	0,773661	-0,00092	0,007	0,008	1

Itga9	0,773661	-0,00092	0,007	0,008	1
Angpt1	0,773661	-0,00092	0,007	0,008	1
Gm20463	0,773661	-0,00092	0,007	0,008	1
Nmrk1	0,773766	0,012669	0,171	0,167	1
Hmgn1	0,773889	-0,00423	0,579	0,593	1
Neurl4	0,773915	0,007776	0,281	0,289	1
Gprasp2	0,773978	-0,00092	0,047	0,044	1
Gm26840	0,774009	0,002054	0,022	0,02	1
H2afj	0,774056	-0,00333	0,954	0,979	1
Gm17555	0,774115	-0,00257	0,004	0,005	1
Zmiz1os1	0,774115	-0,00257	0,004	0,005	1
Mmrn2	0,774115	-0,00257	0,004	0,005	1
Haus4	0,774115	-0,00257	0,004	0,005	1
Aifm3	0,774115	-0,00257	0,004	0,005	1
AC120150.	0,774115	-0,00257	0,004	0,005	1
Slc3a1	0,774115	-0,00257	0,004	0,005	1
Axl	0,774115	-0,00418	0,004	0,005	1
Pitpnm2	0,774221	0,000696	0,007	0,008	1
Ackr1	0,774221	-0,00028	0,007	0,008	1
Pidd1	0,774221	-0,00028	0,007	0,008	1
Myh3	0,774221	-0,00028	0,007	0,008	1
Gm20522	0,774221	-0,00028	0,007	0,008	1
Fam160a2	0,774257	-0,00533	0,16	0,166	1
Nadk	0,77437	-0,01338	0,366	0,371	1
Tomm40l	0,774389	0,005101	0,229	0,237	1
Spred3	0,774458	0,001098	0,021	0,023	1
Fancb	0,774569	0,002655	0,015	0,013	1
Gm26590	0,774569	0,002655	0,015	0,013	1
Top3a	0,774601	0,001863	0,052	0,049	1
Rftn1	0,774624	0,007371	0,677	0,689	1
Kcnh2	0,775181	0,002686	0,022	0,02	1
Stap1	0,775214	-0,00159	0,004	0,005	1
Chtf8	0,775303	-0,00054	0,262	0,27	1
Cdh13	0,775343	0,008436	0,007	0,008	1
Hap1	0,77544	-0,00295	0,028	0,031	1
Arpc1a	0,775448	-0,00679	0,979	0,984	1
Znhit1	0,775554	-0,01074	0,652	0,659	1
Zfat	0,775575	0,00743	0,045	0,042	1
Gm14486	0,77558	-0,00095	0,004	0,005	1
Pltp	0,77558	-0,00095	0,004	0,005	1
Btk	0,77558	-0,00095	0,004	0,005	1
Gm15298	0,77558	-0,00095	0,004	0,005	1
Bbs12	0,77558	-0,00095	0,004	0,005	1
Sis	0,77558	-0,00095	0,004	0,005	1
Olfml3	0,77558	-0,00095	0,004	0,005	1
Gm42918	0,77558	-0,00095	0,004	0,005	1
Cpa5	0,77558	-0,00095	0,004	0,005	1
Oscar	0,77558	-0,00095	0,004	0,005	1
Lrfn3	0,77558	-0,00095	0,004	0,005	1
Gm17102	0,77558	-0,00095	0,004	0,005	1
Ifitm10	0,77558	-0,00095	0,004	0,005	1
Mypn	0,77558	-0,00095	0,004	0,005	1

Npm2	0,77558	-0,00095	0,004	0,005	1
Olfr905	0,77558	-0,00095	0,004	0,005	1
AU023762	0,77558	-0,00095	0,004	0,005	1
Cmtm7	0,77558	-0,00095	0,004	0,005	1
Zfp652os	0,77558	-0,00095	0,004	0,005	1
Gm11592	0,77558	-0,00095	0,004	0,005	1
Rara	0,77558	-0,00095	0,004	0,005	1
Fkbp10	0,77558	-0,00095	0,004	0,005	1
Gm39397	0,77558	-0,00095	0,004	0,005	1
Ripor2	0,77558	-0,00095	0,004	0,005	1
Gm38604	0,77558	-0,00095	0,004	0,005	1
4930592A(	0,77558	-0,00095	0,004	0,005	1
Car15	0,77558	-0,00095	0,004	0,005	1
Zdhhc19	0,77558	-0,00095	0,004	0,005	1
Gm9968	0,77558	-0,00095	0,004	0,005	1
Pnldc1	0,77558	-0,00095	0,004	0,005	1
Col17a1	0,77558	-0,00095	0,004	0,005	1
Gm31282	0,775581	0,00231	0,004	0,005	1
Gm10767	0,775581	-0,00061	0,004	0,005	1
Gm15537	0,775581	-0,00061	0,004	0,005	1
Hoxb3	0,775581	-0,00125	0,004	0,005	1
Wrn	0,775601	-0,01901	0,3	0,302	1
Zc3h12c	0,775618	-0,00384	0,146	0,151	1
Syce2	0,775622	0,000696	0,007	0,008	1
Cables1	0,775622	0,001668	0,007	0,008	1
Eps8	0,775684	0,000468	0,022	0,02	1
Ccnl1	0,775744	0,068179	0,649	0,672	1
Dus3l	0,775794	0,014631	0,309	0,306	1
Gm9856	0,775886	0,009204	0,067	0,063	1
Stk40	0,775908	0,03174	0,153	0,151	1
Cbx8	0,775979	0,004651	0,064	0,06	1
Fanci	0,775992	0,002017	0,015	0,013	1
Zxdb	0,776084	0,00249	0,135	0,141	1
Vps45	0,776153	0,002892	0,251	0,26	1
Sema4g	0,776289	-0,00099	0,078	0,073	1
Ccl2	0,776314	0,00264	0,004	0,005	1
Nucb2	0,776513	0,001806	0,039	0,042	1
Npb	0,776524	-0,02495	0,082	0,086	1
Rrp15	0,776605	0,017186	0,139	0,137	1
Ywhah	0,776646	0,004082	0,605	0,584	1
Sdcbp2	0,77668	0,001005	0,004	0,005	1
Ptp4a3	0,77668	0,001005	0,004	0,005	1
Frmpd1	0,77668	2,86E-05	0,004	0,005	1
Samd11	0,77668	2,86E-05	0,004	0,005	1
Gm42864	0,77668	2,86E-05	0,004	0,005	1
Asprv1	0,77668	2,86E-05	0,004	0,005	1
1700028J1	0,77668	2,86E-05	0,004	0,005	1
Gm26816	0,77668	2,86E-05	0,004	0,005	1
Gm47414	0,77668	2,86E-05	0,004	0,005	1
Gm26521	0,77668	2,86E-05	0,004	0,005	1
Ccdc151	0,77668	2,86E-05	0,004	0,005	1
Gm48393	0,77668	2,86E-05	0,004	0,005	1

Pknox2	0,77668	2,86E-05	0,004	0,005	1
Gm49380	0,77668	2,86E-05	0,004	0,005	1
Gm26767	0,77668	2,86E-05	0,004	0,005	1
Crtap	0,77668	2,86E-05	0,004	0,005	1
Rrm2	0,77668	2,86E-05	0,004	0,005	1
Fam71d	0,77668	2,86E-05	0,004	0,005	1
Gm14424	0,776713	-0,002	0,028	0,031	1
Cd2bp2	0,776881	-0,00398	0,352	0,358	1
B230354K1	0,776892	-0,00027	0,157	0,164	1
Gns	0,776903	0,011788	0,428	0,444	1
9530082P2	0,776948	-0,00238	0,06	0,063	1
Sec14l1	0,777002	0,022525	0,511	0,506	1
Pcdhb3	0,777025	0,00458	0,007	0,008	1
Spg21	0,777027	0,004302	0,343	0,353	1
Zfp689	0,777174	-0,00092	0,052	0,055	1
Dennd5a	0,777226	0,017363	0,957	0,946	1
Xylt2	0,777379	-0,00966	0,097	0,101	1
Ulk1	0,777387	0,018814	0,233	0,228	1
Gstcd	0,777598	0,007028	0,108	0,114	1
Rrm2b	0,777622	0,023064	0,472	0,481	1
Ppfia4	0,77778	0,00198	0,004	0,005	1
Gm42851	0,77778	0,00198	0,004	0,005	1
Gm21671	0,777821	-0,00055	0,015	0,013	1
Arsk	0,77785	0,009144	0,231	0,241	1
Ift74	0,777905	-0,00154	0,181	0,187	1
Rimkla	0,777914	0,004936	0,045	0,042	1
Slx1b	0,778011	-0,00072	0,193	0,2	1
Rabgap1	0,778035	0,00066	0,569	0,563	1
Tshz1	0,778205	-0,01586	0,535	0,53	1
Dlg4	0,77833	-0,0039	0,057	0,06	1
Irx3	0,778428	0,003937	0,007	0,008	1
Fam104a	0,778499	0,006787	0,525	0,525	1
Hsdl1	0,7785	-0,00547	0,24	0,247	1
Arhgap11a	0,778638	0,002036	0,015	0,013	1
Leng1	0,778758	0,00306	0,3	0,312	1
Drg1	0,778766	-0,00381	0,498	0,507	1
Mbd2	0,778793	0,019819	0,628	0,634	1
Wdr53	0,778978	-0,00566	0,116	0,12	1
Gfm2	0,779011	0,009501	0,146	0,141	1
Pim2	0,779043	0,000416	0,015	0,013	1
Lrrc56	0,779226	-0,00057	0,052	0,055	1
Itsn1	0,779231	0,009562	0,129	0,124	1
Dhcr7	0,779349	0,028645	0,596	0,602	1
Sos1	0,779539	-0,01416	0,519	0,522	1
Serf1	0,779615	0,001863	0,11	0,115	1
Atf7ip	0,779699	0,027659	0,727	0,759	1
Snrnp35	0,779702	0,012614	0,183	0,177	1
Prr11	0,780082	0,001729	0,021	0,023	1
Lrrc14	0,780107	0,006953	0,181	0,189	1
Smad2	0,780135	0,011616	0,162	0,158	1
Oxld1	0,780405	-0,0012	0,098	0,102	1
Leng8	0,780466	0,000629	0,643	0,647	1

A430073D:	0,78067	-0,00055	0,015	0,013	1
Macrod2	0,780673	0,00356	0,216	0,223	1
Strn4	0,780701	0,002589	0,641	0,624	1
Parg	0,78073	0,006097	0,327	0,317	1
Zfp90	0,780822	0,000775	0,227	0,218	1
Napb	0,7809	0,011106	0,141	0,148	1
Zfp27	0,780925	-0,00436	0,078	0,081	1
Chfr	0,78107	0,011718	0,393	0,408	1
Bhlhb9	0,781079	-0,00341	0,015	0,013	1
Acvr1	0,781322	-0,01145	0,184	0,189	1
9330175E1	0,781391	-0,00303	0,022	0,02	1
Rps6ka3	0,781403	0,003038	0,125	0,12	1
Katnb1	0,78148	0,006151	0,151	0,146	1
Gm14703	0,781485	-0,00022	0,015	0,013	1
Cnbd2	0,781586	0,006175	0,067	0,063	1
Zfp146	0,781767	-0,00215	0,119	0,124	1
Acad9	0,781815	-0,01759	0,224	0,226	1
Vps35	0,781879	0,010115	0,904	0,893	1
Ccdc148	0,782148	-0,00439	0,1	0,104	1
Fam151b	0,782223	0,011713	0,189	0,184	1
Itpkc	0,782444	0,015558	0,095	0,101	1
Coa5	0,782593	0,005612	0,212	0,207	1
Rrp9	0,782821	0,015671	0,24	0,25	1
Gm44777	0,782908	-0,00839	0,062	0,065	1
Rundc3a	0,782909	0,021972	0,46	0,465	1
Pfas	0,783065	-0,00461	0,2	0,207	1
AC122413.	0,783235	-0,00046	0,028	0,031	1
Ice1	0,78325	0,003436	0,421	0,437	1
Slc13a5	0,783276	-0,00311	0,044	0,047	1
Zfp946	0,783764	-0,00684	0,105	0,109	1
Rab26os	0,783923	-0,00121	0,052	0,055	1
Mindy2	0,783977	-0,00855	0,711	0,733	1
Klf8	0,783984	0,003063	0,045	0,042	1
Oplah	0,78423	-0,00773	0,067	0,07	1
Dscaml1	0,784282	0,015186	0,637	0,646	1
Nkd1	0,784721	-0,01578	0,556	0,559	1
Acaa2	0,784757	-0,00054	0,06	0,063	1
Rif1	0,784913	0,021995	0,685	0,706	1
Tmem128	0,785068	0,008234	0,545	0,527	1
Zfp346	0,78532	-0,00154	0,197	0,203	1
As3mt	0,785385	0,011716	0,331	0,343	1
Pkd1	0,785397	0,002257	0,325	0,335	1
G0s2	0,785404	0,03239	0,124	0,119	1
Fam98b	0,786113	0,003201	0,349	0,363	1
0610010K:	0,786151	-0,00407	0,321	0,327	1
Fnbp4	0,786219	-0,00608	0,364	0,377	1
Gm29107	0,786317	-0,00115	0,018	0,02	1
Ablim2	0,786354	0,006028	0,495	0,511	1
Zfp607a	0,786403	0,017549	0,05	0,047	1
Hnrnpab	0,786418	0,002408	0,787	0,797	1
Pnp	0,786428	0,041386	0,226	0,224	1
Cript	0,786433	0,00041	0,82	0,847	1

Bcl2l2	0,786496	0,008801	0,339	0,351	1
Hoxaas3	0,7866	-0,00069	0,036	0,039	1
Dip2a	0,786791	0,007913	0,96	0,958	1
Tmem64	0,786903	0,015836	0,086	0,083	1
Xxylt1	0,786913	-0,00348	0,207	0,213	1
Adcy6	0,787506	-0,00196	0,073	0,076	1
Abi1	0,787664	0,029057	0,421	0,42	1
Masp2	0,787697	0,0049	0,006	0,005	1
Brinp2	0,787697	0,002954	0,006	0,005	1
Olfr328	0,787697	0,002954	0,006	0,005	1
Lrrc24	0,787697	0,002954	0,006	0,005	1
Gm49226	0,787697	0,002954	0,006	0,005	1
Adgrf4	0,787697	0,002954	0,006	0,005	1
Tbx2	0,787697	0,005871	0,006	0,005	1
Clstn3	0,787697	0,003927	0,006	0,005	1
Psmd12	0,787761	0,005871	0,59	0,615	1
Fcf1	0,787779	0,016824	0,366	0,364	1
Slc35f6	0,787875	0,005425	0,356	0,348	1
Zfp951	0,787987	-0,00647	0,026	0,028	1
Tmem219	0,788407	1,43E-05	0,39	0,405	1
Agt	0,788467	-0,00019	0,018	0,02	1
Trim3	0,788488	0,007756	0,569	0,558	1
Slc15a2	0,788508	0,005871	0,038	0,036	1
Prag1	0,788511	0,008974	0,465	0,457	1
Cabp1	0,788671	0,003927	0,006	0,005	1
4930593A(	0,788671	0,002954	0,006	0,005	1
Gm10399	0,788671	0,002954	0,006	0,005	1
Unc5a	0,788671	0,002954	0,006	0,005	1
Reep2	0,788671	0,002954	0,006	0,005	1
Hsd11b1	0,788671	0,00198	0,006	0,005	1
Gja6	0,788671	0,00198	0,006	0,005	1
Gm30097	0,788671	0,00198	0,006	0,005	1
1700094M	0,788671	0,00198	0,006	0,005	1
Gm44037	0,788671	0,00198	0,006	0,005	1
Il12rb2	0,788671	0,00198	0,006	0,005	1
Ptpn5	0,788671	0,00198	0,006	0,005	1
Rlbp1	0,788671	0,00198	0,006	0,005	1
Hecw1	0,788671	0,00198	0,006	0,005	1
Ppp1r18os	0,788672	0,00231	0,006	0,005	1
Gm32036	0,788767	-0,00339	0,109	0,104	1
Man2a2	0,788901	0,012153	0,806	0,816	1
Drp2	0,788997	0,017755	0,006	0,005	1
Grwd1	0,789025	0,009227	0,149	0,145	1
Slc5a6	0,789067	-0,00208	0,126	0,13	1
Ddx58	0,789083	-0,0093	0,054	0,057	1
Taf8	0,789178	0,004477	0,155	0,15	1
Rmdn2	0,789237	0,007696	0,067	0,063	1
Tbrg4	0,78928	0,014135	0,162	0,158	1
Cpne9	0,789322	0,004254	0,006	0,005	1
Bend7	0,789322	0,00231	0,006	0,005	1
Smyd5	0,789504	-0,00064	0,132	0,137	1
Idh1	0,789522	-0,02514	0,537	0,53	1

2900009J0	0,789646	0,001005	0,006	0,005	1
Kif28	0,789646	0,001005	0,006	0,005	1
Tmem236	0,789646	0,001005	0,006	0,005	1
Ankef1	0,789646	0,001005	0,006	0,005	1
Rhox8	0,789646	0,001005	0,006	0,005	1
Cfap69	0,789646	0,001005	0,006	0,005	1
4933417D1	0,789646	0,001005	0,006	0,005	1
Gm16741	0,789646	0,001005	0,006	0,005	1
Gm35818	0,789646	0,001005	0,006	0,005	1
AC098883.	0,789646	0,001005	0,006	0,005	1
Gm21833	0,789646	0,001005	0,006	0,005	1
AC125141.	0,789646	0,001005	0,006	0,005	1
Csf1r	0,789646	0,001005	0,006	0,005	1
Hspa1l	0,789647	0,001337	0,006	0,005	1
2700081O1	0,789744	0,000913	0,044	0,047	1
Gm26518	0,789873	0,016811	0,227	0,237	1
Sod1	0,789976	0,014425	0,719	0,746	1
Vav2	0,790017	0,002427	0,036	0,039	1
Plekhm3	0,79005	-0,00696	0,331	0,34	1
Cdc20	0,790073	0,002716	0,028	0,031	1
Fam58b	0,790085	0,012403	0,216	0,211	1
Arl13b	0,790317	0,001052	0,46	0,467	1
Rwdd2a	0,790596	0,005563	0,055	0,052	1
Gm45708	0,790622	0,000363	0,006	0,005	1
Slc39a2	0,790622	0,000363	0,006	0,005	1
Trim9	0,790622	0,000363	0,006	0,005	1
Dio2	0,790622	0,000363	0,006	0,005	1
AI987944	0,790667	0,029831	0,285	0,288	1
Eri2	0,790744	-0,00108	0,146	0,151	1
Gtf3c3	0,790853	0,00487	0,233	0,241	1
Pfn1	0,791008	-0,00529	0,984	0,982	1
Serhl	0,791154	0,000442	0,018	0,02	1
Stk24	0,791441	0,00769	0,2	0,207	1
Gm4349	0,791597	-0,00061	0,006	0,005	1
Cdk19os	0,791597	-0,00061	0,006	0,005	1
Gm26765	0,791597	-0,00061	0,006	0,005	1
Gm35256	0,791597	-0,00061	0,006	0,005	1
Pdxp	0,791722	0,00089	0,045	0,042	1
Slc16a2	0,791757	-0,00206	0,09	0,094	1
Dapk3	0,791846	-0,00678	0,374	0,384	1
Thoc5	0,791894	-0,00307	0,203	0,21	1
Aco2	0,791948	0,027106	0,833	0,823	1
Utp11	0,792028	-0,00509	0,518	0,524	1
Gm20939	0,792048	0,001075	0,018	0,02	1
Deptor	0,79231	-0,01306	0,242	0,246	1
Smpd3	0,79272	0,001334	0,101	0,106	1
Tmem81	0,792787	0,006125	0,233	0,226	1
Fzr1	0,792915	0,004132	0,27	0,263	1
Dynlt1a	0,792918	0,010135	0,126	0,122	1
Rbm22	0,79305	0,011885	0,644	0,636	1
Dnase1l1	0,793066	0,002427	0,036	0,039	1
Med7	0,793082	-0,00386	0,294	0,299	1

Smpd2	0,793341	0,008347	0,165	0,161	1
Cactin	0,793502	0,013774	0,173	0,169	1
Zbtb1	0,793766	0,015746	0,214	0,224	1
Nt5c2	0,793795	0,003569	0,229	0,223	1
Ppp4r3a	0,793805	0,019007	0,483	0,486	1
Cul9	0,793949	0,023455	0,268	0,267	1
Iqcg	0,794114	0,002378	0,026	0,024	1
Tmem268	0,794208	0,025076	0,309	0,309	1
Zfp608	0,794311	0,001315	0,378	0,385	1
Zfp651	0,794324	0,018846	0,469	0,465	1
Uck2	0,794342	0,008991	0,135	0,132	1
Kcmf1	0,794381	0,024032	0,445	0,452	1
Plagl2	0,794491	0,002023	0,085	0,089	1
1700019L1	0,794587	0,003991	0,036	0,039	1
Map3k11	0,79467	0,036466	0,386	0,393	1
Ssfa2	0,794858	0,020302	0,509	0,507	1
Itpr2	0,794969	0,007217	0,256	0,263	1
Anxa11	0,794971	-0,00254	0,041	0,044	1
Phlpp1	0,795062	0,001092	0,989	0,987	1
Virma	0,795098	-0,0058	0,296	0,304	1
Ppp1r17	0,795117	0,009379	0,02	0,018	1
Hspbp1	0,795147	0,021938	0,315	0,315	1
Yeats2	0,795161	0,010241	0,253	0,263	1
Adamts4	0,795183	0,003087	0,917	0,919	1
Gm15952	0,795355	-0,00046	0,026	0,028	1
Lrp12	0,795558	-0,0041	0,192	0,198	1
Ncbp2	0,795559	0,01327	0,536	0,538	1
Nsun6	0,795662	-0,00488	0,256	0,262	1
Nup133	0,796583	0,000963	0,256	0,263	1
Nol11	0,796703	0,009794	0,269	0,265	1
Ndufaf1	0,796798	0,000795	0,14	0,135	1
Upp2	0,796813	-0,02164	0,155	0,158	1
Dusp10	0,797033	0,070644	0,417	0,434	1
Tmem18	0,797146	-0,00234	0,192	0,197	1
Prkcsh	0,797222	0,018961	0,69	0,694	1
Rsl1d1	0,797366	-0,01052	0,571	0,559	1
Pacsin3	0,797385	-0,00273	0,56	0,546	1
Crybb1	0,797458	-0,00112	0,026	0,028	1
Tbc1d2b	0,79749	-0,00315	0,041	0,044	1
Oser1	0,79763	-0,00245	0,512	0,515	1
Ppp1r37	0,797949	0,021636	0,54	0,541	1
Ercc2	0,797992	0,007255	0,154	0,161	1
Tspan2	0,798023	-0,01269	0,99	0,989	1
Lamtor2	0,798182	-0,01127	0,75	0,741	1
0610009O	0,798265	0,00654	0,349	0,345	1
Zfp142	0,798371	0,018343	0,184	0,182	1
Utp15	0,798486	-0,0006	0,18	0,174	1
Yae1d1	0,798544	0,018431	0,487	0,48	1
Wtap	0,798719	-0,00077	0,398	0,392	1
Med16	0,799223	0,006121	0,236	0,242	1
Ggcx	0,79934	0,002668	0,101	0,106	1
Rab33b	0,799391	0,002956	0,539	0,55	1

Eno3	0,799493	0,006186	0,028	0,031	1
Nol4l	0,79953	0,015329	0,363	0,361	1
Fuom	0,79955	0,001474	0,21	0,216	1
Alkbh6	0,799553	0,001832	0,251	0,244	1
Irf3	0,79971	0,011833	0,35	0,361	1
Gm2694	0,799958	0,007028	0,101	0,098	1
Cox16	0,799974	-0,01644	0,135	0,138	1
Sytl5	0,800022	-0,00016	0,026	0,028	1
Nipsnap2	0,80017	9,5E-05	0,538	0,527	1
Smc4	0,800285	-0,00699	0,304	0,298	1
Dad1	0,800412	0,010282	0,958	0,964	1
Itfg2	0,80055	0,016413	0,335	0,33	1
Mns1	0,800783	0,004292	0,026	0,028	1
Dpm1	0,800789	-0,01053	0,619	0,629	1
Tor1b	0,800817	-0,01711	0,397	0,405	1
Tmem63b	0,800838	0,017023	0,619	0,628	1
Tbpl1	0,800915	0,001508	0,232	0,224	1
Mthfsd	0,800917	0,002889	0,168	0,174	1
Gm38335	0,801009	0,011167	0,051	0,049	1
Gm26772	0,801033	0,023689	0,013	0,011	1
Gcfc2	0,801061	0,004753	0,132	0,137	1
Casp7	0,801262	-0,00437	0,015	0,016	1
Sms	0,801437	0,015601	0,087	0,085	1
Vps25	0,801479	-0,00111	0,108	0,112	1
Eid3	0,801485	0,006189	0,02	0,018	1
Zfp319	0,801489	0,011663	0,16	0,156	1
Ep400	0,801589	0,032176	0,486	0,498	1
Gnptg	0,801617	-0,01164	0,598	0,584	1
Ppp1r14c	0,801728	-0,0058	0,054	0,057	1
Gm9484	0,801891	0,002072	0,026	0,024	1
Dap3	0,802028	0,015282	0,426	0,424	1
Trmt6	0,802107	0,014071	0,343	0,356	1
Ttc17	0,802201	0,011093	0,21	0,207	1
Xpnpep3	0,802264	0,00669	0,159	0,166	1
Mrps10	0,802548	0,010223	0,367	0,361	1
Plin4	0,803201	0,007278	0,121	0,117	1
Ptprg	0,803227	-0,00312	0,015	0,016	1
Apc2	0,803337	0,006432	0,144	0,14	1
Capn10	0,803396	-0,0062	0,208	0,215	1
Naga	0,803425	-0,00687	0,23	0,234	1
Tchp	0,803453	0,012525	0,107	0,112	1
Cpxm1	0,803668	0,003295	0,013	0,011	1
Neurl2	0,803668	0,003295	0,013	0,011	1
Slc9b1	0,803668	0,004262	0,013	0,011	1
Ccnt1	0,803668	0,032656	0,475	0,489	1
Gm7361	0,8038	0,00112	0,026	0,028	1
Nol12	0,803886	0,013642	0,309	0,307	1
Glrx	0,803951	0,002072	0,026	0,028	1
Ranbp2	0,803997	0,006348	0,582	0,584	1
Gm34006	0,804058	-0,00323	0,033	0,031	1
Coq3	0,804473	-0,00249	0,115	0,119	1
Sema7a	0,804498	-0,02398	0,594	0,584	1

Vps13b	0,804554	0,004063	0,322	0,333	1
Vgll4	0,80458	-0,00029	0,732	0,754	1
Fancf	0,804857	-0,0062	0,206	0,211	1
Gm43137	0,804988	0,002655	0,013	0,011	1
Map1s	0,805029	-0,00539	0,307	0,314	1
Kdm3a	0,805036	0,019614	0,357	0,372	1
Acot9	0,805205	-0,00674	0,163	0,167	1
Gm15747	0,805206	0,008119	0,013	0,011	1
Tmsb15l	0,805206	0,002328	0,013	0,011	1
Map3k21	0,805206	0,002328	0,013	0,011	1
Gpr19	0,805362	0,00173	0,149	0,154	1
Gm36423	0,805648	0,001052	0,013	0,011	1
Ilf2	0,805699	0,020418	0,723	0,737	1
Slc25a45	0,80571	0,006718	0,137	0,133	1
Mars2	0,805744	-0,00227	0,143	0,138	1
Zfp12	0,805966	0,002737	0,127	0,132	1
Tfpt	0,806055	0,010841	0,314	0,309	1
Plekhg2	0,806058	0,014583	0,036	0,039	1
Elmod3	0,806712	-0,00846	0,182	0,185	1
Yars	0,806727	0,008074	0,355	0,367	1
Pip4k2b	0,806733	0,008489	0,21	0,207	1
Capn9	0,806745	0,001359	0,013	0,011	1
Gm6297	0,806894	-0,01239	0,043	0,046	1
Yaf2	0,806932	0,007948	0,474	0,478	1
Tmem9	0,80697	-0,00839	0,109	0,112	1
Naxe	0,806999	-0,02041	0,692	0,683	1
Top1	0,807005	0,031177	0,856	0,865	1
Pcnx3	0,807005	0,002979	0,288	0,278	1
Commd7	0,80705	0,024099	0,401	0,407	1
Lrguk	0,807168	-0,00118	0,015	0,016	1
Neu3	0,807168	-0,00118	0,015	0,016	1
Kazald1	0,807168	-0,00118	0,015	0,016	1
Thop1	0,807446	-0,00995	0,341	0,343	1
Glmp	0,807585	-0,01681	0,638	0,639	1
Mcm5	0,8076	-0,00156	0,046	0,049	1
Akap8	0,807681	0,004045	0,413	0,41	1
Gm29361	0,807847	0,001052	0,013	0,011	1
Copb2	0,807984	0,018891	0,479	0,499	1
Rasa3	0,808018	0,018016	0,761	0,767	1
Gm36899	0,808067	0,000721	0,013	0,011	1
Tsg101	0,808096	0,004683	0,671	0,668	1
Gm21814	0,808152	-0,00055	0,015	0,016	1
Ccnt2	0,808241	0,013666	0,674	0,68	1
Gucd1	0,8084	0,007958	0,089	0,086	1
Comtd1	0,808834	-0,00487	0,223	0,228	1
Nup62	0,808918	0,0067	0,165	0,161	1
March2	0,808974	-0,01449	0,642	0,629	1
Fem1c	0,809534	0,012628	0,384	0,39	1
Uspl1	0,809573	0,010136	0,333	0,328	1
Cnga1	0,809607	-0,00025	0,013	0,011	1
Xylb	0,809607	-0,00025	0,013	0,011	1
Nubp1	0,810101	-0,01488	0,168	0,171	1

Vps41	0,810132	0,011034	0,634	0,621	1
Hook2	0,810159	0,012361	0,051	0,049	1
Lamtor5	0,810239	0,002139	0,679	0,685	1
Cwc27	0,810414	-0,00308	0,284	0,291	1
Ipo8	0,810488	0,006537	0,348	0,345	1
Klc3	0,810711	-0,00055	0,013	0,011	1
Zfp637	0,810924	0,004274	0,455	0,45	1
Lingo2	0,810933	0,000866	0,043	0,041	1
Fam120aos	0,811002	-0,00339	0,051	0,054	1
Zfp518b	0,811053	-0,00238	0,062	0,059	1
Mutyh	0,811117	-0,0049	0,023	0,024	1
Orai1	0,811253	0,005054	0,169	0,164	1
Bcas3os1	0,811372	-0,00215	0,013	0,011	1
Pfkfb2	0,811393	0,008546	0,083	0,088	1
Gm48420	0,811589	-0,00211	0,02	0,018	1
Mob2	0,81162	0,023225	0,384	0,389	1
Selenom	0,81175	-0,00811	0,968	0,972	1
D17Wsu92	0,811946	-0,00191	0,563	0,58	1
Ptcra	0,812034	-0,01536	0,013	0,011	1
Pfkm	0,812076	-0,0024	0,023	0,024	1
0610010F(	0,81231	0,003896	0,245	0,254	1
Zfp524	0,812367	0,00382	0,229	0,236	1
Sfrp1	0,81265	7,55E-06	0,043	0,041	1
Per3	0,812798	0,027876	0,236	0,234	1
Nkapl	0,813097	0,006183	0,033	0,036	1
Pnpla2	0,813183	0,011926	0,358	0,354	1
Trim37	0,813195	0,005596	0,149	0,154	1
Mast2	0,813262	0,009445	0,235	0,229	1
Zfp707	0,813443	0,006763	0,088	0,093	1
Nek1	0,813475	0,011781	0,208	0,203	1
Lsg1	0,813529	0,004753	0,337	0,346	1
Rrp36	0,813649	0,009684	0,358	0,372	1
Ddit3	0,813727	0,069498	0,676	0,688	1
Rras	0,813826	0,009962	0,097	0,094	1
A030001D:	0,813879	-0,00233	0,1	0,104	1
Upf1	0,814009	-0,01284	0,473	0,48	1
Foxo4	0,814055	0,005596	0,154	0,15	1
Fkbpl	0,814072	-0,00781	0,086	0,089	1
Gm26519	0,814291	0,006809	0,033	0,036	1
Hspb6	0,814706	0,00648	0,063	0,06	1
Ndufa7	0,814741	0,01352	0,937	0,928	1
Eny2	0,814934	0,004252	0,673	0,681	1
Lpin2	0,814976	0,004357	0,058	0,055	1
Grsf1	0,8155	-0,0146	0,17	0,172	1
Tmem151a	0,815707	-0,01005	0,998	0,997	1
Lysmd4	0,815736	-0,00486	0,135	0,13	1
Btd	0,816098	-0,00459	0,152	0,156	1
Usp5	0,81615	0,007344	0,594	0,597	1
Pdss1	0,816202	-0,00505	0,064	0,067	1
Fryl	0,816281	0,025073	0,857	0,863	1
Bckdk	0,816396	0,003748	0,305	0,314	1
Synpr	0,816469	-0,00718	0,03	0,033	1

Arhgap35	0,816632	0,00319	0,402	0,411	1
Usp24	0,816913	0,010038	0,341	0,353	1
Guf1	0,816928	0,006407	0,175	0,171	1
Pwwp2a	0,817013	0,014921	0,408	0,407	1
Zfp408	0,817034	0,00069	0,06	0,057	1
Arhgap15	0,817196	0,002187	0,09	0,094	1
Wipf1	0,817257	-0,00772	0,568	0,567	1
Flot1	0,817498	-0,03468	0,327	0,327	1
Nsmce3	0,817536	-0,00762	0,569	0,567	1
Esyt1	0,817586	0,002537	0,065	0,062	1
Khdrbs1	0,817647	-0,00083	0,806	0,818	1
Gmps	0,817688	0,009356	0,402	0,416	1
H2afy2	0,817722	0,008865	0,082	0,08	1
Stard7	0,81794	0,013556	0,449	0,45	1
Lhfpl4	0,817988	-0,00332	0,165	0,169	1
Gm17018	0,817996	0,02269	0,329	0,33	1
Mroh1	0,818087	0,009171	0,155	0,151	1
Leo1	0,818116	-0,00335	0,316	0,319	1
Ube2l3	0,818209	0,011514	0,942	0,946	1
Zfp169	0,818283	0,006155	0,114	0,119	1
Nptxr	0,818292	0,008671	0,041	0,039	1
Pofut2	0,818329	0,006104	0,313	0,309	1
Aox1	0,818826	-0,00474	0,035	0,037	1
Kctd15	0,818984	0,003553	0,099	0,096	1
Dhx8	0,819103	0,008203	0,211	0,207	1
Mrps17	0,819201	0,009655	0,486	0,502	1
Bbs10	0,819322	0,000468	0,023	0,024	1
Ptpn12	0,819366	0,015275	0,202	0,2	1
Mprip	0,819546	0,011367	0,671	0,673	1
Terf1	0,81964	0,000804	0,281	0,275	1
Gnpda2	0,819664	0,006488	0,398	0,411	1
Parp1	0,819739	-0,0491	0,344	0,354	1
2810405F1	0,820089	0,007108	0,046	0,044	1
Gtf2h5	0,820354	0,007297	0,763	0,771	1
Sept2	0,820477	0,002691	0,189	0,195	1
Srrm1	0,820491	-0,00254	0,943	0,941	1
Rnaseh2c	0,820586	0,025083	0,546	0,561	1
Prdm9	0,820609	0,00301	0,023	0,024	1
Yipf5	0,820658	0,007194	0,598	0,592	1
Zmiz2	0,820717	0,00404	0,415	0,421	1
Tpt1	0,820906	0,01588	1	1	1
Supt7l	0,821251	0,006915	0,207	0,213	1
Ikzf5	0,821649	-0,01237	0,43	0,441	1
Gm20219	0,821759	0,000819	0,03	0,033	1
Apba2	0,821969	0,002028	0,128	0,124	1
Gsto1	0,822112	-0,00376	0,162	0,156	1
Cap1	0,822217	0,000126	0,598	0,584	1
Efl1	0,822313	0,011113	0,211	0,208	1
Gm26982	0,822352	0,001098	0,025	0,023	1
Wdr90	0,822355	-0,00016	0,025	0,023	1
Orai3	0,822403	-0,00889	0,131	0,133	1
Vmac	0,822475	0,003962	0,157	0,163	1

Pacs2	0,82263	0,020213	0,993	0,998	1
Smu1	0,822681	0,00837	0,486	0,486	1
Klf9	0,822817	0,032746	0,792	0,798	1
Gm26514	0,822848	-0,00312	0,012	0,013	1
Tspan3	0,822863	0,000362	0,968	0,972	1
Bach1	0,822877	0,00928	0,361	0,371	1
Gar1	0,822967	0,001681	0,309	0,312	1
Cbarp	0,823021	0,001143	0,03	0,033	1
Stard10	0,823268	-0,00837	0,296	0,283	1
B3gnt2	0,823409	0,005554	0,029	0,028	1
Fam45a	0,82345	0,003253	0,374	0,369	1
Gas2l3	0,823511	0,002345	0,012	0,013	1
Fam122a	0,823556	-0,00614	0,249	0,254	1
Dctn6	0,823839	0,012461	0,597	0,589	1
Jph4	0,823936	0,046315	0,688	0,699	1
Taf1b	0,823969	0,002541	0,222	0,228	1
Srm	0,824054	0,024965	0,355	0,363	1
Tmem218	0,824086	-0,00171	0,069	0,072	1
Mtg2	0,824372	0,014853	0,246	0,254	1
Borcs8	0,824484	0,005351	0,21	0,205	1
Actr1b	0,824543	0,015986	0,399	0,42	1
Atp5h	0,824691	0,010691	0,988	0,995	1
Tma7	0,824808	0,000782	0,834	0,849	1
Polr3e	0,824818	0,010128	0,513	0,509	1
1500009C(	0,824838	-0,00248	0,012	0,013	1
Commd2	0,824917	-0,00202	0,291	0,298	1
Lpar1	0,825072	-0,01424	0,977	0,98	1
Fat1	0,825248	0,023832	0,379	0,384	1
Mrpl2	0,825277	0,015045	0,343	0,345	1
BC051226	0,82555	0,006697	0,166	0,163	1
Pfdn2	0,825553	0,004853	0,847	0,857	1
Noc4l	0,825664	0,000981	0,115	0,119	1
Cfap126	0,825722	-0,00185	0,012	0,013	1
Cutc	0,825728	-0,0031	0,081	0,085	1
Rps4x	0,82575	0,003905	0,982	0,979	1
Ang	0,825773	-0,01157	0,045	0,047	1
Zfp607b	0,82581	-0,00944	0,126	0,128	1
Zfp106	0,825844	-0,00959	0,466	0,47	1
Ptprk	0,825858	0,007045	0,532	0,553	1
Cdc40	0,82589	-0,00324	0,439	0,452	1
A630072M	0,8259	0,002522	0,063	0,06	1
Ist1	0,826214	-0,03207	0,578	0,554	1
Rnf2	0,826238	0,012032	0,141	0,138	1
Gm14409	0,826253	0,002624	0,004	0,003	1
Irs3	0,826253	0,002624	0,004	0,003	1
Gm45833	0,826253	0,002624	0,004	0,003	1
Pde8b	0,826253	0,002624	0,004	0,003	1
Gm16222	0,826253	0,004573	0,004	0,003	1
Cmtm4	0,826309	0,005292	0,095	0,099	1
Rabif	0,826476	0,002265	0,226	0,233	1
1110006O:	0,826606	-0,00122	0,012	0,013	1
Zfp459	0,826606	-0,00122	0,012	0,013	1

Trmt44	0,82685	-0,00436	0,074	0,076	1
Tmem151b	0,827058	0,003599	0,004	0,003	1
Gm48342	0,827058	0,002624	0,004	0,003	1
Gm26583	0,827058	0,002624	0,004	0,003	1
Gm5475	0,827058	0,002624	0,004	0,003	1
Cldn8	0,827058	0,002624	0,004	0,003	1
Tmem182	0,827058	0,001648	0,004	0,003	1
Olfr366	0,827058	0,001648	0,004	0,003	1
Ptgfrn	0,827058	0,001648	0,004	0,003	1
Wipf3	0,827058	0,001648	0,004	0,003	1
Gm44662	0,827058	0,001648	0,004	0,003	1
Phxr2	0,827058	0,001648	0,004	0,003	1
Gm35166	0,827058	0,001648	0,004	0,003	1
Islr	0,827058	0,001648	0,004	0,003	1
Slc5a10	0,827058	0,001648	0,004	0,003	1
Serpinf2	0,827058	0,001648	0,004	0,003	1
Ppm1e	0,827058	0,001648	0,004	0,003	1
Gpr22	0,827058	0,001648	0,004	0,003	1
Fkbp11	0,827058	0,001648	0,004	0,003	1
P3h2	0,827058	0,001648	0,004	0,003	1
Popdc2	0,827058	0,001648	0,004	0,003	1
AC159200.	0,827058	0,001648	0,004	0,003	1
Hoxb3os	0,827058	0,003283	0,004	0,003	1
A430005L1	0,827239	0,001545	0,298	0,306	1
Pten	0,82763	-0,00187	0,643	0,646	1
Srp68	0,827657	0,007383	0,377	0,387	1
Uhrf2	0,827706	-0,01744	0,525	0,524	1
Gm15892	0,827714	0,001052	0,012	0,013	1
Ftl1-ps1	0,827794	0,001519	0,166	0,172	1
Igfbp2	0,827862	0,000671	0,004	0,003	1
Gm31728	0,827862	0,000671	0,004	0,003	1
4930447M	0,827862	0,000671	0,004	0,003	1
Zfp385b	0,827862	0,000671	0,004	0,003	1
Olfr1260	0,827862	0,000671	0,004	0,003	1
Bbox1	0,827862	0,000671	0,004	0,003	1
Gm14435	0,827862	0,000671	0,004	0,003	1
Bex4	0,827862	0,000671	0,004	0,003	1
Gm42812	0,827862	0,000671	0,004	0,003	1
1700022I1	0,827862	0,000671	0,004	0,003	1
Gm13212	0,827862	0,000671	0,004	0,003	1
Cort	0,827862	0,000671	0,004	0,003	1
Dnah10	0,827862	0,000671	0,004	0,003	1
Gm44287	0,827862	0,000671	0,004	0,003	1
Pirb	0,827862	0,000671	0,004	0,003	1
Vmn1r76	0,827862	0,000671	0,004	0,003	1
Gm9844	0,827862	0,000671	0,004	0,003	1
B230208H1	0,827862	0,000671	0,004	0,003	1
Gm20597	0,827862	0,000671	0,004	0,003	1
Syt1	0,827862	0,000671	0,004	0,003	1
F420014N2	0,827862	0,000671	0,004	0,003	1
Gm26768	0,827862	0,000671	0,004	0,003	1
Cyp4v3	0,827862	0,000671	0,004	0,003	1

Disc1	0,827862	0,000671	0,004	0,003	1
Gm10873	0,827862	0,000671	0,004	0,003	1
Gm33054	0,827862	0,000671	0,004	0,003	1
Gm5431	0,827862	0,000671	0,004	0,003	1
Zfp750	0,827862	0,000671	0,004	0,003	1
AK157302	0,827862	0,000671	0,004	0,003	1
Gm2762	0,827862	0,000671	0,004	0,003	1
Gm48116	0,827862	0,000671	0,004	0,003	1
Acot12	0,827862	0,000671	0,004	0,003	1
Gm26520	0,827862	0,000671	0,004	0,003	1
Gm49227	0,827862	0,000671	0,004	0,003	1
Angptl4	0,827862	0,000671	0,004	0,003	1
Gm10093	0,827862	0,000671	0,004	0,003	1
Colec12	0,827862	0,000671	0,004	0,003	1
Mppe1	0,827862	0,000671	0,004	0,003	1
Capn1	0,827862	0,000671	0,004	0,003	1
Cyb561a3	0,827862	0,000671	0,004	0,003	1
Pdzd8	0,827907	-0,02139	0,473	0,473	1
Atp5g1	0,828056	-0,0029	0,948	0,945	1
Gm4279	0,828093	-0,00112	0,025	0,023	1
Gm47117	0,828265	0,000363	0,004	0,003	1
Pcgf2	0,828265	0,000363	0,004	0,003	1
Iqch	0,828378	0,000721	0,012	0,013	1
Gm20632	0,828378	-0,00025	0,012	0,013	1
Ube2q1	0,828462	0,004135	0,457	0,468	1
Gm2814	0,828668	2,86E-05	0,004	0,003	1
1700067K(	0,828668	2,86E-05	0,004	0,003	1
Zfp870	0,828901	-2E-05	0,048	0,046	1
Crot	0,828928	-0,00212	0,295	0,304	1
Tmem169	0,829039	0,00109	0,079	0,076	1
Trappc10	0,829195	0,000874	0,843	0,868	1
Cldn12	0,829271	0,002023	0,082	0,086	1
Kcnn1	0,829398	0,008387	0,034	0,033	1
Cand2	0,829473	-0,00095	0,004	0,003	1
Mia	0,829473	-0,00095	0,004	0,003	1
Ppil6	0,829473	-0,00095	0,004	0,003	1
Ttc36	0,829473	-0,00095	0,004	0,003	1
Dhx58	0,829473	-0,00095	0,004	0,003	1
Six4	0,829473	-0,00095	0,004	0,003	1
Dlec1	0,829473	-0,00257	0,004	0,003	1
Rnase1	0,829486	0,001052	0,012	0,013	1
Tpd52l2	0,829524	-0,00097	0,657	0,66	1
Syn1	0,829559	-0,00301	0,113	0,117	1
Elf1	0,829652	0,032233	0,309	0,312	1
Mta3	0,829668	-0,00805	0,554	0,559	1
Blmh	0,829906	0,002725	0,532	0,533	1
Zkscan7	0,829926	-0,00524	0,101	0,104	1
Etfbkmt	0,830107	0,004923	0,029	0,028	1
Zfp78	0,830107	0,004923	0,029	0,028	1
C1ql1	0,83015	0,000721	0,012	0,013	1
Nppc	0,830342	-0,00937	0,161	0,156	1
Sec63	0,830388	0,016318	0,635	0,629	1

Cryl1	0,830595	0,00427	0,012	0,013	1
Setd1b	0,830645	0,001246	0,389	0,397	1
Chd1	0,830784	0,021187	0,372	0,377	1
Gm45902	0,830931	0,010858	0,039	0,037	1
Atpaf2	0,830958	-0,00348	0,102	0,099	1
Ccndbp1	0,830973	0,017951	0,635	0,644	1
9330160F1	0,831025	0,008376	0,034	0,033	1
Bpifb4	0,831084	-0,00257	0,004	0,003	1
1700096K1	0,831378	0,005871	0,189	0,185	1
Rai1	0,831681	-0,00201	0,126	0,13	1
Zfp638	0,831743	-0,01325	0,81	0,813	1
Taf13	0,831772	0,011397	0,555	0,564	1
Gm21847	0,831923	0,003621	0,012	0,013	1
Asf1b	0,831923	0,003621	0,012	0,013	1
Mettl9	0,832031	-0,0157	0,596	0,584	1
Cwc25	0,832225	-0,00198	0,32	0,325	1
Lrrc27	0,832423	0,001381	0,018	0,016	1
C030037D0	0,832423	0,001381	0,018	0,016	1
Calr3	0,832425	0,000113	0,018	0,016	1
Zfp647	0,832506	-0,00072	0,035	0,037	1
Fxyd4	0,832559	-0,00303	0,025	0,023	1
Mettl1	0,832579	0,00848	0,269	0,278	1
Trib2	0,832648	-0,00098	0,035	0,037	1
Lrrc8c	0,832757	-0,002	0,343	0,335	1
Nudcd1	0,832798	0,005588	0,132	0,13	1
Gm15462	0,833033	0,002982	0,012	0,013	1
Nup54	0,833087	0,004831	0,222	0,218	1
Ier3	0,833329	0,009568	0,034	0,033	1
Rapgef3	0,833552	-0,01915	0,171	0,172	1
Nsd1	0,83361	0,011	0,689	0,694	1
Zfp273	0,833629	0,003522	0,092	0,096	1
Ciz1	0,833637	-0,0052	0,237	0,239	1
Camta2	0,833914	0,005607	0,197	0,203	1
Mttp	0,833957	-0,00132	0,112	0,115	1
Rb1cc1	0,834274	0,022629	0,754	0,771	1
Kirrel3	0,834455	0,028953	0,17	0,167	1
Shroom2	0,834506	0,014255	0,585	0,587	1
Men1	0,834606	0,00264	0,258	0,265	1
Usp33	0,834608	0,001759	0,362	0,354	1
Set	0,834865	0,006234	0,963	0,958	1
Aagab	0,834978	0,001847	0,184	0,18	1
Atp5j	0,835038	0,010809	0,969	0,972	1
Ndufs2	0,835327	-0,0114	0,853	0,867	1
Eid2	0,835436	-0,00161	0,077	0,08	1
4921524J1	0,835457	0,003977	0,173	0,169	1
Ascc2	0,83551	-0,00054	0,215	0,221	1
9630013A2	0,83561	0,024062	0,011	0,01	1
Gm13905	0,835682	-0,00082	0,02	0,021	1
Plek	0,835852	-0,00052	0,02	0,021	1
Fam161b	0,835966	0,005316	0,143	0,148	1
Mrpl57	0,835973	0,006666	0,421	0,431	1
Fndc4	0,836014	0,001252	0,048	0,05	1

Fbxo22	0,836335	-0,01228	0,289	0,289	1
Sox13	0,836639	0,001918	0,064	0,067	1
Tmem150a	0,836953	-0,00682	0,296	0,299	1
Tfam	0,836998	-0,0118	0,358	0,346	1
A730015C:	0,837052	0,005871	0,011	0,01	1
Utrn	0,837052	0,009728	0,011	0,01	1
Rptor	0,837228	0,003339	0,236	0,234	1
Gm2000	0,837331	-0,0044	0,098	0,101	1
Brsk2	0,837354	0,020932	0,34	0,354	1
Mgll	0,837406	0,020979	0,245	0,257	1
Zfp85	0,837553	0,003089	0,051	0,049	1
Dhps	0,837639	0,007454	0,426	0,442	1
Gm4673	0,837667	0,014703	0,184	0,18	1
Fbxw16	0,837691	-0,00082	0,018	0,016	1
Eepd1	0,83772	0,004965	0,072	0,075	1
Hspa12a	0,837805	-0,01007	0,037	0,039	1
Bdp1	0,838009	0,057239	0,352	0,359	1
Sfn	0,838495	0,002968	0,011	0,01	1
Gm5577	0,838495	0,002968	0,011	0,01	1
Gm48914	0,838495	0,002968	0,011	0,01	1
Gm5427	0,838495	0,003937	0,011	0,01	1
Zfp382	0,838599	0,011386	0,059	0,057	1
Fam118b	0,838681	0,004734	0,135	0,132	1
Pcgf1	0,838925	0,00264	0,104	0,101	1
Pabpn1	0,839026	0,020928	0,801	0,829	1
Gm28041	0,839152	0,00077	0,02	0,021	1
Atp5b	0,839211	0,007176	0,997	0,995	1
Surf6	0,839234	0,00325	0,204	0,2	1
Uvssa	0,839681	-0,00702	0,258	0,262	1
D730003I1	0,839683	0,014138	0,237	0,236	1
Rag1	0,839699	0,002328	0,011	0,01	1
Gm16192	0,839938	0,001999	0,011	0,01	1
1700105P(	0,839938	0,001999	0,011	0,01	1
Zfyve21	0,840149	0,027684	0,464	0,475	1
Zzz3	0,840503	0,011002	0,57	0,563	1
Ranbp3	0,840504	-0,0123	0,358	0,359	1
Ankmy2	0,840536	-0,0033	0,14	0,145	1
Lrrc58	0,840563	0,009178	0,911	0,914	1
Skp2	0,840631	-0,00534	0,083	0,086	1
Id2	0,840642	0,033464	0,103	0,107	1
Pfkfb4	0,840814	0,007194	0,18	0,176	1
Gm38534	0,840889	-0,00178	0,018	0,016	1
Creb3l2	0,840921	0,025724	0,536	0,533	1
1700040D:	0,841143	0,001359	0,011	0,01	1
Gm43567	0,841143	0,001359	0,011	0,01	1
Gm340	0,841162	0,012795	0,07	0,073	1
Hoxd4	0,841382	0,001028	0,011	0,01	1
Zfp609	0,841387	0,01281	0,267	0,263	1
Abhd14a	0,841496	-0,00345	0,488	0,493	1
Plcb3	0,841627	0,000194	0,027	0,029	1
Lrrtm2	0,841845	0,010694	0,251	0,247	1
1110008P:	0,841994	0,006109	0,289	0,286	1

Kdm5b	0,842042	0,013343	0,387	0,389	1
Becn1	0,842187	-0,01327	0,412	0,407	1
Lman2	0,84224	0,010036	0,565	0,563	1
Eme2	0,842245	0,00209	0,034	0,033	1
Tmem217	0,842347	0,002328	0,011	0,01	1
Shroom4	0,842347	0,000721	0,011	0,01	1
Sprtn	0,842439	0,009961	0,175	0,172	1
C730034F(	0,842458	0,008632	0,059	0,057	1
Usp20	0,842464	0,000656	0,205	0,21	1
Igf2bp3	0,84257	-0,00099	0,117	0,12	1
Sh3d21	0,842587	0,00039	0,011	0,01	1
M6pr	0,842608	-0,00648	0,864	0,865	1
Gnptab	0,842736	0,010189	0,615	0,642	1
Gm32568	0,842828	-0,00122	0,011	0,01	1
Zfp654	0,842838	0,00427	0,185	0,19	1
2510046G:	0,843156	-0,00131	0,04	0,042	1
Trappc9	0,843317	0,006319	0,342	0,351	1
Chic1	0,843586	-0,01295	0,241	0,244	1
Gm47438	0,843604	0,004598	0,023	0,021	1
Sec61a1	0,843622	0,018768	0,636	0,626	1
Cep97	0,843623	0,002505	0,612	0,631	1
Cenpu	0,844032	-0,00058	0,011	0,01	1
Rasd2	0,844032	-0,00058	0,011	0,01	1
Ccdc85b	0,844042	0,007496	0,532	0,541	1
Cask	0,844095	-0,00147	0,282	0,286	1
Cul4a	0,84438	0,021341	0,356	0,358	1
Tmem147	0,844426	0,008515	0,754	0,784	1
Cbx3	0,844547	0,002722	0,933	0,963	1
Hps6	0,844602	-0,00395	0,05	0,052	1
Pds5b	0,844618	0,020591	0,468	0,468	1
Relb	0,844659	0,002518	0,142	0,146	1
Nsf	0,844663	0,018474	0,269	0,268	1
Cisd3	0,844841	0,0122	0,204	0,202	1
Mff	0,845005	0,015802	0,761	0,79	1
Mosmo	0,845029	0,006073	0,414	0,431	1
Ghdc	0,84503	-0,01105	0,208	0,21	1
Insig2	0,845072	0,001876	0,349	0,361	1
Eif4g1	0,845088	-0,00557	0,754	0,774	1
Prkag2	0,845104	-0,00262	0,032	0,034	1
Lias	0,845161	0,004771	0,353	0,363	1
Slc35b4	0,845214	0,003377	0,251	0,247	1
Cdkn2a	0,84541	-0,00907	0,017	0,018	1
Npr2	0,845438	0,00301	0,023	0,021	1
Faap20	0,845482	0,001678	0,142	0,146	1
Gm45495	0,845551	-0,00341	0,079	0,076	1
Larp7	0,845801	0,021791	0,258	0,272	1
Cln5	0,845809	0,013228	0,42	0,423	1
Kmt5c	0,845812	-0,00153	0,162	0,166	1
Lats1	0,845833	0,0013	0,362	0,353	1
Casd1	0,845967	0,007589	0,255	0,25	1
Abraxas2	0,846205	0,003896	0,255	0,263	1
Pex14	0,846477	-0,00396	0,27	0,273	1

Foxn3	0,8465	0,014292	0,996	1	1
Mlkl	0,846538	-0,00596	0,017	0,018	1
Strbp	0,846693	0,010528	0,242	0,239	1
Mtss1l	0,846723	0,001879	0,173	0,177	1
Fam49a	0,846753	-0,00316	0,009	0,01	1
Tor1a	0,84702	0,007882	0,371	0,367	1
Eif2a	0,847476	0,006808	0,487	0,48	1
Mpdu1	0,847519	0,010281	0,511	0,512	1
Cfap74	0,847522	-0,00252	0,009	0,01	1
Dcbld1	0,847522	-0,00252	0,009	0,01	1
C1qbp	0,847567	0,010737	0,597	0,613	1
Zfp37	0,847677	-0,01261	0,264	0,263	1
Smad1	0,847809	0,005395	0,281	0,276	1
Rnf139	0,848008	0,016883	0,329	0,325	1
Kdelc2	0,848359	0,004009	0,044	0,042	1
Ing1	0,848384	0,021184	0,411	0,411	1
Cdc42ep2	0,848424	-0,0019	0,963	0,959	1
Myo10	0,848435	0,00014	0,023	0,021	1
Prtg	0,848503	0,016163	0,106	0,104	1
Tmem181a	0,848692	0,015036	0,357	0,366	1
Ndufaf2	0,848721	0,003336	0,545	0,566	1
Caskin1	0,848725	-0,00394	0,025	0,026	1
Anapc15	0,848999	0,004767	0,153	0,15	1
Gdpd5	0,849068	0,003988	0,388	0,398	1
Tnpo3	0,849089	0,00443	0,487	0,494	1
Gm43518	0,849319	0,00039	0,009	0,01	1
Kcna4	0,849437	0,002054	0,023	0,021	1
Fcor	0,849817	0,00939	0,443	0,444	1
Nebl	0,849832	-0,00092	0,009	0,01	1
Gm14508	0,849832	-0,00092	0,009	0,01	1
Arhgef25	0,849832	-0,00092	0,009	0,01	1
Ewsr1	0,849924	-0,00611	0,827	0,805	1
Wbp4	0,849964	0,014054	0,559	0,569	1
Golga3	0,850028	0,022024	0,321	0,322	1
Cyb5r1	0,850056	0,012448	0,223	0,229	1
Emc9	0,850479	-0,00956	0,281	0,283	1
Arl3	0,850896	0,011447	0,596	0,61	1
Cdc26	0,850999	0,005097	0,476	0,494	1
Tbc1d31	0,851116	0,00817	0,115	0,112	1
Lgals3	0,851116	0,049736	0,064	0,062	1
Zfp473	0,851116	0,005228	0,009	0,01	1
Gm15265	0,851373	0,002968	0,009	0,01	1
Fam110b	0,851373	5,69E-05	0,009	0,01	1
Fbxo47	0,851373	5,69E-05	0,009	0,01	1
5730507C(	0,851373	5,69E-05	0,009	0,01	1
Pnma1	0,851373	5,69E-05	0,009	0,01	1
R3hdm4	0,851468	0,001239	0,642	0,621	1
Bcor	0,851633	-0,00044	0,248	0,254	1
Ypel1	0,851943	0,003127	0,061	0,063	1
Prkcq	0,851964	-0,00931	0,429	0,428	1
Crtc1	0,85201	0,003889	0,131	0,135	1
Fam173b	0,852216	0,009558	0,2	0,207	1

Prrg2	0,852225	-0,0041	0,154	0,158	1
Nfe2l3	0,852589	-0,01541	0,626	0,624	1
Nepro	0,852719	-0,0064	0,055	0,057	1
Zfp316	0,852752	0,002653	0,187	0,184	1
Zfp941	0,852764	-0,00307	0,017	0,018	1
Gm26538	0,852914	0,003937	0,009	0,01	1
Gm10160	0,852914	0,001999	0,009	0,01	1
Wnk1	0,853141	0,006247	0,999	0,998	1
9630009A(	0,853193	-3,8E-05	0,092	0,089	1
Gins2	0,853305	0,007631	0,1	0,104	1
Taf9b	0,853357	0,004539	0,493	0,498	1
Gstm6	0,85341	0,00173	0,144	0,141	1
AL591952.	0,853421	-0,00508	0,037	0,039	1
Npm1	0,853732	0,028156	0,933	0,906	1
Ccpg1	0,853812	0,016636	0,307	0,304	1
Mia2	0,853835	0,009046	0,568	0,593	1
Shroom3	0,853956	0,008552	0,158	0,154	1
Otub1	0,854045	0,001438	0,606	0,607	1
Cdadc1	0,854323	-0,00505	0,41	0,415	1
Ap5s1	0,854572	-0,00566	0,118	0,12	1
Zfp322a	0,854645	0,02038	0,208	0,215	1
Zfp84	0,85468	0,008489	0,199	0,205	1
Tmem132c	0,854704	0,008263	0,077	0,08	1
Ccdc112	0,854741	0,01296	0,184	0,184	1
Acvr2a	0,854747	-0,01152	0,107	0,109	1
Hadhb	0,854782	0,015286	0,733	0,751	1
Zdhhc2	0,854892	0,013285	0,047	0,046	1
Fhit	0,854987	0,003152	0,076	0,073	1
Map3k4	0,855282	-0,02026	0,373	0,369	1
Spin1	0,85534	-0,01255	0,237	0,239	1
Ftsj3	0,855342	0,008694	0,292	0,288	1
Upf2	0,855392	0,006425	0,459	0,481	1
Zfp846	0,855511	0,012302	0,205	0,203	1
Bin3	0,855572	-0,00226	0,172	0,176	1
Gm11508	0,855773	0,002072	0,025	0,026	1
Zfp414	0,855809	0,019415	0,253	0,252	1
Papd4	0,85583	0,017677	0,302	0,304	1
Rad54l	0,855979	-0,0006	0,045	0,047	1
Nuak2	0,855998	0,002968	0,009	0,01	1
Gm31763	0,855998	0,002968	0,009	0,01	1
Fam120a	0,856249	0,013914	0,612	0,595	1
Sec31a	0,856407	0,006641	0,456	0,47	1
Ncbp1	0,856505	0,01455	0,254	0,254	1
Auts2	0,85651	0,012055	0,645	0,65	1
Xrn2	0,856981	0,022775	0,515	0,52	1
Gpr155	0,85706	0,003183	0,644	0,663	1
Rps6kb2	0,857173	0,016382	0,224	0,224	1
Sympk	0,857284	-0,01484	0,521	0,527	1
Mettl8	0,857312	0,00614	0,169	0,174	1
Mon2	0,857314	0,002489	0,693	0,696	1
Nol7	0,857654	0,016507	0,803	0,784	1
Gtdc1	0,858211	0,020912	0,237	0,237	1

Mastl	0,858223	-0,00502	0,042	0,044	1
Tmco4	0,858321	0,003973	0,027	0,026	1
Pes1	0,858569	0,001034	0,28	0,275	1
Spns2	0,858822	0,015878	0,591	0,59	1
Ttc9c	0,85896	0,013117	0,272	0,27	1
Syk	0,858997	0,005231	0,016	0,015	1
Kcnk1	0,859009	0,000274	0,045	0,047	1
Hspa14	0,859032	0,021085	0,314	0,319	1
Rin2	0,859067	0,031072	0,256	0,26	1
Eif2s1	0,859137	0,016738	0,476	0,48	1
Bloc1s5	0,859187	0,022628	0,25	0,252	1
Arhgap20	0,859226	0,002395	0,025	0,026	1
Dynlt1b	0,859596	0,004909	0,016	0,015	1
CAAA0111	0,859815	0,005871	0,264	0,27	1
1810009A	0,859892	0,001825	0,044	0,042	1
Gpr62	0,860169	-0,01048	0,884	0,889	1
Arrb1	0,860264	0,015536	0,062	0,065	1
Nova2	0,86032	0,000746	0,017	0,018	1
Rgs12	0,860476	0,001424	0,025	0,026	1
Ldb1	0,860555	0,025325	0,298	0,304	1
Lmbr1l	0,86057	0,003782	0,085	0,083	1
Gm29562	0,860597	0,005231	0,016	0,015	1
Pank4	0,86061	0,015474	0,109	0,107	1
Brd4	0,860613	0,016758	0,636	0,668	1
Lrfn4	0,860792	0,007624	0,098	0,096	1
Cog7	0,861147	0,004742	0,327	0,333	1
Rpp14	0,861203	-0,01174	0,275	0,275	1
Lrat	0,861395	0,003946	0,016	0,015	1
Ccs	0,861412	-0,00299	0,447	0,437	1
Zbtb38	0,86151	-0,00415	0,825	0,824	1
Slc35e3	0,861561	0,007971	0,206	0,203	1
Snx11	0,861572	0,029846	0,278	0,293	1
Bcan	0,861646	0,005871	0,017	0,018	1
Tmem14c	0,861696	0,010073	0,664	0,68	1
Gm17056	0,8618	-0,01335	0,023	0,021	1
Agtrap	0,861821	0,002611	0,09	0,088	1
Thrb	0,861835	0,003955	0,017	0,018	1
Zfp513	0,86201	-0,00156	0,161	0,156	1
Vapb	0,862078	-0,00635	0,59	0,595	1
Fam241b	0,862213	0,002886	0,09	0,088	1
Abt1	0,862287	0,000573	0,203	0,208	1
Aldh18a1	0,862402	0,001709	0,017	0,018	1
Dnajc5	0,862507	0,012399	0,546	0,545	1
Med30	0,862526	-0,00195	0,579	0,597	1
Ankrd46	0,862591	0,011917	0,688	0,707	1
Npc1	0,862615	0,016141	0,991	0,987	1
Endov	0,862783	-0,00444	0,124	0,127	1
Syt3	0,863156	0,003115	0,054	0,052	1
Usp50	0,86319	-0,01029	0,26	0,262	1
Mtmr3	0,86321	0,020072	0,305	0,307	1
Gm5602	0,863349	0,002036	0,017	0,018	1
Cryab	0,863671	0,002728	0,999	1	1

Cfl1	0,863919	0,006365	0,999	1	1
Sacm1l	0,863968	0,009399	0,411	0,423	1
Dhx35	0,864016	0,001567	0,05	0,052	1
Zfp148	0,864112	0,020432	0,515	0,53	1
Tefm	0,864194	0,000737	0,071	0,073	1
Aim2	0,864397	0,002345	0,016	0,015	1
Gm26516	0,864484	0,002671	0,017	0,018	1
Bend3	0,864484	0,002671	0,017	0,018	1
Trpt1	0,864614	0,001084	0,136	0,14	1
Rab11fip3	0,864683	0,009025	0,273	0,28	1
Tmc6	0,864755	0,003583	0,317	0,324	1
Gm32856	0,865029	0,001143	0,029	0,031	1
Prkaca	0,865062	0,007947	0,559	0,569	1
Zfp39	0,865075	0,004616	0,037	0,036	1
9930104L(	0,865098	-5,4E-05	0,135	0,138	1
Ramp2	0,865198	0,002671	0,016	0,015	1
Top3b	0,86524	0,021084	0,292	0,294	1
Sbf1	0,86557	-0,00098	0,864	0,88	1
Cep164	0,865637	0,012443	0,266	0,265	1
Keap1	0,865651	-0,00515	0,465	0,459	1
Dhx29	0,865723	0,002859	0,237	0,234	1
Eif3k	0,865806	0,008503	0,892	0,876	1
Ercc5	0,866168	0,013683	0,197	0,195	1
Zfp710	0,866317	0,005047	0,161	0,158	1
Mamld1	0,866321	0,012547	0,021	0,02	1
Cs	0,866372	0,006375	0,578	0,579	1
Nphp1	0,866388	0,001397	0,08	0,078	1
Trmu	0,866424	-0,00495	0,132	0,128	1
Gtf3c5	0,866441	0,004022	0,197	0,195	1
Evi5	0,866503	0,002611	0,079	0,081	1
Ccdc6	0,866557	0,005611	0,206	0,211	1
A230107N(	0,866598	-0,00118	0,016	0,015	1
Ccdc94	0,866741	0,000851	0,093	0,096	1
Zfp36l1	0,866846	0,009983	0,021	0,02	1
Vps39	0,866965	-0,00623	0,256	0,259	1
Ift80	0,86697	0,011363	0,238	0,246	1
Ly6g6e	0,867014	-0,00387	0,078	0,08	1
P3h3	0,8672	-0,03149	0,016	0,015	1
Rnft2	0,867288	0,00014	0,076	0,078	1
Rpl7l1	0,867418	0,014482	0,385	0,4	1
Marveld1	0,867419	0,001768	0,032	0,031	1
Tm9sf4	0,867814	0,008335	0,45	0,463	1
Col1a2	0,868207	-0,02854	0,103	0,099	1
Gatad2a	0,868263	0,016556	0,452	0,46	1
Ube2d3	0,868309	0,008168	0,964	0,958	1
Gm5148	0,86844	0,012034	0,052	0,05	1
Blm	0,868569	-0,00138	0,104	0,101	1
Pole3	0,868687	-0,0013	0,373	0,382	1
Gpx1	0,868709	0,056697	0,206	0,213	1
6030443J(	0,868729	0,003356	0,037	0,036	1
Dguok	0,869035	0,028993	0,467	0,48	1
Mfsd10	0,869397	0,008389	0,145	0,143	1

Gm26881	0,86941	-0,00111	0,068	0,07	1
Lrrc41	0,869464	0,012302	0,271	0,281	1
Fndc10	0,869691	0,001881	0,059	0,057	1
Gm38642	0,869723	0,004585	0,192	0,189	1
Pwp2	0,869765	-0,00529	0,1	0,102	1
Zfp970	0,869816	0,008098	0,021	0,02	1
Zfp729b	0,870063	0,004506	0,162	0,166	1
Dip2c	0,87011	0,013457	0,402	0,402	1
Cdipt	0,870287	0,005015	0,365	0,371	1
Etf1	0,870446	0,022481	0,451	0,463	1
Erlin2	0,870598	0,010779	0,37	0,38	1
Fkbp14	0,870737	-0,00396	0,11	0,112	1
Jcad	0,870739	-0,00871	0,07	0,072	1
Zfp68	0,870802	-0,00206	0,336	0,33	1
Ovca2	0,870803	0,001567	0,055	0,057	1
Rbm15b	0,870861	0,010762	0,217	0,213	1
Tmem120a	0,871062	0,003686	0,165	0,169	1
Utp14a	0,871149	8,05E-05	0,299	0,304	1
Nkx2-2os	0,871511	0,000544	0,034	0,036	1
Zswim5	0,871515	-0,00013	0,029	0,031	1
Ppp2cb	0,871581	-0,00344	0,263	0,257	1
Kansl3	0,871732	0,010806	0,305	0,302	1
Cdkn2c	0,871859	-0,00322	0,066	0,063	1
Wwp2	0,871871	0,008705	0,351	0,356	1
Hnrnph2	0,872019	0,002076	0,771	0,767	1
Cisd2	0,872285	0,012386	0,657	0,688	1
Mcl1	0,872635	0,016892	0,716	0,722	1
Gm12525	0,872657	0,00305	0,037	0,036	1
Gm15860	0,872715	-0,00513	0,034	0,036	1
Proser1	0,872828	0,004609	0,237	0,233	1
Krba1	0,872828	-9,3E-05	0,169	0,166	1
Il11	0,872855	0,007712	0,057	0,055	1
Hjurp	0,87286	-0,00149	0,334	0,34	1
Fbxo42	0,872929	0,002446	0,124	0,127	1
Slc39a9	0,873067	0,009602	0,308	0,315	1
Mtm1	0,873145	0,006786	0,314	0,32	1
Fam168b	0,873293	-0,01567	0,409	0,416	1
Fbxo11	0,87332	0,01273	0,426	0,439	1
Pak1ip1	0,873344	-0,00902	0,397	0,402	1
Rpl13a-ps1	0,87335	-0,00145	0,022	0,023	1
Rhbdd2	0,873586	-0,00171	0,299	0,302	1
Gm14322	0,8737	0,001787	0,037	0,036	1
Sbsn	0,873756	0,010336	0,153	0,151	1
Tmcc2	0,873833	-0,00244	0,896	0,912	1
Trmt61a	0,873943	0,004115	0,099	0,102	1
Atad5	0,874054	0,007904	0,099	0,102	1
Sirt6	0,874279	0,001064	0,136	0,14	1
Oxsr1	0,874416	0,022462	0,22	0,221	1
Gm13594	0,874451	-0,00156	0,049	0,047	1
Kdm1b	0,87464	0,00416	0,126	0,124	1
Tmem68	0,874688	0,010176	0,24	0,239	1
Slc25a53	0,875258	-0,00178	0,06	0,062	1

Ect2	0,875543	-0,00223	0,006	0,007	1
Mov10	0,875543	-0,00223	0,006	0,007	1
Gm17529	0,875543	-0,00223	0,006	0,007	1
Lrtm2	0,875568	0,007138	0,026	0,024	1
Atp5l	0,875672	0,002213	0,978	0,982	1
Mrgbp	0,875815	0,007947	0,214	0,22	1
Nsun2	0,875847	0,014397	0,422	0,429	1
Arhgef37	0,875897	-0,00086	0,059	0,057	1
Scn2a	0,875954	-0,0046	0,049	0,047	1
Cdh8	0,876057	0,013554	0,183	0,18	1
Wdr74	0,876065	-0,00632	0,234	0,236	1
Relt	0,876595	-0,00872	0,044	0,046	1
Rpl36a-ps1	0,876704	-0,00082	0,022	0,023	1
Pcolce	0,876806	-0,00125	0,006	0,007	1
Fbxl22	0,876806	-0,00125	0,006	0,007	1
Carmil1	0,876806	-0,00125	0,006	0,007	1
Lefty1	0,876808	0,002682	0,096	0,099	1
Hnrnpk	0,877005	0,015771	0,996	1	1
Atcayos	0,87705	0,002293	0,002	0,002	1
Pithd1	0,87706	-0,00556	0,702	0,701	1
Il6ra	0,877122	0,000696	0,006	0,007	1
Slc18a2	0,877122	-0,00028	0,006	0,007	1
Armcx2	0,877218	0,005061	0,178	0,174	1
Gm15577	0,877438	-0,00061	0,006	0,007	1
Gm44264	0,877438	-0,00061	0,006	0,007	1
Gm44700	0,877438	-0,00061	0,006	0,007	1
Gm45799	0,877438	-0,00061	0,006	0,007	1
Gm11175	0,877438	-0,00061	0,006	0,007	1
Gm10642	0,877438	-0,00061	0,006	0,007	1
Olfr318	0,877438	-0,00061	0,006	0,007	1
Arhgap27	0,877438	-0,00061	0,006	0,007	1
Cdhr2	0,877438	-0,00061	0,006	0,007	1
Zfp457	0,877438	-0,00061	0,006	0,007	1
Gm48610	0,877438	-0,00061	0,006	0,007	1
AC113595.	0,877438	-0,00061	0,006	0,007	1
Gm26749	0,877438	-0,00061	0,006	0,007	1
Slc15a3	0,877438	-0,00061	0,006	0,007	1
Frmd3	0,877626	0,005221	0,002	0,002	1
6030419C:	0,877626	0,004246	0,002	0,002	1
Col14a1	0,877626	0,00327	0,002	0,002	1
Fgl1	0,877626	0,002293	0,002	0,002	1
Sncg	0,877626	0,002293	0,002	0,002	1
Arg2	0,877626	0,002293	0,002	0,002	1
Fsbp	0,877626	0,001315	0,002	0,002	1
Orc1	0,877626	0,001315	0,002	0,002	1
Artn	0,877626	0,001315	0,002	0,002	1
Prom1	0,877626	0,001315	0,002	0,002	1
Ncf1	0,877626	0,001315	0,002	0,002	1
Fam174b	0,877626	0,001315	0,002	0,002	1
Ctsc	0,877626	0,001315	0,002	0,002	1
Ascl2	0,877626	0,001315	0,002	0,002	1
Cnn2	0,877626	0,001315	0,002	0,002	1

Rmst	0,877626	0,001315	0,002	0,002	1
Lrriq1	0,877626	0,001315	0,002	0,002	1
Arhgap10	0,877626	0,001315	0,002	0,002	1
Tbc1d9	0,877626	0,001315	0,002	0,002	1
Plod2	0,877626	0,001315	0,002	0,002	1
Myt1l	0,877626	0,001315	0,002	0,002	1
Mis18bp1	0,877626	0,001315	0,002	0,002	1
Lrr1	0,877626	0,001315	0,002	0,002	1
Efcab1	0,877626	0,001315	0,002	0,002	1
Syt7	0,877626	0,001315	0,002	0,002	1
Gm35339	0,877699	0,00361	0,009	0,008	1
Gm30074	0,877699	0,005548	0,009	0,008	1
Ahnak2	0,8777	0,014834	0,009	0,008	1
Thoc6	0,877863	0,021256	0,227	0,226	1
Cyfip1	0,877948	0,021292	0,538	0,564	1
Vmn1r81	0,87807	-0,00028	0,006	0,007	1
Doc2g	0,87807	5,69E-05	0,006	0,007	1
Get4	0,878094	0,010366	0,595	0,59	1
Naa10	0,878135	-0,00563	0,312	0,302	1
Otos	0,878201	0,000336	0,002	0,002	1
Ptpn7	0,878201	0,000336	0,002	0,002	1
Arhgap30	0,878201	0,000336	0,002	0,002	1
Camk1d	0,878201	0,000336	0,002	0,002	1
Sfmbt2	0,878201	0,000336	0,002	0,002	1
Entpd2	0,878201	0,000336	0,002	0,002	1
Egfl7	0,878201	0,000336	0,002	0,002	1
Scn1a	0,878201	0,000336	0,002	0,002	1
Chrna1os	0,878201	0,000336	0,002	0,002	1
Cst7	0,878201	0,000336	0,002	0,002	1
Ube2c	0,878201	0,000336	0,002	0,002	1
2010308F(	0,878201	0,000336	0,002	0,002	1
Gpr160	0,878201	0,000336	0,002	0,002	1
Crabp2	0,878201	0,000336	0,002	0,002	1
Ankrd34a	0,878201	0,000336	0,002	0,002	1
Ngf	0,878201	0,000336	0,002	0,002	1
Ptpn22	0,878201	0,000336	0,002	0,002	1
Alpk1	0,878201	0,000336	0,002	0,002	1
B230334C(	0,878201	0,000336	0,002	0,002	1
Lrrc7	0,878201	0,000336	0,002	0,002	1
Phf24	0,878201	0,000336	0,002	0,002	1
Bend5	0,878201	0,000336	0,002	0,002	1
Hpca	0,878201	0,000336	0,002	0,002	1
Gabra4	0,878201	0,000336	0,002	0,002	1
Gm10419	0,878201	0,000336	0,002	0,002	1
Myo1h	0,878201	0,000336	0,002	0,002	1
Cd8b1	0,878201	0,000336	0,002	0,002	1
Lrrtm4	0,878201	0,000336	0,002	0,002	1
Fbxo41	0,878201	0,000336	0,002	0,002	1
Gm26588	0,878201	0,000336	0,002	0,002	1
Catsperg1	0,878201	0,000336	0,002	0,002	1
Mir9-3hg	0,878201	0,000336	0,002	0,002	1
Trim34a	0,878201	0,000336	0,002	0,002	1

Mical2	0,878201	0,000336	0,002	0,002	1
Abracl	0,878201	0,000336	0,002	0,002	1
Mtfr2	0,878201	0,000336	0,002	0,002	1
Lyz1	0,878201	0,000336	0,002	0,002	1
Neil3	0,878201	0,000336	0,002	0,002	1
Trim67	0,878201	0,000336	0,002	0,002	1
Vstm4	0,878201	0,000336	0,002	0,002	1
Sox21	0,878201	0,000336	0,002	0,002	1
AB124611	0,878201	0,000336	0,002	0,002	1
Glb1l2	0,878201	0,000336	0,002	0,002	1
Olfr986	0,878201	0,000336	0,002	0,002	1
Osbpl10	0,878201	0,000336	0,002	0,002	1
Rilp	0,878201	0,000336	0,002	0,002	1
Tom1l1	0,878201	0,000336	0,002	0,002	1
Gm28707	0,878201	0,000336	0,002	0,002	1
Adcy2	0,878201	0,000336	0,002	0,002	1
Gm17750	0,878201	0,000336	0,002	0,002	1
Sptb	0,878201	0,000336	0,002	0,002	1
Noxred1	0,878201	0,000336	0,002	0,002	1
Chga	0,878201	0,000336	0,002	0,002	1
Ighm	0,878201	0,000336	0,002	0,002	1
Mapk12	0,878201	0,000336	0,002	0,002	1
Nckap1l	0,878201	0,000336	0,002	0,002	1
Glis2	0,878201	0,000336	0,002	0,002	1
Vav1	0,878201	0,000336	0,002	0,002	1
Vit	0,878201	0,000336	0,002	0,002	1
Arhgef33	0,878201	0,000336	0,002	0,002	1
Nol4	0,878201	0,000336	0,002	0,002	1
Kcnn2	0,878201	0,000336	0,002	0,002	1
Mro	0,878201	0,000336	0,002	0,002	1
Pcsk5	0,878201	0,000336	0,002	0,002	1
Ablim1	0,878201	0,000336	0,002	0,002	1
Rps15a	0,878395	-0,00834	0,982	0,992	1
Pink1	0,878484	0,01636	0,813	0,811	1
Srr	0,878508	-0,0031	0,223	0,226	1
Nxf2	0,878701	0,00231	0,006	0,007	1
Olfr1238	0,878701	0,000363	0,006	0,007	1
Gm26617	0,878701	0,000363	0,006	0,007	1
Prkcd	0,878701	0,000363	0,006	0,007	1
Gm11940	0,878701	0,000363	0,006	0,007	1
Rab3c	0,878701	0,000363	0,006	0,007	1
Serpina3m	0,878701	0,000363	0,006	0,007	1
Hmcn1	0,878777	-0,00031	0,002	0,002	1
Trak1	0,878784	0,012356	0,136	0,135	1
Eftud2	0,878926	0,014889	0,313	0,32	1
Gm20075	0,879036	0,00264	0,009	0,008	1
Ctla2a	0,879036	0,006516	0,009	0,008	1
Eps8l2	0,879036	0,00361	0,009	0,008	1
Fam227a	0,879222	0,001098	0,022	0,023	1
A830018L1	0,879352	-0,00129	0,002	0,002	1
1700003F1	0,879352	-0,00129	0,002	0,002	1
Prdm16	0,879352	-0,00129	0,002	0,002	1

Ccnb2	0,879352	-0,00129	0,002	0,002	1
Rasd1	0,879352	-0,00129	0,002	0,002	1
Slc16a11	0,879352	-0,00129	0,002	0,002	1
Aif1	0,879352	-0,00129	0,002	0,002	1
Celf3	0,879352	-0,00291	0,002	0,002	1
Plppr1	0,879352	-0,00291	0,002	0,002	1
Chrd	0,879352	-0,00291	0,002	0,002	1
Pdp1	0,879395	0,001606	0,13	0,127	1
Atxn2l	0,879446	0,021987	0,605	0,611	1
Fem1a	0,879488	0,00396	0,284	0,288	1
Coa6	0,879735	0,007017	0,332	0,328	1
Ccdc96	0,879838	0,002968	0,009	0,008	1
Myh6	0,879965	0,001337	0,006	0,007	1
Dpf3	0,879965	0,001337	0,006	0,007	1
Setd4	0,880093	0,005251	0,047	0,046	1
Dis3l	0,880202	-0,0053	0,192	0,187	1
Axin2	0,880287	0,007968	0,079	0,081	1
Gm28043	0,880372	0,00264	0,009	0,008	1
Gm17092	0,880372	0,001668	0,009	0,008	1
Dynlrb2	0,880372	0,001668	0,009	0,008	1
2410021H(	0,880372	0,001668	0,009	0,008	1
Raver1	0,88049	0,000442	0,021	0,02	1
Mcm2	0,880581	0,00381	0,096	0,099	1
Faf2	0,8806	0,009807	0,497	0,511	1
Bbs1	0,880684	-0,00178	0,09	0,093	1
Tulp3	0,880813	0,005515	0,349	0,351	1
Abcc10	0,880814	0,010925	0,081	0,085	1
Immt	0,881028	0,016009	0,791	0,795	1
Eif4h	0,881175	-0,00844	0,985	0,99	1
Casp3	0,881588	0,000913	0,039	0,041	1
Tram1l1	0,881593	-0,00168	0,071	0,068	1
Mcm6	0,881595	-0,00554	0,07	0,072	1
Hnrnpul1	0,881596	0,026648	0,615	0,61	1
Smyd1	0,881709	0,000696	0,009	0,008	1
Agap2	0,881709	0,000696	0,009	0,008	1
Sez6	0,881709	0,000696	0,009	0,008	1
Pxdc1	0,881709	0,000696	0,009	0,008	1
Olfr775	0,881736	-0,00118	0,014	0,015	1
Primpol	0,881768	0,007765	0,03	0,029	1
Ptar1	0,881894	0,007572	0,135	0,133	1
Gorasp1	0,881929	0,002889	0,177	0,18	1
Rpp25	0,881977	-0,00058	0,009	0,008	1
Slc4a7	0,882166	0,002504	0,124	0,127	1
Ccdc181	0,882208	0,006451	0,109	0,112	1
Tmem8b	0,882461	0,012403	0,204	0,21	1
Zc3hc1	0,882522	0,001222	0,172	0,169	1
Cyp4f14	0,882533	0,004662	0,072	0,07	1
Mpnd	0,882696	-0,01431	0,404	0,403	1
C030047K2	0,88278	5,69E-05	0,009	0,008	1
A430105J0	0,882782	-0,00055	0,014	0,015	1
Wdr25	0,882783	-0,00394	0,026	0,028	1
Uhrf1bp1l	0,883324	0,009995	0,142	0,146	1

Aebp1	0,883617	-0,01476	0,321	0,314	1
Olfr1160	0,883827	8,49E-05	0,014	0,015	1
Prkag3	0,883925	0,003964	0,022	0,023	1
Plxna3	0,883947	-0,00234	0,06	0,062	1
2810001G:	0,884008	-0,00479	0,117	0,119	1
Palb2	0,884171	-0,00115	0,021	0,02	1
Trim35	0,884543	0,006529	0,97	0,969	1
Casz1	0,884549	0,002701	0,026	0,024	1
Alyref	0,884584	-0,00561	0,638	0,642	1
Inpp5e	0,884618	0,005005	0,113	0,111	1
Dclk2	0,884717	-0,004	0,1	0,098	1
Cfap36	0,884894	0,001577	0,625	0,636	1
Ppfia2	0,885339	-0,00386	0,062	0,063	1
Pycrl	0,885567	-0,01147	0,209	0,21	1
Trmt2b	0,885586	-0,01138	0,192	0,193	1
Scrn3	0,885623	-0,00324	0,286	0,291	1
Gm45890	0,885712	0,001052	0,014	0,015	1
E130114P1	0,885712	0,002017	0,014	0,015	1
AL732506.	0,885991	-0,00345	0,009	0,008	1
Zkscan3	0,886045	-0,00318	0,518	0,527	1
Glmn	0,886431	4,48E-05	0,143	0,146	1
Egln2	0,886675	0,004352	0,47	0,48	1
Anp32a	0,88669	0,00292	0,753	0,759	1
Xiap	0,886793	-0,01496	0,642	0,624	1
Mis12	0,886851	0,004815	0,192	0,195	1
Cog2	0,886915	-0,00729	0,217	0,22	1
Trim33	0,887012	0,0046	0,38	0,387	1
Baz2a	0,887094	0,017272	0,341	0,351	1
Nup155	0,887233	0,007869	0,122	0,12	1
Gpn1	0,887527	-0,00348	0,218	0,221	1
Fbxo34	0,887572	-0,00602	0,109	0,106	1
Gm45869	0,887598	0,002017	0,014	0,015	1
Mpzl3	0,887598	0,007792	0,014	0,015	1
Zc3h14	0,887869	0,000729	0,38	0,372	1
Ilf3	0,887916	-0,00114	0,503	0,496	1
Gm12976	0,887976	0,001953	0,069	0,067	1
Mobp	0,887976	-0,00321	0,999	1	1
Hivep1	0,888027	0,001397	0,072	0,07	1
Wdr60	0,888102	0,002968	0,253	0,259	1
Tmem79	0,888172	0,001098	0,026	0,024	1
Gabpb2	0,888274	0,00425	0,384	0,389	1
Gm26843	0,88842	-0,00174	0,026	0,028	1
Zfp956	0,888521	-0,00069	0,039	0,041	1
2900093K:	0,888555	0,005602	0,18	0,184	1
Slc35c1	0,888621	0,00348	0,081	0,08	1
Vps28	0,888644	0,017387	0,739	0,764	1
Rictor	0,888797	-0,00546	0,575	0,564	1
Pml	0,888798	0,007302	0,117	0,12	1
Uggt1	0,88895	0,008399	0,311	0,309	1
Ndufb8	0,889058	-0,01197	0,916	0,937	1
Dnajc11	0,889293	-0,00114	0,336	0,338	1
Gm44421	0,889499	-0,00282	0,079	0,081	1

Kcnk5	0,889692	-0,00127	0,087	0,089	1
Chst8	0,889722	0,01446	0,318	0,327	1
Rbpj	0,889762	0,00216	0,415	0,41	1
Xpot	0,889864	0,008769	0,199	0,197	1
Kif20b	0,88987	-0,00196	0,03	0,029	1
Spata7	0,890054	0,017999	0,171	0,167	1
AI197445	0,890071	0,002072	0,026	0,024	1
Gpr157	0,890145	0,004639	0,055	0,057	1
Clptm1	0,890156	0,007194	0,803	0,821	1
Fbxo25	0,890973	0,005871	0,361	0,366	1
Ric3	0,891053	0,006999	0,123	0,12	1
Spag1	0,891062	-0,00025	0,057	0,059	1
Hmg20b	0,891086	0,022214	0,403	0,402	1
Ell	0,891288	0,011218	0,192	0,19	1
Bclaf1	0,891292	0,021354	0,805	0,816	1
Gm13293	0,89144	-0,00672	0,363	0,369	1
Nup188	0,891634	0,01112	0,107	0,106	1
Cavin3	0,891722	0,009402	0,822	0,82	1
Med11	0,891846	-0,00809	0,127	0,124	1
Col6a3	0,892002	0,003744	0,062	0,06	1
Fam212a	0,892053	0,000544	0,039	0,041	1
Csmd2	0,892145	0,00327	0,1	0,102	1
Fam120c	0,892221	0,011554	0,206	0,205	1
Romo1	0,892385	0,009935	0,686	0,702	1
Usp11	0,892482	-0,01037	0,29	0,289	1
Mtf2	0,892564	-0,00747	0,375	0,372	1
Homer1	0,892586	0,009944	0,218	0,223	1
Zbtb48	0,892675	0,006171	0,073	0,075	1
Ing2	0,892709	0,015187	0,252	0,25	1
Setd2	0,892885	0,03242	0,565	0,58	1
Zcchc3	0,893088	0,003229	0,182	0,185	1
Mitd1	0,893255	0,003679	0,249	0,247	1
Syn3	0,893277	0,003356	0,035	0,034	1
Bbs4	0,893618	0,002518	0,145	0,143	1
Tmem208	0,893771	0,008552	0,569	0,58	1
Rbm4	0,89397	0,001971	0,075	0,076	1
Gm26779	0,89398	-0,00432	0,019	0,02	1
Nr1d1	0,894016	-0,00112	0,026	0,024	1
Nxt1	0,89415	0,00198	0,15	0,153	1
Chn1	0,894275	0,018082	0,123	0,122	1
Zfp54	0,89434	-0,00562	0,019	0,02	1
Dek	0,894347	0,016126	0,85	0,859	1
Rpl6	0,894496	-0,01299	0,996	0,998	1
Ndufb9	0,894526	-0,00056	0,961	0,976	1
Cluap1	0,89462	0,001724	0,326	0,33	1
Tbc1d25	0,894624	-0,00034	0,044	0,046	1
Map10	0,894776	0,006771	0,079	0,078	1
Ccdc126	0,895037	0,011868	0,082	0,081	1
Lypla1	0,895209	-0,0011	0,214	0,218	1
Pigt	0,895333	0,005415	0,65	0,644	1
Bap1	0,895551	0,00373	0,191	0,189	1
Wdr20	0,89573	0,010807	0,54	0,563	1

Grpel1	0,895764	0,010264	0,598	0,59	1
Kif18a	0,895943	-0,00063	0,044	0,046	1
B4galt4	0,895969	0,003356	0,035	0,034	1
4930572G(	0,895984	0,005249	0,04	0,039	1
Retsat	0,89602	-0,00141	0,11	0,107	1
Gm26720	0,896151	0,010971	0,078	0,076	1
9330175M	0,896265	0,003621	0,014	0,013	1
Dync1i1	0,896274	-0,00486	0,136	0,138	1
2210011C:	0,89632	0,004656	0,06	0,059	1
Rpl36al	0,896336	0,020532	0,856	0,867	1
P4htm	0,896376	0,014304	0,367	0,369	1
Zfp13	0,896404	0,005257	0,06	0,059	1
Kptn	0,896459	0,007571	0,265	0,27	1
Itgb4	0,896566	-0,00185	0,765	0,751	1
Zpr1	0,896597	-3,6E-05	0,337	0,341	1
Zfp280b	0,896625	0,003436	0,065	0,067	1
Celf5	0,896695	0,005231	0,014	0,013	1
Eps15l1	0,896987	0,006907	0,219	0,218	1
Acss2	0,896991	0,001277	0,442	0,442	1
Fyn	0,89711	0,008112	0,544	0,541	1
Ppp1r11	0,8975	0,005703	0,588	0,574	1
B930082K(	0,897538	0,007408	0,047	0,049	1
Pgam5	0,897568	0,007221	0,168	0,171	1
AC134548.	0,897782	-0,00244	0,019	0,02	1
Fzd4	0,897901	-0,00574	0,031	0,033	1
Rnasel	0,89798	0,006513	0,014	0,013	1
Gm35315	0,89798	0,004586	0,014	0,013	1
Ccdc50	0,898133	0,010711	0,603	0,611	1
Epb41l4a	0,898189	0,003854	0,116	0,114	1
Naa25	0,898221	0,007496	0,17	0,176	1
Ssr2	0,89861	0,006856	0,728	0,741	1
Pnrc1	0,898646	0,049486	0,749	0,771	1
Bricd5	0,898813	-0,00463	0,072	0,073	1
Maea	0,899262	-0,0004	0,351	0,358	1
Cldn10	0,899268	0,002017	0,014	0,013	1
Zfp93	0,899347	0,007394	0,065	0,063	1
Ube2e1	0,89967	-0,00195	0,543	0,548	1
Gm2464	0,899696	0,001689	0,014	0,013	1
Cdkl2	0,899741	-0,00454	0,169	0,171	1
Slc4a11	0,899912	0,000416	0,014	0,013	1
Rorb	0,899912	-0,00022	0,014	0,013	1
Gan	0,900156	0,007698	0,29	0,296	1
Efemp1	0,900171	0,003804	0,2	0,198	1
Sfr1	0,900253	0,017608	0,952	0,946	1
Cdk2ap1	0,900332	-0,02653	0,657	0,641	1
Fh1	0,900336	-0,00307	0,357	0,363	1
Eif3f	0,900458	0,019332	0,859	0,87	1
Ndufa5	0,900756	0,008389	0,897	0,899	1
Rnf115	0,900838	0,003059	0,385	0,384	1
Fam184b	0,900865	-0,00115	0,019	0,02	1
Haus8	0,90094	0,009418	0,238	0,236	1
Dach2	0,900969	0,002459	0,045	0,044	1

Zfp652	0,90105	0,004	0,378	0,385	1
Nemp2	0,901227	0,000412	0,115	0,112	1
Rpl30	0,901295	-0,00045	0,996	0,995	1
Epn1	0,901394	0,006991	0,625	0,607	1
Tspan12	0,901413	0,000721	0,014	0,013	1
Olfr1385	0,901413	0,000721	0,014	0,013	1
Rangap1	0,901445	-0,00482	0,604	0,616	1
Mettl14	0,901502	0,00612	0,25	0,246	1
Psmd3	0,90163	0,002516	0,755	0,777	1
Ncaph2	0,901708	0,01535	0,395	0,398	1
Kctd17	0,901776	0,017107	0,496	0,506	1
Ost4	0,901864	0,00662	0,77	0,782	1
Cib1	0,901976	0,00848	0,3	0,309	1
Myef2	0,902221	-0,00286	0,602	0,597	1
Trim44	0,902299	-0,00257	0,636	0,655	1
Gnpat	0,902342	0,001986	0,323	0,32	1
Mrps33	0,902353	0,009268	0,807	0,818	1
Apaf1	0,90248	0,003201	0,082	0,085	1
Itgad	0,902538	-0,00161	0,07	0,068	1
Epb41l4ao:	0,902549	0,020246	0,256	0,259	1
Tnks	0,902575	0,013003	0,673	0,663	1
Mat2b	0,902619	0,007353	0,506	0,506	1
Usf2	0,902781	0,012524	0,762	0,779	1
Ccsap	0,902884	-0,00607	0,081	0,083	1
Eapp	0,902965	0,007926	0,602	0,61	1
Otud4	0,903417	0,026551	0,368	0,379	1
Mrpl17	0,9036	0,01419	0,589	0,597	1
Snd1	0,903784	-0,00382	0,379	0,377	1
Pomgnt2	0,903964	0,009404	0,097	0,096	1
Dpm2	0,90399	0,007137	0,52	0,533	1
Ddx55	0,904017	0,006325	0,315	0,312	1
Clint1	0,90408	0,002429	0,317	0,319	1
Zfp874a	0,904252	0,004997	0,112	0,111	1
Ccdc92	0,90426	0,003985	0,176	0,179	1
Dr1	0,904308	0,007876	0,25	0,255	1
Prps1	0,90442	0,008013	0,184	0,189	1
Chst14	0,904462	0,004322	0,05	0,049	1
Phtf2	0,90454	0,01315	0,063	0,062	1
Cadps	0,90458	0,002977	0,083	0,086	1
Pgm3	0,904611	-0,00185	0,282	0,285	1
9130019O:	0,904725	0,009888	0,048	0,047	1
Rhpn2	0,904924	-0,00038	0,319	0,312	1
Ranbp1	0,905	0,025847	0,674	0,698	1
Hdac7	0,905069	0,030331	0,366	0,382	1
Rack1	0,905158	-0,00038	0,943	0,946	1
Garnl3	0,905184	0,002546	0,142	0,145	1
Nolc1	0,905523	0,030218	0,36	0,369	1
Ptpn11	0,90554	0,011872	0,878	0,876	1
Zfyve1	0,905541	0,012551	0,377	0,376	1
Gnpda1	0,90573	-0,00535	0,182	0,184	1
Thumpd1	0,905842	0,008021	0,432	0,444	1
Gm28050	0,905899	0,003632	0,019	0,018	1

Gm26882	0,906065	0,010017	0,088	0,091	1
Hcfc1r1	0,906345	0,014739	0,81	0,815	1
Tmlhe	0,906356	0,001209	0,044	0,046	1
S100a7a	0,906483	-0,0049	0,024	0,024	1
Bud13	0,906607	0,004961	0,07	0,068	1
Msl1	0,906661	-0,00372	0,493	0,494	1
Zfp784	0,906715	0,002459	0,045	0,044	1
6030407O(	0,906807	0,002737	0,126	0,124	1
Brca2	0,906991	0,002309	0,079	0,078	1
Sec23ip	0,907084	-0,00456	0,325	0,327	1
Trappc13	0,907129	0,00227	0,393	0,398	1
Rc3h1	0,90716	0,028274	0,604	0,613	1
Tsen2	0,907232	0,004301	0,215	0,211	1
Dolk	0,907253	0,003154	0,271	0,273	1
Cdt1	0,907375	0,002996	0,019	0,018	1
Itsn2	0,907775	0,015297	0,77	0,782	1
Fam193a	0,907858	0,00452	0,435	0,428	1
Samd14	0,907927	0,003632	0,019	0,018	1
Swi5	0,908163	0,004337	0,936	0,946	1
Ahctf1	0,908266	0,030781	0,34	0,345	1
Zfp367	0,908286	0,006405	0,181	0,179	1
Nono	0,908287	0,005871	0,656	0,655	1
Ccdc174	0,908629	0,018182	0,413	0,426	1
Atp6v0c	0,908823	0,002506	0,065	0,063	1
Rab21	0,909136	0,00531	0,919	0,893	1
Rnf149	0,909197	-0,00651	0,349	0,35	1
Lekr1	0,909199	0,000331	0,147	0,15	1
Nek3	0,909215	0,006472	0,078	0,08	1
Gm16062	0,909401	0,005553	0,024	0,023	1
Ncoa7	0,909417	0,013601	0,325	0,325	1
Iba57	0,909457	0,001768	0,031	0,033	1
Zc3h13	0,90979	-0,00342	0,509	0,504	1
Gng3	0,909893	0,004377	0,075	0,073	1
Ibtk	0,909896	0,016141	0,341	0,351	1
Ict1os	0,909923	-0,00219	0,011	0,011	1
Syp	0,909994	0,009365	0,219	0,218	1
Setdb2	0,910083	-0,00123	0,091	0,089	1
Taf6l	0,910125	0,005647	0,345	0,351	1
Mzf1	0,910213	-0,00332	0,024	0,024	1
Ccnc	0,910384	0,018607	0,284	0,288	1
A430046D:	0,910442	-0,00302	0,091	0,093	1
Usp18	0,910465	-0,02488	0,03	0,029	1
Gm45767	0,910467	0,002504	0,133	0,135	1
Wdr17	0,910726	0,007452	0,026	0,028	1
Aebp2	0,91099	0,013313	0,541	0,55	1
Pdgfra	0,911064	0,003955	0,019	0,018	1
Jak1	0,911131	-0,00616	0,487	0,472	1
Pced1a	0,911299	0,004435	0,119	0,117	1
Glud1	0,911312	0,005007	0,684	0,678	1
Med24	0,911379	0,003772	0,204	0,208	1
Hectd3	0,911669	0,019116	0,219	0,221	1
Chmp7	0,911854	0,012065	0,806	0,808	1

Hps4	0,911941	0,012403	0,208	0,213	1
Mcu	0,911973	0,009547	0,132	0,132	1
Il1r2	0,912175	-0,00193	0,003	0,003	1
Syt13	0,912175	-0,00193	0,003	0,003	1
Cd72	0,912175	-0,00193	0,003	0,003	1
Poln	0,912175	-0,00193	0,003	0,003	1
Met	0,912175	-0,00193	0,003	0,003	1
B3gnt8	0,912175	-0,00193	0,003	0,003	1
Nps	0,912175	-0,00193	0,003	0,003	1
Akr1b8	0,912175	-0,00354	0,003	0,003	1
Nr3c1	0,912295	0,008858	0,934	0,948	1
Sdha	0,912364	0,005582	0,907	0,909	1
Gm29183	0,912533	-0,00058	0,011	0,011	1
Galnt2	0,912533	-0,00058	0,011	0,011	1
Abcg4	0,912533	-0,00058	0,011	0,011	1
Usp8	0,912568	-0,00313	0,55	0,561	1
Dcun1d1	0,91259	0,005871	0,432	0,439	1
Unc13c	0,912624	-0,00257	0,003	0,003	1
Ube3b	0,912922	-0,00932	0,248	0,249	1
Stx7	0,912928	0,007851	0,812	0,844	1
Efhc2	0,913072	-0,00095	0,003	0,003	1
A930001A:	0,913072	-0,00095	0,003	0,003	1
Dram1	0,913072	-0,00095	0,003	0,003	1
Olfr18	0,913072	-0,00095	0,003	0,003	1
Plcd1	0,913072	-0,00095	0,003	0,003	1
AL591946.	0,913072	-0,00095	0,003	0,003	1
Col4a4	0,913072	-0,00159	0,003	0,003	1
Akap2	0,913111	0,005284	0,097	0,096	1
Mipep	0,913219	0,004978	0,087	0,086	1
Prr7	0,913278	0,002036	0,019	0,018	1
Ncapg2	0,913399	0,016256	0,153	0,153	1
Ppp2r5a	0,913442	0,021012	0,693	0,714	1
Rassf5	0,913521	-0,00031	0,003	0,003	1
Platr23	0,913521	-0,00031	0,003	0,003	1
Nek2	0,913521	-0,00031	0,003	0,003	1
Tspyl3	0,913521	-0,00031	0,003	0,003	1
Nfatc2	0,913521	-0,00031	0,003	0,003	1
Bmp7	0,913521	-0,00031	0,003	0,003	1
9530027J0	0,913521	-0,00031	0,003	0,003	1
Dcx	0,913521	-0,00031	0,003	0,003	1
Ace2	0,913521	-0,00031	0,003	0,003	1
P2ry14	0,913521	-0,00031	0,003	0,003	1
Gm34866	0,913521	-0,00031	0,003	0,003	1
Spag8	0,913521	-0,00031	0,003	0,003	1
Cdkn2b	0,913521	-0,00031	0,003	0,003	1
Heyl	0,913521	-0,00031	0,003	0,003	1
Slc25a34	0,913521	-0,00031	0,003	0,003	1
Zfp993	0,913521	-0,00031	0,003	0,003	1
Jakmip1	0,913521	-0,00031	0,003	0,003	1
Clec4d	0,913521	-0,00031	0,003	0,003	1
Cdca3	0,913521	-0,00031	0,003	0,003	1
Tmem145	0,913521	-0,00031	0,003	0,003	1

4930558N1	0,913521	-0,00031	0,003	0,003	1
Ddias	0,913521	-0,00031	0,003	0,003	1
Olfr633	0,913521	-0,00031	0,003	0,003	1
Olfr675	0,913521	-0,00031	0,003	0,003	1
Shank2	0,913521	-0,00031	0,003	0,003	1
Cdk1	0,913521	-0,00031	0,003	0,003	1
Itgb2	0,913521	-0,00031	0,003	0,003	1
Matk	0,913521	-0,00031	0,003	0,003	1
Parpbp	0,913521	-0,00031	0,003	0,003	1
AC117232.	0,913521	-0,00031	0,003	0,003	1
Slc7a2	0,913521	-0,00031	0,003	0,003	1
Galntl6	0,913521	-0,00031	0,003	0,003	1
Npy1r	0,913521	-0,00031	0,003	0,003	1
Gm26721	0,913521	-0,00031	0,003	0,003	1
Dhrs2	0,913521	-0,00031	0,003	0,003	1
Gpc5	0,913521	-0,00031	0,003	0,003	1
Olfr976	0,913521	-0,00031	0,003	0,003	1
Gm47043	0,913521	-0,00031	0,003	0,003	1
Nefh	0,913521	-0,00031	0,003	0,003	1
Hsd17b1	0,913521	-0,00031	0,003	0,003	1
Etv4	0,913521	-0,00031	0,003	0,003	1
A330076C(	0,913521	-0,00031	0,003	0,003	1
Gm48780	0,913521	-0,00031	0,003	0,003	1
Efcab11	0,913521	-0,00031	0,003	0,003	1
Wnt7b	0,913521	-0,00031	0,003	0,003	1
AU022754	0,913521	-0,00031	0,003	0,003	1
Itga5	0,913521	-0,00031	0,003	0,003	1
AC166832.	0,913521	-0,00031	0,003	0,003	1
Fstl1	0,913521	-0,00031	0,003	0,003	1
AC125199.	0,913521	-0,00031	0,003	0,003	1
2310043M	0,913521	-0,00031	0,003	0,003	1
Gm16196	0,913521	-0,00031	0,003	0,003	1
Tcf19	0,913521	-0,00031	0,003	0,003	1
Gnmt	0,913521	-0,00031	0,003	0,003	1
Crb3	0,913521	-0,00031	0,003	0,003	1
Aqp1	0,913521	2,86E-05	0,003	0,003	1
Exoc4	0,913641	0,018063	0,515	0,528	1
Fbln7	0,913807	0,005557	0,038	0,037	1
Palmd	0,91397	2,86E-05	0,003	0,003	1
Poli	0,914112	0,005871	0,053	0,052	1
Gm21992	0,914195	0,001359	0,011	0,011	1
Gm39214	0,914195	0,00039	0,011	0,011	1
Gm4787	0,914195	0,00039	0,011	0,011	1
Hist2h3c2	0,914417	-0,00456	0,028	0,029	1
Atrnl1	0,914418	0,003599	0,003	0,003	1
Cyp7b1	0,914418	0,002624	0,003	0,003	1
Mme	0,914418	0,002624	0,003	0,003	1
Arhgef17	0,914418	0,002624	0,003	0,003	1
Cyr61	0,914418	0,001648	0,003	0,003	1
Gm26683	0,914418	0,000671	0,003	0,003	1
Capn8	0,914418	0,000671	0,003	0,003	1
Enkur	0,914418	0,000671	0,003	0,003	1

Slc2a6	0,914418	0,000671	0,003	0,003	1
Nr6a1os	0,914418	0,000671	0,003	0,003	1
Erv3	0,914418	0,000671	0,003	0,003	1
Gm26952	0,914418	0,000671	0,003	0,003	1
Hist2h2aa2	0,914418	0,000671	0,003	0,003	1
Bcar3	0,914418	0,000671	0,003	0,003	1
Zfp69	0,914418	0,000671	0,003	0,003	1
Eva1b	0,914418	0,000671	0,003	0,003	1
Mmp23	0,914418	0,000671	0,003	0,003	1
Gm7854	0,914418	0,000671	0,003	0,003	1
Gm29707	0,914418	0,000671	0,003	0,003	1
Add2	0,914418	0,000671	0,003	0,003	1
Gm15612	0,914418	0,000671	0,003	0,003	1
Vmn1r65	0,914418	0,000671	0,003	0,003	1
Omp	0,914418	0,000671	0,003	0,003	1
B430219N1	0,914418	0,000671	0,003	0,003	1
Ccdc38	0,914418	0,000671	0,003	0,003	1
Glipr1	0,914418	0,000671	0,003	0,003	1
Ctrl	0,914418	0,000671	0,003	0,003	1
M5C1000I1	0,914418	0,000671	0,003	0,003	1
Sult4a1	0,914418	0,000671	0,003	0,003	1
Gm16299	0,914418	0,000671	0,003	0,003	1
Cnbp	0,914446	-0,00018	0,965	0,972	1
Arpc5	0,914846	0,01597	0,921	0,935	1
Mxi1	0,914883	0,014426	0,671	0,678	1
Rab4b	0,914952	0,008574	0,181	0,18	1
Slc2a10	0,915316	0,001648	0,003	0,003	1
Lmod3	0,915316	0,001648	0,003	0,003	1
Ahdc1	0,915606	-0,01156	0,153	0,15	1
Slc38a6	0,915619	0,014855	0,255	0,255	1
C230004F1	0,915857	0,001359	0,011	0,011	1
Gm34788	0,915857	0,001359	0,011	0,011	1
Gm44759	0,915857	0,003295	0,011	0,011	1
Wdr75	0,915871	0,005047	0,16	0,163	1
2810454H(	0,916032	0,032753	0,139	0,141	1
Adam4	0,916635	0,003332	0,024	0,023	1
Prr14l	0,916765	0,001561	0,281	0,276	1
Pum3	0,916915	0,004559	0,37	0,369	1
Adcy10	0,916971	-0,00148	0,019	0,018	1
Lrrfip2	0,917254	0,005871	0,388	0,398	1
Csde1	0,917283	-0,00446	0,972	0,98	1
Safb	0,917488	0,008806	0,749	0,772	1
C2cd5	0,917722	0,019158	0,359	0,366	1
Mdh1	0,917792	0,015803	0,785	0,793	1
Zfp160	0,917986	0,003671	0,258	0,263	1
Plekha8	0,918077	0,002776	0,137	0,14	1
Man2b1	0,91849	0,006333	0,305	0,314	1
St6galnac6	0,918494	0,004357	0,067	0,068	1
Rnf215	0,918566	0,008458	0,223	0,221	1
Snx14	0,918808	0,003695	0,276	0,28	1
Maneal	0,918819	-0,0063	0,157	0,159	1
Gm47551	0,918939	0,000795	0,024	0,023	1

Impdh1	0,919183	0,005228	0,011	0,011	1
Timm10	0,919186	0,026039	0,262	0,265	1
Zxdc	0,91926	0,000729	0,269	0,273	1
Irf9	0,919325	-0,03603	0,158	0,156	1
Recql5	0,919705	0,01845	0,144	0,145	1
Naa50	0,919766	0,007684	0,513	0,515	1
Unc5c	0,920095	0,017811	0,63	0,646	1
Amd1	0,920146	0,000123	0,845	0,857	1
Gm16223	0,92022	0,002108	0,036	0,037	1
Tpm4	0,920289	0,009587	0,184	0,182	1
Klf11	0,920415	-0,00194	0,195	0,197	1
Cnpy3	0,92043	0,010777	0,518	0,527	1
Pafah2	0,920466	-0,0091	0,078	0,078	1
Gm7347	0,920482	-0,00145	0,019	0,018	1
Slc48a1	0,920497	0,002472	0,999	1	1
1600012H(	0,920869	0,012139	0,294	0,296	1
Dnajb14	0,920955	0,011736	0,781	0,792	1
Lig4	0,921162	0,009045	0,116	0,115	1
C920006O:	0,921405	0,010815	0,103	0,106	1
Atg12	0,921498	0,000487	0,328	0,33	1
4933404O:	0,921575	0,007655	0,09	0,089	1
Myg1	0,921832	0,001504	0,335	0,338	1
Mrpl44	0,921924	-0,00617	0,126	0,127	1
Nemf	0,922078	0,00234	0,65	0,629	1
Pecr	0,922089	0,011012	0,223	0,223	1
Slc39a11	0,922662	0,000851	0,094	0,096	1
Atp5o.1	0,922712	0,002005	0,931	0,928	1
Rad18	0,922825	-0,00175	0,096	0,098	1
Vrk2	0,922943	0,003744	0,068	0,067	1
Zfp119b	0,922966	-0,00724	0,038	0,039	1
Ext2	0,922969	0,004032	0,335	0,332	1
Ptcd2	0,922972	0,00968	0,237	0,244	1
Snx16	0,922972	0,005392	0,275	0,278	1
Erich6	0,923043	0,003332	0,024	0,024	1
Taf1d	0,923135	0,021407	0,644	0,646	1
Zfp326	0,923264	0,004895	0,575	0,567	1
Sgsm3	0,923308	-0,00182	0,128	0,125	1
Gm9945	0,923356	0,008969	0,266	0,273	1
Fbxw8	0,923495	0,01119	0,357	0,366	1
Sec16a	0,92374	0,004152	0,258	0,262	1
Pex1	0,923898	-0,01475	0,298	0,302	1
Fbxl4	0,924044	0,002278	0,154	0,156	1
Abhd6	0,924126	0,004013	0,302	0,306	1
Trmt1l	0,92418	0,023929	0,344	0,351	1
St3gal6	0,924204	0,016081	0,269	0,272	1
Klhl8	0,924267	0,007496	0,177	0,176	1
Csnk1d	0,924552	0,008894	0,475	0,467	1
Spout1	0,924559	-0,01023	0,261	0,259	1
Gigyf2	0,924836	0,002031	0,524	0,519	1
Ctsa	0,924878	0,003992	0,896	0,904	1
Hscb	0,924898	0,009754	0,221	0,221	1
Gramd1c	0,924915	0,000167	0,028	0,028	1

Gabarapl2	0,924997	-0,00204	0,972	0,974	1
Arih1	0,92503	0,018867	0,539	0,545	1
Zfp60	0,925041	0,013509	0,359	0,371	1
D230025D	0,925069	0,010004	0,265	0,27	1
Tm9sf2	0,925178	0,010164	0,713	0,727	1
Pggt1b	0,925211	0,003388	0,16	0,158	1
Alg10b	0,925312	0,013872	0,156	0,156	1
3110002H	0,926001	0,00694	0,193	0,197	1
Rnf7	0,926186	0,00891	0,99	0,995	1
Pcdhb5	0,926206	-0,01064	0,016	0,016	1
Trpm4	0,926208	0,001787	0,033	0,033	1
Tm9sf3	0,926221	0,004522	0,965	0,945	1
Khsrp	0,926395	0,002621	0,456	0,454	1
Spty2d1	0,926443	0,017316	0,27	0,276	1
Dhtkd1	0,926627	0,001315	0,064	0,065	1
Rprd2	0,926638	0,001168	0,421	0,424	1
Zfp61	0,927132	-0,00581	0,103	0,104	1
Coq8b	0,927297	0,018913	0,219	0,22	1
Arhgef9	0,927457	0,004355	0,237	0,236	1
Tmem97	0,927516	0,00818	0,316	0,314	1
Ddx46	0,927801	0,017996	0,578	0,589	1
Cerk	0,927825	0,012379	0,64	0,649	1
Mars	0,927842	-0,0028	0,308	0,309	1
Nfatc3	0,928128	0,023709	0,42	0,433	1
Bhlhe22	0,928159	0,00231	0,007	0,007	1
Gm12905	0,928159	0,00231	0,007	0,007	1
Alox8	0,928159	0,00231	0,007	0,007	1
C330007P(	0,928316	0,01188	0,502	0,507	1
Gabarap	0,928434	0,012591	0,995	0,998	1
Zfp280d	0,928463	0,01515	0,54	0,545	1
1600020E(	0,928939	-0,00414	0,243	0,246	1
Clic3	0,929373	0,004254	0,007	0,007	1
Gm26620	0,929373	0,00231	0,007	0,007	1
Xkrx	0,929373	0,00231	0,007	0,007	1
Msx1	0,929373	0,001337	0,007	0,007	1
2310040G	0,929373	0,001337	0,007	0,007	1
A230083G	0,929373	0,001337	0,007	0,007	1
Dok4	0,929373	0,001337	0,007	0,007	1
Gm12227	0,929373	0,001337	0,007	0,007	1
Ccdc114	0,929578	-0,00244	0,016	0,016	1
Hells	0,929578	-0,00244	0,016	0,016	1
Rgl3	0,92961	-0,00635	0,033	0,034	1
Sepsecs	0,929663	0,007522	0,155	0,154	1
Tmem53	0,929776	-0,00307	0,016	0,016	1
Dbr1	0,929828	-0,00279	0,114	0,115	1
Gk5	0,929971	-0,00215	0,111	0,112	1
Nfxl1	0,929975	0,013469	0,136	0,137	1
Gspt2	0,93	0,01588	0,091	0,091	1
Pde8a	0,930245	-0,01233	0,901	0,88	1
Extl2	0,930252	0,008267	0,37	0,367	1
Dnajc2	0,930356	-0,00346	0,326	0,319	1
Farsa	0,930429	0,013697	0,288	0,291	1

Sec61a2	0,93052	0,007884	0,558	0,554	1
Mlec	0,93055	-0,0072	0,553	0,554	1
Gm26691	0,930587	0,000363	0,007	0,007	1
Kcnd2	0,930587	0,000363	0,007	0,007	1
Gm26534	0,930587	0,000363	0,007	0,007	1
Trip12	0,930589	0,001015	0,614	0,589	1
1810013L2	0,930602	0,003993	0,293	0,296	1
4930488L2	0,930635	-0,00114	0,061	0,062	1
Klhdc8a	0,931088	0,04938	0,273	0,276	1
Larp1	0,931128	0,018646	0,579	0,585	1
Eed	0,931137	0,009623	0,248	0,247	1
Arsa	0,931137	0,01874	0,352	0,359	1
Emd	0,931325	0,013226	0,358	0,361	1
1110008F1	0,931354	-0,00124	0,638	0,629	1
Eef2k	0,931383	0,016889	0,106	0,109	1
Gm14762	0,931499	-0,00028	0,007	0,007	1
Gm26847	0,931499	-0,00028	0,007	0,007	1
Vegfa	0,931525	0,031539	0,11	0,109	1
Jag2	0,931802	-0,00189	0,007	0,007	1
Eda2r	0,931876	0,011682	0,051	0,05	1
Mettl25	0,931885	0,006984	0,147	0,146	1
Tmtc4	0,931967	-0,00278	0,076	0,076	1
Dhx32	0,932005	0,001002	0,122	0,12	1
Carns1	0,932116	0,000325	0,703	0,686	1
Tmem238	0,932281	0,009441	0,163	0,166	1
Col5a3	0,932349	0,013123	0,026	0,026	1
Kif15	0,93241	-0,00155	0,007	0,007	1
Gm49207	0,932427	0,002611	0,092	0,091	1
Map3k7	0,932432	0,018692	0,363	0,372	1
Dbf4	0,932508	-0,00348	0,038	0,039	1
Mdrl	0,932554	0,000416	0,016	0,016	1
Ascc1	0,932579	0,01677	0,259	0,263	1
Zfp46	0,932588	0,001164	0,148	0,15	1
Palld	0,932713	-0,00125	0,007	0,007	1
Gm10647	0,932713	-0,00125	0,007	0,007	1
Lonp2	0,93272	0,011432	0,447	0,462	1
Cct3	0,932971	0,005871	0,848	0,844	1
Dusp22	0,933212	-0,00121	0,2	0,198	1
Fbxo31	0,933417	0,02004	0,365	0,379	1
Maml2	0,933536	0,002228	0,059	0,06	1
Gpatch2l	0,933636	-0,00031	0,287	0,285	1
Ankfy1	0,93367	0,011399	0,381	0,392	1
Sirt4	0,934089	0,01188	0,174	0,174	1
4930419G1	0,934275	0,00555	0,012	0,011	1
Fam136a	0,934299	0,000879	0,153	0,154	1
Gdf9	0,934426	-0,00376	0,043	0,044	1
9130604C2	0,934539	0,003308	0,016	0,016	1
Washc1	0,934572	0,016484	0,347	0,351	1
Exosc10	0,934617	0,00052	0,311	0,314	1
Rad51c	0,934738	0,006192	0,012	0,011	1
Smco1	0,93484	-0,00447	0,007	0,007	1
Gm20498	0,934958	-0,00037	0,043	0,042	1

Ubiad1	0,935058	-0,00244	0,088	0,089	1
Trpm3	0,93535	0,004923	0,028	0,029	1
Max	0,935474	0,022284	0,425	0,433	1
Mpg	0,935532	0,009531	0,133	0,137	1
Irf2bp1	0,935533	0,013737	0,38	0,392	1
Spon1	0,935533	0,002054	0,016	0,016	1
Nat6	0,935536	-0,00272	0,182	0,182	1
Clu	0,935648	0,089673	0,212	0,216	1
Clk1	0,935732	0,001315	0,919	0,92	1
Cep350	0,935759	-0,00445	0,488	0,486	1
Zfp248	0,935837	0,003532	0,102	0,101	1
Foxred1	0,935873	0,000676	0,216	0,213	1
Gosr2	0,935891	0,012055	0,395	0,398	1
Uri1	0,935913	0,002149	0,395	0,39	1
Fzd5	0,936198	-0,00802	0,105	0,106	1
Surf1	0,93625	-0,00278	0,582	0,571	1
Jtb	0,936343	0,008534	0,663	0,681	1
Tmem131	0,936345	0,015912	0,317	0,319	1
Dynlrb1	0,936503	0,004651	0,907	0,912	1
Dtymk	0,936552	0,017555	0,44	0,45	1
Hsf2	0,936574	0,005618	0,228	0,226	1
Slc26a1	0,936631	0,000519	0,033	0,034	1
Gpr61	0,936918	0,001294	0,063	0,062	1
1700008J0	0,936955	0,001606	0,061	0,062	1
Usp22	0,937166	0,00712	0,844	0,833	1
Amdhd2	0,937304	0,017628	0,394	0,402	1
Myo18a	0,937502	0,017095	0,806	0,81	1
Samd15	0,937519	0,003621	0,012	0,011	1
Mrpl35	0,937643	0,000594	0,286	0,288	1
Foxj1	0,937762	0,015362	0,154	0,158	1
Cavin2	0,937928	-0,00867	0,227	0,226	1
Trmt2a	0,938003	0,015845	0,276	0,281	1
Atat1	0,938304	-0,00277	0,614	0,613	1
Pak2	0,93843	0,00248	0,923	0,932	1
Anxa6	0,938521	-0,00189	0,043	0,042	1
Pygo2	0,938532	-0,00741	0,232	0,231	1
Axin1	0,938585	0,001349	0,237	0,239	1
Fam228b	0,939047	0,020172	0,294	0,306	1
Vkorc1l1	0,939087	0,01228	0,349	0,358	1
Usp42	0,939356	0,023983	0,206	0,208	1
Aste1	0,939454	0,001775	0,096	0,098	1
6330403N1	0,939605	0,002328	0,012	0,011	1
Gm5784	0,939605	0,005228	0,012	0,011	1
A830035O1	0,939605	0,004262	0,012	0,011	1
Arfgef2	0,939647	0,005871	0,364	0,361	1
Dennd6b	0,939872	0,000879	0,151	0,15	1
Snx17	0,940072	0,006155	0,711	0,733	1
Teddm2	0,940265	0,009329	0,026	0,026	1
Cadps2	0,940305	-0,00111	0,097	0,096	1
Med22	0,94032	0,005871	0,27	0,275	1
Cnot11	0,940343	-0,00085	0,189	0,187	1
Bhlhe41	0,94062	-0,00836	0,839	0,836	1

Galnt4	0,940801	0,001475	0,097	0,096	1
Cox5a	0,941042	0,011244	0,817	0,823	1
Tmem222	0,941194	-0,00468	0,479	0,485	1
Btf3	0,941215	0,007362	0,993	0,992	1
Armc2	0,941228	0,003295	0,012	0,011	1
Qser1	0,941523	-0,00993	0,253	0,25	1
Ttc28	0,941786	-0,00701	0,156	0,153	1
Itpkb	0,942077	0,00014	0,021	0,021	1
Fbxl14	0,9421	0,005871	0,194	0,193	1
Alkbh5	0,942135	0,019689	0,716	0,735	1
Cldn34c1	0,942318	0,007528	0,286	0,289	1
Dmac1	0,942371	0,003108	0,327	0,33	1
Intu	0,942378	0,002789	0,051	0,052	1
Ssr1	0,942645	0,012445	0,953	0,959	1
Olfr259	0,942851	0,00039	0,012	0,011	1
Wfdc3	0,942851	0,00039	0,012	0,011	1
Kif24	0,942851	0,00039	0,012	0,011	1
Ndnf	0,942893	-0,00423	0,048	0,047	1
Usp54	0,942917	0,005165	0,85	0,857	1
Pfdn6	0,942932	-6,4E-05	0,838	0,847	1
Nsmce1	0,943003	0,014556	0,409	0,416	1
Inafm1	0,943081	0,010552	0,101	0,101	1
Grk6	0,943299	-0,00828	0,167	0,167	1
Jade3	0,943388	0,004307	0,033	0,034	1
Ddx1	0,943501	0,006923	0,829	0,826	1
Ick	0,943864	0,000408	0,571	0,576	1
Ift43	0,943887	-0,00389	0,594	0,571	1
Rtn4r	0,943931	-0,00098	0,043	0,044	1
Atg16l2	0,944045	-0,00153	0,048	0,049	1
Polr2i	0,944214	0,017142	0,511	0,522	1
Id4	0,94424	-0,00349	0,107	0,107	1
Pgc	0,944514	0,002345	0,017	0,016	1
Nop56	0,944517	0,021218	0,505	0,525	1
Jagn1	0,944618	0,016596	0,454	0,47	1
4933433G	0,94478	0,00332	0,022	0,021	1
Bloc1s6	0,944799	-0,00016	0,118	0,119	1
Kpna1	0,944887	-0,00418	0,388	0,389	1
Wtip	0,945133	0,006498	0,036	0,036	1
B230217C	0,945374	0,002913	0,181	0,182	1
Supt16	0,945507	0,023506	0,614	0,615	1
Shcbp1	0,94556	0,002686	0,021	0,021	1
Ncoa1	0,945585	0,004596	0,371	0,376	1
Gm42413	0,945635	-0,00122	0,012	0,011	1
Lrrc46	0,945635	-0,00282	0,012	0,011	1
Tmem2	0,945635	-0,00055	0,012	0,011	1
Hdac2	0,945678	0,005418	0,337	0,341	1
Mtrf1l	0,945702	-0,00431	0,135	0,133	1
B3gat1	0,945719	0,001658	0,372	0,369	1
Gm26606	0,94588	0,001709	0,017	0,016	1
Zcchc4	0,946329	-0,00096	0,121	0,122	1
0610037L1	0,946415	0,00416	0,467	0,47	1
Ube2cbp	0,946465	0,001381	0,017	0,016	1

Otud5	0,946782	0,002684	0,325	0,322	1
Hipk3	0,946826	0,024141	0,37	0,382	1
Agap1	0,94689	-0,01054	0,98	0,974	1
Ercc1	0,947548	0,012988	0,289	0,296	1
Nt5c3	0,947749	0,007147	0,497	0,502	1
Snx3	0,947786	0,008669	0,94	0,95	1
Zfp358	0,947875	0,001055	0,245	0,247	1
Gatb	0,947919	0,001315	0,129	0,127	1
Tmem98	0,947935	0,017762	0,721	0,738	1
Rbm19	0,947939	-0,00316	0,139	0,14	1
Cenpw	0,948148	0,002716	0,031	0,031	1
Mn1	0,94824	0,002399	0,231	0,234	1
Uggt2	0,948269	0,028063	0,131	0,132	1
Mgmt	0,948691	-0,00155	0,008	0,008	1
Rom1	0,948704	0,004203	0,155	0,154	1
Timm23	0,948824	0,003391	0,046	0,046	1
Mthfd1	0,948912	-0,00644	0,264	0,26	1
Gm49353	0,948971	-0,00189	0,008	0,008	1
Gm16845	0,948971	-0,00189	0,008	0,008	1
Msx2	0,948971	-0,00189	0,008	0,008	1
Pdpk1	0,949048	0,011808	0,413	0,42	1
Rundc3b	0,94929	0,003063	0,038	0,039	1
Shq1	0,949462	0,001918	0,071	0,07	1
Rnf24	0,949496	0,013018	0,158	0,159	1
Oaz3	0,949531	-0,00025	0,008	0,008	1
Sox11	0,949538	0,069893	0,067	0,068	1
Neu1	0,949592	0,008531	0,417	0,424	1
Zfp712	0,949765	-0,00178	0,058	0,059	1
Galnt18	0,949782	-0,00022	0,017	0,016	1
Map2k3os	0,949782	-0,00148	0,017	0,016	1
Acat1	0,950085	0,001545	0,607	0,618	1
St8sia4	0,950091	-0,00252	0,008	0,008	1
Cep128	0,950316	-0,00141	0,102	0,101	1
Tmem72	0,950563	-0,00052	0,017	0,016	1
Mrps18a	0,950731	0,003498	0,543	0,546	1
Gm49042	0,950825	0,001424	0,026	0,026	1
8430432A(	0,951211	-0,00028	0,008	0,008	1
Ccdc160	0,951211	-0,00028	0,008	0,008	1
Kcna3	0,951211	-0,00028	0,008	0,008	1
Fam149a	0,951211	-0,00028	0,008	0,008	1
Gm20687	0,951211	-0,00028	0,008	0,008	1
Hmga1b	0,951211	-0,00028	0,008	0,008	1
Galm	0,951211	-0,00028	0,008	0,008	1
Als2	0,951371	0,009031	0,122	0,122	1
Brms1	0,951568	-0,00193	0,385	0,387	1
1110012L1	0,951584	0,008552	0,194	0,198	1
Btbd19	0,951677	0,005254	0,051	0,05	1
Mrps18c	0,951728	0,00886	0,5	0,522	1
Kcp	0,951733	-0,00118	0,017	0,016	1
Smim26	0,951805	-0,004	0,659	0,665	1
Zfp329	0,951912	0,012651	0,304	0,309	1
Tmem251	0,95213	0,005871	0,201	0,2	1

Atp8b2	0,952333	0,001486	0,036	0,036	1
Mylpf	0,95238	0,00614	0,159	0,158	1
Mcur1	0,952583	-0,0101	0,191	0,19	1
Gnrh1	0,952611	0,00264	0,008	0,008	1
Gm15478	0,952611	0,001668	0,008	0,008	1
Hk1os	0,952611	0,001668	0,008	0,008	1
Ajuba	0,952611	0,000696	0,008	0,008	1
Slc10a1	0,952611	0,000696	0,008	0,008	1
Mgea5	0,953011	0,004751	0,68	0,694	1
Odc1	0,953037	0,007293	0,272	0,276	1
Cuedc2	0,953082	0,022562	0,826	0,841	1
Eef1akmt4	0,9531	-0,00181	0,017	0,016	1
Cops7b	0,953457	0,007522	0,155	0,154	1
Fam171a1	0,953543	-0,00177	0,319	0,317	1
Gm527	0,953576	0,001209	0,046	0,046	1
Nup37	0,953584	0,000819	0,031	0,031	1
Birc5	0,954012	0,001668	0,008	0,008	1
Gm4724	0,954012	0,00361	0,008	0,008	1
Iapp	0,954012	0,00264	0,008	0,008	1
Sgk2	0,954059	-0,00044	0,263	0,263	1
Rasl11b	0,954073	-0,02502	0,392	0,387	1
Gm37459	0,954191	0,000795	0,026	0,026	1
Dpy30	0,954215	0,002242	0,483	0,483	1
A530017D:	0,954228	0,005304	0,132	0,132	1
Ncmap	0,954246	0,000494	0,026	0,026	1
Suco	0,954389	0,016707	0,43	0,444	1
Ctdspl	0,95469	0,005673	0,422	0,428	1
Rnf152	0,954735	0,019826	0,169	0,172	1
4933406C:	0,954777	-0,00286	0,073	0,072	1
Ppcs	0,954815	0,001626	0,071	0,07	1
Lars	0,954917	-0,0054	0,424	0,421	1
Smap2	0,955176	0,003987	0,602	0,607	1
Rbbp6	0,95518	0,060608	0,695	0,733	1
Pxn	0,955509	0,008746	0,197	0,2	1
Cd2ap	0,95552	0,007821	0,498	0,506	1
Jakmip3	0,955523	0,002071	0,559	0,561	1
Usp3	0,955631	0,007764	0,177	0,179	1
4930402H:	0,955698	-0,00711	0,957	0,977	1
Bop1	0,955812	0,006373	0,247	0,25	1
Ap4b1	0,955837	0,001315	0,129	0,13	1
Tet2	0,956226	0,010194	0,311	0,312	1
Pex11b	0,956262	-0,00657	0,278	0,276	1
Cep78	0,956297	0,000751	0,127	0,127	1
Fam220a	0,956383	0,030678	0,496	0,506	1
Rrp7a	0,956566	0,004908	0,492	0,491	1
Tiprl	0,956602	0,008531	0,437	0,442	1
Gtpbp6	0,956649	0,010296	0,361	0,363	1
Nudt14	0,956688	-0,00214	0,154	0,154	1
Dmd	0,956747	0,013873	0,114	0,114	1
Gm29083	0,956905	0,000274	0,046	0,046	1
Gm2415	0,957022	0,004404	0,098	0,098	1
Vars2	0,957034	0,002244	0,071	0,072	1

Zfp961	0,957179	0,004974	0,079	0,078	1
Gm45847	0,957699	0,008026	0,051	0,052	1
Xrcc4	0,957801	-0,00056	0,257	0,259	1
Mob1b	0,958041	0,011856	0,165	0,166	1
Sap130	0,95858	0,000663	0,102	0,102	1
3110001I2	0,958919	0,001486	0,03	0,031	1
Rnf126	0,959041	0,021892	0,368	0,384	1
Mier2	0,959133	0,005871	0,181	0,18	1
Bysl	0,959164	0,002044	0,17	0,171	1
Vegfb	0,959169	0,019958	0,484	0,494	1
Mybbp1a	0,959232	0,007426	0,425	0,421	1
Larp6	0,959235	0,013422	0,806	0,813	1
Cryzl1	0,959427	-0,00152	0,531	0,52	1
Tmem241	0,959457	0,010835	0,044	0,044	1
Ndfip1	0,959465	0,032672	0,37	0,384	1
Epas1	0,959486	-0,00169	0,12	0,119	1
Ncf2	0,959539	0,07347	0,026	0,026	1
Rtf1	0,959623	0,009877	0,71	0,694	1
Arhgef3	0,959786	-0,00167	0,03	0,031	1
Mllt1	0,959998	0,006152	0,144	0,146	1
Gm26733	0,960026	-0,002	0,031	0,031	1
Eif5b	0,960133	0,0186	0,78	0,789	1
2300009A(	0,96055	0,003903	0,261	0,265	1
Pfkl	0,960725	0,007451	0,192	0,193	1
Gm39469	0,960822	-0,00141	0,098	0,099	1
Zcchc11	0,960847	0,01999	0,425	0,439	1
Dctn4	0,960913	-0,01319	0,94	0,937	1
1810037I1	0,960955	0,011654	0,983	0,982	1
Vbp1	0,961132	0,011626	0,86	0,883	1
Gpr21	0,961243	-0,00551	0,026	0,026	1
Mterf1a	0,961253	0,010751	0,122	0,122	1
Zfp937	0,961356	-0,00478	0,063	0,063	1
Rtn4ip1	0,961424	-0,00043	0,08	0,081	1
Fam131a	0,961435	0,007043	0,104	0,106	1
Xrcc1	0,961496	0,011043	0,267	0,268	1
Klhl9	0,961723	-0,02161	0,44	0,436	1
Rab1a	0,961776	0,001853	0,605	0,61	1
Ccdc117	0,962087	0,013748	0,234	0,236	1
Hmgb3	0,962116	-0,00294	0,095	0,096	1
Ankrd50	0,9622	-0,00447	0,16	0,161	1
Faap24	0,962211	0,002155	0,131	0,132	1
AC161376.	0,962407	-0,00065	0,26	0,26	1
Rnh1	0,962588	-0,00693	0,567	0,556	1
Star	0,96282	0,001445	0,03	0,031	1
Bicd1	0,96304	0,010648	0,658	0,637	1
Gm14412	0,96314	0,003063	0,039	0,039	1
Zfp763	0,963181	-0,00524	0,097	0,096	1
Gon7	0,963192	0,017979	0,133	0,135	1
Pou5f2	0,963339	-0,00014	0,078	0,078	1
Mgat2	0,963495	0,015427	0,304	0,311	1
Fbxo36	0,963661	-0,00639	0,28	0,276	1
St6galnac4	0,963833	0,001768	0,03	0,031	1

Rps13	0,964095	0,0008	0,993	0,992	1
Zbed4	0,964154	0,002912	0,093	0,093	1
Trit1	0,964292	-0,0045	0,065	0,065	1
Prdm5	0,964369	-0,00349	0,171	0,169	1
Plcb1	0,964416	0,006504	0,026	0,026	1
Irak1	0,964452	0,009589	0,299	0,301	1
Blvra	0,964531	-0,02033	0,324	0,315	1
Ninl	0,964573	0,007452	0,026	0,026	1
Tamm41	0,964651	-0,00068	0,087	0,088	1
Mrps36	0,964864	0,008282	0,556	0,556	1
Zfp579	0,964972	0,004094	0,399	0,403	1
Akr1a1	0,965079	0,011581	0,929	0,924	1
Uba6	0,96509	0,02216	0,303	0,314	1
Mfhas1	0,965197	-0,0016	0,04	0,041	1
Dock2	0,965488	-0,00025	0,013	0,013	1
Fgd3	0,965538	0,007385	0,231	0,231	1
Zfp799	0,965622	-0,00774	0,148	0,146	1
Frg2f1	0,965659	0,000519	0,035	0,036	1
Gm10073	0,965877	-0,00185	0,05	0,05	1
Adat1	0,965975	0,002417	0,119	0,12	1
Map7	0,966109	-0,00309	0,993	0,998	1
Atg4b	0,966308	0,013465	0,36	0,366	1
Bard1	0,966373	0,002017	0,013	0,013	1
Mtpap	0,966724	0,013278	0,271	0,276	1
Sumo2	0,967005	0,006415	0,917	0,901	1
Pdcd4	0,967147	0,019548	0,379	0,384	1
Zfp867	0,967204	-0,00286	0,041	0,041	1
Amot	0,967257	0,000721	0,013	0,013	1
1700047I1	0,967257	0,000721	0,013	0,013	1
Gm19412	0,967257	0,000721	0,013	0,013	1
Slc38a3	0,967342	0,008774	0,093	0,093	1
Brdt	0,967369	-0,01291	0,323	0,315	1
Elk4	0,967373	0,014892	0,362	0,367	1
Dnaaf2	0,967415	-0,00804	0,027	0,028	1
Rgl2	0,967643	0,00614	0,175	0,174	1
Lsm7	0,967861	0,010797	0,6	0,62	1
B3galt6	0,96788	0,003336	0,14	0,14	1
Ctdspl2	0,968062	0,007455	0,321	0,328	1
Cbs	0,968377	-0,02748	0,213	0,211	1
Tedc1	0,968436	-0,00182	0,055	0,055	1
Mon1b	0,968451	0,000421	0,17	0,169	1
Nudt19	0,968734	0,007194	0,351	0,353	1
Mak16	0,968869	0,006832	0,284	0,286	1
Spata2l	0,969163	0,006801	0,044	0,044	1
Sorcs2	0,96969	0,002017	0,013	0,013	1
Shbg	0,96969	0,002017	0,013	0,013	1
Ptp4a2	0,969835	0,016676	0,931	0,928	1
Adhfe1	0,96989	0,003695	0,044	0,044	1
Umad1	0,969897	-0,00393	0,169	0,167	1
Exoc2	0,9701	0,00487	0,245	0,247	1
Gm15834	0,970556	-0,00181	0,018	0,018	1
AC123724.	0,971062	0,00524	0,03	0,031	1

Ogg1	0,971188	0,002977	0,116	0,115	1
Gm48678	0,97121	0,0107	0,03	0,031	1
Nfe2l2	0,971213	-0,02559	0,512	0,504	1
Rnasek	0,971468	0,003695	0,044	0,044	1
Fam3a	0,971634	0,000464	0,302	0,302	1
Trio	0,97168	0,004909	0,013	0,013	1
H2-Q10	0,97168	0,006191	0,013	0,013	1
Elf2	0,971756	-0,00935	0,561	0,546	1
Dffa	0,971874	0,006697	0,156	0,158	1
6430503K(	0,971984	-0,00169	0,174	0,174	1
Ei24	0,97202	-0,0022	0,677	0,667	1
Fra10ac1	0,972157	0,005119	0,25	0,252	1
Tgfb3	0,972265	0,006425	0,147	0,15	1
Sgms2	0,972552	-0,00342	0,133	0,133	1
Plekha4	0,972565	0,00555	0,013	0,013	1
Hmga1	0,972628	0,00437	0,077	0,076	1
Cep83	0,972928	-0,00342	0,237	0,234	1
Acaa1a	0,973182	0,010203	0,905	0,904	1
Sh3bgrl	0,97321	-0,00387	0,318	0,317	1
Them6	0,973215	-0,00509	0,116	0,115	1
Mplkip	0,97329	0,016159	0,291	0,296	1
Faap100	0,973326	0,004086	0,225	0,224	1
Zfp574	0,973485	-0,00525	0,125	0,125	1
Nmt2	0,973581	0,01281	0,263	0,267	1
Gm13054	0,973764	0,000442	0,018	0,018	1
D6Wsu163	0,973765	-0,00581	0,126	0,125	1
Esf1	0,973822	-0,01344	0,47	0,47	1
Slc25a23	0,973854	0,01783	0,608	0,62	1
Ddx41	0,97402	0,003804	0,223	0,224	1
Fkbp9	0,974061	-0,00338	0,189	0,189	1
Fbxl6	0,97425	0,003038	0,138	0,138	1
Zfp931	0,974338	0,003247	0,106	0,106	1
Edrf1	0,974362	0,01238	0,165	0,166	1
Stx17	0,974574	0,005871	0,386	0,389	1
Ubxn8	0,974651	0,019326	0,202	0,207	1
Adamts1	0,974766	0,090173	0,358	0,363	1
Gm45509	0,974807	0,013217	0,053	0,054	1
Sh3yl1	0,974892	0,00069	0,064	0,063	1
Commd9	0,975122	0,0037	0,511	0,535	1
Rfx1	0,975126	0,005871	0,088	0,089	1
Phf20l1	0,975395	0,019644	0,643	0,662	1
Atp6v0d1	0,975424	0,00544	0,873	0,872	1
Pigb	0,975787	0,005282	0,09	0,091	1
Syt4	0,975811	0,031402	0,025	0,024	1
Rfng	0,975822	0,018848	0,181	0,184	1
1700029I1	0,97584	0,001402	0,018	0,018	1
Rsu1	0,975845	0,000434	0,775	0,763	1
Zfp687	0,976029	0,004881	0,258	0,259	1
Cmtr2	0,976044	0,000326	0,054	0,054	1
Zmat1	0,976046	0,018332	0,351	0,358	1
Rsf1	0,976169	-0,00909	0,75	0,754	1
Raly	0,97625	0,006085	0,877	0,888	1

Ccdc157	0,976322	0,007958	0,08	0,081	1
Psmd1	0,976385	-0,00314	0,735	0,74	1
Txndc15	0,976445	0,003975	0,691	0,685	1
Slc41a2	0,976492	0,001806	0,039	0,039	1
Gadd45a	0,976729	0,032638	0,342	0,351	1
Rora	0,976735	0,002329	0,295	0,293	1
Zfp960	0,976783	0,001075	0,018	0,018	1
Isoc1	0,976797	-0,00275	0,084	0,085	1
Zfp874b	0,976833	0,013221	0,169	0,169	1
Aaed1	0,976921	-0,00399	0,269	0,267	1
Thada	0,976957	0,015037	0,121	0,122	1
Hpf1	0,977007	-0,00134	0,5	0,496	1
Map4	0,977013	0,008657	0,802	0,815	1
Cbfa2t2	0,977026	0,004628	0,253	0,255	1
Mbd6	0,977039	0,006795	0,314	0,317	1
Plbd2	0,977194	0,007869	0,43	0,429	1
4930480K2	0,97737	-0,00057	0,054	0,054	1
Sh3bgrl3	0,977697	0,000153	0,988	0,995	1
Ssbp3	0,977728	0,020801	0,365	0,374	1
Ergic2	0,977824	-0,00653	0,628	0,613	1
Klhl12	0,978007	0,001055	0,219	0,22	1
Tgfb1	0,978104	0,004592	0,018	0,018	1
Scg5	0,978208	0,035492	0,674	0,712	1
Umps	0,978362	-0,00252	0,112	0,112	1
Clcn2	0,978391	0,00203	0,373	0,376	1
Psmg2	0,978467	0,014455	0,267	0,272	1
Lonrf3	0,978596	0,004913	0,02	0,02	1
1810030O	0,978657	0,017524	0,27	0,273	1
Tnfrsf13c	0,978679	-0,00096	0,084	0,085	1
Arl4d	0,978735	-0,00425	0,242	0,241	1
Rnf8	0,978887	0,005336	0,189	0,19	1
Nsdhl	0,979251	0,000669	0,281	0,281	1
Gm44386	0,979313	0,005871	0,112	0,112	1
Pold1	0,979354	-0,0053	0,118	0,117	1
Cd99l2	0,979781	-0,00234	0,338	0,333	1
Slc25a33	0,979962	-0,00348	0,161	0,161	1
Chtf18	0,980035	0,00332	0,02	0,02	1
Asic3	0,98018	0,002671	0,018	0,018	1
Tmub2	0,98022	0,011507	0,386	0,393	1
Dus4l	0,980354	0,002989	0,113	0,114	1
Ddx49	0,980498	-0,00623	0,261	0,26	1
Wasf1	0,980683	-0,00604	0,317	0,317	1
4831440E1	0,980754	0,002996	0,02	0,02	1
Usp1	0,980797	0,013082	0,351	0,358	1
Eya4	0,980961	0,027983	0,211	0,216	1
Mrpl11	0,980989	0,004184	0,503	0,512	1
Slc22a5	0,981238	-0,00775	0,17	0,169	1
Wdr89	0,981255	0,01238	0,182	0,184	1
Stx5a	0,981535	0,009304	0,531	0,537	1
Npepl1	0,98163	0,016947	0,272	0,278	1
Arih2	0,981634	0,005647	0,344	0,345	1
Cc2d2a	0,9817	0,014213	0,197	0,2	1

Psmb1	0,981758	0,00852	0,956	0,938	1
9130221H1	0,981779	0,001466	0,034	0,034	1
Sec23b	0,981829	0,004225	0,429	0,429	1
Slain2	0,982077	0,014133	0,341	0,343	1
Olfr46	0,982141	0,001768	0,029	0,029	1
Htra1	0,982172	0,004528	0,768	0,74	1
Ybey	0,982232	0,009909	0,105	0,106	1
Bzw1	0,982352	-0,00045	0,93	0,912	1
6430573F1	0,982381	0,00276	0,045	0,046	1
Cend1	0,982418	0,014637	0,411	0,418	1
Cdc16	0,982436	0,005871	0,329	0,328	1
Syne2	0,982447	0,007884	0,182	0,182	1
Fancg	0,982515	0,007347	0,097	0,098	1
Abhd3	0,982596	-0,01182	0,28	0,276	1
Idua	0,982736	0,000938	0,11	0,111	1
Lpgat1	0,982884	0,025414	0,902	0,915	1
Arf6	0,983046	0,012086	0,255	0,259	1
Itga4	0,983161	0,015449	0,076	0,076	1
Mtor	0,983281	0,004152	0,27	0,27	1
Zfp771	0,983574	0,036966	0,77	0,767	1
Mier3	0,983942	-0,00323	0,234	0,233	1
Slc35f5	0,983963	0,002121	0,187	0,189	1
Aamp	0,984006	-0,00648	0,895	0,912	1
Gatd1	0,98406	0,004236	0,312	0,312	1
Atp8b5	0,984164	0,002597	0,082	0,083	1
Rbak	0,984479	0,018244	0,198	0,203	1
Prcp	0,984521	0,006094	0,336	0,343	1
Tmem17	0,984537	0,009159	0,083	0,085	1
Stag1	0,984647	0,033285	0,475	0,499	1
Sppl3	0,984708	0,01558	0,421	0,429	1
Snhg18	0,985146	0,005266	0,068	0,068	1
Senp1	0,985224	0,006601	0,256	0,257	1
Sh3bp4	0,985937	0,000401	0,065	0,065	1
Anapc10	0,985968	0,00144	0,207	0,207	1
Nav1	0,986045	0,020413	0,587	0,587	1
Yars2	0,986091	-0,0029	0,129	0,128	1
Eid1	0,986277	0,009967	0,977	0,987	1
Atp9b	0,986285	0,007298	0,285	0,286	1
4930550C1	0,986509	0,000442	0,02	0,02	1
Dvl3	0,986591	0,010558	0,342	0,345	1
Morc3	0,986698	-0,00874	0,234	0,233	1
Adprhl2	0,986891	0,005171	0,328	0,33	1
Clk2	0,986912	0,00511	0,235	0,237	1
Rab3a	0,987054	-0,01541	0,524	0,515	1
Vstm2b	0,987154	0,001052	0,015	0,015	1
Mfsd9	0,987338	-0,00043	0,032	0,033	1
Lmtk3	0,987534	-0,00214	0,047	0,047	1
Zfp790	0,987675	0,00014	0,075	0,075	1
Usp45	0,987684	0,010717	0,165	0,166	1
Tomm6os	0,988103	0,002968	0,01	0,01	1
D7Ertd443	0,988151	0,001989	0,622	0,626	1
Tbc1d20	0,988155	0,017947	0,309	0,315	1

Lca5	0,988232	0,003224	0,091	0,091	1
Ccdc25	0,98832	0,002594	0,288	0,291	1
Acsf3	0,98844	-0,00071	0,084	0,085	1
Zfp772	0,988944	0,004009	0,047	0,047	1
Lrsam1	0,989008	0,005871	0,139	0,14	1
Tmem69	0,989295	0,003414	0,055	0,055	1
Emsy	0,989343	0,018139	0,56	0,571	1
Arhgef19	0,98962	0,016014	0,071	0,072	1
Gm26668	0,989622	0,001028	0,01	0,01	1
Gm10353	0,989622	0,001028	0,01	0,01	1
Zfp507	0,989954	0,006409	0,182	0,184	1
Bmf	0,989956	0,013839	0,05	0,05	1
Efr3a	0,990042	-0,00746	0,298	0,298	1
4430402I1	0,990262	-0,00055	0,015	0,015	1
Gm26526	0,990283	0,002925	0,267	0,27	1
Rdx	0,990459	0,003092	0,998	0,998	1
Gm21663	0,990634	0,00039	0,01	0,01	1
Ctbp2	0,990634	0,00039	0,01	0,01	1
Gm32369	0,990634	0,00039	0,01	0,01	1
Anapc1	0,99089	0,007309	0,4	0,407	1
Arfrp1	0,99097	-0,00348	0,388	0,385	1
Antxr1	0,991048	0,003819	0,094	0,094	1
Adarb1	0,991091	0,000746	0,015	0,015	1
Trim17	0,99114	5,69E-05	0,01	0,01	1
Aard	0,99114	5,69E-05	0,01	0,01	1
Arid1a	0,99124	0,002316	0,653	0,654	1
Ppm1b	0,991271	0,005696	0,525	0,535	1
Rab5c	0,991296	0,00598	0,846	0,86	1
Atxn1l	0,9913	-0,00605	0,228	0,226	1
Pik3r1	0,991482	0,014259	0,964	0,976	1
Bcl2l12	0,991742	0,002626	0,092	0,093	1
Tcaf2	0,991787	0,00198	0,005	0,005	1
Pmel	0,991787	0,00198	0,005	0,005	1
Htr1b	0,991787	0,00198	0,005	0,005	1
Serpinf1	0,991787	0,00198	0,005	0,005	1
Calhm2	0,991787	0,00198	0,005	0,005	1
Atrip	0,991938	0,003164	0,171	0,171	1
Calr	0,991978	0,009321	0,98	0,99	1
Wars2	0,992039	0,001863	0,057	0,057	1
Eif1a	0,992073	0,027073	0,276	0,281	1
Spocd1	0,992153	-0,00058	0,01	0,01	1
Btaf1	0,992189	0,026879	0,276	0,285	1
Fbxo32	0,992246	0,014723	0,731	0,743	1
Ctbp1	0,992288	0,00105	0,851	0,854	1
Plcxd1	0,992501	0,001337	0,005	0,005	1
Ldb3	0,992799	-0,00055	0,272	0,27	1
Bbip1	0,992826	0,010982	0,628	0,652	1
Tceal5	0,992858	0,001005	0,005	0,005	1
Fam151a	0,992858	0,001005	0,005	0,005	1
Parva	0,992858	0,001005	0,005	0,005	1
Scamp4	0,992858	0,001005	0,005	0,005	1
Sh2d4b	0,992858	0,001005	0,005	0,005	1

Gm34934	0,992858	0,001005	0,005	0,005	1
Gm34868	0,992858	0,001005	0,005	0,005	1
Amhr2	0,992858	0,001005	0,005	0,005	1
Gm15956	0,992858	0,001005	0,005	0,005	1
Tesmin	0,992858	0,001005	0,005	0,005	1
Kif11	0,992858	0,001005	0,005	0,005	1
Nt5c3b	0,993224	-0,00389	0,281	0,278	1
Med29	0,99328	-0,003	0,382	0,379	1
Magi3	0,993321	-0,00217	0,044	0,044	1
Gm10791	0,993388	0,002709	0,101	0,101	1
Kras	0,993393	0,000608	0,509	0,504	1
Map3k20	0,993672	-0,00155	0,01	0,01	1
Hirip3	0,993757	0,021258	0,181	0,184	1
Ahr	0,993801	0,006188	0,023	0,023	1
Itfg1	0,993813	0,007405	0,753	0,764	1
Slc7a6	0,993815	0,009101	0,098	0,099	1
Ccdc137	0,993846	0,004517	0,17	0,171	1
3110035E1	0,993929	2,86E-05	0,005	0,005	1
Knl1	0,993929	2,86E-05	0,005	0,005	1
Gm42957	0,993929	2,86E-05	0,005	0,005	1
Glipr2	0,993929	2,86E-05	0,005	0,005	1
Gm43660	0,993929	2,86E-05	0,005	0,005	1
Nos1	0,993929	2,86E-05	0,005	0,005	1
Ache	0,993929	2,86E-05	0,005	0,005	1
2900089D1	0,993929	2,86E-05	0,005	0,005	1
Rasgrp4	0,993929	2,86E-05	0,005	0,005	1
Gm4353	0,993929	2,86E-05	0,005	0,005	1
Gm49083	0,993929	2,86E-05	0,005	0,005	1
Lingo1	0,993929	2,86E-05	0,005	0,005	1
Camkv	0,993929	2,86E-05	0,005	0,005	1
4833445I0	0,993929	2,86E-05	0,005	0,005	1
Gm46332	0,993929	2,86E-05	0,005	0,005	1
9030624G1	0,993929	2,86E-05	0,005	0,005	1
D430001F1	0,993929	2,86E-05	0,005	0,005	1
Ndc80	0,993929	2,86E-05	0,005	0,005	1
Rasgrp2	0,993929	2,86E-05	0,005	0,005	1
Rsad1	0,993973	-0,0022	0,079	0,078	1
Il2	0,994038	-0,00367	0,054	0,054	1
Rprm	0,994286	0,000363	0,005	0,005	1
Ahcy	0,994346	0,000843	0,037	0,037	1
Aimp2	0,994587	0,024171	0,291	0,302	1
Gm11851	0,994643	-0,00061	0,005	0,005	1
Pitpnc1	0,994643	-0,00061	0,005	0,005	1
Prkab1	0,994691	0,00674	0,115	0,115	1
Gm26909	0,994773	0,009185	0,149	0,151	1
S100a3	0,995	-0,00286	0,005	0,005	1
Pigu	0,995088	0,006516	0,379	0,384	1
Ppan	0,995147	0,01466	0,162	0,164	1
Thnsl1	0,995276	4,48E-05	0,151	0,151	1
Gps1	0,995294	0,009727	0,631	0,66	1
Gal3st1	0,9955	0,012449	0,949	0,94	1
Pitrm1	0,995566	0,004099	0,094	0,094	1

Tbcc	0,99563	-0,00155	0,286	0,286	1
Gm21988	0,995671	0,002859	0,079	0,08	1
Prpf3	0,9957	0,016386	0,157	0,159	1
Ccdc190	0,995715	-0,00159	0,005	0,005	1
Gm11261	0,995715	-0,00159	0,005	0,005	1
Msantd1	0,995715	-0,00159	0,005	0,005	1
Rbfox3	0,995715	-0,00159	0,005	0,005	1
Gm29675	0,995715	-0,00159	0,005	0,005	1
C030017D(	0,995715	-0,00159	0,005	0,005	1
Pcdhga1	0,995715	-0,00159	0,005	0,005	1
Dlg5	0,995858	0,01004	0,201	0,203	1
Stk17b	0,995941	-0,00259	0,072	0,072	1
Plxnb3	0,996111	0,003547	0,697	0,704	1
Fbxo38	0,996333	0,007804	0,284	0,288	1
Thap3	0,996573	0,004353	0,372	0,376	1
Ttyh3	0,996732	-0,00163	0,143	0,143	1
Evl	0,996858	0,000326	0,052	0,052	1
Chil1	0,996942	0,001825	0,047	0,047	1
Stoml2	0,997078	0,005447	0,401	0,403	1
Dpy19l4	0,997173	0,024876	0,386	0,402	1
Rbl1	0,9975	-0,0032	0,005	0,005	1
Cyb5b	0,997946	0,03135	0,413	0,431	1
Hnrnpul2	0,997951	0,003639	0,703	0,704	1
Kpna2	0,99798	0,000913	0,052	0,052	1
Necap2	0,998138	0,005237	0,612	0,624	1
Ap3d1	0,998203	0,015378	0,425	0,428	1
B230344G1	0,998204	-0,01094	0,14	0,138	1
Tbc1d19	0,99824	0,014592	0,279	0,283	1
Mcrs1	0,998663	-0,01014	0,468	0,465	1
Zdhhc14	0,998702	-0,00439	0,692	0,678	1
1700088E(	0,998798	0,004949	0,055	0,055	1
Prrc2a	0,99883	0,023902	0,686	0,688	1
Cxxc1	0,998898	0,016667	0,321	0,328	1
Thap12	0,998984	-0,01763	0,364	0,359	1
Macrod1	0,998987	0,003695	0,366	0,366	1
Cdk12	0,99909	0,010147	0,593	0,608	1
Smarcad1	0,999218	0,013974	0,329	0,333	1
Slc26a2	0,999268	0,01613	0,167	0,169	1
Spata31d1a	0,999282	0,006411	0,176	0,177	1
Atm	0,99936	-0,00038	0,238	0,237	1
Nthl1	0,999639	0,001755	0,217	0,218	1
Zfp503	0,999744	0,001793	0,094	0,094	1

	p_val	avg_logFC	pct.1	pct.2	p_val_adj
Gm8797	7,51E-22	-0,6487	0,058	0,523	1,18E-17
Psmb8	3,32E-14	-1,80806	0,042	0,354	5,24E-10
Tapbpl	9,11E-14	-0,67438	0,027	0,308	1,44E-09
Cd274	7,24E-13	-1,41388	0,027	0,292	1,14E-08
Xist	2,75E-11	1,8526	0,669	0,308	4,34E-07
Psmb9	3,56E-11	-0,95195	0,038	0,292	5,61E-07
Psmb10	4,57E-11	-0,95991	0,135	0,477	7,22E-07
Gbp3	6,22E-11	-0,96421	0,035	0,277	9,81E-07
Tap2	7,15E-11	-0,94485	0,054	0,323	1,13E-06
Tap1	6,67E-10	-0,99515	0,069	0,338	1,05E-05
Psme1	1,19E-09	-0,66256	0,173	0,508	1,88E-05
Ube2l6	1,54E-09	-0,27264	0,019	0,215	2,42E-05
Gbp7	2,12E-09	-1,23112	0,085	0,354	3,35E-05
Parp10	2,77E-09	-0,325	0,008	0,169	4,37E-05
Ifit3	1E-08	-1,16667	0,019	0,2	0,000158
Cmpk2	4,14E-08	-0,33488	0,023	0,2	0,000653
Isg15	9,41E-08	-0,40032	0	0,108	0,001484
Parp14	1,32E-07	-0,39561	0,015	0,169	0,00209
Irgm1	1,35E-07	-0,62362	0,208	0,523	0,002125
Tmem140	1,63E-07	-0,50867	0,027	0,2	0,002574
Bst2	1,98E-07	-0,87758	0,069	0,292	0,00312
Oasl2	2,71E-07	-0,46814	0,023	0,185	0,004283
Igtp	3,01E-07	-1,3938	0,112	0,354	0,004744
Tvp23a	6,32E-07	-0,26209	0,05	0,246	0,009977

	p_val	avg_logFC	pct.1	pct.2	p_val_adj
Tsix	2,21E-09	0,495146	0,448	0,096	3,49E-05
Hspa8	3,08E-09	-0,33313	1	1	4,85E-05
Xist	1,66E-08	0,841434	0,612	0,205	0,000261
Cpox	2,02E-08	-0,36404	1	1	0,000318
Mt1	2,72E-08	0,756803	0,866	0,658	0,000429
Serpind1	2,75E-07	-0,628	0,343	0,678	0,004339
Atp1b3	2,85E-07	0,438092	1	0,993	0,004494
Ncam1	5,29E-07	0,260971	1	1	0,008344

	ReactomeTerms[[i]]
1	No_Genes

	p_val	avg_logFC	pct.1	pct.2	p_val_adj
Tsix	6,39E-11	0,693147	0,625	0	1,01E-06
Flrt3	6,31E-07	0,318454	0,375	0	0,009956
Gm31805	6,31E-07	0,318454	0,375	0	0,009956
Pitrm1	6,31E-07	0,318454	0,375	0	0,009956
Gsto2	6,31E-07	0,318454	0,375	0	0,009956

	p_val	avg_logFC	pct.1	pct.2	p_val_adj
Xist	2,41E-19	1,400388	0,844	0,391	3,81E-15
Hspa8	1,5E-16	-0,43974	1	1	2,37E-12
Ddx3y	4,27E-10	-0,53033	0,163	0,489	6,73E-06
Apoe	9,76E-10	-0,75145	0,966	1	1,54E-05
Sept7	1,04E-08	-0,33548	1	1	0,000164
Sqle	1,41E-08	0,555746	0,782	0,496	0,000223
Msmo1	6,01E-08	0,492094	0,973	0,782	0,000949
Hmgcs1	9,91E-08	0,706599	0,973	0,932	0,001564
Tsix	1,02E-07	0,4149	0,299	0,053	0,001603
Tmod2	4,59E-07	-0,37394	0,932	0,962	0,007242
Idi1	4,92E-07	0,523064	0,81	0,579	0,007755

	p_val	avg_logFC	pct.1	pct.2	p_val_adj
Gstp1	5,1E-15	0,917306	1	0,96	8,05E-11
C4b	3,13E-13	0,820673	1	0,641	4,93E-09
Gatm	1,47E-12	-0,33053	1	1	2,33E-08
Etv1	2,03E-12	-0,569	0,966	0,996	3,21E-08
Xist	2,05E-12	1,005354	0,655	0,191	3,23E-08
Eml1	2,32E-12	-0,67327	0,534	0,9	3,65E-08
Cd63	4,29E-12	0,570363	1	0,976	6,77E-08
Tubb4a	8,75E-12	-0,53309	1	1	1,38E-07
mt-Co1	1,51E-11	-0,34353	1	1	2,38E-07
Pex5l	3,02E-11	-0,58985	1	1	4,77E-07
Gstm7	9,34E-11	-0,61032	0,862	0,972	1,47E-06
Qdpr	1,02E-10	-0,42445	1	1	1,61E-06
Gng11	3,16E-10	-0,5556	0,983	0,996	4,99E-06
Sparc	4,58E-10	0,591209	0,897	0,594	7,22E-06
mt-Nd4	2,23E-09	-0,32688	1	1	3,52E-05
Ppp1r14a	2,67E-09	-0,36966	1	1	4,21E-05
Slc44a1	3,97E-09	0,35717	1	1	6,26E-05
mt-Cytb	7,1E-09	-0,3127	1	1	0,000112
Spock3	7,4E-09	-0,48541	0,845	0,972	0,000117
H2-D1	8,09E-09	0,48963	0,879	0,566	0,000128
S100a16	9,07E-09	-0,44297	0,983	1	0,000143
Atg4c	1,34E-08	-0,41723	0,362	0,765	0,000211
B2m	3,11E-08	0,384635	0,845	0,482	0,000491
Slc38a2	3,74E-08	-0,45938	1	1	0,00059
Ctsk	5,33E-08	-0,45809	0,241	0,625	0,000841
Fos	7E-08	0,506909	0,81	0,394	0,001105
Anxa5	1,07E-07	0,457369	0,81	0,458	0,001692
Prnp	1,37E-07	0,2698	1	1	0,002162
Ndrg2	1,75E-07	-0,53333	0,155	0,526	0,002757
Psat1	1,96E-07	-0,3477	0,966	0,996	0,003092
Oaz1	2,72E-07	-0,32798	1	0,996	0,00429
Calm1	2,81E-07	-0,31177	1	1	0,004429
Sept7	2,83E-07	-0,30956	1	1	0,004457
Ndrg1	3,96E-07	0,286817	1	1	0,006247
Psap	5,46E-07	0,328064	1	1	0,008606
Asrgl1	5,84E-07	-0,43211	0,621	0,884	0,009211
Ankub1	5,92E-07	-0,47266	0,259	0,629	0,009339

	ReactomeTerms[[i]]
1	No_Genes

	p_val	avg_logFC	pct.1	pct.2	p_val_adj
Cd81	3,61E-08	0,277028	1	1	0,000569
Cisd1	1,93E-07	0,292458	0,974	0,89	0,003044
H2-D1	3,22E-07	-0,61542	0,479	0,726	0,005079
Fos	3,64E-07	-0,98254	0,359	0,623	0,005739
Hist1h2bc	5,33E-07	0,351682	0,94	0,877	0,008411
Selenof	5,45E-07	0,27004	0,957	0,966	0,008597

	p_val	avg_logFC	pct.1	pct.2	p_val_adj
Slc34a3	9,64E-10	0,77319	0,833	0,031	1,52E-05
3110082J2	8,69E-09	0,405465	0,5	0	0,000137
Gsto2	8,69E-09	0,405465	0,5	0	0,000137
Nppc	1,17E-07	0,48052	0,667	0,031	0,001838
Gm996	1,17E-07	0,48052	0,667	0,031	0,001838
Serpind1	1,51E-07	0,561015	0,667	0,031	0,002381
Sgsm3	1,89E-07	0,465705	0,667	0,031	0,002976
Aff3	1,89E-07	0,451106	0,667	0,031	0,002976
Mest	3,01E-07	0,394753	0,667	0,031	0,004754

	p_val	avg_logFC	pct.1	pct.2	p_val_adj
Pdlim2	1,27E-08	0,494462	0,989	0,977	0,0002
H2-D1	2,13E-08	-1,04767	0,685	0,887	0,000336
B2m	3,24E-08	-0,97289	0,618	0,85	0,000512

	p_val	avg_logFC	pct.1	pct.2	p_val_adj
Scnm1	2,99E-11	0,298091	0,676	0,398	4,72E-07
AY036118	1,08E-07	-0,25534	0,947	0,976	0,00171

	p_val	avg_logFC	pct.1	pct.2	p_val_adj
Abca1	5,11E-11	-0,82082	0,158	0,8	8,06E-07
Fxyd7	4,77E-10	-0,97548	0,05	0,467	7,53E-06
Ptgr1	3,93E-09	-0,28768	0	0,133	6,2E-05
Rnf219	8,29E-09	-0,33055	0,038	0,4	0,000131
Kcna4	1,97E-08	-0,26863	0,015	0,267	0,000311
Fxyd1	4,52E-08	-1,35148	0,127	0,6	0,000713
Gfap	8,13E-08	-0,4296	0,065	0,467	0,001283
Tmem176a	2,49E-07	-0,72055	0,1	0,533	0,003932
Apoe	2,95E-07	-1,39594	0,946	1	0,004652

	p_val	avg_logFC	pct.1	pct.2	p_val_adj
B2m	4,5E-15	1,244604	0,94	0,487	7,09E-11
Ncam1	7,56E-12	0,410157	1	1	1,19E-07
H2-D1	5,32E-11	1,14695	0,851	0,479	8,39E-07
Atp1a1	8,22E-11	0,412963	1	1	1,3E-06
Dip2a	9,56E-11	0,483992	1	0,966	1,51E-06
Serpind1	1,01E-10	-0,7342	0,343	0,803	1,59E-06
Shisa4	2,91E-10	-0,54986	0,94	1	4,59E-06
Bmyc	3,94E-10	-0,55461	0,657	0,923	6,21E-06
Sox11	7,19E-10	0,751188	0,403	0,043	1,13E-05
Sys1	9,13E-10	-0,49293	0,97	0,966	1,44E-05
Sptbn1	1,02E-09	0,559562	1	0,915	1,61E-05
Inf2	1,16E-09	0,459289	0,985	0,957	1,83E-05
Tulp4	1,18E-09	0,439698	1	1	1,87E-05
C4b	1,36E-09	0,755365	0,985	0,744	2,15E-05
Npc1	1,36E-09	0,432136	1	0,974	2,15E-05
Selenof	1,55E-09	-0,46053	0,91	0,957	2,45E-05
Plekhb1	2,13E-09	0,578697	1	1	3,37E-05
Hspa8	2,75E-09	-0,32147	1	1	4,34E-05
Gpc1	4,57E-09	0,504991	0,731	0,299	7,2E-05
Rexo2	6,14E-09	-0,47824	0,761	0,949	9,68E-05
Atp5f1	7,43E-09	-0,44251	0,925	0,991	0,000117
AY036118	9,91E-09	0,591383	1	0,769	0,000156
Rpl13a	1,01E-08	0,422307	0,985	0,872	0,000159
Cox5b	1,27E-08	-0,38801	0,925	0,991	0,0002
Cisd1	1,63E-08	-0,42894	0,791	0,974	0,000257
S100a16	1,63E-08	-0,44922	0,955	1	0,000257
Pacs2	1,9E-08	0,389276	1	1	0,000299
Sort1	1,96E-08	0,353612	1	1	0,000309
Hopx	2,13E-08	-0,4649	0,97	1	0,000336
Usmg5	2,21E-08	-0,43983	0,701	0,949	0,000348
Myrf	2,49E-08	0,373178	0,985	0,991	0,000392
Ppp1r14a	3,76E-08	-0,37445	0,985	1	0,000592
Bhlhe40	4,42E-08	0,350467	0,358	0,051	0,000698
Grhpr	4,75E-08	-0,4983	0,701	0,915	0,000749
Junb	4,84E-08	0,913498	0,507	0,154	0,000763
Ndufab1	6,31E-08	-0,42178	0,687	0,94	0,000995
Tmcc3	6,67E-08	0,368289	0,985	1	0,001053
Abca2	7,29E-08	0,315443	1	1	0,00115
Eef2	7,43E-08	0,338267	1	1	0,001172
Psmb2	7,62E-08	-0,40019	0,836	0,957	0,001203
Arhgap23	9,6E-08	0,452692	0,97	0,897	0,001515
Eif1	9,68E-08	-0,35536	1	1	0,001527
Taok1	9,96E-08	0,472064	0,94	0,795	0,001571
Phlda3	1,13E-07	-0,49026	0,672	0,88	0,001791
Trf	1,23E-07	-0,27952	1	1	0,00194
Fez1	1,23E-07	-0,35185	1	1	0,001946
Ypel3	1,24E-07	-0,40569	0,776	0,966	0,001959
Cox5a	1,26E-07	-0,43396	0,701	0,932	0,001994
Sept9	1,27E-07	0,461089	0,746	0,436	0,002004
Cox6c	1,28E-07	-0,33128	0,985	0,991	0,002027
Tmem50a	1,3E-07	-0,33464	0,955	1	0,002044

Tspan3	1,31E-07	-0,36603	0,925	1	0,002074
Nipa1	1,36E-07	-0,39889	0,91	0,983	0,00214
Chchd2	1,39E-07	-0,31433	1	1	0,002192
Gstm7	1,56E-07	-0,41907	0,866	0,991	0,002459
Prdx1	1,64E-07	-0,2833	1	1	0,002585
Slc25a27	1,66E-07	-0,57637	0,672	0,863	0,002626
Spcs1	1,7E-07	-0,42095	0,776	0,923	0,002685
Mt1	1,83E-07	0,784698	0,866	0,692	0,002885
Ndufa7	1,91E-07	-0,31593	0,791	0,974	0,003007
Tmem258	2,07E-07	-0,49593	0,701	0,88	0,003272
Rnf130	2,16E-07	-0,33213	1	1	0,003401
Pkp4	2,48E-07	0,552366	0,94	0,752	0,003911
Fos	2,54E-07	1,58391	0,687	0,359	0,004006
Egr1	2,82E-07	1,301922	0,299	0,034	0,004453
Dhcr24	3,44E-07	0,784601	0,791	0,573	0,005423
Gng11	3,64E-07	-0,41905	1	0,991	0,005743
Mrps33	3,91E-07	-0,47507	0,701	0,872	0,006167
Scarb2	3,93E-07	0,346034	1	1	0,006208
H2-K1	4,19E-07	0,777267	0,597	0,274	0,006607
Tfrc	4,2E-07	-0,41668	0,284	0,667	0,006631
Gstm5	4,22E-07	-0,49461	0,94	0,983	0,006659
Etv1	4,45E-07	-0,37361	0,97	0,991	0,007022
Brd2	4,47E-07	0,544903	0,851	0,675	0,007056
Mrps21	4,54E-07	-0,33384	0,582	0,915	0,007166
Cd63	4,56E-07	0,374134	0,925	0,564	0,007188
Anks1b	4,71E-07	0,485161	0,97	0,735	0,007428
Kif5a	4,89E-07	0,454827	0,672	0,342	0,007714
Edil3	5,4E-07	0,27271	1	1	0,008518
Adh5	5,64E-07	-0,38391	0,806	0,94	0,008905
Glo1	5,81E-07	-0,43224	0,687	0,932	0,009164
5031439G	6,2E-07	0,350832	0,97	0,88	0,009774
Dock7	6,22E-07	0,476136	0,731	0,41	0,00981
Glod4	6,33E-07	-0,40086	0,761	0,897	0,009981

	ReactomeTerms[[i]]
1	No_Genes

	p_val	avg_logFC	pct.1	pct.2	p_val_adj
Apoe	1,47E-18	-1,29259	0,966	0,978	2,31E-14
B2m	7,51E-18	1,165618	0,98	0,618	1,18E-13
Aplp1	6,87E-16	-0,43147	1	1	1,08E-11
H2-D1	2,03E-15	1,194204	0,952	0,685	3,2E-11
AY036118	1,3E-14	0,686309	1	0,933	2,06E-10
H2-K1	4,34E-14	1,008112	0,762	0,281	6,85E-10
Hmgcs1	2E-12	1,049022	0,973	0,91	3,15E-08
Tfrc	1,03E-11	-0,40038	0,17	0,629	1,63E-07
Pdlim2	1,13E-11	-0,61391	0,959	0,989	1,78E-07
Opalin	1,68E-10	-0,59544	0,952	0,978	2,64E-06
Sqle	8,14E-10	0,694601	0,782	0,449	1,28E-05
Itih3	9,18E-10	-0,3998	0,075	0,404	1,45E-05
Hspa8	2,43E-09	-0,37756	1	0,989	3,83E-05
Masp1	4,18E-09	-0,41988	0,116	0,461	6,59E-05
Sept4	6,39E-09	-0,39597	1	0,989	0,000101
Pmp22	9,34E-09	0,496511	0,993	0,809	0,000147
Idi1	3,61E-08	0,730779	0,81	0,517	0,000569
Dhcr24	3,9E-08	0,647321	0,68	0,337	0,000615
Ncam1	4,16E-08	0,339156	1	0,989	0,000656
Ppp1r14a	6,14E-08	-0,39144	1	0,989	0,000968
Msmo1	6,51E-08	0,578367	0,973	0,73	0,001027
Mag	7,21E-08	-0,3249	1	0,989	0,001138
Gfap	7,35E-08	-0,39236	0,088	0,371	0,00116
Cwc25	8,76E-08	-0,31839	0,156	0,494	0,001382
Enpp6	9,83E-08	0,38713	0,966	0,596	0,00155
Sept7	2,13E-07	-0,35071	1	0,989	0,003355
S100b	2,44E-07	0,674981	0,891	0,685	0,003851
1500011B(	2,99E-07	-0,29114	0,136	0,449	0,004718
Bin1	4,26E-07	-0,34032	0,993	1	0,006722
Hsp90aa1	5,29E-07	-0,32277	1	0,989	0,008352
Tmem163	5,3E-07	-0,39218	0,163	0,449	0,008364

	p_val	avg_logFC	pct.1	pct.2	p_val_adj
B2m	6,09E-16	1,065993	0,845	0,381	9,6E-12
C4b	7,97E-14	0,847311	1	0,537	1,26E-09
Gstm7	2,51E-13	-0,68616	0,862	0,967	3,97E-09
H2-D1	8E-13	1,220854	0,879	0,508	1,26E-08
Fos	9,07E-13	0,854886	0,81	0,303	1,43E-08
Aplp1	2,46E-12	-0,30493	1	1	3,88E-08
Gng11	2,47E-12	-0,65626	0,983	0,996	3,9E-08
Tubb4a	6,88E-12	-0,52051	1	1	1,09E-07
Tuba1a	1,05E-11	-0,56438	1	1	1,66E-07
Egr1	1,43E-11	0,463739	0,328	0,041	2,26E-07
Eml1	1,77E-11	-0,65682	0,534	0,885	2,8E-07
Edil3	2,22E-11	0,397961	1	1	3,5E-07
AY036118	2,66E-11	0,819474	1	0,947	4,19E-07
Etv1	3,1E-11	-0,53584	0,966	1	4,89E-07
Ppp1r14a	5,71E-11	-0,42711	1	1	9E-07
Gstp1	7,38E-11	0,724706	1	0,992	1,16E-06
Eif1	1,46E-10	-0,29239	1	1	2,31E-06
Psap	1,67E-10	0,443951	1	1	2,64E-06
mt-Co1	1,69E-10	-0,30513	1	1	2,67E-06
Cd63	3,39E-10	0,507862	1	0,963	5,35E-06
Tfrc	4,01E-10	-0,53028	0,138	0,598	6,32E-06
S100a16	6,8E-10	-0,49202	0,983	1	1,07E-05
Spock3	7,95E-10	-0,52931	0,845	0,988	1,25E-05
Oaz1	1,89E-09	-0,37731	1	0,996	2,98E-05
Calm1	2,03E-09	-0,35788	1	1	3,2E-05
Pex5l	2,64E-09	-0,50552	1	1	4,17E-05
Qdpr	3,43E-09	-0,37933	1	1	5,41E-05
Epb41l3	7,82E-09	-0,38946	0,983	0,988	0,000123
H2-K1	8,51E-09	0,764092	0,707	0,357	0,000134
Prdx1	1,14E-08	-0,35338	1	1	0,00018
Mif	1,29E-08	-0,52472	0,948	0,984	0,000203
Ywhaq	2,19E-08	-0,28137	1	1	0,000345
Sparc	2,25E-08	0,538191	0,897	0,623	0,000355
Junb	4,82E-08	0,542576	0,517	0,18	0,000761
Slc44a1	5,13E-08	0,325695	1	1	0,00081
Cyp2j6	5,66E-08	-0,44098	0,276	0,721	0,000892
Gpc1	5,94E-08	0,503905	0,69	0,361	0,000937
Cfl1	8,62E-08	-0,26802	1	1	0,001359
Bpgm	9,21E-08	-0,32954	0,983	1	0,001453
Fam102a	1,02E-07	0,372138	1	0,885	0,001607
Cox6c	1,16E-07	-0,27561	1	1	0,001829
Atg4c	1,29E-07	-0,41566	0,362	0,738	0,002036
Ndrg2	1,35E-07	-0,52968	0,155	0,533	0,002136
Anxa5	1,4E-07	0,470757	0,81	0,512	0,002203
Uqcr10	1,58E-07	-0,33256	0,948	0,992	0,002487
Sept7	1,82E-07	-0,31199	1	1	0,002872
Mt1	1,84E-07	0,909965	0,983	0,934	0,002901
Psat1	1,97E-07	-0,34959	0,966	1	0,003114
Gab1	2,15E-07	0,399967	0,966	0,959	0,00339
Prnp	2,52E-07	0,254135	1	1	0,003972
Apoe	2,86E-07	-0,73949	1	1	0,004516

Erbin	3,08E-07	0,495243	0,983	0,967	0,004858
Hint1	4,9E-07	-0,29113	0,983	0,996	0,007734
Il18	4,93E-07	-0,37225	0,448	0,779	0,007775
Ankub1	4,99E-07	-0,36457	0,259	0,664	0,007872
Fam96a	5,91E-07	-0,37537	0,19	0,574	0,00932
