# Supplementary material for: Distinct oligodendrocyte populations have spatial preference and different responses to spinal cord injury
Source: Nat Commun. 2020 Nov 17;11:5860. doi: 10.1038/s41467-020-19453-x (PMC7673029; doi:10.1038/s41467-020-19453-x)
Supplement: Supplementary file 11 — Reporting Summary [file 41467_2020_19453_MOESM11_ESM.pdf]

nature research

Corresponding author(s): Elisa Floriddia, Gonçalo Castelo-Branco

Last updated by author(s): Sep 13, 2020

Reporting Summary

Nature Research wishes to improve the reproducibility of the work that we publish. This form provides structure for consistency and transparency in reporting. For further information on Nature Research policies, see Authors & Referees and the Editorial Policy Checklist .

Statistics

For all statistical analyses, confirm that the following items are present in the figure legend, table legend, main text, or Methods section.

n/a	Confirmed	
☐	☒	The exact sample size (*n*) for each experimental group/condition, given as a discrete number and unit of measurement
☐	☒	A statement on whether measurements were taken from distinct samples or whether the same sample was measured repeatedly
☐	☒	The statistical test(s) used AND whether they are one- or two-sided<br>*Only common tests should be described solely by name; describe more complex techniques in the Methods section.*
☒	☐	A description of all covariates tested
☐	☒	A description of any assumptions or corrections, such as tests of normality and adjustment for multiple comparisons
☐	☒	A full description of the statistical parameters including central tendency (e.g. means) or other basic estimates (e.g. regression coefficient) AND variation (e.g. standard deviation) or associated estimates of uncertainty (e.g. confidence intervals)
☐	☒	For null hypothesis testing, the test statistic (e.g. *F*, *t*, *r*) with confidence intervals, effect sizes, degrees of freedom and *P* value noted<br>*Give P values as exact values whenever suitable.*
☒	☐	For Bayesian analysis, information on the choice of priors and Markov chain Monte Carlo settings
☒	☐	For hierarchical and complex designs, identification of the appropriate level for tests and full reporting of outcomes
☒	☐	Estimates of effect sizes (e.g. Cohen's *d*, Pearson's *r*), indicating how they were calculated

Our web collection on statistics for biologists contains articles on many of the points above.

Software and code

Policy information about availability of computer code

Data collection	For scRNA-seq data, Fast-Q alignment done using STAR aligner.<br><br>Images for immunohistochemistry, immunocytochemistry and RNASCOPE were acquired using the LSM800 confocal microscope set up (Zeiss).<br><br>Images for In Situ Sequencing were acquired using a Zeiss Axio Imager Z2 epifluorescence microscope (Zeiss Oberkochen, Germany). A series of images (10% overlap between two neighboring images) at different focal depths was obtained and the stacks of images were merged to a single image thereafter using the maximum-intensity projection (MIP) in the Zeiss ZEN 3 software. A custom-made Cellprofiler 2.2.1 pipeline was used to extract the fluorescence intensity from each of the signal (each detected RCA product) and to save all intensity information and coordinates, followed by signal decoding using a custom-made Matlab pipeline.

Data analysis

All tools described in the methods section of the manuscript, source code and notebooks are available at https://github.com/Castelo-Branco-lab under the repository https://github.com/Castelo-Branco-lab/Floriddia_et_al_2019

R statistical computing platform (Microsoft R Open 3.4.2), we also provide a full R session info file containing all packages and their versions used. Furthermore, essential packages used include Seurat R package, umap-learn 0.4.3, e1071 R package (https://CRAN.R-project.org/package=e1071, spdep 0.6-15, facto extra 1.0.5 , MAST R package v1.4.1, clusterProfiler package v3.6.0, MetaNeighbor v1, Cytoscape v3.5.1, Zen 3 software, Fiji version 1.0.

Confocal images were processed with FiJi (ImageJ, NIH) to select the regions of interest (ROIs). ROI images were segmented with a customized CellProfiler pipeline. The image analysis pipeline is available at the following link: https://github.com/Castelo-Branco-lab/Floriddia_et_al_2019

For manuscripts utilizing custom algorithms or software that are central to the research but not yet described in published literature, software must be made available to editors/reviewers. We strongly encourage code deposition in a community repository (e.g. GitHub). See the Nature Research guidelines for submitting code & software for further information.

Data

Policy information about availability of data

All manuscripts must include a data availability statement. This statement should provide the following information, where applicable:

- Accession codes, unique identifiers, or web links for publicly available datasets
- A list of figures that have associated raw data
- A description of any restrictions on data availability

We declare that the data supporting the findings of this study are available within the paper. Source data are provided with this paper. Single-cell RNA Sequencing dataset is available in GEO (accession number GSE128525). The sequencing dataset can also be explored at and visualized at https://ki.se/en/ mbb/oligointernode. Additional information is available upon reasonable request to the Authors.

Field-specific reporting

Please select the one below that is the best fit for your research. If you are not sure, read the appropriate sections before making your selection.

☒ Life sciences ☐ Behavioural & social sciences ☐ Ecological, evolutionary & environmental sciences

For a reference copy of the document with all sections, see nature.com/documents/nr-reporting-summary-flat.pdf

Life sciences study design

All studies must disclose on these points even when the disclosure is negative.

Sample size

No sample size calculation was performed to pre-determine sample sizes. Our sample sizes are in the same range of other studies in the field and following the 3Rs principle (https://ec.europa.eu/environment/chemicals/lab_animals/3r/alternative_en.htm).

For single cell RNA-seq from corpus callosum of P60 Emx1::Cre-Sox10::Cre-LoxP-GFP-STOP-TdTom, samples were collected from 2 mice, which was were sufficient to obtain the number of cells necessary to perform a confident data analysis and comparisons between ventral and dorsal derived OL populations. The overall findings were also confirmed by lineage tracing analysis combined with RNASCOPE.

For single cell RNA-seq from spinal cord injury, the injury site, rostral and caudal wallerian degeneration regions or control spinal cord of three months Sox10::Cre-GFP mice were microdissected from eight injured and five laminectomy mice. The dissected tissue was pooled and dissociated into a single-cell suspension. The overall findings were also confirmed by RNASCOPE.

For Immunohistochemistry/RNAscope ISH performed in mouse tissue, the sample size was n=3-9, while for In Situ Sequencing (ISS), the sample size was n=2. This sample size of ISS was found sufficient for validations of findings from RNAscope ISH.

For Immunocytochemistry of microfiber experiments: n=3-11

Data exclusions

For single cell RNA-Seq data, we excluded data points through our quality control pipeline, as indicated in the methods section in the paper. In short, for the single cell analysis of corpus callosum, thresholds were set for a minimum of 200 genes expressed per cells, with a maximum of 3000 genes. We also set a threshold for mitochondrial expression as a percentage of total measure UMI counts, permitting cells to pass the threshold of less than 5% of mitochondrial UMI counts of the total number of UMI counts. For the single cell analysis of spinal cord tissue, thresholds were set for a minimum of 500 genes expressed per cells, with a maximum of 7000 genes. We also set a threshold for mitochondrial expression as a percentage of total measure UMI counts, permitting cells to pass the threshold of less than 10% of mitochondrial UMI counts of the total number of UMI counts.

Replication

Several orthogonal techniques (RNAScope ISH, IHC, and ISS, lineage tracing and scRNA-Seq) were used throughout the manuscript to validated our findings. RNAScope ISH, IHC and lineage tracing were replicated with a minimum of three independent experiments and replication attempts provided consistent results. scRNAseq were not replicated but the sample size and number of analyzed cells were sufficiently powered.

Randomization	Randomization was not applied because we worked with WT animals or transgenic mice of known genotype from the same litters. All final experiments included WT and trangenic mice from minimum two litters from different breeding pairs.
Blinding	Blinding to the animal genotypes or injury vs naive conditions of the researchers was not necessary because the tissue (RNAscope ISH, IHC, ISS, ICC) and molecular (SCRNAseq) analyses were automated. For image acquisition, the region of interests were acquired on randomly selected tissue sections.

Reporting for specific materials, systems and methods

We require information from authors about some types of materials, experimental systems and methods used in many studies. Here, indicate whether each material, system or method listed is relevant to your study. If you are not sure if a list item applies to your research, read the appropriate section before selecting a response.

Materials & experimental systems

n/a	Involved in the study
☐	☒ Antibodies
☒	☐ Eukaryotic cell lines
☒	☐ Palaeontology
☐	☒ Animals and other organisms
☒	☐ Human research participants
☒	☐ Clinical data

Methods

n/a	Involved in the study
☒	☐ ChIP-seq
☒	☐ Flow cytometry
☒	☐ MRI-based neuroimaging

Antibodies

Antibodies used

For Immunocytochemistry and Immunohistochemistry the antibodies description is the following:

chicken anti-GFP (AbCam, ab 13970, RRID:AB_300798)
goat anti-Sox10 (Santa Cruz, sc-17342, RRID:AB_2195374)
rat anti-MBP (AbCam, ab 7349, RRID:AB_305869)
goat anti-PDGFR-alpha (R&D Systems, AF1062, RRID:AB_305869)

goat anti-chicken AlexaFluor 488 conjugated (AbCam, ab150169)
donkey anti-goat AlexaFluor 647 conjugated (LifeTech, A21447)
donkey anti-rat AlexaFluor 488 conjugated (Invitrogen (ThermoFisher Scientific); A21208; polyclonal, LOT 1229697)

RNAscope probes:

Sox10-C1 or -C2 (ACD Biotechne, 435931)
Ptgds-C1 (ACD Biotechne, 492781)
Klk6-C3 (ACD Biotechne, 493751)
Egr2-C3 (ACD Biotechne, 407871)
Ptprz1-C1 (ACD Biotechne, 460991)
Itpr2-C1 (ACD Biotechne, 462071)
Dusp1-C1 (ACD Biotechne, 424501)

Validation

All antibodies used in this study have been cited by other authors:

chicken anti-GFP (AbCam, ab 13970, RRID:AB_300798)
Recommended by manufacturer: IHC-P, WB, ICC/IF, IHC-Fr, IHC-FoFr
Citations:
Disease-specific oligodendrocyte lineage cells arise in multiple sclerosis. doi:10.1038/s41591-018-0236-y.
Oligodendrocyte heterogeneity in the mouse juvenile and adult central nervous system. doi:10.1126/science.aaf6463.
GABAergic regulation of cerebellar NG2-cell development is altered in perinatal white matter injury. doi:10.1038/nn.3990.
CNS-Resident Glial Progenitor/Stem Cells Produce Schwann Cells as well as Oligodendrocytes during Repair of CNS Demyelination. doi:10.1016/j.stem.2010.04.002.
A Common Progenitor for Retinal Astrocytes and Oligodendrocytes. DOI:10.1523/JNEUROSCI.3456-09.2010

goat anti-Sox10 (Santa Cruz, sc-17342, RRID:AB_2195374)
Recommended by manufacturer: -
Citations:
Oligodendrocyte heterogeneity in the mouse juvenile and adult central nervous system. doi:10.1126/science.aaf6463.

Gammill, L.S. and Roffers-Agarwal, J. 2010. Division of labor during trunk neural crest development. Dev. Biol. 344: 555-565.
Heanue, T.A., et al. 2011. Prospective identification and isolation of enteric nervous system progenitors using Sox2. Stem Cells 29: 128-140.
Guo, F., et al. 2011. Macroglial plasticity and the origins of reactive astroglia in experimental autoimmune encephalomyelitis. J. Neurosci. 31: 11914-11928
Distinct adhesion-independent functions of β-catenin control stage-specific sensory neurogenesis and proliferation. DOI 10.1186/s12915-015-0134-4

rat anti-MBP (AbCam, ab 7349, RRID:AB_305869)
Recommended by manufacturer: IHC-P, WB; Use at an assay dependent concentration
Citations:
PAD2-Mediated Citrullination Contributes to Efficient Oligodendrocyte Differentiation and Myelination. doi: 10.1016/j.celrep.2019.03.108.
Astrocytes Are Required for Oligodendrocyte Survival and Maintenance of Myelin Compaction and Integrity. DOI: 10.3389/fncel.2020.00074
Oligodendrocyte Intrinsic miR-27a Controls Myelination and Remyelination. DOI: 10.1016/j.celrep.2019.09.020
Circulating transforming growth factor-β1 facilitates remyelination in the adult central nervous system. doi: 10.7554/eLife.41869.
Functional genomic analysis of oligodendrocyte differentiation. doi: 10.1523/JNEUROSCI.2572-06.2006.
A competitive advantage by neonatally engrafted human glial progenitors yields mice whose brains are chimeric for human glia. DOI:10.1523/JNEUROSCI.1510-14.2014
Oligodendrocyte-encoded Kir4.1 function is required for axonal integrity. . DOI: https://doi.org/10.7554/eLife.36428
Dicer1 and miR-219 Are Required for Normal Oligodendrocyte Differentiation and Myelination. doi:10.1016/j.neuron.2010.01.027.

goat anti-PDGFR-alpha (R&D Systems, AF1062, RRID:AB_305869)
Recommended by manufacturer: ELISA, IHC-FoFr, IHC-Fr, IHC-P, RIA, WB; Immunohistochemistry; Other; Western Blot; Chromatography; Immunohistochemistry - fixed; Immunofluorescence; ELISA; Immunohistochemistry - frozen; Radioimmunoassay
Citations:
Transcriptional Convergence of Oligodendrocyte Lineage Progenitors during Development. https://doi.org/10.1016/j.devcel.2018.07.005
Neuronal Activity Promotes Oligodendrogenesis and Adaptive Myelination in the Mammalian Brain. DOI: 10.1126/science.1252304
Changes in the Oligodendrocyte Progenitor Cell Proteome with Ageing. https://doi.org/10.1074/mcp.RA120.002102

In addition, regarding antibodies used in IHC and ICC, we have further evaluated the specificity of the antibodies in our tissue by analyzing the presence of the antibody signal in regions where the protein should be expressed and its absence in regions where the protein shouldn't be expressed. We have further evaluated the location /morphology of the signal within the cell, for transcription factors where we expect a nuclear signal, whether for proteins like MBP, the signal was cytoplasmic, as expected.

Animals and other organisms

Policy information about studies involving animals; ARRIVE guidelines recommended for reporting animal research

Laboratory animals	Mouse lines used in this study are Pdgfrα::CreERTM-RCE::LoxP- RCE (Z/EG, mixed C57BL/6NJ and CD1 background) (The Jackson Laboratory, stock nr. 018280), Sox10::CreERT2-ROSA26::LoxP-GFP (C57B6/J, http://www.informatics.jax.org/allele/MGI:5301107, from Vasillis Pachnis, Francis Crick Institute, and William Richardson, University College of London), Emx1::Cre-Sox10::Cre-LoxP-GFP-STOP-TdTom (from William Richardson, University College of London, mixed CBA and C57BL/6 background)25 and Olig2::Cre+-TFEBfl/fl. Further information regarding strains, animal husbandry and experimental details on lineage tracing, spinal cord injury and post-operative care and Experimental Autoimmune Encephalomyelitis, can be found in the methods sections of the manuscript. For all the mouse strains, we used animals of both sexes at P20 or P60. General housing parameters such as relative humidity, temperature, and ventilation follow the European convention for the protection of vertebrate animals used for experimental and other scientific purposes treaty ETS 123. Briefly, consistent relative air humidity of 50%, 22°C and the air quality is controlled with the use of stand-alone air handling units supplemented with HEPA filtrated air. Monitoring of husbandry parameters is done using ScanClime (Scanbur) units.
Wild animals	The study did not involve wild animals
Field-collected samples	The study did not involve samples collected from the field.
Ethics oversight	All experimental procedures in this study were conducted in accordance with the European directive 2010/63/EU, local Swedish directive L150/SJVFS/2019:9, Saknr L150 and Karolinska Institutet complementary guidelines for procurement and use of laboratory animals, Dnr 1937/03-640. The procedures described here were approved by Stockholms Norra Djurförsöksetiska nämnd, the local committee for ethical experiments on laboratory animals in Sweden, lic.nr. 130/15, 144/16, & 1995/2019.

Note that full information on the approval of the study protocol must also be provided in the manuscript.
